# Height and body-mass index trajectories of school-aged children and adolescents from 1985 to 2019 in 200 countries and territories: a pooled analysis of 2181 population-based studies with 65 million participants

**DOI:** 10.1016/S0140-6736(20)31859-6

**Published:** 2020-11-07

**Authors:** NCD Risk Factor Collaboration (NCD-RisC), Andrea Rodriguez-Martinez, Bin Zhou, Marisa K Sophiea, James Bentham, Christopher J Paciorek, Maria LC Iurilli, Rodrigo M Carrillo-Larco, James E Bennett, Mariachiara Di Cesare, Cristina Taddei, Honor Bixby, Gretchen A Stevens, Leanne M Riley, Melanie J Cowan, Stefan Savin, Goodarz Danaei, Adela Chirita-Emandi, Andre P Kengne, Young-Ho Khang, Avula Laxmaiah, Reza Malekzadeh, J Jaime Miranda, Jin Soo Moon, Stevo R Popovic, Thorkild IA Sørensen, Maroje Soric, Gregor Starc, Ahmad A Zainuddin, Edward W Gregg, Zulfiqar A Bhutta, Robert Black, Leandra Abarca-Gómez, Ziad A Abdeen, Shynar Abdrakhmanova, Suhaila Abdul Ghaffar, Hanan F Abdul Rahim, Niveen M Abu-Rmeileh, Jamila Abubakar Garba, Benjamin Acosta-Cazares, Robert J Adams, Wichai Aekplakorn, Kaosar Afsana, Shoaib Afzal, Imelda A Agdeppa, Javad Aghazadeh-Attari, Carlos A Aguilar-Salinas, Charles Agyemang, Mohamad Hasnan Ahmad, Noor Ani Ahmad, Ali Ahmadi, Naser Ahmadi, Soheir H Ahmed, Wolfgang Ahrens, Gulmira Aitmurzaeva, Kamel Ajlouni, Hazzaa M Al-Hazzaa, Amani Rashed Al-Othman, Rajaa Al-Raddadi, Monira Alarouj, Fadia AlBuhairan, Shahla AlDhukair, Mohamed M Ali, Abdullah Alkandari, Ala'a Alkerwi, Kristine Allin, Mar Alvarez-Pedrerol, Eman Aly, Deepak N Amarapurkar, Parisa Amiri, Norbert Amougou, Philippe Amouyel, Lars Bo Andersen, Sigmund A Anderssen, Lars Ängquist, Ranjit Mohan Anjana, Alireza Ansari-Moghaddam, Hajer Aounallah-Skhiri, Joana Araújo, Inger Ariansen, Tahir Aris, Raphael E Arku, Nimmathota Arlappa, Krishna K Aryal, Thor Aspelund, Felix K Assah, Maria Cecília F Assunção, May Soe Aung, Juha Auvinen, Mária Avdicová, Ana Azevedo, Mohsen Azimi-Nezhad, Fereidoun Azizi, Mehrdad Azmin, Bontha V Babu, Maja Bæksgaard Jørgensen, Azli Baharudin, Suhad Bahijri, Jennifer L Baker, Nagalla Balakrishna, Mohamed Bamoshmoosh, Maciej Banach, Piotr Bandosz, José R Banegas, Joanna Baran, Carlo M Barbagallo, Alberto Barceló, Amina Barkat, Aluisio JD Barros, Mauro Virgílio Gomes Barros, Abdul Basit, Joao Luiz D Bastos, Iqbal Bata, Anwar M Batieha, Rosangela L Batista, Zhamilya Battakova, Assembekov Batyrbek, Louise A Baur, Robert Beaglehole, Silvia Bel-Serrat, Antonisamy Belavendra, Habiba Ben Romdhane, Judith Benedics, Mikhail Benet, James E Bennett, Salim Berkinbayev, Antonio Bernabe-Ortiz, Gailute Bernotiene, Heloísa Bettiol, Jorge Bezerra, Aroor Bhagyalaxmi, Sumit Bharadwaj, Santosh K Bhargava, Zulfiqar A Bhutta, Hongsheng Bi, Yufang Bi, Daniel Bia, Elysée Claude Bika Lele, Mukharram M Bikbov, Bihungum Bista, Dusko J Bjelica, Peter Bjerregaard, Espen Bjertness, Marius B Bjertness, Cecilia Björkelund, Katia V Bloch, Anneke Blokstra, Simona Bo, Martin Bobak, Lynne M Boddy, Bernhard O Boehm, Heiner Boeing, Jose G Boggia, Elena Bogova, Carlos P Boissonnet, Stig E Bojesen, Marialaura Bonaccio, Vanina Bongard, Alice Bonilla-Vargas, Matthias Bopp, Herman Borghs, Pascal Bovet, Lien Braeckevelt, Lutgart Braeckman, Marjolijn CE Bragt, Imperia Brajkovich, Francesco Branca, Juergen Breckenkamp, João Breda, Hermann Brenner, Lizzy M Brewster, Garry R Brian, Lacramioara Brinduse, Sinead Brophy, Graziella Bruno, H Bas Bueno-de-Mesquita, Anna Bugge, Marta Buoncristiano, Genc Burazeri, Con Burns, Antonio Cabrera de León, Joseph Cacciottolo, Hui Cai, Tilema Cama, Christine Cameron, José Camolas, Günay Can, Ana Paula C Cândido, Felicia Cañete, Mario V Capanzana, Nadežda Capková, Eduardo Capuano, Vincenzo Capuano, Marloes Cardol, Viviane C Cardoso, Axel C Carlsson, Esteban Carmuega, Joana Carvalho, José A Casajús, Felipe F Casanueva, Ertugrul Celikcan, Laura Censi, Marvin Cervantes-Loaiza, Juraci A Cesar, Snehalatha Chamukuttan, Angelique W Chan, Queenie Chan, Himanshu K Chaturvedi, Nish Chaturvedi, Norsyamlina Che Abdul Rahim, Chien-Jen Chen, Fangfang Chen, Huashuai Chen, Shuohua Chen, Zhengming Chen, Ching-Yu Cheng, Bahman Cheraghian, Angela Chetrit, Ekaterina Chikova-Iscener, Arnaud Chiolero, Shu-Ti Chiou, Adela Chirita-Emandi, María-Dolores Chirlaque, Belong Cho, Kaare Christensen, Diego G Christofaro, Jerzy Chudek, Renata Cifkova, Michelle Cilia, Eliza Cinteza, Frank Claessens, Janine Clarke, Els Clays, Emmanuel Cohen, Hans Concin, Susana C Confortin, Cyrus Cooper, Tara C Coppinger, Eva Corpeleijn, Simona Costanzo, Dominique Cottel, Chris Cowell, Cora L Craig, Amelia C Crampin, Ana B Crujeiras, Semánová Csilla, Alexandra M Cucu, Liufu Cui, Felipe V Cureau, Graziella D'Arrigo, Eleonora d'Orsi, Liliana Dacica, María Ángeles Dal Re Saavedra, Jean Dallongeville, Albertino Damasceno, Camilla T Damsgaard, Goodarz Danaei, Rachel Dankner, Thomas M Dantoft, Parasmani Dasgupta, Saeed Dastgiri, Luc Dauchet, Kairat Davletov, Guy De Backer, Dirk De Bacquer, Giovanni de Gaetano, Stefaan De Henauw, Paula Duarte de Oliveira, David De Ridder, Karin De Ridder, Susanne R de Rooij, Delphine De Smedt, Mohan Deepa, Alexander D Deev, Vincent Jr DeGennaro, Abbas Dehghan, Hélène Delisle, Francis Delpeuch, Stefaan Demarest, Elaine Dennison, Katarzyna Deren, Valérie Deschamps, Klodian Dhana, Meghnath Dhimal, Augusto F Di Castelnuovo, Juvenal Soares Dias-da-Costa, María Elena Díaz-Sánchez, Alejandro Diaz, Zivka Dika, Shirin Djalalinia, Visnja Djordjic, Ha TP Do, Annette J Dobson, Maria Benedetta Donati, Chiara Donfrancesco, Silvana P Donoso, Angela Döring, Maria Dorobantu, Ahmad Reza Dorosty, Kouamelan Doua, Wojciech Drygas, Jia Li Duan, Charmaine A Duante, Priscilla Duboz, Rosemary B Duda, Vesselka Duleva, Virginija Dulskiene, Samuel C Dumith, Anar Dushpanova, Vilnis Dzerve, Elzbieta Dziankowska-Zaborszczyk, Ricky Eddie, Ebrahim Eftekhar, Eruke E Egbagbe, Robert Eggertsen, Sareh Eghtesad, Gabriele Eiben, Ulf Ekelund, Mohammad El-Khateeb, Jalila El Ati, Denise Eldemire-Shearer, Marie Eliasen, Paul Elliott, Reina Engle-Stone, Macia Enguerran, Rajiv T Erasmus, Raimund Erbel, Cihangir Erem, Louise Eriksen, Johan G Eriksson, Jorge Escobedo-de la Peña, Saeid Eslami, Ali Esmaeili, Alun Evans, David Faeh, Albina A Fakhretdinova, Caroline H Fall, Elnaz Faramarzi, Mojtaba Farjam, Victoria Farrugia Sant'Angelo, Farshad Farzadfar, Mohammad Reza Fattahi, Asher Fawwad, Francisco J Felix-Redondo, Trevor S Ferguson, Romulo A Fernandes, Daniel Fernández-Bergés, Daniel Ferrante, Thomas Ferrao, Marika Ferrari, Marco M Ferrario, Catterina Ferreccio, Eldridge Ferrer, Jean Ferrieres, Thamara Hubler Figueiró, Anna Fijalkowska, Günther Fink, Krista Fischer, Bernhard Föger, Leng Huat Foo, Maria Forsner, Heba M Fouad, Damian K Francis, Maria do Carmo Franco, Oscar H Franco, Ruth Frikke-Schmidt, Guillermo Frontera, Flavio D Fuchs, Sandra C Fuchs, Isti I Fujiati, Yuki Fujita, Matsuda Fumihiko, Takuro Furusawa, Zbigniew Gaciong, Mihai Gafencu, Andrzej Galbarczyk, Henrike Galenkamp, Daniela Galeone, Myriam Galfo, Fabio Galvano, Jingli Gao, Manoli Garcia-de-la-Hera, Marta García-Solano, Dickman Gareta, Sarah P Garnett, Jean-Michel Gaspoz, Magda Gasull, Adroaldo Cesar Araujo Gaya, Anelise Reis Gaya, Andrea Gazzinelli, Ulrike Gehring, Harald Geiger, Johanna M Geleijnse, Ali Ghanbari, Erfan Ghasemi, Oana-Florentina Gheorghe-Fronea, Simona Giampaoli, Francesco Gianfagna, Tiffany K Gill, Jonathan Giovannelli, Glen Gironella, Aleksander Giwercman, Konstantinos Gkiouras, Justyna Godos, Sibel Gogen, Rebecca A Goldsmith, David Goltzman, Santiago F Gómez, Aleksandra Gomula, Bruna Goncalves Cordeiro da Silva, Helen Gonçalves, David A Gonzalez-Chica, Marcela Gonzalez-Gross, Margot González-Leon, Juan P González-Rivas, Clicerio González-Villalpando, María-Elena González-Villalpando, Angel R Gonzalez, Frederic Gottrand, Antonio Pedro Graça, Sidsel Graff-Iversen, Dušan Grafnetter, Aneta Grajda, Maria G Grammatikopoulou, Ronald D Gregor, Tomasz Grodzicki, Else Karin Grøholt, Anders Grøntved, Giuseppe Grosso, Gabriella Gruden, Dongfeng Gu, Emanuela Gualdi-Russo, Pilar Guallar-Castillón, Andrea Gualtieri, Elias F Gudmundsson, Vilmundur Gudnason, Ramiro Guerrero, Idris Guessous, Andre L Guimaraes, Martin C Gulliford, Johanna Gunnlaugsdottir, Marc J Gunter, Xiu-Hua Guo, Yin Guo, Prakash C Gupta, Rajeev Gupta, Oye Gureje, Beata Gurzkowska, Enrique Gutiérrez-González, Laura Gutierrez, Felix Gutzwiller, Seongjun Ha, Farzad Hadaegh, Charalambos A Hadjigeorgiou, Rosa Haghshenas, Hamid Hakimi, Jytte Halkjær, Ian R Hambleton, Behrooz Hamzeh, Dominique Hange, Abu AM Hanif, Sari Hantunen, Rachakulla Hari Kumar, Seyed Mohammad Hashemi-Shahri, Maria Hassapidou, Jun Hata, Teresa Haugsgjerd, Alison J Hayes, Jiang He, Yuan He, Yuna He, Regina Heidinger-Felso, Mirjam Heinen, Tatjana Hejgaard, Marleen Elisabeth Hendriks, Rafael dos Santos Henrique, Ana Henriques, Leticia Hernandez Cadena, Sauli Herrala, Victor M Herrera, Isabelle Herter-Aeberli, Ramin Heshmat, Allan G Hill, Sai Yin Ho, Suzanne C Ho, Michael Hobbs, Albert Hofman, Ingunn Holden Bergh, Michelle Holdsworth, Reza Homayounfar, Clara Homs, Wilma M Hopman, Andrea RVR Horimoto, Claudia M Hormiga, Bernardo L Horta, Leila Houti, Christina Howitt, Thein Thein Htay, Aung Soe Htet, Maung Maung Than Htike, Yonghua Hu, José María Huerta, Ilpo Tapani Huhtaniemi, Constanta Huidumac Petrescu, Abdullatif Husseini, Chinh Nguyen Huu, Inge Huybrechts, Nahla Hwalla, Jolanda Hyska, Licia Iacoviello, Jesús M Ibarluzea, Mohsen M Ibrahim, Norazizah Ibrahim Wong, Nayu Ikeda, M Arfan Ikram, Violeta Iotova, Vilma E Irazola, Takafumi Ishida, Muhammad Islam, Sheikh Mohammed Shariful Islam, Masanori Iwasaki, Rod T Jackson, Jeremy M Jacobs, Hashem Y Jaddou, Tazeen Jafar, Kenneth James, Kazi M Jamil, Konrad Jamrozik, Imre Janszky, Edward Janus, Juel Jarani, Marjo-Riitta Jarvelin, Grazyna Jasienska, Ana Jelakovic, Bojan Jelakovic, Garry Jennings, Anjani Kumar Jha, Chao Qiang Jiang, Ramon O Jimenez, Karl-Heinz Jöckel, Michel Joffres, Mattias Johansson, Jari J Jokelainen, Jost B Jonas, Torben Jørgensen, Pradeep Joshi, Farahnaz Joukar, Dragana P Jovic, Jacek J Józwiak, Anne Juolevi, Gregor Jurak, Iulia Jurca Simina, Vesna Juresa, Rudolf Kaaks, Felix O Kaducu, Anthony Kafatos, Eero O Kajantie, Zhanna Kalmatayeva, Ofra Kalter-Leibovici, Yves Kameli, Kodanda R Kanala, Srinivasan Kannan, Efthymios Kapantais, Khem B Karki, Marzieh Katibeh, Joanne Katz, Peter T Katzmarzyk, Jussi Kauhanen, Prabhdeep Kaur, Maryam Kavousi, Gyulli M Kazakbaeva, Ulrich Keil, Lital Keinan Boker, Sirkka Keinänen-Kiukaanniemi, Roya Kelishadi, Cecily Kelleher, Han CG Kemper, Andre P Kengne, Maryam Keramati, Alina Kerimkulova, Mathilde Kersting, Timothy Key, Yousef Saleh Khader, Davood Khalili, Young-Ho Khang, Kay-Tee Khaw, Bahareh Kheiri, Motahareh Kheradmand, Alireza Khosravi, Ilse MSL Khouw, Ursula Kiechl-Kohlendorfer, Stefan Kiechl, Japhet Killewo, Dong Wook Kim, Hyeon Chang Kim, Jeongseon Kim, Jenny M Kindblom, Heidi Klakk, Magdalena Klimek, Jeannette Klimont, Jurate Klumbiene, Michael Knoflach, Bhawesh Koirala, Elin Kolle, Patrick Kolsteren, Jürgen König, Raija Korpelainen, Paul Korrovits, Magdalena Korzycka, Jelena Kos, Seppo Koskinen, Katsuyasu Kouda, Viktoria A Kovacs, Sudhir Kowlessur, Slawomir Koziel, Wolfgang Kratzer, Susi Kriemler, Peter Lund Kristensen, Steiner Krokstad, Daan Kromhout, Branimir Krtalic, Herculina S Kruger, Ruzena Kubinova, Renata Kuciene, Urho M Kujala, Enisa Kujundzic, Zbigniew Kulaga, R Krishna Kumar, Marie Kunešová, Pawel Kurjata, Yadlapalli S Kusuma, Kari Kuulasmaa, Catherine Kyobutungi, Quang Ngoc La, Fatima Zahra Laamiri, Tiina Laatikainen, Carl Lachat, Youcef Laid, Tai Hing Lam, Christina-Paulina Lambrinou, Edwige Landais, Vera Lanska, Georg Lappas, Bagher Larijani, Tint Swe Latt, Laura Lauria, Avula Laxmaiah, Maria Lazo-Porras, Khanh Le Nguyen Bao, Agnès Le Port, Tuyen D Le, Jeannette Lee, Jeonghee Lee, Paul H Lee, Nils Lehmann, Terho Lehtimäki, Daniel Lemogoum, Naomi S Levitt, Yanping Li, Merike Liivak, Christa L Lilly, Wei-Yen Lim, M Fernanda Lima-Costa, Hsien-Ho Lin, Xu Lin, Yi-Ting Lin, Lars Lind, Allan Linneberg, Lauren Lissner, Mieczyslaw Litwin, Jing Liu, Lijuan Liu, Wei-Cheng Lo, Helle-Mai Loit, Khuong Quynh Long, Luis Lopes, Oscar Lopes, Esther Lopez-Garcia, Tania Lopez, Paulo A Lotufo, José Eugenio Lozano, Janice L Lukrafka, Dalia Luksiene, Annamari Lundqvist, Robert Lundqvist, Nuno Lunet, Charles Lunogelo, Michala Lustigová, Edyta Luszczki, Guansheng Ma, Jun Ma, Xu Ma, George LL Machado-Coelho, Aristides M Machado-Rodrigues, Suka Machi, Luisa M Macieira, Ahmed A Madar, Stefania Maggi, Dianna J Magliano, Sara Magnacca, Emmanuella Magriplis, Gowri Mahasampath, Bernard Maire, Marjeta Majer, Marcia Makdisse, Päivi Mäki, Fatemeh Malekzadeh, Reza Malekzadeh, Rahul Malhotra, Kodavanti Mallikharjuna Rao, Sofia K Malyutina, Lynell V Maniego, Yannis Manios, Jim I Mann, Fariborz Mansour-Ghanaei, Enzo Manzato, Paula Margozzini, Anastasia Markaki, Oonagh Markey, Eliza Markidou Ioannidou, Pedro Marques-Vidal, Larissa Pruner Marques, Jaume Marrugat, Yves Martin-Prevel, Rosemarie Martin, Reynaldo Martorell, Eva Martos, Stefano Marventano, Luis P Mascarenhas, Shariq R Masoodi, Ellisiv B Mathiesen, Prashant Mathur, Alicia Matijasevich, Tandi E Matsha, Christina Mavrogianni, Artur Mazur, Jean Claude N Mbanya, Shelly R McFarlane, Stephen T McGarvey, Martin McKee, Stela McLachlan, Rachael M McLean, Scott B McLean, Breige A McNulty, Sounnia Mediene-Benchekor, Jurate Medzioniene, Parinaz Mehdipour, Kirsten Mehlig, Amir Houshang Mehrparvar, Aline Meirhaeghe, Jørgen Meisfjord, Christa Meisinger, Ana Maria B Menezes, Geetha R Menon, Gert BM Mensink, Maria Teresa Menzano, Alibek Mereke, Indrapal I Meshram, Andres Metspalu, Jie Mi, Kim F Michaelsen, Nathalie Michels, Kairit Mikkel, Karolina Milkowska, Jody C Miller, Cláudia S Minderico, GK Mini, Juan Francisco Miquel, J Jaime Miranda, Mohammad Reza Mirjalili, Daphne Mirkopoulou, Erkin Mirrakhimov, Marjeta Mišigoj-Durakovic, Antonio Mistretta, Veronica Mocanu, Pietro A Modesti, Sahar Saeedi Moghaddam, Bahram Mohajer, Mostafa K Mohamed, Shukri F Mohamed, Kazem Mohammad, Zahra Mohammadi, Noushin Mohammadifard, Reza Mohammadpourhodki, Viswanathan Mohan, Salim Mohanna, Muhammad Fadhli Mohd Yusoff, Iraj Mohebbi, Farnam Mohebi, Marie Moitry, Drude Molbo, Line T Møllehave, Niels C Møller, Dénes Molnár, Amirabbas Momenan, Charles K Mondo, Michele Monroy-Valle, Eric Monterrubio-Flores, Kotsedi Daniel K Monyeki, Jin Soo Moon, Mahmood Moosazadeh, Leila B Moreira, Alain Morejon, Luis A Moreno, Karen Morgan, Suzanne N Morin, Erik Lykke Mortensen, George Moschonis, Malgorzata Mossakowska, Aya Mostafa, Anabela Mota-Pinto, Jorge Mota, Mohammad Esmaeel Motlagh, Jorge Motta, Marcos André Moura-dos-Santos, Malay K Mridha, Kelias P Msyamboza, Thet Thet Mu, Magdalena Muc, Boban Mugoša, Maria L Muiesan, Parvina Mukhtorova, Martina Müller-Nurasyid, Neil Murphy, Jaakko Mursu, Elaine M Murtagh, Kamarul Imran Musa, Sanja Music Milanovic, Vera Musil, Norlaila Mustafa, Iraj Nabipour, Shohreh Naderimagham, Gabriele Nagel, Balkish M Naidu, Farid Najafi, Harunobu Nakamura, Jana Námešná, Ei Ei K Nang, Vinay B Nangia, Martin Nankap, Sameer Narake, Paola Nardone, Matthias Nauck, William A Neal, Azim Nejatizadeh, Keiu Nelis, Liis Nelis, Ilona Nenko, Martin Neovius, Flavio Nervi, Chung T Nguyen, D Nguyen, Quang Ngoc Nguyen, Ramfis E Nieto-Martínez, Yury P Nikitin, Guang Ning, Toshiharu Ninomiya, Sania Nishtar, Marianna Noale, Oscar A Noboa, Helena Nogueira, Teresa Norat, Maria Nordendahl, Børge G Nordestgaard, Davide Noto, Natalia Nowak-Szczepanska, Mohannad Al Nsour, Irfan Nuhoglu, Eha Nurk, Terence W O'Neill, Dermot O'Reilly, Galina Obreja, Caleb Ochimana, Angélica M Ochoa-Avilés, Eiji Oda, Kyungwon Oh, Kumiko Ohara, Claes Ohlsson, Ryutaro Ohtsuka, Örn Olafsson, Maria Teresa A Olinto, Isabel O Oliveira, Mohd Azahadi Omar, Altan Onat, Sok King Ong, Lariane M Ono, Pedro Ordunez, Rui Ornelas, Ana P Ortiz, Pedro J Ortiz, Merete Osler, Clive Osmond, Sergej M Ostojic, Afshin Ostovar, Johanna A Otero, Kim Overvad, Ellis Owusu-Dabo, Fred Michel Paccaud, Cristina Padez, Ioannis Pagkalos, Elena Pahomova, Karina Mary de Paiva, Andrzej Pajak, Domenico Palli, Alberto Palloni, Luigi Palmieri, Wen-Harn Pan, Songhomitra Panda-Jonas, Arvind Pandey, Francesco Panza, Dimitrios Papandreou, Soon-Woo Park, Suyeon Park, Winsome R Parnell, Mahboubeh Parsaeian, Ionela M Pascanu, Patrick Pasquet, Nikhil D Patel, Mangesh S Pednekar, Nasheeta Peer, Sergio Viana Peixoto, Markku Peltonen, Alexandre C Pereira, Marco A Peres, Napoleón Pérez-Farinós, Cynthia M Pérez, Valentina Peterkova, Annette Peters, Astrid Petersmann, Janina Petkeviciene, Ausra Petrauskiene, Emanuela Pettenuzzo, Niloofar Peykari, Son Thai Pham, Rafael N Pichardo, Daniela Pierannunzio, Iris Pigeot, Hynek Pikhart, Aida Pilav, Lorenza Pilotto, Francesco Pistelli, Freda Pitakaka, Aleksandra Piwonska, Andreia N Pizarro, Pedro Plans-Rubió, Bee Koon Poh, Hermann Pohlabeln, Raluca M Pop, Stevo R Popovic, Miquel Porta, Georg Posch, Anil Poudyal, Dimitrios Poulimeneas, Hamed Pouraram, Farhad Pourfarzi, Akram Pourshams, Hossein Poustchi, Rajendra Pradeepa, Alison J Price, Jacqueline F Price, Rui Providencia, Jardena J Puder, Iveta Pudule, Soile E Puhakka, Maria Puiu, Margus Punab, Radwan F Qasrawi, Mostafa Qorbani, Tran Quoc Bao, Ivana Radic, Ricardas Radisauskas, Salar Rahimikazerooni, Mahfuzar Rahman, Mahmudur Rahman, Olli Raitakari, Manu Raj, Ellina Rakhimova, Sherali Rakhmatulloev, Ivo Rakovac, Sudha Ramachandra Rao, Ambady Ramachandran, Jacqueline Ramke, Elisabete Ramos, Rafel Ramos, Lekhraj Rampal, Sanjay Rampal, Vayia Rarra, Ramon A Rascon-Pacheco, Mette Rasmussen, Cassiano Ricardo Rech, Josep Redon, Paul Ferdinand M Reganit, Valéria Regecová, Luis Revilla, Abbas Rezaianzadeh, Lourdes Ribas-Barba, Robespierre Ribeiro, Elio Riboli, Adrian Richter, Fernando Rigo, Natascia Rinaldo, Tobias F Rinke de Wit, Ana Rito, Raphael M Ritti-Dias, Juan A Rivera, Cynthia Robitaille, Romana Roccaldo, Daniela Rodrigues, Fernando Rodríguez-Artalejo, María del Cristo Rodriguez-Perez, Laura A Rodríguez-Villamizar, Ulla Roggenbuck, Rosalba Rojas-Martinez, Nipa Rojroongwasinkul, Dora Romaguera, Elisabetta L Romeo, Rafaela V Rosario, Annika Rosengren, Ian Rouse, Joel GR Roy, Adolfo Rubinstein, Frank J Rühli, Jean-Bernard Ruidavets, Blanca Sandra Ruiz-Betancourt, Emma Ruiz Moreno, Iuliia A Rusakova, Kenisha Russell Jonsson, Paola Russo, Petra Rust, Marcin Rutkowski, Charumathi Sabanayagam, Elena Sacchini, Harshpal S Sachdev, Alireza Sadjadi, Ali Reza Safarpour, Sare Safi, Saeid Safiri, Olfa Saidi, Nader Saki, Benoit Salanave, Eduardo Salazar Martinez, Diego Salmerón, Veikko Salomaa, Jukka T Salonen, Massimo Salvetti, Margarita Samoutian, Jose Sánchez-Abanto, Sandjaja, Susana Sans, Loreto Santa Marina, Diana A Santos, Ina S Santos, Lèlita C Santos, Maria Paula Santos, Osvaldo Santos, Rute Santos, Sara Santos Sanz, Jouko L Saramies, Luis B Sardinha, Nizal Sarrafzadegan, Thirunavukkarasu Sathish, Kai-Uwe Saum, Savvas Savva, Mathilde Savy, Norie Sawada, Mariana Sbaraini, Marcia Scazufca, Beatriz D Schaan, Angelika Schaffrath Rosario, Herman Schargrodsky, Anja Schienkiewitz, Karin Schindler, Sabine Schipf, Carsten O Schmidt, Ida Maria Schmidt, Peter Schnohr, Ben Schöttker, Sara Schramm, Stine Schramm, Helmut Schröder, Constance Schultsz, Aletta E Schutte, Sylvain Sebert, Aye Aye Sein, Rusidah Selamat, Vedrana Sember, Abhijit Sen, Idowu O Senbanjo, Sadaf G Sepanlou, Victor Sequera, Luis Serra-Majem, Jennifer Servais, Ludmila Ševcíková, Svetlana A Shalnova, Teresa Shamah-Levy, Morteza Shamshirgaran, Coimbatore Subramaniam Shanthirani, Maryam Sharafkhah, Sanjib K Sharma, Jonathan E Shaw, Amaneh Shayanrad, Ali Akbar Shayesteh, Lela Shengelia, Zumin Shi, Kenji Shibuya, Hana Shimizu-Furusawa, Dong Wook Shin, Youchan Shin, Majid Shirani, Rahman Shiri, Namuna Shrestha, Khairil Si-Ramlee, Alfonso Siani, Rosalynn Siantar, Abla M Sibai, Antonio M Silva, Diego Augusto Santos Silva, Mary Simon, Judith Simons, Leon A Simons, Agneta Sjöberg, Michael Sjöström, Gry Skodje, Jolanta Slowikowska-Hilczer, Przemyslaw Slusarczyk, Liam Smeeth, Hung-Kwan So, Fernanda Cunha Soares, Grzegorz Sobek, Eugène Sobngwi, Morten Sodemann, Stefan Söderberg, Moesijanti YE Soekatri, Agustinus Soemantri, Reecha Sofat, Vincenzo Solfrizzi, Mohammad Hossein Somi, Emily Sonestedt, Yi Song, Thorkild IA Sørensen, Elin P Sørgjerd, Maroje Soric, Charles Sossa Jérome, Victoria E Soto-Rojas, Aïcha Soumaré, Slavica Sovic, Bente Sparboe-Nilsen, Karen Sparrenberger, Angela Spinelli, Igor Spiroski, Jan A Staessen, Hanspeter Stamm, Gregor Starc, Maria G Stathopoulou, Kaspar Staub, Bill Stavreski, Jostein Steene-Johannessen, Peter Stehle, Aryeh D Stein, George S Stergiou, Jochanan Stessman, Ranko Stevanovic, Jutta Stieber, Doris Stöckl, Tanja Stocks, Jakub Stokwiszewski, Ekaterina Stoyanova, Gareth Stratton, Karien Stronks, Maria Wany Strufaldi, Lela Sturua, Ramón Suárez-Medina, Machi Suka, Chien-An Sun, Johan Sundström, Yn-Tz Sung, Jordi Sunyer, Paibul Suriyawongpaisal, Boyd A Swinburn, Rody G Sy, Holly E Syddall, René Charles Sylva, Moyses Szklo, Lucjan Szponar, E Shyong Tai, Mari-Liis Tammesoo, Abdonas Tamosiunas, Eng Joo Tan, Xun Tang, Frank Tanser, Yong Tao, Mohammed Rasoul Tarawneh, Jakob Tarp, Carolina B Tarqui-Mamani, Radka Taxová Braunerová, Anne Taylor, Julie Taylor, Félicité Tchibindat, William R Tebar, Grethe S Tell, Tania Tello, KR Thankappan, Holger Theobald, Xenophon Theodoridis, Lutgarde Thijs, Nihal Thomas, Betina H Thuesen, Lubica Tichá, Erik J Timmermans, Anne Tjonneland, Hanna K Tolonen, Janne S Tolstrup, Murat Topbas, Roman Topór-Madry, Liv Elin Torheim, María José Tormo, Michael J Tornaritis, Maties Torrent, Laura Torres-Collado, Stefania Toselli, Pierre Traissac, Thi Tuyet-Hanh Tran, Dimitrios Trichopoulos, Antonia Trichopoulou, Oanh TH Trinh, Atul Trivedi, Lechaba Tshepo, Maria Tsigga, Shoichiro Tsugane, Azaliia M Tuliakova, Marshall K Tulloch-Reid, Fikru Tullu, Tomi-Pekka Tuomainen, Jaakko Tuomilehto, Maria L Turley, Per Tynelius, Themistoklis Tzotzas, Christophe Tzourio, Peter Ueda, Eunice Ugel, Flora AM Ukoli, Hanno Ulmer, Belgin Unal, Zhamyila Usupova, Hannu MT Uusitalo, Nalan Uysal, Justina Vaitkeviciute, Gonzalo Valdivia, Susana Vale, Damaskini Valvi, Rob M van Dam, Johan Van der Heyden, Yvonne T van der Schouw, Koen Van Herck, Hoang Van Minh, Irene GM van Valkengoed, Dirk Vanderschueren, Diego Vanuzzo, Anette Varbo, Gregorio Varela-Moreiras, Patricia Varona-Pérez, Senthil K Vasan, Tomas Vega, Toomas Veidebaum, Gustavo Velasquez-Melendez, Biruta Velika, Giovanni Veronesi, WM Monique Verschuren, Cesar G Victora, Giovanni Viegi, Lucie Viet, Salvador Villalpando, Paolo Vineis, Jesus Vioque, Jyrki K Virtanen, Marjolein Visser, Sophie Visvikis-Siest, Bharathi Viswanathan, Mihaela Vladulescu, Tiina Vlasoff, Dorja Vocanec, Henry Völzke, Ari Voutilainen, Sari Voutilainen, Martine Vrijheid, Tanja GM Vrijkotte, Alisha N Wade, Aline Wagner, Thomas Waldhör, Janette Walton, Elvis OA Wambiya, Wan Mohamad Wan Bebakar, Wan Nazaimoon Wan Mohamud, Rildo de Souza Wanderley Júnior, Ming-Dong Wang, Ningli Wang, Qian Wang, Xiangjun Wang, Ya Xing Wang, Ying-Wei Wang, S Goya Wannamethee, Nicholas Wareham, Adelheid Weber, Niels Wedderkopp, Deepa Weerasekera, Daniel Weghuber, Wenbin Wei, Aneta Weres, Bo Werner, Peter H Whincup, Kurt Widhalm, Indah S Widyahening, Andrzej Wiecek, Rainford J Wilks, Johann Willeit, Peter Willeit, Julianne Williams, Tom Wilsgaard, Bogdan Wojtyniak, Roy A Wong-McClure, Andrew Wong, Jyh Eiin Wong, Tien Yin Wong, Jean Woo, Mark Woodward, Frederick C Wu, Jianfeng Wu, Li Juan Wu, Shouling Wu, Haiquan Xu, Liang Xu, Nor Azwany Yaacob, Uruwan Yamborisut, Weili Yan, Ling Yang, Xiaoguang Yang, Yang Yang, Nazan Yardim, Mehdi Yaseri, Tabara Yasuharu, Xingwang Ye, Panayiotis K Yiallouros, Moein Yoosefi, Akihiro Yoshihara, Qi Sheng You, San-Lin You, Novie O Younger-Coleman, Safiah Md Yusof, Ahmad Faudzi Yusoff, Luciana Zaccagni, Vassilis Zafiropulos, Ahmad A Zainuddin, Seyed Rasoul Zakavi, Farhad Zamani, Sabina Zambon, Antonis Zampelas, Hana Zamrazilová, Maria Elisa Zapata, Abdul Hamid Zargar, Ko Ko Zaw, Tomasz Zdrojewski, Tajana Zeljkovic Vrkic, Yi Zeng, Luxia Zhang, Zhen-Yu Zhang, Dong Zhao, Ming-Hui Zhao, Wenhua Zhao, Shiqi Zhen, Wei Zheng, Yingfeng Zheng, Bekbolat Zholdin, Maigeng Zhou, Dan Zhu, Yanina Zocalo, Julio Zuñiga Cisneros, Monika Zuziak, Majid Ezzati

## Abstract

**Background:**

Comparable global data on health and nutrition of school-aged children and adolescents are scarce. We aimed to estimate age trajectories and time trends in mean height and mean body-mass index (BMI), which measures weight gain beyond what is expected from height gain, for school-aged children and adolescents.

**Methods:**

For this pooled analysis, we used a database of cardiometabolic risk factors collated by the Non-Communicable Disease Risk Factor Collaboration. We applied a Bayesian hierarchical model to estimate trends from 1985 to 2019 in mean height and mean BMI in 1-year age groups for ages 5–19 years. The model allowed for non-linear changes over time in mean height and mean BMI and for non-linear changes with age of children and adolescents, including periods of rapid growth during adolescence.

**Findings:**

We pooled data from 2181 population-based studies, with measurements of height and weight in 65 million participants in 200 countries and territories. In 2019, we estimated a difference of 20 cm or higher in mean height of 19-year-old adolescents between countries with the tallest populations (the Netherlands, Montenegro, Estonia, and Bosnia and Herzegovina for boys; and the Netherlands, Montenegro, Denmark, and Iceland for girls) and those with the shortest populations (Timor-Leste, Laos, Solomon Islands, and Papua New Guinea for boys; and Guatemala, Bangladesh, Nepal, and Timor-Leste for girls). In the same year, the difference between the highest mean BMI (in Pacific island countries, Kuwait, Bahrain, The Bahamas, Chile, the USA, and New Zealand for both boys and girls and in South Africa for girls) and lowest mean BMI (in India, Bangladesh, Timor-Leste, Ethiopia, and Chad for boys and girls; and in Japan and Romania for girls) was approximately 9–10 kg/m^2^. In some countries, children aged 5 years started with healthier height or BMI than the global median and, in some cases, as healthy as the best performing countries, but they became progressively less healthy compared with their comparators as they grew older by not growing as tall (eg, boys in Austria and Barbados, and girls in Belgium and Puerto Rico) or gaining too much weight for their height (eg, girls and boys in Kuwait, Bahrain, Fiji, Jamaica, and Mexico; and girls in South Africa and New Zealand). In other countries, growing children overtook the height of their comparators (eg, Latvia, Czech Republic, Morocco, and Iran) or curbed their weight gain (eg, Italy, France, and Croatia) in late childhood and adolescence. When changes in both height and BMI were considered, girls in South Korea, Vietnam, Saudi Arabia, Turkey, and some central Asian countries (eg, Armenia and Azerbaijan), and boys in central and western Europe (eg, Portugal, Denmark, Poland, and Montenegro) had the healthiest changes in anthropometric status over the past 3·5 decades because, compared with children and adolescents in other countries, they had a much larger gain in height than they did in BMI. The unhealthiest changes—gaining too little height, too much weight for their height compared with children in other countries, or both—occurred in many countries in sub-Saharan Africa, New Zealand, and the USA for boys and girls; in Malaysia and some Pacific island nations for boys; and in Mexico for girls.

**Interpretation:**

The height and BMI trajectories over age and time of school-aged children and adolescents are highly variable across countries, which indicates heterogeneous nutritional quality and lifelong health advantages and risks.

**Funding:**

Wellcome Trust, AstraZeneca Young Health Programme, EU.

## Introduction

Growth and development through childhood and adolescence are affected by social, nutritional, and environmental factors at home, at school, and in the community. During school ages (typically 5–19 years), these factors amplify or mitigate adversity in infancy and early childhood and, if healthy, can help consolidate gains from early childhood and correct some nutritional inadequacies and imbalances.^1^, ^2^, ^3^ Therefore, investing in the nutrition of school-aged children and adolescents is crucial for a healthy transition to adulthood.

**Research in context**

**Evidence before this study**

We searched MEDLINE (through PubMed) for articles published from inception up to Aug 2, 2020, with no language restrictions, using the following search terms: (“body size”[mh:noexp] OR “body height”[mh:noexp] OR “body weight”[mh:noexp] OR “birth weight”[mh:noexp] OR “overweight”[mh:noexp] OR “obesity”[mh] OR “thinness”[mh:noexp] OR “Waist-Hip Ratio”[mh:noexp] OR “Waist Circumference”[mh:noexp] OR “body mass index”[mh:noexp]) AND (“Humans”[mh]) AND (“Health Surveys”[mh] OR “Epidemiological Monitoring”[mh] OR “Prevalence”[mh]) NOT Comment[ptyp] NOT Case Reports[ptyp]. Articles were screened according to the inclusion and exclusion criteria described in the Methods section. We found global or multicountry studies on trends over time in height for adults and for children younger than 5 years, but not for school-aged children and adolescents. One multicountry study used cross-sectional height data in 53 community-based samples and reported height differences in children aged 10–17 years. We found three studies on trends in body-mass index (BMI) or overweight in children and adolescents, but only one of these studies separately reported trends for children aged 5–19 years. We found multiple studies in individual or small groups of countries on trends in height, BMI, or both. In terms of considering combined changes in height and BMI, the *Lancet* Series on the double burden of malnutrition used data on stunting in children younger than 5 years together with data on various measures of underweight and overweight at different ages, but did not have data on height in older children and adolescents, nor did it analyse trends.

**Added value of this study**

To our knowledge, this study presents the first comparable estimates of height in school-aged children and adolescents for all countries in the world and does so alongside estimates of BMI, which together are pathways from nutrition and environment during childhood and adolescence to lifelong health. We also analysed age trajectories of mean height and BMI to investigate ages when growth in different countries was more versus less healthy and to identify the need for intervention.

**Implications of all the available evidence**

Age trajectories and time trends in mean height and BMI of school-aged children and adolescents were highly variable across countries and indicated heterogeneous nutritional quality and life-long health advantages and risks. Global and national nutrition and health programmes should extend to children and adolescents in school years to consolidate gains in children younger than 5 years and enable healthy growth through the entire developmental period.

Height and body-mass index (BMI) are anthropometric measures of the quality of nutrition and healthiness of the living environment during childhood and adolescence and are highly predictive of health and developmental outcomes throughout life.^4^, ^5^, ^6^, ^7^ Having low height and excessively low weight for one's height, represented by low BMI, increases the risk of morbidity and mortality, impairs cognitive development, and reduces educational performance and work productivity in later life.^4^, ^5^, ^7^ High BMI is associated with higher risk of disability and premature death in adulthood and with poor mental health and educational outcomes.^6^, ^8^

Much of global health and nutrition research and policy has focused on the period from preconception to age 5 years.^9^, ^10^ For school-aged children and adolescents, global information is available only for BMI^11^ and, to our knowledge, no study has reported global trends in height for these ages. In this study, we present consistent and comparable global estimates of height and BMI for school-aged children from 1985 to 2019 and assess how countries perform in terms of children and adolescents growing taller without excessive weight gain. We also evaluate height and BMI trajectories by age to understand when growth is more or less healthy and to identify the need for intervention.

## Methods

### Data sources

For this pooled analysis, we used a database of cardiometabolic risk factors collated by the Non-Communicable Disease Risk Factor Collaboration (NCD-RisC). The database and its criteria for data inclusion and exclusion are described in the appendix (pp 39–42). We used data from the NCD-RisC database from 1985 to 2019 for analysis of BMI and from 1971 to 2019 for analysis of height. Children aged 5 years in data from 1971 were born in 1966, and hence were 19 years old in 1985, as were children aged 6 years in data from 1972 through to 19-year-old adolescents in data from 1985. Additionally, for analysis of height, participants aged 20–30 years were included and assigned to their corresponding birth cohort, because mean height in these ages would be at least that when they were aged 19 years, given that the decline of height with age begins in the third and fourth decades of life. The inclusion of data from different years provided multiple observations of each birth cohort during their life course, which in turn helped to estimate the relevant parameters in the height model that used birth year as its time scale. A list of the data sources we used in this analysis and their characteristics is provided in the appendix (pp 49–89).

### Primary outcomes

Our primary outcomes were population mean height and mean BMI from ages 5 to 19 years. BMI accounts for the weight gain that is simply due to becoming taller, and hence measures being underweight or overweight for a person's height. When presenting results, we refer to gains in height as a healthy trend because the relationship between height and health is positive and continuous. We refer to BMI gain as unhealthy except in countries where mean BMI was more than 1 SD lower than the median of the WHO reference (ie, lower than 18·7 kg/m^2^ for girls and 19·6 kg/m^2^ for boys at age 19 years). We also compared mean height and BMI with the median of the WHO growth reference^12^ (appendix pp 90–93) at each age from 5 to 19 years. We used the WHO reference because it provides growth curves for both height and BMI and is used for monitoring in most countries. We started our analysis from age 5 years because children enter school at or around this age, and their nutrition, physical activity, and health are influenced by food and environment at their homes, schools, and communities.

### Statistical analysis

We used a Bayesian hierarchical model to estimate mean height and mean BMI by country, year, sex, and age. The model is described in detail in a statistical paper^13^ and related substantive papers^11^, ^14^ and is summarised in the appendix (pp 43–45). Briefly, the model had a hierarchical structure in which estimates for each country and year were informed by its own data, if available, and by data from other years in the same country and from other countries, especially those in the same region and super-region, with data for similar time periods. The extent to which estimates for each country-year were influenced by data from other years and other countries depended on whether the country had data, the sample size of the data, whether they were national, and the within-country and within-region variability of the available data.

The model allowed for non-linear time trends and non-linear changes in mean height and BMI with age, including periods of rapid growth during puberty, and the earlier age of these growth spurts in girls than in boys. We used observation year—the year in which data were collected—as the time scale for the analysis of BMI and birth year as the time scale for the analysis of height, consistent with previous analyses.^11^, ^14^ For BMI, substantial societal changes that affect nutrition and physical activity might affect children of different ages simultaneously, whereas for height, these effects accumulate in each birth cohort and a cohort's height-for-age monotonically increases from childhood to late adolescence.

The computer code for the model is available online, as are our country and regional estimates both in numerical format and as interactive visualisations. All analyses were done with R (version 3.5.1).

### Role of the funding source

The funders of the study had no role in study design, data collection, analysis, interpretation, or writing of the paper. Country and Regional Data Group members, ARM, BZ, and MS had full access to the data in the study. The corresponding author had final responsibility for the decision to submit for publication.

## Results

We pooled 2181 population-based measurement surveys and studies, with anthropometric measurements on 50 million people aged 5–19 years and 15 million people aged 20–30 years. We used at least one data source for 193 of 200 countries and territories for which estimates were made, covering 98·7% of the world's population in 2019 (appendix p 94–95), and at least two data sources for 177 countries, covering 98·0% of the world's population. Of these 2181 data sources, 1289 (59·1%) were sampled from national populations, 360 (16·5%) covered one or more subnational regions, and the remaining 532 (24·4%) were from one or a small number of communities. Regionally, data availability ranged from approximately three data sources per country in Oceania to approximately 46 sources per country in the high-income Asia-Pacific region.

**Figure 1:**
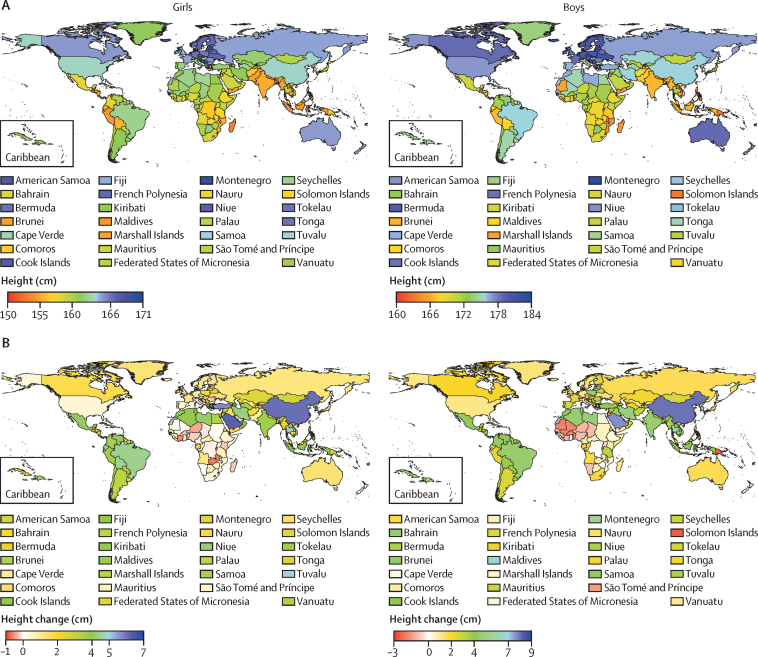
Height and height change by country and territory (A) Mean height of 19-year-olds in 2019. (B) Change in mean height of 19-year-olds from 1985 to 2019.

**Figure 2:**
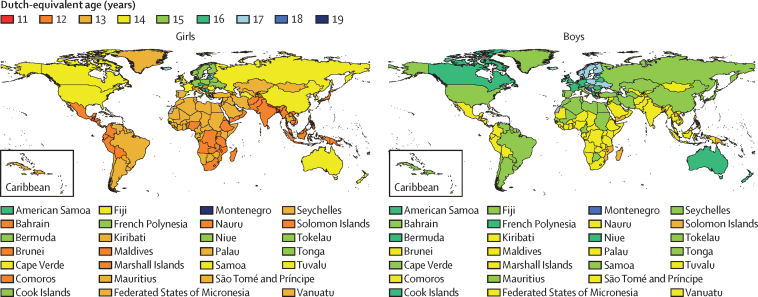
Growth gap for 19-year-olds in 2019 by country and territory The growth gap is the difference between 19 years and the age at which a Dutch girl or boy, who had the highest height in the world, achieved the height of 19-year-olds in different countries.

In 2019, the 19-year-olds who were on average the tallest in the world lived in northwestern and central European countries: the Netherlands (mean height 183·8 cm, 95% credible interval [CrI] 181·5–186·2), followed by Montenegro, Estonia, and Bosnia and Herzegovina for boys; and the Netherlands (170·4 cm, 168·3–172·4), followed by Montenegro, Denmark, and Iceland for girls (figure 1A). The 19-year-olds who were on average the shortest in 2019 lived in south and southeast Asia, Latin America, and east Africa: Timor-Leste (160·1 cm, 158·0–162·2), followed by Laos, Solomon Islands, and Papua New Guinea for boys; and Guatemala (150·9 cm; 149·4–152·4), followed by Bangladesh, Nepal, and Timor-Leste for girls. The 20 cm or higher difference between countries with the tallest and shortest mean height represents approximately 8 years of growth gap for girls and approximately 6 years for boys. For example, 19-year-old girls in four countries (Guatemala, Bangladesh, Nepal, and Timor-Leste) had the same mean height as that of 11-year-old Dutch girls, and those in another 53 countries—such as Burundi, India, Indonesia, Laos, Pakistan, Peru, the Philippines, and Yemen—had the same mean height as that of 12-year-old Dutch girls (figure 2). Similarly, 19-year-old boys in 11 countries throughout Asia, Latin America, and sub-Saharan Africa had the same mean height as that of Dutch boys aged 13 years.

Although northwestern European children and adolescents were on average the tallest in the world in 2019, much of this advantage was achieved before the late 20th century, and many of these countries had below median height change from 1985 to 2019 (figure 1B, appendix pp 96–296). By contrast, central European countries such as Montenegro and Poland achieved a substantial part of their height advantage since 1985, especially in boys. However, the largest gains in height over the past 3·5 decades were those in some emerging economies, including China (largest gain for boys and third largest for girls) and South Korea (third largest for boys and second largest for girls), and through parts of southeast Asia, the Middle East and north Africa, and Latin America and the Caribbean. Nonetheless, how much mean height changed from 1985 to 2019 varied substantially, even within this group of countries. For example, gains in mean height at age 19 years in China were larger than in India by 3·5 cm (95% CrI 1·8–5·1) for boys and 2·3 cm (0·9–3·7) for girls. By contrast with emerging economies, the height of children and adolescents, especially boys, has on average stagnated or become shorter since 1985 in many countries in sub-Saharan Africa.^10^

**Figure 3:**
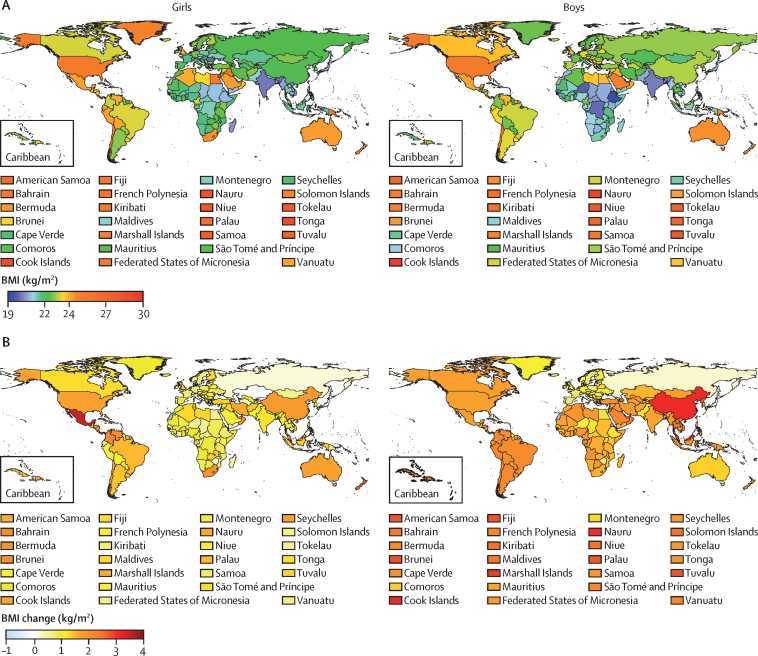
BMI and BMI change by country and territory (A) Mean BMI of 19-year-olds in 2019. (B) Change in mean BMI of 19-year-olds from 1985 to 2019. BMI=body-mass index.

Pacific island countries in Oceania had the highest mean BMI in the world in 2019, surpassing 28 kg/m^2^ for 19-year-olds in many of these nations (figure 3A). Late-adolescence BMI was also high for boys and girls in Middle Eastern and north African countries such as Kuwait and Bahrain; in Caribbean islands such as the Bahamas; in Chile, the USA, and New Zealand; and, for girls, in South Africa. The mean BMI of 19-year-old boys and girls was lowest (approximately 21 kg/m^2^ or lower) in countries in south Asia (eg, India and Bangladesh), southeast Asia (eg, Timor-Leste), and east and central Africa (eg, Ethiopia and Chad), as was it for 19-year-old girls in Japan and some central European countries (eg, Romania and Bosnia and Herzegovina). The highest and lowest worldwide BMIs were approximately 9–10 kg/m^2^ apart, equivalent to about 25 kg of weight.

Change in late-adolescence BMI from 1985 to 2019 ranged from small changes (less than 0·5 kg/m^2^) in both sexes in Japan and some European countries (eg, Italy, Russia, and Denmark) and, for girls, in some central Asian (eg, Armenia) and sub-Saharan African countries, to increases higher than 3 kg/m^2^ in Malaysia and some countries in Oceania for both sexes, in China for boys, and in Mexico for girls (figure 3B).

**Figure 4:**
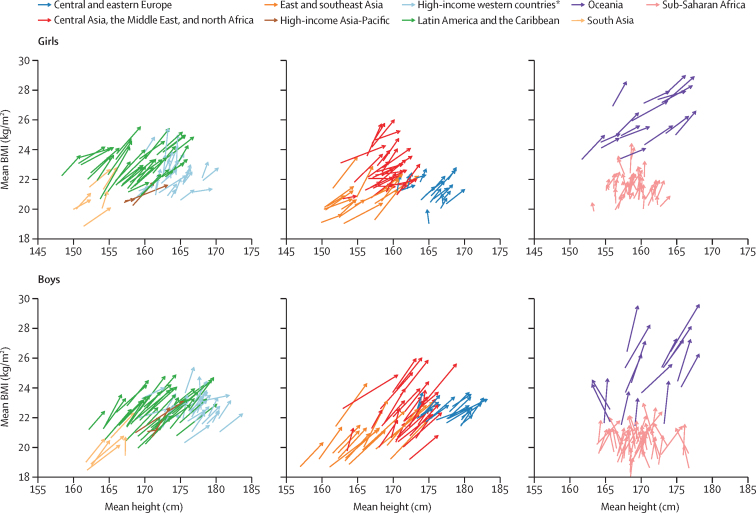
Combined change from 1985 to 2019 in mean height and mean BMI of 19-year-olds Each arrow shows one country. For each country, the arrow begins at mean height and BMI values in 1985 and ends in mean height and BMI values in 2019. Each arrow colour refers to countries in one region. BMI=body-mass index. *Countries in northwestern Europe, southwestern Europe, and English-speaking high-income countries (Australia, Canada, Ireland, New Zealand, the UK and the USA).

From 1985 to 2019, 19-year-old girls in some countries in central Asia (eg, Armenia and Azerbaijan) and 19-year-old boys in some European countries (eg, Portugal, Denmark, Poland, and Montenegro) had moderate-to-large gains in height alongside small or no increases in BMI (figure 4). Meanwhile, children grew much taller in some countries (eg, girls in South Korea, Turkey, Vietnam, and Saudi Arabia), while their BMI increased about the same as the global median. Both these trends were healthier than those of boys and girls in much of sub-Saharan Africa and in New Zealand and the USA, boys in Malaysia and some countries in Oceania, and girls in Mexico, where little or no height gain occurred, much larger weight was gained, or both, relative to other countries.

**Figure 5:**
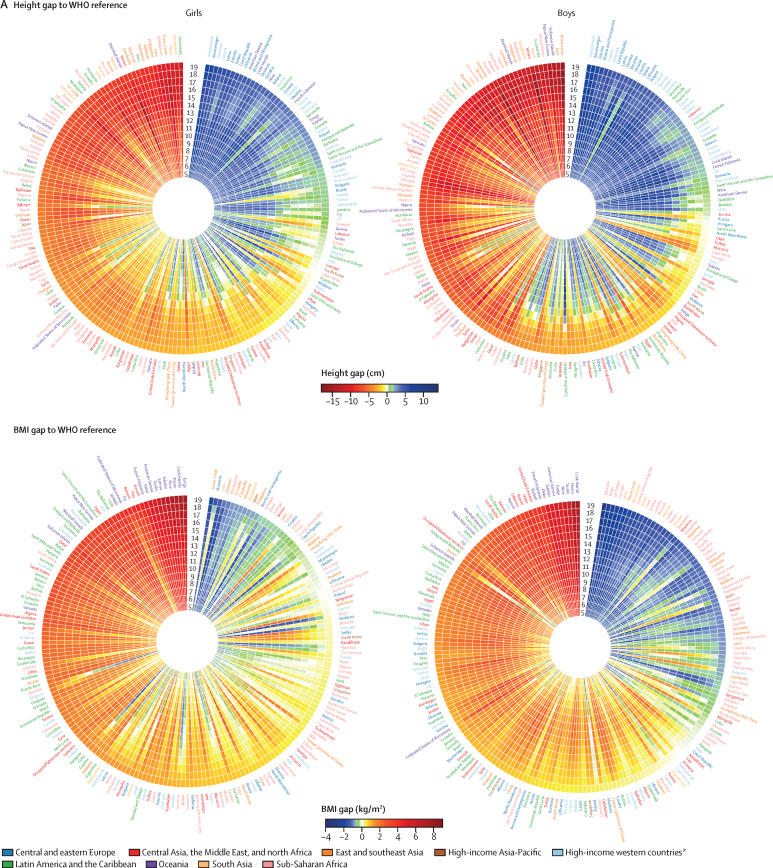

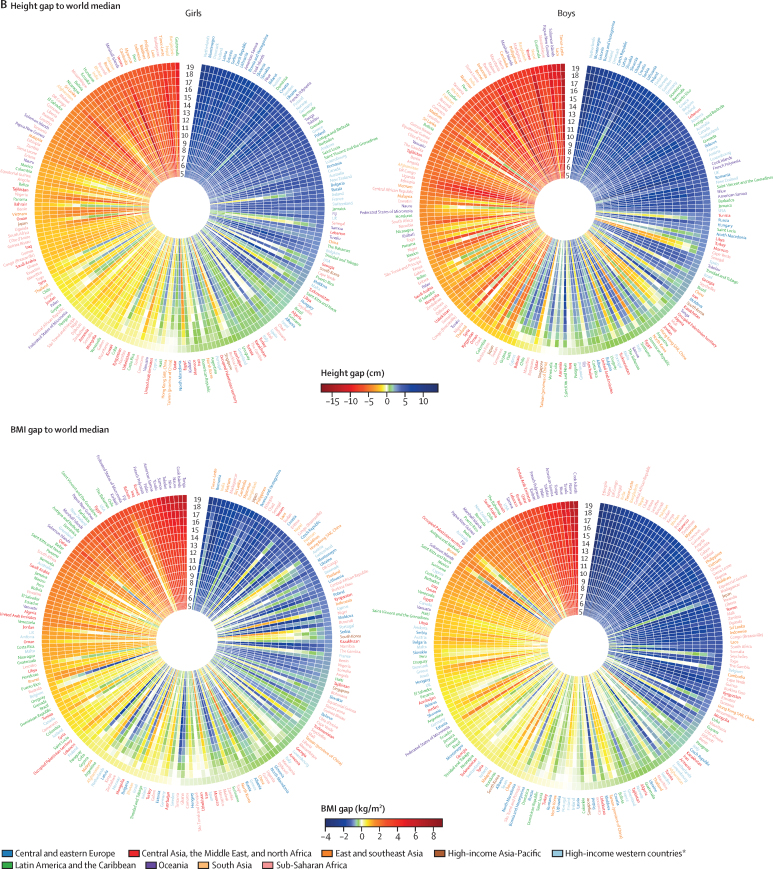
Age trajectory of height and BMI for 19-year-olds in 2019 Mean height and BMI of 19-year-olds in 2019 (ie, those born in 2000) at each age from 5 to 19 years compared with the median of the WHO growth reference^12^ (A) and the world median (B). Each cell represents the difference between the height or BMI of children and adolescents in one country and the median value for a given age of the WHO growth reference (A) and all countries (B). Countries are ordered by decreasing height or increasing BMI in adolescents at age 19 years in 2019. The median of the WHO growth reference and world median are presented in the appendix (pp 90–93). Results reported as Z scores of the WHO growth reference are presented in the appendix (pp 297–98). A comparison of height and BMI gap between boys and girls is presented in the appendix (pp 299–300). BMI=body-mass index. SAR=Special Administrative Region. *Countries in northwestern Europe, southwestern Europe, and English-speaking high-income countries (Australia, Canada, Ireland, New Zealand, the UK and the USA).

Boys born in 2000 (ie, who were aged 19 years in 2019) gained from 53·4 cm to 71·3 cm of height from their 5th to 19th birthday in different countries (appendix pp 96–296); for girls born in the same year, height gain from their 5th to 19th birthday ranged from 43·8 cm to 55·5 cm in different countries. We compared the mean height and mean BMI of children born in 2000 in each country with the median of the respective WHO growth reference^12^ at each age from 5 to 19 years (figure 5A). This comparison showed that, in many countries, mean height throughout late childhood and adolescence was lower than the median of the WHO growth reference (figure 5A, appendix pp 297–98). Exceptions to this pattern were much of Europe and a few countries in the Caribbean and Polynesia (eg, Dominica for boys and girls and French Polynesia for girls), where mean height throughout late childhood and adolescence was higher than the median of the WHO reference by about 3 cm or more. Elsewhere, either height advantage (ie, having mean height higher than the WHO reference median) at 5 years was diminished or reversed as children grew older, or height disadvantage (ie, having mean height lower than the WHO reference median) increased. This progressive falling behind as children grew older was especially noticeable in middle-income countries in Latin America and the Caribbean (eg, Chile and Uruguay), the Middle East and north Africa (eg, United Arab Emirates), and sub-Saharan Africa (eg, Mauritius and South Africa), where children had optimal height at age 5 years, but by the time they reached age 19 years, their height was shorter than the median of the WHO reference, by about 2 cm or more. A small number of countries (eg, Russia for boys and girls and Iran for boys) slightly reduced the gap to the WHO reference median during late childhood and adolescence.

For BMI, the deficit relative to the WHO reference median at age 5 years, which was seen mainly in sub-Saharan Africa and south and southeast Asia, generally became smaller or disappeared as children grew to adolescence and reached age 19 years (figure 5A). For girls in South Africa and girls and boys in Canada, China, and some countries in Oceania, the Middle East and north Africa, and Latin America and the Caribbean, mean BMI was similar to the WHO reference median for 5-year-old children, but exceeded the WHO reference median as the children became older.

Comparing height and BMI in each country with the median of all countries (figure 5B) showed that children and adolescents in some countries had a consistent height advantage or disadvantage relative to those in other countries at every age. This was especially the case for countries at the top (eg, the Netherlands, Denmark, Montenegro, Estonia, and Iceland) and the bottom (eg, Timor-Leste, Laos, Nepal, Yemen, and Guatemala) of the global ranks at age 19 years. For other countries, children's height caught up with or fell behind their comparators during school ages. For example, children in some European countries (eg, girls in Belgium and boys in Austria) and Latin America and the Caribbean (eg, girls in Puerto Rico and boys in Barbados) had about the same height as Dutch children at age 5 years, but progressively fell behind such that, by the time they were 19 years old, they were more than 5 cm shorter than Dutch adolescents. By contrast, the height of children and adolescents in Latvia, Czech Republic, Morocco, and Iran progressively improved relative to others as they approached age 19 years.

When age-specific mean BMI was compared with the global median (figure 5B), whether a country had low (eg, countries in south and southeast Asia) or high (eg, the USA, Chile, and countries in Oceania) mean BMI relative to others, persisted more than was the case for height. Nonetheless, some differences in age trajectories of BMI occurred across countries. For example, girls and boys in some European countries (eg, Italy, France, and Croatia), Japan, and Seychelles progressively moved towards healthier BMIs relative to other countries, and the difference between their BMI and the global median changed from positive to negative. By contrast, girls and boys in countries such as Kuwait, Bahrain, Fiji, Jamaica, and Mexico; and girls in South Africa and New Zealand had a progressively higher BMI relative to the global median as they became older.

## Discussion

We identified highly variable age trajectories and trends over time in the height and BMI of school-aged children and adolescents across countries and territories. These cross-country differences show that childhood and adolescence are crucial periods in differentiating countries in terms of how they shape these determinants of lifelong health.

Our results are consistent with findings from both studies of adolescents in individual countries and global studies of adult height, which show substantial variation in how much height has changed throughout the world.^14^ One study,^15^ assessing cross-sectional height in 53 community-based samples, found substantial cross-population variation in height differences from ages 10–17 years, which is consistent with our findings on age trajectories. Our results are also consistent with previous global analyses^11^ in terms of regions and countries with the highest and lowest BMI, but previous studies had not considered age trajectories.

Our study has strengths in scope, data, and methods: we presented novel estimates of height in school-aged children and adolescents for all countries in the world, and we did so alongside estimates of BMI. We used an unprecedented scale of population-based data from 193 countries and territories covering approximately 99% of the world's population, while maintaining a high standard of data representativeness and quality. Data were analysed according to a consistent protocol, and the characteristics and quality of data from each country were rigorously verified through repeated checks by NCD-RisC members. We used a statistical model that accounted for non-linear changes in height and BMI throughout childhood and adolescence, and we used all available data while giving more weight to national data than to subnational and community sources.

As with all global analyses, our study has some limitations. Despite our extensive efforts to identify and access worldwide population-based data, some countries, especially those in the Caribbean, Polynesia and Micronesia, Melanesia, and sub-Saharan Africa, had fewer data sources than in other regions. The scarcity of data is reflected in the larger uncertainty of our estimates for these countries and regions compared with those for other countries. Of the studies used, less than half had data for children aged 5–9 years compared with nearly 90% with data for children aged 10–19 years, which increases the uncertainty of findings for the younger age groups. BMI is an imperfect measure of the extent and distribution of fat in the body, but it has the major advantage of having consistent and comparable data in many population-based surveys, especially compared with measures such as body fat measured by dual-energy x-ray absorptiometry (DEXA), which is complex and costly and cannot be used in surveys. A systematic review reported that BMI and DEXA-measured body fat were highly correlated.^16^ We compared height and BMI in each country with the median of the WHO growth reference.^12^ Although the reference is the current international comparison tool,^17^ unlike that of children younger than 5 years, it is not based on a multicountry sample of predominantly healthy and well-nourished children.^12^ Consequently, the reference might be affected by slower growth as the sample children grew older.^18^ Future studies should also evaluate the socioeconomic and geographical patterns of height and BMI in these ages, as has been done for children younger than 5 years and adults.^19^, ^20^

Several factors that interact throughout childhood and adolescence, and possibly across generations, might be responsible for the heterogeneous worldwide age trajectories and trends of height and BMI.^21^ First, there is an important genetic component to height^22^, ^23^ and, to a lesser extent, to BMI^24^ within populations. However, genetics explains a small part of the variation across countries or the changes over time, especially for BMI.^25^, ^26^, ^27^, ^28^ That genetics has a small role in height and BMI at the population level relative to nutrition and environment is also supported by the finding that the height of migrant descendents typically converges to the height of their new country within a few generations.^29^, ^30^, ^31^ Second, some of the observed differences in height and BMI might be intergenerational or due to exposures and experiences during pregnancy, mediated through birth length and weight.^21^ Third, the age of puberty onset, which is influenced by diet, physical activity, and weight gain during childhood, might affect height gain during the adolescent growth spurt and in late adolescence.^32^ Although some studies have found a negative association between age of pubertal onset and final height,^33^ others have found that age of pubertal onset does not affect final height, because an earlier puberty onset might be compensated by a more intense or longer period of peak height velocity.^34^ No comparable global data exist on age at menarche and timing of pubertal growth, but national data indicate substantial changes in some countries. Finally, all of these pathways are influenced by food and nutrition,^28^, ^35^, ^36^ including energy balance, and adequacy and quality of nutrients, especially proteins, fats, and micronutrients.^18^, ^21^, ^37^ There is also an important effect from the occurrence and treatment of infections, which itself is influenced by water and sanitation, and whether episodes of infections are effectively treated in a timely manner. Similarly, physical activity at home and school can influence BMI. Fully establishing the drivers of the observed height and BMI trajectories and trends requires data on these determinants and their distributions in different countries.

Our findings on the heterogeneous age trajectories and time trends of height and BMI in late childhood and adolescence raise the need to rethink and revise two common features of global health and nutrition programmes. First, we need to overcome the disconnect in research and practice between reducing undernutrition, particularly short stature, and preventing and managing overweight and obesity.^11^, ^19^, ^21^ Second, the finding that children in some countries grow healthily to age 5 years but do not continue to do so during school years shows an imbalance between investment in improving nutrition and growth before age 5 years and doing so in school-aged children and adolescents.^38^ Therefore, our findings should motivate policies and interventions at home, at school, in the community, and through the health system to support healthy growth during the entire period from birth to adolescence through enhanced nutritional quality, healthier living environment, and provision of high-quality preventive and curative care. These measures include agricultural and food system policies^39^ that increase the availability and reduce the cost of nutritious foods that help children grow taller without gaining excessive weight for their height; (conditional) cash transfers and food vouchers towards nutritious foods for low-income families; free healthy school meal programmes; fiscal and regulatory policies that restrict the consumption of unhealthy foods, especially processed carbohydrates; the provision of affordable healthy housing, clean water, and sanitation; and the provision of facilities for play and sports in the community and at school. Taking these actions would enable children to grow taller without gaining excessive weight, with lifelong benefits for their health and wellbeing.

Correspondence to:Prof Majid Ezzati, School of Public Health, Imperial College London, London W2 1PG, UK majid.ezzati@imperial.ac.uk
