# Supplementary material for: Height and body-mass index trajectories of school-aged children and adolescents from 1985 to 2019 in 200 countries and territories: a pooled analysis of 2181 population-based studies with 65 million participants
Source: Lancet. 2020 Nov 7;396(10261):1511–24. doi: 10.1016/S0140-6736(20)31859-6 (PMC7658740; doi:10.1016/S0140-6736(20)31859-6)
Supplement: Supplementary appendix [file mmc1.pdf]

THE LANCET

Supplementary appendix

This appendix formed part of the original submission and has been peer reviewed. We post it as supplied by the authors.

Supplement to: NCD Risk Factor Collaboration (NCD-RisC). Height and body-mass index trajectories of school-aged children and adolescents from 1985 to 2019 in 200 countries and territories: a pooled analysis of 2181 population-based studies with 65 million participants. *Lancet* 2020; **396:** 1511–24.

Table of Contents

Appendix Text 1. NCD Risk Factor Collaboration (NCD-RisC) ... 2

Appendix Text 2. Data sources ... 39

Appendix Text 3. Statistical methods ... 43

Appendix Table 1. List of analysis regions and "super-regions", and countries in each region ... 46

Appendix Table 2. Data sources used in the analysis ... 49

Appendix Figure 1. Median height of the WHO reference population and median height of all countries in the world for children born in the year 2000, by age. ... 90

Appendix Figure 2. Median body-mass index (BMI) of the WHO reference population and median BMI of all countries in the world for children born in the year 2000, by age ... 92

Appendix Figure 3. Number of data sources by country. ... 94

Appendix Figure 4. Trends and age trajectories of height and BMI, by country ... 96

Appendix Figure 5. Age trajectory of height and BMI for 2019 19-year-olds ... 297

Appendix Figure 6. Difference between the height and BMI of boys and girls born who were 19 years old in 2019, by age ... 299

References ... 301

Appendix Text 1. NCD Risk Factor Collaboration (NCD-RisC)

Pooled Analysis and Writing

Andrea Rodriguez-Martinez (PhD) (Imperial College London, UK); Bin Zhou (PhD) (Imperial College London, UK); Marisa K Sophiea (MSc) (Imperial College London, UK); James Bentham (PhD) (University of Kent, UK); Prof Christopher J Paciorek (PhD) (University of California Berkeley, USA); Maria LC Iurilli (MRes) (Imperial College London, UK); Rodrigo M Carrillo-Larco (MD) (Imperial College London, UK); James E Bennett (PhD) (Imperial College London, UK); Mariachiara Di Cesare (PhD) (Middlesex University, UK); Cristina Taddei (MD) (Imperial College London, UK); Honor Bixby (PhD) (Imperial College London, UK); Gretchen A Stevens (DSc) (Independent researcher, USA; Imperial College London, UK); Leanne M Riley (MSc) (World Health Organization, Switzerland); Melanie J Cowan (MPH) (World Health Organization, Switzerland); Stefan Savin (MPH) (World Health Organization, Switzerland); Goodarz Danaei (ScD) (Harvard TH Chan School of Public Health, USA); Adela Chirita-Emandi (MD) (Victor Babeş University of Medicine and Pharmacy Timisoara, Romania); Prof Andre P Kengne (PhD) (South African Medical Research Council, South Africa); Prof Young-Ho Khang (MD) (Seoul National University, Republic of Korea); Prof Avula Laxmaiah (MBBS) (ICMR - National Institute of Nutrition, India); Prof Reza Malekzadeh (MD) (Tehran University of Medical Sciences, Iran); Prof J Jaime Miranda (PhD) (Universidad Peruana Cayetano Heredia, Peru); Prof Jin Soo Moon (MD) (Seoul National University Children's Hospital, Republic of Korea); Stevo R Popovic (PhD) (University of Montenegro, Montenegro); Prof Thorkild IA Sørensen (DrMedSci) (University of Copenhagen, Denmark); Maroje Sorić (PhD) (University of Zagreb, Croatia); Gregor Starc (PhD) (University of Ljubljana, Slovenia); Ahmad A Zainuddin (MSc) (Ministry of Health, Malaysia); Prof Edward W Gregg (PhD) (Imperial College London, UK); Prof Zulfiqar A Bhutta (PhD) (The Hospital for Sick Children, Canada; Aga Khan University, Pakistan); Prof Robert Black (MD) (Johns

Hopkins University, USA); Prof Majid Ezzati (FMedSci) (Imperial College London, UK; University of Ghana, Ghana)

Country and Regional Data (* equal contribution; listed alphabetically)

Leandra Abarca-Gómez (Caja Costarricense de Seguro Social, Costa Rica)*; Ziad A Abdeen (Al-Quds University, Palestine)*; Shynar Abdrakhmanova (National Center of Public Healthcare, Kazakhstan)*; Suhaila Abdul Ghaffar (Ministry of Health, Malaysia)*; Hanan F Abdul Rahim (Qatar University, Qatar)*; Niveen M Abu-Rmeileh (Birzeit University, Palestine)*; Jamila Abubakar Garba (Usmanu Danfodiyo University Teaching Hospital, Nigeria)*; Benjamin Acosta-Cazares (Instituto Mexicano del Seguro Social, Mexico)*; Robert J Adams (Flinders University, Australia)*; Wichai Aekplakorn (Mahidol University, Thailand)*; Kaosar Afsana (BRAC University, Bangladesh)*; Shoaib Afzal (Copenhagen University Hospital, Denmark)*; Imelda A Agdeppa (Food and Nutrition Research Institute, Philippines)*; Javad Aghazadeh-Attari (Urmia University of Medical Sciences, Iran)*; Carlos A Aguilar-Salinas (Instituto Nacional de Ciencias Médicas y Nutrición, Mexico)*; Charles Agyemang (University of Amsterdam, The Netherlands)*; Mohamad Hasnan Ahmad (Ministry of Health, Malaysia)*; Noor Ani Ahmad (Ministry of Health, Malaysia)*; Ali Ahmadi (Modeling in Health Research Center, Iran)*; Naser Ahmadi (Non-Communicable Diseases Research Center, Iran)*; Soheir H Ahmed (University of Oslo, Norway)*; Wolfgang Ahrens (University of Bremen, Germany)*; Gulmira Aitmurzaeva (Republican Center for Health Promotion, Kyrgyzstan)*; Kamel Ajlouni (National Center for Diabetes, Endocrinology and Genetics, Jordan)*; Hazzaa M Al-Hazzaa (Princess Nourah bint Abdulrahman University, Saudi Arabia)*; Amani Rashed Al-Othman (Kuwait Institute for Scientific Research, Kuwait)*; Rajaa Al-Raddadi (King Abdulaziz University, Saudi Arabia)*; Monira Alarouj (Dasman Diabetes Institute, Kuwait)*; Fadia AlBuhairan (Aldara Hospital and Medical Center, Saudi Arabia)*; Shahla AlDhukair (King Abdullah International Medical Research Center, Saudi Arabia)*; Mohamed M Ali (World Health Organization, Switzerland)*; Abdullah Alkandari (Dasman Diabetes Institute,

Kuwait)*; Ala'a Alkerwi (Luxembourg Institute of Health, Luxembourg)*; Kristine Allin (Bispebjerg and Frederiksberg Hospital, Denmark)*; Mar Alvarez-Pedrerol (ISGlobal Centre for Research in Environmental Epidemiology, Spain)*; Eman Aly (World Health Organization Regional Office for the Eastern Mediterranean, Egypt)*; Deepak N Amarapurkar (Bombay Hospital and Medical Research Centre, India)*; Parisa Amiri (Research Center for Social Determinants of Health, Iran)*; Norbert Amougou (UMR CNRS-MNHN 7206 Eco-anthropologie, France)*; Philippe Amouyel (University of Lille, France; Lille University Hospital, France)*; Lars Bo Andersen (Western Norway University of Applied Sciences, Norway)*; Sigmund A Anderssen (Norwegian School of Sport Sciences, Norway)*; Lars Ängquist (University of Copenhagen, Denmark)*; Ranjit Mohan Anjana (Madras Diabetes Research Foundation, India)*; Alireza Ansari-Moghaddam (Zahedan University of Medical Sciences, Iran)*; Hajer Aounallah-Skhiri (National Institute of Public Health, Tunisia)*; Joana Araújo (Institute of Public Health of the University of Porto, Portugal)*; Inger Ariansen (Norwegian Institute of Public Health, Norway)*; Tahir Aris (Ministry of Health, Malaysia)*; Raphael E Arku (University of Massachusetts, USA)*; Nimmathota Arlappa (ICMR - National Institute of Nutrition, India)*; Krishna K Aryal (Abt Associates, Nepal)*; Thor Aspelund (University of Iceland, Iceland)*; Felix K Assah (University of Yaoundé 1, Cameroon)*; Maria Cecília F Assunção (Federal University of Pelotas, Brazil)*; May Soe Aung (University of Medicine 1, Myanmar)*; Juha Auvinen (University of Oulu, Finland; Oulu University Hospital, Finland)*; Mária Avdicová (Banska Bystrica Regional Authority of Public Health, Slovakia)*; Ana Azevedo (University of Porto Medical School, Portugal)*; Mohsen Azimi-Nezhad (Neyshabur University of Medical Sciences, Iran)*; Fereidoun Azizi (Research Institute for Endocrine Sciences, Iran)*; Mehrdad Azmin (Non-Communicable Diseases Research Center, Iran)*; Bontha V Babu (Indian Council of Medical Research, India)*; Maja Bæksgaard Jørgensen (National Institute of Public Health, Denmark)*; Azli Baharudin (Ministry of Health, Malaysia)*; Suhad Bahijri (King Abdulaziz University, Saudi Arabia)*; Jennifer L Baker (Bispebjerg and Frederiksberg Hospital, Denmark)*; Nagalla Balakrishna (ICMR - National Institute of Nutrition, India)*;

Mohamed Bamoshmoosh (University of Science and Technology, Yemen)*; Maciej Banach (Medical University of Lodz, Poland)*; Piotr Bandosz (Medical University of Gdansk, Poland)*; José R Banegas (Universidad Autónoma de Madrid/CIBERESP, Spain)*; Joanna Baran (University of Rzeszów, Poland)*; Carlo M Barbagallo (University of Palermo, Italy)*; Alberto Barceló (Pan American Health Organization, USA)*; Amina Barkat (Mohammed V University de Rabat, Morocco)*; Aluisio JD Barros (Federal University of Pelotas, Brazil)*; Mauro Virgílio Gomes Barros (University of Pernambuco, Brazil)*; Abdul Basit (Baqai Medical University, Pakistan)*; Joao Luiz D Bastos (Federal University of Santa Catarina, Brazil)*; Iqbal Bata (Dalhousie University, Canada)*; Anwar M Batieha (Jordan University of Science and Technology, Jordan)*; Rosangela L Batista (Federal University of Maranhão, Brazil)*; Zhamilya Battakova (National Center of Public Healthcare, Kazakhstan)*; Assembekov Batyrbek (Al-Farabi Kazakh National University, Kazakhstan)*; Louise A Baur (University of Sydney, Australia)*; Robert Beaglehole (University of Auckland, New Zealand)*; Silvia Bel-Serrat (University College Dublin, Ireland)*; Antonisamy Belavendra (Christian Medical College, India)*; Habiba Ben Romdhane (University Tunis El Manar, Tunisia)*; Judith Benedics (Federal Ministry of Social Affairs, Health, Care and Consumer Protection, Austria)*; Mikhail Benet (Cafam University Foundation, Colombia)*; James E Bennett (Imperial College London, UK)*; Salim Berkinbayev (Kazakh National Medical University, Kazakhstan)*; Antonio Bernabe-Ortiz (Universidad Peruana Cayetano Heredia, Peru)*; Gailute Bernotiene (Lithuanian University of Health Sciences, Lithuania)*; Heloísa Bettiol (University of São Paulo, Brazil)*; Jorge Bezerra (University of Pernambuco, Brazil)*; Aroor Bhagyalaxmi (B J Medical College, India)*; Sumit Bharadwaj (Chirayu Medical College, India)*; Santosh K Bhargava (Sunder Lal Jain Hospital, India)*; Zulfiqar A Bhutta (The Hospital for Sick Children, Canada; Aga Khan University, Pakistan)*; Hongsheng Bi (Shandong University of Traditional Chinese Medicine, China)*; Yufang Bi (Shanghai Jiao-Tong University School of Medicine, China)*; Daniel Bia (Republic University, Uruguay)*; Elysée Claude Bika Lele (Institute of Medical Research and Medicinal Plant Studies,

Cameroon)*; Mukharram M Bikbov (Ufa Eye Research Institute, Russia)*; Bihungum Bista (Nepal Health Research Council, Nepal)*; Dusko J Bjelica (University of Montenegro, Montenegro)*; Peter Bjerregaard (University of Southern Denmark, Denmark)*; Espen Bjertness (University of Oslo, Norway)*; Marius B Bjertness (University of Oslo, Norway)*; Cecilia Björkelund (University of Gothenburg, Sweden)*; Katia V Bloch (Universidade Federal do Rio de Janeiro, Brazil)*; Anneke Blokstra (National Institute for Public Health and the Environment, The Netherlands)*; Simona Bo (University of Turin, Italy)*; Martin Bobak (University College London, UK)*; Lynne M Boddy (Liverpool John Moores University, UK)*; Bernhard O Boehm (Nanyang Technological University Singapore, Singapore)*; Heiner Boeing (German Institute of Human Nutrition, Germany)*; Jose G Boggia (Republic University, Uruguay)*; Elena Bogova (Endocrinology Research Centre, Russia)*; Carlos P Boissonnet (Centro de Educación Médica e Investigaciones Clínicas, Argentina)*; Stig E Bojesen (Copenhagen University Hospital, Denmark; University of Copenhagen, Denmark)*; Marialaura Bonaccio (IRCCS Neuromed, Italy)*; Vanina Bongard (Toulouse University School of Medicine, France)*; Alice Bonilla-Vargas (Caja Costarricense de Seguro Social, Costa Rica)*; Matthias Bopp (University of Zurich, Switzerland)*; Herman Borghs (University Hospital KU Leuven, Belgium)*; Pascal Bovet (Ministry of Health, Seychelles; University of Lausanne, Switzerland)*; Lien Braeckevelt (Flemish Agency for Care and Health, Belgium)*; Lutgart Braeckman (Ghent University, Belgium)*; Marjolijn CE Bragt (FrieslandCampina, The Netherlands)*; Imperia Brajkovich (Universidad Central de Venezuela, Venezuela)*; Francesco Branca (World Health Organization, Switzerland)*; Juergen Breckenkamp (Bielefeld University, Germany)*; João Breda (World Health Organization Regional Office for Europe, Russia)*; Hermann Brenner (German Cancer Research Center, Germany)*; Lizzy M Brewster (University of Amsterdam, The Netherlands)*; Garry R Brian (The Fred Hollows Foundation, New Zealand)*; Lacramioara Brinduse (University of Medicine and Pharmacy Bucharest, Romania)*; Sinead Brophy (Swansea University, UK)*; Graziella Bruno (University of Turin, Italy)*; H Bas Bueno-de-Mesquita (National Institute for Public Health and the Environment,

The Netherlands)*; Anna Bugge (University College Copenhagen, Denmark)*; Marta Buoncristiano (World Health Organization Regional Office for Europe, Russia)*; Genc Burazeri (Institute of Public Health, Albania)*; Con Burns (Cork Institute of Technology, Ireland)*; Antonio Cabrera de León (Universidad de La Laguna, Spain)*; Joseph Cacciottolo (University of Malta, Malta)*; Hui Cai (Vanderbilt University, USA)*; Tilema Cama (Ministry of Health, Tonga)*; Christine Cameron (Canadian Fitness and Lifestyle Research Institute, Canada)*; José Camolas (Hospital Santa Maria, Portugal)*; Günay Can (Istanbul University - Cerrahpasa, Turkey)*; Ana Paula C Cândido (Universidade Federal de Juiz de Fora, Brazil)*; Felicia Cañete (Ministry of Public Health, Paraguay)*; Mario V Capanzana (Food and Nutrition Research Institute, Philippines)*; Nadežda Capková (National Institute of Public Health, Czech Republic)*; Eduardo Capuano (Gaetano Fucito Hospital, Italy)*; Vincenzo Capuano (Gaetano Fucito Hospital, Italy)*; Marloes Cardol (University of Groningen, The Netherlands)*; Viviane C Cardoso (University of São Paulo, Brazil)*; Axel C Carlsson (Karolinska Institutet, Sweden)*; Esteban Carmuega (Centro de Estudios sobre Nutrición Infantil, Argentina)*; Joana Carvalho (University of Porto, Portugal)*; José A Casajús (University of Zaragoza, Spain)*; Felipe F Casanueva (Santiago de Compostela University, Spain)*; Ertugrul Celikcan (Ministry of Health, Turkey)*; Laura Censi (Council for Agricultural Research and Economics, Italy)*; Marvin Cervantes-Loaiza (Caja Costarricense de Seguro Social, Costa Rica)*; Juraci A Cesar (Federal University of Rio Grande, Brazil)*; Snehalatha Chamukuttan (India Diabetes Research Foundation, India)*; Angelique W Chan (Duke-NUS Medical School, Singapore)*; Queenie Chan (Imperial College London, UK)*; Himanshu K Chaturvedi (ICMR - National Institute of Medical Statistics, India)*; Nish Chaturvedi (University College London, UK)*; Norsyamlina Che Abdul Rahim (Ministry of Health, Malaysia)*; Chien-Jen Chen (Academia Sinica, Taiwan)*; Fangfang Chen (Capital Institute of Pediatrics, China)*; Huashuai Chen (Duke University, USA)*; Shuohua Chen (Kailuan General Hospital, China)*; Zhengming Chen (University of Oxford, UK)*; Ching-Yu Cheng (Duke-NUS Medical School, Singapore)*; Bahman Cheraghian (Ahvaz Jundishapur University of Medical Sciences,

Iran)*; Angela Chetrit (The Gertner Institute for Epidemiology and Health Policy Research, Israel)*; Ekaterina Chikova-Iscener (National Centre of Public Health and Analyses, Bulgaria)*; Arnaud Chiolero (University of Fribourg, Switzerland)*; Shu-Ti Chiou (Ministry of Health and Welfare, Taiwan)*; Adela Chirita-Emandi (Victor Babes University of Medicine and Pharmacy Timisoara, Romania)*; María-Dolores Chirlaque (Murcia Health Council, Spain)*; Belong Cho (Seoul National University, Republic of Korea)*; Kaare Christensen (University of Southern Denmark, Denmark)*; Diego G Christofaro (Universidade Estadual Paulista, Brazil)*; Jerzy Chudek (Medical University of Silesia, Poland)*; Renata Cifkova (Charles University, Czech Republic; Thomayer Hospital, Czech Republic)*; Michelle Cilia (Primary Health Care, Malta)*; Eliza Cinteza (Carol Davila University of Medicine and Pharmacy, Romania)*; Frank Claessens (Katholieke Universiteit Leuven, Belgium)*; Janine Clarke (Statistics Canada, Canada)*; Els Clays (Ghent University, Belgium)*; Emmanuel Cohen (UMR CNRS-MNHN 7206 Eco-anthropologie, France)*; Hans Concin (Agency for Preventive and Social Medicine, Austria)*; Susana C Confortin (Federal University of Maranhão, Brazil)*; Cyrus Cooper (University of Southampton, UK)*; Tara C Coppinger (Cork Institute of Technology, Ireland)*; Eva Corpeleijn (University of Groningen, The Netherlands)*; Simona Costanzo (IRCCS Neuromed, Italy)*; Dominique Cottel (Institut Pasteur de Lille, France)*; Chris Cowell (University of Sydney, Australia)*; Cora L Craig (Canadian Fitness and Lifestyle Research Institute, Canada)*; Amelia C Crampin (Malawi Epidemiology and Intervention Research Unit, Malawi)*; Ana B Crujeiras (CIBEROBN, Spain)*; Semánová Csilla (University of Debrecen, Hungary)*; Alexandra M Cucu (University of Medicine and Pharmacy Carol Davila, Romania)*; Liufu Cui (Kailuan General Hospital, China)*; Felipe V Cureau (Universidade Federal do Rio Grande do Sul, Brazil)*; Graziella D'Arrigo (National Council of Research, Italy)*; Eleonora d'Orsi (Federal University of Santa Catarina, Brazil)*; Liliana Dacica (Eftimie Murgu University Resita, Romania)*; María Ángeles Dal Re Saavedra (Spanish Agency of Food Safety and Nutrition, Spain)*; Jean Dallongeville (Institut Pasteur de Lille, France)*; Albertino Damasceno (Eduardo Mondlane

University, Mozambique)*; Camilla T Damsgaard (University of Copenhagen, Denmark)*; Goodarz Danaei (Harvard TH Chan School of Public Health, USA)*; Rachel Dankner (The Gertner Institute for Epidemiology and Health Policy Research, Israel)*; Thomas M Dantoft (Bispebjerg and Frederiksberg Hospital, Denmark)*; Parasmani Dasgupta (Indian Statistical Institute, India)*; Saeed Dastgiri (Tabriz Health Services Management Centre, Iran)*; Luc Dauchet (University of Lille, France; Lille University Hospital, France)*; Kairat Davletov (Al-Farabi Kazakh National University, Kazakhstan)*; Guy De Backer (Ghent University, Belgium)*; Dirk De Bacquer (Ghent University, Belgium)*; Giovanni de Gaetano (IRCCS Neuromed, Italy)*; Stefaan De Henauw (Ghent University, Belgium)*; Paula Duarte de Oliveira (Federal University of Pelotas, Brazil)*; David De Ridder (Geneva University Hospitals, Switzerland)*; Karin De Ridder (Sciensano, Belgium)*; Susanne R de Rooij (Amsterdam University Medical Centers, The Netherlands; University of Amsterdam, The Netherlands)*; Delphine De Smedt (Ghent University, Belgium)*; Mohan Deepa (Madras Diabetes Research Foundation, India)*; Alexander D Deev (National Research Centre for Preventive Medicine, Russia)*; Vincent Jr DeGennaro (Innovating Health International, Haiti)*; Abbas Dehghan (Imperial College London, UK)*; Hélène Delisle (University of Montreal, Canada)*; Francis Delpeuch (French National Research Institute for Sustainable Development, France)*; Stefaan Demarest (Sciensano, Belgium)*; Elaine Dennison (University of Southampton, UK)*; Katarzyna Dereń (University of Rzeszów, Poland)*; Valérie Deschamps (French Public Health Agency, France)*; Klodian Dhana (Erasmus Medical Center Rotterdam, The Netherlands)*; Meghnath Dhimal (Nepal Health Research Council, Nepal)*; Augusto F Di Castelnuovo (Mediterranea Cardiocentro, Italy)*; Juvenal Soares Dias-da-Costa (Universidade do Vale do Rio dos Sinos, Brazil)*; María Elena Díaz-Sánchez (National Institute of Hygiene, Epidemiology and Microbiology, Cuba)*; Alejandro Diaz (National Council of Scientific and Technical Research, Argentina)*; Zivka Dika (University of Zagreb, Croatia)*; Shirin Djalalinia (Ministry of Health and Medical Education, Iran)*; Visnja Djordjic (University of Novi Sad, Serbia)*; Ha TP Do (National Institute of Nutrition, Vietnam)*; Annette J Dobson (University of Queensland,

Australia)*; Maria Benedetta Donati (IRCCS Neuromed, Italy)*; Chiara Donfrancesco (Istituto Superiore di Sanità, Italy)*; Silvana P Donoso (Universidad de Cuenca, Ecuador)*; Angela Döring (Helmholtz Zentrum München, Germany)*; Maria Dorobantu (Carol Davila University of Medicine and Pharmacy, Romania)*; Ahmad Reza Dorosty (Tehran University of Medical Sciences, Iran)*; Kouamelan Doua (Ministère de la Santé et de l'Hygiène Publique, Côte d'Ivoire)*; Wojciech Drygas (National Institute of Cardiology, Poland; Medical University of Lodz, Poland)*; Jia Li Duan (Beijing Center for Disease Prevention and Control, China)*; Charmaine A Duante (Food and Nutrition Research Institute, Philippines)*; Priscilla Duboz (UMI 3189 ESS, France)*; Rosemary B Duda (Beth Israel Deaconess Medical Center, USA; Harvard Medical School, USA)*; Vesselka Duleva (National Centre of Public Health and Analyses, Bulgaria)*; Virginija Dulskiene (Lithuanian University of Health Sciences, Lithuania)*; Samuel C Dumith (Federal University of Rio Grande, Brazil)*; Anar Dushpanova (Al-Farabi Kazakh National University, Kazakhstan)*; Vilnis Dzerve (University of Latvia, Latvia)*; Elzbieta Dziankowska-Zaborszczyk (Medical University of Lodz, Poland)*; Ricky Eddie (Ministry of Health and Medical Services, Solomon Islands)*; Ebrahim Eftekhar (Hormozgan University of Medical Sciences, Iran)*; Eruke E Egbagbe (University of Benin, Nigeria)*; Robert Eggertsen (University of Gothenburg, Sweden)*; Sareh Eghtesad (Tehran University of Medical Sciences, Iran)*; Gabriele Eiben (University of Skövde, Sweden)*; Ulf Ekelund (Norwegian School of Sport Sciences, Norway)*; Mohammad El-Khateeb (National Center for Diabetes, Endocrinology and Genetics, Jordan)*; Jalila El Ati (National Institute of Nutrition and Food Technology, Tunisia)*; Denise Eldemire-Shearer (The University of the West Indies, Jamaica)*; Marie Eliasen (Bispebjerg and Frederiksberg Hospital, Denmark)*; Paul Elliott (Imperial College London, UK)*; Reina Engle-Stone (University of California Davis, USA)*; Macia Enguerran (UMI 3189 ESS, France)*; Rajiv T Erasmus (University of Stellenbosch, South Africa)*; Raimund Erbel (University of Duisburg-Essen, Germany)*; Cihangir Erem (Karadeniz Technical University, Turkey)*; Louise Eriksen (University of Southern Denmark, Denmark)*; Johan G Eriksson (University of Helsinki, Finland)*; Jorge Escobedo-de la Peña (Instituto Mexicano del

Seguro Social, Mexico)*; Saeid Eslami (Mashhad University of Medical Sciences, Iran)*; Ali Esmaeili (Rafsanjan University of Medical Sciences, Iran)*; Alun Evans (Queen's University of Belfast, UK)*; David Faeh (University of Zurich, Switzerland)*; Albina A Fakhretdinova (Ufa Eye Research Institute, Russia)*; Caroline H Fall (University of Southampton, UK)*; Elnaz Faramarzi (Tabriz University of Medical Sciences, Iran)*; Mojtaba Farjam (Fasa University of Medical Sciences, Iran)*; Victoria Farrugia Sant'Angelo (Primary Health Care, Malta)*; Farshad Farzadfar (Non-Communicable Diseases Research Center, Iran)*; Mohammad Reza Fattahi (Shiraz University of Medical Sciences, Iran)*; Asher Fawwad (Baqai Medical University, Pakistan)*; Francisco J Felix-Redondo (Centro de Salud Villanueva Norte, Spain)*; Trevor S Ferguson (The University of the West Indies, Jamaica)*; Romulo A Fernandes (Universidade Estadual Paulista, Brazil)*; Daniel Fernández-Bergés (Hospital Don Benito-Villanueva de la Serena, Spain)*; Daniel Ferrante (Ministry of Health, Argentina)*; Thomas Ferrao (Statistics Canada, Canada)*; Marika Ferrari (Council for Agricultural Research and Economics, Italy)*; Marco M Ferrario (University of Insubria, Italy)*; Catterina Ferreccio (Pontificia Universidad Católica de Chile, Chile)*; Eldridge Ferrer (Food and Nutrition Research Institute, Philippines)*; Jean Ferrieres (Toulouse University School of Medicine, France)*; Thamara Hubler Figueiró (Federal University of Santa Catarina, Brazil)*; Anna Fijalkowska (Institute of Mother and Child, Poland)*; Günther Fink (Swiss Tropical and Public Health Institute, Switzerland; University of Basel, Switzerland)*; Krista Fischer (University of Tartu, Estonia)*; Bernhard Föger (Agency for Preventive and Social Medicine, Austria)*; Leng Huat Foo (Universiti Sains Malaysia, Malaysia)*; Maria Forsner (Umeå University, Sweden)*; Heba M Fouad (World Health Organization Regional Office for the Eastern Mediterranean, Egypt)*; Damian K Francis (The University of the West Indies, Jamaica)*; Maria do Carmo Franco (Federal University of São Paulo, Brazil)*; Oscar H Franco (Erasmus Medical Center Rotterdam, The Netherlands)*; Ruth Frikke-Schmidt (University of Copenhagen, Denmark)*; Guillermo Frontera (Hospital Universitario Son Espases, Spain)*; Flavio D Fuchs (Hospital de Clinicas de Porto Alegre, Brazil)*; Sandra C Fuchs (Universidade Federal do Rio

Grande do Sul, Brazil)*; Isti I Fujiati (Universitas Sumatera Utara, Indonesia)*; Yuki Fujita (Kindai University, Japan)*; Matsuda Fumihiko (Kyoto University, Japan)*; Takuro Furusawa (Kyoto University, Japan)*; Zbigniew Gaciong (Medical University of Warsaw, Poland)*; Mihai Gafencu (Victor Babes University of Medicine and Pharmacy Timisoara, Romania)*; Andrzej Galbarczyk (Jagiellonian University Medical College, Poland)*; Henrike Galenkamp (University of Amsterdam, The Netherlands)*; Daniela Galeone (Ministero della Salute DG Prevenzione Sanitaria, Italy)*; Myriam Galfo (Council for Agricultural Research and Economics, Italy)*; Fabio Galvano (University of Catania, Italy)*; Jingli Gao (Kailuan General Hospital, China)*; Manoli Garcia-de-la-Hera (CIBER en Epidemiología y Salud Pública, Spain)*; Marta García-Solano (Spanish Agency of Food Safety and Nutrition, Spain)*; Dickman Gareta (Africa Health Research Institute, South Africa)*; Sarah P Garnett (University of Sydney, Australia)*; Jean-Michel Gaspoz (Geneva University Medical School, Switzerland)*; Magda Gasull (CIBER en Epidemiología y Salud Pública, Spain)*; Adroaldo Cesar Araujo Gaya (Universidade Federal do Rio Grandre do Sul, Brazil)*; Anelise Reis Gaya (Universidade Federal do Rio Grandre do Sul, Brazil)*; Andrea Gazzinelli (Universidade Federal de Minas Gerais, Brazil)*; Ulrike Gehring (Utrecht University, The Netherlands)*; Harald Geiger (Agency for Preventive and Social Medicine, Austria)*; Johanna M Geleijnse (Wageningen University, The Netherlands)*; Ali Ghanbari (Non-Communicable Diseases Research Center, Iran)*; Erfan Ghasemi (Non-Communicable Diseases Research Center, Iran)*; Oana-Florentina Gheorghe-Fronea (Carol Davila University of Medicine and Pharmacy, Romania)*; Simona Giampaoli (Istituto Superiore di Sanità, Italy)*; Francesco Gianfagna (University of Insubria, Italy; Mediterranea Cardiocentro, Italy)*; Tiffany K Gill (University of Adelaide, Australia)*; Jonathan Giovannelli (University of Lille, France; Lille University Hospital, France)*; Glen Gironella (Food and Nutrition Research Institute, Philippines)*; Aleksander Giwercman (Lund University, Sweden)*; Konstantinos Gkiouras (Aristotle University of Thessaloniki, Greece)*; Justyna Godos (Oasi Research Institute-IRCCS, Italy)*; Sibel Gogen (Ministry of Health, Turkey)*; Rebecca A Goldsmith (Ministry of Health, Israel)*; David Goltzman

(McGill University, Canada)*; Santiago F Gómez (Gasol Foundation, Spain; University of Lleida, Spain)*; Aleksandra Gomula (PASs Hirszfeld Institute of Immunology and Experimental Therapy, Poland)*; Bruna Goncalves Cordeiro da Silva (Federal University of Pelotas, Brazil)*; Helen Gonçalves (Federal University of Pelotas, Brazil)*; David A Gonzalez-Chica (University of Adelaide, Australia)*; Marcela Gonzalez-Gross (Universidad Politécnica de Madrid, Spain)*; Margot González-Leon (Instituto Mexicano del Seguro Social, Mexico)*; Juan P González-Rivas (St Anne's University Hospital, Czech Republic)*; Clicerio González-Villalpando (National Institute of Public Health, Mexico)*; María-Elena González-Villalpando (Centro de Estudios en Diabetes A.C., Mexico)*; Angel R Gonzalez (Universidad Autónoma de Santo Domingo, Dominican Republic)*; Frederic Gottrand (University of Lille, France)*; Antonio Pedro Graça (Ministry of Health, Portugal)*; Sidsel Graff-Iversen (Norwegian Institute of Public Health, Norway)*; Dušan Grafnetter (Institute for Clinical and Experimental Medicine, Czech Republic)*; Aneta Grajda (Children's Memorial Health Institute, Poland)*; Maria G Grammatikopoulou (Aristotle University of Thessaloniki, Greece)*; Ronald D Gregor (Dalhousie University, Canada)*; Tomasz Grodzicki (Jagiellonian University Medical College, Poland)*; Else Karin Grøholt (Norwegian Institute of Public Health, Norway)*; Anders Grøntved (University of Southern Denmark, Denmark)*; Giuseppe Grosso (University of Catania, Italy)*; Gabriella Gruden (University of Turin, Italy)*; Dongfeng Gu (National Center of Cardiovascular Diseases, China)*; Emanuela Gualdi-Russo (University of Ferrara, Italy)*; Pilar Guallar-Castillón (Universidad Autónoma de Madrid/CIBERESP, Spain)*; Andrea Gualtieri (Authority Sanitaria San Marino, San Marino)*; Elias F Gudmundsson (Icelandic Heart Association, Iceland)*; Vilmundur Gudnason (University of Iceland, Iceland)*; Ramiro Guerrero (Universidad Icesi, Colombia)*; Idris Guessous (Geneva University Hospitals, Switzerland)*; Andre L Guimaraes (State University of Montes Claros, Brazil)*; Martin C Gulliford (King's College London, UK)*; Johanna Gunnlaugsdottir (Icelandic Heart Association, Iceland)*; Marc J Gunter (International Agency for Research on Cancer, France)*; Xiu-Hua Guo (Capital Medical University, China)*; Yin Guo (Capital Medical University

Beijing Tongren Hospital, China)*; Prakash C Gupta (Healis-Sekhsaria Institute for Public Health, India)*; Rajeev Gupta (Eternal Heart Care Centre and Research Institute, India)*; Oye Gureje (University of Ibadan, Nigeria)*; Beata Gurzkowska (Children's Memorial Health Institute, Poland)*; Enrique Gutiérrez-González (Spanish Agency of Food Safety and Nutrition, Spain)*; Laura Gutierrez (Institute for Clinical Effectiveness and Health Policy, Argentina)*; Felix Gutzwiller (University of Zurich, Switzerland)*; Seongjun Ha (National Health Insurance Service, Republic of Korea)*; Farzad Hadaegh (Prevention of Metabolic Disorders Research Center, Iran)*; Charalambos A Hadjigeorgiou (Research and Education Institute of Child Health, Cyprus)*; Rosa Haghshenas (Non-Communicable Diseases Research Center, Iran)*; Hamid Hakimi (Rafsanjan University of Medical Sciences, Iran)*; Jytte Halkjær (Danish Cancer Society Research Center, Denmark)*; Ian R Hambleton (The University of the West Indies, Barbados)*; Behrooz Hamzeh (Kermanshah University of Medical Sciences, Iran)*; Dominique Hange (University of Gothenburg, Sweden)*; Abu AM Hanif (BRAC James P Grant School of Public Health, Bangladesh)*; Sari Hantunen (University of Eastern Finland, Finland)*; Rachakulla Hari Kumar (ICMR - National Institute of Nutrition, India)*; Seyed Mohammad Hashemi-Shahri (Zahedan University of Medical Sciences, Iran)*; Maria Hassapidou (International Hellenic University, Greece)*; Jun Hata (Kyushu University, Japan)*; Teresa Haugsgjerd (University of Bergen, Norway)*; Alison J Hayes (University of Sydney, Australia)*; Jiang He (Tulane University, USA)*; Yuan He (National Research Institute for Health and Family Planning, China)*; Yuna He (Chinese Center for Disease Control and Prevention, China)*; Regina Heidinger-Felso (University of Pécs, Hungary)*; Mirjam Heinen (University College Dublin, Ireland)*; Tatjana Hejgaard (Danish Health Authority, Denmark)*; Marleen Elisabeth Hendriks (Joep Lange Institute, The Netherlands)*; Rafael dos Santos Henrique (Federal University of Pernambuco, Brazil)*; Ana Henriques (Institute of Public Health of the University of Porto, Portugal)*; Leticia Hernandez Cadena (National Institute of Public Health, Mexico)*; Sauli Herrala (Oulu University Hospital, Finland)*; Victor M Herrera (Universidad Autónoma de Bucaramanga, Colombia)*; Isabelle Herter-Aeberli (ETH

Zurich, Switzerland)*; Ramin Heshmat (Chronic Diseases Research Center, Iran)*; Allan G Hill (University of Southampton, UK)*; Sai Yin Ho (University of Hong Kong, China)*; Suzanne C Ho (The Chinese University of Hong Kong, China)*; Michael Hobbs (University of Western Australia, Australia)*; Albert Hofman (Erasmus Medical Center Rotterdam, The Netherlands)*; Ingunn Holden Bergh (Norwegian Institute of Public Health, Norway)*; Michelle Holdsworth (French National Research Institute for Sustainable Development, France)*; Reza Homayounfar (Fasa University of Medical Sciences, Iran)*; Clara Homs (Gasol Foundation, Spain; University Ramon Llull, Spain)*; Wilma M Hopman (Kingston Health Sciences Centre, Canada)*; Andrea RVR Horimoto (University of São Paulo, Brazil)*; Claudia M Hormiga (Fundación Oftalmológica de Santander, Colombia)*; Bernardo L Horta (Federal University of Pelotas, Brazil)*; Leila Houti (University Oran 1, Algeria)*; Christina Howitt (The University of the West Indies, Barbados)*; Thein Thein Htay (Independent Public Health Specialist, Myanmar)*; Aung Soe Htet (Ministry of Health and Sports, Myanmar)*; Maung Maung Than Htike (Ministry of Health and Sports, Myanmar)*; Yonghua Hu (Peking University, China)*; José María Huerta (CIBER en Epidemiología y Salud Pública, Spain)*; Ilpo Tapani Huhtaniemi (Imperial College London, UK)*; Constanta Huidumac Petrescu (National Institute of Public Health, Romania)*; Abdullatif Husseini (Birzeit University, Palestine)*; Chinh Nguyen Huu (National Institute of Nutrition, Vietnam)*; Inge Huybrechts (International Agency for Research on Cancer, France)*; Nahla Hwalla (American University of Beirut, Lebanon)*; Jolanda Hyska (Institute of Public Health, Albania)*; Licia Iacoviello (IRCCS Neuromed, Pozzilli, Italy; University of Insubria, Italy)*; Jesús M Ibarluzea (CIBER en Epidemiología y Salud Pública, Spain)*; Mohsen M Ibrahim (Cairo University, Egypt)*; Norazizah Ibrahim Wong (Ministry of Health, Malaysia)*; Nayu Ikeda (National Institutes of Biomedical Innovation, Health and Nutrition, Japan)*; M Arfan Ikram (Erasmus Medical Center Rotterdam, The Netherlands)*; Violeta Iotova (Medical University Varna, Bulgaria)*; Vilma E Irazola (Institute for Clinical Effectiveness and Health Policy, Argentina)*; Takafumi Ishida (The University of Tokyo, Japan)*; Muhammad Islam (The Hospital for Sick Children, Canada)*; Sheikh

Mohammed Shariful Islam (Deakin University, Australia)*; Masanori Iwasaki (Tokyo Metropolitan Institute of Gerontology, Japan)*; Rod T Jackson (University of Auckland, New Zealand)*; Jeremy M Jacobs (Hadassah University Medical Center, Israel)*; Hashem Y Jaddou (Jordan University of Science and Technology, Jordan)*; Tazeen Jafar (Duke-NUS Medical School, Singapore)*; Kenneth James (The University of the West Indies, Jamaica)*; Kazi M Jamil (Kuwait Institute for Scientific Research, Kuwait)*; Konrad Jamrozik (University of Adelaide, Australia; deceased)*; Imre Janszky (Norwegian University of Science and Technology, Norway)*; Edward Janus (University of Melbourne, Australia)*; Juel Jarani (Sports University of Tirana, Albania)*; Marjo-Riitta Jarvelin (Imperial College London, UK; University of Oulu, Finland)*; Grazyna Jasienska (Jagiellonian University Medical College, Poland)*; Ana Jelakovic (University Hospital Center Zagreb, Croatia)*; Bojan Jelakovic (University of Zagreb School of Medicine, Croatia)*; Garry Jennings (Heart Foundation, Australia)*; Anjani Kumar Jha (Nepal Health Research Council, Nepal)*; Chao Qiang Jiang (Guangzhou 12th Hospital, China)*; Ramon O Jimenez (Universidad Eugenio Maria de Hostos, Dominican Republic)*; Karl-Heinz Jöckel (University of Duisburg-Essen, Germany)*; Michel Joffres (Simon Fraser University, Canada)*; Mattias Johansson (International Agency for Research on Cancer, France)*; Jari J Jokelainen (Oulu University Hospital, Finland)*; Jost B Jonas (Institute of Molecular and Clinical Ophthalmology Basel, Switzerland)*; Torben Jørgensen (Bispebjerg and Frederiksberg Hospital, Denmark)*; Pradeep Joshi (World Health Organization Country Office, India)*; Farahnaz Joukar (Guilan University of Medical Sciences, Iran)*; Dragana P Jovic (Institute of Public Health of Serbia, Serbia)*; Jacek J Jóźwiak (University of Opole, Poland)*; Anne Juolevi (Finnish Institute for Health and Welfare, Finland)*; Gregor Jurak (University of Ljubljana, Slovenia)*; Iulia Jurca Simina (Victor Babes University of Medicine and Pharmacy Timisoara, Romania)*; Vesna Juresa (University of Zagreb, Croatia)*; Rudolf Kaaks (German Cancer Research Center, Germany)*; Felix O Kaducu (Gulu University, Uganda)*; Anthony Kafatos (University of Crete, Greece)*; Eero O Kajantie (Finnish Institute for Health and Welfare, Finland)*; Zhanna Kalmatayeva (Al-Farabi Kazakh National

University, Kazakhstan)*; Ofra Kalter-Leibovici (The Gertner Institute for Epidemiology and Health Policy Research, Israel)*; Yves Kameli (French National Research Institute for Sustainable Development, France)*; Kodanda R Kanala (Sri Venkateswara University, India)*; Srinivasan Kannan (Sree Chitra Tirunal Institute for Medical Sciences and Technology, India)*; Efthymios Kapantais (Hellenic Medical Association for Obesity, Greece)*; Khem B Karki (Maharajgunj Medical Campus, Nepal)*; Marzieh Katibeh (Aarhus University, Denmark)*; Joanne Katz (Johns Hopkins Bloomberg School of Public Health, USA)*; Peter T Katzmarzyk (Pennington Biomedical Research Center, USA)*; Jussi Kauhanen (University of Eastern Finland, Finland)*; Prabhdeep Kaur (National Institute of Epidemiology, India)*; Maryam Kavousi (Erasmus Medical Center Rotterdam, The Netherlands)*; Gyulli M Kazakbaeva (Ufa Eye Research Institute, Russia)*; Ulrich Keil (University of Münster, Germany)*; Lital Keinan Boker (Israel Center for Disease Control, Israel)*; Sirkka Keinänen-Kiukaanniemi (Oulu University Hospital, Finland)*; Roya Kelishadi (Research Institute for Primordial Prevention of Non-communicable Disease, Iran)*; Cecily Kelleher (University College Dublin, Ireland)*; Han CG Kemper (Amsterdam UMC Public Health Research Institute, The Netherlands)*; Andre P Kengne (South African Medical Research Council, South Africa)*; Maryam Keramati (Mashhad University of Medical Sciences, Iran)*; Alina Kerimkulova (Kyrgyz State Medical Academy, Kyrgyzstan)*; Mathilde Kersting (Research Institute of Child Nutrition, Germany)*; Timothy Key (University of Oxford, UK)*; Yousef Saleh Khader (Jordan University of Science and Technology, Jordan)*; Davood Khalili (Shahid Beheshti University of Medical Sciences, Iran)*; Young-Ho Khang (Seoul National University, Republic of Korea)*; Kay-Tee Khaw (University of Cambridge, UK)*; Bahareh Kheiri (Shahid Beheshti University of Medical Sciences, Iran)*; Motahareh Kheradmand (Mazandaran University of Medical Sciences, Iran)*; Alireza Khosravi (Hypertension Research Center, Iran)*; Ilse MSL Khouw (FrieslandCampina, The Netherlands)*; Ursula Kiechl-Kohlendorfer (Medical University of Innsbruck, Austria)*; Stefan Kiechl (Medical University of Innsbruck, Austria; VASCage, Austria)*; Japhet Killewo (Muhimbili University of Health and Allied Sciences, Tanzania)*; Dong Wook Kim

(National Health Insurance Service, Republic of Korea)*; Hyeon Chang Kim (Yonsei University College of Medicine, Republic of Korea)*; Jeongseon Kim (National Cancer Center, Republic of Korea)*; Jenny M Kindblom (University of Gothenburg, Sweden; Sahlgrenska University Hospital, Sweden)*; Heidi Klakk (University College South Denmark, Denmark)*; Magdalena Klimek (Jagiellonian University Medical College, Poland)*; Jeannette Klimont (Statistics Austria, Austria)*; Jurate Klumbiene (Lithuanian University of Health Sciences, Lithuania)*; Michael Knoflach (Medical University of Innsbruck, Austria)*; Bhawesh Koirala (B P Koirala Institute of Health Sciences, Nepal)*; Elin Kolle (Norwegian School of Sport Sciences, Norway)*; Patrick Kolsteren (Ghent University, Belgium)*; Jürgen König (University of Vienna, Austria)*; Raija Korpelainen (University of Oulu, Finland; Oulu Deaconess Institute Foundation sr, Finland)*; Paul Korrovits (Tartu University Clinics, Estonia)*; Magdalena Korzycka (Institute of Mother and Child, Poland)*; Jelena Kos (University Hospital Center Zagreb, Croatia)*; Seppo Koskinen (Finnish Institute for Health and Welfare, Finland)*; Katsuyasu Kouda (Kansai Medical University, Japan)*; Viktoria A Kovacs (Hungarian School Sport Federation, Hungary)*; Sudhir Kowlessur (Ministry of Health and Quality of Life, Mauritius)*; Slawomir Koziel (PASs Hirszfeld Institute of Immunology and Experimental Therapy, Poland)*; Wolfgang Kratzer (University Hospital Ulm, Germany)*; Susi Kriemler (University of Zurich, Switzerland)*; Peter Lund Kristensen (University of Southern Denmark, Denmark)*; Steiner Krokstad (Norwegian University of Science and Technology, Norway)*; Daan Kromhout (University of Groningen, The Netherlands)*; Branimir Krtalic (University Hospital Center Zagreb, Croatia)*; Herculina S Kruger (North-West University, South Africa)*; Ruzena Kubinova (National Institute of Public Health, Czech Republic)*; Renata Kuciene (Lithuanian University of Health Sciences, Lithuania)*; Urho M Kujala (University of Jyväskylä, Finland)*; Enisa Kujundzic (Institute of Public Health of Montenegro, Montenegro)*; Zbigniew Kulaga (Children's Memorial Health Institute, Poland)*; R Krishna Kumar (Amrita Institute of Medical Sciences, India)*; Marie Kunešová (Institute of Endocrinology, Czech Republic)*; Pawel Kurjata (National Institute of Cardiology, Poland)*; Yadlapalli S Kusuma (All India Institute of

Medical Sciences, India)*; Kari Kuulasmaa (Finnish Institute for Health and Welfare, Finland)*; Catherine Kyobutungi (African Population and Health Research Center, Kenya)*; Quang Ngoc La (Hanoi University of Public Health, Vietnam)*; Fatima Zahra Laamiri (Hassan First University of Settat, Morocco)*; Tiina Laatikainen (Finnish Institute for Health and Welfare, Finland)*; Carl Lachat (Ghent University, Belgium)*; Youcef Laid (Ministry of Health, Algeria)*; Tai Hing Lam (University of Hong Kong, China)*; Christina-Paulina Lambrinou (Harokopio University, Greece)*; Edwige Landais (French National Research Institute for Sustainable Development, France)*; Vera Lanska (Institute for Clinical and Experimental Medicine, Czech Republic)*; Georg Lappas (Sahlgrenska Academy, Sweden)*; Bagher Larijani (Endocrinology and Metabolism Research Center, Iran)*; Tint Swe Latt (University of Public Health, Myanmar)*; Laura Lauria (Istituto Superiore di Sanità, Italy)*; Avula Laxmaiah (ICMR - National Institute of Nutrition, India)*; Maria Lazo-Porras (Universidad Peruana Cayetano Heredia, Peru)*; Khanh Le Nguyen Bao (National Institute of Nutrition, Vietnam)*; Agnès Le Port (International Food Policy Research Institute, Senegal)*; Tuyen D Le (National Institute of Nutrition, Vietnam)*; Jeannette Lee (National University of Singapore, Singapore)*; Jeonghee Lee (National Cancer Center, Republic of Korea)*; Paul H Lee (Hong Kong Polytechnic University, China)*; Nils Lehmann (University of Duisburg-Essen, Germany)*; Terho Lehtimäki (Tampere University Hospital, Finland; Tampere University, Finland)*; Daniel Lemogoum (University of Douala, Cameroon)*; Naomi S Levitt (University of Cape Town, South Africa)*; Yanping Li (Harvard TH Chan School of Public Health, USA)*; Merike Liivak (National Institute for Health Development, Estonia)*; Christa L Lilly (West Virginia University, USA)*; Wei-Yen Lim (National University of Singapore, Singapore)*; M Fernanda Lima-Costa (Oswaldo Cruz Foundation Rene Rachou Research Institute, Brazil)*; Hsien-Ho Lin (National Taiwan University, Taiwan)*; Xu Lin (University of Chinese Academy of Sciences, China)*; Yi-Ting Lin (Uppsala University, Sweden)*; Lars Lind (Uppsala University, Sweden)*; Allan Linneberg (Bispebjerg and Frederiksberg Hospital, Denmark)*; Lauren Lissner (University of Gothenburg, Sweden)*; Mieczyslaw Litwin (Children's Memorial Health Institute,

Poland)*; Jing Liu (Capital Medical University Beijing An Zhen Hospital, China)*; Lijuan Liu (Capital Medical University Beijing Tongren Hospital, China)*; Wei-Cheng Lo (Taipei Medical University, Taiwan)*; Helle-Mai Loit (National Institute for Health Development, Estonia)*; Khuong Quynh Long (Hanoi University of Public Health, Vietnam)*; Luis Lopes (University of Porto, Portugal)*; Oscar Lopes (Sports Medical Center of Minho, Portugal)*; Esther Lopez-Garcia (Universidad Autónoma de Madrid/CIBERESP, Spain)*; Tania Lopez (Universidad San Martín de Porres, Peru)*; Paulo A Lotufo (University of São Paulo, Brazil)*; José Eugenio Lozano (Consejería de Sanidad Junta de Castilla y León, Spain)*; Janice L Lukrafka (Universidade Federal de Ciências da Saúde de Porto Alegre, Brazil)*; Dalia Luksiene (Lithuanian University of Health Sciences, Lithuania)*; Annamari Lundqvist (Finnish Institute for Health and Welfare, Finland)*; Robert Lundqvist (Norrbotten County Council, Sweden)*; Nuno Lunet (University of Porto, Portugal)*; Charles Lunogelo (Ilembula Lutheran Hospital, Tanzania)*; Michala Lustigová (Charles University, Czech Republic; National Institute of Public Health, Czech Republic)*; Edyta Łuszczki (University of Rzeszów, Poland)*; Guansheng Ma (Peking University, China)*; Jun Ma (Peking University, China)*; Xu Ma (National Research Institute for Health and Family Planning, China)*; George LL Machado-Coelho (Universidade Federal de Ouro Preto, Brazil)*; Aristides M Machado-Rodrigues (University of Coimbra, Portugal)*; Suka Machi (The Jikei University School of Medicine, Japan)*; Luisa M Macieira (Coimbra University Hospital Center, Portugal)*; Ahmed A Madar (University of Oslo, Norway)*; Stefania Maggi (Institute of Neuroscience of the National Research Council, Italy)*; Dianna J Magliano (Baker Heart and Diabetes Institute, Australia)*; Sara Magnacca (Mediterranea Cardiocentro, Italy)*; Emmanuella Magriplis (Agricultural University of Athens, Greece)*; Gowri Mahasampath (Christian Medical College, India)*; Bernard Maire (French National Research Institute for Sustainable Development, France)*; Marjeta Majer (University of Zagreb, Croatia)*; Marcia Makdisse (Hospital Israelita Albert Einstein, Brazil)*; Päivi Mäki (Finnish Institute for Health and Welfare, Finland)*; Fatemeh Malekzadeh (Tehran University of Medical Sciences, Iran)*; Reza Malekzadeh (Tehran University of Medical Sciences, Iran)*;

Rahul Malhotra (Duke-NUS Medical School, Singapore)*; Kodavanti Mallikharjuna Rao (ICMR - National Institute of Nutrition, India)*; Sofia K Malyutina (SB RAS Federal Research Center Institute of Cytology and Genetics, Russia)*; Lynell V Maniego (Food and Nutrition Research Institute, Philippines)*; Yannis Manios (Harokopio University, Greece)*; Jim I Mann (University of Otago, New Zealand)*; Fariborz Mansour-Ghanaei (Guilan University of Medical Sciences, Iran)*; Enzo Manzato (University of Padua, Italy)*; Paula Margozzini (Pontificia Universidad Católica de Chile, Chile)*; Anastasia Markaki (Hellenic Mediterranean University, Greece)*; Oonagh Markey (Loughborough University, UK)*; Eliza Markidou Ioannidou (Ministry of Health, Cyprus)*; Pedro Marques-Vidal (Lausanne University Hospital, Switzerland)*; Larissa Pruner Marques (Escola Nacional de Saúde Pública Sergio Arouca, Brazil)*; Jaume Marrugat (CIBERCV, Spain; Institut Hospital del Mar d'Investigacions Mèdiques, Spain)*; Yves Martin-Prevel (French National Research Institute for Sustainable Development, France)*; Rosemarie Martin (Mary Immaculate College, Ireland)*; Reynaldo Martorell (Emory University, USA)*; Eva Martos (Hungarian Society of Sports Medicine, Hungary)*; Stefano Marventano (University of Catania, Italy)*; Luis P Mascarenhas (Universidade Estadual do Centro-Oeste, Brazil)*; Shariq R Masoodi (Sher-i-Kashmir Institute of Medical Sciences, India)*; Ellisiv B Mathiesen (UiT The Arctic University of Norway, Norway)*; Prashant Mathur (ICMR - National Centre for Disease Informatics and Research, India)*; Alicia Matijasevich (University of São Paulo, Brazil)*; Tandi E Matsha (Cape Peninsula University of Technology, South Africa)*; Christina Mavrogianni (Harokopio University, Greece)*; Artur Mazur (University of Rzeszów, Poland)*; Jean Claude N Mbanya (University of Yaoundé 1, Cameroon)*; Shelly R McFarlane (The University of the West Indies, Jamaica)*; Stephen T McGarvey (Brown University, USA)*; Martin McKee (London School of Hygiene & Tropical Medicine, UK)*; Stela McLachlan (University of Edinburgh, UK)*; Rachael M McLean (University of Otago, New Zealand)*; Scott B McLean (Statistics Canada, Canada)*; Breige A McNulty (University College Dublin, Ireland)*; Sounnia Mediene-Benchekor (University Oran 1, Algeria)*; Jurate Medzioniene (Lithuanian University of Health Sciences, Lithuania)*; Parinaz

Mehdipour (Non-Communicable Diseases Research Center, Iran)*; Kirsten Mehlig (University of Gothenburg, Sweden)*; Amir Houshang Mehrparvar (Shahid Sadoughi University of Medical Sciences, Iran)*; Aline Meirhaeghe (Institut National de la Santé et de la Recherche Médicale, France)*; Jørgen Meisfjord (Norwegian Institute of Public Health, Norway)*; Christa Meisinger (Helmholtz Zentrum München, Germany)*; Ana Maria B Menezes (Federal University of Pelotas, Brazil)*; Geetha R Menon (ICMR - National Institute of Medical Statistics, India)*; Gert BM Mensink (Robert Koch Institute, Germany)*; Maria Teresa Menzano (Ministero della Salute DG Prevenzione Sanitaria, Italy)*; Alibek Mereke (Al-Farabi Kazakh National University, Kazakhstan)*; Indrapal I Meshram (ICMR - National Institute of Nutrition, India)*; Andres Metspalu (University of Tartu, Estonia)*; Jie Mi (Capital Institute of Pediatrics, China)*; Kim F Michaelsen (University of Copenhagen, Denmark)*; Nathalie Michels (Ghent University, Belgium)*; Kairit Mikkel (University of Tartu, Estonia)*; Karolina Milkowska (Jagiellonian University Medical College, Poland)*; Jody C Miller (University of Otago, New Zealand)*; Cláudia S Minderico (Universidade de Lisboa, Portugal)*; GK Mini (Women's Social and Health Studies Foundation, India)*; Juan Francisco Miquel (Pontificia Universidad Católica de Chile, Chile)*; J Jaime Miranda (Universidad Peruana Cayetano Heredia, Peru)*; Mohammad Reza Mirjalili (Shahid Sadoughi University of Medical Sciences, Iran)*; Daphne Mirkopoulou (Democritus University, Greece)*; Erkin Mirrakhimov (Kyrgyz State Medical Academy, Kyrgyzstan)*; Marjeta Mišigoj-Durakovic (University of Zagreb, Croatia)*; Antonio Mistretta (University of Catania, Italy)*; Veronica Mocanu (Grigore T Popa University of Medicine and Pharmacy, Romania)*; Pietro A Modesti (Università degli Studi di Firenze, Italy)*; Sahar Saeedi Moghaddam (Non-Communicable Diseases Research Center, Iran)*; Bahram Mohajer (Non-Communicable Diseases Research Center, Iran)*; Mostafa K Mohamed (Ain Shams University, Egypt)*; Shukri F Mohamed (African Population and Health Research Center, Kenya)*; Kazem Mohammad (Tehran University of Medical Sciences, Iran)*; Zahra Mohammadi (Tehran University of Medical Sciences, Iran)*; Noushin Mohammadifard (Hypertension Research Center, Iran)*; Reza Mohammadpourhodki

(Mashhad University of Medical Sciences, Iran)*; Viswanathan Mohan (Madras Diabetes Research Foundation, India)*; Salim Mohanna (Universidad Peruana Cayetano Heredia, Peru)*; Muhammad Fadhli Mohd Yusoff (Ministry of Health, Malaysia)*; Iraj Mohebbi (Urmia University of Medical Sciences, Iran)*; Farnam Mohebi (Non-Communicable Diseases Research Center, Iran)*; Marie Moitry (University of Strasbourg, France; Strasbourg University Hospital, France)*; Drude Molbo (University of Copenhagen, Denmark)*; Line T Møllehave (Bispebjerg and Frederiksberg Hospital, Denmark)*; Niels C Møller (University of Southern Denmark, Denmark)*; Dénes Molnár (University of Pécs, Hungary)*; Amirabbas Momenan (Shahid Beheshti University of Medical Sciences, Iran)*; Charles K Mondo (Mulago Hospital, Uganda)*; Michele Monroy-Valle (University of San Carlos of Guatemala, Guatemala)*; Eric Monterrubio-Flores (National Institute of Public Health, Mexico)*; Kotsedi Daniel K Monyeki (University of Limpopo, South Africa)*; Jin Soo Moon (Seoul National University, Republic of Korea)*; Mahmood Moosazadeh (Mazandaran University of Medical Sciences, Iran)*; Leila B Moreira (Universidade Federal do Rio Grande do Sul, Brazil)*; Alain Morejon (University of Medical Sciences of Cienfuegos, Cuba)*; Luis A Moreno (University of Zaragoza, Spain; CIBEROBN, Spain)*; Karen Morgan (Royal College of Surgeons in Ireland, Ireland)*; Suzanne N Morin (McGill University, Canada)*; Erik Lykke Mortensen (University of Copenhagen, Denmark)*; George Moschonis (La Trobe University, Australia)*; Malgorzata Mossakowska (International Institute of Molecular and Cell Biology, Poland)*; Aya Mostafa (Ain Shams University, Egypt)*; Anabela Mota-Pinto (University of Coimbra, Portugal)*; Jorge Mota (University of Porto, Portugal)*; Mohammad Esmaeel Motlagh (Ahvaz Jundishapur University of Medical Sciences, Iran)*; Jorge Motta (Instituto Conmemorativo Gorgas de Estudios de la Salud, Panama)*; Marcos André Moura-dos-Santos (University of Pernambuco, Brazil)*; Malay K Mridha (BRAC James P Grant School of Public Health, Bangladesh)*; Kelias P Msyamboza (World Health Organization Country Office, Malawi)*; Thet Thet Mu (Department of Public Health, Myanmar)*; Magdalena Muc (University of Coimbra, Portugal)*; Boban Mugoša (Institute of Public Health of Montenegro, Montenegro)*; Maria L Muiesan (University of Brescia,

Italy)*; Parvina Mukhtorova (Ministry of Health and Social Protection, Tajikistan)*; Martina Müller-Nurasyid (Helmholtz Zentrum München, Germany)*; Neil Murphy (International Agency for Research on Cancer, France)*; Jaakko Mursu (University of Eastern Finland, Finland)*; Elaine M Murtagh (University of Limerick, Ireland)*; Kamarul Imran Musa (Universiti Sains Malaysia, Malaysia)*; Sanja Music Milanovic (Croatian Institute of Public Health, Croatia; University of Zagreb, Croatia)*; Vera Musil (University of Zagreb, Croatia)*; Norlaila Mustafa (Universiti Kebangsaan Malaysia, Malaysia)*; Iraj Nabipour (Bushehr University of Medical Sciences, Iran)*; Shohreh Naderimagham (Non-Communicable Diseases Research Center, Iran)*; Gabriele Nagel (Ulm University, Germany)*; Balkish M Naidu (Department of Statistics, Malaysia)*; Farid Najafi (Kermanshah University of Medical Sciences, Iran)*; Harunobu Nakamura (Kobe University, Japan)*; Jana Námešná (Banska Bystrica Regional Authority of Public Health, Slovakia)*; Ei Ei K Nang (National University of Singapore, Singapore)*; Vinay B Nangia (Suraj Eye Institute, India)*; Martin Nankap (UNICEF, Cameroon)*; Sameer Narake (Healis-Sekhsaria Institute for Public Health, India)*; Paola Nardone (Istituto Superiore di Sanità, Italy)*; Matthias Nauck (University Medicine Greifswald, Germany)*; William A Neal (West Virginia University, USA)*; Azim Nejatizadeh (Hormozgan University of Medical Sciences, Iran)*; Keiu Nelis (National Institute for Health Development, Estonia)*; Liis Nelis (National Institute for Health Development, Estonia)*; Ilona Nenko (Jagiellonian University Medical College, Poland)*; Martin Neovius (Karolinska Institutet, Sweden)*; Flavio Nervi (Pontificia Universidad Católica de Chile, Chile)*; Chung T Nguyen (National Institute of Hygiene and Epidemiology, Vietnam)*; Nguyen D Nguyen (University of Medicine and Pharmacy at Ho Chi Minh City, Vietnam)*; Quang Ngoc Nguyen (Hanoi Medical University, Vietnam)*; Ramfis E Nieto-Martínez (LifeDoc Health, USA)*; Yury P Nikitin (SB RAS Federal Research Center Institute of Cytology and Genetics, Russia)*; Guang Ning (Shanghai Jiao-Tong University School of Medicine, China)*; Toshiharu Ninomiya (Kyushu University, Japan)*; Sania Nishtar (Heartfile, Pakistan)*; Marianna Noale (Institute of Neuroscience of the National Research Council, Italy)*; Oscar A Noboa (Republic University,

Uruguay)*; Helena Nogueira (University of Coimbra, Portugal)*; Teresa Norat (Imperial College London, UK)*; Maria Nordendahl (Umeå University, Sweden)*; Børge G Nordestgaard (Copenhagen University Hospital, Denmark; University of Copenhagen, Denmark)*; Davide Noto (University of Palermo, Italy)*; Natalia Nowak-Szczepanska (PASs Hirszfeld Institute of Immunology and Experimental Therapy, Poland)*; Mohannad Al Nsour (Eastern Mediterranean Public Health Network, Jordan)*; Irfan Nuhoglu (Karadeniz Technical University, Turkey)*; Eha Nurk (National Institute for Health Development, Estonia)*; Terence W O'Neill (University of Manchester, UK)*; Dermot O'Reilly (Queen's University of Belfast, UK)*; Galina Obreja (State University of Medicine and Pharmacy, Moldova)*; Caleb Ochimana (Harvard TH Chan School of Public Health, USA)*; Angélica M Ochoa-Avilés (Universidad de Cuenca, Ecuador)*; Eiji Oda (Tachikawa General Hospital, Japan)*; Kyungwon Oh (Korea Centers for Disease Control and Prevention, Republic of Korea)*; Kumiko Ohara (Kindai University, Japan)*; Claes Ohlsson (University of Gothenburg, Sweden; Sahlgrenska University Hospital, Sweden)*; Ryutaro Ohtsuka (Japan Wildlife Research Center, Japan)*; Örn Olafsson (Icelandic Heart Association, Iceland)*; Maria Teresa A Olinto (Universidade do Vale do Rio dos Sinos, Brazil)*; Isabel O Oliveira (Federal University of Pelotas, Brazil)*; Mohd Azahadi Omar (Ministry of Health, Malaysia)*; Altan Onat (Istanbul University, Turkey; deceased)*; Sok King Ong (Ministry of Health, Brunei)*; Lariane M Ono (Universidade Federal do Paraná, Brazil)*; Pedro Ordunez (Pan American Health Organization, USA)*; Rui Ornelas (University of Madeira, Portugal)*; Ana P Ortiz (University of Puerto Rico, Puerto Rico)*; Pedro J Ortiz (Universidad Peruana Cayetano Heredia, Peru)*; Merete Osler (Center for Clinical Research and Prevention, Denmark)*; Clive Osmond (MRC Lifecourse Epidemiology Unit, UK)*; Sergej M Ostojic (University of Novi Sad, Serbia)*; Afshin Ostovar (Tehran University of Medical Sciences, Iran)*; Johanna A Otero (Fundación Oftalmológica de Santander, Colombia)*; Kim Overvad (Aarhus University, Denmark)*; Ellis Owusu-Dabo (Kwame Nkrumah University of Science and Technology, Ghana)*; Fred Michel Paccaud (UniSanté, Switzerland)*; Cristina Padez (University of Coimbra, Portugal)*; Ioannis

Pagkalos (International Hellenic University, Greece)*; Elena Pahomova (University of Latvia, Latvia)*; Karina Mary de Paiva (Federal University of Santa Catarina, Brazil)*; Andrzej Pajak (Jagiellonian University Medical College, Poland)*; Domenico Palli (Institute for Cancer Research, Prevention and Clinical Network, Italy)*; Alberto Palloni (University of Wisconsin-Madison, USA)*; Luigi Palmieri (Istituto Superiore di Sanità, Italy)*; Wen-Harn Pan (Academia Sinica, Taiwan)*; Songhomitra Panda-Jonas (Ruprecht-Karls-University of Heidelberg, Germany)*; Arvind Pandey (ICMR - National Institute of Medical Statistics, India)*; Francesco Panza (IRCCS Ente Ospedaliero Specializzato in Gastroenterologia S. de Bellis, Italy)*; Dimitrios Papandreou (Zayed University, United Arab Emirates)*; Soon-Woo Park (Catholic University of Daegu, Republic of Korea)*; Suyeon Park (Korea Centers for Disease Control and Prevention, Republic of Korea)*; Winsome R Parnell (University of Otago, New Zealand)*; Mahboubeh Parsaeian (Tehran University of Medical Sciences, Iran)*; Ionela M Pascanu (University of Medicine, Pharmacy, Science and Technology of Târgu Mures, Romania)*; Patrick Pasquet (UMR CNRS-MNHN 7206 Eco-anthropologie, France)*; Nikhil D Patel (Jivandeep Hospital, India)*; Mangesh S Pednekar (Healis-Sekhsaria Institute for Public Health, India)*; Nasheeta Peer (South African Medical Research Council, South Africa)*; Sergio Viana Peixoto (Oswaldo Cruz Foundation Rene Rachou Research Institute, Brazil)*; Markku Peltonen (Finnish Institute for Health and Welfare, Finland)*; Alexandre C Pereira (University of São Paulo, Brazil)*; Marco A Peres (National Dental Care Centre Singapore, Singapore)*; Napoleón Pérez-Farinós (University of Malaga, Spain)*; Cynthia M Pérez (University of Puerto Rico, Puerto Rico)*; Valentina Peterkova (Endocrinology Research Centre, Russia)*; Annette Peters (Helmholtz Zentrum München, Germany)*; Astrid Petersmann (University Medicine Greifswald, Germany)*; Janina Petkeviciene (Lithuanian University of Health Sciences, Lithuania)*; Ausra Petrauskiene (Lithuanian University of Health Sciences, Lithuania)*; Emanuela Pettenuzzo (University Hospital of Varese, Italy)*; Niloofar Peykari (Ministry of Health and Medical Education, Iran)*; Son Thai Pham (Vietnam National Heart Institute, Vietnam)*; Rafael N Pichardo (Clínica de Medicina Avanzada Dr. Abel González, Dominican Republic)*;

Daniela Pierannunzio (Istituto Superiore di Sanità, Italy)*; Iris Pigeot (Leibniz Institute for Prevention Research and Epidemiology - BIPS, Germany)*; Hynek Pikhart (University College London, UK)*; Aida Pilav (University of Sarajevo, Bosnia and Herzegovina)*; Lorenza Pilotto (Cardiovascular Prevention Centre Udine, Italy)*; Francesco Pistelli (University Hospital of Pisa, Italy)*; Freda Pitakaka (Ministry of Health and Medical Services, Solomon Islands)*; Aleksandra Piwonska (National Institute of Cardiology, Poland)*; Andreia N Pizarro (University of Porto, Portugal)*; Pedro Plans-Rubió (Public Health Agency of Catalonia, Spain)*; Bee Koon Poh (Universiti Kebangsaan Malaysia, Malaysia)*; Hermann Pohlabeln (Leibniz Institute for Prevention Research and Epidemiology - BIPS, Germany)*; Raluca M Pop (University of Medicine, Pharmacy, Science and Technology of Târgu Mures, Romania)*; Stevo R Popovic (University of Montenegro, Montenegro)*; Miquel Porta (Institut Hospital del Mar d'Investigacions Mèdiques, Spain)*; Georg Posch (Agency for Preventive and Social Medicine, Austria)*; Anil Poudyal (Nepal Health Research Council, Nepal)*; Dimitrios Poulimeneas (International Hellenic University, Greece)*; Hamed Pouraram (Tehran University of Medical Sciences, Iran)*; Farhad Pourfarzi (Ardabil University of Medical Sciences, Iran)*; Akram Pourshams (Tehran University of Medical Sciences, Iran)*; Hossein Poustchi (Tehran University of Medical Sciences, Iran)*; Rajendra Pradeepa (Madras Diabetes Research Foundation, India)*; Alison J Price (London School of Hygiene & Tropical Medicine, UK)*; Jacqueline F Price (University of Edinburgh, UK)*; Rui Providencia (University College London, UK)*; Jardena J Puder (Lausanne University Hospital, Switzerland)*; Iveta Pudule (Centre for Disease Prevention and Control, Latvia)*; Soile E Puhakka (University of Oulu, Finland; Oulu Deaconess Institute Foundation sr, Finland)*; Maria Puiu (Victor Babes University of Medicine and Pharmacy Timisoara, Romania)*; Margus Punab (Tartu University Clinics, Estonia)*; Radwan F Qasrawi (Al-Quds University, Palestine)*; Mostafa Qorbani (Alborz University of Medical Sciences, Iran)*; Tran Quoc Bao (Ministry of Health, Vietnam)*; Ivana Radic (University of Novi Sad, Serbia)*; Ricardas Radisauskas (Lithuanian University of Health Sciences, Lithuania)*; Salar Rahimikazerooni (Shiraz University of Medical

Sciences, Iran)*; Mahfuzar Rahman (Centre for Injury Prevention and Research in Bangladesh, Bangladesh)*; Mahmudur Rahman (Institute of Epidemiology Disease Control and Research, Bangladesh)*; Olli Raitakari (University of Turku, Finland)*; Manu Raj (Amrita Institute of Medical Sciences, India)*; Ellina Rakhimova (Ufa Eye Research Institute, Russia)*; Sherali Rakhmatulloev (Ministry of Health and Social Protection, Tajikistan)*; Ivo Rakovac (World Health Organization Regional Office for Europe, Russia)*; Sudha Ramachandra Rao (National Institute of Epidemiology, India)*; Ambady Ramachandran (India Diabetes Research Foundation, India)*; Jacqueline Ramke (University of Auckland, New Zealand)*; Elisabete Ramos (University of Porto Medical School, Portugal)*; Rafel Ramos (Institut Universitari d'Investigació en Atenció Primària Jordi Gol, Spain)*; Lekhraj Rampal (Universiti Putra Malaysia, Malaysia)*; Sanjay Rampal (University of Malaya, Malaysia)*; Vayia Rarra (Sotiria Hospital, Greece)*; Ramon A Rascon-Pacheco (Instituto Mexicano del Seguro Social, Mexico)*; Mette Rasmussen (University of Southern Denmark, Denmark)*; Cassiano Ricardo Rech (Federal University of Santa Catarina, Brazil)*; Josep Redon (University of Valencia, Spain)*; Paul Ferdinand M Reganit (University of the Philippines, Philippines)*; Valéria Regecová (Slovak Academy of Sciences, Slovakia)*; Luis Revilla (Universidad San Martín de Porres, Peru)*; Abbas Rezaianzadeh (Shiraz University of Medical Sciences, Iran)*; Lourdes Ribas-Barba (Nutrition Research Foundation, Spain)*; Robespierre Ribeiro (Minas Gerais State Secretariat for Health, Brazil; deceased)*; Elio Riboli (Imperial College London, UK)*; Adrian Richter (University Medicine Greifswald, Germany)*; Fernando Rigo (CS S. Agustín Ibsalut, Spain)*; Natascia Rinaldo (University of Ferrara, Italy)*; Tobias F Rinke de Wit (Amsterdam Institute for Global Health and Development, The Netherlands)*; Ana Rito (National Institute of Health Doutor Ricardo Jorge, Portugal)*; Raphael M Ritti-Dias (Universidade Nove de Julho, Brazil)*; Juan A Rivera (National Institute of Public Health, Mexico)*; Cynthia Robitaille (Public Health Agency of Canada, Canada)*; Romana Roccaldo (Council for Agricultural Research and Economics, Italy)*; Daniela Rodrigues (University of Coimbra, Portugal)*; Fernando Rodríguez-Artalejo (Universidad Autónoma de

Madrid/CIBERESP, Spain)*; María del Cristo Rodriguez-Perez (Canarian Health Service, Spain)*; Laura A Rodríguez-Villamizar (Universidad Industrial de Santander, Colombia)*; Ulla Roggenbuck (University of Duisburg-Essen, Germany)*; Rosalba Rojas-Martinez (National Institute of Public Health, Mexico)*; Nipa Rojroongwasinkul (Mahidol University, Thailand)*; Dora Romaguera (CIBEROBN, Spain)*; Elisabetta L Romeo (Associazione Calabrese di Epatologia, Italy)*; Rafaela V Rosario (University of Minho, Portugal)*; Annika Rosengren (University of Gothenburg, Sweden; Sahlgrenska University Hospital, Sweden)*; Ian Rouse (Fiji National University, Fiji)*; Joel GR Roy (Statistics Canada, Canada)*; Adolfo Rubinstein (Institute for Clinical Effectiveness and Health Policy, Argentina)*; Frank J Rühli (University of Zurich, Switzerland)*; Jean-Bernard Ruidavets (Toulouse University School of Medicine, France)*; Blanca Sandra Ruiz-Betancourt (Instituto Mexicano del Seguro Social, Mexico)*; Emma Ruiz Moreno (Spanish Nutrition Foundation, Spain)*; Iuliia A Rusakova (Ufa Eye Research Institute, Russia)*; Kenisha Russell Jonsson (Public Health Agency of Sweden, Sweden)*; Paola Russo (Institute of Food Sciences of the National Research Council, Italy)*; Petra Rust (University of Vienna, Austria)*; Marcin Rutkowski (Medical University of Gdansk, Poland)*; Charumathi Sabanayagam (Singapore Eye Research Institute, Singapore)*; Elena Sacchini (Authority Sanitaria San Marino, San Marino)*; Harshpal S Sachdev (Sitaram Bhartia Institute of Science and Research, India)*; Alireza Sadjadi (Tehran University of Medical Sciences, Iran)*; Ali Reza Safarpour (Shiraz University of Medical Sciences, Iran)*; Sare Safi (Shahid Beheshti University of Medical Sciences, Iran)*; Saeid Safiri (Tabriz University of Medical Sciences, Iran)*; Olfa Saidi (University Tunis El Manar, Tunisia)*; Nader Saki (Ahvaz Jundishapur University of Medical Sciences, Iran)*; Benoit Salanave (French Public Health Agency, France)*; Eduardo Salazar Martinez (National Institute of Public Health, Mexico)*; Diego Salmerón (CIBER en Epidemiología y Salud Pública, Spain)*; Veikko Salomaa (Finnish Institute for Health and Welfare, Finland)*; Jukka T Salonen (University of Helsinki, Finland)*; Massimo Salvetti (University of Brescia, Italy)*; Margarita Samoutian (Kindergarten of Avlonari, Greece)*; Jose Sánchez-Abanto (National

Institute of Health, Peru)*; Sandjaja (Ministry of Health, Indonesia)*; Susana Sans (Catalan Department of Health, Spain)*; Loreto Santa Marina (Biodonostia Health Research Institute, Spain)*; Diana A Santos (Universidade de Lisboa, Portugal)*; Ina S Santos (Federal University of Pelotas, Brazil)*; Lèlita C Santos (Coimbra University Hospital Center, Portugal)*; Maria Paula Santos (University of Porto, Portugal)*; Osvaldo Santos (Instituto de Saúde Ambiental, Portugal)*; Rute Santos (University of Porto, Portugal)*; Sara Santos Sanz (Spanish Food Safety and Nutrition Agency, Spain)*; Jouko L Saramies (South Karelia Social and Health Care District, Finland)*; Luis B Sardinha (Universidade de Lisboa, Portugal)*; Nizal Sarrafzadegan (Isfahan Cardiovascular Research Center, Iran)*; Thirunavukkarasu Sathish (McMaster University, Canada)*; Kai-Uwe Saum (German Cancer Research Center, Germany)*; Savvas Savva (Research and Education Institute of Child Health, Cyprus)*; Mathilde Savy (French National Research Institute for Sustainable Development, France)*; Norie Sawada (National Cancer Center, Japan)*; Mariana Sbaraini (Universidade Federal do Rio Grande do Sul, Brazil)*; Marcia Scazufca (University of São Paulo Clinics Hospital, Brazil)*; Beatriz D Schaan (Universidade Federal do Rio Grande do Sul, Brazil)*; Angelika Schaffrath Rosario (Robert Koch Institute, Germany)*; Herman Schargrodsky (Hospital Italiano de Buenos Aires, Argentina)*; Anja Schienkiewitz (Robert Koch Institute, Germany)*; Karin Schindler (Medical University of Vienna, Austria)*; Sabine Schipf (University Medicine Greifswald, Germany)*; Carsten O Schmidt (University Medicine Greifswald, Germany)*; Ida Maria Schmidt (Rigshospitalet, Denmark)*; Peter Schnohr (Copenhagen University Hospital, Denmark)*; Ben Schöttker (German Cancer Research Center, Germany)*; Sara Schramm (University of Duisburg-Essen, Germany)*; Stine Schramm (University of Southern Denmark, Denmark)*; Helmut Schröder (CIBER en Epidemiología y Salud Pública, Spain)*; Constance Schultsz (Academic Medical Center of University of Amsterdam, The Netherlands)*; Aletta E Schutte (University of New South Wales, Australia; The George Institute for Global Health, Australia)*; Sylvain Sebert (University of Oulu, Finland)*; Aye Aye Sein (Ministry of Health and Sports, Myanmar)*; Rusidah Selamat (Ministry of

Health, Malaysia)*; Vedrana Sember (University of Ljubljana, Slovenia)*; Abhijit Sen (Center for Oral Health Services and Research Mid-Norway, Norway)*; Idowu O Senbanjo (Lagos State University College of Medicine, Nigeria)*; Sadaf G Sepanlou (Tehran University of Medical Sciences, Iran)*; Victor Sequera (Ministry of Public Health, Paraguay)*; Luis Serra-Majem (University of Las Palmas de Gran Canaria, Spain)*; Jennifer Servais (Statistics Canada, Canada)*; Ludmila Ševcíková (Comenius University, Slovakia)*; Svetlana A Shalnova (National Research Centre for Preventive Medicine, Russia)*; Teresa Shamah-Levy (National Institute of Public Health, Mexico)*; Morteza Shamshirgaran (Neyshabur University of Medical Sciences, Iran)*; Coimbatore Subramaniam Shanthirani (Madras Diabetes Research Foundation, India)*; Maryam Sharafkhah (Tehran University of Medical Sciences, Iran)*; Sanjib K Sharma (B P Koirala Institute of Health Sciences, Nepal)*; Jonathan E Shaw (Baker Heart and Diabetes Institute, Australia)*; Amaneh Shayanrad (Tehran University of Medical Sciences, Iran)*; Ali Akbar Shayesteh (Ahvaz Jundishapur University of Medical Sciences, Iran)*; Lela Shengelia (National Center for Disease Control and Public Health, Georgia)*; Zumin Shi (Qatar University, Qatar)*; Kenji Shibuya (King's College London, UK)*; Hana Shimizu-Furusawa (Nippon Medical School, Japan)*; Dong Wook Shin (Sungkyunkwan University, Republic of Korea)*; Youchan Shin (Singapore Eye Research Institute, Singapore)*; Majid Shirani (Shahrekord University of Medical Sciences, Iran)*; Rahman Shiri (Finnish Institute of Occupational Health, Finland)*; Namuna Shrestha (Nepal Health Research Council, Nepal)*; Khairil Si-Ramlee (Ministry of Health, Brunei)*; Alfonso Siani (Institute of Food Sciences of the National Research Council, Italy)*; Rosalynn Siantar (Singapore Eye Research Institute, Singapore)*; Abla M Sibai (American University of Beirut, Lebanon)*; Antonio M Silva (Federal University of Maranhão, Brazil)*; Diego Augusto Santos Silva (Federal University of Santa Catarina, Brazil)*; Mary Simon (India Diabetes Research Foundation, India)*; Judith Simons (St Vincent's Hospital, Australia)*; Leon A Simons (University of New South Wales, Australia)*; Agneta Sjöberg (University of Gothenburg, Sweden)*; Michael Sjöström (Karolinska Institutet, Sweden)*; Gry Skodje (Nes Municipality,

Norway)*; Jolanta Slowikowska-Hilczer (Medical University of Lodz, Poland)*; Przemyslaw Slusarczyk (International Institute of Molecular and Cell Biology, Poland)*; Liam Smeeth (London School of Hygiene & Tropical Medicine, UK)*; Hung-Kwan So (University of Hong Kong, China)*; Fernanda Cunha Soares (University of Pernambuco, Brazil)*; Grzegorz Sobek (University of Rzeszów, Poland)*; Eugène Sobngwi (University of Yaoundé 1, Cameroon)*; Morten Sodemann (University of Southern Denmark, Denmark)*; Stefan Söderberg (Umeå University, Sweden)*; Moesijanti YE Soekatri (Health Polytechnic Jakarta II Institute, Indonesia)*; Agustinus Soemantri (Diponegoro University, Indonesia)*; Reecha Sofat (University College London, UK)*; Vincenzo Solfrizzi (University of Bari, Italy)*; Mohammad Hossein Somi (Tabriz University of Medical Sciences, Iran)*; Emily Sonestedt (Lund University, Sweden)*; Yi Song (Peking University, China)*; Thorkild IA Sørensen (University of Copenhagen, Denmark)*; Elin P Sørgjerd (Norwegian University of Science and Technology, Norway)*; Maroje Sorić (University of Zagreb, Croatia)*; Charles Sossa Jérome (Institut Régional de Santé Publique, Benin)*; Victoria E Soto-Rojas (Universidad Icesi, Colombia)*; Aïcha Soumaré (University of Bordeaux, France)*; Slavica Sovic (University of Zagreb School of Medicine, Croatia)*; Bente Sparboe-Nilsen (Oslo Metropolitan University, Norway)*; Karen Sparrenberger (Universidade Federal do Rio Grande do Sul, Brazil)*; Angela Spinelli (Istituto Superiore di Sanità, Italy)*; Igor Spiroski (Institute of Public Health, North Macedonia; Ss. Cyril and Methodius University, North Macedonia)*; Jan A Staessen (University of Leuven, Belgium)*; Hanspeter Stamm (Lamprecht und Stamm Sozialforschung und Beratung AG, Switzerland)*; Gregor Starc (University of Ljubljana, Slovenia)*; Maria G Stathopoulou (Institut National de la Santé et de la Recherche Médicale, France)*; Kaspar Staub (University of Zurich, Switzerland)*; Bill Stavreski (Heart Foundation, Australia)*; Jostein Steene-Johannessen (Norwegian School of Sport Sciences, Norway)*; Peter Stehle (Bonn University, Germany)*; Aryeh D Stein (Emory University, USA)*; George S Stergiou (Sotiria Hospital, Greece)*; Jochanan Stessman (Hadassah University Medical Center, Israel)*; Ranko Stevanovic (Croatian Institute of Public Health, Croatia)*; Jutta Stieber (Helmholtz Zentrum

München, Germany; deceased)*; Doris Stöckl (Helmholtz Zentrum München, Germany)*; Tanja Stocks (Lund University, Sweden)*; Jakub Stokwiszewski (National Institute of Public Health-National Institute of Hygiene, Poland)*; Ekaterina Stoyanova (Kalina Malina Kindergarten, Bulgaria)*; Gareth Stratton (Swansea University, UK)*; Karien Stronks (University of Amsterdam, The Netherlands)*; Maria Wany Strufaldi (Federal University of São Paulo, Brazil)*; Lela Sturua (National Center for Disease Control and Public Health, Georgia)*; Ramón Suárez-Medina (National Institute of Hygiene, Epidemiology and Microbiology, Cuba)*; Machi Suka (The Jikei University School of Medicine, Japan)*; Chien-An Sun (Fu Jen Catholic University, Taiwan)*; Johan Sundström (Uppsala University, Sweden)*; Yn-Tz Sung (The Chinese University of Hong Kong, China)*; Jordi Sunyer (ISGlobal Centre for Research in Environmental Epidemiology, Spain)*; Paibul Suriyawongpaisal (Mahidol University, Thailand)*; Boyd A Swinburn (University of Auckland, New Zealand)*; Rody G Sy (University of the Philippines, Philippines)*; Holly E Syddall (University of Southampton, UK)*; René Charles Sylva (National Statistical Office, Cabo Verde)*; Moyses Szklo (Johns Hopkins Bloomberg School of Public Health, USA)*; Lucjan Szponar (National Institute of Public Health-National Institute of Hygiene, Poland)*; E Shyong Tai (National University of Singapore, Singapore)*; Mari-Liis Tammesoo (University of Tartu, Estonia)*; Abdonas Tamosiunas (Lithuanian University of Health Sciences, Lithuania)*; Eng Joo Tan (University of Sydney, Australia)*; Xun Tang (Peking University, China)*; Frank Tanser (University of KwaZulu-Natal, South Africa)*; Yong Tao (Peking University, China)*; Mohammed Rasoul Tarawneh (Ministry of Health, Jordan)*; Jakob Tarp (Norwegian School of Sport Sciences, Norway)*; Carolina B Tarqui-Mamani (National Institute of Health, Peru)*; Radka Taxová Braunerová (Institute of Endocrinology, Czech Republic)*; Anne Taylor (University of Adelaide, Australia)*; Julie Taylor (University College London, UK)*; Félicité Tchibindat (UNICEF, Niger)*; William R Tebar (Universidade Estadual Paulista, Brazil)*; Grethe S Tell (University of Bergen, Norway)*; Tania Tello (Universidad Peruana Cayetano Heredia, Peru)*; KR Thankappan (Central University of Kerala, India)*; Holger Theobald (Karolinska Institutet, Sweden)*; Xenophon

Theodoridis (Aristotle University of Thessaloniki, Greece)*; Lutgarde Thijs (University of Leuven, Belgium)*; Nihal Thomas (Christian Medical College, India)*; Betina H Thuesen (Bispebjerg and Frederiksberg Hospital, Denmark)*; Lubica Tichá (Comenius University, Slovakia)*; Erik J Timmermans (Amsterdam Public Health Research Institute, The Netherlands)*; Anne Tjonneland (Danish Cancer Society Research Center, Denmark)*; Hanna K Tolonen (Finnish Institute for Health and Welfare, Finland)*; Janne S Tolstrup (University of Southern Denmark, Denmark)*; Murat Topbas (Karadeniz Technical University, Turkey)*; Roman Topór-Madry (Jagiellonian University Medical College, Poland)*; Liv Elin Torheim (Oslo Metropolitan University, Norway)*; María José Tormo (Health Service of Murcia, Spain)*; Michael J Tornaritis (Research and Education Institute of Child Health, Cyprus)*; Maties Torrent (Institut d'Investigacio Sanitaria Illes Balears, Spain)*; Laura Torres-Collado (CIBER en Epidemiología y Salud Pública, Spain)*; Stefania Toselli (University of Bologna, Italy)*; Pierre Traissac (French National Research Institute for Sustainable Development, France)*; Thi Tuyet-Hanh Tran (Hanoi University of Public Health, Vietnam)*; Dimitrios Trichopoulos (Harvard TH Chan School of Public Health, USA; deceased)*; Antonia Trichopoulou (Hellenic Health Foundation, Greece)*; Oanh TH Trinh (University of Medicine and Pharmacy at Ho Chi Minh City, Vietnam)*; Atul Trivedi (Government Medical College, India)*; Lechaba Tshepo (Sefako Makgatho Health Science University, South Africa)*; Maria Tsigga (International Hellenic University, Greece)*; Shoichiro Tsugane (National Cancer Center, Japan)*; Azaliia M Tuliakova (Ufa Eye Research Institute, Russia)*; Marshall K Tulloch-Reid (The University of the West Indies, Jamaica)*; Fikru Tullu (Addis Ababa University, Ethiopia)*; Tomi-Pekka Tuomainen (University of Eastern Finland, Finland)*; Jaakko Tuomilehto (Finnish Institute for Health and Welfare, Finland)*; Maria L Turley (Ministry of Health, New Zealand)*; Per Tynelius (Karolinska Institutet, Sweden)*; Themistoklis Tzotzas (Hellenic Medical Association for Obesity, Greece)*; Christophe Tzourio (University of Bordeaux, France)*; Peter Ueda (Karolinska Institutet, Sweden)*; Eunice Ugel (Universidad Centro-Occidental Lisandro Alvarado, Venezuela)*; Flora AM Ukoli (Meharry Medical College, USA)*; Hanno Ulmer (Medical

University of Innsbruck, Austria)*; Belgin Unal (Dokuz Eylul University, Turkey)*; Zhamyila Usupova (Republican Center for Health Promotion, Kyrgyzstan)*; Hannu MT Uusitalo (University of Tampere Tays Eye Center, Finland)*; Nalan Uysal (Sabiha Gokcen Ilkokulu, Turkey)*; Justina Vaitkeviciute (Lithuanian University of Health Sciences, Lithuania)*; Gonzalo Valdivia (Pontificia Universidad Católica de Chile, Chile)*; Susana Vale (Polytechnic Institute of Porto, Portugal)*; Damaskini Valvi (Icahn School of Medicine at Mount Sinai, USA)*; Rob M van Dam (National University of Singapore, Singapore)*; Johan Van der Heyden (Sciensano, Belgium)*; Yvonne T van der Schouw (Utrecht University, The Netherlands)*; Koen Van Herck (Ghent University, Belgium)*; Hoang Van Minh (Hanoi University of Public Health, Vietnam)*; Irene GM van Valkengoed (University of Amsterdam, The Netherlands)*; Dirk Vanderschueren (Katholieke Universiteit Leuven, Belgium)*; Diego Vanuzzo (Cardiovascular Prevention Centre Udine, Italy)*; Anette Varbo (Copenhagen University Hospital, Denmark; University of Copenhagen, Denmark)*; Gregorio Varela-Moreiras (Universidad CEU San Pablo, Spain)*; Patricia Varona-Pérez (National Institute of Hygiene, Epidemiology and Microbiology, Cuba)*; Senthil K Vasan (University of Southampton, UK)*; Tomas Vega (Consejería de Sanidad Junta de Castilla y León, Spain)*; Toomas Veidebaum (National Institute for Health Development, Estonia)*; Gustavo Velasquez-Melendez (Universidade Federal de Minas Gerais, Brazil)*; Biruta Velika (Centre for Disease Prevention and Control, Latvia)*; Giovanni Veronesi (University of Insubria, Italy)*; WM Monique Verschuren (National Institute for Public Health and the Environment, The Netherlands)*; Cesar G Victora (Federal University of Pelotas, Brazil)*; Giovanni Viegi (National Research Council, Italy)*; Lucie Viet (National Institute for Public Health and the Environment, The Netherlands)*; Salvador Villalpando (National Institute of Public Health, Mexico)*; Paolo Vineis (Imperial College London, UK)*; Jesus Vioque (Instituto de Investigación Sanitaria y Biomédica de Alicante, Spain)*; Jyrki K Virtanen (University of Eastern Finland, Finland)*; Marjolein Visser (Vrije Universiteit Amsterdam, The Netherlands)*; Sophie Visvikis-Siest (Institut National de la Santé et de la Recherche Médicale, France)*; Bharathi Viswanathan (Ministry of Health, Seychelles)*;

Mihaela Vladulescu (Sunflower Nursery School, Romania)*; Tiina Vlasoff (North Karelia Center for Public Health, Finland)*; Dorja Vocanec (University of Zagreb School of Medicine, Croatia)*; Henry Völzke (University Medicine Greifswald, Germany)*; Ari Voutilainen (University of Eastern Finland, Finland)*; Sari Voutilainen (University of Eastern Finland, Finland)*; Martine Vrijheid (ISGlobal Centre for Research in Environmental Epidemiology, Spain)*; Tanja GM Vrijkotte (Amsterdam University Medical Centers, The Netherlands; University of Amsterdam, The Netherlands)*; Alisha N Wade (University of the Witwatersrand, South Africa)*; Aline Wagner (University of Strasbourg, France)*; Thomas Waldhör (Medical University of Vienna, Austria)*; Janette Walton (Cork Institute of Technology, Ireland)*; Elvis OA Wambiya (African Population and Health Research Center, Kenya)*; Wan Mohamad Wan Bebakar (Universiti Sains Malaysia, Malaysia)*; Wan Nazaimoon Wan Mohamud (Institute for Medical Research, Malaysia)*; Rildo de Souza Wanderley Júnior (University of Pernambuco, Brazil)*; Ming-Dong Wang (Public Health Agency of Canada, Canada)*; Ningli Wang (Capital Medical University Beijing Tongren Hospital, China)*; Qian Wang (Xinjiang Medical University, China)*; Xiangjun Wang (Shanghai Educational Development Co. Ltd., China)*; Ya Xing Wang (Capital Medical University, China)*; Ying-Wei Wang (Ministry of Health and Welfare, Taiwan)*; S Goya Wannamethee (University College London, UK)*; Nicholas Wareham (University of Cambridge, UK)*; Adelheid Weber (Federal Ministry of Social Affairs, Health, Care and Consumer Protection, Austria)*; Niels Wedderkopp (University of Southern Denmark, Denmark)*; Deepa Weerasekera (Ministry of Health, New Zealand)*; Daniel Weghuber (Paracelsus Medical University, Austria)*; Wenbin Wei (Capital Medical University, China)*; Aneta Weres (University of Rzeszów, Poland)*; Bo Werner (Örebro University, Sweden)*; Peter H Whincup (St George's, University of London, UK)*; Kurt Widhalm (Medical University of Vienna, Austria)*; Indah S Widyahening (Universitas Indonesia, Indonesia)*; Andrzej Wiecek (Medical University of Silesia, Poland)*; Rainford J Wilks (The University of the West Indies, Jamaica)*; Johann Willeit (Medical University of Innsbruck, Austria)*; Peter Willeit (Medical University of Innsbruck, Austria)*; Julianne Williams (World Health

Organization Regional Office for Europe, Russia)*; Tom Wilsgaard (UiT The Arctic University of Norway, Norway)*; Bogdan Wojtyniak (National Institute of Public Health-National Institute of Hygiene, Poland)*; Roy A Wong-McClure (Caja Costarricense de Seguro Social, Costa Rica)*; Andrew Wong (University College London, UK)*; Jyh Eiin Wong (Universiti Kebangsaan Malaysia, Malaysia)*; Tien Yin Wong (Duke-NUS Medical School, Singapore)*; Jean Woo (The Chinese University of Hong Kong, China)*; Mark Woodward (University of New South Wales, Australia; University of Oxford, UK)*; Frederick C Wu (University of Manchester, UK)*; Jianfeng Wu (Shandong University of Traditional Chinese Medicine, China)*; Li Juan Wu (Capital Medical University, China)*; Shouling Wu (Kailuan General Hospital, China)*; Haiquan Xu (Institute of Food and Nutrition Development of Ministry of Agriculture and Rural Affairs, China)*; Liang Xu (Beijing Institute of Ophthalmology, China)*; Nor Azwany Yaacob (Universiti Sains Malaysia, Malaysia)*; Uruwan Yamborisut (Mahidol University, Thailand)*; Weili Yan (Children's Hospital of Fudan University, China)*; Ling Yang (University of Oxford, UK)*; Xiaoguang Yang (Chinese Center for Disease Control and Prevention, China)*; Yang Yang (Shanghai Educational Development Co. Ltd, China)*; Nazan Yardim (Ministry of Health, Turkey)*; Mehdi Yaseri (Shahid Beheshti University of Medical Sciences, Iran)*; Tabara Yasuharu (Kyoto University, Japan)*; Xingwang Ye (University of Chinese Academy of Sciences, China)*; Panayiotis K Yiallouros (University of Cyprus, Cyprus)*; Moein Yoosefi (Non-Communicable Diseases Research Center, Iran)*; Akihiro Yoshihara (Niigata University, Japan)*; Qi Sheng You (Capital Medical University, China)*; San-Lin You (Fu Jen Catholic University, Taiwan)*; Novie O Younger-Coleman (The University of the West Indies, Jamaica)*; Safiah Md Yusof (International Medical University, Malaysia)*; Ahmad Faudzi Yusoff (Ministry of Health, Malaysia)*; Luciana Zaccagni (University of Ferrara, Italy)*; Vassilis Zafiropulos (Hellenic Mediterranean University, Greece)*; Ahmad A Zainuddin (Ministry of Health, Malaysia)*; Seyed Rasoul Zakavi (Mashhad University of Medical Sciences, Iran)*; Farhad Zamani (Iran University of Medical Sciences, Iran)*; Sabina Zambon (University of Padua, Italy)*; Antonis Zampelas (Agricultural University of Athens, Greece)*; Hana

Zamrazilová (Institute of Endocrinology, Czech Republic)*; Maria Elisa Zapata (Centro de Estudios sobre Nutrición Infantil, Argentina)*; Abdul Hamid Zargar (Center for Diabetes and Endocrine Care, India)*; Ko Ko Zaw (University of Public Health, Myanmar)*; Tomasz Zdrojewski (Medical University of Gdansk, Poland)*; Tajana Zeljkovic Vrkic (University Hospital Center Zagreb, Croatia)*; Yi Zeng (Peking University, China; Duke University, USA)*; Luxia Zhang (Peking University First Hospital, China)*; Zhen-Yu Zhang (University of Leuven, Belgium)*; Dong Zhao (Capital Medical University Beijing An Zhen Hospital, China)*; Ming-Hui Zhao (Peking University First Hospital, China)*; Wenhua Zhao (Chinese Center for Disease Control and Prevention, China)*; Shiqi Zhen (Jiangsu Provincial Center for Disease Control and Prevention, China)*; Wei Zheng (Vanderbilt University, USA)*; Yingfeng Zheng (Sun Yat-sen University, China)*; Bekbolat Zholdin (West-Kazakhstan Medical State Medical University, Kazakhstan)*; Maigeng Zhou (Chinese Center for Disease Control and Prevention, China)*; Dan Zhu (Inner Mongolia Medical University, China)*; Yanina Zocalo (Republic University, Uruguay)*; Julio Zuñiga Cisneros (Instituto Conmemorativo Gorgas de Estudios de la Salud, Panama)*; Monika Zuziak (Przedszkole No. 81, Poland)*

Appendix Text 2. Data sources

We used a database on cardiometabolic risk factors collated by the Non-Communicable Disease Risk Factor Collaboration (NCD-RisC). Data were obtained from publicly available multi-country and national measurement surveys (e.g., Demographic and Health Surveys (DHS), WHO-STEPwise approach to Surveillance (STEPS) surveys, and those identified via the Inter-University Consortium for Political and Social Research and European Health Interview & Health Examination Surveys Database). With the help of World Health Organization (WHO) and its regional and country offices as well as World Heart Federation, we identified and accessed population-based survey data from national health and statistical agencies. We searched and reviewed published studies as detailed previously[1] and invited eligible studies to join NCD-RisC, as did we with data holders from earlier pooled analysis of cardiometabolic risk factors.[2-5]

We carefully checked that each data source meets our inclusion criteria listed below. Potential duplicate data sources were first identified by comparing studies from the same country and year, followed by checking with NCD-RisC members that had provided data about whether the sources from the same country and year were the same or distinct. If two sources were confirmed as duplicates, one was discarded. All NCD-RisC members are also periodically asked to review the list of sources from their country, to verify that the included data meet the inclusion criteria and are not duplicates, and to suggest additional sources. The NCD-RisC database is continuously updated through all the above routes. For each data source, we recorded the study population, sampling approach, years of measurement, and measurement methods. Only population-based data were included, and these were assessed in terms of whether they covered the whole country, multiple sub-national regions, or one or a small number of communities, and whether rural, urban, or both participants were included. All submitted data were checked by at least two independent persons. Questions and clarifications were discussed with NCD-RisC members and resolved before data were incorporated in the database.

Anonymised individual data for participants aged 5 to 19 years from the studies in the NCD-RisC database were reanalysed according to a common protocol. We calculated mean height and mean BMI and the associated standard errors by sex and single year of age from 5 to 19 years. Additionally, for analysis of height, participants aged 20 to 30 years were included, assigned to their corresponding birth cohort, because mean height in these ages would be at least that when they were aged 19 years, given that the decline of height with age begins in the third and fourth decades of life.[6] All analyses incorporated sample weights and complex survey design, when applicable, in calculating summary statistics, with computer code provided to NCD-RisC members who requested assistance.

Additionally, summary statistics for nationally representative data from sources that were identified but not accessed via the above routes were extracted from published reports. Data were also extracted for nine STEPS surveys that were not publicly available, one Countrywide Integrated Non-communicable Diseases Intervention (CINDI) survey, and five sites of the WHO Multinational MONItoring of trends and determinants in CArdiovascular disease (MONICA) project that were not deposited in the MONICA Data Centre. We also included those data from a previous global-data pooling study,[5] when not accessed through the above routes.

Data inclusion and exclusion

Data sources were included if:

- measured data on height and weight were available;
- study participants were five years of age and older;
- data were collected using a probabilistic sampling method with a defined sampling frame;

- data were from population samples at the national, sub-national (i.e., covering one or more sub-national regions, with more than three urban or five rural communities), or community level;
- data were from the countries and territories listed in Appendix Table 1.

We excluded all data sources that only used self-reported weight and height without measurement because these data are subject to biases that vary with geography, time, age, sex and socioeconomic characteristics.[7-9] We also excluded data on population subgroups whose anthropometric status may differ systematically from the general population, including:

- studies that had included or excluded people based on their health status or cardiovascular risk;
- studies whose participants were only ethnic minorities;
- specific educational, occupational, or socioeconomic subgroups, with the exception noted below;
- those recruited through health facilities, with the exception noted below; and
- females aged 15-19 years in surveys which only sampled ever-married women or measured height and weight only among mothers.

We included school-based data in countries and age-sex groups with enrolment of 70% or higher because when enrolment is low, children who go to school tend to have higher socioeconomic status. We used primary school enrolment rates for ages 5-12 years and secondary school years for those older. We also included data whose sampling frame was health insurance schemes in countries where at least 80% of the population were insured. Finally, we included data collected through primary care in high-income and central European countries with universal insurance,

because contact with the primary care systems tends to be as good as or better than response rates for population-based surveys.

We excluded <0.2% of all participants who had implausible recorded height (defined as <60 cm or >180 cm for ages <10 years; <80 cm or >200 cm for ages 10-14 years; <100 cm or >250 cm for ages >14 years), weight (<5 kg or >90 kg for age <10 years; <8 kg or >150 kg for ages 10-14 years; <12 kg or >300 kg for ages >14 years), or BMI (<6 kg/m^2 or >40 kg/m^2 for ages < 10 years; <8 kg/m^2 or >60 kg/m^2 for ages 10-14 years; <10 kg/m^2 or >80 kg/m^2 for ages >14 years).

Conversion of BMI prevalence metrics to mean BMI

In less than 2% of our data points, mostly extracted from published reports or a previous pooling analysis,[5] mean BMI was not reported, but data were available for the prevalence of one or more BMI categories. We used previously validated conversion regressions[1] to estimate mean BMI from the available metric(s). To account for the uncertainty of mean BMI estimated through these conversions, we took repeated draws from the sampling uncertainty distribution of the original data points, and from the joint posterior distribution of regression coefficients, random effects and residuals, accounting for the correlations among the uncertainties of regression coefficients and random effects.

Appendix Text 3. Statistical methods

We used a Bayesian hierarchical model to estimate mean height and mean BMI by country, year, sex and age. The model is described in detail in statistical[10] and related substantive papers;[1-5,11] the computer code for the model is available at www.ncdrisc.org.

In summary, the model had a hierarchical structure in which estimates for each country and year were informed by its own data, if available, and by data from other years in the same country and from other countries, especially those in the same region and super-region, with data for similar time periods. The extent to which estimates for each country-year were influenced by data from other years and other countries depended on whether the country had data, the sample size of the data, whether they were national, and the within-country and within-region variability of the available data. For the purpose of hierarchical analysis, countries were organised into 21 regions (Appendix Table 1), mostly based on geography and national income. Regions were in turn organised into nine super-regions.

We used observation year, i.e., the year in which data were collected, as the time-scale for the analysis of BMI and birth year as the time scale for the analysis of height, consistent with previous analyses.[11,12] For BMI, significant societal changes that affect nutrition and physical activity may affect children of different ages simultaneously, whereas for height, these effects accumulate in each birth cohort and a cohort's height-for-age monotonically increases from childhood to late adolescence. The statistical model incorporated non-linear time trends, by having two components, a linear term and a second-order random walk,[13] both modelled hierarchically. The age associations of height and BMI were modelled to allow non-linear changes over age, including periods of rapid as well as slow rise, the former representing adolescent growth spurts.[14] We used a cubic spline to model age non-linearly and flexibly, with the spline parameters permitted to vary across countries based on their own data as well as in a hierarchical structure. We selected the

number and position of splines' knots based on a combination of physiological and statistical considerations. Physiologically, growth spurts during puberty occur earlier in girls than in boys, followed by a slow-down of height gain.[14-16] To allow the age model to have sufficient flexibility to capture such patterns, we used four knots in different positions for boys and girls. Statistically, we evaluated the residuals of the age model and used the model that minimized the sum of squares of residuals. Based on these considerations, the four knots were placed at ages 8, 10, 12, 14 years for girls and at 10, 12, 14, 16 years for boys. For BMI, we used only two spline knots (at ages 10 and 15 years) because, at the population level, changes in BMI with age are smoother than those in height.[14,16]

The statistical model accounted for the possibility that height or BMI in sub-national and community samples might differ systematically from nationally representative samples and have larger variation than in national studies. These features were taken into account by including data-driven fixed-effect and random-effect terms for sub-national and community data. The fixed effects adjusted for systematic differences between sub-national or community studies and national studies; and the random effects allowed national data to have greater influence on the estimates than sub-national or community data with similar sample sizes.

All analyses were done separately by sex because there are differences between girls and boys in height and BMI, and their time trends and age associations.[1,11,16] We fitted the statistical model with the Markov chain Monte Carlo (MCMC) algorithm and obtained 5,000 post-burn in samples (or draws) from the posterior distribution of model parameters, which were used to obtain the posterior distributions of mean height and mean BMI. Posterior estimates were made in one-year age groups from five to 19 years of age for every year from 1985 to 2019. We applied the pool-adjacent-violators algorithm, a monotonic regression that uses an iterative algorithm based on least squares to fit a free-form line to a sequence of observations such that the fitted line is non-

decreasing,[17,18] on the posterior height estimates to ensure that each birth cohort's height increased monotonically with age. In practice, this had little effect on the results, with height at age 19 years adjusted by an average of less than 0.15 cm for both boys and girls. This was because in only three countries for boys and six for girls was height at 19 years estimated to be >1cm lower than at peak age. The reported credible intervals represent the 2.5th and the 97.5th percentiles of the posterior distributions.

Appendix Table 1. List of analysis regions and “super-regions”, and countries in each region. The hierarchical structure of the statistical model consisted of country, region, super-region, and world.

Super-region	Region
**Sub-Saharan Africa (48)**	**Central Africa (6)**: Angola, Central African Republic, Congo, DR Congo, Equatorial Guinea, Gabon
	**East Africa (17)**: Burundi, Comoros, Djibouti, Eritrea, Ethiopia, Kenya, Madagascar, Malawi, Mauritius, Mozambique, Rwanda, Seychelles, Somalia, Sudan (former), Tanzania, Uganda, Zambia
	**Southern Africa (6)**: Botswana, Eswatini, Lesotho, Namibia, South Africa, Zimbabwe
	**West Africa (19)**: Benin, Burkina Faso, Cabo Verde, Cameroon, Chad, Cote d'Ivoire, Ghana, Guinea, Guinea Bissau, Liberia, Mali, Mauritania, Niger, Nigeria, Sao Tome and Principe, Senegal, Sierra Leone, The Gambia, Togo
**Central Asia, Middle East and north Africa (28)**	**Central Asia (9)**: Armenia, Azerbaijan, Georgia, Kazakhstan, Kyrgyzstan, Mongolia, Tajikistan, Turkmenistan, Uzbekistan
	**Middle East and north Africa (19)**: Algeria, Bahrain, Egypt, Iran, Iraq, Jordan, Kuwait, Lebanon, Libya, Morocco, Oman, Qatar, Saudi Arabia, State of Palestine, Syrian Arab Republic, Tunisia, Turkey, United Arab Emirates, Yemen
**South Asia (6)**	**South Asia (6)**: Afghanistan, Bangladesh, Bhutan, India, Nepal, Pakistan
**East and southeast Asia (16)**	**East Asia (4)**: China, China (Hong Kong SAR), North Korea, Taiwan (province of China)
	**Southeast Asia (12)**: Brunei Darussalam, Cambodia, Indonesia, Lao PDR, Malaysia, Maldives, Myanmar, Philippines, Sri Lanka, Thailand, Timor-Leste, Viet Nam
**Oceania (17)**	**Polynesia and Micronesia (13)**: American Samoa, Cook Islands, French Polynesia, Kiribati, Marshall Islands, Micronesia (Federated States of), Nauru, Niue, Palau, Samoa, Tokelau, Tonga, Tuvalu
	**Melanesia (4):** Fiji, Papua New Guinea, Solomon Islands, Vanuatu
**High-income Asia Pacific (3)**	**High-income Asia Pacific (3)**: Japan, Singapore, South Korea
**Latin America and Caribbean (35)**	**Andean Latin America (3)**: Bolivia, Ecuador, Peru
	**Caribbean (18)**: Antigua and Barbuda, Bahamas, Barbados, Belize, Bermuda, Cuba, Dominica, Dominican Republic, Grenada, Guyana, Haiti, Jamaica, Puerto Rico, Saint Kitts and Nevis, Saint Lucia, Saint Vincent and the Grenadines, Suriname, Trinidad and Tobago
	**Central Latin America (9)**: Colombia, Costa Rica, El Salvador, Guatemala, Honduras, Mexico, Nicaragua, Panama, Venezuela
	**Southern Latin America (5)**: Argentina, Brazil, Chile, Paraguay, Uruguay

**High-income western countries (27)**	**High-income English-speaking countries* (6)**: Australia, Canada, Ireland, New Zealand, United Kingdom, United States of America
	**Northwestern Europe (12)**: Austria, Belgium, Denmark, Finland, Germany, Greenland, Iceland, Luxembourg, Netherlands, Norway, Sweden, Switzerland
	**Southwestern Europe (9)**: Andorra, Cyprus, France, Greece, Israel, Italy, Malta, Portugal, Spain
**Central and eastern Europe (20)**	**Central Europe (13)**: Albania, Bosnia and Herzegovina, Bulgaria, Croatia, Czech Republic, Hungary, Montenegro, North Macedonia, Poland, Romania, Serbia, Slovakia, Slovenia
	**Eastern Europe (7)**: Belarus, Estonia, Latvia, Lithuania, Moldova, Russian Federation, Ukraine

* Although high-income English-speaking countries are geographically separated, they exhibit remarkably similar trends in cardiometabolic risk factors and outcomes.[1,11,19-21] They were therefore grouped together so that the statistical model shares information amongst them more than it does with other countries that are geographically closer but epidemiologically more distinct.

Appendix Table 2. Data sources used in the analysis.

	Country	Study years	Survey/Study name/Citation	Level of representativeness	Rural, urban or both	Age range as in NCD-RisC database*		Sample size as in height analysis		Sample size as in BMI analysis		Note
						Male	Female	Male	Female	Male	Female	
1	Afghanistan	2013	National Nutrition Survey	National	Both		10-49		13,343		6,443	
2	Afghanistan	2018	STEPS	National	Both	18-69	18-69	692	638	139	145	
3	Albania	2008-2009	DHS	National	Both	15-49	15-49	1,305	3,267	659	1,488	
4	Albania	2013	Childhood Obesity Surveillance Initiative 3	National	Both	7-9	7-9	2,971	2,794	2,971	2,794	1
5	Albania	2013-2015	Balkan Survey of Inactivity in Children (BASIC)	Subnational	Both	5-16	5-16	4,986	4,985	4,986	4,982	
6	Albania	2015-2016	Childhood Obesity Surveillance Initiative 4	National	Both	7-9	7-9	3,359	3,062	3,358	3,062	1
7	Albania	2017-2018	DHS	National	Both	15-59	15-59	2,044	4,557	727	1,649	
8	Algeria	2003	STEPS	Subnational	Both	25-64	25-64	256	478			
9	Algeria	2016-2017	STEPS	National	Both	18-69	18-69	626	788	99	120	
10	American Samoa	1976-1978	McGarvey, Am J Clin Nutr 53(6 Suppl):1586S-1594S, 1991	National	Both	5+	5+	89	79			
11	American Samoa	2004	STEPS	National	Both	25-64	25-64	126	158			
12	Antigua and Barbuda	2009	Global School-based Student Health Survey	National	Both	13-17	13-17	70	122	70	122	
13	Argentina	2005	Encuensta Nacional de Nutrición y Salud 2005	National	Both		10-49		3,939		2,171	
14	Argentina	2004-2005	Cardiovascular Risk Factors Multiple Evaluation in Latin America	Community	Urban	25-64	25-64	195	184			
15	Argentina	2008-2011	The VELA Project	Community	Rural	5+	5+	281	361	246	291	
16	Argentina	2011	Primera Encuesta Alimentaria y Nutricional de la Ciudad Autónoma de Buenos Aires - EAN CABA	Community	Urban	5-18; 60+	5-49; 60+	982	1,332	982	1,012	
17	Argentina	2012	Global School-based Student Health Survey	National	Both	13-17	13-17	8,337	8,906	8,333	8,904	
18	Argentina	2012-2013	Primer estudio sobre el estado nutricional y los hábitos alimentarios de la población adulta de Rosario	Community	Urban	18-70	18-70	129	312	17	54	
19	Argentina	2018	Encuesta Nacional de Factores de Riesgo 2018	National	Both	18+	18+	1,605	1,931	291	250	
20	Argentina	2018	Global School-based Student Health Survey	National	Both	12-17	12-17	16,801	18,979	16,773	18,935	
21	Armenia	1998	The health and nutritional status of children and women in Armenia	National	Both		18-45		1,519		72	
22	Armenia	2000	DHS	National	Both		15-49		2,794		1,090	
23	Armenia	2005	DHS	National	Both	15-49	15-49	606	3,027	245	1,086	
24	Armenia	2015-2016	DHS	National	Both		15-49		2,640		707	
25	Armenia	2016	STEPS	National	Both	18-69	18-69	168	270	14	26	
26	Australia	1985	Australian Council for Health, Physical Education and Recreation survey	National	Both	7-12	7-12	707	702	707	702	
27	Australia	1994	MONICA, Perth inner	Community	Urban	25-64	25-64	23	28			
28	Australia	1994	MONICA, Perth outer	Community	Urban	25-64	25-64	26	36			
29	Australia	1995	National Nutrition Survey 1995	National	Both	5+	5+	2,152	2,149	1,236	1,171	
30	Australia	1996	The Nepean Longitudinal Cohort Study	Community	Urban	7-8	7-8	221	215	221	215	
31	Australia	1999-2000	The Australian Diabetes, Obesity and Lifestyle Study 1999-2000	National	Both	25+	25+	263	319			
32	Australia	1999-2003	North West Adelaide Health Study	Community	Urban	18+	18+	236	225	31	37	
33	Australia	2004	The Longitudinal Study of Australian Children, K cohort (child)	National	Both	5	5	425	431	425	431	
34	Australia	2004	The Nepean Longitudinal Cohort Study	Community	Urban	14-15	14-15	143	150	143	149	
35	Australia	2004-2006	North West Adelaide Health Study	Community	Urban	20+	20+	77	62			
36	Australia	2006	The Longitudinal Study of Australian Children, K cohort (child)	National	Both	6-7	6-7	2,249	2,164	2,245	2,156	
37	Australia	2007	Children's Nutrition and Physical Activity Survey	National	Both	5-16	5-16	1,652	1,672	1,649	1,669	
38	Australia	2008	The Longitudinal Study of Australian Children, B cohort (infant)	National	Both	5	5	508	514	508	513	
39	Australia	2008	The Longitudinal Study of Australian Children, K cohort (child)	National	Both	8-9	8-9	2,124	2,025	2,121	2,023	
40	Australia	2007-2008	National Health Survey	National	Both	18+	18+	970	939	148	136	
41	Australia	2008-2010	North West Adelaide Health Study	Community	Urban	24+	24+	27	23			
42	Australia	2010	The Longitudinal Study of Australian Children, B cohort (infant)	National	Both	6-7	6-7	2,153	2,022	2,148	2,014	
43	Australia	2010	The Longitudinal Study of Australian Children, K cohort (child)	National	Both	10-11	10-11	2,054	1,948	2,052	1,945	
44	Australia	2011-2013	International Study of Childhood Obesity, Lifestyle and the Environment (ISCOLE)	Community	Urban	9-11	9-11	243	285	243	285	
45	Australia	2012	The Longitudinal Study of Australian Children, B cohort (infant)	National	Both	8-9	8-9	2,053	1,953	2,041	1,947	
46	Australia	2012	The Longitudinal Study of Australian Children, K cohort (child)	National	Both	12-13	12-13	1,963	1,883	1,948	1,851	
47	Australia	2011-2013	Australian Health Survey 2011-13	National	Both	5+	5+	3,947	3,976	2,451	2,386	2
48	Australia	2014	The Longitudinal Study of Australian Children, B cohort (infant)	National	Both	10-11	10-11	1,848	1,763	1,829	1,742	
49	Australia	2014	The Longitudinal Study of Australian Children, K cohort (child)	National	Both	14-15	14-15	1,709	1,634	1,694	1,581	
50	Australia	2014-2015	National Health Survey	National	Both	5+	5+	1,824	1,675	1,824	1,675	
51	Australia	2016	The Longitudinal Study of Australian Children, B cohort (infant)	National	Both	12-13	12-13	1,648	1,560	1,639	1,529	
52	Australia	2016	The Longitudinal Study of Australian Children, K cohort (child)	National	Both	16-17	16-17	1,484	1,427	1,477	1,380	
53	Australia	2017-2018	National Health Survey	National	Both	18+	18+	1,116	1,176	155	166	
54	Austria	1983	The Austrian Conscription Database	National	Both	17-18		30,463		58,517		3
55	Austria	1984	The Austrian Conscription Database	National	Both	17-18		58,937		58,830		3

	Country	Study years	Survey/Study name/Citation	Level of representativeness	Rural, urban or both	Age range as in NCD-RisC database*		Sample size as in height analysis		Sample size as in BMI analysis		Note
						Male	Female	Male	Female	Male	Female	
56	Austria	1985	The Austrian Conscription Database	National	Both	17-18		58,725		58,638		
57	Austria	1986	The Austrian Conscription Database	National	Both	17-18		58,135		57,870		
58	Austria	1987	The Austrian Conscription Database	National	Both	17-18		55,333		55,292		
59	Austria	1988	The Austrian Conscription Database	National	Both	17-18		51,051		51,050		
60	Austria	1989	The Austrian Conscription Database	National	Both	17-18		49,381		49,380		
61	Austria	1990	The Austrian Conscription Database	National	Both	17-18		47,103		47,102		
62	Austria	1991	The Austrian Conscription Database	National	Both	17-18		44,288		44,288		
63	Austria	1992	The Austrian Conscription Database	National	Both	17-18		43,555		43,553		
64	Austria	1992	Vorarlberg Health Monitoring and Promotion Programme (VHM&PP)	Subnational	Both	18+	18+	2,379	3,238	54	73	
65	Austria	1993	The Austrian Conscription Database	National	Both	17-18		42,357		42,356		
66	Austria	1994	The Austrian Conscription Database	National	Both	17-18		40,402		40,401		
67	Austria	1995	The Austrian Conscription Database	National	Both	17-18		39,813		39,809		
68	Austria	1996	The Austrian Conscription Database	National	Both	17-18		39,587		39,587		
69	Austria	1997	The Austrian Conscription Database	National	Both	17-18		40,408		40,408		
70	Austria	1998	The Austrian Conscription Database	National	Both	17-18		43,131		43,131		
71	Austria	1998	Vorarlberg Health Monitoring and Promotion Programme (VHM&PP)	Subnational	Both	18+	18+	1,403	2,339	18	30	
72	Austria	1999	The Austrian Conscription Database	National	Both	17-18		44,163		44,163		
73	Austria	1998-1999	CINDI Survey Vorarlberg/Austria	Subnational	Both	25-64	25-64	37	48			
74	Austria	2000	The Austrian Conscription Database	National	Both	17-18		44,278		44,275		
75	Austria	2001	The Austrian Conscription Database	National	Both	17-18		43,318		43,315		
76	Austria	2002	The Austrian Conscription Database	National	Both	17-18		42,899		42,899		
77	Austria	2003	The Austrian Conscription Database	National	Both	17-18		42,389		42,389		
78	Austria	2004	The Austrian Conscription Database	National	Both	17-18		42,774		42,774		
79	Austria	2004	Vorarlberg Health Monitoring and Promotion Programme (VHM&PP)	Subnational	Both	18+	18+	1,346	2,052	30	68	
80	Austria	2005	The Austrian Conscription Database	National	Both	17-18		42,907		42,906		
81	Austria	2004-2005	Vorarlberg Health Monitoring and Promotion Programme (VHM&PP)	Subnational	Both	6-17	6-17	17,504	15,823	17,504	15,823	
82	Austria	2006	The Austrian Conscription Database	National	Both	17-18		44,572		44,571		
83	Austria	2007	The Austrian Conscription Database	National	Both	17-18		44,756		44,755		
84	Austria	2006-2007	HELENA	Community	Urban	12-17	12-17	191	211	191	211	
85	Austria	2008	The Austrian Conscription Database	National	Both	17-18		44,880		44,880		
86	Austria	2009	The Austrian Conscription Database	National	Both	17-18		45,594		45,594		
87	Austria	2008-2009	Vorarlberg Health Monitoring and Promotion Programme (VHM&PP)	Subnational	Both	6-17	6-17	16,847	15,295	16,847	15,295	
88	Austria	2010	The Austrian Conscription Database	National	Both	17-18		44,968		44,968		
89	Austria	2009-2011	Mayer et al., Ann Hum Biol 42(1):45-55, 2015	National	Both	5-17	5-17	6,638	6,339	6,634	6,340	
90	Austria	2010-2012	Austrian Study on Nutritional Status 2012	National	Both	6-80	6-80	230	265	198	198	
91	Austria	2011	The Austrian Conscription Database	National	Both	17-18		44,076		44,076		
92	Austria	2012	The Austrian Conscription Database	National	Both	17-18		42,768		42,768		
93	Austria	2011-2012	BMI in Upper Austrian Children and Adolescents	Subnational	Both	6-17	6-17			7,808	6,853	
94	Austria	2013	The Austrian Conscription Database	National	Both	17-18		41,574		41,574		
95	Austria	2012-2013	Vorarlberg Health Monitoring and Promotion Programme (VHM&PP)	Subnational	Both	6-17	6-17	14,760	13,648	14,760	13,648	
96	Austria	2014	The Austrian Conscription Database	National	Both	17-18		41,740		41,740		
97	Austria	2013-2014	Prevalence of obesity and motor performance in Tyrolean preschool children	Subnational	Both	5	5	550	513	550	513	
98	Austria	2015	The Austrian Conscription Database	National	Both	17-18		39,154		39,154		
99	Austria	2014-2015	Influence of selected risk factors on the motor performance of 10- to 11-year-old schoolchildren	Subnational	Both	10-11	10-11	197	129	197	129	
100	Austria	2016	The Austrian Conscription Database	National	Both	17-18		38,076		38,076		
101	Austria	2015-2016	Childhood Obesity Surveillance Initiative 4	National	Both	8-9	8-9	1,220	1,175	1,220	1,175	1
102	Austria	2015-2017	EVA Tyrol Study Austria North East Tyrol	Subnational	Both	14-17	14-17	680	831	680	831	
103	Austria	2015-2016	Vorarlberg Health Monitoring and Promotion Programme (VHM&PP)	Subnational	Both	6-17	6-17	13,037	12,048	13,037	12,048	
104	Austria	2017	The Austrian Conscription Database	National	Both	17-18		36,081		36,080		
105	Azerbaijan	1996	Health and Nutrition Survey	National	Both	19-59	19-59	34	137		3	
106	Azerbaijan	2006	DHS	National	Both	15-59	15-49	1,028	3,814	382	1,402	
107	Azerbaijan	2017	STEPS	National	Both	18-69	18-69	212	252	21	28	
108	Bahamas	2011-2012	STEPS	National	Both	25-64	25-64	111	132			
109	Bahamas	2013	Global School-based Student Health Survey	National	Both	13-17	13-17	460	533	460	533	
110	Bahrain	1998-1999	National Nutrition Survey	National	Both	19+	19+	212	203			
111	Bahrain	2001-2004	Global database on growth and malnutrition of school children and adolescents, WHO	National	Both	6-19	6-20			1,268	1,326	
112	Bahrain	2016	Global School-based Student Health Survey	National	Both	12-17	12-17	3,416	3,262	3,416	3,262	

	Country	Study years	Survey/Study name/Citation	Level of representativeness	Rural, urban or both	Age range as in NCD-RisC database*		Sample size as in height analysis		Sample size as in BMI analysis		Note
						Male	Female	Male	Female	Male	Female	
113	Bangladesh	1996-1997	DHS	National	Both		20-49		2,536			
114	Bangladesh	1999-2000	DHS	National	Both		20-49		2,852			
115	Bangladesh	2002	STEPS	National	Rural	25-64	25-64	304	278			
116	Bangladesh	2002	STEPS	National	Urban	25-64	25-64	608	704			
117	Bangladesh	2004	DHS	National	Both		20-49		4,187			
118	Bangladesh	2006	Urban Health Survey	Subnational	Urban	20-59	20-59	2,349	2,562			
119	Bangladesh	2007	DHS	National	Both		20-49		4,060			
120	Bangladesh	2009-2010	STEPS	National	Both	25+	25+	588	1,139			
121	Bangladesh	2011	DHS	National	Both	15+	20+	859	6,837	17		
122	Bangladesh	2013	STEPS	National	Both	25+	25+	279	495			
123	Bangladesh	2014	Global School-based Student Health Survey	National	Both		11-13		447		447	
124	Bangladesh	2014	DHS	National	Both		20-49		6,441			
125	Bangladesh	2015	An assessment of BRAC Health Nutrition and Population Programme and benchmark survey of Sustainable Development Goal – 2015	National	Rural	35+	11+		10,262		4,025	
126	Bangladesh	2018	STEPS	National	Both	18-69	18-69	765	1,079	118	162	
127	Bangladesh	2018-2019	National Nutrition Surveillance	National	Both	10+	10+	6,136	6,521	4,894	4,849	
128	Barbados	2011	Global School-based Student Health Survey	National	Both	13-17	13-17	628	708	627	708	
129	Barbados	2011-2013	Health of the Nation (HotN)	National	Both	25+	25+	24	42			
130	Belarus	2016-2017	STEPS	National	Both	18-69	18-69	331	344	32	29	
131	Belgium	1996-1998	Flemish Study on Environment, Genes and Health Outcomes	Community	Rural	10-84	10-84	132	129	72	76	
132	Belgium	1998-2000	Flemish Study on Environment, Genes and Health Outcomes	Community	Rural	10-80	10-80	64	64	25	32	
133	Belgium	1999-2001	Flemish Study on Environment, Genes and Health Outcomes	Community	Rural	10-81	10-81	56	60	29	34	
134	Belgium	2001	Flemish Study on Environment, Genes and Health Outcomes	Community	Rural	10-78	10-78	67	62	19	22	
135	Belgium	2002-2003	Flemish Study on Environment, Genes and Health Outcomes	Community	Rural	10-81	10-81	30	46	9	21	
136	Belgium	2002-2005	Flemish Study on Environment, Genes and Health Outcomes	Community	Rural	10-88	10-88	123	125	70	68	
137	Belgium	2005-2008	Flemish Study on Environment, Genes and Health Outcomes	Community	Rural	10-89	10-89	82	65	24	23	
138	Belgium	2006-2007	HELENA	Community	Urban	12-17	12-17	156	180	156	180	
139	Belgium	2007-2008	Childhood Obesity Surveillance Initiative 1	Subnational	Both	6-9	6-9	64,322	61,755	64,322	61,754	1
140	Belgium	2007-2010	Identification and prevention of Dietary- and lifestyle-induced health EFfects In Children and infantS (IDEFICS)	Community	Urban	5-9	5-9	822	834	822	834	
141	Belgium	2009-2010	Childhood Obesity Surveillance Initiative 2	Subnational	Both	6-9	6-9	67,783	65,373	67,775	65,365	1
142	Belgium	2009-2013	Flemish Study on Environment, Genes and Health Outcomes	Community	Rural	20-88	20-88	16	24			
143	Belgium	2012-2013	Childhood Obesity Surveillance Initiative 3	Subnational	Both	6-9	6-9	70,442	67,876	70,441	67,874	1
144	Belgium	2010-2015	Flemish Study on Environment, Genes and Health Outcomes	Community	Rural	15-87	15-87	38	48	2	2	
145	Belgium	2014-2015	Food Consumption Survey	National	Urban	5-64	5-64	1,050	1,036	908	892	
146	Belgium	2018-2019	European Health Examination Survey	National	Both	18+	18+	65	97	7	7	
147	Belize	2004-2005	CAMDI	National	Both	20+	20+	95	200			
148	Belize	2011	Global School-based Student Health Survey	National	Both	13	13	163	188	163	188	
149	Benin	1996	DHS	National	Both		20-49		1,310			
150	Benin	2001	DHS	National	Both		15-49		3,576		1,118	
151	Benin	2006	DHS	National	Both		15-49		9,174		2,602	
152	Benin	2007	STEPS	Community	Urban	25-64	25-64	243	406			
153	Benin	2008	STEPS	National	Both	25-64	25-64	631	563			
154	Benin	2011-2012	DHS	National	Both		15-49		8,635		2,699	
155	Benin	2015	STEPS	National	Both	18-69	18-69	659	833	70	101	
156	Benin	2017-2018	DHS	National	Both		15-49		4,589		1,538	
157	Bhutan	2007	STEPS	Community	Urban	25-74	25-74	119	152			
158	Bhutan	2014	STEPS	National	Both	18-69	18-69	196	417	11	28	
159	Bolivia	1994	DHS	National	Both		20-49		1,322			
160	Bolivia	1998	DHS	National	Both		20-49		2,094			
161	Bolivia	2003	DHS	National	Both		15-49		9,505		3,625	
162	Bolivia	2005-2007	Baya Botti et al., Nutr Hosp 24(3):304-11, 2009	National	Both	12-18	12-18	1,504	1,849	1,499	1,841	
163	Bolivia	2008	DHS	National	Both		15-49		8,826		3,239	
164	Bosnia and Herzegovina	2002	Non-communicable disease risk factor survey, Federation of Bosnia and Herzegovina	Subnational	Both	25-64	25-64	73	148			
165	Bosnia and Herzegovina	2012	Non-communicable disease risk factor survey, Federation of Bosnia and Herzegovina	Subnational	Rural	18+	18+	272	238	50	38	
166	Bosnia and Herzegovina	2012	Non-communicable disease risk factor survey, Federation of Bosnia and Herzegovina	Subnational	Urban	18+	18+	136	133	22	17	
167	Botswana	2007	STEPS	National	Both	25-64	25-64	363	666			

	Country	Study years	Survey/Study name/Citation	Level of representativeness	Rural, urban or both	Age range as in NCD-RisC database*		Sample size as in height analysis		Sample size as in BMI analysis		Note
						Male	Female	Male	Female	Male	Female	
168	Botswana	2014	STEPS	National	Both	15-69	15-69	554	932	126	206	
169	Brazil	1989	Pesquisa Nacional sobre Saude e Nutricao	National	Both	5+	5+	11,063	10,844	11,049	10,832	
170	Brazil	1995	The 1982 Pelotas (Brazil) Birth Cohort: 13 years follow-up	Community	Urban	13	13	352	363	352	363	
171	Brazil	1996-1997	Pesquisa sobre Padrões de Vida (PPV)	Subnational	Both	5+	5+	4,207	4,405	2,901	2,843	
172	Brazil	1995-1996	Cohort study from Porto Alegre	Community	Urban	18+	18+	151	148	22	26	
173	Brazil	1996	DHS	National	Both		20-49		1,817			
174	Brazil	1996-1997	The Bambui Cohort Study of Ageing	Community	Urban	18+	18+	129	131	25	20	
175	Brazil	1997	The 1982 Pelotas (Brazil) Birth Cohort: 15 years follow-up	Community	Urban	15	15	559	513	559	513	
176	Brazil	1998	Belo Horizonte Heart Study	Community	Urban	6-18	6-18	658	738	658	738	
177	Brazil	1999-2000	Projeto Esporte Brasil	National	Urban	6-11	6-11	107	102	107	102	
178	Brazil	2000	The 1982 Pelotas (Brazil) Birth Cohort: 18 years follow-up	Community	Urban	18		2,229		2,228		
179	Brazil	1999-2000	Prevalence of Risk Factors for Coronary Artery Disease in the State of Rio Grande do Sul	Subnational	Urban	20+	20+	125	118			
180	Brazil	1999-2000	Pelotas cross-sectional survey	Community	Urban	20-69	20-69	229	220			
181	Brazil	2001	Projeto Esporte Brasil	National	Urban	6-17	6-17	251	225	249	225	
182	Brazil	2001	The 1982 Pelotas (Brazil) Birth Cohort: 19 years follow-up	Community	Urban		19		920		919	
183	Brazil	2002-2003	Pesquisa de Orcamentos Familiares	National	Both	5+	5+	44,862	41,332	29,276	27,119	
184	Brazil	2003	Projeto Esporte Brasil	National	Urban	6-17	6-17	2,023	2,078	2,016	2,073	
185	Brazil	2003	Women's Health in Southern Brazil	Community	Urban		20-60		262			
186	Brazil	2004	Caju & Virgen das Gracas	Community	Rural	18+	18+	73	77	15	14	
187	Brazil	2004	Projeto Esporte Brasil	National	Urban	6-17	6-17	14,509	12,304	14,447	12,254	
188	Brazil	2002-2004	Ribeira Preto Birth Cohort	Community	Urban	22-25	22-25	1,013	1,083			
189	Brazil	2004-2006	Hearts of Brazil	National	Urban	18+	18+	113	129	11	20	
190	Brazil	2004-2005	The 1993 Pelotas (Brazil) Birth Cohort: 11 years follow-up	Community	Urban	10-12	10-12	2,184	2,258	2,184	2,257	
191	Brazil	2005	Projeto Esporte Brasil	National	Urban	6-17	6-17	3,743	3,388	3,615	3,287	
192	Brazil	2005	Syndrome of Obesity and Risk Factors for Cardiovascular Disease Study	Community	Urban	18-100	18-100	209	211	74	69	
193	Brazil	2005	Syndrome of Obesity and Risk Factors for Cardiovascular Disease Study Among Teenagers	Community	Urban	11-18	11-18	231	238	230	236	
194	Brazil	2004-2005	The 1982 Pelotas (Brazil) Birth Cohort: 23 years follow-up	Community	Urban	23	23	2,207	2,082			
195	Brazil	2006	ATITUDE	Subnational	Both	14-21	14-21	2,406	3,484	1,669	2,477	
196	Brazil	2006	The Ouro Preto Study	Community	Urban	7-14	7-14	364	399	364	399	
197	Brazil	2006	Pesquisa Nacional de Demografia e Saude 2006	National	Both		15-49		6,916		2,329	
198	Brazil	2006	Projeto Esporte Brasil	National	Urban	6-17	6-17	7,521	6,220	7,505	6,211	
199	Brazil	2005-2006	Sao Luis Birth Cohort	Community	Urban	7-8	7-8	347	325	347	325	
200	Brazil	2007	Projeto Esporte Brasil	National	Urban	6-17	6-17	4,772	4,219	4,768	4,215	
201	Brazil	2006-2007	Syndrome of Obesity and Risk Factors for Cardiovascular Disease Study Among Teenagers	Community	Urban	11-18	11-18	231	238	230	236	
202	Brazil	2006-2007	Syndrome of Obesity and Risk Factors for Cardiovascular Disease Study	Community	Urban	18+	18+	209	205	74	70	
203	Brazil	2008	Caju & Virgen das Gracas	Community	Rural	18+	18+	62	76	14	24	
204	Brazil	2007-2008	Scan J Med Sci Sports, 23(3):317-22	Community	Urban	10-16	10-16	493	528	493	528	
205	Brazil	2008	The 1993 Pelotas (Brazil) Birth Cohort: 15 years follow-up	Community	Urban	14-15	14-15	2,001	2,095	2,001	2,095	
206	Brazil	2008	Projeto Esporte Brasil	National	Urban	6-17	6-17	3,511	2,397	3,474	2,386	
207	Brazil	2008-2010	Machado-Rodrigues et al., Ann Hum Biol 2013; 41: 271-6	Community	Urban	10-18	10-18	360	484	360	482	
208	Brazil	2008-2009	Pesquisa de Orcamentos Familiares	National	Both	5+	5+	43,488	42,858	27,098	25,710	
209	Brazil	2009	Projeto Esporte Brasil	National	Urban	6-17	6-17	970	783	943	767	
210	Brazil	2010	Longitudinal Study of Health and Wellbeing in Preschool Age (Project ELOS-Pré)	Community	Urban	5	5	255	247	255	247	
211	Brazil	2010	Projeto Esporte Brasil	National	Urban	6-17	6-17	1,097	1,051	1,095	1,047	
212	Brazil	2010	San Pedro	Community	Rural	18+	18+	37	48	8	10	
213	Brazil	2009-2010	EpiFloripa Adults Cohort Study (EpiFloripa)	Community	Urban	20-59	20-59	262	266			
214	Brazil	2011	ATITUDE	Subnational	Both	14-19	14-19	2,421	3,658	2,420	3,658	
215	Brazil	2010-2011	The 2004 Pelotas (Brazil) Birth Cohort: 6 years follow-up	Community	Urban	6-7	6-7	1,864	1,740	1,721	1,631	
216	Brazil	2011	Pregnancy in adolescence in municipalities of small size in the Northeast of Brazil	Community	Both	13-19	13-19	512	563	512	563	
217	Brazil	2011	Projeto Esporte Brasil	National	Urban	6-17	6-17	1,260	933	1,243	929	
218	Brazil	2012	Longitudinal Study of Health and Wellbeing in Preschool Age (Project ELOS-Pré)	Community	Urban	5-7	5-7	388	349	388	348	
219	Brazil	2011-2013	International Study of Childhood Obesity, Lifestyle and the Environment (ISCOLE)	Community	Urban	9-11	9-11	287	297	277	287	
220	Brazil	2011-2012	The 1993 Pelotas (Brazil) Birth Cohort: 18 years follow-up	Community	Urban	17-19	17-19	1,970	2,004	1,970	2,004	
221	Brazil	2012	Projeto Esporte Brasil	National	Urban	6-17	6-17	3,133	1,994	3,116	1,976	
222	Brazil	2012	EpiFloripa Adults Cohort Study (EpiFloripa)	Community	Urban	22-62	22-62	103	121			
223	Brazil	2010-2015	Baependi Heart Study	Community	Rural	18+	18+	240	293	47	48	
224	Brazil	2013	Pesquisas Nacional de Saude	National	Both	18+	18+	6,060	7,421	1,005	1,101	

	Country	Study years	Survey/Study name/Citation	Level of representativeness	Rural, urban or both	Age range as in NCD-RisC database*		Sample size as in height analysis		Sample size as in BMI analysis		Note
						Male	Female	Male	Female	Male	Female	
225	Brazil	2012-2013	Prevalence of Leptin Polymorphism Gln223Arg	Community	Urban	18+	18+	71	137	15	24	
226	Brazil	2013	Projeto Esporte Brasil	National	Urban	6-17	6-17	1,270	965	1,269	960	
227	Brazil	2011-2014	Profile of Risk Factors for Coronary Arterial Disease in Rio Grande Do Sul - Revaluation after 10 Years	Subnational	Urban	20+	20+	60	63			
228	Brazil	2012-2013	The 1982 Pelotas (Brazil) Birth Cohort: 30 years follow-up	Community	Urban	30	30	1,754	1,853			
229	Brazil	2014	Longitudinal Study of Health and Wellbeing in Preschool Age (Project ELOS-Pré)	Community	Urban	7-9	7-9	246	213	223	200	
230	Brazil	2013-2014	Estudo de Riscos Cardiovasculares em Adolescentes (ERICA)	National	Both	12-17	12-17	32,724	40,675	32,723	40,675	
231	Brazil	2014	Projeto Esporte Brasil	National	Urban	6-17	6-17	267	212	267	212	
232	Brazil	2014	Brazilian Guide to the Physical Fitness Related to Health Assessment and Lifestyle Habits	Community	Urban	14-19	14-19	473	538	473	535	
233	Brazil	2015	The 2004 Pelotas (Brazil) Birth Cohort: 11 years follow-up	Community	Urban	10-11	10-11	1,736	1,632	1,736	1,632	
234	Brazil	2015	Projeto Esporte Brasil	National	Urban	6-17	6-17	639	497	639	496	
235	Brazil	2014-2015	EpiFloripa Adults Cohort Study (EpiFloripa)	Community	Urban	25-65	25-65	41	55			
236	Brazil	2016	ATITUDE	Subnational	Both	14-21	14-21	2,592	3,222	2,575	3,198	
237	Brazil	2016	Projeto Esporte Brasil	National	Urban	6-17	6-17	175	110	175	110	
238	Brazil	2015-2016	The 1993 Pelotas (Brazil) Birth Cohort: 22 years follow-up	Community	Urban	21-23	21-23	1,703	1,886			
239	Brazil	2017	Projeto Esporte Brasil	National	Urban	6-17	6-17	2,738	1,358	2,736	1,350	
240	Brazil	2017	Intervention in physical education classes to reduce sedentary behavior and improve cognitive function: SACODE	Community	Both	14-19	14-19	501	632	501	631	
241	Brazil	2016-2017	Study in Presidente Prudente	Community	Urban	18+	18+	30	50	7	7	
242	Brazil	2018	Healthy Living Study in Lagoa do Carro	Community	Urban	5-18	5-18	637	595	637	595	
243	Brazil	2018	Projeto Esporte Brasil	National	Urban	6-17	6-17	1,331	1,160	1,315	1,151	
244	Brazil	2019	Projeto Esporte Brasil	National	Urban	6-17	6-17	1,623	1,397	1,587	1,356	
245	Brazil	2018-2019	Epidemiology in the health (Santo Anastácio Edition)	Community	Urban	18+	18+	34	38	4	8	
246	Brunei Darussalam	2010-2011	National Health And Nutritional Status Survey (NHANSS)	National	Both	5-75	5-75	581	578	348	349	
247	Brunei Darussalam	2014	Global School-based Student Health Survey	National	Both	12-17	12-17	1,145	1,326	1,145	1,326	
248	Brunei Darussalam	2015-2016	National Non-Communicable Diseases Survey (NNCDS)	National	Both	18-69	18-69	221	283	51	48	
249	Bulgaria	2008	Childhood Obesity Surveillance Initiative 1	National	Both	7-8	7-8	1,657	1,661	1,657	1,661	1
250	Bulgaria	2013	Childhood Obesity Surveillance Initiative 3	National	Both	7	7	1,671	1,677	1,671	1,677	1
251	Bulgaria	2015-2016	Childhood Obesity Surveillance Initiative 4	National	Both	7	7	1,702	1,698	1,702	1,698	1
252	Bulgaria	2016	Feel4Diabetes	Community	Urban	6-10	6-10	1,447	1,522	1,447	1,522	
253	Bulgaria	2016-2017	Erasmus plus KA2, Healthyland	Community	Urban	5	5	26	24	26	24	
254	Bulgaria	2017-2018	Erasmus plus KA2, Healthyland	Community	Urban	5-6	5-6	49	51	49	51	
255	Burkina Faso	1992-1993	DHS	National	Both		20-49		1,894			
256	Burkina Faso	1998-1999	DHS	National	Both		20-49		1,818			
257	Burkina Faso	2002	Vulnérabilité Alimentaire et Sécurité Nutritionnelle dans la Gnagna (VASN-Gnagna)	Subnational	Rural	5+	5+	1,025	2,219	787	882	
258	Burkina Faso	2003	DHS	National	Both		15-49		6,887		2,520	
259	Burkina Faso	2010	DHS	National	Both		15-49		4,800		1,587	
260	Burkina Faso	2013	STEPS	National	Both	25-64	25-64	571	634			
261	Burundi	2010	DHS	National	Both		15-49		2,906		1,143	
262	Burundi	2016-2017	DHS	National	Both		15-49		5,079		1,922	
263	Cabo Verde	2007	STEPS	National	Both	25-64	25-64	119	130			
264	Cambodia	2000	DHS	National	Both		15-49		3,676		1,620	
265	Cambodia	2005	DHS	National	Both		15-49		4,462		1,765	
266	Cambodia	2010	DHS	National	Both		15-49		5,157		1,894	
267	Cambodia	2010	STEPS	National	Both	25-64	25-64	303	504			
268	Cambodia	2014	DHS	National	Both		15-49		5,901		1,842	
269	Cameroon	1998	DHS	National	Both		20-49		988			
270	Cameroon	1998-1999	ENHIP	Community	Rural	15+	15+	114	182	48	51	
271	Cameroon	1998-1999	ENHIP	Community	Urban	15+	15+	310	372	138	160	
272	Cameroon	2003	STEPS	Subnational	Urban	15+	15+	1,744	2,648	631	898	
273	Cameroon	2004	DHS	National	Both		15-49		3,223		1,179	
274	Cameroon	2007	Cameroon Burden of Diabetes - Second Survey	Subnational	Urban	18+	18+	766	1,238	2		
275	Cameroon	2009	National Survey of Micronutrient Status and Consumption of Fortifiable Foods	National	Both		15-49		617		76	
276	Cameroon	2011	DHS	National	Both		15-49		4,945		1,741	
277	Cameroon	2009-2012	Anthropologie nutritionnelle des migrants d'Afrique centrale à la ville et en France	Subnational	Both	18-76	18-76	157	203	26	43	
278	Cameroon	2014-2015	Cardiovascular risk factors screening in urban and rural areas in the Far-North Region Cameroon	Subnational	Both	20+	20+	208	108			
279	Cameroon	2018-2019	DHS	National	Both		15-64		4,141		1,558	

	Country	Study years	Survey/Study name/Citation	Level of representativeness	Rural, urban or both	Age range as in NCD-RisC database*		Sample size as in height analysis		Sample size as in BMI analysis		Note
						Male	Female	Male	Female	Male	Female	
280	Canada	1970-1972	Anthropometry report: Height, weight and body dimensions - a report from Nutrition Canada	National	Both	5-19	5-19	167	165			
281	Canada	1981	Canada Fitness Survey	National	Both	7-64	7-64	1,904	1,786			
282	Canada	1986-1992	Canada Heart Health Survey	National	Both	18-74	18-74	301	316	300	309	
283	Canada	1995	MONICA, Halifax	Community	Both	25-64	25-64	23	26			
284	Canada	1995-1997	Canadian Multicentre Osteoporosis Study (CaMos)	Subnational	Both	35+	25+		84			
285	Canada	2007-2009	Canadian Health Measures Survey, Cycle 1	National	Both	6-79	6-79	1,280	1,284	1,053	1,028	
286	Canada	2009-2011	Canadian Health Measures Survey, Cycle 2	National	Both	5-79	5-79	1,402	1,394	1,189	1,148	
287	Canada	2011-2013	International Study of Childhood Obesity, Lifestyle and the Environment (ISCOLE)	Community	Urban	9-11	9-11	239	328	238	327	
288	Canada	2012-2013	Canadian Health Measures Survey, Cycle 3	National	Both	5-79	5-79	1,289	1,277	1,106	1,095	
289	Canada	2014-2015	Canadian Health Measures Survey, Cycle 4	National	Both	5-79	5-79	1,297	1,297	1,131	1,116	
290	Canada	2016-2017	Canadian Health Measures Survey, Cycle 5	National	Both	6-79	6-79	1,190	1,175	1,034	1,028	
291	Central African Republic	1994-1995	DHS	National	Both		20-49		1,225			
292	Central African Republic	2010	STEPS	Subnational	Both	25-64	25-64	390	540			
293	Chad	1996-1997	DHS	National	Both		20-49		2,254			
294	Chad	2004	DHS	National	Both		20-49		1,746			
295	Chad	2008	STEPS	Community	Urban	25-64	25-64	240	220			
296	Chad	2014-2015	DHS	National	Both		15-49		6,515		2,172	
297	Chile	1992-1993	Miquel et al., Gastroenterology 115: 937-46, 1998	Community	Urban	18+	18+	206	304	8	14	
298	Chile	2000	Nervi et al., J Hepatol 45:299 -305, 2006	Community	Urban	18+	18+	2	15			
299	Chile	2003	Encuesta Nacional de Salud	National	Both	17+	17+	306	307	63	77	
300	Chile	2004-2005	Cardiovascular Risk Factors Multiple Evaluation in Latin America	Community	Urban	25-64	25-64	182	202			
301	Chile	2009-2010	Encuesta Nacional de Salud	National	Both	15+	15+	441	623	169	191	
302	Chile	2010-2011	Encuesta Nacional de Consumo Alimentario	National	Both	5+	5+	837	978	591	631	
303	Chile	2013	Global School-based Student Health Survey	National	Both	13-17	13-17	799	793	799	793	
304	Chile	2016-2017	Encuesta Nacional de Salud	National	Both	15+	15+	453	631	159	183	
305	China	1982	China National Nutrition Survey	National	Both	7+	7+	1,266	1,200	8,232	6,592	3
306	China	1985	Chinese National Surveys on Students Constitution and Health	National	Both	7-18	7-18	205,041	204,796	205,041	204,796	
307	China	1989	China Health and Nutrition Study	National	Both	5-45	5-45	200	167	197	166	4
308	China	1991	China Health and Nutrition Study	National	Both	5+	5+	2,762	2,850	1,730	1,661	4
309	China	1992	China National Nutrition Survey	National	Both	5+	5+	8,807	10,303	10,998	10,377	4
310	China	1993	China Health and Nutrition Study	National	Both	5+	5+	2,574	2,462	1,677	1,500	4
311	China	1993	Sino-MONICA Anhui	Subnational	Urban	25-64	25-64	26	39			
312	China	1993	Sino-MONICA Beijing	Subnational	Both	25-64	25-64	48	66			
313	China	1993	Sino-MONICA Jiangsu	Subnational	Urban	25-64	25-64	49	63			
314	China	1993	Sino-MONICA Liaoning	Subnational	Both	25-64	25-64	56	40			
315	China	1995	Chinese National Surveys on Students Constitution and Health	National	Both	7-18	7-18	103,009	101,772	103,009	101,772	
316	China	1997	China Health and Nutrition Study	National	Both	5+	5+	2,418	2,223	1,532	1,366	4
317	China	2000	China Health and Nutrition Study	National	Both	5+	5+	2,192	2,041	1,452	1,277	4
318	China	2000	Chinese National Surveys on Students Constitution and Health	National	Both	7-18	7-18	107,997	108,096	107,997	108,096	
319	China	1996-2003	Wu et al., Osteoporos Int 15:751-59, 2004	Community	Urban		18+				114	
320	China	2002	China National Nutrition and Health Survey	National	Both	5-101	5-101	29,026	28,161	20,760	18,743	
321	China	2004	Beijing Child and Adolescent Metabolic Syndrome Study	Community	Both	5-18	5-18	10,564	10,413	10,562	10,412	
322	China	2004	China Health and Nutrition Study	National	Both	5+	5+	1,487	1,331	968	828	4
323	China	2004-2005	Xinjiang Children and Adolescent Survey	Community	Urban	6-18	6-18	2,035	2,240	2,030	2,233	
324	China	2005	Chinese National Surveys on Students Constitution and Health	National	Both	7-18	7-18	117,598	116,704	117,598	116,704	
325	China	2006	China Health and Nutrition Study	National	Both	5+	5+	1,189	1,096	787	676	4
326	China	2007	Beijing Child and Adolescent Metabolic Syndrome Study	Community	Urban	7-18	7-18	863	664	863	664	
327	China	2009	China Health and Nutrition Study	National	Both	5+	5+	1,170	1,080	740	602	4
328	China	2009	The nutrition-based comprehensive intervention study on childhood obesity in China	Subnational	Urban	6-11	6-11	4,495	4,269	4,495	4,269	
329	China	2009-2010	China National Survey of Chronic Kidney Disease	National	Both	18+	18+	2,651	2,717	123	92	
330	China	2010	China Noncommunicable Disease Surveillance	National	Both	18+	18+	7,204	7,443	1,229	1,254	
331	China	2010	Chinese National Surveys on Students Constitution and Health	National	Both	7-18	7-18	107,611	107,611	107,611	107,611	
332	China	2011	Beijing Childhood Eye Study	Community	Both	7-18	7-18	6,886	7,301	6,686	6,967	
333	China	2011	Beijing Children Eye Study	Community	Both	5-13	5-13	306	275	291	261	
334	China	2011	China Health and Nutrition Study	National	Both	5+	5+	1,457	1,506	943	881	4
335	China	2012	Beijing Children Eye Study	Community	Both	5-13	5-13	284	257	283	251	
336	China	2012	Beijing High School Eye Students Study	Community	Both	16-18	16-18	2,103	2,407	2,088	2,340	

	Country	Study years	Survey/Study name/Citation	Level of representativeness	Rural, urban or both	Age range as in NCD-RisC database*		Sample size as in height analysis		Sample size as in BMI analysis		Note
						Male	Female	Male	Female	Male	Female	
337	China	2011-2013	International Study of Childhood Obesity, Lifestyle and the Environment (ISCOLE)	Community	Urban	9-11	9-11	293	259	293	258	
338	China	2012	Shandong Children Study	Community	Rural	5-18	5-18	1,663	1,385	1,663	1,385	
339	China	2012	Shandong Children Study	Community	Urban	5-18	5-18	1,423	1,381	1,423	1,381	
340	China	2010-2014	National Free Preconception Health Examination Project	National	Rural	20-64		10,576,140				
341	China	2013	Gobi Desert Children Eye Study	Community	Urban	6-21	6-21	800	761	797	761	
342	China	2012-2013	The Kailuan Study	Community	Urban	18+	18+	6,475	1,260	39		
343	China	2014	Shanghai Municipal Surveys on Students Constitution and Health	Community	Both	7-18	7-18	7,758	7,665	7,758	7,665	
344	China	2015	China Health and Nutrition Study	National	Both	5+	5+	1,149	966	665	608	4
345	China	2014-2015	The Kailuan Study	Community	Urban	18+	18+	6,170	880	2		
346	China	2016	Greater Beijing School Children Myopia Study	Subnational	Rural	6-18	6-18	12,874	12,866	12,873	12,866	
347	China	2016	Greater Beijing School Children Myopia Study	Subnational	Urban	6-18	6-18	4,385	3,650	4,384	3,650	
348	China (Hong Kong SAR)	1995-1996	Hong Kong Cardiovascular Risk Factor Prevalence Study 1995-1996	National	Both	25-74	25-74	132	104			
349	China (Hong Kong SAR)	2005-2006	Hong Kong Growth Survey	National	Both	7-19	7-19	7,472	7,370	7,472	7,370	
350	China (Hong Kong SAR)	2016-2018	Smart device usage, lifestyles behaviors, physical fitness, and eye problems: A prospective study in Hong Kong adolescents	Community	Urban	7-15	7-15	703	741	703	741	
351	Colombia	1995	DHS	National	Both		20-49		2,117			
352	Colombia	2000	DHS	National	Both		20-49		1,944			
353	Colombia	2002	Factores de riesgo cardiovascular en la localidad de Santa Fe de la ciudad de Bogotá. Resultados obtenidos en el área demostrativa Carmen	Community	Urban	15-69	15-69	168	269	60	86	
354	Colombia	2002	Factores de riesgo cardiovascular en la localidad de Tunjuelito de la ciudad de Bogotá. Resultados obtenidos en el área demostrativa Carmen	Community	Urban	15-29	15-29	208	312	93	127	
355	Colombia	2005	DHS	National	Both	5-64	5-64	27,551	33,673	20,154	21,521	
356	Colombia	2005	Encuesta Nacional de Situacion Nutricional	National	Both	5-12	5-49	2,646	5,005	2,644	3,534	
357	Colombia	2004-2005	Cardiovascular Risk Factors Multiple Evaluation in Latin America	Community	Urban	25-64	25-64	169	182			
358	Colombia	2007	Encuesta Nacional de Salud	National	Both	18-69	18-69	1,860	2,489	350	394	
359	Colombia	2010	DHS	National	Both	5-64	5-64	39,428	43,675	28,359	28,434	
360	Colombia	2010	STEPS	Subnational	Both	15-64	15-64	403	527	167	195	
361	Colombia	2011-2013	International Study of Childhood Obesity, Lifestyle and the Environment (ISCOLE)	Community	Urban	9-11	9-11	454	462	454	462	
362	Colombia	2015	Encuesta Nacional de Situacion Nutricional	National	Both	5-64	5-64	2,769	2,957	1,848	1,798	
363	Colombia	2015	STEPS	Subnational	Both	15-64	15-64	394	324	173	75	
364	Comoros	1996	DHS	National	Both		20-49		431			
365	Comoros	2011	STEPS	National	Both	25-64	25-64	299	837			
366	Comoros	2012	DHS	National	Both		15-49		3,113		1,207	
367	Congo	1986	Enquête Brazzaville 1986	Community	Urban	5-50	5-50	129	197	129	196	
368	Congo	1987	Enquête Nationale Congo 1987	National	Rural		13-49		264		252	
369	Congo	1991	Enquête Brazzaville 1991	Community	Urban	5-90	5-90	1,692	2,503	1,309	1,516	
370	Congo	1996	Enquête Brazzaville 1996	Community	Urban	5-90	5-90	1,716	2,372	1,283	1,455	
371	Congo	2004	STEPS	Community	Urban	25-64	25-64	276	303			
372	Congo	2005	DHS	National	Both		15-49		4,177		1,388	
373	Congo	2011-2012	DHS	National	Both		15-49		3,121		1,031	
374	Cook Islands	2003	STEPS	National	Both	25-64	25-64	80	113			
375	Cook Islands	2011	Global School-based Student Health Survey	National	Both	13-17	13-17	530	543	530	543	
376	Cook Islands	2013-2015	STEPS	National	Both	18-64	18-64	77	119	10	23	
377	Cook Islands	2015	Global School-based Student Health Survey	National	Both	13-17	13-17	305	313	304	313	
378	Costa Rica	2004	CAMDI	Community	Urban	20+	20+	75	162			
379	Costa Rica	2009	Global School-based Student Health Survey	National	Both	13	13-17	356	1,308	356	1,308	
380	Costa Rica	2010	Costa Rican National Cardiovascular Risk Factors Survey, 2010	National	Both	20+	20+	136	323			
381	Costa Rica	2014	Costa Rican National Cardiovascular Risk Factors Survey, 2014	National	Both	20+	20+	159	391			
382	Cote d'Ivoire	1994	DHS	National	Both		20-49		1,774			
383	Cote d'Ivoire	1998-1999	DHS	National	Both		15-49		1,913		735	
384	Cote d'Ivoire	2005	STEPS	Subnational	Rural	15-64	15-64	322	481	88	108	
385	Cote d'Ivoire	2005	STEPS	Subnational	Urban	15-64	15-64	515	875	152	265	
386	Cote d'Ivoire	2011-2012	DHS	National	Both		15-49		3,004		958	
387	Croatia	2003	Croatian Adult Health Survey 2003	National	Both	18+	18+	241	627			
388	Croatia	2003-2004	School Health Survey	National	Both	6-19	6-19	1,504	1,299	1,504	1,299	
389	Croatia	2002-2007	Epidemiology of arterial hypertension in Croatia (EH-UH)	National	Both	18+	18+	49	60			
390	Croatia	2005	Endemic Nephropathy and Arterial Hypertension (ENAH)	Subnational	Rural	18+	18+	37	45		3	

	Country	Study years	Survey/Study name/Citation	Level of representativeness	Rural, urban or both	Age range as in NCD-RisC database*		Sample size as in height analysis		Sample size as in BMI analysis		Note
						Male	Female	Male	Female	Male	Female	
391	Croatia	2006-2008	The Cardiovascular risk factors in school age – intervention model development	National	Both	6-20	6-20	6,012	5,625	6,011	5,625	
392	Croatia	2008	Endemic Nephropathy and Arterial Hypertension (ENAH)	Subnational	Rural	18+	18+	45	63	10	8	
393	Croatia	2010	Endemic Nephropathy and Arterial Hypertension (ENAH)	Subnational	Rural	18+	18+	30	54	3	5	
394	Croatia	2014	Croatian Physical Activity in Adolescence Longitudinal Study (CRO-PALS)	Community	Urban	14-17	14-17	429	413	428	410	
395	Croatia	2015	Endemic Nephropathy and Arterial Hypertension (ENAH) Follow-up Study	Subnational	Rural	18+	18+	10	15			
396	Croatia	2015-2016	Childhood Obesity Surveillance Initiative 4	National	Both	8	8	1,365	1,365	1,364	1,364	1
397	Croatia	2016-2017	Croatian Physical Activity in Adolescence Longitudinal Study (CRO-PALS)	Community	Urban	17-20	17-20	383	384	383	384	
398	Cuba	2010	National Risk Factor Survey	National	Both	15+	15+	786	782	244	255	
399	Cuba	2011	Non communicable disease risk factor in Cienfuegos	Community	Urban	15-80	15-80	129	176	41	56	
400	Cyprus	2007-2008	Asthma Study Cyprus	National	Both	15-18	15-18	352	506	368	490	
401	Cyprus	2007-2008	Childhood asthma and atopy in Cyprus	Subnational	Both	7-9, 13-15	7-9, 13-15	566	590	566	590	
402	Cyprus	2007-2010	Identification and prevention of Dietary- and lifestyle-induced health EFfects In Children and infantS (IDEFICS)	Community	Urban	5-9	5-9	1,129	1,106	1,129	1,106	
403	Cyprus	2015-2016	Childhood Obesity Surveillance Initiative 4	National	Urban	6-9	6-9	687	623	685	623	1
404	Czech Republic	1971	3rd Nationwide Anthropometric Survey of Children and Adolescents 1971	National	Both	5-17	5-17	2,832	2,933			
405	Czech Republic	1981	4th Nationwide Anthropometric Survey of Children and Adolescents 1981	National	Both	5-17	5-17	37,006	38,420			
406	Czech Republic	1991	5th Nationwide Anthropometric Survey of Children and Adolescents 1991	National	Both	5-17	5-17	34,641	35,659			
407	Czech Republic	1997-1998	Czech post-MONICA	National	Both	25-64	25-64	140	159			
408	Czech Republic	2000-2001	Czech post-MONICA	National	Both	25-64	25-64	151	166			
409	Czech Republic	2001	6th Nationwide Anthropometric Survey of Children and Adolescents 2001	National	Both	5-19	5-20			18,960	22,426	
410	Czech Republic	2008	Childhood Obesity Surveillance Initiative 1	National	Both	6-7	6-7	834	838	834	838	1
411	Czech Republic	2006-2009	Czech post-MONICA	National	Both	25-64	25-64	135	156			
412	Czech Republic	2010	Childhood Obesity Surveillance Initiative 2	National	Both	6-7	6-7	1,203	1,239	1,203	1,239	1
413	Czech Republic	2013	Childhood Obesity Surveillance Initiative 3	National	Both	6-7	6-7	1,267	1,200	1,267	1,200	1
414	Czech Republic	2014-2015	European Heath Examination Survey	National	Both	25-64	25-64	43	50			
415	Czech Republic	2015-2016	Childhood Obesity Surveillance Initiative 4	National	Both	6-7	6-7	809	884	809	883	1
416	Czech Republic	2015-2018	MONICA	National	Both	25-65	25-65	61	87			
417	Denmark	1972	Copenhagen School Health Records Register	Community	Urban	6-13	6-13	549	605			
418	Denmark	1973	Copenhagen School Health Records Register	Community	Urban	6-13	6-13	2,986	3,051			
419	Denmark	1974	Copenhagen School Health Records Register	Community	Urban	6-13	6-13	4,760	4,758			
420	Denmark	1975	Copenhagen School Health Records Register	Community	Urban	6-13	6-13	6,096	5,931			
421	Denmark	1976	Copenhagen School Health Records Register	Community	Urban	6-13	6-13	7,670	7,724			
422	Denmark	1977	Copenhagen School Health Records Register	Community	Urban	6-13	6-13	8,980	8,683			
423	Denmark	1978	Copenhagen School Health Records Register	Community	Urban	6-13	6-13	10,749	10,633			
424	Denmark	1979	Copenhagen School Health Records Register	Community	Urban	6-13	6-13	11,659	11,624			
425	Denmark	1980	Copenhagen School Health Records Register	Community	Urban	6-13	6-13	11,234	11,194			
426	Denmark	1981	Copenhagen School Health Records Register	Community	Urban	6-13	6-13	11,433	11,349			
427	Denmark	1982	Copenhagen School Health Records Register	Community	Urban	6-13	6-13	10,971	10,746			
428	Denmark	1983	Copenhagen School Health Records Register	Community	Urban	6-13	6-13	7,956	7,945			
429	Denmark	1984	Copenhagen School Health Records Register	Community	Urban	6-13	6-13	4,886	4,947			
430	Denmark	1985	Copenhagen School Health Records Register	Community	Urban	6-13	6-13	4,266	4,275	4,266	4,275	
431	Denmark	1986	Copenhagen School Health Records Register	Community	Urban	6-13	6-13	4,533	4,462	4,533	4,462	
432	Denmark	1987	Copenhagen School Health Records Register	Community	Urban	6-13	6-13	4,686	4,544	4,686	4,544	
433	Denmark	1988	Copenhagen School Health Records Register	Community	Urban	6-13	6-13	4,738	4,501	4,738	4,501	
434	Denmark	1989	Copenhagen School Health Records Register	Community	Urban	6-13	6-13	4,845	4,598	4,845	4,598	
435	Denmark	1990	Copenhagen School Health Records Register	Community	Urban	6-13	6-13	4,669	4,449	4,669	4,449	
436	Denmark	1991	Copenhagen School Health Records Register	Community	Urban	6-13	6-13	4,844	4,811	4,844	4,811	
437	Denmark	1992	Copenhagen School Health Records Register	Community	Urban	6-13	6-13	5,243	5,104	5,243	5,104	
438	Denmark	1993	Copenhagen School Health Records Register	Community	Urban	6-13	6-13	4,968	4,890	4,968	4,890	
439	Denmark	1991-1994	Copenhagen City Heart Study	Subnational	Urban	20+	20+	224	258			
440	Denmark	1994	Copenhagen School Health Records Register	Community	Urban	6-13	6-13	4,065	4,005	4,065	4,005	
441	Denmark	1995	Copenhagen School Health Records Register	Community	Urban	6-13	6-13	5,437	5,379	5,437	5,379	
442	Denmark	1996	Copenhagen School Health Records Register	Community	Urban	6-13	6-13	4,674	4,670	4,674	4,670	
443	Denmark	1997	Copenhagen School Health Records Register	Community	Urban	7-13	7-13	4,105	4,025	4,105	4,025	
444	Denmark	1998	Copenhagen School Health Records Register	Community	Urban	8-13	8-13	3,203	3,253	3,203	3,253	
445	Denmark	1997-1998	The European Youth Heart Study	Community	Urban	8-18	8-18	485	532	485	532	
446	Denmark	1999	Copenhagen School Health Records Register	Community	Urban	9-13	9-13	2,860	2,777	2,860	2,777	

	Country	Study years	Survey/Study name/Citation	Level of representativeness	Rural, urban or both	Age range as in NCD-RisC database*		Sample size as in height analysis		Sample size as in BMI analysis		Note
						Male	Female	Male	Female	Male	Female	
447	Denmark	2000	Copenhagen School Health Records Register	Community	Urban	10-13	10-13	1,911	1,923	1,911	1,923	
448	Denmark	2001	Copenhagen School Health Records Register	Community	Urban	11-13	11-13	1,594	1,595	1,594	1,595	
449	Denmark	2001-2002	The Copenhagen School Child Intervention Study	Community	Urban	5-8	5-8	362	329	362	329	
450	Denmark	2002	Copenhagen School Health Records Register	Community	Urban	12-13	12-13	860	895	860	895	
451	Denmark	2001-2003	Copenhagen City Heart Study	Subnational	Urban	20+	20+	171	281			
452	Denmark	2003	Copenhagen School Health Records Register	Community	Urban	13	13	322	369	322	369	
453	Denmark	2003-2004	The European Youth Heart Study	Community	Urban	8-17	8-17	392	509	392	509	
454	Denmark	2003-2004	Copenhagen General Population Study 1	Subnational	Urban	20+	20+	92	136			
455	Denmark	2004-2005	The Copenhagen School Child Intervention Study	Community	Urban	8-11	8-11	121	130	121	130	
456	Denmark	2005	Copenhagen General Population Study 1	Subnational	Urban	20+	20+	85	123			
457	Denmark	2006	Danish Conscript Register	National	Both	17-26		25,232		18,951		
458	Denmark	2006	Copenhagen General Population Study 1	Subnational	Urban	20+	20+	59	81			
459	Denmark	2007	Danish Conscript Register	National	Both	17-26		27,366		20,778		
460	Denmark	2006-2008	The Health2006 Cohort	Subnational	Urban	18-71	18-71	109	196	14	13	
461	Denmark	2007	Copenhagen General Population Study 1	Subnational	Urban	20+	20+	211	243			
462	Denmark	2008	The Childhood Health Activity and Motor Performance School Study	Community	Both	5-11	5-11	551	629	550	628	
463	Denmark	2008	Danish Conscript Register	National	Both	17-26		24,583		19,631		
464	Denmark	2008	The Copenhagen School Child Intervention Study	Community	Urban	12-14	12-14	99	112	99	111	
465	Denmark	2007-2008	The Danish Health Examination Survey 2007-2008	National	Both	18+	18+	365	703	63	100	
466	Denmark	2008	Copenhagen General Population Study 1	Subnational	Urban	20+	20+	100	129			
467	Denmark	2009	The Childhood Health Activity and Motor Performance School Study	Community	Both	6-11	6-11	256	258	256	258	
468	Denmark	2009	Danish Conscript Register	National	Both	17-26		27,100		20,100		
469	Denmark	2009	Copenhagen General Population Study 1	Subnational	Urban	20+	20+	43	43			
470	Denmark	2010	The Childhood Health Activity and Motor Performance School Study	Community	Both	7-13	7-13	262	258	262	258	
471	Denmark	2010	Danish Conscript Register	National	Both	17-26		30,815		23,061		
472	Denmark	2009-2010	The European Youth Heart Study	Community	Both	14-28	14-28	482	565	176	220	
473	Denmark	2010	Copenhagen General Population Study 1	Subnational	Urban	20+	20+	64	84			
474	Denmark	2011	The Childhood Health Activity and Motor Performance School Study	Community	Both	8-13	8-13	245	241	245	241	
475	Denmark	2011	Danish Conscript Register	National	Both	17-26		30,721		23,699		
476	Denmark	2011	Copenhagen General Population Study 1	Subnational	Urban	20+	20+	46	74			
477	Denmark	2012	The Childhood Health Activity and Motor Performance School Study	Community	Both	9-14	9-14	274	251	273	249	
478	Denmark	2012	Danish Conscript Register	National	Both	17-26		29,654		23,401		
479	Denmark	2011-2012	The OPUS School Meal Study	Subnational	Both	8-11	8-11	427	388	427	388	
480	Denmark	2012	Copenhagen General Population Study 1	Subnational	Urban	20+	20+	52	47			
481	Denmark	2011-2012	The Health2006 cohort - 5-year follow-up	Subnational	Urban	24-76	24-76	39	58			
482	Denmark	2013	The Childhood Health Activity and Motor Performance School Study	Community	Both	10-15	10-15	225	210	225	208	
483	Denmark	2013	Danish Conscript Register	National	Both	17-26		30,567		24,490		
484	Denmark	2013	LCoMotion	Subnational	Both	11-14	11-14	353	365	353	365	
485	Denmark	2013	Copenhagen General Population Study 1	Subnational	Urban	20+	20+	97	129			
486	Denmark	2014	Danish Conscript Register	National	Both	17-26		32,401		26,615		
487	Denmark	2012-2015	The Danish study of Functional Disorders (DanFunD)	Subnational	Urban	18-72	18-72	289	357	17	29	
488	Denmark	2014	Copenhagen General Population Study 2	Subnational	Urban	20+	20+	22	32			
489	Denmark	2015	The Childhood Health Activity and Motor Performance School Study	Community	Both	12-17	12-17	122	122	122	120	
490	Denmark	2015	Danish Conscript Register	National	Both	17-26		28,907		24,482		
491	Denmark	2014-2015	Copenhagen General Population Study 1	Subnational	Urban	20+	20+	50	65			
492	Denmark	2015	Copenhagen General Population Study 2	Subnational	Urban	20+	20+	87	93			
493	Denmark	2015-2016	Childhood Obesity Surveillance Initiative 4	National	Both	6-7	6-7	1,340	1,283	1,339	1,281	1
494	Denmark	2016	Conscript	National	Both	17-29		29,057		24,145		
495	Denmark	2016	Copenhagen General Population Study 2	Subnational	Urban	20+	20+	89	103			
496	Denmark	2017	Conscript	National	Both	17-29		31,057		26,415		
497	Denmark	2017	Copenhagen General Population Study 2	Subnational	Urban	20+	20+	31	46			
498	Denmark	2018	Conscript	National	Both	17-29		27,597		24,085		
499	Denmark	2019	Conscript	National	Both	17-29		27,412		22,663		
500	Dominica	2007	STEPS	National	Both	15-64	15-64	131	161	67	76	
501	Dominica	2009	Global School-based Student Health Survey	National	Both	13-17	13-17	509	542	508	542	
502	Dominican Republic	1991	DHS	National	Both		20-49		1,436			
503	Dominican Republic	1996	DHS	National	Both		15-49		4,494		1,613	

	Country	Study years	Survey/Study name/Citation	Level of representativeness	Rural, urban or both	Age range as in NCD-RisC database*		Sample size as in height analysis		Sample size as in BMI analysis		Note
						Male	Female	Male	Female	Male	Female	
504	Dominican Republic	1996-1998	Estudio factores de riesgo cardiovascular y sindrome metabolico en la Republica Dominicana I (EFRICARD I)	National	Both	18-75	18-75	228	594	18	49	
505	Dominican Republic	2010-2012	Estudio factores de riesgo cardiovascular y sindrome metabolico en la Republica Dominicana II (EFRICARD II)	National	Both	18-75	18-75	299	726	65	164	
506	Dominican Republic	2013	DHS	National	Both	15-59	15-49	4,872	4,967	1,896	1,767	
507	DR Congo	2001	Multiple Indicator Cluster Survey Round 2	National	Both		15-49		2,977		464	
508	DR Congo	2005	STEPS	Subnational	Urban	15+	15+	362	557	150	204	
509	DR Congo	2007	DHS	National	Both		15-49		2,901		859	
510	DR Congo	2013-2014	DHS	National	Both		15-49		5,544		1,791	
511	Ecuador	2004	Encuesta Demografica y de Salud Materno e Infantil/Reproductive Health Survey	National	Both		15-49		2,443		308	
512	Ecuador	2004-2005	Cardiovascular Risk Factors Multiple Evaluation in Latin America	Community	Urban	25-64	25-64	195	190			
513	Ecuador	2008-2009	Food Nutrition and Health	Community	Both	10-16	10-16	382	376	379	375	
514	Ecuador	2011-2013	Encuesta Nacional de Salud y Nutrición (ENSANUT)	National	Both	5-59	5-59	14,822	15,100	10,711	9,292	
515	Ecuador	2018	Encuesta Nacional de Salud y Nutrición	National	Both	5+	5+	34,423	37,486	23,716	22,970	
516	Egypt	1992	DHS	National	Both		20-49		2,650			
517	Egypt	1995	DHS	National	Both		20-49		3,836			
518	Egypt	2000	DHS	National	Both		20-49		5,026			
519	Egypt	2002	National Survey of Smoking, Obesity, Blood Pressure and Blood Glucose	National	Both	5+	5+	2,371	2,836	1,685	1,793	
520	Egypt	2003	DHS	National	Both		20-49		3,159			
521	Egypt	2003-2004	Marzouk et al., Gut 56(8):1105-10, 2007	Community	Rural	25+	25+	39	66			
522	Egypt	2005	STEPS	National	Both	15-65	15-65	2,148	2,102	812	659	
523	Egypt	2005	DHS	National	Both		20-49		6,725			
524	Egypt	2008	DHS	National	Both	10-59	20-49	11,328	6,570	9,694		
525	Egypt	2011	STEPS	National	Both	15-65	15-65	533	965	185	222	
526	Egypt	2011	Global School-based Student Health Survey	National	Both	13-17	13-17	325	454	325	454	
527	Egypt	2014	DHS	National	Both		20-49		7,717			
528	Egypt	2015	DHS	National	Both	15-59	15-59	3,035	3,988	1,226	1,330	
529	Egypt	2017	STEPS	National	Both	15-69	15-69	638	947	279	316	
530	El Salvador	2002-2003	Ecuesta Nacional de Salud Familiar	National	Both		15-49		2,677		381	
531	El Salvador	2004	CAMDI	Community	Urban	20+	20+	107	173			
532	El Salvador	2008	Ecuesta Nacional de Salud Familiar	National	Both		15-49		3,798		942	
533	El Salvador	2014-2015	Encuesta Nacional de Enfermedades Crónicas 2014-2015 (ENECA-ELS)	National	Both	20+	20+	399	749			
534	Eritrea	1995	DHS	National	Both		15-49		966			
535	Eritrea	2002	DHS	National	Both		15-49		1,784			
536	Eritrea	2004	STEPS	National	Both	15-64	15-64	356	375	139	123	
537	Eritrea	2010	STEPS	National	Both	25-74	25-74	147	911			
538	Estonia	1997	Pomerleau et al., Public Health Nutrition 3:3-10, 2000	National	Both	19-64	19-64	183	225	13	12	
539	Estonia	2002	Estonian Biobank	National	Both	18+	18+	21	53	5	10	
540	Estonia	2003	Estonian Biobank	National	Both	18+	18+	646	1,114	153	186	
541	Estonia	2004	Estonian Biobank	National	Both	18+	18+	145	218	38	42	
542	Estonia	2007	Estonian Biobank	National	Both	18+	18+	258	475	56	98	
543	Estonia	2008	Estonian Biobank	National	Both	18+	18+	1,644	2,830	324	508	
544	Estonia	2009	Estonian Biobank	National	Both	18+	18+	1,177	1,384	210	293	
545	Estonia	2007-2010	Identification and prevention of Dietary- and lifestyle-induced health EFfects In Children and infantS (IDEFICS)	Community	Urban	5-9	5-9	558	634	558	634	
546	Estonia	2010	Estonian Biobank	National	Both	18+	18+	1,381	1,679	291	311	
547	Estonia	2011	Estonian Biobank	National	Both	18+	18+	26	63	3	14	
548	Estonia	2012	Estonian Biobank	National	Both	18+	18+	26	56		7	
549	Estonia	2013	Estonian Biobank	National	Both	18+	18+	32	57	5	11	
550	Estonia	2013-2015	National Dietary Survey (RTU) 2014	National	Both	5-74	5-74	574	754	400	476	
551	Estonia	2015-2016	Childhood Obesity Surveillance Initiative 4	National	Both	7-8	7-8	6,508	6,202	6,502	6,198	1
552	Estonia	2018-2019	Childhood Obesity Surveillance Initiative 5	National	Both	7-11	7-11	6,067	6,038	6,066	6,038	1
553	Eswatini	2006-2007	DHS	National	Both	15-49	15-49	2,726	3,025	1,249	1,197	
554	Eswatini	2007	STEPS	National	Both	25-64	25-64	109	164			
555	Eswatini	2014	STEPS	National	Both	15-69	15-69	509	738	204	231	
556	Ethiopia	2000	DHS	National	Both		15-49		9,051		3,384	
557	Ethiopia	2005	DHS	National	Both		15-49		4,018		1,486	

	Country	Study years	Survey/Study name/Citation	Level of representativeness	Rural, urban or both	Age range as in NCD-RisC database*		Sample size as in height analysis		Sample size as in BMI analysis		Note
						Male	Female	Male	Female	Male	Female	
558	Ethiopia	2006	STEPS	Subnational	Urban	25-64	25-64	369	372			
559	Ethiopia	2011	DHS	National	Both	15-59	15-49	7,484	9,981	2,821	3,676	
560	Ethiopia	2016	DHS	National	Both	15-59	15-49	6,283	8,919	2,446	3,226	
561	Fiji	2002	STEPS	National	Both	15-64	15-64	954	1,366	321	412	
562	Fiji	2005-2007	Pacific Obesity Prevention in Communities – Healthy Youth Health Communities Study	Subnational	Urban	11-19	11-19	3,730	4,109	3,730	4,109	
563	Fiji	2007-2008	Pacific Obesity Prevention in Communities – Healthy Youth Health Communities Study	Subnational	Urban	13-22	13-22	1,492	1,832	1,479	1,813	
564	Fiji	2010	Global School-based Student Health Survey	National	Both	13-17	13-17	630	892	629	892	
565	Fiji	2011	STEPS	National	Both	25-64	25-64	123	162			
566	Fiji	2016	Global School-based Student Health Survey	National	Both	13-17	13-17	1,394	1,469	1,394	1,469	
567	Finland	1980	Young Finns Study 1980	National	Rural	5-18	5-18	480	507			
568	Finland	1980	Young Finns Study 1980	National	Urban	5-18	5-18	440	443			
569	Finland	1983	Young Finns Study 1983	National	Rural	6-21	6-21	561	575	666	692	3
570	Finland	1983	Young Finns Study 1983	National	Urban	6-21	6-21	472	481	566	597	3
571	Finland	1986	Young Finns Study 1986	National	Rural	9-24	9-24	477	503	476	502	
572	Finland	1986	Young Finns Study 1986	National	Urban	9-24	9-24	420	455	420	454	
573	Finland	1997	The National FINRISK Study	National	Both	25-74	25-74	343	402			
574	Finland	2001	Young Finns Study 2001	National	Rural	24-39	24-39	146	172			
575	Finland	2001	Young Finns Study 2001	National	Urban	24-39	24-39	341	409			
576	Finland	2001-2002	North Finland Birth Cohort 1986	Community	Both	15-17	15-17	3,259	3,356	3,251	3,348	
577	Finland	2002	The National FINRISK Study	National	Both	25-74	25-74	278	426			
578	Finland	2007	Young Finns Study 2007	National	Rural	30-45	30-45	57	64			
579	Finland	2007	Young Finns Study 2007	National	Urban	30-45	30-45	103	97			
580	Finland	2007	The National FINRISK Study	National	Both	25-74	25-74	216	295			
581	Finland	2011-2013	International Study of Childhood Obesity, Lifestyle and the Environment (ISCOLE)	Community	Urban	9-11	9-11	253	283	253	282	
582	Finland	2012	The National FINRISK Study	National	Both	25-74	25-74	203	262			
583	Finland	2015-2016	Childhood Obesity Surveillance Initiative 4	National	Both	7-9	7-9	6,598	6,388	6,598	6,388	1
584	Finland	2017	The FinHealth Survey	National	Both	18+	18+	241	280	11	19	
585	France	2000	Corpulence 7-9 ans	Subnational	Both	7-9	7-9	786	796	786	796	
586	France	2006-2007	HELENA	Community	Urban	12-17	12-17	122	165	122	165	
587	France	2007	Corpulence 7-9 ans	National	Both	7-9	7-9	1,281	1,244	1,281	1,244	
588	France	2006-2007	Etude Nationale Nutrition Santé	National	Both	5-74	5-74	819	876	726	730	
589	France	2014-2016	Esteban	National	Both	6-74	6-74	641	625	569	552	
590	France	2015-2016	Childhood Obesity Surveillance Initiative 4	National	Both	7-9	7-9	2,510	2,561	2,510	2,561	1
591	French Polynesia	2010	STEPS	National	Both	18-64	18-64	396	524	69	92	
592	French Polynesia	2015	Global School-based Student Health Survey	National	Both	12-17	12-17	1,310	1,438	1,310	1,438	
593	Gabon	2000	DHS	National	Both		20-49		1,385			
594	Gabon	2009	STEPS	Subnational	Urban	15-64	15-64	321	695	77	157	
595	Gabon	2012	DHS	National	Both		15-49		3,131		1,123	
596	Georgia	2010	STEPS	National	Both	18-64	18-64	392	678	97	144	
597	Georgia	2016	STEPS	National	Both	18-69	18-69	204	352	26	49	
598	Georgia	2015-2016	Childhood Obesity Surveillance Initiative 4	National	Both	7-8	7-8	1,687	1,585	1,685	1,585	1
599	Germany	1985	The German Conscription Database	Subnational	Both	19		402,641		402,487		
600	Germany	1986	The German Conscription Database	Subnational	Both	19		382,672		382,632		
601	Germany	1987	The German Conscription Database	Subnational	Both	19		349,092		349,083		
602	Germany	1988	The German Conscription Database	Subnational	Both	19		303,277		303,265		
603	Germany	1989	The German Conscription Database	Subnational	Both	19		245,755		245,740		
604	Germany	1990	The German Conscription Database	Subnational	Both	19		206,615		206,599		
605	Germany	1991	The German Conscription Database	National	Both	19		138,205		138,195		
606	Germany	1992	The German Conscription Database	National	Both	19		220,992		220,956		
607	Germany	1993	The German Conscription Database	National	Both	19		188,700		188,655		
608	Germany	1994	The German Conscription Database	National	Both	19		155,442		155,426		
609	Germany	1993-1994	MONICA, Chemnitz	Community	Urban	25-64	25-64	37	34			
610	Germany	1993-1994	MONICA, Zwickau	Community	Urban	25-64	25-64	13	20			
611	Germany	1995	The German Conscription Database	National	Both	19		185,793		185,762		
612	Germany	1994-1995	MONICA, Augsburg	Community	Both	25-64	25-64	194	217			
613	Germany	1996	The German Conscription Database	National	Both	19		191,265		191,260		
614	Germany	1997	The German Conscription Database	National	Both	19		148,774		148,738		

	Country	Study years	Survey/Study name/Citation	Level of representativeness	Rural, urban or both	Age range as in NCD-RisC database*		Sample size as in height analysis		Sample size as in BMI analysis		Note
						Male	Female	Male	Female	Male	Female	
615	Germany	1997-1999	German National Health Interview and Examination Survey (GNHIES98)	National	Both	18-79	18-79	645	634	142	124	
616	Germany	1998	The German Conscription Database	National	Both	19		146,563		146,528		
617	Germany	1999	The German Conscription Database	National	Both	19		292,829		292,732		
618	Germany	1997-2001	Study of Health in Pomerania (SHIP-0) baseline study	Subnational	Both	20-80	20-80	274	318			
619	Germany	1999-2001	KORA S4 Study: Kooperative Research in the Region of Augsburg Survey 4	Community	Both	24-75	24-75	150	173			
620	Germany	2002	Echinoccoccus Multilocularis and Internal Diseases in Leutkirch	Community	Urban	12-65	12-65	313	316	162	177	
621	Germany	2002-2006	Study of Health in Pomerania (SHIP-1) 5-year follow-up	Subnational	Both	25-85	25-85	60	86			
622	Germany	2003-2006	German Health Interview and Examination Survey for Children and Adolescents (KiGGS)	National	Both	5-17	5-17	6,622	6,286	6,602	6,260	
623	Germany	2006-2007	HELENA	Community	Urban	12-17	12-17	282	194	282	194	
624	Germany	2008	The German Conscription Database	National	Both	19		98,942		98,926		
625	Germany	2009	The German Conscription Database	National	Both	19		111,461		111,455		
626	Germany	2007-2010	Identification and prevention of Dietary- and lifestyle-induced health EFfects In Children and infantS (IDEFICS)	Community	Urban	5-9	5-9	772	762	772	762	
627	Germany	2010	The German Conscription Database	National	Both	19		101,928		101,911		
628	Germany	2008-2011	German Health Interview and Examination Survey for adults 2008-11 (DEGS1)	National	Both	18-79	18-79	520	545	123	92	
629	Germany	2008-2012	Study of Health in Pomerania, second cohort (SHIP-TREND)	Subnational	Both	20-79	20-79	190	194			
630	Germany	2014-2017	German Health Interview and Examination Survey for Children and Adolescents (KiGGS2)	National	Both	5-17	5-17	1,541	1,584	1,541	1,584	
631	Ghana	1993	DHS	National	Both		20-49		946			
632	Ghana	1998	DHS	National	Both		20-49		1,098			
633	Ghana	2003	DHS	National	Both		15-49		2,940		989	
634	Ghana	2003	Women's Health Study of Accra (WHSA-I)	Community	Urban		18+		265		57	
635	Ghana	2006	STEPS	Community	Urban	25+	25+	162	321			
636	Ghana	2008	DHS	National	Both		15-49		2,665		959	
637	Ghana	2008-2010	Women's Health Study of Accra (WHSA-II)	Community	Urban		18+		621			
638	Ghana	2012-2014	Research on Obesity and Diabetes among African Migrants (RODAM), control group	Subnational	Rural	25+	25+	54	66			
639	Ghana	2012-2014	Research on Obesity and Diabetes among African Migrants (RODAM), control group	Subnational	Urban	25+	25+	40	108			
640	Ghana	2014	DHS	National	Both	15-59	15-49	2,103	2,523	896	883	
641	Greece	1991-1999	EPIC	National	Both	19-86	19-86	174	256			
642	Greece	1997	The Didima Study	Community	Rural	20+	18+	30	49		5	
643	Greece	2003	National Epidemiological Survey	National	Both	13-19	13-19	6,677	7,779	6,675	7,778	
644	Greece	2004-2005	Arsakeion School Study	Community	Urban	6-18	6-18	358	420	358	420	
645	Greece	2005	Daphne	Community	Rural	17-18	17-18	41	57	41	57	
646	Greece	2006	Samos	Community	Both	5-13	5-13	53	67	55	65	
647	Greece	2006-2007	HELENA, Athens	Community	Urban	12-17	12-17	158	162	158	162	
648	Greece	2006-2007	HELENA, Heraklion	Community	Urban	12-17	12-17	135	149	135	149	
649	Greece	2007-2009	Healthy Growth Study	Subnational	Both	9-13	9-13	1,307	1,291	1,305	1,284	
650	Greece	2008-2009	Greek Childhood Obesity Study (GRECO)	National	Both	10-12	10-12	2,046	2,164	2,033	2,160	
651	Greece	2010-2012	ADONUT	National	Both	12-19	12-19	18,669	18,675	18,668	18,675	
652	Greece	2010-2011	Childhood Obesity Surveillance Initiative 2	National	Both	7-9	7-9	2,581	2,688	2,581	2,688	1
653	Greece	2013-2015	Hellenic National Nutrition and Health Survey (HNNHS)	National	Both	5+	5+	747	959	309	326	
654	Greece	2014-2015	Evaluation of a Web-based Dietary Intervention Among Primary School Children (NUTRI-WEB Children Project)	Community	Both	7-12	7-12	387	434	387	433	
655	Greece	2015-2016	Childhood Obesity Surveillance Initiative 4	National	Both	7-9	7-9	1,908	1,873	1,907	1,872	1
656	Greece	2016-2017	Erasmus plus KA2, Healthyland	Community	Rural	5	5	13	9	13	9	
657	Greece	2017-2018	Erasmus plus KA2, Healthyland	Community	Rural	5-6	5-6	33	12	33	12	
658	Greenland	2005-2010	Population Health Survey in Greenland	National	Both	18+	18+	242	333	40	50	
659	Grenada	2011	STEPS	National	Both	25-64	25-64	77	103			
660	Guatemala	1995	DHS	National	Both		20-49		2,737			
661	Guatemala	1998-1999	DHS	National	Both		20-49		1,368			
662	Guatemala	2002	Reproductive Health Survey	National	Both	15-59	15-49	1,012	4,466	387	1,305	
663	Guatemala	2001-2002	CAMDI	Community	Urban	20+	20+	76	176			
664	Guatemala	2003-2005	The Institute of Nutrition of Central America and Panama Nutrition Supplementation Trial Cohort	Community	Both	25-41	25-41	84	98			
665	Guatemala	2008-2009	Encuesta Nacional de Salud Materno Infantil	National	Both	15-59	15-49	3,032	8,722	1,244	2,632	
666	Guatemala	2014-2015	DHS	National	Both		15-49		13,387		5,398	
667	Guinea	1999	DHS	National	Both		20-49		1,715			
668	Guinea	2005	DHS	National	Both		15-49		2,033		740	
669	Guinea	2009	STEPS	Subnational	Both	15-64	15-64	432	657	149	203	

	Country	Study years	Survey/Study name/Citation	Level of representativeness	Rural, urban or both	Age range as in NCD-RisC database*		Sample size as in height analysis		Sample size as in BMI analysis		Note
						Male	Female	Male	Female	Male	Female	
670	Guinea	2012	DHS	National	Both		15-49		2,708		997	
671	Guinea	2018	DHS	National	Both		15-49		3,069		1,176	
672	Guinea Bissau	2010	Multiple Indicator Cluster Survey	National	Both		15-49		5,116		1,734	
673	Guyana	2009	DHS	National	Both	15-49	15-49	1,641	2,392	702	913	
674	Guyana	2010	Global School-based Student Health Survey	National	Both	13-17	13-17	988	1,261	987	1,261	
675	Guyana	2016	STEPS	National	Both	18-69	18-69	273	448	54	72	
676	Haiti	1994-1995	DHS	National	Both		20-49		1,013			
677	Haiti	2000	DHS	National	Both		15-49		5,696		2,277	
678	Haiti	2005-2006	DHS	National	Both		15-49		3,227		1,320	
679	Haiti	2012	DHS	National	Both		15-49		5,761		2,266	
680	Haiti	2015-2016	Carrefour	Community	Urban	25-65	25-65	149	196			
681	Haiti	2015-2016	Thomonde	Community	Rural	25-65	25-65	38	74			
682	Haiti	2016-2017	DHS	National	Both		15-49		5,526		2,169	
683	Honduras	1996	Honduras National Micronutrient Survey	National	Both		20-40		481			
684	Honduras	2003-2004	CAMDI	Community	Urban	20+	20+	117	216			
685	Honduras	2005-2006	DHS	National	Both		15-49		11,181		4,132	
686	Honduras	2011-2012	DHS	National	Both		15-49		12,697		4,802	
687	Hungary	2006-2007	HELENA	Community	Urban	12-17	12-17	197	197	197	197	
688	Hungary	2007-2010	Identification and prevention of Dietary- and lifestyle-induced health EFfects In Children and infantS (IDEFICS)	Community	Urban	5-9	5-9	1,128	1,169	1,128	1,169	
689	Hungary	2010	Childhood Obesity Surveillance Initiative 2	National	Both	7	7	553	682	553	682	1
690	Hungary	2015-2016	Childhood Obesity Surveillance Initiative 4	National	Both	6-8	6-8	2,753	2,579	2,750	2,572	1
691	Hungary	2016	Feel4Diabetes	Community	Both	6-10	6-10	1,447	1,522	1,447	1,522	
692	Iceland	1993-1994	MONICA, Arnes County	Community	Rural	25-64	25-64	43	56			
693	Iceland	1993-1994	MONICA, Reykjavik	Subnational	Urban	25-64	25-64	16	40			
694	Iceland	2005-2011	Risk Evaluation For INfarct Estimates (REFINE)	Subnational	Urban	20-73	20-73	232	249			
695	Iceland	2010-2012	Risk Evaluation For INfarct Estimates (REFINE) follow-up visit (REFINELO)	Subnational	Urban	26-74	26-74	6	13			
696	India	1974	National Nutrition Monitoring Bureau rural survey (Kerala)	Subnational	Rural	1-59	1-59	85	62			
697	India	1974	National Nutrition Monitoring Bureau rural survey (Tamil Nadu)	Subnational	Rural	1-59	1-59	140	129			
698	India	1974	National Nutrition Monitoring Bureau rural survey (Karnataka)	Subnational	Rural	1-59	1-59	98	75			
699	India	1974	National Nutrition Monitoring Bureau rural survey (Andhra Pradesh)	Subnational	Rural	1-59	1-59	179	195			
700	India	1974	National Nutrition Monitoring Bureau rural survey (Maharashtra)	Subnational	Rural	1-59	1-59	51	39			
701	India	1974	National Nutrition Monitoring Bureau rural survey (Gujarat)	Subnational	Rural	1-59	1-59	256	248			
702	India	1974	National Nutrition Monitoring Bureau rural survey (Madhya Pradesh)	Subnational	Rural	1-59	1-59	309	227			
703	India	1974	National Nutrition Monitoring Bureau rural survey (West Bengal)	Subnational	Rural	1-59	1-59	200	175			
704	India	1974	National Nutrition Monitoring Bureau rural survey (Uttar Pradesh)	Subnational	Rural	1-59	1-59	64	47			
705	India	1975-1979	National Nutrition Monitoring Bureau Rural Survey	National	Rural	5+	5+	14,170	9,831			
706	India	1980	National Nutrition Monitoring Bureau rural survey (Kerala)	Subnational	Rural	5-59	5-59	212	177			
707	India	1980	National Nutrition Monitoring Bureau rural survey (Tamil Nadu)	Subnational	Rural	5-59	5-59	643	489			
708	India	1980	National Nutrition Monitoring Bureau rural survey (Karnataka)	Subnational	Rural	5-59	5-59	734	697			
709	India	1980	National Nutrition Monitoring Bureau rural survey (Andhra Pradesh)	Subnational	Rural	5-59	5-59	1,257	920			
710	India	1980	National Nutrition Monitoring Bureau rural survey (Gujarat)	Subnational	Rural	5-59	5-59	317	230			
711	India	1980	National Nutrition Monitoring Bureau rural survey (Orissa)	Subnational	Rural	5-59	5-59	276	307			
712	India	1980	National Nutrition Monitoring Bureau rural survey (West Bengal)	Subnational	Rural	5-59	5-59	673	641			
713	India	1980	National Nutrition Monitoring Bureau rural survey (Uttar Pradesh)	Subnational	Rural	5-59	5-59	511	283			
714	India	1981	National Nutrition Monitoring Bureau rural survey (Tamil Nadu)	Subnational	Rural	5-59	5-59	687	506			
715	India	1981	National Nutrition Monitoring Bureau rural survey (Karnataka)	Subnational	Rural	5-59	5-59	503	418			
716	India	1981	National Nutrition Monitoring Bureau rural survey (Andhra Pradesh)	Subnational	Rural	5-59	5-59	783	574			
717	India	1981	National Nutrition Monitoring Bureau rural survey (Maharashtra)	Subnational	Rural	5-59	5-59	371	336			
718	India	1981	National Nutrition Monitoring Bureau rural survey (Orissa)	Subnational	Rural	5-59	5-59	163	154			
719	India	1981	National Nutrition Monitoring Bureau rural survey (West Bengal)	Subnational	Rural	5-59	5-59	361	334			
720	India	1981	National Nutrition Monitoring Bureau rural survey (Uttar Pradesh)	Subnational	Rural	5-59	5-59	330	195			
721	India	1982	National Nutrition Monitoring Bureau rural survey (Kerala)	Subnational	Rural	5-59	5-59	251	245			
722	India	1982	National Nutrition Monitoring Bureau rural survey (Tamil Nadu)	Subnational	Rural	5-59	5-59	550	490			
723	India	1982	National Nutrition Monitoring Bureau rural survey (Karnataka)	Subnational	Rural	5-59	5-59	481	417			
724	India	1982	National Nutrition Monitoring Bureau rural survey (Andhra Pradesh)	Subnational	Rural	5-59	5-59	493	358			
725	India	1982	National Nutrition Monitoring Bureau rural survey (Maharashtra)	Subnational	Rural	5-59	5-59	623	490			

	Country	Study years	Survey/Study name/Citation	Level of representativeness	Rural, urban or both	Age range as in NCD-RisC database*		Sample size as in height analysis		Sample size as in BMI analysis		Note
						Male	Female	Male	Female	Male	Female	
726	India	1982	National Nutrition Monitoring Bureau rural survey (Gujarat)	Subnational	Rural	5-59	5-59	209	195			
727	India	1982	National Nutrition Monitoring Bureau rural survey (Orissa)	Subnational	Rural	5-59	5-59	141	155			
728	India	1982	National Nutrition Monitoring Bureau rural survey (West Bengal)	Subnational	Rural	5-59	5-59	65	57			
729	India	1982-1983	Bengali School Children	Community	Urban	7-21		816				
730	India	1983-1984	National Nutrition Monitoring Bureau rural survey (Tamil Nadu)	Subnational	Rural	5-59	5-59	792	805			
731	India	1983-1984	National Nutrition Monitoring Bureau rural survey (Andhra Pradesh)	Subnational	Rural	5-59	5-59	660	777			
732	India	1983-1984	National Nutrition Monitoring Bureau rural survey (Orissa)	Subnational	Rural	5-59	5-59	708	794			
733	India	1983-1984	National Nutrition Monitoring Bureau rural survey (Gujarat)	Subnational	Rural	5-59	5-59	925	872			
734	India	1990	National Nutrition Monitoring Bureau rural survey	National	Rural	5+	5+	3,141	3,464	3,139	3,464	
735	India	1992-1994	Jaipur Heart Watch 1	Community	Rural	20-80	20-80	560	374			
736	India	1992-1994	Jaipur Heart Watch 1	Community	Urban	20-80	20-80	505	124			
737	India	1995	Shobana et al., Diabetes Res Clin Pract 42(3):181–86, 1998	Community	Urban	20-74	20-74	192	233			
738	India	1995-1996	Epidemiology of blood pressure across cross-cultural populations of Visakhapatnam district, Andhra Pradesh, India	Community	Rural	20-76	19-76	47	54		2	
739	India	1996	Nutrition Profile of Indians (Jind District, Haryana)	Subnational	Rural	5-59	5-59	342	403			
740	India	1996	Nutrition Profile of Indians (Kaithal District, Haryana)	Subnational	Rural	5-59	5-59	368	381			
741	India	1996	Nutrition Profile of Indians (Karnal District, Haryana)	Subnational	Rural	5-59	5-59	156	295			
742	India	1996	Nutrition Profile of Indians (Kurkeshetra District, Haryana)	Subnational	Rural	5-59	5-59	250	345			
743	India	1996	Nutrition Profile of Indians (Mahendragarh District, Haryana)	Subnational	Rural	5-59	5-59	338	423			
744	India	1996	Nutrition Profile of Indians (Bhatinda District, Punjab)	Subnational	Rural	5-59	5-59	328	418			
745	India	1996	Nutrition Profile of Indians (Faridkot District, Punjab)	Subnational	Rural	5-59	5-59	329	440			
746	India	1996	Nutrition Profile of Indians (Ferozpur District, Punjab)	Subnational	Rural	5-59	5-59	361	499			
747	India	1996	Nutrition Profile of Indians (Gurdaspur District, Punjab)	Subnational	Rural	5-59	5-59	388	481			
748	India	1996	Nutrition Profile of Indians (Hoshiarpur District, Punjab)	Subnational	Rural	5-59	5-59	260	318			
749	India	1996	Nutrition Profile of Indians (Amritsar District, Punjab)	Subnational	Rural	5-59	5-59	376	507			
750	India	1996-1997	National Nutrition Monitoring Bureau rural survey	National	Rural	5+	5+	13,591	17,589	10,093	10,790	
751	India	1997	Ramachandran et al., Diabetes Res Clin Pract 44(3):207–13, 1999	Community	Rural	20-74	20-74	264	385			
752	India	1996-1999	Chennai Urban Population Study	Community	Urban	20+	20+	138	136			
753	India	1998-1999	DHS	National	Both		20-49		31,749			
754	India	2000	Ramachandran et al., Diabet Med,20(3):220-24, 2003	Subnational	Urban	20-75	20-75	1,257	1,299			
755	India	1999-2001	Jaipur Heart Watch 2	Community	Urban	20-75	20-75	91	88			
756	India	1998-2002	Vellore Birth Cohort	Subnational	Both	25-31	25-31	1,094	963			
757	India	1999-2002	Bengali School Children	Community	Urban	7-21		1,152		1,152		
758	India	2000-2001	National Nutrition Monitoring Bureau rural survey	National	Rural	5+	5+	10,281	14,500	7,615	8,479	
759	India	1999-2002	New Delhi Birth Cohort	Community	Urban	26-33	26-33	633	457			
760	India	2001-2002	Nutrition Profile of Indians (Uttarkashi District, Uttar Pradesh)	Subnational	Rural	5-59	5-59	367	471			
761	India	2001-2002	Nutrition Profile of Indians (Chamoli District, Uttar Pradesh)	Subnational	Rural	5-59	5-59	350	432			
762	India	2001-2002	Nutrition Profile of Indians (Tehrigarhwal District, Uttar Pradesh)	Subnational	Rural	5-59	5-59	291	354			
763	India	2001-2002	Nutrition Profile of Indians (Dehradun District, Uttar Pradesh)	Subnational	Rural	5-59	5-59	321	421			
764	India	2001-2002	Nutrition Profile of Indians (Pauri District, Uttar Pradesh)	Subnational	Rural	5-59	5-59	221	286			
765	India	2001-2002	Nutrition Profile of Indians (West Bengal)	Subnational	Rural	5-59	5-59	4,460	6,856			
766	India	2001-2002	Nutrition Profile of Indians (Orissa)	Subnational	Rural	5-59	5-59	12,019	13,886			
767	India	2002-2003	Blood Pressure epidemiology in tribal, rural and urban communities of Orissa with special reference to physical and social parameters	Community	Rural	18-80	18-80	56	61	7	9	
768	India	2001-2004	Chennai Urban Rural Epidemiology Study	Community	Urban	20+	20+	237	327			
769	India	2003-2004	ICMR RF RHD Registry, Jai Vigyan Mission Mode, Kochi	Subnational	Both	5-16	5-16	11,327	13,515	11,327	13,515	
770	India	2003-2005	India STEPS Ballabgarh	Subnational	Rural	15-69	15-69	476	496	164	88	
771	India	2003-2005	India STEPS Ballabgarh	Subnational	Urban	15-69	15-69	365	387	119	99	
772	India	2003-2005	India STEPS Chennai	Subnational	Rural	15-69	15-69	399	396	133	148	
773	India	2003-2005	India STEPS Chennai	Subnational	Urban	15-69	15-69	389	391	119	64	
774	India	2003-2005	India STEPS Delhi	Subnational	Urban	15-69	15-69	397	344	137	145	
775	India	2003-2005	India STEPS Dibrugarh	Subnational	Rural	15-69	15-69	414	512	119	106	
776	India	2003-2005	India STEPS Dibrugarh	Subnational	Urban	15-69	15-69	355	389	99	95	
777	India	2003-2005	India STEPS Nagpur	Subnational	Rural	15-69	15-69	386	393	112	71	
778	India	2003-2005	India STEPS Nagpur	Subnational	Urban	15-69	15-69	386	375	86	87	
779	India	2003-2005	India STEPS Trivandrum	Subnational	Rural	15-69	15-69	331	405	130	92	
780	India	2003-2005	India STEPS Trivandrum	Subnational	Urban	15-69	15-69	360	357	132	108	

	Country	Study years	Survey/Study name/Citation	Level of representativeness	Rural, urban or both	Age range as in NCD-RisC database*		Sample size as in height analysis		Sample size as in BMI analysis		Note
						Male	Female	Male	Female	Male	Female	
781	India	2005-2006	DHS	National	Both	15-54	15-49	35,133	66,021	12,515	22,318	
782	India	2005-2006	Risk factor profile for chronic non-communicable diseases: Results of a community-based study in Kerala, India	Community	Both	15-64	15-64	796	870	273	209	
783	India	2005-2006	National Nutrition Monitoring Bureau rural survey	National	Rural	5+	5+	10,564	13,320	7,133	7,648	
784	India	2005-2006	ICMR RF RHD Registry, Jai Vigyan Mission Mode, Kochi	Subnational	Both	5-16	5-16	9,754	10,509	9,754	10,509	
785	India	2006	Ramachandran et al., Diabetes Care 31(5):893-98, 2008	Community	Both	20+	20+	1,085	979			
786	India	2005-2007	Prevalence of cardiovascular risk factors in rural Tamil Nadu	Community	Rural	25-65	25-65	1,001	1,090			
787	India	2006-2008	Kashmiri Young Adults	Subnational	Both	20-40	20-40	916	445			
788	India	2005-2011	Bengali School Children	Community	Urban	7-21	7-21	847	2,183	580	1,884	
789	India	2007-2008	Integrated Disease Surveillance Project Non-communicable Disease Risk Factors Survey Andhra Pradesh	Subnational	Both	15-64	15-64	1,106	1,567	180	194	
790	India	2007-2008	Integrated Disease Surveillance Project Non-communicable Disease Risk Factors Survey Kerala	Subnational	Both	15-64	15-64	605	1,122	147	130	
791	India	2007-2008	Integrated Disease Surveillance Project Non-communicable Disease Risk Factors Survey Madhya Pradesh	Subnational	Both	15-64	15-64	1,378	1,359	308	221	
792	India	2007-2008	Integrated Disease Surveillance Project Non-communicable Disease Risk Factors Survey Maharashtra	Subnational	Both	15-64	15-64	1,268	1,303	258	220	
793	India	2007-2008	Integrated Disease Surveillance Project Non-communicable Disease Risk Factors Survey Mizoram	Subnational	Both	15-64	15-64	1,151	1,317	209	224	
794	India	2007-2008	Integrated Disease Surveillance Project Non-communicable Disease Risk Factors Survey Tamil Nadu	Subnational	Both	15-64	15-64	811	1,341	144	155	
795	India	2007-2008	Integrated Disease Surveillance Project Non-communicable Disease Risk Factors Survey Uttarakhand	Subnational	Both	15-64	15-64	1,010	1,665	273	283	
796	India	2007-2009	Prevalence of NCD risk factor in people above 15 year in rural area Nagpur using WHO STEP approach	Community	Rural	15+	15+	694	609	262	256	
797	India	2008-2010	ICMR India Diabetes Study	National	Both	20+	20+	1,800	2,033			
798	India	2009-2010	Baseline survey for the assessment of prevalence of risk factors of NCDs in Gandhinagar district	Community	Rural	15-64	15-64	309	244	121	67	
799	India	2009-2010	Baseline survey for the assessment of prevalence of risk factors of NCDs in Gandhinagar district	Community	Urban	15-64	15-64	289	280	103	79	
800	India	2010	Kerala 2010 follow-up	Community	Rural	21-70	21-70	41	32			
801	India	2011-2012	Body Mass Index, Social Conditions and Environmental Effect on High Blood Pressure Among the Adolescent School Children	National	Both	12-16	12-16	1,097	1,161	1,096	1,161	
802	India	2011-2013	International Study of Childhood Obesity, Lifestyle and the Environment (ISCOLE)	Community	Urban	9-11	9-11	292	328	292	328	
803	India	2011-2012	National Nutrition Monitoring Bureau Rural Survey	National	Rural	5+	5+	17,159	21,149	11,621	12,403	
804	India	2012-2013	Health Survey in Anand School Children	Community	Both	5-13	5-13	1,630	961	1,628	959	
805	India	2012-2013	District Level Household and Facility Survey (DLHS) 4	National	Both	5+	5+	253,224	271,681	157,993	152,769	
806	India	2012-2013	ICMR-India Diabetes (INDIAB) Study, Phase II	Subnational	Both	20+	20+	1,676	2,461			
807	India	2014	Annual Health Survey-Chemical, Anthropometric	National	Both	5+	5+	361,281	366,551	245,191	230,014	
808	India	2012-2015	ICMR-India Diabetes (INDIAB) Study, North East Phase	National	Both	20+	20+	2,209	3,273			
809	India	2015-2016	DHS	National	Both	15-54	15-49	50,210	356,491	18,490	118,744	
810	India	2015-2016	Diet and nutritional status of urban population and prevalence of hypertension	National	Urban	5+	5+	30,065	34,512	19,180	18,822	
811	Indonesia	1993-1994	Indonesian Family Life Surveys	National	Both	5+	5+	4,038	4,821	3,216	3,275	
812	Indonesia	1997-1998	Indonesian Family Life Surveys	National	Both	5+	5+	6,737	7,314	4,963	5,007	
813	Indonesia	2000-2001	Indonesian Family Life Surveys	National	Both	5+	5+	8,637	9,134	5,580	5,561	
814	Indonesia	2003	A genetic-ecological study of the risk factors for lifestyle-related diseases in Oceanian populations, Study A	Community	Rural	18-79	18-79	23	44	3	13	
815	Indonesia	2003	A genetic-ecological study of the risk factors for lifestyle-related diseases in Oceanian populations, Study B	Community	Rural	18-79	18-79	45	58	13	12	
816	Indonesia	2007-2008	Indonesian Family Life Surveys	National	Both	5+	5+	9,395	10,037	5,779	5,630	
817	Indonesia	2011	SEANUTS	National	Both	5-12	5-12	1,363	1,380	1,363	1,380	
818	Indonesia	2013	Population Health Basic Health Research 2013 (Riskesdas 2013)	National	Both	5-19	5-19	155,850	146,291	155,332	145,926	
819	Indonesia	2013	Population Health Basic Health Research 2013 (Riskesdas 2013)	National	Both	20+	20+	60,693	66,157			
820	Indonesia	2015	Global School-based Student Health Survey	National	Both	13-17	13-17	3,845	4,688	3,845	4,688	
821	Indonesia	2014-2015	Indonesian Family Life Surveys	National	Both	5+	5+	10,008	10,772	6,814	6,639	
822	Iran	1990-1991	National Health Survey I	National	Both	5-18	5-18	9,083	9,220	8,883	9,038	
823	Iran	1999-2000	National Health Survey II	National	Both	5+	5+	14,738	16,429	11,032	11,446	
824	Iran	1999-2001	Tehran Lipid and Glucose Study	Community	Urban	5+	5+	2,878	3,626	2,033	2,226	
825	Iran	2001	Isfahan Healthy Heart Program, Arak rural	Community	Rural	19+	19+	345	346			
826	Iran	2001	Isfahan Healthy Heart Program, Arak urban	Community	Urban	19+	19+	685	641			
827	Iran	2001	Isfahan Healthy Heart Program, Isfahan rural	Community	Rural	19+	19+	81	82			
828	Iran	2001	Isfahan Healthy Heart Program, Isfahan urban	Community	Urban	19+	19+	596	584			
829	Iran	2001	Isfahan Healthy Heart Program, Najaf Abad rural	Community	Rural	19+	19+	139	122			

	Country	Study years	Survey/Study name/Citation	Level of representativeness	Rural, urban or both	Age range as in NCD-RisC database*		Sample size as in height analysis		Sample size as in BMI analysis		Note
						Male	Female	Male	Female	Male	Female	
830	Iran	2001	Isfahan Healthy Heart Program, Najaf Abad urban	Community	Urban	19+	19+	188	181			
831	Iran	2001	Isfahan Healthy Heart Program in Students, Arak rural	Community	Rural	11-18	11-18			145	193	
832	Iran	2001	Isfahan Healthy Heart Program in Students, Arak urban	Community	Urban	11-18	11-18			326	306	
833	Iran	2001	Isfahan Healthy Heart Program in Students, Isfahan rural	Community	Rural	11-18	11-18			89	120	
834	Iran	2001	Isfahan Healthy Heart Program in Students, Isfahan urban	Community	Urban	11-18	11-18			243	225	
835	Iran	2001	Isfahan Healthy Heart Program in Students, Najaf Abad rural	Community	Rural	11-18	11-18			61	74	
836	Iran	2001	Isfahan Healthy Heart Program in Students, Najaf Abad urban	Community	Urban	11-18	11-18			62	72	
837	Iran	2003-2004	Childhood and Adolescence Surveillance and Prevention of Adult Noncommunicable Disease (CASPIAN)	National	Both	6-18	6-18	10,805	10,180	10,791	10,170	
838	Iran	2002-2005	Tehran Lipid and Glucose Study	Community	Urban	5+	5+	1,079	1,385	698	790	
839	Iran	2003-2004	The Persian Gulf Healthy Heart Study	Subnational	Urban	25-75	25-75	326	350			
840	Iran	2005	Dastgiri et al., J Public Health Nutr 2006; 9: 996-1000	Subnational	Urban	15-70	15-70	51	66	14	21	
841	Iran	2005	STEPS	National	Both	15-64	15-64	12,409	12,022	4,546	4,069	
842	Iran	2006	STEPS	National	Both	16-65	16-65	4,437	4,224	1,470	1,238	
843	Iran	2007	STEPS	National	Both	15-64	15-64	749	703	258	195	
844	Iran	2007	STEPS	National	Both	15-64	15-64	4,640	4,358	1,678	1,347	
845	Iran	2005-2008	Tehran Lipid and Glucose Study	Community	Urban	5+	5+	1,201	1,448	660	701	
846	Iran	2007	Isfahan Healthy Heart Program, Arak rural	Community	Rural	19+	19+	391	280			
847	Iran	2007	Isfahan Healthy Heart Program, Arak urban	Community	Urban	19+	19+	503	476			
848	Iran	2007	Isfahan Healthy Heart Program, Isfahan rural	Community	Rural	19+	19+	60	47			
849	Iran	2007	Isfahan Healthy Heart Program, Isfahan urban	Community	Urban	19+	19+	493	436			
850	Iran	2007	Isfahan Healthy Heart Program, Najaf Abad rural	Community	Rural	19+	19+	99	88			
851	Iran	2007	Isfahan Healthy Heart Program, Najaf Abad urban	Community	Urban	19+	19+	161	206			
852	Iran	2007	Isfahan Healthy Heart Program in Students, Arak rural	Community	Rural	11-18	11-18			177	164	
853	Iran	2007	Isfahan Healthy Heart Program in Students, Arak urban	Community	Urban	11-18	11-18			329	342	
854	Iran	2007	Isfahan Healthy Heart Program in Students, Isfahan rural	Community	Rural	11-18	11-18			16	19	
855	Iran	2007	Isfahan Healthy Heart Program in Students, Isfahan urban	Community	Urban	11-18	11-18			398	338	
856	Iran	2007	Isfahan Healthy Heart Program in Students, Najaf Abad rural	Community	Rural	11-18	11-18			38	38	
857	Iran	2007	Isfahan Healthy Heart Program in Students, Najaf Abad urban	Community	Urban	11-18	11-18			43	67	
858	Iran	2008	STEPS	National	Both	15-64	15-64	4,596	4,384	1,706	1,366	
859	Iran	2009	STEPS	National	Both	15-64	15-64	4,717	4,370	1,594	1,347	
860	Iran	2009-2010	Childhood and Adolescence Surveillance and Prevention of Adult Noncommunicable Disease (CASPIAN)	National	Both	10-18	10-18	2,799	2,814	2,799	2,814	
861	Iran	2008-2011	Tehran Lipid and Glucose Study	Community	Urban	20+	20+	999	1,318			
862	Iran	2011	STEPS	National	Both	6-69	6-69	2,203	2,507	1,018	1,027	
863	Iran	2011-2012	Amol county study	Community	Rural	10+	10+	546	325	258	124	
864	Iran	2011-2012	Amol county study	Community	Urban	10+	10+	471	375	176	142	
865	Iran	2011-2012	Childhood and Adolescence Surveillance and Prevention of Adult Noncommunicable Disease (CASPIAN)	National	Both	6-18	6-18	6,659	6,448	6,649	6,443	
866	Iran	2012	National Integrated Micronutrient Survey (NIMS) 2012	National	Both	6-60	6-60	6,066	6,510	5,649	5,978	
867	Iran	2012-2013	Tehran City	Community	Urban	10-90	10-90	116	136	39	41	
868	Iran	2012-2013	Zahedan City	Community	Urban	10-90	10-90	578	471	306	187	
869	Iran	2013-2014	Isfahan Salt Study (ISS)	Community	Urban	6-18	6-18	400	383	400	383	
870	Iran	2014-2015	Childhood and Adolescence Surveillance and Prevention of Adult Noncommunicable Disease (CASPIAN)	National	Both	7-18	7-18	7,177	6,974	7,164	6,966	
871	Iran	2015	Iranian School Measurement Database	National	Both	6-18	6-18	912,264	913,385	911,584	912,687	
872	Iran	2016	STEPS	National	Both	18+	18+	2,832	3,098	204	221	
873	Iran	2018-2019	Prevalence of risk factors for cardiovascular disease among a rural population in eastern Iran	Subnational	Rural	18+	18+	32	20		2	
874	Iran	2016-2019	The Khuzestan Comprehensive Health Study: A platform for NCDs, blood borne and mental diseases research	Subnational	Both	20-65	20-65	1,910	3,786			
875	Iraq	2006	STEPS	National	Both	25-64	25-64	1,046	948			
876	Iraq	2015	STEPS	National	Both	18+	18+	411	556	78	88	
877	Ireland	1998	Survey of Lifestyle, Attitudes and Nutritional in Ireland 1998	National	Both	18+	18+	25	46		6	
878	Ireland	2002	Survey of Lifestyle, Attitudes and Nutritional in Ireland 2002	National	Both	18+	18+	18	32		6	
879	Ireland	2003-2004	National Children Food Survey	National	Both	5-12	5-12	293	301	293	301	
880	Ireland	2005-2006	National Teens Food Survey	National	Both	13-17	13-17	223	218	224	216	
881	Ireland	2006-2007	Survey of Lifestyle, Attitudes and Nutritional in Ireland 2006-2007	National	Both	18+	18+	162	171	27	28	
882	Ireland	2008	Childhood Obesity Surveillance Initiative 1	National	Both	7	7	1,098	1,285	1,098	1,285	1

	Country	Study years	Survey/Study name/Citation	Level of representativeness	Rural, urban or both	Age range as in NCD-RisC database*		Sample size as in height analysis		Sample size as in BMI analysis		Note
						Male	Female	Male	Female	Male	Female	
883	Ireland	2008-2010	National Adult Nutrition Survey	National	Both	18+	18+	192	168	36	19	
884	Ireland	2010	Murtagh et al., Pediatric Exercise 25(2):300-07, 2013; School A	Community	Rural	7-12	7-12	20	11	20	11	
885	Ireland	2010	Murtagh et al., Pediatric Exercise 25(2):300-07, 2014; School B	Community	Rural	7-12	7-12	19	19	19	19	
886	Ireland	2010	Murtagh et al., Pediatric Exercise 25(2):300-07, 2015; School C	Community	Rural	7-12	7-12	14	12	14	12	
887	Ireland	2010	Murtagh et al., Pediatric Exercise 25(2):300-07, 2016; School D	Community	Rural	7-12	7-12	16	21	16	21	
888	Ireland	2010	Childhood Obesity Surveillance Initiative 2	National	Both	6-9	6-9	1,452	1,533	1,452	1,533	1
889	Ireland	2012-2013	Childhood Obesity Surveillance Initiative 3	National	Both	6-9	6-9	1,087	1,054	1,087	1,054	1
890	Ireland	2015	Active Classrooms Study	Community	Both	8-11	8-11	124	120	124	120	
891	Ireland	2013-2016	Project Spraoi	Community	Both	5-11	5-11	474	429	474	429	
892	Ireland	2015-2016	Childhood Obesity Surveillance Initiative 4	National	Both	6-10	6-10	1,441	1,681	1,441	1,679	1
893	Israel	1999-2001	Mabat First Israeli National Health and Nutrition Survey	National	Both	25-64	25-64	191	191			
894	Israel	2003-2004	Mabat Youth First Israeli National Health and Nutrition Survey in 7th-12th grade students	National	Both	12-18	12-18	2,579	3,152	2,553	3,073	
895	Israel	2002-2007	Hadera District Study	Subnational	Urban	25-78	25-78	35	43			
896	Israel	2014-2016	Mabat Second Israeli National Health and Nutrition Survey	National	Both	18-64	18-64	284	282	31	29	
897	Israel	2015-2016	Mabat Youth Second Israeli National Health and Nutrition Survey in 7th-12th grade students	National	Both	12-18	12-18	2,256	2,431	2,221	2,415	
898	Italy	1980-1982	Po River Delta Epidemiological Study - first survey	Community	Rural	8-64	8-64	289	288			
899	Italy	1986-1987	Malattie cardiovascolari ATerosclerotiche Istituto Superiore di Sanità (MATISS)	Community	Rural	20-72	19-72		6		6	
900	Italy	1985-1988	Pisa Epidemiological Study - first survey	Community	Urban	5-90	5-90	388	309	388	309	
901	Italy	1989	Ventimiglia Heart Study	Community	Rural	6+	6+	124	132	124	132	
902	Italy	1988-1991	Po River Delta Epidemiological Study - second survey	Community	Rural	8-73	8-73	251	253	251	253	
903	Italy	1991-1993	Pisa Epidemiological Study - second survey	Community	Urban	8-97	8-97	356	352	166	146	
904	Italy	1993-1996	Malattie cardiovascolari ATerosclerotiche Istituto Superiore di Sanità (MATISS)	Community	Rural	20-77	20-77	199	212			
905	Italy	1994	MONICA, Friuli	Subnational	Urban	25-64	25-64	100	97			
906	Italy	1993-1994	MONICA, Brianza	Subnational	Urban	25-64	25-64	81	95			
907	Italy	1993-1998	EPIC Florence	Community	Urban	24-72	30-72	7				
908	Italy	1998-1999	Progetto VIP	Community	Both	25-74	25-74	60	60			
909	Italy	2000	Sorveglianza Nutrizionale Infanzia e Adolescenza (SoNIA)	Subnational	Both	13-15	13-15	236	244	236	244	
910	Italy	2000-2001	Sorveglianza Nutrizionale Infanzia e Adolescenza (SoNIA)	Subnational	Both	8-9	8-9	444	413	444	413	
911	Italy	2003	Sorveglianza Nutrizionale Infanzia e Adolescenza (SoNIA)	Subnational	Both	5-6	5-6	1,355	1,327	1,355	1,327	
912	Italy	2005-2007	Moli-family Study	Subnational	Both	14+	14+	99	128	43	55	
913	Italy	2006-2007	HELENA	Community	Urban	12-17	12-17	119	185	119	185	
914	Italy	2008	Childhood Obesity Surveillance Initiative 1	National	Both	8-9	8-9	4,101	3,896	4,100	3,896	1
915	Italy	2009	The ZOOM8 study: Nutrition and physical activity of primary school children	National	Both	6-11	6-11	1,083	1,045	1,083	1,045	
916	Italy	2007-2010	Identification and prevention of Dietary- and lifestyle-induced health EFfects In Children and infantS (IDEFICS)	Community	Urban	5-9	5-9	893	847	893	847	
917	Italy	2008-2009	Progetto VIP	Community	Both	25-74	25-74	46	49			
918	Italy	2010	Childhood Obesity Surveillance Initiative 2	National	Both	8-9	8-9	21,476	20,194	21,474	20,190	1
919	Italy	2009-2011	Pisa Epidemiological Study - third survey	Community	Urban	6+	6+	54	55	19	22	
920	Italy	2009-2010	Grosso et al., J Epidemiol 24(4):327-33, 2014	Community	Both	19+	19+	105	152	8	10	
921	Italy	2010-2012	CAMELIA	Community	Both	18-75	18-75	56	78	10	10	
922	Italy	2011	Grosso et al., Nutrients 5(12):4908-23, 2013	Community	Rural	13-16	13-16	115	89	115	89	
923	Italy	2011	Grosso et al., Nutrients 5(12):4908-23, 2013	Community	Urban	13-16	13-16	512	419	512	419	
924	Italy	2011	CONVERGI Study	Community	Urban	13-19	13-19	159	269	159	269	
925	Italy	2011-2012	ALImentazione e stile di vita negli ADOlescenti (ALIADO) (Nutrition and lifestyle in adolescents)	Community	Urban	15-16	15-16	149	194	149	194	
926	Italy	2012	Childhood Obesity Surveillance Initiative 3	National	Both	8-9	8-9	23,139	22,407	23,137	22,405	1
927	Italy	2012-2014	Mistretta et al., Obes Res Clin Pract 13(6):511-21, 2019	Community	Urban	11-16	11-16	885	758	878	753	
928	Italy	2014	OKkio alla SALUTE	National	Both	8-9	8-9	24,462	22,855	24,457	22,855	
929	Italy	2014-2016	Mediterranean healthy Eating, Aging and Lifestyles (MEAL) study	Subnational	Urban	20+	20+	114	188			
930	Italy	2015-2016	Childhood Obesity Surveillance Initiative 4	National	Both	8-9	8-9	22,737	21,457	22,732	21,454	1
931	Italy	2016	EVA Tyrol Study South Italy	Subnational	Both	14-18	14-18	108	200	108	200	
932	Italy	2016	OKkio alla SALUTE	National	Both	8-9	8-9	22,736	21,457	22,732	21,454	
933	Italy	2018-2019	Progetto VIP	Community	Both	25-74	25-74	60	60			
934	Jamaica	2000-2001	Jamaica Health and Lifestyle Survey	National	Both	15-74	15-74	204	408	72	93	
935	Jamaica	2005	Jamaican Youth Risk and Resiliency Behaviour Survey 2005	National	Both	10-15	10-15	1,379	1,519	1,328	1,386	
936	Jamaica	2006-2007	Jamaica Youth Risk and Resiliency Behaviour Survey 2006	National	Both	15-19	15-19	585	701	585	701	
937	Jamaica	2007-2008	Jamaica Health and Lifestyle Survey	National	Both	15-74	15-74	261	525	86	148	
938	Jamaica	2010	Global School-based Student Health Survey	National	Both		13-17		788		787	

	Country	Study years	Survey/Study name/Citation	Level of representativeness	Rural, urban or both	Age range as in NCD-RisC database*		Sample size as in height analysis		Sample size as in BMI analysis		Note
						Male	Female	Male	Female	Male	Female	
939	Japan	1975	National Nutrition Survey	National	Both	5+	5+	847	872			
940	Japan	1976	National Nutrition Survey	National	Both	5+	5+	1,222	1,141			
941	Japan	1977	National Nutrition Survey	National	Both	5+	5+	1,215	1,088			
942	Japan	1978	National Nutrition Survey	National	Both	5+	5+	1,474	1,404			
943	Japan	1979	National Nutrition Survey	National	Both	5+	5+	1,656	1,577			
944	Japan	1980	National Cardiovascular Survey	National	Both	5+	5+	1,785	1,705			
945	Japan	1981	National Nutrition Survey	National	Both	5+	5+	1,613	1,607			
946	Japan	1982	National Nutrition Survey	National	Both	5+	5+	1,988	2,003	2,217	2,295	3
947	Japan	1983	National Nutrition Survey	National	Both	5+	5+	2,032	1,930	2,172	2,075	3
948	Japan	1984	National Nutrition Survey	National	Both	5+	5+	1,913	1,849	1,990	1,934	3
949	Japan	1985	National Nutrition Survey	National	Both	5+	5+	2,248	2,178	2,247	2,178	
950	Japan	1986	National Nutrition Survey	National	Both	5+	5+	2,187	2,075	2,187	2,074	
951	Japan	1987	National Nutrition Survey	National	Both	5+	5+	1,865	1,846	1,863	1,845	
952	Japan	1988	National Nutrition Survey	National	Both	5+	5+	1,912	1,840	1,912	1,838	
953	Japan	1989	National Nutrition Survey	National	Both	5+	5+	1,607	1,453	1,607	1,453	
954	Japan	1990	National Nutrition Survey and National Cardiovascular Survey	National	Both	5+	5+	1,657	1,527	1,655	1,526	
955	Japan	1991	National Nutrition Survey	National	Both	5+	5+	2,211	2,285	1,546	1,450	
956	Japan	1991	Konan Town Study	Community	Rural	20-79	20-79	5	4			
957	Japan	1992	National Nutrition Survey	National	Both	5+	5+	2,027	2,053	1,425	1,337	
958	Japan	1992	Konan Town Study	Community	Rural	20-79	20-79	6	4			
959	Japan	1993	Iwata kids health study	Community	Urban	10	10	513	485	513	485	
960	Japan	1993	National Nutrition Survey	National	Both	5+	5+	2,025	2,108	1,450	1,398	
961	Japan	1993	Konan Town Study	Community	Rural	20-79	20-79	2	4			
962	Japan	1994	Iwata kids health study	Community	Urban	10	10	569	567	569	567	
963	Japan	1994	National Nutrition Survey	National	Both	5+	5+	1,910	2,001	1,281	1,235	
964	Japan	1994	Konan Town Study	Community	Rural	20-79	20-79	6	9			
965	Japan	1995	Iwata kids health study	Community	Urban	10	10	524	567	524	567	
966	Japan	1995	National Nutrition Survey	National	Both	5+	5+	1,932	1,997	1,307	1,211	
967	Japan	1995	Konan Town Study	Community	Rural	20-79	20-79	3	4			
968	Japan	1996	Iwata kids health study	Community	Urban	10	10	552	480	552	480	
969	Japan	1996	National Nutrition Survey	National	Both	5+	5+	1,739	1,871	1,104	1,090	
970	Japan	1997	Iwata kids health study	Community	Urban	10	10	506	537	506	537	
971	Japan	1997	National Nutrition Survey	National	Both	5+	5+	1,669	1,809	1,065	1,023	
972	Japan	1998	Iwata kids health study	Community	Urban	10	10	527	464	527	464	
973	Japan	1998	National Nutrition Survey	National	Both	5+	5+	1,684	1,740	1,090	1,043	
974	Japan	1999	Iwata kids health study	Community	Urban	10	10	468	463	468	463	
975	Japan	1999	National Nutrition Survey	National	Both	5+	5+	1,321	1,498	835	816	
976	Japan	2000	Iwata kids health study	Community	Urban	10	10	440	401	440	401	
977	Japan	2000	National Nutrition Survey and National Cardiovascular Survey	National	Both	5+	5+	1,422	1,480	902	925	
978	Japan	2001	Iwata kids health study	Community	Urban	10	10	452	414	452	414	
979	Japan	2001	National Nutrition Survey	National	Both	5+	5+	1,331	1,417	913	862	
980	Japan	2001	The Japan Association of Health Service Database	Subnational	Both	20+	20+	247,264	186,355			
981	Japan	2002	Iwata kids health study	Community	Urban	10	10	496	398	496	398	
982	Japan	2002	National Nutrition Survey	National	Both	5+	5+	1,101	1,194	719	736	
983	Japan	2003	Iwata kids health study	Community	Urban	10	10	415	399	415	399	
984	Japan	2003	National Health and Nutrition Survey	National	Both	5+	5+	1,061	1,157	674	703	
985	Japan	2004	Iwata kids health study	Community	Urban	10	10	463	412	463	412	
986	Japan	2004	National Health and Nutrition Survey	National	Both	5+	5+	941	913	642	546	
987	Japan	2005	Iwata kids health study	Community	Urban	10	10	476	420	476	420	
988	Japan	2005	National Health and Nutrition Survey	National	Both	5+	5+	797	835	510	529	
989	Japan	2006	Iwata kids health study	Community	Urban	10	10	417	391	417	391	
990	Japan	2006	National Health and Nutrition Survey	National	Both	5+	5+	891	918	615	584	
991	Japan	2007	Fukuroi kids health study	Community	Urban	13-14	13-14	395	372	395	372	
992	Japan	2007	Iwata kids health study	Community	Urban	10	10	439	394	439	394	
993	Japan	2007	National Health and Nutrition Survey	National	Both	5+	5+	832	904	590	596	
994	Japan	2008	Fukuroi kids health study	Community	Urban	13-14	13-14	381	346	381	346	
995	Japan	2008	Iwata kids health study	Community	Urban	10	10	406	417	406	417	

	Country	Study years	Survey/Study name/Citation	Level of representativeness	Rural, urban or both	Age range as in NCD-RisC database*		Sample size as in height analysis		Sample size as in BMI analysis		Note
						Male	Female	Male	Female	Male	Female	
996	Japan	2008	MEXT School Health Statistics	National	Both	5-17	5-17	326,407	326,966	326,401	326,957	
997	Japan	2008	National Health and Nutrition Survey	National	Both	5+	5+	769	785	506	489	
998	Japan	2009	Fukuroi kids health study	Community	Urban	13-14	13-14	388	357	388	357	
999	Japan	2009	MEXT School Health Statistics	National	Both	5-17	5-17	326,529	327,108	326,523	327,097	
1000	Japan	2009	National Health and Nutrition Survey	National	Both	5+	5+	810	872	564	575	
1001	Japan	2010	Fukuroi kids health study	Community	Urban	13-14	13-14	360	387	360	387	
1002	Japan	2010	MEXT School Health Statistics	National	Both	5-17	5-17	326,517	326,414	326,508	326,401	
1003	Japan	2010	National Health and Nutrition Survey	National	Both	5+	5+	707	721	487	459	
1004	Japan	2011	Fukuroi kids health study	Community	Urban	13-14	13-14	402	369	402	369	
1005	Japan	2011	MEXT School Health Statistics	National	Both	5-17	5-17	305,274	306,389	305,267	306,382	
1006	Japan	2011	National Health and Nutrition Survey	National	Both	5+	5+	644	723	437	457	
1007	Japan	2011	The Tokyo Health Service Association Database	Community	Urban	20+	20+	15,068	17,931			
1008	Japan	2012	Fukuroi kids health study	Community	Urban	13-14	13-14	432	353	432	353	
1009	Japan	2012	MEXT School Health Statistics	National	Both	5-17	5-17	326,527	326,577	326,524	326,572	
1010	Japan	2012	National Health and Nutrition Survey	National	Both	5+	5+	2,380	2,485	1,610	1,602	
1011	Japan	2013	Awaji Child Health Study	Community	Urban	10-14	10-14	198	203	198	203	
1012	Japan	2013	Fukuroi kids health study	Community	Urban	13-14	13-14	387	404	387	404	
1013	Japan	2013	MEXT School Health Statistics	National	Both	5-17	5-17	327,926	327,581	327,923	327,577	
1014	Japan	2013	National Health and Nutrition Survey	National	Both	5+	5+	721	695	460	438	
1015	Japan	2014	Awaji Child Health Study	Community	Urban	10-14	10-14	229	218	229	218	
1016	Japan	2014	MEXT School Health Statistics	National	Both	5-17	5-17	327,062	326,885	327,062	326,884	
1017	Japan	2014	National Health and Nutrition Survey	National	Both	5+	5+	642	678	462	441	
1018	Japan	2014-2015	Nagaoka Health Screening	Community	Both	20-89	20-89	294	473			
1019	Japan	2015	Awaji Child Health Study	Community	Urban	10-14	10-14	230	228	230	228	
1020	Japan	2015	MEXT School Health Statistics	National	Both	5-17	5-17	326,383	327,212	326,382	327,210	
1021	Japan	2015	National Health and Nutrition Survey	National	Both	5+	5+	612	632	437	418	
1022	Japan	2016	MEXT School Health Statistics	National	Both	5-17	5-17	334,735	334,447	334,731	334,444	
1023	Japan	2016	National Health and Nutrition Survey	National	Both	5+	5+	1,929	1,926	1,346	1,269	
1024	Japan	2017	MEXT School Health Statistics	National	Both	5-17	5-17	333,185	333,725	333,182	333,722	
1025	Japan	2017	National Health and Nutrition Survey	National	Both	5+	5+	498	516	331	348	
1026	Japan	2017	The Tokyo Health Service Association Database	Community	Urban	20+	20+	11,117	14,319			
1027	Japan	2018	MEXT School Health Statistics	National	Both	5-17	5-17	337,022	336,954	337,022	336,953	
1028	Jordan	1997	DHS	National	Both		20-49		1,674			
1029	Jordan	2002	DHS	National	Both		20-49		1,862			
1030	Jordan	2004	Behavioural Risk Factor Surveillance Survey	National	Rural	18+	18+	57	116	12	14	
1031	Jordan	2007	Behavioural Risk Factor Surveillance Survey	National	Both	18+	18+	76	85	12	17	
1032	Jordan	2007	DHS	National	Both		20-49		1,601			
1033	Jordan	2009	Metabolic abnormalities and vitamin D study	National	Both	7+	7+	638	1,295	488	618	
1034	Jordan	2009	DHS	National	Both		20-49		1,455			
1035	Jordan	2012	DHS	National	Both		20-49		2,096			
1036	Jordan	2016-2017	National Cardiovascular Diseases and Diabetes Study (NCDDS)	National	Both	18+	18+	154	579	33	120	
1037	Jordan	2017-2018	DHS	National	Both		15-49		2,096			
1038	Jordan	2019	STEPS	National	Both	18-69	18-69	574	790	145	134	
1039	Kazakhstan	1995	DHS	National	Both		15-49		1,745		638	
1040	Kazakhstan	1999	DHS	National	Both		15-49		1,027		341	
1041	Kazakhstan	2015	Almaty STEPS	Subnational	Both	18-69	18-69	67	172	9	9	
1042	Kazakhstan	2015	Shymkent STEPS	Subnational	Both	18-69	18-69	90	152	19	22	
1043	Kazakhstan	2015-2016	Childhood Obesity Surveillance Initiative 4	National	Both	8-10	8-10	2,756	2,683	2,755	2,683	1
1044	Kazakhstan	2015-2016	Aktobe STEPS	Subnational	Both	18-69	18-69	73	179	3	12	
1045	Kazakhstan	2019	A health status assessment of a population of Karaganda urban region	Community	Urban	18+	18+	67	92		6	
1046	Kenya	1993	DHS	National	Both		20-49		1,966			
1047	Kenya	1998	DHS	National	Both		20-49		1,995			
1048	Kenya	2003	DHS	National	Both		15-49		4,641		1,614	
1049	Kenya	2008-2009	DHS	National	Both		15-49		4,949		1,674	
1050	Kenya	2011-2013	International Study of Childhood Obesity, Lifestyle and the Environment (ISCOLE)	Community	Urban	9-11	9-11	262	301	262	301	
1051	Kenya	2014	DHS	National	Both		15-49		8,090		2,660	
1052	Kenya	2015	STEPS	National	Both	18-69	18-69	544	839	75	115	

	Country	Study years	Survey/Study name/Citation	Level of representativeness	Rural, urban or both	Age range as in NCD-RisC database*		Sample size as in height analysis		Sample size as in BMI analysis		Note
						Male	Female	Male	Female	Male	Female	
1053	Kenya	2018	Assessing the gaps in healthcare and determining the feasibility for the setup of a social enterprise - Viwandani Lown Community Health Center, Kenya	Community	Urban	19-73	19-73	97	109	54	58	
1054	Kiribati	2004	STEPS	National	Both	15-64	15-64	262	301	89	85	
1055	Kiribati	2011	Global School-based Student Health Survey	National	Both		13		156		156	
1056	Kiribati	2015-2016	STEPS	National	Both	18-69	18-69	177	211	15	11	
1057	Kuwait	1993-1994	al-Isa, Ann Nutr Metab 41(5):307-14, 1997	Community	Both	18+				210		
1058	Kuwait	2001	Kuwait nutrition surveillance system	National	Both	5-18	5-18			4,378	5,255	
1059	Kuwait	2002	Kuwait nutrition surveillance system	National	Both	5-18	5-18			5,444	5,146	
1060	Kuwait	2003	Kuwait nutrition surveillance system	National	Both	5-18	5-18			5,028	4,965	
1061	Kuwait	2004	Kuwait nutrition surveillance system	National	Both	5-18	5-18			4,900	4,792	
1062	Kuwait	2005	Kuwait nutrition surveillance system	National	Both	5-18	5-18			4,915	5,006	
1063	Kuwait	2006	STEPS	National	Both	20-64	20-64	222	309			
1064	Kuwait	2006	Kuwait nutrition surveillance system	National	Both	5-18	5-18			5,127	4,931	
1065	Kuwait	2007	Kuwait nutrition surveillance system	National	Both	5-18	5-18			5,330	5,317	
1066	Kuwait	2008	Kuwait nutrition surveillance system	National	Both	5-18	5-18			5,620	5,425	
1067	Kuwait	2008-2010	Gulf Cooperation Council World Health Survey	National	Both	18+	18+	360	528	42	57	
1068	Kuwait	2008-2009	National Nutrition Program for the State of Kuwait	National	Urban	5+	5+	414	402	314	294	
1069	Kuwait	2009	Kuwait nutrition surveillance system	National	Both	5-18	5-18			5,249	5,464	
1070	Kuwait	2011	Global School-based Student Health Survey	National	Both	13-17	13-17	1,265	1,274	1,265	1,274	
1071	Kuwait	2011-2014	Kuwait Diabetes Epidemiology Program	National	Urban	18-82	18-82	213	190	5	5	
1072	Kuwait	2014	STEPS	National	Both	18-69	18-69	506	692	62	47	
1073	Kuwait	2015	Global School-based Student Health Survey	National	Both	13-17	13-17	1,364	1,553	1,363	1,553	
1074	Kyrgyzstan	1997	DHS	National	Both		15-49		1,904		699	
1075	Kyrgyzstan	2012	DHS	National	Both		15-49		4,343		1,526	
1076	Kyrgyzstan	2013	STEPS	National	Both	25-64	25-64	134	221			
1077	Kyrgyzstan	2015-2016	Childhood Obesity Surveillance Initiative 4	National	Both	6-9	6-9	4,027	3,956	3,945	3,905	1
1078	Lao PDR	2006	Multiple Indicator Cluster Survey 3	National	Both		15-49		409		164	
1079	Lao PDR	2008	STEPS	Community	Both	25-64	25-64	173	330			
1080	Lao PDR	2013	STEPS	National	Both	18-64	18-64	228	430	38	53	
1081	Latvia	2008	Childhood Obesity Surveillance Initiative 1	National	Both	7-8	7-8	2,283	2,101	2,283	2,101	1
1082	Latvia	2008-2009	Cardiovascular risk factor study	National	Both	25-74	25-74	135	218			
1083	Latvia	2010	Childhood Obesity Surveillance Initiative 2	National	Both	7-8	7-8	2,093	2,053	2,093	2,053	1
1084	Latvia	2012	Childhood Obesity Surveillance Initiative 3	National	Both	6-7	6-7	1,804	1,677	1,804	1,677	1
1085	Latvia	2015-2016	Childhood Obesity Surveillance Initiative 4	National	Both	7-9	7-9	2,952	2,991	2,952	2,991	1
1086	Lebanon	1997	Obesity in Lebanon: National Survey	National	Both	5+	5+	468	624	370	450	
1087	Lebanon	2008-2009	STEPS	National	Both	5+	5+	851	886	485	464	
1088	Lebanon	2017	STEPS	National	Both	18-69	18-69	98	123	4	11	
1089	Lesotho	2004-2005	DHS	National	Both		15-49		2,022		803	
1090	Lesotho	2009-2010	DHS	National	Both	15-59	15-49	1,878	2,368	821	926	
1091	Lesotho	2012	STEPS	National	Both	25-64	25-64	122	275			
1092	Lesotho	2014	DHS	National	Both	15-59	15-49	1,583	1,996	678	760	
1093	Liberia	2006-2007	DHS	National	Both		15-49		3,925		1,233	
1094	Liberia	2011	STEPS	National	Both	25-64	25-64	227	353			
1095	Liberia	2013	DHS	National	Both	15-49	15-49	2,183	2,588	865	939	
1096	Libya	2009	STEPS	National	Both	25-64	25-64	256	273			
1097	Lithuania	1992-1993	Countrywide Integrated Noncommunicable Diseases Intervention Programme (CINDI) survey	Subnational	Rural	25-64	25-64	44	52			
1098	Lithuania	1998-1999	Countrywide Integrated Noncommunicable Diseases Intervention Programme (CINDI) survey	Subnational	Rural	25-64	25-64	48	61			
1099	Lithuania	2002	Pomerleau et al., Public Health Nutrition 3: 3-10, 2000	National	Both	24-70	24-70	69	189			
1100	Lithuania	2006-2007	Countrywide Integrated Noncommunicable Diseases Intervention Programme (CINDI) survey	Subnational	Rural	25-64	25-64	45	74			
1101	Lithuania	2008	Childhood Obesity Surveillance Initiative 1	National	Both	7-8	7-8	2,532	2,344	2,532	2,344	1
1102	Lithuania	2010	Childhood Obesity Surveillance Initiative 2	National	Both	7-9	7-9	3,306	3,414	3,306	3,414	1
1103	Lithuania	2010-2012	Prevalence and risk factors of high blood pressure in 12-15-year-old Lithuanian children and adolescents 2010-2012 (Study 1)	Community	Both	12-15	12-15	3,494	3,963	3,494	3,963	
1104	Lithuania	2013	Childhood Obesity Surveillance Initiative 3	National	Both	7-8	7-8	1,890	1,896	1,890	1,895	1
1105	Lithuania	2012-2013	Prevalence and risk factors of high blood pressure in 12-15-year-old Lithuanian children and adolescents 2012-2013 (Study 2)	Community	Both	12-15	12-15	962	985	962	985	
1106	Lithuania	2015-2016	Childhood Obesity Surveillance Initiative 4	National	Both	7-8	7-8	1,944	1,898	1,924	1,876	1

	Country	Study years	Survey/Study name/Citation	Level of representativeness	Rural, urban or both	Age range as in NCD-RisC database*		Sample size as in height analysis		Sample size as in BMI analysis		Note
						Male	Female	Male	Female	Male	Female	
1107	Luxembourg	2007-2009	Observation of cardiovascular risk factors in Luxembourg (ORISCAV-LUX)	National	Both	18-69	18-69	106	115	16	13	
1108	Madagascar	1997	DHS	National	Both		20-49		1,512			
1109	Madagascar	2003-2004	DHS	National	Both		15-49		4,211		1,369	
1110	Madagascar	2005	STEPS	Subnational	Both	25-64	25-64	581	584			
1111	Madagascar	2008-2009	DHS	National	Both		15-49		4,828		1,833	
1112	Malawi	1992	DHS	National	Both		20-49		1,331			
1113	Malawi	2000	DHS	National	Both		15-49		8,139		2,557	
1114	Malawi	2004	DHS	National	Both		15-49		6,999		2,000	
1115	Malawi	2009	STEPS	National	Both	25-64	25-64	426	955			
1116	Malawi	2010	DHS	National	Both		15-49		4,711		1,599	
1117	Malawi	2013-2017	NCD Survey Malawi Epidemiology and Intervention Research Unit	Community	Rural	18+	18+	2,257	2,993	500	550	
1118	Malawi	2013-2017	NCD Survey Malawi Epidemiology and Intervention Research Unit	Community	Urban	18+	18+	2,950	5,589	649	995	
1119	Malawi	2015-2016	DHS	National	Both		15-49		4,767		1,569	
1120	Malawi	2017	STEPS	National	Both	18-69	18-69	449	815	71	114	
1121	Malaysia	1996	National Health and Morbidity Survey (NHMS)	National	Both	18+	18+	4,451	5,419	730	927	
1122	Malaysia	2002-2003	Malaysian Adult Nutrition Survey	National	Both	18-59	18-59	501	418	73	62	
1123	Malaysia	2004	Rampal et al., Public Health 122(1):11-8, 2008	National	Both	15+	15+	2,230	2,980	941	1,211	
1124	Malaysia	2005	STEPS	National	Both	25-64	25-64	438	443			
1125	Malaysia	2006	National Health and Morbidity Survey (NHMS)	National	Both	5+	5+	12,090	12,391	8,767	8,540	
1126	Malaysia	2008	National Iodine Deficiency Disorder (IDD) Survey	National	Both	7-10	7-10	9,391	8,660	9,388	8,659	
1127	Malaysia	2008	Metabolic Syndrome Study in Malaysia	National	Rural	18+	18+	98	140	16	23	
1128	Malaysia	2008	Metabolic Syndrome Study in Malaysia	National	Urban	18+	18+	116	177	15	31	
1129	Malaysia	2011	National Health and Morbidity Survey (NHMS)	National	Both	5+	5+	2,342	2,253	389	364	
1130	Malaysia	2010-2011	SEANUTS	National	Both	5-12	5-12	1,306	1,352	1,306	1,352	
1131	Malaysia	2012	Global School-based Student Health Survey	National	Both	13	13-17	2,536	12,295	2,536	12,295	
1132	Malaysia	2012	Malaysian School-Based Health Survey	National	Both	9-17	9-17	20,264	19,661	20,254	19,652	
1133	Malaysia	2012-2013	Petaling District	Subnational	Urban	12-17	12-17	882	1,364	882	1,364	
1134	Malaysia	2013-2014	Batang Padang District	Subnational	Both	12-17	12-17	2,928	3,319	2,928	3,319	
1135	Malaysia	2014	Malaysian Adult Nutrition Survey	National	Both	18-59	18-59	391	377	79	44	
1136	Malaysia	2015	National Health and Morbidity Survey (NHMS)	National	Both	18+	18+	2,328	2,185	402	370	
1137	Maldives	2001	Multiple Indicator Cluster Survey	National	Both		15-50		578			
1138	Maldives	2004	STEPS	Subnational	Urban	25-64	25-64	292	311			
1139	Maldives	2009	Global School-based Student Health Survey	National	Both	13-17		807		806		
1140	Maldives	2009	DHS	National	Both		20-49		2,299			
1141	Maldives	2011	STEPS	Subnational	Urban	15-64	15-64	312	395	131	113	
1142	Maldives	2014	Global School-based Student Health Survey	National	Both	13-17		931		931		
1143	Maldives	2016-2017	DHS	National	Both	15-49	15-49	2,032	3,298	851	934	
1144	Mali	1995-1996	DHS	National	Both		20-49		2,331			
1145	Mali	1997	Programme Intégré de Développement de Bafoulabé	Community	Rural	15-45	15-45	242	422	94	159	
1146	Mali	1999-2000	Bafoulabe Iodine Study	Community	Rural		15-45		271		104	
1147	Mali	2001	DHS	National	Both		15-49		6,786		2,102	
1148	Mali	2006	DHS	National	Both		15-49		8,211		2,734	
1149	Mali	2007	STEPS	Subnational	Both	15-64	15-64	538	785	191	299	
1150	Mali	2012-2013	DHS	National	Both		15-49		2,948		839	
1151	Mali	2013	Santé Nutritionnelle à Assise Comunautaire dans la région de Kayes (SNACK)	Subnational	Rural		20-68		2,484			
1152	Mali	2018	DHS	National	Both		15-49		2,908		924	
1153	Malta	2008	Childhood Obesity Surveillance Initiative 1	National	Both	6	6	1,084	1,031	1,084	1,031	1
1154	Malta	2010	Childhood Obesity Surveillance Initiative 2	National	Both	6	6	1,151	1,170	1,151	1,170	1
1155	Malta	2013	Childhood Obesity Surveillance Initiative 3	National	Both	7-8	7-8	1,758	1,693	1,757	1,693	1
1156	Malta	2015-2016	Childhood Obesity Surveillance Initiative 4	National	Both	7-8	7-8	2,056	1,906	2,056	1,905	1
1157	Marshall Islands	2002	STEPS	National	Both	15-64	15-64	367	623	155	212	
1158	Mauritania	2000-2001	DHS	National	Both		15-49		1,432			
1159	Mauritania	2006	STEPS	Community	Urban	15-64	15-64	413	505	128	154	
1160	Mauritius	1998	Mauritius Noncommunicable Disease Survey	National	Both	25-74	25-74	99	174			
1161	Mauritius	2009	Mauritius Noncommunicable Disease Survey	National	Both	19-74	19-74	390	467			
1162	Mauritius	2011	Global School-based Student Health Survey	National	Both	13-17	13-17	859	1,043	859	1,043	
1163	Mauritius	2011	Global School-based Student Health Survey, Rodrigues	Subnational	Both	13-17	13-17	425	546	425	546	

	Country	Study years	Survey/Study name/Citation	Level of representativeness	Rural, urban or both	Age range as in NCD-RisC database*		Sample size as in height analysis		Sample size as in BMI analysis		Note
						Male	Female	Male	Female	Male	Female	
1164	Mauritius	2017	Global School-based Student Health Survey	National	Both	13-17	13-17	1,358	1,526	1,358	1,525	
1165	Mexico	1988-1989	Encuesta Nacional de Nutricion	National	Both		12-49		5,974		5,828	
1166	Mexico	1992-1993	Encuesta Nacional de Enfermedades Cronicas	National	Urban	20-69	20-69	2,235	3,102			
1167	Mexico	1998-1999	Encuesta Nacional de Nutricion	National	Both		12-49		10,194		5,096	
1168	Mexico	1999	National Survey on School Children	National	Both	5-10	5-10	4,910	5,040	4,898	5,031	
1169	Mexico	2000	Encuesta Nacional de Salud	National	Both	10+	10+	12,635	19,512	9,122	10,897	
1170	Mexico	2002	Encuesta Nacional Sobre Niveles de vida de los Hogares	National	Both	5+	5+	6,738	7,638	4,771	5,052	
1171	Mexico	2005	Encuesta Nacional Sobre Niveles de vida de los Hogares	National	Both	5+	5+	6,480	7,016	4,634	4,664	
1172	Mexico	2004-2005	Cardiovascular Risk Factors Multiple Evaluation in Latin America	Community	Urban	25-64	25-64	207	213			
1173	Mexico	2006	Encuesta Nacional de Salud y Nutrición	National	Both	5+	5+	17,464	19,604	14,622	15,038	
1174	Mexico	2006	PREVENIMSS National Coverage Surveys	National	Urban	20+	20+	1,944	2,265			
1175	Mexico	2010	PREVENIMSS National Coverage Surveys	National	Urban	20+	20+	1,381	990			
1176	Mexico	2009-2012	Encuesta Nacional Sobre Niveles de vida de los Hogares	National	Both	5+	5+	2,046	2,371	763	864	
1177	Mexico	2011-2012	Encuesta Nacional de Salud Y Nutricion	National	Both	5+	5+	18,678	19,978	15,319	15,145	
1178	Mexico	2016	Encuesta Nacional de Salud Y Nutricion	National	Both	5+	5+	3,331	4,108	2,815	2,976	
1179	Mexico	2018-2019	Encuesta Nacional de Salud Y Nutricion	National	Both	5+	5+	1,020	1,422			
1180	Micronesia (Federated States of)	2002	STEPS	Subnational	Both	25-64	25-64	86	149			
1181	Micronesia (Federated States of)	2006	STEPS	Subnational	Both	15-64	15-64	380	577	154	211	
1182	Micronesia (Federated States of)	2008	STEPS	Subnational	Both	25-64	25-64	119	205			
1183	Micronesia (Federated States of)	2009	STEPS, Yap	Subnational	Both	15-64	15-64	118	127	32	11	
1184	Micronesia (Federated States of)	2009	STEPS, Kosrae	Subnational	Both	15-64	15-64	31	64			
1185	Micronesia (Federated States of)	2016	STEPS	Subnational	Both	18-69	18-69	62	124	7	15	
1186	Moldova	2005	DHS	National	Both		15-49		3,445		1,364	
1187	Moldova	2013	STEPS	National	Both	18-69	18-69	314	464	60	67	
1188	Moldova	2013	Childhood Obesity Surveillance Initiative 3	National	Both	7-8	7-8	1,931	1,751	1,931	1,751	1
1189	Mongolia	2005	STEPS	National	Both	15-64	15-64	512	519	227	194	
1190	Mongolia	2009	STEPS	National	Both	15-64	15-64	661	932	180	228	
1191	Mongolia	2013	STEPS	National	Both	15-64	15-64	1,124	1,234	492	471	
1192	Mongolia	2013	Global School-based Student Health Survey	National	Both	13-17	13-17	2,095	2,360	2,095	2,360	
1193	Mongolia	2019	STEPS	National	Both	15-69	15-69	654	755	163	176	
1194	Montenegro	1982	Anthropometric Characteristics of Montenegrin Recruiters from '70 and 80's	National	Both	17-28				5,267		3
1195	Montenegro	1983	Anthropometric Characteristics of Montenegrin Recruiters from '70 and 80's	National	Both	17-28		52		9,296		3
1196	Montenegro	1984	Anthropometric Characteristics of Montenegrin Recruiters from '70 and 80's	National	Both	17-28		9,842		10,219		3
1197	Montenegro	1985	Anthropometric Characteristics of Montenegrin Recruiters from '70 and 80's	National	Both	17-28		9,437		9,437		
1198	Montenegro	1986	Anthropometric Characteristics of Montenegrin Recruiters from '70 and 80's	National	Both	17-28		9,590		9,590		
1199	Montenegro	1987	Anthropometric Characteristics of Montenegrin Recruiters from '70 and 80's	National	Both	17-28		9,934		9,934		
1200	Montenegro	1988	Anthropometric Characteristics of Montenegrin Recruiters from '70 and 80's	National	Both	17-28		86		86		
1201	Montenegro	2016	Anthropometric parameters as an indicator of obesity at adolescents in Montenegro	National	Both	14-18	14-18	678	771	678	771	
1202	Montenegro	2015-2016	Childhood Obesity Surveillance Initiative 4	National	Both	6-8	6-8	1,802	1,623	1,802	1,623	1
1203	Montenegro	2019	Body composition of high school students in Montenegro and its relationship with their eating habits	National	Both	18-20	18-20	504	497	501	495	
1204	Montenegro	2019	Initiative for monitoring obesity of children aged 11 to 12 in Montenegro	National	Both	11-12	11-12	666	619	666	619	
1205	Montenegro	2019	Initiative for monitoring obesity of children aged 5 to 6 in Montenegro and Slovenia	National	Both	5-6	5-6	231	214	231	214	
1206	Montenegro	2018-2019	Initiative for monitoring obesity of children aged 6 to 9 in Montenegro and Slovenia	National	Both	6-9	6-9	1,111	999	1,111	999	
1207	Morocco	1992	DHS	National	Both		20-49		1,290			
1208	Morocco	2003-2004	DHS	National	Both		15-49		8,904		3,230	
1209	Morocco	2017	STEPS	National	Both	18+	18+	335	651	63	89	
1210	Mozambique	1997	DHS	National	Both		20-49		1,915			
1211	Mozambique	2000	Growth of adolescents in Mozambique	Community	Urban	9-17	9-17	690	727	690	727	
1212	Mozambique	2003	DHS	National	Both		15-49		6,878		2,207	
1213	Mozambique	2005	STEPS	National	Both	25-64	25-64	267	417			
1214	Mozambique	2011	DHS	National	Both		15-49		7,796		2,708	
1215	Mozambique	2014-2015	STEPS	National	Both	15-64	15-64	487	711	173	226	
1216	Myanmar	2003-2004	STEPS	Subnational	Both	25-74	25-74	156	186			
1217	Myanmar	2009	STEPS	National	Both	15-64	15-64	720	1,025	176	262	
1218	Myanmar	2011	Underweight prevalence among young adults from rural areas, Salin Township, Magwe Region	Community	Rural	15-35	15-35	140	183	51	60	
1219	Myanmar	2014	STEPS	National	Both	25-64	25-64	314	492			

	Country	Study years	Survey/Study name/Citation	Level of representativeness	Rural, urban or both	Age range as in NCD-RisC database*		Sample size as in height analysis		Sample size as in BMI analysis		Note
						Male	Female	Male	Female	Male	Female	
1220	Myanmar	2013-2014	STEPS 2013-2014 Yangon	Subnational	Both	25-74	25-74	60	67			
1221	Myanmar	2015-2016	DHS	National	Both		15-49		5,510		1,777	
1222	Namibia	1992	DHS	National	Both		20-49		1,250			
1223	Namibia	2005	STEPS	National	Both	25-64	25-64	242	310			
1224	Namibia	2006-2007	DHS	National	Both		15-49		5,497		2,047	
1225	Namibia	2009	Okambilimbili Survey	Community	Urban	5+	5+	490	550	265	295	
1226	Namibia	2013	DHS	National	Both		15-64		2,501		841	
1227	Nauru	1975-1976	Trends in the prevalence and incidence of non-insulin-dependent diabetes mellitus and impaired glucose tolerance	Subnational	Both	10+	10+	4	4			
1228	Nauru	1994	Trends in the prevalence and incidence of non-insulin-dependent diabetes mellitus and impaired glucose tolerance	National	Both	25+	25+	133	166			
1229	Nauru	2004	STEPS	National	Both	15-64	15-64	404	430	122	119	
1230	Nauru	2006	STEPS	National	Both	16-65	16-65	87	87	24	18	
1231	Nauru	2011	Global School-based Student Health Survey	National	Both		13-17		259		259	
1232	Nauru	2015	STEPS	National	Rural	18-69	18-69	200	188	30	22	
1233	Nepal	1996	DHS	National	Both		20-49		2,216			
1234	Nepal	2001	DHS	National	Both		20-49		3,290			
1235	Nepal	2005	STEPS	Subnational	Both	15-64	15-64	1,066	1,311	358	346	
1236	Nepal	2006	DHS	National	Both		15-49		6,224		2,283	
1237	Nepal	2007-2008	STEPS	National	Both	15-64	15-64	687	827	259	245	
1238	Nepal	2006-2011	Early detection and management of Kidney disease, Hypertension, Diabetes and Cardiovascular disease (KHDC Nepal), Tarahara	Community	Rural	18+	18+	280	661	48	97	
1239	Nepal	2006-2011	Early detection and management of Kidney disease, Hypertension, Diabetes and Cardiovascular disease (KHDC Nepal), Damak	Community	Urban	18+	18+	256	387	37	43	
1240	Nepal	2006-2011	Early detection and management of Kidney disease, Hypertension, Diabetes and Cardiovascular disease (KHDC Nepal), Dharan	Community	Urban	18+	18+	1,418	2,083	351	352	
1241	Nepal	2011	DHS	National	Both		15-49		3,510		1,288	
1242	Nepal	2012-2013	STEPS	National	Both	15-69	15-69	286	682	81	117	
1243	Nepal	2015	Community based intervention for prevention and control of non-communicable diseases risk factors (CIPCON) baseline survey, Dhankuta	Subnational	Rural	15-69	15-69	78	159	20	24	
1244	Nepal	2015	Community based intervention for prevention and control of non-communicable diseases risk factors (CIPCON) baseline survey, Ilam	Subnational	Rural	15-69	15-69	96	165	18	26	
1245	Nepal	2016	DHS	National	Both	15-49	15-49	2,109	3,540	961	1,251	
1246	Nepal	2016-2018	The Population Based Prevalence of Selected Non-Communicable Diseases In Nepal	National	Both	20+	20+	561	1,264			
1247	Netherlands	1993-1997	EPIC Bilthoven	Community	Urban	20-59	20-59	1,573	2,232			
1248	Netherlands	1998-2001	Regenboog Project	National	Both	12-89	12-89	411	484	181	206	
1249	Netherlands	2004-2006	Prevention and Incidence of Asthma and Mite Allergy (PIAMA)	National	Both	7-9	7-9	1,110	1,104	1,110	1,104	
1250	Netherlands	2008-2011	Prevention and Incidence of Asthma and Mite Allergy (PIAMA)	National	Both	12-13	12-13	739	771	739	769	
1251	Netherlands	2008-2011	Amsterdam Born Children and their Development Study (ABCD)	Community	Urban	5-7	5-7	1,544	1,529	1,541	1,528	
1252	Netherlands	2011-2013	GECKO Drenthe Onderzoek	Subnational	Rural	5-7	5-7	1,139	1,133	1,139	1,133	
1253	Netherlands	2012-2014	Prevention and Incidence of Asthma and Mite Allergy (PIAMA)	National	Both	15-17	15-17	386	416	386	414	
1254	Netherlands	2011-2015	Healthy Life in an Urban Setting (HELIUS)	Community	Urban	18-71	18-71	289	471	30	50	
1255	Netherlands	2012-2016	Amsterdam Born Children and their Development Study (ABCD)	Community	Urban	9-12	9-12	1,407	1,122	1,121	1,135	
1256	Netherlands	2016-2018	GECKO Drenthe Onderzoek	Subnational	Rural	8-12	8-12	1,085	1,117	1,083	1,114	
1257	New Zealand	1989	The Life in New Zealand Survey	National	Both	15+	15+	97	104	97	104	
1258	New Zealand	1990-1993	Williams, N Z Med J 113(1114):308-11, 2000	Community	Both	18-21	18-21			450	417	
1259	New Zealand	1996-1997	National Nutrition Survey	National	Both	15+	15+	383	587	128	156	
1260	New Zealand	2002	National Children's Nutrition Survey	National	Both	5-14	5-14	1,558	1,482	1,556	1,479	
1261	New Zealand	2002-2003	New Zealand Health Survey	National	Both	15+	15+	935	1,381	293	325	
1262	New Zealand	2006-2007	New Zealand Health Survey	National	Both	5+	5+	2,685	2,844	2,014	1,901	
1263	New Zealand	2008-2009	New Zealand Adult Nutrition Survey	National	Both	15+	15+	573	709	339	404	
1264	New Zealand	2011-2012	New Zealand Health Survey	National	Both	5+	5+	2,154	2,190	1,511	1,406	
1265	New Zealand	2012-2013	New Zealand Health Survey	National	Both	5+	5+	2,301	2,426	1,573	1,452	
1266	New Zealand	2013-2014	New Zealand Health Survey	National	Both	5+	5+	2,491	2,661	1,681	1,644	
1267	New Zealand	2014-2015	New Zealand Health Survey	National	Both	5+	5+	2,580	2,768	1,779	1,745	
1268	New Zealand	2015-2016	New Zealand Health Survey	National	Both	5+	5+	2,702	2,652	1,870	1,653	
1269	Nicaragua	1997-1998	DHS	National	Both		15-49		7,617		2,966	
1270	Nicaragua	2001	DHS	National	Both		15-49		7,292		2,838	

	Country	Study years	Survey/Study name/Citation	Level of representativeness	Rural, urban or both	Age range as in NCD-RisC database*		Sample size as in height analysis		Sample size as in BMI analysis		Note
						Male	Female	Male	Female	Male	Female	
1271	Nicaragua	2003-2004	CAMDI	Community	Urban	20+	20+	235	204			
1272	Nicaragua	2006-2007	Encuesta Nicaraguense de Demografia y Salud	National	Both		15-49		7,317		2,383	
1273	Nicaragua	2011-2012	Encuesta Nicaraguense de Demografia y Salud	National	Both		15-49		7,451		2,562	
1274	Niger	1992	DHS	National	Both		20-49		1,997			
1275	Niger	1998	DHS	National	Both		20-49		1,856			
1276	Niger	2006	DHS	National	Both		15-49		2,636		850	
1277	Niger	2007	STEPS	National	Both	15-64	15-64	390	539	116	135	
1278	Niger	2012	DHS	National	Both		15-49		2,794		775	
1279	Nigeria	1999	DHS	National	Both		20-49		1,295			
1280	Nigeria	2003	DHS	National	Both		15-49		4,456		1,576	
1281	Nigeria	2006	Senbanjo et al., West Afr J Med 30(6):425-31, 2011	Community	Urban	5-19	5-19	296	274	296	274	
1282	Nigeria	2008	DHS	National	Both		15-49		18,426		5,850	
1283	Nigeria	2009	Community Health Plan - Kwara Central Survey	Community	Rural	5+	5+	1,334	1,301	1,096	1,004	
1284	Nigeria	2011	Community Health Plan - Kwara Central Survey	Community	Rural	5+	5+	504	491	436	386	
1285	Nigeria	2013	DHS	National	Both		15-49		21,338		7,206	
1286	Nigeria	2013	Community Health Plan - Kwara Central Survey	Community	Rural	5+	5+	453	416	394	355	
1287	Nigeria	2018	Hypertension Prevalence, Awareness, Treatment and Control in Rural Area, Nigeria	Community	Rural	18+	18+	72	64	11	20	
1288	Niue	2010	Global School-based Student Health Survey	National	Both	13-17	13-17	63	38	63	38	
1289	Niue	2011	STEPS	National	Both	15+	15+	117	130	55	36	
1290	North Macedonia	1999	Multiple Indicator Cluster Survey	National	Both		15-45		733		46	
1291	North Macedonia	2009	Annual assessment of nutritional status of school children aged	National	Both	7	7	1,088	983	1,087	982	
1292	North Macedonia	2010	Childhood Obesity Surveillance Initiative 2	National	Both	7	7	1,427	1,311	1,427	1,311	1
1293	North Macedonia	2013	Childhood Obesity Surveillance Initiative 3	National	Both	6-7	6-7	1,655	1,511	1,655	1,511	1
1294	North Macedonia	2015-2016	Childhood Obesity Surveillance Initiative 4	National	Both	7-8	7-8	1,809	1,727	1,809	1,727	1
1295	Norway	1994-1995	The Tromsø Study: Tromsø 4	Community	Both	25+	25+	1,506	1,782			
1296	Norway	1995-1997	Young-HUNT1 Study	Subnational	Rural	12-21	12-21	4,196	4,251	4,194	4,241	
1297	Norway	1995-1997	HUNT2 study	Subnational	Rural	20+	20+	3,963	4,764			
1298	Norway	1999-2000	European Youth Heart Study	Community	Urban	9-15	9-15	365	369	364	369	
1299	Norway	2000-2001	Young-HUNT2 Study	Subnational	Rural	16-21	16-21	764	901	755	884	
1300	Norway	2000-2003	The Oslo cohort (HUBRO), the Oppland and Hedmark cohort (OPPHED), and the Troms and Finnmark cohort (TROFINN) of COHORT NORWAY	Subnational	Both	30-76	30-76	3,031	3,967			
1301	Norway	2005-2006	Physical Activity among Norwegian Children and Adolescents	National	Both	8-16	8-16	1,186	1,058	1,186	1,055	
1302	Norway	2006-2008	Young-HUNT3 Study	Subnational	Rural	12-21	12-21	3,809	3,797	3,788	3,777	
1303	Norway	2006-2008	HUNT3 Study	Subnational	Rural	20+	20+	1,859	2,638			
1304	Norway	2008	Childhood Obesity Surveillance Initiative 1	National	Both	8	8	1,435	1,399	1,435	1,399	1
1305	Norway	2010	Childhood Obesity Surveillance Initiative 2	National	Both	8	8	1,335	1,286	1,335	1,286	1
1306	Norway	2012	Childhood Obesity Surveillance Initiative 3	National	Both	8	8	1,492	1,381	1,492	1,381	1
1307	Norway	2015-2016	Childhood Obesity Surveillance Initiative 4	National	Both	7-8	7-8	1,690	1,645	1,690	1,645	1
1308	Oman	2008	Gulf Cooperation Council World Health Survey	National	Both	18+	18+	882	850	134	129	
1309	Oman	2010	Global School-based Student Health Survey	National	Both	13-17	13-17	251	300	251	300	
1310	Oman	2015	Global School-based Student Health Survey	National	Both	13-17	13-17	1,330	1,551	1,330	1,551	
1311	Oman	2017	STEPS	National	Both	15+	15+	886	813	165	169	
1312	Pakistan	1990-1994	National Health Survey of Pakistan 1990-1994	National	Both	5+	5+	4,779	4,834	3,636	3,391	
1313	Pakistan	1990-1994	Metroville Health Study (MHS)	Community	Urban	18+	18+			39	31	
1314	Pakistan	2005	STEPS	National	Both	25-65	25-65	170	252			
1315	Pakistan	2011	National Nutrition Survey	National	Both	5-49	5-49	17,373	34,697	14,906	20,335	
1316	Pakistan	2012-2013	DHS	National	Both		20-49		1,645			
1317	Pakistan	2014	STEPS	Subnational	Both	18-69	18-69	957	1,402	191	264	
1318	Pakistan	2016-2017	National Diabetes Survey of Pakistan	National	Both	20+	20+	654	753			
1319	Pakistan	2017-2018	DHS	National	Both		15-49		1,978			
1320	Palau	2011-2013	STEPS	National	Both	25-64	25-64	88	90			
1321	Panama	2003	Encuesta de Niveles de Vida	National	Both	5+	5+	5,983	5,942	4,154	3,980	
1322	Panama	2010-2011	Prevalencia de factores de riesgo asociados a enfermedad cardiovascular 2010-2011	Subnational	Both	18+	18+	183	549	29	98	
1323	Panama	2018	Global School-based Student Health Survey	National	Both	13-17	13-17	1,121	1,385	1,121	1,384	
1324	Papua New Guinea	2007	STEPS	National	Both	15-64	15-64	555	638	194	211	
1325	Paraguay	2011	Primera Encuesta Nacional de Factores de Riesgo de Enfermedades No Transmisibles en Poblacion General	National	Both	15-75	15-75	351	529	119	125	

	Country	Study years	Survey/Study name/Citation	Level of representativeness	Rural, urban or both	Age range as in NCD-RisC database*		Sample size as in height analysis		Sample size as in BMI analysis		Note
						Male	Female	Male	Female	Male	Female	
1326	Peru	1991-1992	DHS	National	Both		15-49		2,860			
1327	Peru	1996	DHS	National	Both		20-49		5,656			
1328	Peru	2000	DHS	National	Both		15-49		14,126		5,340	
1329	Peru	2003	Factores de Riesgo de Enfermedades No Transmisibles	Community	Urban	16+	16+	102	105	23	12	
1330	Peru	2004	Factores de Riesgo de Enfermedades No Transmisibles	Community	Urban	15+	15+	71	132	26	28	
1331	Peru	2004-2006	DHS	National	Both		15-49		3,034		1,175	
1332	Peru	2005	Factores de Riesgo de Enfermedades No Transmisibles	Community	Urban	15+	15+	78	160	24	42	
1333	Peru	2004-2005	Encuesta Nacional de Indicadores Nutricionales, Bioquímicos, Socioeconómicos y Culturales Relacionados con las Enfermedades Crónicas Degenerativas	National	Both	20+	20+	474	549			
1334	Peru	2004-2005	Cardiovascular Risk Factors Multiple Evaluation in Latin America	Community	Urban	25-64	25-64	219	239			
1335	Peru	2006	Factores de Riesgo de Enfermedades No Transmisibles	Community	Urban	15+	15+	256	320	87	98	
1336	Peru	2007-2008	DHS	National	Both		15-49		10,850		3,940	
1337	Peru	2007-2008	Monitoreo de Indicadores Nutricionales en la ENAHO 2007-2008	National	Both	5+	5+	8,344	8,670	5,965	5,807	
1338	Peru	2007-2010	Monitoreo Nacional de Indicadores Nutricionales	National	Both		12-49		2,421		734	
1339	Peru	2009	DHS	National	Both		15-49		11,951		4,409	
1340	Peru	2010	DHS	National	Both		15-49		11,416		4,358	
1341	Peru	2009-2011	Monitoreo de Indicadores Nutricionales en la ENAHO 2009-2010	National	Both	5+	5+	14,729	15,477	11,184	10,943	
1342	Peru	2010	Global School-based Student Health Survey	National	Both	13-17	13-17	1,267	1,306	1,267	1,306	
1343	Peru	2011	DHS	National	Both		15-49		11,180		4,171	
1344	Peru	2012	DHS	National	Both		15-49		12,002		4,551	
1345	Peru	2011-2012	Monitoreo de Indicadores Nutricionales en la ENAHO 2011	National	Both	5+	5+	3,730	4,006	2,816	2,820	
1346	Peru	2013	DHS	National	Both	15+	15+	850	11,280	309	4,093	
1347	Peru	2014	DHS	National	Both	15+	15+	3,685	11,558	1,386	4,176	
1348	Peru	2014	Launching a salt substitute to reduce blood pressure at the population level: a cluster randomized stepped wedge trial in Peru	Subnational	Both	18+	18+	286	334	51	54	
1349	Peru	2015	DHS	National	Both	15+	15+	4,806	17,724	1,496	5,190	
1350	Peru	2016	DHS	National	Both	15+	15+	4,400	16,141	1,418	4,674	
1351	Peru	2017	DHS	National	Both	15+	15+	4,469	16,855	1,354	4,743	
1352	Peru	2018	DHS	National	Both	15+	15+	4,241	17,282	1,355	4,807	
1353	Peru	2019	DHS	National	Both	15+	15+	4,132	16,403	1,306	4,732	
1354	Philippines	1983-1984	Cebu Longitudinal Health and Nutrition Survey Baseline 2-Month Follow-up	Community	Both		15-50		83			
1355	Philippines	1983-1984	Cebu Longitudinal Health and Nutrition Survey Baseline 4-Month Follow-up	Community	Both		15-50		148			
1356	Philippines	1983-1984	Cebu Longitudinal Health and Nutrition Survey Baseline 6-Month Follow-up	Community	Both		15-50		124			
1357	Philippines	1984-1985	Cebu Longitudinal Health and Nutrition Survey Baseline 8-Month Follow-up	Community	Both		15-50		102			
1358	Philippines	1984-1985	Cebu Longitudinal Health and Nutrition Survey Baseline 10-Month Follow-up	Community	Both		15-50		96			
1359	Philippines	1984-1985	Cebu Longitudinal Health and Nutrition Survey Baseline 12-Month Follow-up	Community	Both		15-50		80			
1360	Philippines	1984-1985	Cebu Longitudinal Health and Nutrition Survey Baseline 14-Month Follow-up	Community	Both		15-50		72			
1361	Philippines	1985-1986	Cebu Longitudinal Health and Nutrition Survey Baseline 20-Month Follow-up	Community	Both		15-50		96		69	
1362	Philippines	1985-1986	Cebu Longitudinal Health and Nutrition Survey Baseline 22-Month Follow-up	Community	Both		15-50		90		65	
1363	Philippines	1985-1986	Cebu Longitudinal Health and Nutrition Survey Baseline 24-Month Follow-up	Community	Both		15-50		81		57	
1364	Philippines	1984-1985	Cebu Longitudinal Health and Nutrition Survey Baseline 16-Month Follow-up	Community	Both		15-50		126		92	
1365	Philippines	1984-1985	Cebu Longitudinal Health and Nutrition Survey Baseline 18-Month Follow-up	Community	Both		15-50		108		78	
1366	Philippines	1991-1992	Cebu Longitudinal Health and Nutrition Survey 1991 Child Follow-up	Community	Both	8	8	1,202	1,076	1,202	1,076	
1367	Philippines	1993	National Safe Motherhood Survey	National	Both		15-49		4,086			
1368	Philippines	1994-1995	Cebu Longitudinal Health and Nutrition Survey 1994-1995 Mother Follow-up	Community	Both		15-59		633		244	
1369	Philippines	1998-1999	Cebu Longitudinal Health and Nutrition Survey 1998-1999 Child Follow-up	Community	Both	14-16	14-16	1,102	999	1,102	999	
1370	Philippines	1998-1999	Cebu Longitudinal Health and Nutrition Survey 1998-1999 Mother Follow-up	Community	Both		15-59		3			
1371	Philippines	2002	Cebu Longitudinal Health and Nutrition Survey 2002 Child Follow-up	Community	Both	17-19	17-19	1,087	961	1,087	907	
1372	Philippines	2003	Global School-based Student Health Survey	National	Both		13		291		291	
1373	Philippines	2003	6th National Nutrition Survey	National	Both	5+	5+	6,489	6,725	4,600	4,441	
1374	Philippines	2005	Cebu Longitudinal Health and Nutrition Survey 2005 Child Follow-up	Community	Both	20-22	20-22	1,006	903			
1375	Philippines	2007	Global School-based Student Health Survey	National	Both		13		254		254	
1376	Philippines	2007	Cebu Longitudinal Health and Nutrition Survey 2007 Child Follow-up	Community	Both	23-24	23-24	937	816			
1377	Philippines	2008	7th National Nutrition Survey	National	Both	5+	5+	37,502	33,529	28,914	26,630	
1378	Philippines	2009	Cebu Longitudinal Health and Nutrition Survey 2009 Child Follow-up	Community	Both	24-26	24-26	864	796			
1379	Philippines	2011	Global School-based Student Health Survey	National	Both		13		540		540	
1380	Philippines	2011	2011 Updating of Nutritional Status of Filipino Children	National	Both	5+	5+	37,670	35,989	29,544	26,983	

	Country	Study years	Survey/Study name/Citation	Level of representativeness	Rural, urban or both	Age range as in NCD-RisC database*		Sample size as in height analysis		Sample size as in BMI analysis		Note
						Male	Female	Male	Female	Male	Female	
1381	Philippines	2013-2014	8th National Nutrition Survey	National	Both	5+	5+	31,628	30,662	24,303	22,865	
1382	Philippines	2015	Global School-based Student Health Survey	National	Both		13-17		3,730		3,730	
1383	Philippines	2015	2015 Updating of Nutritional Status of Filipino Children and Other Population Groups	National	Both	5+	5+	38,158	37,206	28,876	27,178	
1384	Poland	1986	Poland Conscripts 10% Sample Cohort	National	Both	18-19		29,464		29,421		
1385	Poland	1988	Fourth National Survey	Subnational	Both	7-19	7-19	12,350	10,372	12,345	10,371	
1386	Poland	1995	Poland Conscripts 10% Sample Cohort	National	Both	18-19		31,062		31,043		
1387	Poland	1995-1996	Polish Program CINDI (CINDI Lodz 1995)	Community	Urban	17-64	17-64	300	387	79	111	
1388	Poland	1997	Wroclaw survey in adolescents	Community	Both	12-17	12-17	2,198	2,141	2,198	2,141	
1389	Poland	2000	The health status, risk factors of chronic diseases and health behaviors of residents of Torun (CINDI Torun 2000)	Community	Urban	16-83	16-83	256	264	111	85	
1390	Poland	2000	Binkowska-Bury et al., Neuro Endocrinol Lett 34(8):814-20, 2013	Subnational	Both	19-20		3,004		3,003		
1391	Poland	2001	Poland Conscripts 10% Sample Cohort	National	Both	18-19		31,255		31,213		
1392	Poland	2000-2001	Household Food Consumption and Anthropometric Survey	National	Both	5+	5+	799	829	555	572	
1393	Poland	2001	Binkowska-Bury et al., Neuro Endocrinol Lett 34(8):814-20, 2013	Subnational	Both	19-20		3,425		3,420		
1394	Poland	2001-2002	The health status, risk factors of chronic diseases and health behaviors of residents of Lodz (CINDI Lodz 2001)	Community	Urban	18-64	18-64	261	258	61	39	
1395	Poland	2002	NATPOL	National	Both	18+	18+	201	222	32	34	
1396	Poland	2002	Binkowska-Bury et al., Neuro Endocrinol Lett 34(8):814-20, 2013	Subnational	Both	19-20		3,547		3,544		
1397	Poland	2003	Binkowska-Bury et al., Neuro Endocrinol Lett 34(8):814-20, 2013	Subnational	Both	19-20		3,637		3,633		
1398	Poland	2004	Binkowska-Bury et al., Neuro Endocrinol Lett 34(8):814-20, 2013	Subnational	Both	19-20		3,540		3,538		
1399	Poland	2003-2005	National Multicenter Health Survey in Poland. Project WOBASZ	National	Both	20-74	20-74	1,204	1,379			
1400	Poland	2005	Binkowska-Bury et al., Neuro Endocrinol Lett 34(8):814-20, 2013	Subnational	Both	19-20		3,308		3,308		
1401	Poland	2003-2006	Mogielica Human Ecology Study Site	Community	Rural	22+	21+	8	60			
1402	Poland	2006	The health, risk factors for chronic diseases, attitudes and behaviors of health residents of Torun (CINDI Torun 2006)	Community	Urban	15-65	15-65	194	257	70	74	
1403	Poland	2006	Binkowska-Bury et al., Neuro Endocrinol Lett 34(8):814-20, 2013	Subnational	Both	19-20		3,702		3,701		
1404	Poland	2007	Binkowska-Bury et al., Neuro Endocrinol Lett 34(8):814-20, 2013	Subnational	Both	19-20		3,612		3,612		
1405	Poland	2007-2009	Elaboration of the reference range of arterial blood pressure for the population of children and adolescents in Poland – PL0080 OLAF	National	Both	6-18	6-18	8,386	9,172	8,383	9,162	
1406	Poland	2008	Binkowska-Bury et al., Neuro Endocrinol Lett 34(8):814-20, 2013	Subnational	Both	19-20		3,435		3,435		
1407	Poland	2009	Binkowska-Bury et al., Neuro Endocrinol Lett 34(8):814-20, 2013	Subnational	Both	19-20		3,407		3,405		
1408	Poland	2007-2010	Mogielica Human Ecology Study Site	Community	Rural	30+	21+		50			
1409	Poland	2010	Binkowska-Bury et al., Neuro Endocrinol Lett 34(8):814-20, 2013	Subnational	Both	19-20		3,317		3,317		
1410	Poland	2011	NATPOL	National	Both	18-79	18-79	239	266	39	29	
1411	Poland	2010-2012	Blood pressure references for Polish preschool children - the OLA study	National	Both	5-6	5-6	926	905	926	904	
1412	Poland	2012-2013	Fifth National Survey	Subnational	Both	6-19	6-19	3,100	2,687	3,091	2,686	
1413	Poland	2011-2014	Mogielica Human Ecology Study Site	Community	Rural	30+	20+		8			
1414	Poland	2013-2014	National Multicenter Health Survey in Poland. Project WOBASZ II	National	Both	20+	20+	392	408			
1415	Poland	2014-2017	The impact of physical activity and selected perinatal risk factors on the occurrence of overweight and obesity and hypertension in children	Subnational	Both	5-15	5-15	516	454	516	454	
1416	Poland	2015-2016	Childhood Obesity Surveillance Initiative 4	National	Both	8	8	1,675	1,667	1,675	1,664	1
1417	Poland	2015-2016	LIPIDOGRAM2015 & LIPIDOGEN2015 Study - National epidemiological study of lipid disorders and selected risk factors of cardiovascular disease in primary health care in Poland	National	Both	18+	18+	214	356	24	21	
1418	Poland	2016-2017	Erasmus plus KA2, Healthyland	Community	Urban	6	6	25	25	25	25	
1419	Poland	2017-2018	Erasmus plus KA2, Healthyland	Community	Urban	5-6	5-6	24	26	24	26	
1420	Poland	2018	Mogielica Human Ecology Study Site	Community	Rural	26+	20+	4	8			
1421	Portugal	1985	Body Mass Index of Portuguese Conscripts	National	Both	18-20		28,716		28,716		
1422	Portugal	1986	Body Mass Index of Portuguese Conscripts	National	Both	18-20		70,504		25,422		
1423	Portugal	1987	Body Mass Index of Portuguese Conscripts	National	Both	18-20		68,079		22,797		
1424	Portugal	1988	Body Mass Index of Portuguese Conscripts	National	Both	18-20		67,576		22,917		
1425	Portugal	1989	Body Mass Index of Portuguese Conscripts	National	Both	18-20		68,827		24,459		
1426	Portugal	1990	Body Mass Index of Portuguese Conscripts	National	Both	18-20		44,360		1,054		
1427	Portugal	1991	Body Mass Index of Portuguese Conscripts	National	Both	18-20		22,412		16,803		
1428	Portugal	1992	Body Mass Index of Portuguese Conscripts	National	Both	18-20		63,455		34,576		
1429	Portugal	1993	Body Mass Index of Portuguese Conscripts	National	Both	18-20		71,524		57,668		
1430	Portugal	1994	Body Mass Index of Portuguese Conscripts	National	Both	18-20		66,013		50,559		
1431	Portugal	1995	Body Mass Index of Portuguese Conscripts	National	Both	18-20		83,173		62,636		
1432	Portugal	1996	Body Mass Index of Portuguese Conscripts	National	Both	18-21		124,049		87,926		

	Country	Study years	Survey/Study name/Citation	Level of representativeness	Rural, urban or both	Age range as in NCD-RisC database*		Sample size as in height analysis		Sample size as in BMI analysis		Note
						Male	Female	Male	Female	Male	Female	
1433	Portugal	1997	Body Mass Index of Portuguese Conscripts	National	Both	18-21		61,236		50,808		
1434	Portugal	1998	Body Mass Index of Portuguese Conscripts	National	Both	18-21		41,037		30,619		
1435	Portugal	1999	Body Mass Index of Portuguese Conscripts	National	Both	18-21		54,187		42,736		
1436	Portugal	1998-2000	European Youth Heart Study	Community	Both	9-16	9-16	554	535	554	535	
1437	Portugal	2000	Body Mass Index of Portuguese Conscripts	National	Both	18-21		53,326		41,849		
1438	Portugal	1999-2003	EPIPorto Study	Community	Urban	18+	18+	86	126	5	10	
1439	Portugal	2004	Growth of adolescents in Coimbra	Community	Both	9-16	9-16	265	409	265	408	
1440	Portugal	2004	Growth of adolescents in Gouveia	Community	Rural	10-19	10-19	238	246	238	246	
1441	Portugal	2003-2004	EPITeen - Epidemiological Health Investigation of Teenagers in Porto	Community	Urban	13-14	13-14	983	1,049	981	1,048	
1442	Portugal	2003-2005	Estudo de Prevalência da Obesidade e Consumos Alimentares em Portugal	National	Both	18-64	18-64	1,052	1,606	202	351	
1443	Portugal	2007	Growth of adolescents in Tondela	Community	Rural	6-19	6-19	315	312	314	312	
1444	Portugal	2008	Childhood Obesity Surveillance Initiative 1	National	Both	6-8	6-8	1,801	1,792	1,801	1,792	1
1445	Portugal	2007-2008	European Youth Heart Study	Community	Both	8-17	8-17	315	311	315	311	
1446	Portugal	2007-2010	Promoção do Exercício e Saúde no Sedentarismo e Obesidade da Adolescência (PESSOA Program)	Community	Urban	9-16	9-16	1,931	1,813	1,931	1,813	
1447	Portugal	2007-2008	Primary schools health promotion	Community	Urban	6-12	6-12	224	238	224	238	
1448	Portugal	2008	Azorean Physical Activity and Health Study II	Subnational	Both	15-18	15-18	608	893	608	893	
1449	Portugal	2007-2008	EPITeen - Epidemiological Health Investigation of Teenagers in Porto	Community	Urban	16-17	16-17	1,192	1,261	1,186	1,251	
1450	Portugal	2007-2009	Portuguese National Survey of Physical Activity and Physical Fitness	National	Both	9+	9+	11,681	12,385	10,706	11,408	
1451	Portugal	2007-2010	The Midland Adolescent Lifestyle Study	Subnational	Both	12-16	12-16	196	235	196	235	
1452	Portugal	2009-2010	Portuguese Prevalence Study of Obesity in Childhood	National	Both	5-10	5-10	6,806	7,099	6,804	7,099	
1453	Portugal	2009	Bracara Study	Community	Urban	8-14	8-14	398	336	398	336	
1454	Portugal	2010	Childhood Obesity Surveillance Initiative 2	National	Both	6-8	6-8	1,830	1,826	1,830	1,826	1
1455	Portugal	2010-2012	Promoção do Exercício e Saúde no Sedentarismo e Obesidade da Adolescência (PESSOA Program)	Community	Urban	9-14	9-14	287	275	287	275	
1456	Portugal	2008-2013	Preschool Physical Activity, Body Composition and Lifestyle Study (PRESTYLE)	Community	Urban	5-6	5-6	703	635	651	607	
1457	Portugal	2011-2012	The association of childhood obesity with asthma and rhinitis symptoms in 6-8years old children living in the Coimbra district, Portugal: the role of environmental, family and socioeconomic factors	Community	Both	5-9	5-9	480	509	480	509	
1458	Portugal	2011-2013	International Study of Childhood Obesity, Lifestyle and the Environment (ISCOLE)	Community	Urban	9-11	9-11	358	419	358	419	
1459	Portugal	2011-2013	Environmental Support for Leisure and Active Transport	Subnational	Urban	10-15	10-15	294	340	294	340	
1460	Portugal	2011-2013	EPITeen - Epidemiological Health Investigation of Teenagers in Porto	Community	Urban	20-23	20-23	855	905			
1461	Portugal	2013	Childhood Obesity Surveillance Initiative 3	National	Both	6-8	6-8	2,951	2,982	2,951	2,982	1
1462	Portugal	2013	Childhood obesity in Lousao	Community	Rural	5-14	5-14	444	418	444	418	
1463	Portugal	2011-2014	Longitudinal Analysis of Biomarkers and Environmental Determinants of Physical activity (LABMED Study)	Subnational	Urban	12-18	12-18	535	461	546	471	
1464	Portugal	2013-2014	Cultural, social, economic, and environmental factors that can influence children's sport participation and obesity levels	Community	Both	6-10	6-10	385	408	385	408	
1465	Portugal	2015-2016	Childhood Obesity Surveillance Initiative 4	National	Both	6-8	6-8	3,346	3,399	3,346	3,399	1
1466	Portugal	2016-2017	Overweight and obesity and their associated factors among early adolescence school children in urban and rural Portugal (rural only)	National	Rural	10-12	10-12	38	33	38	33	
1467	Portugal	2016-2017	Portuguese Prevalence Study of Obesity in Childhood	Subnational	Both	5-10	5-10	3,572	3,522	3,572	3,522	
1468	Portugal	2018-2019	Childhood Obesity Surveillance Initiative 5	National	Both	6-7	6-7	2,918	2,795	2,903	2,772	1
1469	Puerto Rico	2010-2013	HPV Infection in a Population-Based Sample of Puerto Rican Women	Subnational	Both		16-64		118		22	
1470	Qatar	2006	World Health Survey	National	Both	18+	18+	399	540	67	60	
1471	Qatar	2011	Global School-based Student Health Survey	National	Both	13	13	102	126	102	126	
1472	Qatar	2012	STEPS	National	Both	18-64	18-64	296	399	70	88	
1473	Romania	1999	Romania physical development rural data	National	Rural	5-18	5-18	20,467	20,070	20,452	20,043	
1474	Romania	1999	Romania physical development urban data	National	Urban	5-18	5-18	21,423	23,605	21,423	23,605	
1475	Romania	2006-2008	Hypertension in Romanian Children and Adolescents: A Cross-Sectional Survey	Subnational	Both	5-17	5-17	2,313	2,339	2,313	2,339	
1476	Romania	2008	Healthy traditions for healthy children	Community	Rural	7-11	7-11	74	69	74	69	
1477	Romania	2008-2009	Healthy traditions for healthy children	Community	Urban	7-11	7-11	562	525	562	525	
1478	Romania	2009-2011	Study on children in Dolj County, South Romania	Subnational	Both	5-21	5-21	746	672	734	665	
1479	Romania	2010-2013	Healthy traditions for healthy children	Community	Urban	5-11	5-11	1,736	1,798	1,736	1,798	
1480	Romania	2011-2012	Study for the Evaluation of Prevalence of Hypertension and cArdiovascular Risk among the Adult Population of Romania - SEPHAR II	National	Both	18-80	18-80	134	124	4	5	
1481	Romania	2013	Childhood Obesity Surveillance Initiative 3	National	Both	8	8	2,175	2,173	2,175	2,173	1
1482	Romania	2013	Healthy traditions for healthy children	Subnational	Rural	5-11	5-11	123	107	123	107	

	Country	Study years	Survey/Study name/Citation	Level of representativeness	Rural, urban or both	Age range as in NCD-RisC database*		Sample size as in height analysis		Sample size as in BMI analysis		Note
						Male	Female	Male	Female	Male	Female	
1483	Romania	2013-2014	Auxological evaluation of school children in Mures County	Subnational	Both	6-14	6-14	936	957	936	957	
1484	Romania	2014	Timis County Study	Community	Urban	6-19	6-19	238	205	237	205	
1485	Romania	2014-2015	Healthy traditions for healthy children	Subnational	Rural	5-10	5-10	599	561	599	561	
1486	Romania	2015	Healthy traditions for healthy children	Community	Urban	5-10	5-10	183	211	183	210	
1487	Romania	2015-2016	Childhood Obesity Surveillance Initiative 4	National	Both	7-9	7-9	3,779	3,674	3,699	3,595	1
1488	Romania	2015-2016	Study for the Evaluation of Prevalence of Hypertension and cArdiovascular Risk among the Adult Population of Romania - SEPHAR III	National	Both	18-80	18-80	154	184	26	26	
1489	Romania	2016-2017	Erasmus plus KA2, Healthyland	Community	Urban	5-6	5-6	14	16	14	16	
1490	Romania	2017-2018	Erasmus plus KA2, Healthyland	Community	Urban	5-7	5-7	31	34	31	34	
1491	Romania	2018	Healthy traditions for healthy children	Community	Both	5-13	5-13	1,144	1,085	1,142	1,085	
1492	Romania	2019	Resita preschool measurements	Community	Urban	5-8	5-8	232	236	232	236	
1493	Russian Federation	1992-1993	Russia Longitudinal Monitoring Survey-Higher School of Economics Round II	National	Both	5+	5+	1,277	1,444	547	577	
1494	Russian Federation	1993	Russia Longitudinal Monitoring Survey-Higher School of Economics Round III	National	Both	5+	5+	2,371	2,454	1,631	1,536	
1495	Russian Federation	1993-1994	Russia Longitudinal Monitoring Survey-Higher School of Economics Round IV	National	Both	5+	5+	2,287	2,427	1,618	1,605	
1496	Russian Federation	1994	Russia Longitudinal Monitoring Survey-Higher School of Economics Round V	National	Both	5+	5+	1,980	2,056	1,268	1,242	
1497	Russian Federation	1994-1995	MONICA, Novosibirsk (intervention)	Community	Urban	25-64	25-64	85	90			
1498	Russian Federation	1995	Russia Longitudinal Monitoring Survey-Higher School of Economics Round VI	National	Both	5+	5+	1,878	1,950	1,210	1,201	
1499	Russian Federation	1995	MONICA, Novosibirsk, Kirowsky district	Community	Urban	25-64	25-64	77	82			
1500	Russian Federation	1996	Russia Longitudinal Monitoring Survey-Higher School of Economics Round VII	National	Both	5+	5+	1,845	1,970	1,179	1,167	
1501	Russian Federation	1997	Russian Karelia Survey in Pitkaranta	Community	Both	25-64	25-64	33	53			
1502	Russian Federation	1998-1999	Russia Longitudinal Monitoring Survey-Higher School of Economics Round VIII	National	Both	5+	5+	1,918	2,019	1,202	1,195	
1503	Russian Federation	2000	Russia Longitudinal Monitoring Survey-Higher School of Economics Round IX	National	Both	5+	5+	2,019	2,098	1,225	1,174	
1504	Russian Federation	2001	Russia Longitudinal Monitoring Survey-Higher School of Economics Round X	National	Both	5+	5+	2,178	2,326	1,298	1,269	
1505	Russian Federation	2002	Russia Longitudinal Monitoring Survey-Higher School of Economics Round XI	National	Both	5+	5+	2,244	2,372	1,301	1,291	
1506	Russian Federation	2002	Russian Karelia Survey in Pitkaranta	Community	Both	25-64	25-64	35	40			
1507	Russian Federation	2003	Russia Longitudinal Monitoring Survey-Higher School of Economics Round XII	National	Both	5+	5+	2,284	2,385	1,315	1,260	
1508	Russian Federation	2004	Russia Longitudinal Monitoring Survey-Higher School of Economics Round XIII	National	Both	5+	5+	2,225	2,347	1,267	1,231	
1509	Russian Federation	2005	Russia Longitudinal Monitoring Survey-Higher School of Economics Round XIV	National	Both	5+	5+	2,156	2,228	1,214	1,143	
1510	Russian Federation	2007	Russian Karelia Survey in Pitkaranta	Community	Both	25-64	25-64	17	26			
1511	Russian Federation	2015-2016	Childhood Obesity Surveillance Initiative 4	Community	Urban	6-8	6-8	1,499	1,529	1,499	1,529	1
1512	Rwanda	2000	DHS	National	Both		15-49		6,107		2,543	
1513	Rwanda	2005	DHS	National	Both		15-49		3,286		1,300	
1514	Rwanda	2010	DHS	National	Both	15-59	15-49	3,722	4,288	1,459	1,564	
1515	Rwanda	2012	STEPS	National	Both	15-64	15-64	1,001	1,646	264	414	
1516	Rwanda	2014-2015	DHS	National	Both	15-59	15-49	3,319	3,868	1,306	1,388	
1517	Saint Kitts and Nevis	2007	STEPS	Subnational	Both	25-64	25-64	63	130			
1518	Saint Kitts and Nevis	2011	Global School-based Student Health Survey	National	Both	13-17	13-17	651	814	650	814	
1519	Saint Lucia	2012	STEPS	National	Both	25-64	25-64	82	127			
1520	Saint Vincent and the Grenadines	2013-2014	STEPS	National	Both	18-69	18-69	295	462	57	68	
1521	Saint Vincent and the Grenadines	2018	Global School-based Student Health Survey	National	Both	13-17	13-17	738	841	737	839	
1522	Samoa	1979-1982	McGarvey, Am J Clin Nutr 53(6 Suppl):1586S-1594S, 1991	National	Both	5+	5+	207	176			
1523	Samoa	1995	McGarvey, Pac Health Dialog 8(1):157-62, 2001	National	Both	29+	30+	2				
1524	Samoa	2002	STEPS	National	Both	25-64	25-64	223	242			
1525	Samoa	2010	Samoan Genome-Wide Association Study	National	Both	24-65	24-65	182	250			
1526	Samoa	2013	STEPS	National	Both	18-64	18-64	202	297	29	41	
1527	Sao Tome and Principe	2008-2009	DHS	National	Both	15-59	15-49	1,150	1,448	455	465	
1528	Sao Tome and Principe	2009	STEPS	National	Both	25-64	25-64	284	339			
1529	Saudi Arabia	1985-1988	National Nutrition Survey	National	Both	5-75	5-75	2,960	3,287	273	542	
1530	Saudi Arabia	1989-1994	National Nutrition Survey	National	Both	18-40	18-40			1,452	1,905	
1531	Saudi Arabia	2005	STEPS	National	Both	15-64	15-64	982	1,115			
1532	Saudi Arabia	2005	El Mouzan et al., Ann Saudi Med 30(3):203Ð208, 2010	National	Both	5-18	5-18			9,853	9,519	
1533	Saudi Arabia	2007	Gulf Cooperation Council World Health Survey	National	Both	18+	18+	1,328	1,287	187	192	
1534	Saudi Arabia	2009-2010	Arab Teens Lifestyle Study (ATLS)	Subnational	Urban	14-19	14-19	1,384	1,479	1,384	1,479	
1535	Saudi Arabia	2011-2013	Jeddah City Study	Community	Urban	5+	5+	601	431	438	226	
1536	Saudi Arabia	2011-2012	Jeeluna Study- National Assessment of the Health Needs of Adolescents in Saudi Arabia	National	Both	12-19	12-19	6,245	5,796	6,234	5,790	
1537	Saudi Arabia	2013	Saudi Health Information Survey	National	Both	15+	15+	1,721	1,937	654	526	
1538	Senegal	1992-1993	DHS	National	Both		20-49		1,556			

	Country	Study years	Survey/Study name/Citation	Level of representativeness	Rural, urban or both	Age range as in NCD-RisC database*		Sample size as in height analysis		Sample size as in BMI analysis		Note
						Male	Female	Male	Female	Male	Female	
1539	Senegal	2003	Perceptions of healthy and desirable body size in urban Senegalese women	Community	Urban		20-50		117			
1540	Senegal	2005	DHS	National	Both		15-49		2,810		1,107	
1541	Senegal	2010-2011	DHS	National	Both	15-59	15-49	2,671	3,594	1,217	1,351	
1542	Senegal	2010-2012	Biocultural determinants of overweight and obesity in the context of nutrition transition in Senegal: a holistic anthropological approach	Subnational	Both	18+	18+	110	124	16	34	
1543	Senegal	2015	Les maladies chroniques au Sénégal: Une écologie de la santé comparative entre Dakar et Widou Thiengoly	Community	Both	20-100	20-100	306	320			
1544	Serbia	1994-1995	MONICA, Novi Sad	Community	Urban	25-64	25-64	66	67			
1545	Serbia	2000	Health Status, Health Needs and Utilization of Health Care of the Population of Serbia	National	Both	7+	7+	1,623	1,933	883	932	
1546	Serbia	2006	The 2006 National Health Survey for the Population of Serbia	National	Both	7+	7+	2,361	2,382	1,331	1,303	
1547	Serbia	2013	The National Health Survey of the Republic of Serbia, 2013	National	Both	7+	7+	1,970	2,018	1,097	1,093	
1548	Serbia	2013-2014	Stay Fit for Lifelong Health; the Prevalence of Lifestyle Health Conditions in Serbian Population	National	Urban	18-65		221		4		
1549	Serbia	2015-2016	Childhood Obesity Surveillance Initiative 4	National	Both	6-8	6-8	2,475	2,386	2,475	2,386	1
1550	Seychelles	1994	Seychelles Heart Survey II	National	Both	25-64	25-64	59	67			
1551	Seychelles	1998	School Screening Program	National	Both	5-16	5-16	1,567	1,424	1,567	1,424	
1552	Seychelles	1999	School Screening Program	National	Both	5-16	5-16	2,657	2,781	2,657	2,781	
1553	Seychelles	2000	School Screening Program	National	Both	5-16	5-16	1,781	1,831	1,781	1,831	
1554	Seychelles	2001	School Screening Program	National	Both	5-16	5-16	2,622	2,595	2,622	2,595	
1555	Seychelles	2002	School Screening Program	National	Both	5-16	5-16	2,495	2,536	2,495	2,536	
1556	Seychelles	2003	School Screening Program	National	Both	5-16	5-16	2,814	2,832	2,814	2,832	
1557	Seychelles	2004	School Screening Program	National	Both	5-16	5-16	2,359	2,317	2,359	2,317	
1558	Seychelles	2004	Seychelles Heart Survey III	National	Both	25-64	25-64	55	70			
1559	Seychelles	2005	School Screening Program	National	Both	5-16	5-16	2,738	2,775	2,738	2,774	
1560	Seychelles	2006	School Screening Program	National	Both	5-16	5-16	2,694	2,622	2,693	2,622	
1561	Seychelles	2007	Global School-based Student Health Survey	National	Both	13-17	13-17	387	467	385	467	
1562	Seychelles	2011	School Screening Program	National	Both	5-16	5-16	2,318	2,283	2,312	2,277	
1563	Seychelles	2012	School Screening Program	National	Both	5-16	5-16	2,283	2,327	2,283	2,326	
1564	Seychelles	2013	School Screening Program	National	Both	5-16	5-16	1,942	2,091	1,942	2,090	
1565	Seychelles	2014	School Screening Program	National	Both	5-16	5-16	2,145	2,219	2,144	2,219	
1566	Seychelles	2013-2014	Seychelles Heart Survey IV	National	Both	25-64	25-64	45	70			
1567	Seychelles	2015	Global School-based Student Health Survey	National	Both	13-17	13-17	770	888	770	888	
1568	Seychelles	2015	School Screening Program	National	Both	5-16	5-16	1,998	1,998	1,997	1,998	
1569	Seychelles	2016	School Screening Program	National	Both	5-16	5-16	1,783	1,895	1,780	1,894	
1570	Sierra Leone	2008	DHS	National	Both		15-49		2,007		543	
1571	Sierra Leone	2009	STEPS	National	Both	25-64	25-64	430	814			
1572	Sierra Leone	2013	DHS	National	Both	15-59	15-49	3,409	4,529	1,449	1,744	
1573	Singapore	1982-1985	Thyroid Heart Study	National	Both	18+	18+	28	31	61	66	3
1574	Singapore	1993-1995	NUH Heart Study	National	Both	26-89	26-89	19	9			
1575	Singapore	2004-2007	Combined follow up of Singapore Cardiovascular Cohort study and Singapore Prospective study	National	Both	24+	24+	90	126			
1576	Singapore	2012-2013	Singapore Health Study 2012	National	Both	18-79	18-79	224	218	36	44	
1577	Singapore	2014-2015	Singapore Health 2	National	Urban	18-80	18-80	115	138	15	21	
1578	Slovakia	1985	Effects of somatic development and environmental factors on blood pressure in children	Community	Urban	5-7	5-7	412	388	412	388	
1579	Slovakia	1993	Countrywide Integrated Noncommunicable Diseases Intervention Programme (CINDI)	National	Both	15-64	15-64	292	316	199	135	
1580	Slovakia	1998	Countrywide Integrated Noncommunicable Diseases Intervention Programme (CINDI)	National	Both	15-64	15-64	249	289	112	122	
1581	Slovakia	2001	Nation-wide Anthropological Survey	National	Both	6-18	6-18	11,193	11,023	11,191	10,953	
1582	Slovakia	2003	Countrywide Integrated Noncommunicable Diseases Intervention Programme (CINDI)	National	Both	15-64	15-64	188	200	89	60	
1583	Slovakia	2008	Countrywide Integrated Noncommunicable Diseases Intervention Programme (CINDI)	National	Both	15-64	15-64	92	108	36	36	
1584	Slovakia	2011	Nation-wide Anthropological Survey	National	Both	6-19	6-18	9,074	9,041	9,072	9,041	
1585	Slovakia	2011-2012	European Health Examination Survey	National	Both	18-64	18-64	213	240	46	68	
1586	Slovakia	2015-2016	Childhood Obesity Surveillance Initiative 4	National	Both	7	7	1,390	1,382	1,390	1,379	1
1587	Slovenia	1982	The SLOFIT monitoring system	National	Both	6-19	6-19	15,657	15,650	16,381	17,183	3
1588	Slovenia	1983	Analysis of Children's Development in Slovenia (ACDSi)	National	Both	7-14	7-14	1,580	1,576	1,580	1,576	3
1589	Slovenia	1983	The SLOFIT monitoring system	National	Both	6-19	6-19	16,022	16,432	16,365	17,435	3
1590	Slovenia	1984	The SLOFIT monitoring system	National	Both	6-19	6-19	21,381	22,694	21,438	22,933	3
1591	Slovenia	1985	The SLOFIT monitoring system	National	Both	6-19	6-19	22,505	23,046	22,496	23,043	
1592	Slovenia	1986	The SLOFIT monitoring system	National	Both	6-19	6-19	22,891	23,011	22,891	23,009	
1593	Slovenia	1987	The SLOFIT monitoring system	National	Both	6-19	6-19	49,090	48,788	49,089	48,784	

	Country	Study years	Survey/Study name/Citation	Level of representativeness	Rural, urban or both	Age range as in NCD-RisC database*		Sample size as in height analysis		Sample size as in BMI analysis		Note
						Male	Female	Male	Female	Male	Female	
1594	Slovenia	1988	The SLOFIT monitoring system	National	Both	6-19	6-19	85,446	83,394	85,444	83,390	
1595	Slovenia	1989	The SLOFIT monitoring system	National	Both	6-19	6-19	106,541	106,642	106,538	106,639	
1596	Slovenia	1990	The SLOFIT monitoring system	National	Both	6-19	6-19	129,317	128,572	129,317	128,572	
1597	Slovenia	1991	The SLOFIT monitoring system	National	Both	6-19	6-19	130,726	129,843	130,726	129,842	
1598	Slovenia	1992	The SLOFIT monitoring system	National	Both	6-19	6-19	135,243	134,855	135,239	134,853	
1599	Slovenia	1993	Analysis of Children's Development in Slovenia (ACDSi)	National	Both	6-14	6-14	1,674	1,678	1,674	1,678	
1600	Slovenia	1993	The SLOFIT monitoring system	National	Both	6-19	6-19	143,183	141,967	143,182	141,966	
1601	Slovenia	1994	Analysis of Children's Development in Slovenia (ACDSi)	National	Both	14-18	14-18	683	696	683	696	
1602	Slovenia	1994	The SLOFIT monitoring system	National	Both	6-19	6-19	145,348	143,290	145,348	143,290	
1603	Slovenia	1995	The SLOFIT monitoring system	National	Both	6-19	6-19	142,246	140,692	142,245	140,692	
1604	Slovenia	1996	The SLOFIT monitoring system	National	Both	6-19	6-19	140,718	138,122	140,714	138,120	
1605	Slovenia	1997	The SLOFIT monitoring system	National	Both	6-19	6-19	127,551	120,331	127,551	120,331	
1606	Slovenia	1998	The SLOFIT monitoring system	National	Both	6-19	6-19	126,342	121,550	126,342	121,549	
1607	Slovenia	1999	The SLOFIT monitoring system	National	Both	6-19	6-19	122,655	119,408	122,655	119,408	
1608	Slovenia	2000	The SLOFIT monitoring system	National	Both	6-19	6-19	121,918	116,281	121,918	116,281	
1609	Slovenia	2001	The SLOFIT monitoring system	National	Both	6-19	6-19	118,997	114,607	118,997	114,606	
1610	Slovenia	2002	The SLOFIT monitoring system	National	Both	6-19	6-19	114,716	110,691	114,716	110,691	
1611	Slovenia	2003	Analysis of Children's Development in Slovenia (ACDSi)	National	Both	5-14	5-14	2,061	1,939	2,061	1,939	
1612	Slovenia	2003	The SLOFIT monitoring system	National	Both	6-19	6-19	117,278	112,944	117,278	112,944	
1613	Slovenia	2004	Analysis of Children's Development in Slovenia (ACDSi)	National	Both	14-19	14-19	950	712	950	712	
1614	Slovenia	2004	The SLOFIT monitoring system	National	Both	6-19	6-19	117,768	112,576	117,768	112,575	
1615	Slovenia	2005	The SLOFIT monitoring system	National	Both	6-19	6-19	114,966	109,546	114,966	109,542	
1616	Slovenia	2006	The SLOFIT monitoring system	National	Both	6-19	6-19	109,878	102,840	109,877	102,838	
1617	Slovenia	2007	The SLOFIT monitoring system	National	Both	6-19	6-19	103,108	100,366	103,105	100,364	
1618	Slovenia	2008	The SLOFIT monitoring system	National	Both	6-19	6-19	103,736	98,071	103,735	98,070	
1619	Slovenia	2009	The SLOFIT monitoring system	National	Both	6-19	6-19	103,673	98,894	103,669	98,891	
1620	Slovenia	2010	The SLOFIT monitoring system	National	Both	6-19	6-19	101,165	96,687	101,163	96,686	
1621	Slovenia	2011	The SLOFIT monitoring system	National	Both	6-19	6-19	100,811	96,314	100,802	96,309	
1622	Slovenia	2012	The SLOFIT monitoring system	National	Both	6-19	6-19	101,178	95,917	101,172	95,917	
1623	Slovenia	2013	The SLOFIT monitoring system	National	Both	6-19	6-19	99,198	95,669	99,191	95,666	
1624	Slovenia	2013-2014	Analysis of Children's Development in Slovenia (ACDSi), 10-15 year olds	National	Both	6-15	6-15	1,665	1,627	1,665	1,627	
1625	Slovenia	2014	Analysis of Children's Development in Slovenia (ACDSi)	National	Both	14-19	14-19	704	724	703	724	
1626	Slovenia	2014	The SLOFIT monitoring system	National	Both	6-21	6-21	102,793	97,864	102,558	97,736	
1627	Slovenia	2015	The SLOFIT monitoring system	National	Both	6-19	6-19	103,570	99,295	103,568	99,293	
1628	Slovenia	2016	The SLOFIT monitoring system	National	Both	6-19	6-19	107,427	102,180	107,421	102,174	
1629	Slovenia	2017	The SLOFIT monitoring system	National	Both	6-19	6-19	108,784	104,582	108,780	104,582	
1630	Slovenia	2018	The SLOFIT monitoring system	National	Both	6-19	6-19	110,216	105,866	110,213	105,861	
1631	Slovenia	2019	The SLOFIT monitoring system	National	Both	6-19	6-19	99,448	94,793	99,445	94,791	
1632	Solomon Islands	2004	A genetic-ecological study of the risk factors for lifestyle-related diseases in Oceanian populations	Community	Rural	18-74	18-74	39	40	3	5	
1633	Solomon Islands	2004	A genetic-ecological study of the risk factors for lifestyle-related diseases in Oceanian populations	Community	Urban	18-79	20-79	11	18	4		
1634	Solomon Islands	2006	STEPS	Subnational	Both	15-64	15-64	480	637	116	146	
1635	Solomon Islands	2009-2010	Furusawa et al., N Z Med J 124(1333):17-28, 2011	Subnational	Rural	5+	5+	126	154	108	108	
1636	Solomon Islands	2009-2010	Furusawa et al., N Z Med J 124(1333):17-28, 2011	Subnational	Urban	5-70	5-70	46	68	40	47	
1637	Solomon Islands	2015	STEPS	National	Both	18-69	18-69	169	215	29	45	
1638	Somalia	2016	The prevalence of selected risk factors for non-communicable diseases in Hargeisa, Somaliland: a cross-sectional study	Community	Urban	20-69	20-69	55	301			
1639	South Africa	1996	Ellisras Longitudinal Study	Community	Rural	5-10	5-10	584	535	583	535	
1640	South Africa	1997	Ellisras Longitudinal Study	Community	Rural	5-11	5-11	1,046	968	1,046	968	
1641	South Africa	1998	DHS	National	Both	15+	15+	2,424	2,900	1,066	1,048	
1642	South Africa	1998	Ellisras Longitudinal Study	Community	Rural	5-12	5-12	958	856	958	856	
1643	South Africa	1999	Ellisras Longitudinal Study	Community	Rural	5-13	5-13	991	917	991	917	
1644	South Africa	2000	Ellisras Longitudinal Study	Community	Rural	5-14	5-14	936	877	936	877	
1645	South Africa	2001	Ellisras Longitudinal Study	Community	Rural	6-15	6-15	962	904	962	904	
1646	South Africa	2000-2001	Transition and Health during Urbanisation of South Africans: Children	Subnational	Both	9-15	9-15	606	640	606	639	
1647	South Africa	2002	Ellisras Longitudinal Study	Community	Rural	7-16	7-16	890	823	890	823	
1648	South Africa	2003	DHS	National	Both	15+	15+	1,433	1,711	634	648	
1649	South Africa	2003	Ellisras Longitudinal Study	Community	Rural	8-17	8-17	911	858	911	858	

	Country	Study years	Survey/Study name/Citation	Level of representativeness	Rural, urban or both	Age range as in NCD-RisC database*		Sample size as in height analysis		Sample size as in BMI analysis		Note
						Male	Female	Male	Female	Male	Female	
1650	South Africa	2003-2004	Africa Centre Biomeasure Survey	Community	Rural	25-49	25-49	176	355			
1651	South Africa	2008	National Income Dynamics Study Wave I	National	Both	5+	5+	5,251	5,873	3,804	3,748	
1652	South Africa	2007-2008	Cardiometabolic risk profile of South African Learners	Subnational	Both	10-16	10-16	496	776	496	776	
1653	South Africa	2010	Africa Centre Biomeasure Survey	Community	Rural	15+	15+	1,836	2,782	1,022	1,045	
1654	South Africa	2010-2011	National Income Dynamics Study Wave II	National	Both	5+	5+	5,440	6,051	3,575	3,681	
1655	South Africa	2011-2013	International Study of Childhood Obesity, Lifestyle and the Environment (ISCOLE)	Community	Urban	9-11	9-11	222	327	222	327	
1656	South Africa	2012	National Income Dynamics Study Wave III	National	Both	5+	5+	7,219	7,919	5,087	5,085	
1657	South Africa	2012	South African National Health and Nutrition Examination Survey	National	Both	5+	5+	414	837			
1658	South Africa	2014-2015	National Income Dynamics Study Wave IV	National	Both	5+	5+	8,926	9,539	6,080	6,071	
1659	South Africa	2016	DHS	National	Both	15-59	15-49	1,420	1,736	565	563	
1660	South Africa	2017	National Income Dynamics Study Wave V	National	Both	5+	5+	9,006	9,820	6,166	6,323	
1661	South Korea	1975	Kim et al., Am J Phys Anthropol 136:230-6, 2008; Nationwide Cross-sectional Anthropometric Survey	National	Both	5-20	5-20	9,864	9,329			
1662	South Korea	1984	Kim et al., Am J Phys Anthropol 136:230-6, 2008; Nationwide Cross-sectional Anthropometric Survey	National	Both	5-20	5-20	42,347	40,797			
1663	South Korea	1998	Korea National Health and Nutrition Examination Survey	National	Both	10+	10+	1,576	1,726	917	891	
1664	South Korea	1997-1998	National Anthropometric Survey in Korean Children and Adolescents	National	Both	5-19	5-19	29,318	26,469	29,318	26,469	
1665	South Korea	1999	The South Korean Conscription Database	National	Both	19		401,721		401,721		
1666	South Korea	2000	The South Korean Conscription Database	National	Both	19		402,758		402,758		
1667	South Korea	2001	Korea National Health and Nutrition Examination Survey	National	Both	5+	5+	1,751	1,796	1,286	1,163	
1668	South Korea	2001	The South Korean Conscription Database	National	Both	19		398,653		398,653		
1669	South Korea	2002	The South Korean Conscription Database	National	Both	19		367,024		367,024		
1670	South Korea	2003	The South Korean Conscription Database	National	Both	19		329,626		329,626		
1671	South Korea	2004	The South Korean Conscription Database	National	Both	19		323,001		323,001		
1672	South Korea	2005	Korea National Health and Nutrition Examination Survey	National	Both	5+	5+	1,131	1,202	858	800	
1673	South Korea	2005	The South Korean Conscription Database	National	Both	19		313,378		313,378		
1674	South Korea	2005	National Anthropometric Survey in Korean Children and Adolescents	National	Both	5-19	5-19	41,723	39,197	41,727	39,200	
1675	South Korea	2006	The South Korean Conscription Database	National	Both	19		302,587		302,587		
1676	South Korea	2007	Korea National Health and Nutrition Examination Survey	National	Both	5+	5+	634	642	497	460	
1677	South Korea	2007	The South Korean Conscription Database	National	Both	19		312,795		312,795		
1678	South Korea	2008	Korea National Health and Nutrition Examination Survey	National	Both	5+	5+	1,386	1,451	1,056	957	
1679	South Korea	2008	The South Korean Conscription Database	National	Both	19		312,919		312,919		
1680	South Korea	2009	Korea National Health and Nutrition Examination Survey	National	Both	5+	5+	1,524	1,531	1,083	1,008	
1681	South Korea	2009	Korea National School Health Examination Survey (KNSHES)	National	Both	6-20	6-20	104,042	89,897	103,997	89,881	
1682	South Korea	2009	The South Korean Conscription Database	National	Both	19		324,818		324,818		
1683	South Korea	2007-2012	JS High-School Study	Community	Rural	14-17	14-17	553	508	553	508	
1684	South Korea	2010	Korea National Health and Nutrition Examination Survey	National	Both	5+	5+	1,178	1,226	897	802	
1685	South Korea	2010	Korea National School Health Examination Survey (KNSHES)	National	Both	6-20	6-20	99,965	86,244	56,924	46,287	
1686	South Korea	2010	The South Korean Conscription Database	National	Both	19		347,249		347,249		
1687	South Korea	2011	Korea National Health and Nutrition Examination Survey	National	Both	5+	5+	1,022	1,063	752	700	
1688	South Korea	2011	Korea National School Health Examination Survey (KNSHES)	National	Both	6-20	6-20	97,377	83,059	97,363	83,046	
1689	South Korea	2011	The South Korean Conscription Database	National	Both	19		364,982		364,982		
1690	South Korea	2012	Korea National Health and Nutrition Examination Survey	National	Both	5+	5+	961	982	722	619	
1691	South Korea	2012	Korea National School Health Examination Survey (KNSHES)	National	Both	6-20	6-20	45,075	42,009	45,066	42,005	
1692	South Korea	2012	The South Korean Conscription Database	National	Both	19		361,009		361,009		
1693	South Korea	2013	Korea National Health and Nutrition Examination Survey	National	Both	5+	5+	1,052	1,071	771	702	
1694	South Korea	2013	Korea National School Health Examination Survey (KNSHES)	National	Both	6-20	6-20	43,685	40,791	43,675	40,785	
1695	South Korea	2013	The South Korean Conscription Database	National	Both	19		363,914		363,914		
1696	South Korea	2014	Korea National Health and Nutrition Examination Survey	National	Both	5+	5+	845	895	621	554	
1697	South Korea	2014	Korea National School Health Examination Survey (KNSHES)	National	Both	6-20	6-20	42,580	39,991	42,570	39,987	
1698	South Korea	2014	The South Korean Conscription Database	National	Both	19		363,597		363,597		
1699	South Korea	2015	Korea National Health and Nutrition Examination Survey	National	Both	5+	5+	909	853	612	539	
1700	South Korea	2015	Korea National School Health Examination Survey (KNSHES)	National	Both	6-20	6-20	43,152	41,654	43,141	41,645	
1701	South Korea	2015	The South Korean Conscription Database	National	Both	19		350,518		350,518		
1702	South Korea	2016	Korea National Health and Nutrition Examination Survey	National	Both	5+	5+	952	998	676	636	
1703	South Korea	2016	Korea National School Health Examination Survey (KNSHES)	National	Both	6-20	6-20	42,245	40,635	42,242	40,631	
1704	South Korea	2016	The South Korean Conscription Database	National	Both	19		339,410		339,410		

	Country	Study years	Survey/Study name/Citation	Level of representativeness	Rural, urban or both	Age range as in NCD-RisC database*		Sample size as in height analysis		Sample size as in BMI analysis		Note
						Male	Female	Male	Female	Male	Female	
1705	South Korea	2017	Korea National Health and Nutrition Examination Survey	National	Both	5+	5+	942	963	624	608	
1706	South Korea	2017	The South Korean Conscription Database	National	Both	19		323,457		323,457		
1707	Spain	1989	Cardiovascular Risk Factors Study in Catalonia	Subnational	Both	15+	15+	20	28	20	28	
1708	Spain	1991-1993	Encuesta de Factores de Riesgo Cardiovascular en la Region de Murcia (Cardiovascular Risk Factors Survey)	Subnational	Both	18-69	18-69	389	382	9	11	
1709	Spain	1994-1995	Encuesta de Nutrición y Salud Comunidad Valenciana 1994-95 (ENCV)	Subnational	Urban	15+	15+	299	317	109	124	
1710	Spain	1998-2000	EnKID study	National	Both	5-24	5-24	1,452	1,730	1,030	1,120	
1711	Spain	2001-2002	Catalan Health Interview Survey	Subnational	Both	18-74	18-74	102	158	15	23	
1712	Spain	2001-2003	DIabetes, Nutrición y Obesidad en la población adulta de la Región de Murcia (DINO)	Subnational	Both	20+	20+	135	157			
1713	Spain	2000-2005	CDC of the Canary Islands	Subnational	Both	18-75	18-75	484	647	36	71	
1714	Spain	2004	Cardiovascular Risk Study in Castilla y León (RECCyL)	Subnational	Both	15+	15+	311	340	96	88	
1715	Spain	2004	Vioque J et al. Obesity 16:664-70, 2008	Community	Urban	24+	24+	12	13			
1716	Spain	2006-2007	HELENA	Community	Urban	12-17	12-17	188	193	188	193	
1717	Spain	2006-2008	Biblioni Mdel et al., Br J Nutr 103(1):99-106, 2010	Community	Both	12-17	12-17			571	652	
1718	Spain	2007-2009	Harmonizing Equation of Risk in Mediterranean countries Extremadura (HERMEX)	Subnational	Both	25-79	25-79	72	101			
1719	Spain	2008-2010	Study on Nutrition and Cardiovascular Risk in Spain	National	Both	18+	18+	1,001	1,147	147	164	
1720	Spain	2007-2010	Identification and prevention of Dietary- and lifestyle-induced health EFfects In Children and infantS (IDEFICS)	Community	Urban	5-9	5-9	474	468	474	468	
1721	Spain	2009	Cardiovascular Risk Study in Castilla y León (RECCyL)	Subnational	Both	20+	20+	105	128			
1722	Spain	2010-2011	Childhood Obesity Surveillance Initiative 2	National	Both	6-9	6-9	3,839	3,817	3,837	3,817	1
1723	Spain	2012	Effects of a lifestyle intervention on the prevention of childhood obesity: a community-based model	Subnational	Urban	8-12	8-12	1,171	1,081	1,171	1,081	
1724	Spain	2013	ANIBES Study	National	Both	9-75	9-75	444	344	302	189	
1725	Spain	2013	Childhood Obesity Surveillance Initiative 3	National	Both	7-8	7-8	1,682	1,744	1,682	1,744	1
1726	Spain	2012-2013	Valencia Cohort INMA Project	Subnational	Both	7-8	7-8	229	232	229	232	
1727	Spain	2012-2013	Brain Development and Air Pollution Ultrafine Particles in School Children-BREATHE Project	Subnational	Urban	7-12	7-12	1,338	1,325	1,338	1,325	
1728	Spain	2012-2013	AMICS-INfancia y Medio Ambiente (Childhood and Environment) Project - Menorca	Subnational	Both	14-15	14-15	162	165	162	165	
1729	Spain	2012-2013	Infancia y Medio Ambiente (Childhood and Environment) Birth Cohort study - Sabadell	Subnational	Urban	5-8	5-8	280	260	280	260	
1730	Spain	2013-2014	Effects of a lifestyle intervention on the prevention of childhood obesity: a community-based model	Subnational	Urban	9-13	9-13	1,086	1,004	1,086	1,004	
1731	Spain	2014	Cardiovascular Risk Study in Castilla y León (RECCyL)	Subnational	Both	20+	20+	52	52			
1732	Spain	2015-2016	Childhood Obesity Surveillance Initiative 4	National	Both	6-9	6-9	5,532	5,367	5,532	5,367	1
1733	Spain	2015-2016	Infancia y Medio Ambiente (Childhood and Environment) Birth Cohort study – Gipuzkoa	Subnational	Both	7-8	7-8	192	189	192	189	
1734	Spain	2018	Childhood obesity cohort study of Sant Boi de Llobregat - SantBoiSà	Community	Urban	5-10	5-10	299	266	299	266	
1735	Spain	2019	Physical Activity, Sedentarism and Obesity of Spanish youth	National	Both	5-19	5-19	1,824	1,929	1,824	1,928	
1736	Sri Lanka	2006	STEPS	National	Both	15-64	15-64	1,822	1,857	688	519	
1737	Sri Lanka	2014	STEPS	National	Both	18-69	18-69	260	447	57	70	
1738	Sri Lanka	2016	Global School-based Student Health Survey	National	Both	13-17	13-17	671	928	671	923	
1739	State of Palestine	1996-1998	Kobar, rural	Community	Rural	15-64	15-64		259		83	
1740	State of Palestine	1996-1998	Old Ramallah, urban	Community	Urban	15-64	15-64		228		63	
1741	State of Palestine	1999-2000	The First National Health and Nutrition Survey	National	Both	18-64	18-64	546	711	71	85	
1742	State of Palestine	2010	STEPS	National	Both	15-64	15-64	961	1,153	419	358	
1743	State of Palestine	2010	Global School-based Student Health Survey	National	Both	13-17	13-17	1,823	1,865	1,822	1,865	
1744	Sudan (former)	2005	STEPS	Subnational	Both	25-64	25-64	166	265			
1745	Sudan (former)	2016	STEPS	National	Both	18-69	18-69	704	1,448	155	236	
1746	Suriname	2013-2015	The Healthy Life in Suriname Study (HELISUR)	Subnational	Urban	18-70	18-70	93	151	5	6	
1747	Sweden	1973	BMI Epidemiology Study	Community	Urban	7		257				
1748	Sweden	1974	BMI Epidemiology Study	Community	Urban	8		386				
1749	Sweden	1975	BMI Epidemiology Study	Community	Urban	9		35				
1750	Sweden	1978	BMI Epidemiology Study	Community	Urban	7		212				
1751	Sweden	1979	BMI Epidemiology Study	Community	Urban	8		409				
1752	Sweden	1979-1980	1973 Birth Cohort	National	Both	6	6	451	441			
1753	Sweden	1980	BMI Epidemiology Study	Community	Urban	9		324				
1754	Sweden	1980-1981	1973 Birth Cohort	National	Both	7	7	1,428	1,334			
1755	Sweden	1981-1982	1973 Birth Cohort	National	Both	8	8	935	902	890	869	3
1756	Sweden	1982	The Swedish Conscription Database	National	Both	17-18				54,288		3
1757	Sweden	1982-1983	1973 Birth Cohort	National	Both	9	9	717	669	670	635	3

	Country	Study years	Survey/Study name/Citation	Level of representativeness	Rural, urban or both	Age range as in NCD-RisC database*		Sample size as in height analysis		Sample size as in BMI analysis		Note
						Male	Female	Male	Female	Male	Female	
1758	Sweden	1983	The Swedish Conscription Database	National	Both	17-18		22,425		52,895		3
1759	Sweden	1983	BMI Epidemiology Study	Community	Urban	7		103				
1760	Sweden	1983-1984	1973 Birth Cohort	National	Both	10	10	1,488	1,367	1,448	1,340	3
1761	Sweden	1984	The Swedish Conscription Database	National	Both	17-18		36,094		36,273		3
1762	Sweden	1984	BMI Epidemiology Study	Community	Urban	8		407				
1763	Sweden	1984-1985	1973 Birth Cohort	National	Both	11	11	523	542	487	498	
1764	Sweden	1985	BMI Epidemiology Study	Community	Urban	9		357		357		
1765	Sweden	1985	The Swedish Conscription Database	National	Both	17-18		10,981		14,918		
1766	Sweden	1985-1986	1973 Birth Cohort	National	Both	12	12	1,323	1,226	1,238	1,158	
1767	Sweden	1986	The Swedish Conscription Database	National	Both	17-18		44,812		48,454		
1768	Sweden	1986-1987	1973 Birth Cohort	National	Both	13	13	799	823	709	729	
1769	Sweden	1986-1987	1981 Birth Cohort	National	Both	5	5	940	961	935	958	
1770	Sweden	1987	The Swedish Conscription Database	National	Both	17-18		48,893		49,306		
1771	Sweden	1987-1988	1973 Birth Cohort	National	Both	14	14	1,455	1,342	1,405	1,294	
1772	Sweden	1987-1988	1981 Birth Cohort	National	Both	6	6	811	781	808	780	
1773	Sweden	1988	BMI Epidemiology Study	Community	Urban	7		65		65		
1774	Sweden	1988	The Swedish Conscription Database	National	Both	17-18		47,454		47,923		
1775	Sweden	1988-1989	1973 Birth Cohort	National	Both	15	15	1,069	1,008	964	929	
1776	Sweden	1988-1989	1981 Birth Cohort	National	Both	7	7	1,521	1,499	1,508	1,489	
1777	Sweden	1989	BMI Epidemiology Study	Community	Urban	8		407		407		
1778	Sweden	1989	The Swedish Conscription Database	National	Both	17-18		47,595		48,117		
1779	Sweden	1989-1990	1973 Birth Cohort	National	Both	16	16	971	856	912	785	
1780	Sweden	1989-1990	1981 Birth Cohort	National	Both	8	8	1,515	1,535	1,497	1,511	
1781	Sweden	1990	BMI Epidemiology Study	Community	Urban	9		379		379		
1782	Sweden	1990	The Swedish Conscription Database	National	Both	17-18		48,195		48,882		
1783	Sweden	1990-1991	1973 Birth Cohort	National	Both	17	17	910	610	881	588	
1784	Sweden	1990	1981 Birth Cohort	National	Both	9	9	1,102	1,118	1,091	1,108	
1785	Sweden	1991	The Swedish Conscription Database	National	Both	17-18		48,404		49,150		
1786	Sweden	1992	1973 Birth Cohort	National	Both	18	18	1,296	402	1,290	390	
1787	Sweden	1991-1992	1981 Birth Cohort	National	Both	10	10	1,411	1,434	1,405	1,415	
1788	Sweden	1992	The Swedish Conscription Database	National	Both	17-18		46,441		47,275		
1789	Sweden	1992-1993	1981 Birth Cohort	National	Both	11	11	807	812	785	785	
1790	Sweden	1993	BMI Epidemiology Study	Community	Urban	7		125		125		
1791	Sweden	1993	The Swedish Conscription Database	National	Both	17-18		45,286		46,245		
1792	Sweden	1993-1994	1981 Birth Cohort	National	Both	12	12	1,343	1,405	1,318	1,378	
1793	Sweden	1994	BMI Epidemiology Study	Community	Urban	8		376		376		
1794	Sweden	1994	The Swedish Conscription Database	National	Both	17-18		42,646		43,648		
1795	Sweden	1994	MONICA Northern Sweden	Subnational	Both	25-74	25-74	85	88			
1796	Sweden	1994-1995	1981 Birth Cohort	National	Both	13	13	964	1,015	924	954	
1797	Sweden	1995	BMI Epidemiology Study	Community	Urban	9		293		293		
1798	Sweden	1995	The Swedish Conscription Database	National	Both	17-18		42,519		43,674		
1799	Sweden	1995	MONICA Gothenburg	Community	Urban	25-64	25-64	63	84			
1800	Sweden	1995-1996	1981 Birth Cohort	National	Both	14	14	1,238	1,316	1,202	1,266	
1801	Sweden	1996	The Swedish Conscription Database	National	Both	17-18		42,349		43,794		
1802	Sweden	1996-1997	1981 Birth Cohort	National	Both	15	15	1,187	1,190	1,143	1,134	
1803	Sweden	1997	The Swedish Conscription Database	National	Both	17-18		36,048		37,078		
1804	Sweden	1997-1998	1981 Birth Cohort	National	Both	16	16	850	897	819	862	
1805	Sweden	1998	BMI Epidemiology Study	Community	Urban	7		707		707		
1806	Sweden	1998	The Swedish Conscription Database	National	Both	17-18		41,084		42,564		
1807	Sweden	1998-1999	1981 Birth Cohort	National	Both	17	17	416	178	406	171	
1808	Sweden	1999	1981 Birth Cohort	National	Both	18	18	831	23	831	23	
1809	Sweden	1999	BMI Epidemiology Study	Community	Urban	8		980		980		
1810	Sweden	1998-1999	European Youth Heart Study (EYHS) I	Subnational	Urban	8-16	8-16	526	603	525	602	
1811	Sweden	1999	The Swedish Conscription Database	National	Both	17-18		36,319		37,852		
1812	Sweden	1999	MONICA Northern Sweden	Subnational	Both	25-74	25-74	73	74			
1813	Sweden	2000	BMI Epidemiology Study	Community	Urban	9		860		860		
1814	Sweden	2000	The Swedish Conscription Database	National	Both	17-18		29,913		31,328		

	Country	Study years	Survey/Study name/Citation	Level of representativeness	Rural, urban or both	Age range as in NCD-RisC database*		Sample size as in height analysis		Sample size as in BMI analysis		Note
						Male	Female	Male	Female	Male	Female	
1815	Sweden	2000-2002	The COMPASS study	Community	Urban	14-16	14-16	1,718	1,597	1,718	1,597	
1816	Sweden	2001	The Swedish Conscription Database	National	Both	17-18		28,297		29,809		
1817	Sweden	2002	The Swedish Conscription Database	National	Both	17-18		23,303		24,580		
1818	Sweden	2003	BMI Epidemiology Study	Community	Urban	7		15		15		
1819	Sweden	2003	The Swedish Conscription Database	National	Both	17-18		27,600		29,198		
1820	Sweden	2001-2004	Swedish INTERGENE Cohort Study	Subnational	Both	24-76	24-76	82	107			
1821	Sweden	2004	BMI Epidemiology Study	Community	Urban	8		285		285		
1822	Sweden	2004	The Swedish Conscription Database	National	Both	17-18		26,439		27,857		
1823	Sweden	2004	MONICA Northern Sweden	Subnational	Both	26-75	26-75	69	58			
1824	Sweden	2005	BMI Epidemiology Study	Community	Urban	9		301		301		
1825	Sweden	2004-2005	European Youth Heart Study (EYHS) II	Subnational	Urban	15-21	15-21	196	262	126	151	
1826	Sweden	2005	The Swedish Conscription Database	National	Both	17-18				25,836		
1827	Sweden	2007	BMI Epidemiology Study	Community	Urban	7		1,327		1,327		
1828	Sweden	2006-2007	HELENA	Community	Urban	12-17	12-17	132	208	132	208	
1829	Sweden	2008	BMI Epidemiology Study	Community	Urban	7-8		2,501		2,501		
1830	Sweden	2008	Childhood Obesity Surveillance Initiative 1	National	Both	7-9	7-9	2,374	2,189	2,374	2,189	1
1831	Sweden	2009	BMI Epidemiology Study	Community	Urban	7-9		3,706		3,706		
1832	Sweden	2007-2010	Identification and prevention of Dietary- and lifestyle-induced health EFfects In Children and infantS (IDEFICS)	Community	Urban	5-9	5-9	557	557	557	557	
1833	Sweden	2009	MONICA Northern Sweden	Subnational	Both	25-74	25-74	77	99			
1834	Sweden	2010	BMI Epidemiology Study	Community	Urban	8-9		2,425		2,425		
1835	Sweden	2011	BMI Epidemiology Study	Community	Urban	9		1,412		1,412		
1836	Sweden	2012	BMI Epidemiology Study	Community	Urban	7		1,551		1,551		
1837	Sweden	2013	BMI Epidemiology Study	Community	Urban	7-8		2,875		2,875		
1838	Sweden	2014	BMI Epidemiology Study	Community	Urban	7-9		4,251		4,251		
1839	Sweden	2014	MONICA Northern Sweden	Subnational	Both	25-74	25-74	58	52			
1840	Sweden	2015	BMI Epidemiology Study	Community	Urban	8-9		1,917		1,917		
1841	Sweden	2016	BMI Epidemiology Study	Community	Urban	9		35		35		
1842	Sweden	2015-2016	Childhood Obesity Surveillance Initiative 4	National	Both	6-9	6-9	4,071	3,798	4,070	3,798	1
1843	Switzerland	1992-1993	The Swiss MONICA Study Wave III	Subnational	Both	25-74	25-74	86	101			
1844	Switzerland	1993-1994	The Swiss Conscription Database	National	Both	19		23,499				
1845	Switzerland	2002	Prevalence of overweight and obesity in 6-12-year old children in Switzerland	National	Both	6-12	6-12	1,196	1,235	1,196	1,235	
1846	Switzerland	2004	The Swiss Conscription Database	National	Both	18-20		20,491		16,987		
1847	Switzerland	2005	Kinder- und Jugendsportstudie (KISS)	Subnational	Both	6-13	6-13	239	256	239	256	
1848	Switzerland	2005	The Swiss Conscription Database	National	Both	18-20		32,131		24,432		
1849	Switzerland	2005-2006	Chiolero et al., J Hypertens 25(11):2209-17, 2007	Subnational	Both	10-14	10-14	2,621	2,586	2,621	2,586	
1850	Switzerland	2006	Kinder- und Jugendsportstudie (KISS)	Subnational	Both	7-14	7-14	100	105	100	105	
1851	Switzerland	2005-2006	BMI Monitoring for Switzerland - Study 1	Community	Urban	5-11	5-11	4,477	4,103	4,477	4,103	
1852	Switzerland	2006	The Swiss Conscription Database	National	Both	18-20		34,530		25,845		
1853	Switzerland	2007	Prevalence of overweight and obesity in 6-12-year old children in Switzerland	National	Both	6-12	6-12	1,082	1,136	1,082	1,136	
1854	Switzerland	2006-2007	BMI Monitoring for Switzerland - Study 1	Community	Urban	5-11	5-11	4,061	3,896	4,061	3,896	
1855	Switzerland	2007	The Swiss Conscription Database	National	Both	18-20		36,194		27,229		
1856	Switzerland	2007-2008	BMI Monitoring for Switzerland - Study 1	Community	Urban	5-11	5-11	4,067	4,009	4,067	4,009	
1857	Switzerland	2008	The Swiss Conscription Database	National	Both	18-20		34,497		25,977		
1858	Switzerland	2009	Kinder- und Jugendsportstudie (KISS)	Subnational	Both	10-17	10-17	44	65	44	65	
1859	Switzerland	2008-2009	BMI Monitoring for Switzerland - Study 1	Community	Urban	5-11	5-11	3,998	3,847	3,998	3,847	
1860	Switzerland	2008-2010	BMI Monitoring for Switzerland - Study 2	Subnational	Both	6	6	975	1,034	975	1,034	
1861	Switzerland	2009	The Swiss Conscription Database	National	Both	18-20		34,896		25,811		
1862	Switzerland	2009-2010	BMI Monitoring for Switzerland - Study 1	Community	Urban	5-11	5-11	4,051	3,913	4,051	3,913	
1863	Switzerland	2010	The Swiss Conscription Database	National	Both	18-20		37,214		27,970		
1864	Switzerland	2007-2012	Bus Santé Study	Subnational	Urban	20-80	20-80	60	57			
1865	Switzerland	2010-2011	BMI Monitoring for Switzerland - Study 1	Community	Urban	5-11	5-11	4,092	3,734	4,092	3,734	
1866	Switzerland	2011	The Swiss Conscription Database	National	Both	18-20		38,108		28,965		
1867	Switzerland	2012	Prevalence of overweight and obesity in 6-12-year old children in Switzerland	National	Both	6-12	6-12	1,499	1,464	1,499	1,464	
1868	Switzerland	2011-2012	BMI Monitoring for Switzerland - Study 1	Community	Urban	5-11	5-11	4,085	3,940	4,085	3,940	
1869	Switzerland	2010-2013	BMI Monitoring for Switzerland - Study 2	Subnational	Both	5-11	5-11	11,371	10,525	11,371	10,525	
1870	Switzerland	2012	The Swiss Conscription Database	National	Both	18-20		36,938		28,348		

	Country	Study years	Survey/Study name/Citation	Level of representativeness	Rural, urban or both	Age range as in NCD-RisC database*		Sample size as in height analysis		Sample size as in BMI analysis		Note
						Male	Female	Male	Female	Male	Female	
1871	Switzerland	2012-2013	BMI Monitoring for Switzerland - Study 1	Community	Urban	5-11	5-11	4,224	4,042	4,224	4,042	
1872	Switzerland	2013	The Swiss Conscription Database	National	Both	18-20		32,890		25,315		
1873	Switzerland	2013-2014	BMI Monitoring for Switzerland - Study 1	Community	Urban	5-11	5-11	4,333	4,073	4,333	4,073	
1874	Switzerland	2014	The Swiss Conscription Database	National	Both	18-20		32,691		26,521		
1875	Switzerland	2014-2016	BMI Monitoring for Switzerland - Study 2	Subnational	Both	5-11	5-11	8,104	7,786	8,104	7,786	
1876	Switzerland	2015	The Swiss Conscription Database	National	Both	18-20		32,616		26,558		
1877	Switzerland	2013-2016	Bus Santé Study	Subnational	Urban	20-74	20-74	306	331			
1878	Switzerland	2014-2015	BMI Monitoring for Switzerland - Study 1	Community	Urban	5-11	5-11			4,657	4,625	
1879	Switzerland	2015-2016	BMI Monitoring for Switzerland - Study 1	Community	Urban	5-11	5-11	3,326	3,058	3,326	3,058	
1880	Switzerland	2016-2017	BMI Monitoring for Switzerland - Study 1	Community	Urban	5-11	5-11	3,457	3,121	3,457	3,121	
1881	Switzerland	2017-2018	National Studie Gesundheit und Ernährung von Primarschülern (CHildHNS)	National	Both	6-12	6-12	1,135	1,144	1,135	1,144	
1882	Taiwan (province of China)	1993-1996	Nutrition and Health Survey in Taiwan 1993-1996	National	Both	5+	5+	1,683	1,793	1,536	1,610	
1883	Taiwan (province of China)	2001-2002	Nutrition and Health Survey in Taiwan 2001-2002	National	Both	6-12	6-12	1,334	1,139	1,334	1,139	
1884	Taiwan (province of China)	2005-2008	Nutrition and Health Survey in Taiwan 2005-2008	National	Both	19+	19+	151	174	13	7	
1885	Taiwan (province of China)	2007	Taiwanese Survey on Hypertension, Hyperglycemia and Hyperlipidemia	National	Both	20+	20+	368	370			
1886	Taiwan (province of China)	2010	Nutrition and Health Survey in Taiwan	National	Both	13-15	13-15	852	927	852	927	
1887	Taiwan (province of China)	2011	Nutrition and Health Survey in Taiwan	National	Both	16-18	16-18	580	591	580	591	
1888	Taiwan (province of China)	2012	Global School-based Student Health Survey	National	Both	13-17	13-17	2,998	2,927	2,998	2,927	
1889	Taiwan (province of China)	2012	Nutrition and Health Survey in Taiwan	National	Both	7-12	7-12	510	499	510	499	
1890	Taiwan (province of China)	2013-2016	Nutrition and Health Survey in Taiwan	National	Both	5+	5+	1,226	1,311	1,056	1,105	
1891	Tajikistan	2003	Micronutrient Status Survey	National	Both		15-49		1,179		355	
1892	Tajikistan	2012	DHS	National	Both		15-49		5,455		1,923	
1893	Tajikistan	2016	STEPS	National	Both	18-69	18-69	310	394	38	38	
1894	Tajikistan	2015-2016	Childhood Obesity Surveillance Initiative 4	National	Both	7	7	1,502	1,519	1,438	1,457	1
1895	Tajikistan	2017	DHS	National	Both		15-49		5,714		1,815	
1896	Tanzania	1991-1992	DHS	National	Both		20-49		2,649			
1897	Tanzania	1996	DHS	National	Both		20-49		2,191			
1898	Tanzania	1998-1999	Bovet et al., Int J Epidemiol 31(1):240-7, 2002	Community	Urban	25-64	25-64	1,176	2,459			
1899	Tanzania	2004-2005	DHS	National	Both		15-49		6,028		2,096	
1900	Tanzania	2009	Ilembula School study	Community	Rural	8-18	8-18	449	450	449	450	
1901	Tanzania	2010	DHS	National	Both		15-49		5,642		2,054	
1902	Tanzania	2011	STEPS	Subnational	Both	25-64	25-64	193	322			
1903	Tanzania	2012	STEPS	National	Both	25-64	25-64	412	569			
1904	Tanzania	2015-2016	DHS	National	Both		15-49		7,463		2,694	
1905	Thailand	1991	Thailand National Health Examination Survey I	National	Both	5+	5+	4,408	5,158	3,197	3,228	
1906	Thailand	1997	Thailand National Health Examination Survey II	National	Both	5-59	5-59	3,276	3,451	2,987	2,944	
1907	Thailand	2004	Thailand National Health Examination Survey III	National	Both	15+	15+	2,144	1,827	702	502	
1908	Thailand	2003-2004	The Fifth National Nutrition Survey of Thailand	National	Both	19+	19+	265	635			
1909	Thailand	2009	Thailand National Health Examination Survey IV	National	Both	5+	5+	4,619	4,579	3,874	3,839	
1910	Thailand	2011	SEANUTS	National	Both	5-12	5-12	922	939	922	939	
1911	Thailand	2015	Global School-based Student Health Survey	National	Both	12-17	12-17	2,240	3,106	2,240	3,106	
1912	The Gambia	2010	STEPS	National	Both	25-64	25-64	293	775			
1913	The Gambia	2013	DHS	National	Both		15-49		2,794		1,011	
1914	Timor-Leste	2009-2010	DHS	National	Both		15-49		7,262		3,065	
1915	Timor-Leste	2014	STEPS	National	Both	18-69	18-69	220	404	29	70	
1916	Timor-Leste	2016	DHS	National	Both	15-59	15-49	2,199	7,010	1,037	3,019	
1917	Togo	1998	DHS	National	Both		20-49		1,779			
1918	Togo	2010	STEPS	National	Both	15-64	15-64	841	1,001	245	248	
1919	Togo	2013-2014	DHS	National	Both		15-49		2,566		869	
1920	Togo	2014	Impact evaluation of a cash transfer program in North Togo	Subnational	Rural		20-65		1,959			
1921	Tokelau	2005	STEPS	National	Both	15-64	15-64	101	105	44	43	
1922	Tokelau	2014	STEPS	National	Both	18-64	18-64	88	85	19	16	
1923	Tonga	2004	STEPS	National	Both	15-64	15-64	95	107	27	24	
1924	Tonga	2005-2007	Pacific Obesity Prevention in Communities - Ma'alahi Youth Project	Subnational	Rural	11-19	11-19	1,206	1,445	1,206	1,445	
1925	Tonga	2007-2008	Pacific Obesity Prevention in Communities - Ma'alahi Youth Project	Subnational	Rural	13-22	13-22	437	584	407	529	
1926	Tonga	2010	Global School-based Student Health Survey	National	Both	13-17	13-17	927	1,069	926	1,069	
1927	Tonga	2011	STEPS	National	Both	15-64	15-64	78	175			

	Country	Study years	Survey/Study name/Citation	Level of representativeness	Rural, urban or both	Age range as in NCD-RisC database*		Sample size as in height analysis		Sample size as in BMI analysis		Note
						Male	Female	Male	Female	Male	Female	
1928	Tonga	2017	Global School-based Student Health Survey	National	Both	12-17	12-17	1,254	1,452	1,254	1,452	
1929	Trinidad and Tobago	1999	Child Health Survey	National	Both	5-9	5-9	3,064	3,273	3,060	3,272	
1930	Trinidad and Tobago	2001	Adult Survey	National	Rural	25+	25+	18	40			
1931	Trinidad and Tobago	2003	Child Health Survey	National	Both	5-9	5-9	1,833	1,978	1,832	1,974	
1932	Trinidad and Tobago	2003	National Survey of Senior School Health	National	Both	15-16	15-16	830	1,113	828	1,112	
1933	Tunisia	1996-1997	Tunisian National Nutrition Survey 1996-1997	National	Both	5+	5+	1,614	2,227	1,411	1,606	
1934	Tunisia	2005	Aounallah et al., Public Health 12(1):98, 2012	National	Both	15-19	15-19	1,290	1,566	1,290	1,566	
1935	Tunisia	2009-2010	ObeMaghreb	Subnational	Urban	5-49	5-49	1,148	1,510	911	1,001	
1936	Turkey	1993	DHS	National	Both		20-49		1,582			
1937	Turkey	1998	DHS	National	Both		20-49		1,493			
1938	Turkey	1998	Turkish Adult Risk Factor Study	National	Both	28+	28+	38	64			
1939	Turkey	2003	Prevalence, awareness, treatment and control of hypertension in Turkey in 2003	National	Both	18+	18+	526	784	102	129	
1940	Turkey	2003	DHS	National	Both		20-49		1,858			
1941	Turkey	2003-2005	Prevalence of prehypertension and associated risk factors among Turkish adults: Trabzon Hypertension Study	Subnational	Both	20+	20+	607	699			
1942	Turkey	2008	DHS	National	Both		15-49		2,095			
1943	Turkey	2011	Chronic Diseases and Risk Factors Survey in Turkey	National	Both	15+	15+	2,377	2,612	986	940	
1944	Turkey	2009-2012	Prevalence of diabetes and associated risk factors among adult population in Trabzon city	Subnational	Both	20+	20+	314	478			
1945	Turkey	2013	DHS	National	Both		15-49		3,937		1,367	
1946	Turkey	2013	Childhood Obesity Surveillance Initiative 3	National	Both	7-8	7-8	2,483	2,475	2,483	2,475	1
1947	Turkey	2015-2016	Childhood Obesity Surveillance Initiative 4	National	Both	6-7	6-7	5,480	5,336	5,479	5,336	1
1948	Turkey	2017	STEPS	National	Both	15+	15+	469	612	142	151	
1949	Turkey	2016-2017	Erasmus plus KA2, Healthyland	Community	Urban	6	6	29	22	29	22	
1950	Turkey	2017-2018	Erasmus plus KA2, Healthyland	Community	Urban	6	6	50	50	50	50	
1951	Turkmenistan	2000	DHS	National	Both		15-49		1,390			
1952	Turkmenistan	2013	STEPS	National	Both	18-64	18-64	559	767	51	121	
1953	Turkmenistan	2015-2016	Childhood Obesity Surveillance Initiative 4	National	Both	7-8	7-8	1,973	1,976	1,952	1,956	1
1954	Turkmenistan	2018	STEPS	National	Both	18-69	18-69	430	523	30	65	
1955	Tuvalu	1976	The Funafuti Survey	Subnational	Urban	10+	10+	10	5			
1956	Tuvalu	2013	Global School-based Student Health Survey	National	Both	13-17	13-17	211	215	210	215	
1957	Tuvalu	2015	STEPS	National	Both	18-69	18-69	134	132	22	17	
1958	Uganda	1995	DHS	National	Both		20-49		2,108			
1959	Uganda	2000-2001	DHS	National	Both		15-49		4,152		1,370	
1960	Uganda	2006	DHS	National	Both	15-54	15-49	1,300	1,727	565	589	
1961	Uganda	2011	DHS	National	Both	15-54	15-49	1,292	1,693	565	600	
1962	Uganda	2011-2013	Gulu Health and Demographic Surveillance Site (HDSS)	Community	Rural	5+	5+	2,847	3,295	2,328	2,392	
1963	Uganda	2011-2012	The Prevalence and Distribution of Non-communicable Diseases and Their Risk Factors in Kasese District, Uganda	Subnational	Both	25-79	25-79	50	40			
1964	Uganda	2014	STEPS	National	Both	18-69	18-69	641	793	124	160	
1965	Uganda	2014-2015	Gulu Health and Demographic Surveillance Site (HDSS)	Community	Rural	15-24	15-24	671	559	448	331	
1966	Uganda	2016	DHS	National	Both	15-54	15-54	2,880	3,625	1,241	1,285	
1967	Ukraine	2013-2014	The prevalence of underweight, overweight and obesity in children and adolescents from Ukraine	National	Both	6-18	6-18	6,596	7,143	6,596	7,143	
1968	Ukraine	2019	STEPS	National	Both	18-69	18-69	256	299	28	41	
1969	United Arab Emirates	2005	Global School-based Student Health Survey	National	Both	12-15	12-15	5,595	6,268	5,595	6,268	
1970	United Arab Emirates	2009	Gulf Cooperation Council World Health Survey	National	Both	18+	18+	148	182	31	24	
1971	United Arab Emirates	2010	Global School-based Student Health Survey	National	Both	13-17	13-17	948	1,257	948	1,257	
1972	United Arab Emirates	2016	Global School-based Student Health Survey	National	Both	12-17	12-17	2,323	2,675	2,323	2,674	
1973	United Arab Emirates	2017-2018	STEPS	National	Both	18+	18+	368	514	36	43	
1974	United Kingdom	1975	British Cohort Study 1970	National	Both	5	5	6,674	6,218			
1975	United Kingdom	1980	British Cohort Study 1970	National	Both	10	10	6,557	6,170			
1976	United Kingdom	1986	British Cohort Study 1970	National	Both	16	16	2,808	2,995	2,773	2,950	
1977	United Kingdom	1986-1987	Dietary and Nutritional Survey of British Adults 1986-1987	National	Both	16-64	16-64	112	85	111	84	
1978	United Kingdom	1991-1992	Health Survey for England	National	Both	16+	16+	792	891	201	215	
1979	United Kingdom	1993	Health Survey for England	National	Both	16+	16+	1,764	1,963	441	442	
1980	United Kingdom	1994	Health Survey for England	National	Both	16+	16+	1,623	1,907	375	388	
1981	United Kingdom	1995	Health Survey for England	National	Both	5+	5+	2,860	3,190	1,689	1,711	
1982	United Kingdom	1995	Scottish Health Survey (SHeS)	Subnational	Both	16-64	16-64	838	1,056	176	170	

	Country	Study years	Survey/Study name/Citation	Level of representativeness	Rural, urban or both	Age range as in NCD-RisC database*		Sample size as in height analysis		Sample size as in BMI analysis		Note
						Male	Female	Male	Female	Male	Female	
1983	United Kingdom	1995	MONICA, Glasgow	Community	Urban	25-64	25-64	79	113			
1984	United Kingdom	1996	Health Survey for England	National	Both	5+	5+	3,031	3,297	1,904	1,840	
1985	United Kingdom	1996	British Cohort Study 1970	National	Both	26	26	2,550	4,802			
1986	United Kingdom	1997	Health Survey for England	National	Both	5+	5+	3,463	3,592	2,815	2,811	
1987	United Kingdom	1997	National Diet and Nutrition Survey (NDNS)	National	Both	5-18	5-18	934	899	933	896	
1988	United Kingdom	1993-2000	EPIC Oxford	Subnational	Both	20-98	20-98	1,393	7,220			
1989	United Kingdom	1998	Health Survey for England	National	Both	5+	5+	2,851	3,082	1,775	1,738	
1990	United Kingdom	1998	Scottish Health Survey (SHeS)	Subnational	Both	5-74	5-74	2,191	2,329	1,613	1,551	
1991	United Kingdom	1999	Health Survey for England	National	Both	5+	5+	1,353	1,403	872	849	
1992	United Kingdom	1998-1999	SportsLinx	Community	Urban	9-10	9-10	1,433	1,369	1,429	1,364	
1993	United Kingdom	2000	Health Survey for England	National	Both	5+	5+	1,400	1,426	903	851	
1994	United Kingdom	1999-2000	SportsLinx	Community	Urban	9-10	9-10	1,504	1,468	1,469	1,439	
1995	United Kingdom	2001	Health Survey for England	National	Both	5+	5+	2,461	2,832	1,538	1,626	
1996	United Kingdom	2000-2001	National Diet and Nutrition Survey (NDNS)	National	Both	19-64	19-64	140	165	9	9	
1997	United Kingdom	2000-2001	SportsLinx	Community	Urban	9-10	9-10	1,175	1,159	1,166	1,154	
1998	United Kingdom	2002	Health Survey for England	National	Both	5+	5+	4,533	4,798	3,469	3,376	
1999	United Kingdom	2001-2002	SportsLinx	Community	Urban	9-10	9-10	867	743	866	743	
2000	United Kingdom	2003	Health Survey for England	National	Both	5+	5+	2,300	2,577	1,488	1,559	
2001	United Kingdom	2003	Scottish Health Survey (SHeS)	Subnational	Both	5+	5+	1,495	1,631	1,135	1,156	
2002	United Kingdom	2002-2003	SportsLinx	Community	Urban	9-10	9-10	728	754	725	749	
2003	United Kingdom	2004	Health Survey for England	National	Both	5+	5+	967	1,028	656	613	
2004	United Kingdom	2003-2004	SportsLinx	Community	Urban	9-10	9-10	1,907	1,931	1,906	1,931	
2005	United Kingdom	2005	Health Survey for England	National	Both	5+	5+	1,547	1,691	1,140	1,138	
2006	United Kingdom	2004-2005	SportsLinx	Community	Urban	9-10	9-10	1,736	1,739	1,724	1,712	
2007	United Kingdom	2006	Health Survey for England	National	Both	5+	5+	3,438	3,666	2,777	2,739	
2008	United Kingdom	2006	Millennium Cohort Study	National	Both	5-6	5-6	6,265	6,006	6,258	5,999	
2009	United Kingdom	2005-2006	SportsLinx	Community	Urban	9-10	9-10	1,460	1,421	1,455	1,409	
2010	United Kingdom	2007	Health Survey for England	National	Both	5+	5+	3,185	3,153	2,771	2,639	
2011	United Kingdom	2006-2007	National Child Measurement Programme	National	Both	5-11	5-11	380,595	358,287	380,586	358,276	
2012	United Kingdom	2006-2007	SportsLinx	Community	Urban	9-10	9-10	1,761	1,688	1,759	1,683	
2013	United Kingdom	2007	Welsh Health Survey (WHS)	Subnational	Both	5-15	5-15	788	762	782	751	
2014	United Kingdom	2008	Health Survey for England	National	Both	5+	5+	3,617	3,933	2,801	2,849	
2015	United Kingdom	2008	Millennium Cohort Study	National	Both	6-8	6-8	7,005	6,859	6,961	6,836	
2016	United Kingdom	2007-2008	National Child Measurement Programme	National	Both	5-11	5-11	396,801	374,787	396,795	374,775	
2017	United Kingdom	2008	Scottish Health Survey (SHeS)	Subnational	Both	5+	5+	883	1,024	620	632	
2018	United Kingdom	2007-2008	SportsLinx	Community	Urban	9-10	9-10	1,855	1,821	1,852	1,815	
2019	United Kingdom	2008	Welsh Health Survey (WHS)	Subnational	Both	5-15	5-15	741	635	737	624	
2020	United Kingdom	2009	Health Survey for England	National	Both	5+	5+	1,733	1,669	1,505	1,374	
2021	United Kingdom	2008-2009	National Child Measurement Programme	National	Both	5-11	5-11	398,497	377,149	398,496	377,146	
2022	United Kingdom	2009	Scottish Health Survey (SHeS)	Subnational	Both	5+	5+	1,237	1,307	918	843	
2023	United Kingdom	2008-2009	SportsLinx	Community	Urban	9-10	9-10	1,837	1,864	1,824	1,854	
2024	United Kingdom	2009	Welsh Health Survey (WHS)	Subnational	Both	5-15	5-15	912	865	907	857	
2025	United Kingdom	2010	Health Survey for England	National	Both	5+	5+	2,396	2,469	1,980	1,875	
2026	United Kingdom	2009-2010	National Child Measurement Programme	National	Both	5-11	5-11	398,272	377,962	398,272	377,960	
2027	United Kingdom	2008-2012	National Diet and Nutrition Survey (NDNS)	National	Both	5+	5+	1,451	1,534	1,272	1,259	
2028	United Kingdom	2010	Scottish Health Survey (SHeS)	Subnational	Both	5+	5+	976	1,051	639	575	
2029	United Kingdom	2009-2010	SportsLinx	Community	Urban	9-10	9-10	1,497	1,449	1,493	1,429	
2030	United Kingdom	2010	Welsh Health Survey (WHS)	Subnational	Both	5-15	5-15	901	870	892	859	
2031	United Kingdom	2011	Health Survey for England	National	Both	5+	5+	1,045	1,283	640	713	
2032	United Kingdom	2010-2011	National Child Measurement Programme	National	Both	5-11	5-11	393,149	373,473	393,146	373,472	
2033	United Kingdom	2011	Scottish Health Survey (SHeS)	Subnational	Both	5+	5+	955	1,043	618	601	
2034	United Kingdom	2010-2011	SportsLinx	Community	Urban	9-10	9-10	1,338	1,261	1,332	1,252	
2035	United Kingdom	2011	Welsh Health Survey (WHS)	Subnational	Both	5-15	5-15	953	847	949	836	
2036	United Kingdom	2012	Health Survey for England	National	Both	5+	5+	1,069	1,237	695	672	
2037	United Kingdom	2011-2013	International Study of Childhood Obesity, Lifestyle and the Environment (ISCOLE)	Community	Urban	9-11	9-11	237	288	237	287	
2038	United Kingdom	2012	Millennium Cohort Study	National	Both	10-12	10-12	6,600	6,479	6,574	6,451	
2039	United Kingdom	2011-2012	National Child Measurement Programme	National	Both	5-11	5-11	390,385	373,068	390,383	373,067	

	Country	Study years	Survey/Study name/Citation	Level of representativeness	Rural, urban or both	Age range as in NCD-RisC database*		Sample size as in height analysis		Sample size as in BMI analysis		Note
						Male	Female	Male	Female	Male	Female	
2040	United Kingdom	2012	Scottish Health Survey (SHeS)	Subnational	Both	5+	5+	778	858	569	590	
2041	United Kingdom	2011-2012	SportsLinx	Community	Urban	9-10	9-10	1,334	1,378	1,286	1,330	
2042	United Kingdom	2012	Welsh Health Survey (WHS)	Subnational	Both	5-15	5-15	808	721	804	712	
2043	United Kingdom	2013	Health Survey for England	National	Both	5+	5+	1,118	1,307	737	758	
2044	United Kingdom	2012-2013	National Child Measurement Programme	National	Both	5-11	5-11	396,704	377,113	396,700	377,109	
2045	United Kingdom	2013	Scottish Health Survey (SHeS)	Subnational	Both	5+	5+	830	837	594	544	
2046	United Kingdom	2014	Health Survey for England	National	Both	5+	5+	1,084	1,186	717	711	
2047	United Kingdom	2013-2014	National Child Measurement Programme	National	Both	5-11	5-11	407,634	389,424	407,631	389,421	
2048	United Kingdom	2013-2014	National Diet and Nutrition Survey (NDNS)	National	Both	5+	5+	525	574	469	464	
2049	United Kingdom	2014	Scottish Health Survey (SHeS)	Subnational	Both	5+	5+	761	822	558	563	
2050	United Kingdom	2013-2014	Swan-Linx Project	Community	Both	9-11	9-11	335	348	329	333	
2051	United Kingdom	2015	Health Survey for England	National	Both	5+	5+	2,239	2,436	1,896	1,915	
2052	United Kingdom	2015	Millennium Cohort Study	National	Both	13-15	13-15	5,705	5,703	5,635	5,474	
2053	United Kingdom	2014-2015	National Child Measurement Programme	National	Both	5-11	5-11	412,685	395,006	412,685	395,006	
2054	United Kingdom	2015	Scottish Health Survey (SHeS)	Subnational	Both	5+	5+	679	671	459	397	
2055	United Kingdom	2015	Swan-Linx Project	Community	Both	9-11	9-11	421	403	420	395	
2056	United Kingdom	2016	Health Survey for England	National	Both	5+	5+	1,005	1,179	642	668	
2057	United Kingdom	2015-2016	National Child Measurement Programme	National	Both	5-11	5-11	427,039	410,085	427,039	410,085	
2058	United Kingdom	2015-2016	National Diet and Nutrition Survey (NDNS)	National	Both	5+	5+	549	580	490	456	
2059	United Kingdom	2016	Scottish Health Survey (SHeS)	Subnational	Both	5+	5+	683	714	494	477	
2060	United Kingdom	2016	Swan-Linx Project	Community	Both	9-11	9-11	606	661	604	653	
2061	United Kingdom	2017	Health Survey for England	National	Both	5+	5+	927	1,074	601	643	
2062	United Kingdom	2016-2017	National Child Measurement Programme	National	Both	5-11	5-11	430,101	412,604	430,101	412,604	
2063	United Kingdom	2016-2017	National Diet and Nutrition Survey (NDNS)	National	Both	5+	5+	255	267	224	215	
2064	United Kingdom	2017	Scottish Health Survey	Subnational	Both	5+	5+	672	641	499	433	
2065	United Kingdom	2017-2018	National Child Measurement Programme	National	Both	5-11	5-11	444,135	426,632	444,135	426,632	
2066	United States of America	1971-1975	US NHANES I	National	Both	5-74	5-74	616	659			
2067	United States of America	1973-1974	The Bogalusa Heart Study	Community	Rural	5-19	5-19	389	365			
2068	United States of America	1976-1977	The Bogalusa Heart Study	Community	Rural	5-25	5-25	1,089	1,047			
2069	United States of America	1976-1980	US NHANES II	National	Both	5-74	5-74	137	136			
2070	United States of America	1978-1979	The Bogalusa Heart Study	Community	Rural	5-22	5-22	1,320	1,305			
2071	United States of America	1981-1982	The Bogalusa Heart Study	Community	Rural	5-22	5-22	1,554	1,575			
2072	United States of America	1983-1985	The Bogalusa Heart Study	Community	Rural	6-22	6-22	1,265	1,323			
2073	United States of America	1985-1986	Coronary Artery Risk Development in Young Adults (CARDIA)	Subnational	Urban	18-30	18-30	241	313	241	312	
2074	United States of America	1987-1988	The Bogalusa Heart Study	Community	Rural	5-22	5-22	1,687	1,623	1,679	1,612	
2075	United States of America	1988-1994	US NHANES III	National	Both	5+	5+	5,301	5,733	3,634	3,775	
2076	United States of America	1992-1994	The Bogalusa Heart Study	Community	Rural	5-21	5-21	1,581	1,627	1,576	1,627	
2077	United States of America	1992-1993	Coronary Artery Risk Development in Young Adults (CARDIA)	Subnational	Urban	25-37	25-37	505	568			
2078	United States of America	1996	National Longitudinal Study of Adolescent Health Wave II	National	Both	11-21	11-21	2,299	2,499	2,259	2,437	5
2079	United States of America	1995-1996	Coronary Artery Risk Development in Young Adults (CARDIA)	Subnational	Urban	28-40	28-40	151	219			
2080	United States of America	1998-1999	Coronary Artery Risk Detection in Appalachian Communities (CARDIAC), 5th Grade	Subnational	Both	10-12	10-12	541	462	541	461	
2081	United States of America	2000-2001	Coronary Artery Risk Detection in Appalachian Communities (CARDIAC), 5th Grade	Subnational	Both	10-12	10-12	639	569	639	569	
2082	United States of America	1999-2000	US NHANES 1999-2000	National	Both	5+	5+	2,090	2,210	1,766	1,714	
2083	United States of America	2001	Coronary Artery Risk Detection in Appalachian Communities (CARDIAC), 5th Grade	Subnational	Both	10-12	10-12	1,732	1,635	1,732	1,631	
2084	United States of America	2001-2002	National Longitudinal Study of Adolescent Health Wave III	National	Both	18-28	18-28	2,178	2,521	227	318	5
2085	United States of America	2002-2003	Coronary Artery Risk Detection in Appalachian Communities (CARDIAC), 5th Grade	Subnational	Both	10-12	10-12	2,643	2,597	2,635	2,594	
2086	United States of America	2001-2002	US NHANES 2001-2002	National	Both	5+	5+	2,281	2,470	1,826	1,812	
2087	United States of America	2003-2004	Coronary Artery Risk Detection in Appalachian Communities (CARDIAC), 5th Grade	Subnational	Both	10-12	10-12	4,589	4,069	4,581	4,061	
2088	United States of America	2004-2005	Coronary Artery Risk Detection in Appalachian Communities (CARDIAC), 5th Grade	Subnational	Both	10-12	10-12	4,350	4,281	4,340	4,277	
2089	United States of America	2003-2004	US NHANES 2003-2004	National	Both	5+	5+	2,108	2,157	1,701	1,644	
2090	United States of America	2005-2006	Coronary Artery Risk Detection in Appalachian Communities (CARDIAC), 2nd Grade	Subnational	Both	7-9	7-9	328	271	327	270	
2091	United States of America	2005-2006	Coronary Artery Risk Detection in Appalachian Communities (CARDIAC), 5th Grade	Subnational	Both	10-12	10-12	4,867	4,212	4,859	4,205	
2092	United States of America	2006-2007	Coronary Artery Risk Detection in Appalachian Communities (CARDIAC), 2nd Grade	Subnational	Both	7-9	7-9	845	799	843	798	
2093	United States of America	2006-2007	Coronary Artery Risk Detection in Appalachian Communities (CARDIAC), 5th Grade	Subnational	Both	10-12	10-12	4,045	3,424	4,039	3,418	
2094	United States of America	2005-2006	US NHANES 2005-2006	National	Both	5+	5+	2,154	2,368	1,748	1,716	
2095	United States of America	2007-2008	Coronary Artery Risk Detection in Appalachian Communities (CARDIAC), 2nd Grade	Subnational	Both	7-9	7-9	3,873	3,977	3,873	3,975	
2096	United States of America	2007-2008	Coronary Artery Risk Detection in Appalachian Communities (CARDIAC), 5th Grade	Subnational	Both	10-12	10-12	4,050	3,638	4,046	3,634	

	Country	Study years	Survey/Study name/Citation	Level of representativeness	Rural, urban or both	Age range as in NCD-RisC database*		Sample size as in height analysis		Sample size as in BMI analysis		Note
						Male	Female	Male	Female	Male	Female	
2097	United States of America	2008-2009	Coronary Artery Risk Detection in Appalachian Communities (CARDIAC), 2nd Grade	Subnational	Both	7-9	7-9	4,919	5,187	4,907	5,178	
2098	United States of America	2008-2009	Coronary Artery Risk Detection in Appalachian Communities (CARDIAC), 5th Grade	Subnational	Both	10-12	10-12	4,153	3,595	4,152	3,593	
2099	United States of America	2007-2008	US NHANES 2007-2008	National	Both	5+	5+	1,765	1,666	1,340	1,234	
2100	United States of America	2009-2010	Coronary Artery Risk Detection in Appalachian Communities (CARDIAC), 2nd Grade	Subnational	Both	7-9	7-9	5,224	5,567	5,217	5,562	
2101	United States of America	2009-2010	Coronary Artery Risk Detection in Appalachian Communities (CARDIAC), 5th Grade	Subnational	Both	10-12	10-12	3,992	3,515	3,990	3,513	
2102	United States of America	2008-2009	National Longitudinal Study of Adolescent Health Wave IV	National	Both	24-34	24-34	1,462	1,808			5
2103	United States of America	2010-2011	Coronary Artery Risk Detection in Appalachian Communities (CARDIAC), 2nd Grade	Subnational	Both	7-9	7-9	4,995	5,288	4,994	5,286	
2104	United States of America	2010-2011	Coronary Artery Risk Detection in Appalachian Communities (CARDIAC), 5th Grade	Subnational	Both	10-12	10-12	3,316	2,819	3,313	2,819	
2105	United States of America	2009-2010	US NHANES 2009-2010	National	Both	5+	5+	1,872	1,845	1,402	1,297	
2106	United States of America	2011-2012	Coronary Artery Risk Detection in Appalachian Communities (CARDIAC), 2nd Grade	Subnational	Both	7-9	7-9	4,124	4,415	4,122	4,412	
2107	United States of America	2011-2012	Coronary Artery Risk Detection in Appalachian Communities (CARDIAC), 5th Grade	Subnational	Both	10-12	10-12	2,646	2,115	2,644	2,112	
2108	United States of America	2012-2013	Coronary Artery Risk Detection in Appalachian Communities (CARDIAC), 2nd Grade	Subnational	Both	7-9	7-9	4,569	5,192	4,565	5,184	
2109	United States of America	2012-2013	Coronary Artery Risk Detection in Appalachian Communities (CARDIAC), 5th Grade	Subnational	Both	10-12	10-12	2,796	2,332	2,796	2,332	
2110	United States of America	2011-2013	International Study of Childhood Obesity, Lifestyle and the Environment (ISCOLE)	Community	Urban	9-11	9-11	281	370	281	368	
2111	United States of America	2011-2012	US NHANES 2011-2012	National	Both	5+	5+	1,854	1,757	1,366	1,292	
2112	United States of America	2013-2014	Coronary Artery Risk Detection in Appalachian Communities (CARDIAC), 2nd Grade	Subnational	Both	7-9	7-9	4,998	5,421	4,994	5,415	
2113	United States of America	2013-2014	Coronary Artery Risk Detection in Appalachian Communities (CARDIAC), 5th Grade	Subnational	Both	10-12	10-12	2,409	1,987	2,408	1,987	
2114	United States of America	2014-2015	Coronary Artery Risk Detection in Appalachian Communities (CARDIAC), 2nd Grade	Subnational	Both	7-9	7-9	5,184	5,497	5,182	5,495	
2115	United States of America	2014-2015	Coronary Artery Risk Detection in Appalachian Communities (CARDIAC), 5th Grade	Subnational	Both	10-12	10-12	2,286	1,889	2,286	1,889	
2116	United States of America	2013-2014	US NHANES 2013-2014	National	Both	5+	5+	1,917	1,875	1,467	1,409	
2117	United States of America	2015-2016	US NHANES 2015-2016	National	Both	5+	5+	1,806	1,831	1,376	1,337	
2118	United States of America	2015-2016	National Social Life Health and Aging Project	Community	Both	24-99	24-99		2			6
2119	Uruguay	2004	CUiiDARTE Project (Adolescent)	National	Urban	5-8	5-8	129	149	129	149	
2120	Uruguay	2005	CUiiDARTE Project (Adolescent)	National	Urban	6-10	6-10	105	105	105	105	
2121	Uruguay	2006	STEPS	National	Both	25-64	25-64	33	73			
2122	Uruguay	2009-2010	CUiiDARTE Project (Adolescent)	National	Urban	10-14	10-14	117	130	117	129	
2123	Uruguay	2012	Global School-based Student Health Survey	National	Both		13-15		1,377		1,377	
2124	Uruguay	2013	STEPS	National	Urban	15-64	15-64	212	317	75	92	
2125	Uruguay	2012-2016	Genotype, Phenotype and Environment of Hypertension in Uruguay (GEFA-HT-UY)	Community	Urban	19+	19+	12	13			
2126	Uruguay	2015	CUiiDARTE Project (Adolescent)	National	Urban	17-20	17-20	135	142	135	142	
2127	Uruguay	2016-2017	CUiiDARTE Project (Children)	Community	Urban	5-6	5-6	395	375	395	375	
2128	Uzbekistan	1996	DHS	National	Both		15-49		2,440		898	
2129	Uzbekistan	2002	DHS	National	Both	15-59	15-49	1,165	3,008	418	1,104	
2130	Uzbekistan	2014	STEPS	National	Both	18-64	18-64	461	582	83	75	
2131	Uzbekistan	2019	STEPS	National	Both	18-69	18-69	174	258	12	25	
2132	Vanuatu	2005	STEPS	Subnational	Both	15-60	15-60	316	382	104	122	
2133	Vanuatu	2011	STEPS	National	Both	25-64	25-64	384	431			
2134	Venezuela	2004-2005	Cardiovascular Risk Factors Multiple Evaluation in Latin America	Community	Urban	25-64	25-64	178	224			
2135	Venezuela	2005-2006	Brajkovich et al., Rev Ven Endoc Metab 4(3):31-32, 2006	Community	Urban	20-65	20-65	22	73			
2136	Venezuela	2007-2008	Venezuelan Study of Metabolic Syndrome, Obesity and Lifestyle (VEMSOLS)	Community	Urban	20+	20+	20	27			
2137	Venezuela	2008-2009	Venezuelan Study of Metabolic Syndrome, Obesity and Lifestyle (VEMSOLS)	Community	Rural	20+	20+	16	29			
2138	Venezuela	2010-2011	Venezuelan Study of Metabolic Syndrome, Obesity and Lifestyle (VEMSOLS)	Community	Urban	20+	20+	15	35			
2139	Venezuela	2015-2017	Cardio-Metabolic Health Venezuelan Study (EVESCAM)	National	Both	20+	20+	126	302			
2140	Viet Nam	1987-1989	General Nutrition Survey	National	Both	15-70	15-70			2,236	2,303	
2141	Viet Nam	1992-1993	Living Standard Survey	National	Both	5+	5+	5,784	6,054	4,172	4,107	
2142	Viet Nam	1997-1998	Living Standard Survey	National	Both	5+	5+	6,954	6,866	5,059	4,908	
2143	Viet Nam	2000	National Nutrition Survey	National	Both	20+	20+	2,455	2,347			
2144	Viet Nam	2001-2002	Viet Nam National Health Survey 2001-2002	National	Both	5+	5+	36,636	36,661	27,137	25,748	
2145	Viet Nam	2001-2003	The National Epidemiological Survey on Hypertension and its Risk Factors (North)	Subnational	Both	25-74	25-74	265	427			
2146	Viet Nam	2003-2004	The Survey on Heart Failure and its Risk Factors	Subnational	Both	25-74	25-74	195	291			
2147	Viet Nam	2004	The Hypertension Management Programme in Rural Communes (Hanoi)	Community	Rural	25-74	25-74	61	85			
2148	Viet Nam	2005	The Survey on Non-Communicable Disease Risk Factors	Subnational	Both	25-74	25-74	105	104			
2149	Viet Nam	2005	National Adult Overweight Survey	National	Both	25-64	25-64	1,038	1,039			
2150	Viet Nam	2005	Non-communicable disease risk factors in Ho Chi Minh City	Community	Urban	25-64	25-64	110	101			
2151	Viet Nam	2006	Qualitative and quantitative assessment of nutritional status and lifestyles of Vietnamese adolescents, rural	Subnational	Rural	15-17	15-17	252	363	252	363	

	Country	Study years	Survey/Study name/Citation	Level of representativeness	Rural, urban or both	Age range as in NCD-RisC database*		Sample size as in height analysis		Sample size as in BMI analysis		Note
						Male	Female	Male	Female	Male	Female	
2152	Viet Nam	2006	Qualitative and quantitative assessment of nutritional status and lifestyles of Vietnamese adolescents, urban	Subnational	Urban	15-19	15-19	254	334	254	334	
2153	Viet Nam	2006	The Hypertension Management Programme in Rural Communes (Bavi)	Community	Rural	25-74	25-74	17	45			
2154	Viet Nam	2007	The Hypertension Management Programme in Rural Communes (Phu Phuong)	Community	Rural	25-74	25-74	15	52			
2155	Viet Nam	2006-2008	The National Epidemiological Survey on Hypertension and its Risk Factors (South)	Subnational	Both	25-74	25-74	115	177			
2156	Viet Nam	2009	STEPS	National	Both	25-64	25-64	636	834			
2157	Viet Nam	2008-2009	The Survey on Diabetes and its Risk Factors	Subnational	Both	25+	25+	44	83			
2158	Viet Nam	2009	The Hypertension Management Programme in Rural Communes (Phu Cuong)	Community	Rural	25-74	25-74	9	42			
2159	Viet Nam	2009-2010	Vietnam National Nutrition Survey 2009-2010	National	Both	5+	5+	8,067	8,444	5,210	5,160	
2160	Viet Nam	2011	SEANUTS	National	Both	5-11	5-11	975	980	975	980	
2161	Viet Nam	2013	Global School-based Student Health Survey	National	Both	13-17	13-17	1,368	1,578	1,368	1,578	
2162	Viet Nam	2015	STEPS	National	Both	18-69	18-69	220	252	26	20	
2163	Viet Nam	2019	Global School-based Student Health Survey	National	Both	13-18	13-18	3,572	4,118	3,572	4,117	
2164	Yemen	1997	DHS	National	Both		15-49		3,240			
2165	Yemen	2005-2006	Yemen Household Budget Survey 2005-2006	National	Both	5+	5+	2,451	2,386	1,914	1,733	
2166	Yemen	2007-2009	Hypertension and Diabetes in Yemen (HYDY)	National	Rural	6-70	6-70	1,325	1,356	795	787	
2167	Yemen	2007-2009	Hypertension and Diabetes in Yemen (HYDY)	National	Urban	6-70	6-70	1,307	1,349	763	759	
2168	Yemen	2013	DHS	National	Both		15-49		15,621		5,930	
2169	Zambia	1992	DHS	National	Both		20-49		1,963			
2170	Zambia	1996	DHS	National	Both		20-49		2,394			
2171	Zambia	2001-2002	DHS	National	Both		15-49		4,739		1,616	
2172	Zambia	2007	DHS	National	Both		15-49		4,407		1,447	
2173	Zambia	2008	STEPS	Subnational	Urban	25+	25+	207	412			
2174	Zambia	2013-2014	DHS	National	Both		15-49		9,429		3,401	
2175	Zambia	2017	STEPS	National	Both	18-69	18-69	584	883	109	207	
2176	Zimbabwe	1991	Zinyowera et al., Cent Afr J Med 40(2):33-8, 1994	Community	Both	18+	18+			119	113	
2177	Zimbabwe	1994	DHS	National	Both		20-49		1,151			
2178	Zimbabwe	1999	DHS	National	Both		15-49		3,533		1,315	
2179	Zimbabwe	2005-2006	DHS	National	Both		15-49		5,413		1,962	
2180	Zimbabwe	2010-2011	DHS	National	Both	15-54	15-49	4,312	5,408	1,812	1,818	
2181	Zimbabwe	2015	DHS	National	Both	15-54	15-49	4,597	5,633	2,051	2,049	

*As described in Methods section, participants aged 5-19 years were used in the BMI analysis and participants aged 5-29 years were used in the height analysis.

1. We thank Jelena Jakovljevic, Henrique Loyola, Harry Rutter, Joop van Raaij, Stephen Whiting and Ivo Rakovac for data from the COSI Project.

2. This research uses data from Australia Health Survey (AHS). We thank the Health Section, Australian Bureau of Statistics, Belconnen, ACT, Australia for support for AHS 2011-2012.

3. National studies for the 3 years prior to 1985 were assigned to 1985 in the BMI analysis so that they can inform the estimates in countries with slightly earlier national data.

4. This research uses data from China Health and Nutrition Survey (CHNS). We thank the National Institute of Nutrition and Food Safety, China Center for Disease Control and Prevention, Carolina Population Center (5 R24 HD050924), the University of North Carolina at Chapel Hill, the NIH (R01-HD30880, DK056350, R24HD050924, and R01-HD38700) and the Fogarty International Center, NIH for financial support for the CHNS data collection and analysis files from 1989 to 2011 and future surveys, and the China-Japan Friendship Hospital, Ministry of Health for support for CHNS 2009.

5. This research uses data from Add Health, a program project designed by J. Richard Udry, Peter S. Bearman, and Kathleen Mullan Harris, and funded by a grant P01-HD31921 from the Eunice Kennedy Shriver National Institute of Child Health and Human Development, with cooperative funding from 17 other agencies. Special acknowledgment is due Ronald R. Rindfuss and Barbara Entwisle for assistance in the original design. Persons interested in obtaining data files from Add Health should contact Add Health, Carolina Population Center, 123 W. Franklin Street, Chapel Hill, NC 27516-2524 (addhealth@unc.edu). No direct support was received from grant P01-HD31921 for this analysis.

6. The bibliographic citation for this data source is: Waite, Linda J., Kathleen Cagney, William Dale, Elbert Huang, Edward O. Laumann, Martha McClintock, Colm A. O'Muircheartaigh, L. Phillip Schumm, and Benjamin Cornwell. National Social Life, Health, and Aging Project (NSHAP): Wave 2 and Partner Data Collection. ICPSR34921-v1. Ann Arbor, MI: Inter-university Consortium for Political and Social Research [distributor], 2014-04-29. https://doi.org/10.3886/ICPSR34921.v1

Appendix Figure 1. Median height of the WHO reference population[16] and median height of all countries in the world for children born in the year 2000, by age.

The WHO growth reference was constructed by merging the original sample from 5 to 19 years from the 1977 NCHS/WHO growth reference (a non-obese sample with expected heights), supplemented with data from the WHO Child Growth Standards to smooth the transition between the two samples.[16]

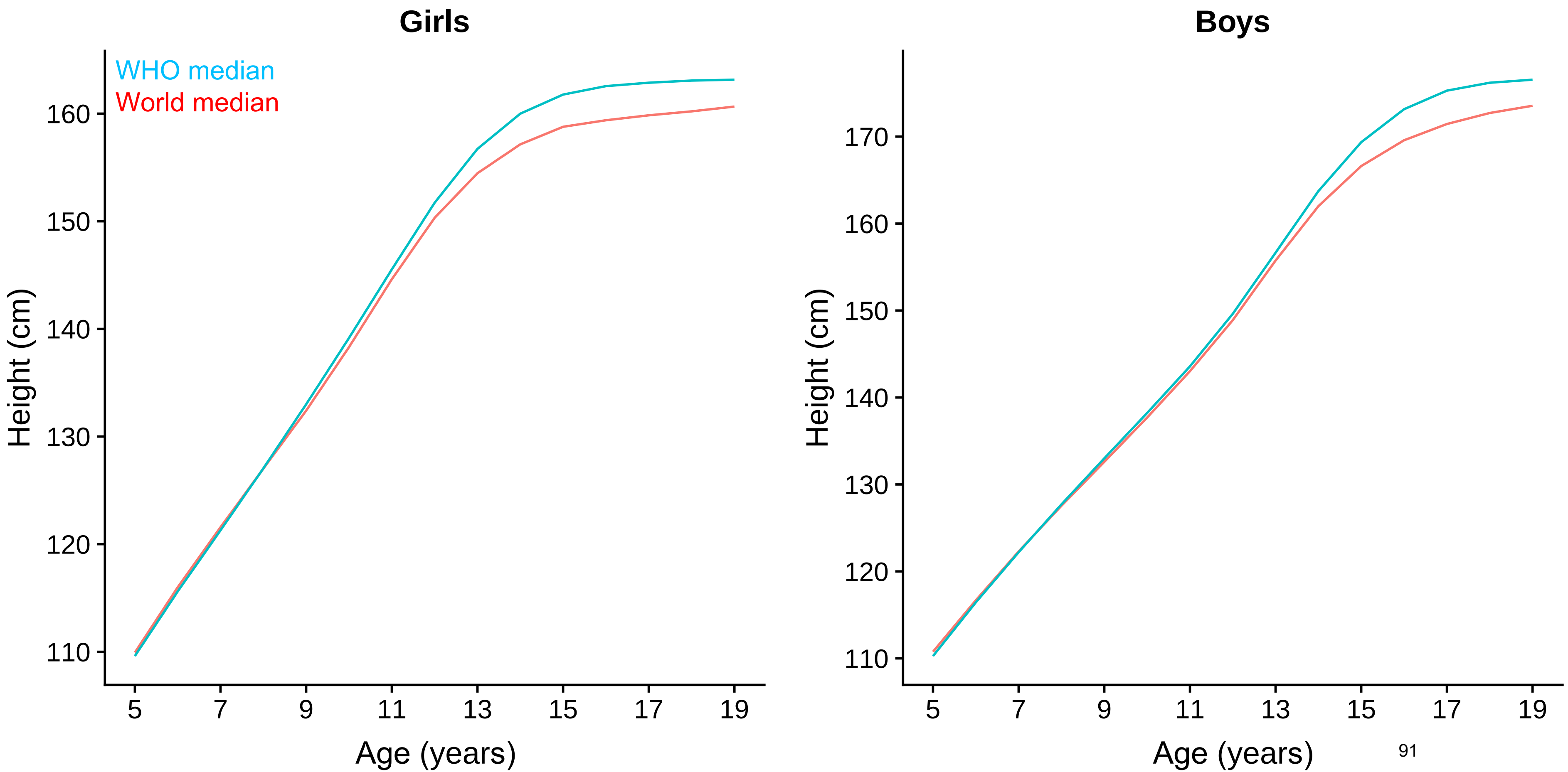
Girls
Boys
WHO median
World median
Height (cm)
Age (years)
110
120
130
140
150
160
170
5
7
9
11
13
15
17
19

Appendix Figure 2. Median body-mass index (BMI) of the WHO reference population[16] and median BMI of all countries in the world for children born in the year 2000, by age.

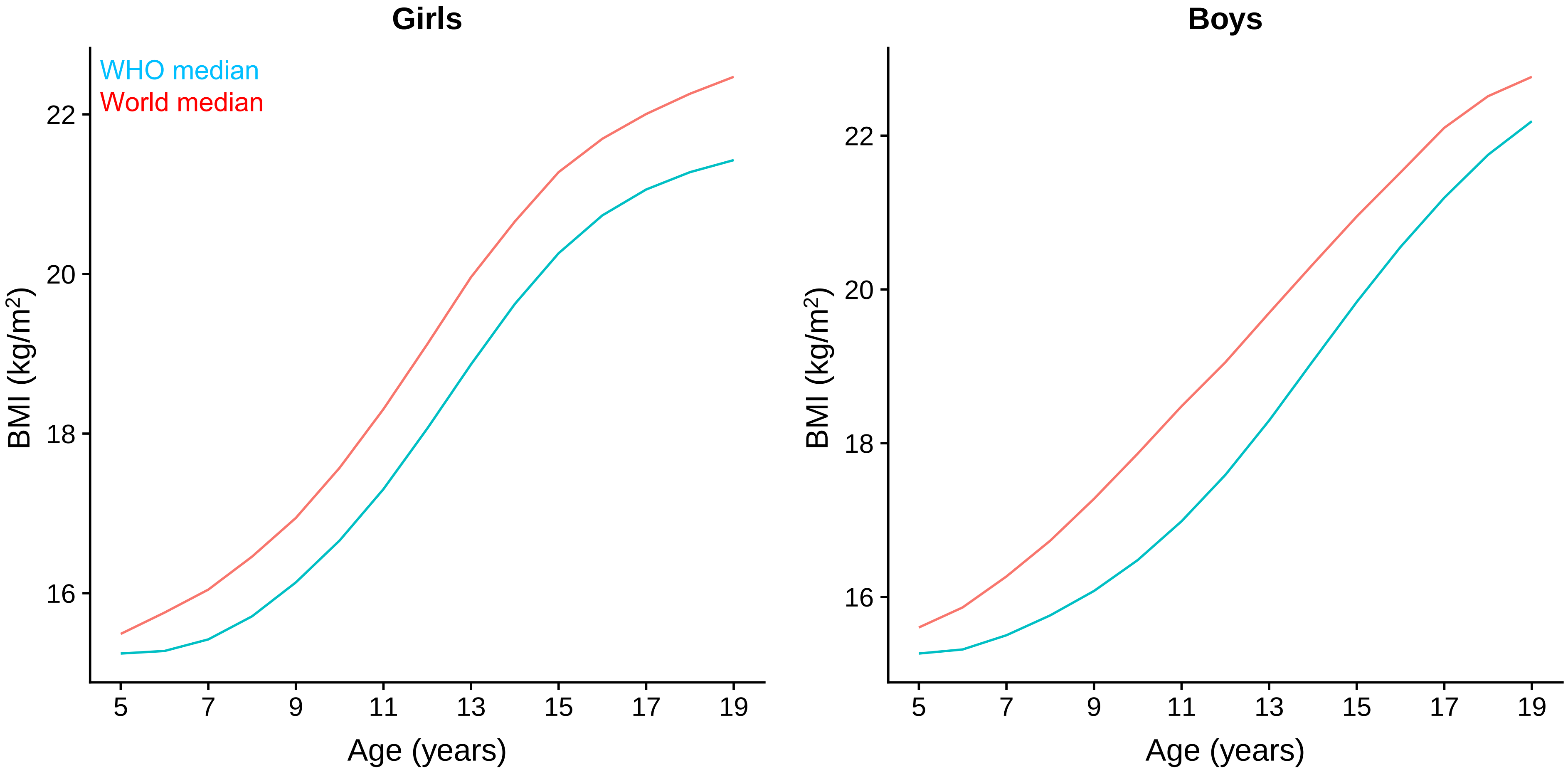
Girls
Boys
WHO median
World median
BMI (kg/m²)
Age (years)
16
18
20
22
5
7
9
11
13
15
17
19

Appendix Figure 3. Number of data sources by country.

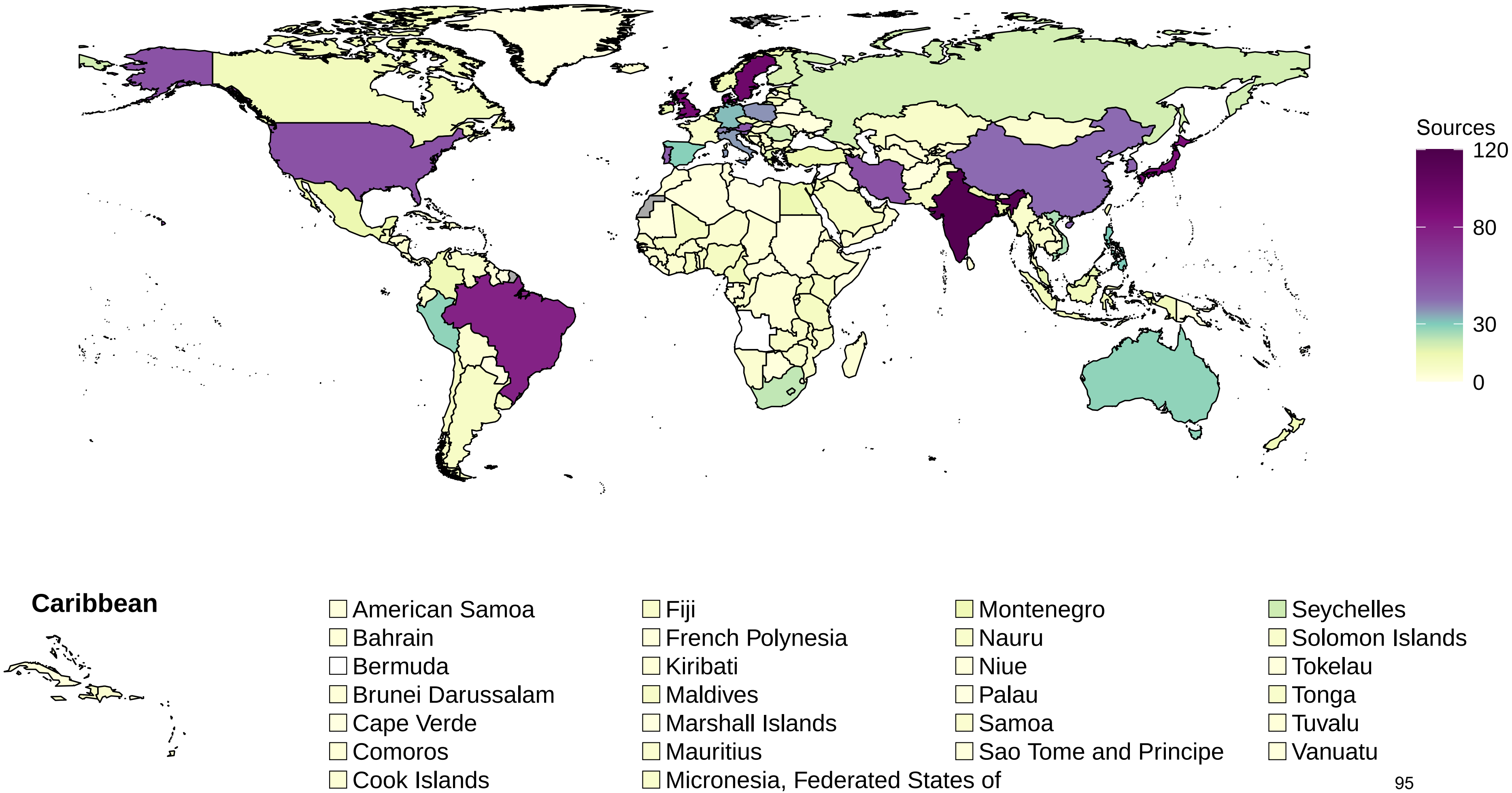
Sources
120
80
30
0
Caribbean
American Samoa
Bahrain
Bermuda
Brunei Darussalam
Cape Verde
Comoros
Cook Islands
Fiji
French Polynesia
Kiribati
Maldives
Marshall Islands
Mauritius
Micronesia, Federated States of
Montenegro
Nauru
Niue
Palau
Samoa
Sao Tome and Principe
Seychelles
Solomon Islands
Tokelau
Tonga
Tuvalu
Vanuatu

Appendix Figure 4. Trends and age trajectories of height and BMI, by country. Solid lines are trends in mean height and body-mass index (BMI) of 19-year-olds and dashed lines are age-trajectory of height and BMI of 19-year-olds in 2019 (i.e., those born in 2000) at each age from 5 years to 19 years.

Afghanistan

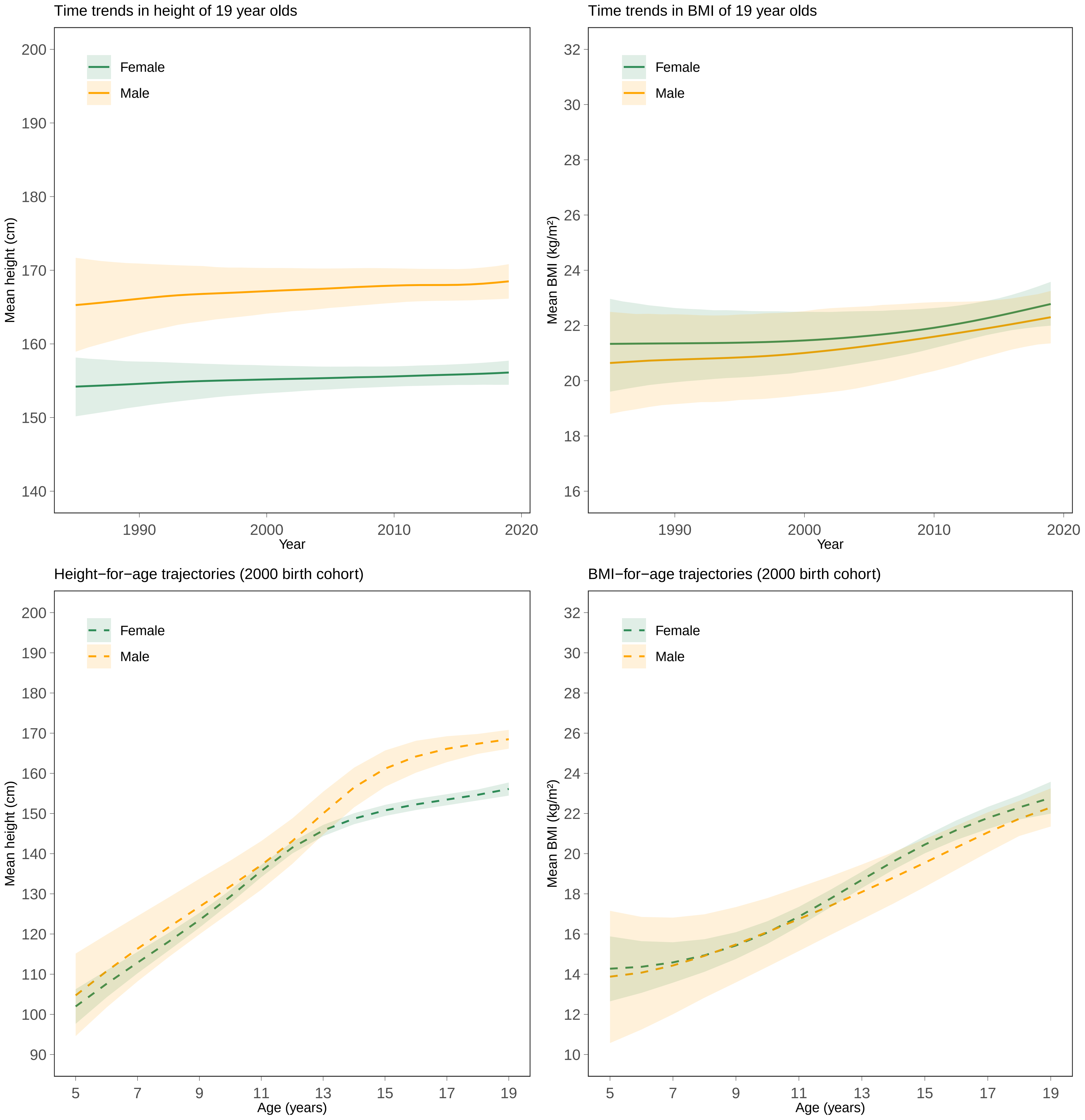

Albania

Time trends in height of 19 year olds

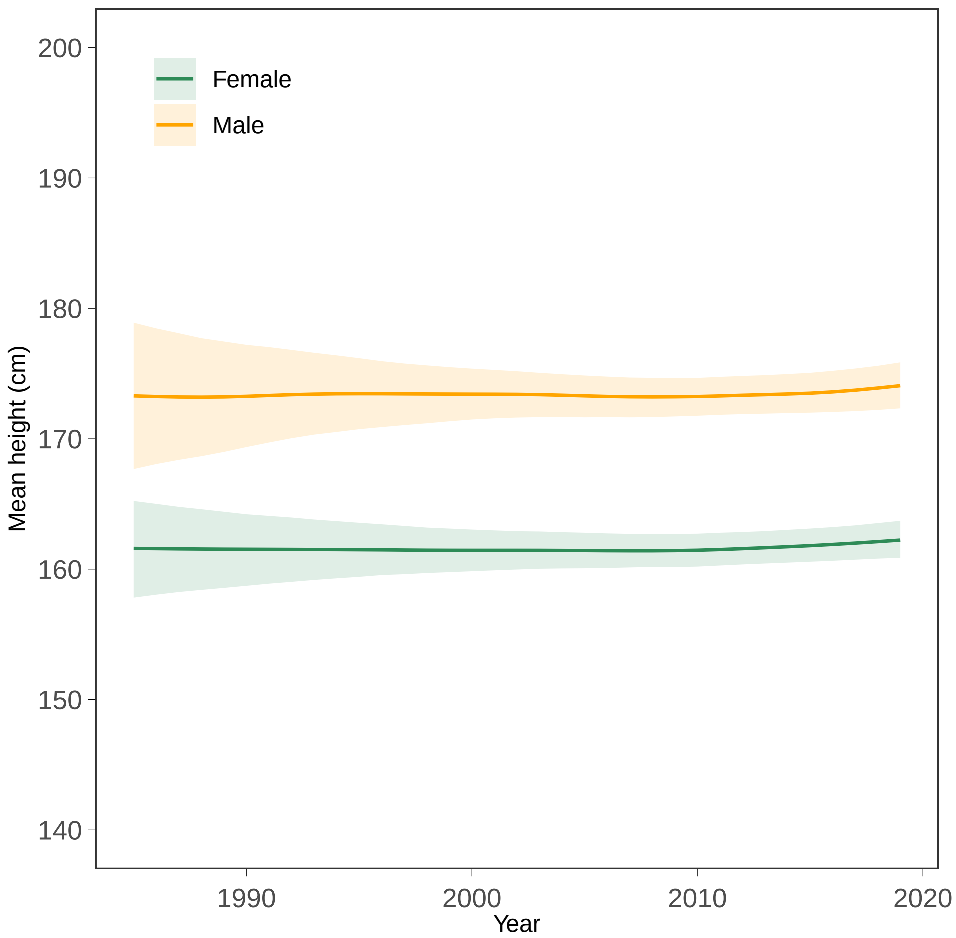

Time trends in BMI of 19 year olds

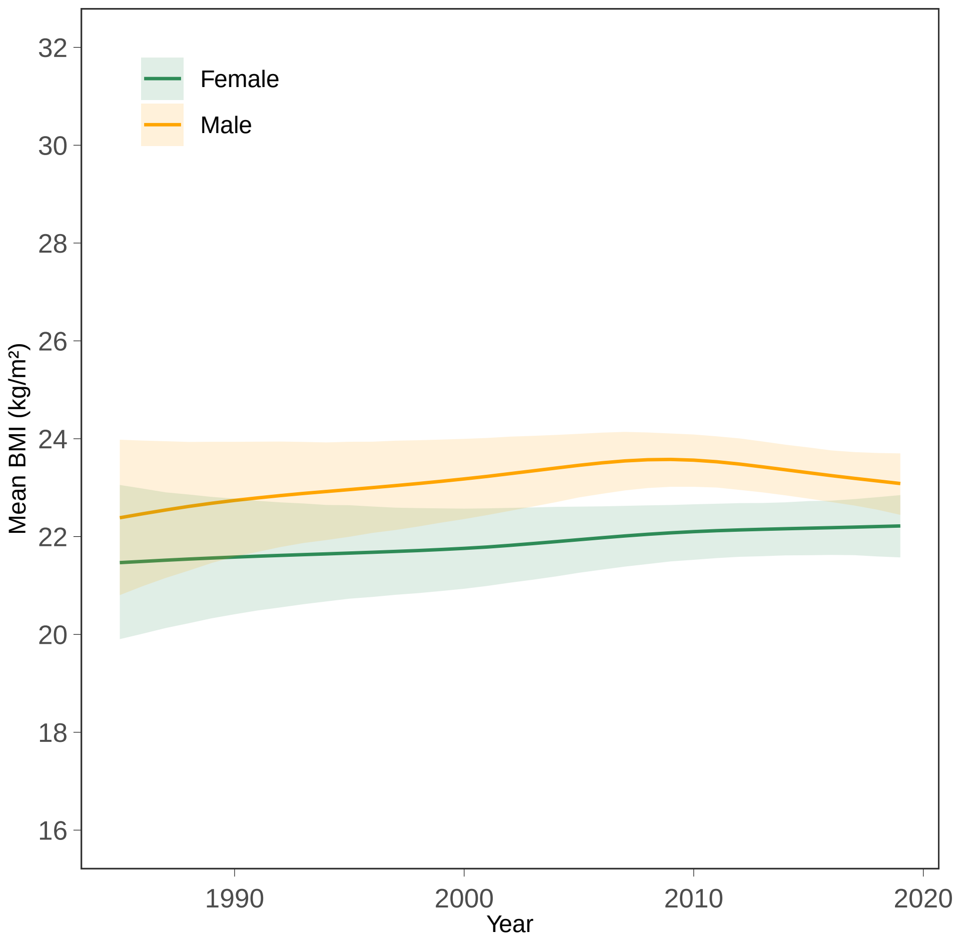

Height−for−age trajectories (2000 birth cohort)

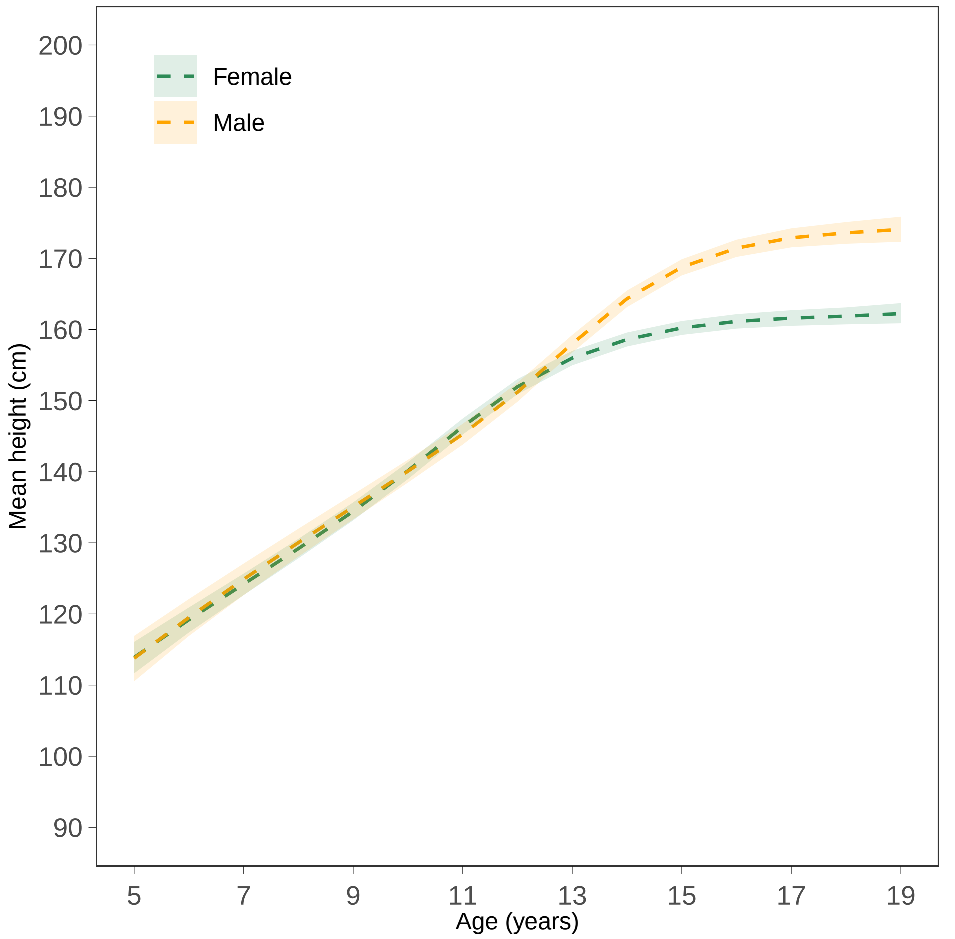

BMI−for−age trajectories (2000 birth cohort)

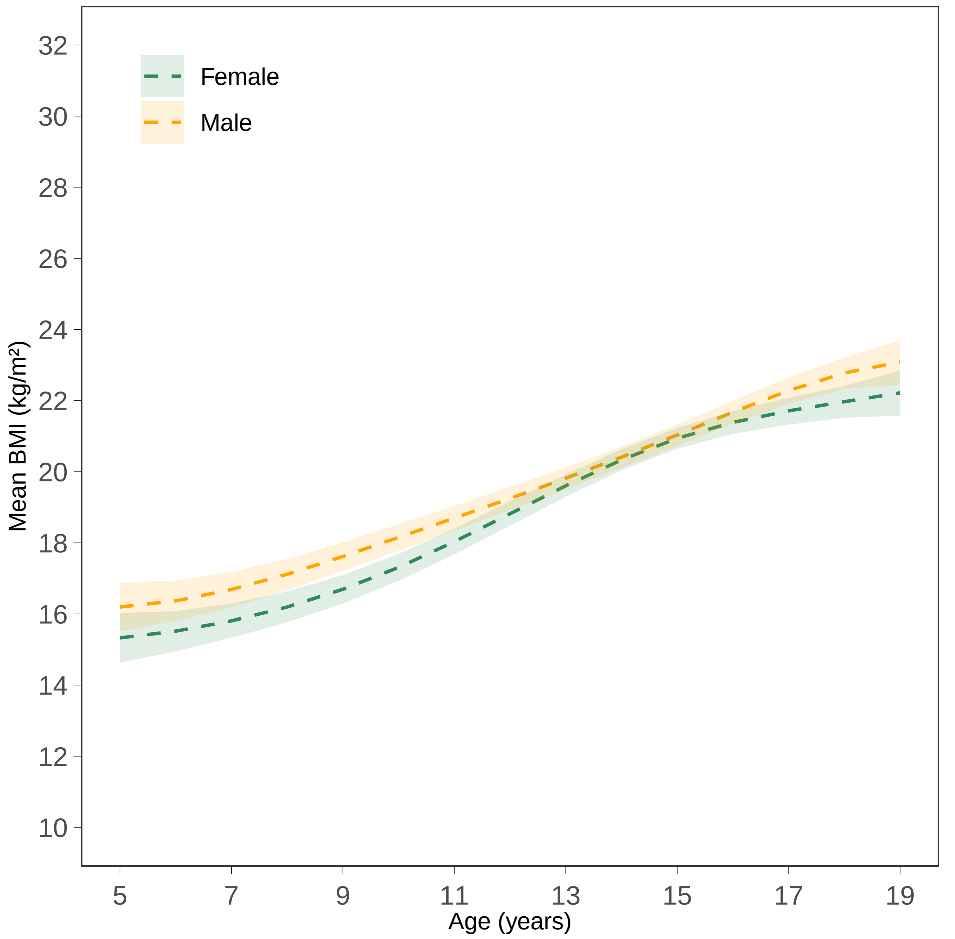

Algeria

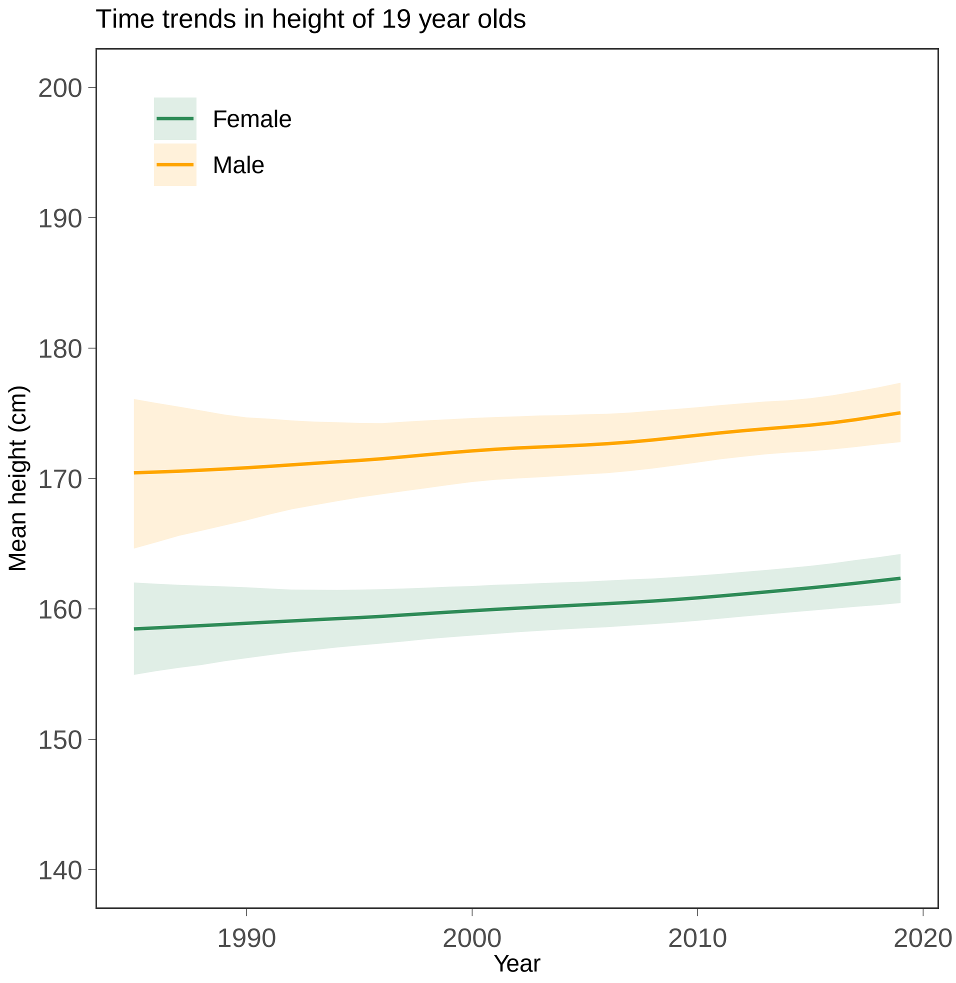

Time trends in height of 19 year olds
Female
Male
Mean height (cm)
200
190
180
170
160
150
140
1990
2000
2010
2020
Year

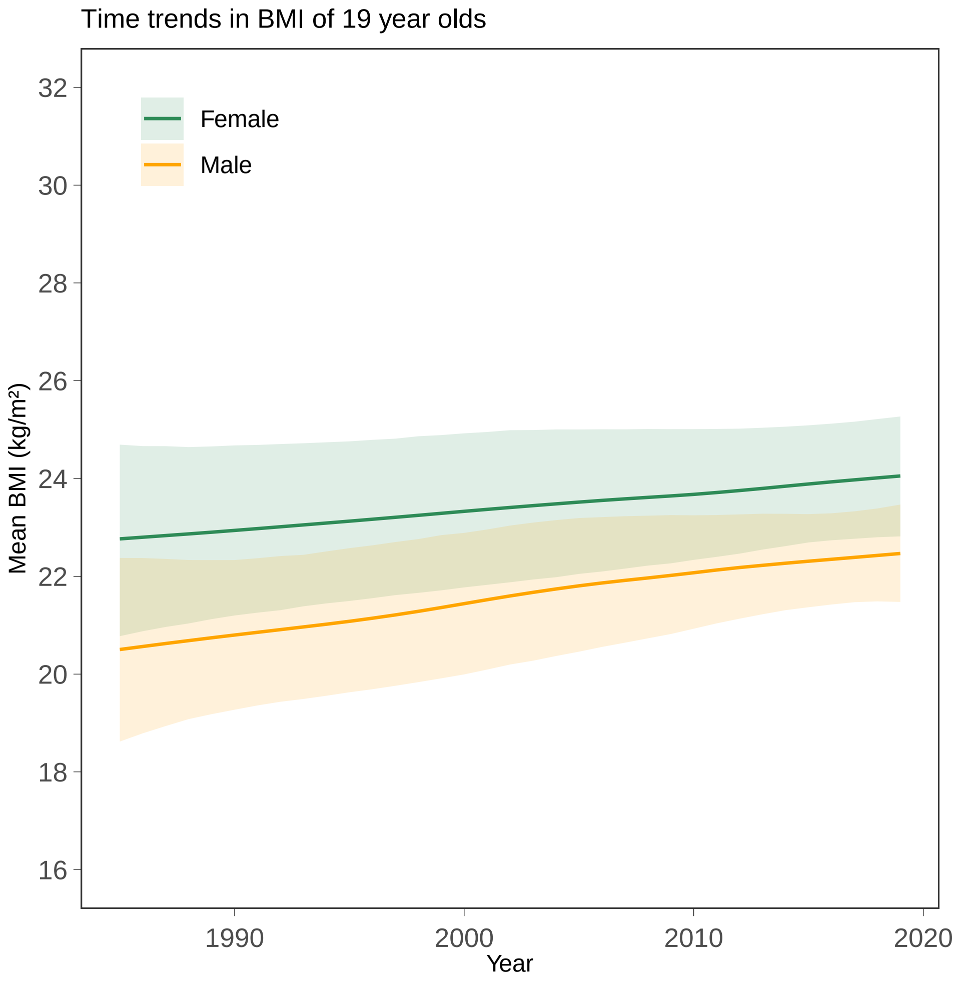

Time trends in BMI of 19 year olds
Female
Male
Mean BMI (kg/m²)
32
30
28
26
24
22
20
18
16
1990
2000
2010
2020
Year

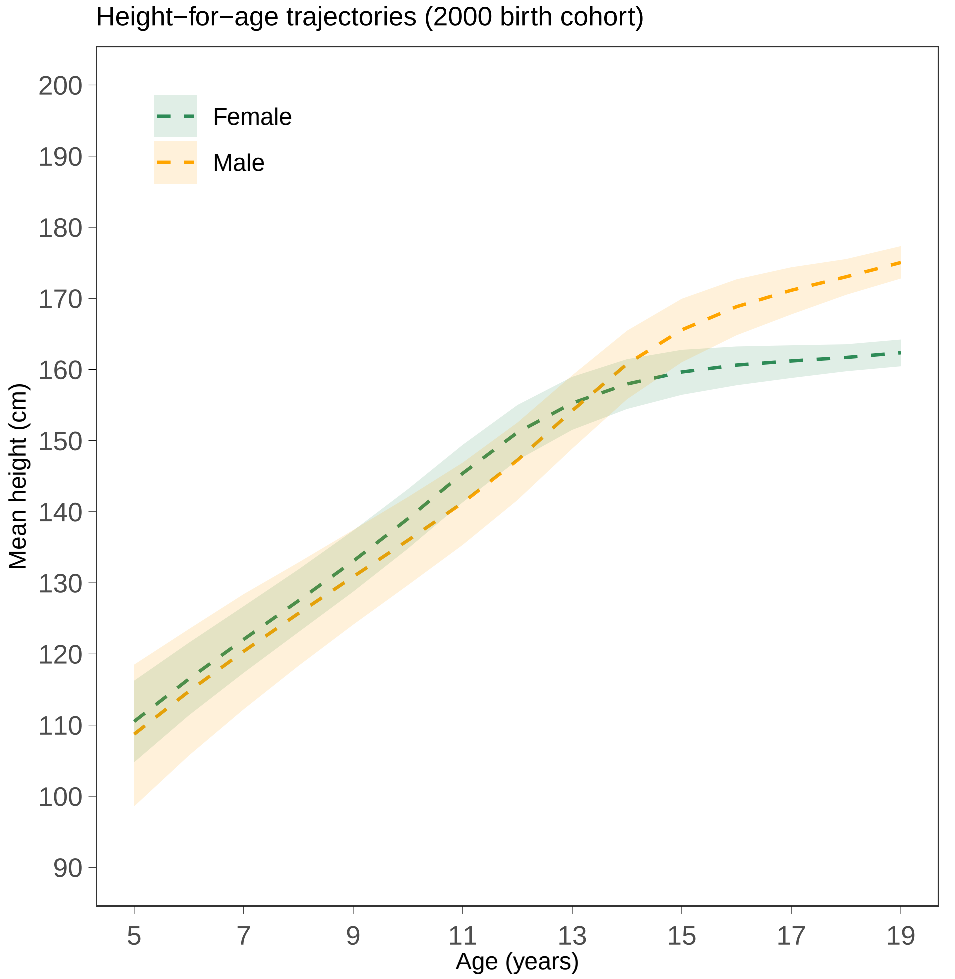

Height−for−age trajectories (2000 birth cohort)
Female
Male
Mean height (cm)
200
190
180
170
160
150
140
130
120
110
100
90
5
7
9
11
13
15
17
19
Age (years)

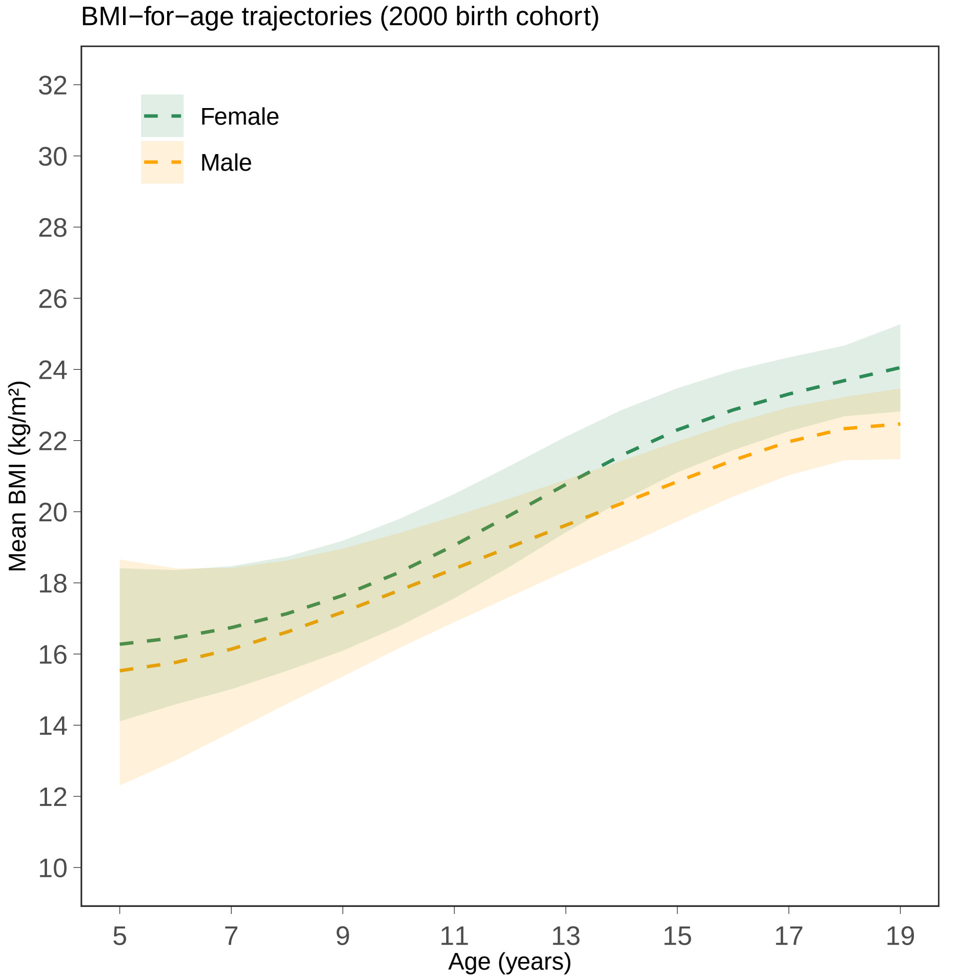

BMI−for−age trajectories (2000 birth cohort)
Female
Male
Mean BMI (kg/m²)
32
30
28
26
24
22
20
18
16
14
12
10
5
7
9
11
13
15
17
19
Age (years)

American Samoa

Time trends in height of 19 year olds

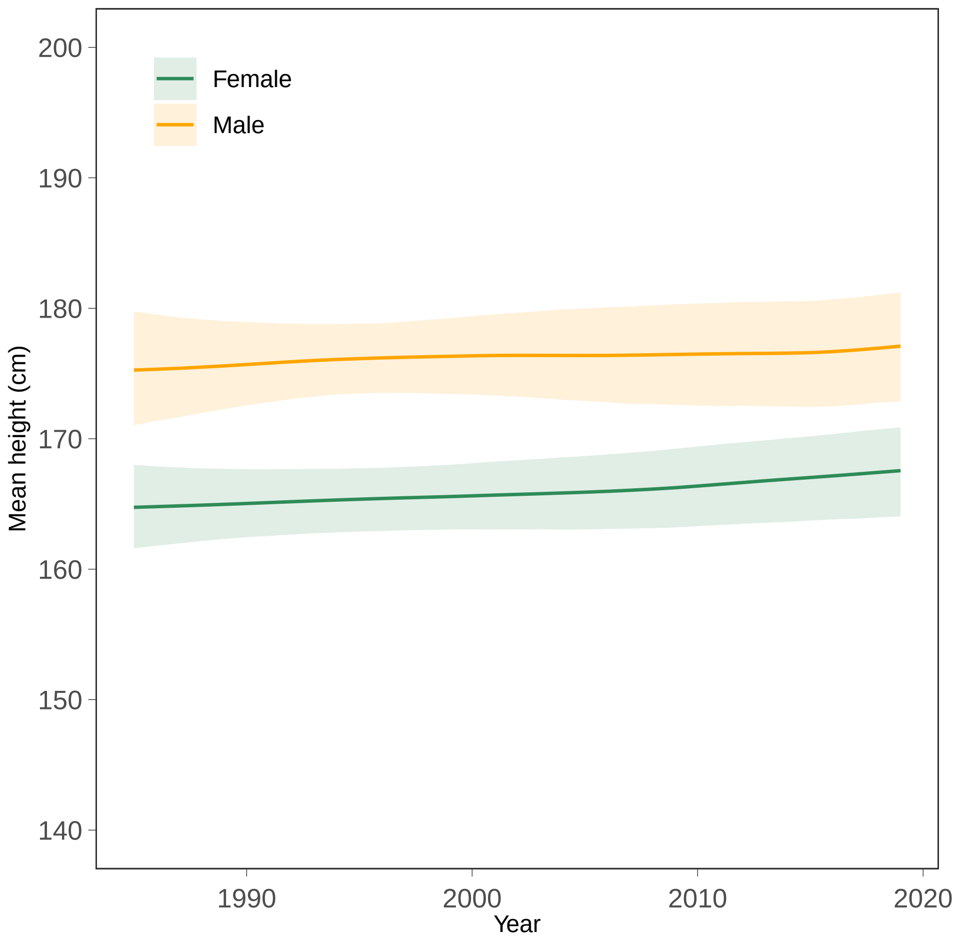

Time trends in BMI of 19 year olds

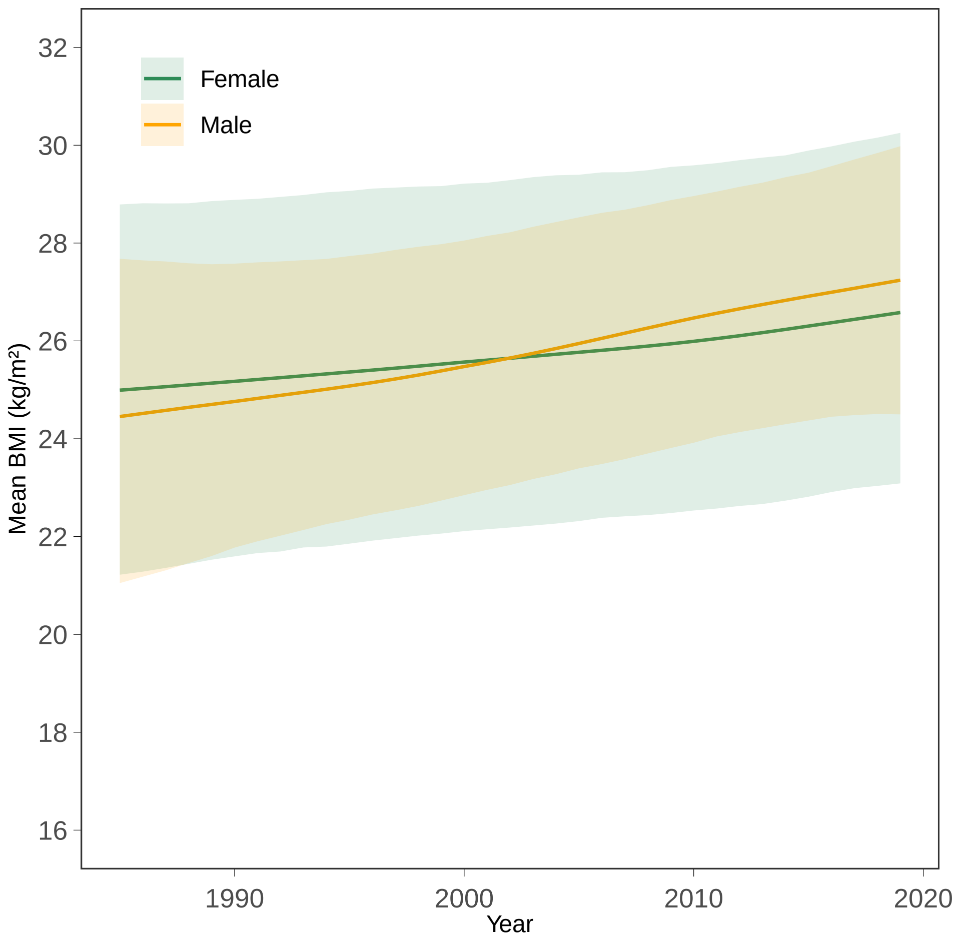

Height−for−age trajectories (2000 birth cohort)

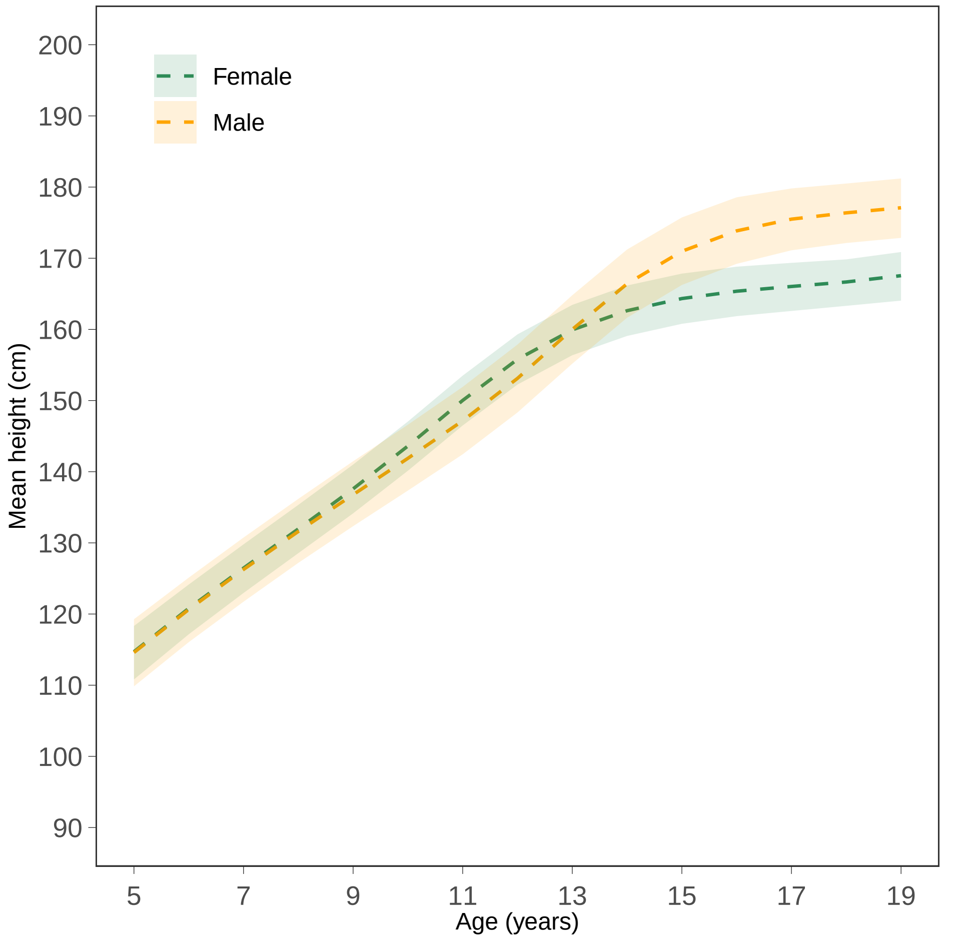

BMI−for−age trajectories (2000 birth cohort)

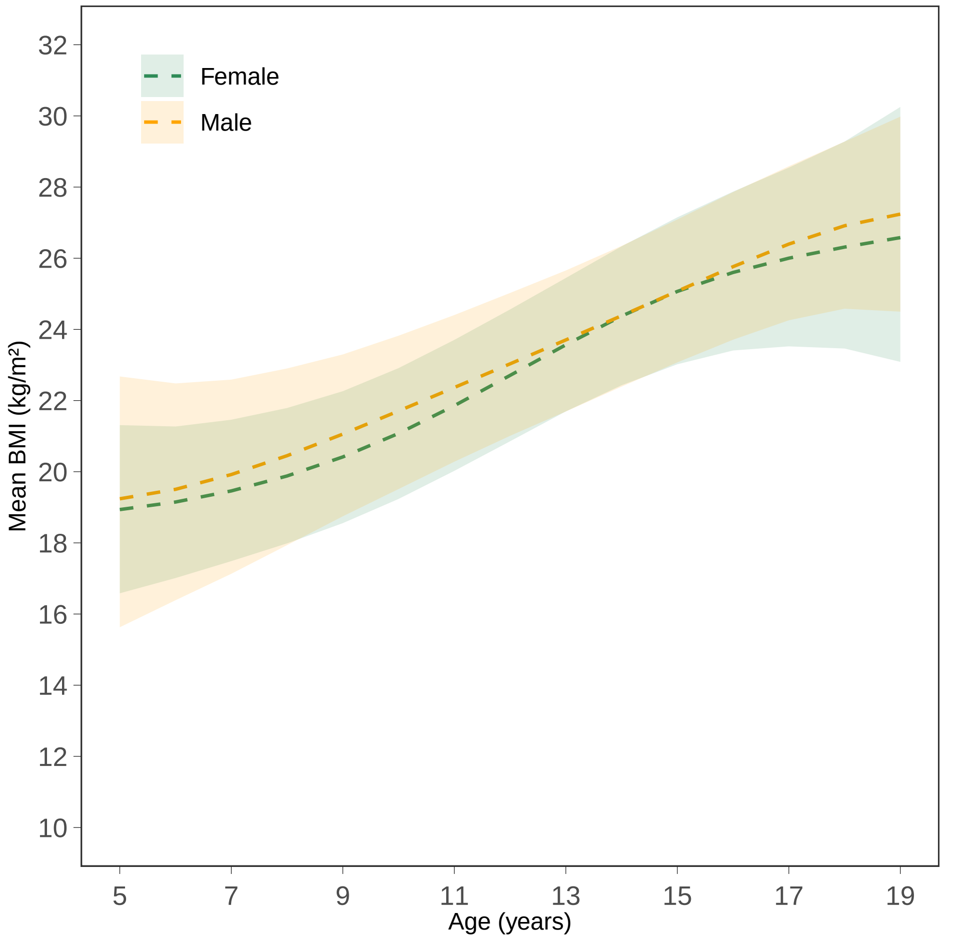

Andorra

Time trends in height of 19 year olds

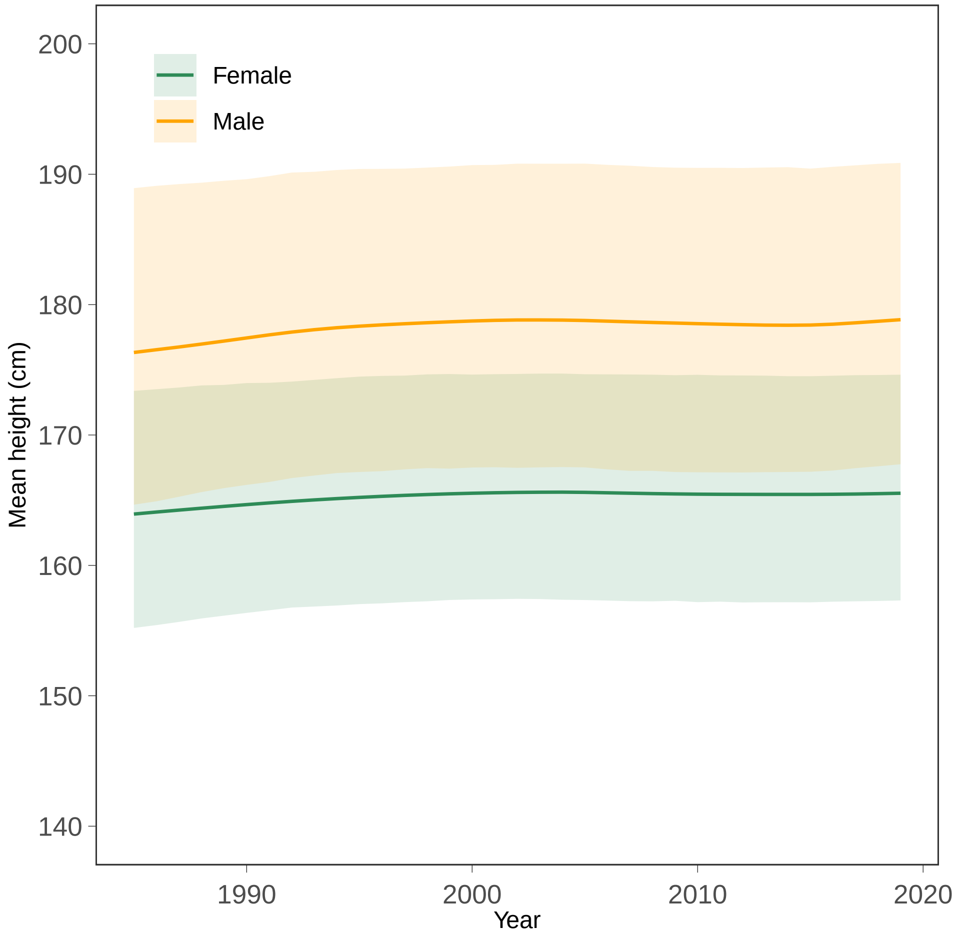

Time trends in BMI of 19 year olds

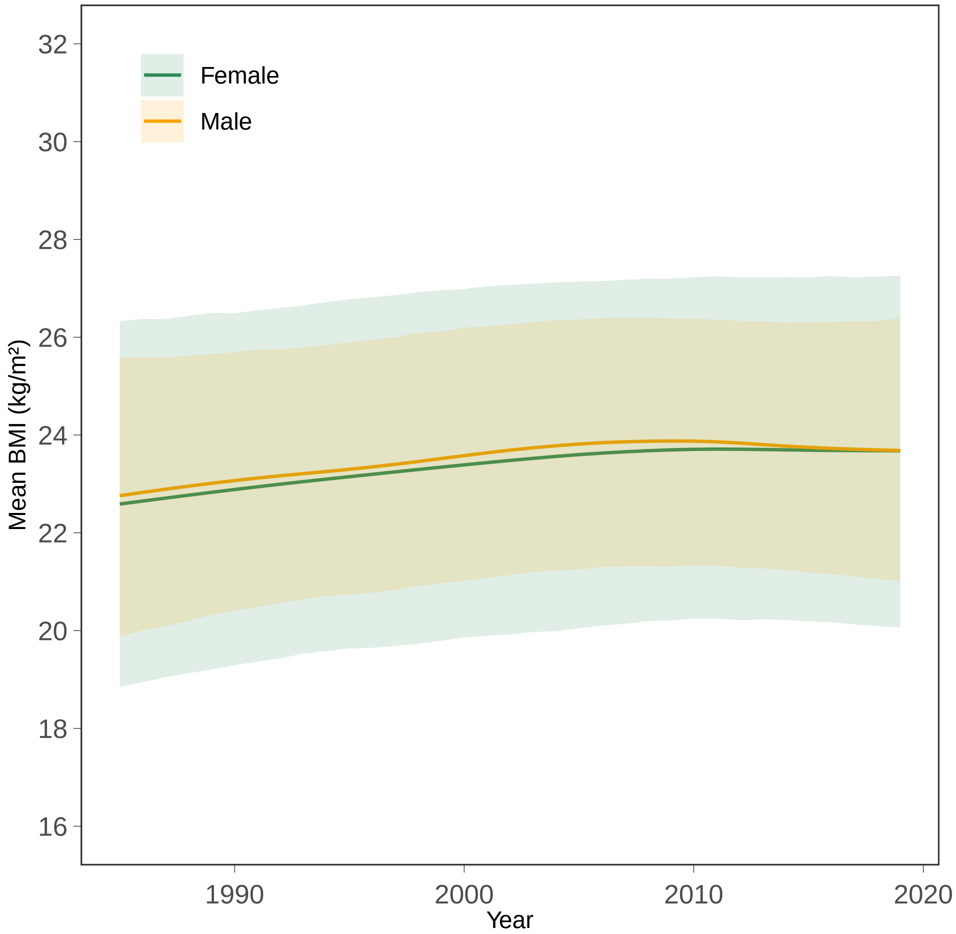

Height−for−age trajectories (2000 birth cohort)

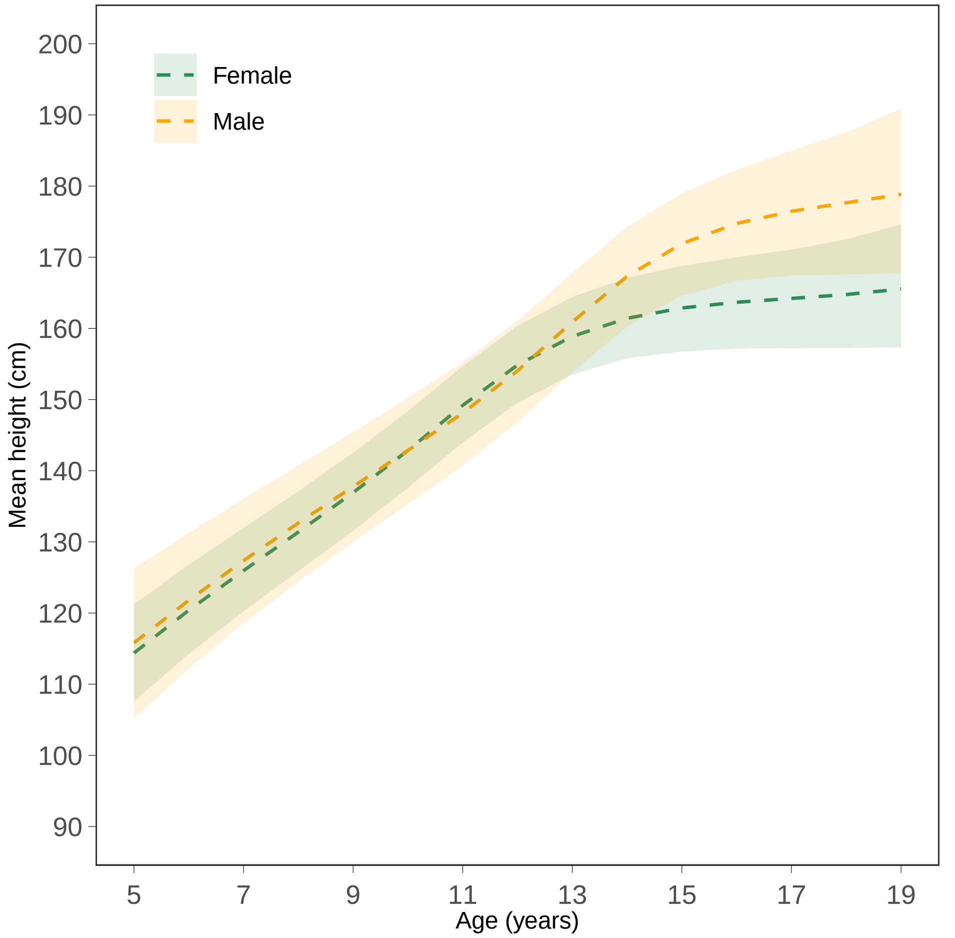

BMI−for−age trajectories (2000 birth cohort)

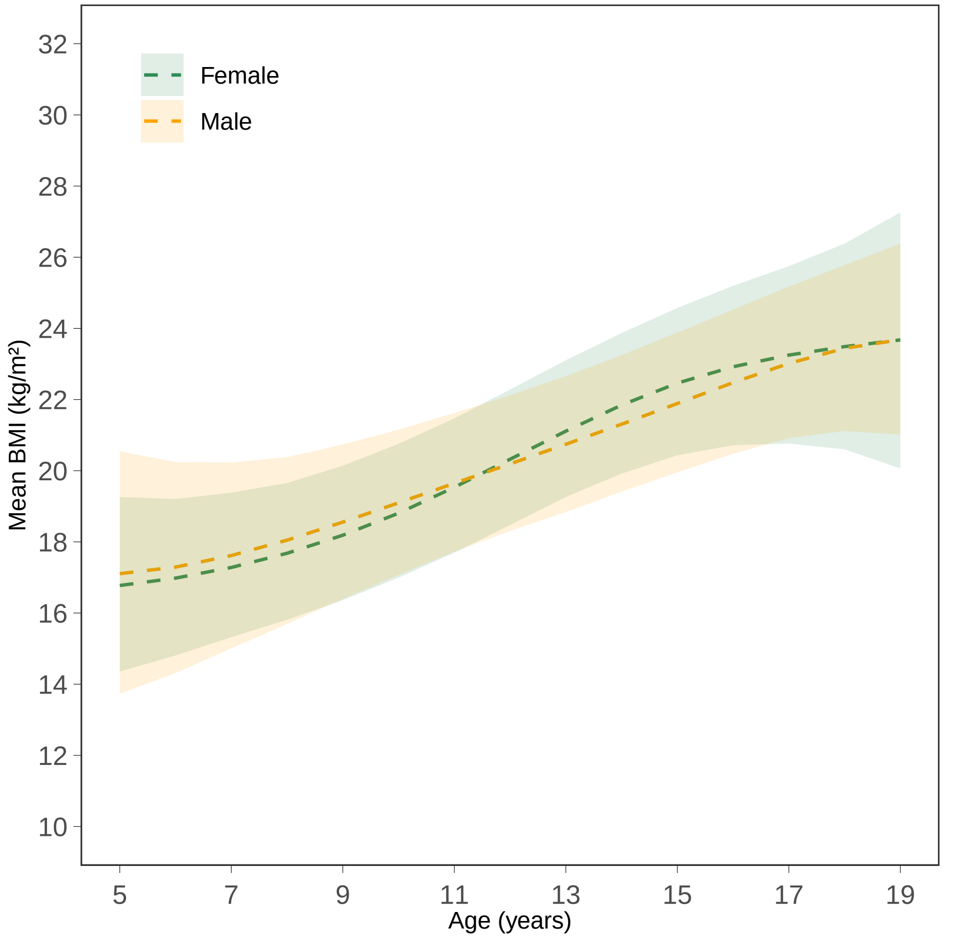

Angola

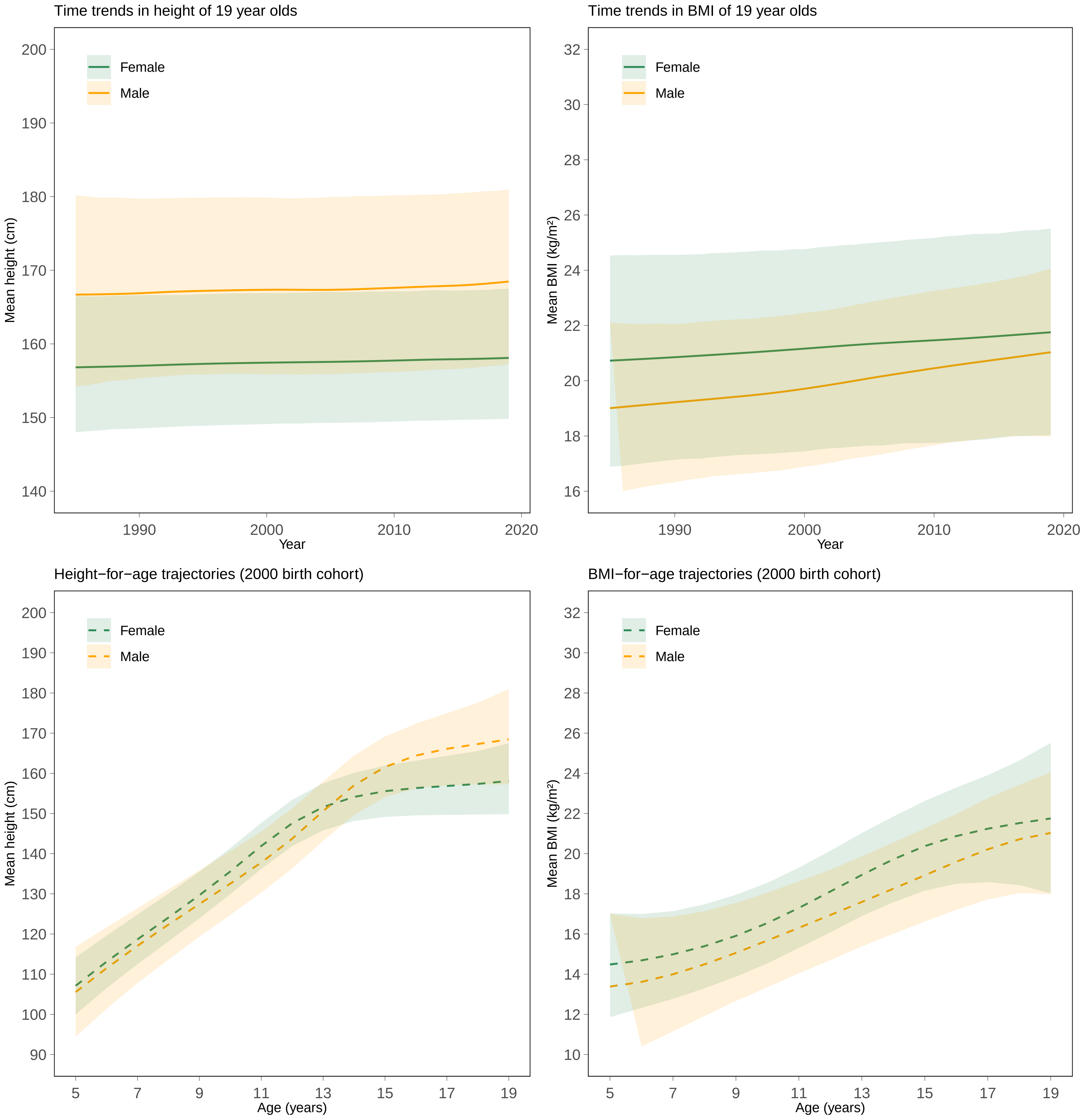

Time trends in height of 19 year olds
Female
Male
Mean height (cm)
200
190
180
170
160
150
140
1990
2000
2010
2020
Year
Time trends in BMI of 19 year olds
Female
Male
Mean BMI (kg/m²)
32
30
28
26
24
22
20
18
16
1990
2000
2010
2020
Year
Height−for−age trajectories (2000 birth cohort)
Female
Male
Mean height (cm)
200
190
180
170
160
150
140
130
120
110
100
90
5
7
9
11
13
15
17
19
Age (years)
BMI−for−age trajectories (2000 birth cohort)
Female
Male
Mean BMI (kg/m²)
32
30
28
26
24
22
20
18
16
14
12
10
5
7
9
11
13
15
17
19
Age (years)

Antigua and Barbuda

Time trends in height of 19 year olds

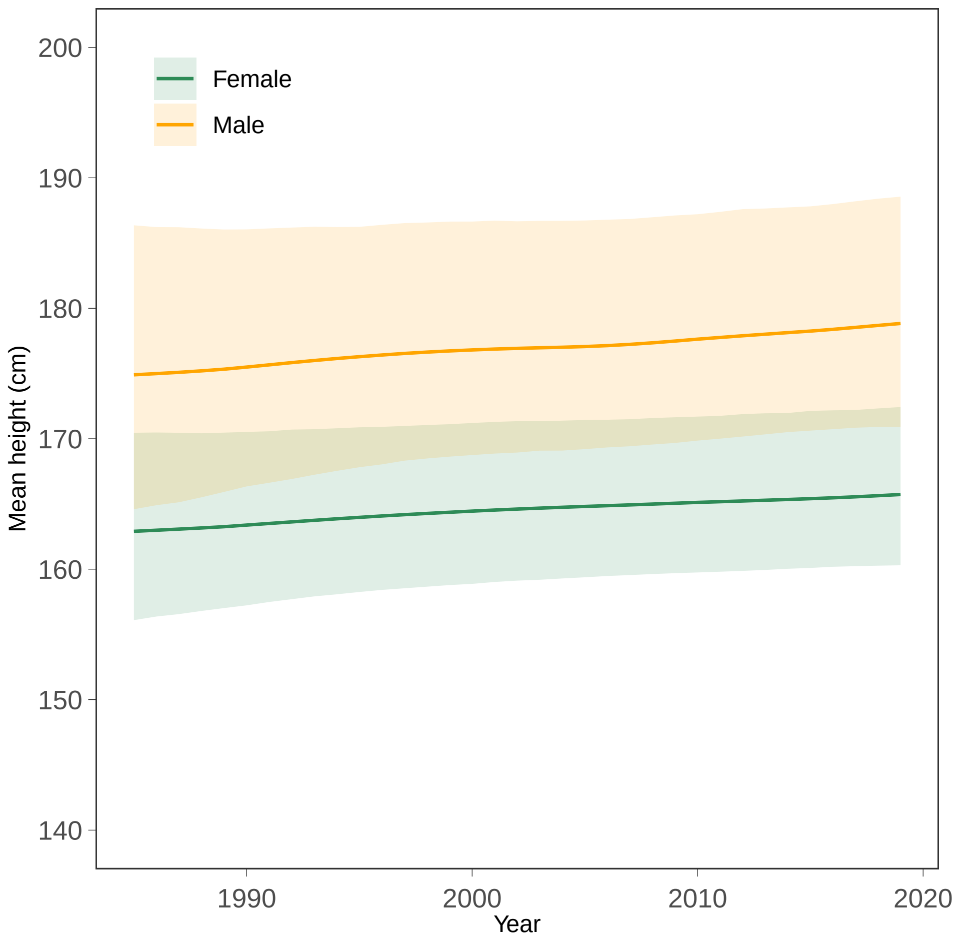

Time trends in BMI of 19 year olds

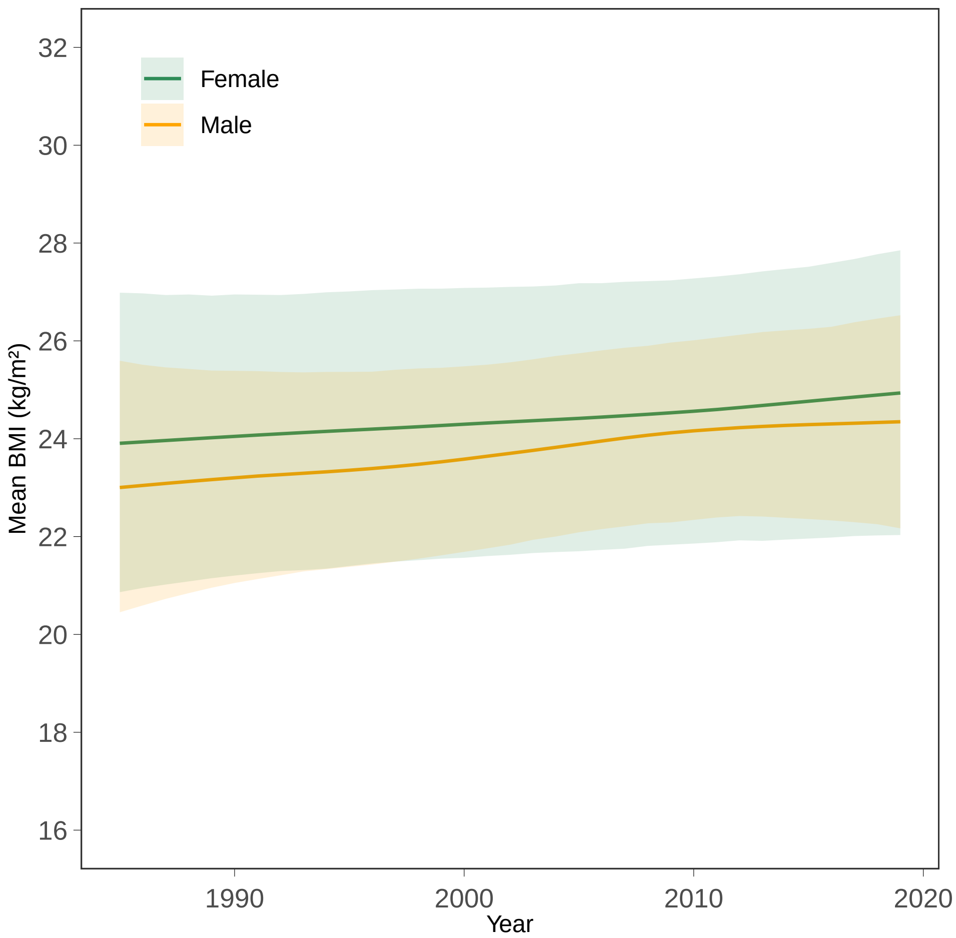

Height−for−age trajectories (2000 birth cohort)

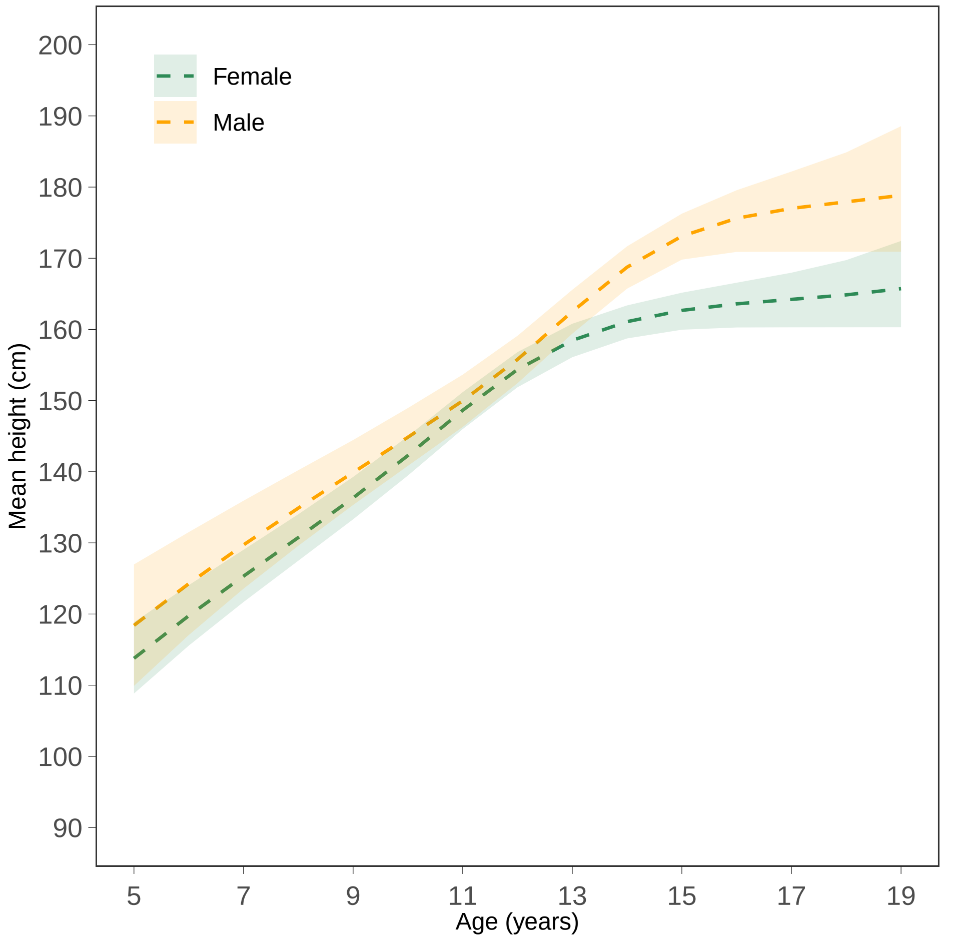

BMI−for−age trajectories (2000 birth cohort)

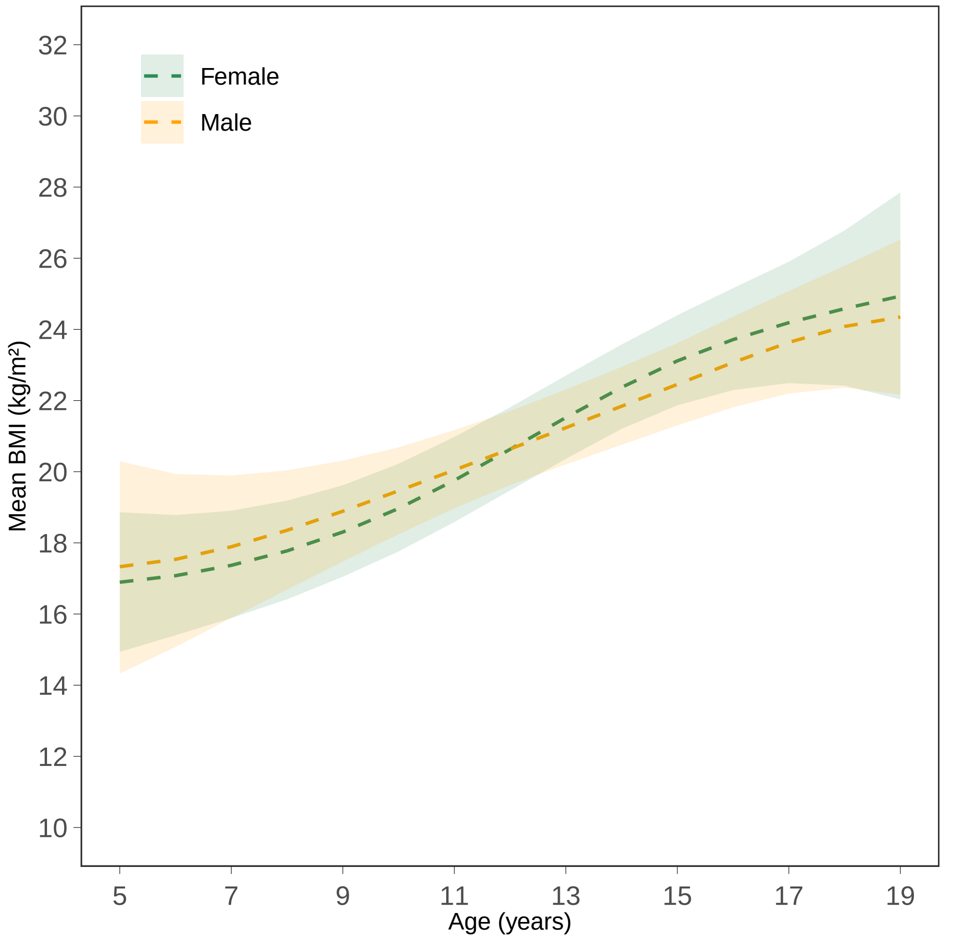

Argentina

Time trends in height of 19 year olds

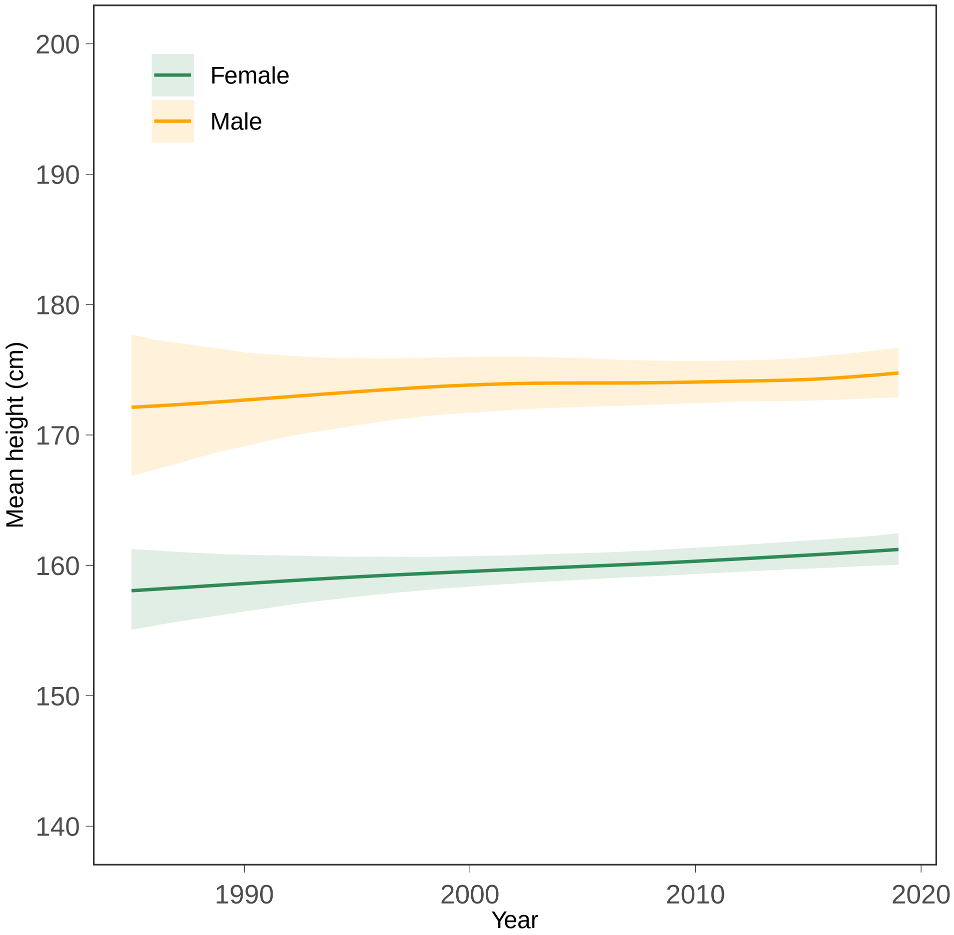

Time trends in BMI of 19 year olds

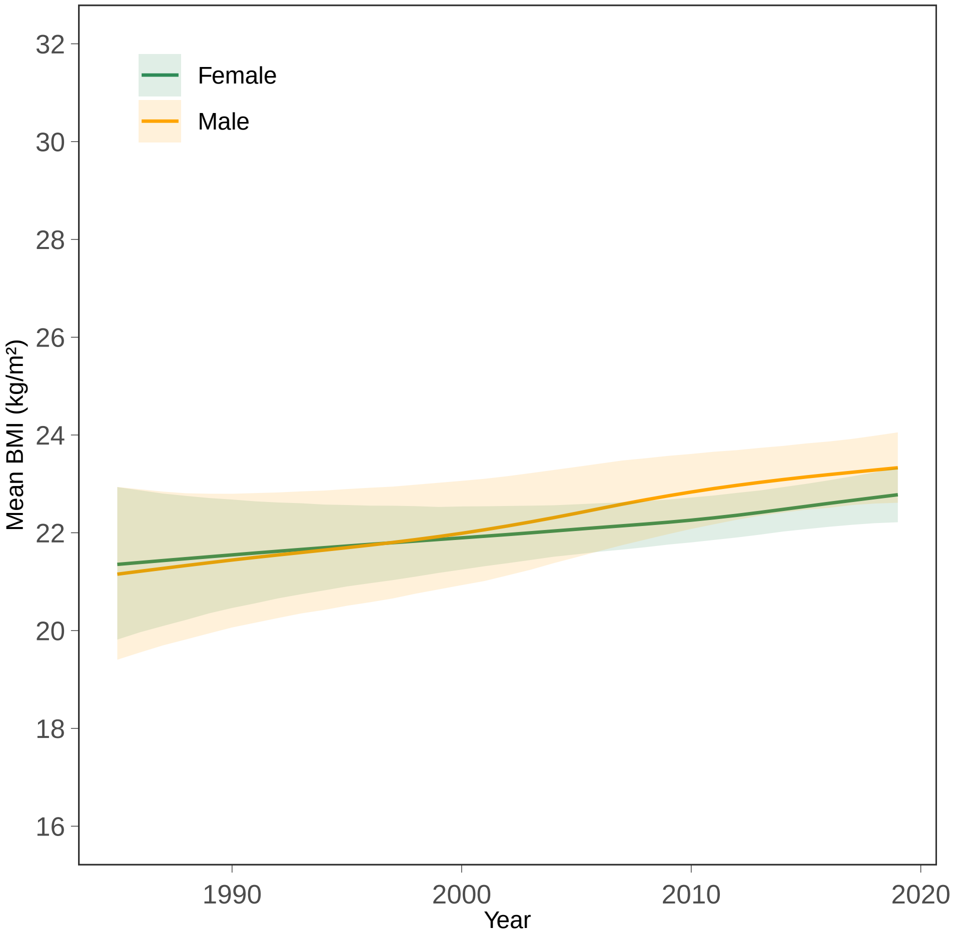

Height−for−age trajectories (2000 birth cohort)

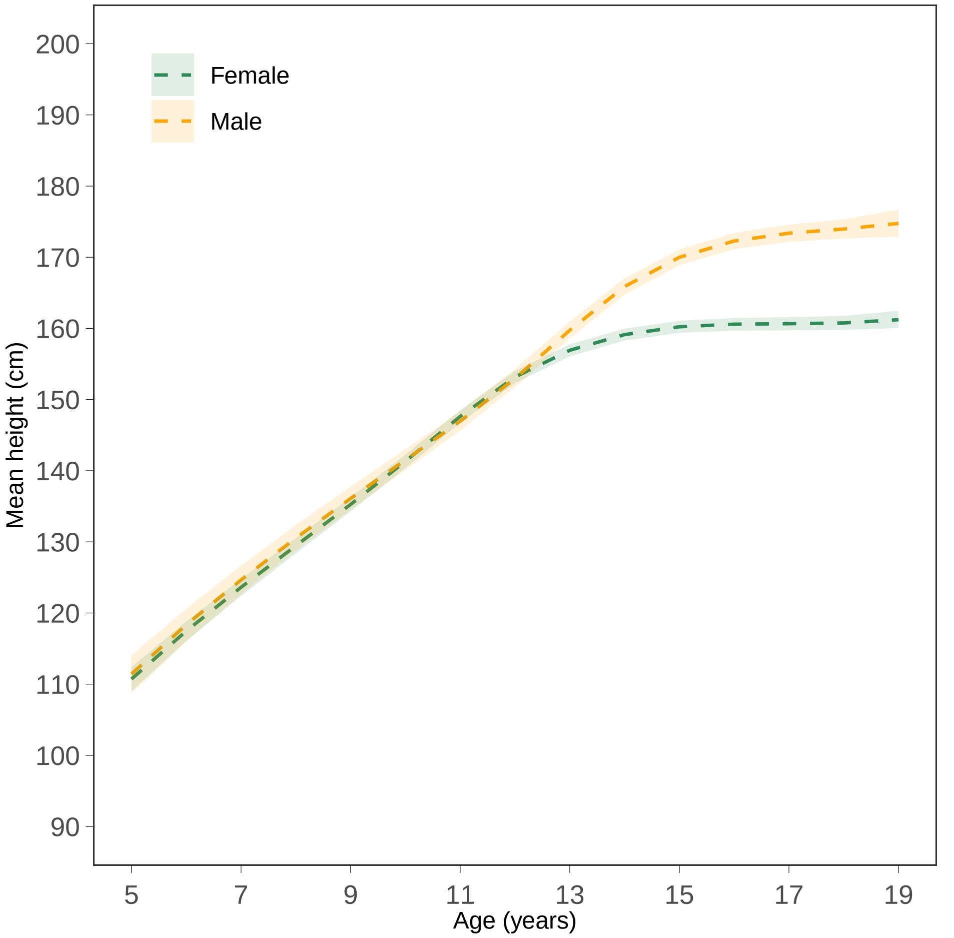

BMI−for−age trajectories (2000 birth cohort)

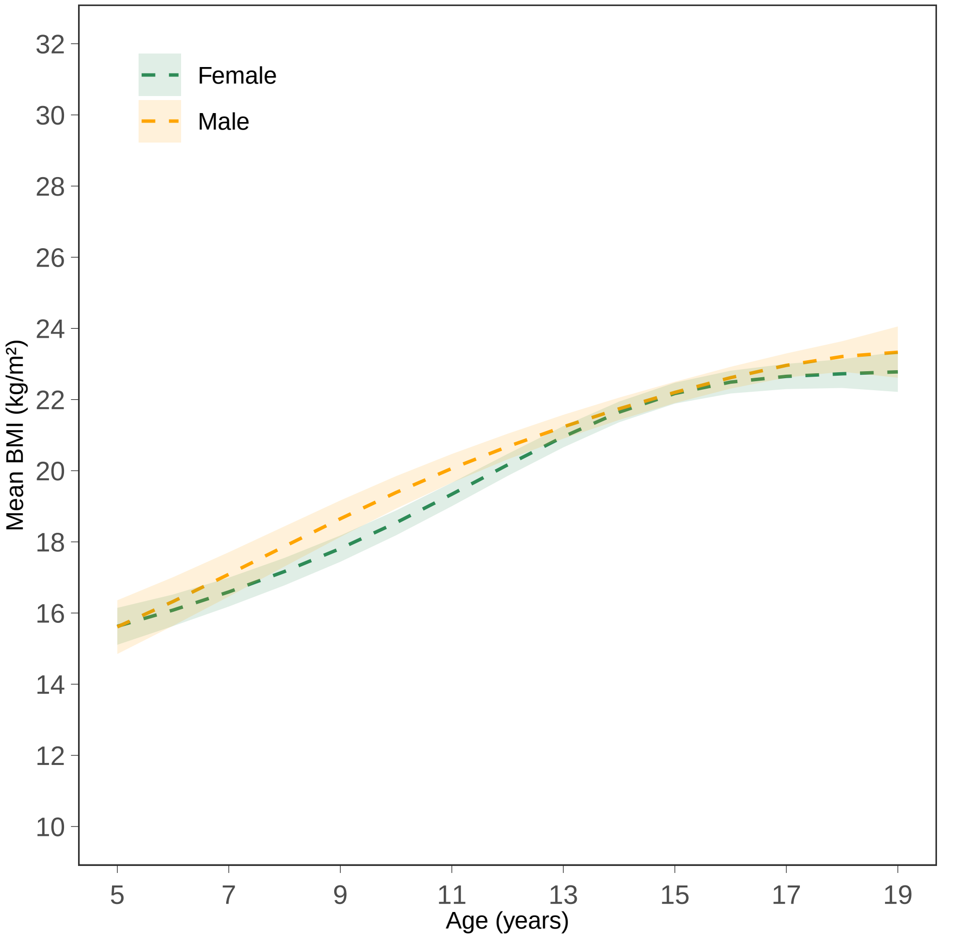

Armenia

Time trends in height of 19 year olds

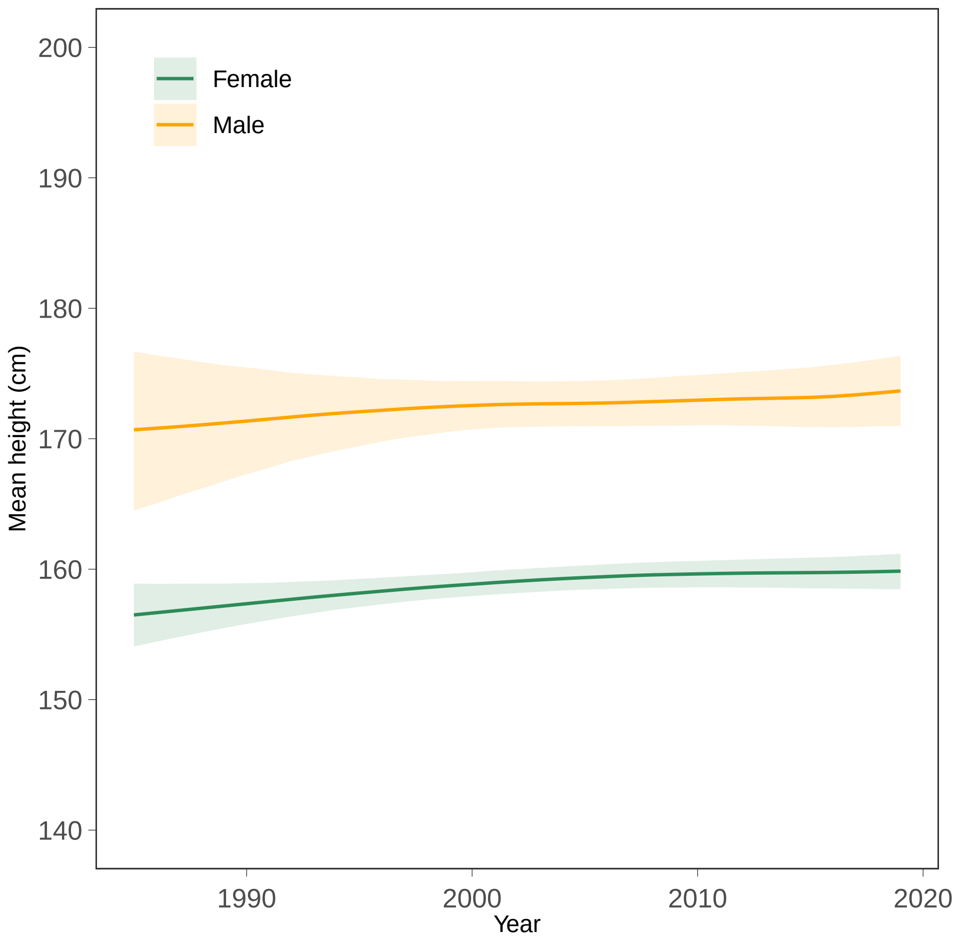

Time trends in BMI of 19 year olds

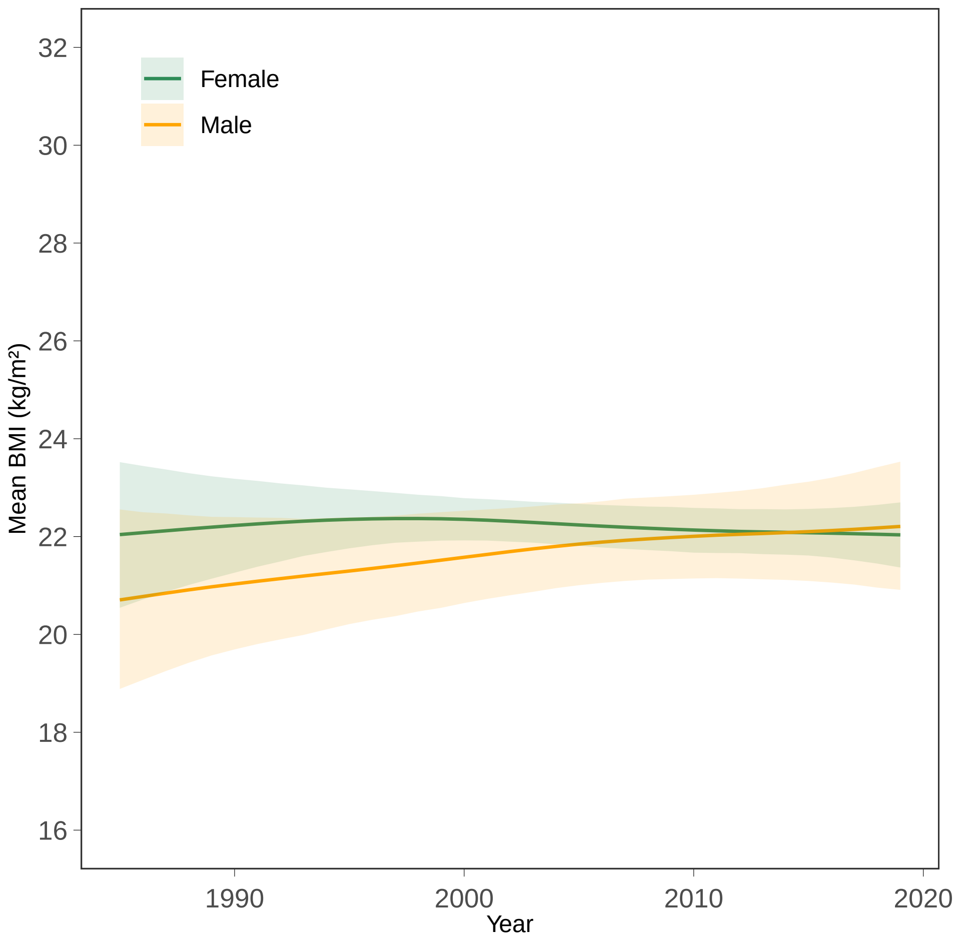

Height−for−age trajectories (2000 birth cohort)

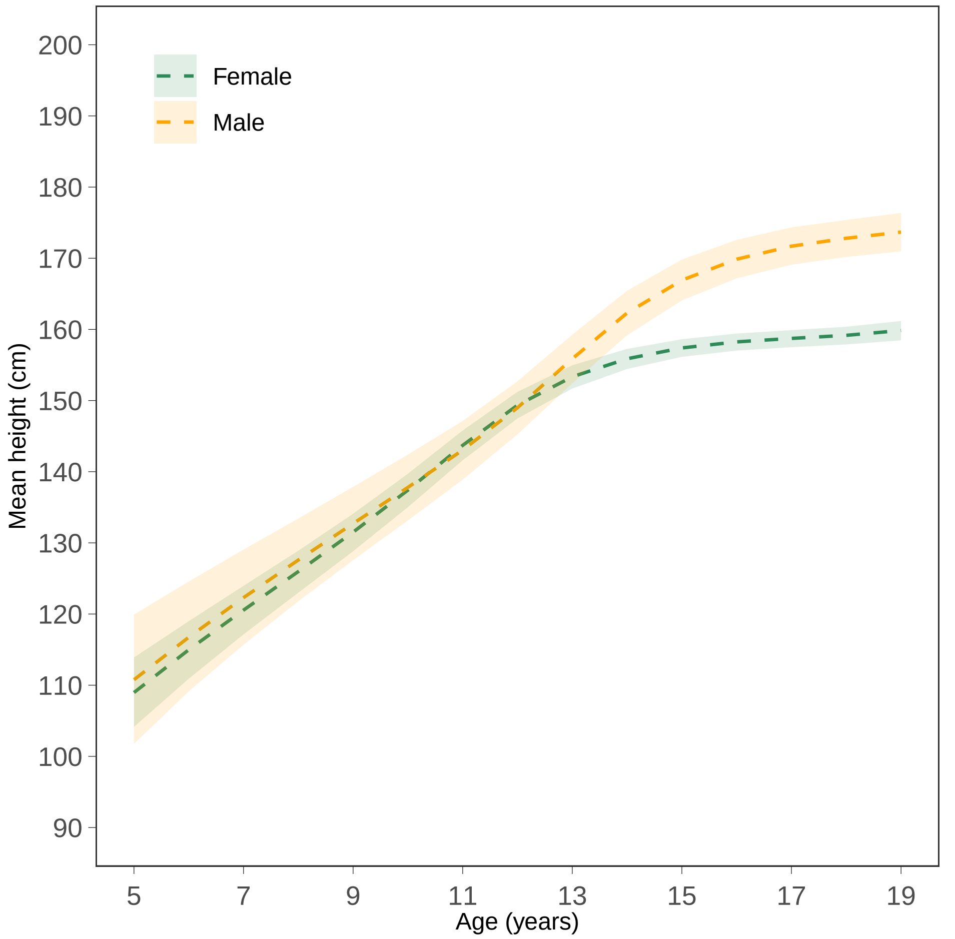

BMI−for−age trajectories (2000 birth cohort)

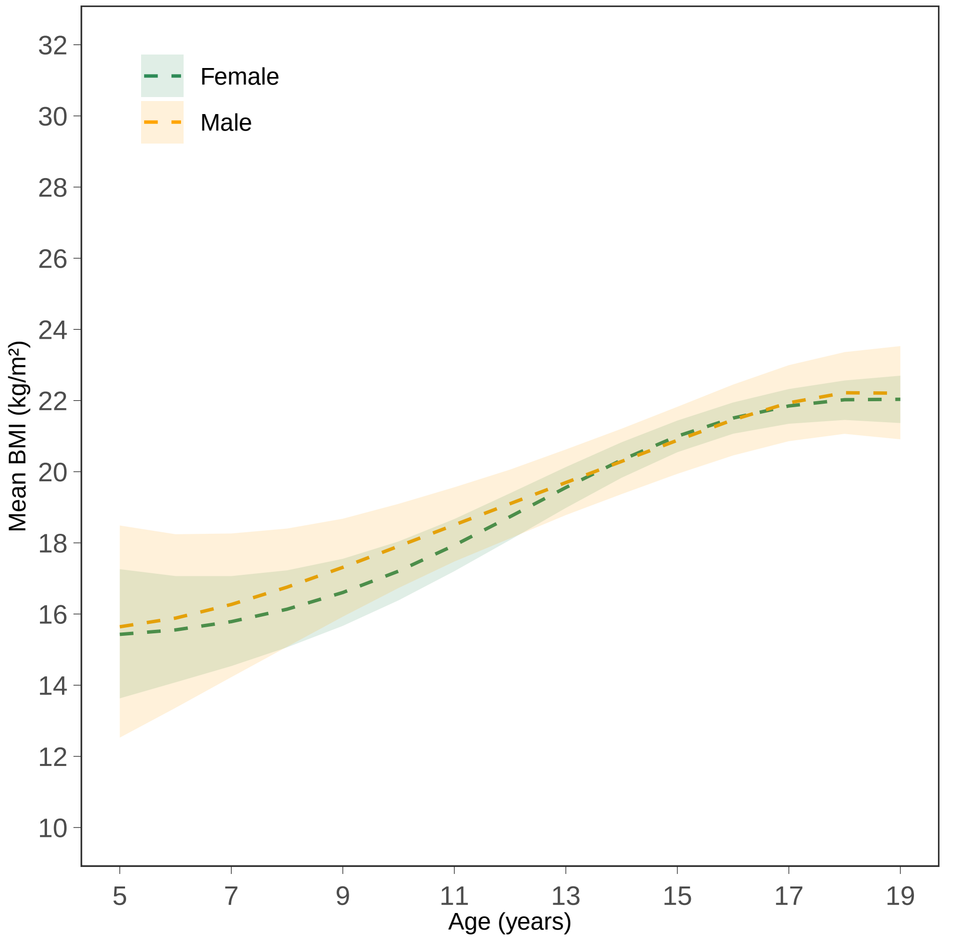

Australia

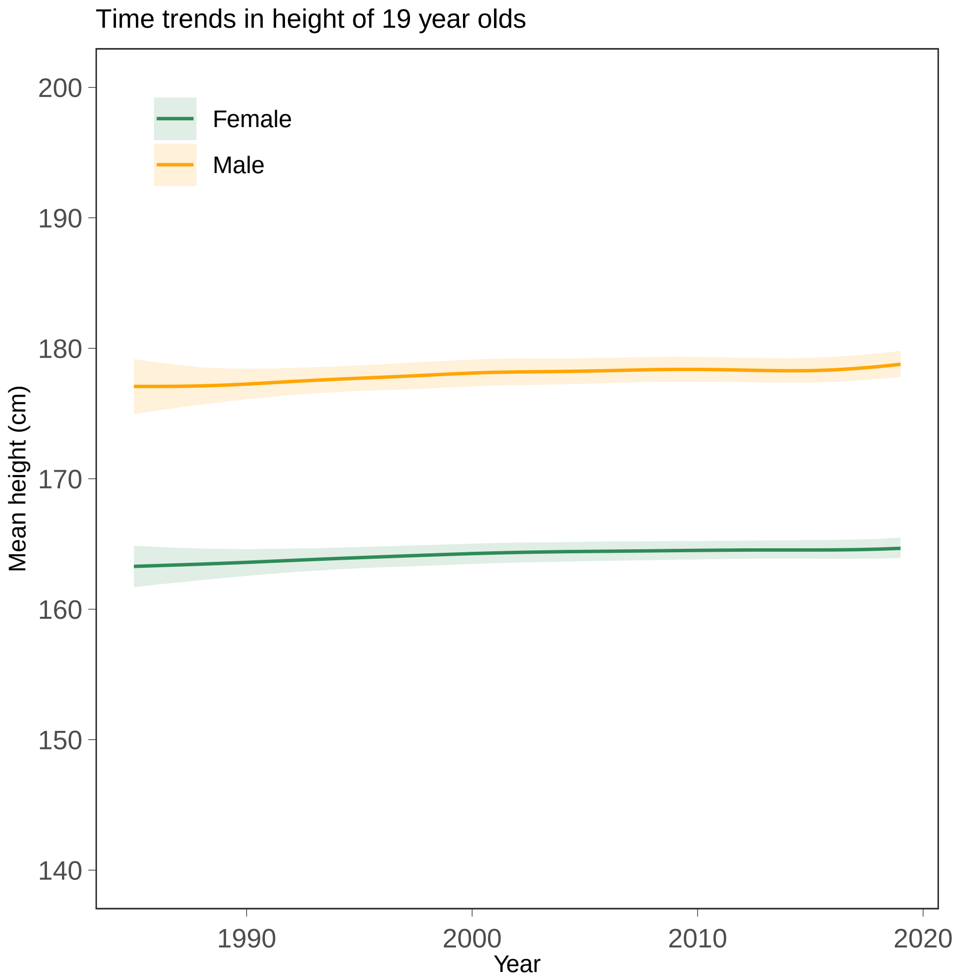
Time trends in height of 19 year olds
Female
Male
Mean height (cm)
200
190
180
170
160
150
140
1990
2000
2010
2020
Year

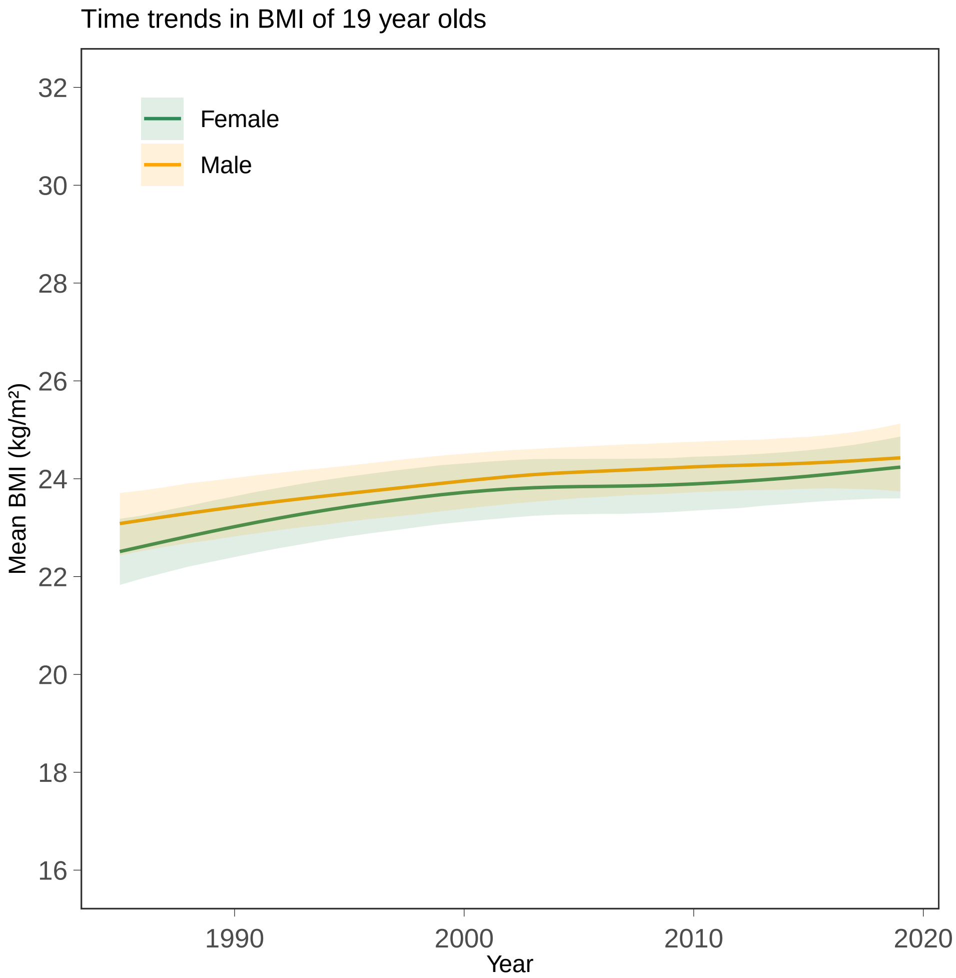
Time trends in BMI of 19 year olds
Female
Male
Mean BMI (kg/m²)
32
30
28
26
24
22
20
18
16
1990
2000
2010
2020
Year

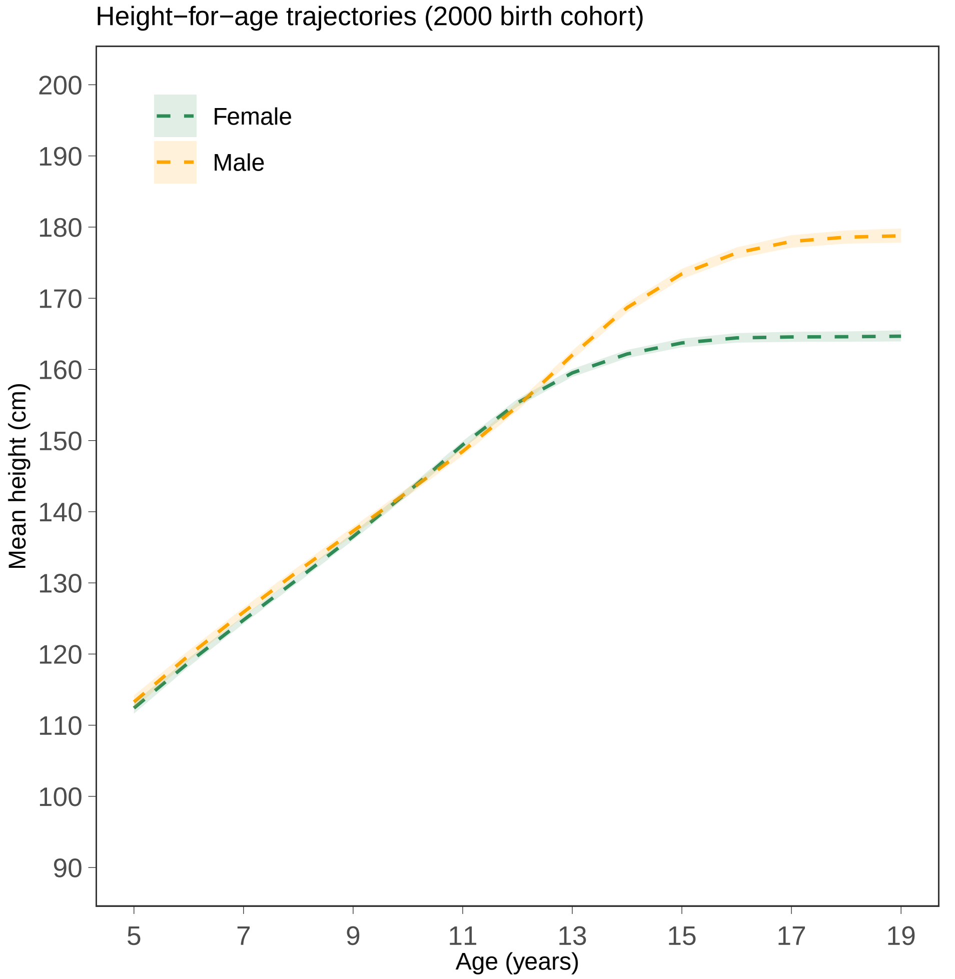
Height−for−age trajectories (2000 birth cohort)
Female
Male
Mean height (cm)
200
190
180
170
160
150
140
130
120
110
100
90
5
7
9
11
13
15
17
19
Age (years)

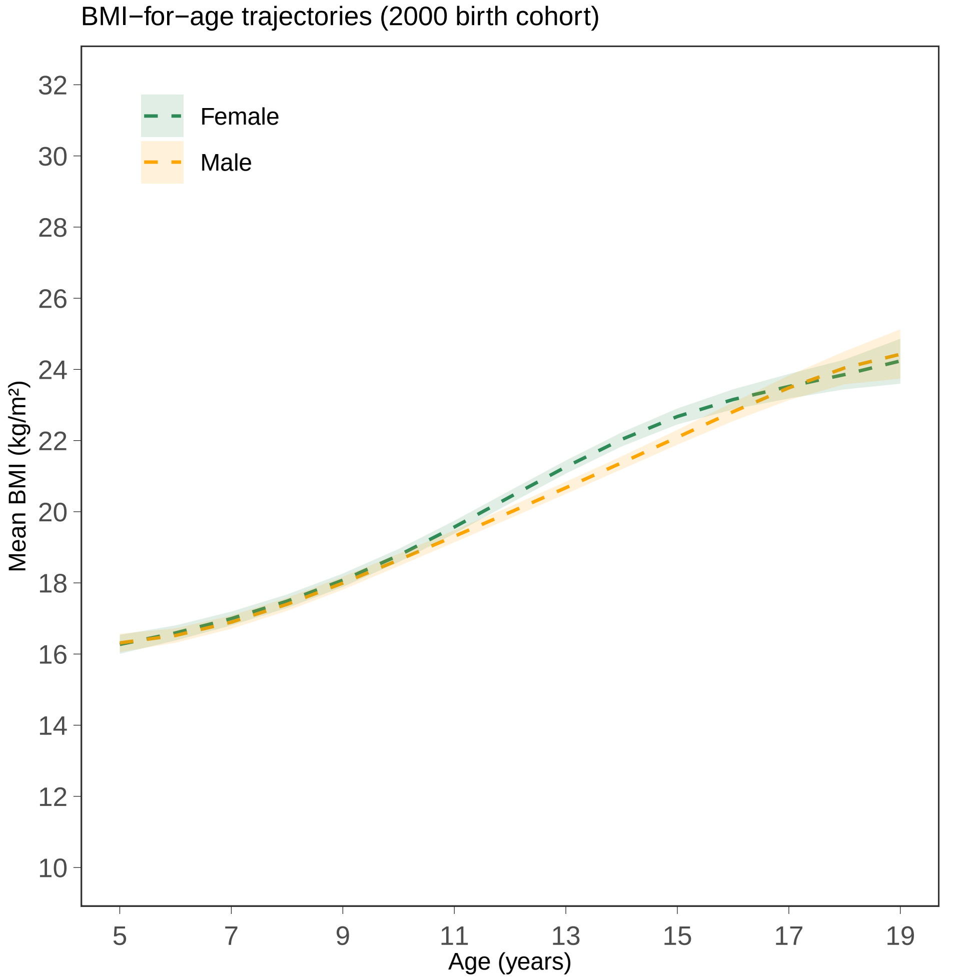
BMI−for−age trajectories (2000 birth cohort)
Female
Male
Mean BMI (kg/m²)
32
30
28
26
24
22
20
18
16
14
12
10
5
7
9
11
13
15
17
19
Age (years)

Austria

Time trends in height of 19 year olds

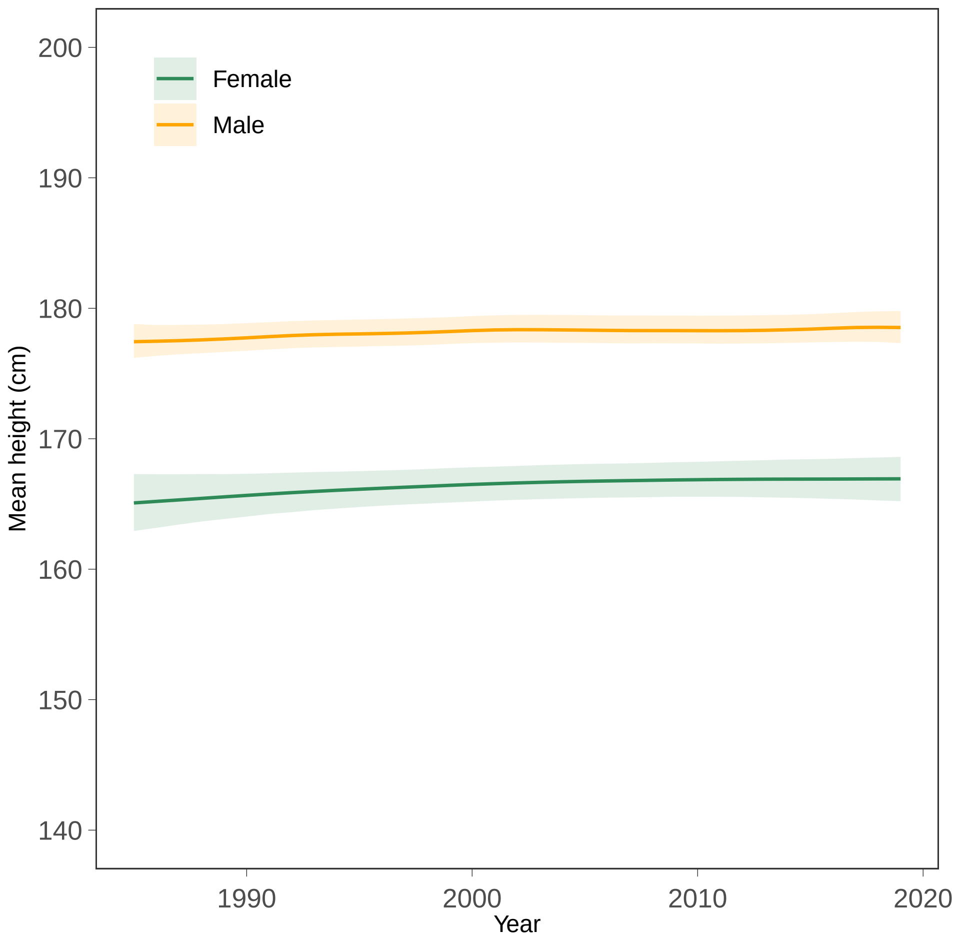

Time trends in BMI of 19 year olds

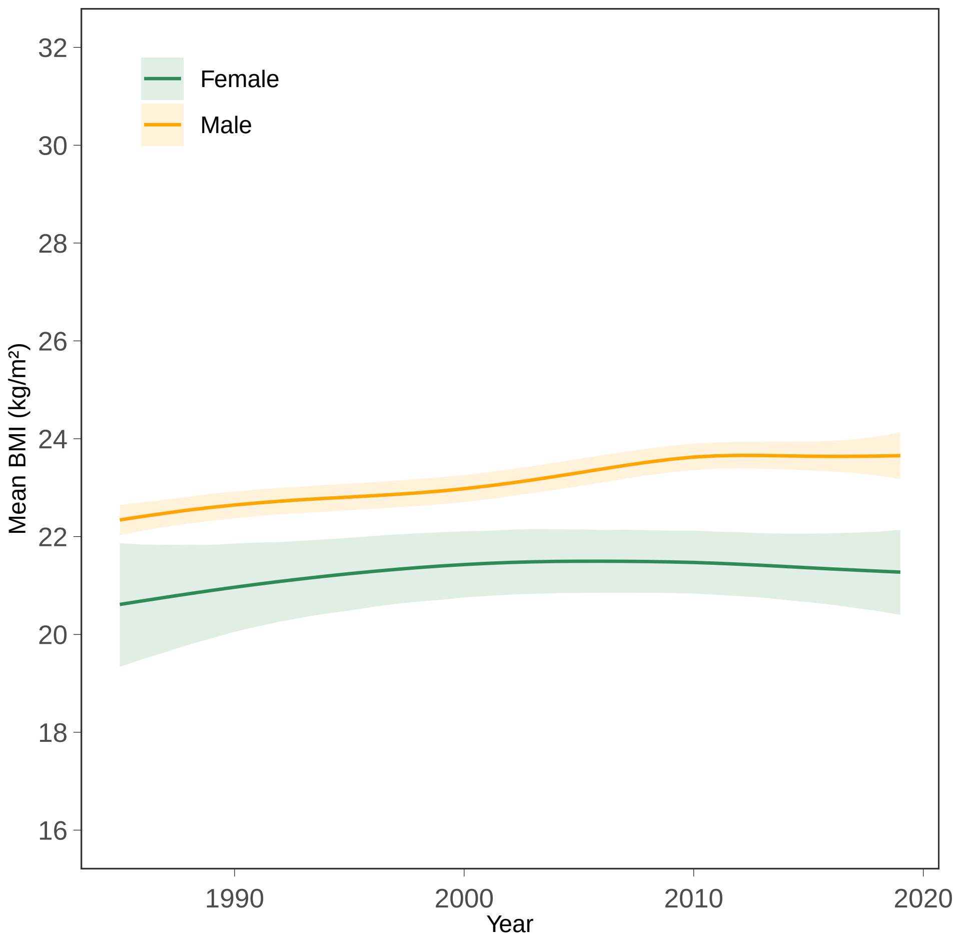

Height−for−age trajectories (2000 birth cohort)

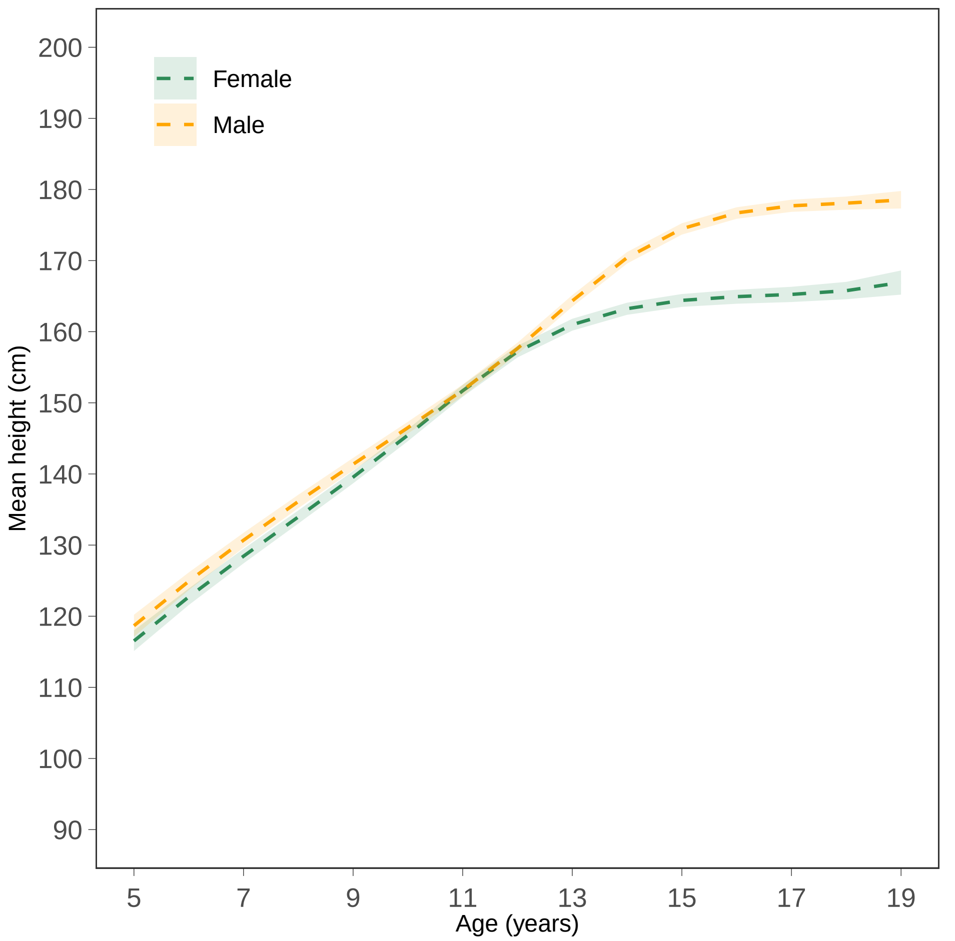

BMI−for−age trajectories (2000 birth cohort)

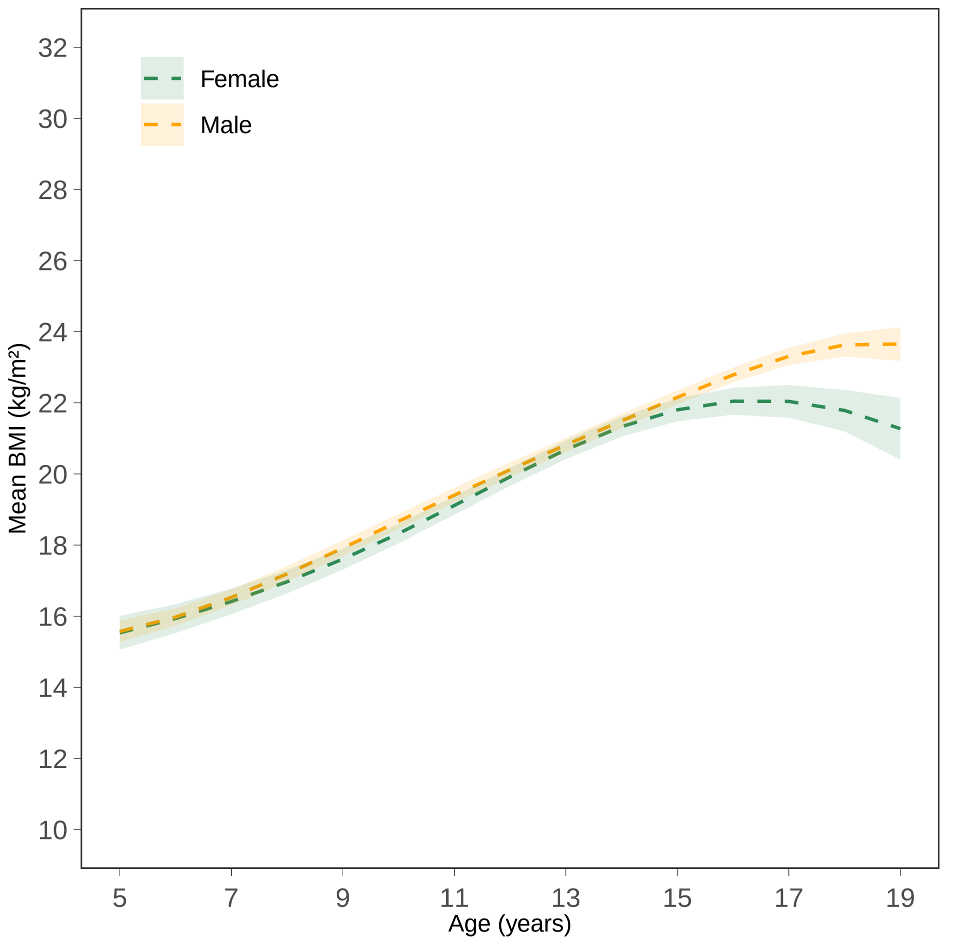

Azerbaijan

Time trends in height of 19 year olds

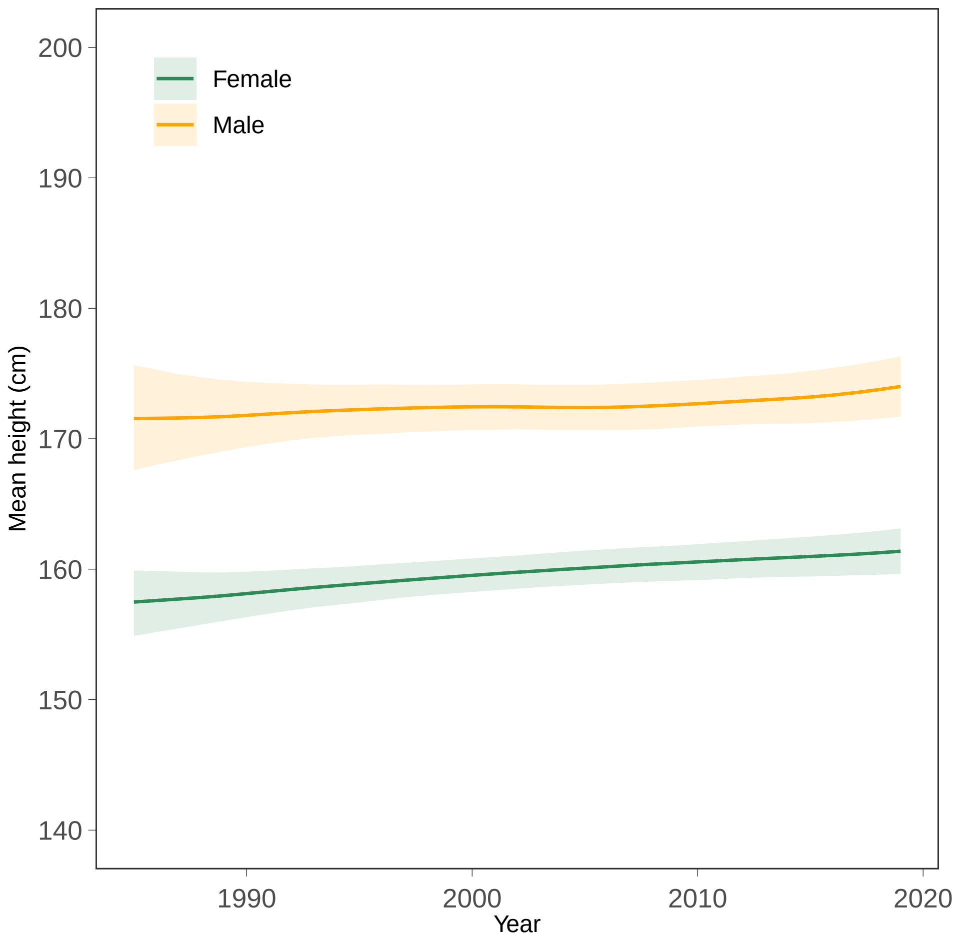

Time trends in BMI of 19 year olds

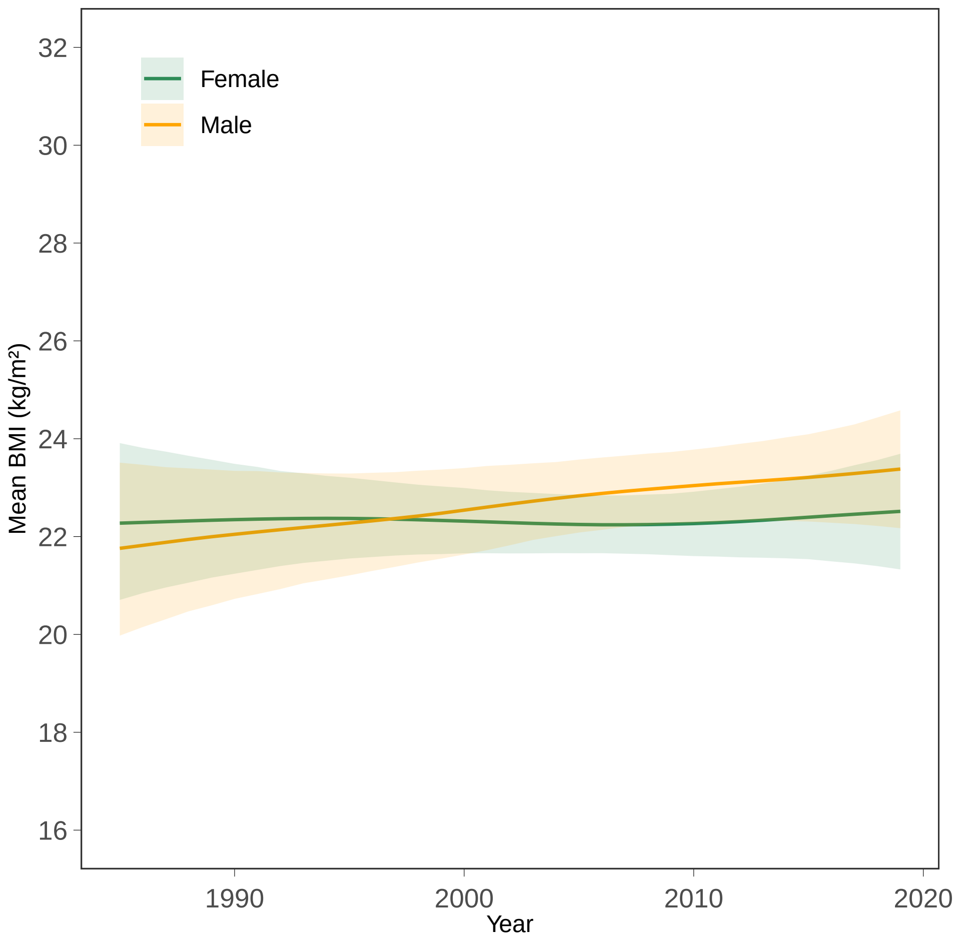

Height−for−age trajectories (2000 birth cohort)

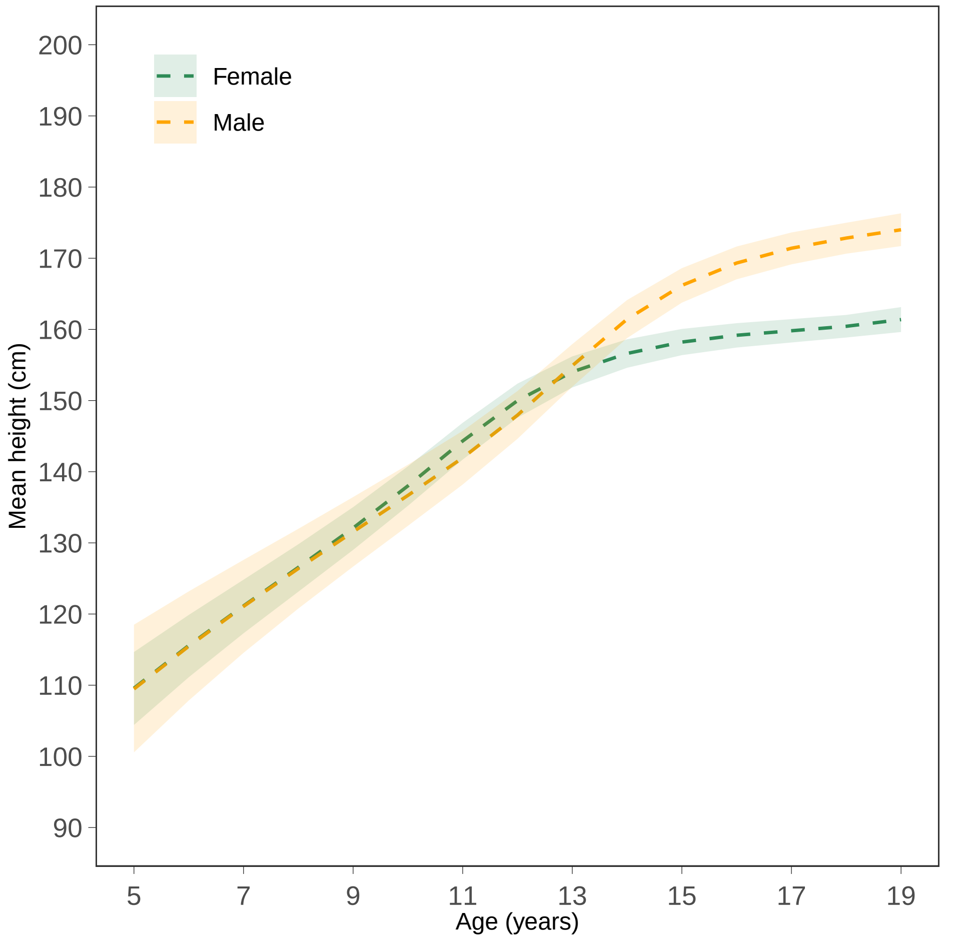

BMI−for−age trajectories (2000 birth cohort)

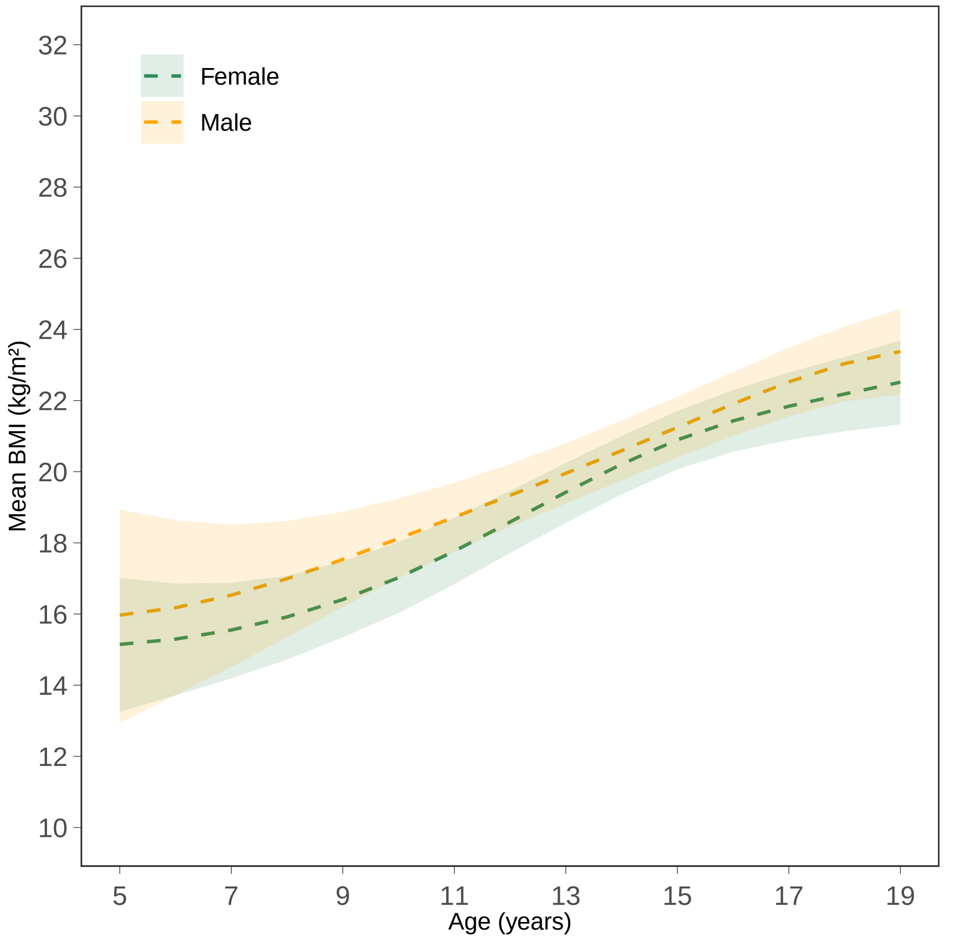

Bahamas

Time trends in height of 19 year olds

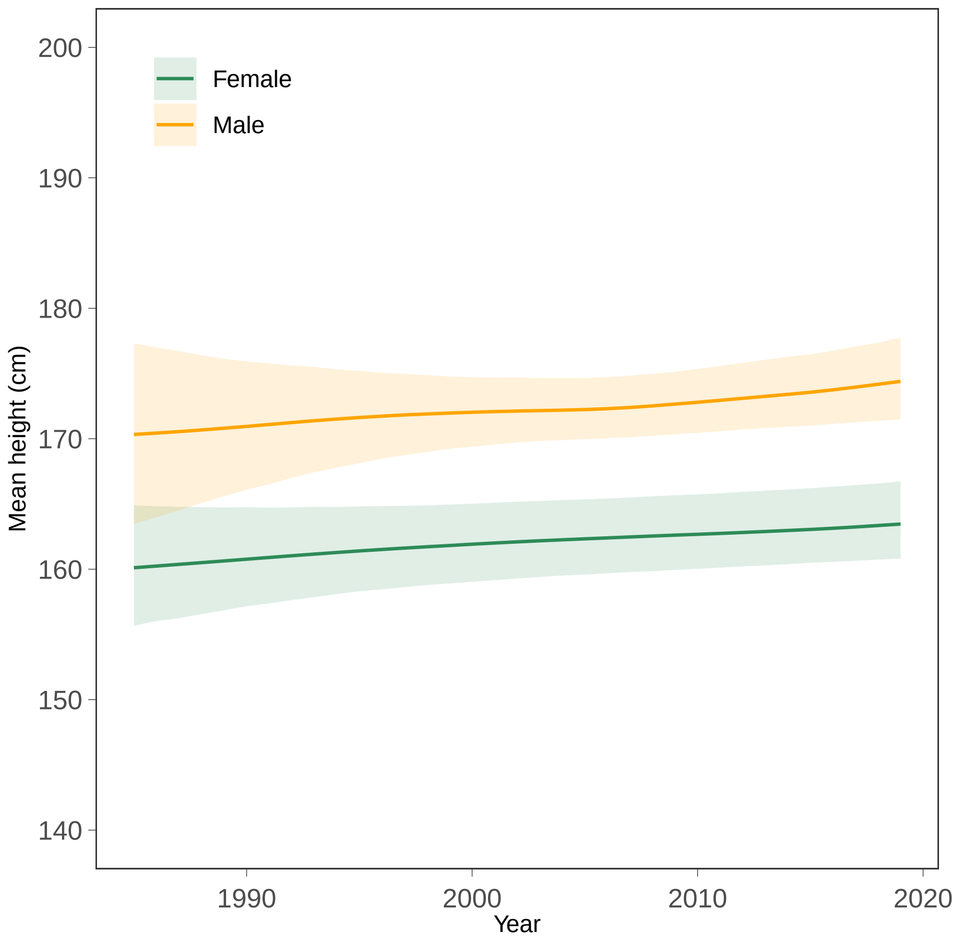

Time trends in BMI of 19 year olds

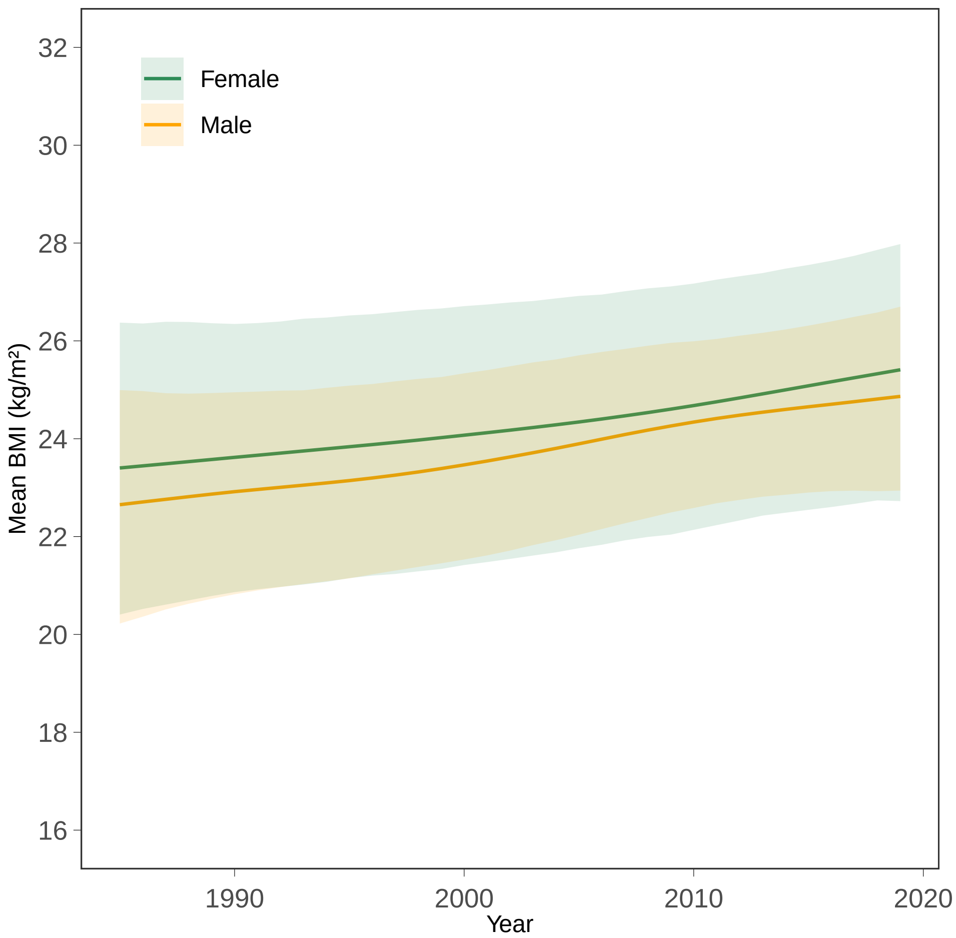

Height−for−age trajectories (2000 birth cohort)

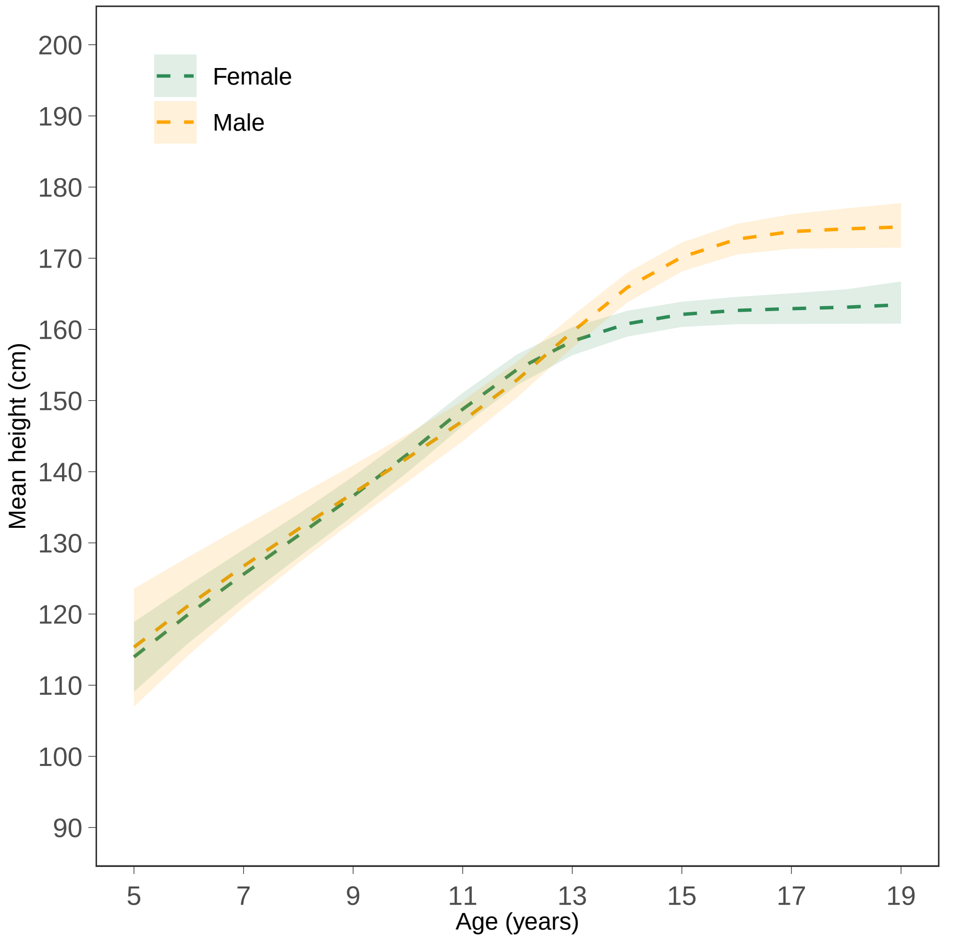

BMI−for−age trajectories (2000 birth cohort)

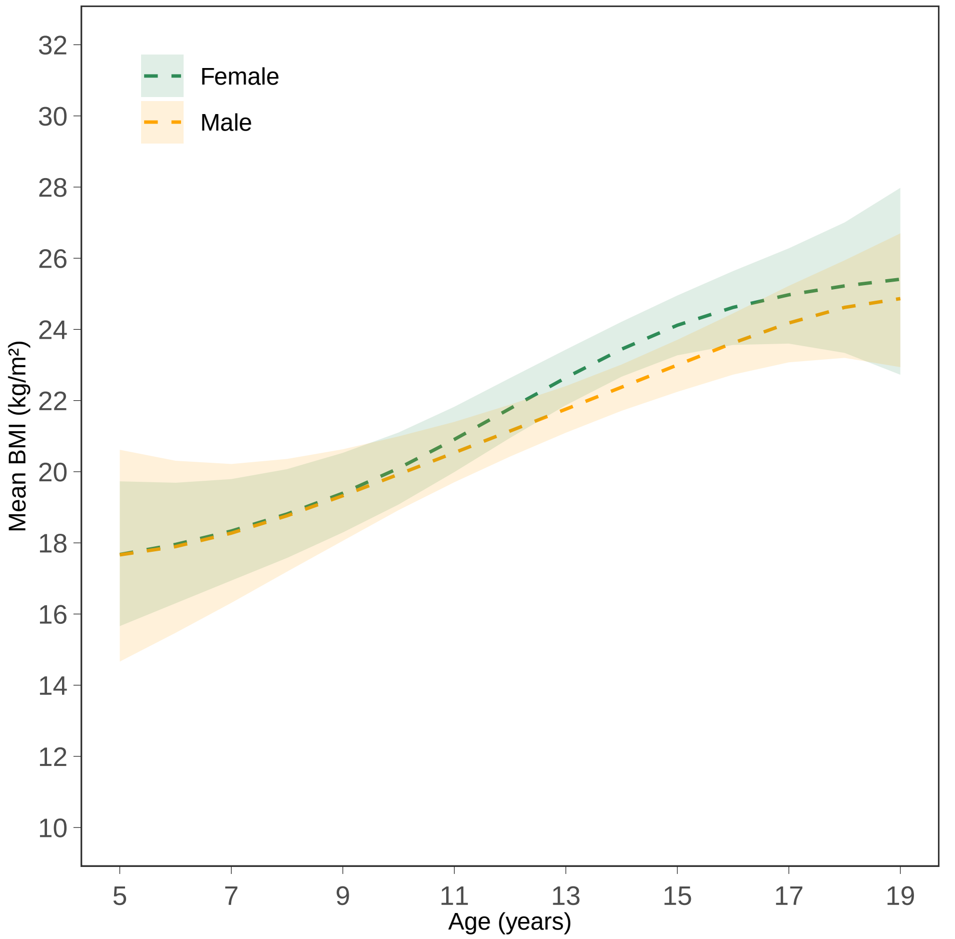

Bahrain

Time trends in height of 19 year olds

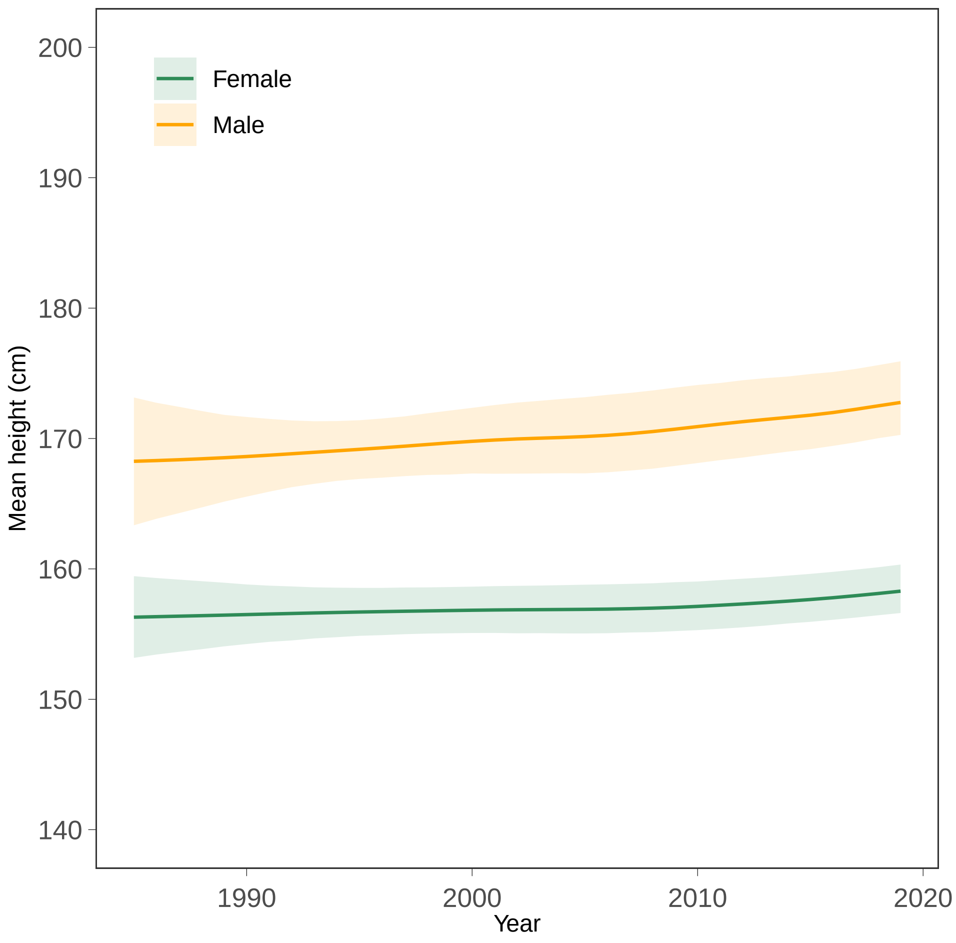

Time trends in BMI of 19 year olds

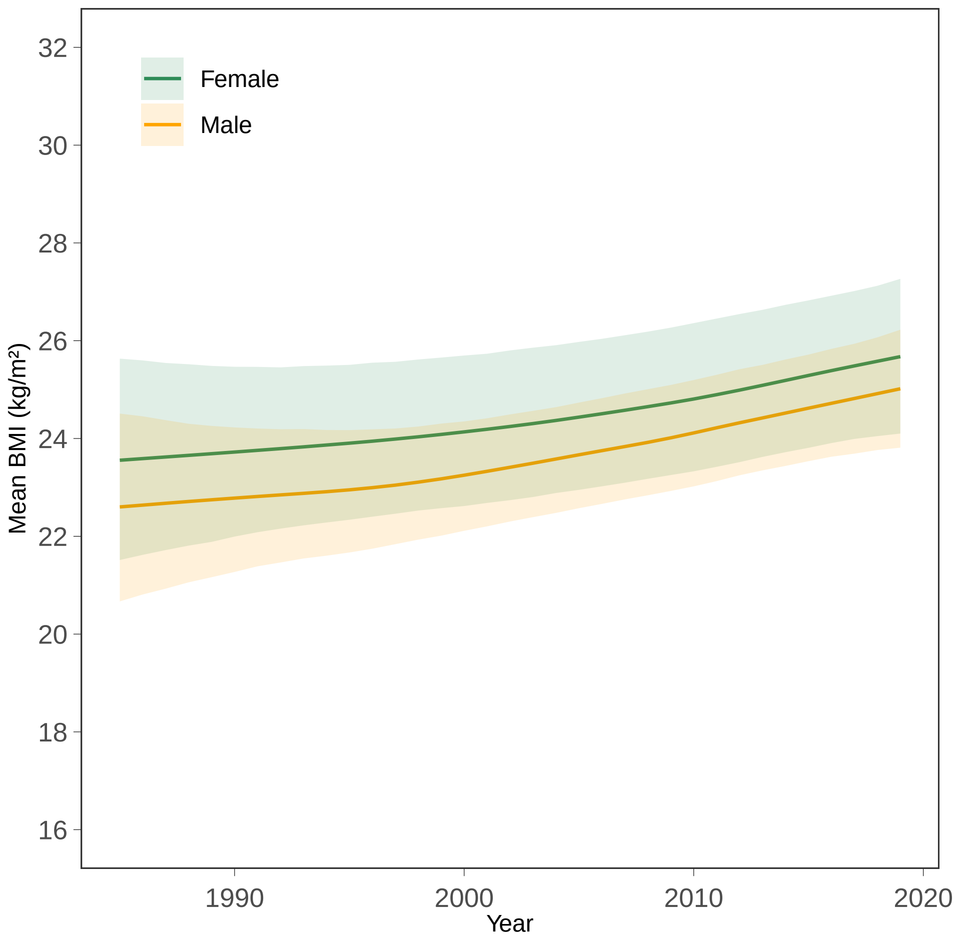

Height−for−age trajectories (2000 birth cohort)

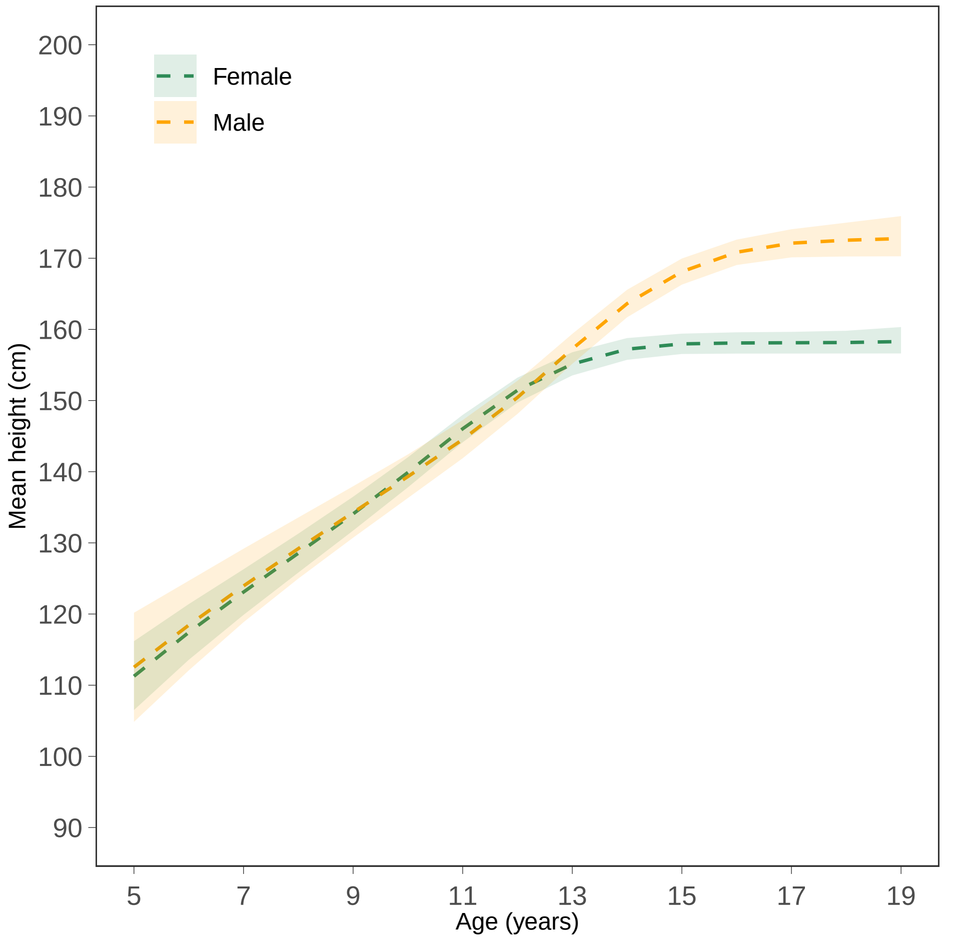

BMI−for−age trajectories (2000 birth cohort)

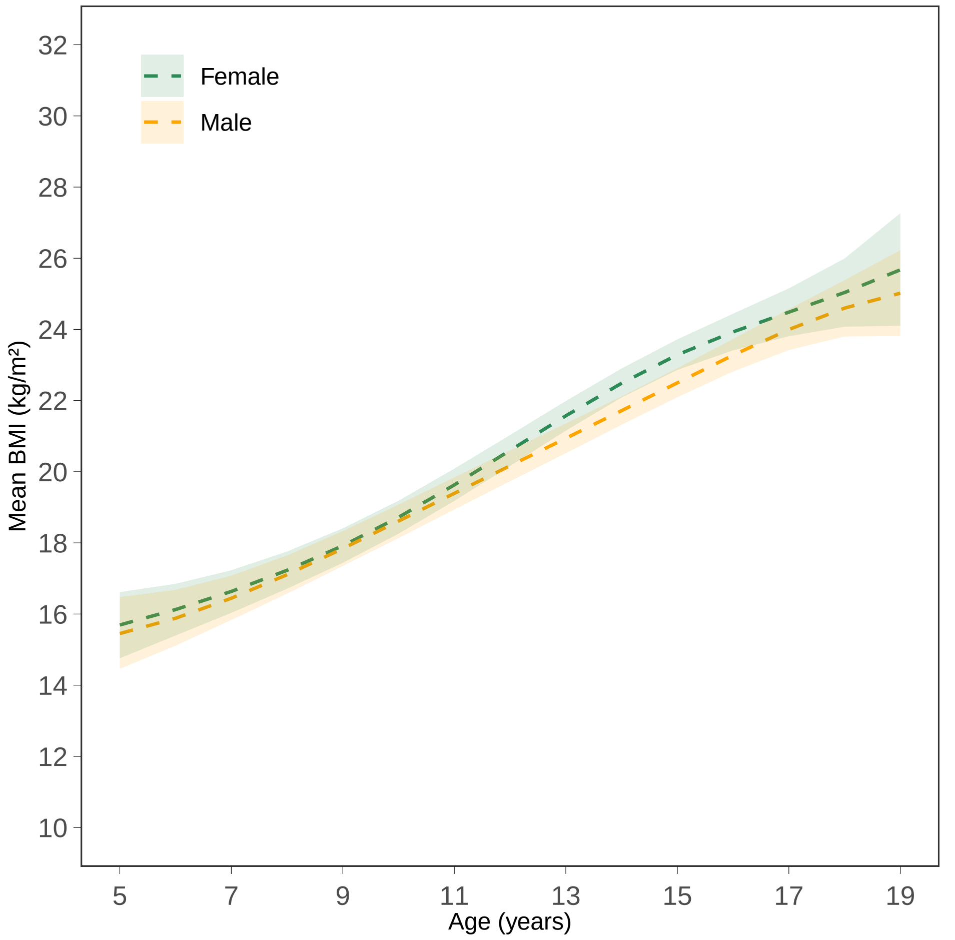

Bangladesh

Time trends in height of 19 year olds

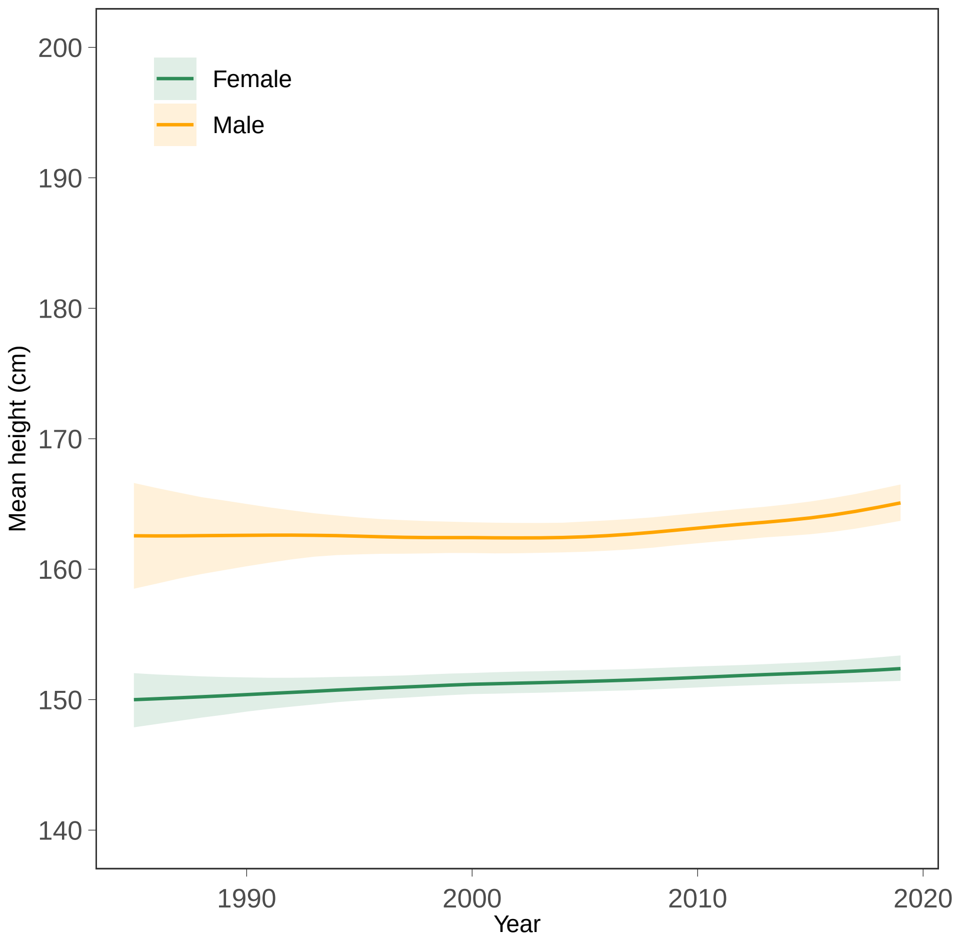

Time trends in BMI of 19 year olds

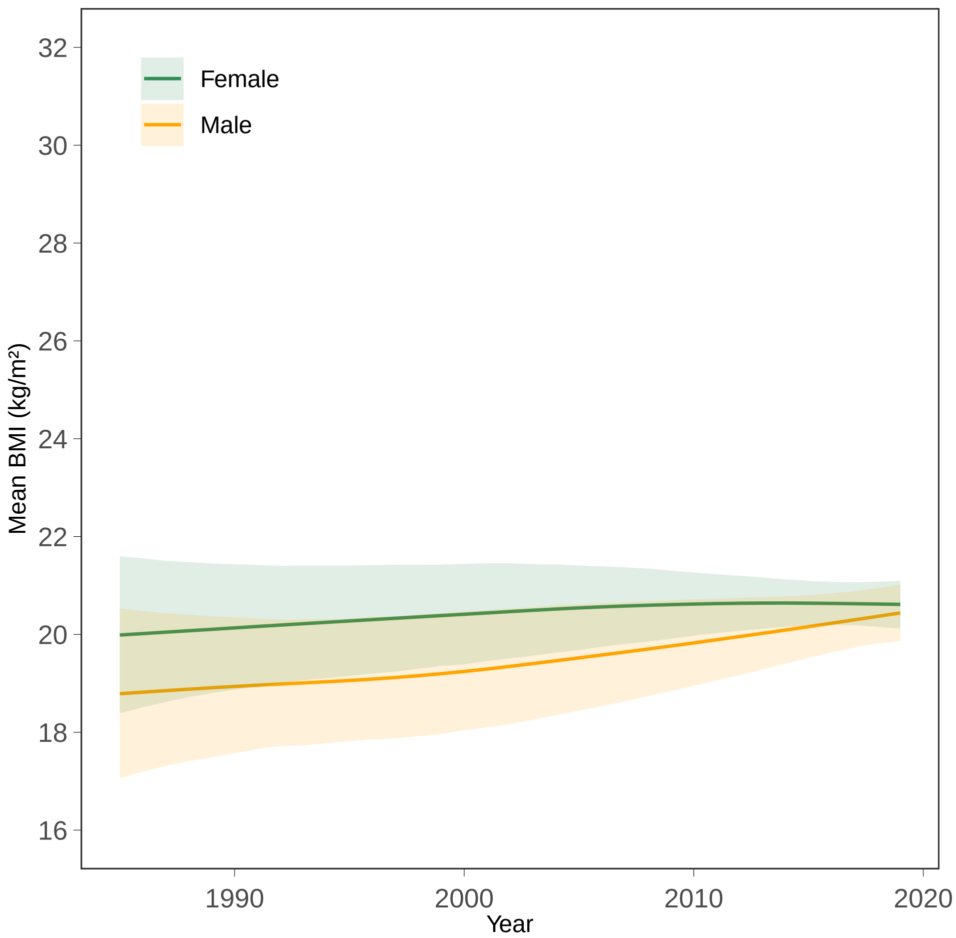

Height−for−age trajectories (2000 birth cohort)

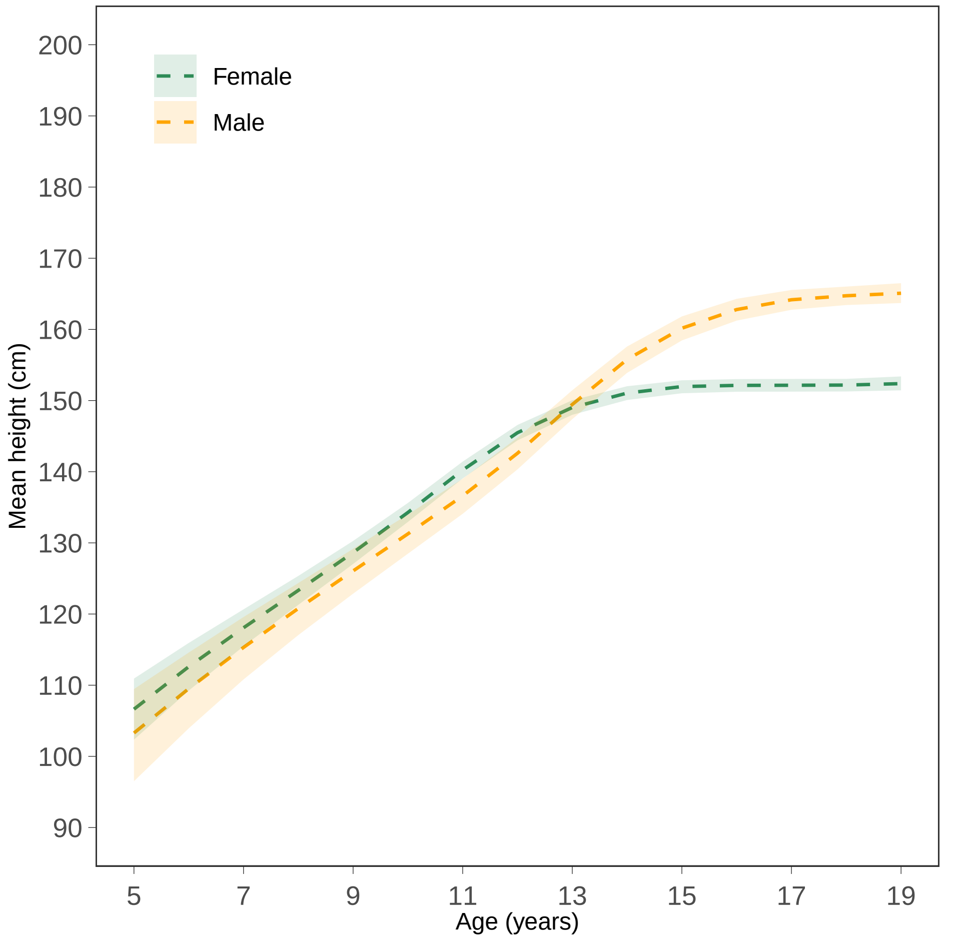

BMI−for−age trajectories (2000 birth cohort)

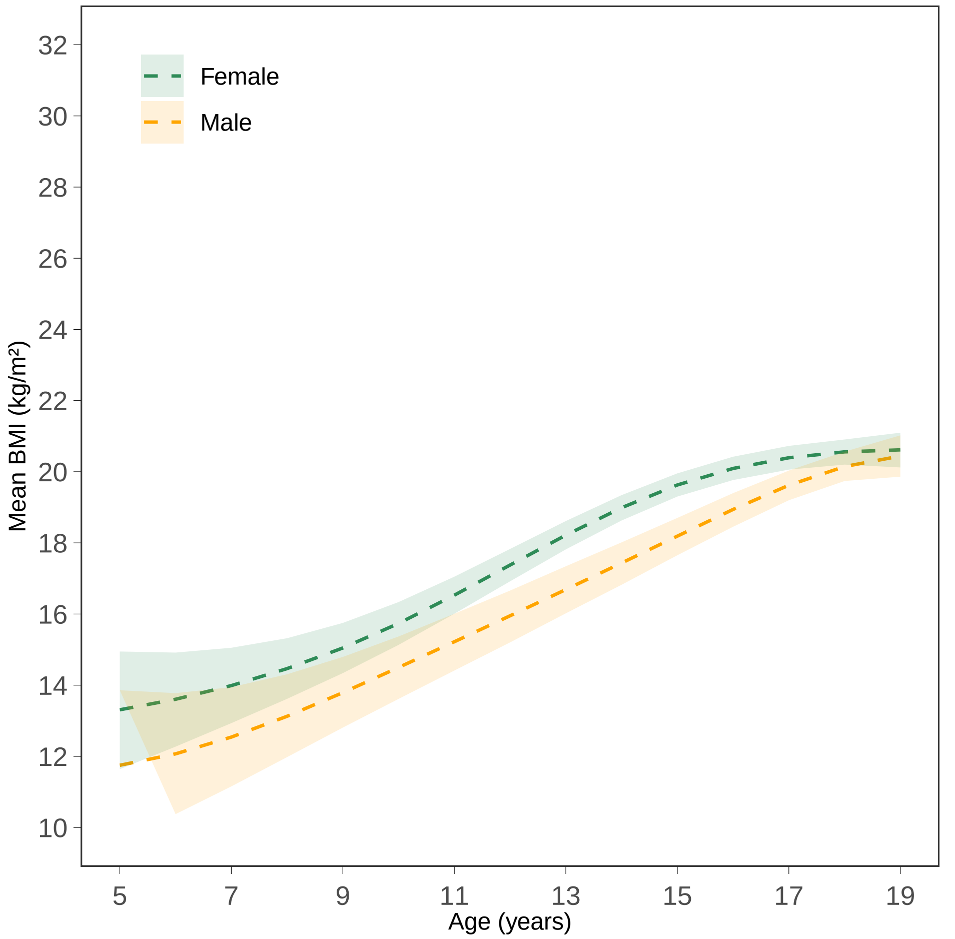

Barbados

Time trends in height of 19 year olds

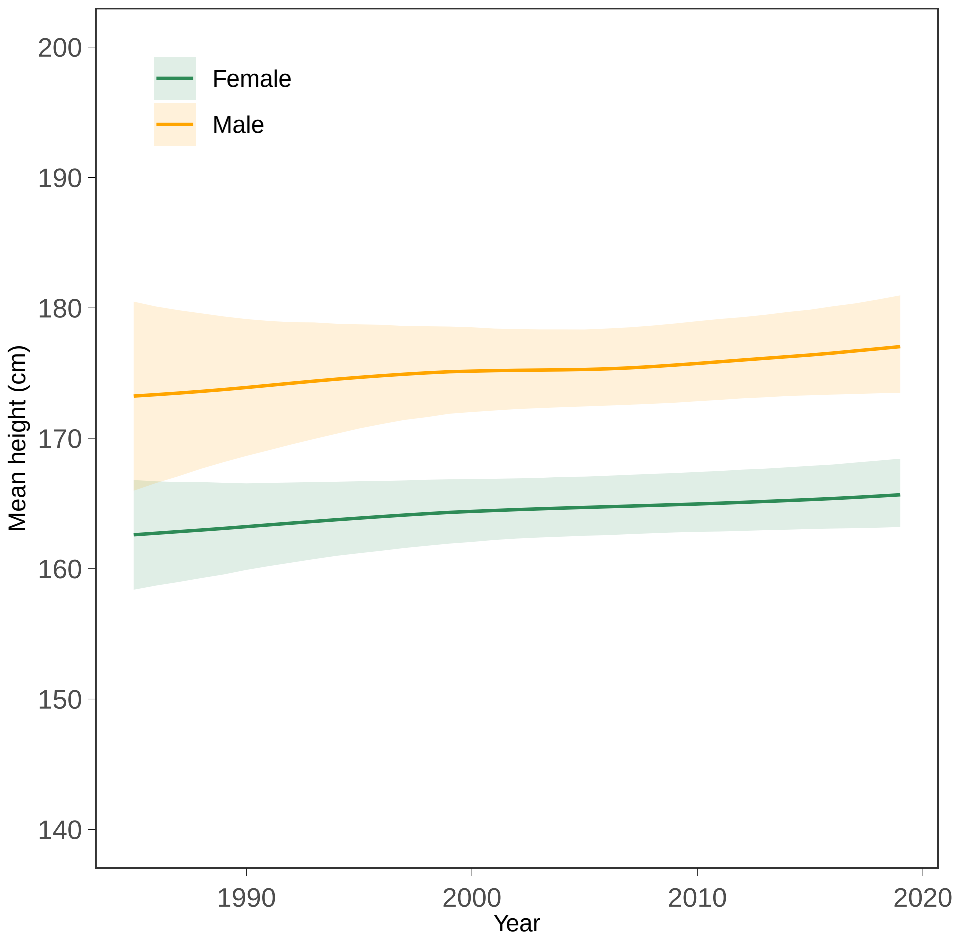

Time trends in BMI of 19 year olds

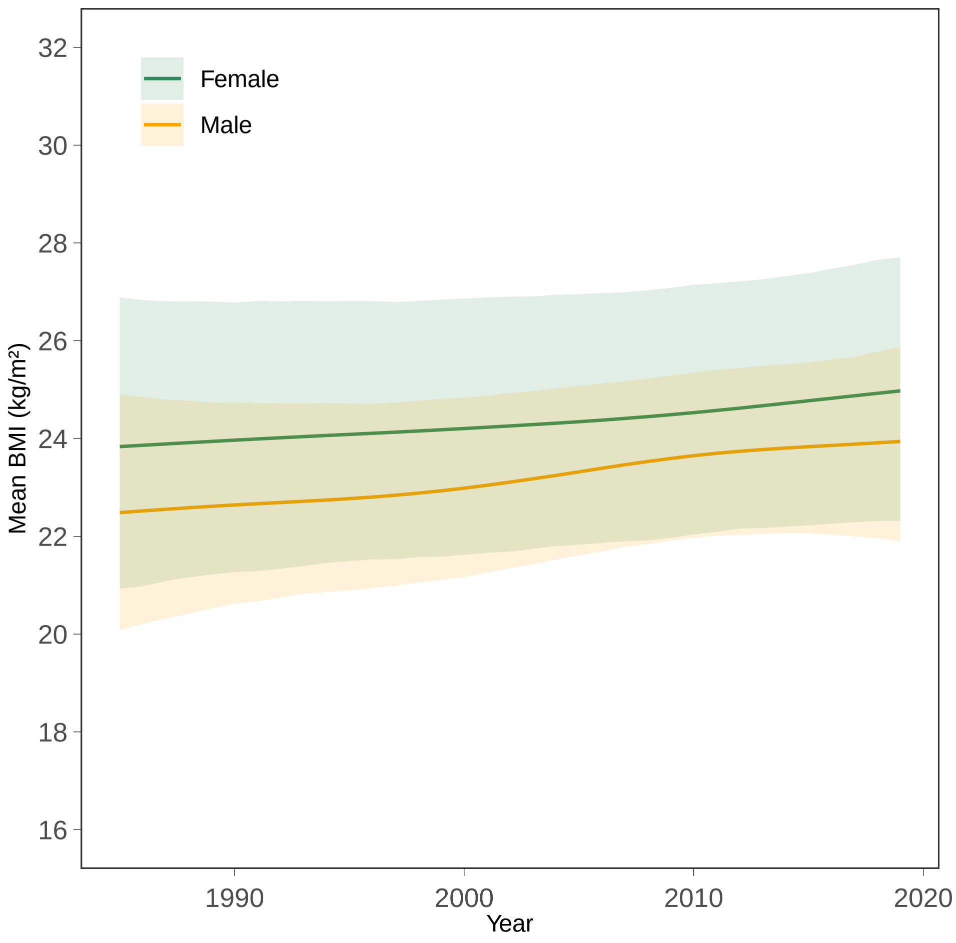

Height−for−age trajectories (2000 birth cohort)

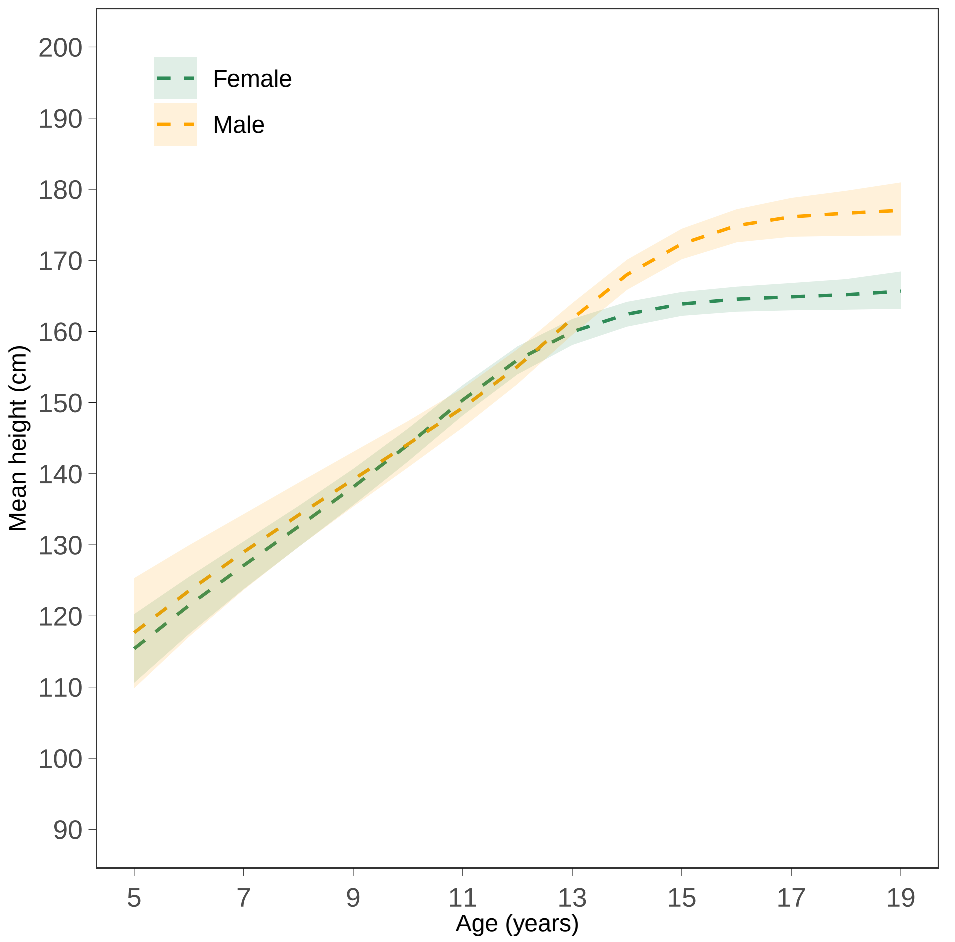

BMI−for−age trajectories (2000 birth cohort)

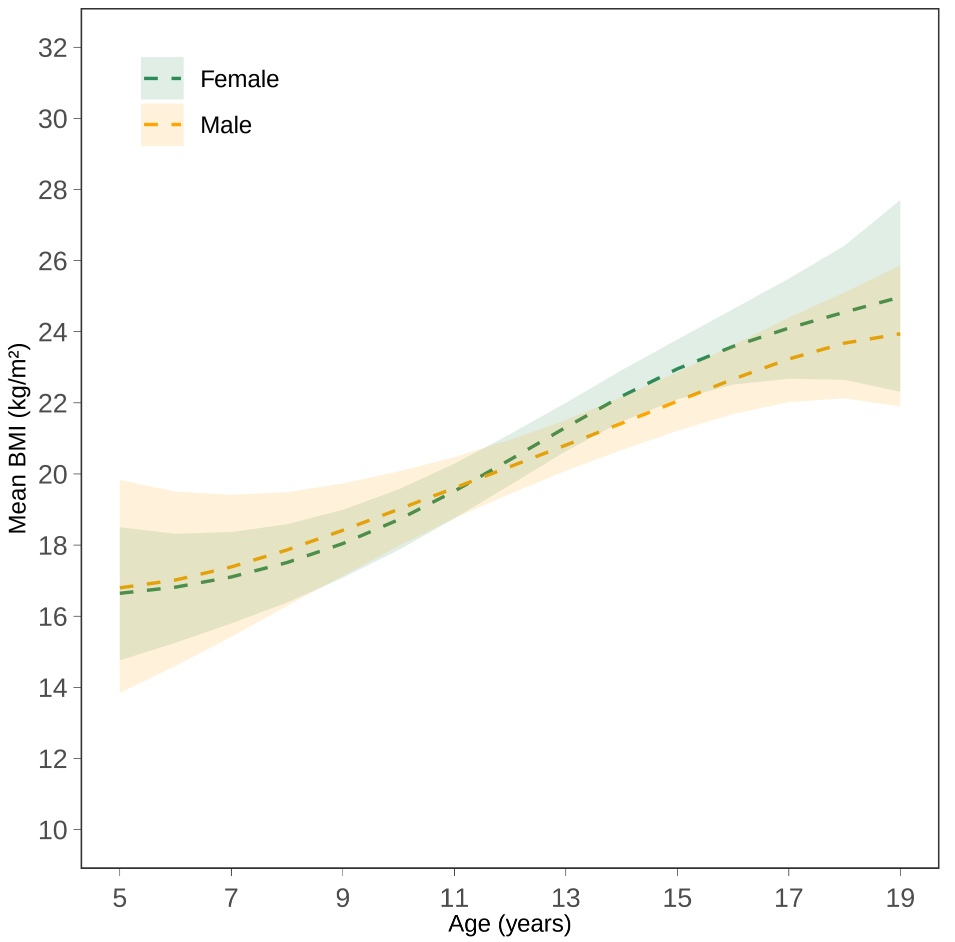

Belarus

Time trends in height of 19 year olds

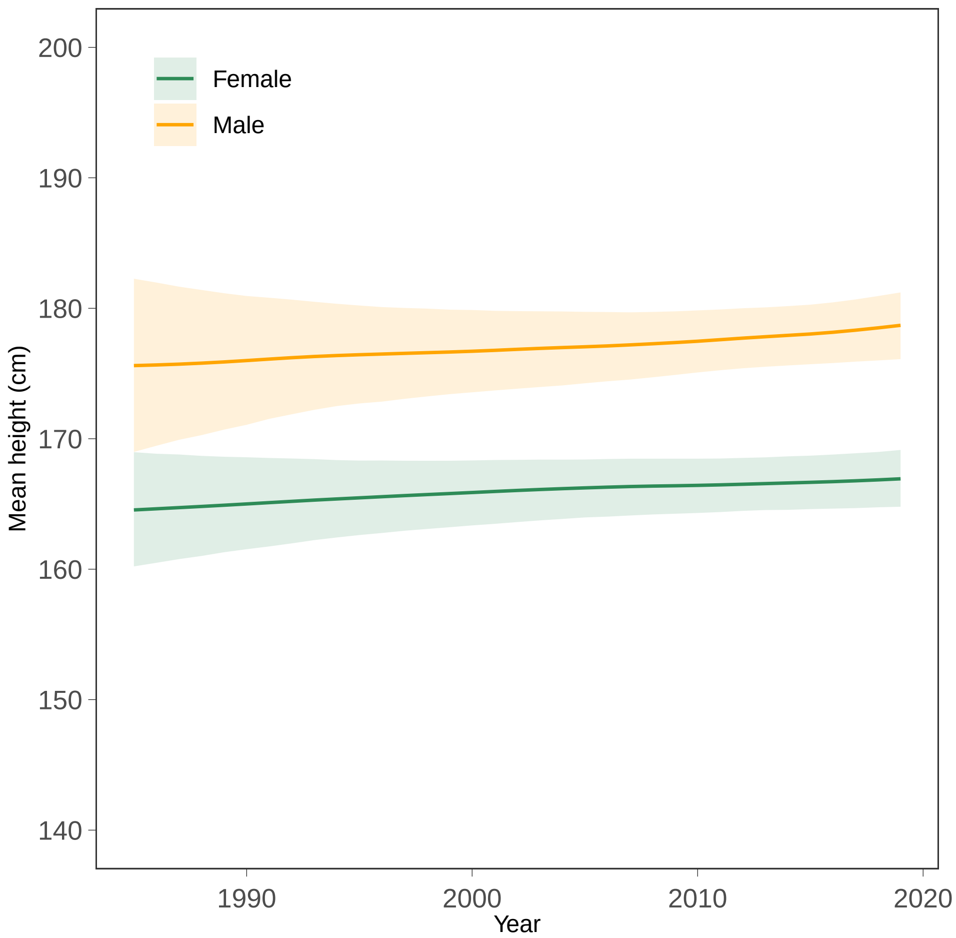

Time trends in BMI of 19 year olds

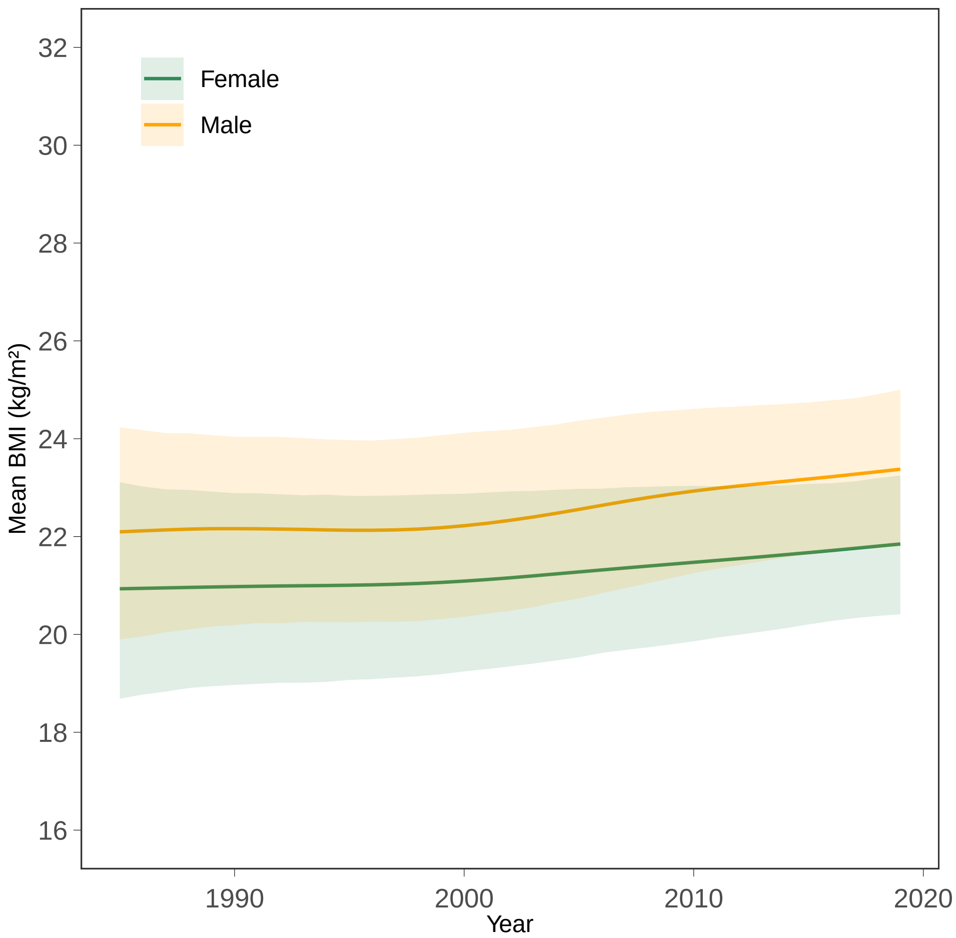

Height−for−age trajectories (2000 birth cohort)

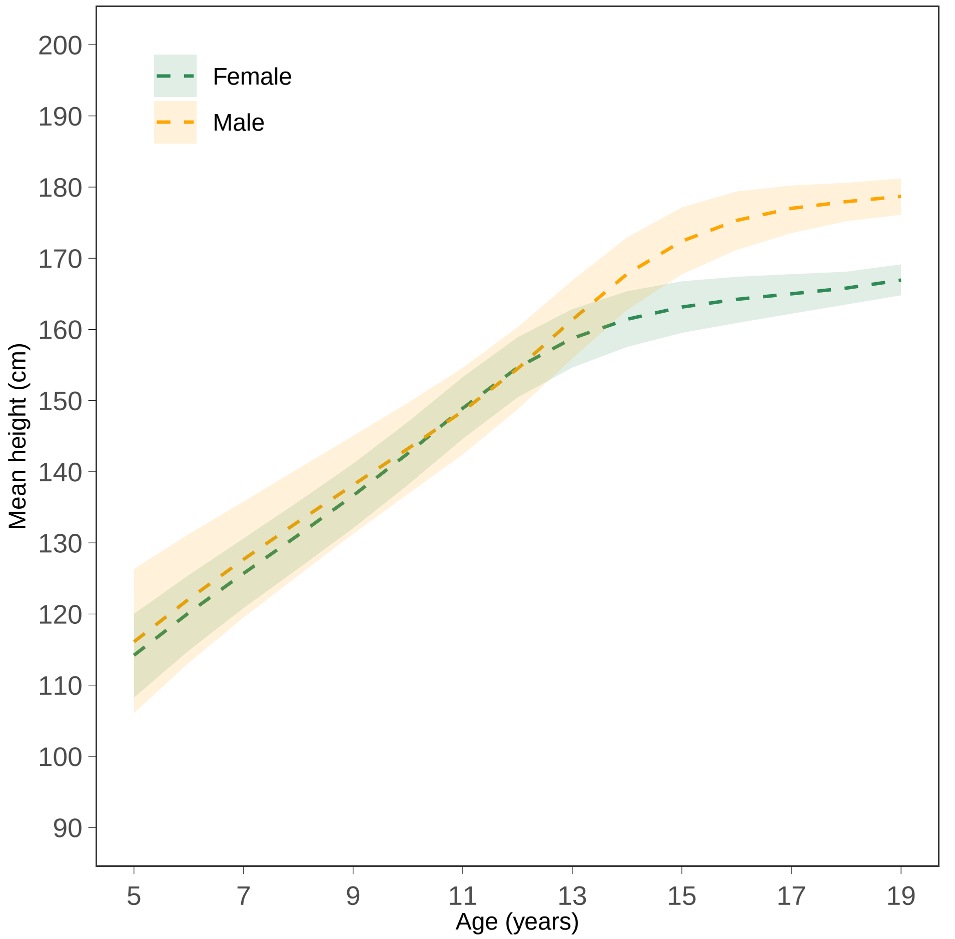

BMI−for−age trajectories (2000 birth cohort)

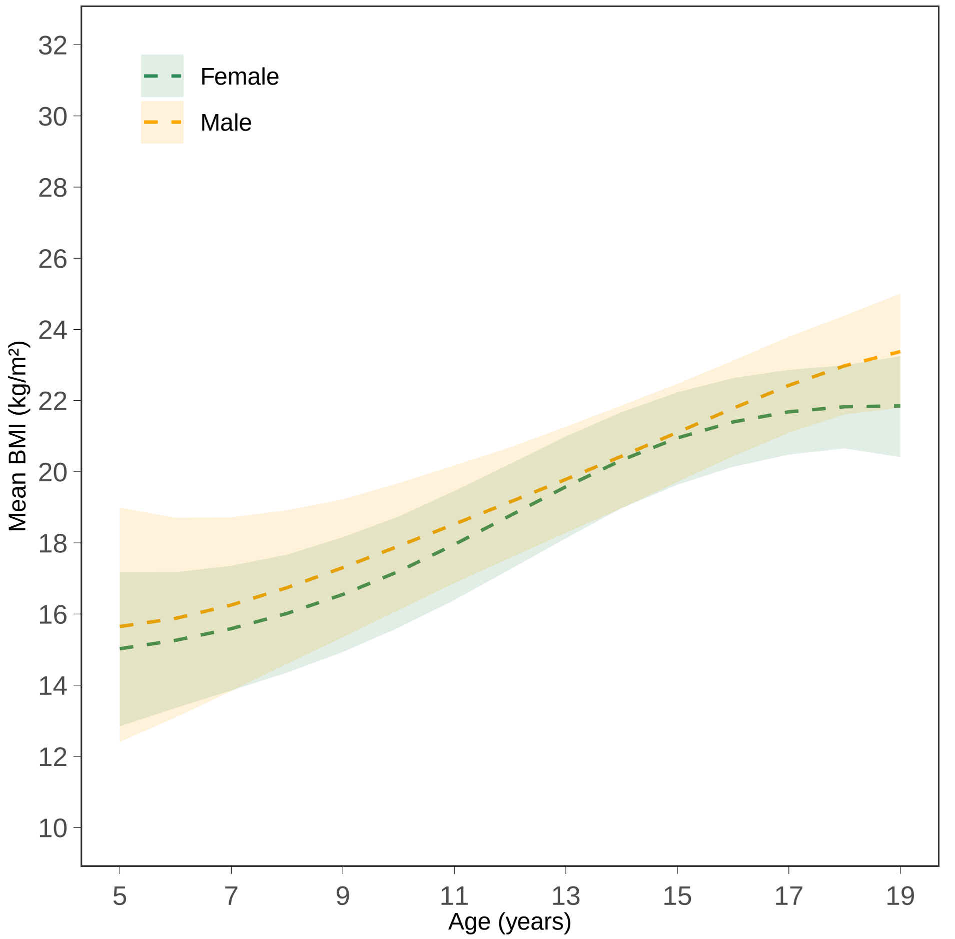

Belgium

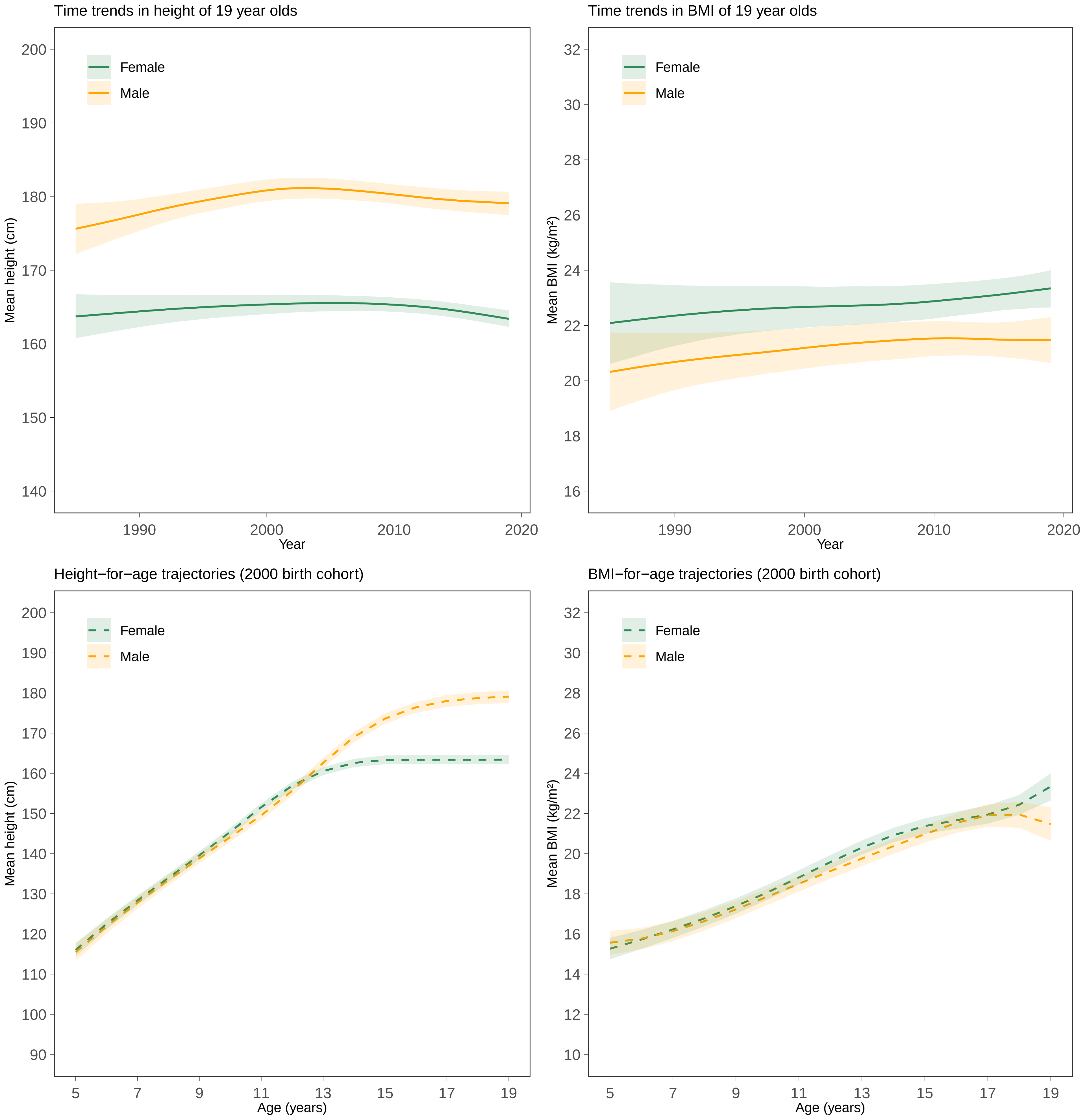
Time trends in height of 19 year olds
Female
Male
Mean height (cm)
200
190
180
170
160
150
140
1990
2000
2010
2020
Year
Time trends in BMI of 19 year olds
Female
Male
Mean BMI (kg/m²)
32
30
28
26
24
22
20
18
16
1990
2000
2010
2020
Year
Height−for−age trajectories (2000 birth cohort)
Female
Male
Mean height (cm)
200
190
180
170
160
150
140
130
120
110
100
90
5
7
9
11
13
15
17
19
Age (years)
BMI−for−age trajectories (2000 birth cohort)
Female
Male
Mean BMI (kg/m²)
32
30
28
26
24
22
20
18
16
14
12
10
5
7
9
11
13
15
17
19
Age (years)

Belize

Time trends in height of 19 year olds

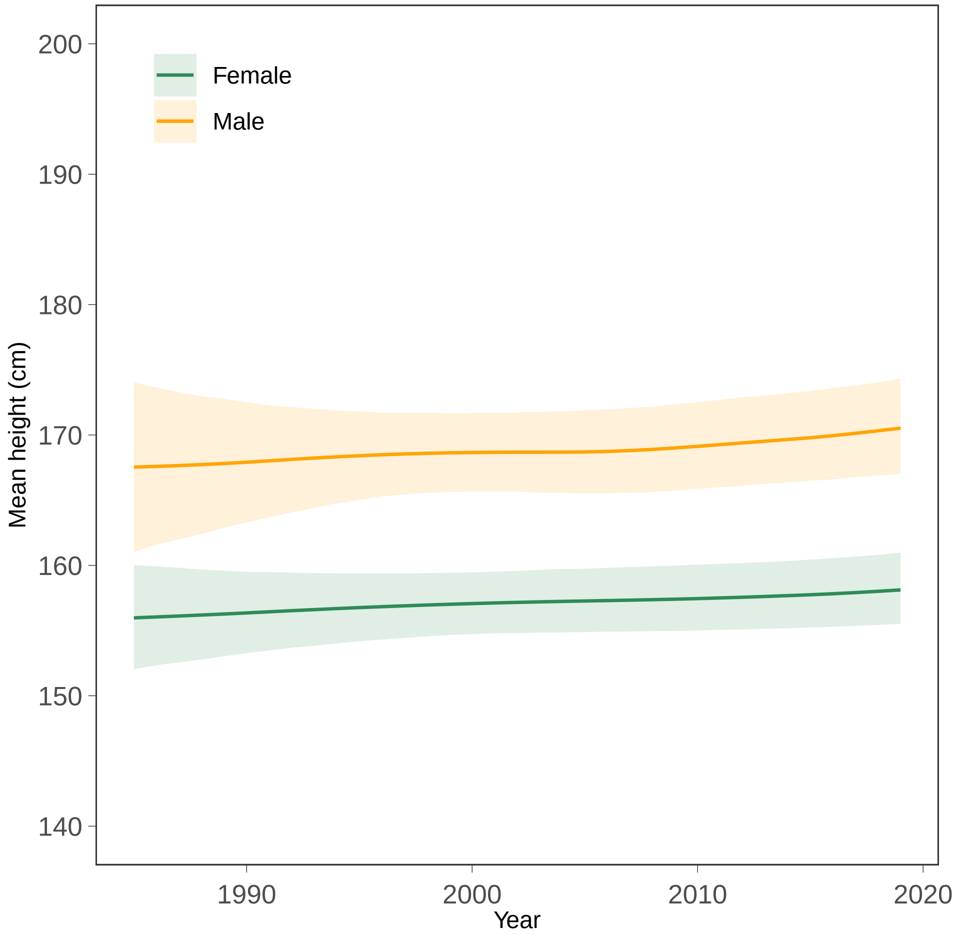

Time trends in BMI of 19 year olds

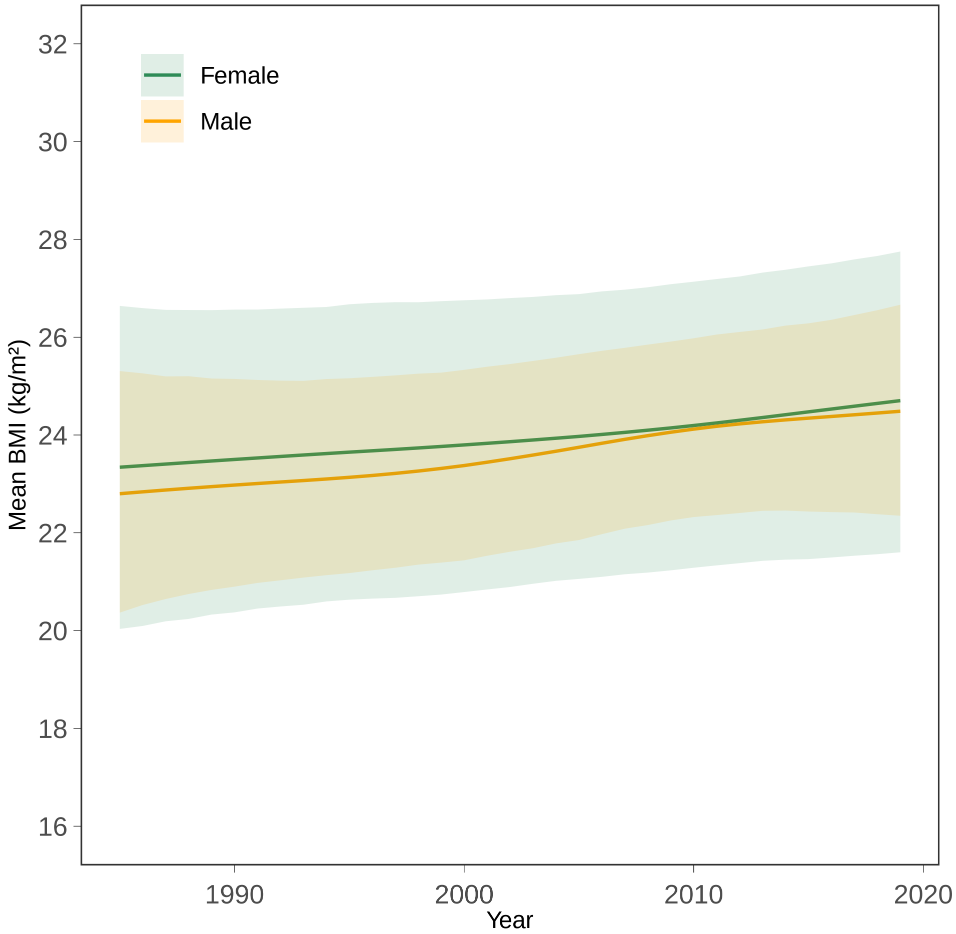

Height−for−age trajectories (2000 birth cohort)

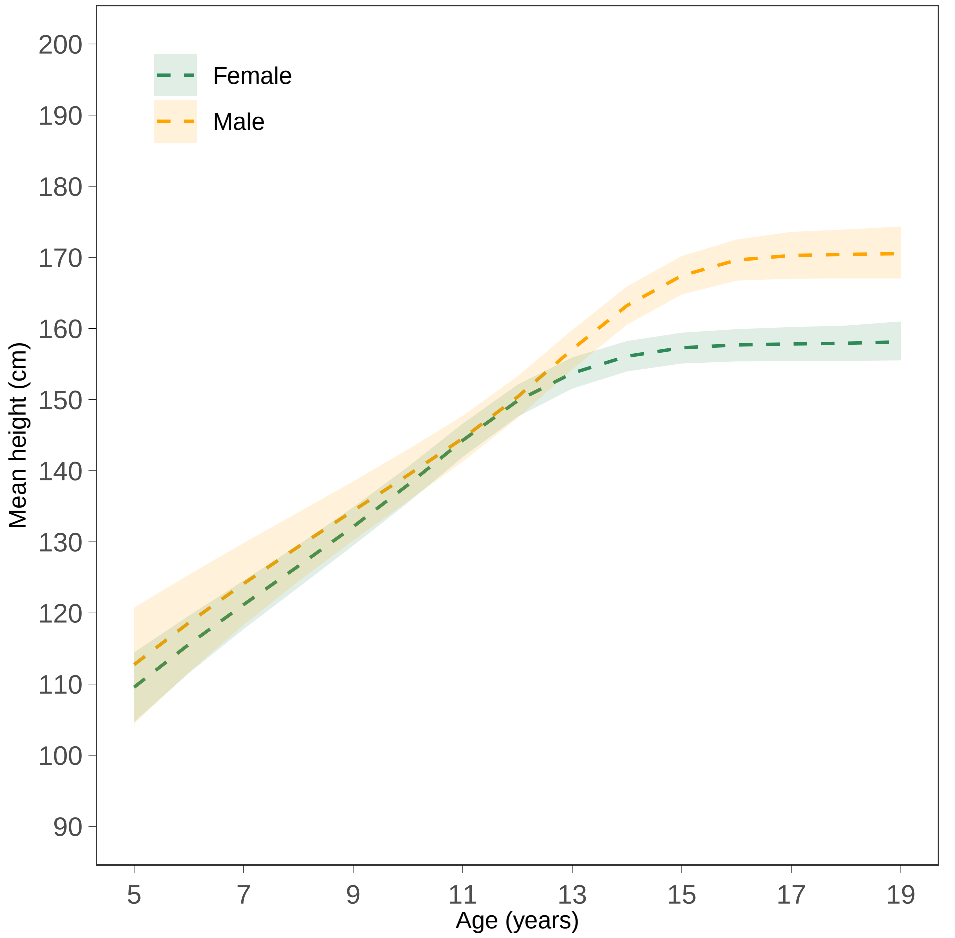

BMI−for−age trajectories (2000 birth cohort)

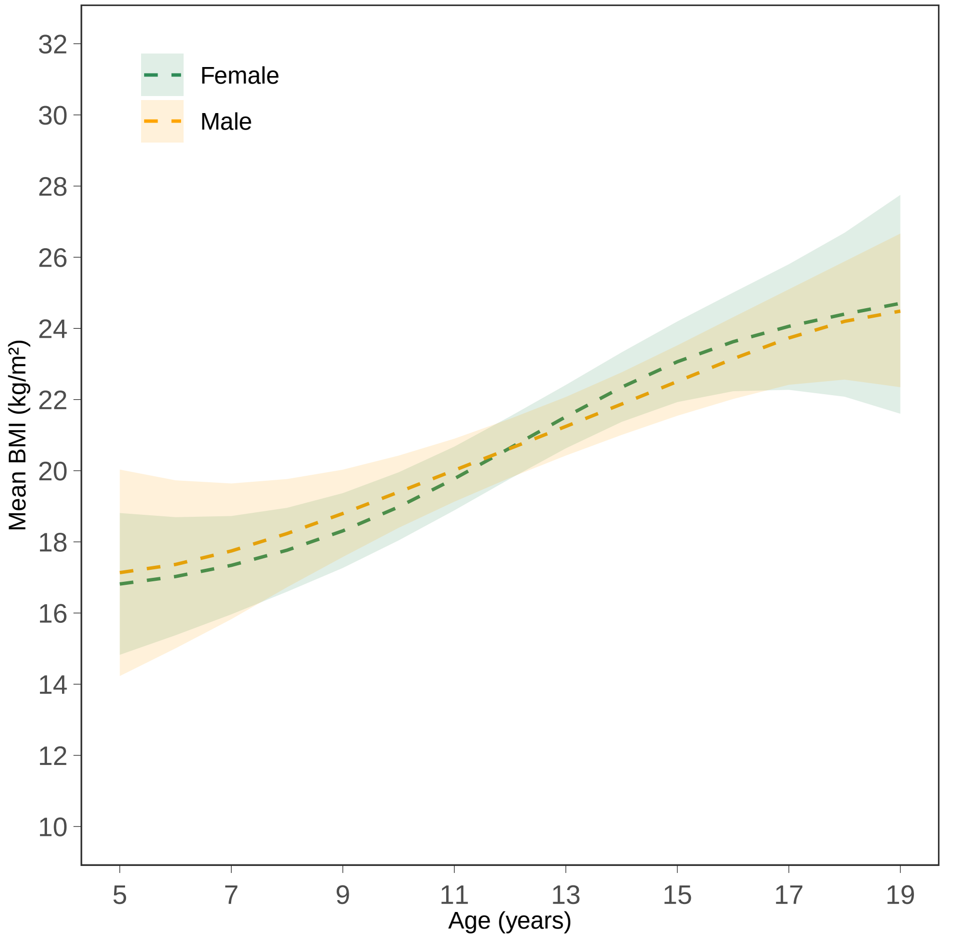

Benin

Time trends in height of 19 year olds

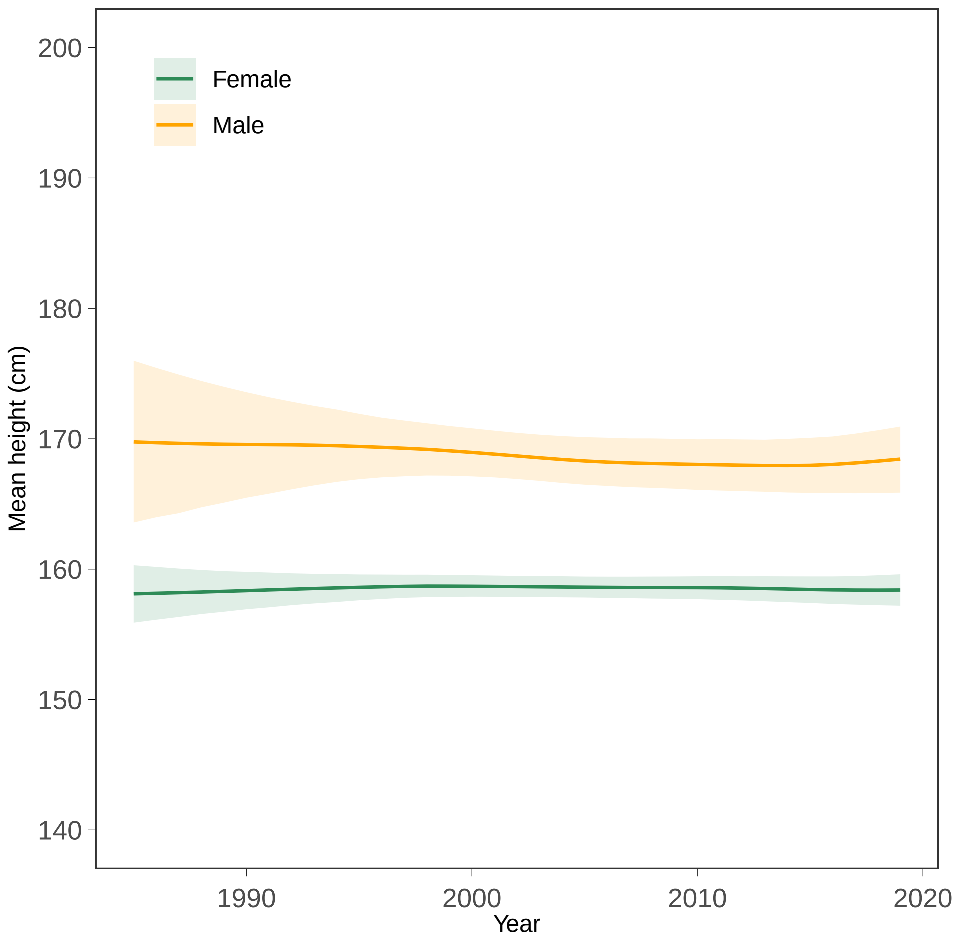

Time trends in BMI of 19 year olds

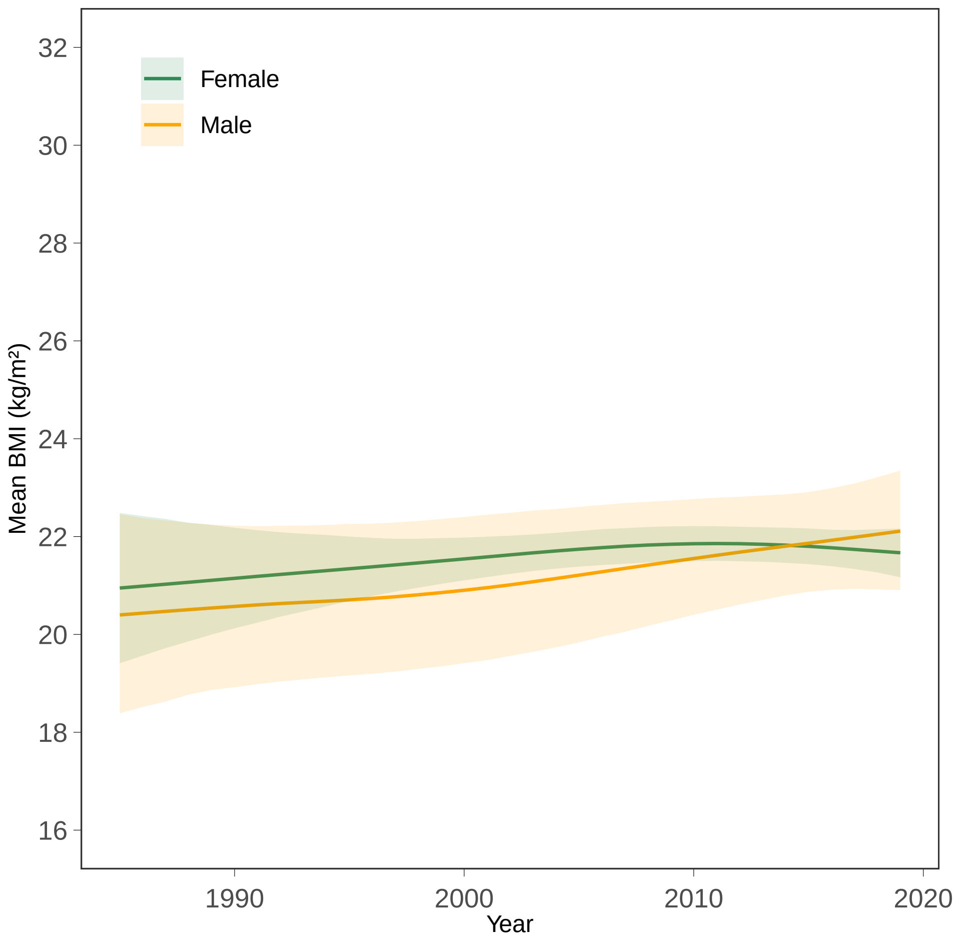

Height−for−age trajectories (2000 birth cohort)

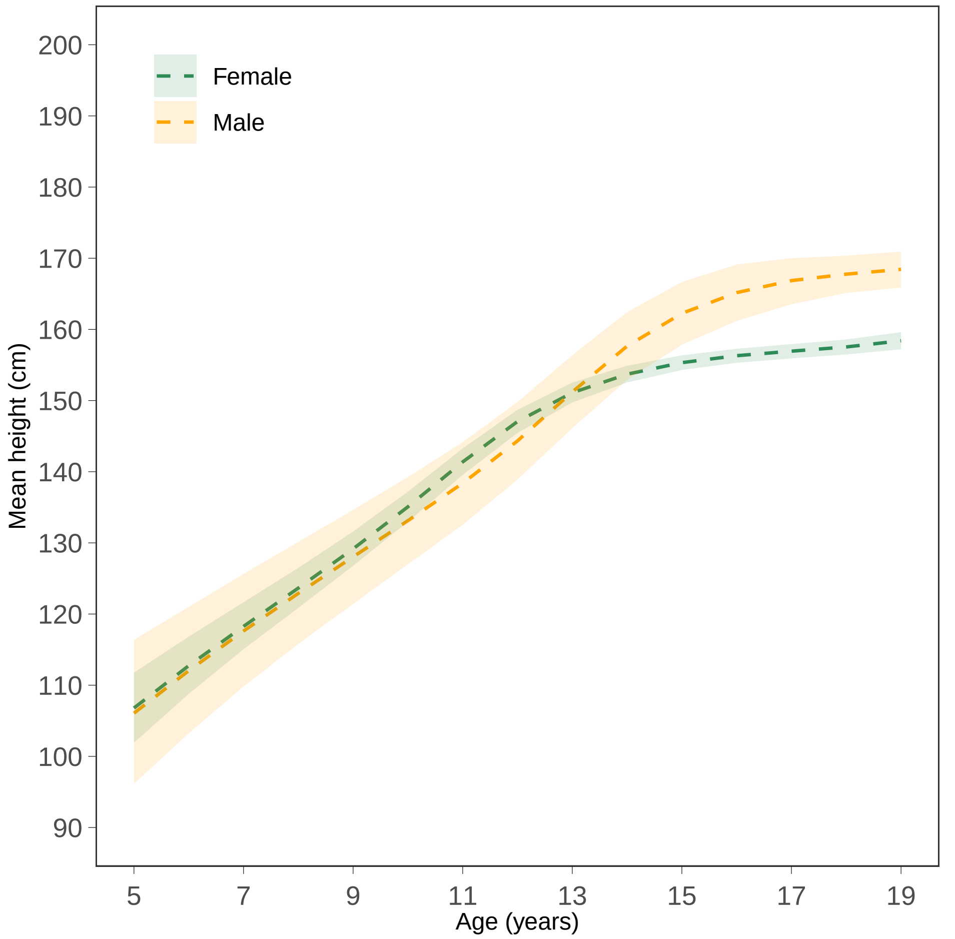

BMI−for−age trajectories (2000 birth cohort)

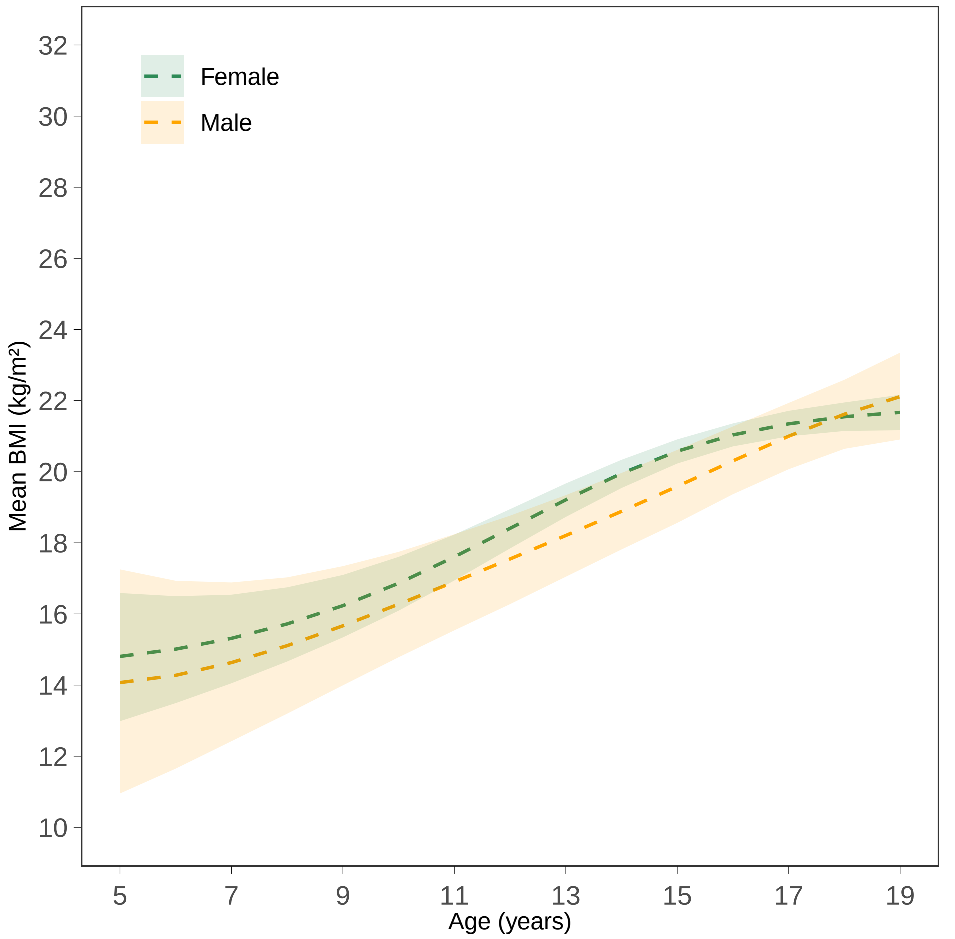

Bermuda

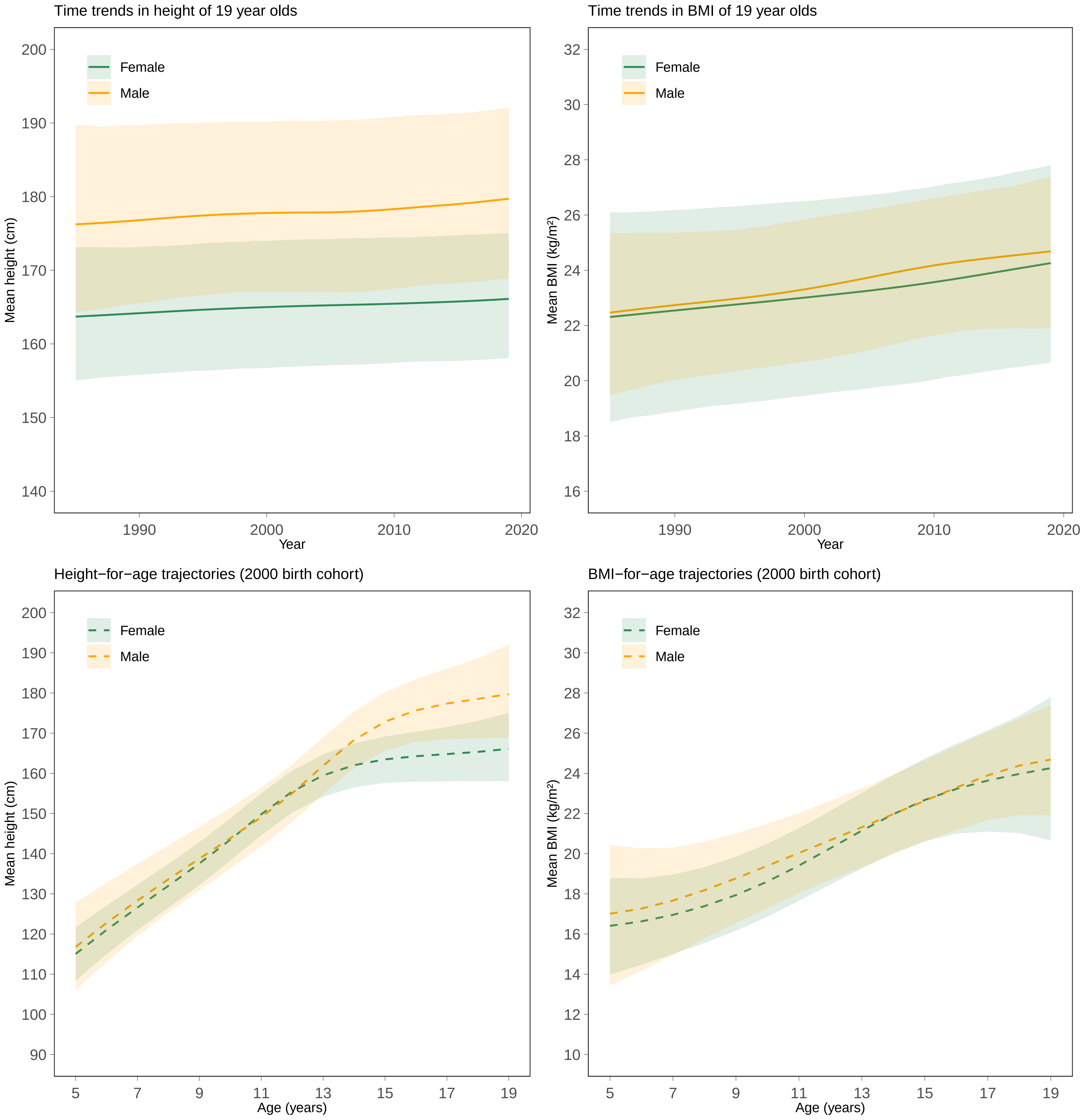

Time trends in height of 19 year olds
Female
Male
Mean height (cm)
Year
200
190
180
170
160
150
140
1990
2000
2010
2020
Time trends in BMI of 19 year olds
Mean BMI (kg/m²)
32
30
28
26
24
22
20
18
16
Height−for−age trajectories (2000 birth cohort)
Mean height (cm)
Age (years)
100
90
5
7
9
11
13
15
17
19
BMI−for−age trajectories (2000 birth cohort)
Mean BMI (kg/m²)
14
12
10

Bhutan

Time trends in height of 19 year olds

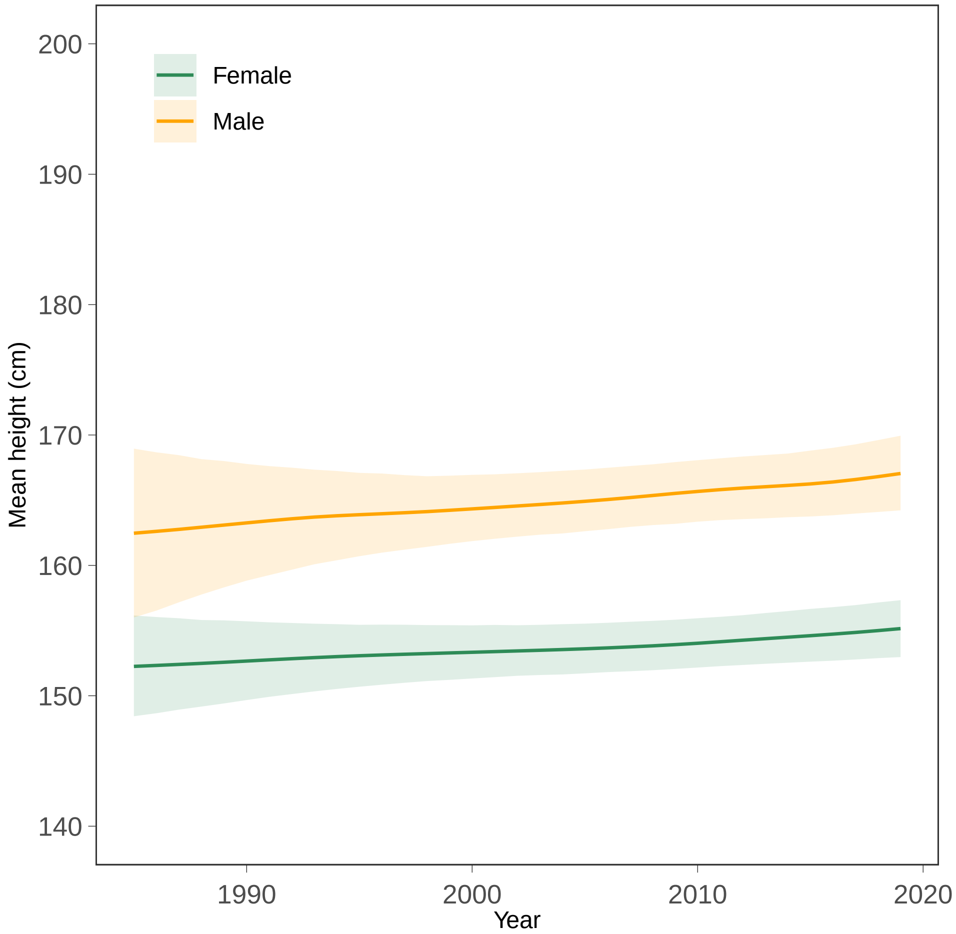

Time trends in BMI of 19 year olds

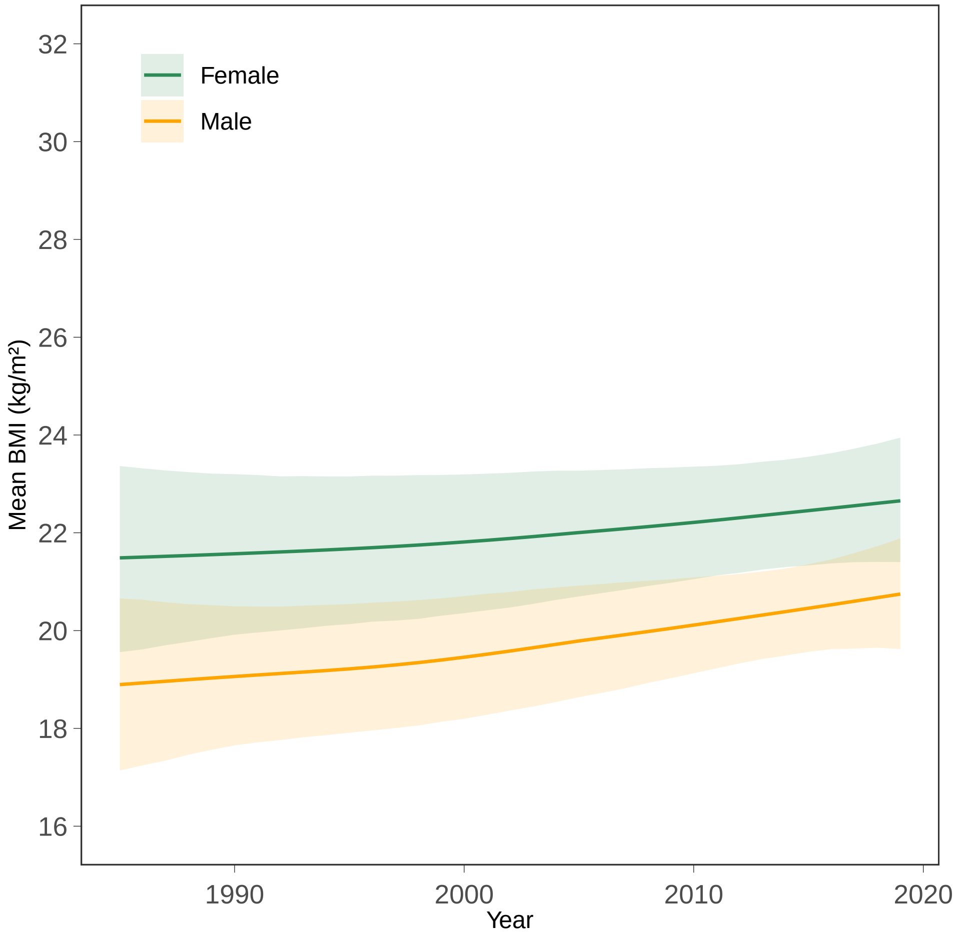

Height−for−age trajectories (2000 birth cohort)

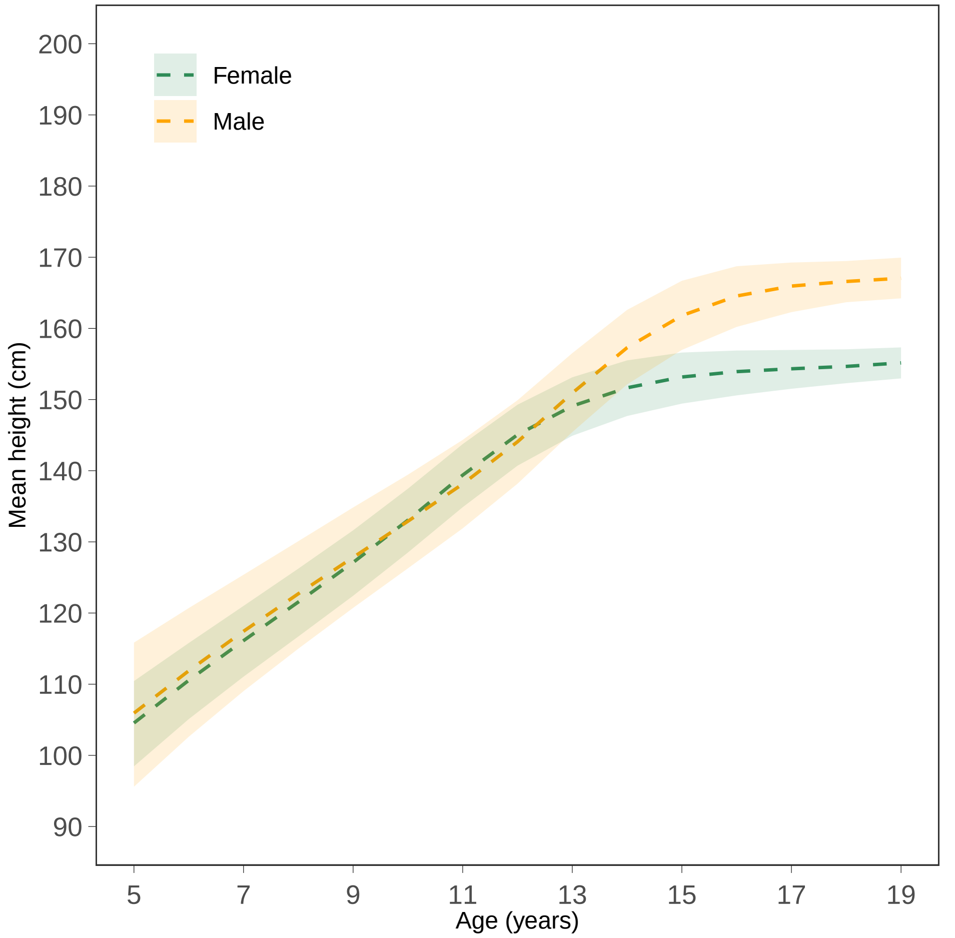

BMI−for−age trajectories (2000 birth cohort)

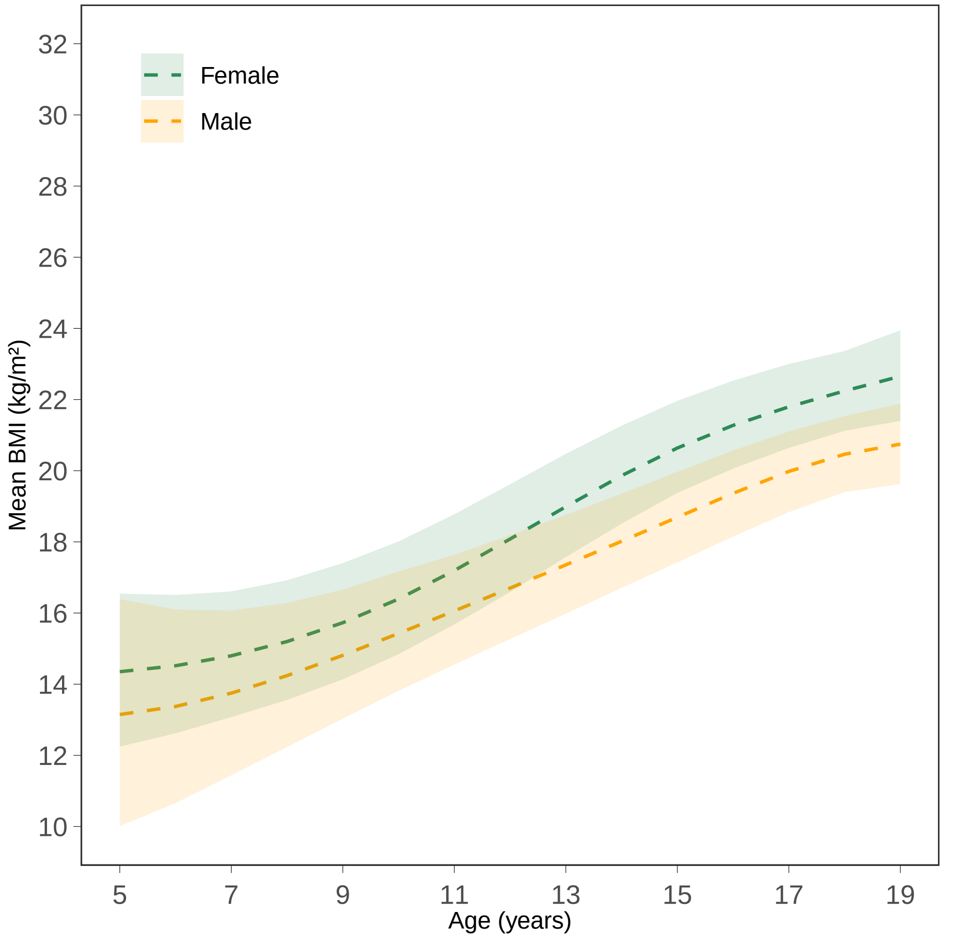

Bolivia

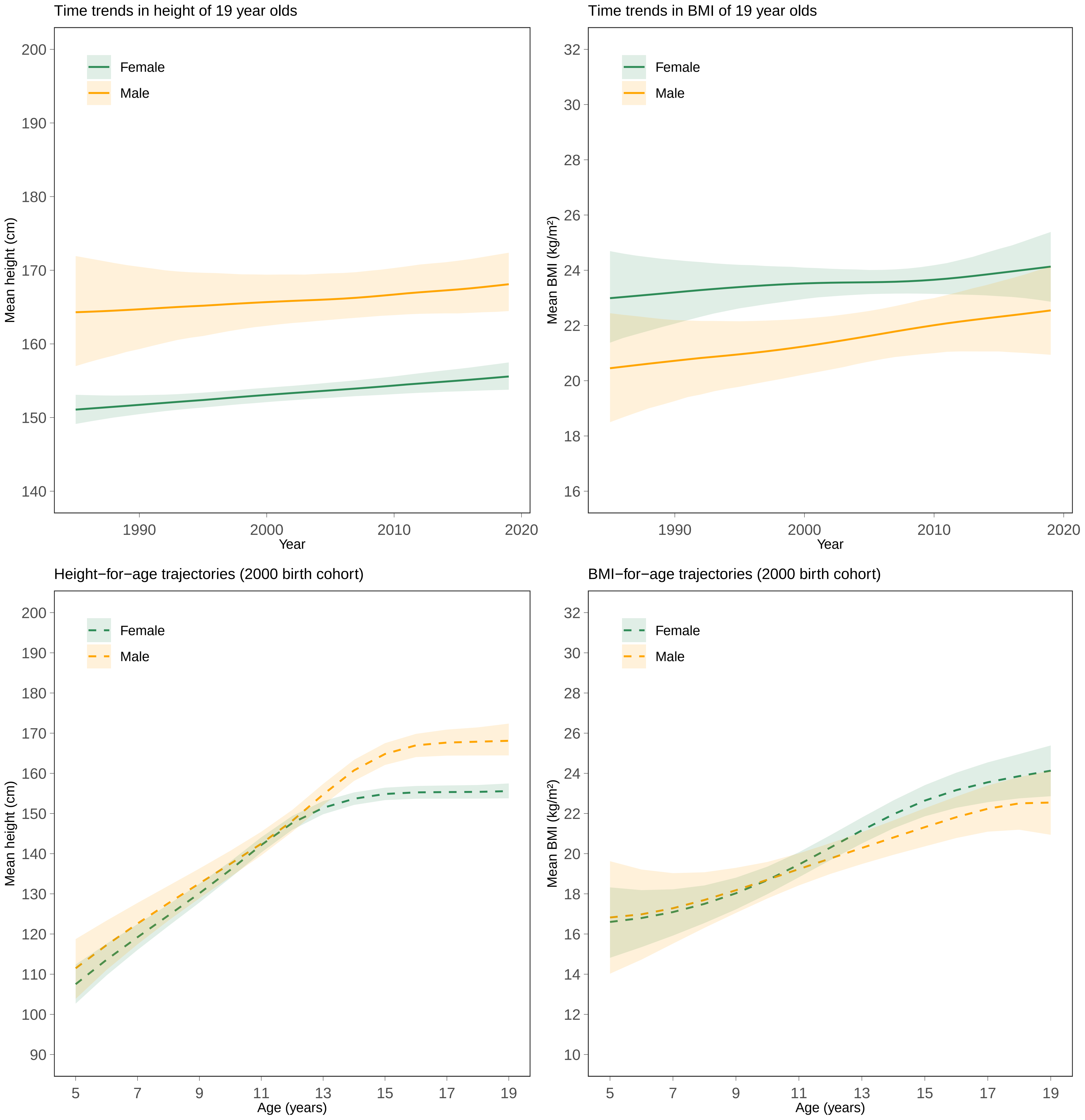
Time trends in height of 19 year olds
Female
Male
Mean height (cm)
Year
Time trends in BMI of 19 year olds
Mean BMI (kg/m²)
Height−for−age trajectories (2000 birth cohort)
Age (years)
BMI−for−age trajectories (2000 birth cohort)

Bosnia and Herzegovina

Time trends in height of 19 year olds

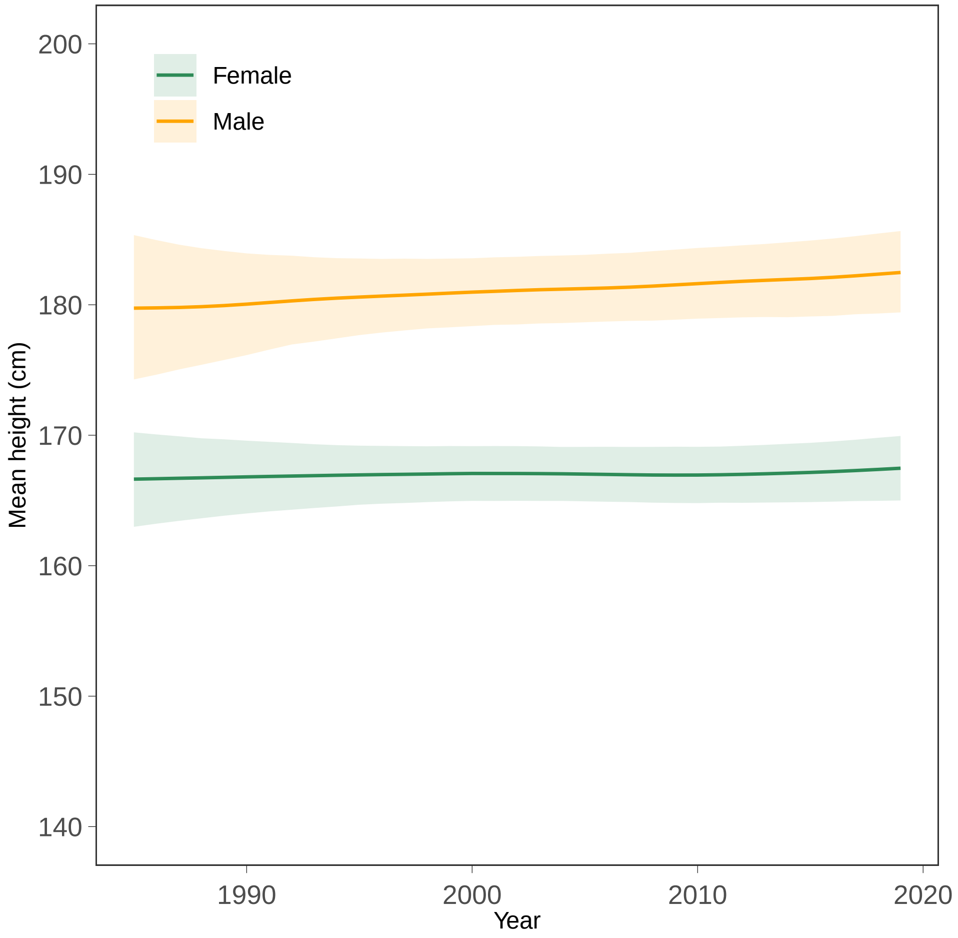

Time trends in BMI of 19 year olds

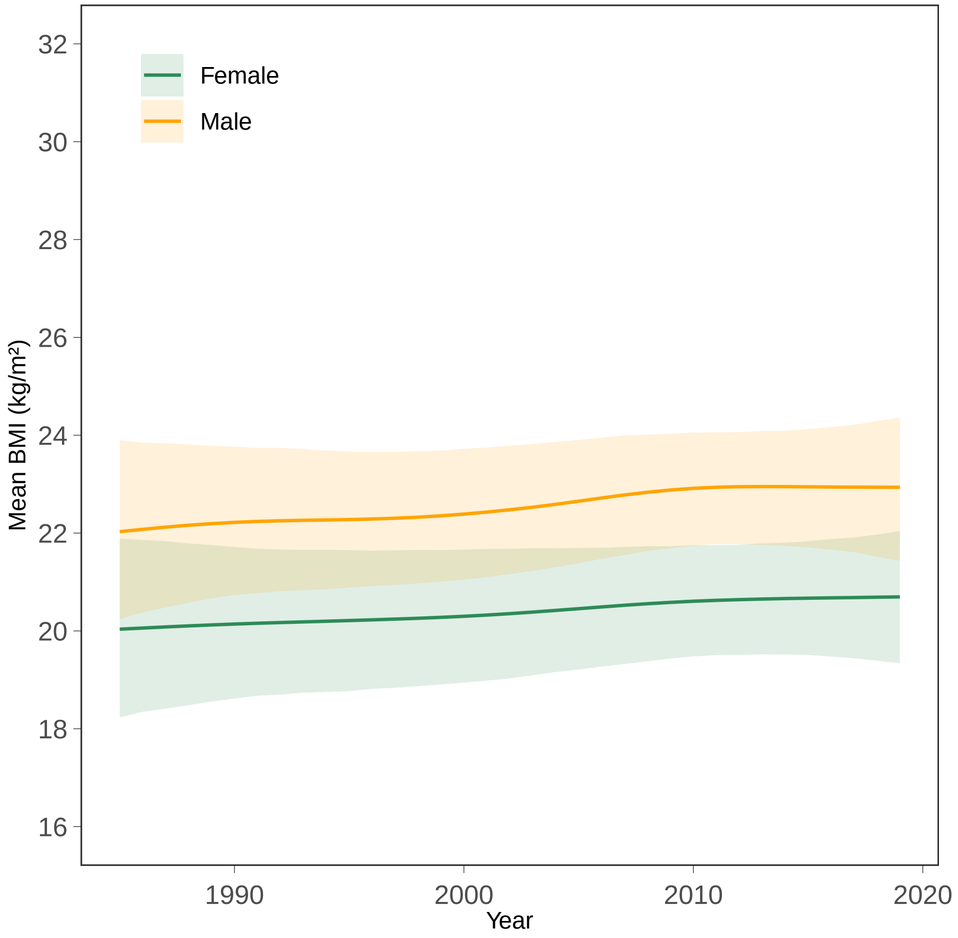

Height−for−age trajectories (2000 birth cohort)

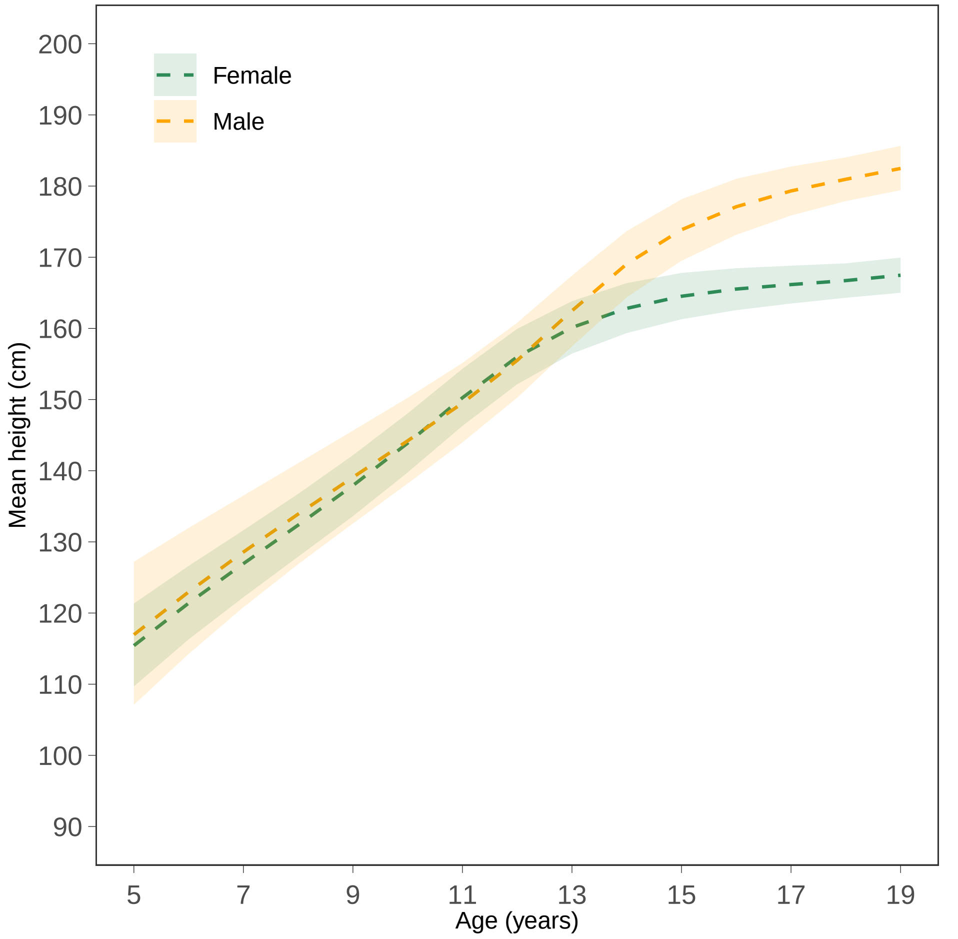

BMI−for−age trajectories (2000 birth cohort)

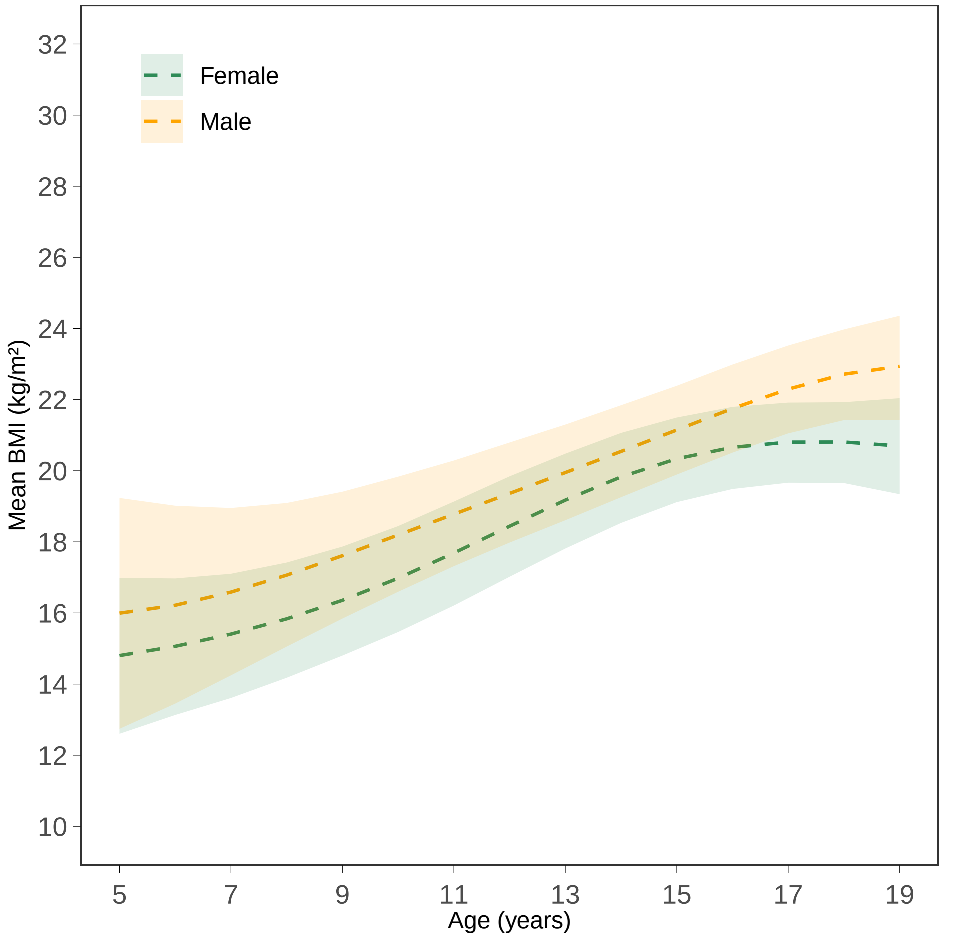

Botswana

Time trends in height of 19 year olds

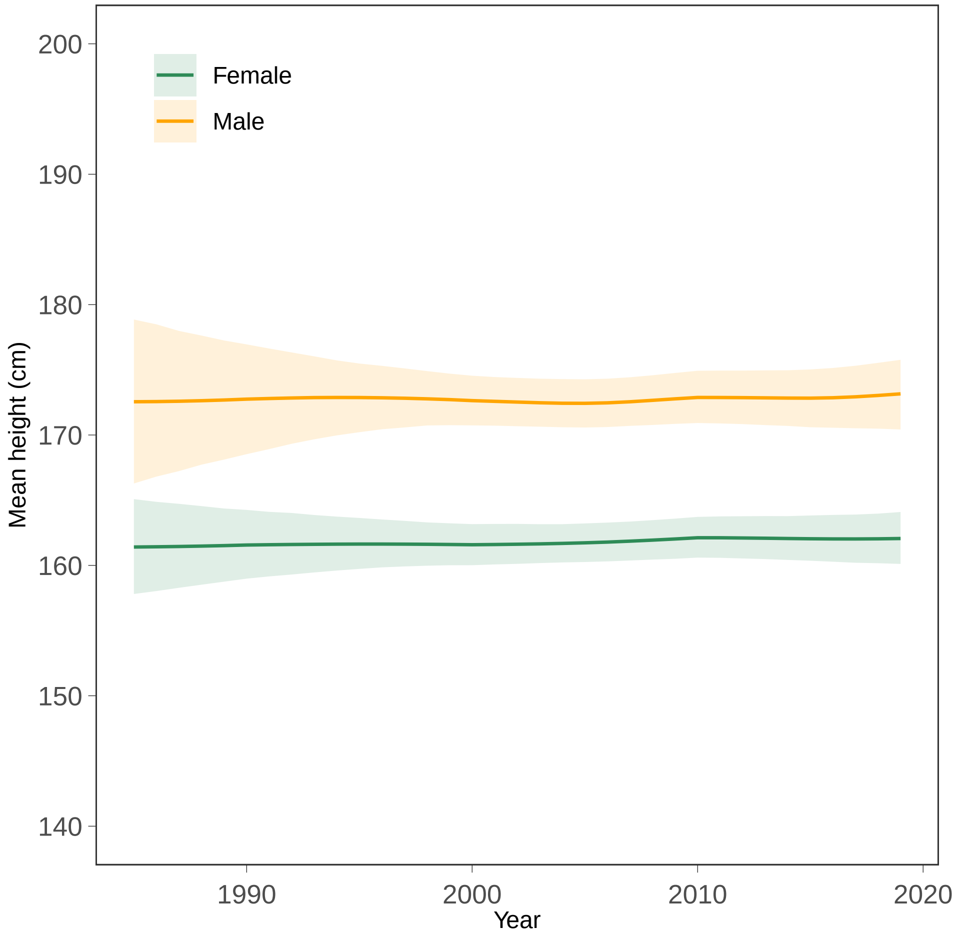

Time trends in BMI of 19 year olds

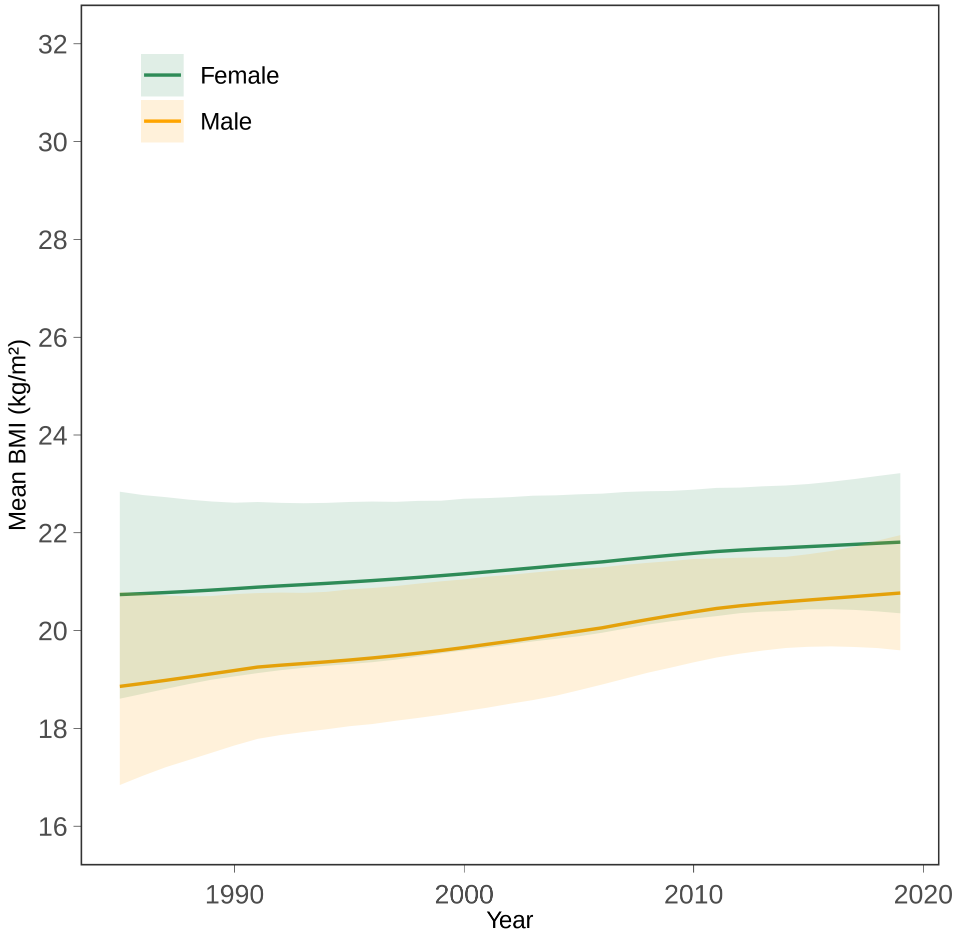

Height−for−age trajectories (2000 birth cohort)

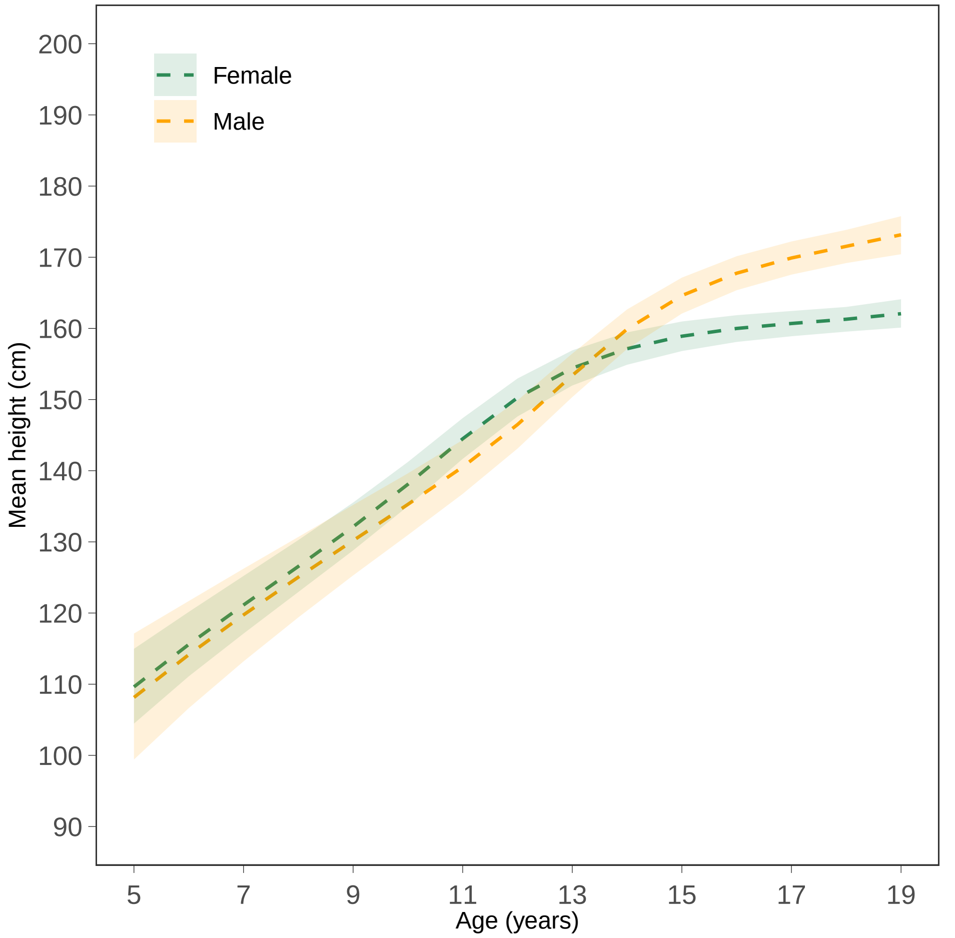

BMI−for−age trajectories (2000 birth cohort)

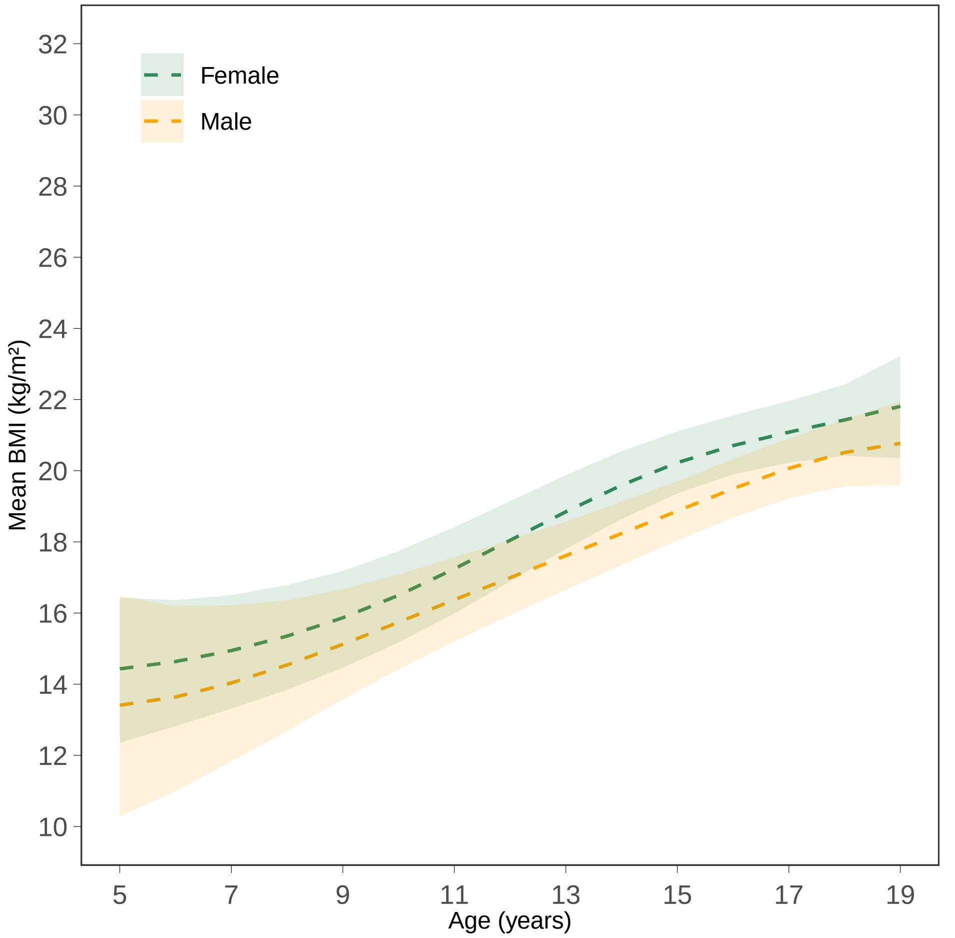

Brazil

Time trends in height of 19 year olds

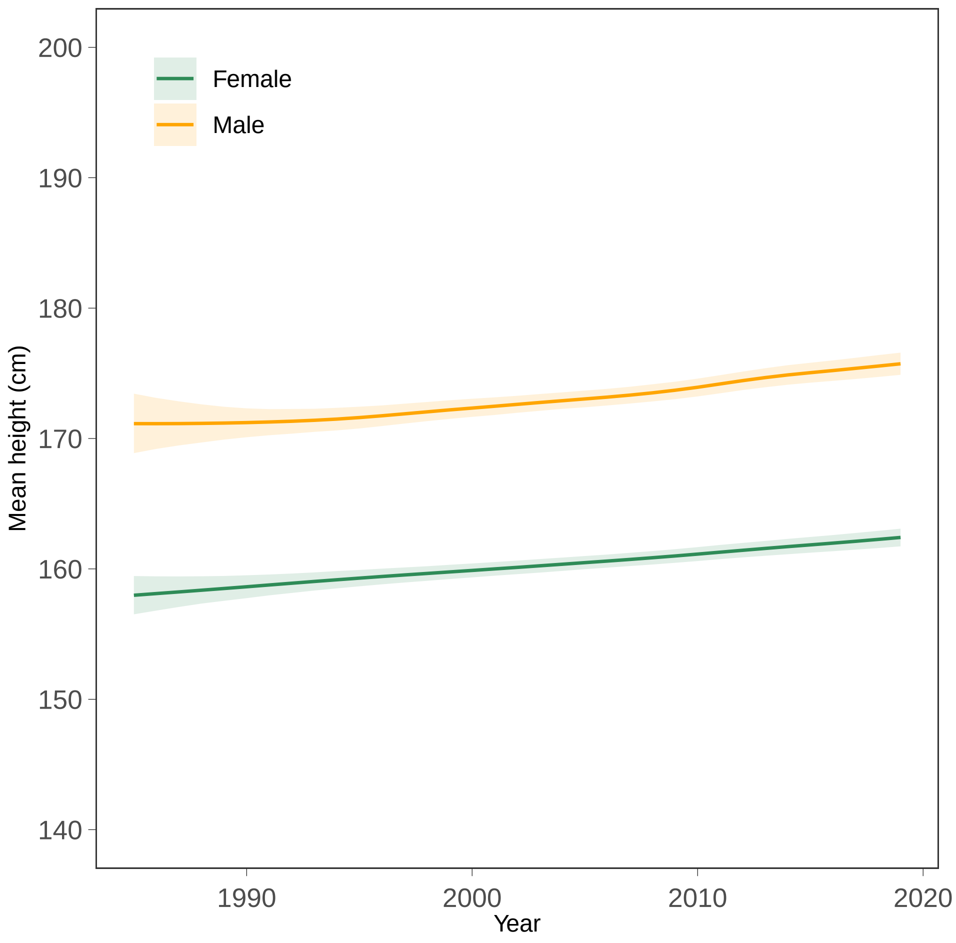

Time trends in BMI of 19 year olds

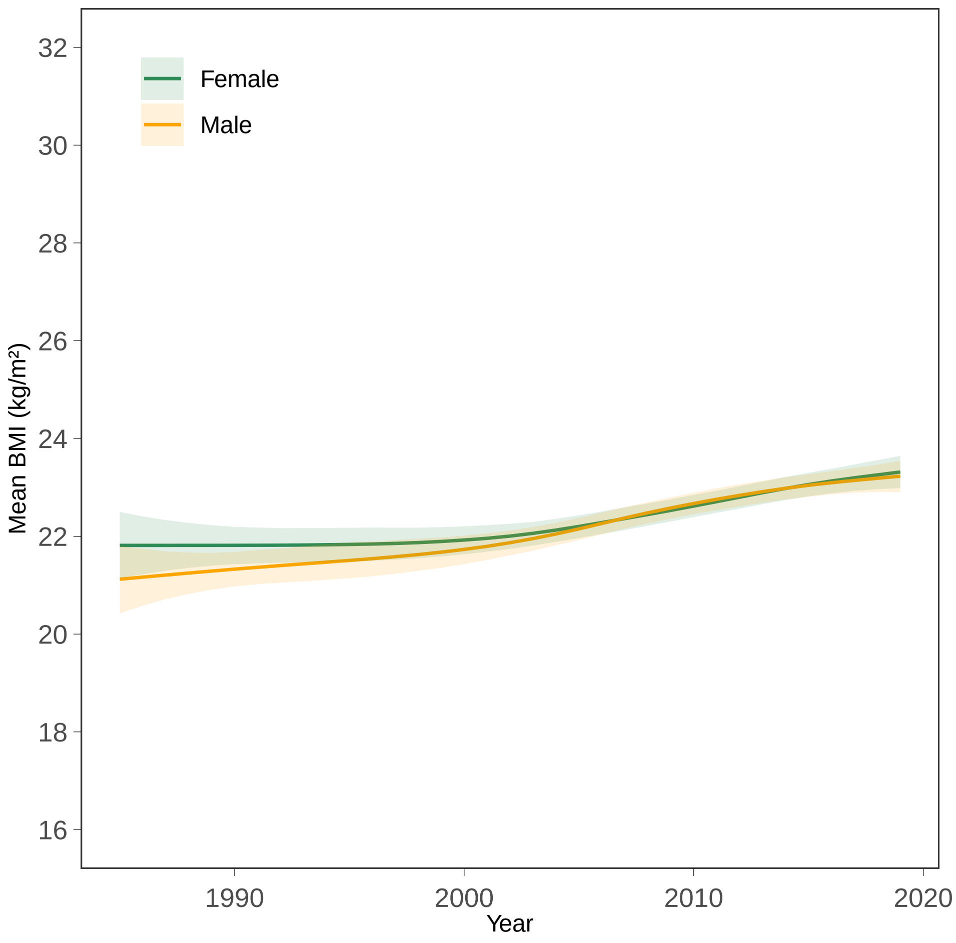

Height−for−age trajectories (2000 birth cohort)

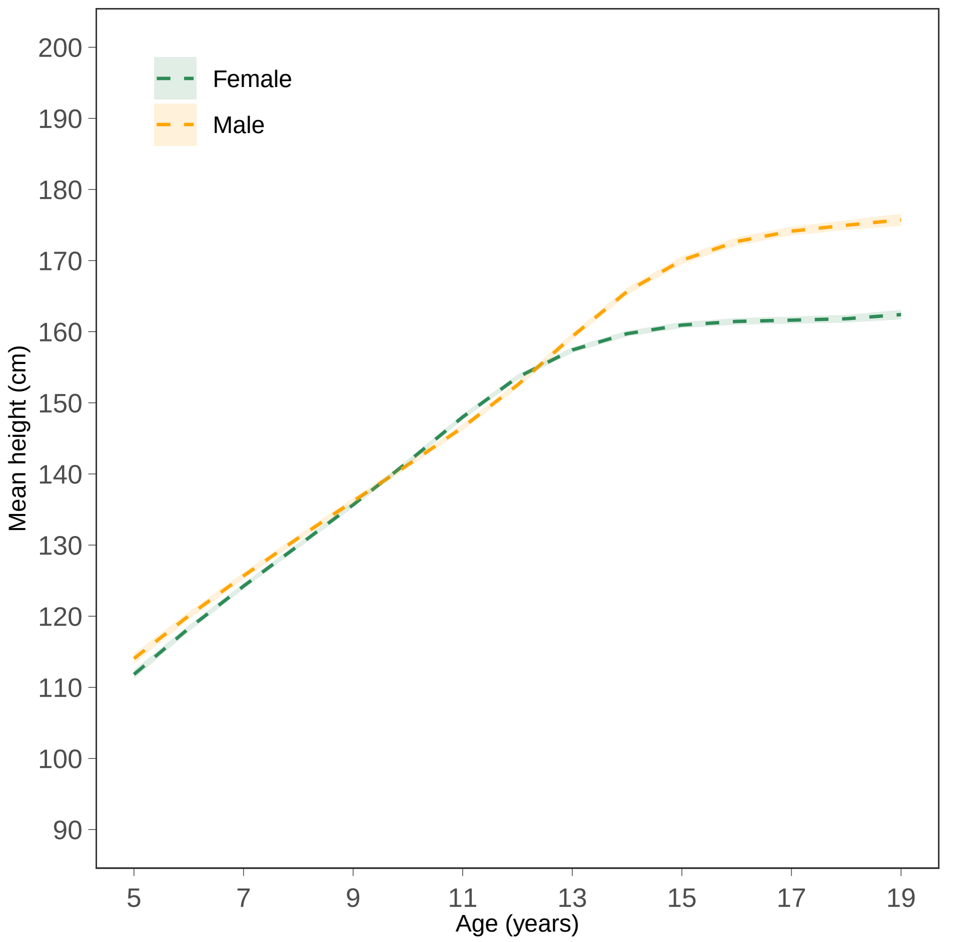

BMI−for−age trajectories (2000 birth cohort)

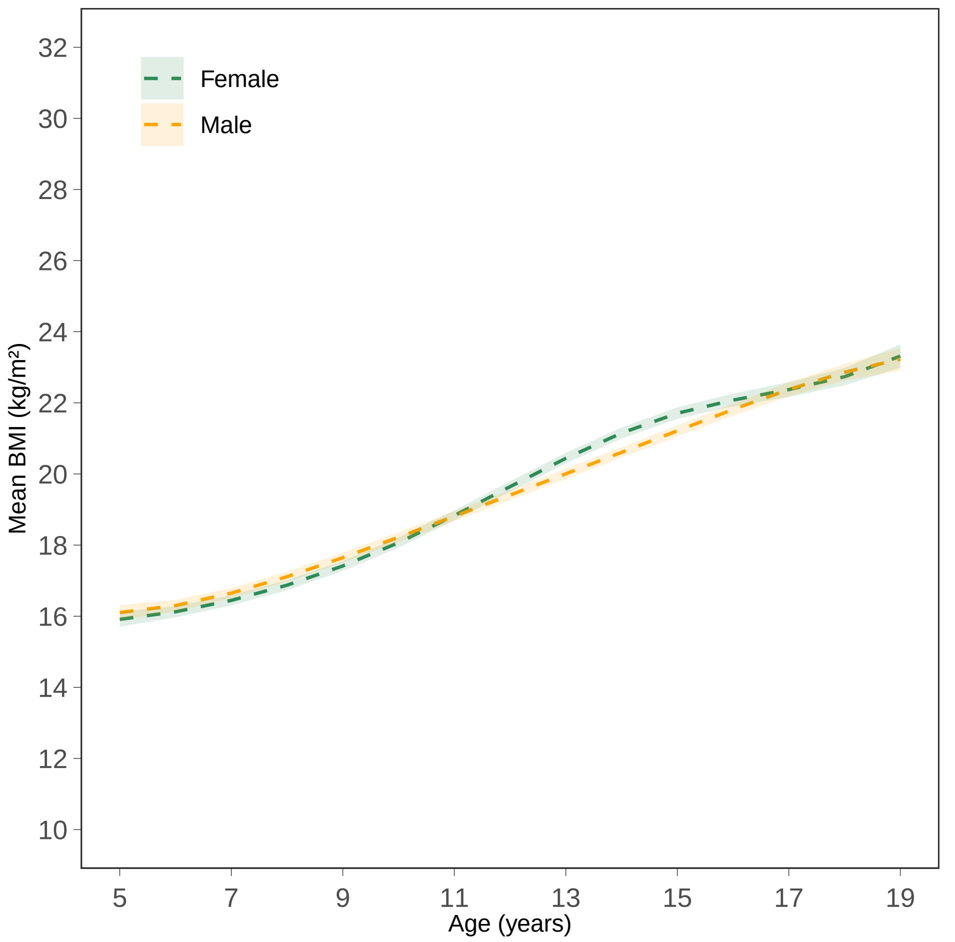

Brunei Darussalam

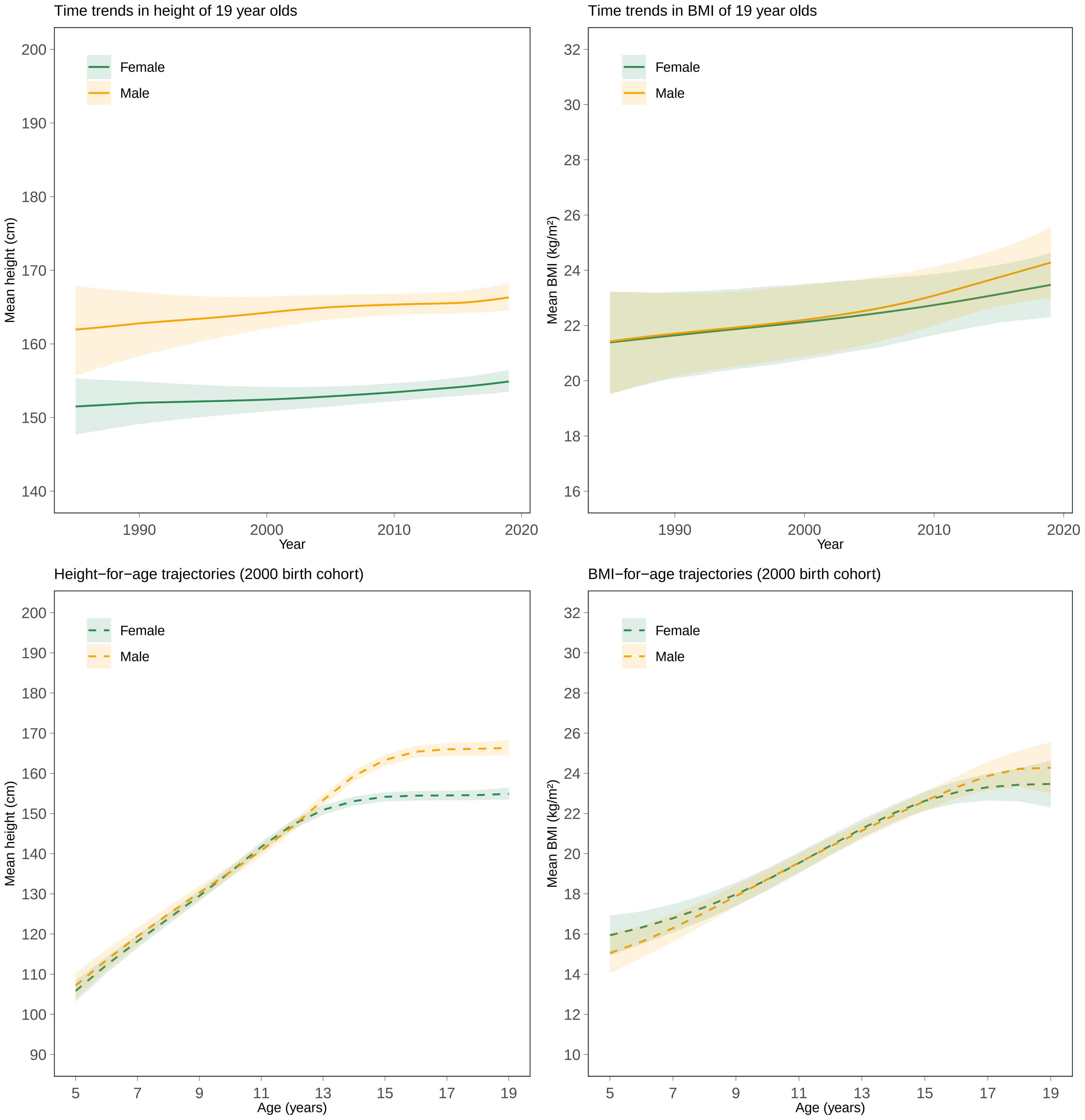

Bulgaria

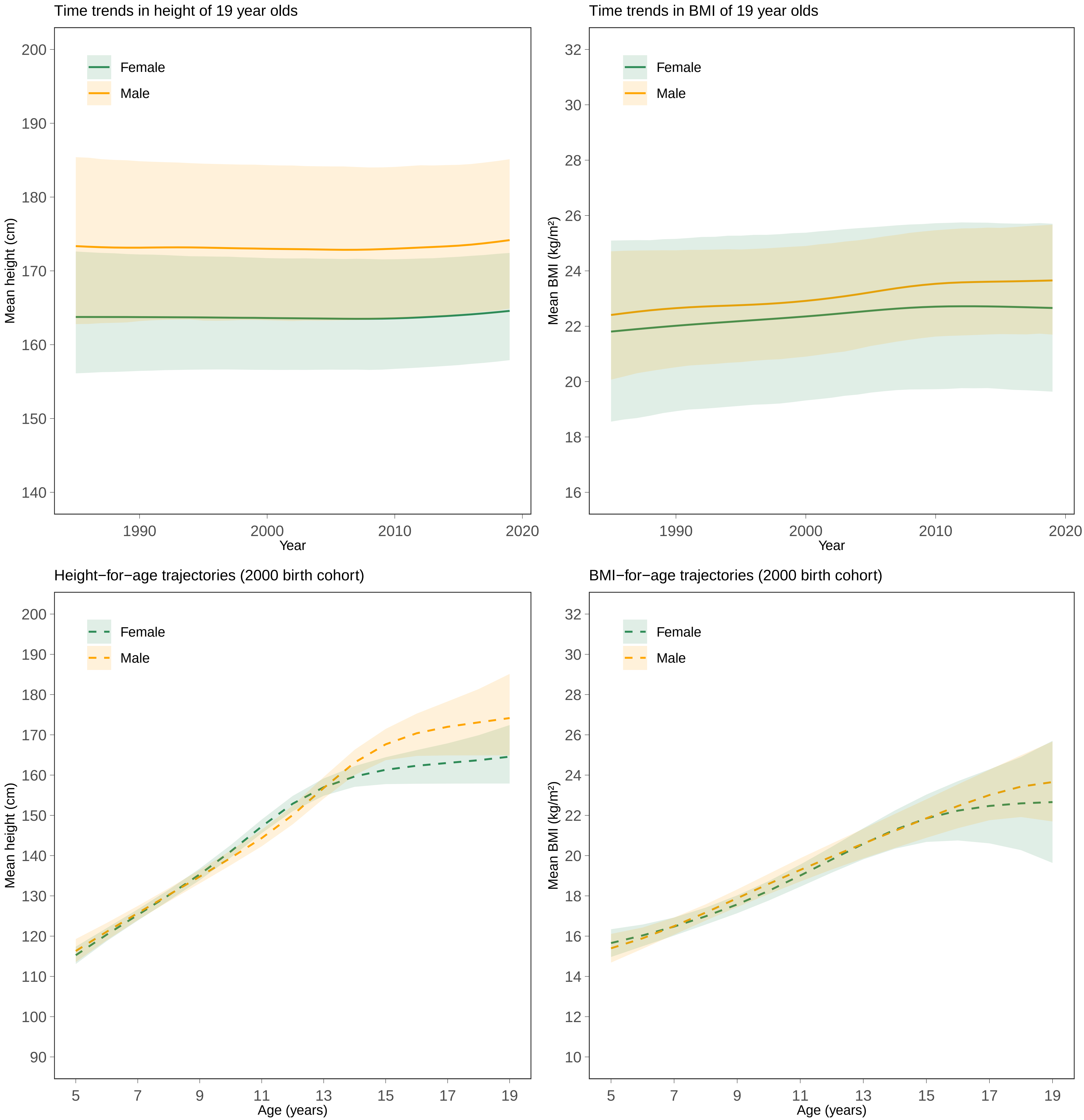
Time trends in height of 19 year olds
Female
Male
Mean height (cm)
Year
Time trends in BMI of 19 year olds
Mean BMI (kg/m²)
Height−for−age trajectories (2000 birth cohort)
Age (years)
BMI−for−age trajectories (2000 birth cohort)

Burkina Faso

Time trends in height of 19 year olds

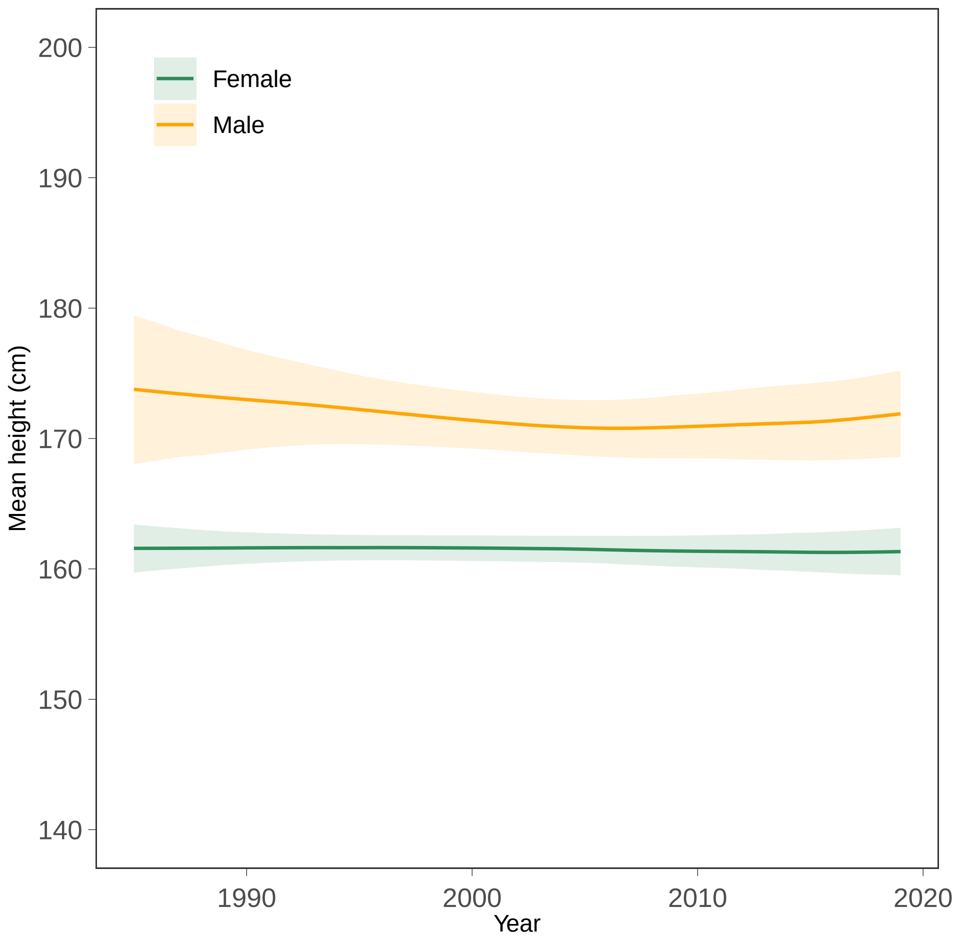

Time trends in BMI of 19 year olds

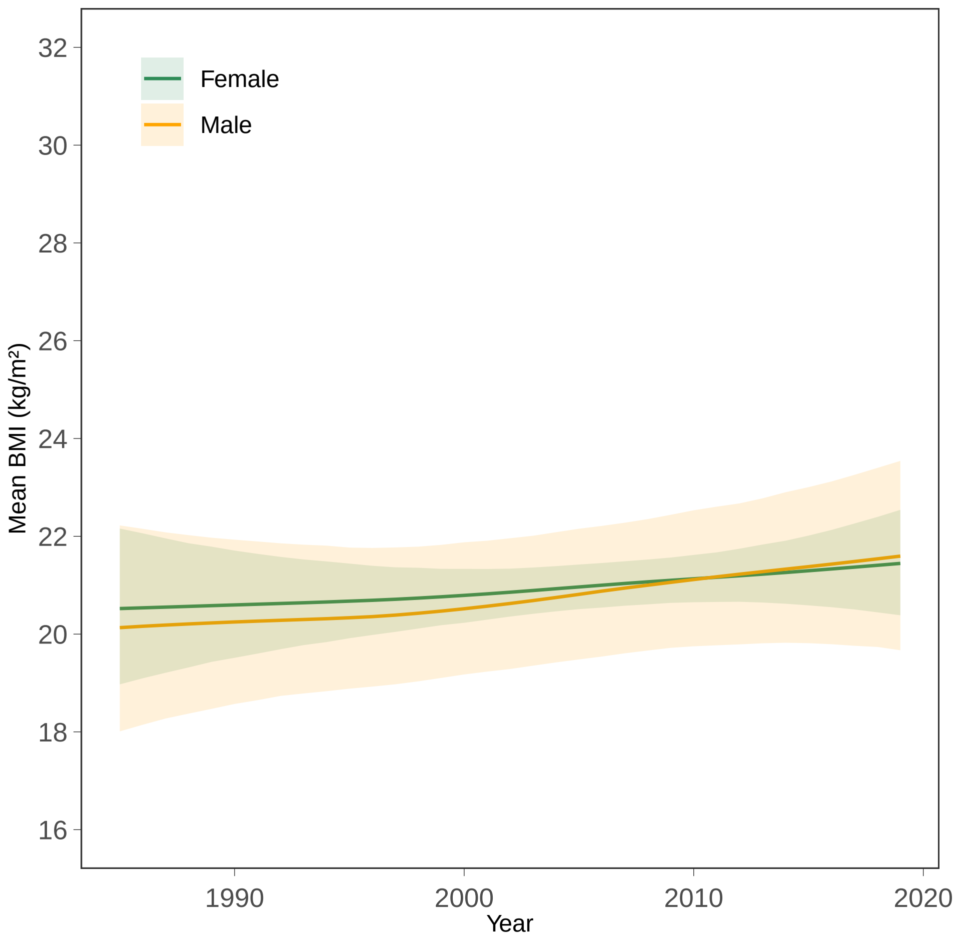

Height−for−age trajectories (2000 birth cohort)

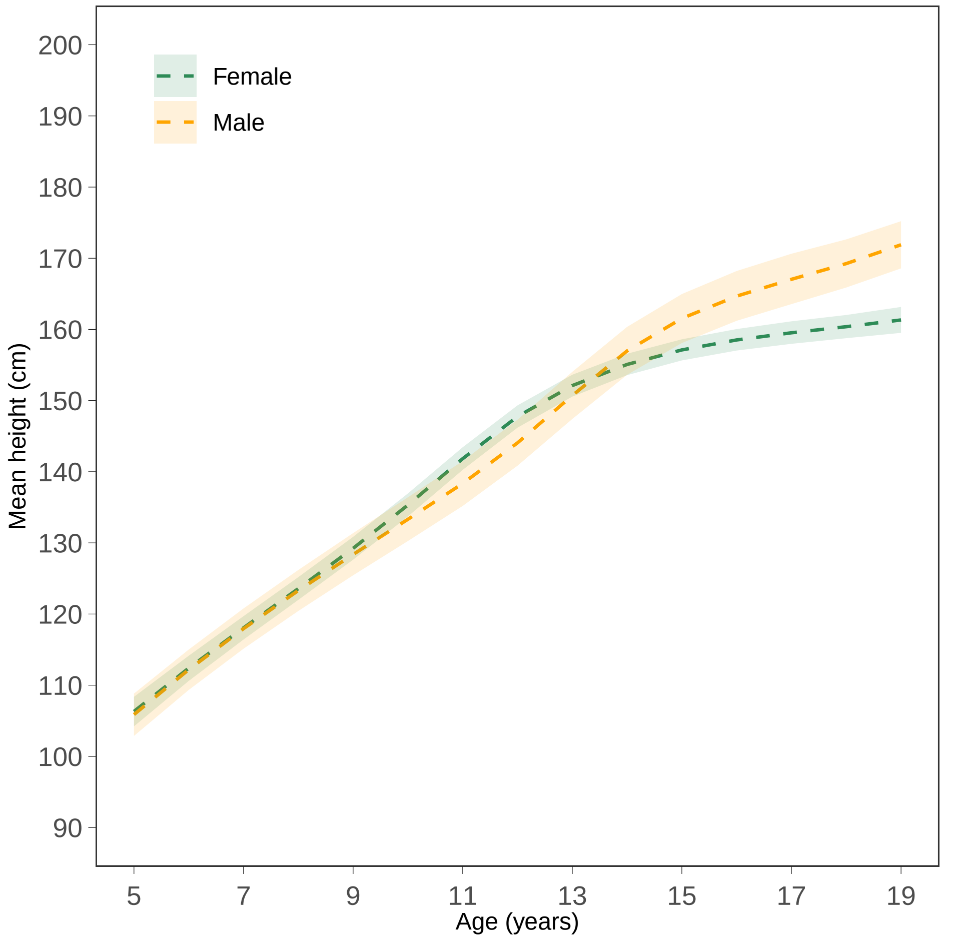

BMI−for−age trajectories (2000 birth cohort)

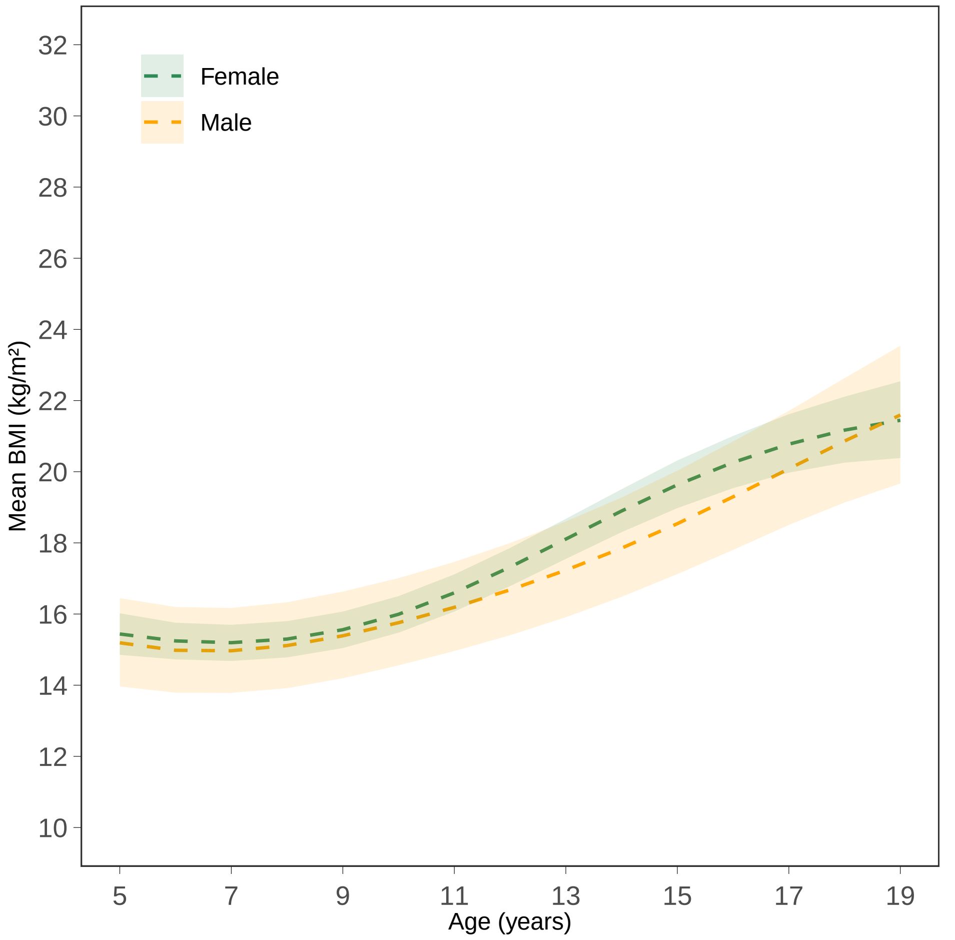

Burundi

Time trends in height of 19 year olds

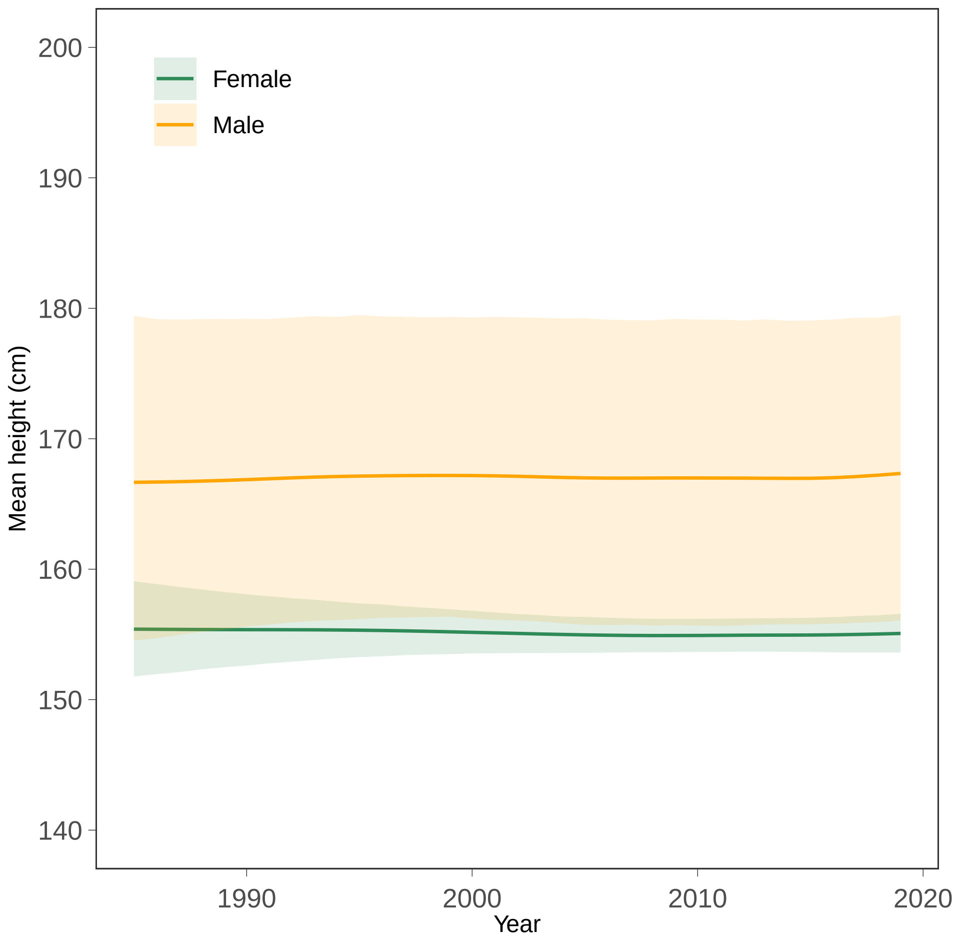

Time trends in BMI of 19 year olds

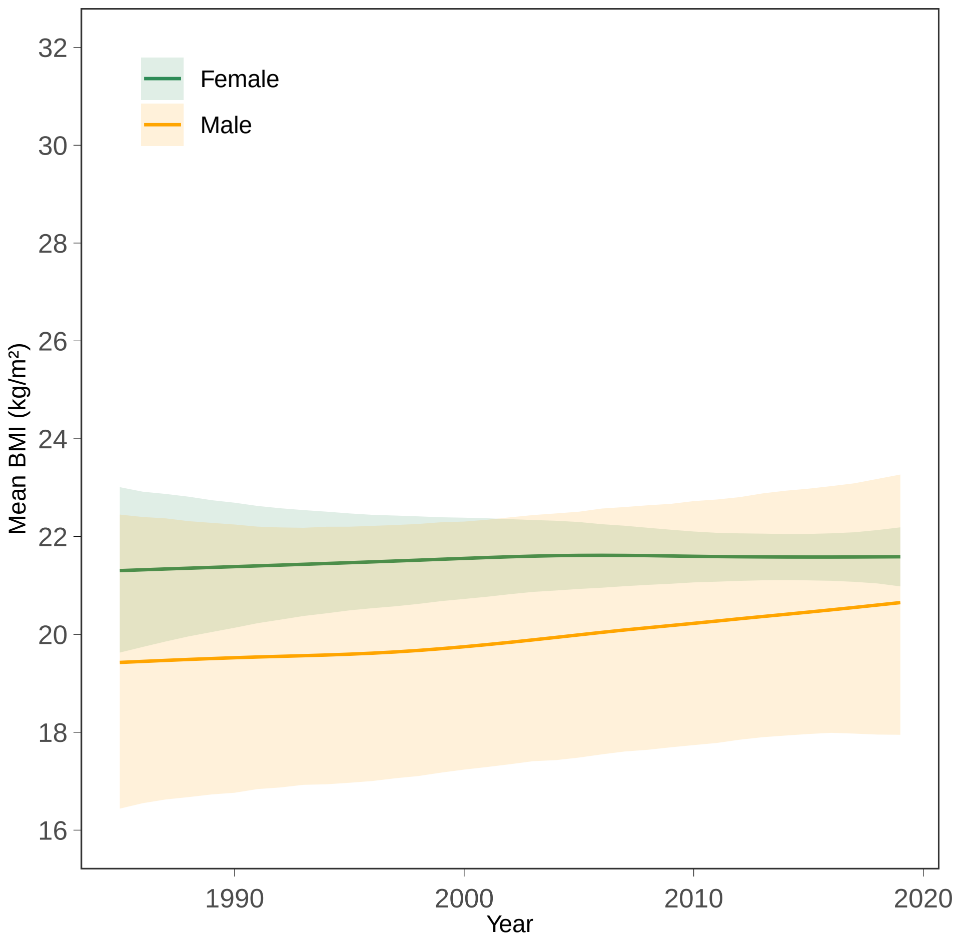

Height−for−age trajectories (2000 birth cohort)

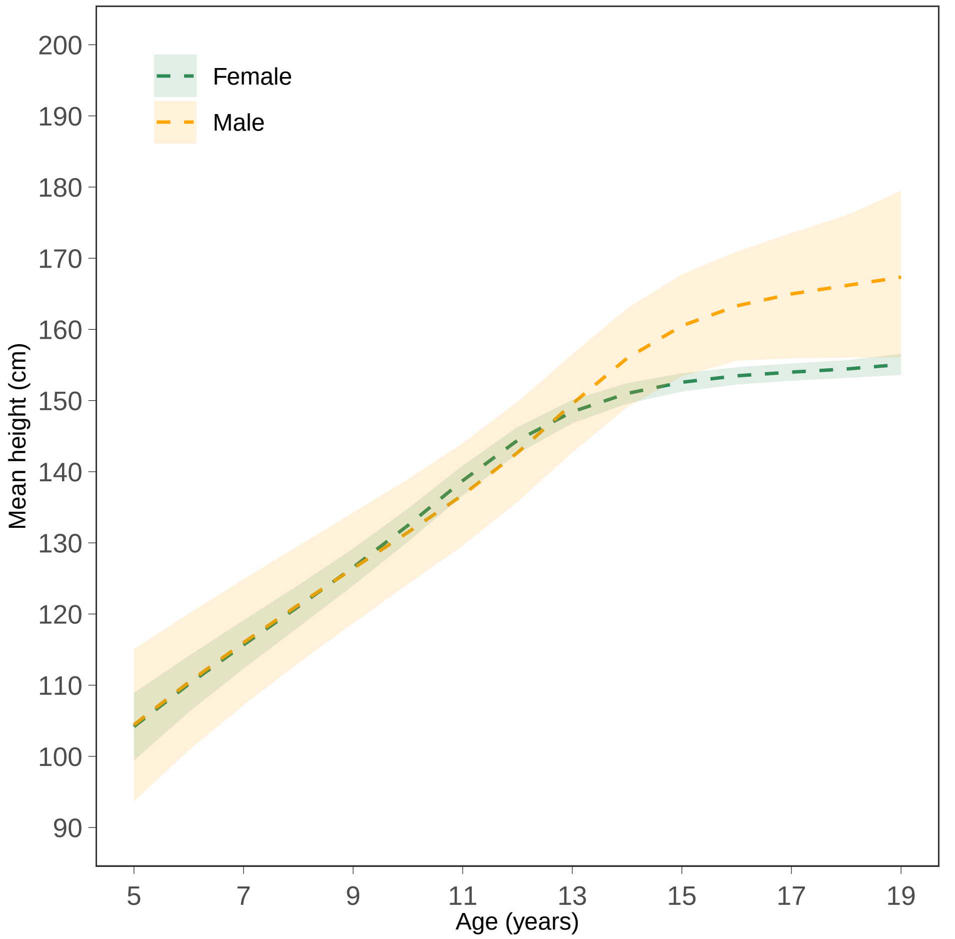

BMI−for−age trajectories (2000 birth cohort)

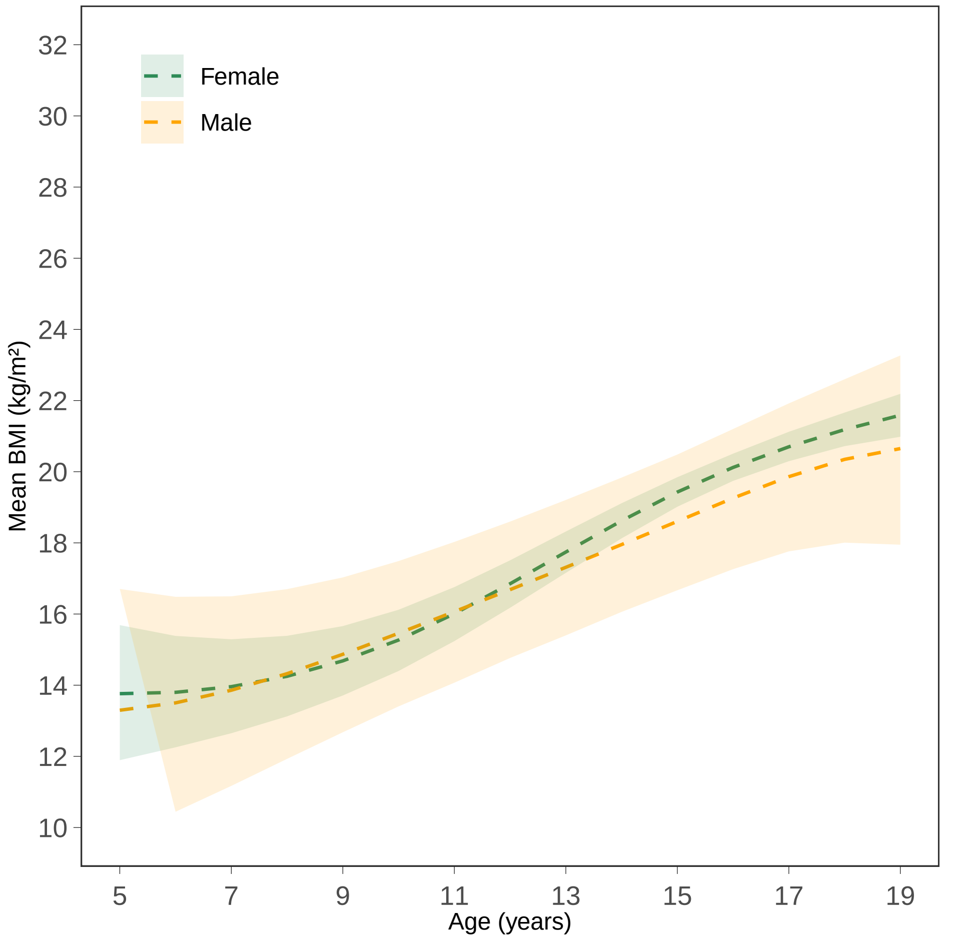

Cabo Verde

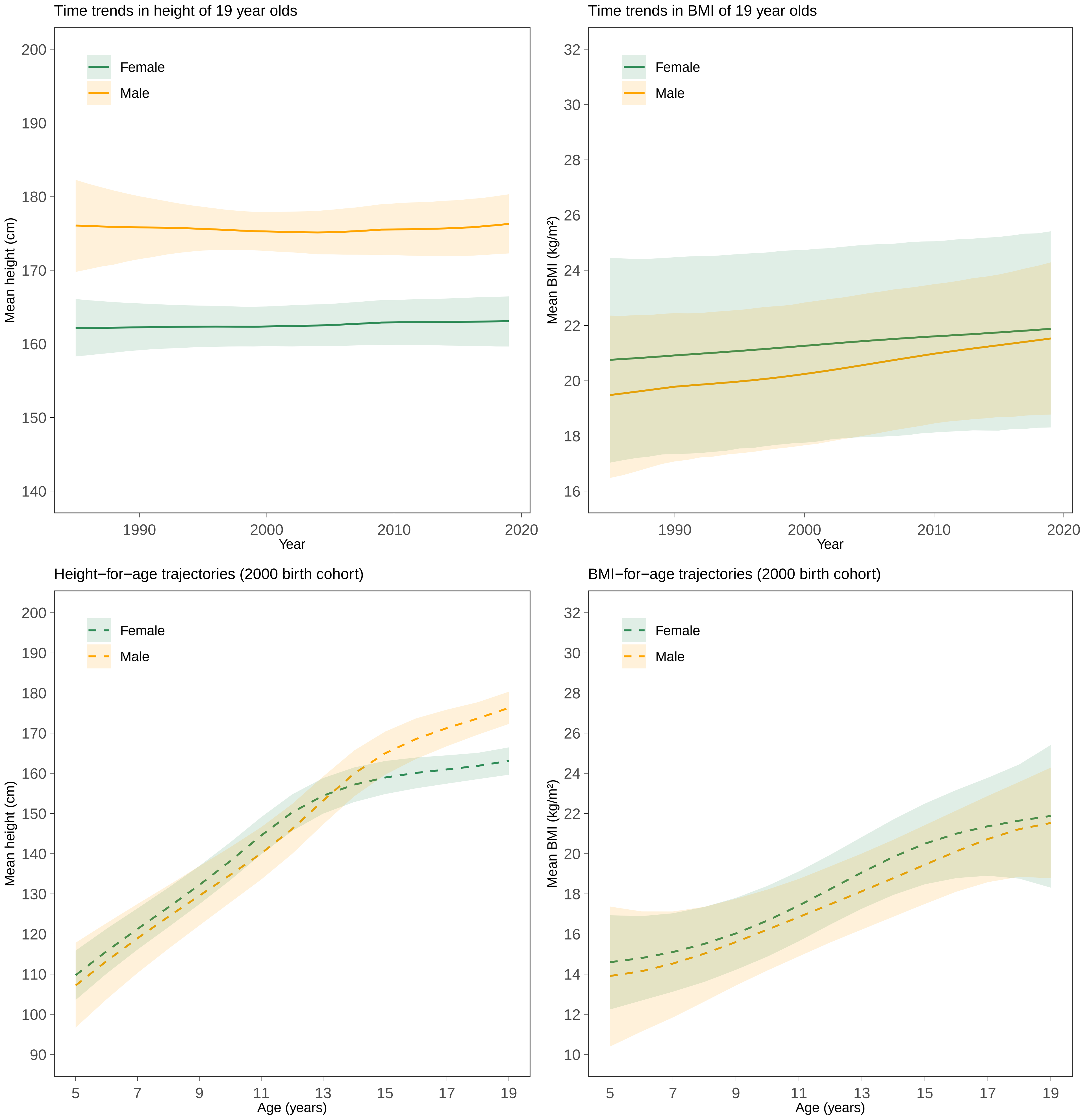
Time trends in height of 19 year olds
Female
Male
Mean height (cm)
Year
Time trends in BMI of 19 year olds
Mean BMI (kg/m²)
Height−for−age trajectories (2000 birth cohort)
Age (years)
BMI−for−age trajectories (2000 birth cohort)

Cambodia

Time trends in height of 19 year olds

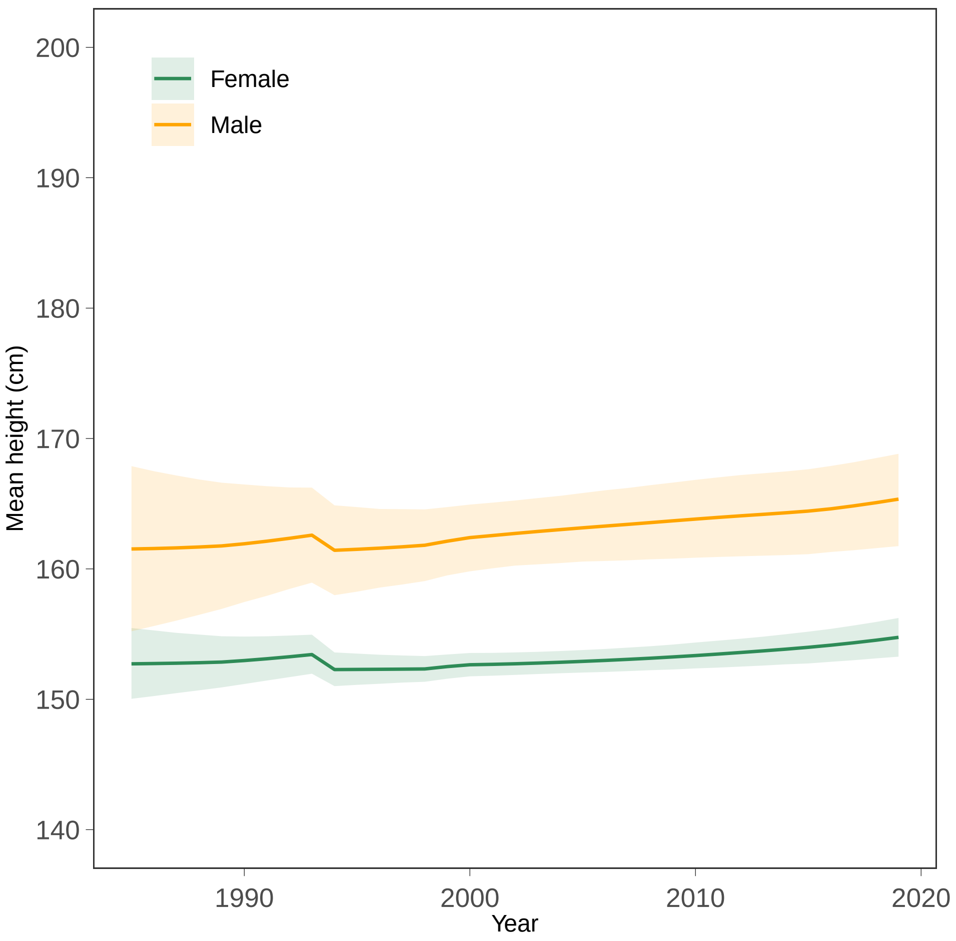

Time trends in BMI of 19 year olds

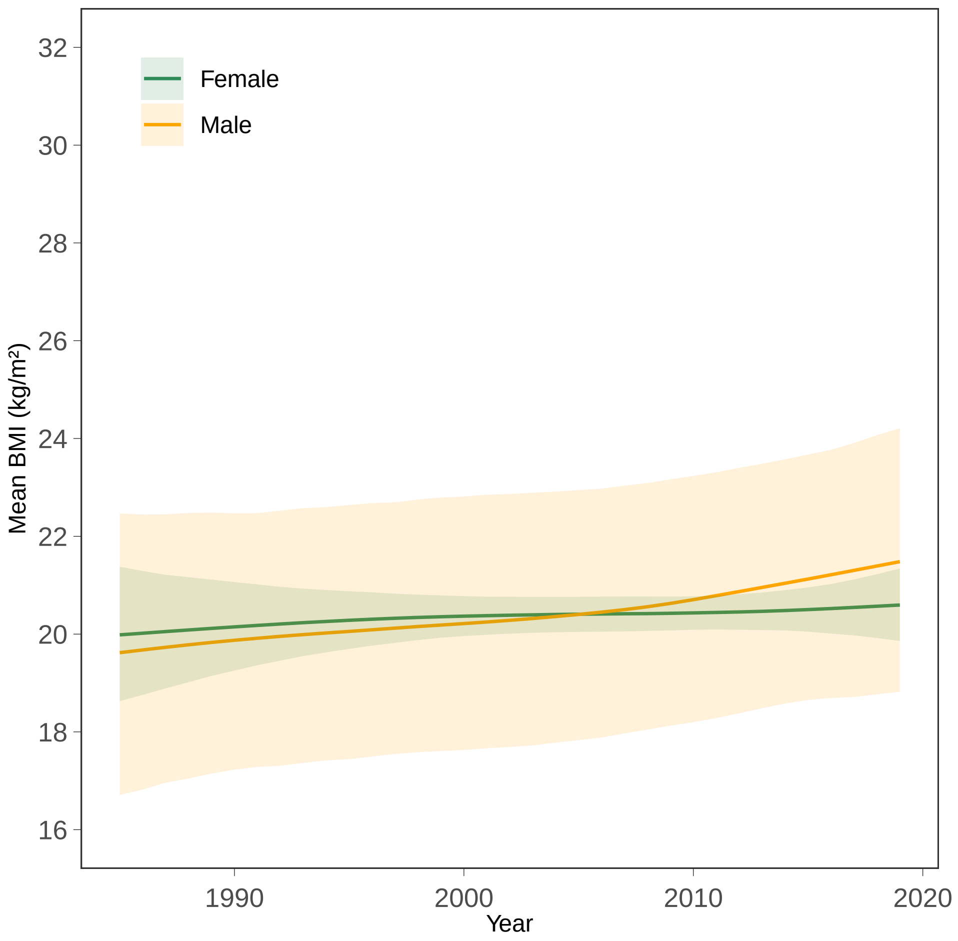

Height−for−age trajectories (2000 birth cohort)

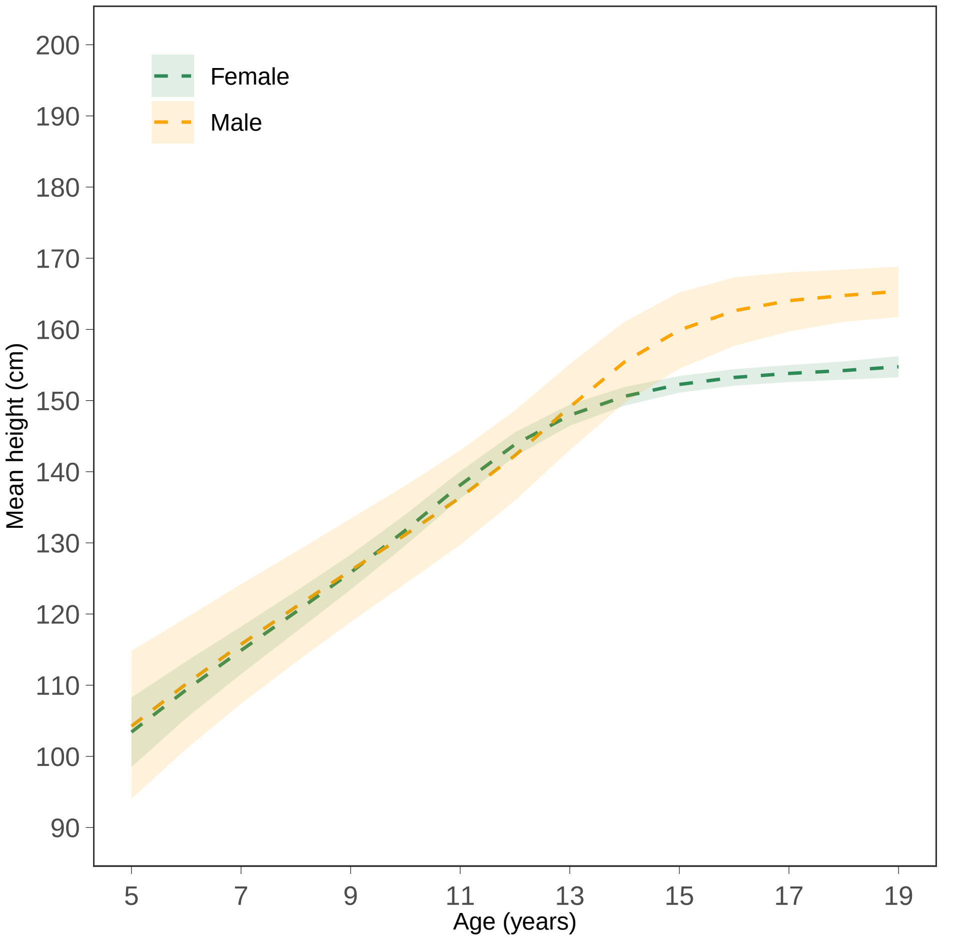

BMI−for−age trajectories (2000 birth cohort)

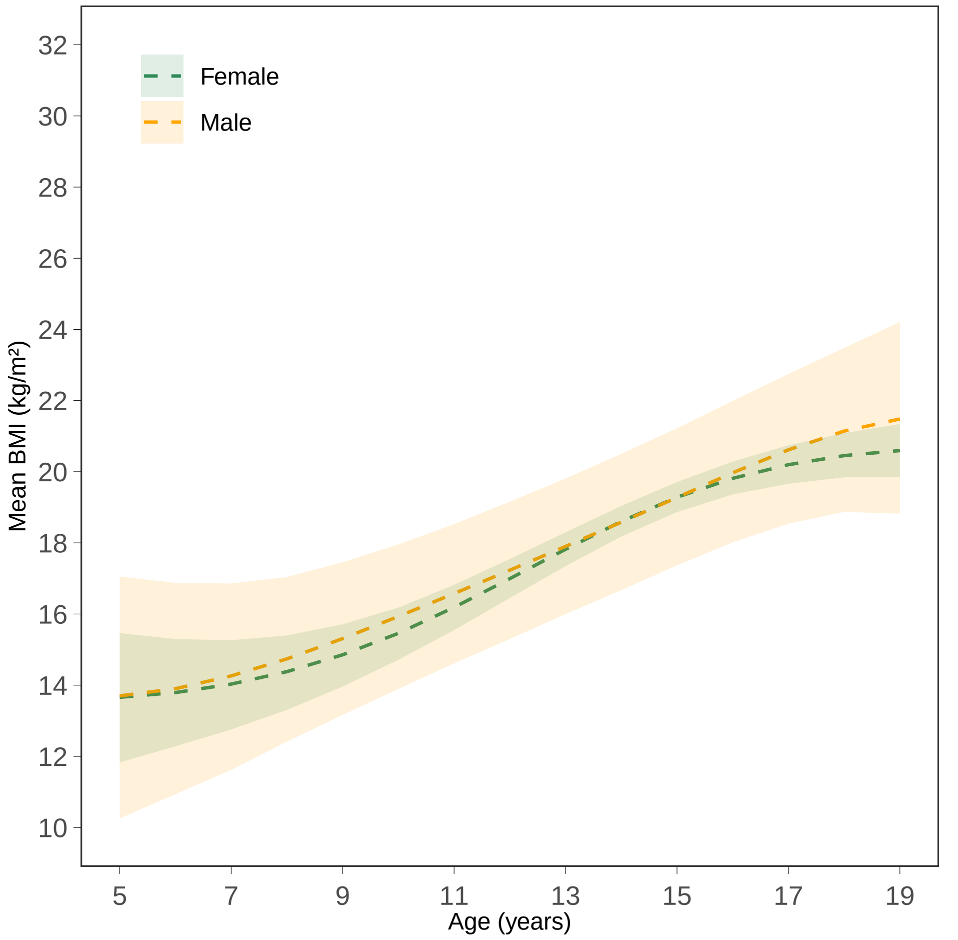

Cameroon

Time trends in height of 19 year olds

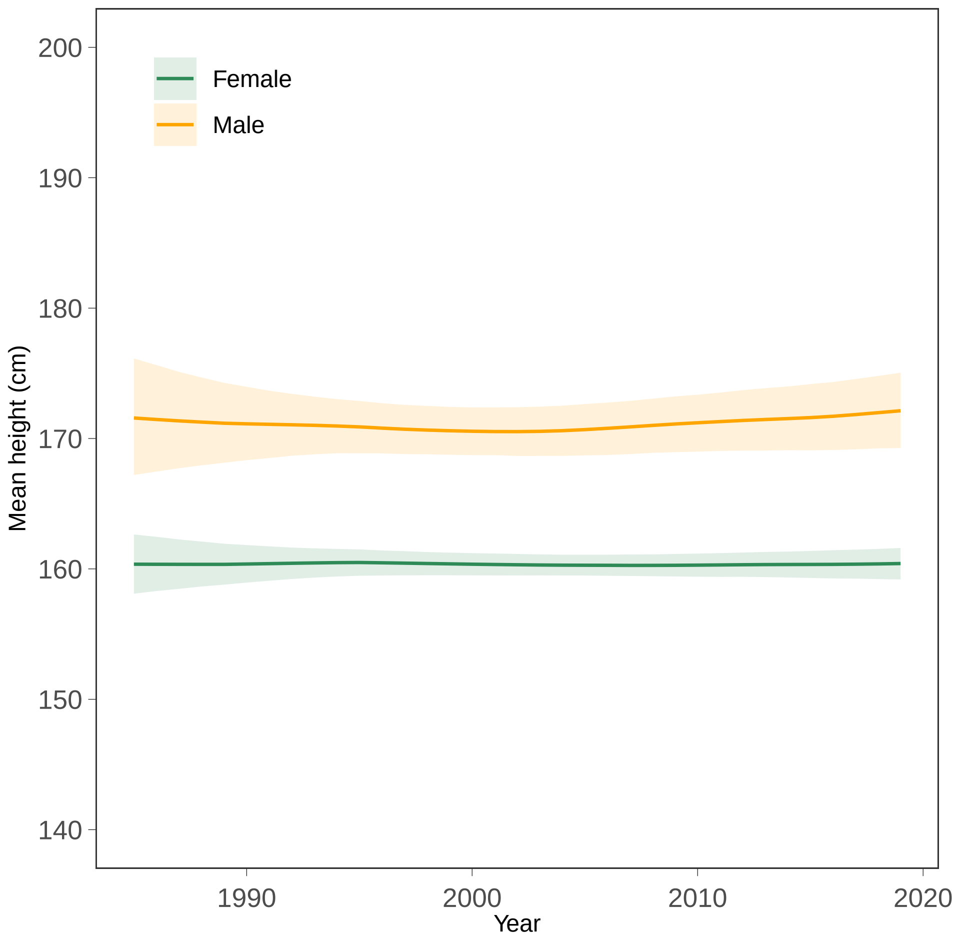

Time trends in BMI of 19 year olds

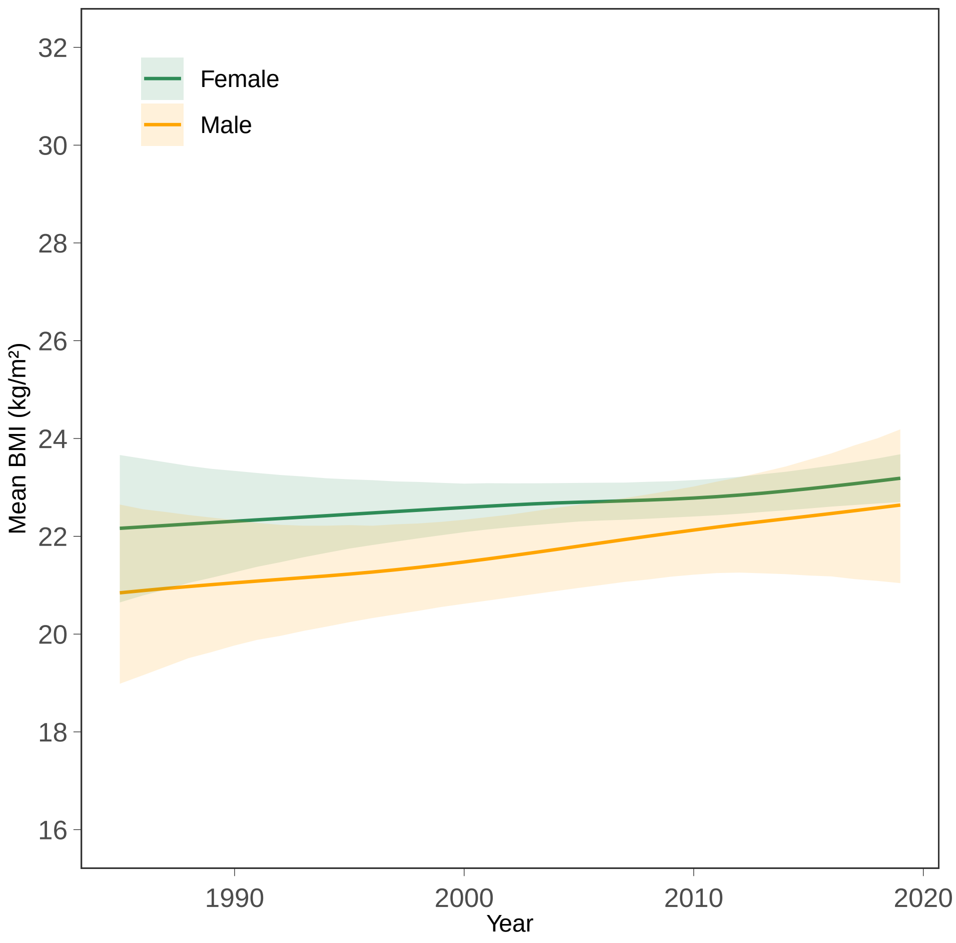

Height−for−age trajectories (2000 birth cohort)

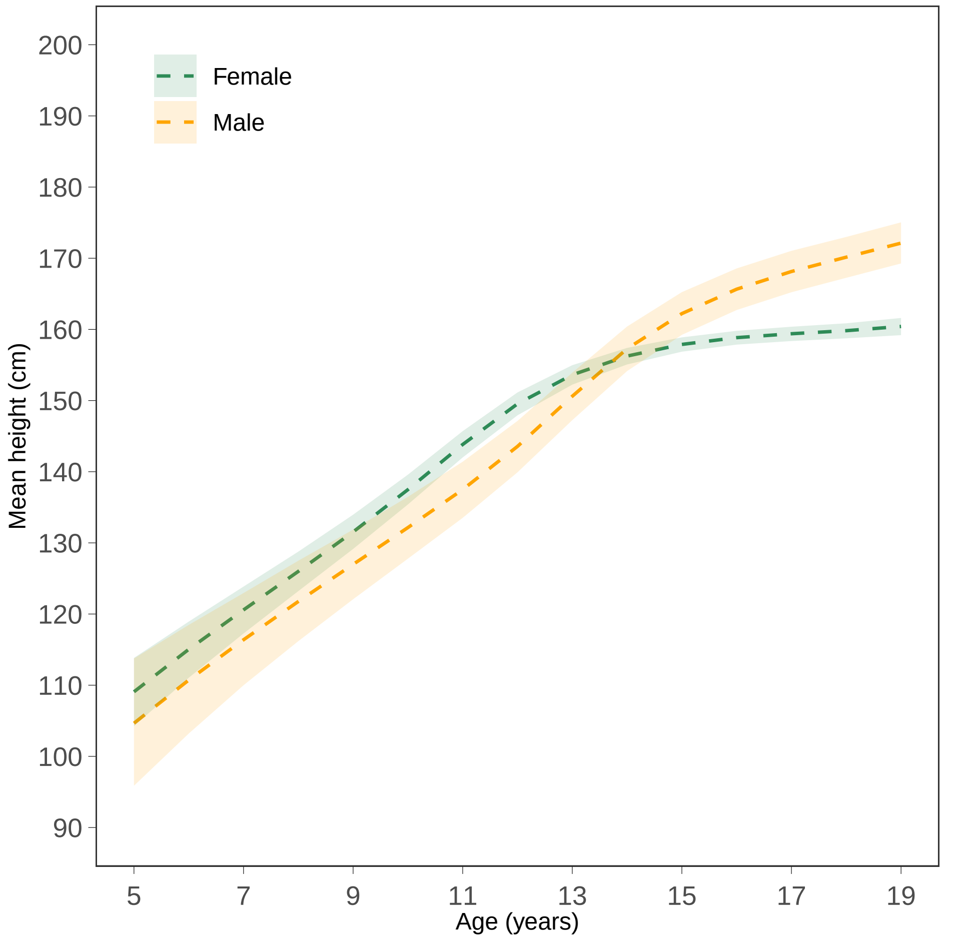

BMI−for−age trajectories (2000 birth cohort)

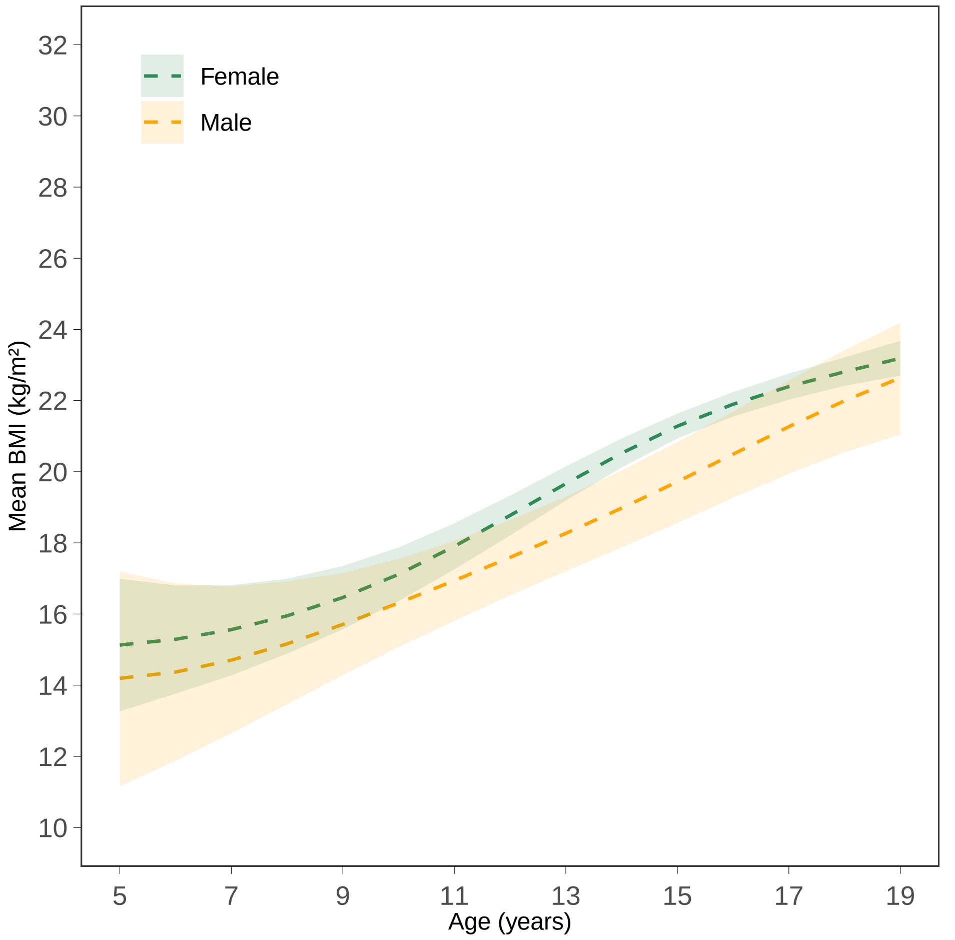

Canada

Time trends in height of 19 year olds

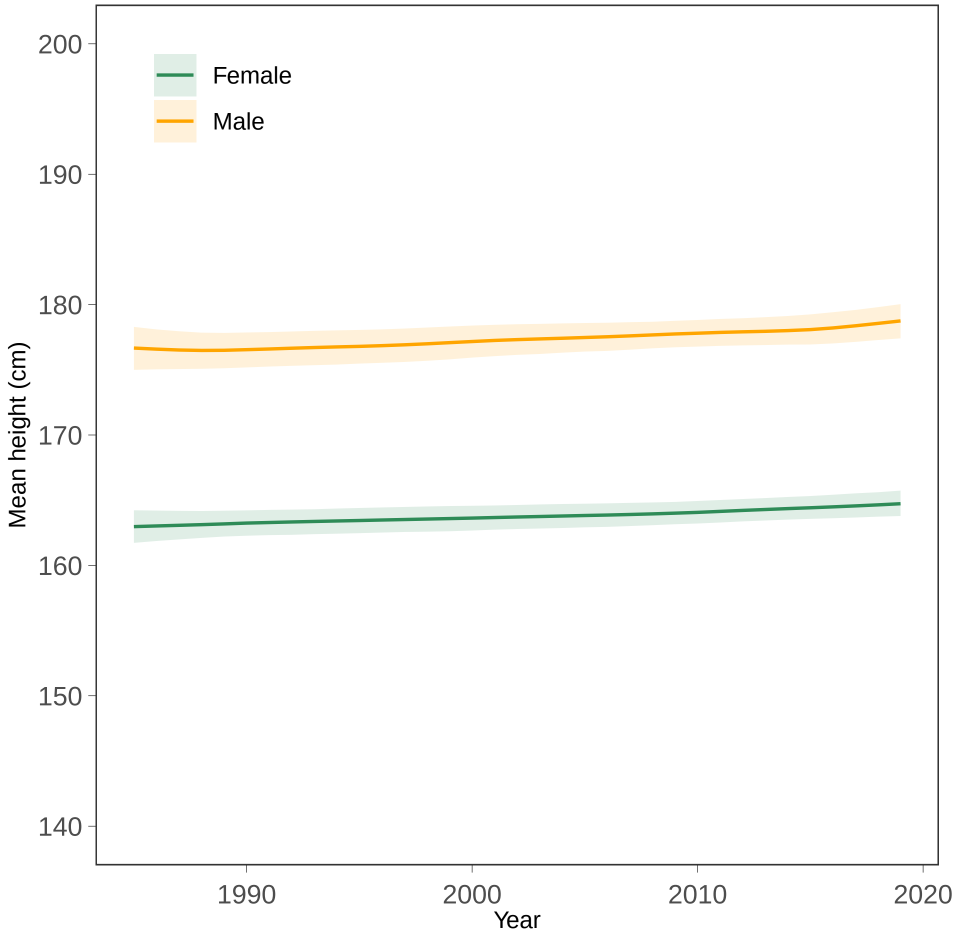

Time trends in BMI of 19 year olds

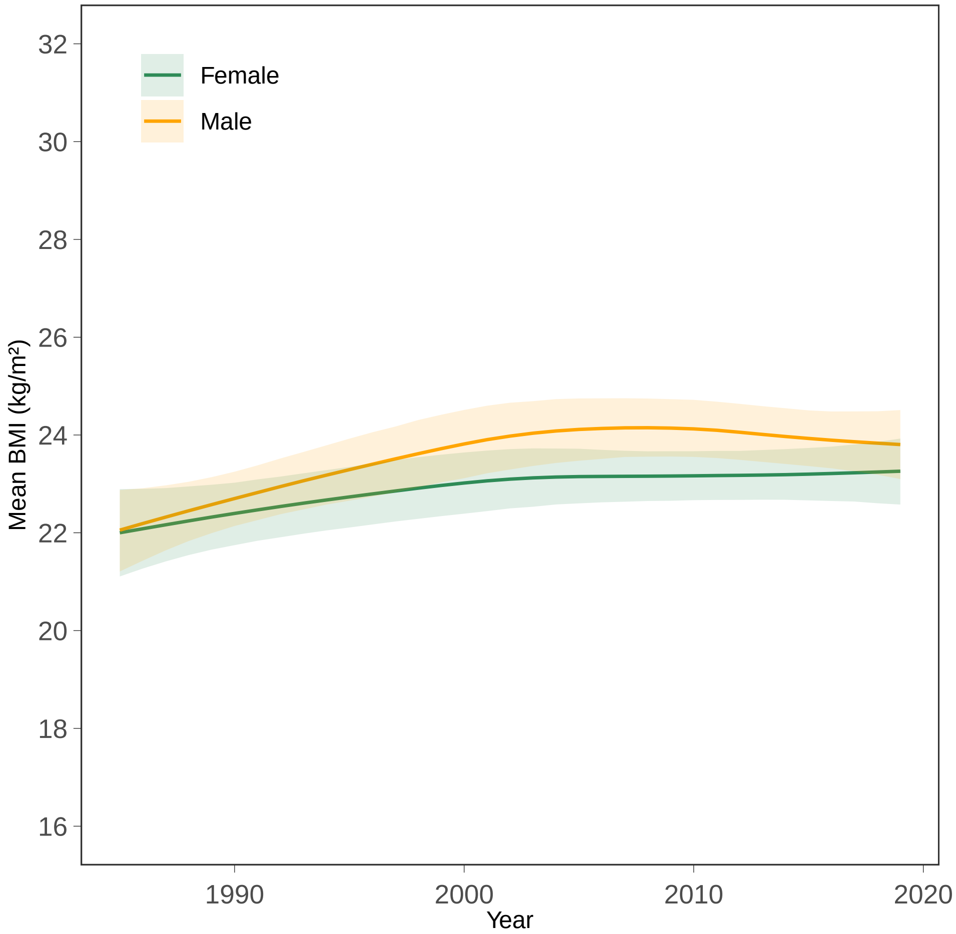

Height−for−age trajectories (2000 birth cohort)

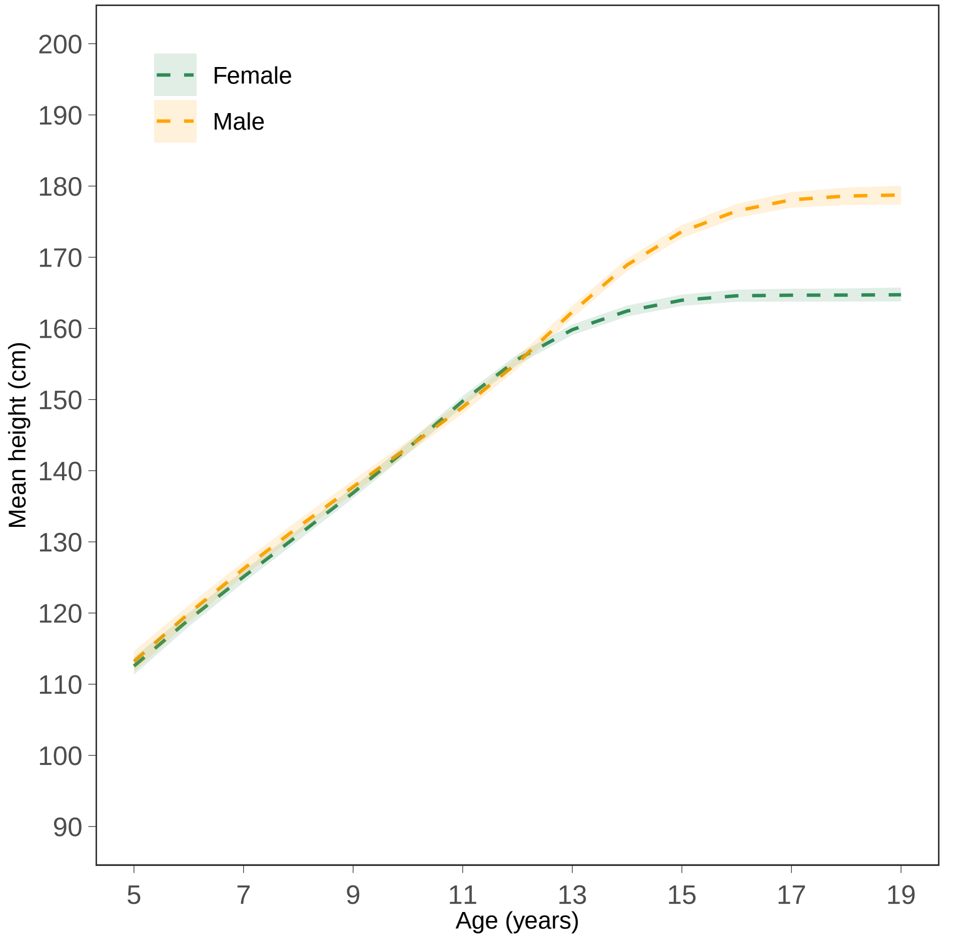

BMI−for−age trajectories (2000 birth cohort)

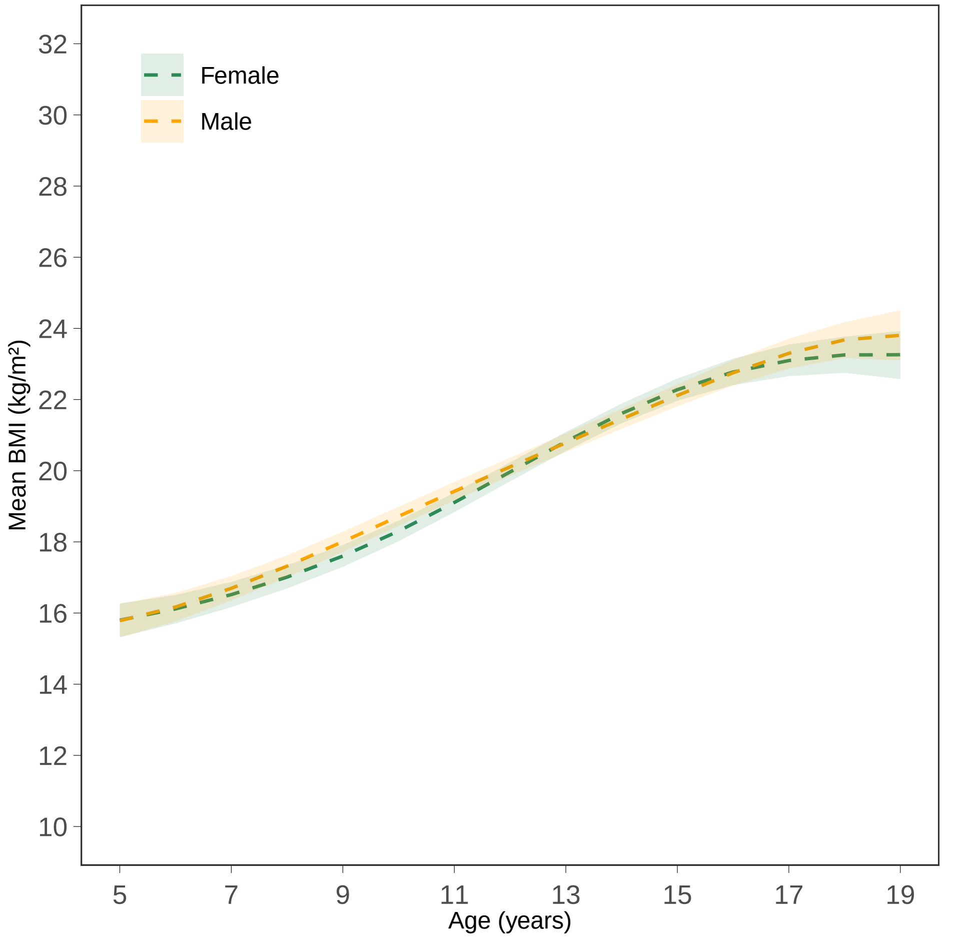

Central African Republic

Time trends in height of 19 year olds

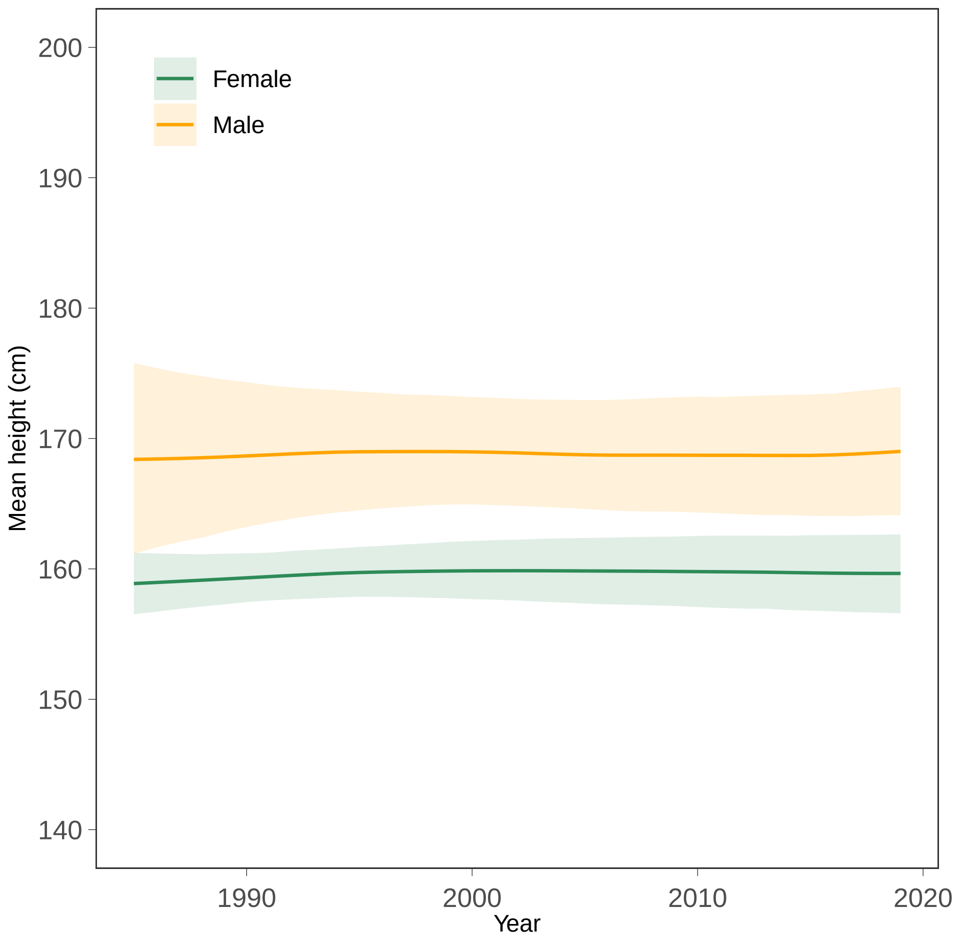

Time trends in BMI of 19 year olds

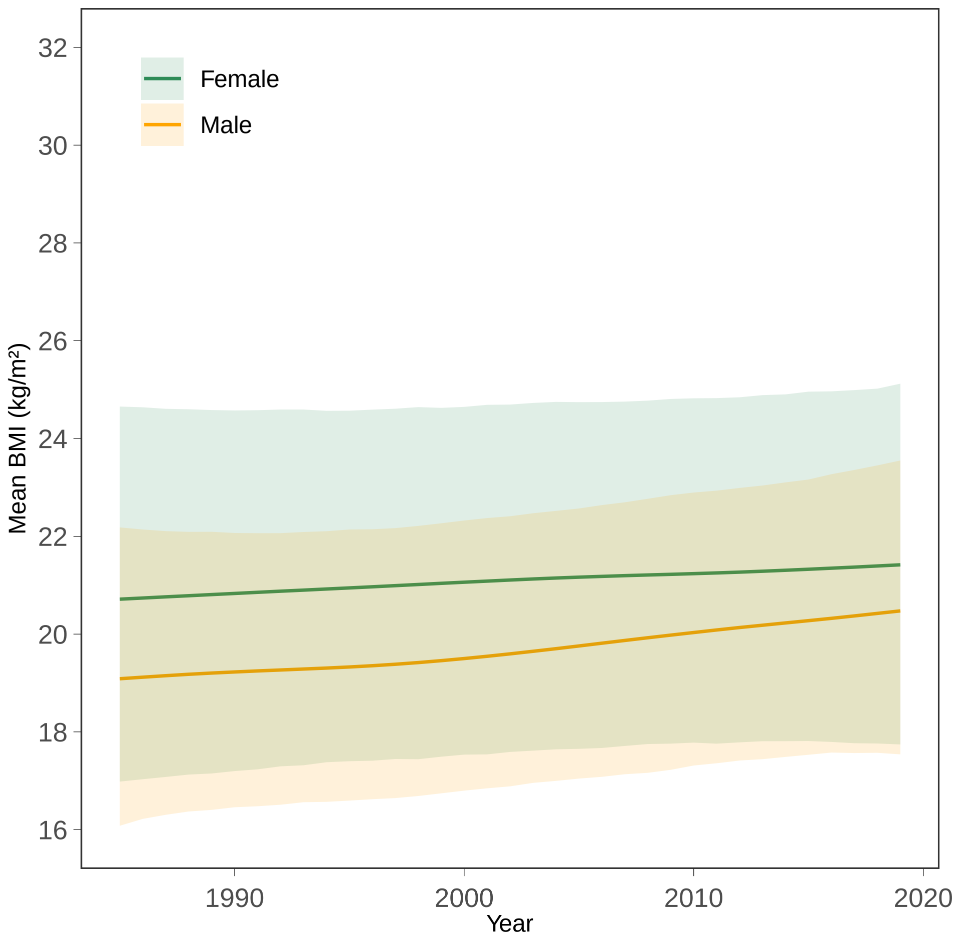

Height−for−age trajectories (2000 birth cohort)

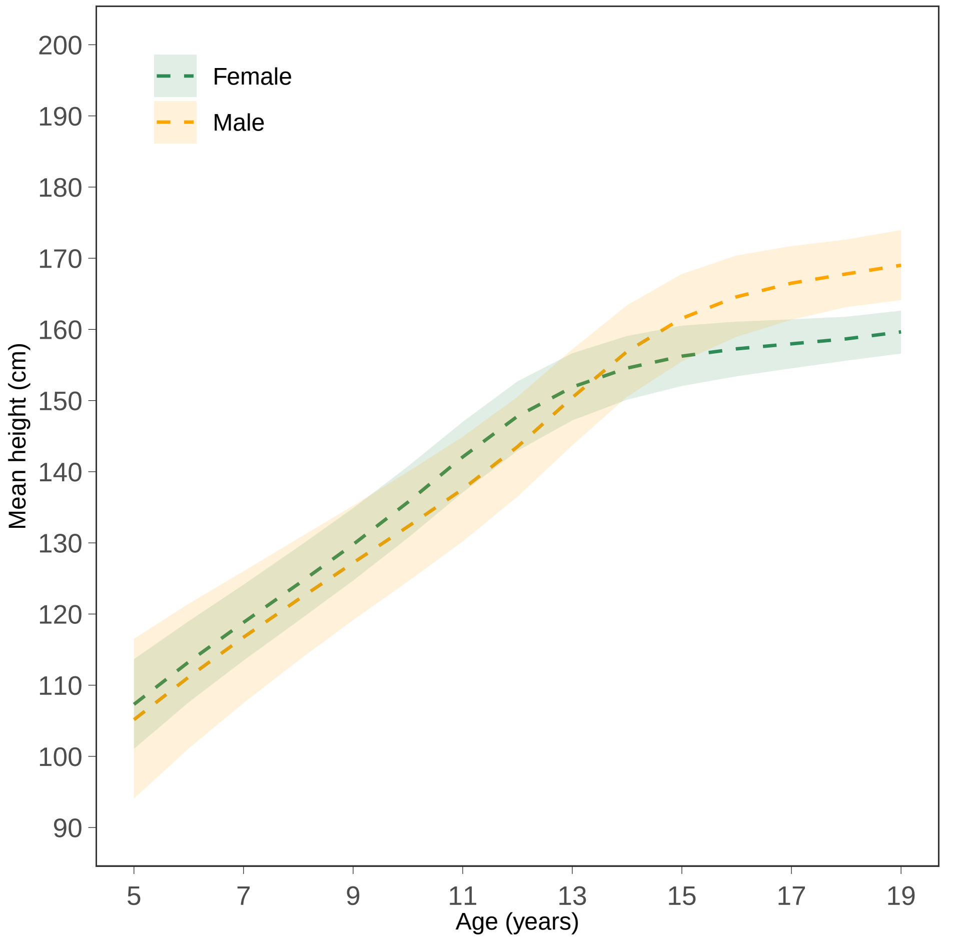

BMI−for−age trajectories (2000 birth cohort)

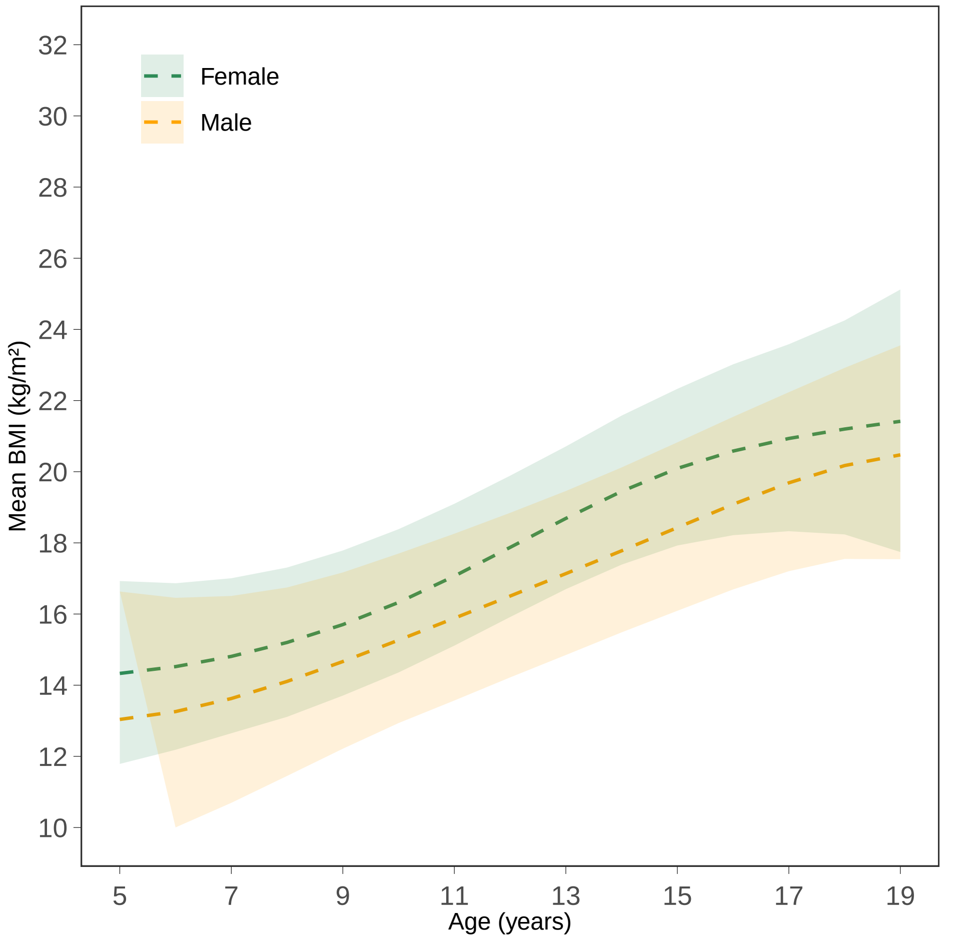

Chad

Time trends in height of 19 year olds

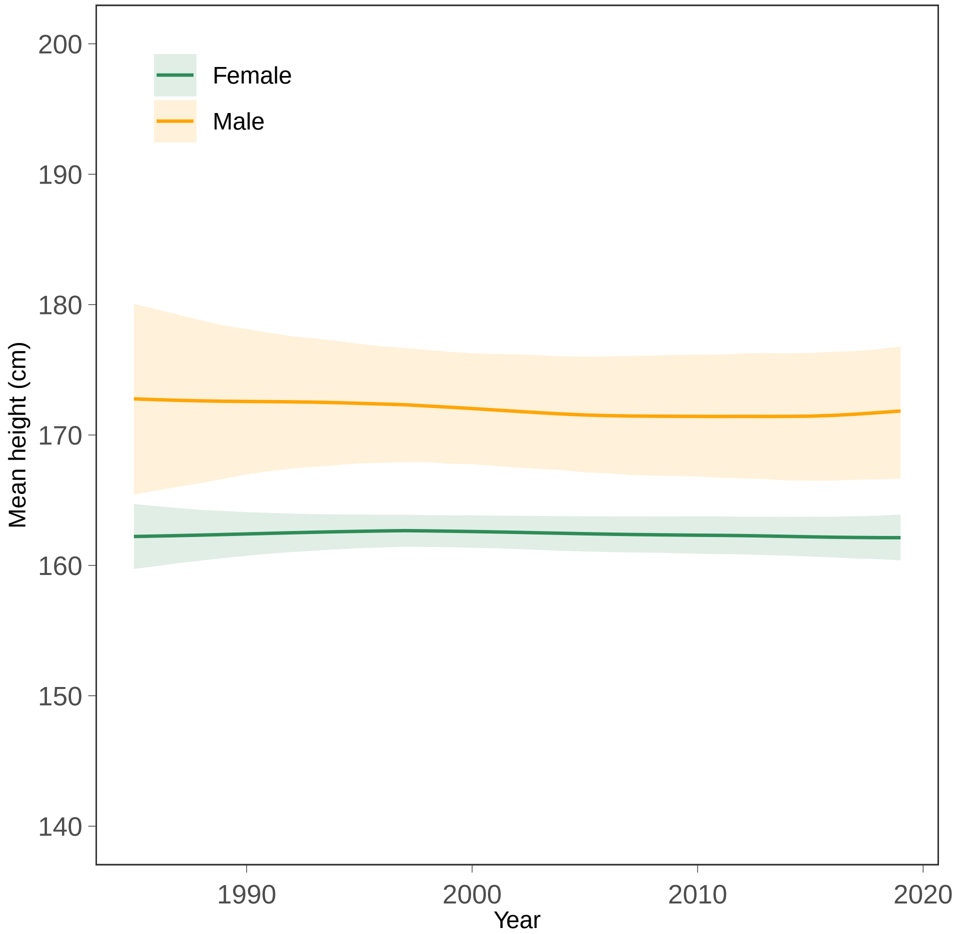

Time trends in BMI of 19 year olds

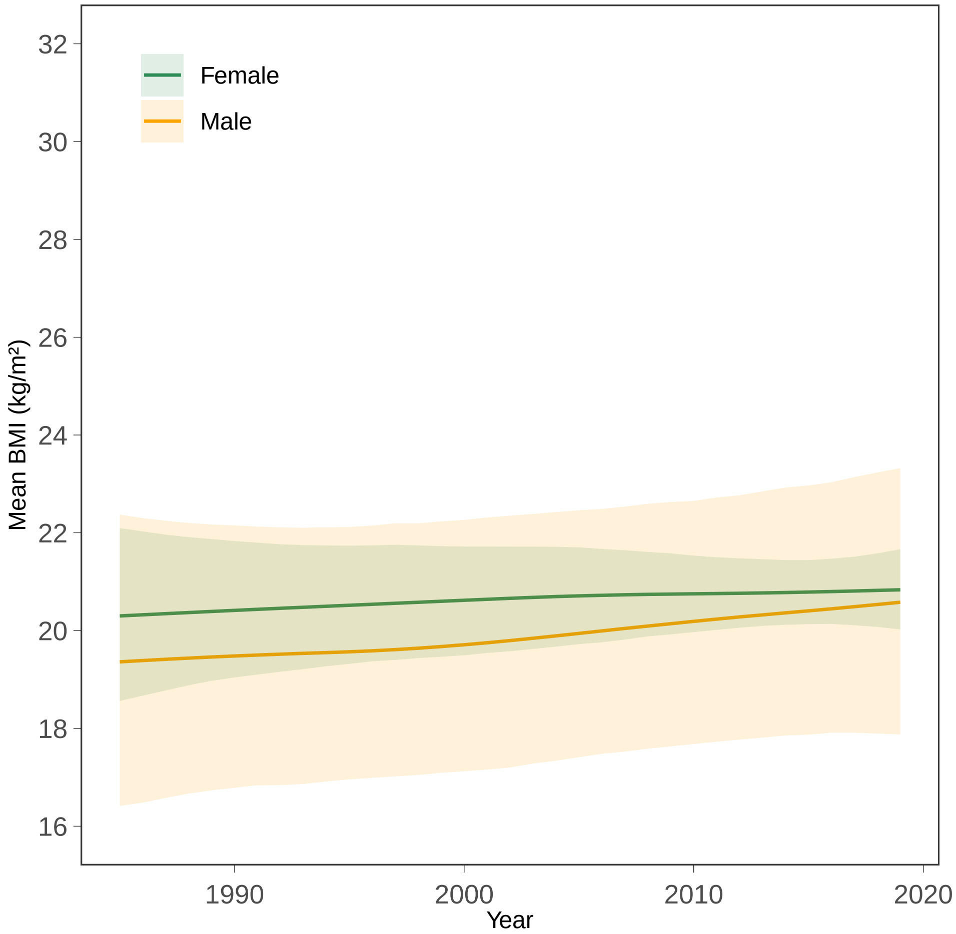

Height−for−age trajectories (2000 birth cohort)

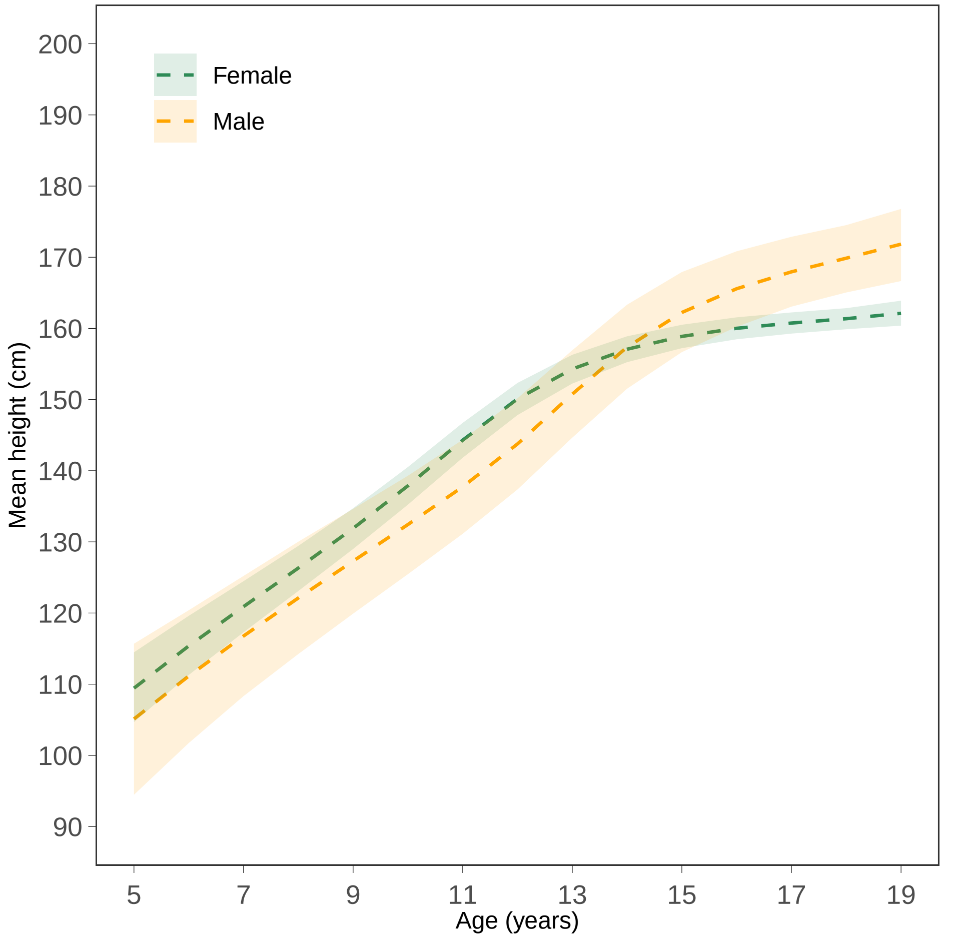

BMI−for−age trajectories (2000 birth cohort)

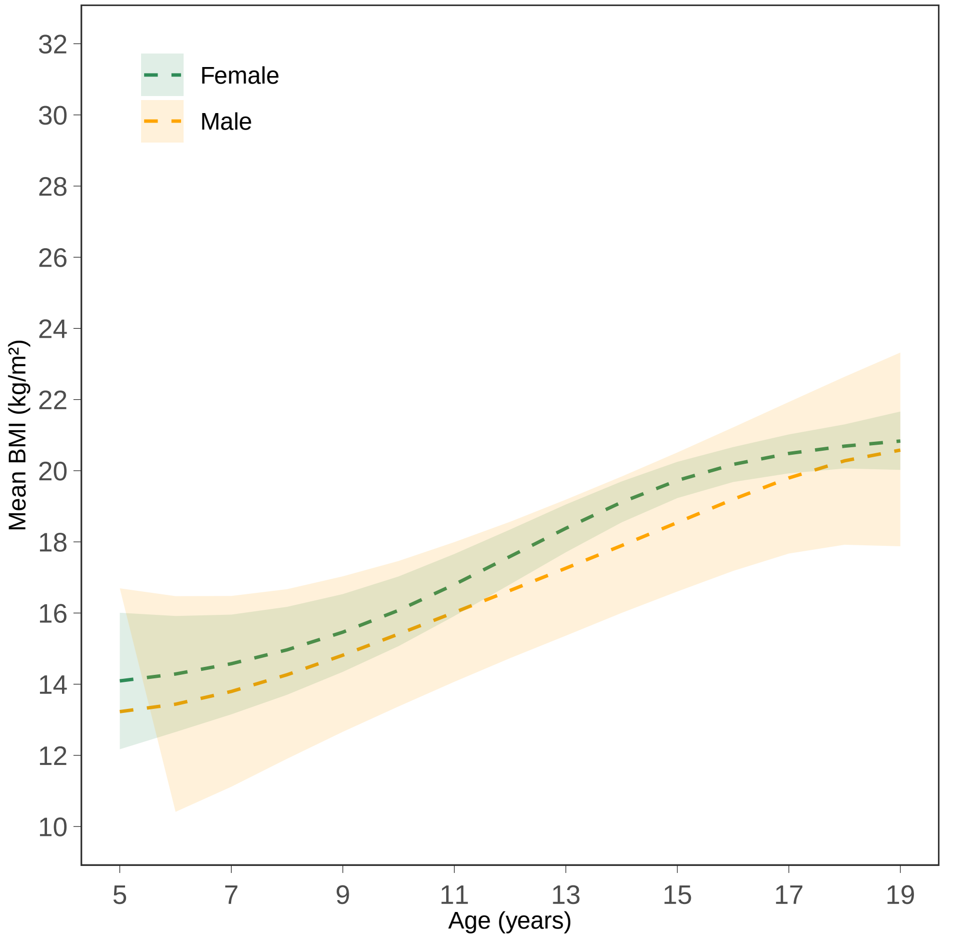

Chile

Time trends in height of 19 year olds

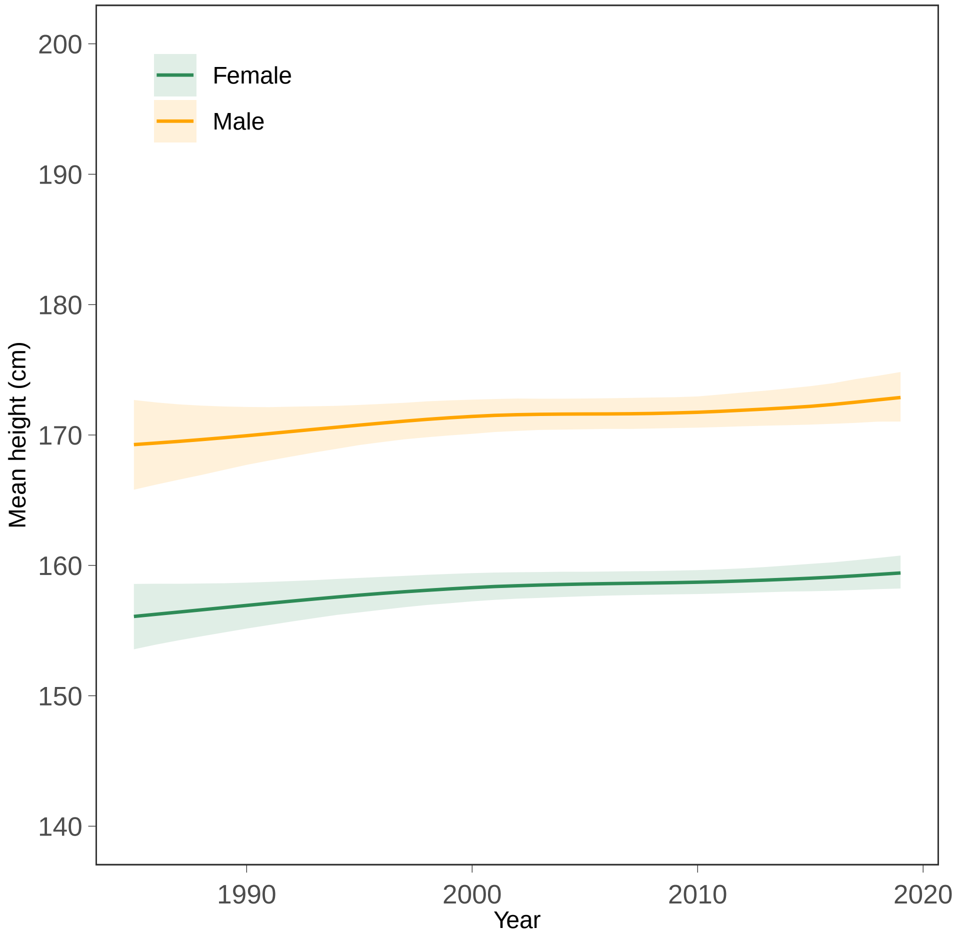

Time trends in BMI of 19 year olds

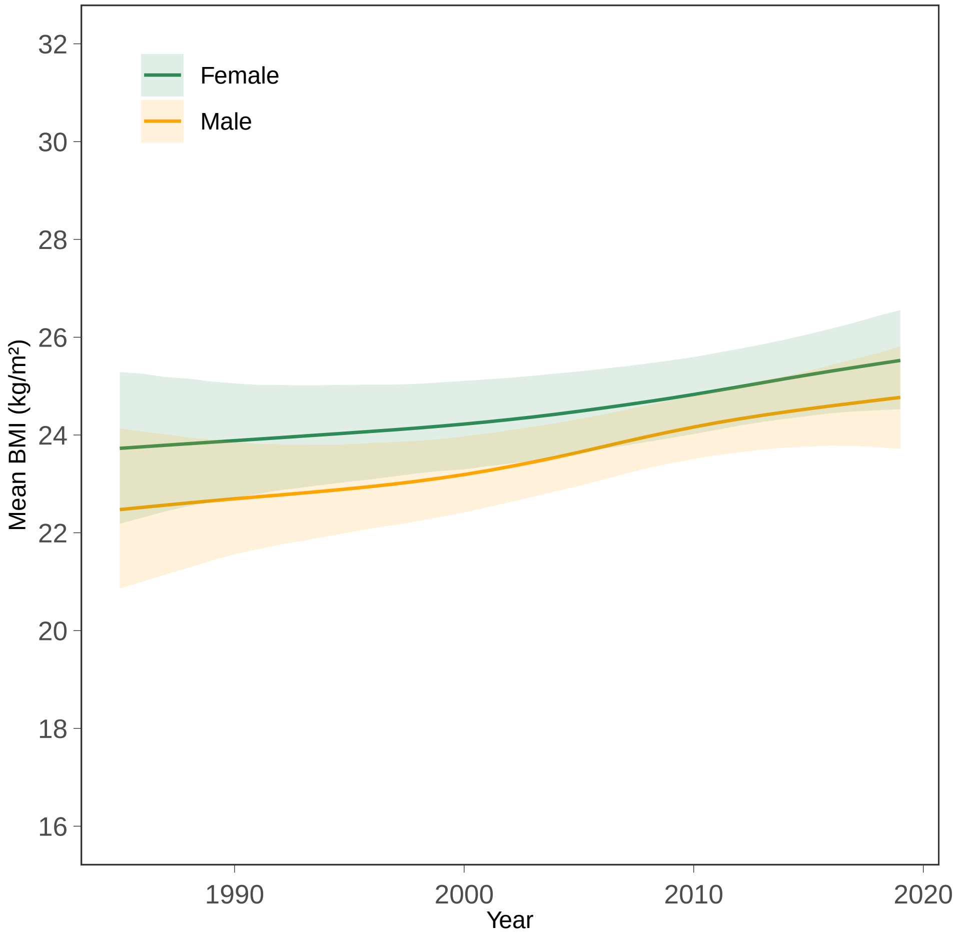

Height−for−age trajectories (2000 birth cohort)

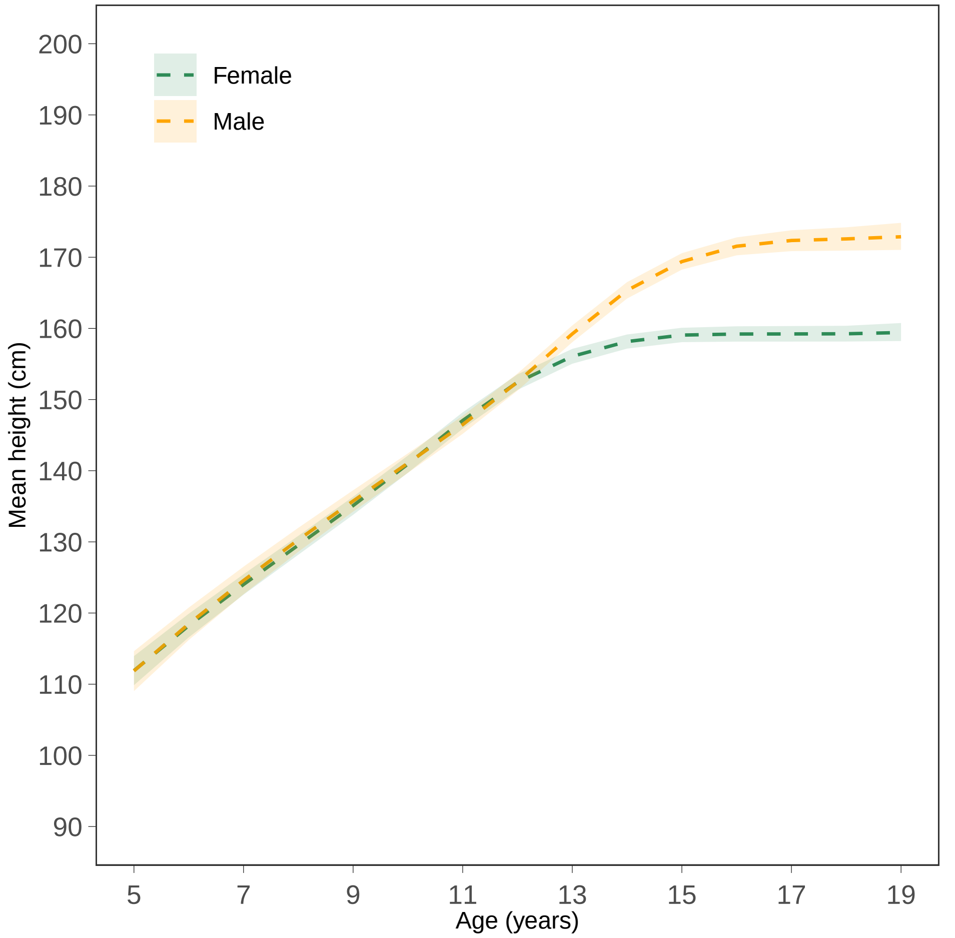

BMI−for−age trajectories (2000 birth cohort)

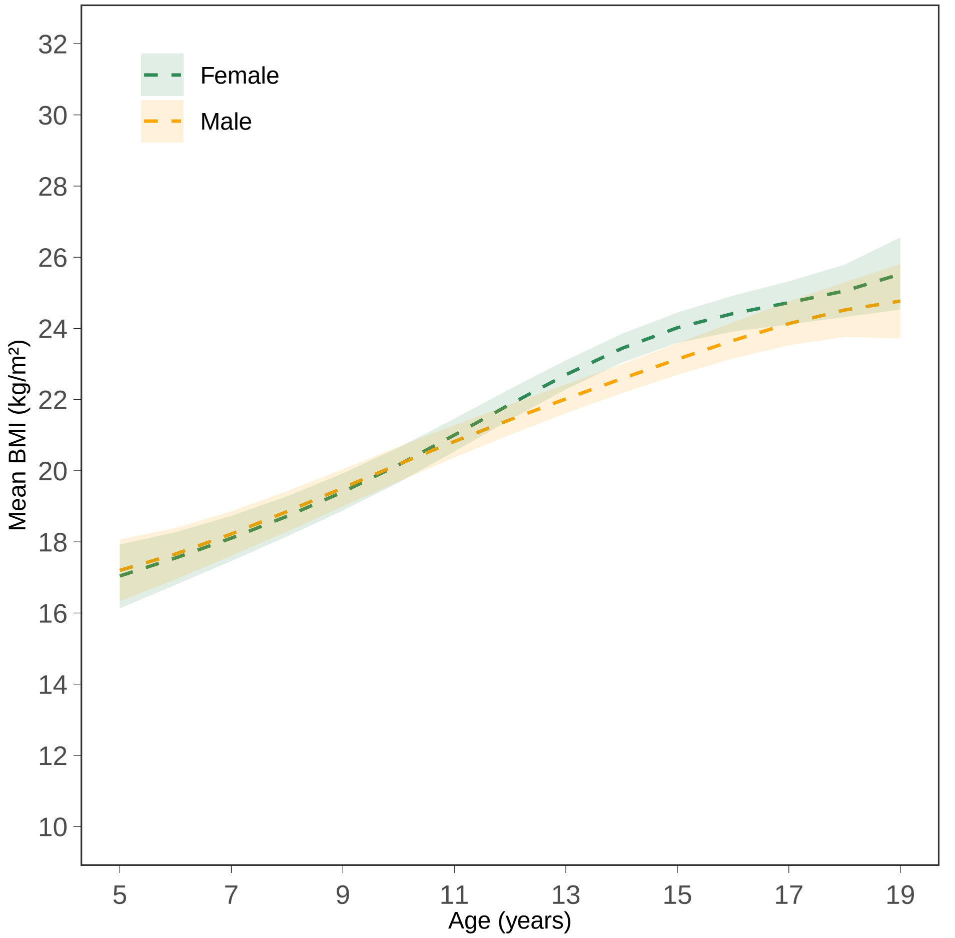

China (Hong Kong SAR)

Time trends in height of 19 year olds

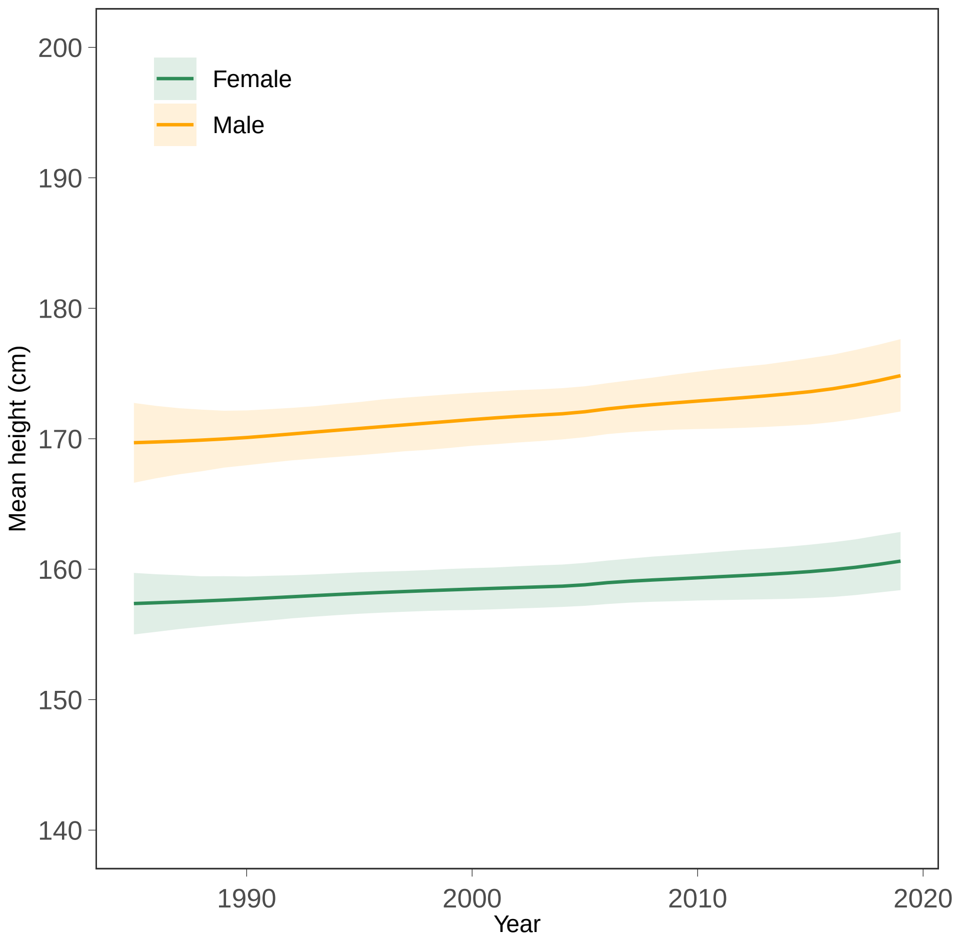

Time trends in BMI of 19 year olds

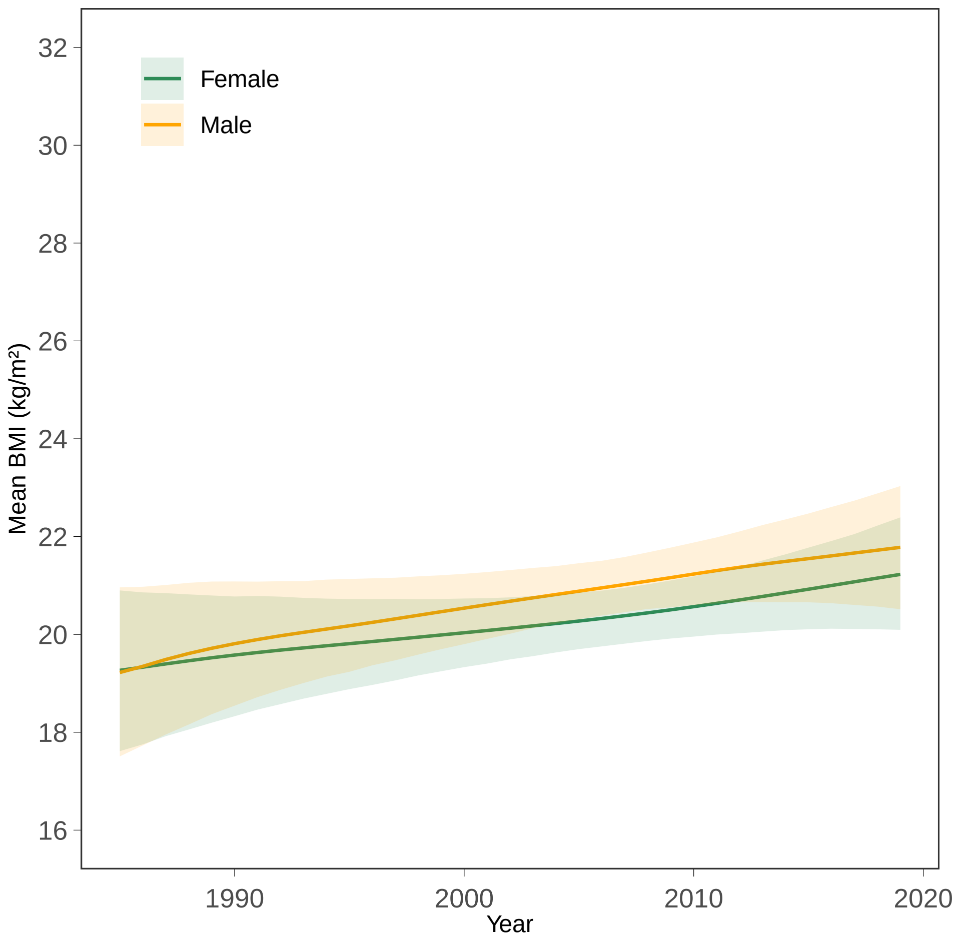

Height−for−age trajectories (2000 birth cohort)

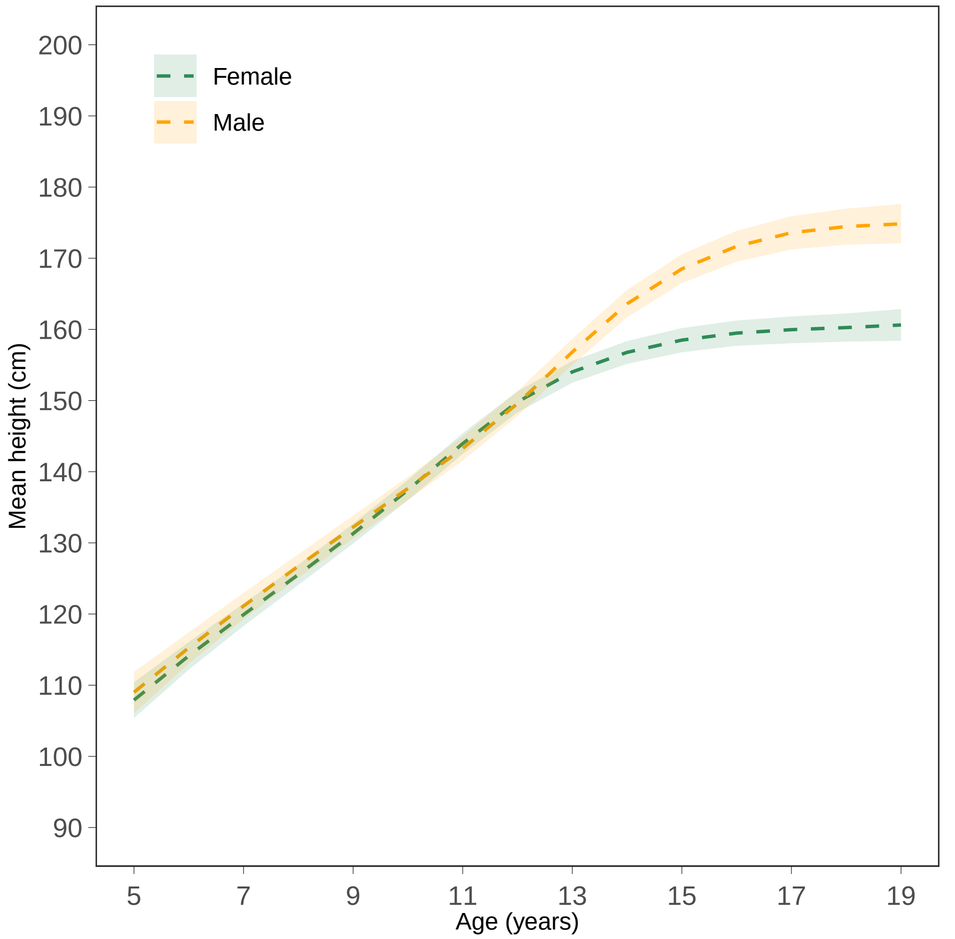

BMI−for−age trajectories (2000 birth cohort)

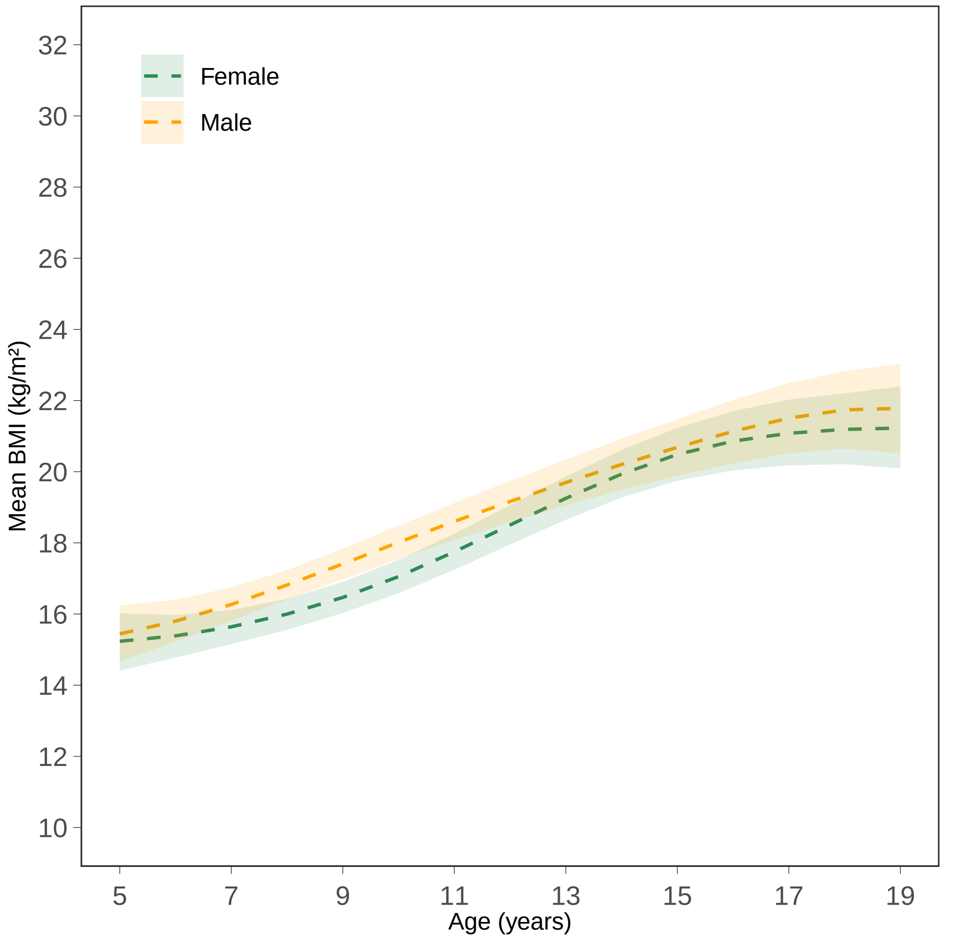

China

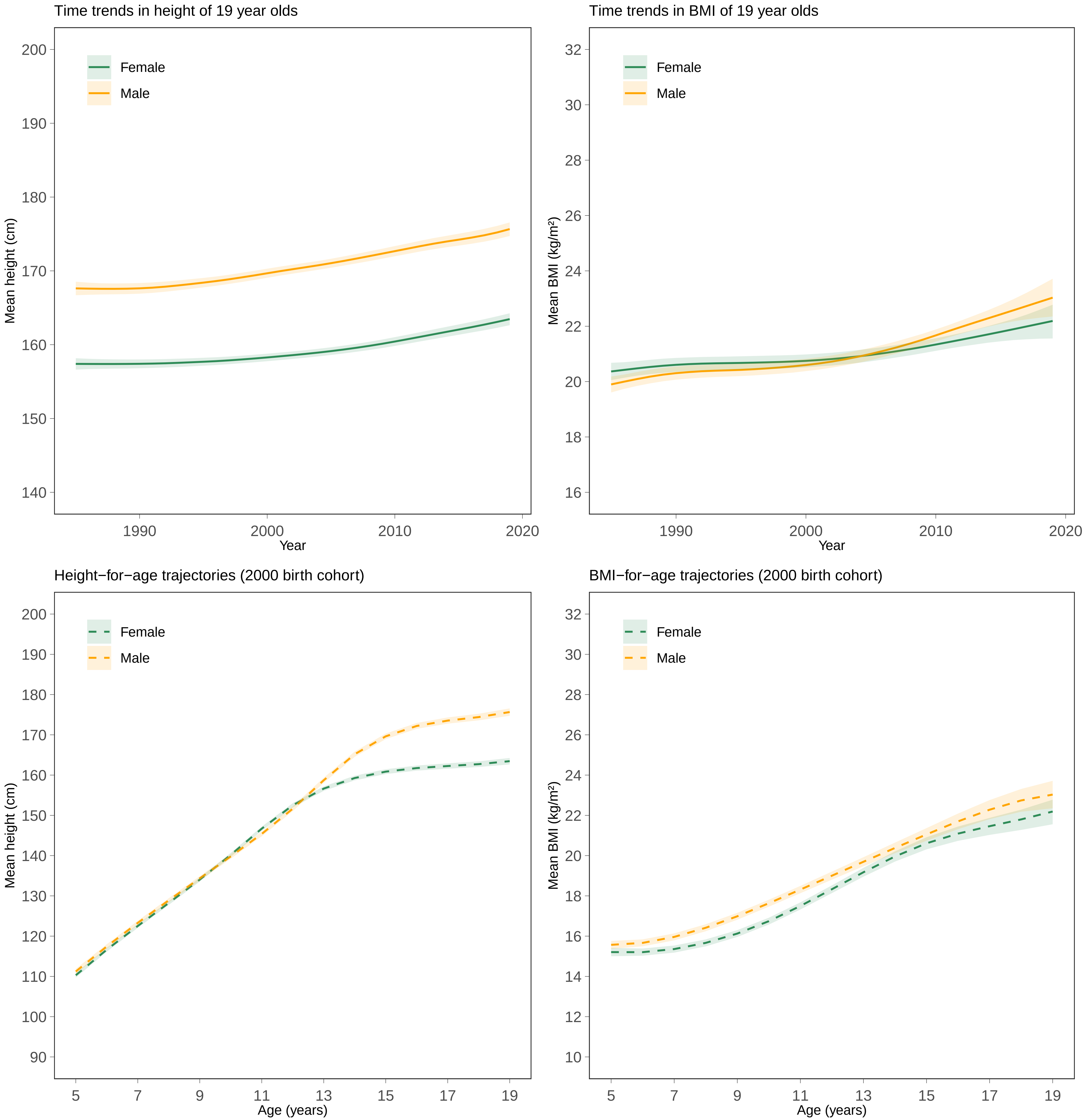
Time trends in height of 19 year olds
Female
Male
Mean height (cm)
200
190
180
170
160
150
140
1990
2000
2010
2020
Year
Time trends in BMI of 19 year olds
Female
Male
Mean BMI (kg/m²)
32
30
28
26
24
22
20
18
16
1990
2000
2010
2020
Year
Height−for−age trajectories (2000 birth cohort)
Female
Male
Mean height (cm)
200
190
180
170
160
150
140
130
120
110
100
90
5
7
9
11
13
15
17
19
Age (years)
BMI−for−age trajectories (2000 birth cohort)
Female
Male
Mean BMI (kg/m²)
32
30
28
26
24
22
20
18
16
14
12
10
5
7
9
11
13
15
17
19
Age (years)

Colombia

Time trends in height of 19 year olds

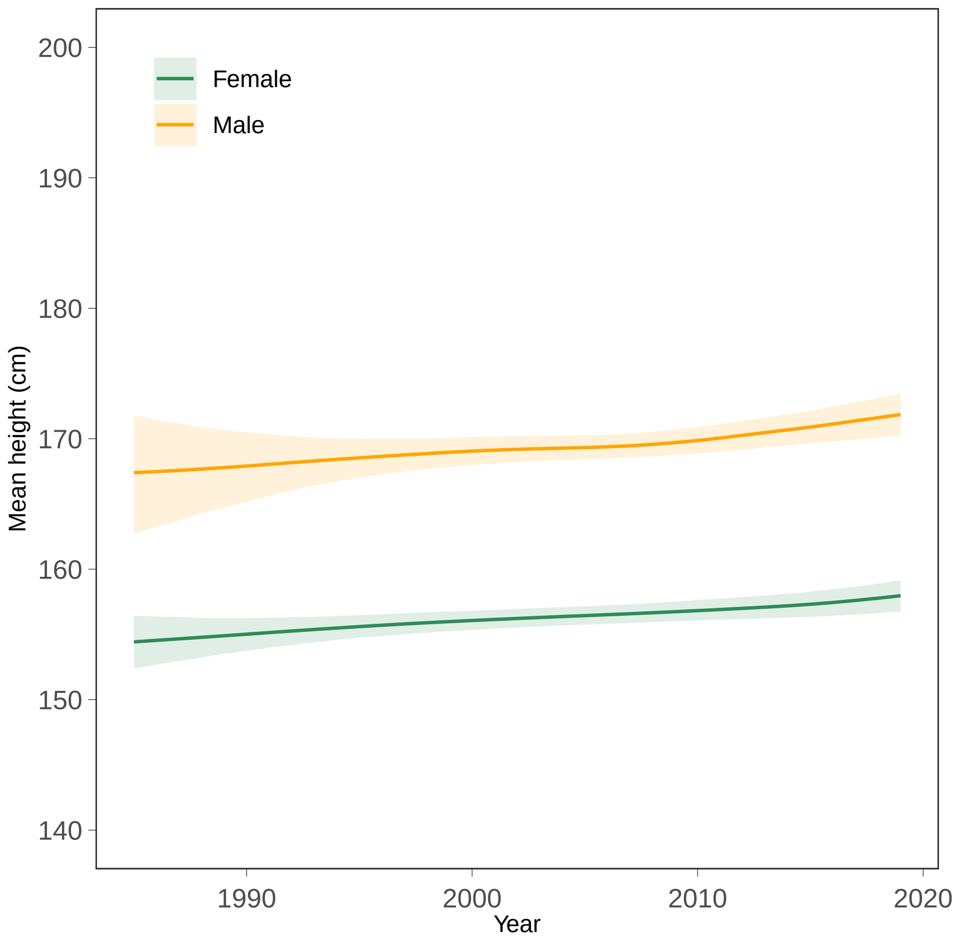

Time trends in BMI of 19 year olds

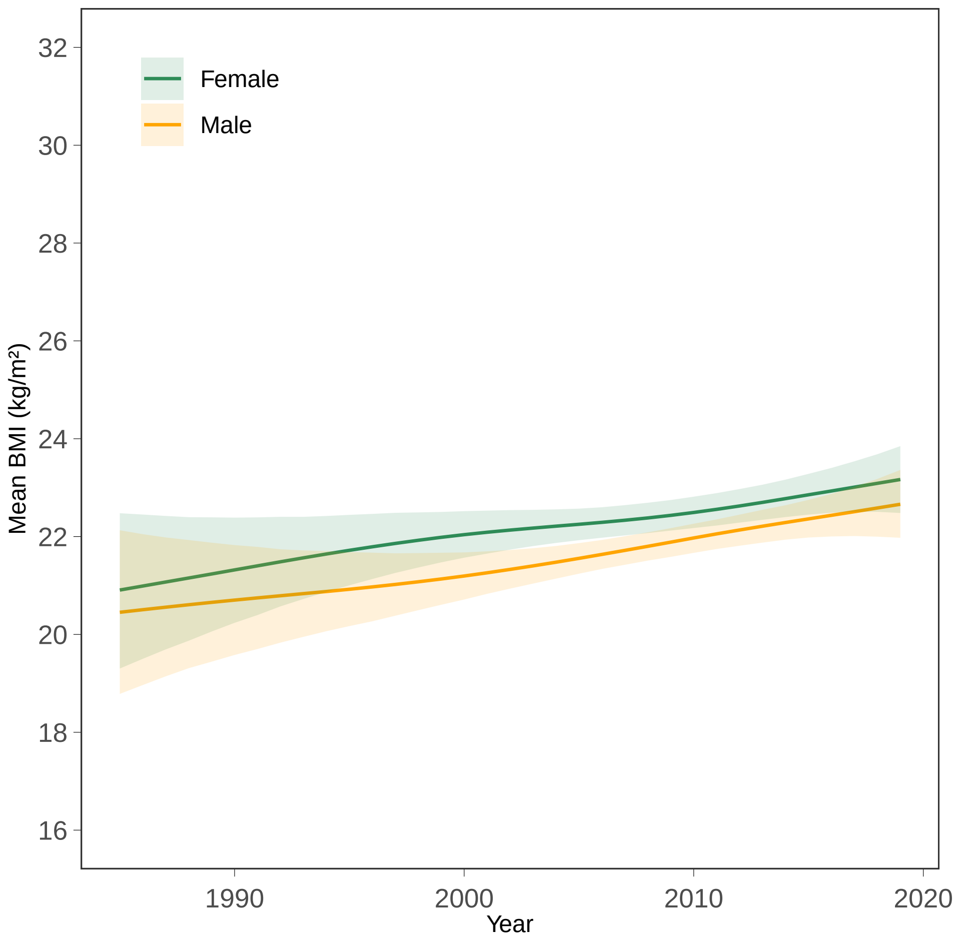

Height−for−age trajectories (2000 birth cohort)

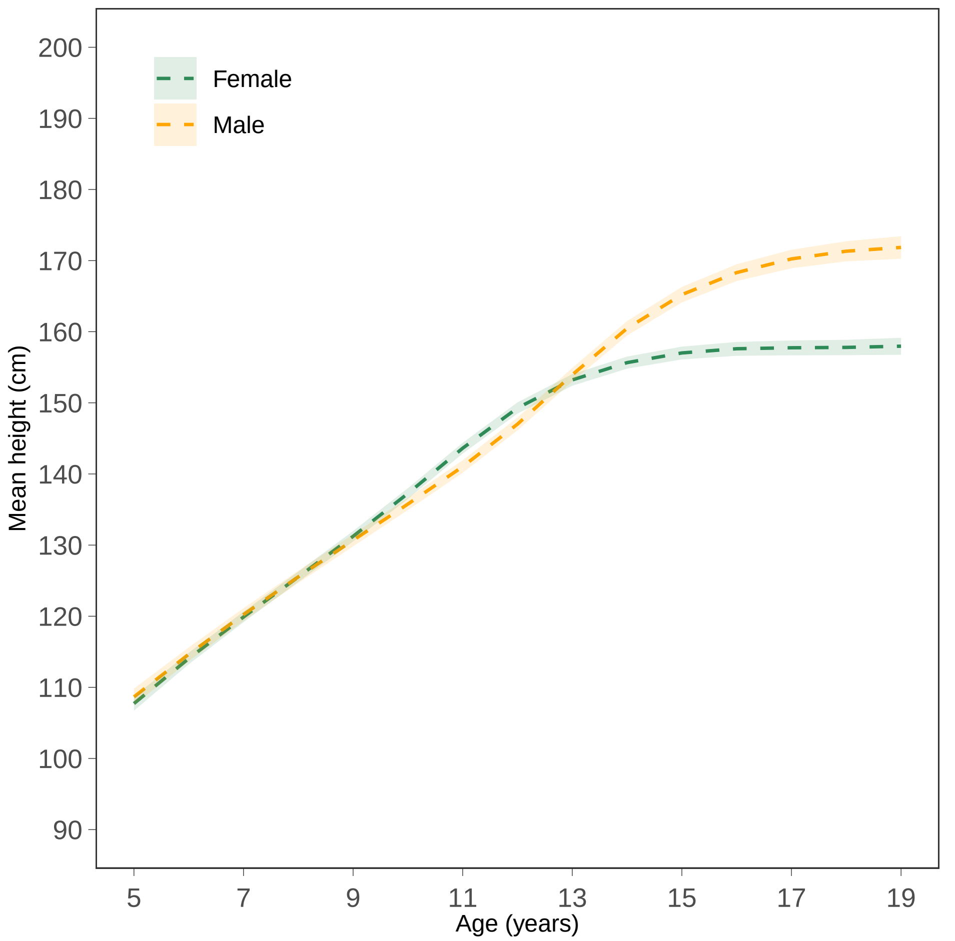

BMI−for−age trajectories (2000 birth cohort)

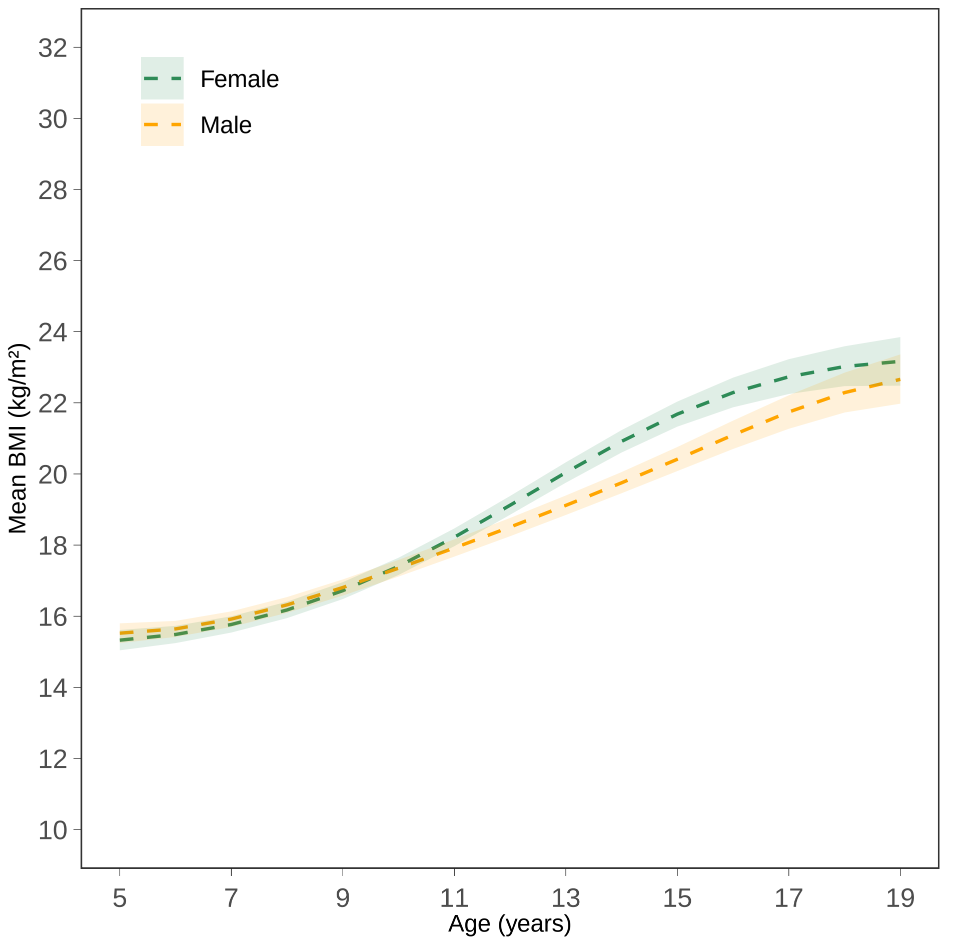

Comoros

Time trends in height of 19 year olds

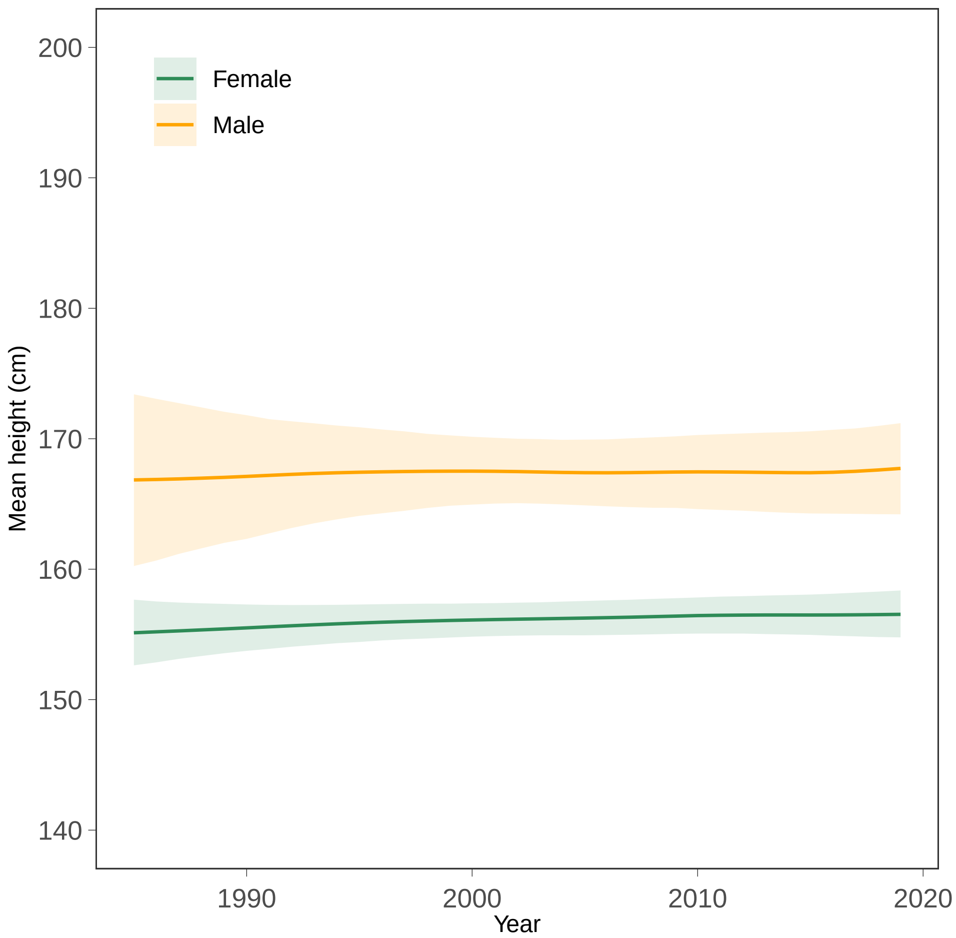

Time trends in BMI of 19 year olds

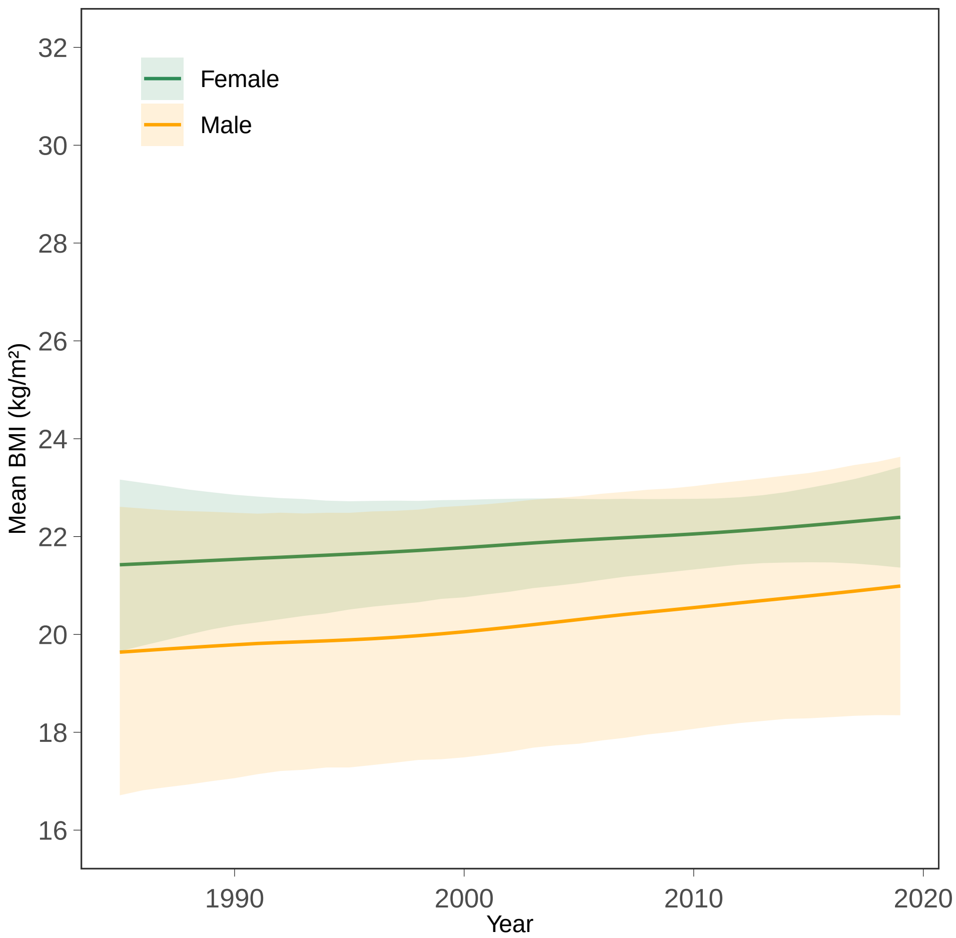

Height−for−age trajectories (2000 birth cohort)

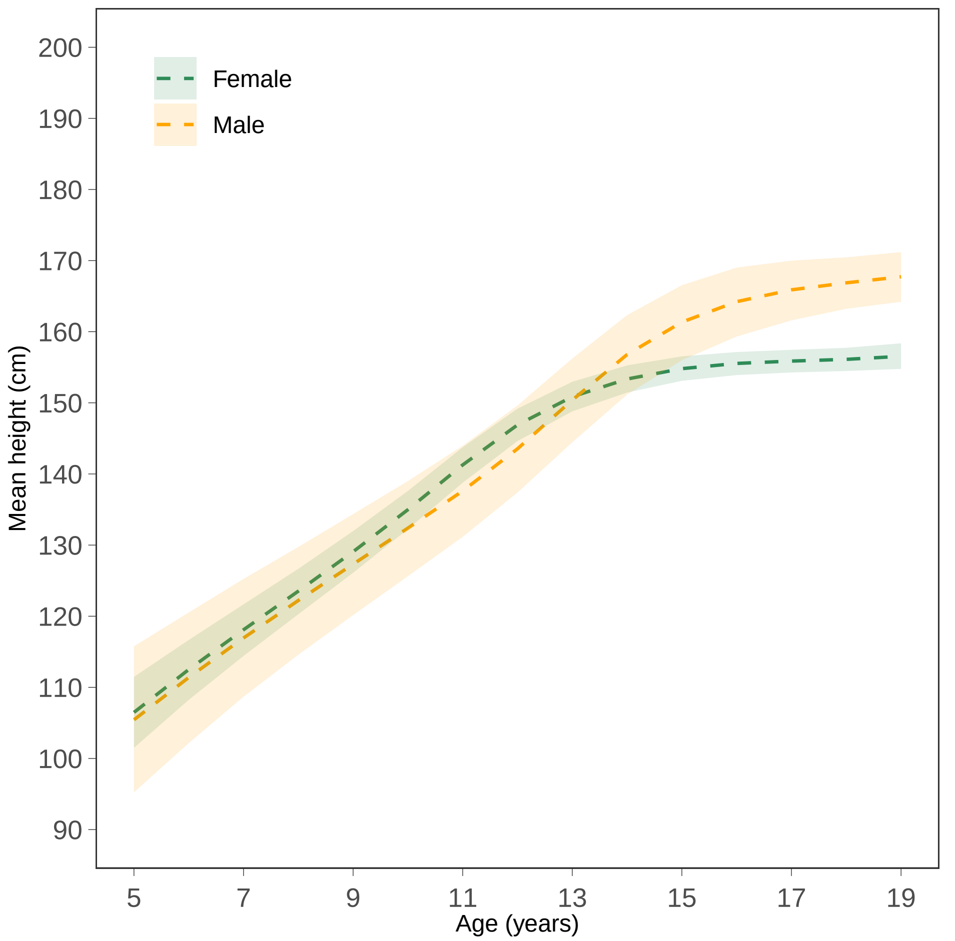

BMI−for−age trajectories (2000 birth cohort)

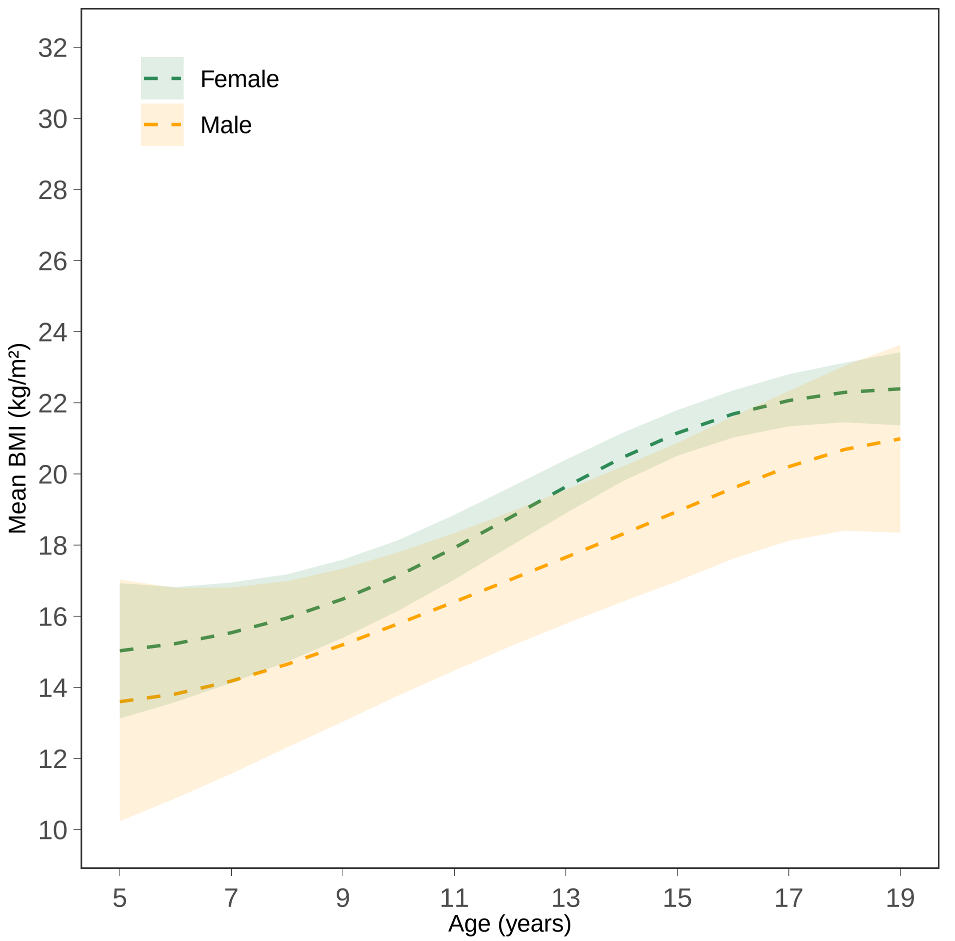

Congo

Time trends in height of 19 year olds

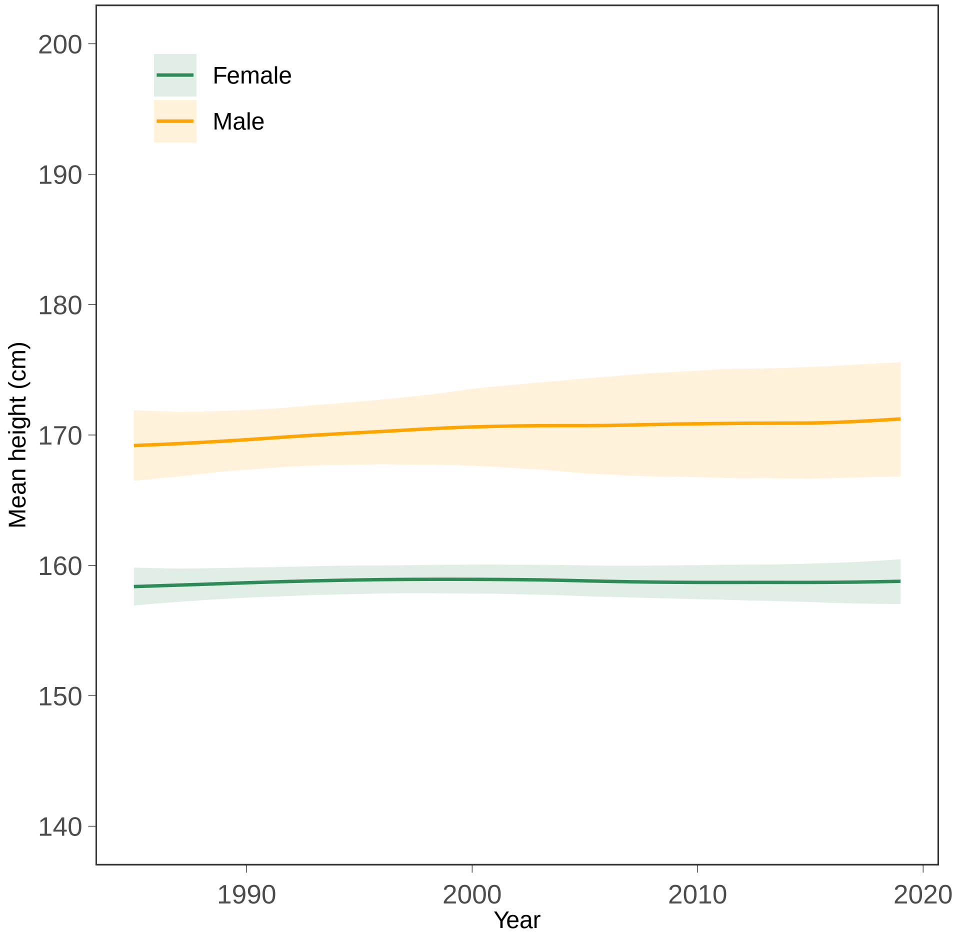

Time trends in BMI of 19 year olds

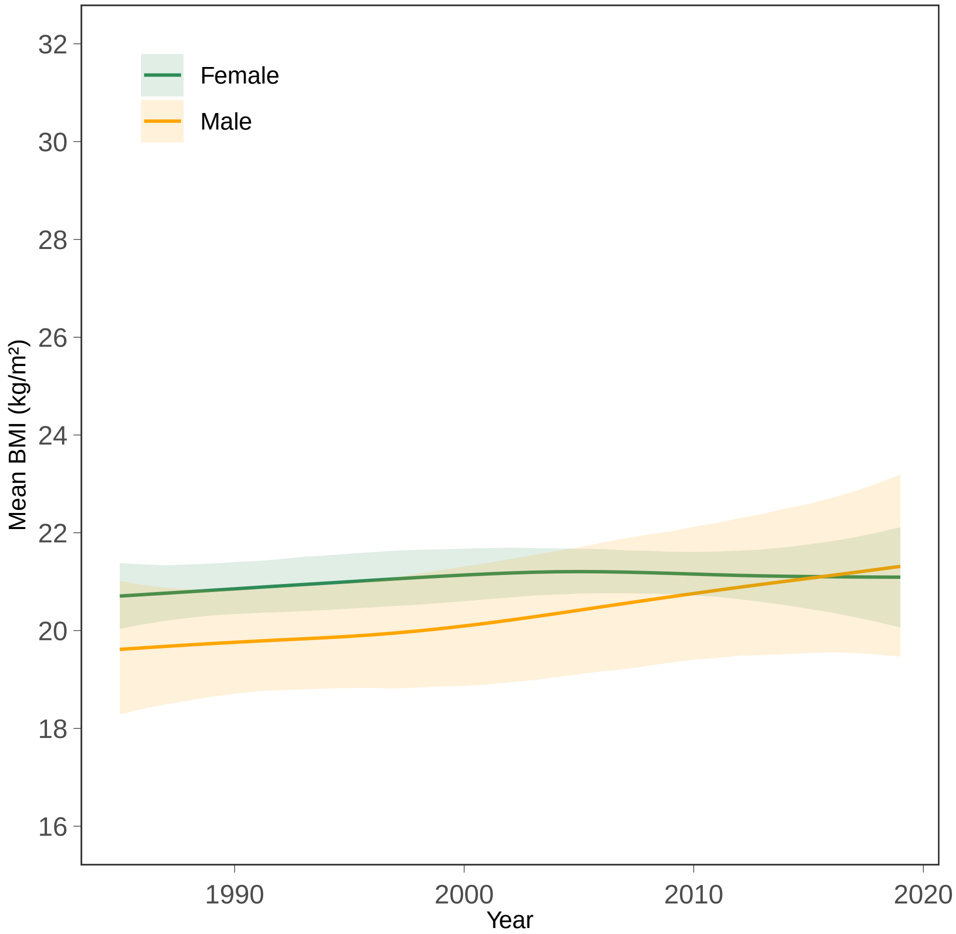

Height−for−age trajectories (2000 birth cohort)

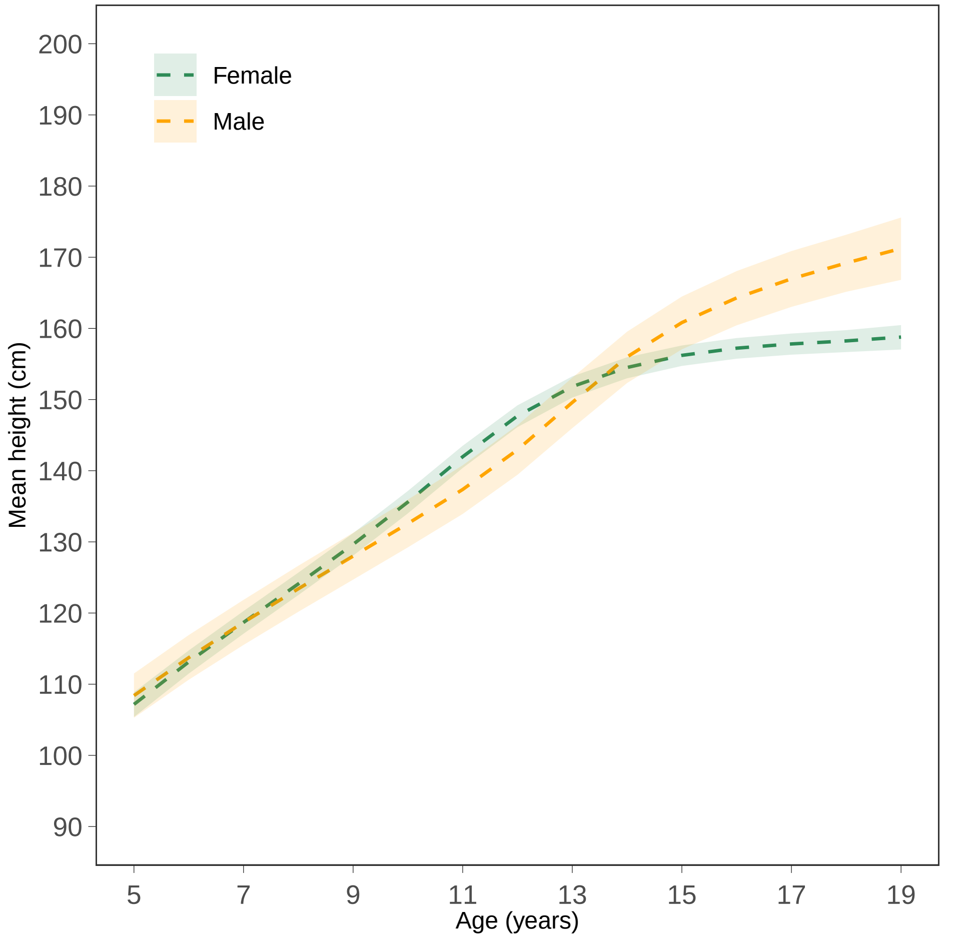

BMI−for−age trajectories (2000 birth cohort)

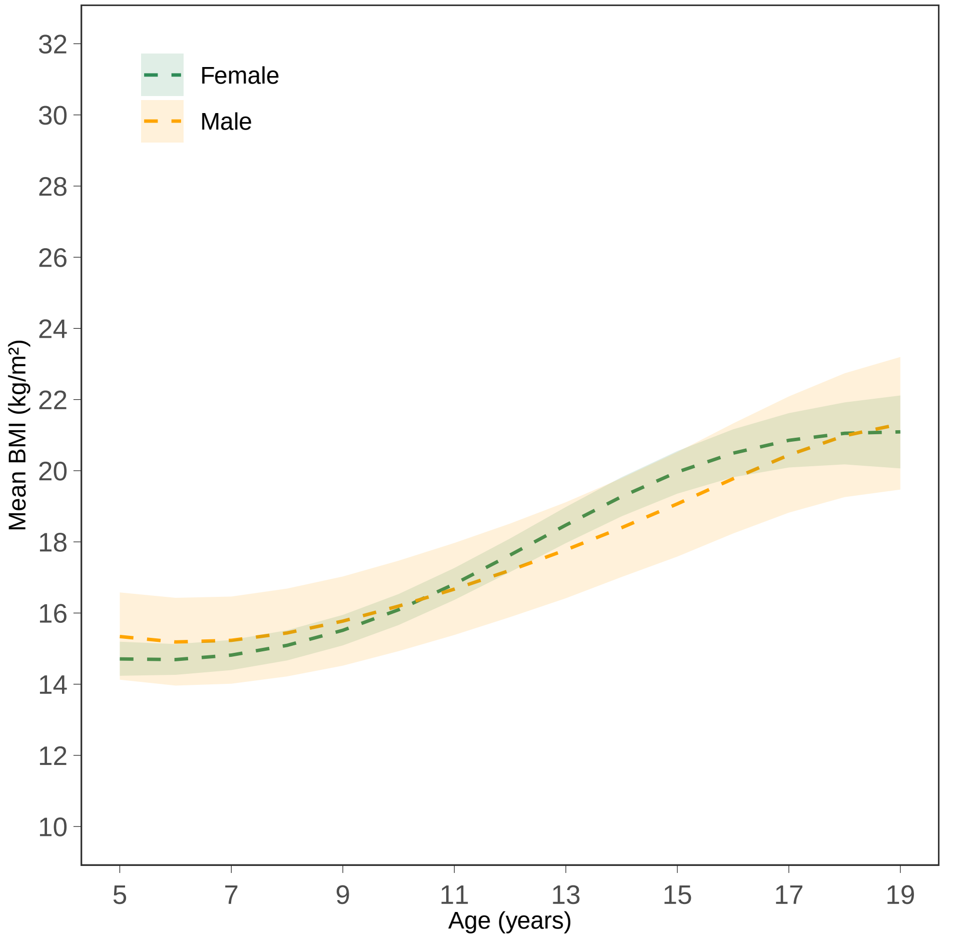

Cook Islands

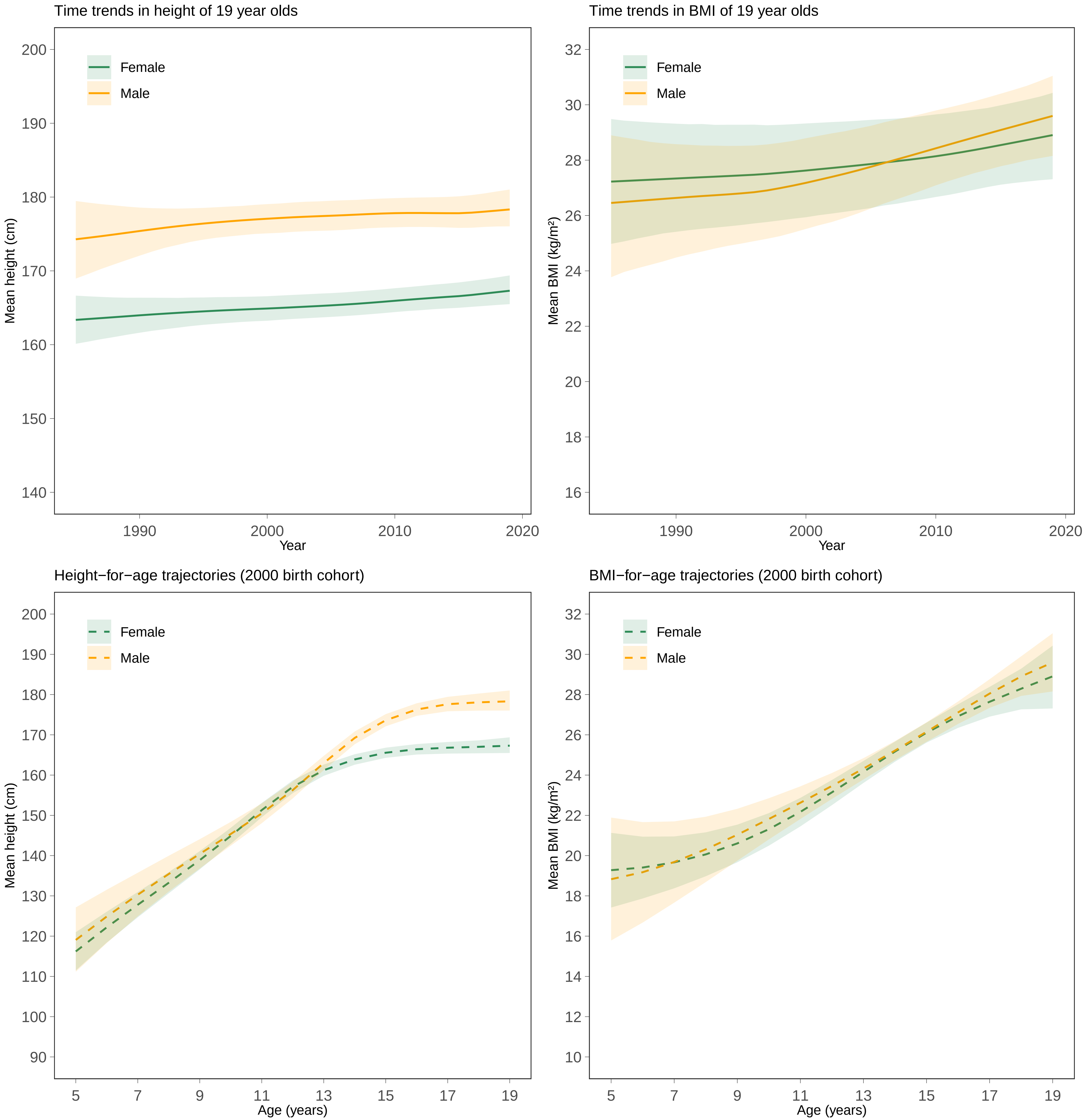

Costa Rica

Time trends in height of 19 year olds

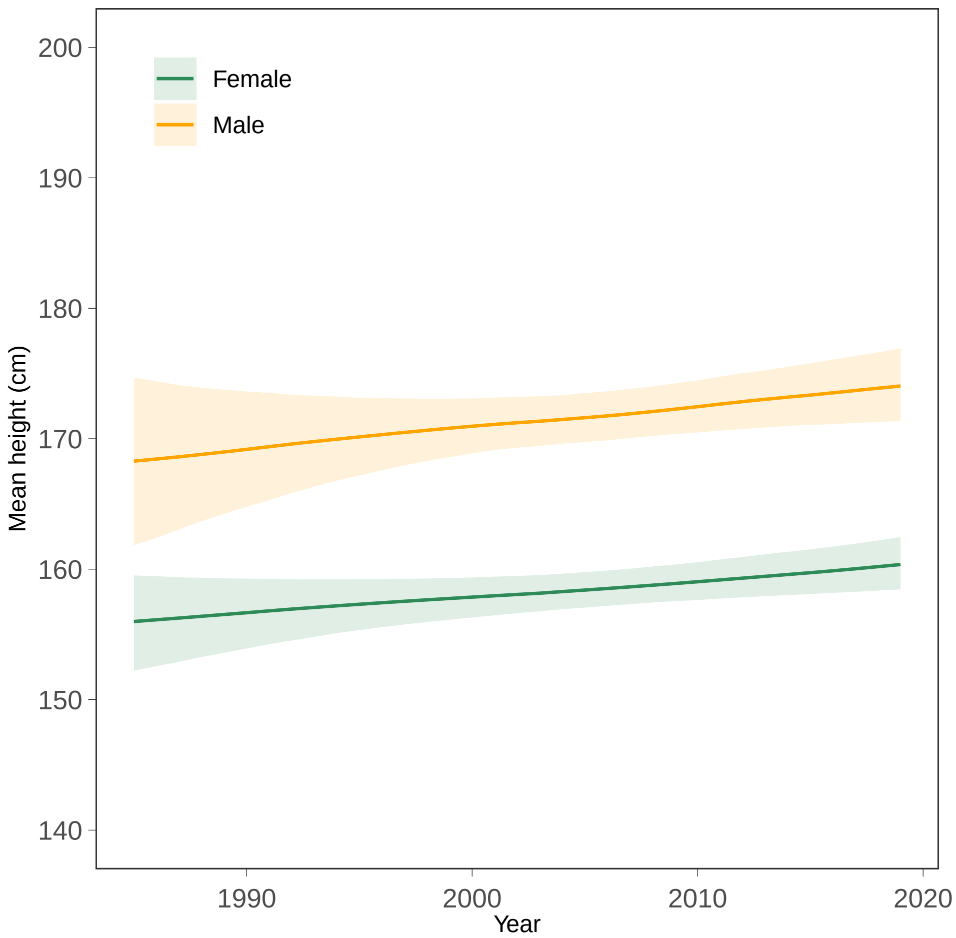

Time trends in BMI of 19 year olds

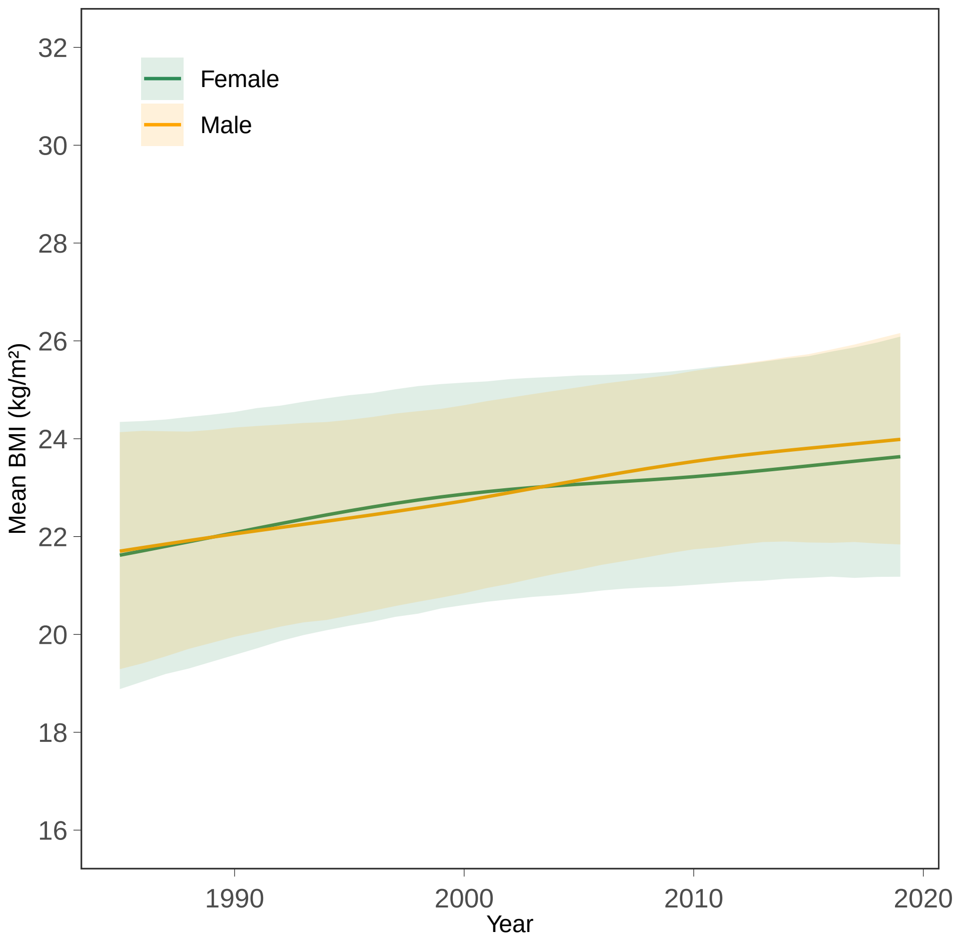

Height−for−age trajectories (2000 birth cohort)

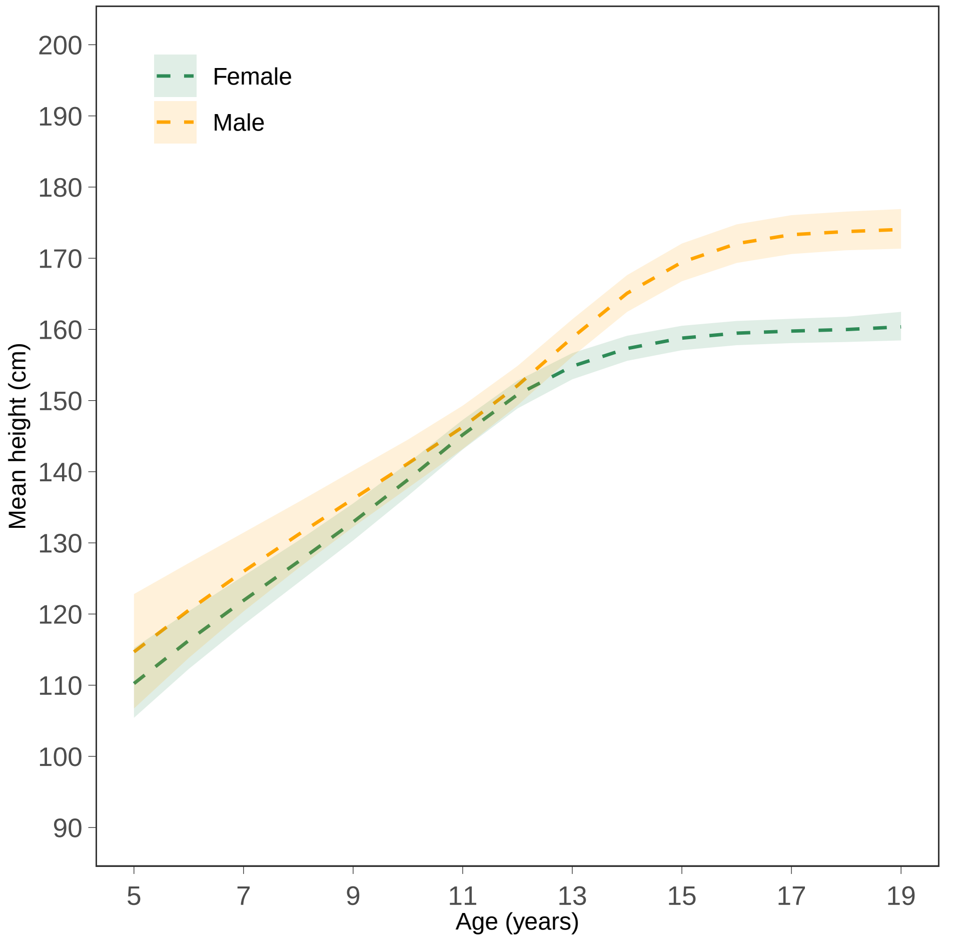

BMI−for−age trajectories (2000 birth cohort)

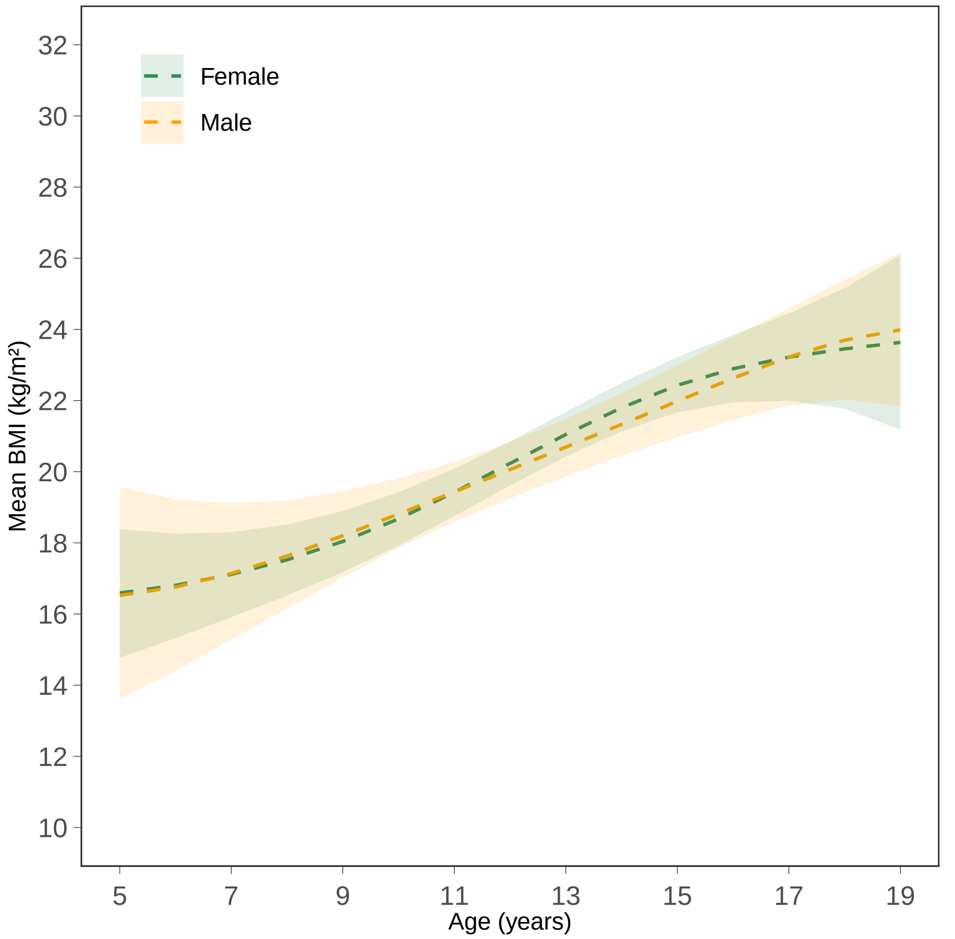

Cote d'Ivoire

Time trends in height of 19 year olds

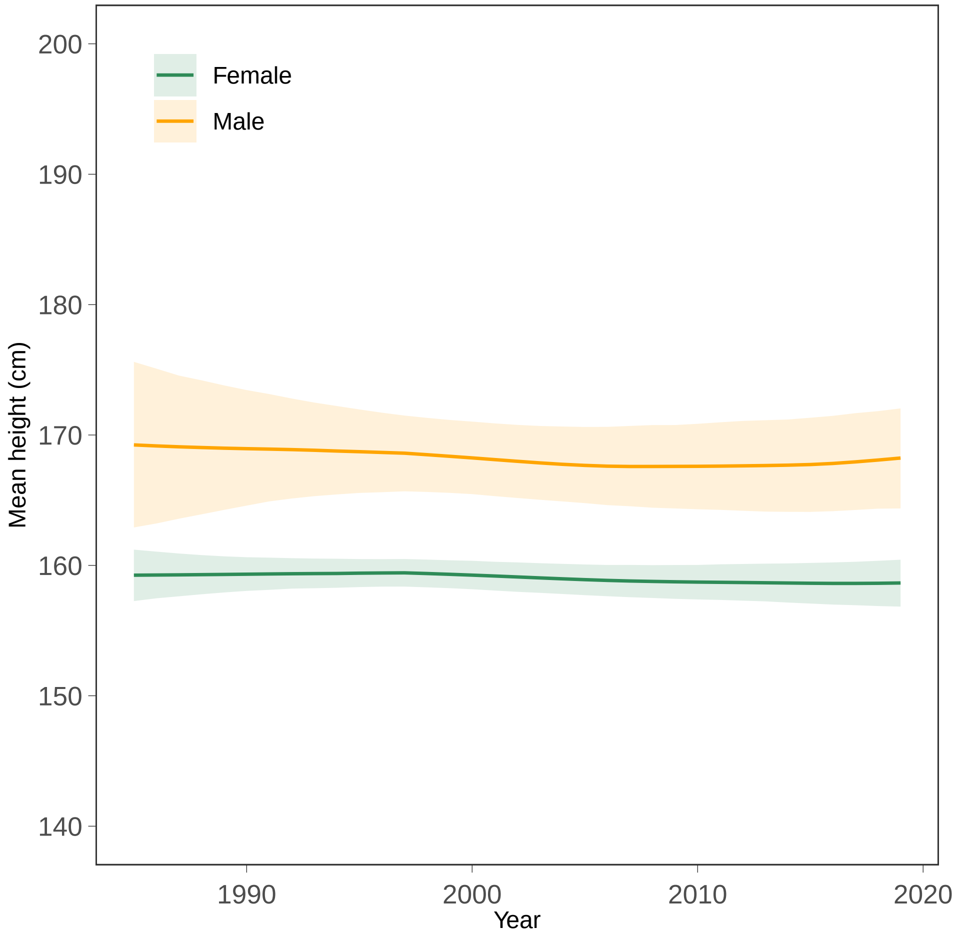

Time trends in BMI of 19 year olds

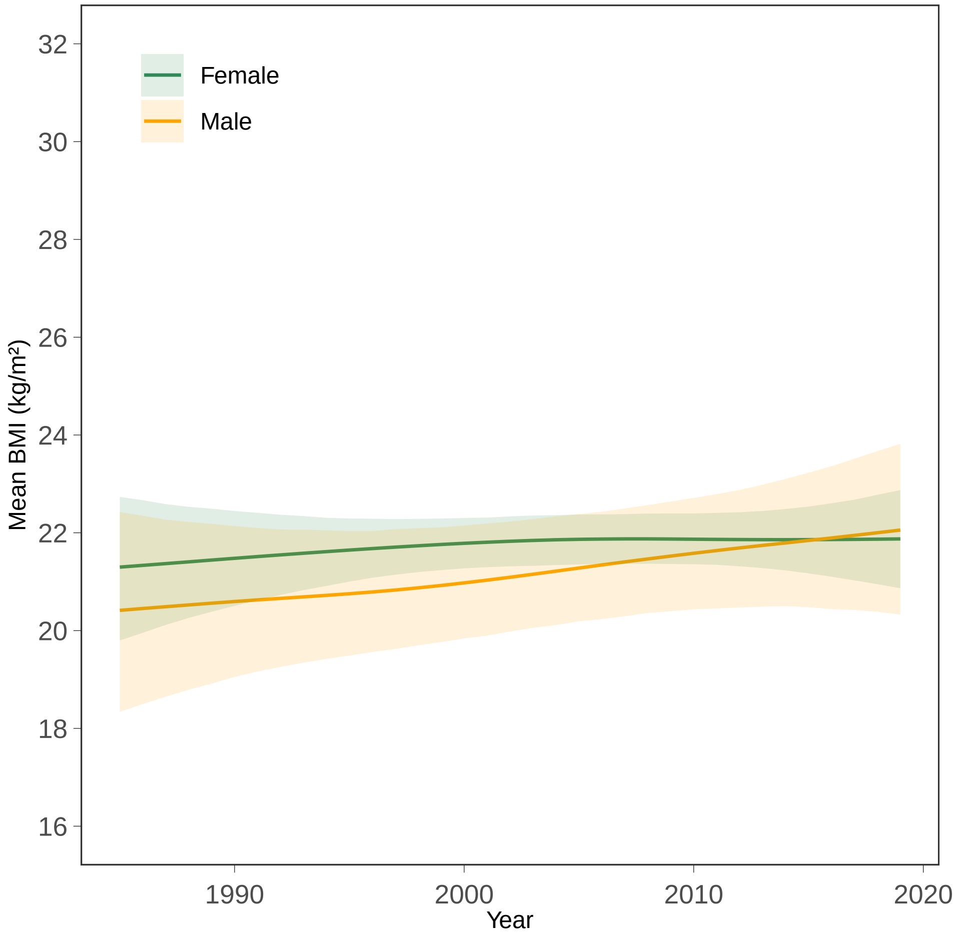

Height−for−age trajectories (2000 birth cohort)

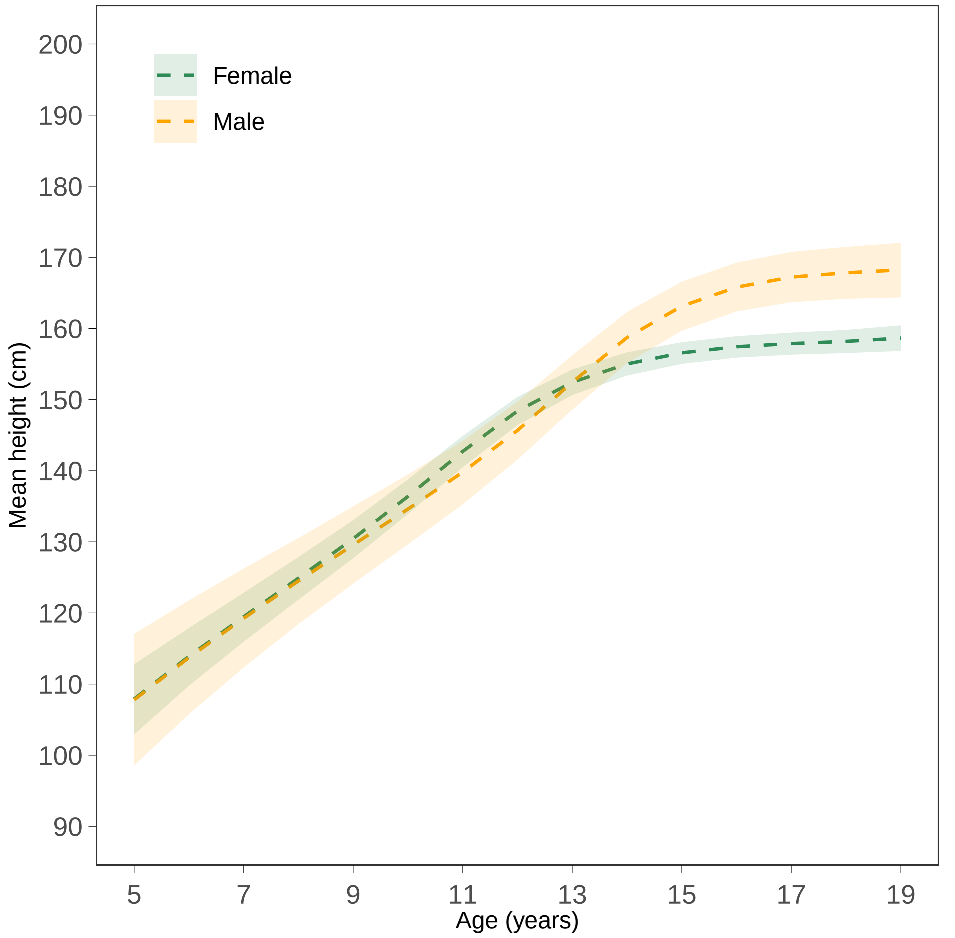

BMI−for−age trajectories (2000 birth cohort)

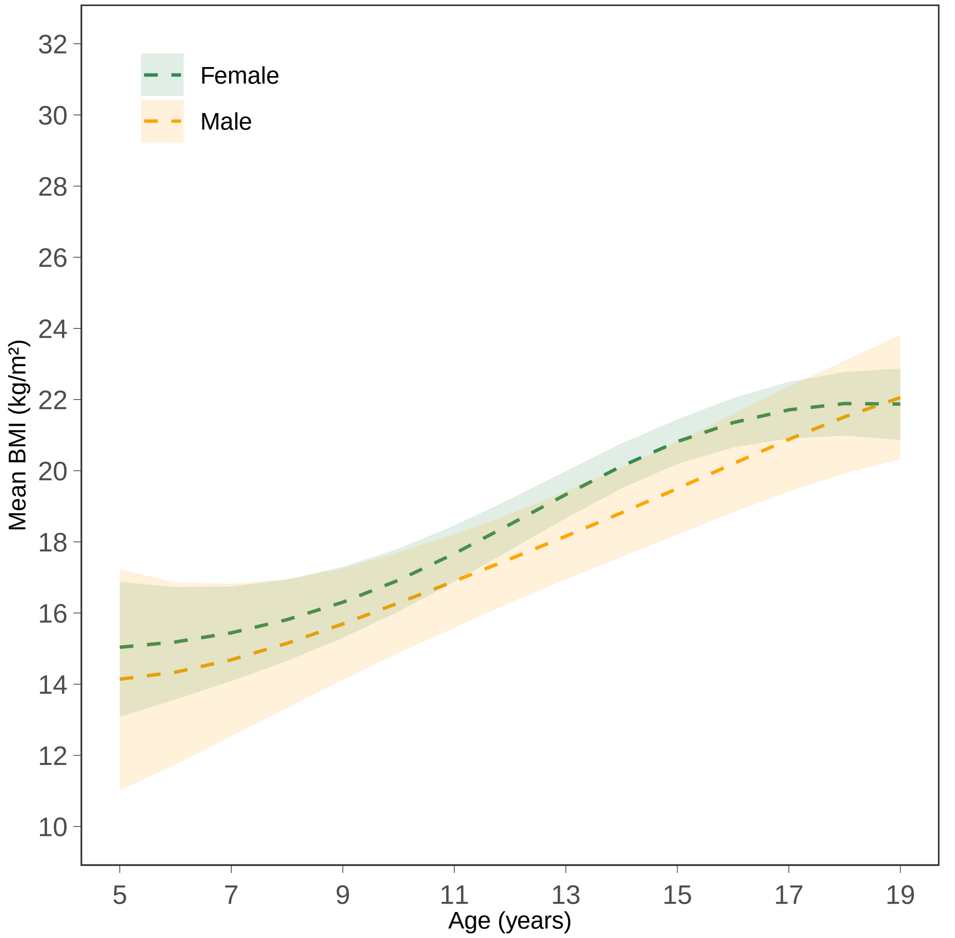

Croatia

Time trends in height of 19 year olds

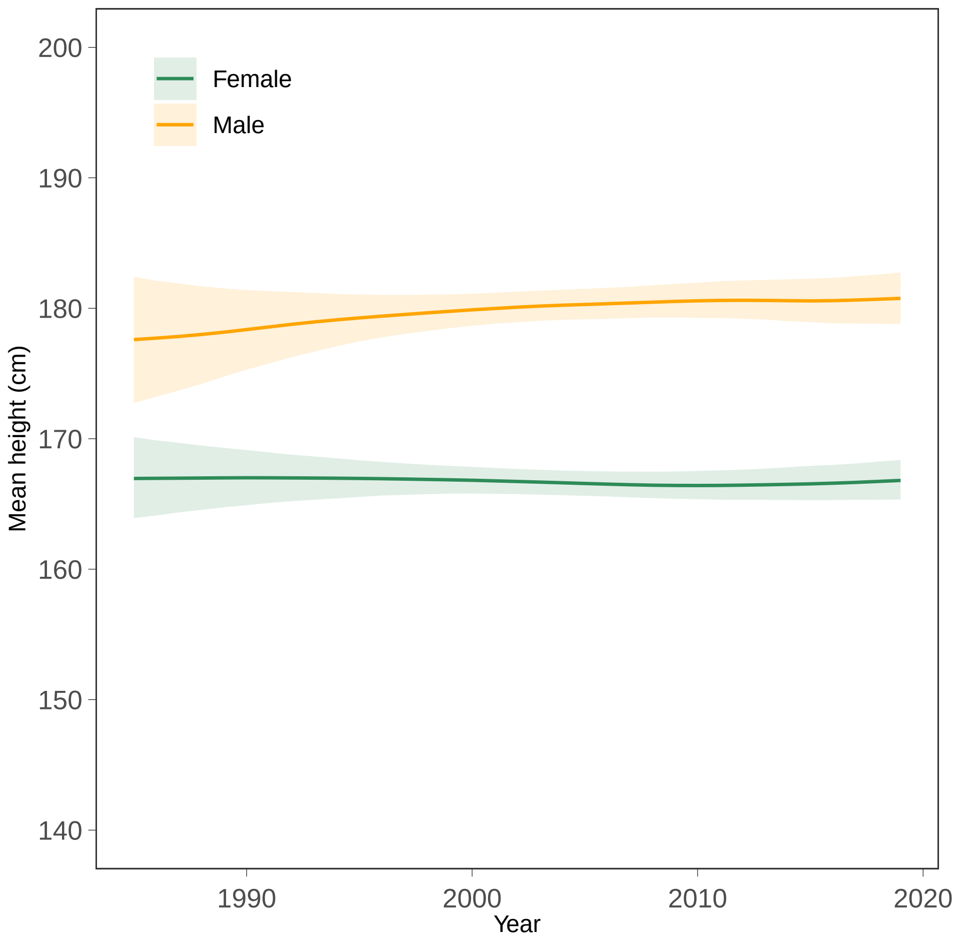

Time trends in BMI of 19 year olds

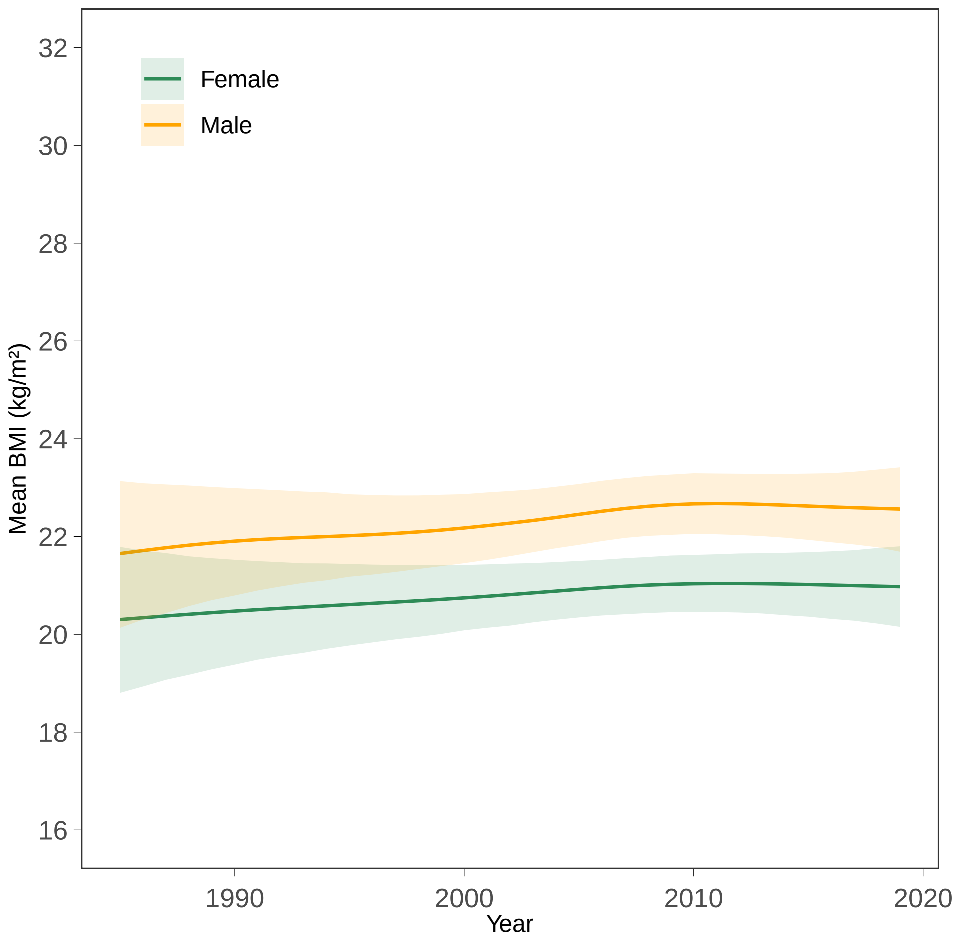

Height−for−age trajectories (2000 birth cohort)

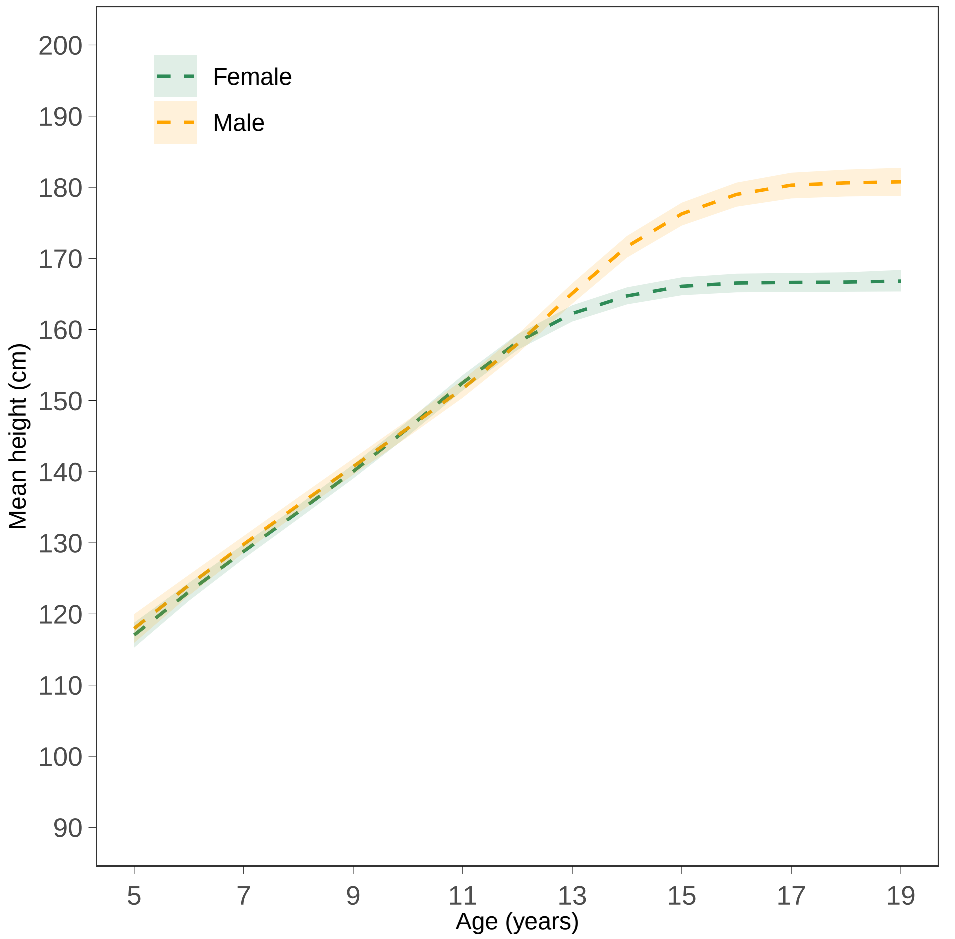

BMI−for−age trajectories (2000 birth cohort)

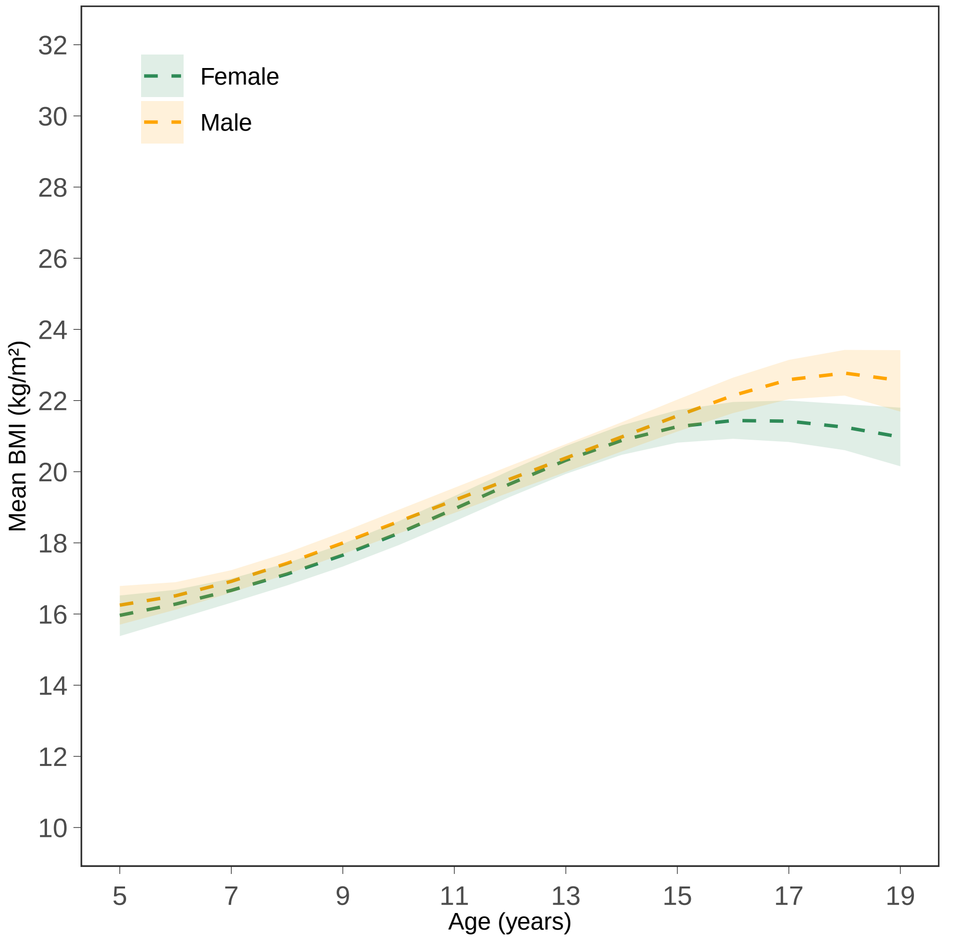

Cuba

Time trends in height of 19 year olds

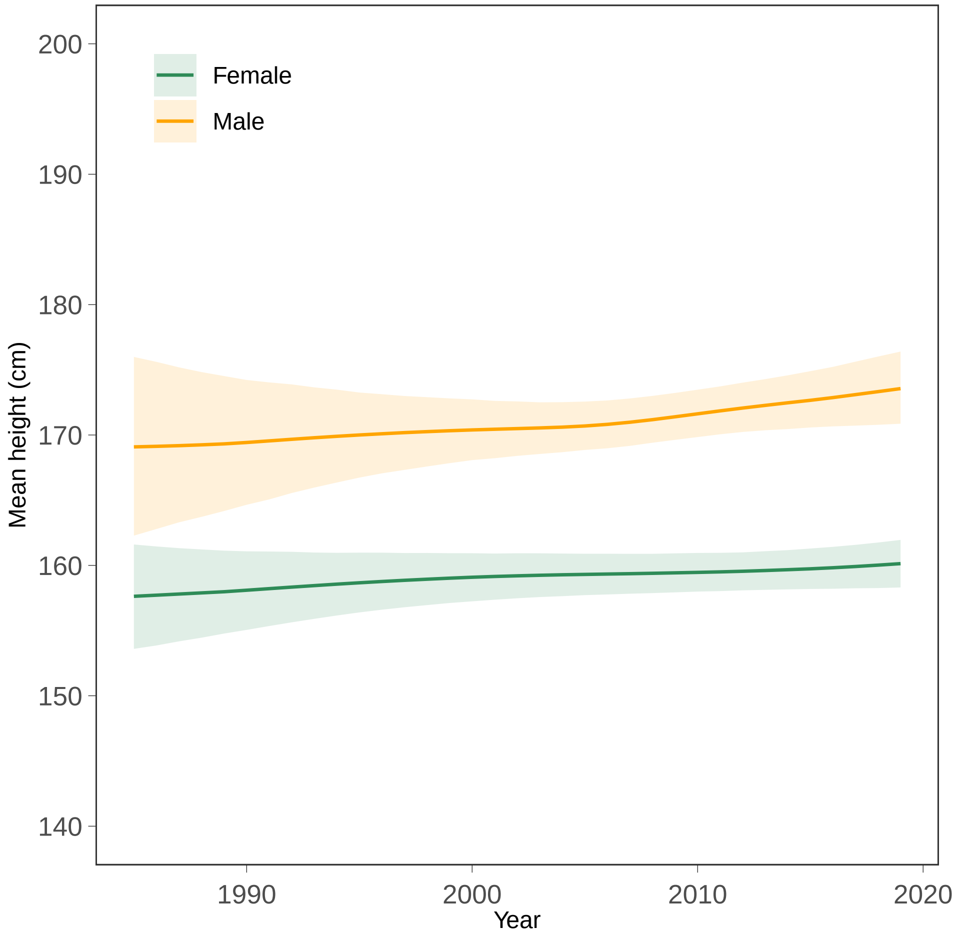

Time trends in BMI of 19 year olds

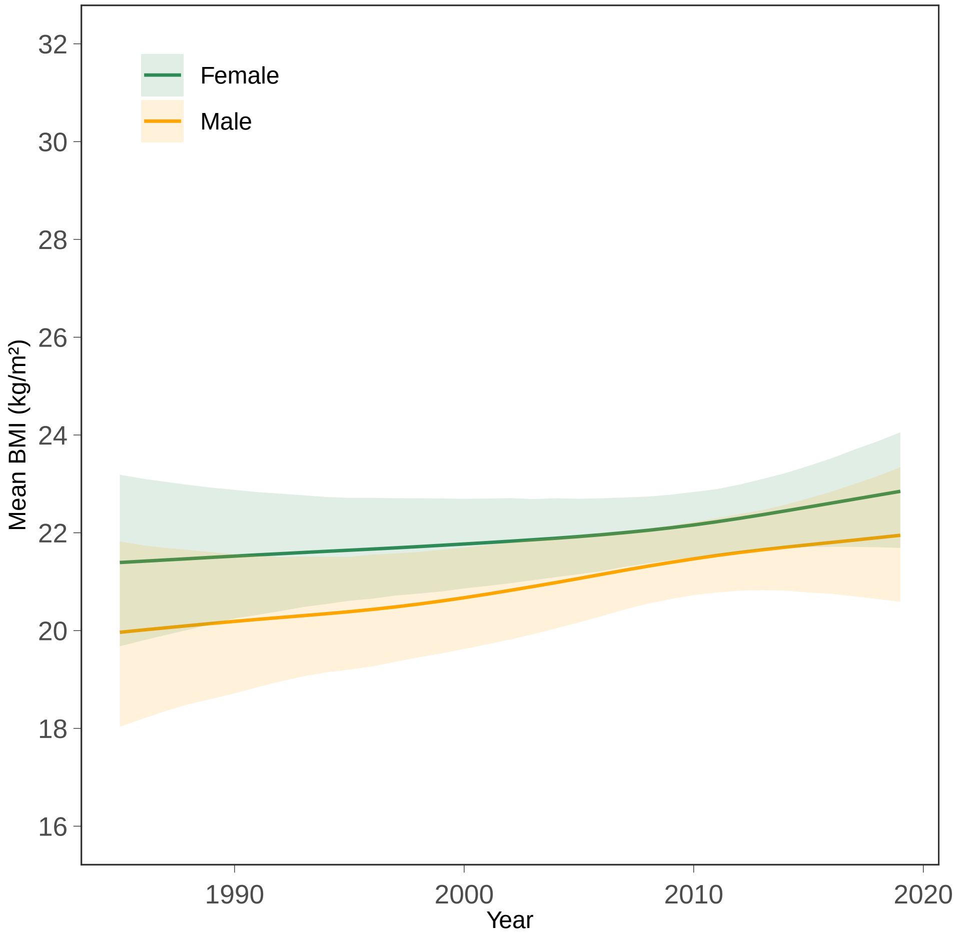

Height−for−age trajectories (2000 birth cohort)

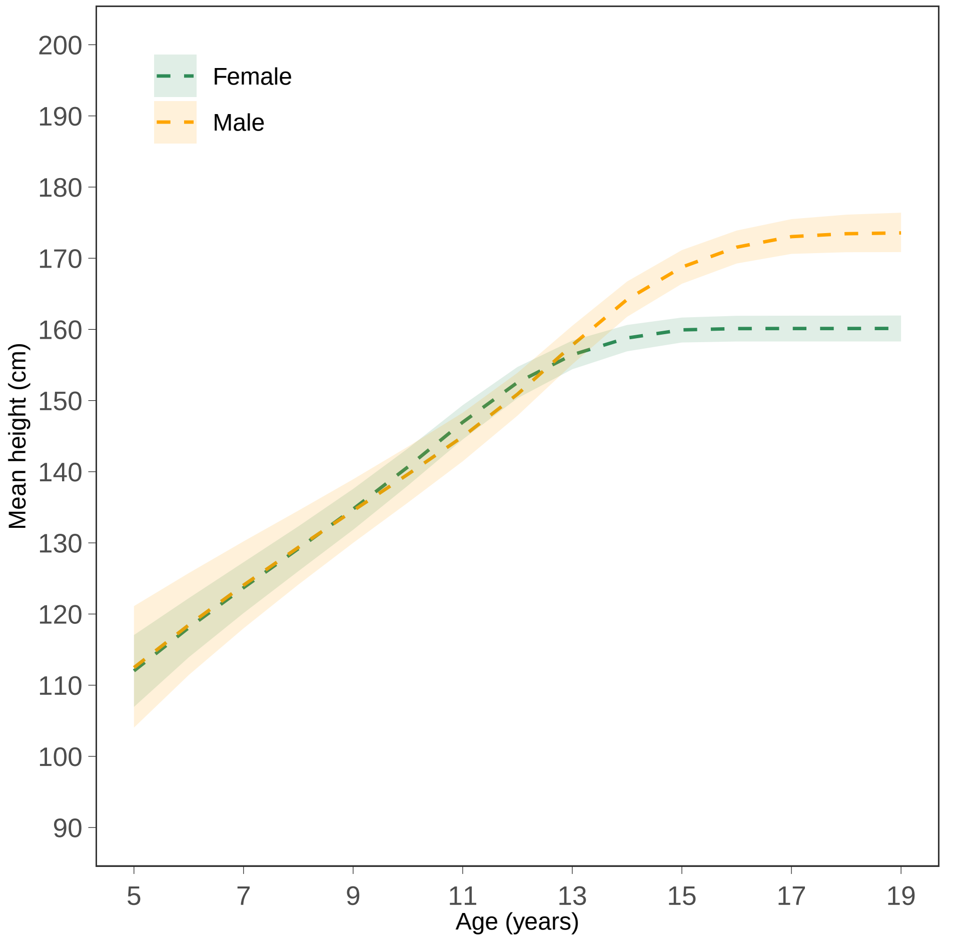

BMI−for−age trajectories (2000 birth cohort)

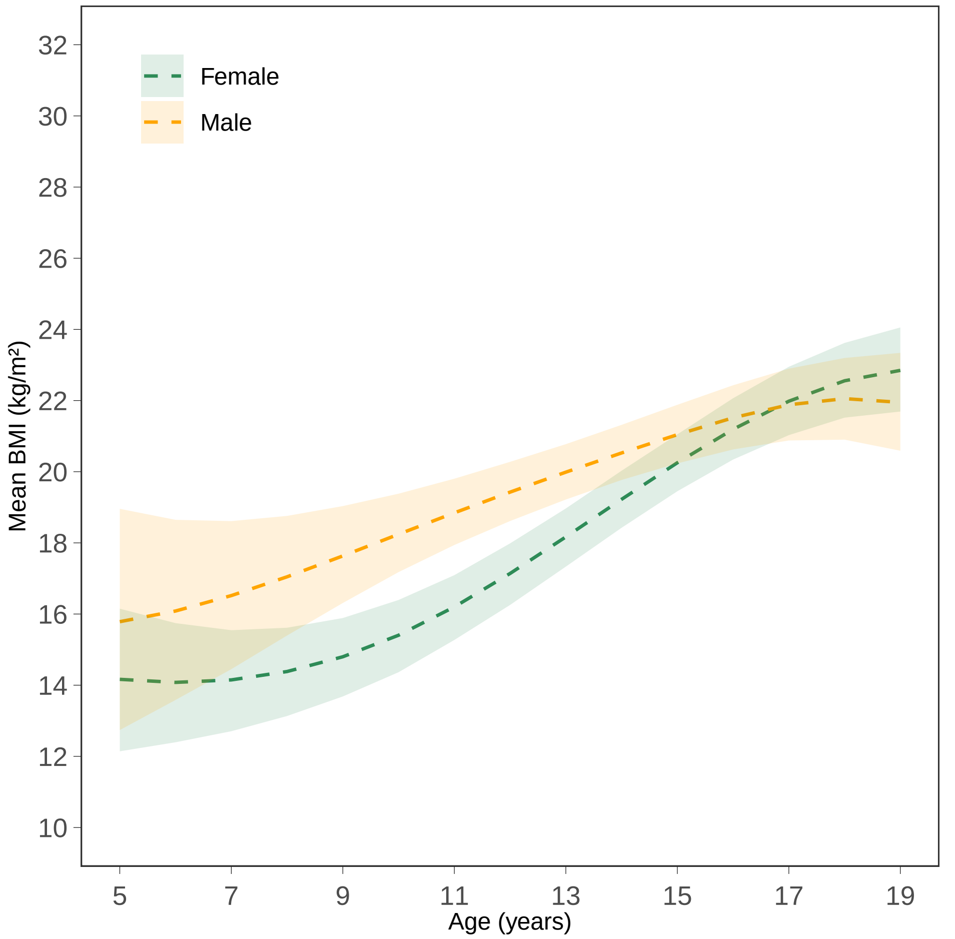

Cyprus

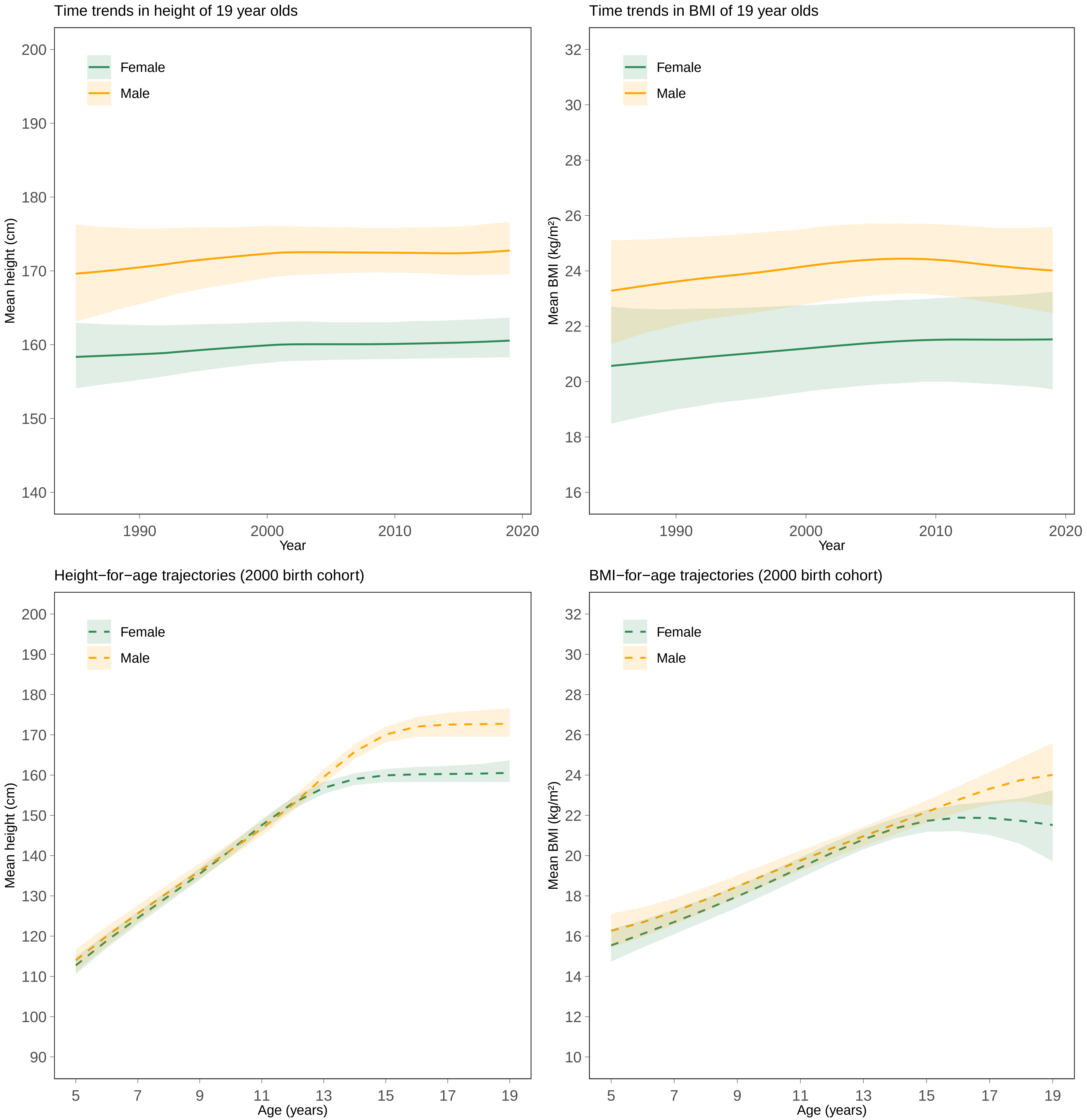

Time trends in height of 19 year olds
Female
Male
Mean height (cm)
200
190
180
170
160
150
140
1990
2000
2010
2020
Year
Time trends in BMI of 19 year olds
Female
Male
Mean BMI (kg/m²)
32
30
28
26
24
22
20
18
16
1990
2000
2010
2020
Year
Height−for−age trajectories (2000 birth cohort)
Female
Male
Mean height (cm)
200
190
180
170
160
150
140
130
120
110
100
90
5
7
9
11
13
15
17
19
Age (years)
BMI−for−age trajectories (2000 birth cohort)
Female
Male
Mean BMI (kg/m²)
32
30
28
26
24
22
20
18
16
14
12
10
5
7
9
11
13
15
17
19
Age (years)

Czech Republic

Time trends in height of 19 year olds

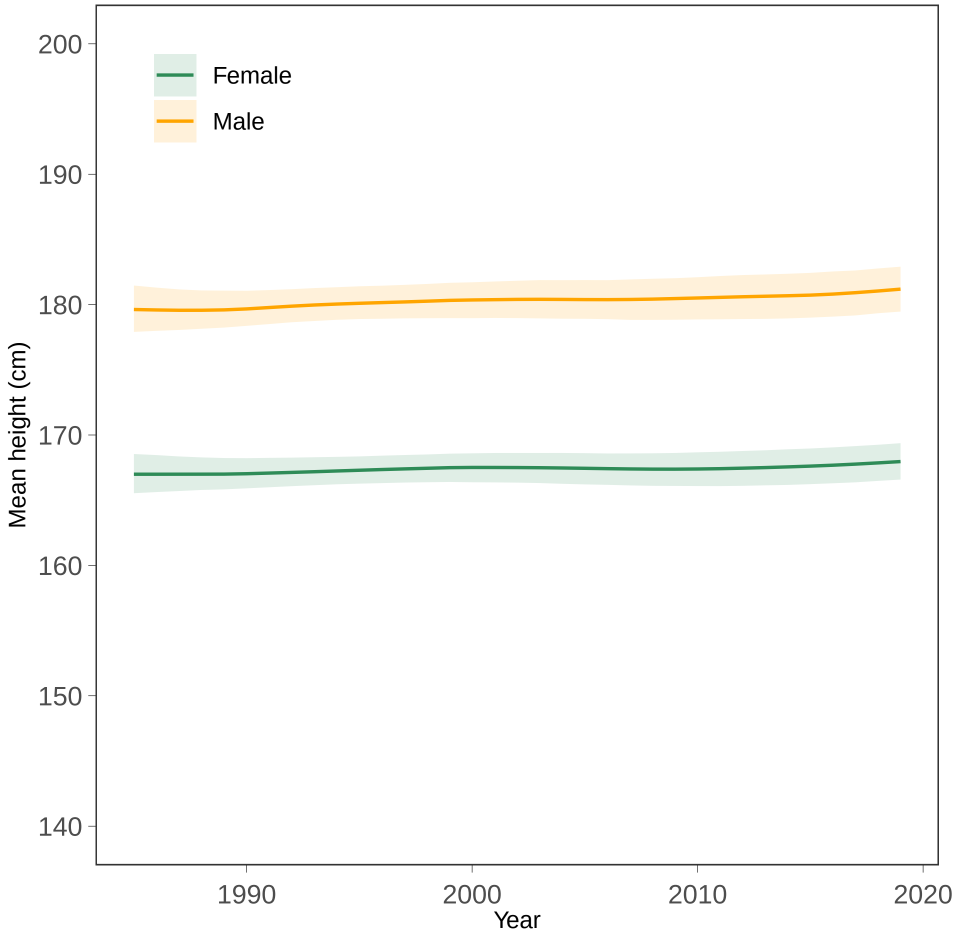

Time trends in BMI of 19 year olds

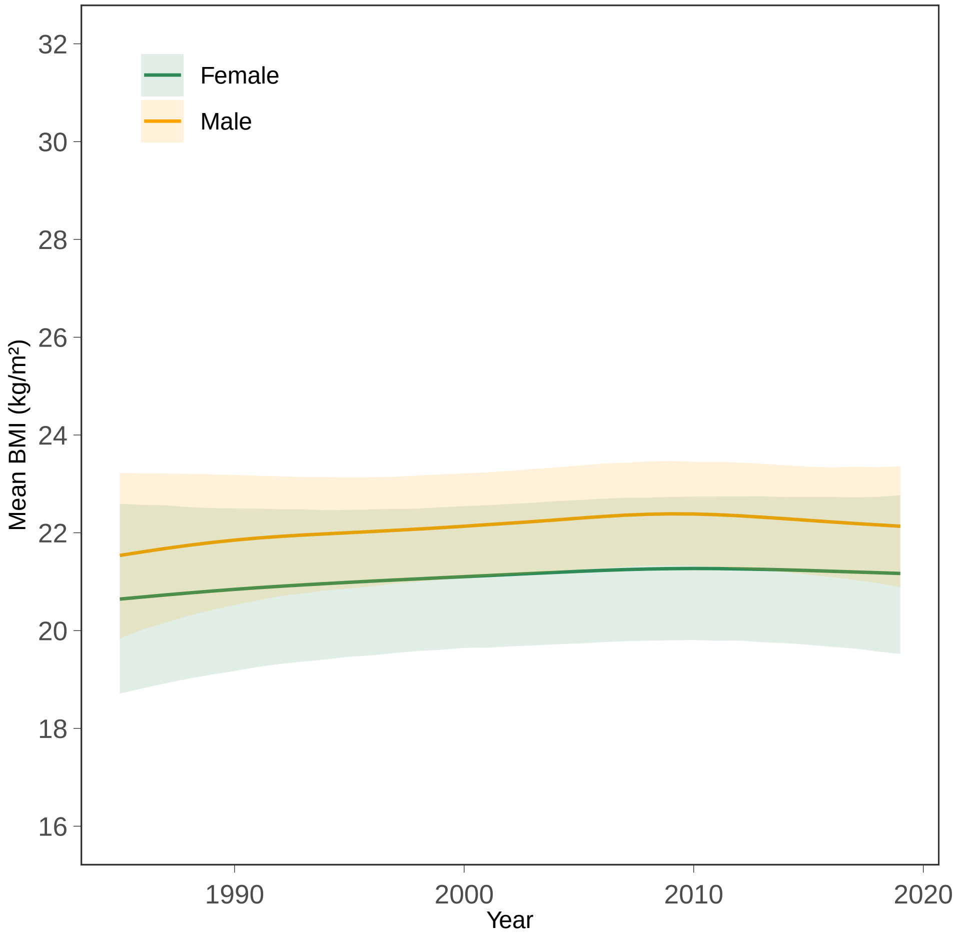

Height−for−age trajectories (2000 birth cohort)

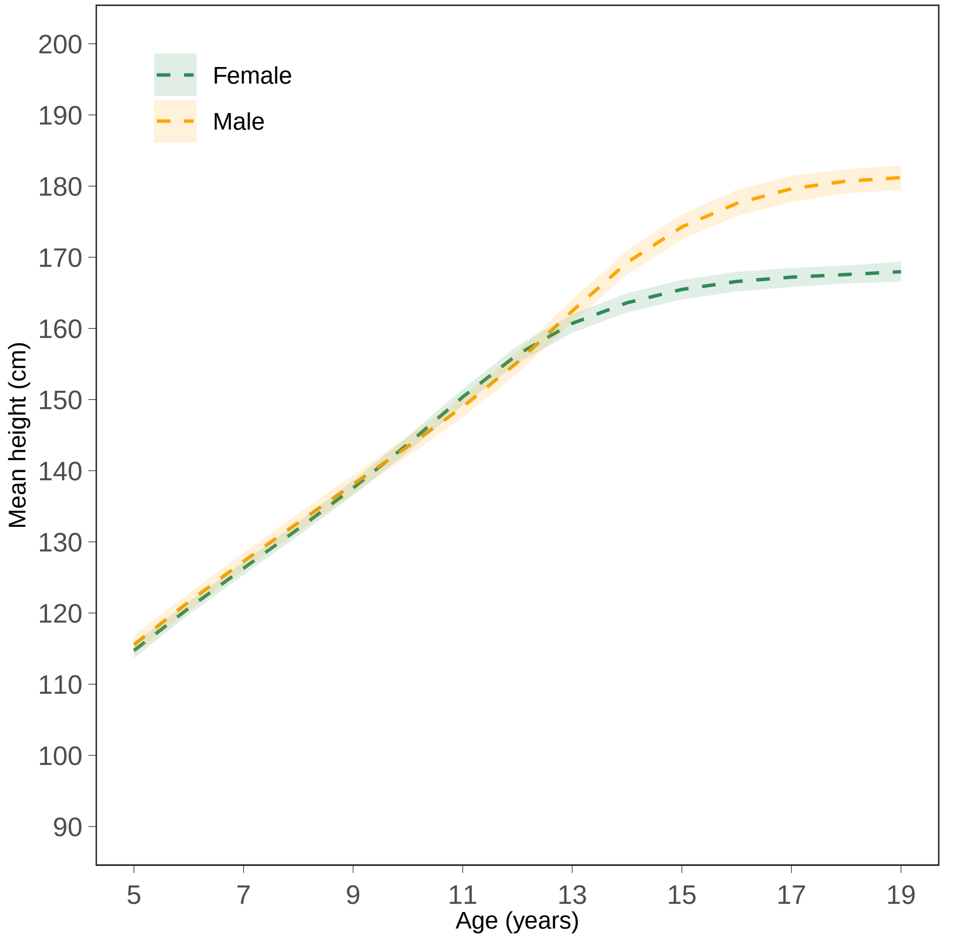

BMI−for−age trajectories (2000 birth cohort)

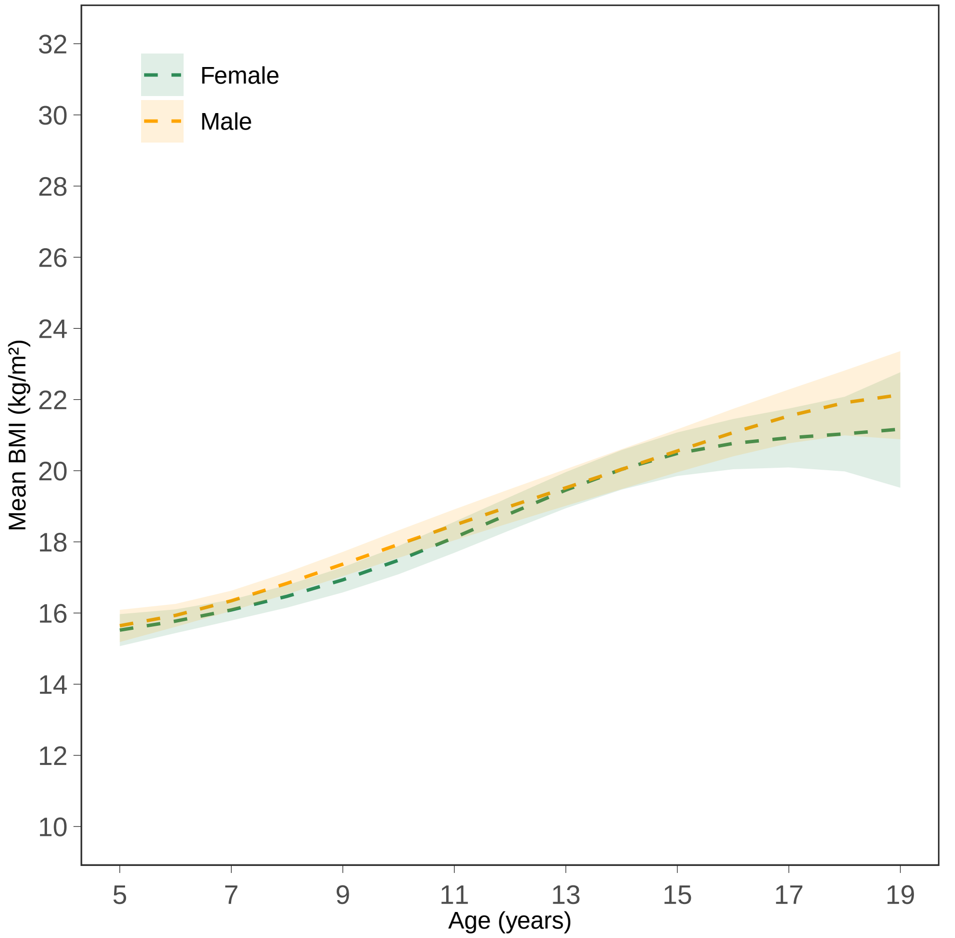

Denmark

Time trends in height of 19 year olds

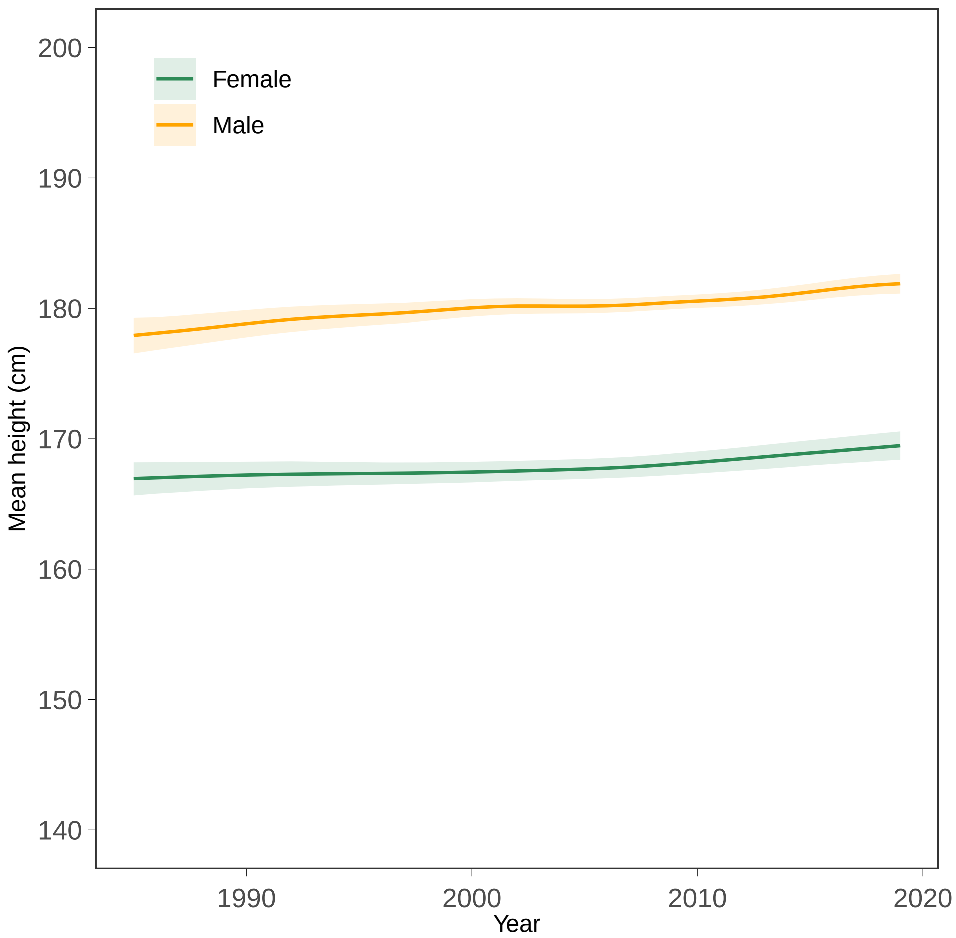

Time trends in BMI of 19 year olds

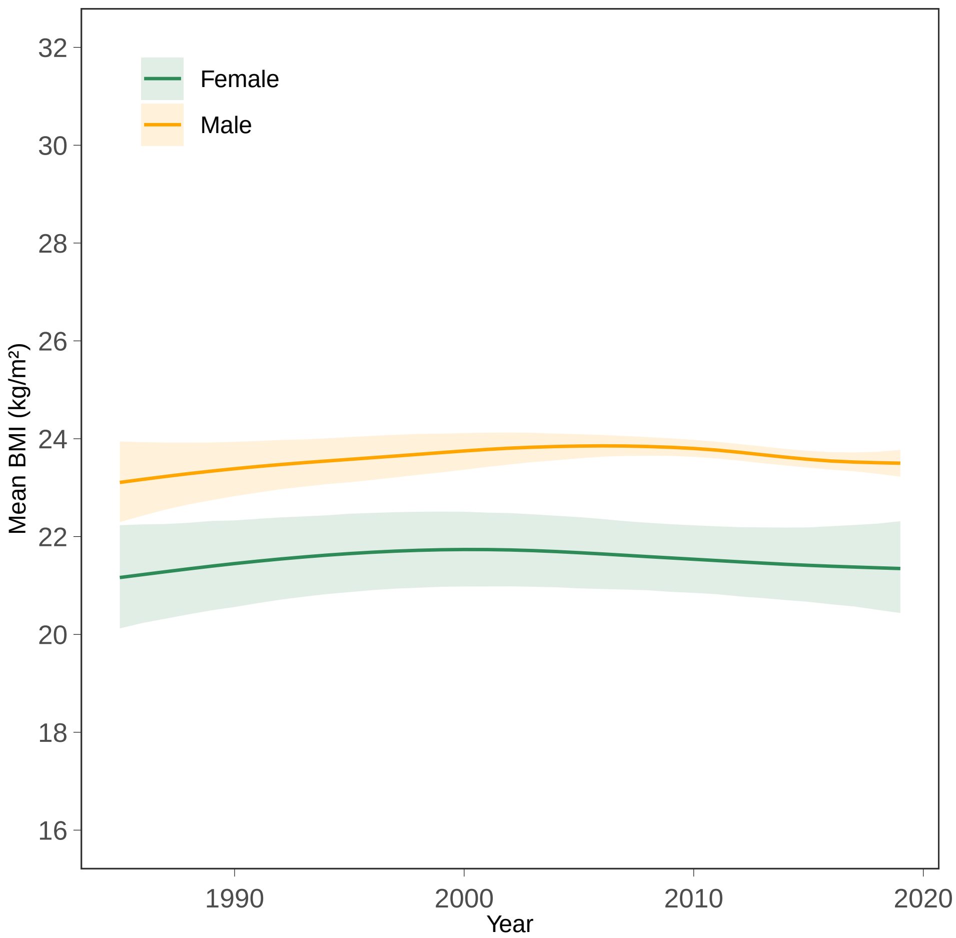

Height−for−age trajectories (2000 birth cohort)

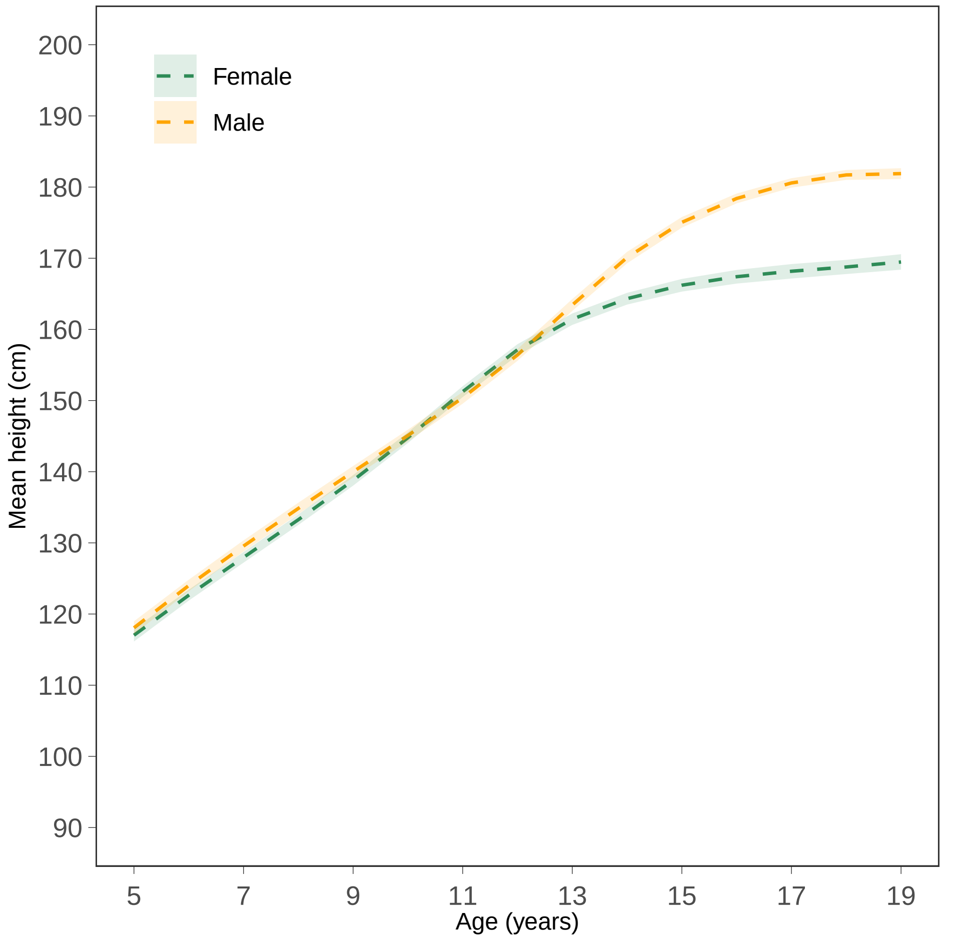

BMI−for−age trajectories (2000 birth cohort)

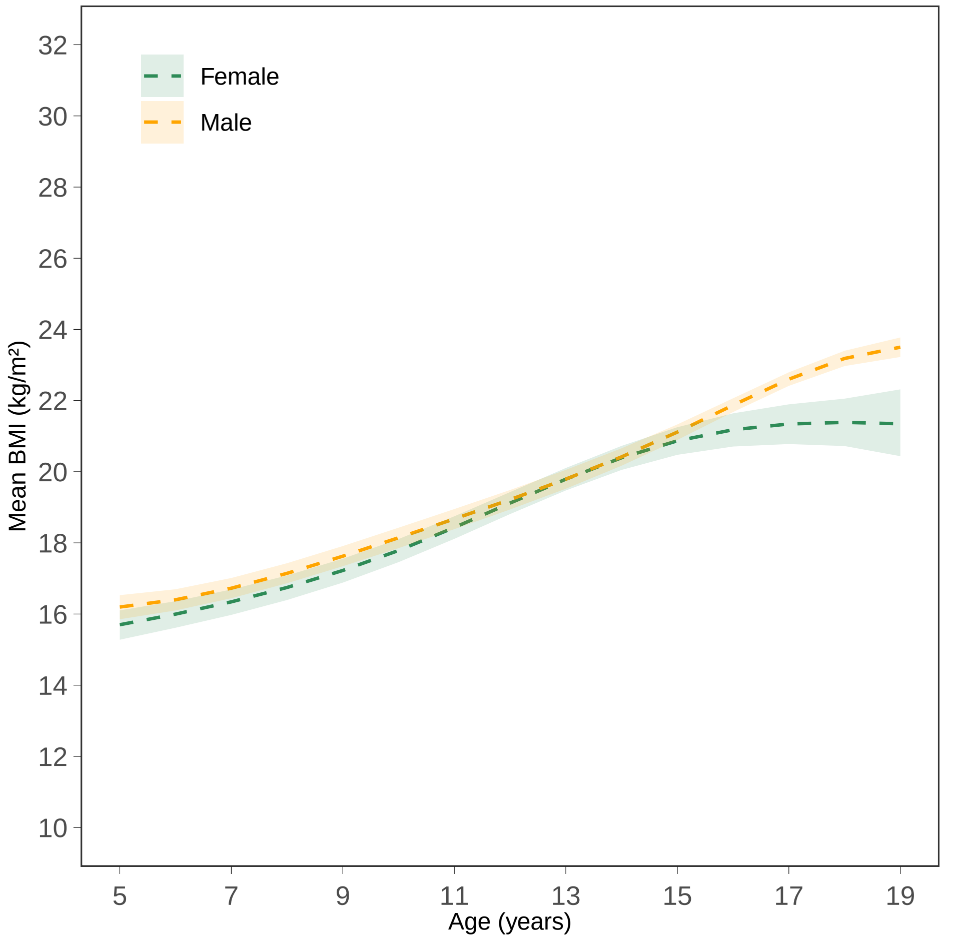

Djibouti

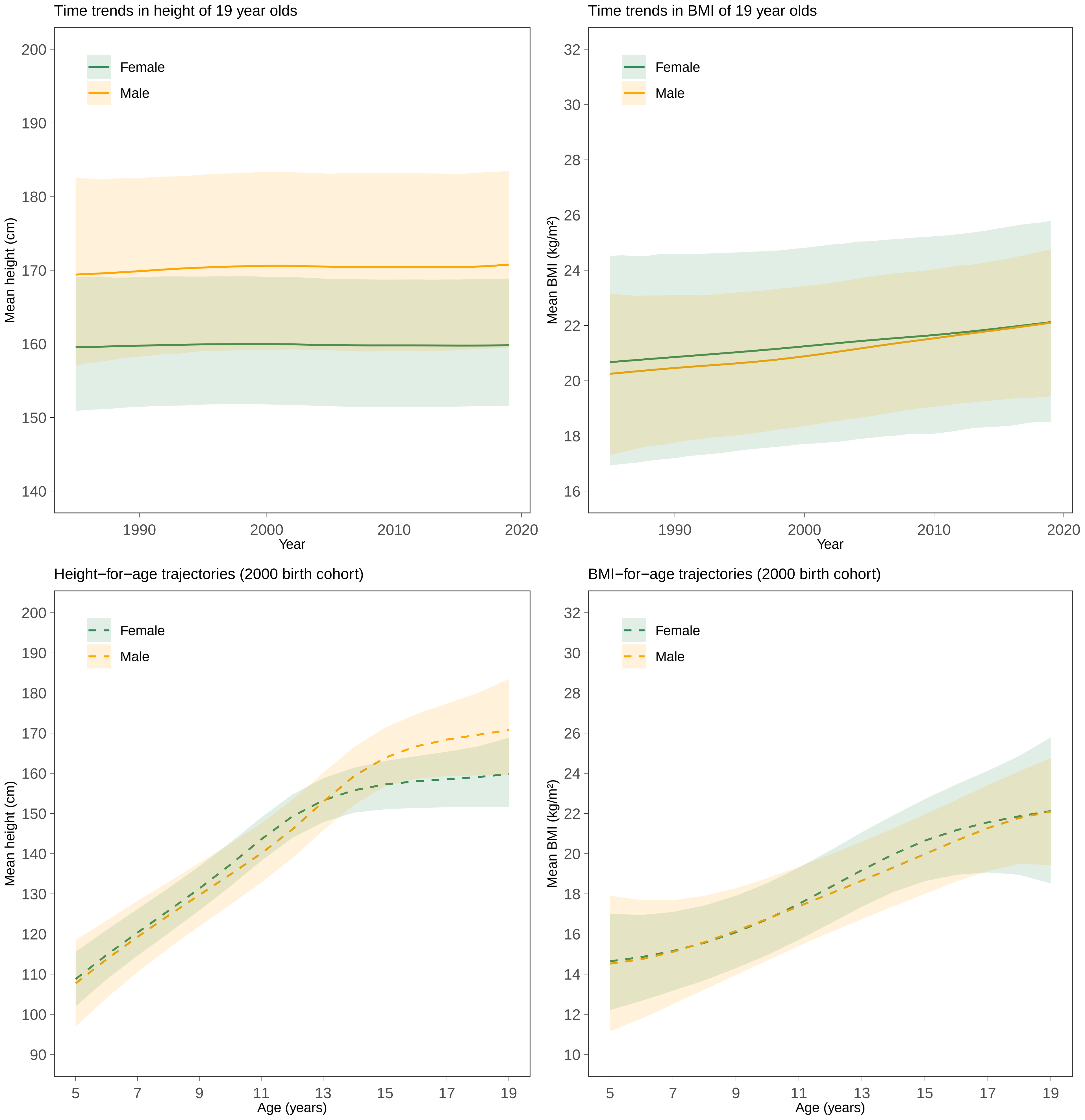

Time trends in height of 19 year olds
Female
Male
Mean height (cm)
Year
Time trends in BMI of 19 year olds
Mean BMI (kg/m²)
Height−for−age trajectories (2000 birth cohort)
Age (years)
BMI−for−age trajectories (2000 birth cohort)

Dominica

Time trends in height of 19 year olds

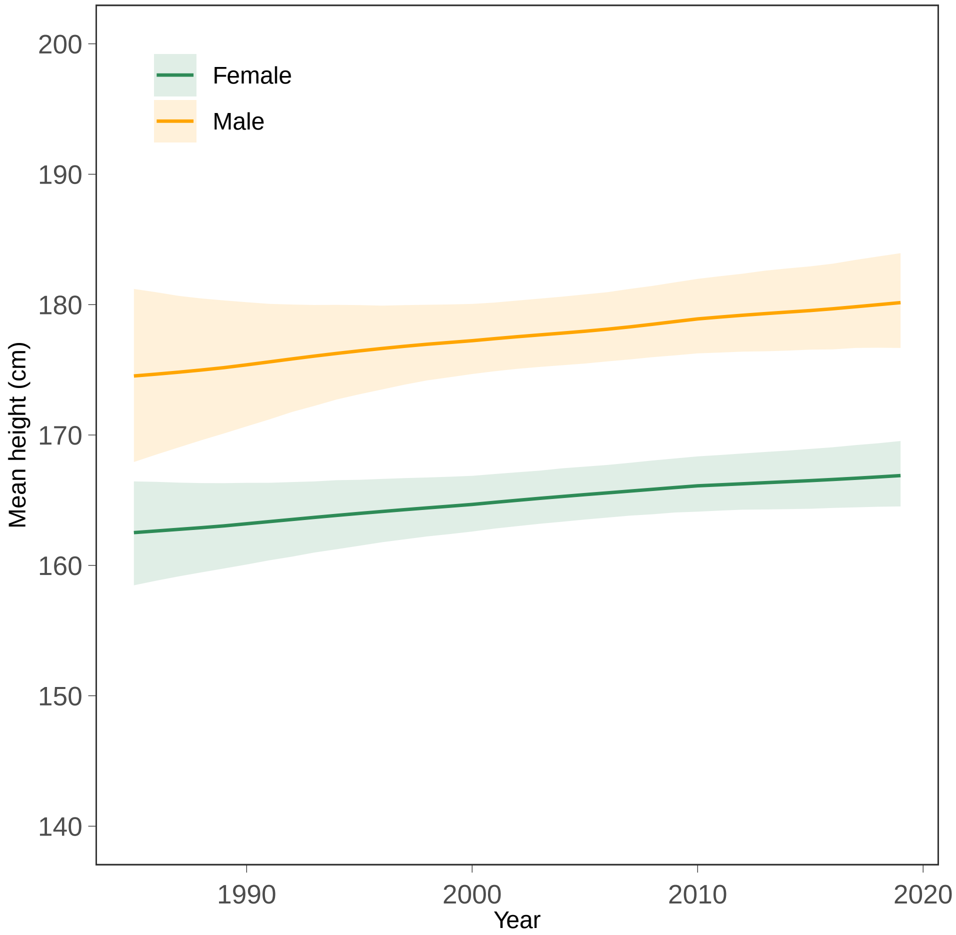

Time trends in BMI of 19 year olds

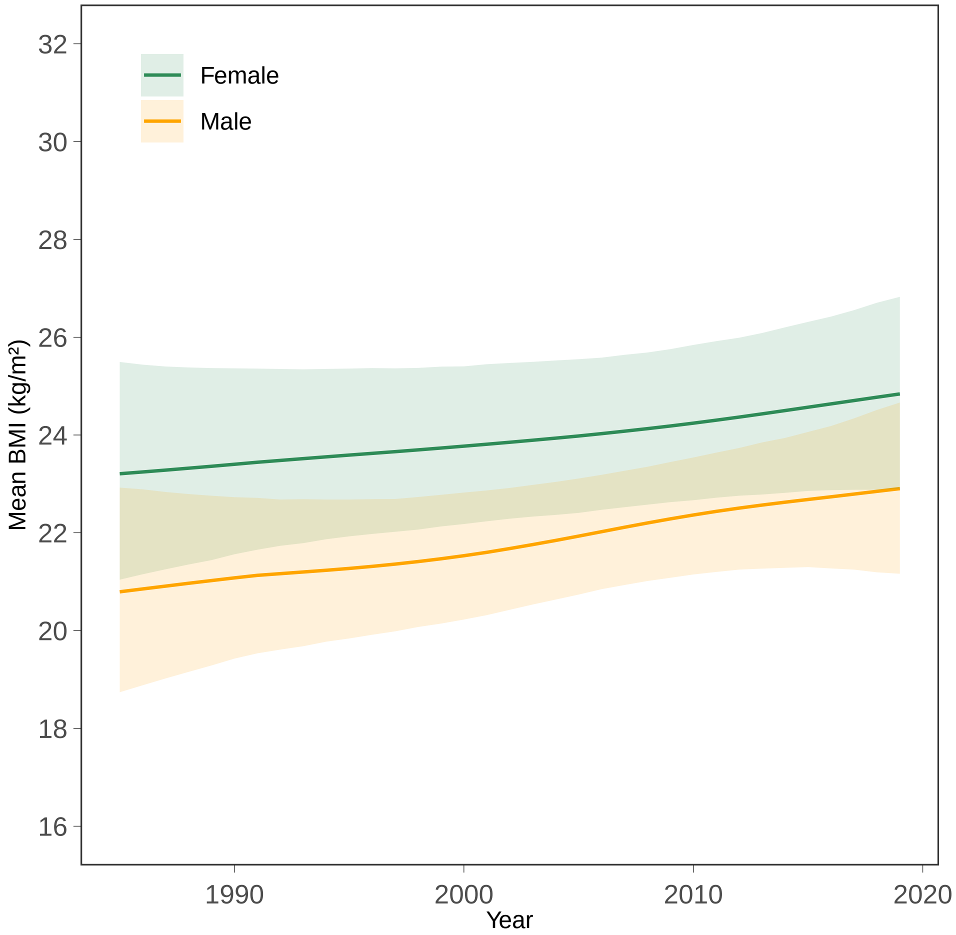

Height−for−age trajectories (2000 birth cohort)

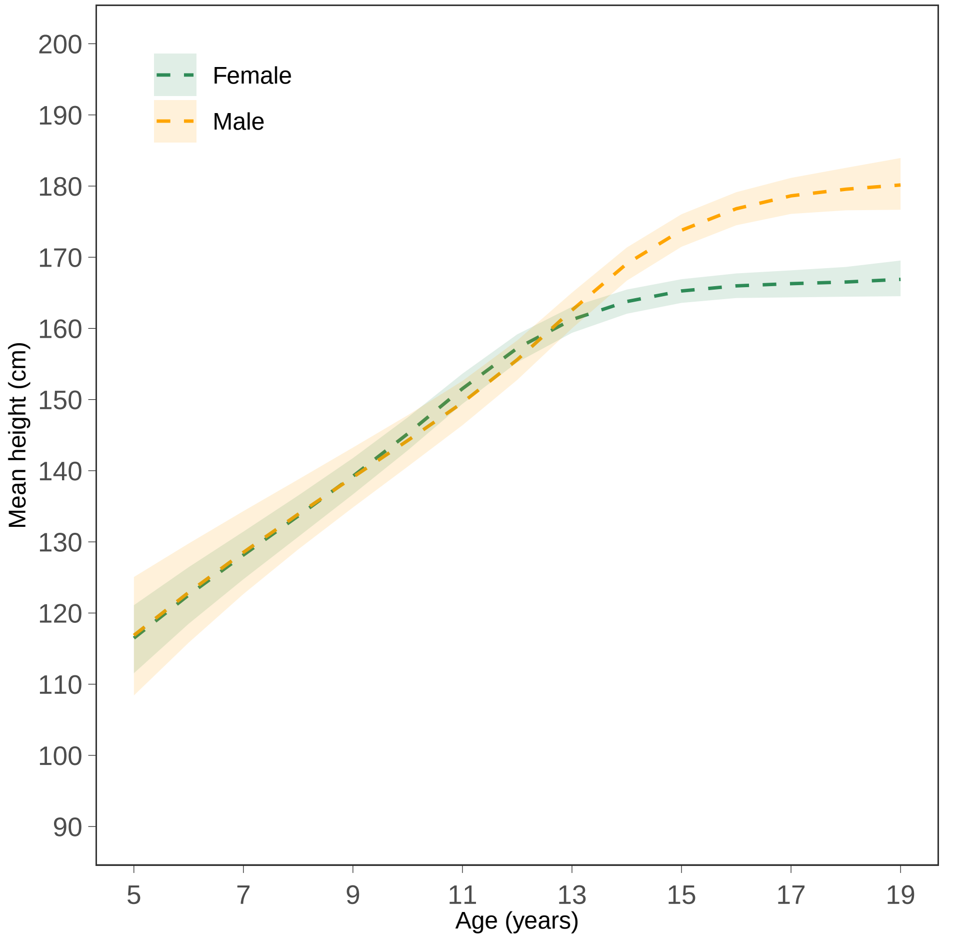

BMI−for−age trajectories (2000 birth cohort)

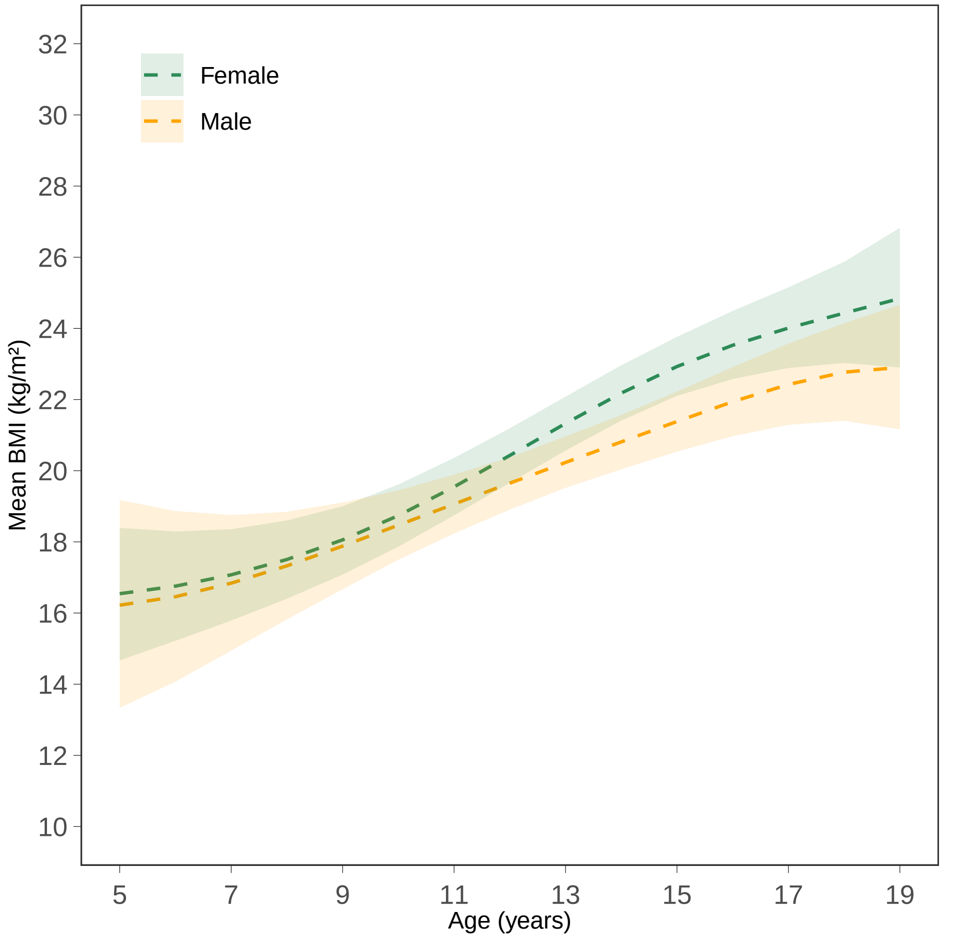

Dominican Republic

Time trends in height of 19 year olds

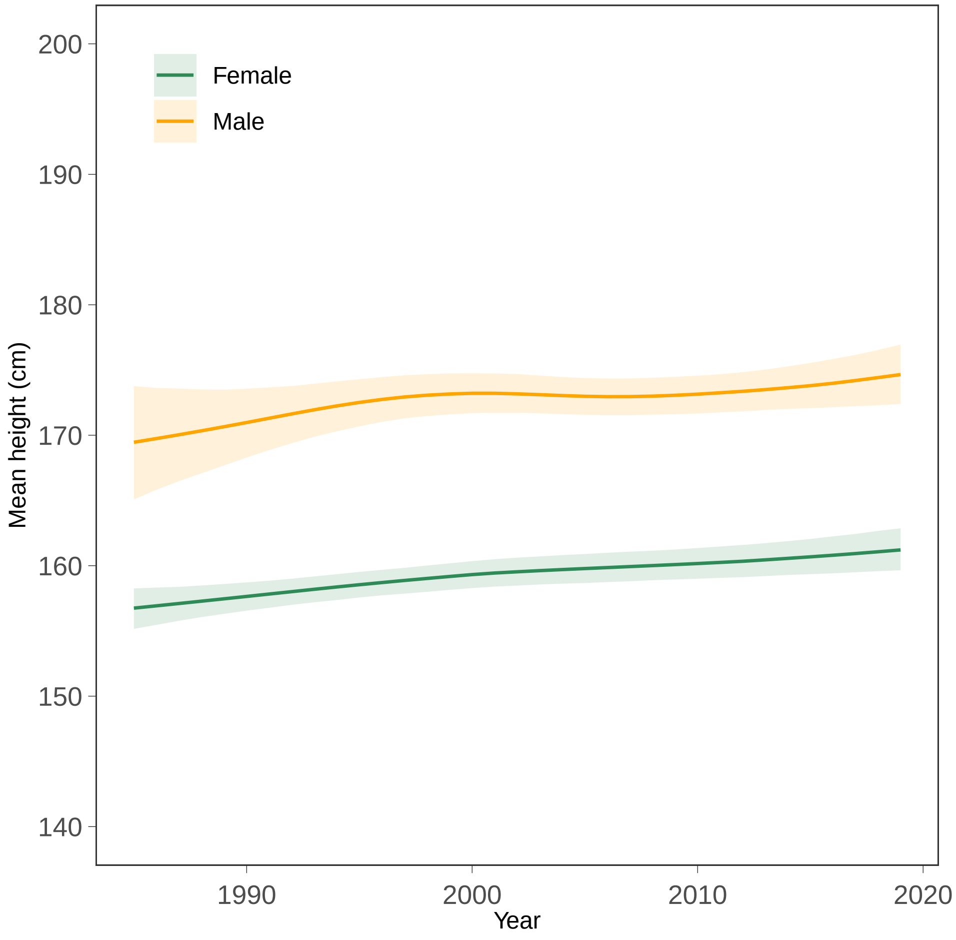

Time trends in BMI of 19 year olds

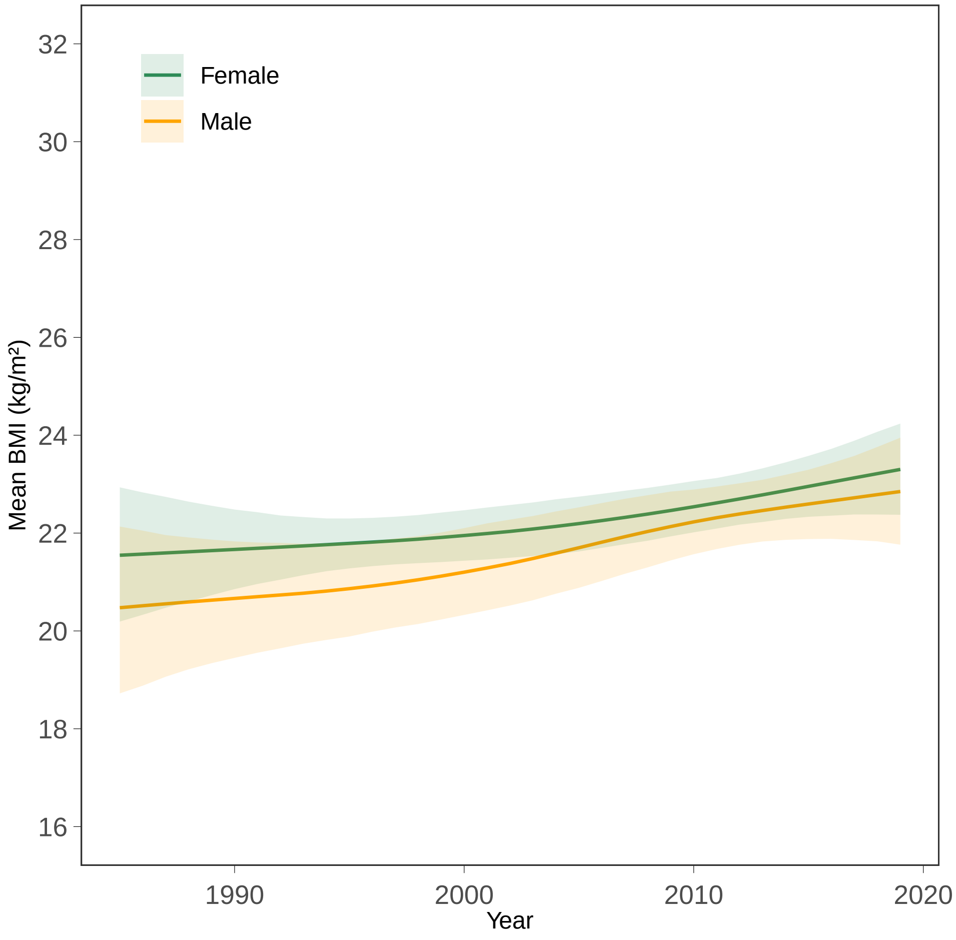

Height−for−age trajectories (2000 birth cohort)

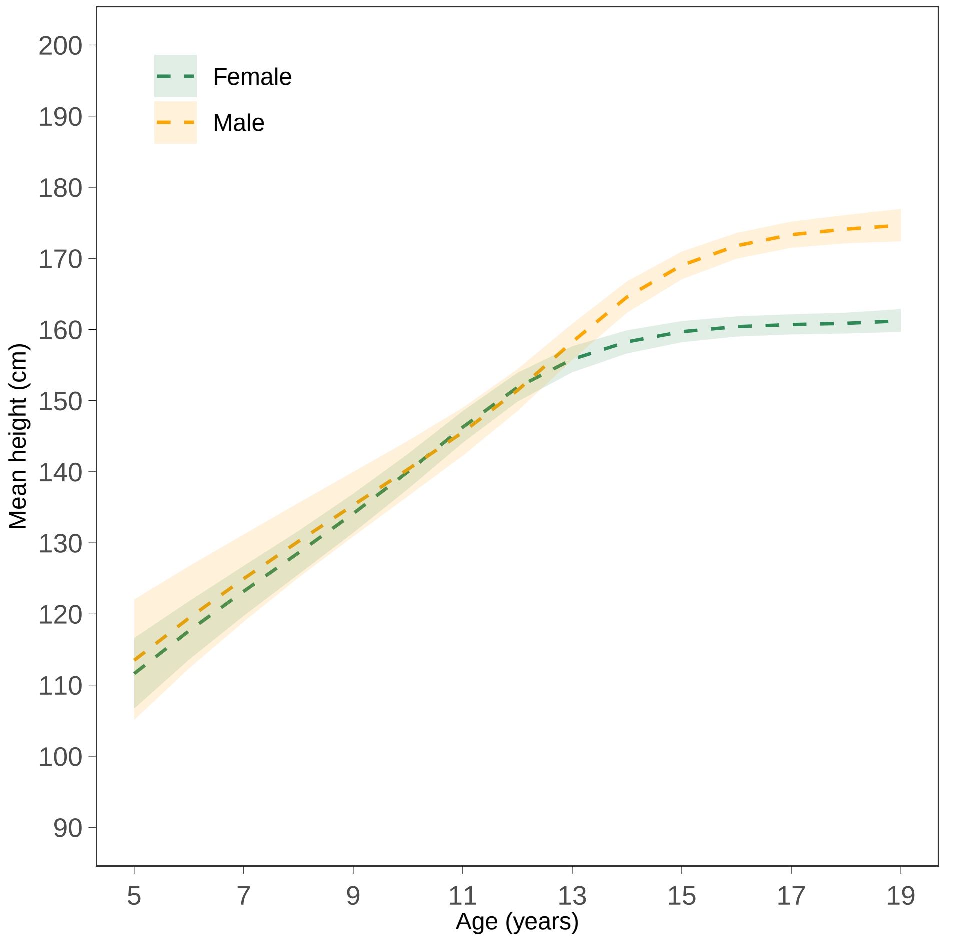

BMI−for−age trajectories (2000 birth cohort)

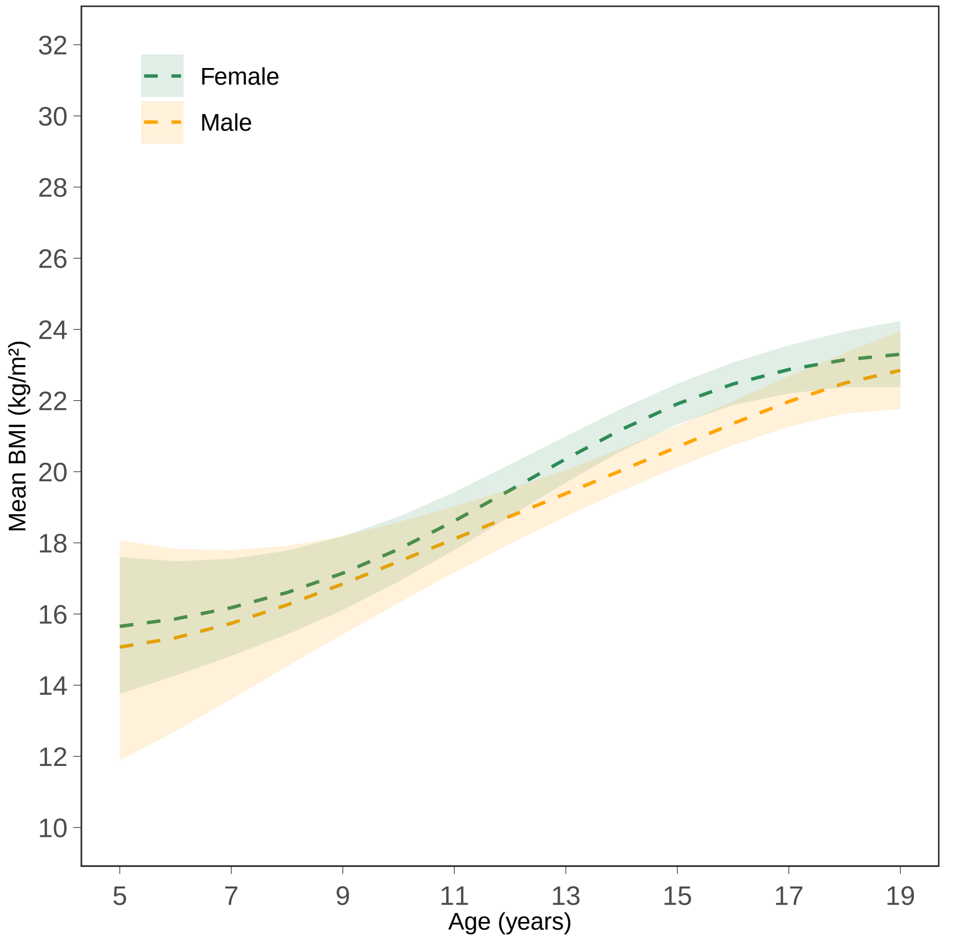

DR Congo

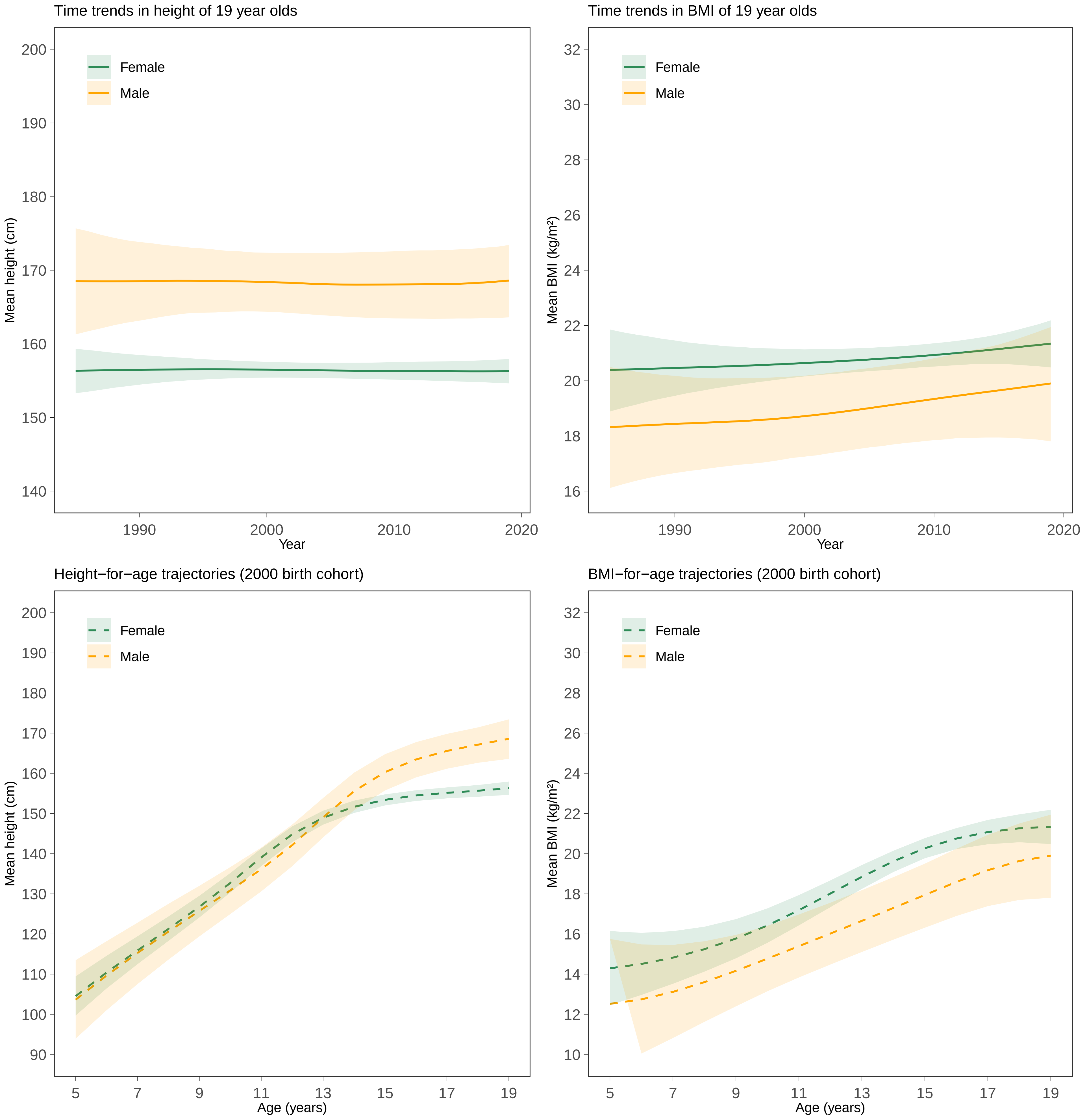

Time trends in height of 19 year olds
Female
Male
Mean height (cm)
Year
Time trends in BMI of 19 year olds
Mean BMI (kg/m²)
Height−for−age trajectories (2000 birth cohort)
Age (years)
BMI−for−age trajectories (2000 birth cohort)

Ecuador

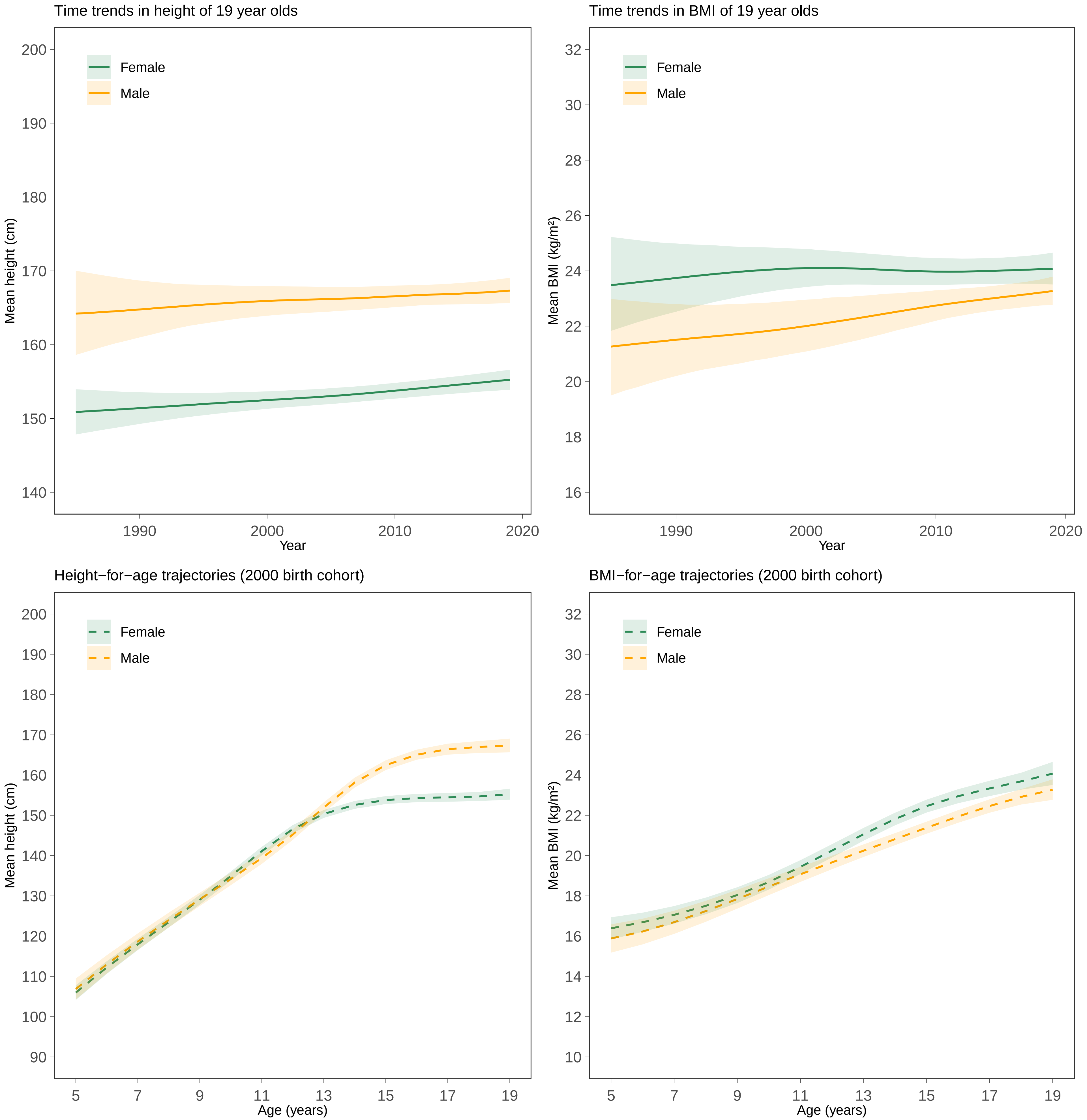

Time trends in height of 19 year olds
Female
Male
Mean height (cm)
200
190
180
170
160
150
140
1990
2000
2010
2020
Year
Time trends in BMI of 19 year olds
Female
Male
Mean BMI (kg/m²)
32
30
28
26
24
22
20
18
16
1990
2000
2010
2020
Year
Height−for−age trajectories (2000 birth cohort)
Female
Male
Mean height (cm)
200
190
180
170
160
150
140
130
120
110
100
90
5
7
9
11
13
15
17
19
Age (years)
BMI−for−age trajectories (2000 birth cohort)
Female
Male
Mean BMI (kg/m²)
32
30
28
26
24
22
20
18
16
14
12
10
5
7
9
11
13
15
17
19
Age (years)

Egypt

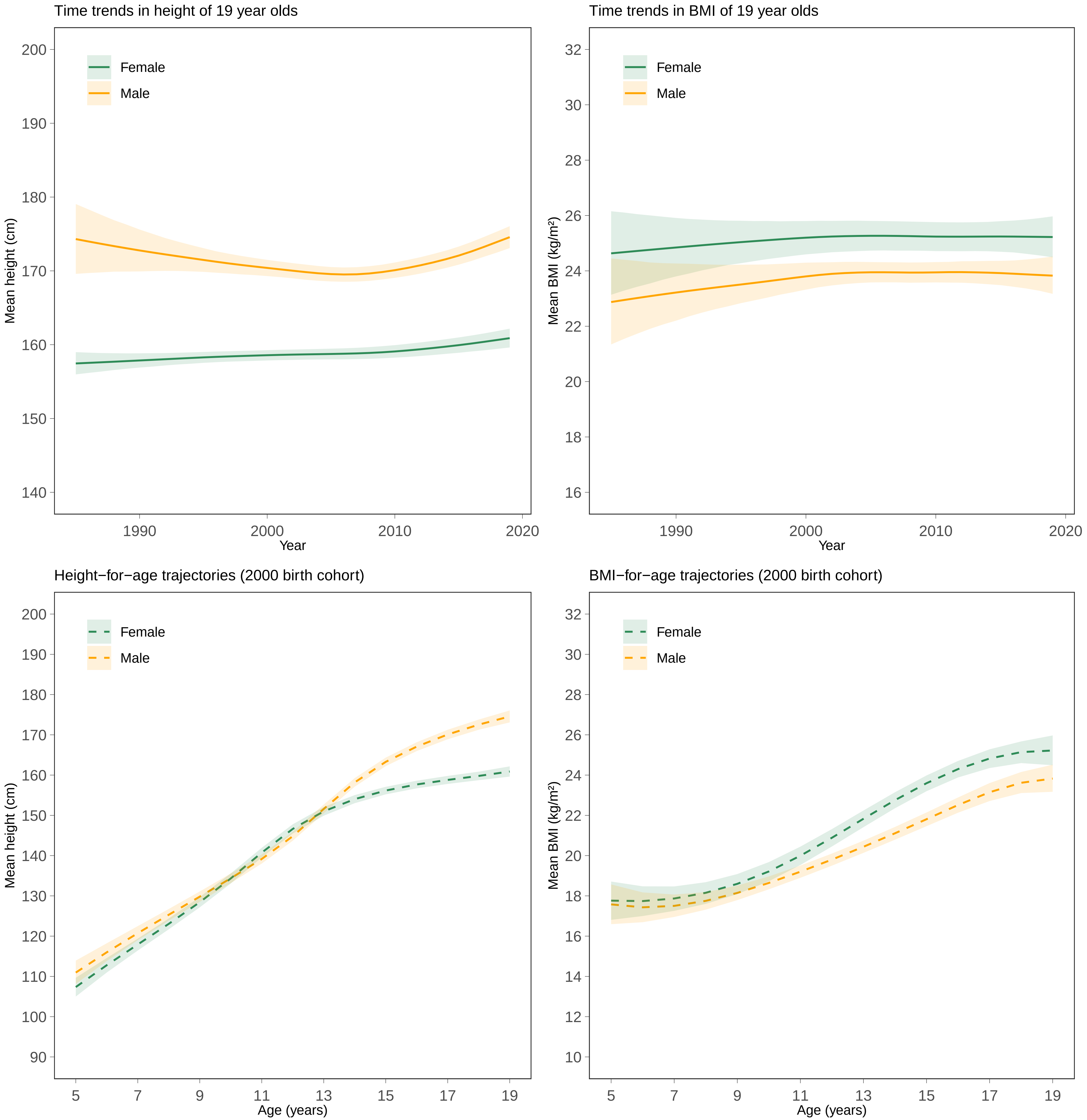

Time trends in height of 19 year olds
Female
Male
Mean height (cm)
Year
Time trends in BMI of 19 year olds
Mean BMI (kg/m²)
Height−for−age trajectories (2000 birth cohort)
Age (years)
BMI−for−age trajectories (2000 birth cohort)

El Salvador

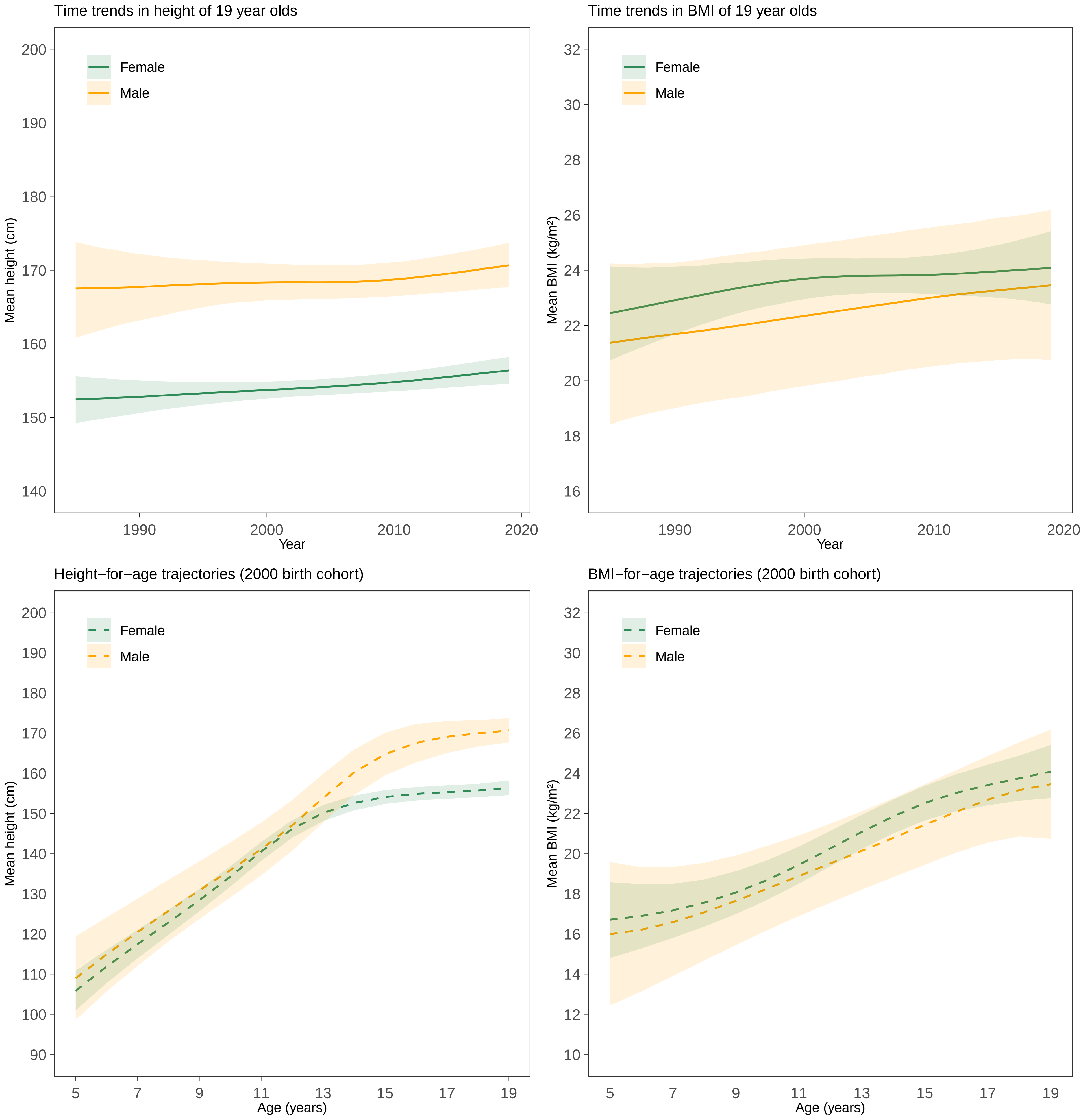

Time trends in height of 19 year olds
Female
Male
Mean height (cm)
Year
Time trends in BMI of 19 year olds
Mean BMI (kg/m²)
Height−for−age trajectories (2000 birth cohort)
Age (years)
BMI−for−age trajectories (2000 birth cohort)

Equatorial Guinea

Time trends in height of 19 year olds

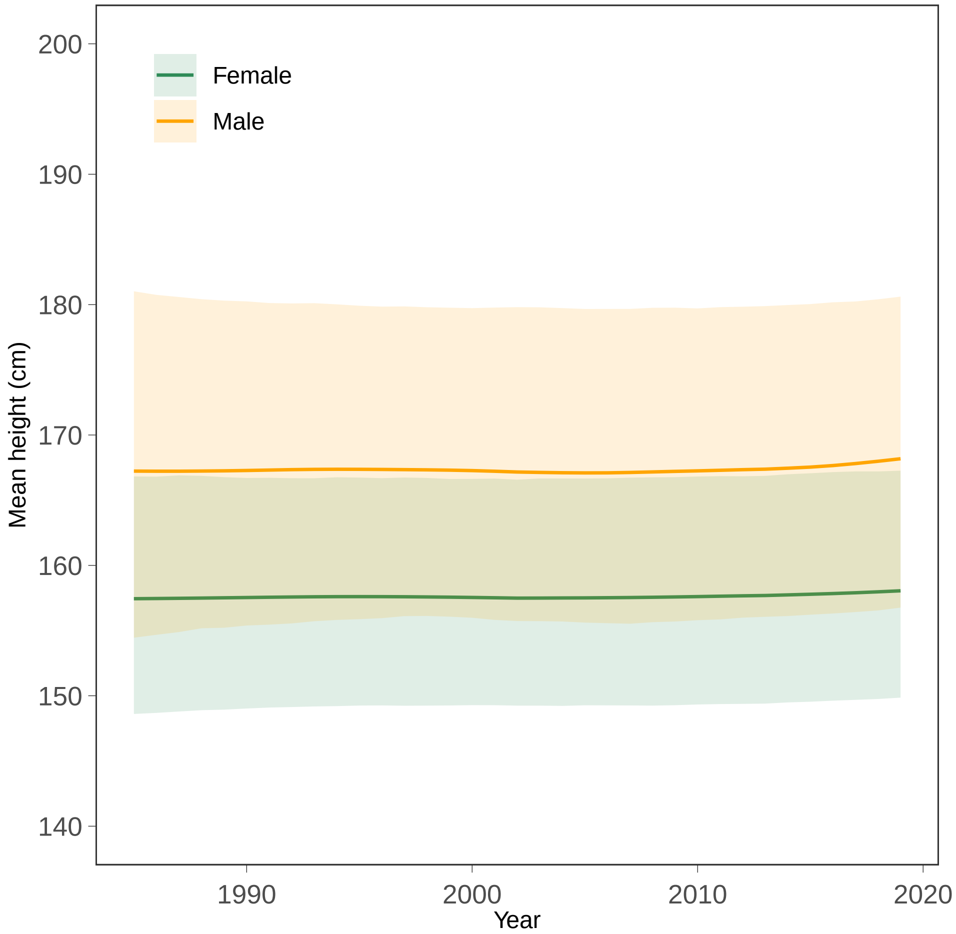

Time trends in BMI of 19 year olds

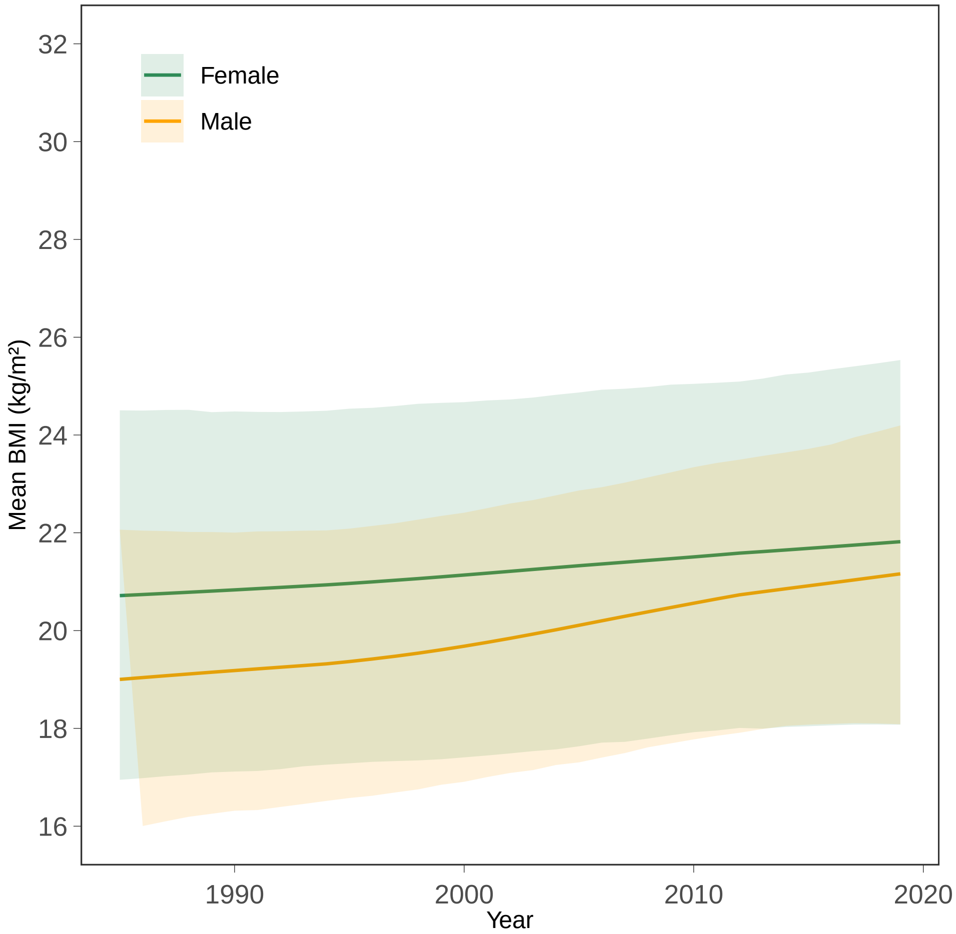

Height−for−age trajectories (2000 birth cohort)

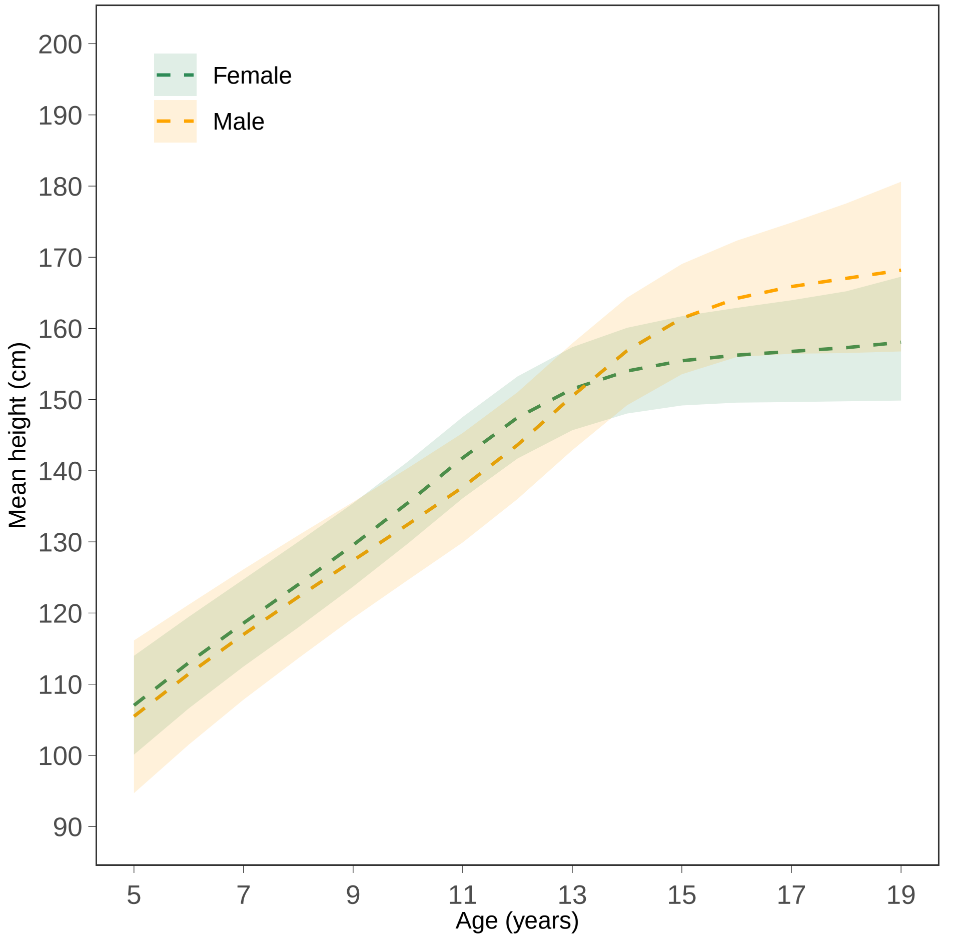

BMI−for−age trajectories (2000 birth cohort)

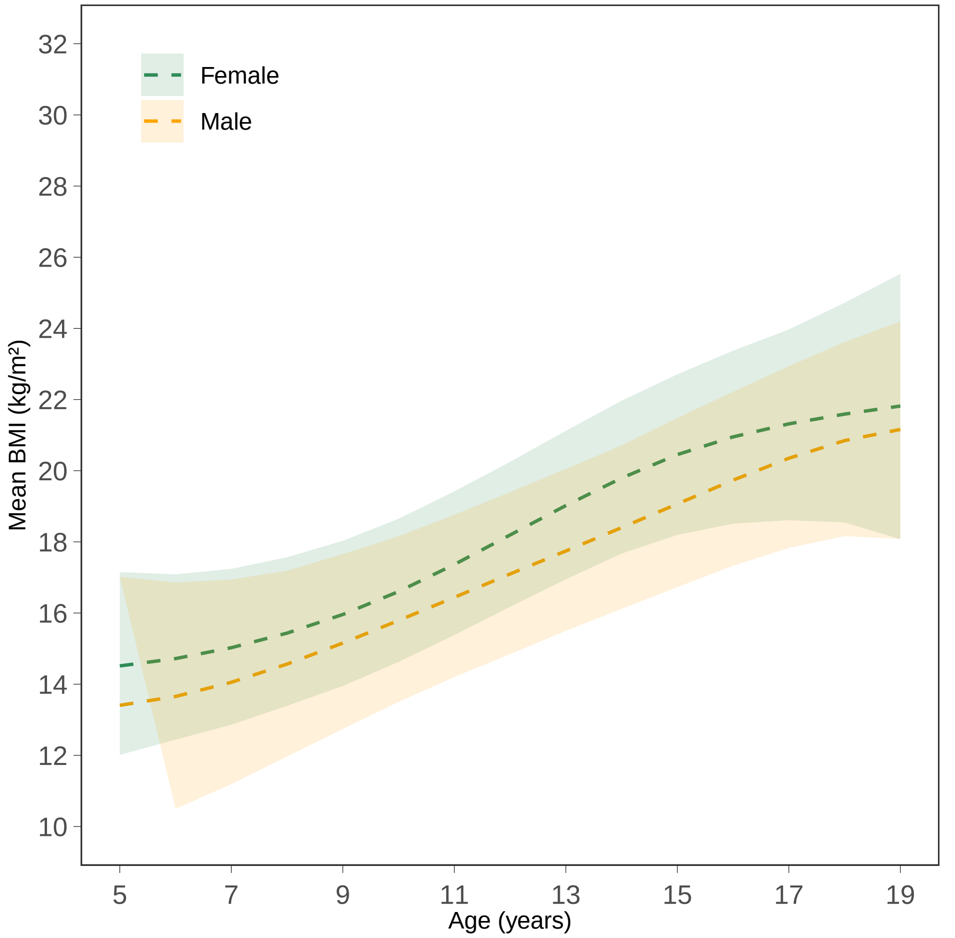

Eritrea

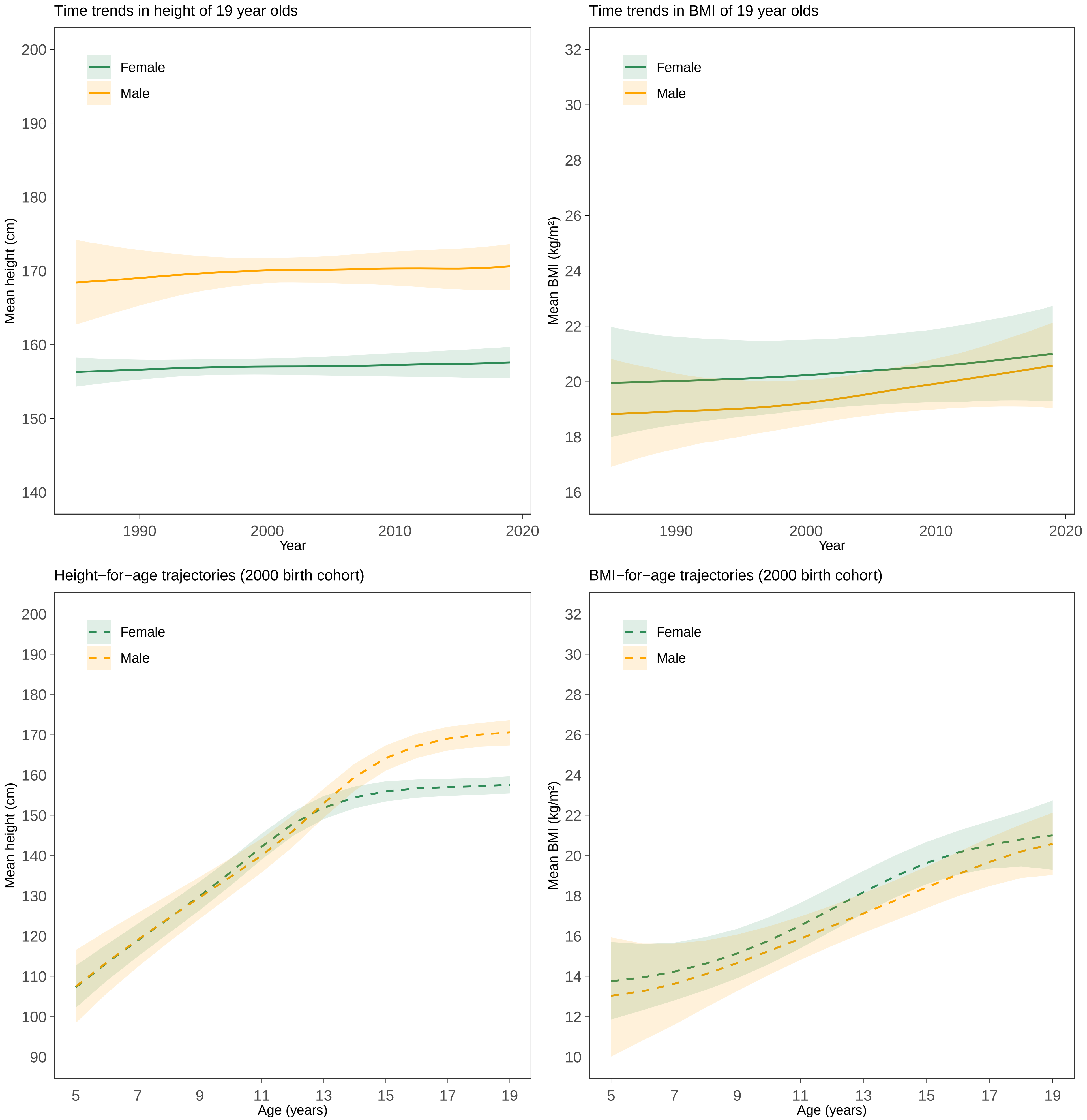
Time trends in height of 19 year olds
Female
Male
Mean height (cm)
200
190
180
170
160
150
140
1990
2000
2010
2020
Year
Time trends in BMI of 19 year olds
Female
Male
Mean BMI (kg/m²)
32
30
28
26
24
22
20
18
16
1990
2000
2010
2020
Year
Height−for−age trajectories (2000 birth cohort)
Female
Male
Mean height (cm)
200
190
180
170
160
150
140
130
120
110
100
90
5
7
9
11
13
15
17
19
Age (years)
BMI−for−age trajectories (2000 birth cohort)
Female
Male
Mean BMI (kg/m²)
32
30
28
26
24
22
20
18
16
14
12
10
5
7
9
11
13
15
17
19
Age (years)

Estonia

Time trends in height of 19 year olds

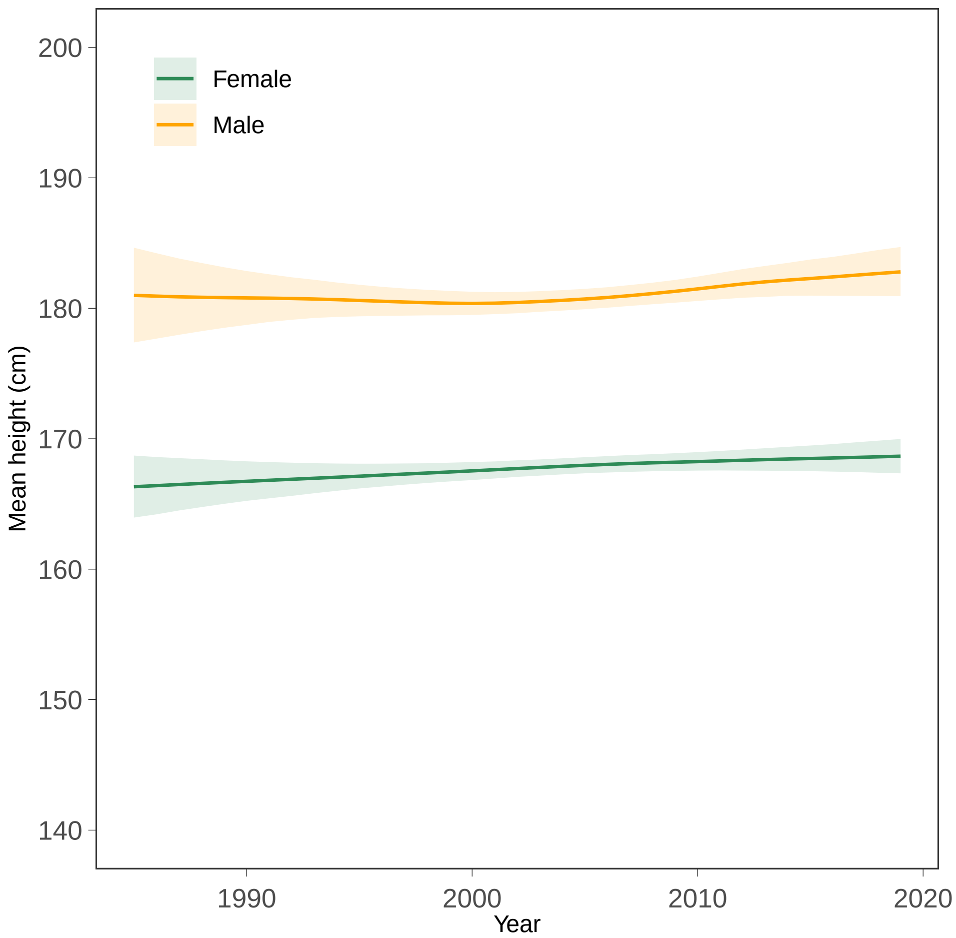

Time trends in BMI of 19 year olds

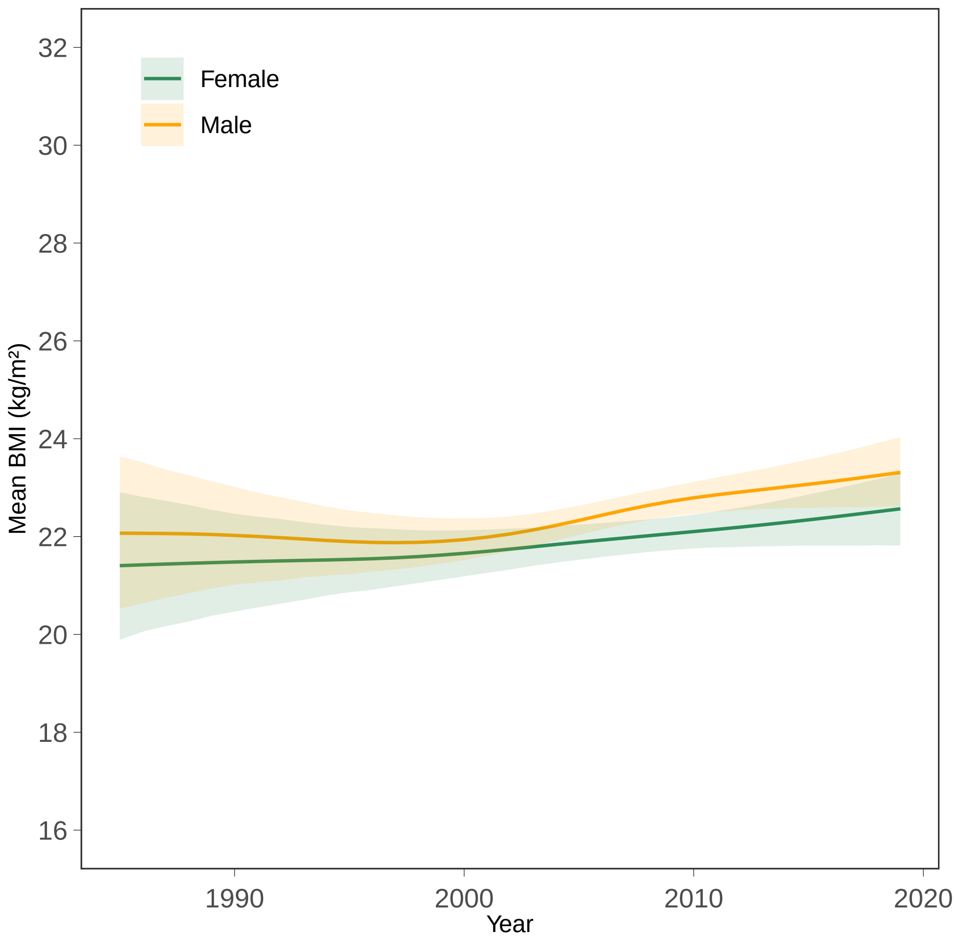

Height−for−age trajectories (2000 birth cohort)

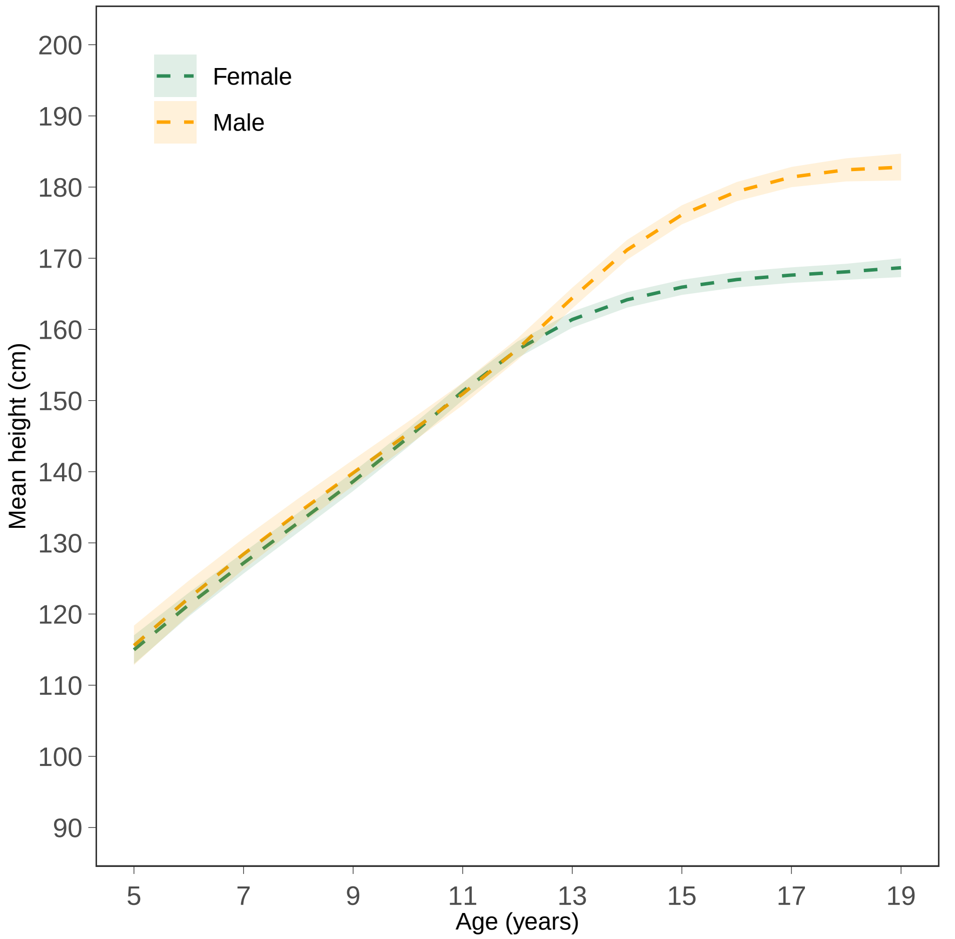

BMI−for−age trajectories (2000 birth cohort)

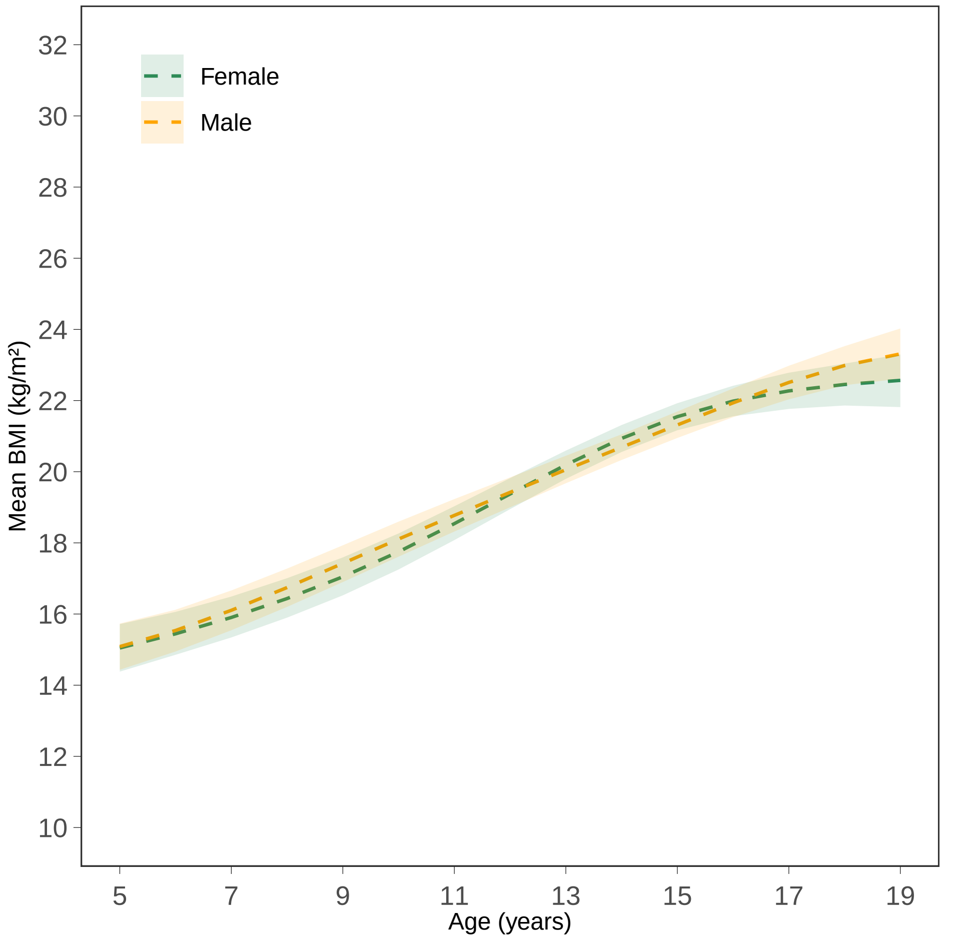

Eswatini

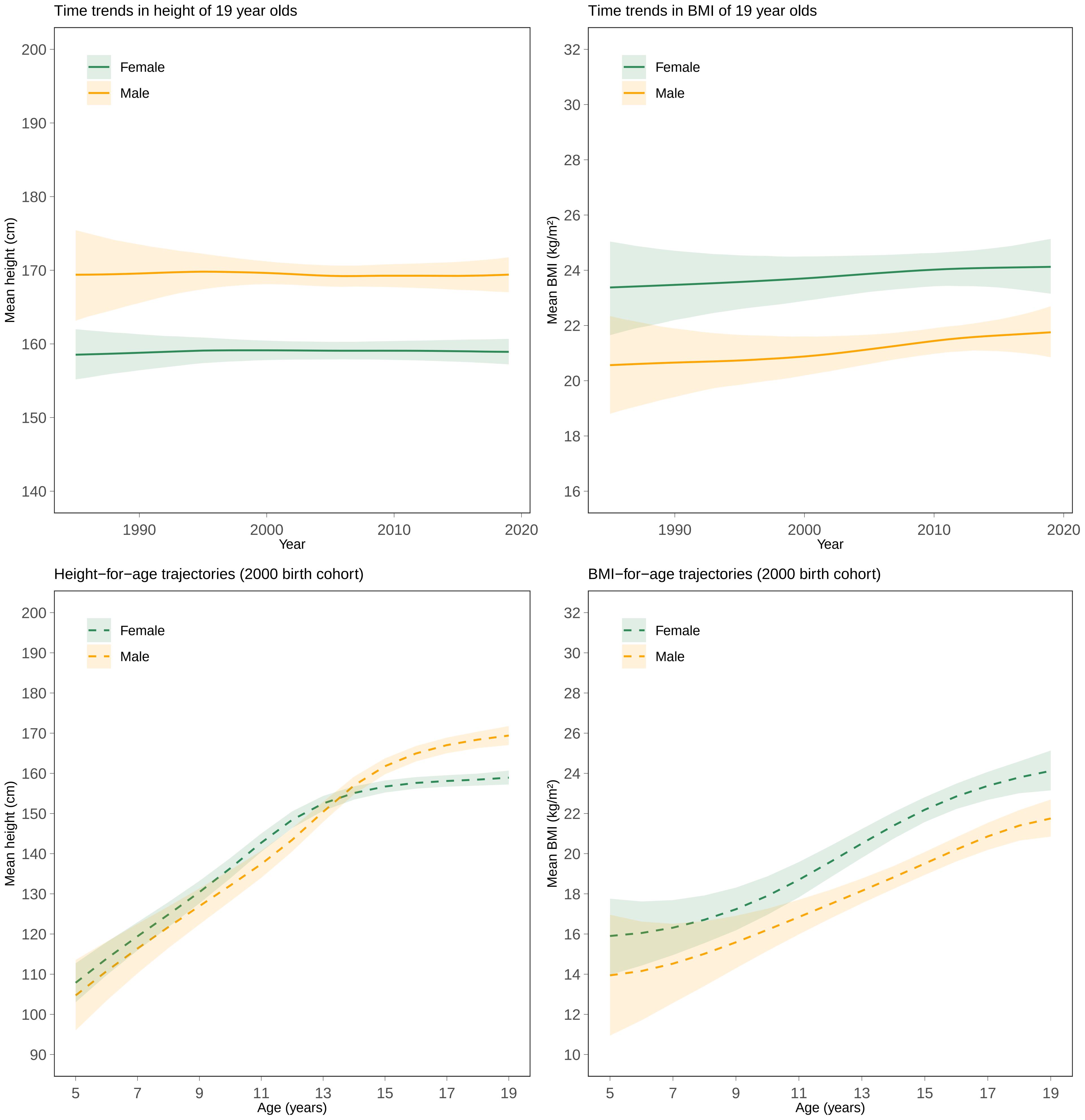

Time trends in height of 19 year olds
Female
Male
Mean height (cm)
200
190
180
170
160
150
140
1990
2000
2010
2020
Year
Time trends in BMI of 19 year olds
Female
Male
Mean BMI (kg/m²)
32
30
28
26
24
22
20
18
16
1990
2000
2010
2020
Year
Height−for−age trajectories (2000 birth cohort)
Female
Male
Mean height (cm)
200
190
180
170
160
150
140
130
120
110
100
90
5
7
9
11
13
15
17
19
Age (years)
BMI−for−age trajectories (2000 birth cohort)
Female
Male
Mean BMI (kg/m²)
32
30
28
26
24
22
20
18
16
14
12
10
5
7
9
11
13
15
17
19
Age (years)

Ethiopia

Time trends in height of 19 year olds

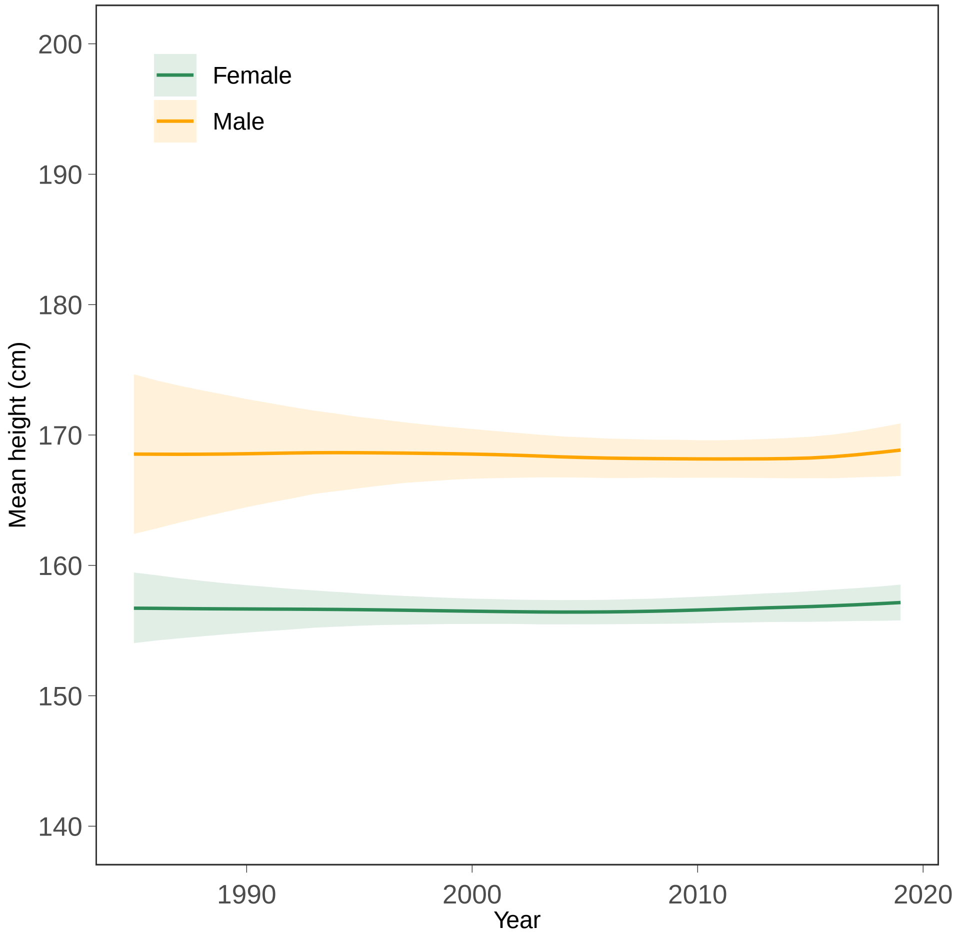

Time trends in BMI of 19 year olds

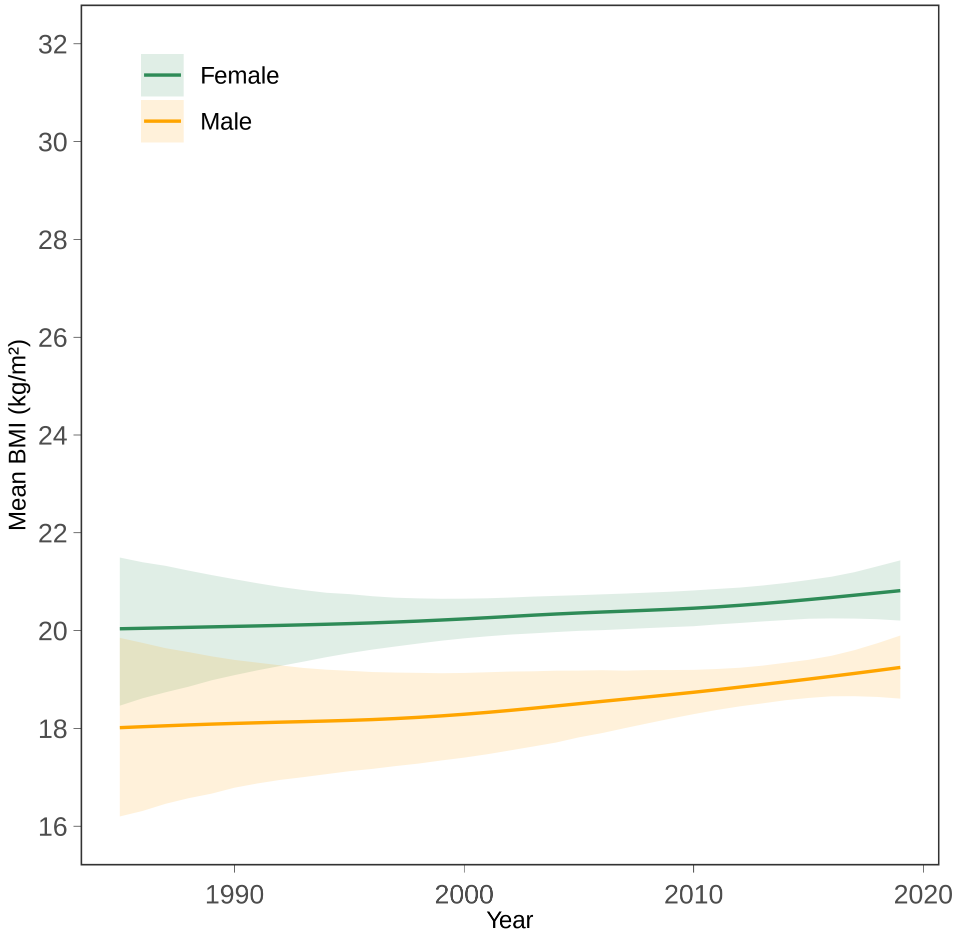

Height−for−age trajectories (2000 birth cohort)

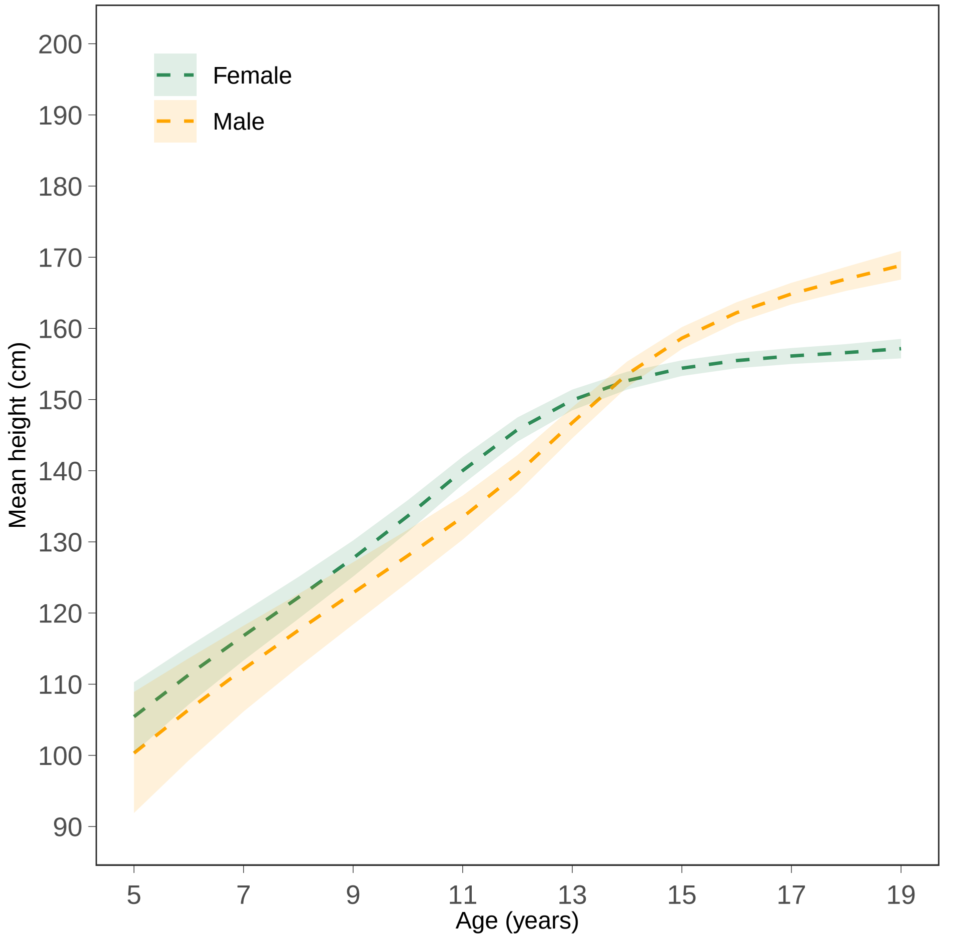

BMI−for−age trajectories (2000 birth cohort)

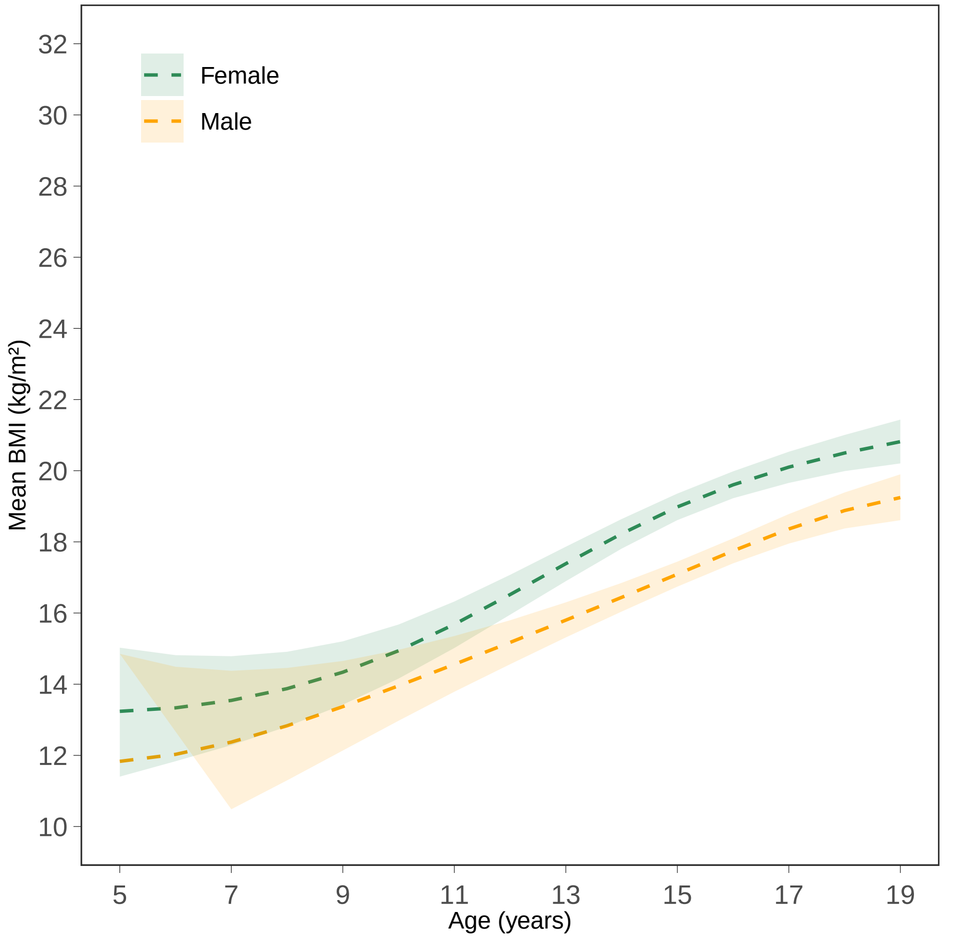

Fiji

Time trends in height of 19 year olds

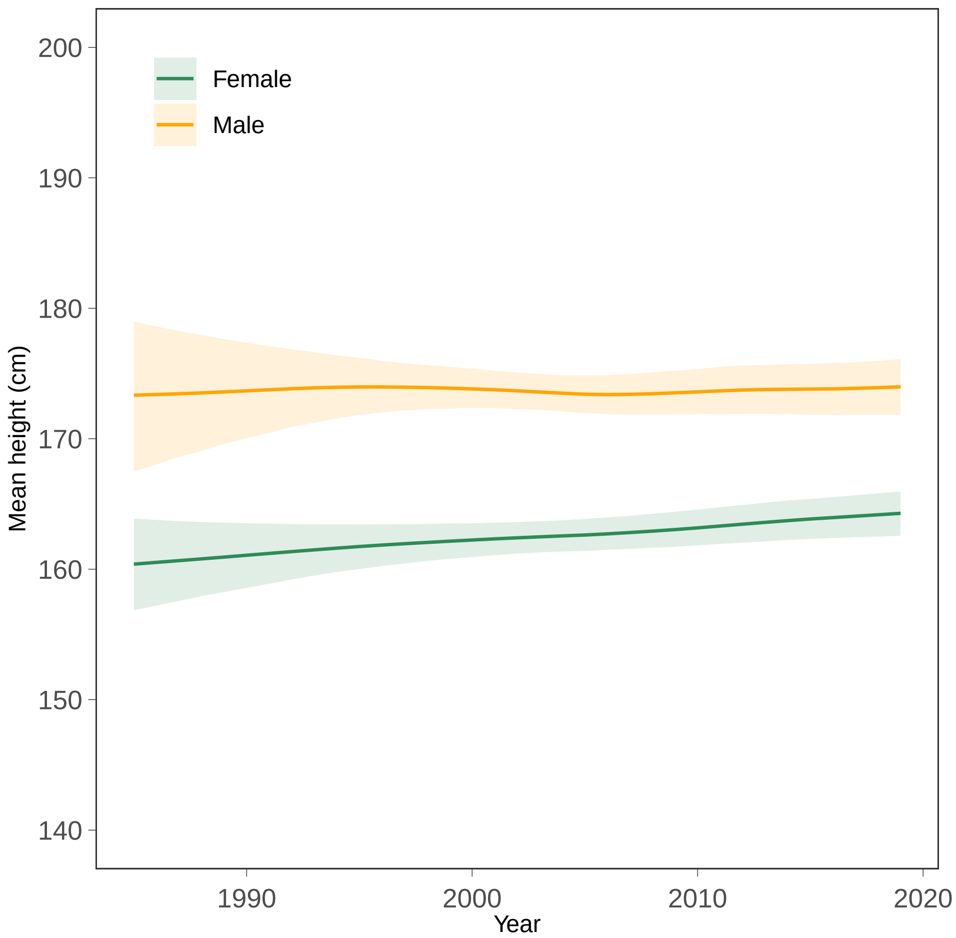

Time trends in BMI of 19 year olds

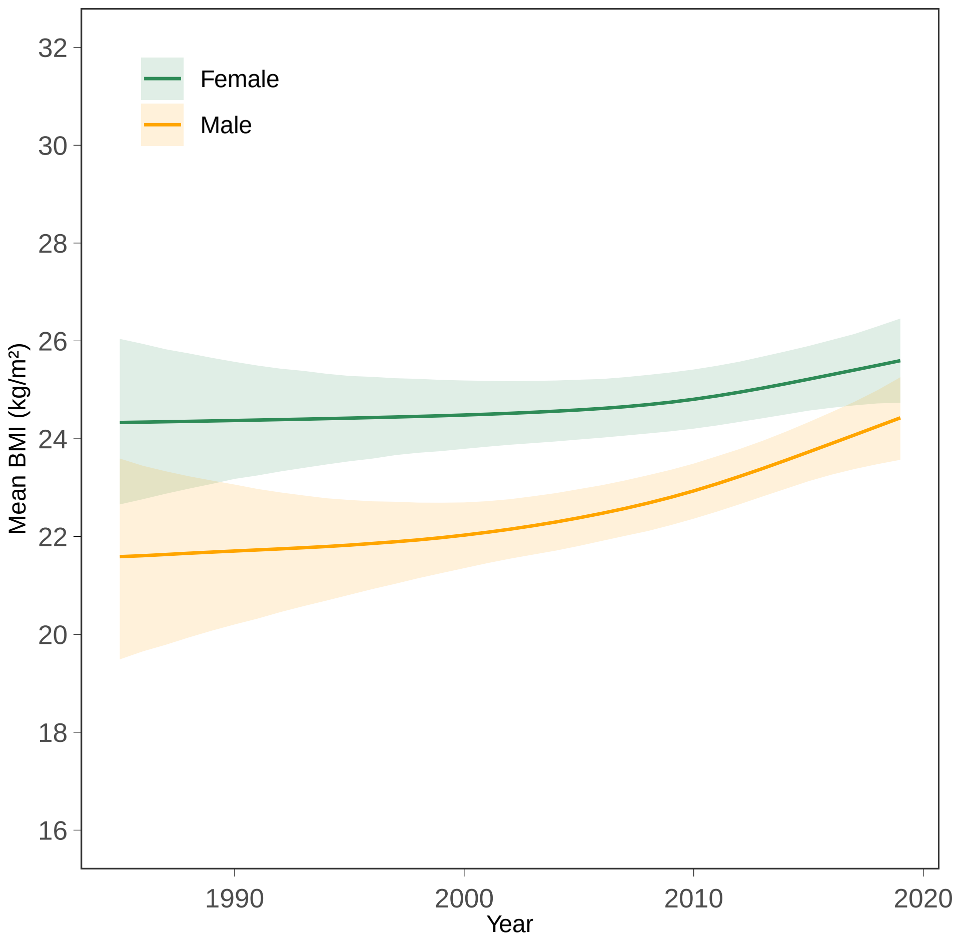

Height−for−age trajectories (2000 birth cohort)

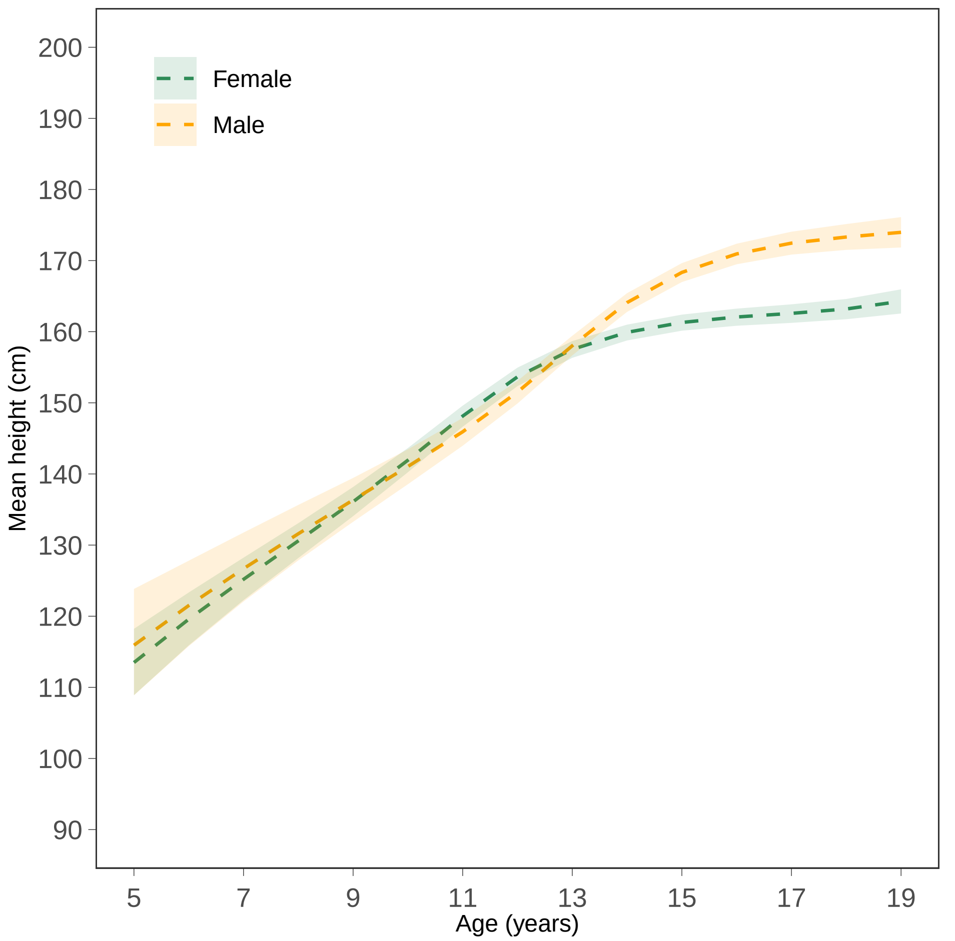

BMI−for−age trajectories (2000 birth cohort)

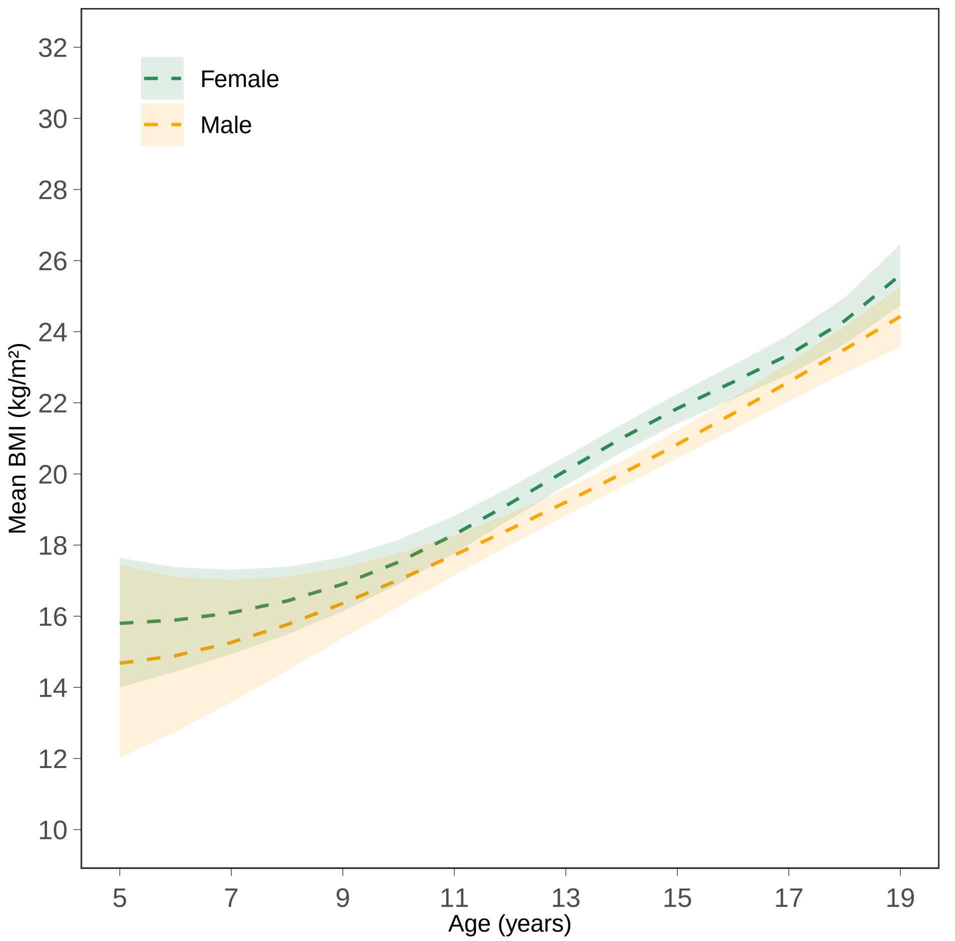

Finland

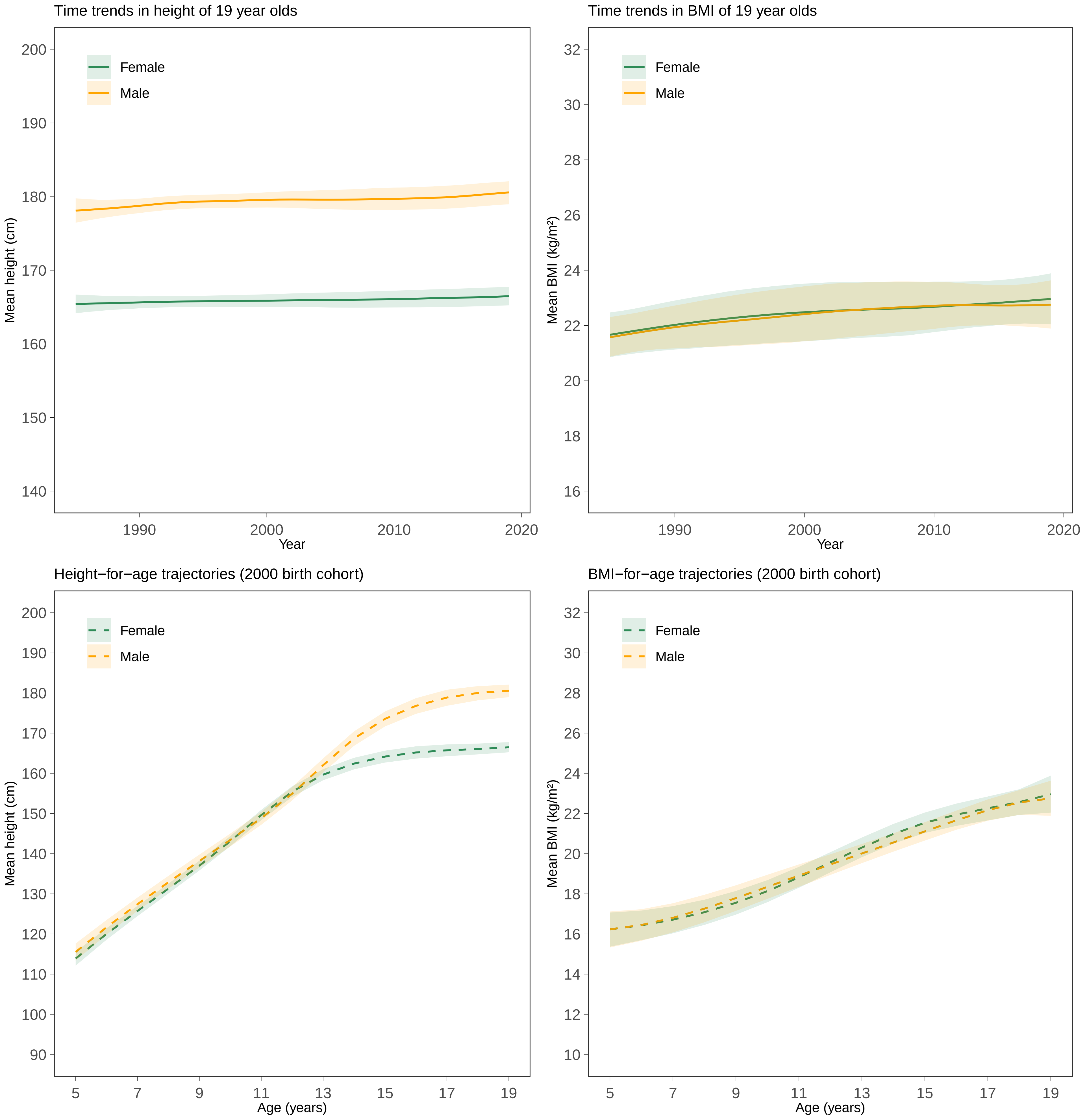
Time trends in height of 19 year olds
Female
Male
Mean height (cm)
200
190
180
170
160
150
140
1990
2000
2010
2020
Year
Time trends in BMI of 19 year olds
Female
Male
Mean BMI (kg/m²)
32
30
28
26
24
22
20
18
16
1990
2000
2010
2020
Year
Height−for−age trajectories (2000 birth cohort)
Female
Male
Mean height (cm)
200
190
180
170
160
150
140
130
120
110
100
90
5
7
9
11
13
15
17
19
Age (years)
BMI−for−age trajectories (2000 birth cohort)
Female
Male
Mean BMI (kg/m²)
32
30
28
26
24
22
20
18
16
14
12
10
5
7
9
11
13
15
17
19
Age (years)

France

Time trends in height of 19 year olds

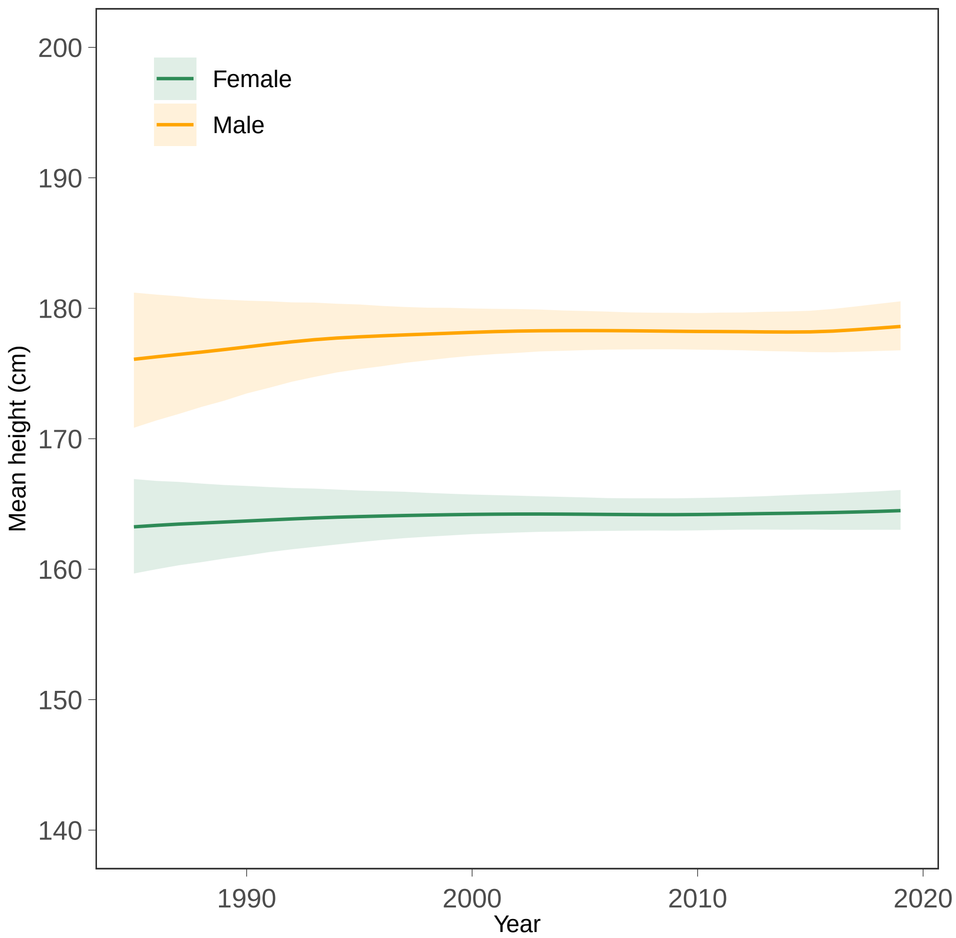

Time trends in BMI of 19 year olds

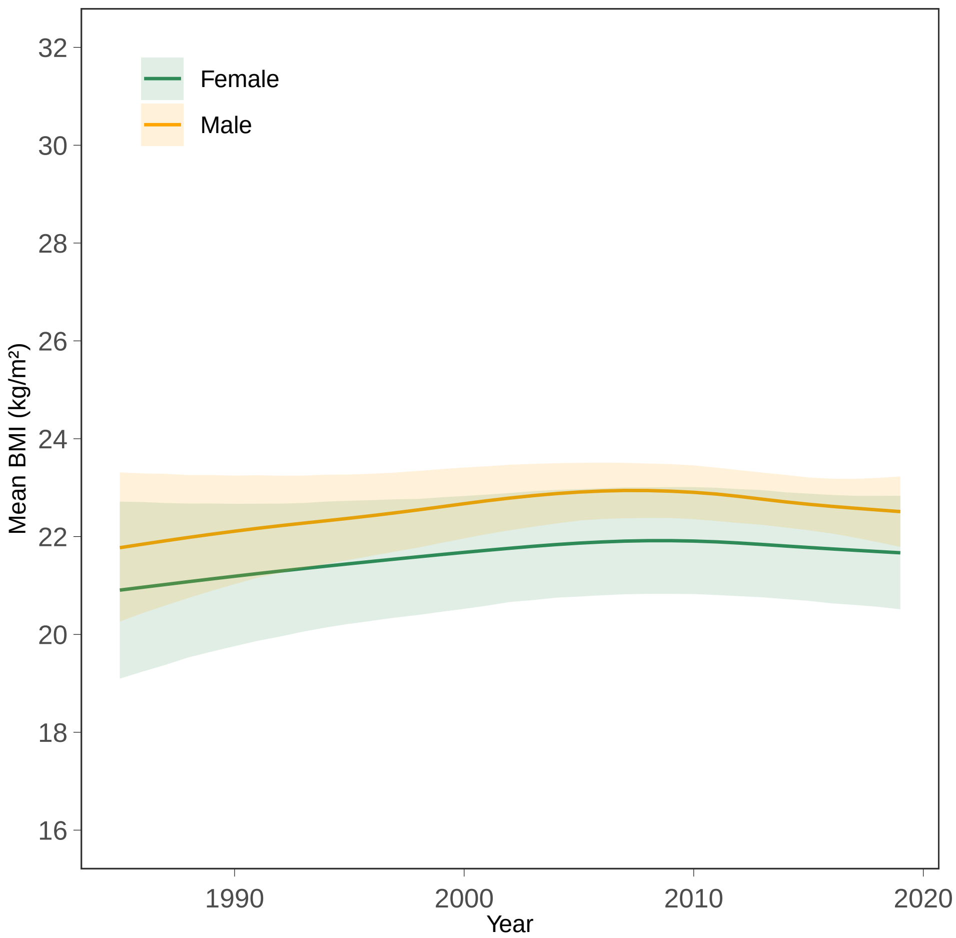

Height−for−age trajectories (2000 birth cohort)

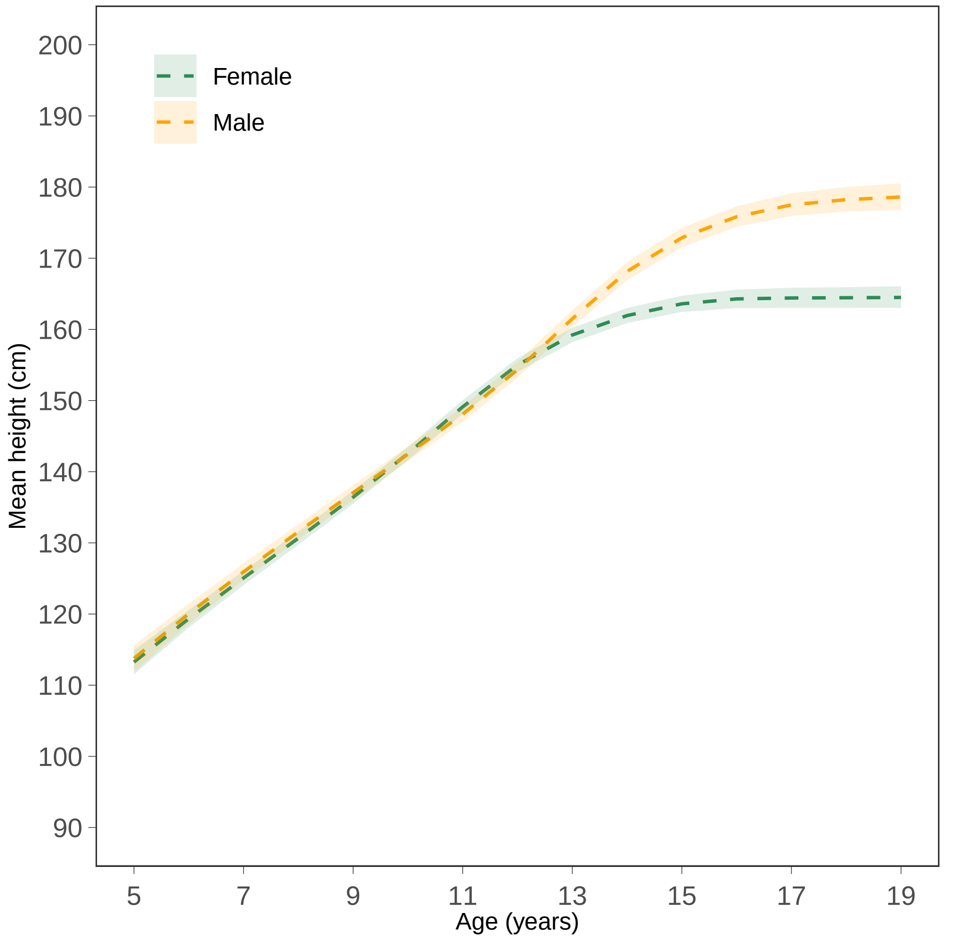

BMI−for−age trajectories (2000 birth cohort)

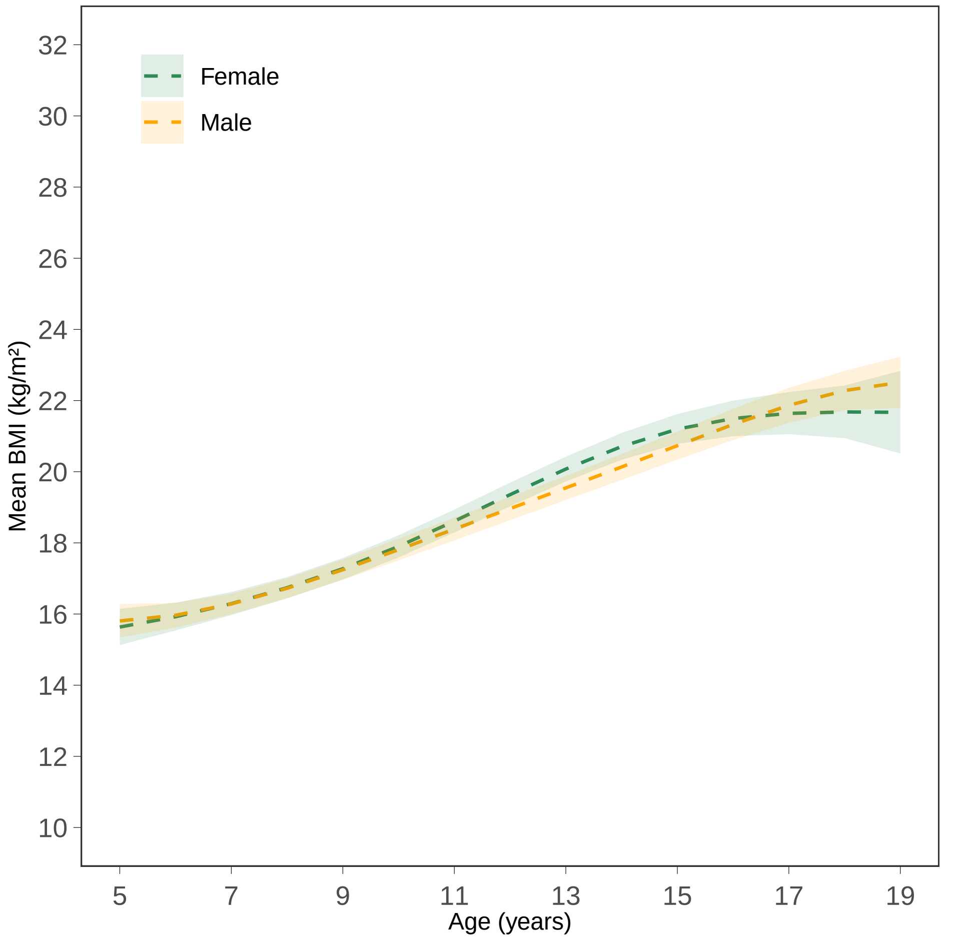

French Polynesia

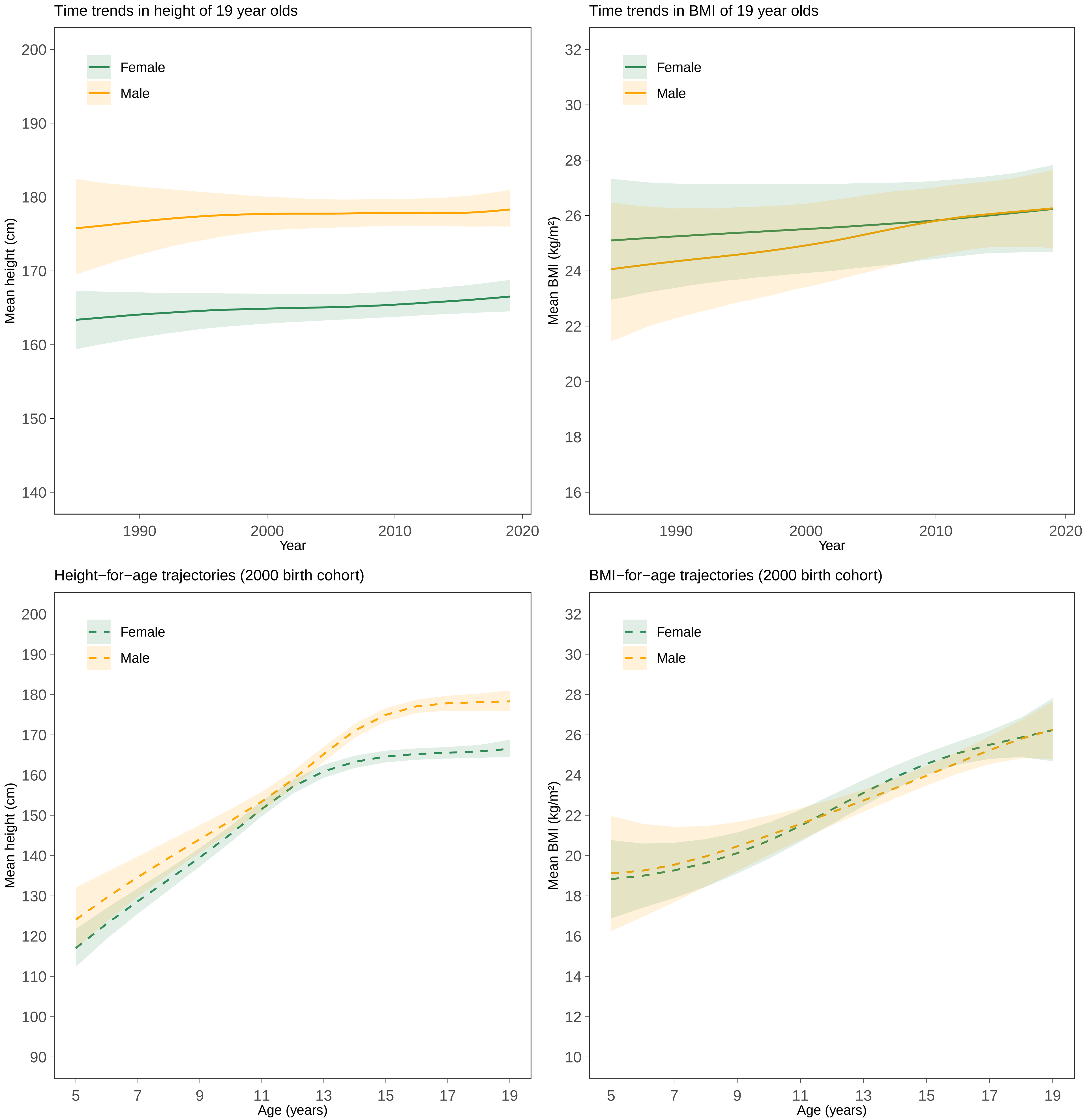

Gabon

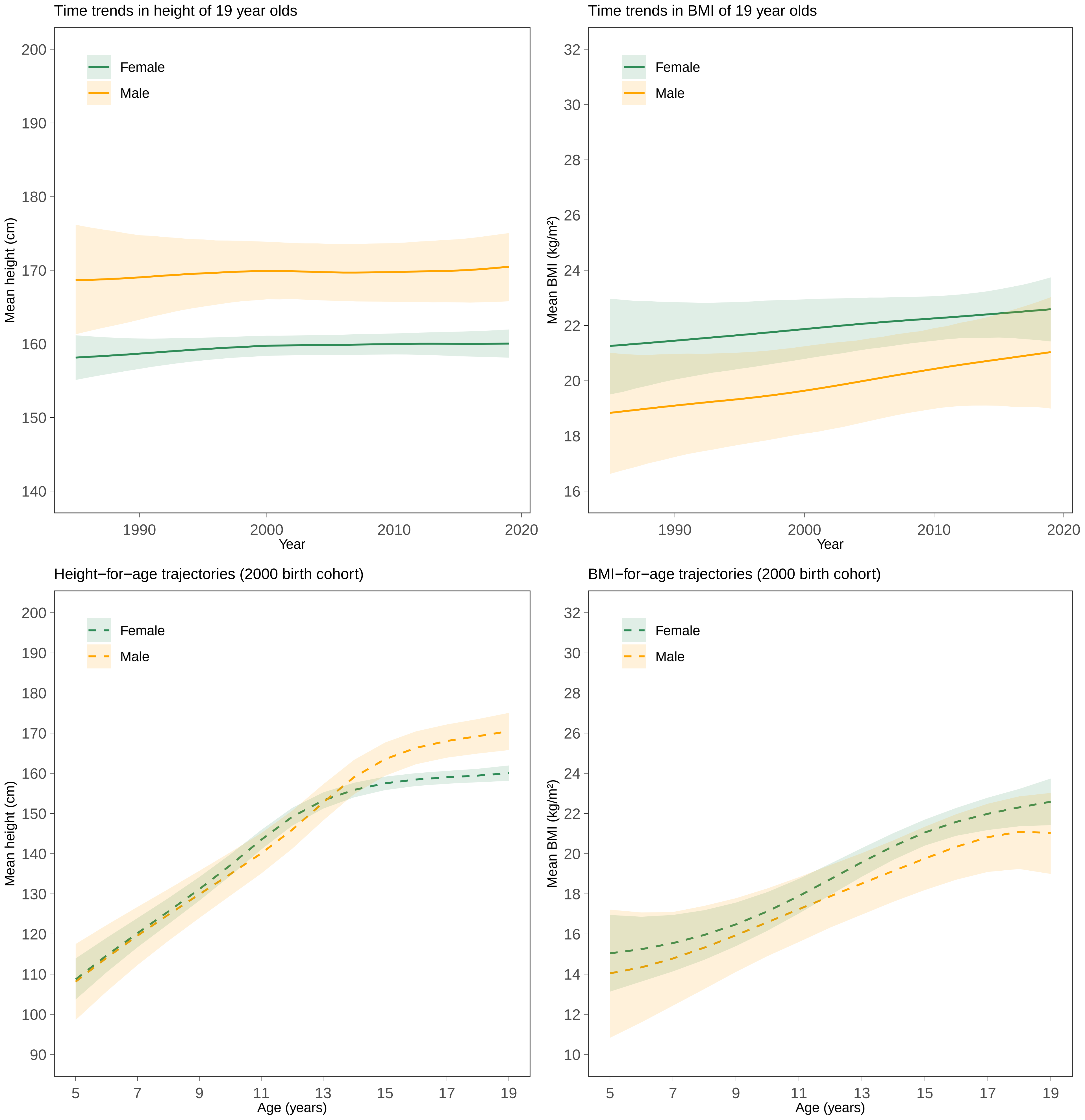

Time trends in height of 19 year olds
Female
Male
Mean height (cm)
200
190
180
170
160
150
140
1990
2000
2010
2020
Year
Time trends in BMI of 19 year olds
Female
Male
Mean BMI (kg/m²)
32
30
28
26
24
22
20
18
16
1990
2000
2010
2020
Year
Height−for−age trajectories (2000 birth cohort)
Female
Male
Mean height (cm)
200
190
180
170
160
150
140
130
120
110
100
90
5
7
9
11
13
15
17
19
Age (years)
BMI−for−age trajectories (2000 birth cohort)
Female
Male
Mean BMI (kg/m²)
32
30
28
26
24
22
20
18
16
14
12
10
5
7
9
11
13
15
17
19
Age (years)

Georgia

Time trends in height of 19 year olds

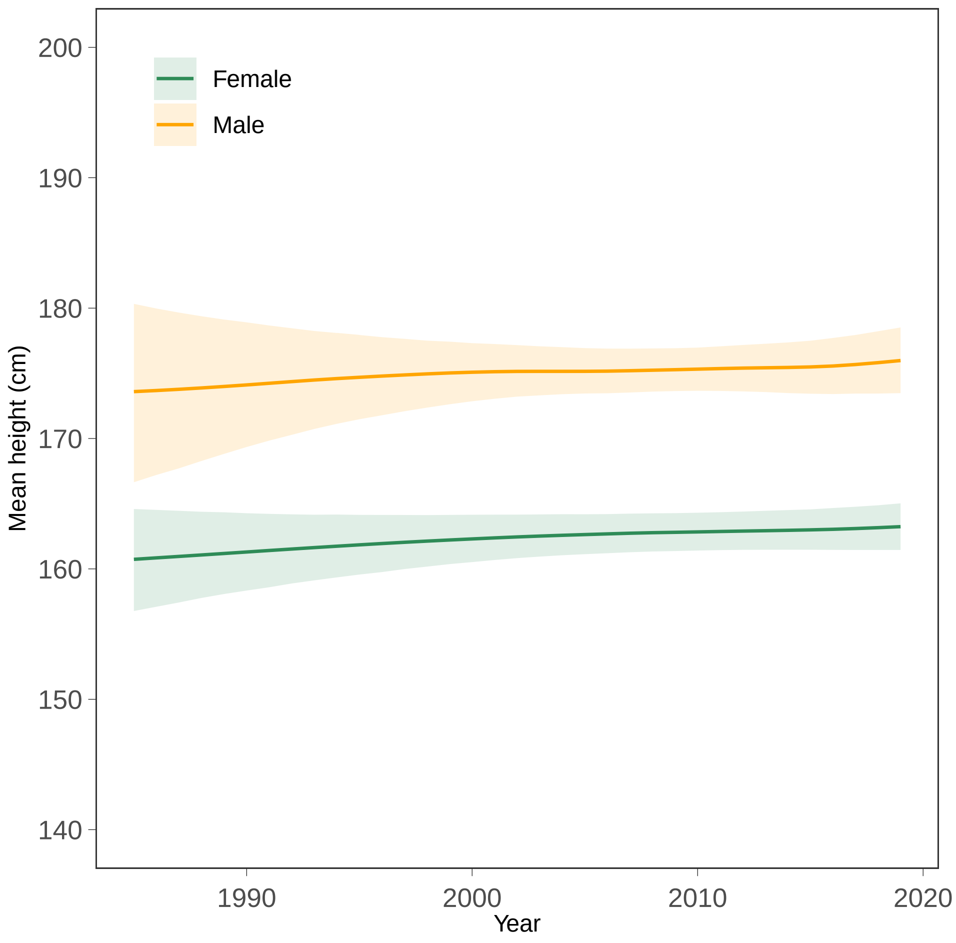

Time trends in BMI of 19 year olds

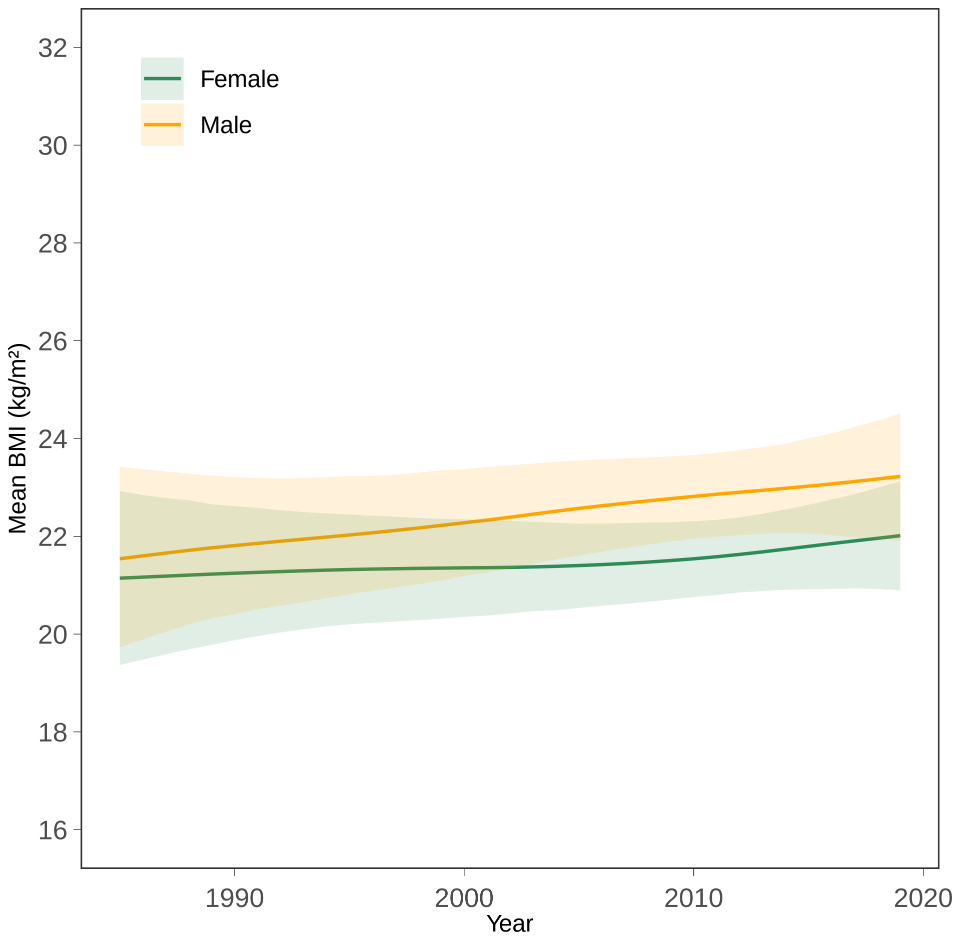

Height−for−age trajectories (2000 birth cohort)

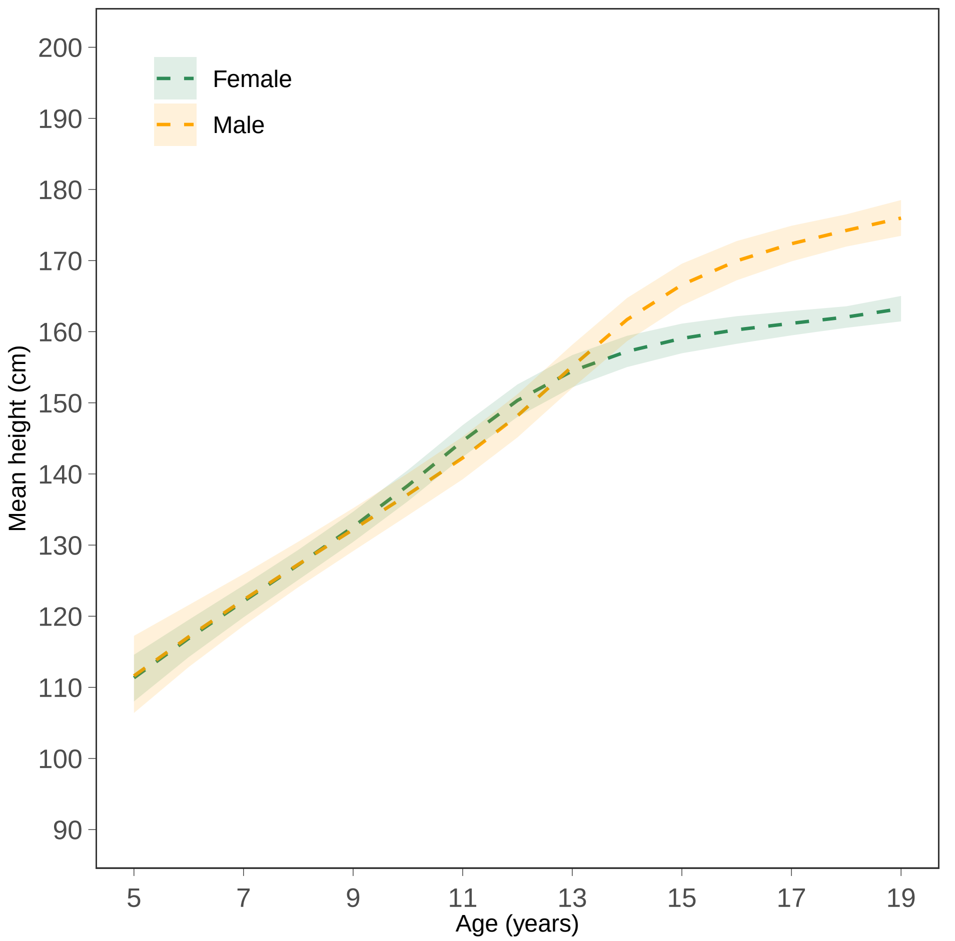

BMI−for−age trajectories (2000 birth cohort)

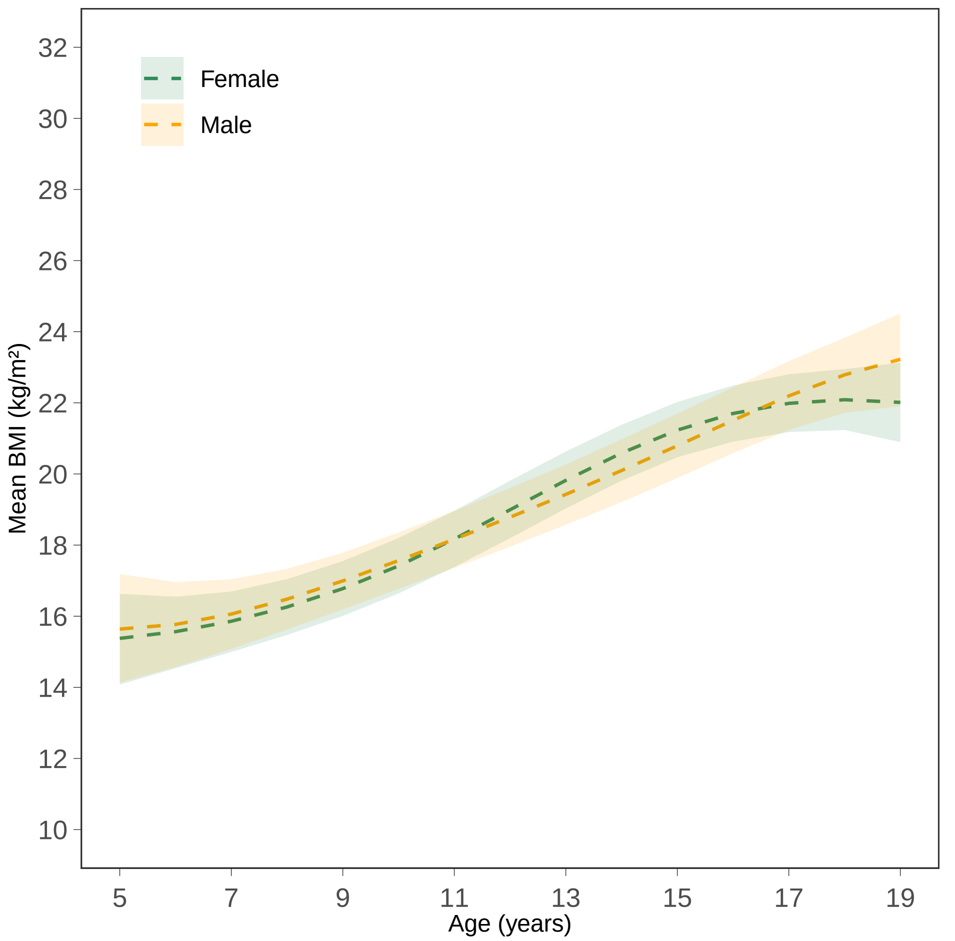

Germany

Time trends in height of 19 year olds

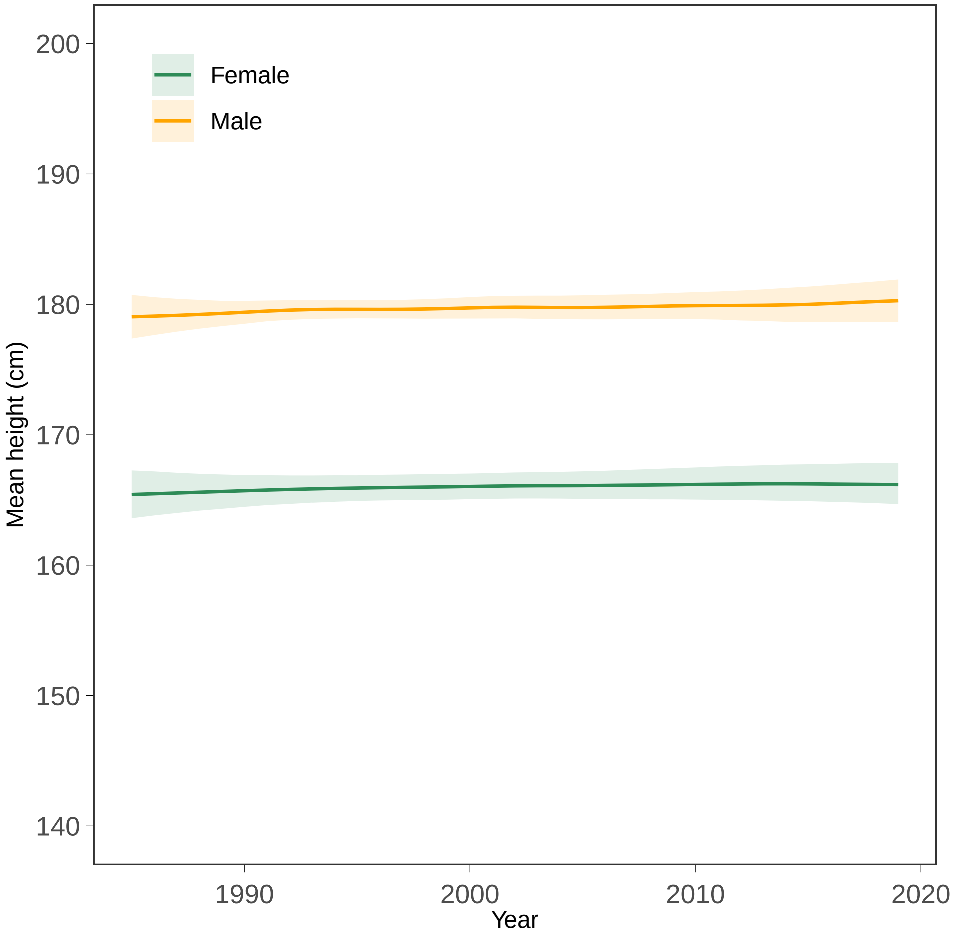

Time trends in BMI of 19 year olds

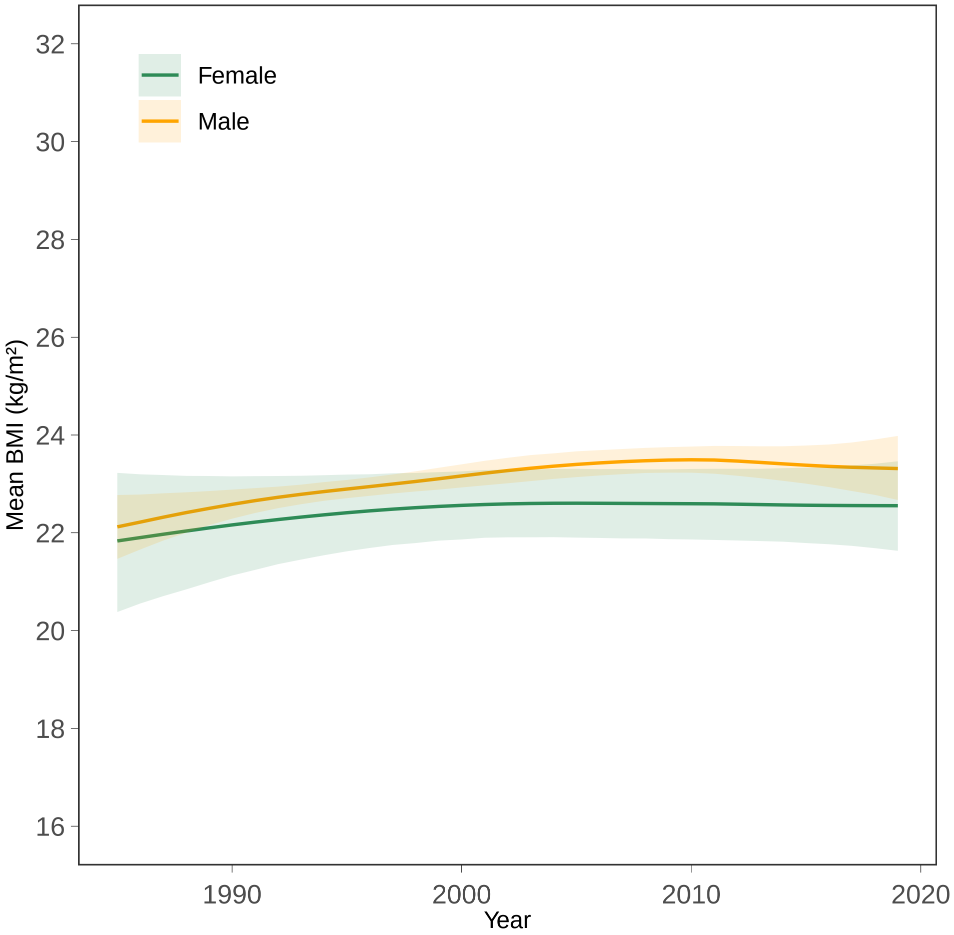

Height−for−age trajectories (2000 birth cohort)

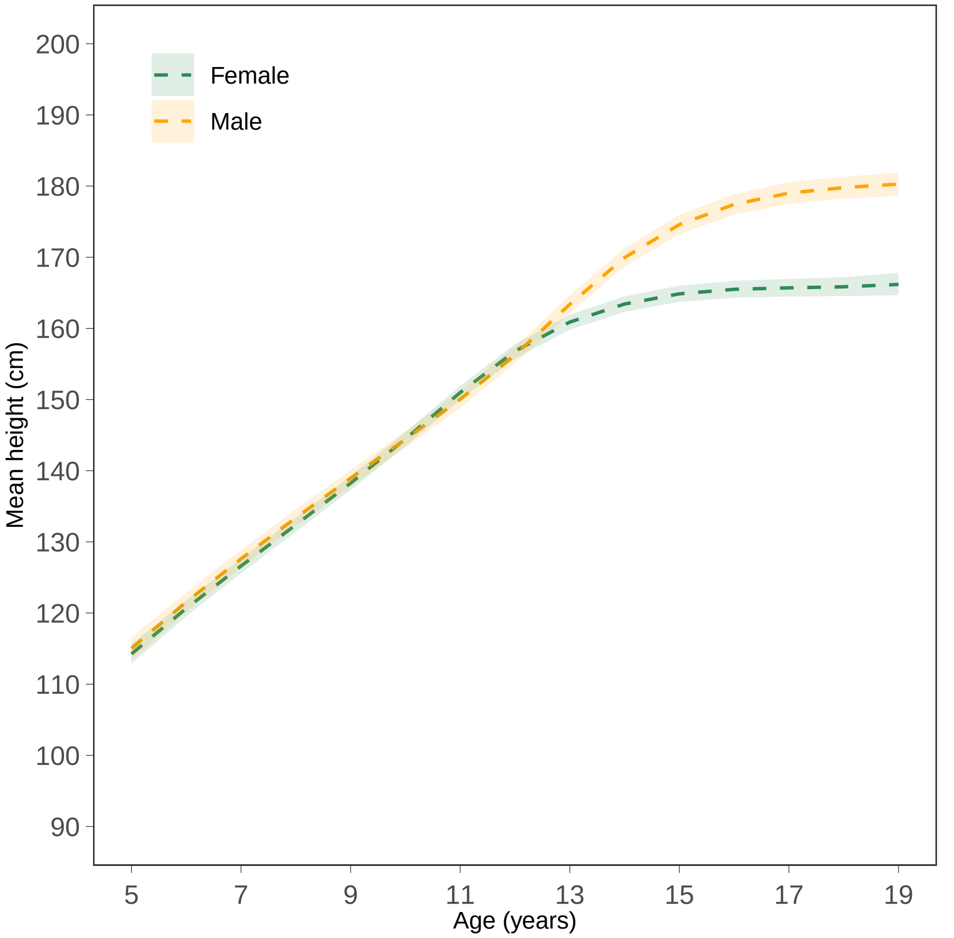

BMI−for−age trajectories (2000 birth cohort)

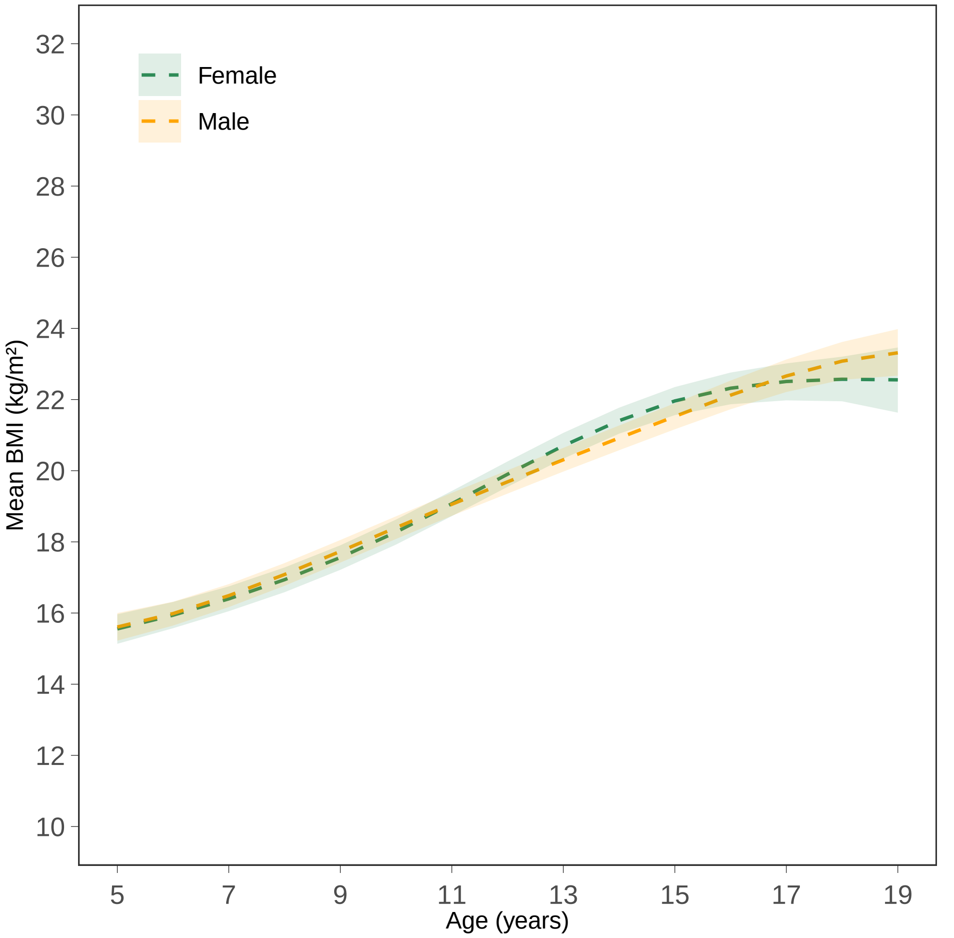

Ghana

Time trends in height of 19 year olds

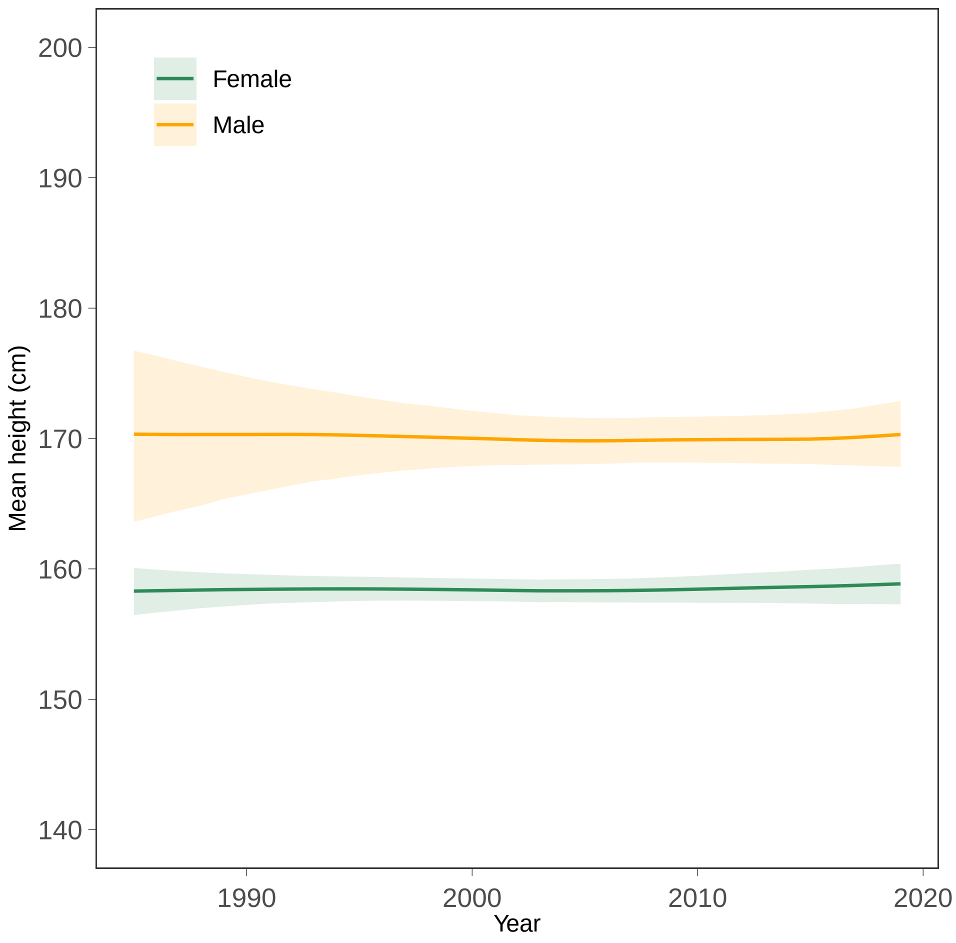

Time trends in BMI of 19 year olds

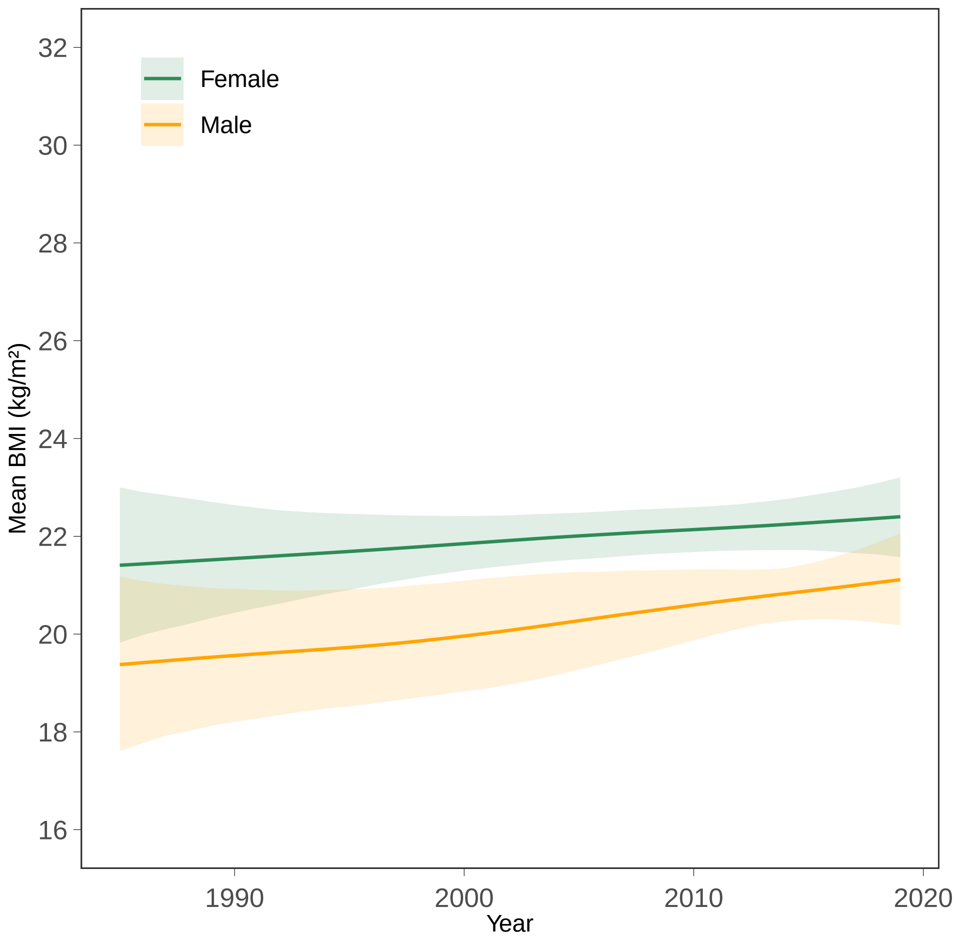

Height−for−age trajectories (2000 birth cohort)

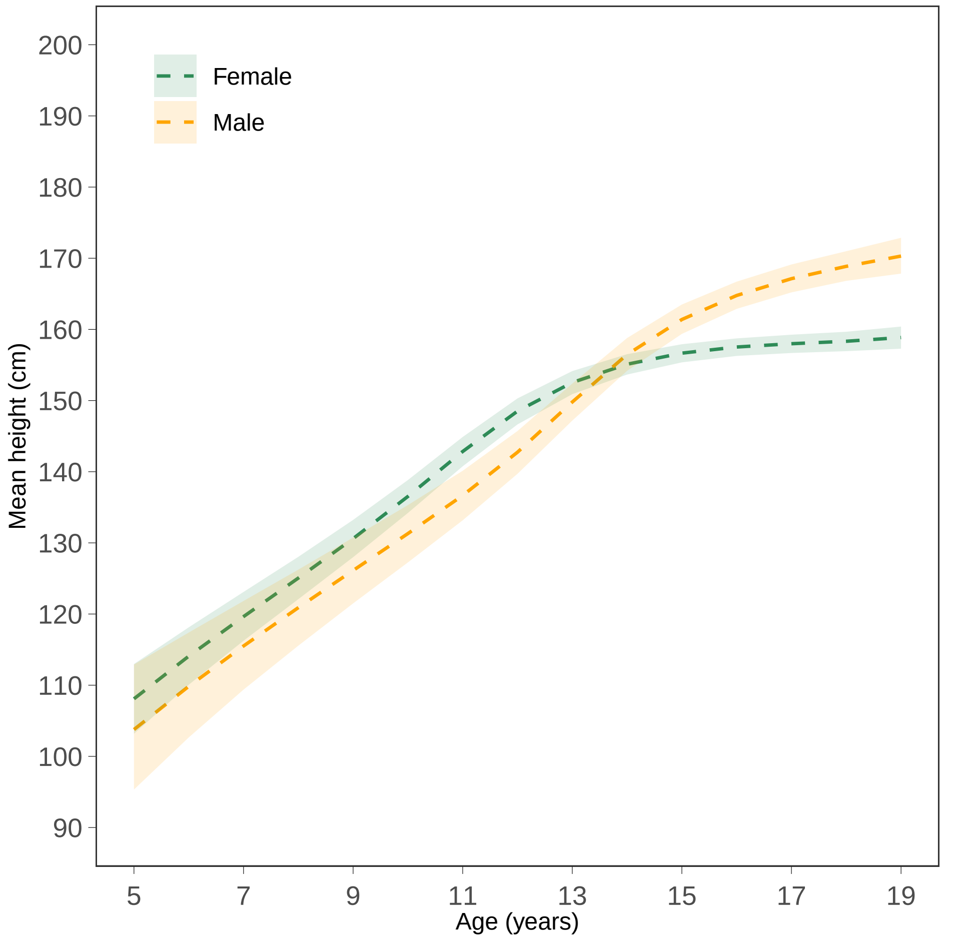

BMI−for−age trajectories (2000 birth cohort)

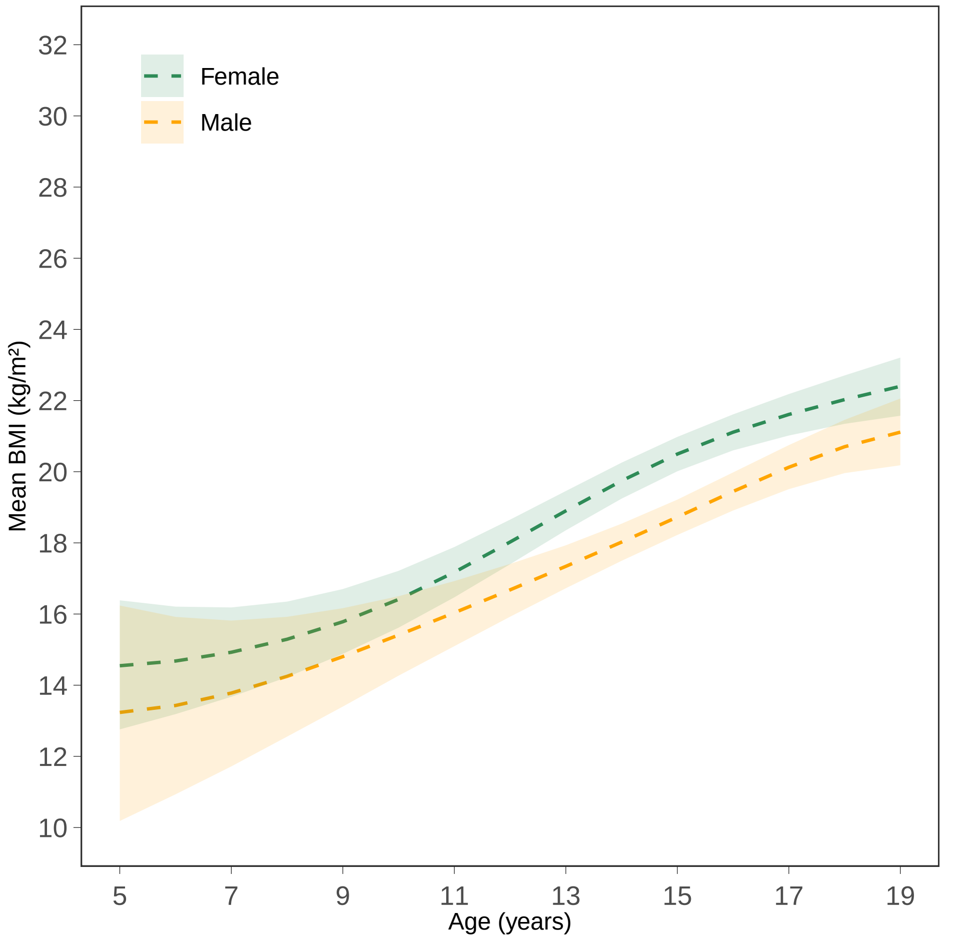

Greece

Time trends in height of 19 year olds

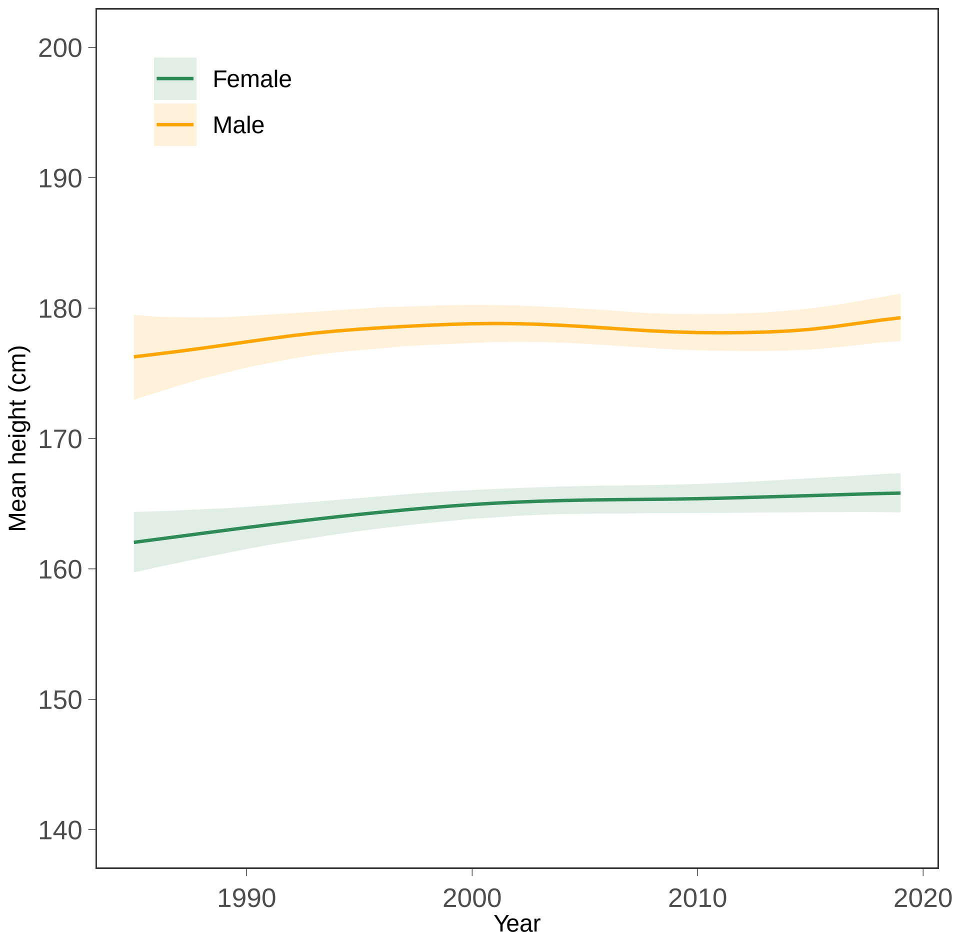

Time trends in BMI of 19 year olds

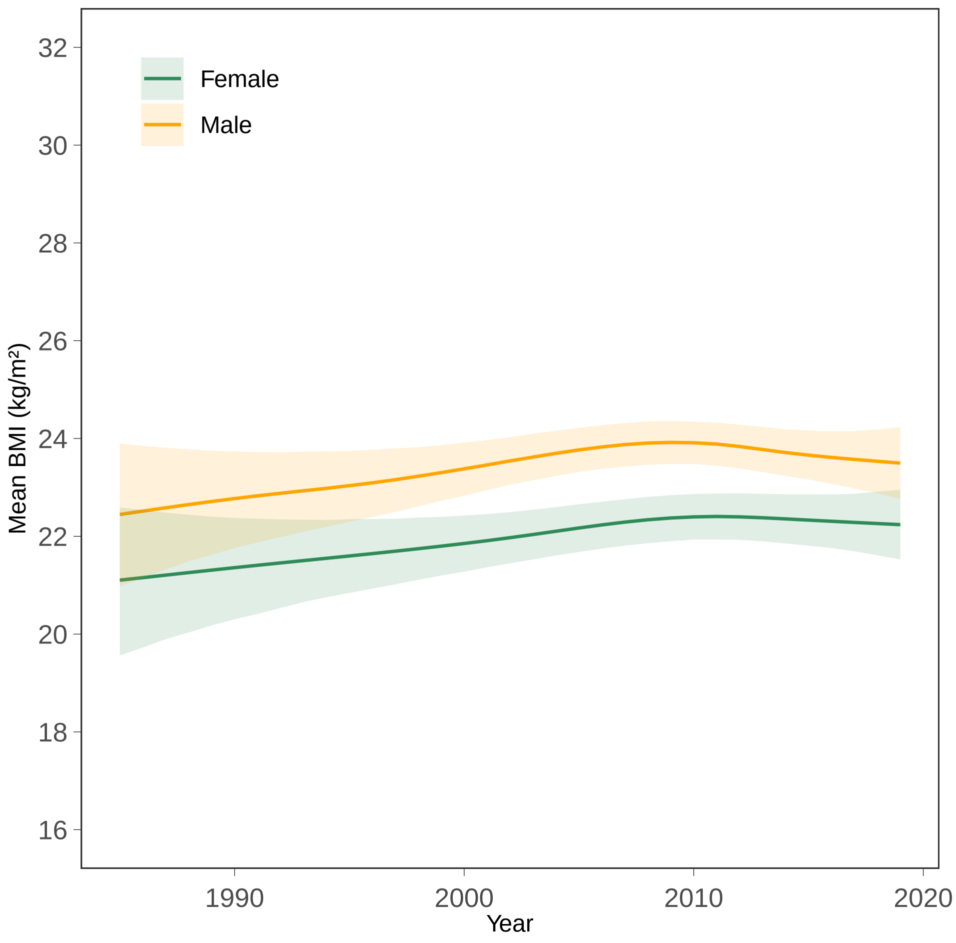

Height−for−age trajectories (2000 birth cohort)

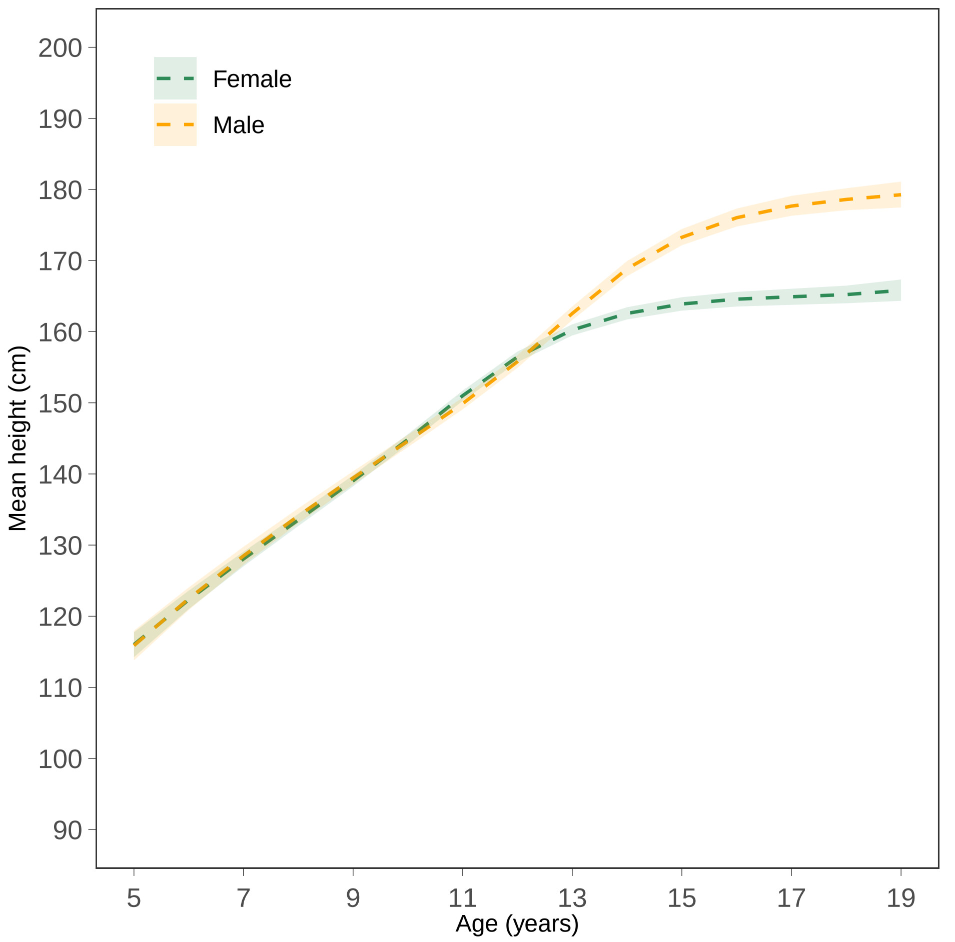

BMI−for−age trajectories (2000 birth cohort)

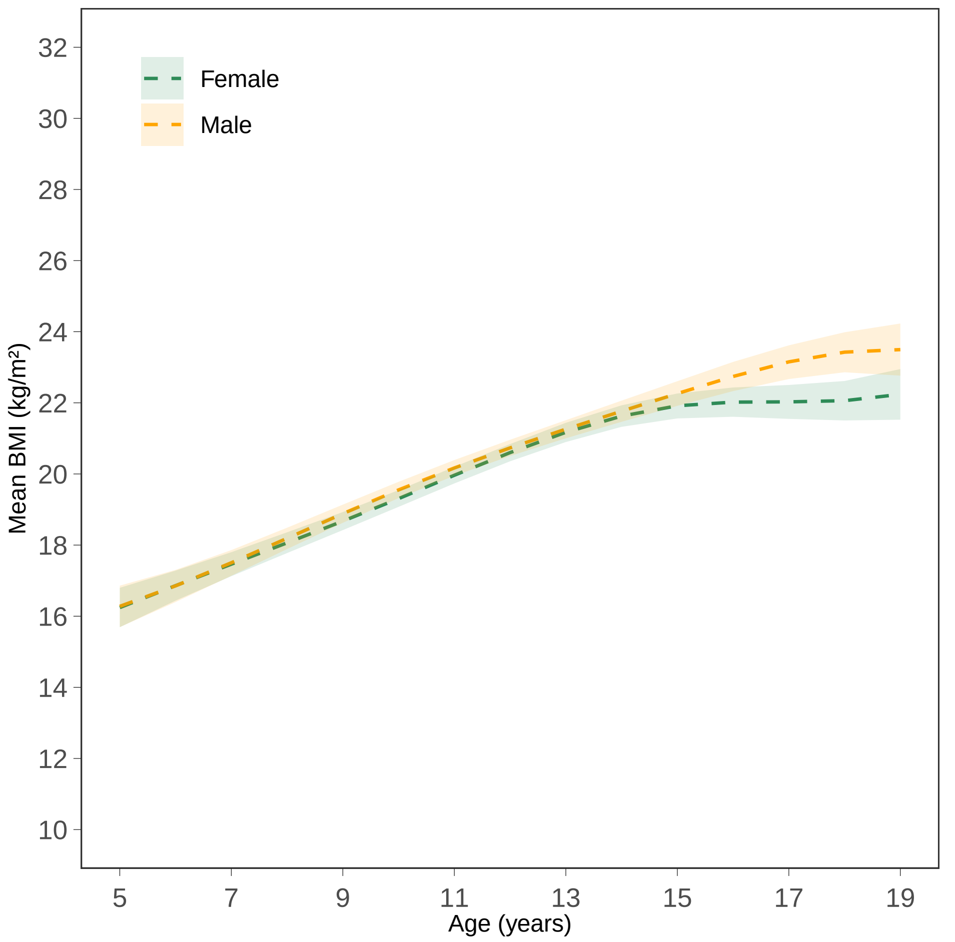

Greenland

Time trends in height of 19 year olds

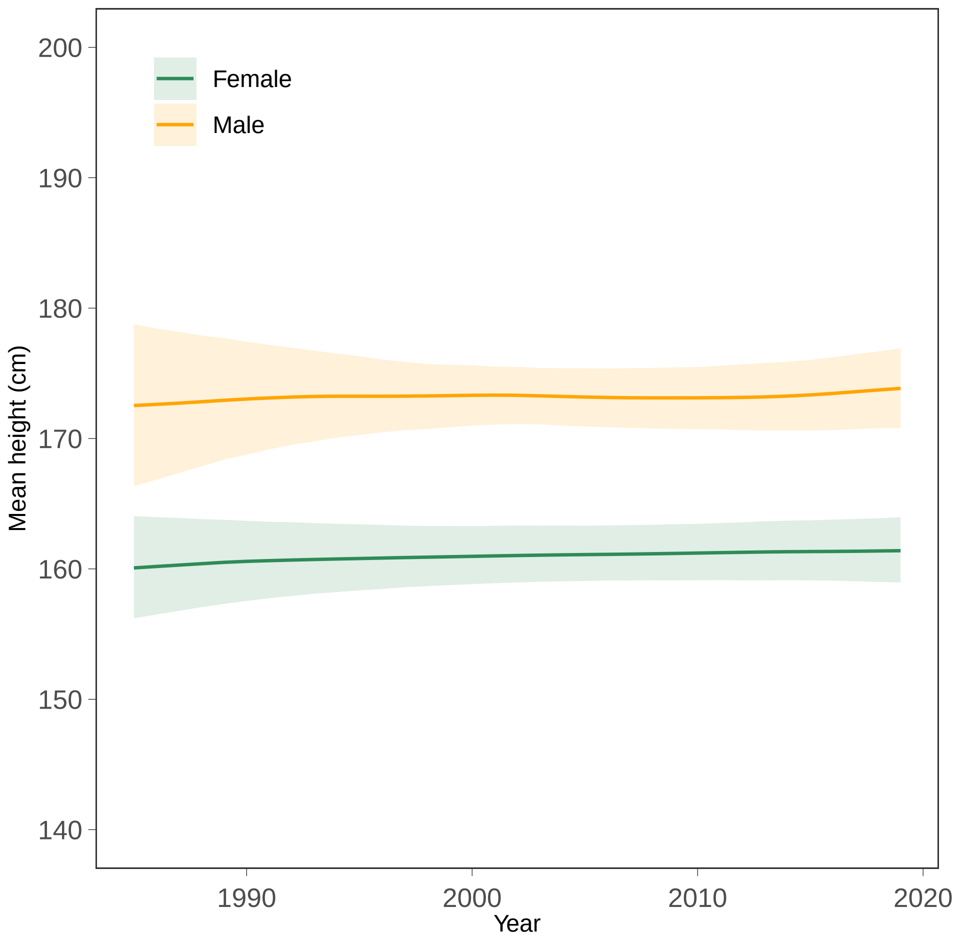

Time trends in BMI of 19 year olds

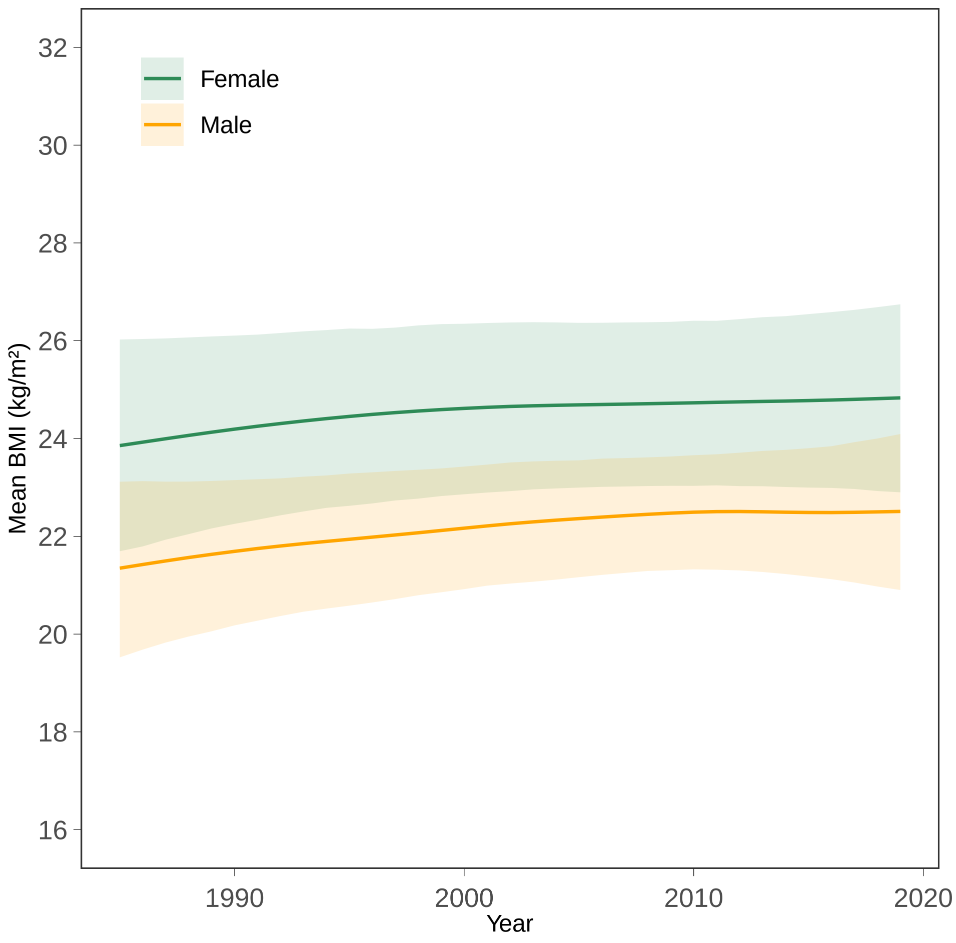

Height−for−age trajectories (2000 birth cohort)

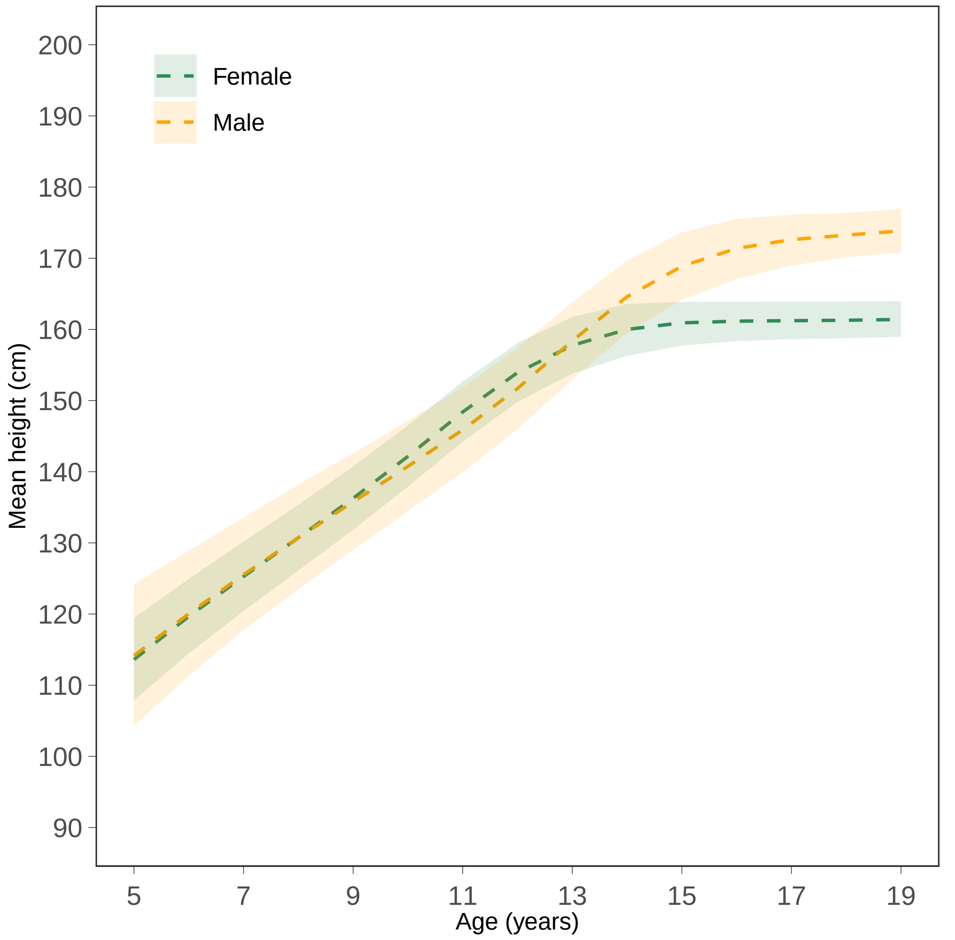

BMI−for−age trajectories (2000 birth cohort)

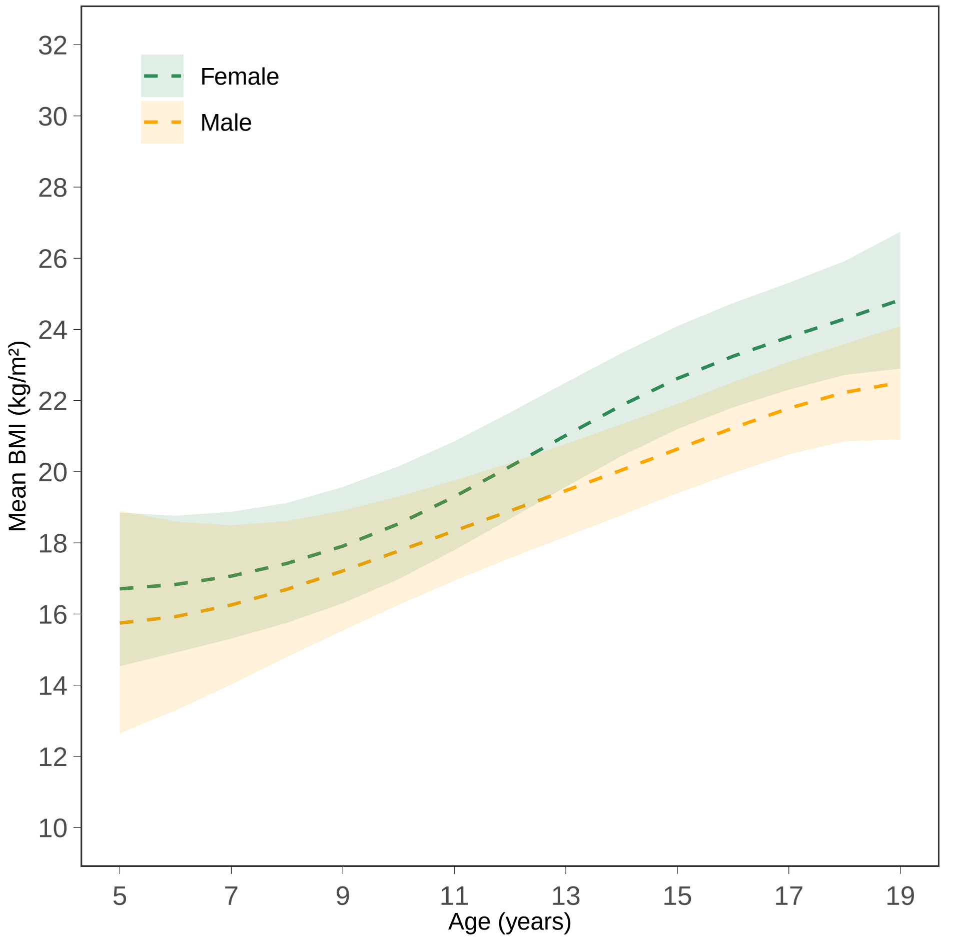

Grenada

Time trends in height of 19 year olds

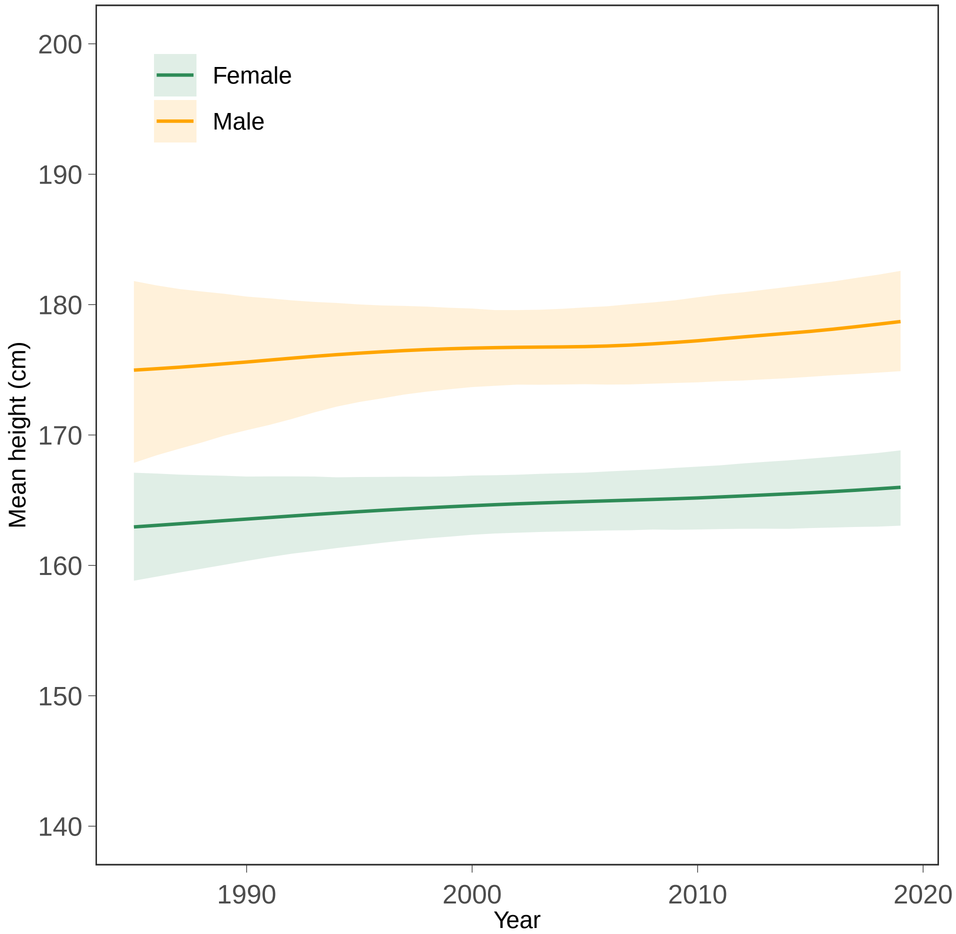

Time trends in BMI of 19 year olds

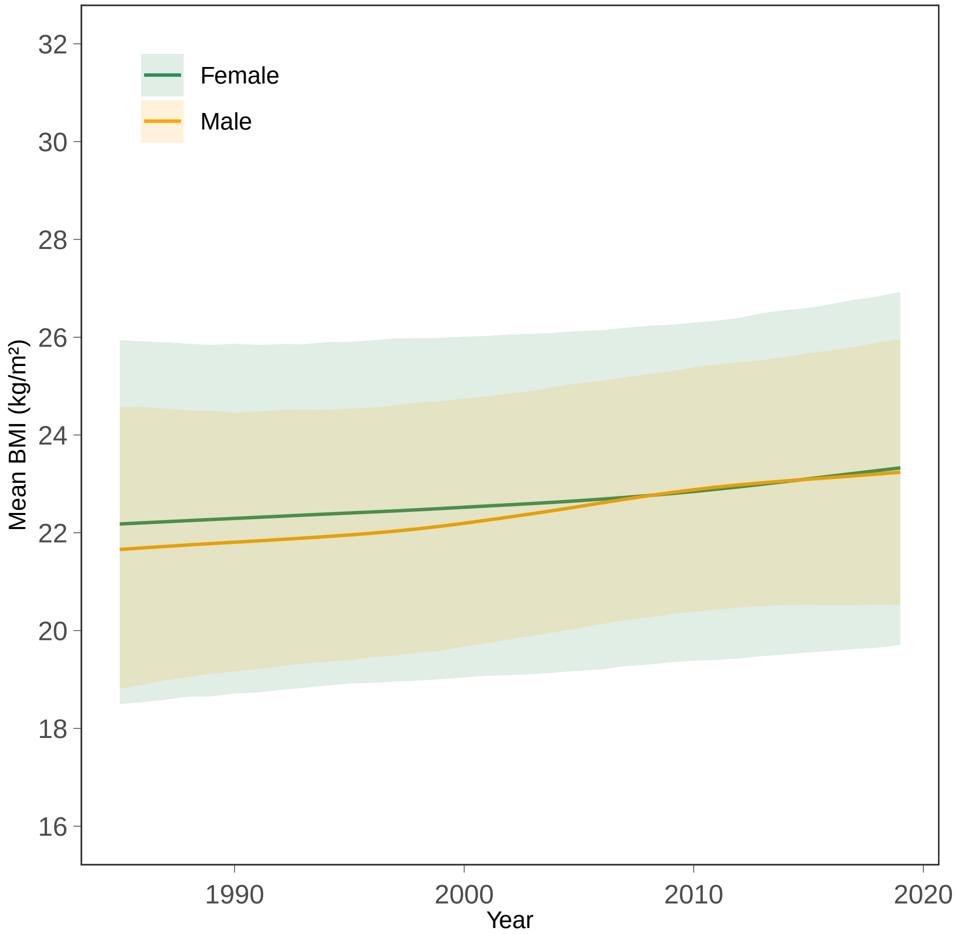

Height−for−age trajectories (2000 birth cohort)

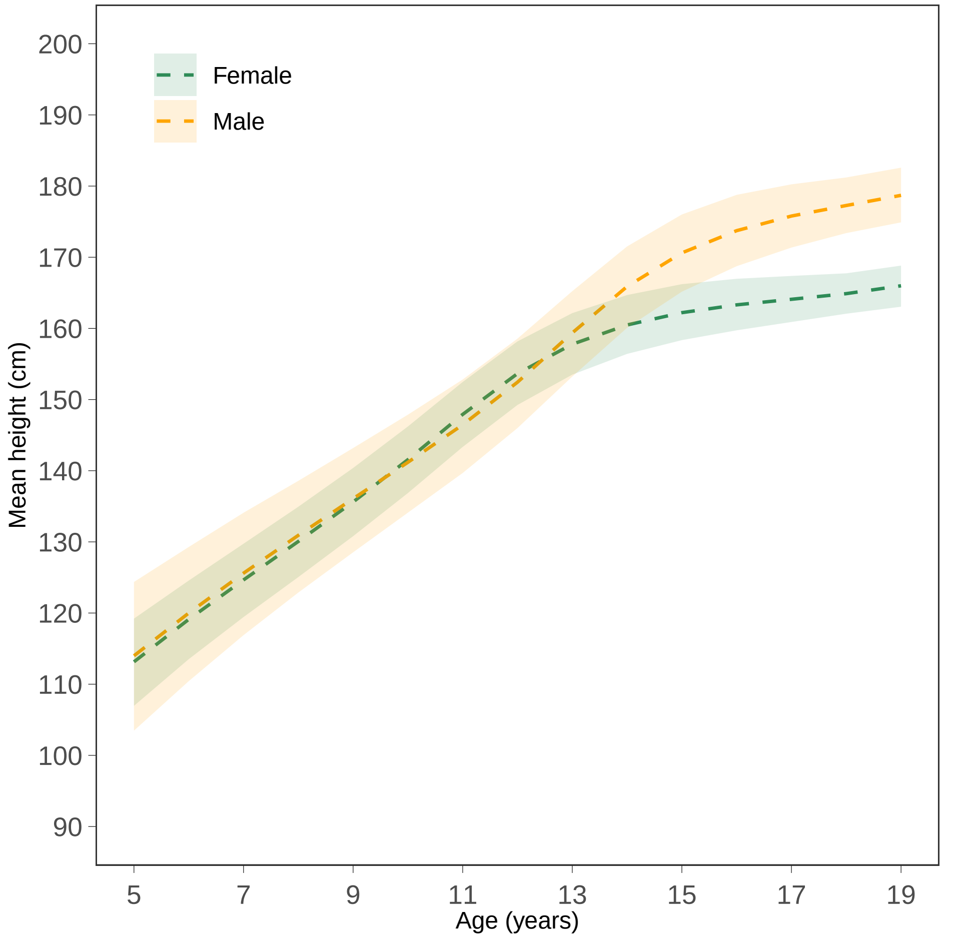

BMI−for−age trajectories (2000 birth cohort)

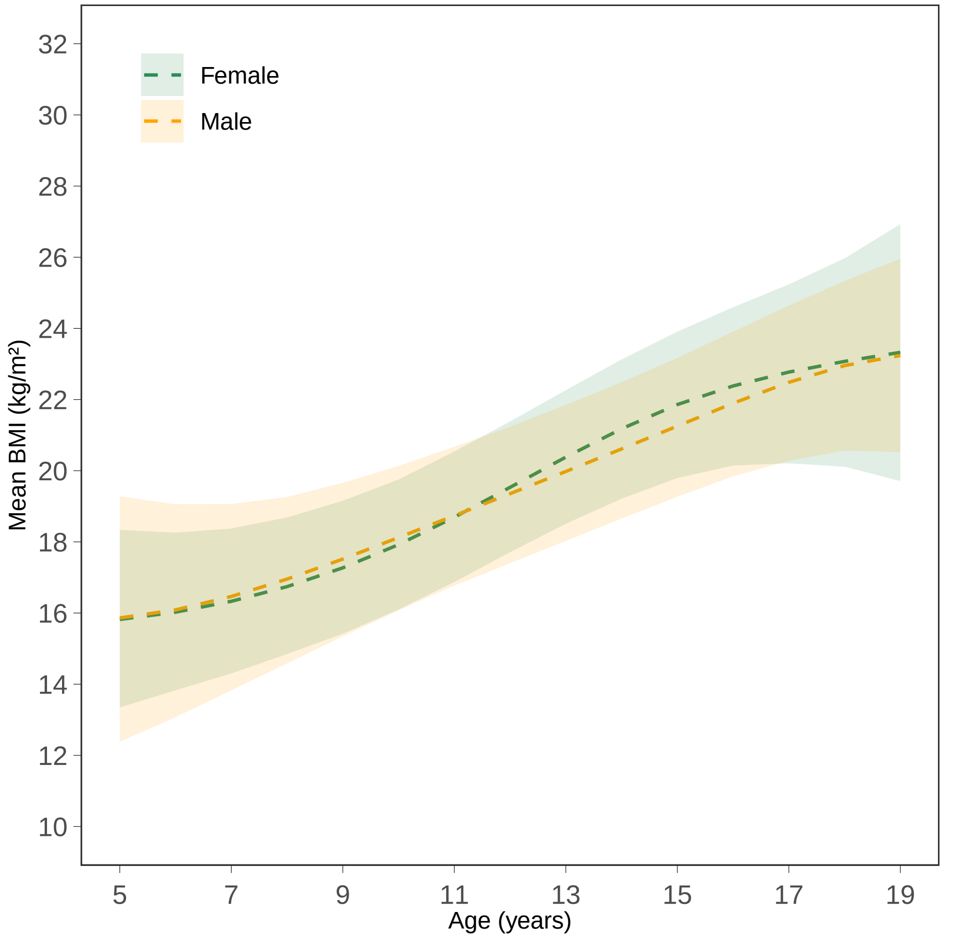

Guatemala

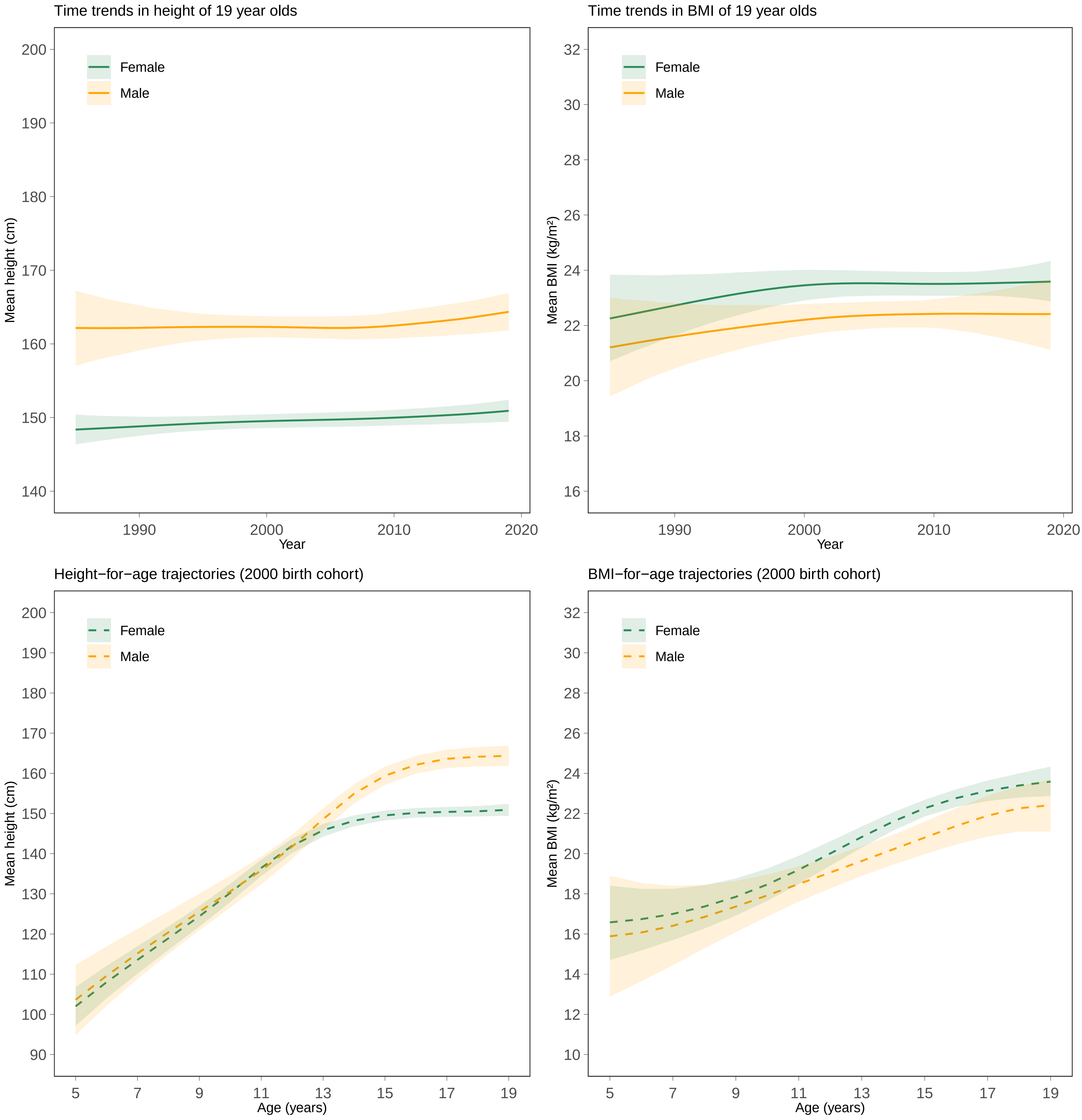

Time trends in height of 19 year olds
Female
Male
Mean height (cm)
Year
Time trends in BMI of 19 year olds
Mean BMI (kg/m²)
Height−for−age trajectories (2000 birth cohort)
Age (years)
BMI−for−age trajectories (2000 birth cohort)

Guinea Bissau

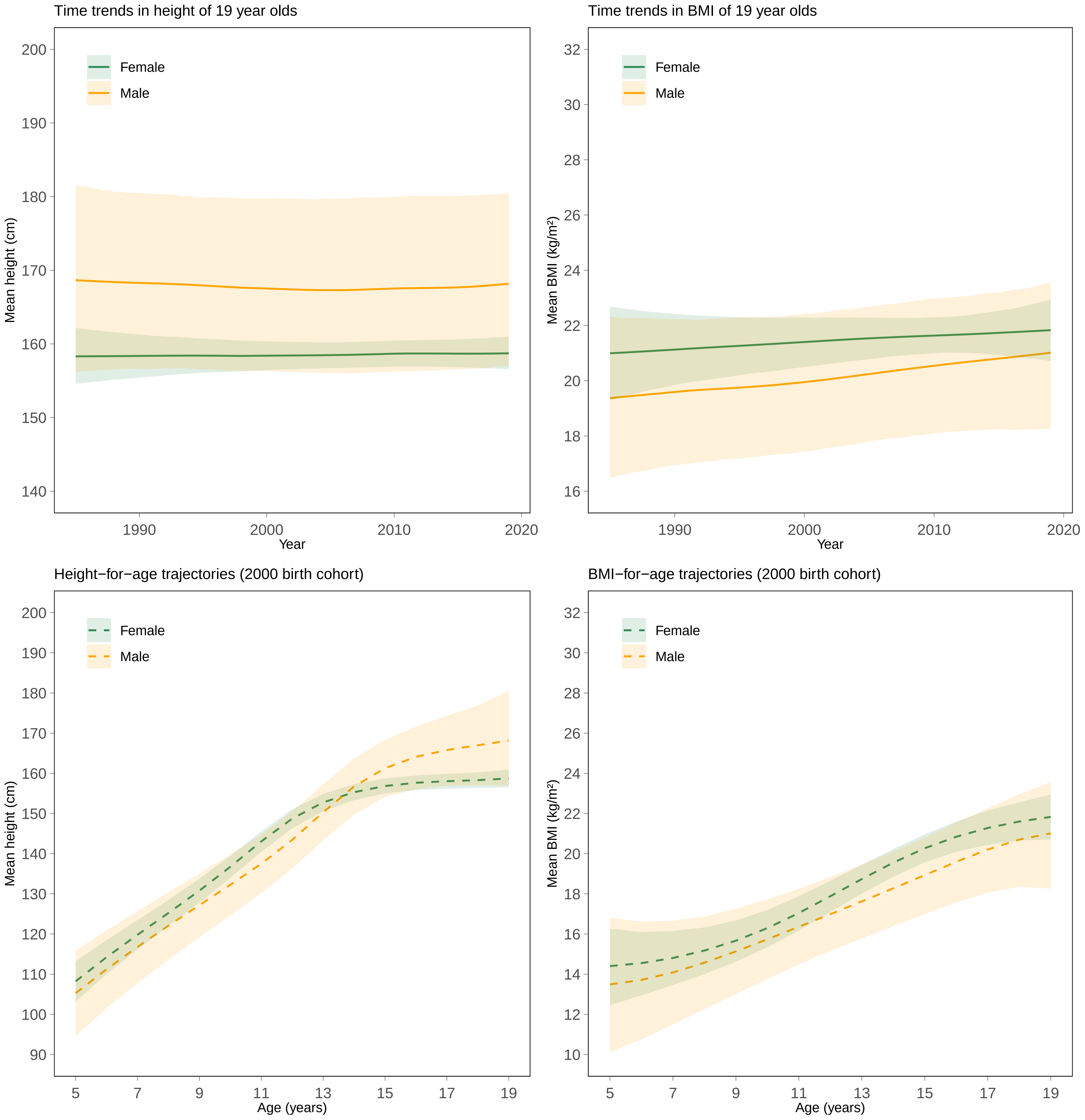

Guinea

Time trends in height of 19 year olds

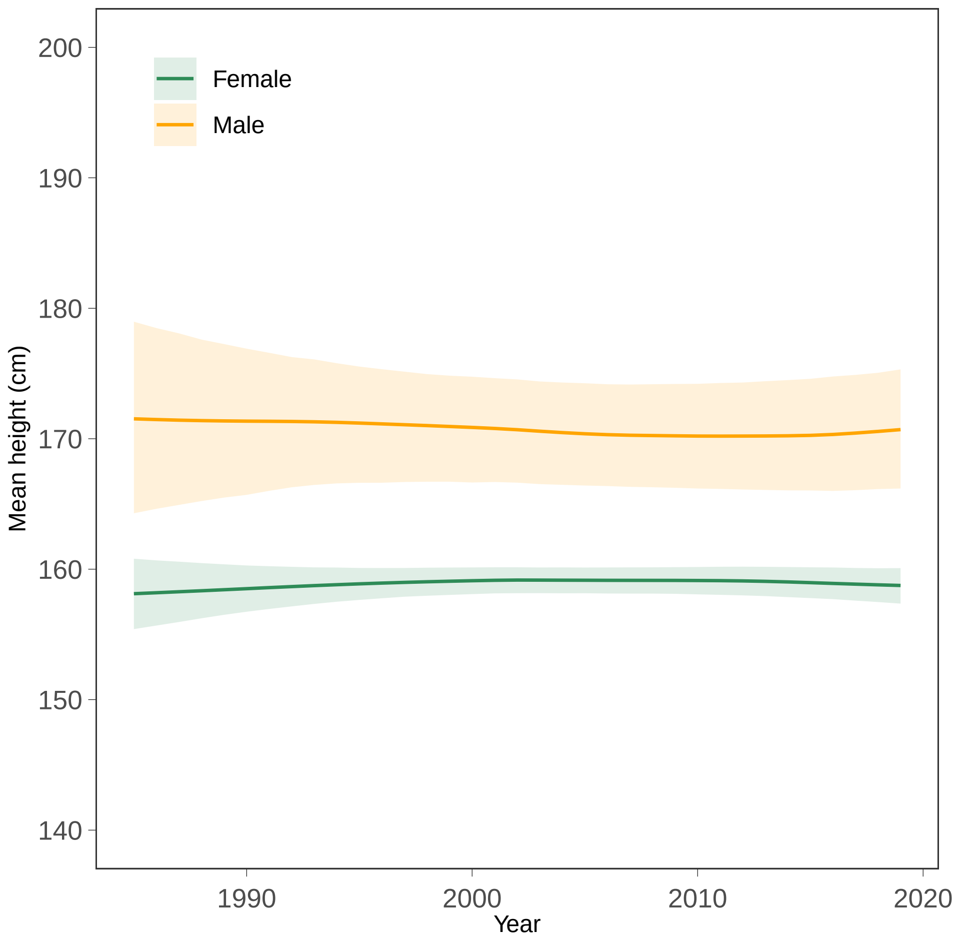

Time trends in BMI of 19 year olds

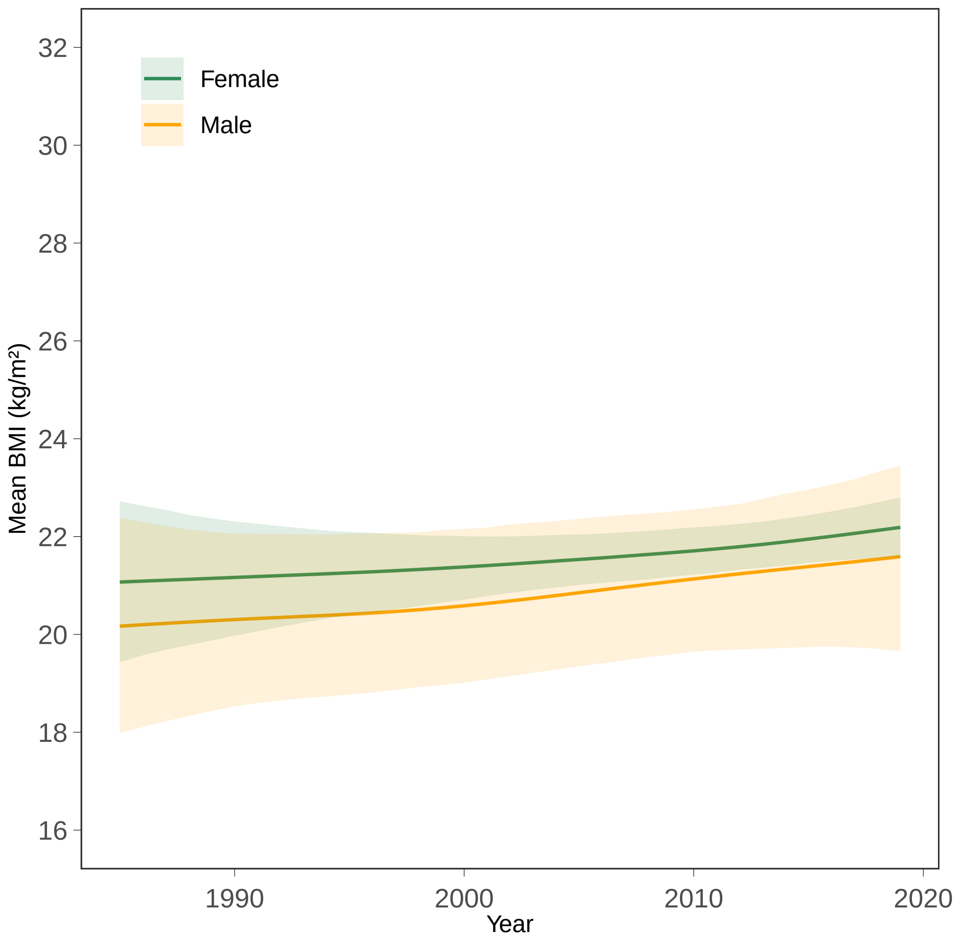

Height−for−age trajectories (2000 birth cohort)

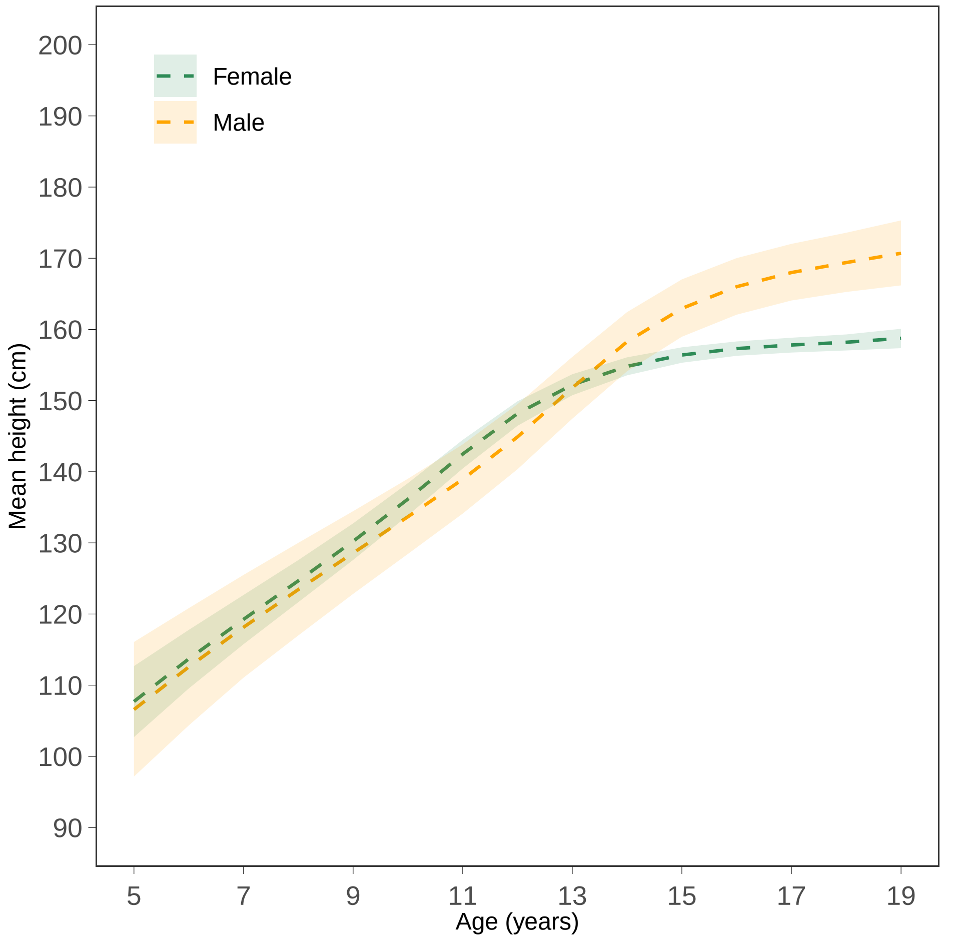

BMI−for−age trajectories (2000 birth cohort)

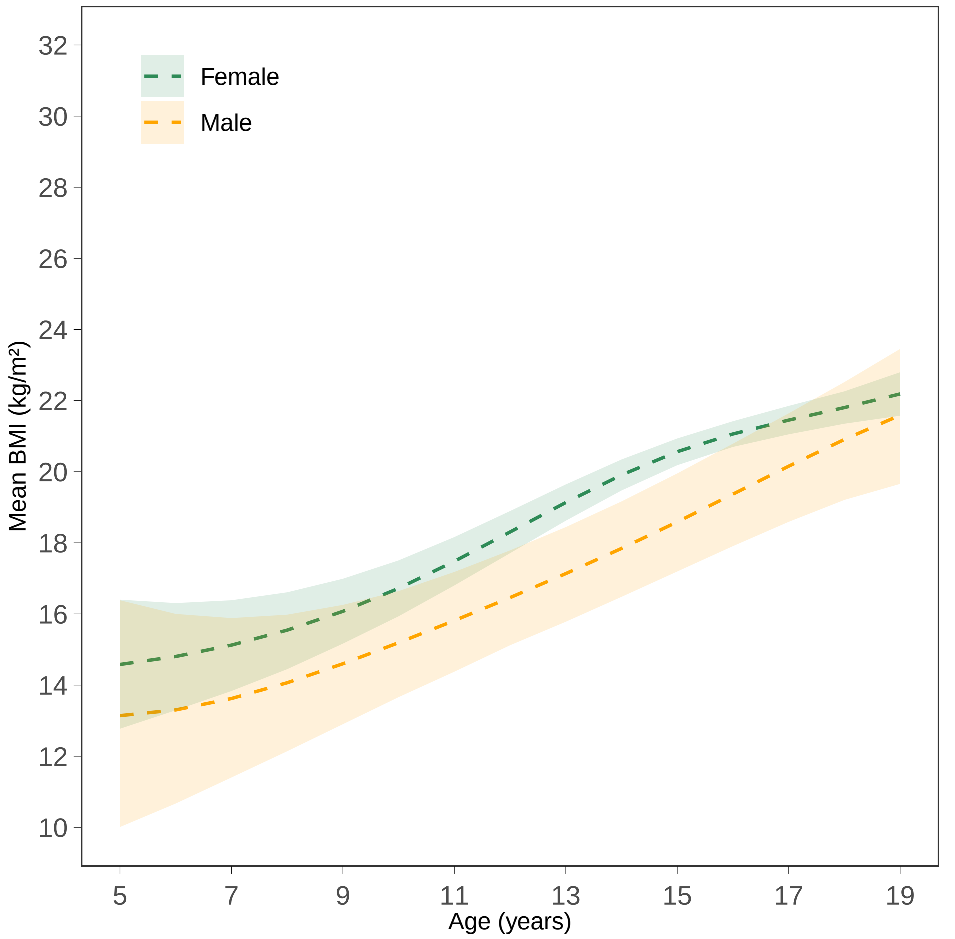

Guyana

Time trends in height of 19 year olds

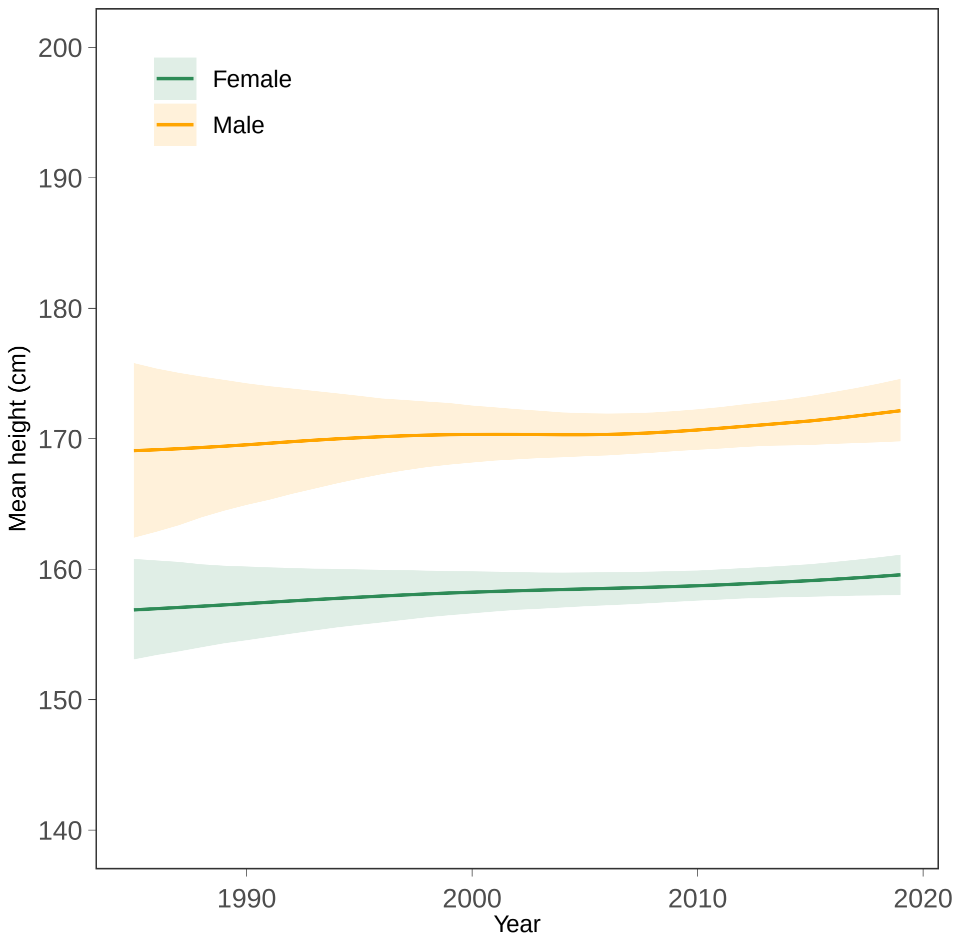

Time trends in BMI of 19 year olds

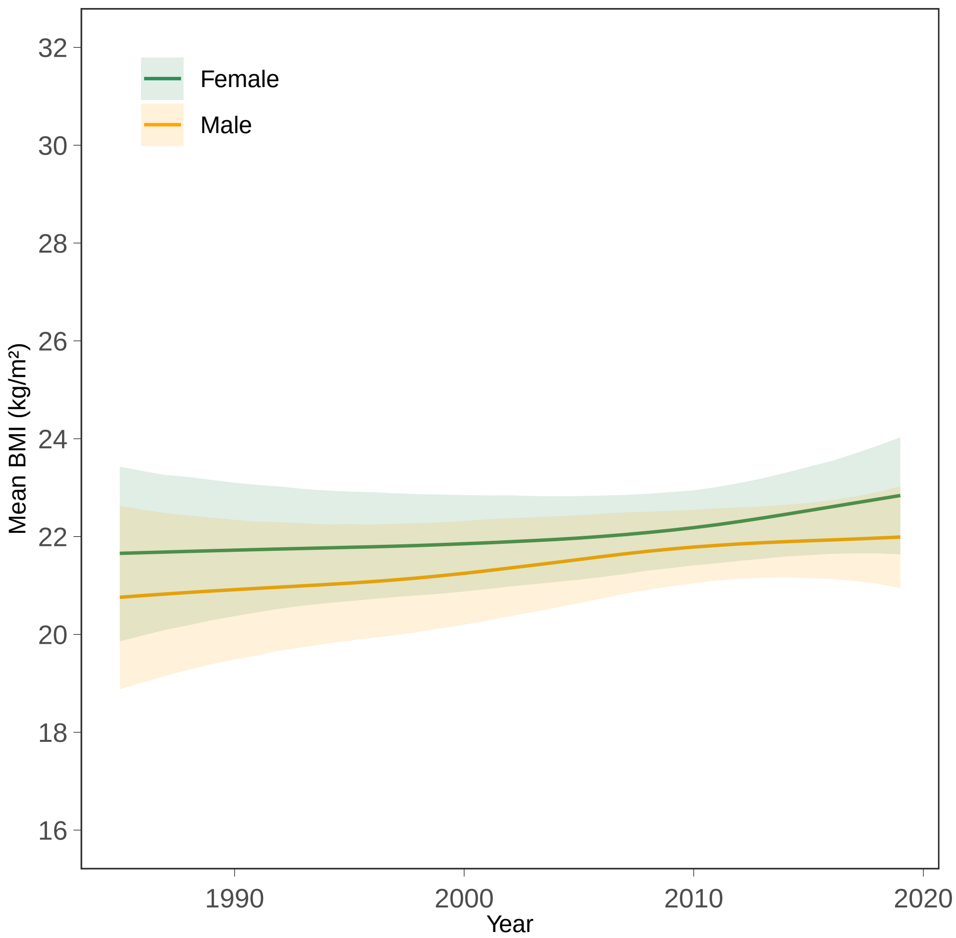

Height−for−age trajectories (2000 birth cohort)

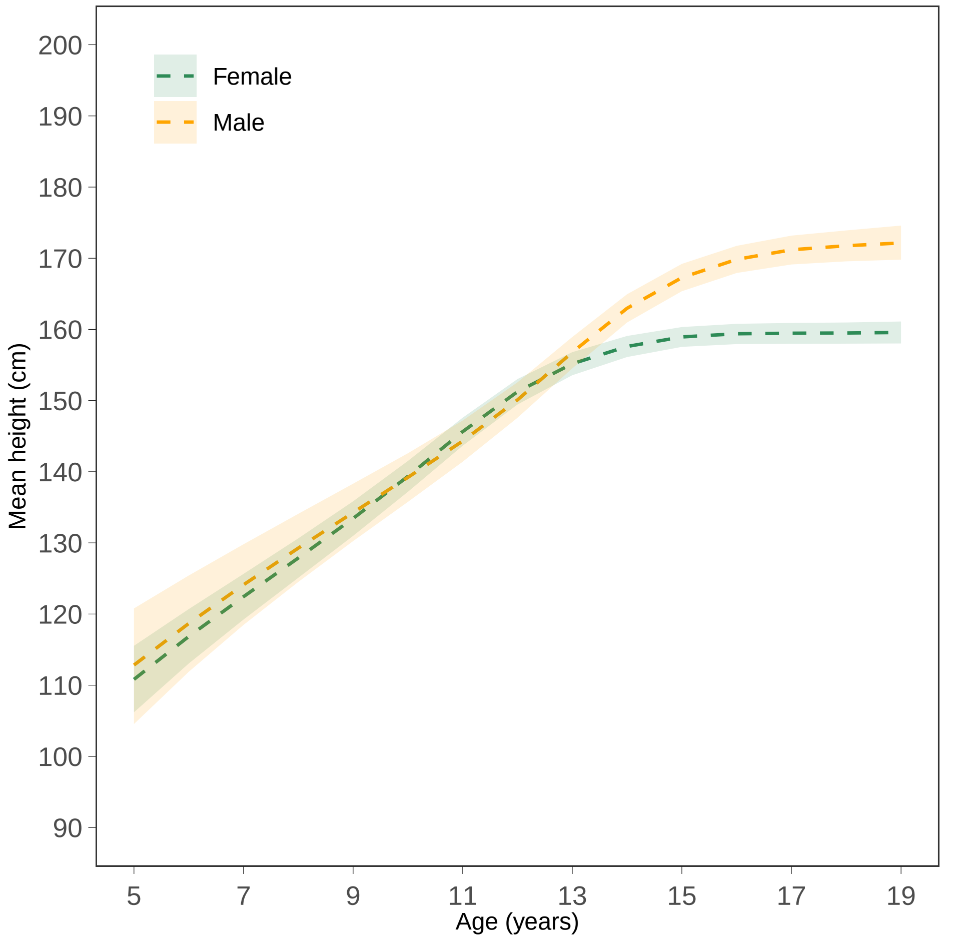

BMI−for−age trajectories (2000 birth cohort)

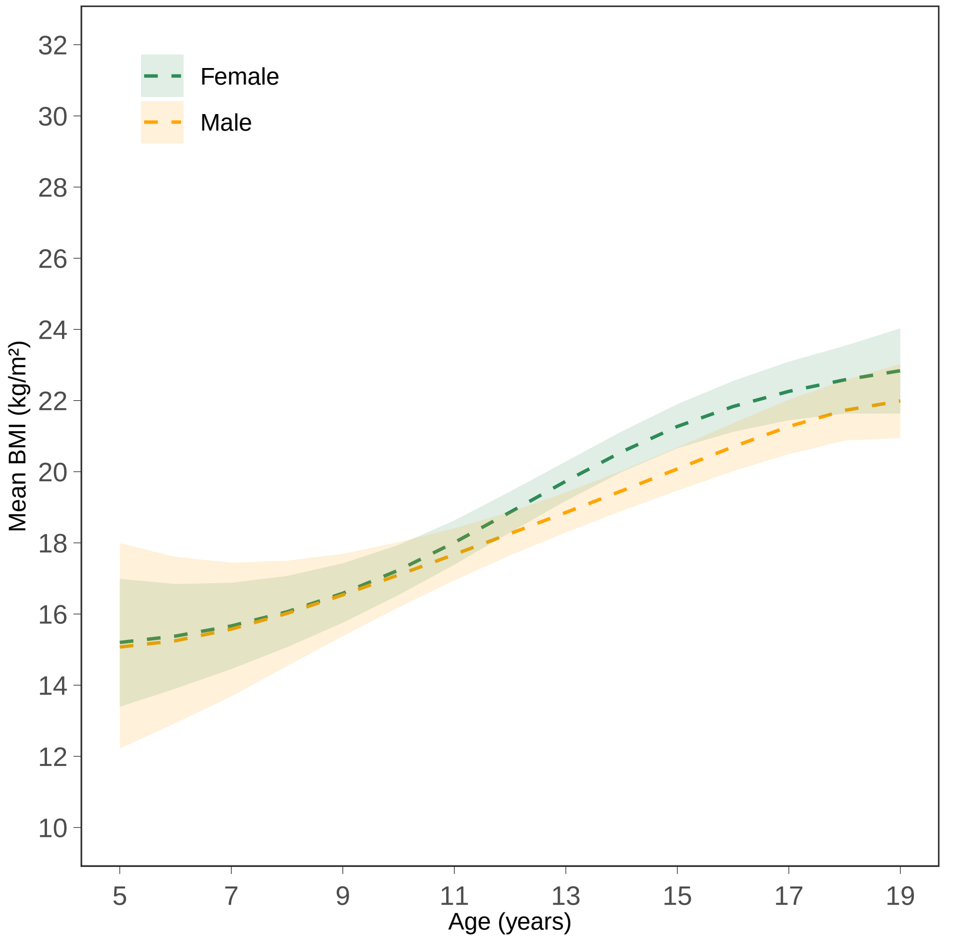

Haiti

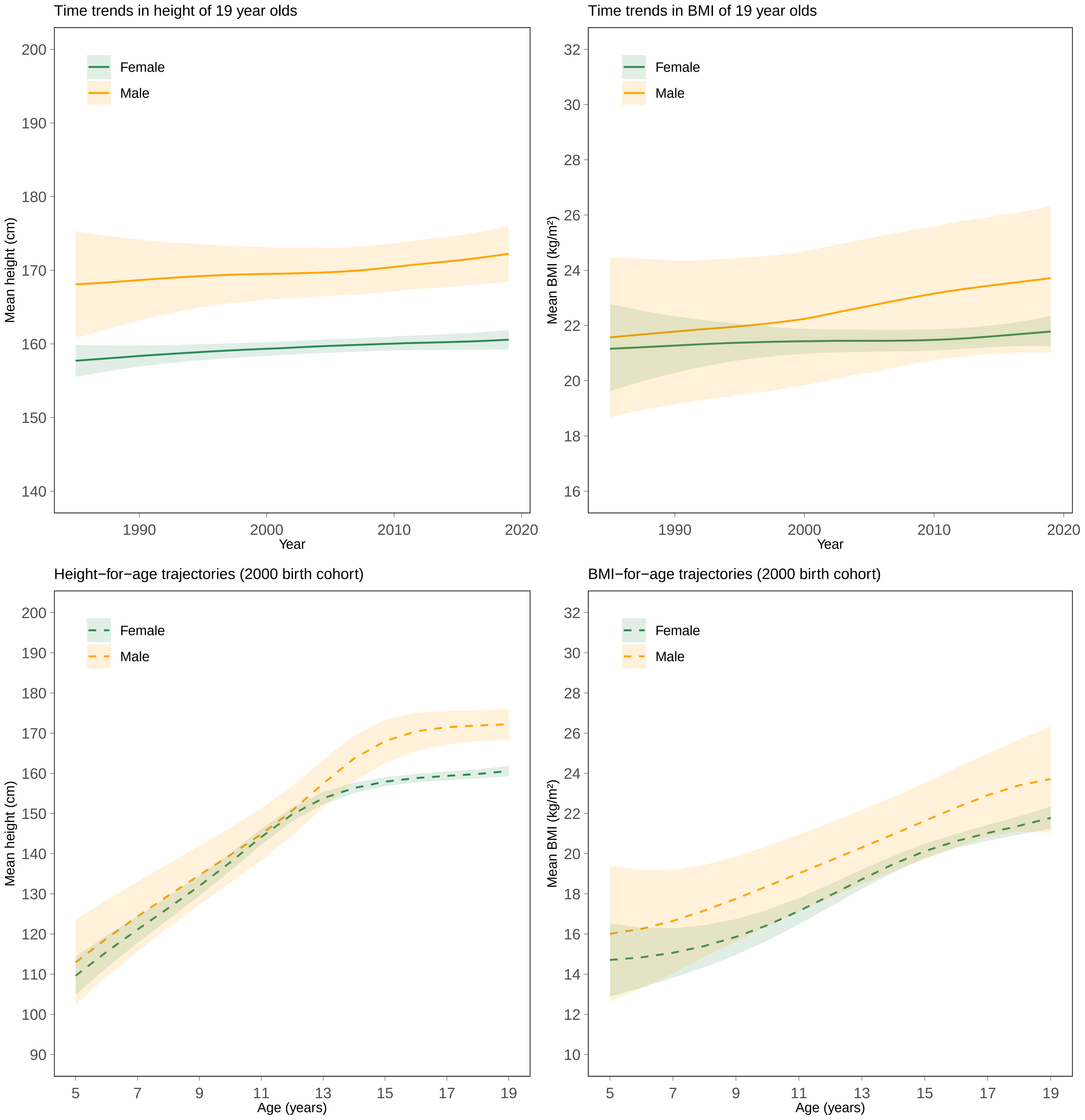

Time trends in height of 19 year olds
Female
Male
Mean height (cm)
200
190
180
170
160
150
140
1990
2000
2010
2020
Year
Time trends in BMI of 19 year olds
Female
Male
Mean BMI (kg/m²)
32
30
28
26
24
22
20
18
16
1990
2000
2010
2020
Year
Height−for−age trajectories (2000 birth cohort)
Female
Male
Mean height (cm)
200
190
180
170
160
150
140
130
120
110
100
90
5
7
9
11
13
15
17
19
Age (years)
BMI−for−age trajectories (2000 birth cohort)
Female
Male
Mean BMI (kg/m²)
32
30
28
26
24
22
20
18
16
14
12
10
5
7
9
11
13
15
17
19
Age (years)

Honduras

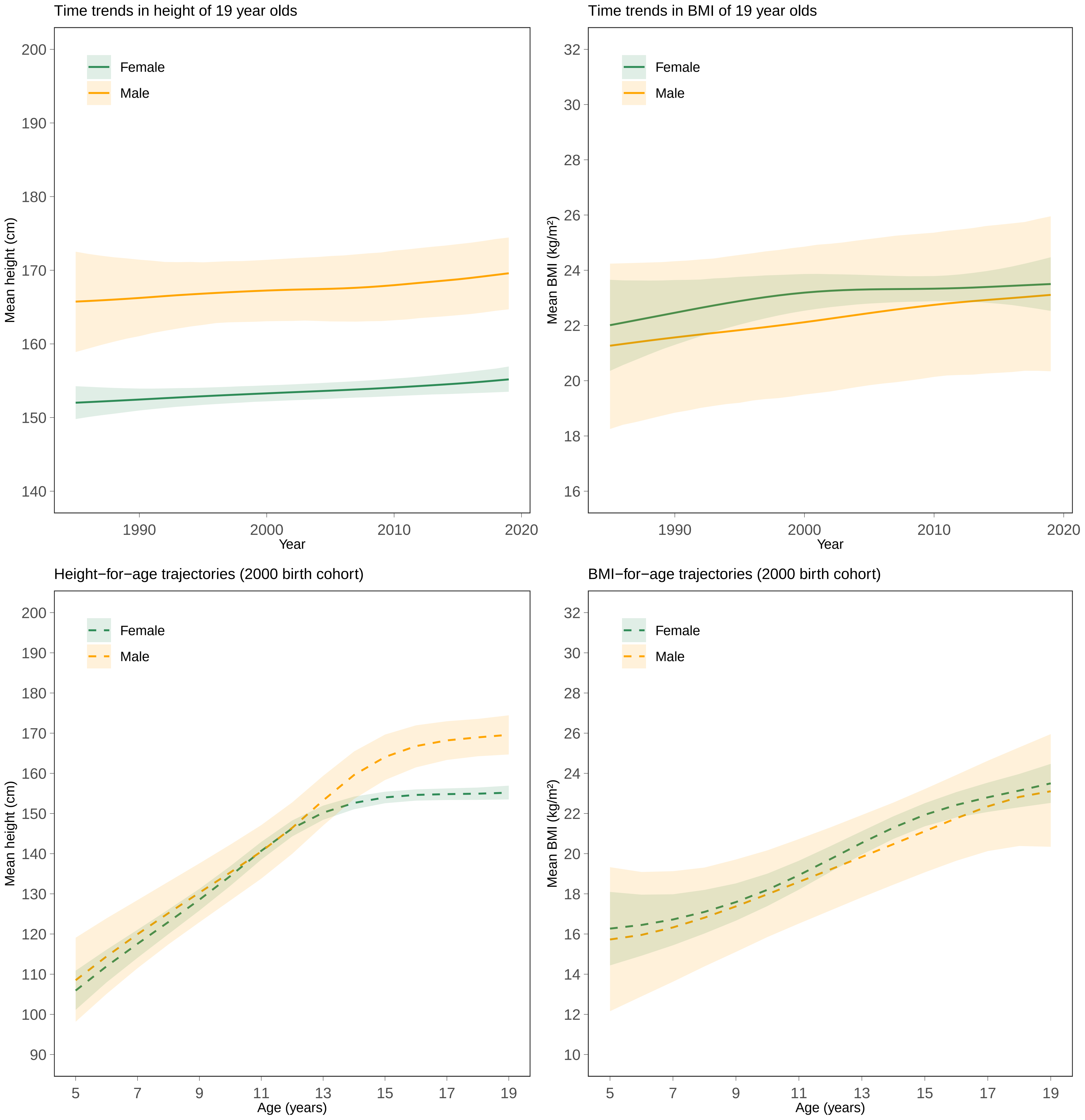

Time trends in height of 19 year olds
Female
Male
Mean height (cm)
200
190
180
170
160
150
140
1990
2000
2010
2020
Year
Time trends in BMI of 19 year olds
Female
Male
Mean BMI (kg/m²)
32
30
28
26
24
22
20
18
16
1990
2000
2010
2020
Year
Height−for−age trajectories (2000 birth cohort)
Female
Male
Mean height (cm)
200
190
180
170
160
150
140
130
120
110
100
90
5
7
9
11
13
15
17
19
Age (years)
BMI−for−age trajectories (2000 birth cohort)
Female
Male
Mean BMI (kg/m²)
32
30
28
26
24
22
20
18
16
14
12
10
5
7
9
11
13
15
17
19
Age (years)

Hungary

Time trends in height of 19 year olds

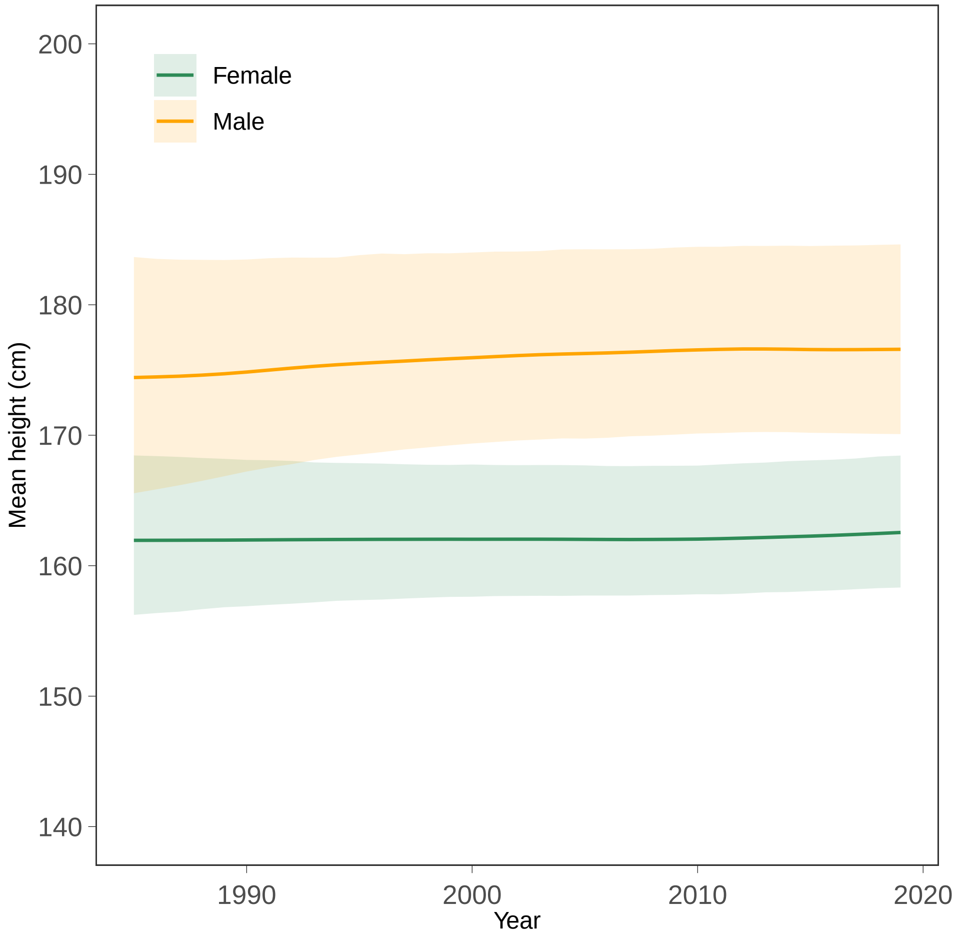

Time trends in BMI of 19 year olds

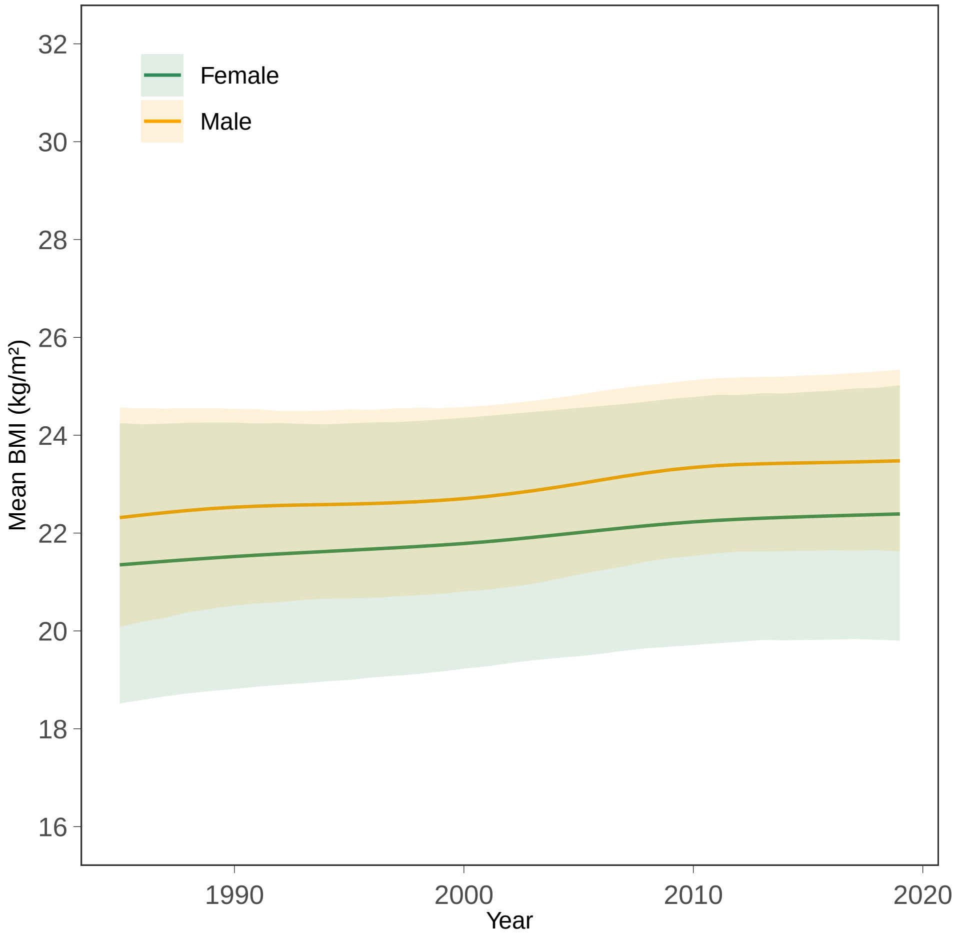

Height−for−age trajectories (2000 birth cohort)

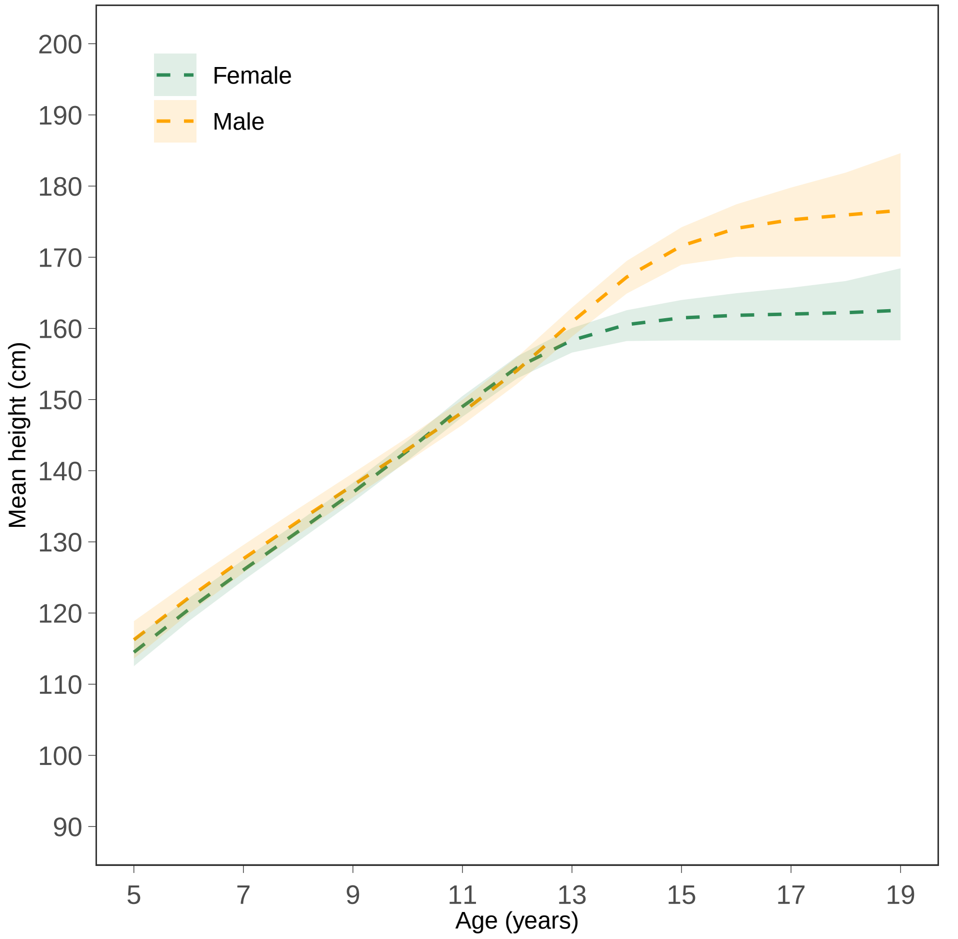

BMI−for−age trajectories (2000 birth cohort)

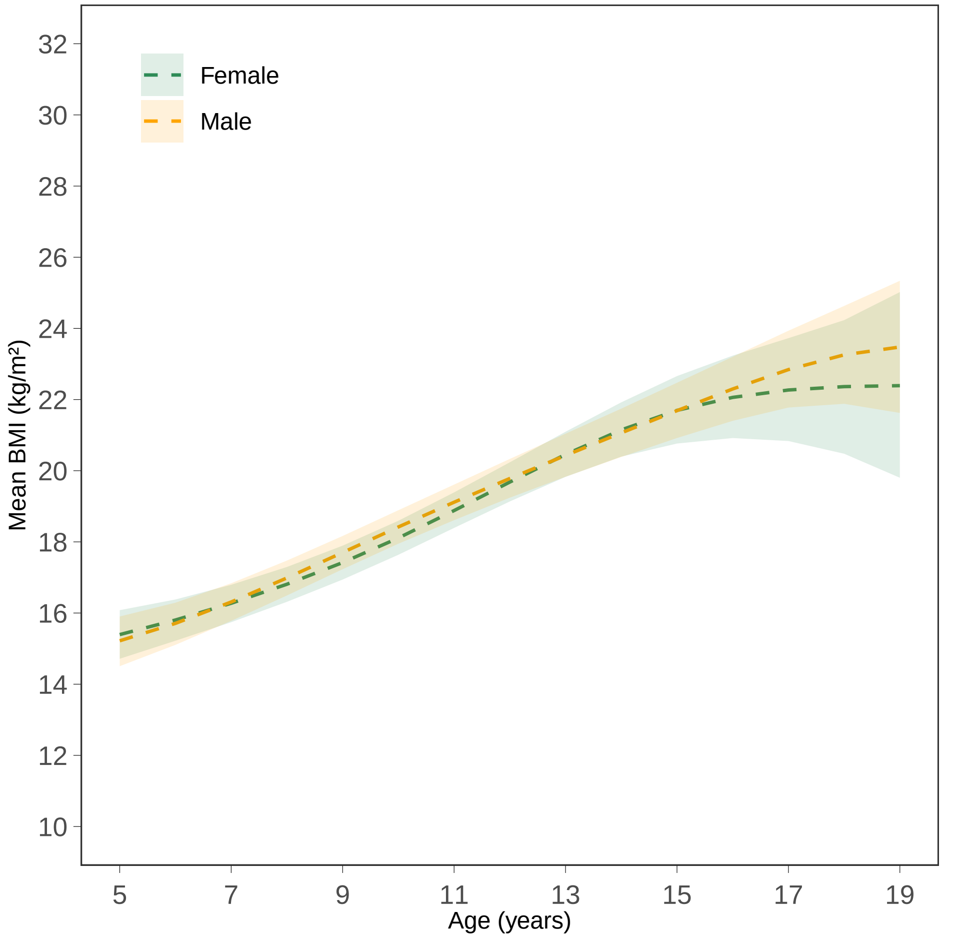

Iceland

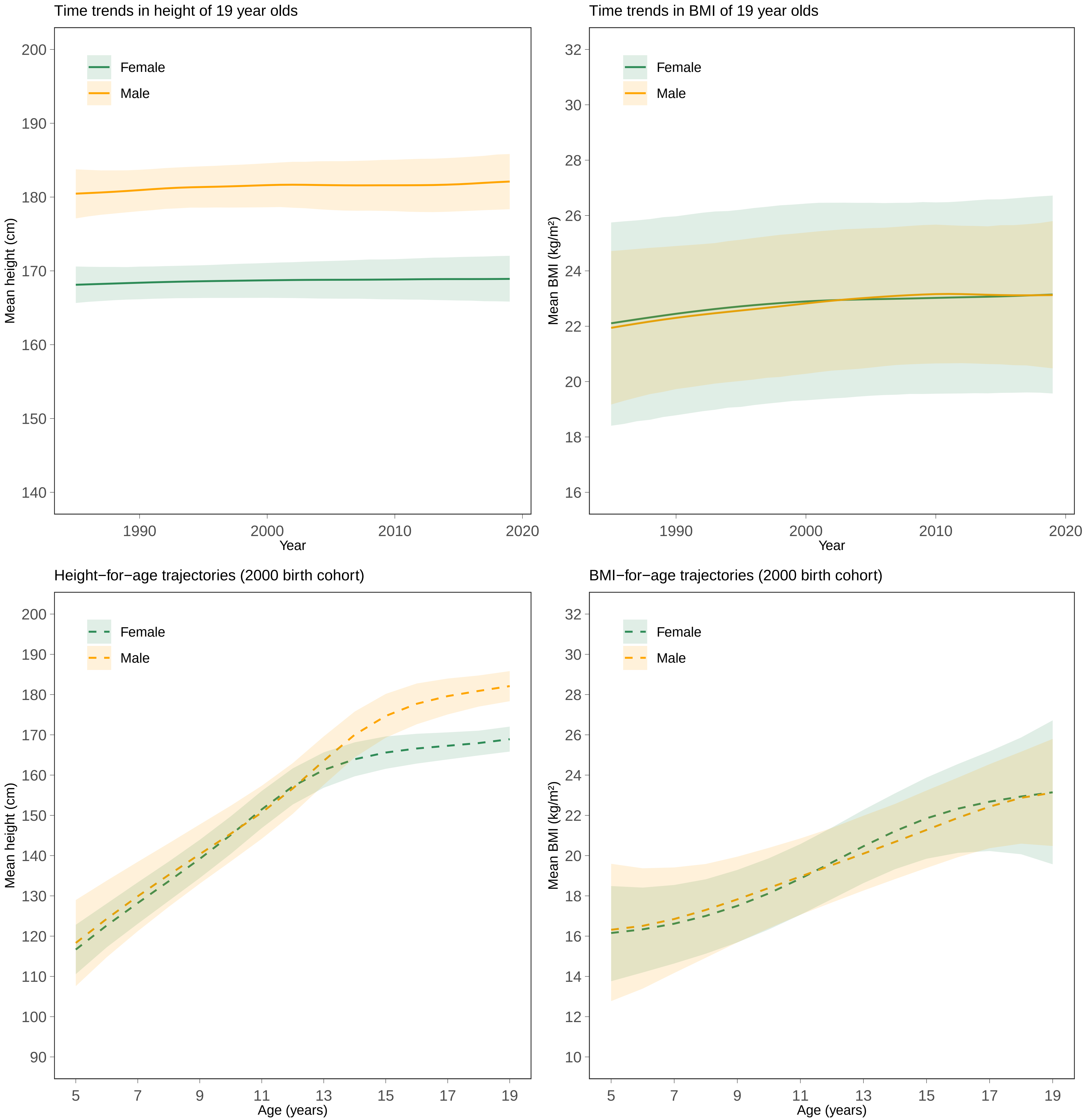

Time trends in height of 19 year olds
Female
Male
Mean height (cm)
200
190
180
170
160
150
140
1990
2000
2010
2020
Year
Time trends in BMI of 19 year olds
Female
Male
Mean BMI (kg/m²)
32
30
28
26
24
22
20
18
16
1990
2000
2010
2020
Year
Height−for−age trajectories (2000 birth cohort)
Female
Male
Mean height (cm)
200
190
180
170
160
150
140
130
120
110
100
90
5
7
9
11
13
15
17
19
Age (years)
BMI−for−age trajectories (2000 birth cohort)
Female
Male
Mean BMI (kg/m²)
32
30
28
26
24
22
20
18
16
14
12
10
5
7
9
11
13
15
17
19
Age (years)

India

Time trends in height of 19 year olds

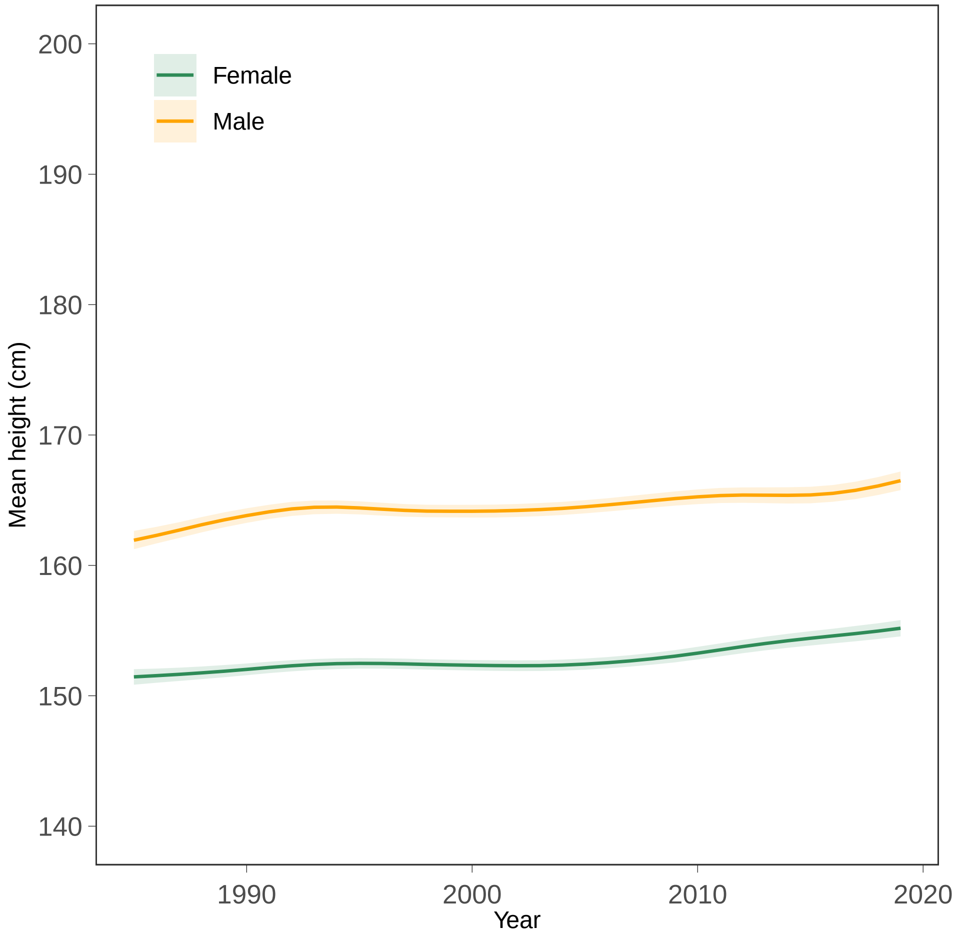

Time trends in BMI of 19 year olds

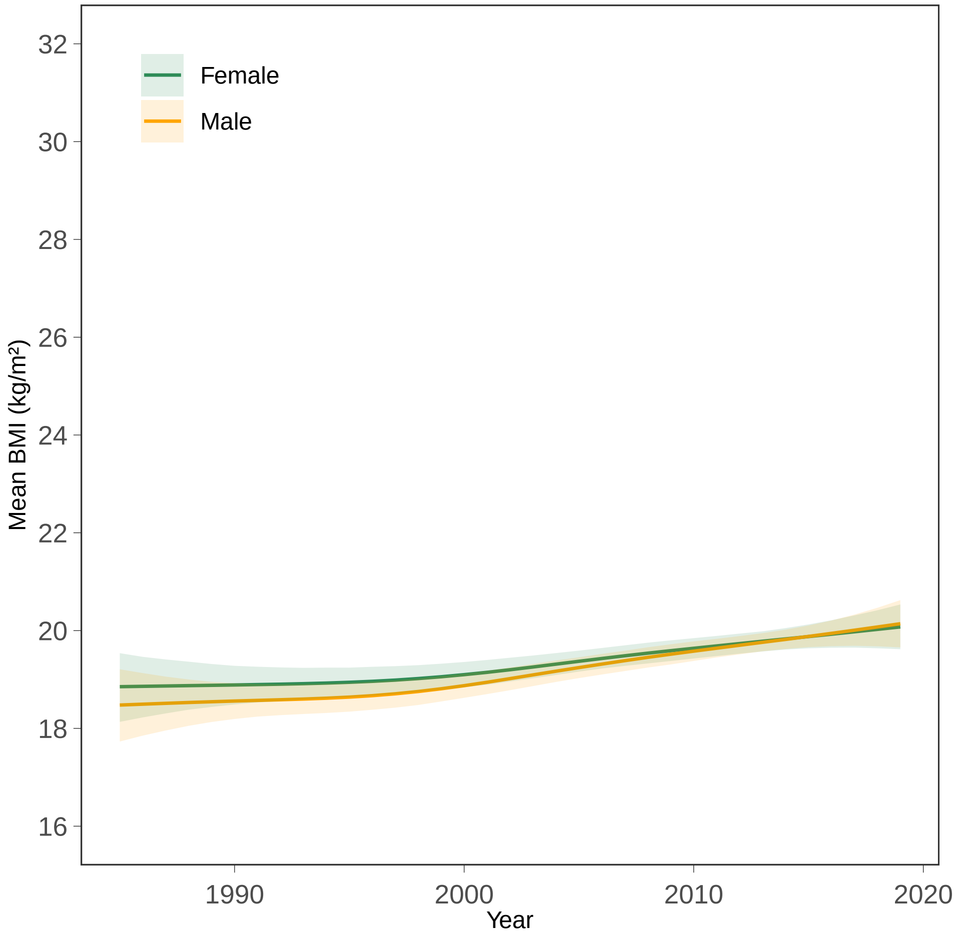

Height−for−age trajectories (2000 birth cohort)

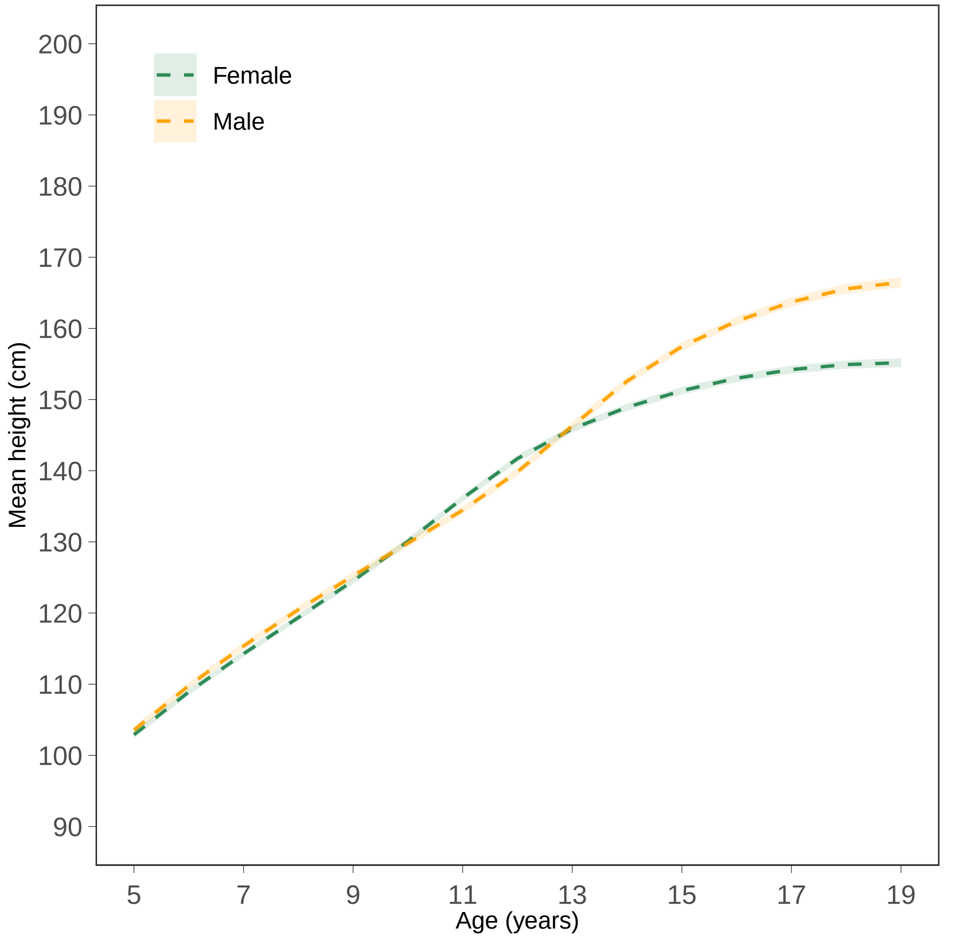

BMI−for−age trajectories (2000 birth cohort)

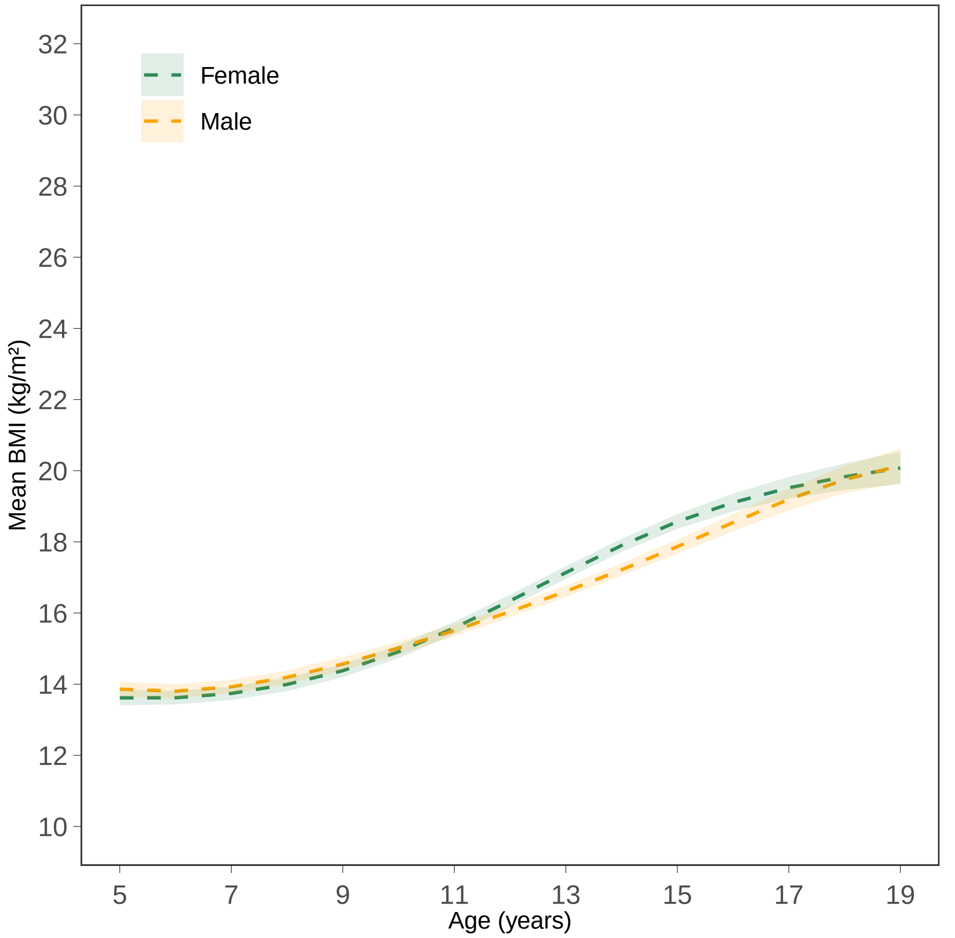

Indonesia

Time trends in height of 19 year olds

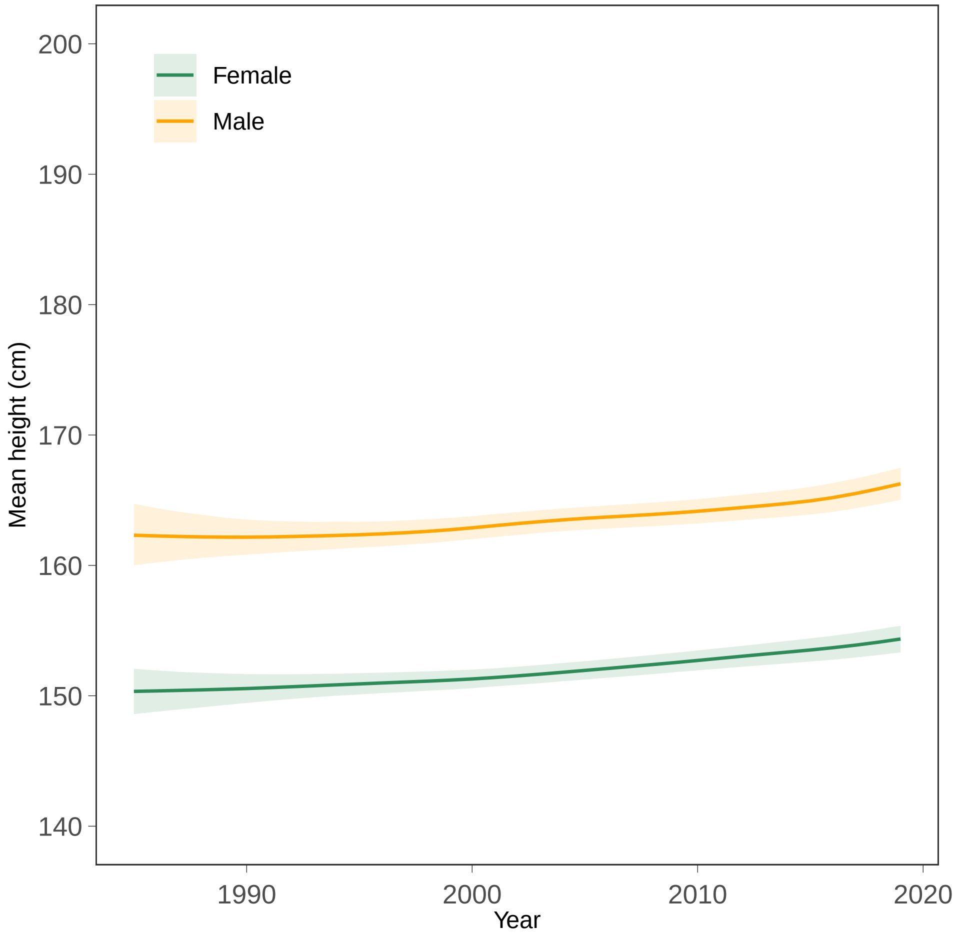

Time trends in BMI of 19 year olds

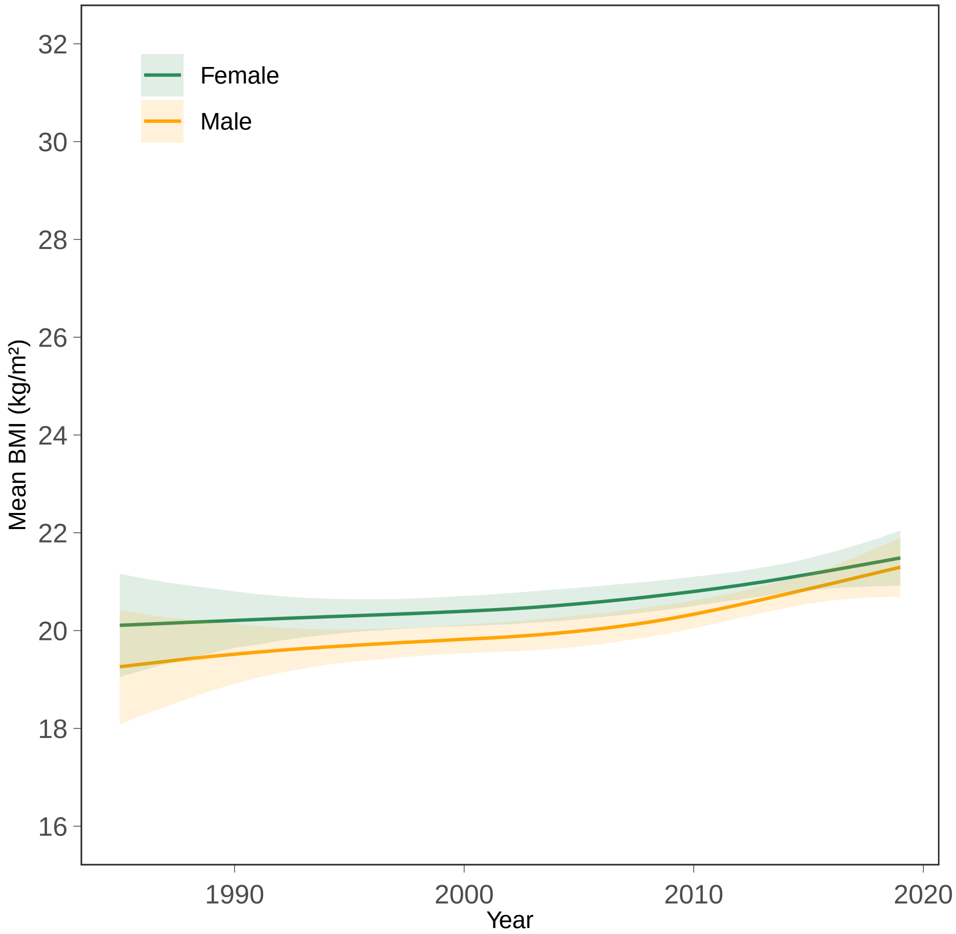

Height−for−age trajectories (2000 birth cohort)

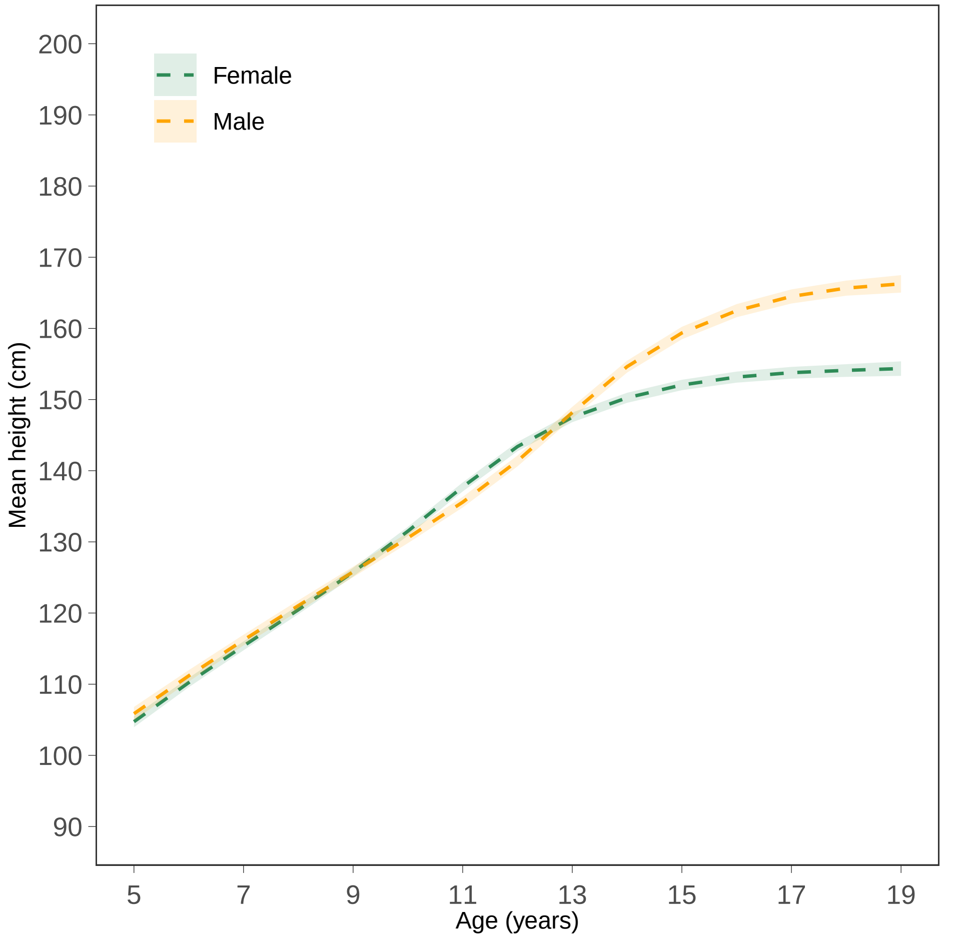

BMI−for−age trajectories (2000 birth cohort)

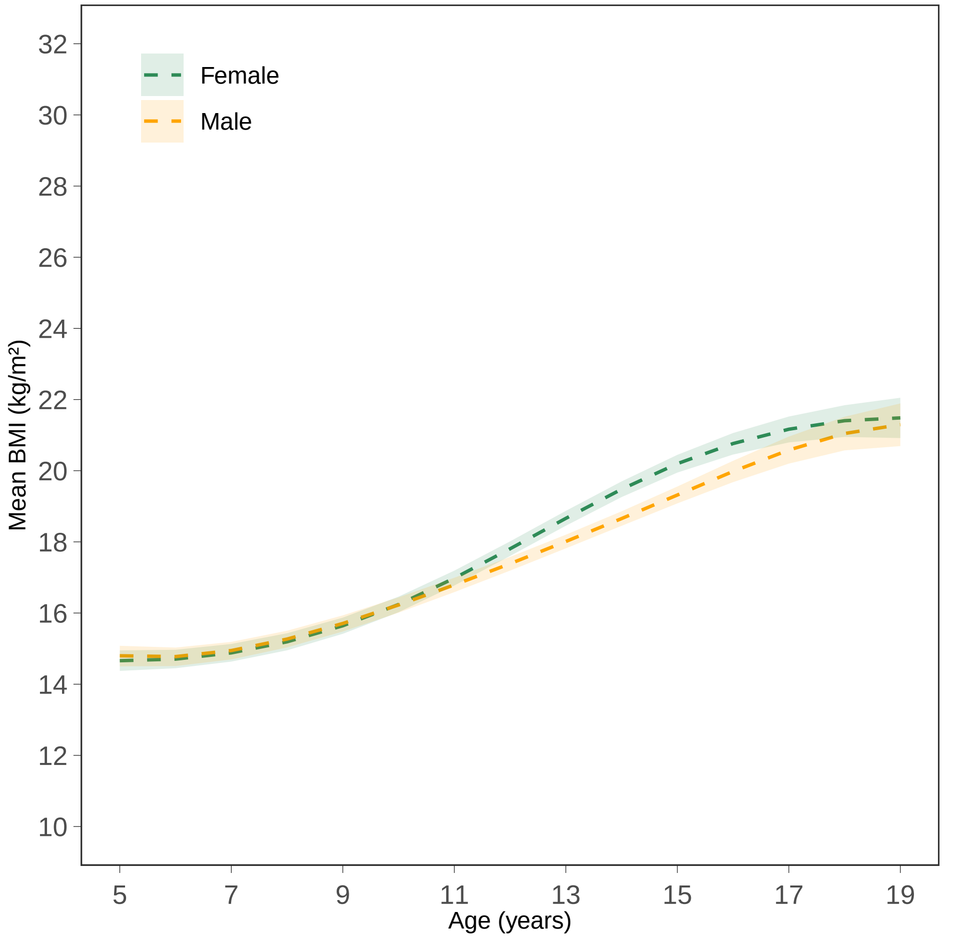

Iran

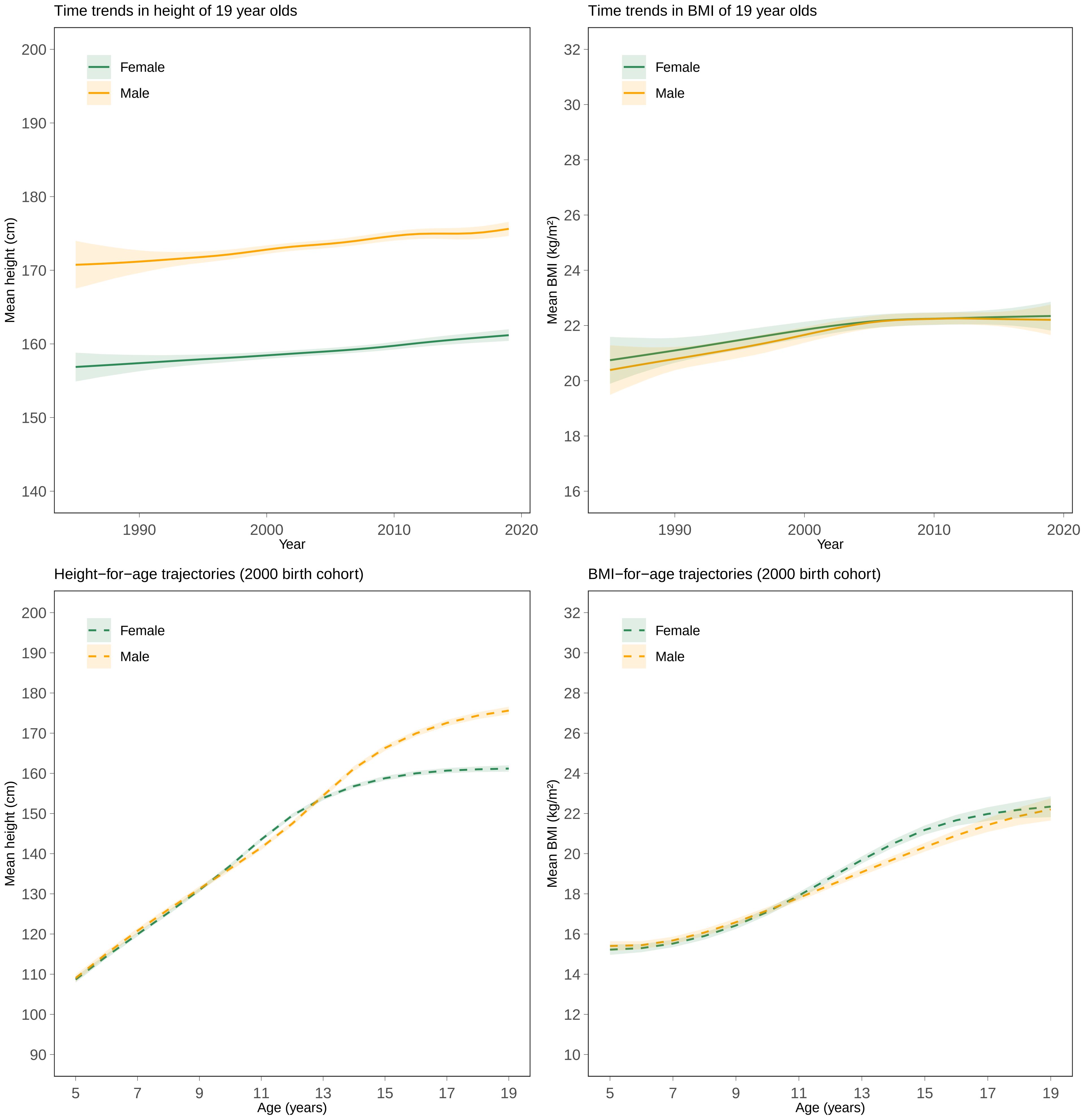

Time trends in height of 19 year olds
Mean height (cm)
Year
Female
Male
Time trends in BMI of 19 year olds
Mean BMI (kg/m²)
Year
Female
Male
Height−for−age trajectories (2000 birth cohort)
Mean height (cm)
Age (years)
Female
Male
BMI−for−age trajectories (2000 birth cohort)
Mean BMI (kg/m²)
Age (years)
Female
Male

Iraq

Time trends in height of 19 year olds

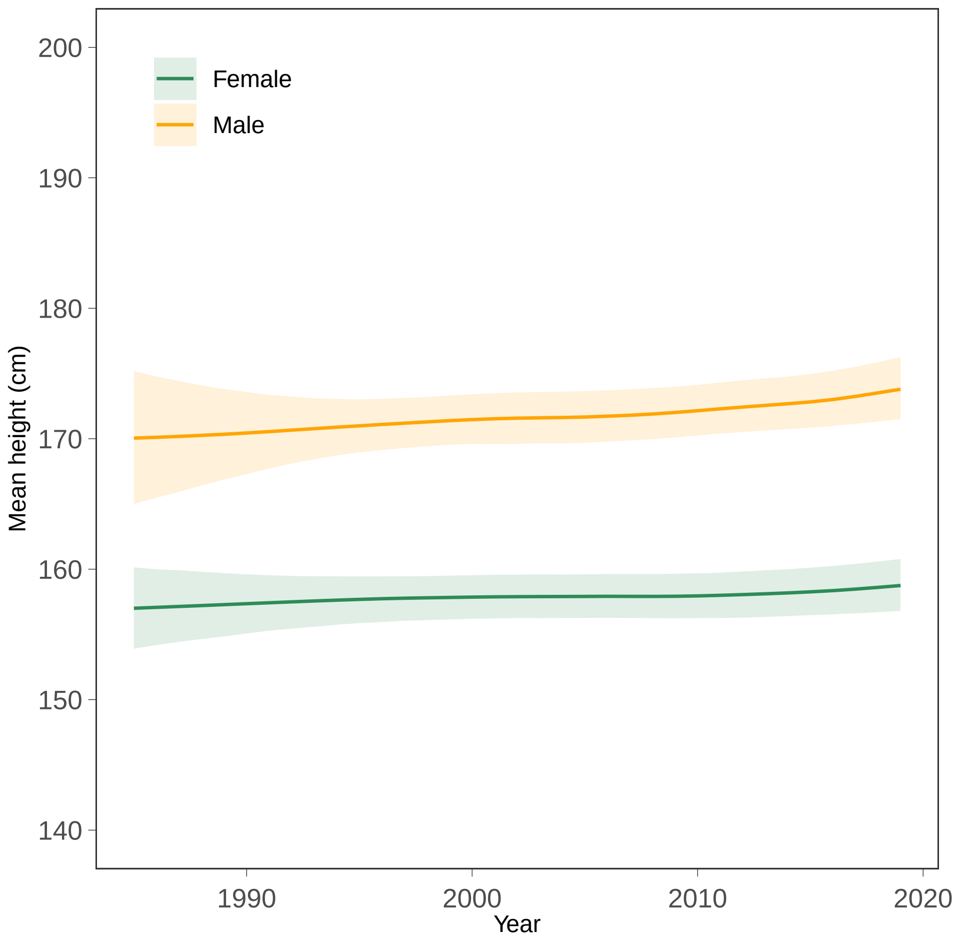

Time trends in BMI of 19 year olds

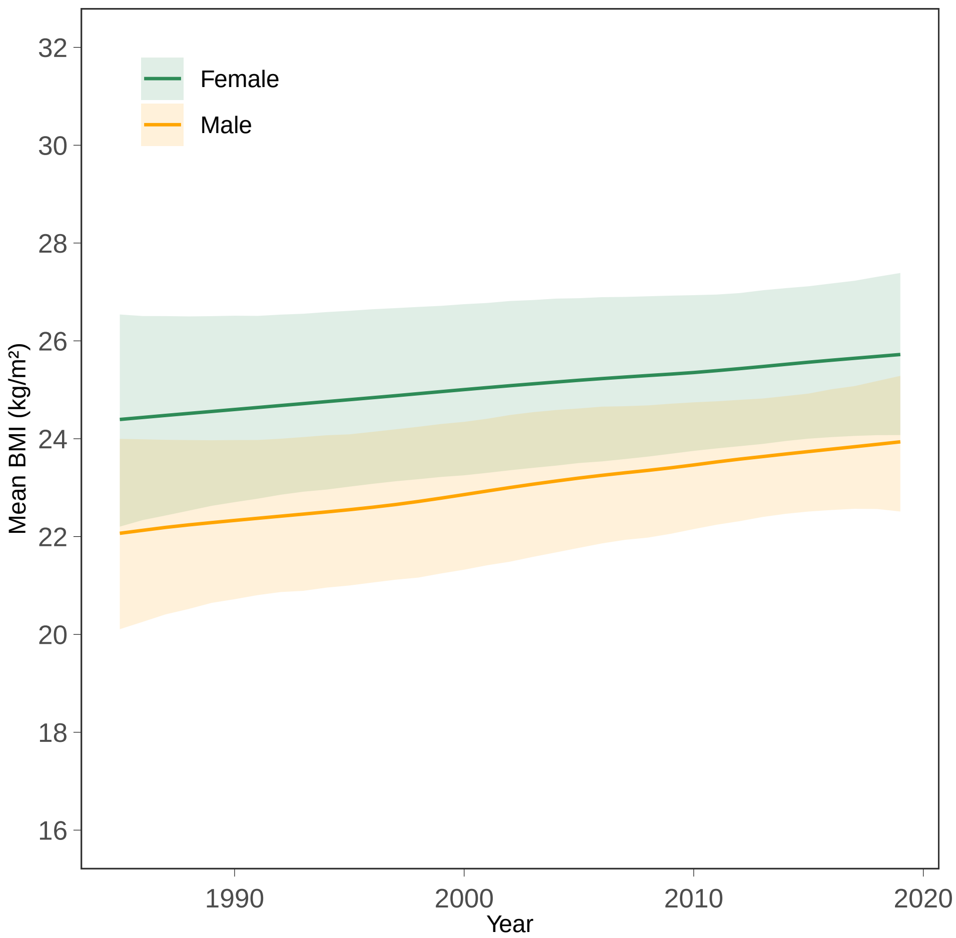

Height−for−age trajectories (2000 birth cohort)

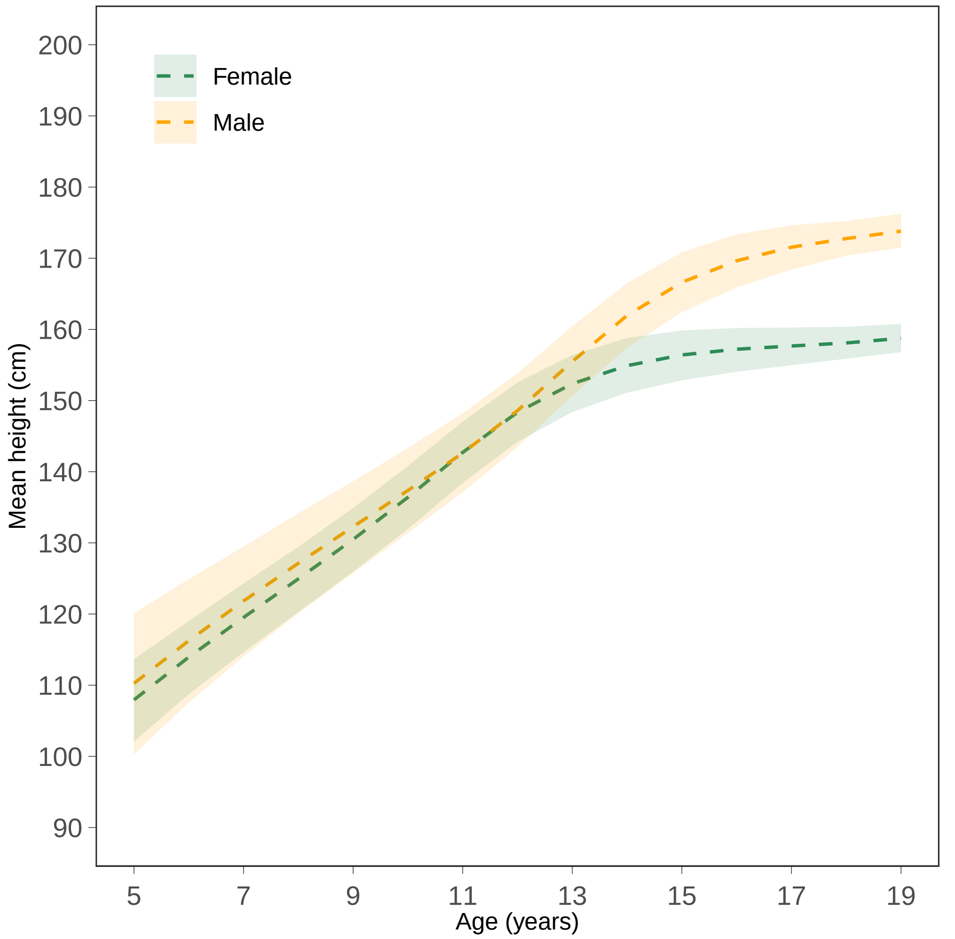

BMI−for−age trajectories (2000 birth cohort)

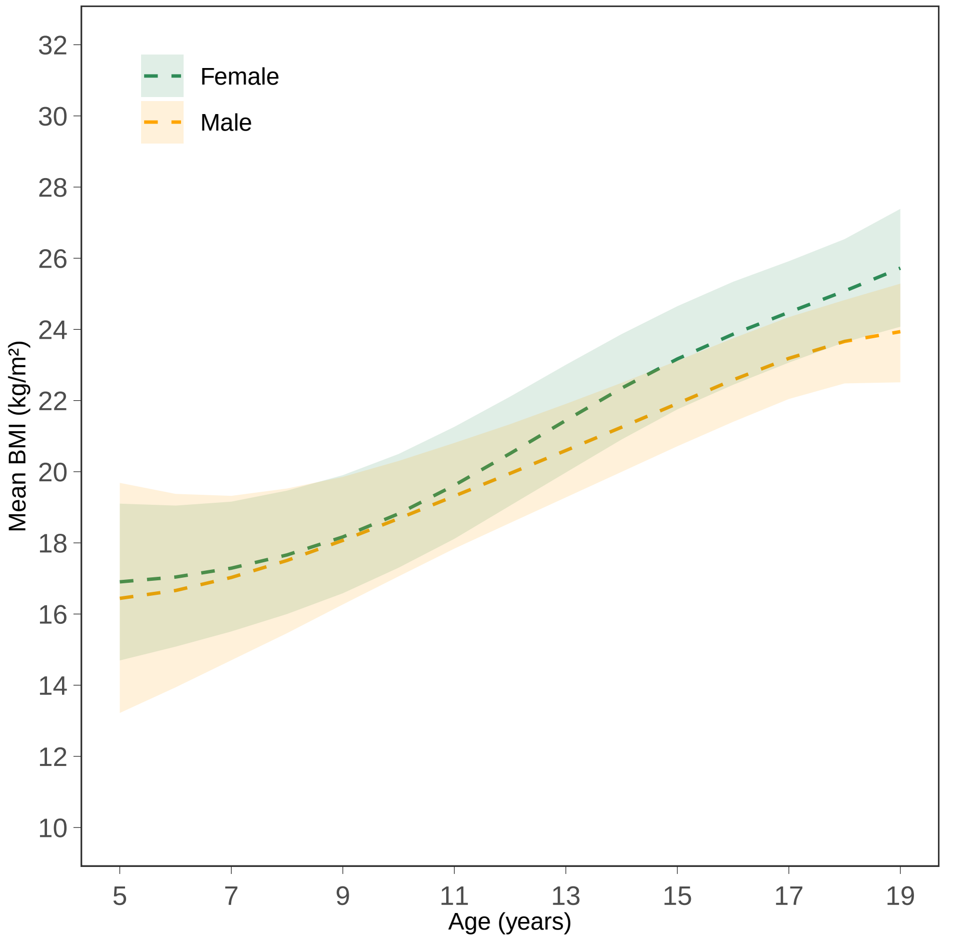

Ireland

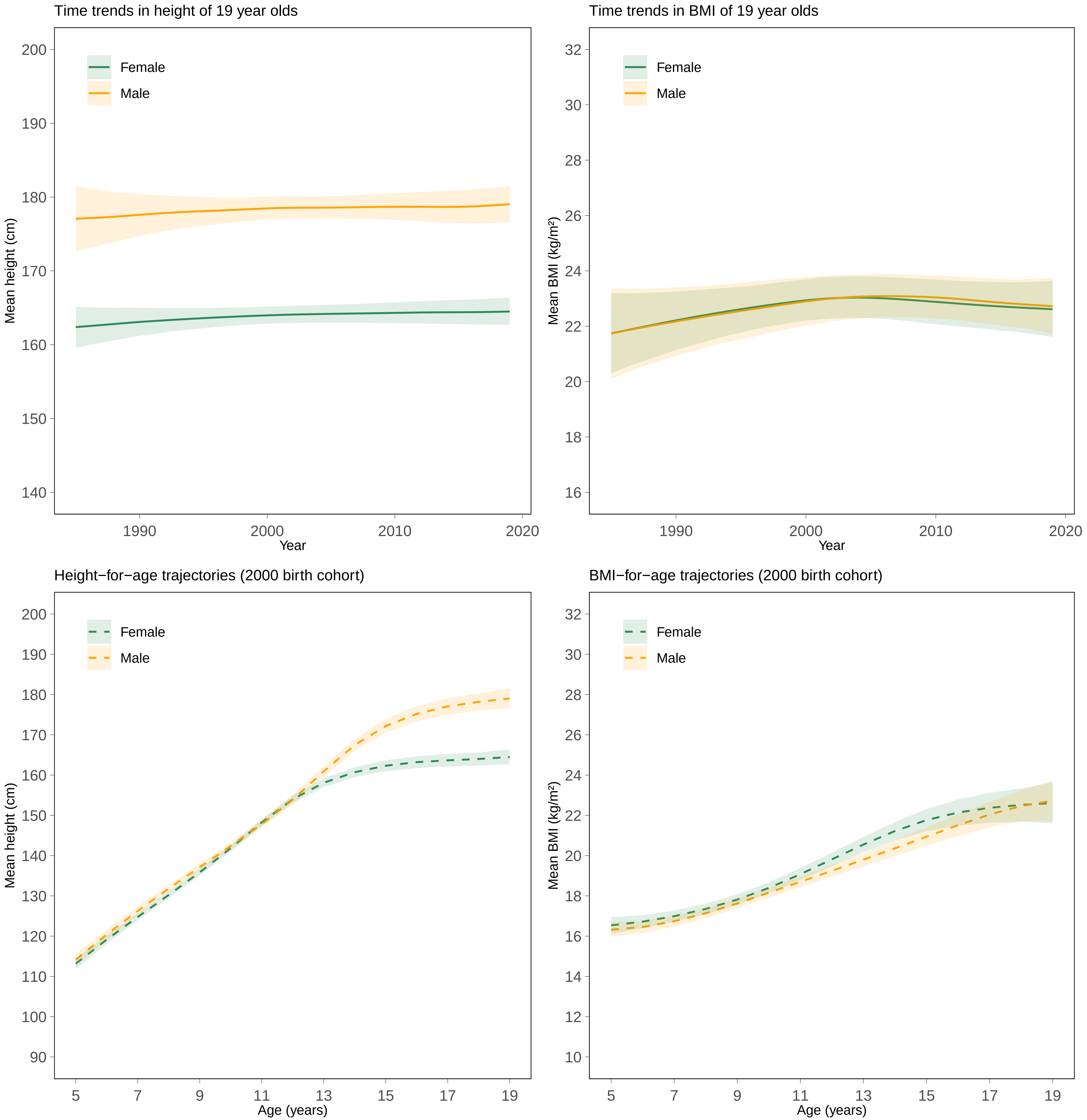

Time trends in height of 19 year olds
Female
Male
Mean height (cm)
Year
Time trends in BMI of 19 year olds
Mean BMI (kg/m²)
Height−for−age trajectories (2000 birth cohort)
Age (years)
BMI−for−age trajectories (2000 birth cohort)

Israel

Time trends in height of 19 year olds

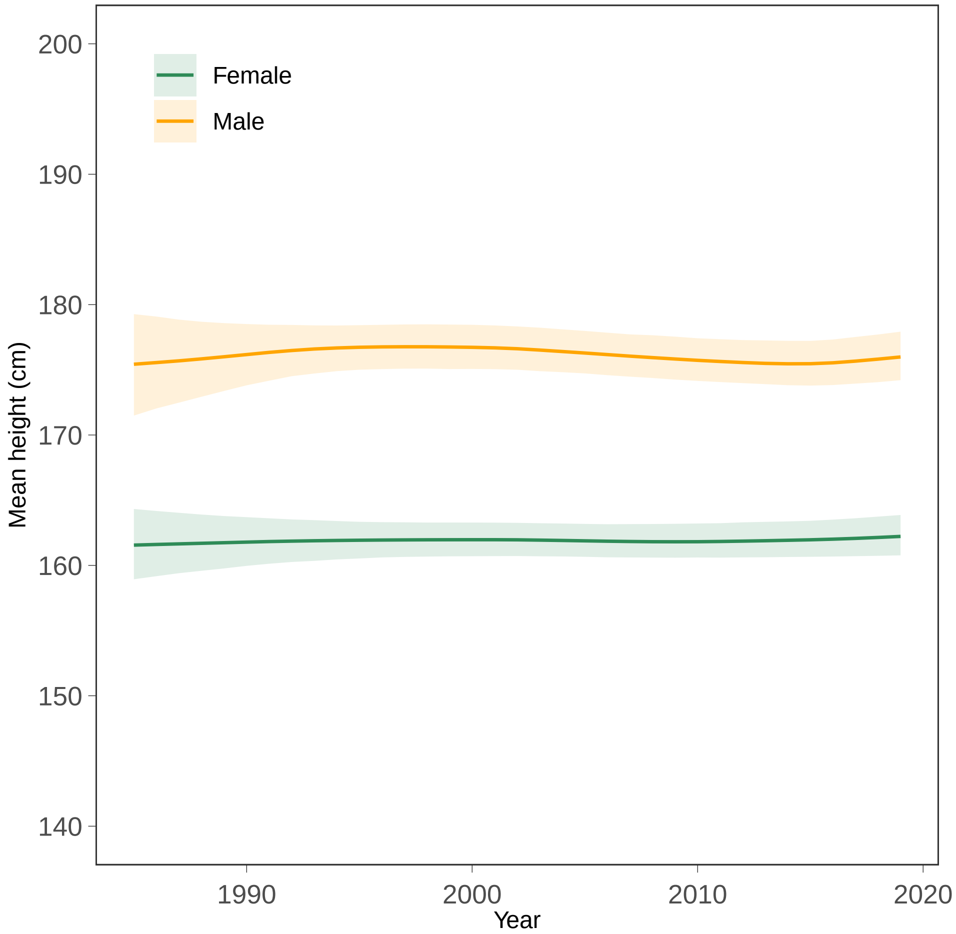

Time trends in BMI of 19 year olds

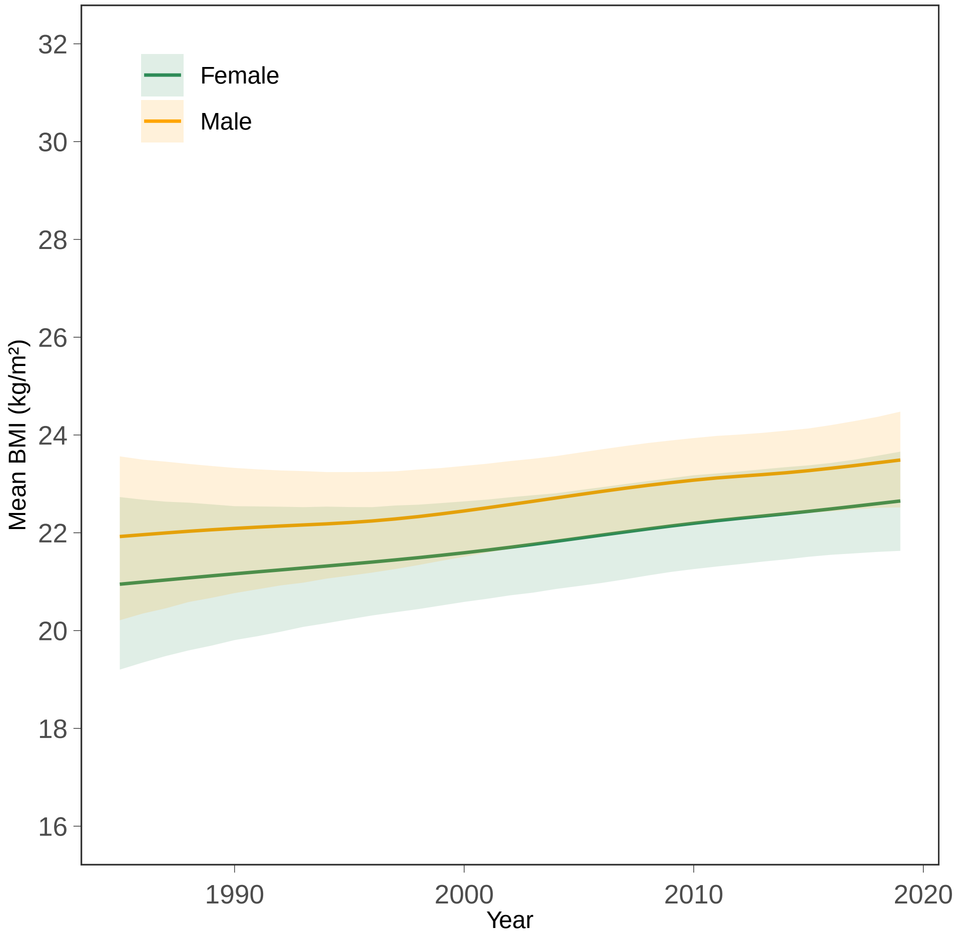

Height−for−age trajectories (2000 birth cohort)

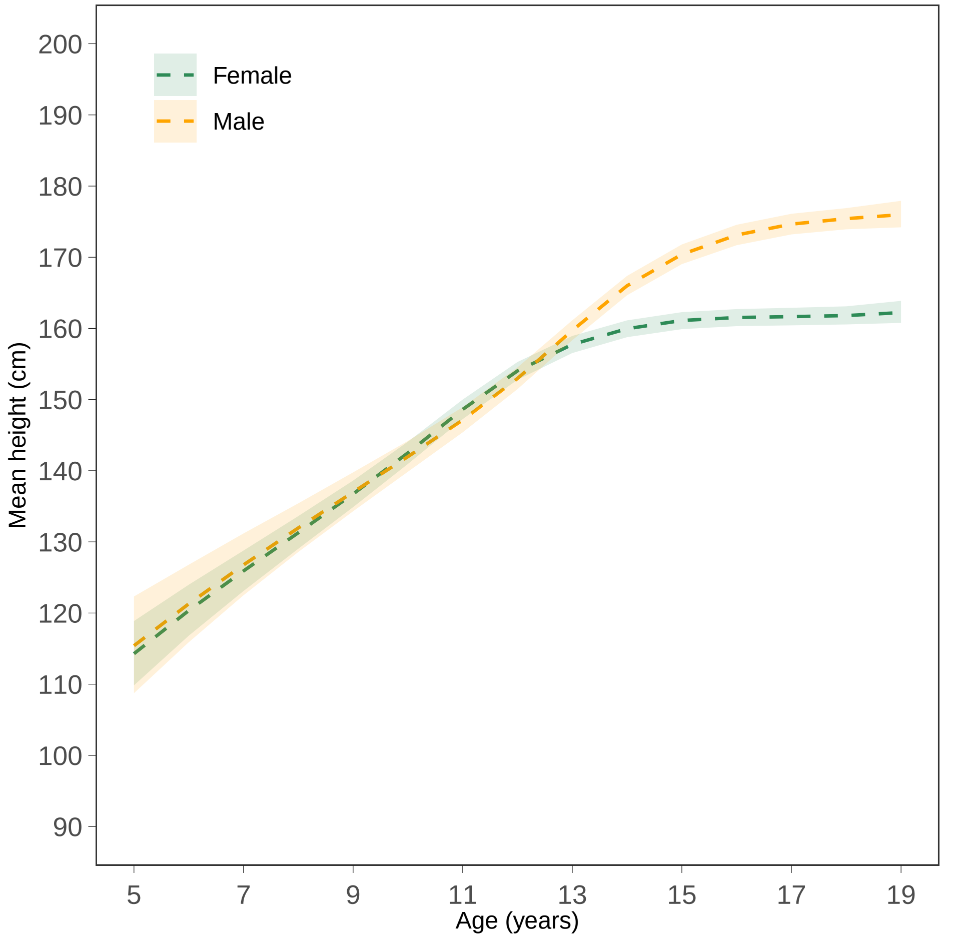

BMI−for−age trajectories (2000 birth cohort)

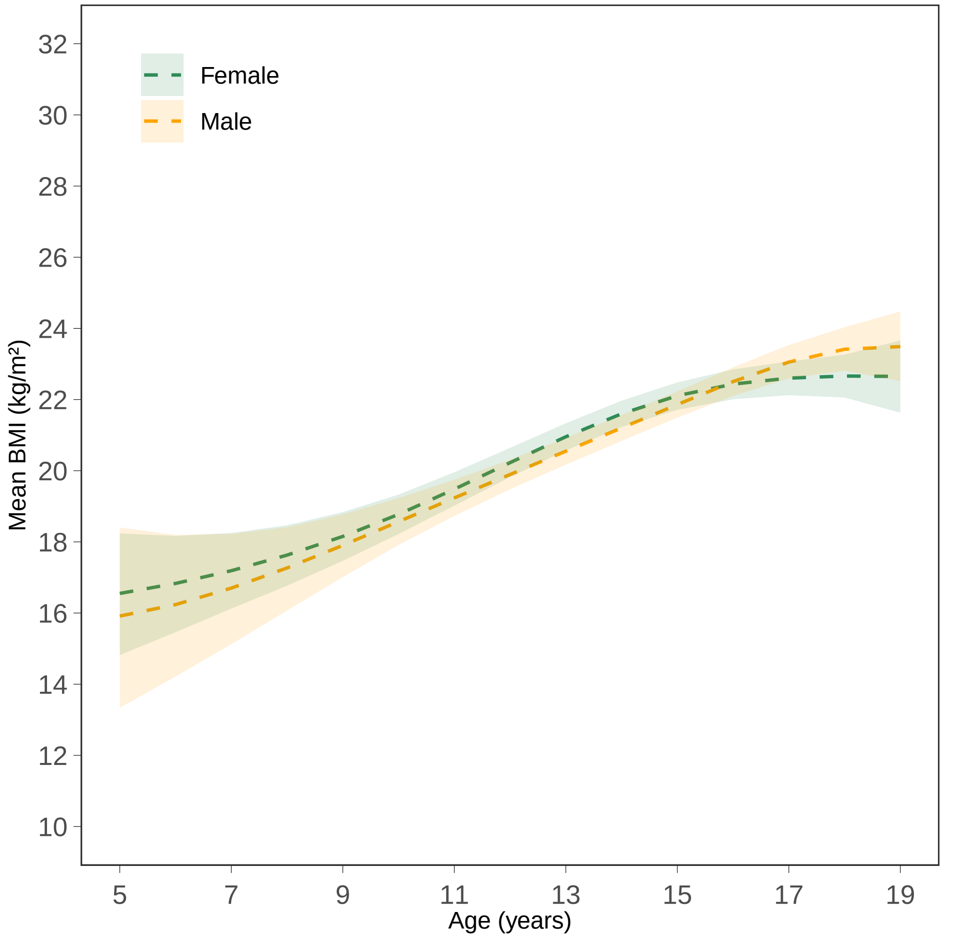

Italy

Time trends in height of 19 year olds

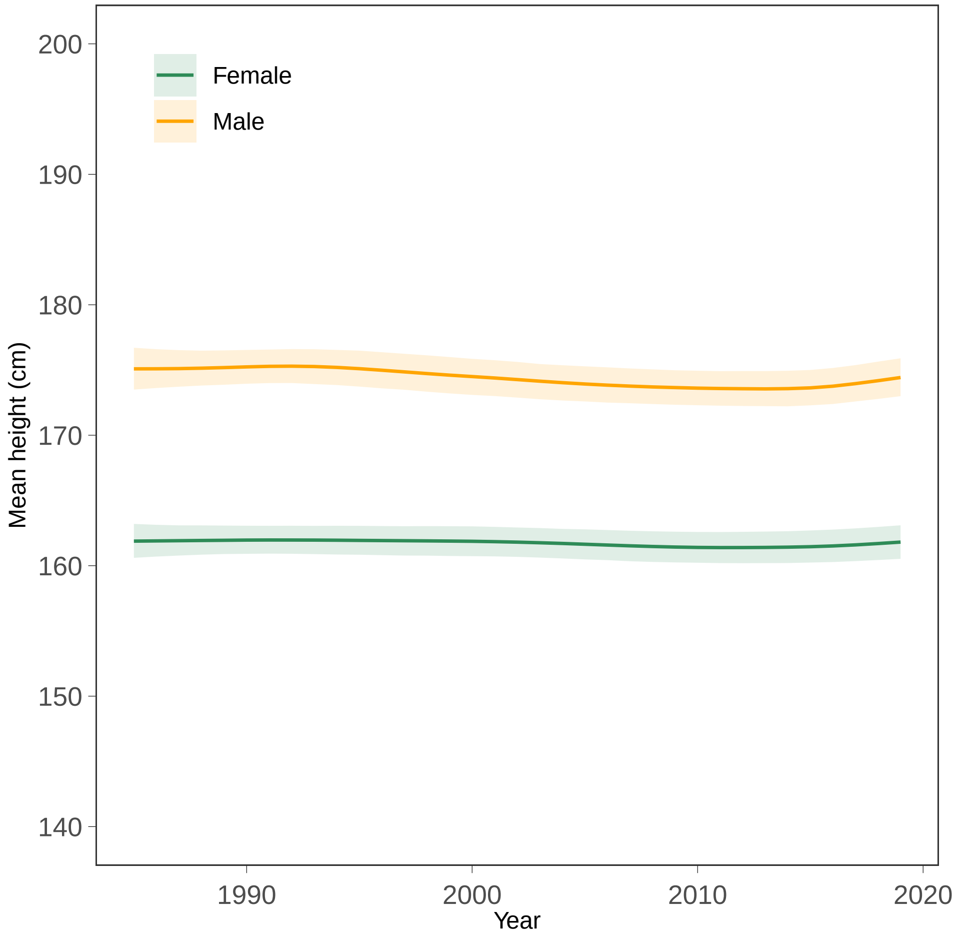

Time trends in BMI of 19 year olds

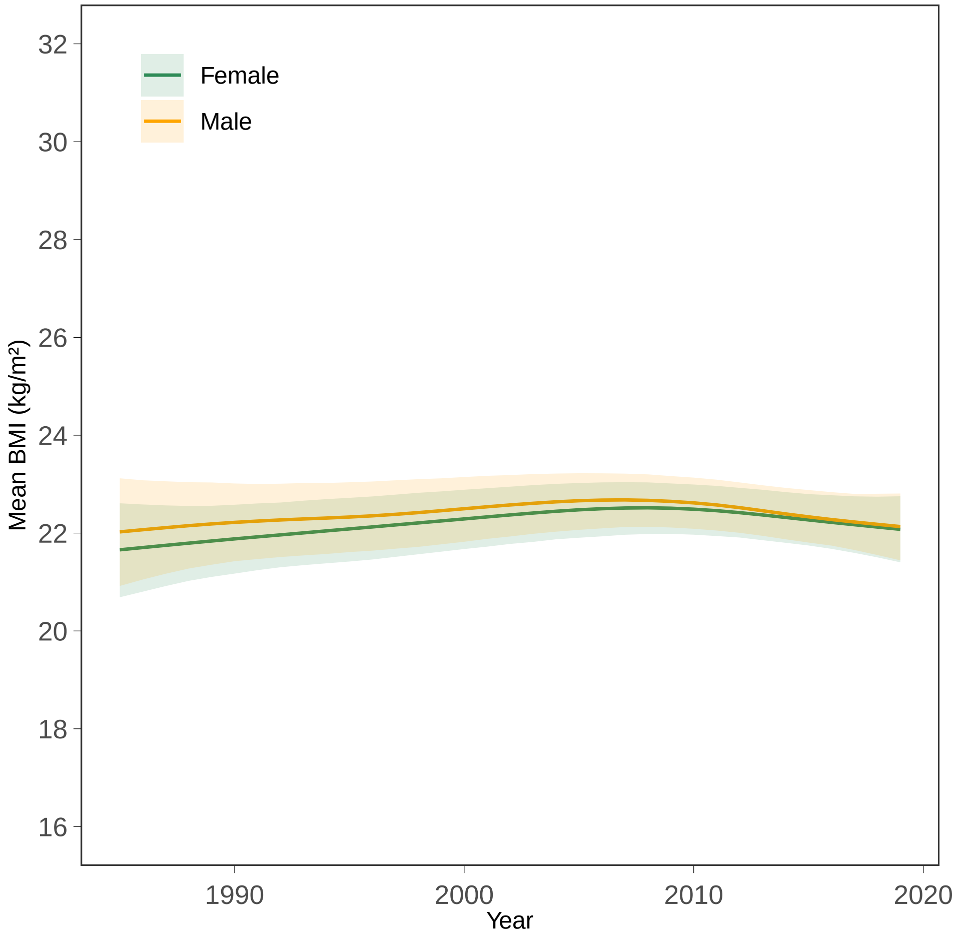

Height−for−age trajectories (2000 birth cohort)

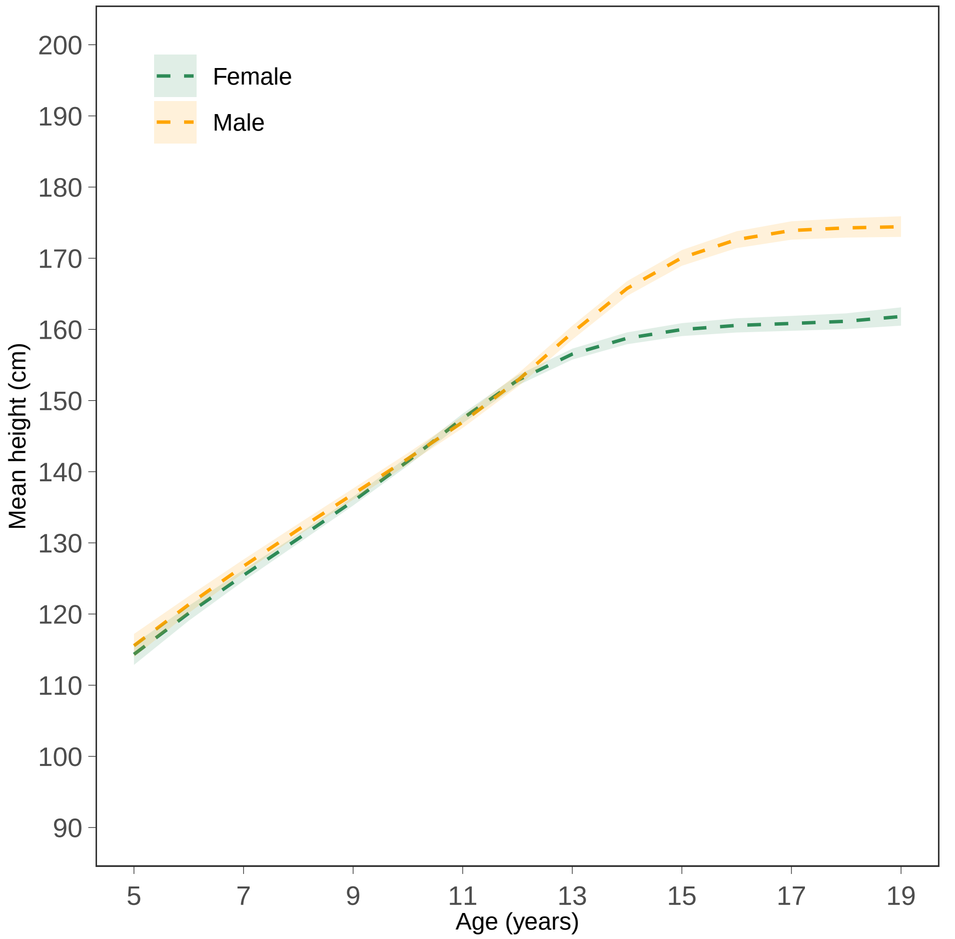

BMI−for−age trajectories (2000 birth cohort)

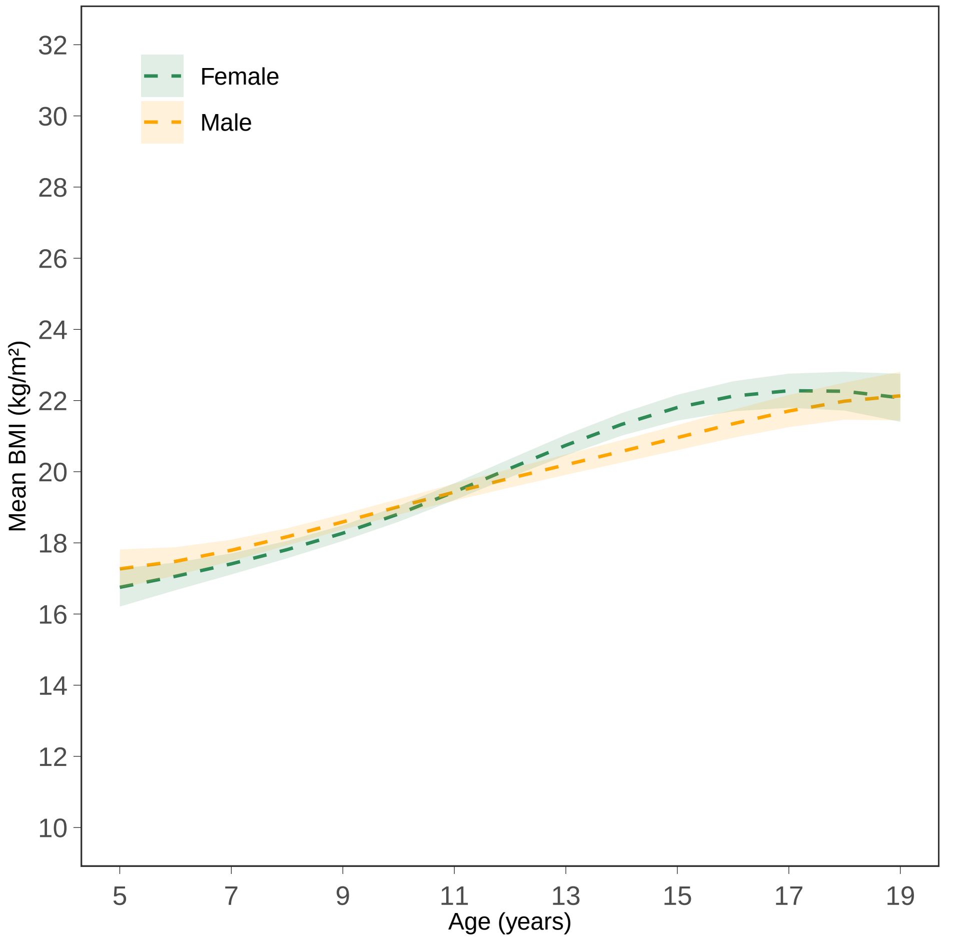

Jamaica

Time trends in height of 19 year olds

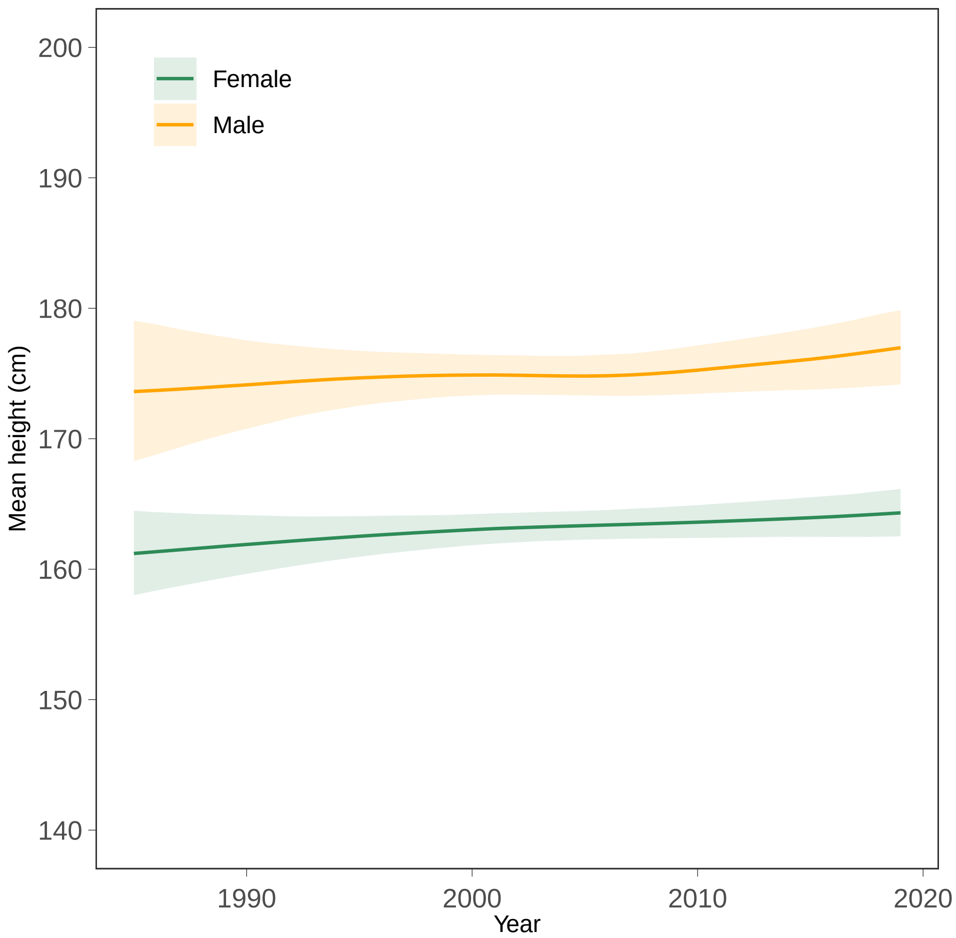

Time trends in BMI of 19 year olds

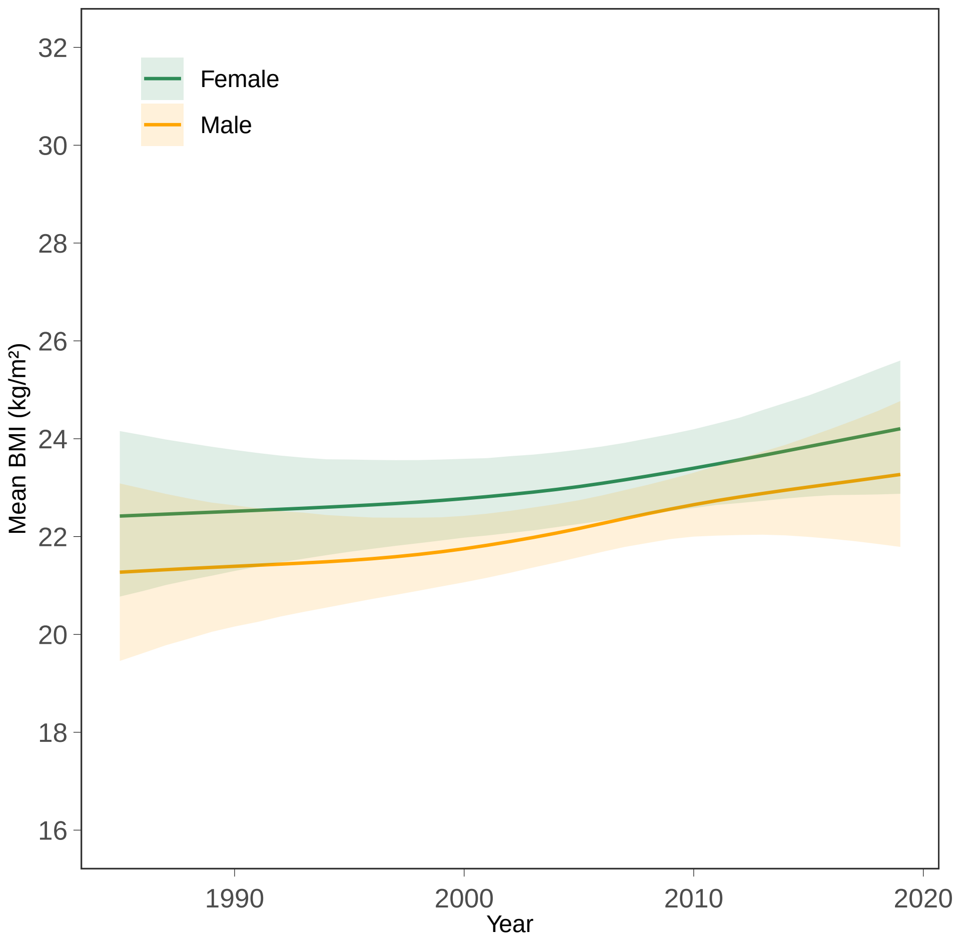

Height−for−age trajectories (2000 birth cohort)

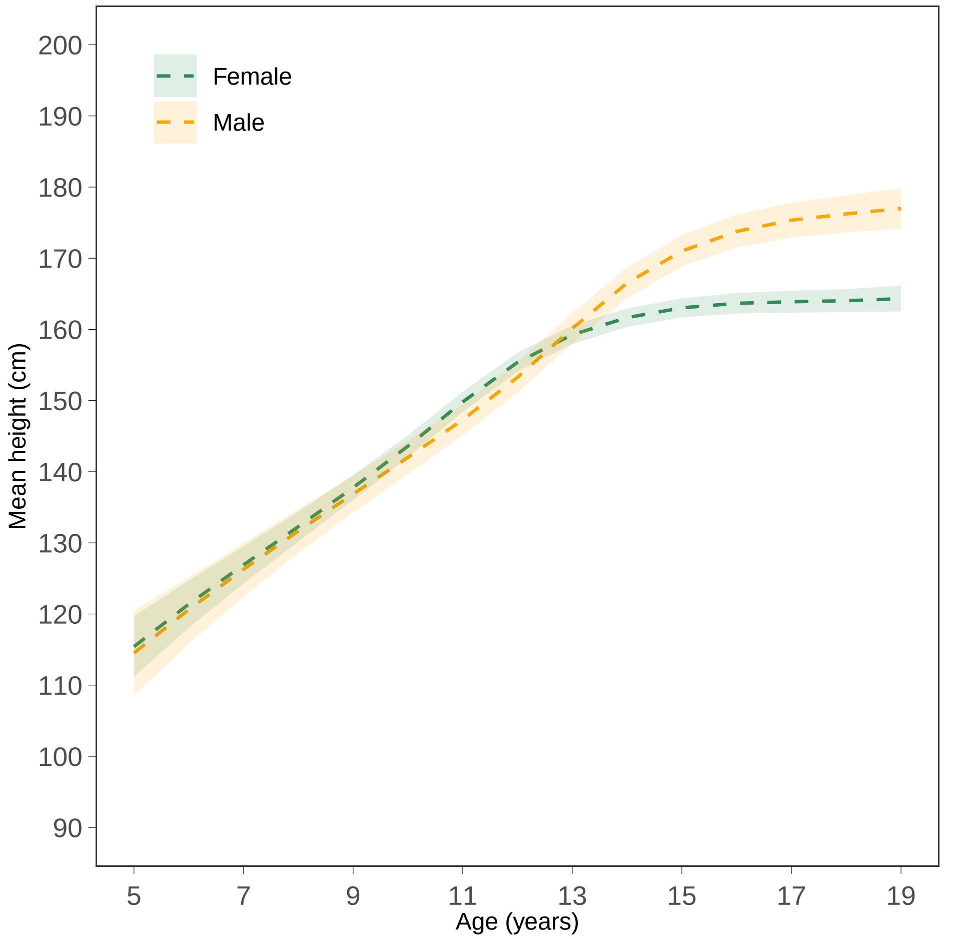

BMI−for−age trajectories (2000 birth cohort)

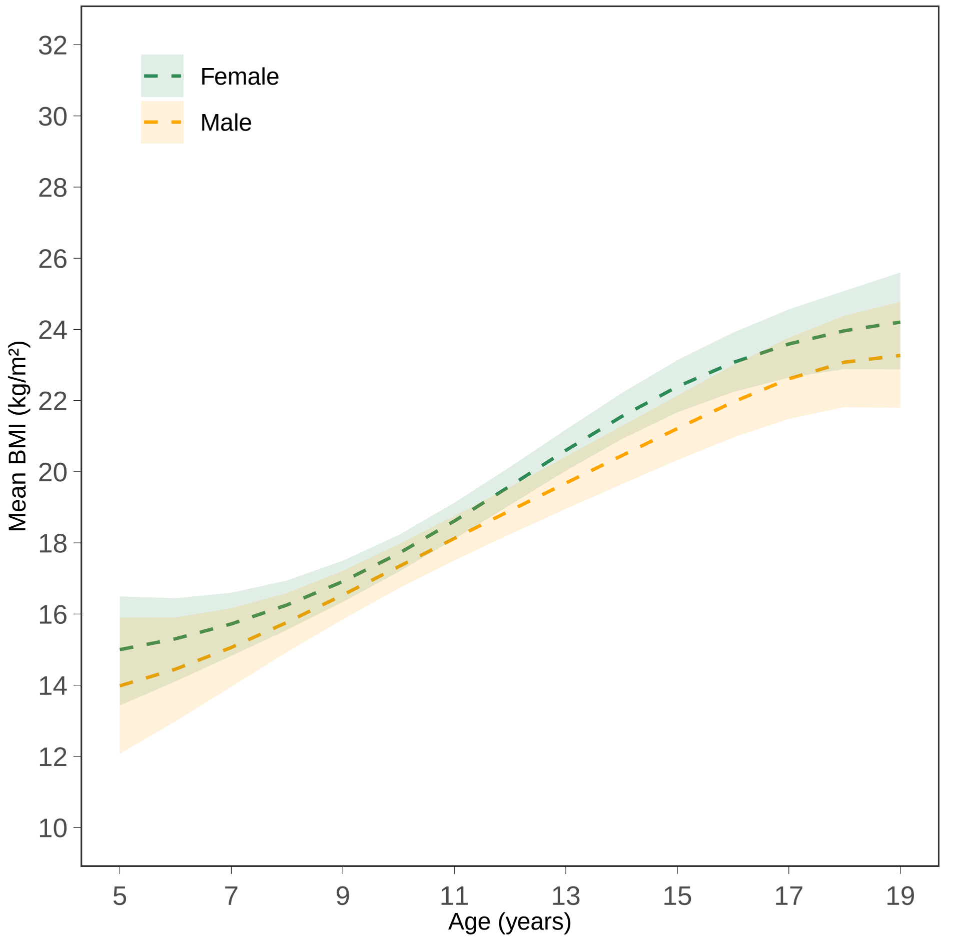

Japan

Time trends in height of 19 year olds

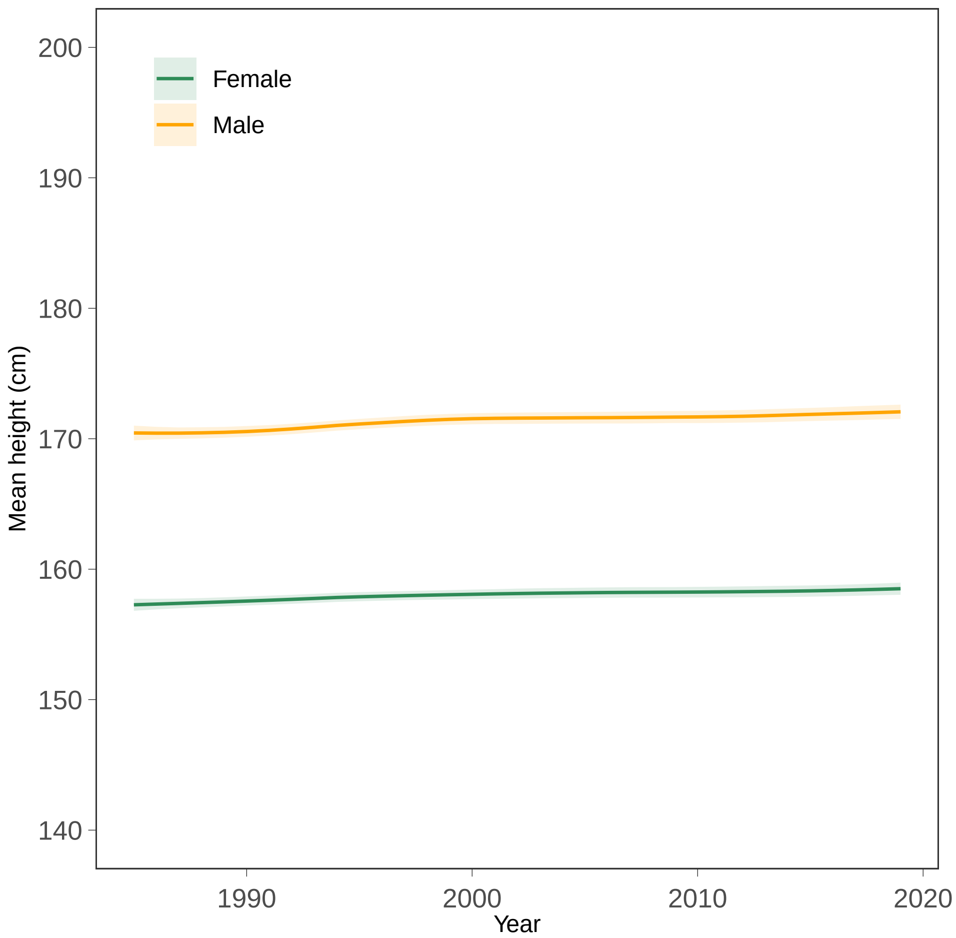

Time trends in BMI of 19 year olds

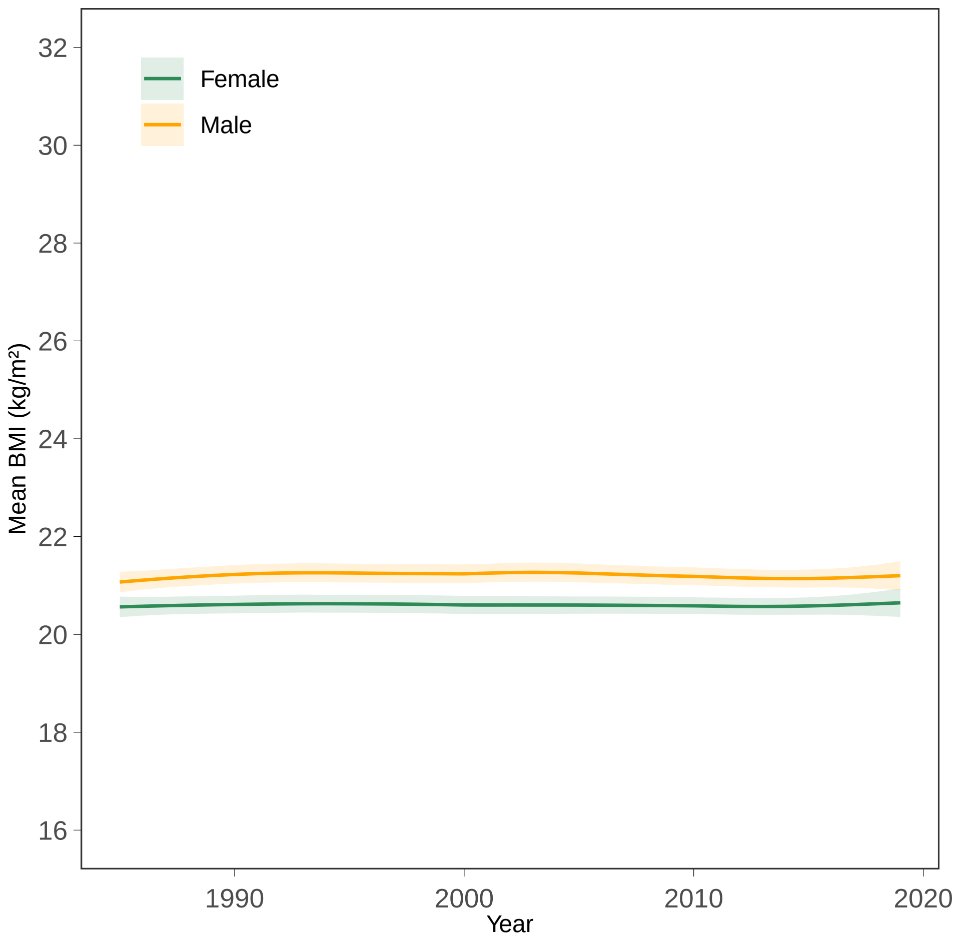

Height−for−age trajectories (2000 birth cohort)

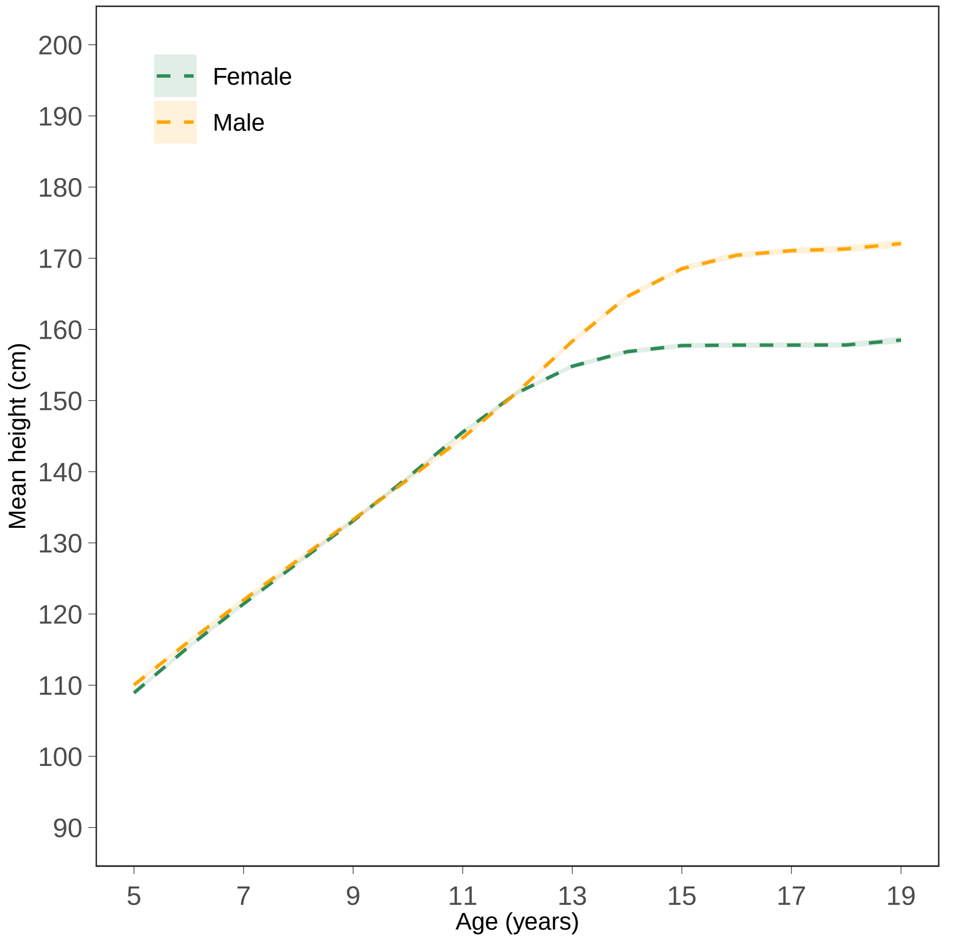

BMI−for−age trajectories (2000 birth cohort)

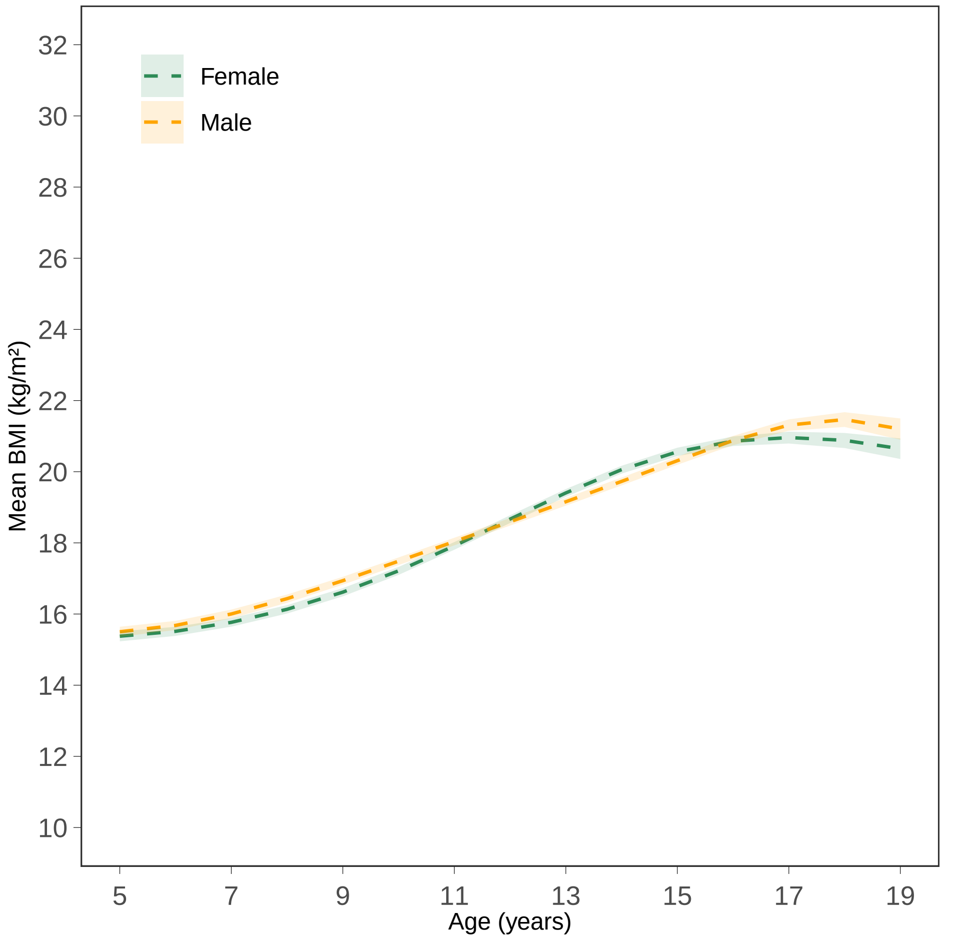

Jordan

Time trends in height of 19 year olds

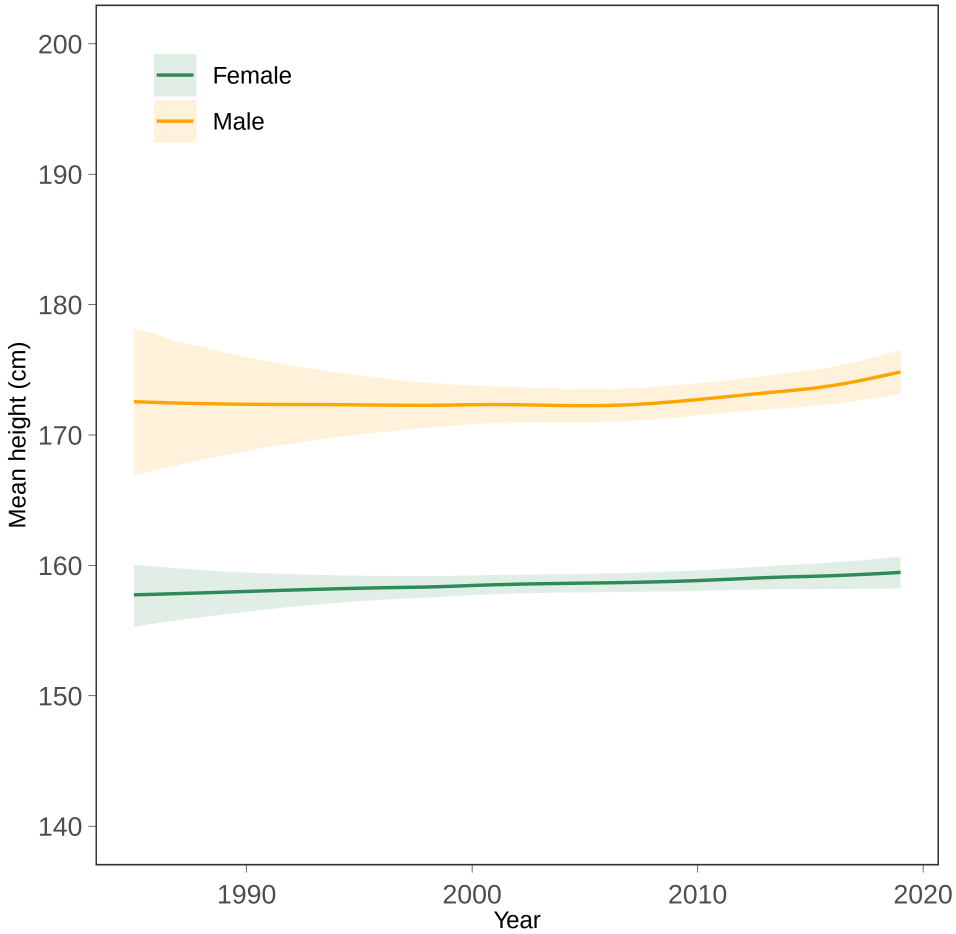

Time trends in BMI of 19 year olds

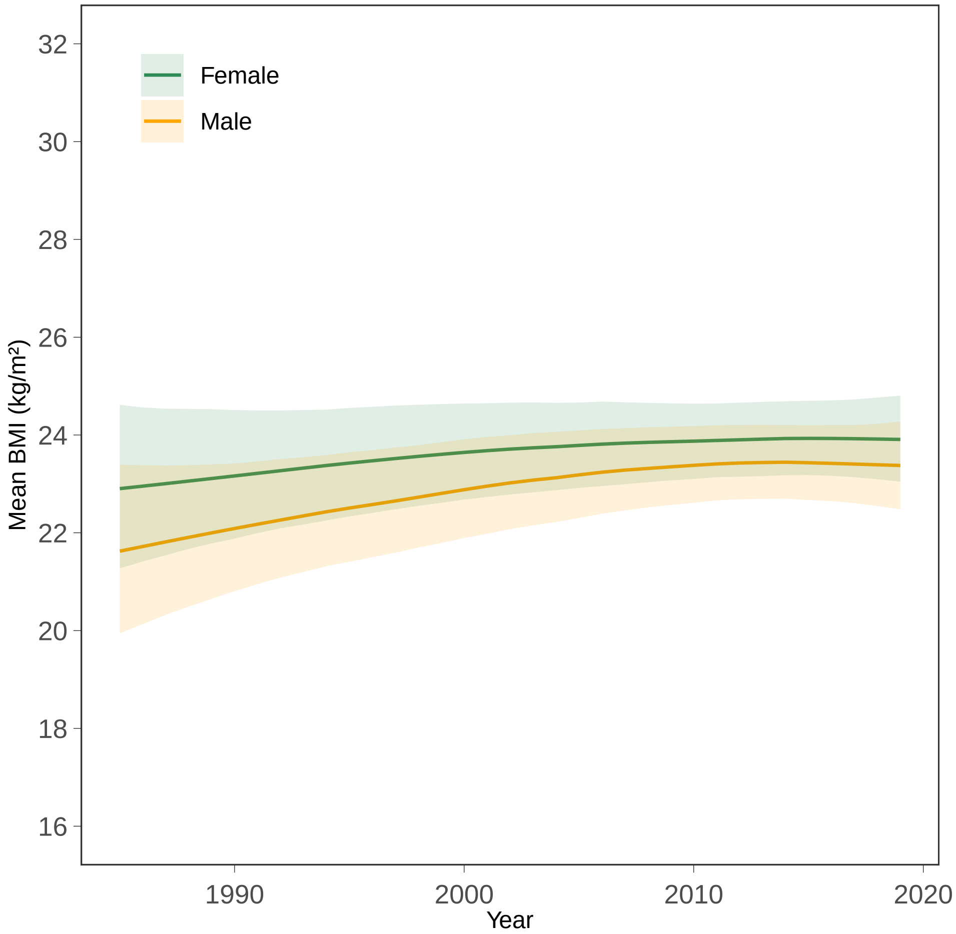

Height−for−age trajectories (2000 birth cohort)

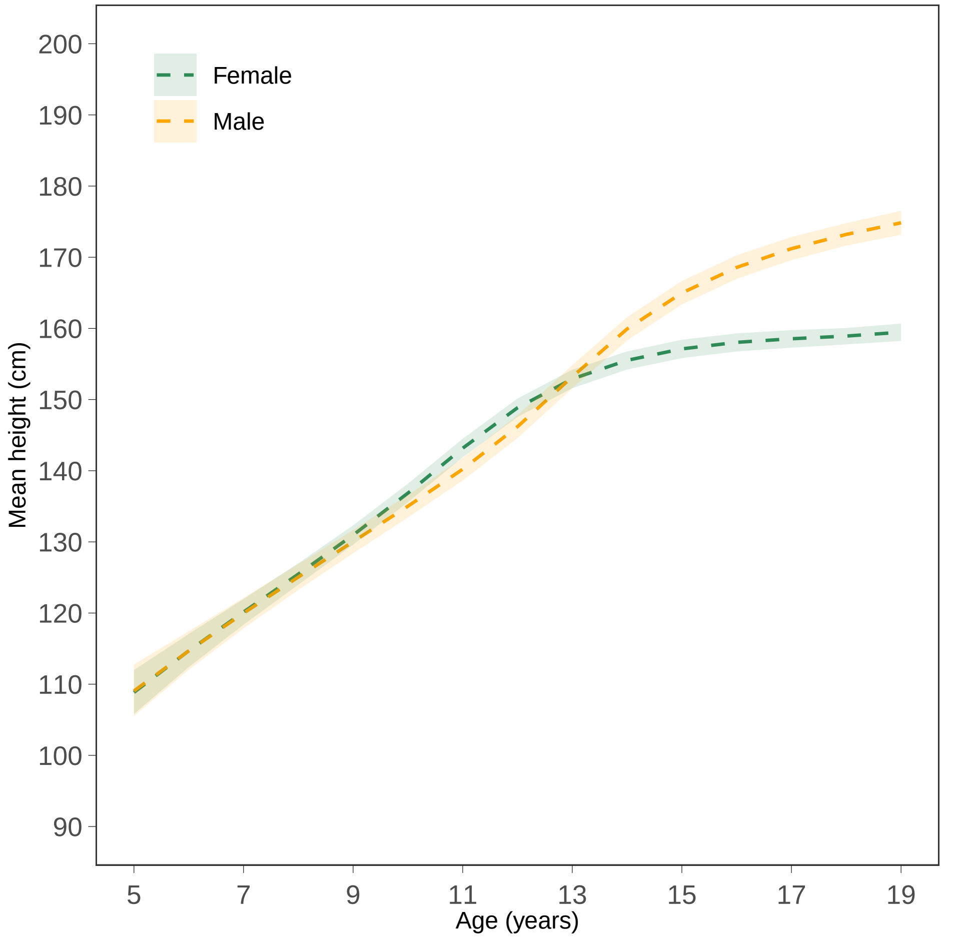

BMI−for−age trajectories (2000 birth cohort)

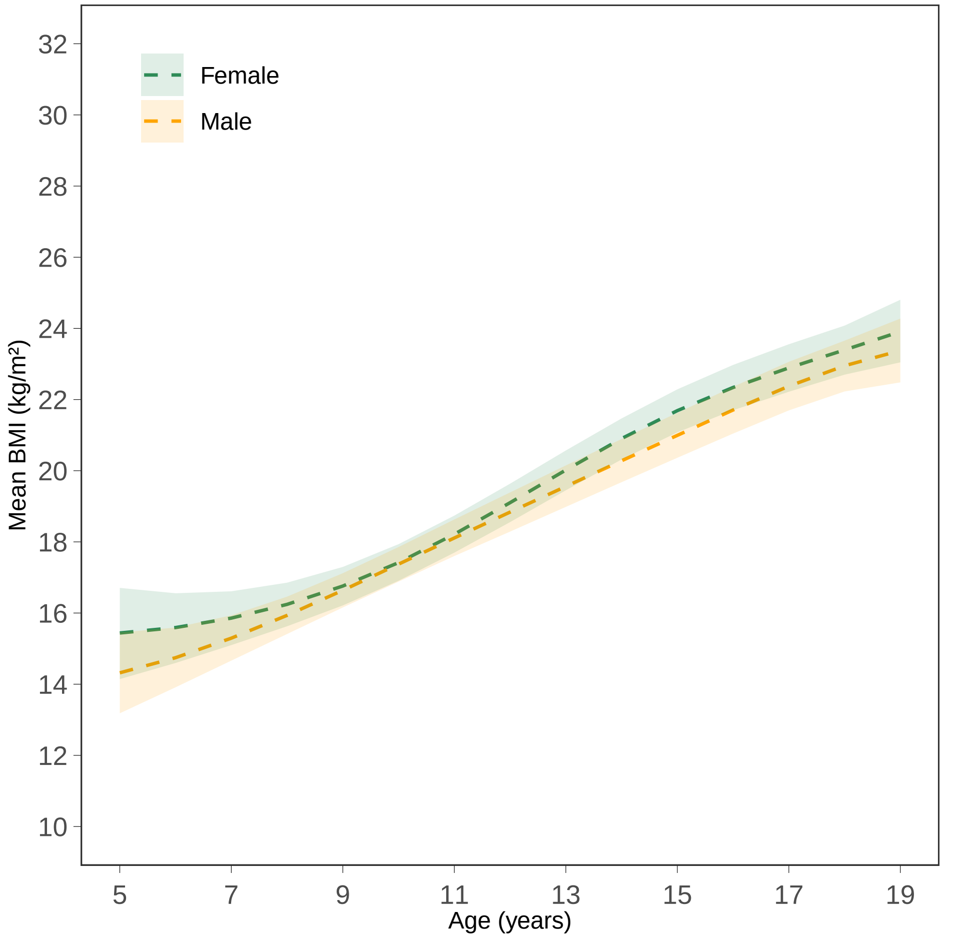

Kazakhstan

Time trends in height of 19 year olds

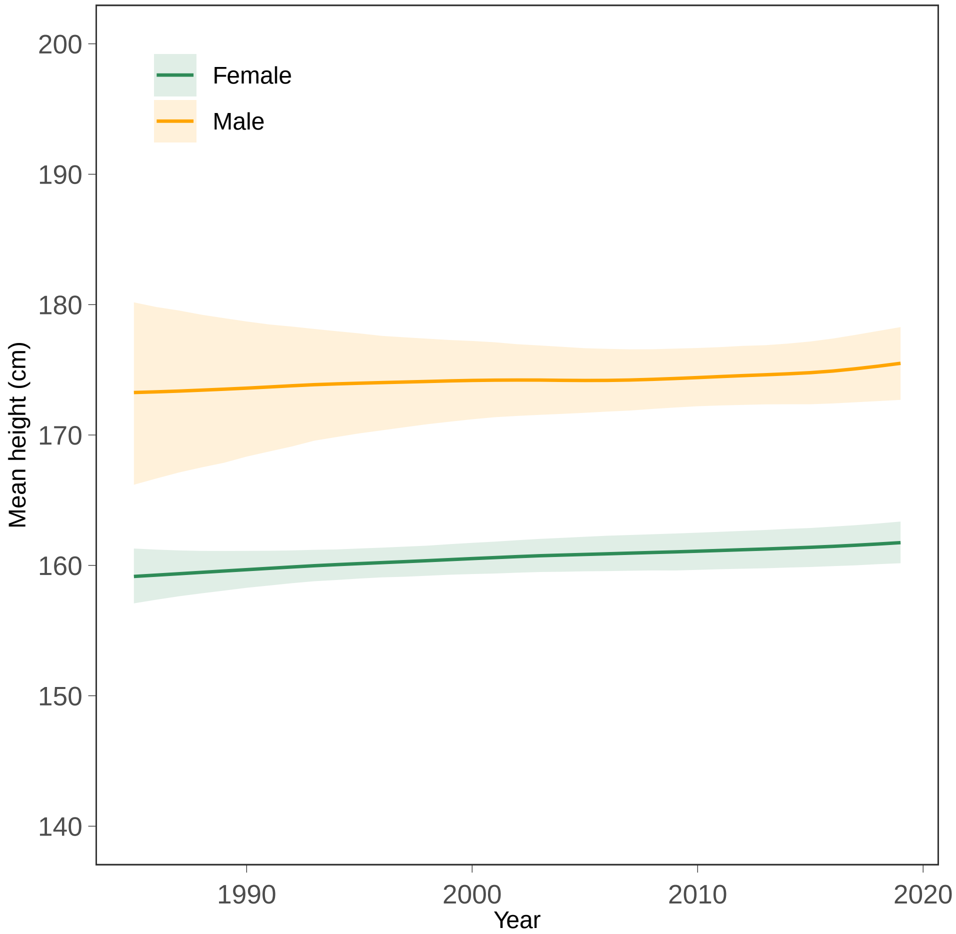

Time trends in BMI of 19 year olds

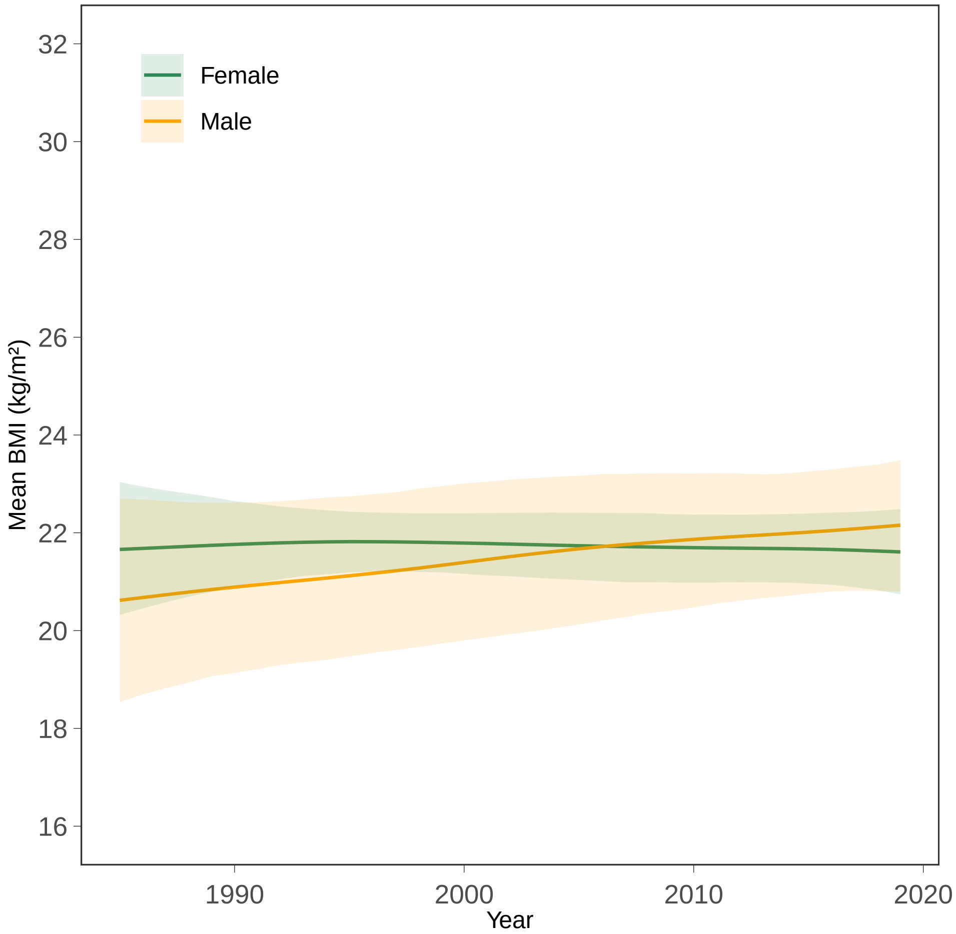

Height−for−age trajectories (2000 birth cohort)

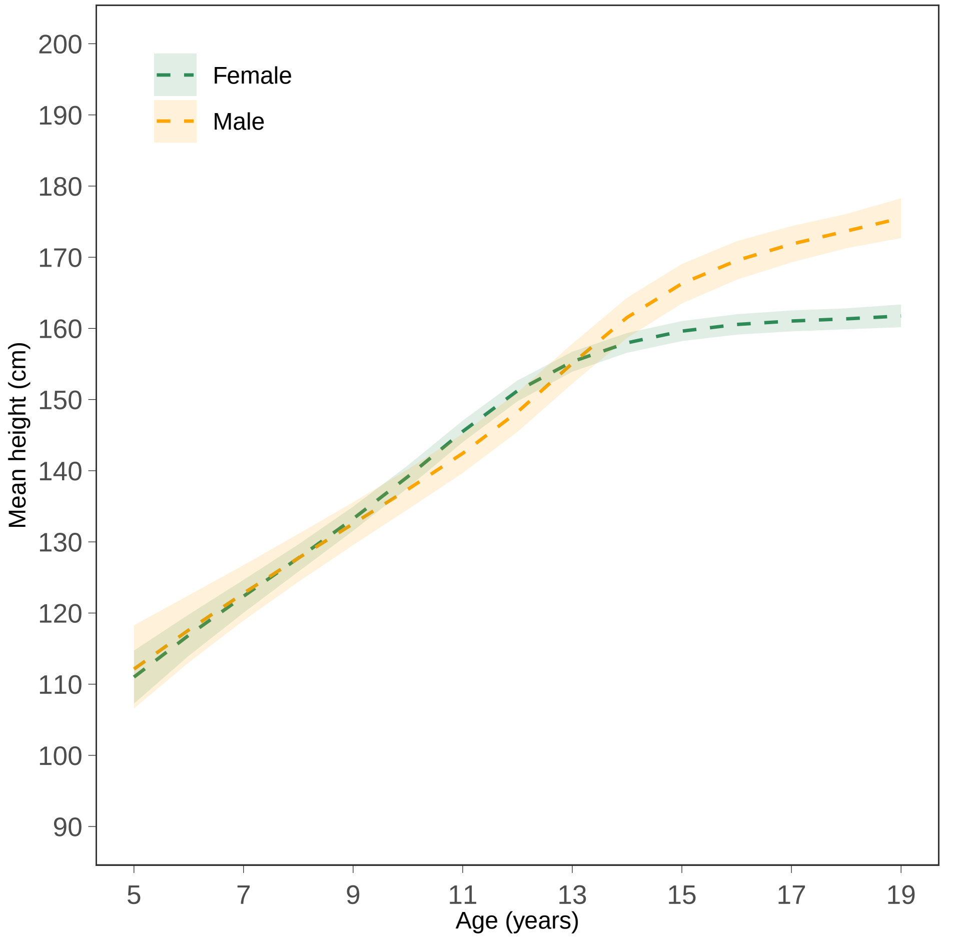

BMI−for−age trajectories (2000 birth cohort)

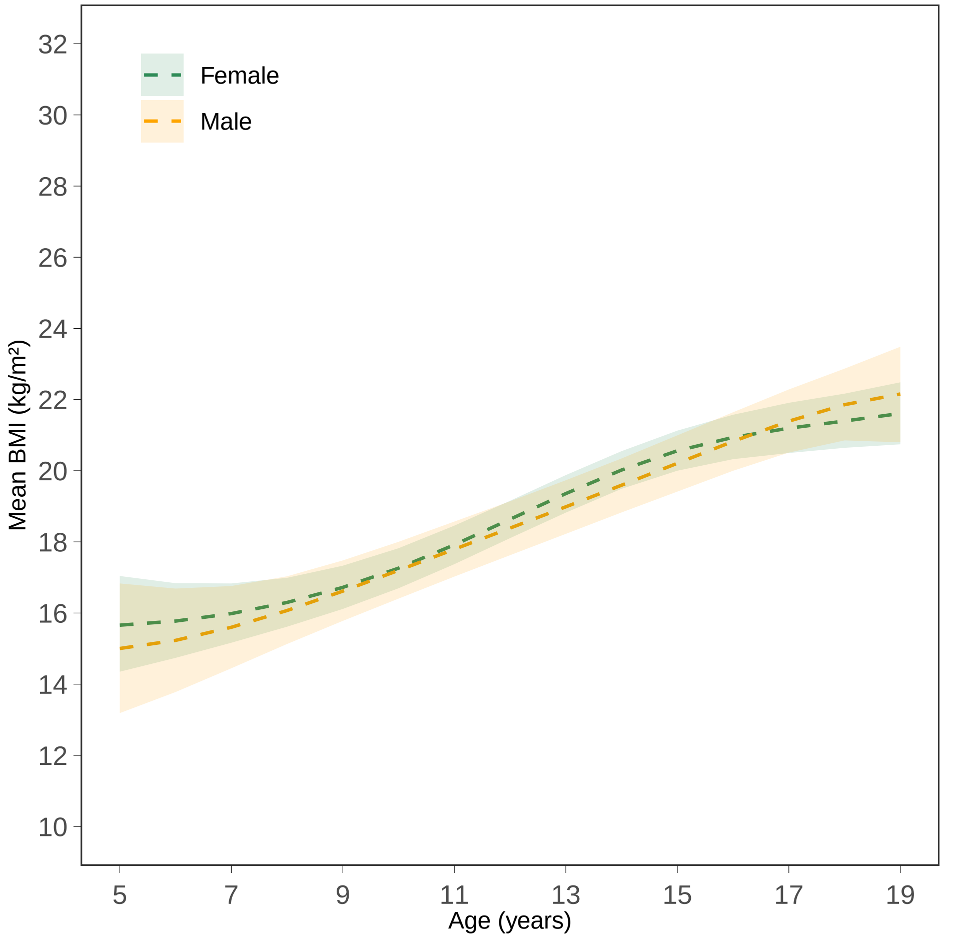

Kenya

Time trends in height of 19 year olds

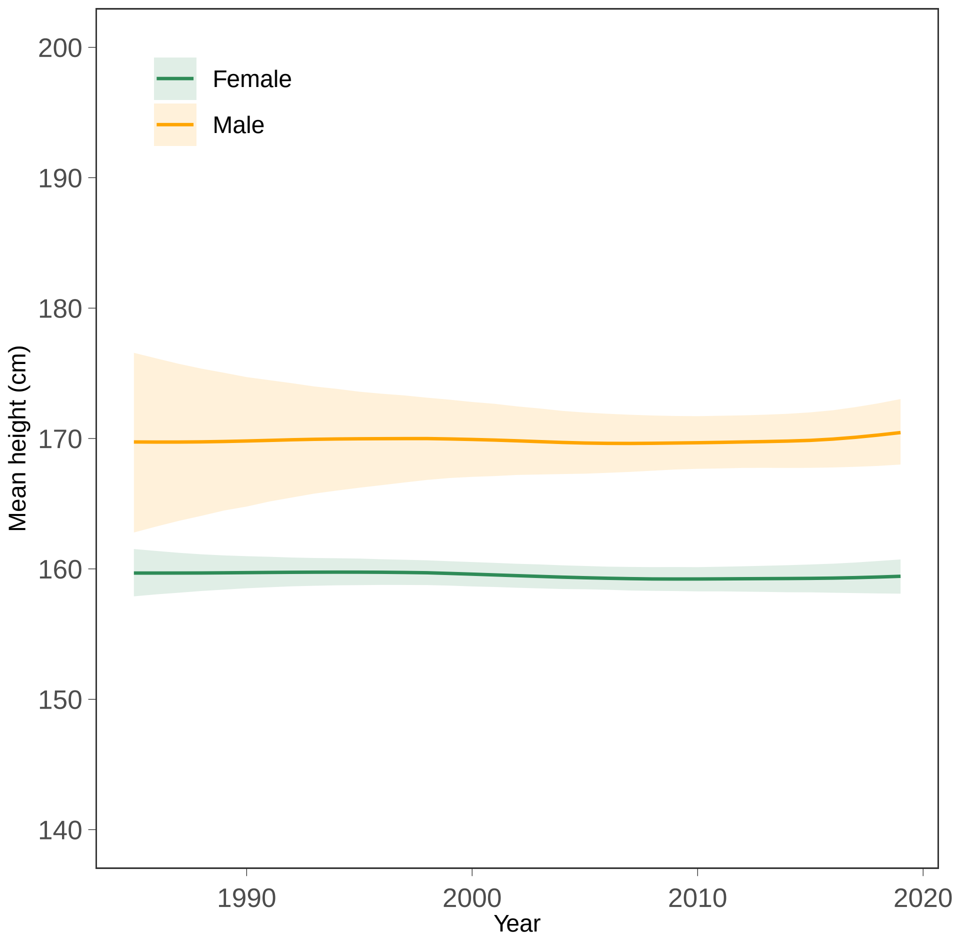

Time trends in BMI of 19 year olds

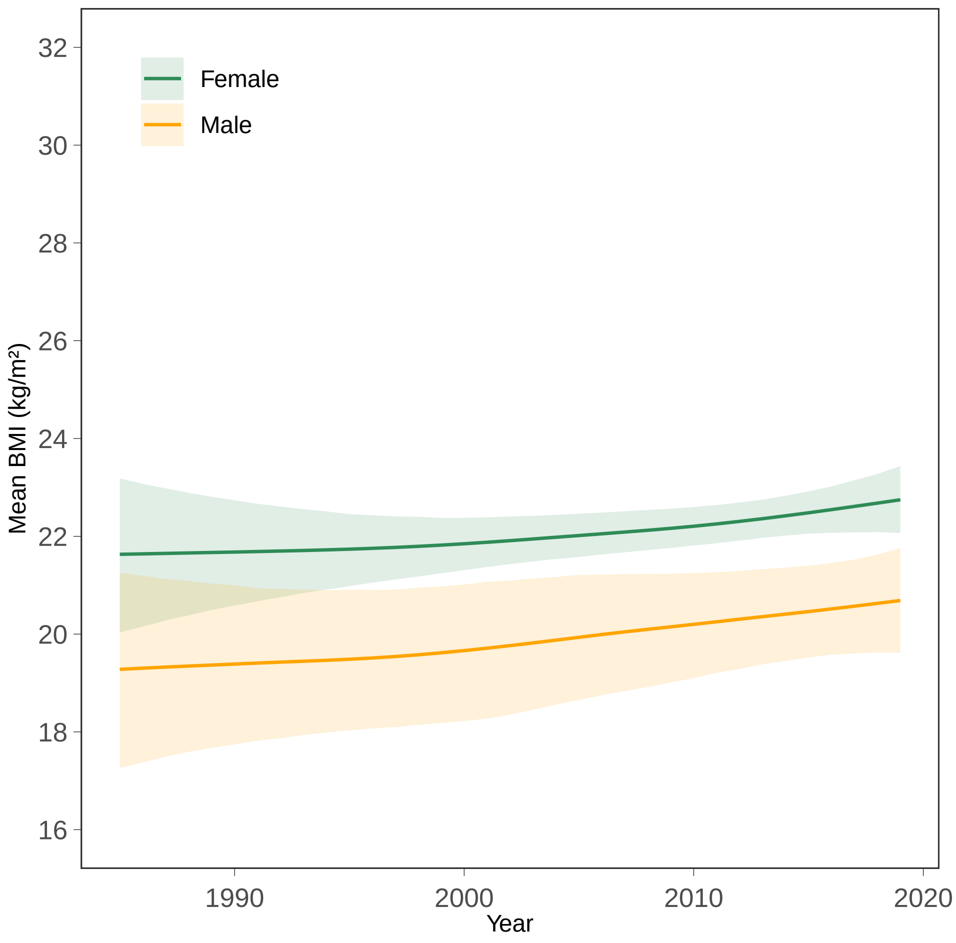

Height−for−age trajectories (2000 birth cohort)

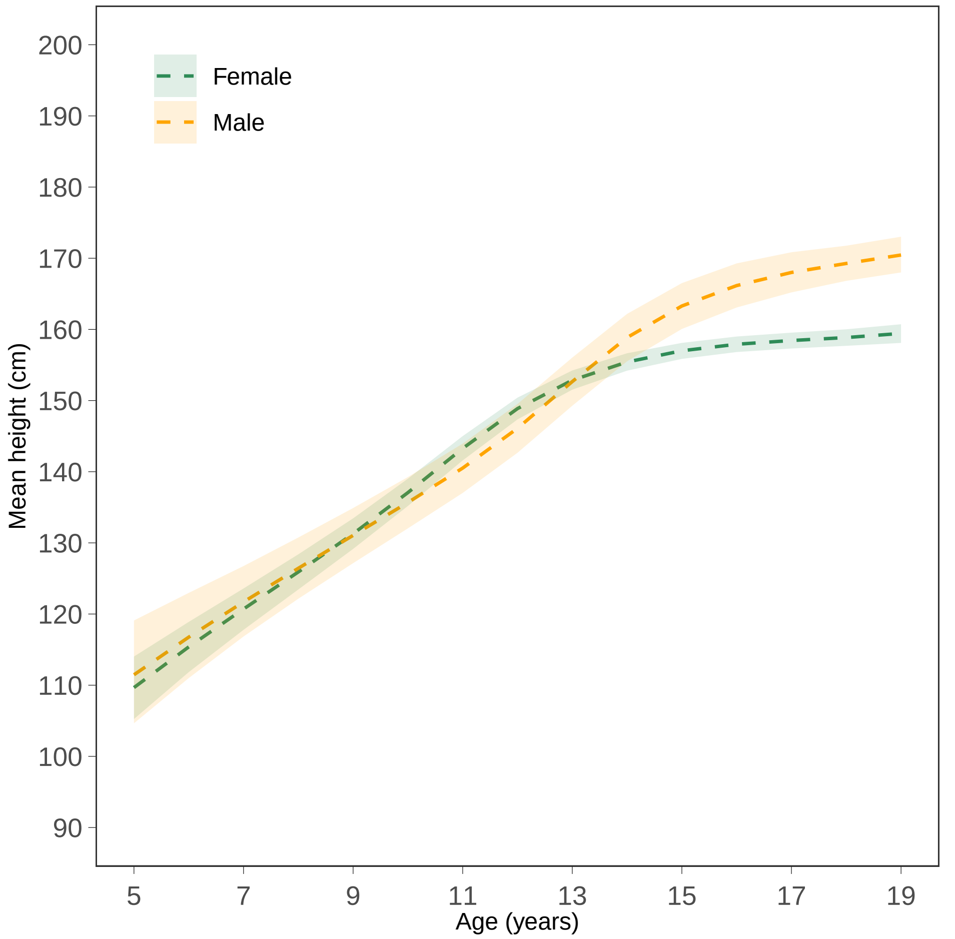

BMI−for−age trajectories (2000 birth cohort)

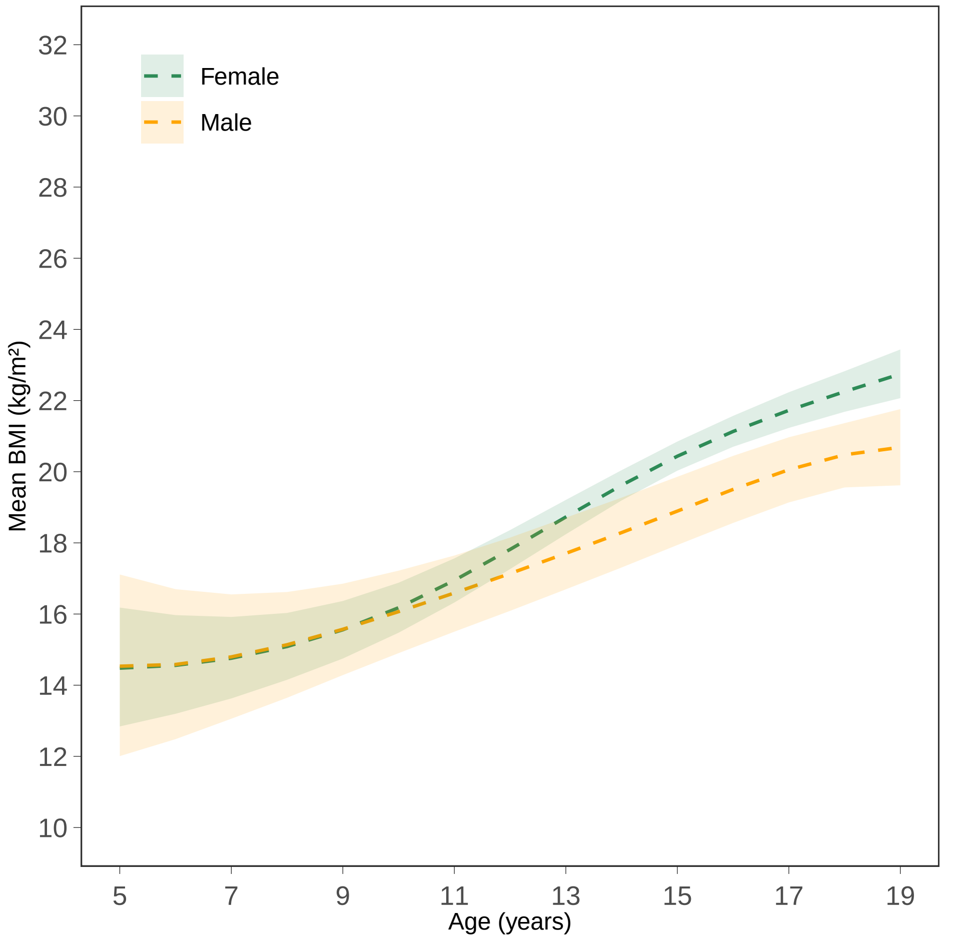

Kiribati

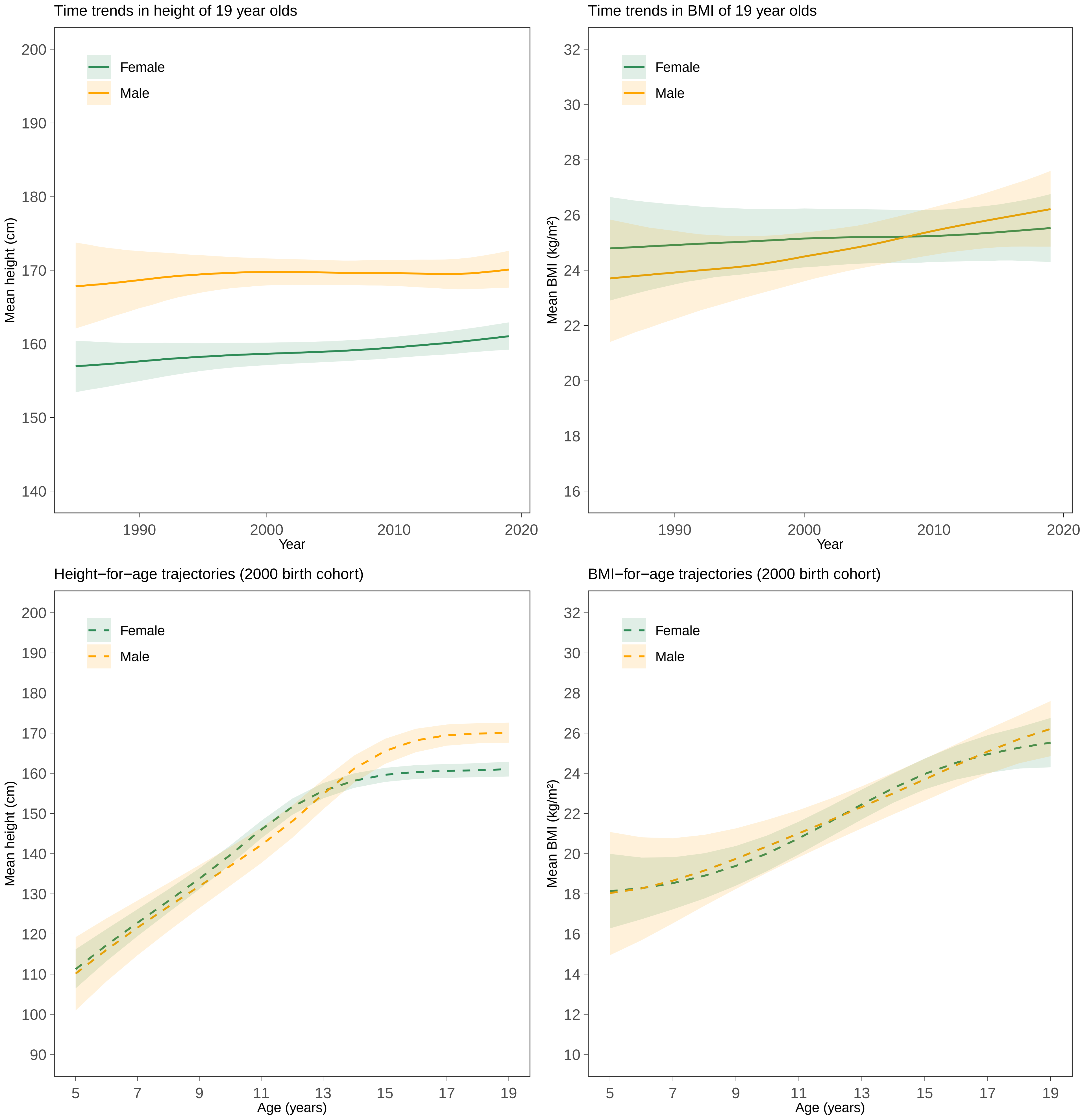

Time trends in height of 19 year olds
Female
Male
Mean height (cm)
Year
Time trends in BMI of 19 year olds
Mean BMI (kg/m²)
Height−for−age trajectories (2000 birth cohort)
Age (years)
BMI−for−age trajectories (2000 birth cohort)

Kuwait

Time trends in height of 19 year olds

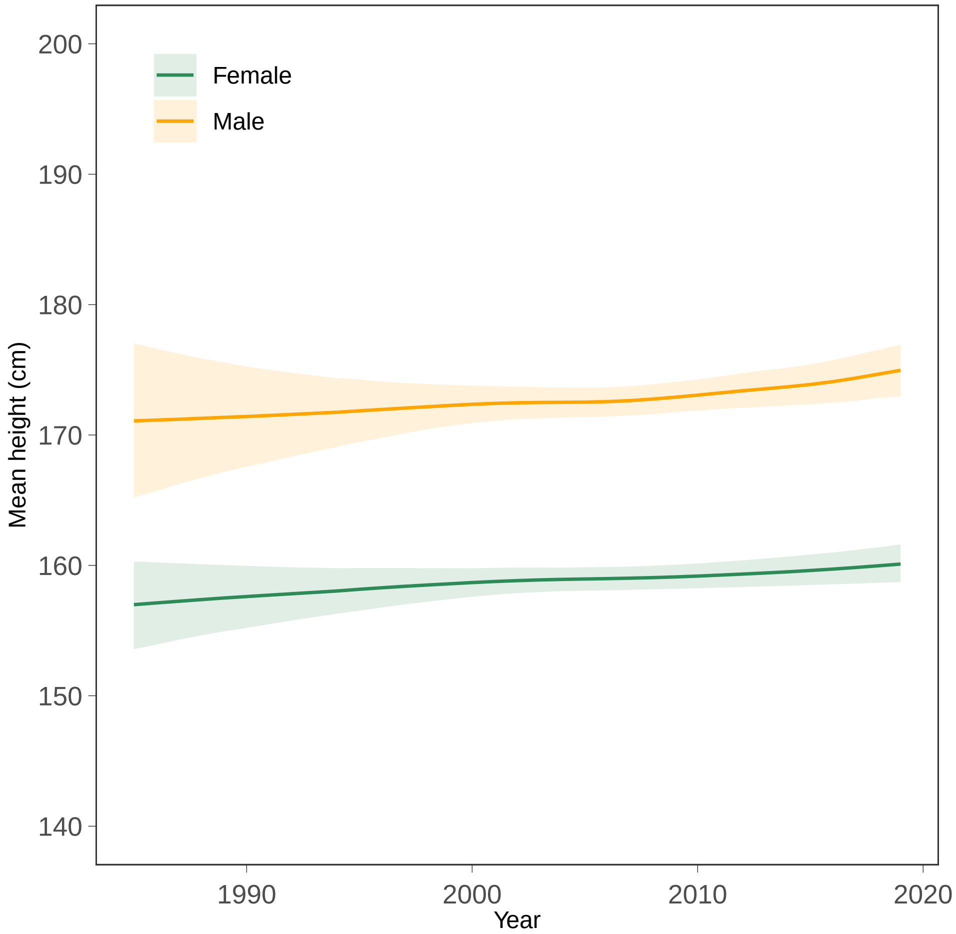

Time trends in BMI of 19 year olds

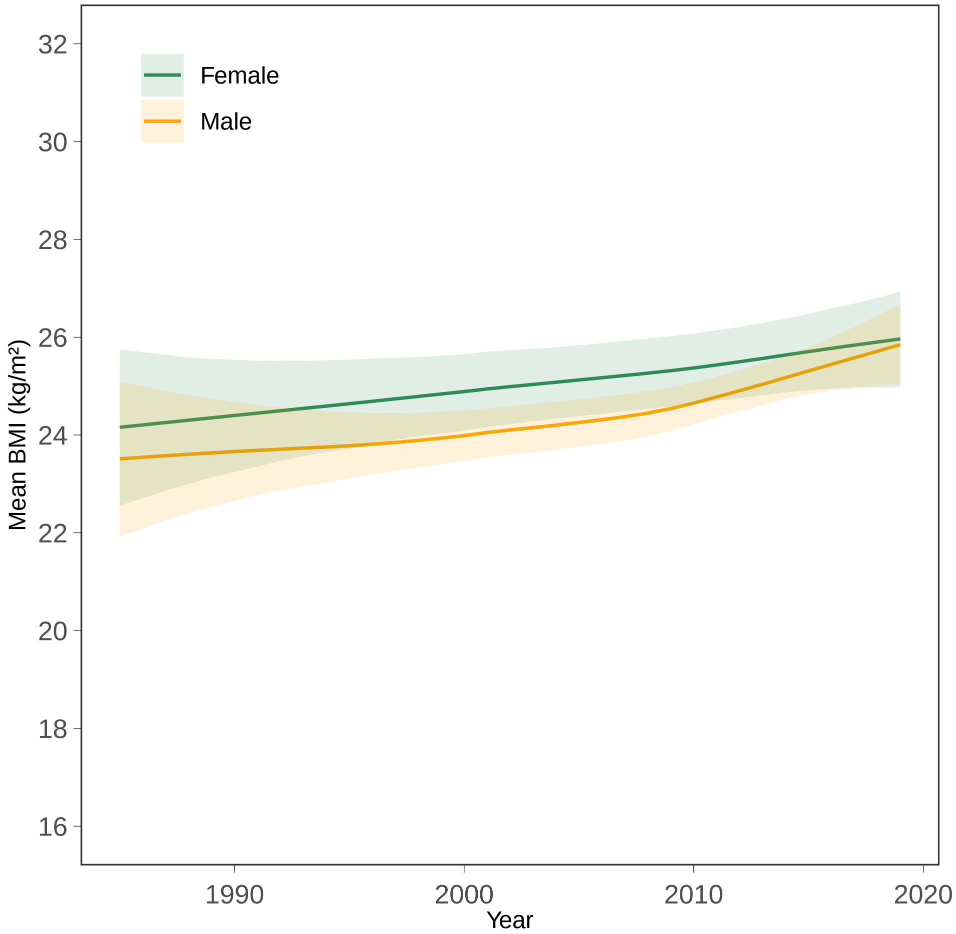

Height−for−age trajectories (2000 birth cohort)

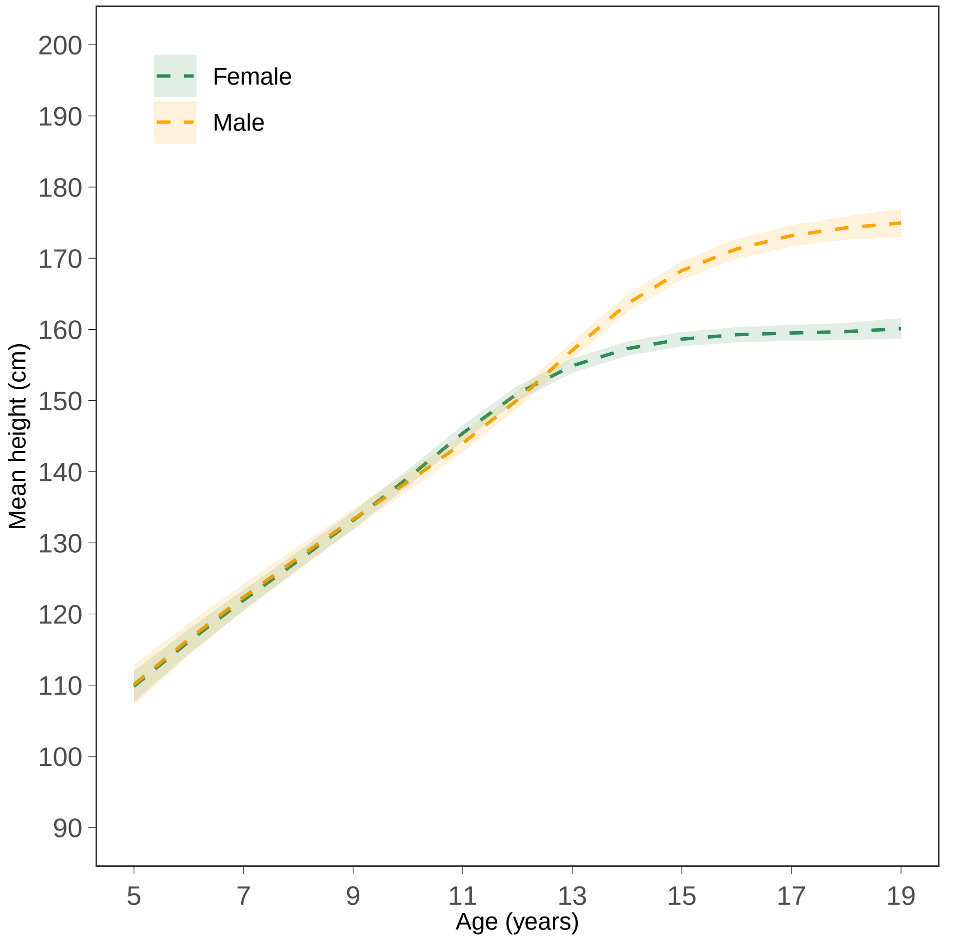

BMI−for−age trajectories (2000 birth cohort)

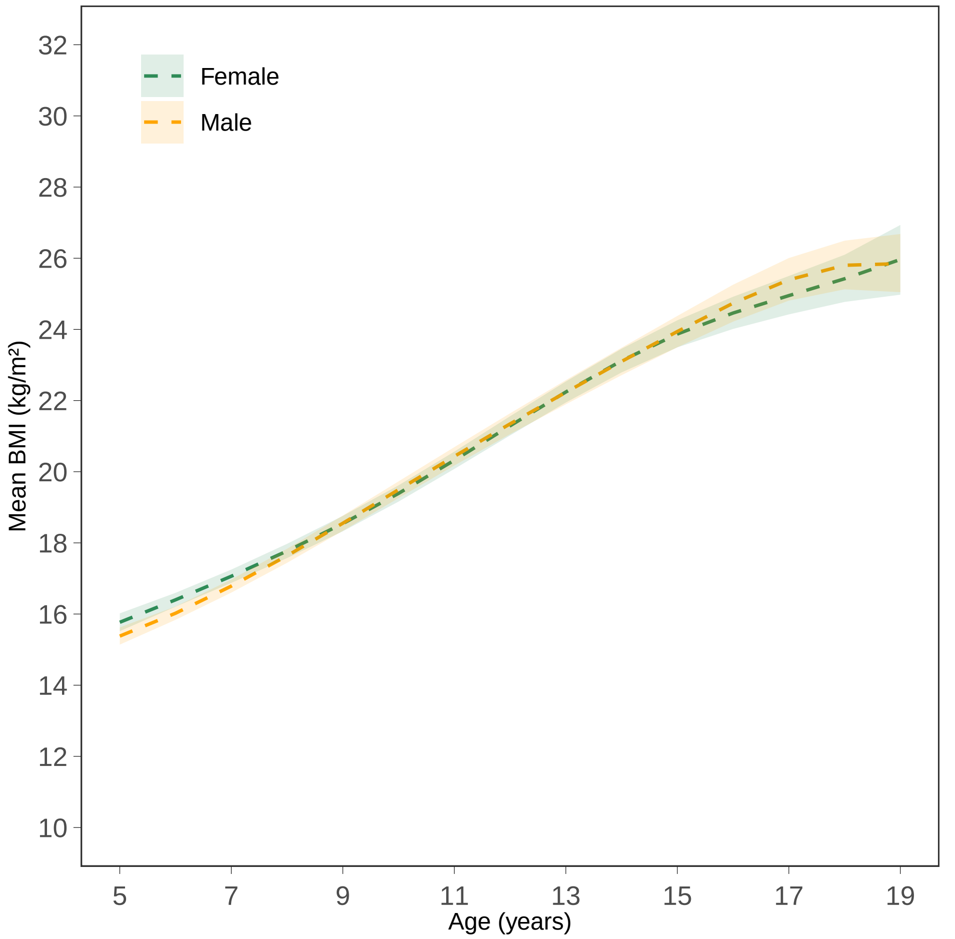

Kyrgyzstan

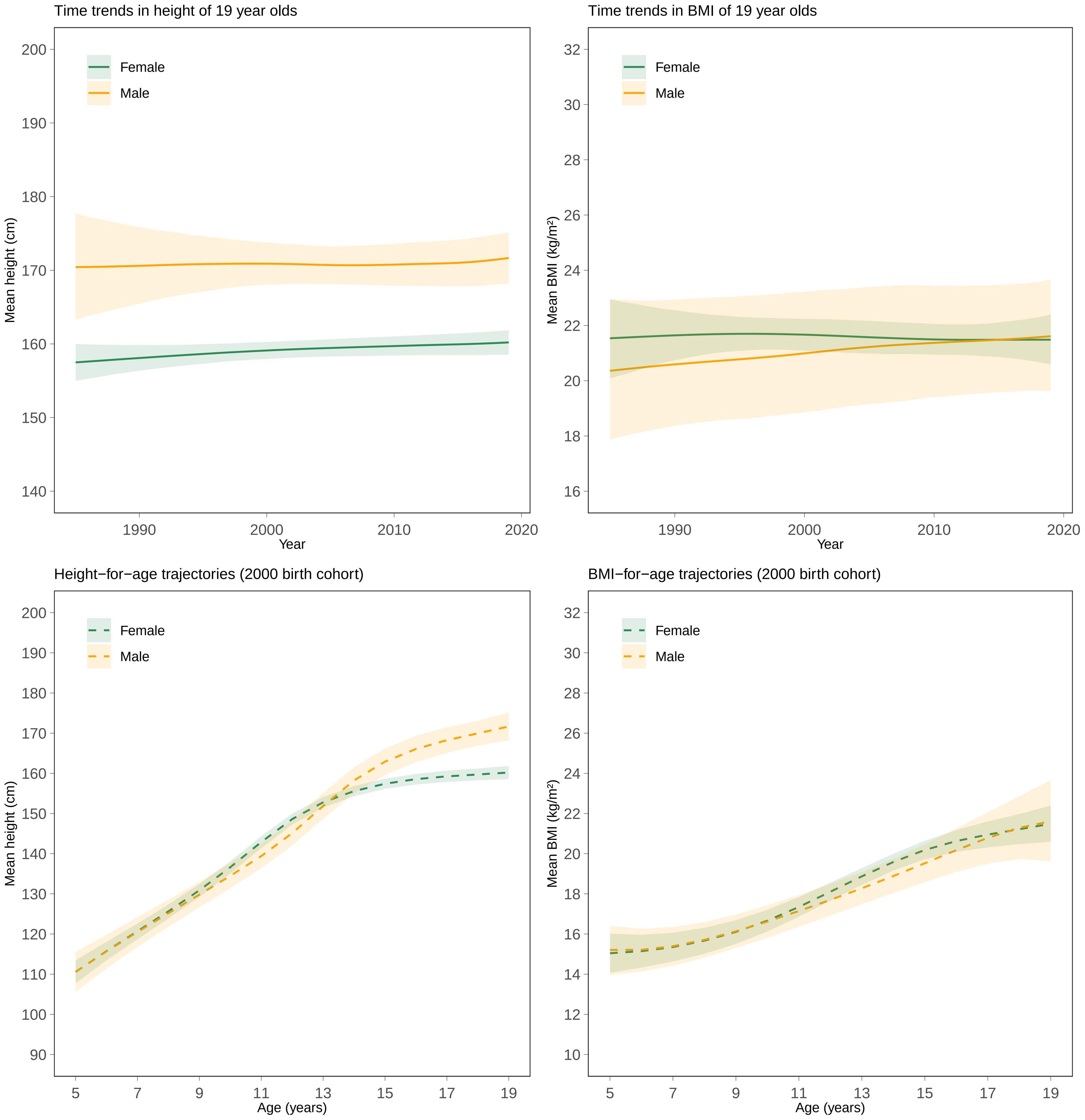

Time trends in height of 19 year olds
Female
Male
Mean height (cm)
200
190
180
170
160
150
140
1990
2000
2010
2020
Year
Time trends in BMI of 19 year olds
Female
Male
Mean BMI (kg/m²)
32
30
28
26
24
22
20
18
16
1990
2000
2010
2020
Year
Height−for−age trajectories (2000 birth cohort)
Female
Male
Mean height (cm)
200
190
180
170
160
150
140
130
120
110
100
90
5
7
9
11
13
15
17
19
Age (years)
BMI−for−age trajectories (2000 birth cohort)
Female
Male
Mean BMI (kg/m²)
32
30
28
26
24
22
20
18
16
14
12
10
5
7
9
11
13
15
17
19
Age (years)

Lao PDR

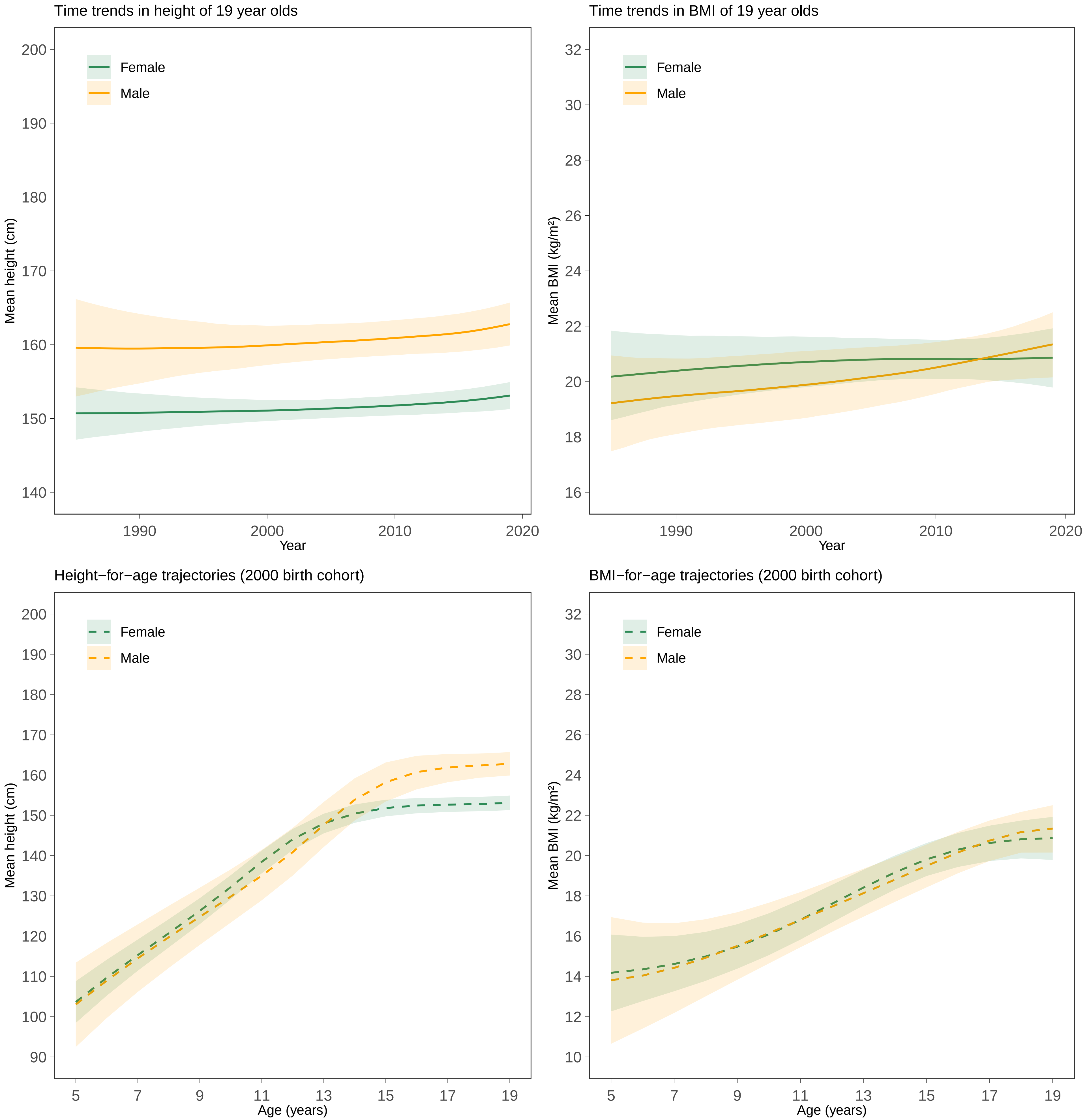

Time trends in height of 19 year olds
Female
Male
Mean height (cm)
Year
Time trends in BMI of 19 year olds
Mean BMI (kg/m²)
Height−for−age trajectories (2000 birth cohort)
Age (years)
BMI−for−age trajectories (2000 birth cohort)

Latvia

Time trends in height of 19 year olds

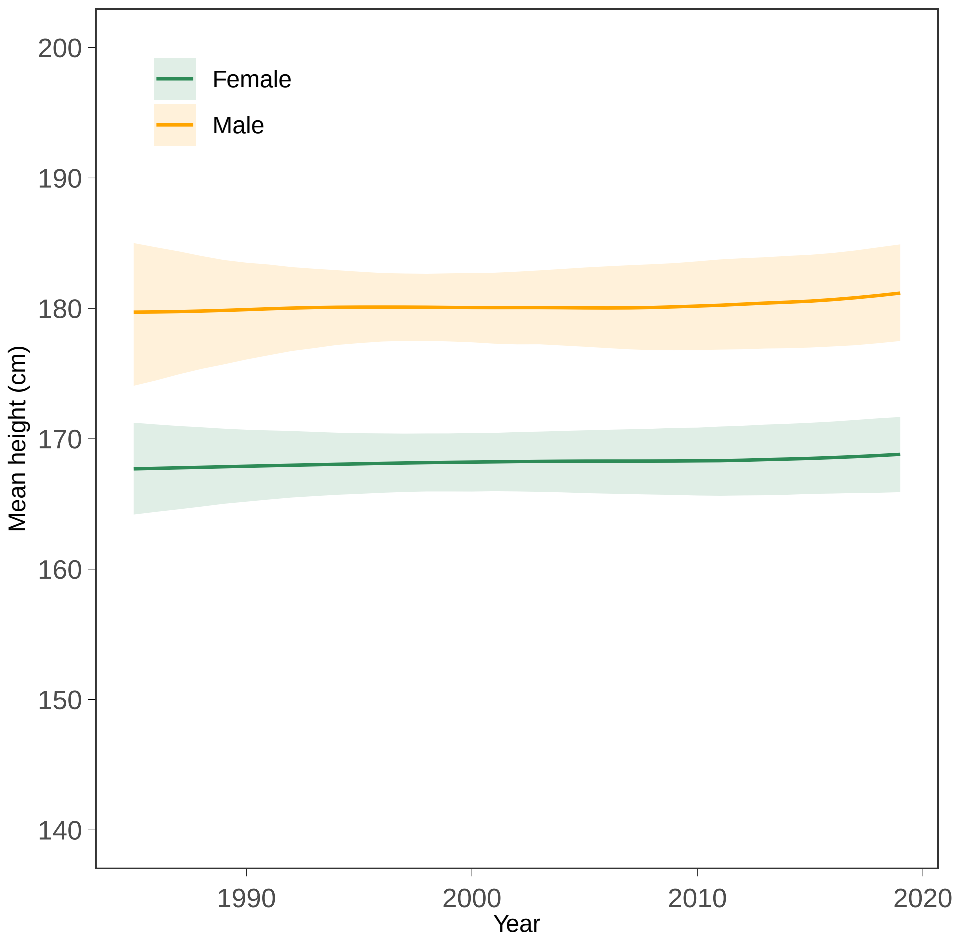

Time trends in BMI of 19 year olds

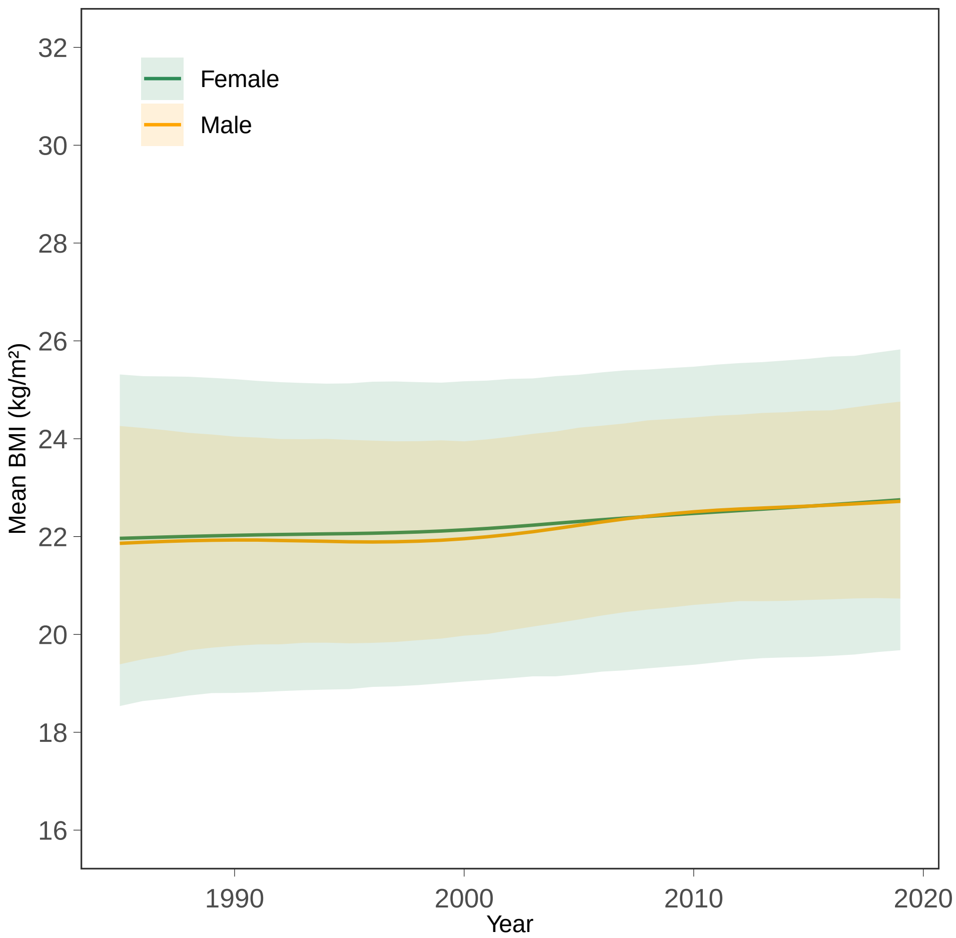

Height−for−age trajectories (2000 birth cohort)

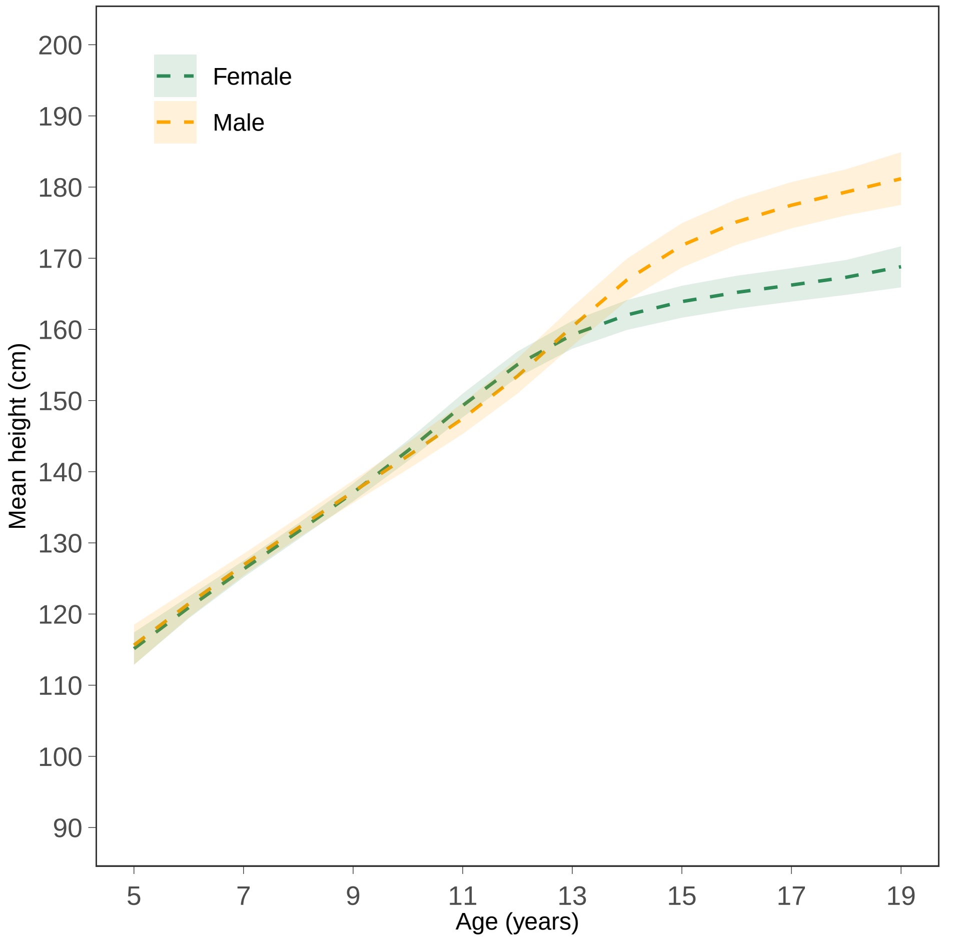

BMI−for−age trajectories (2000 birth cohort)

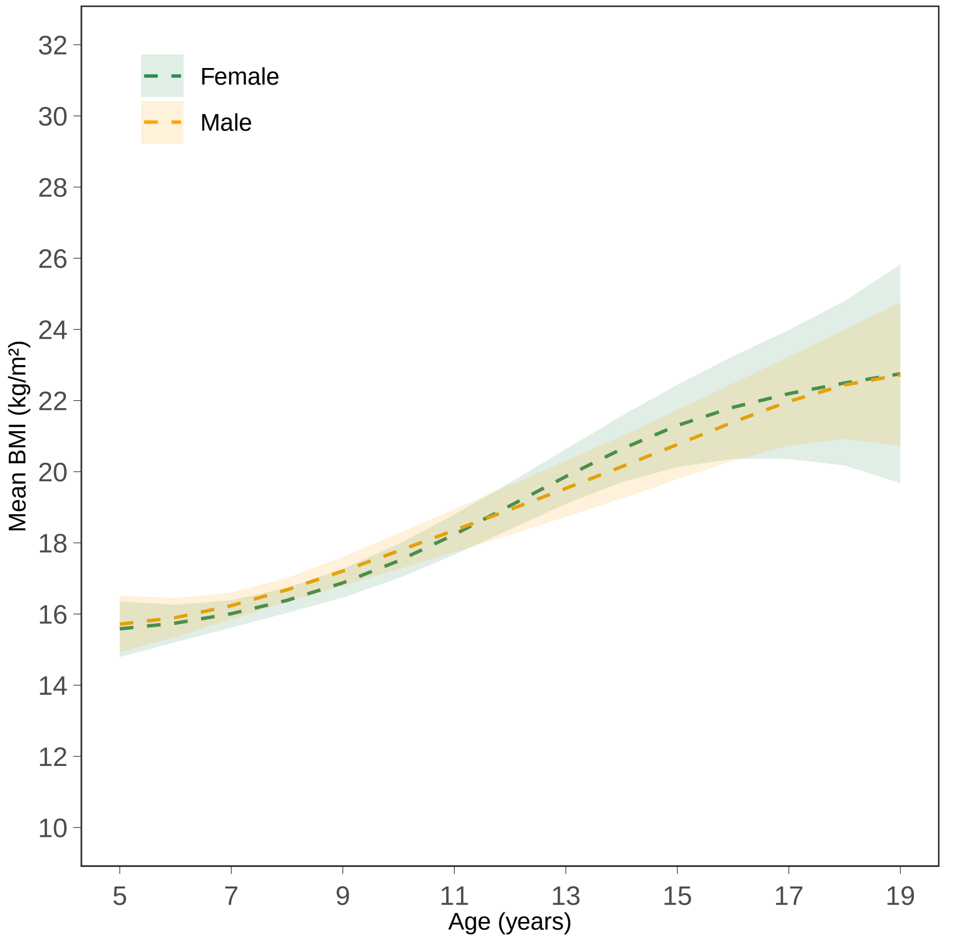

Lebanon

Time trends in height of 19 year olds

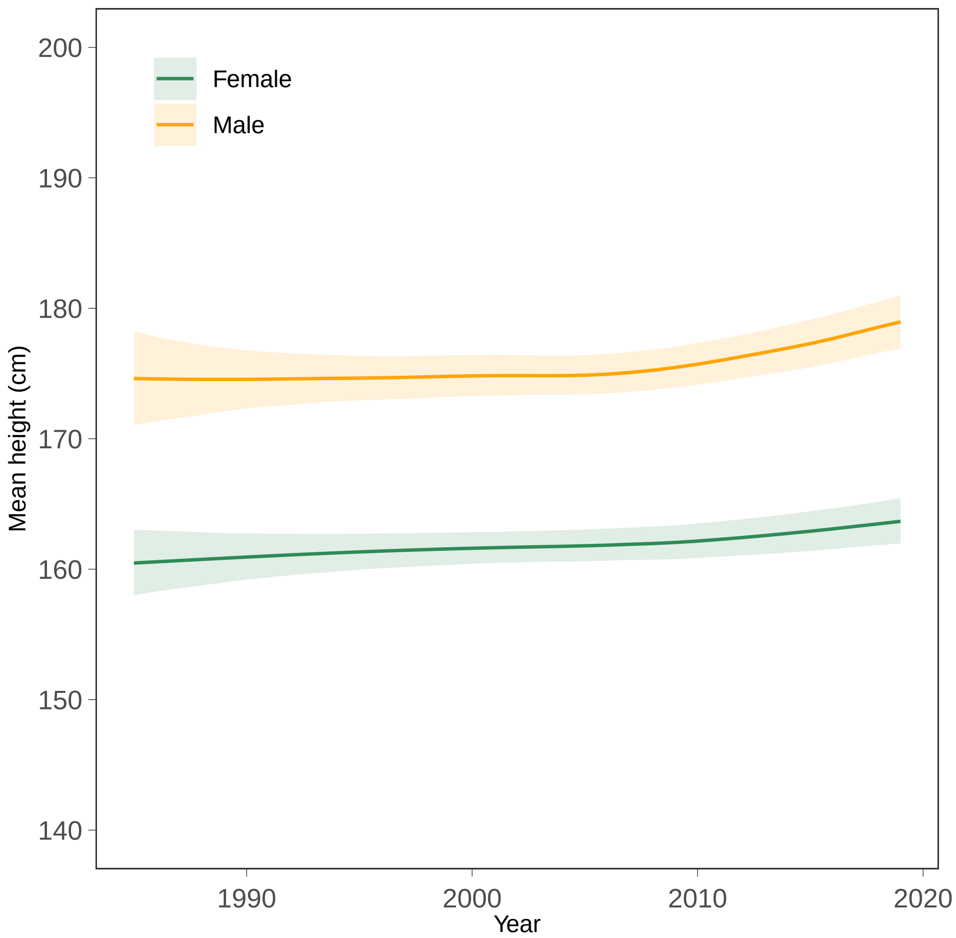

Time trends in BMI of 19 year olds

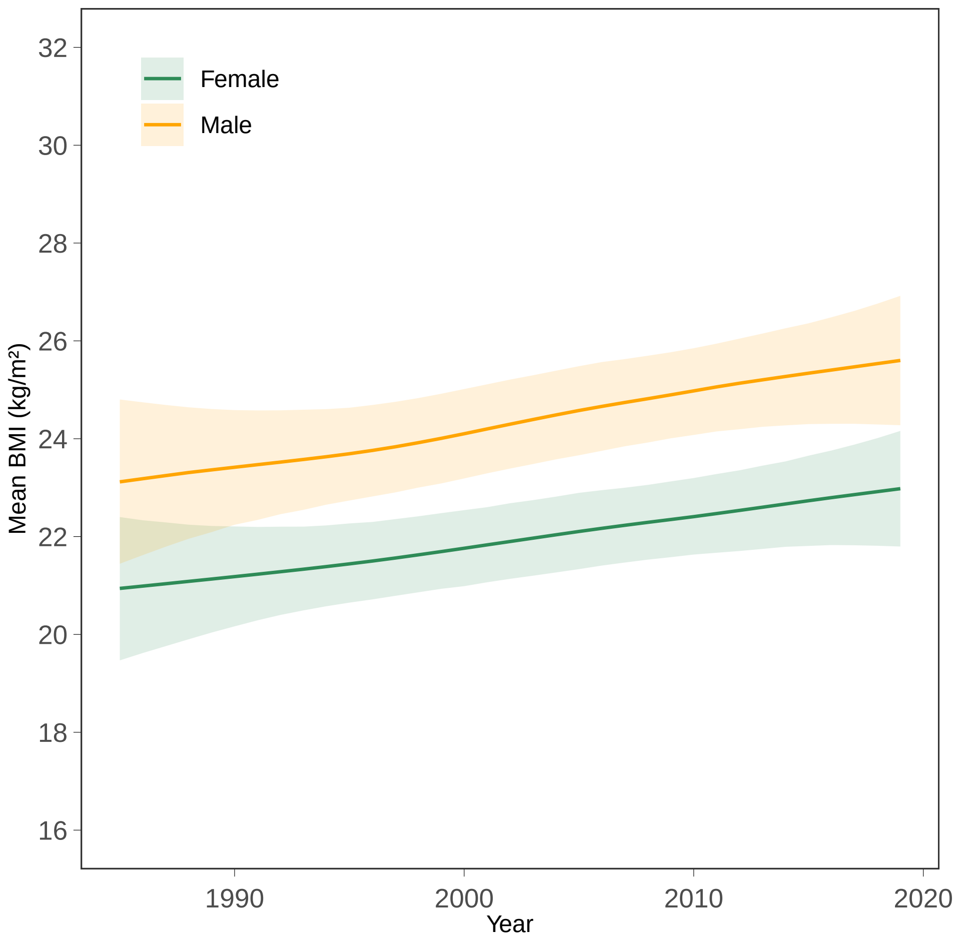

Height−for−age trajectories (2000 birth cohort)

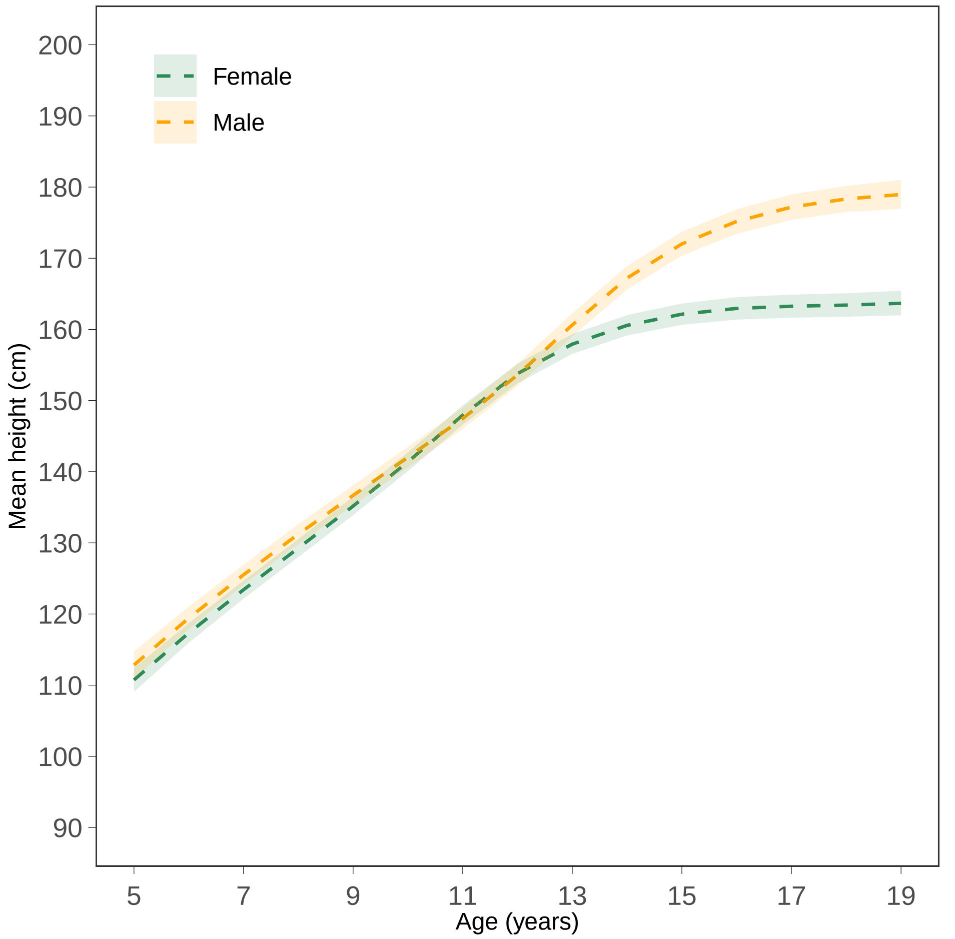

BMI−for−age trajectories (2000 birth cohort)

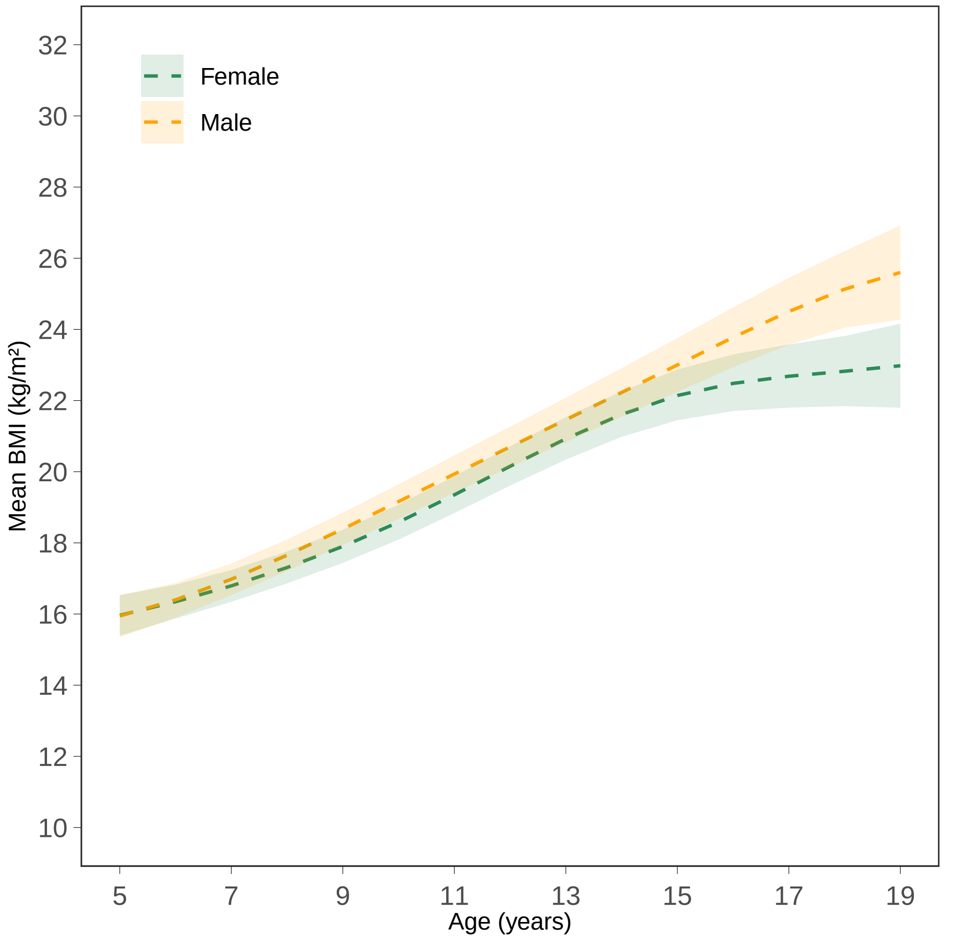

Lesotho

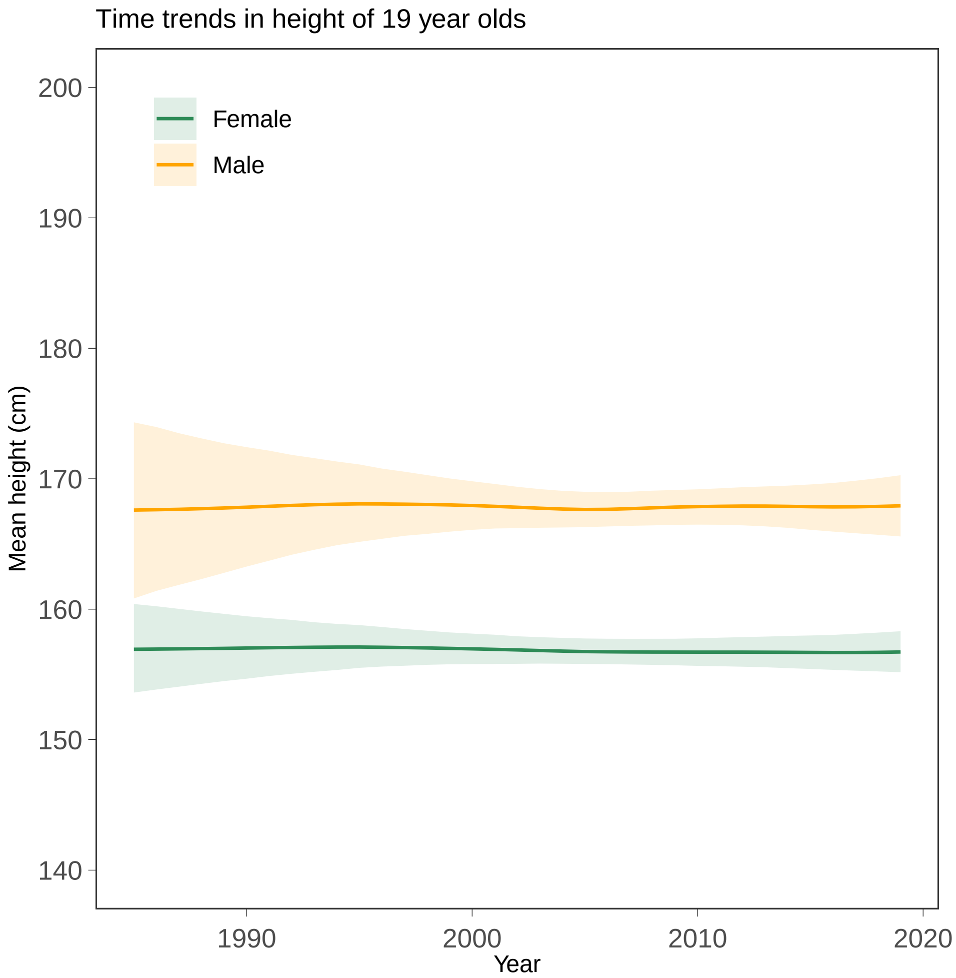

Time trends in height of 19 year olds
Female
Male
Mean height (cm)
200
190
180
170
160
150
140
1990
2000
2010
2020
Year

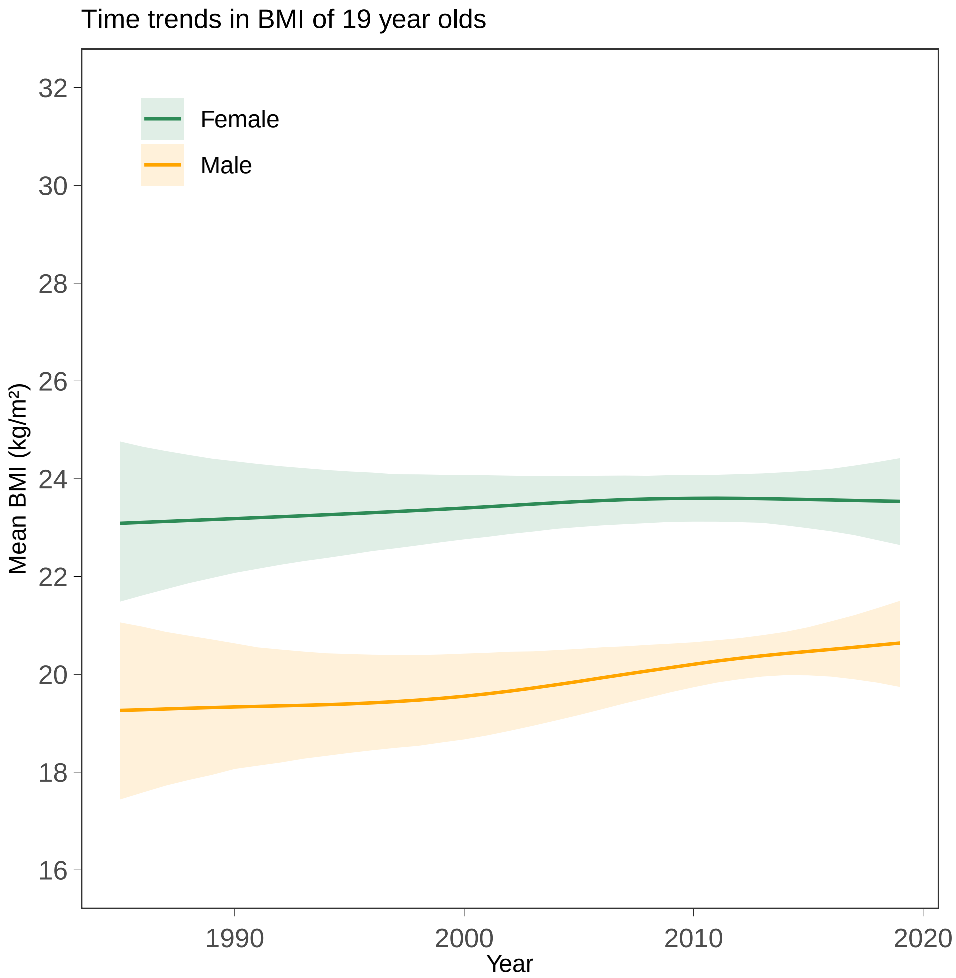

Time trends in BMI of 19 year olds
Female
Male
Mean BMI (kg/m²)
32
30
28
26
24
22
20
18
16
1990
2000
2010
2020
Year

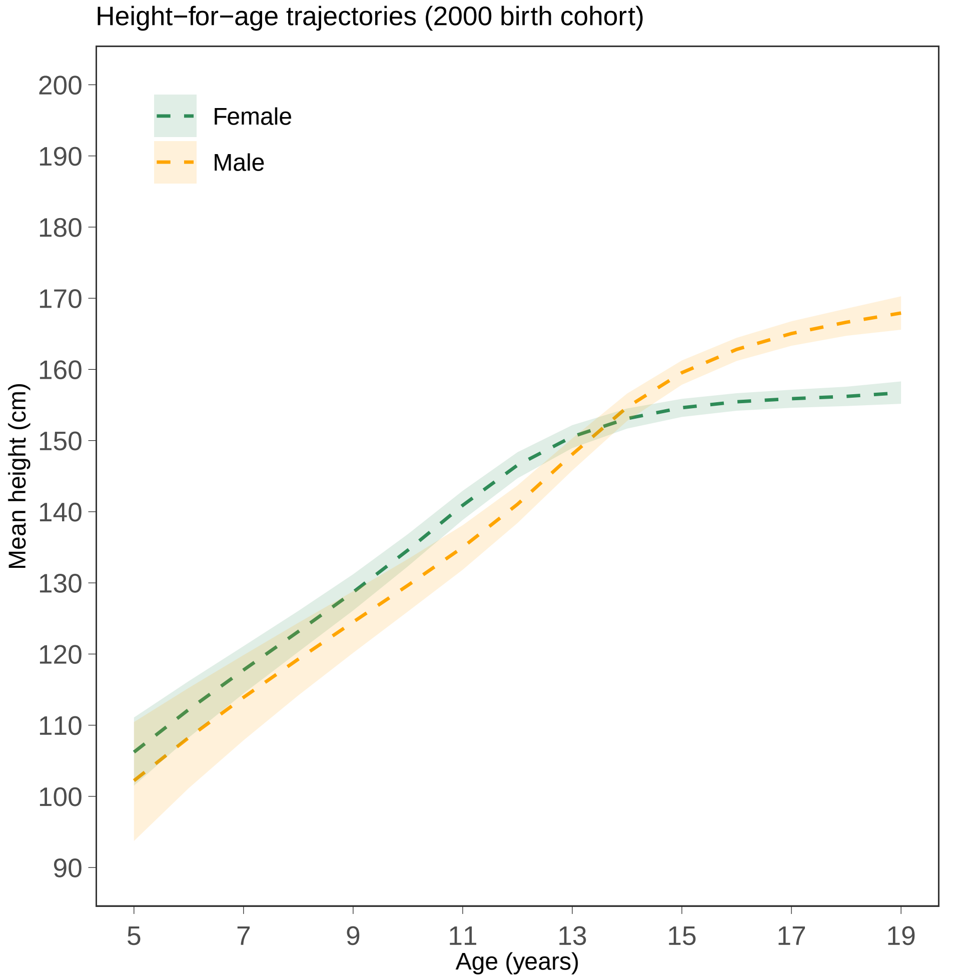

Height−for−age trajectories (2000 birth cohort)
Female
Male
Mean height (cm)
200
190
180
170
160
150
140
130
120
110
100
90
5
7
9
11
13
15
17
19
Age (years)

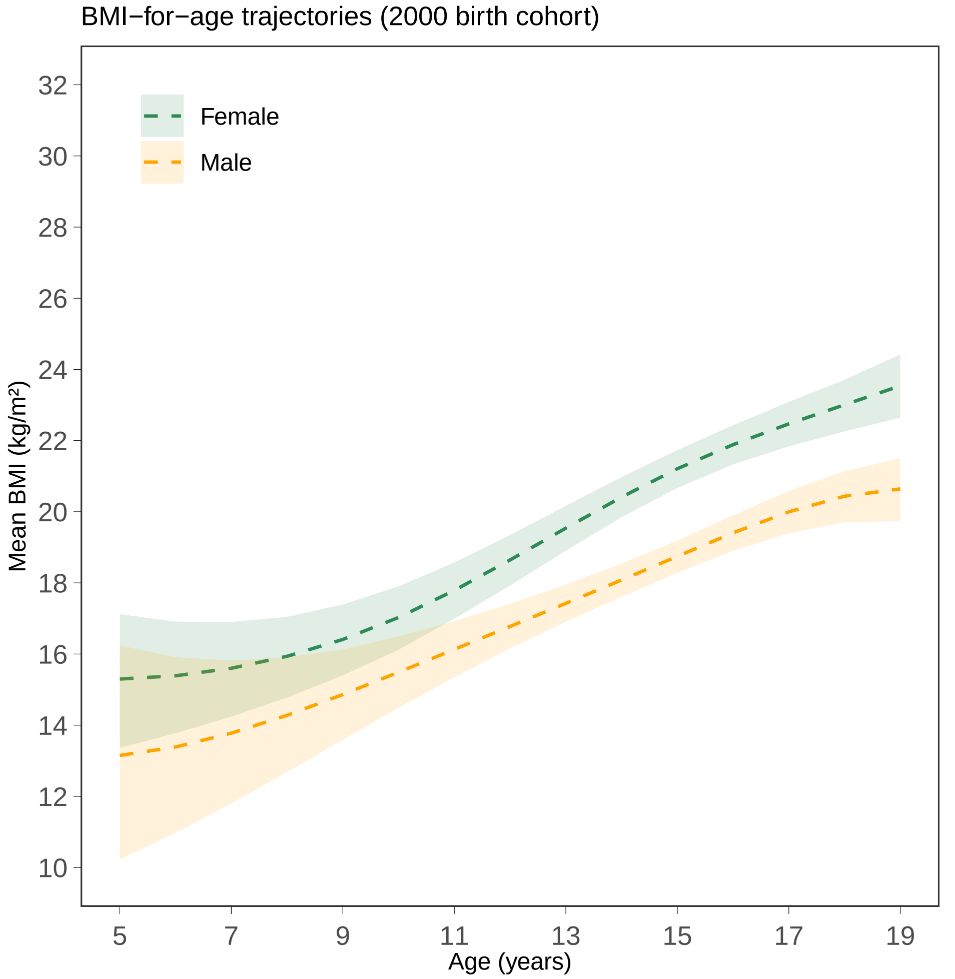

BMI−for−age trajectories (2000 birth cohort)
Female
Male
Mean BMI (kg/m²)
32
30
28
26
24
22
20
18
16
14
12
10
5
7
9
11
13
15
17
19
Age (years)

Liberia

Time trends in height of 19 year olds

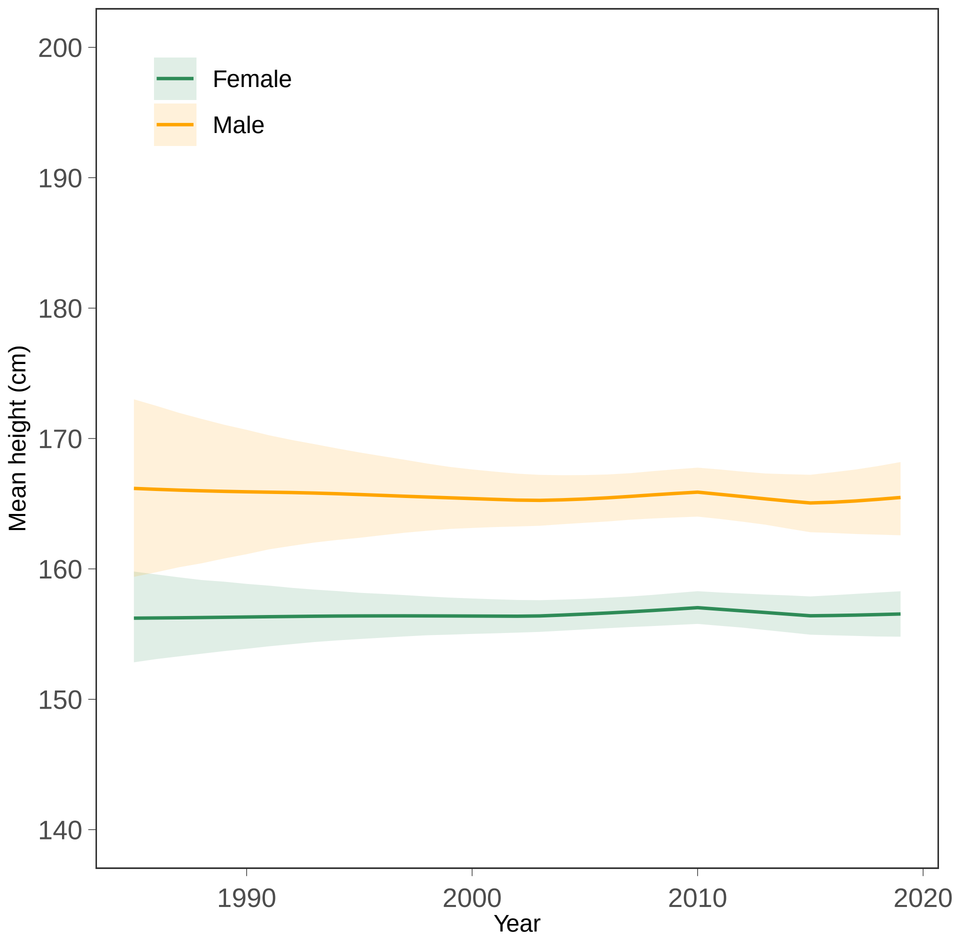

Time trends in BMI of 19 year olds

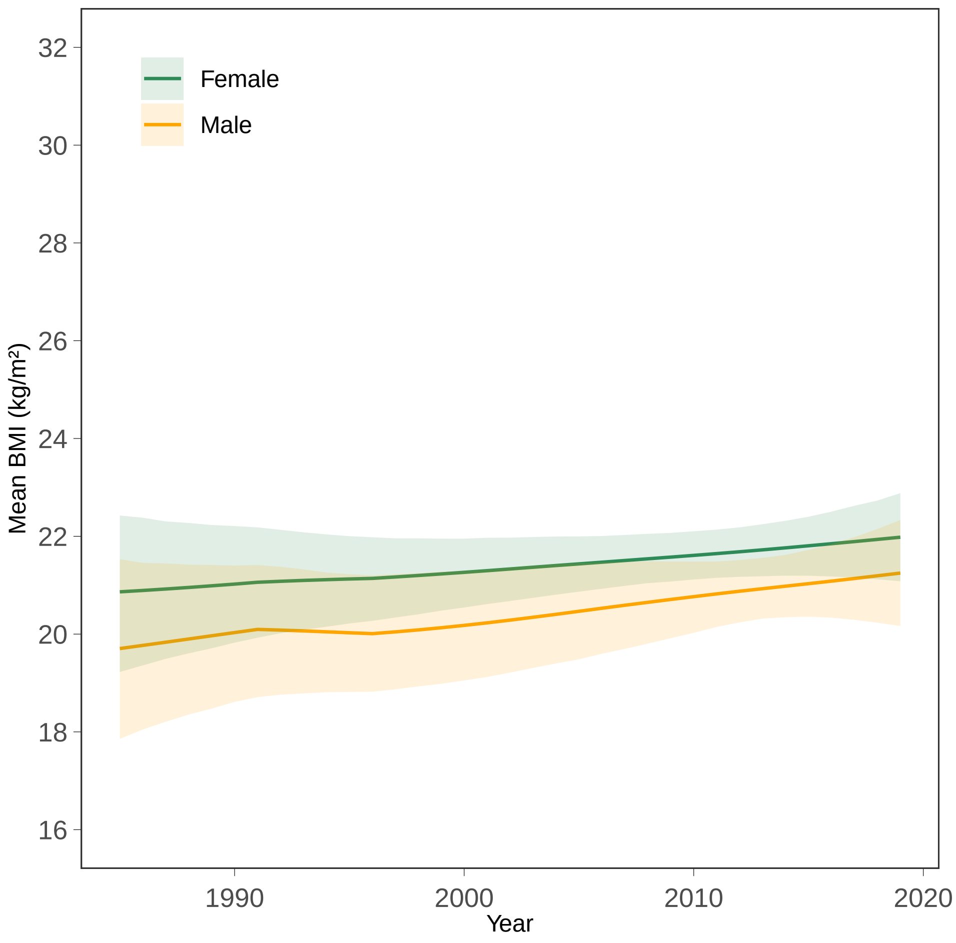

Height−for−age trajectories (2000 birth cohort)

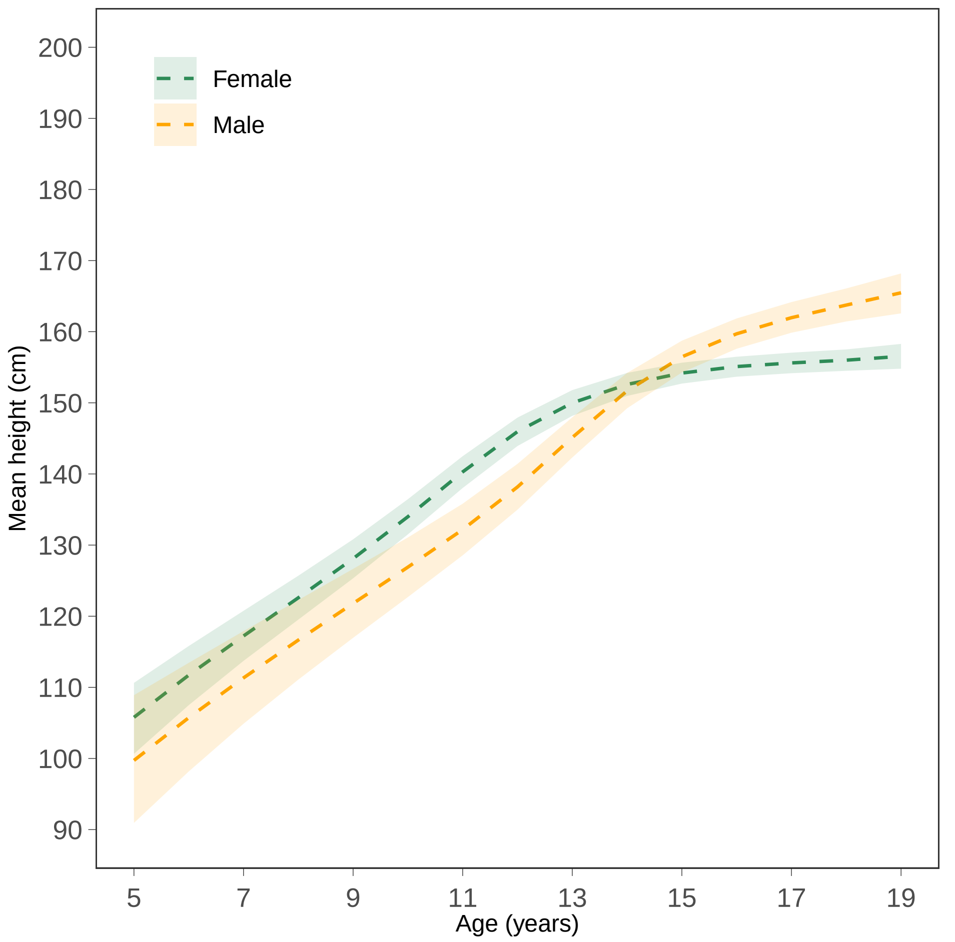

BMI−for−age trajectories (2000 birth cohort)

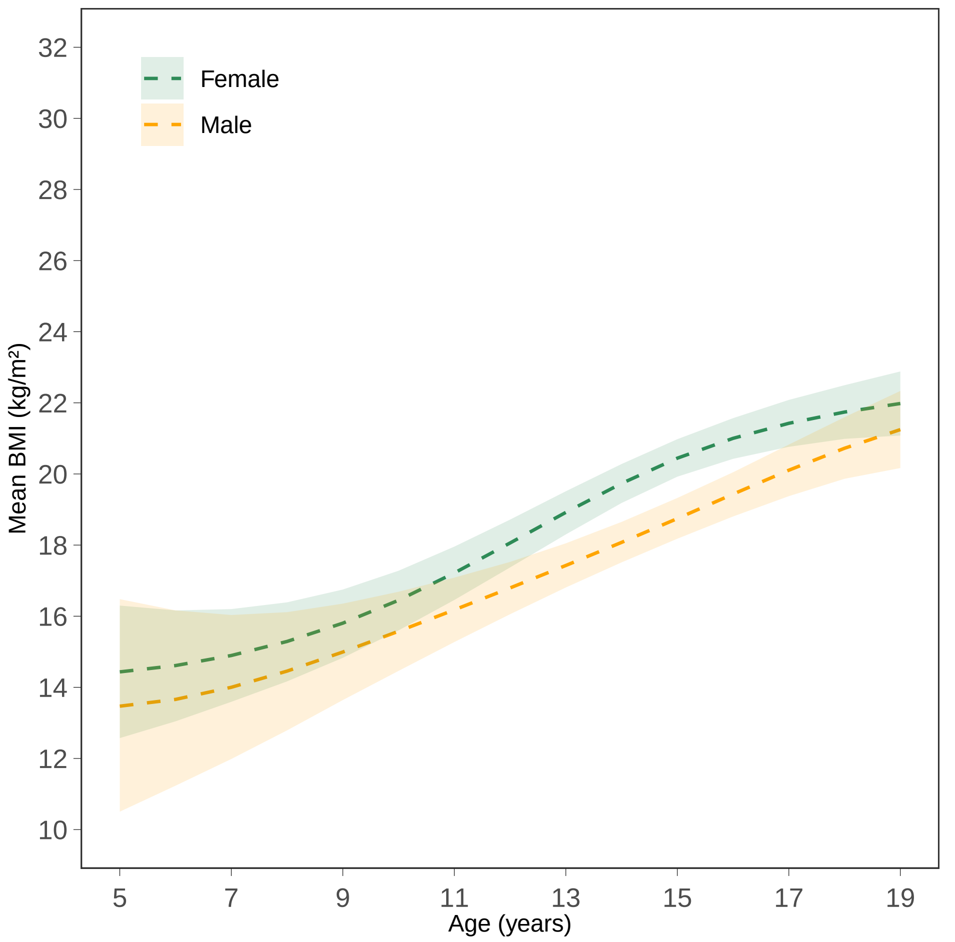

Libya

Time trends in height of 19 year olds

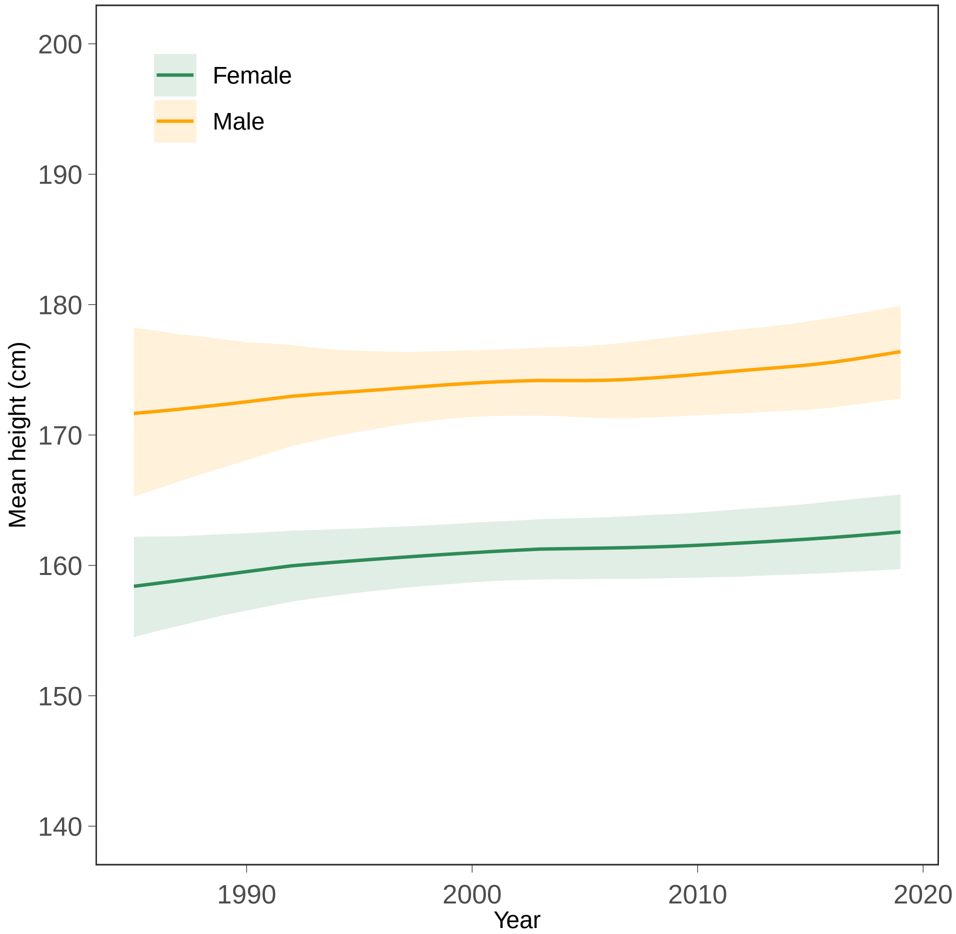

Time trends in BMI of 19 year olds

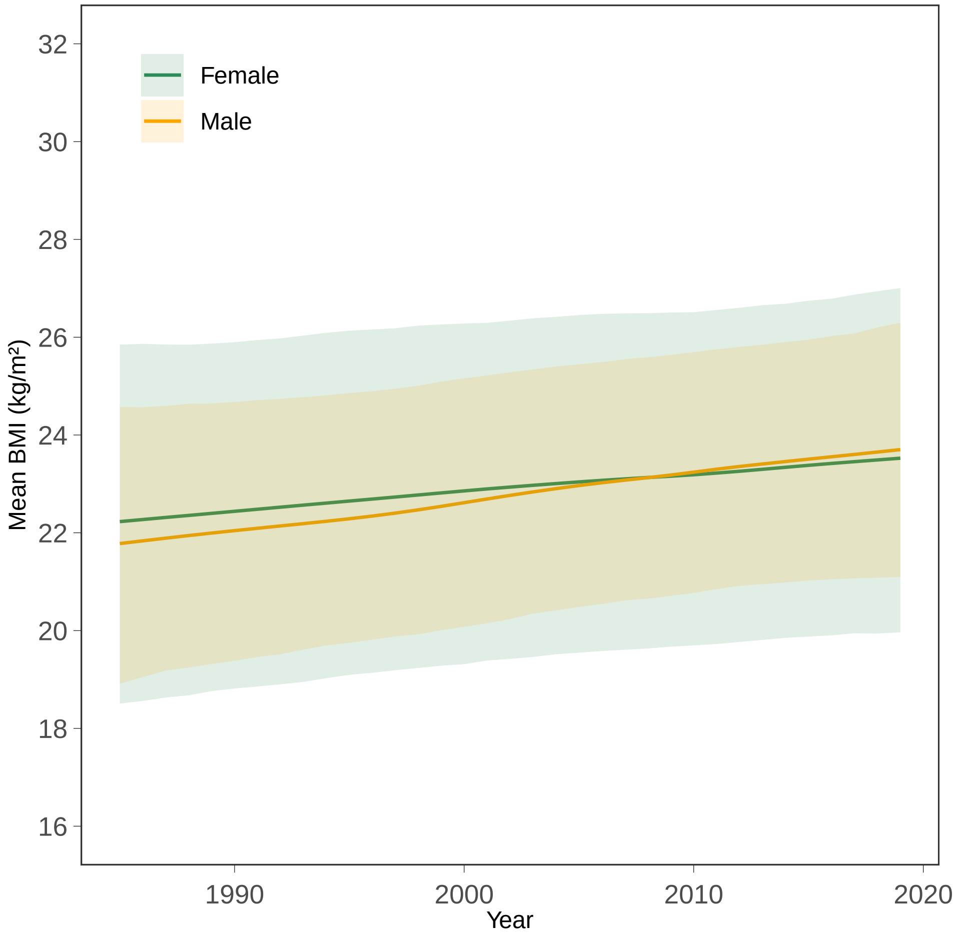

Height−for−age trajectories (2000 birth cohort)

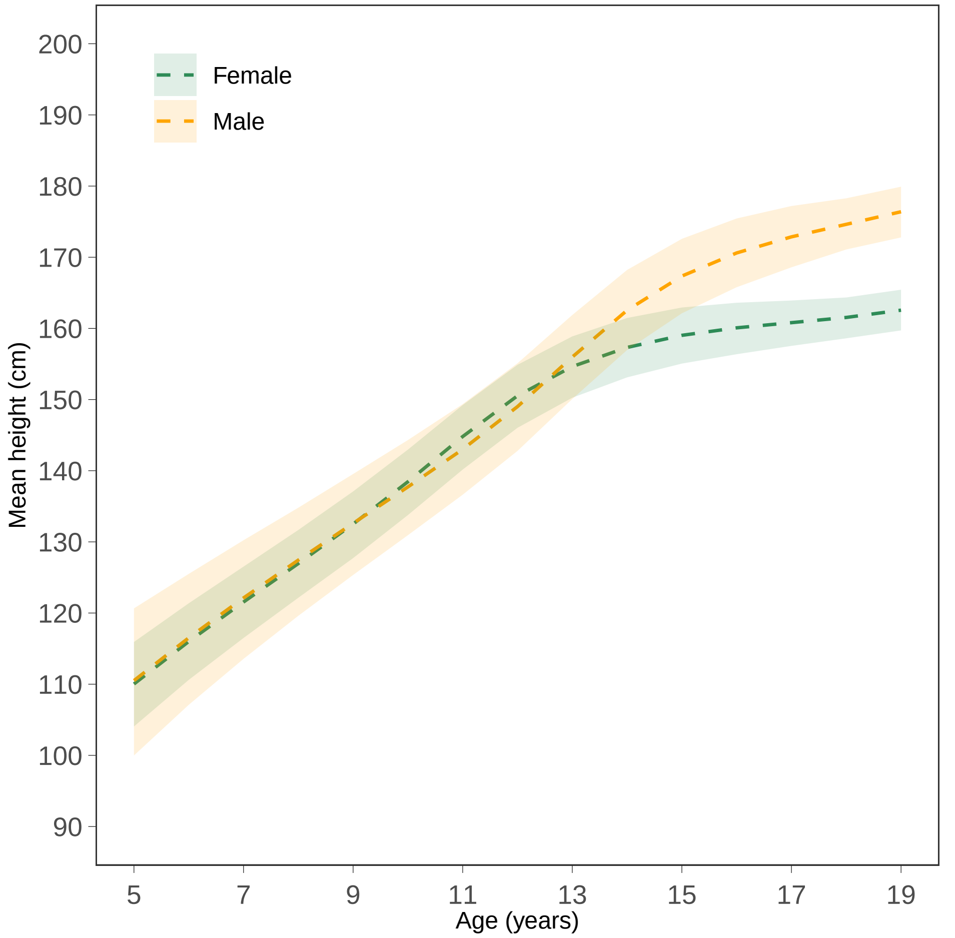

BMI−for−age trajectories (2000 birth cohort)

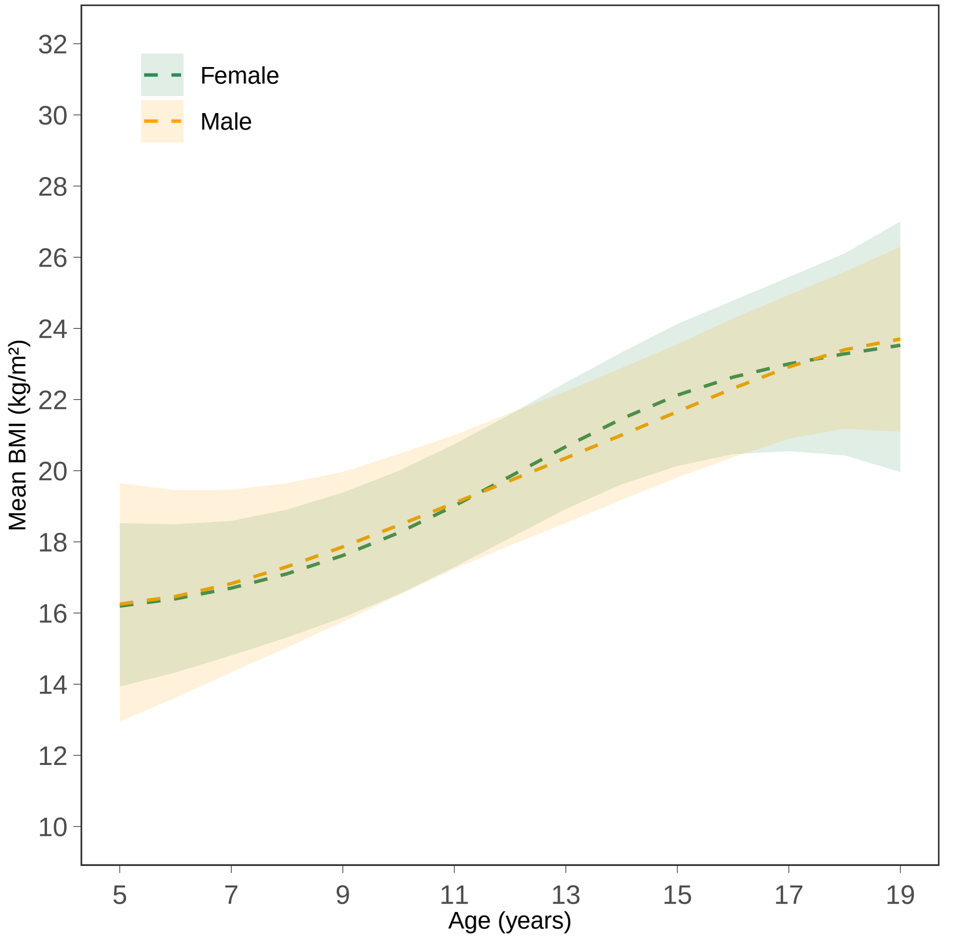

Lithuania

Time trends in height of 19 year olds

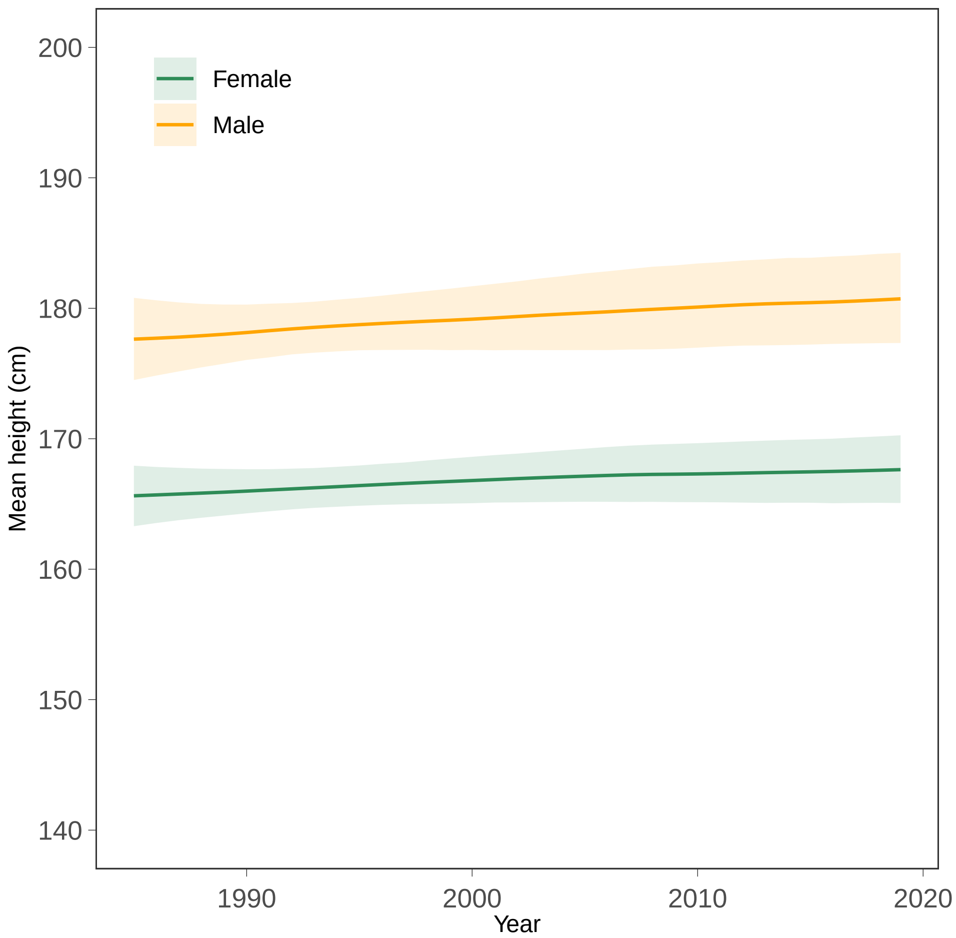

Time trends in BMI of 19 year olds

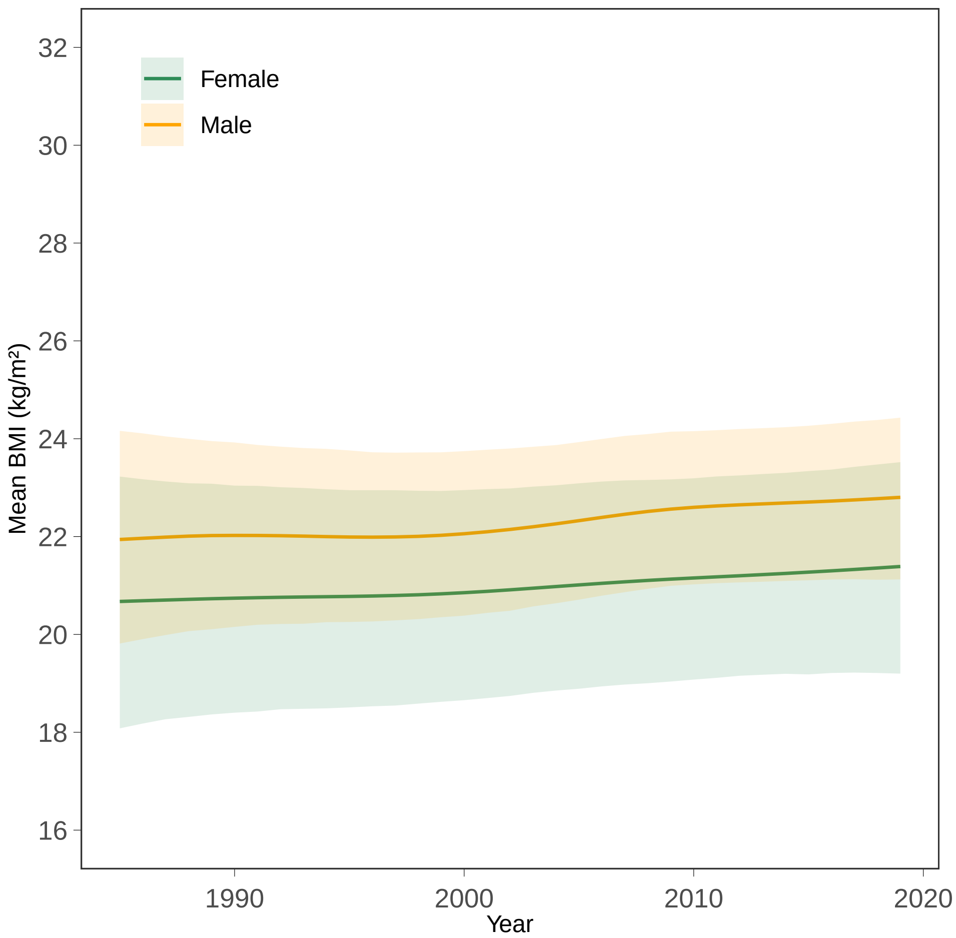

Height−for−age trajectories (2000 birth cohort)

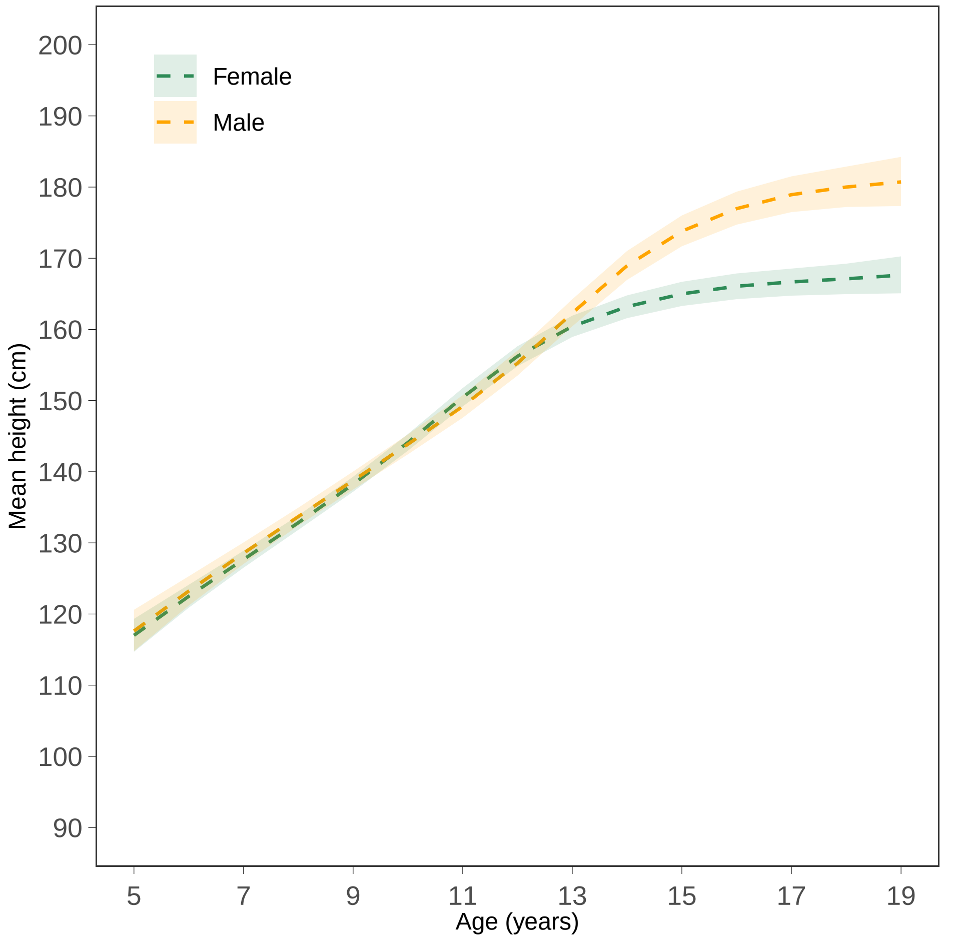

BMI−for−age trajectories (2000 birth cohort)

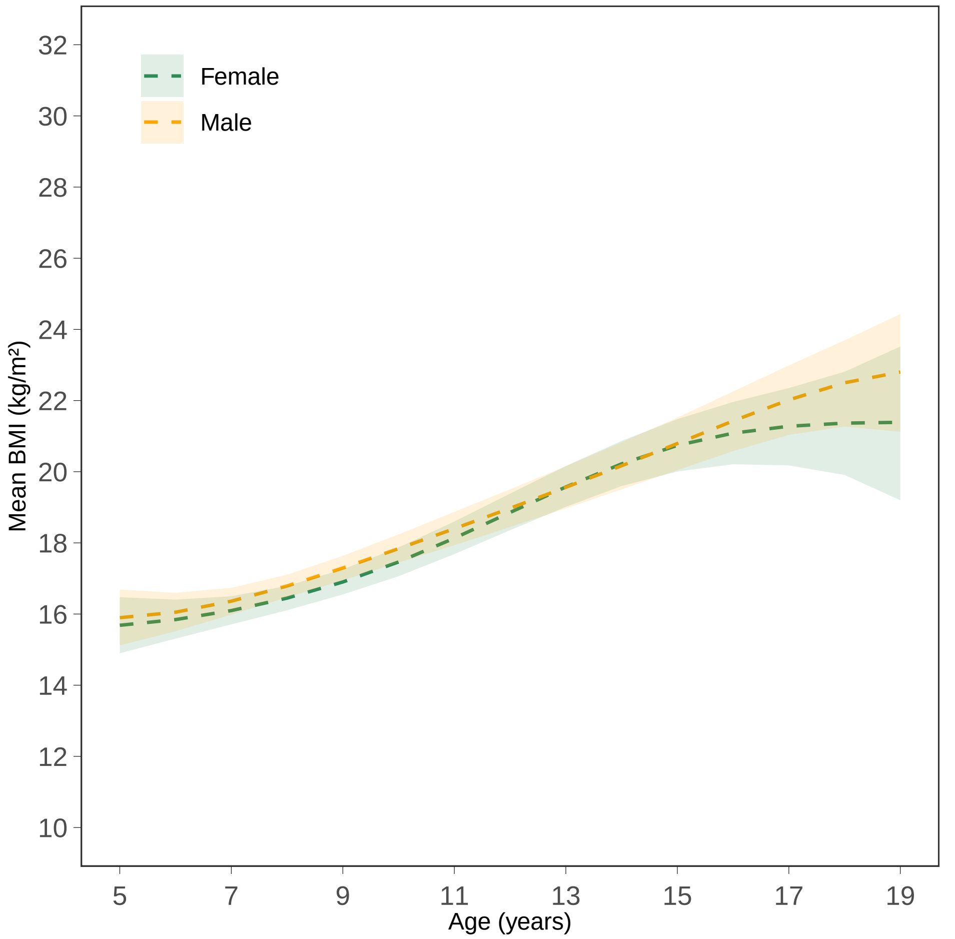

Luxembourg

Time trends in height of 19 year olds

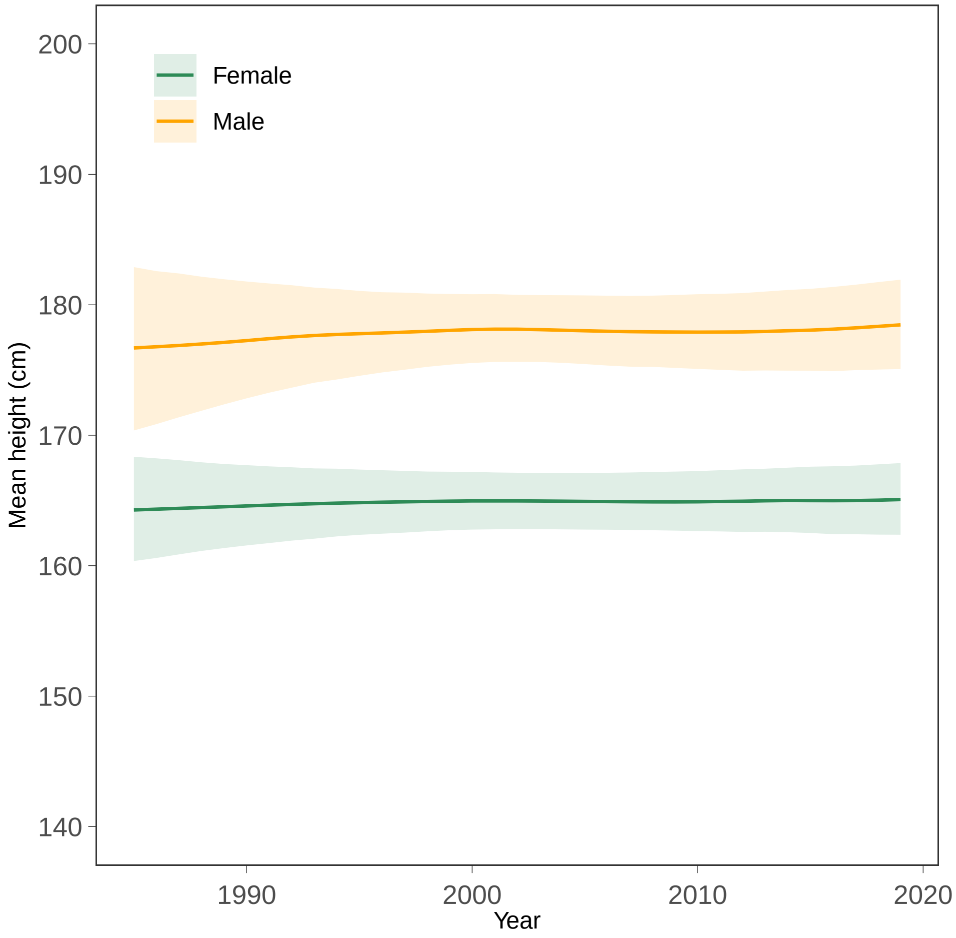

Time trends in BMI of 19 year olds

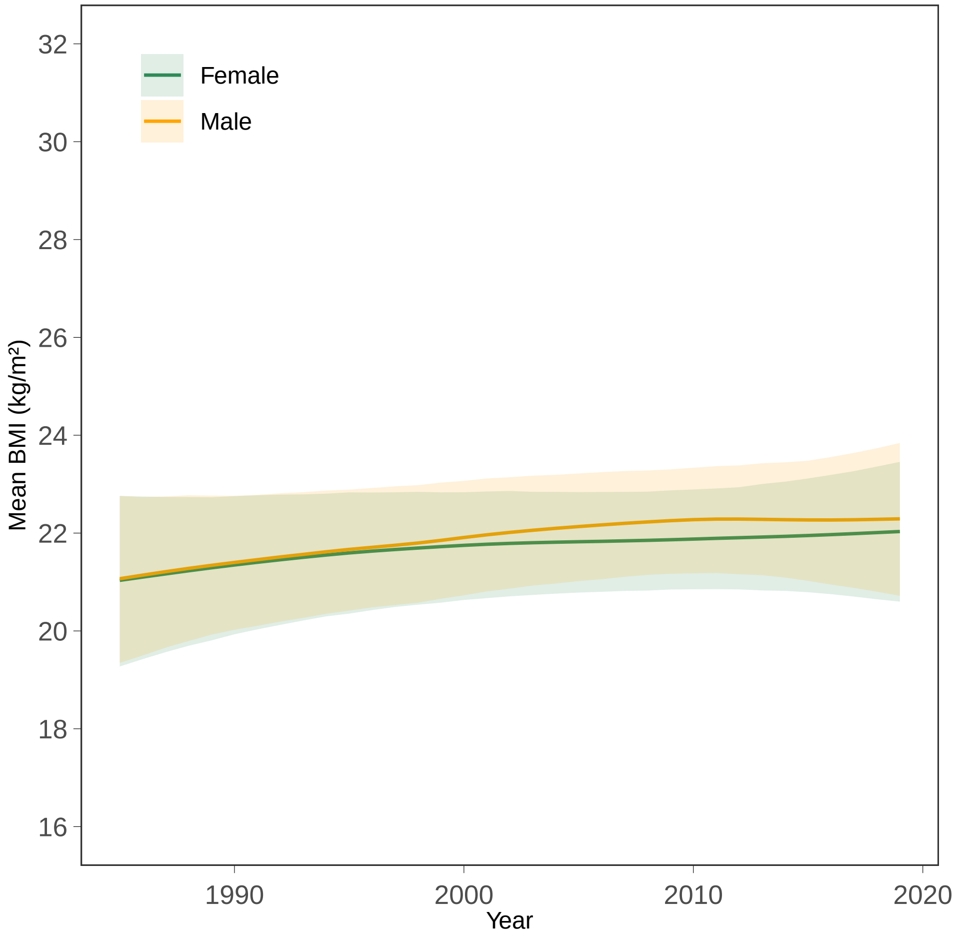

Height−for−age trajectories (2000 birth cohort)

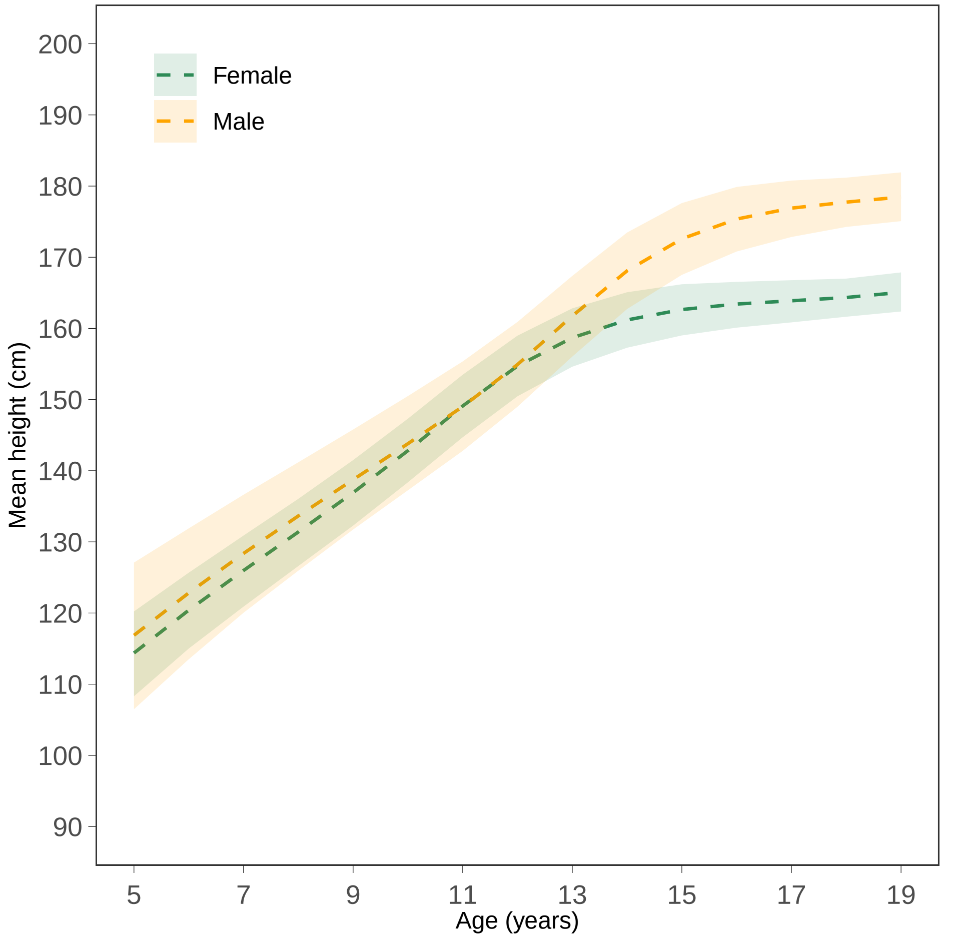

BMI−for−age trajectories (2000 birth cohort)

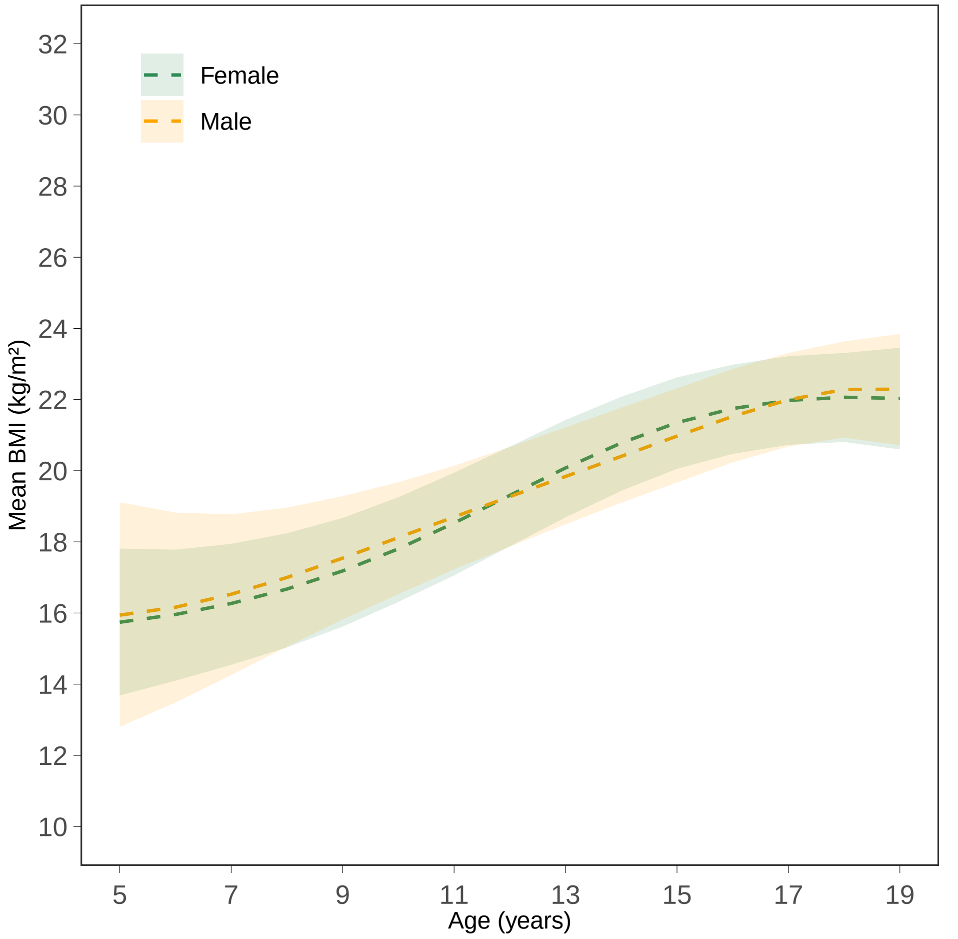

Madagascar

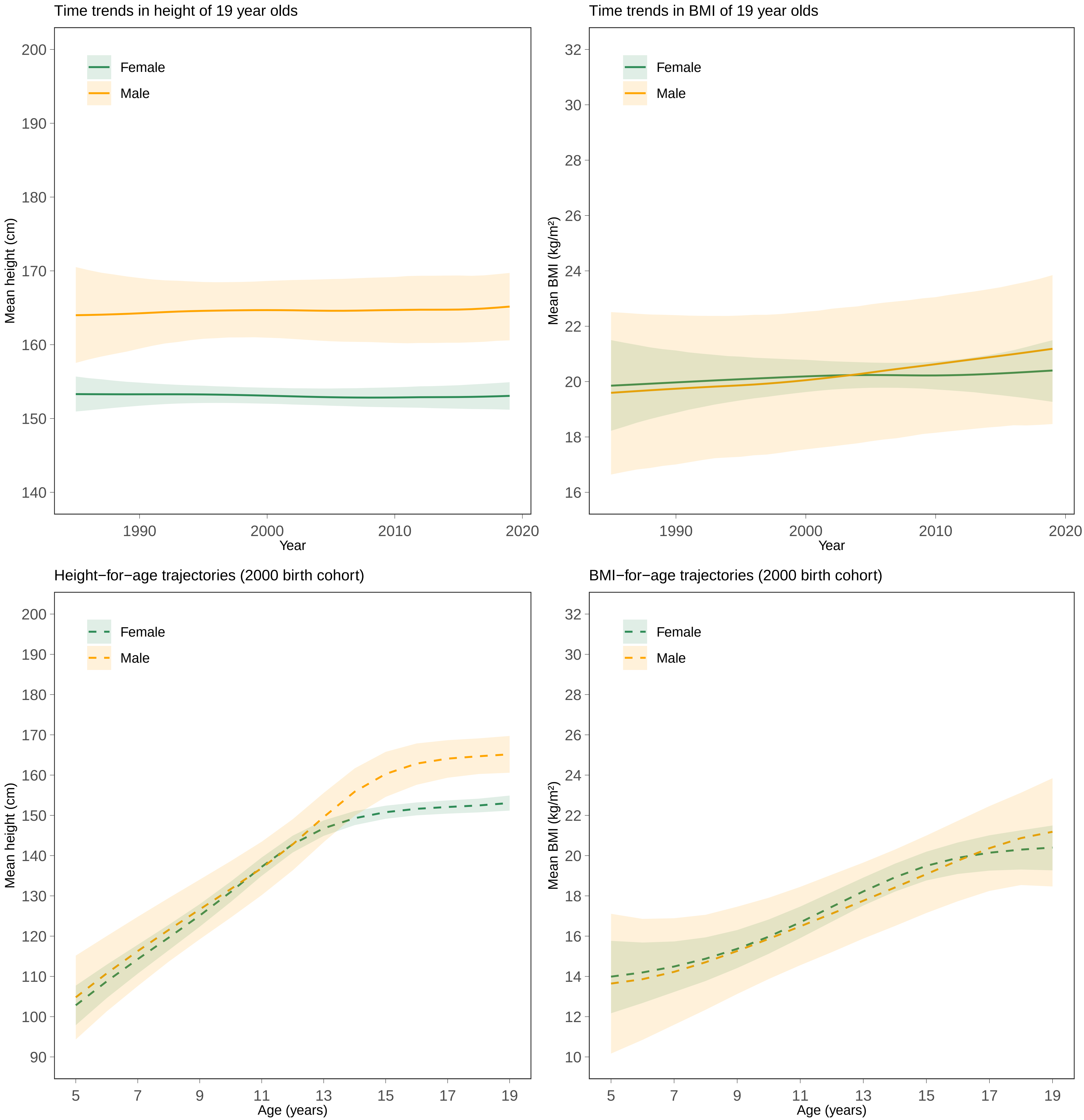

Time trends in height of 19 year olds
Female
Male
Mean height (cm)
200
190
180
170
160
150
140
1990
2000
2010
2020
Year
Time trends in BMI of 19 year olds
Female
Male
Mean BMI (kg/m²)
32
30
28
26
24
22
20
18
16
1990
2000
2010
2020
Year
Height−for−age trajectories (2000 birth cohort)
Female
Male
Mean height (cm)
200
190
180
170
160
150
140
130
120
110
100
90
5
7
9
11
13
15
17
19
Age (years)
BMI−for−age trajectories (2000 birth cohort)
Female
Male
Mean BMI (kg/m²)
32
30
28
26
24
22
20
18
16
14
12
10
5
7
9
11
13
15
17
19
Age (years)

Malawi

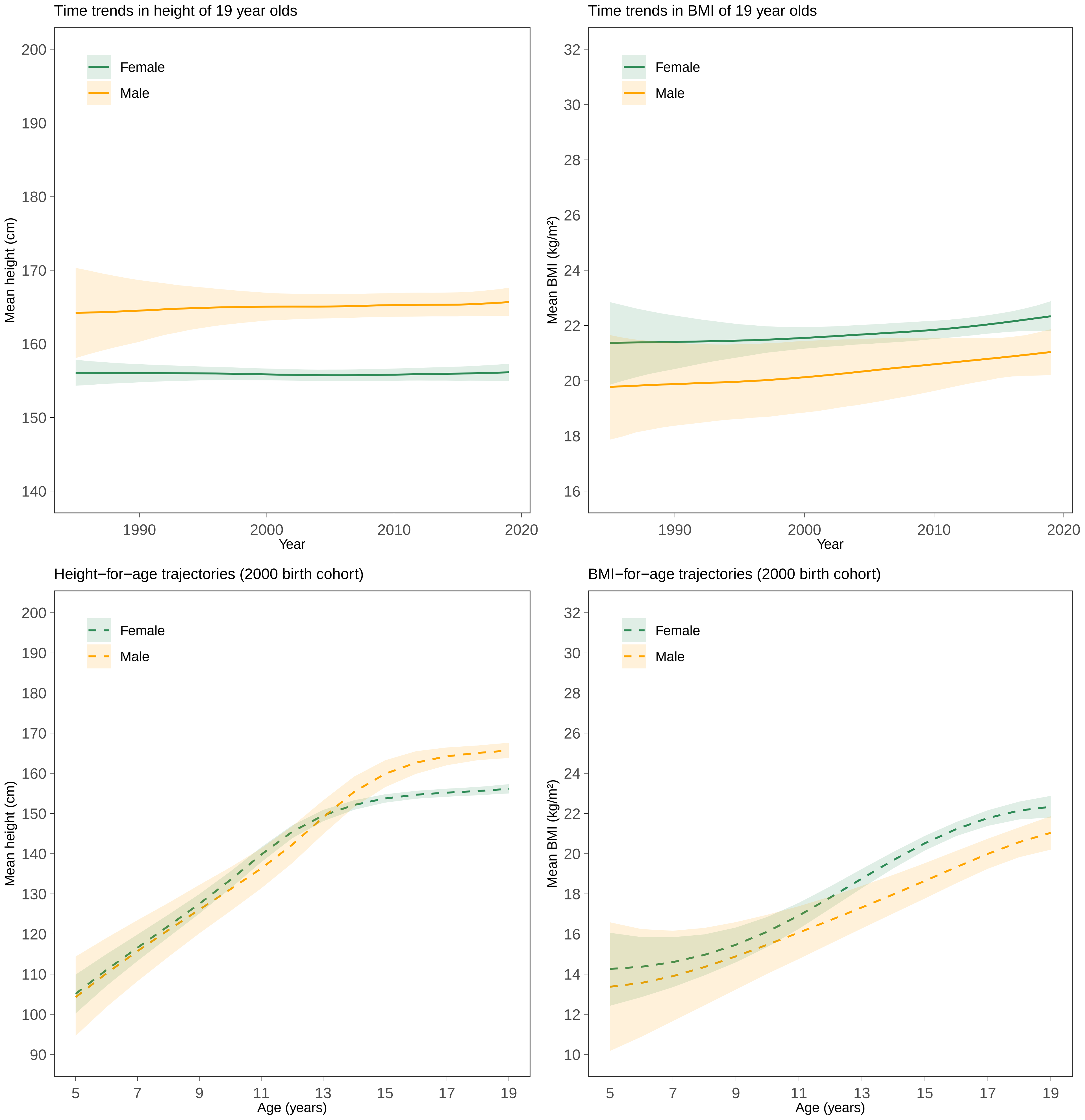
Time trends in height of 19 year olds
Female
Male
Mean height (cm)
200
190
180
170
160
150
140
1990
2000
2010
2020
Year
Time trends in BMI of 19 year olds
Female
Male
Mean BMI (kg/m²)
32
30
28
26
24
22
20
18
16
1990
2000
2010
2020
Year
Height−for−age trajectories (2000 birth cohort)
Female
Male
Mean height (cm)
200
190
180
170
160
150
140
130
120
110
100
90
5
7
9
11
13
15
17
19
Age (years)
BMI−for−age trajectories (2000 birth cohort)
Female
Male
Mean BMI (kg/m²)
32
30
28
26
24
22
20
18
16
14
12
10
5
7
9
11
13
15
17
19
Age (years)

Malaysia

Time trends in height of 19 year olds

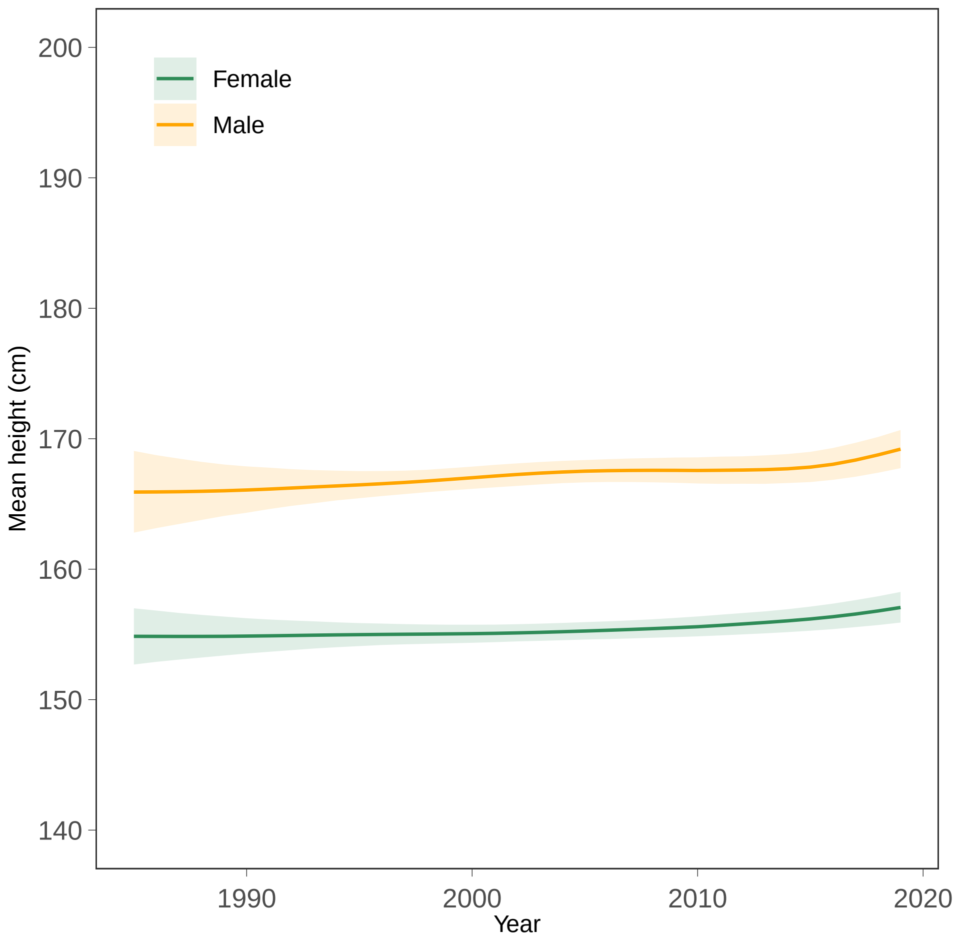

Time trends in BMI of 19 year olds

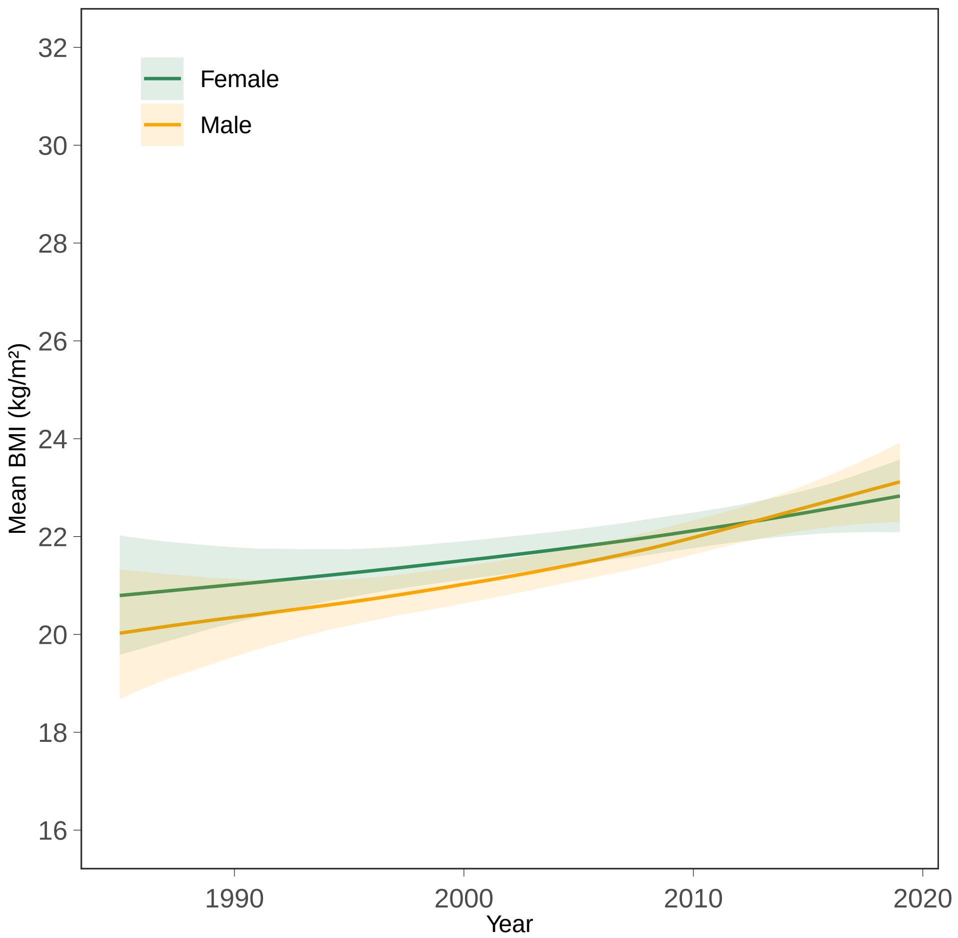

Height−for−age trajectories (2000 birth cohort)

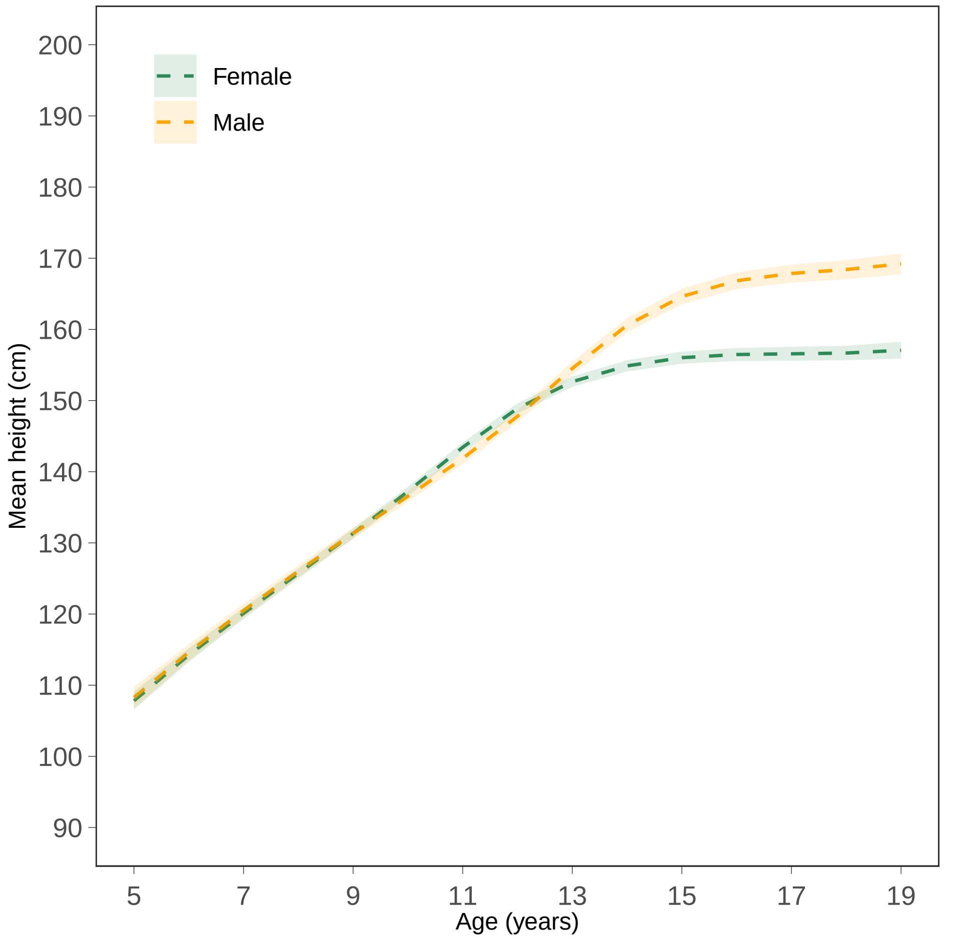

BMI−for−age trajectories (2000 birth cohort)

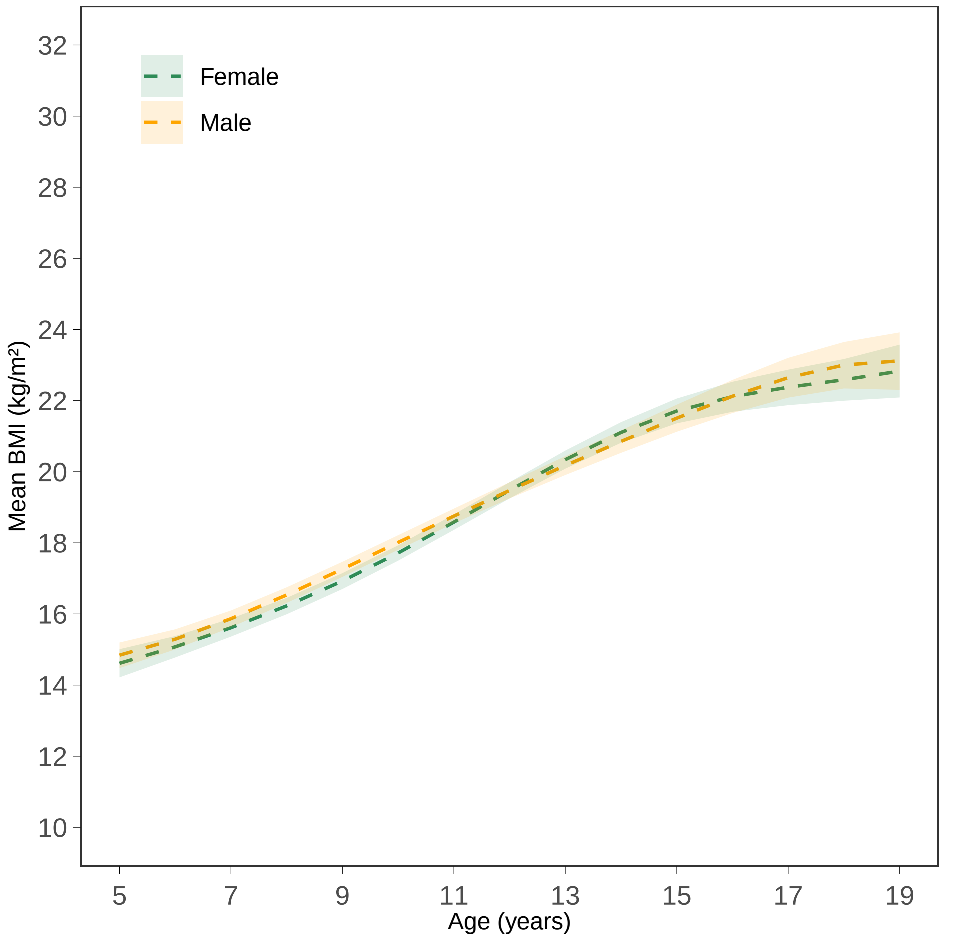

Maldives

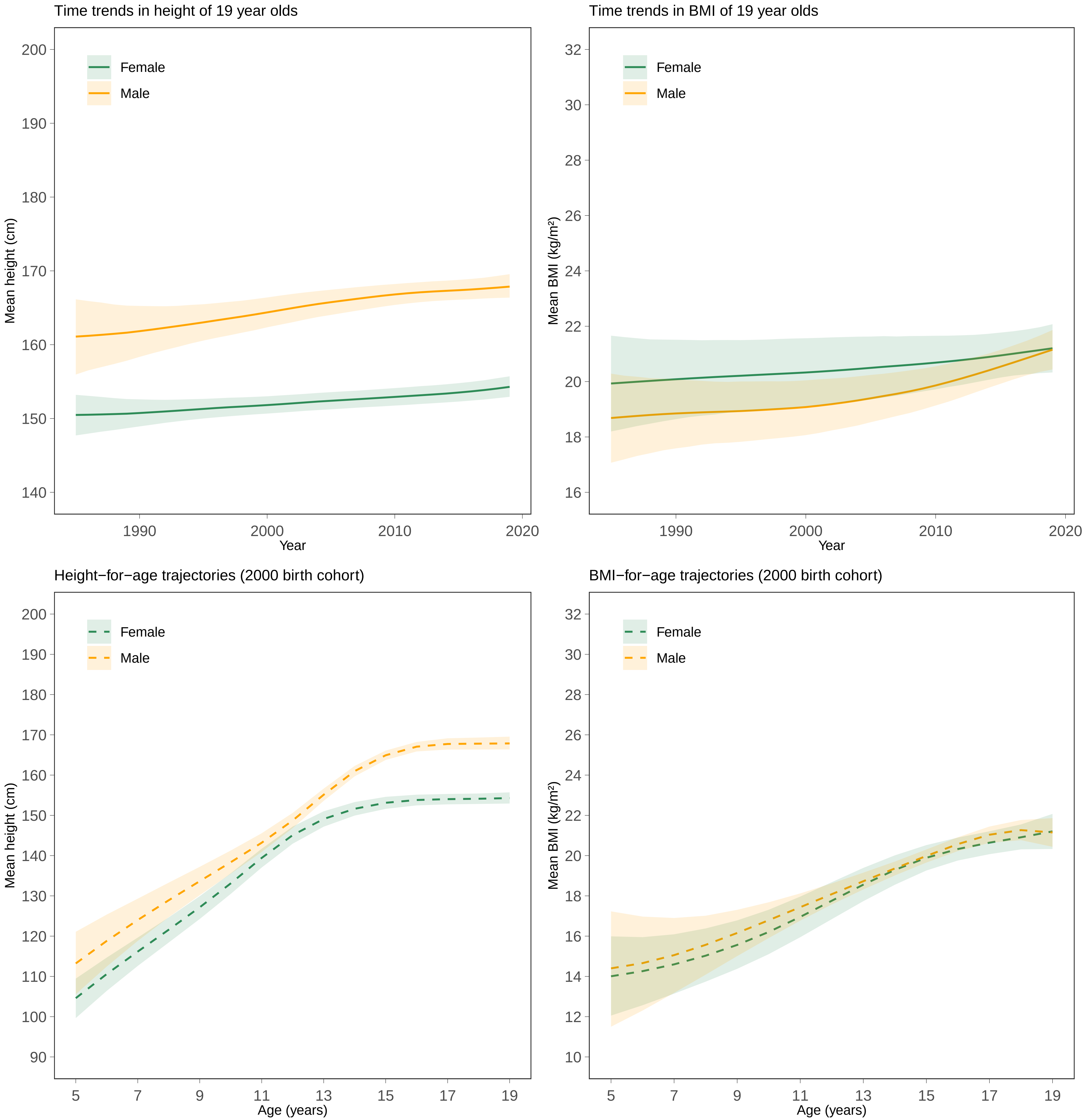

Time trends in height of 19 year olds
Female
Male
Mean height (cm)
200
190
180
170
160
150
140
1990
2000
2010
2020
Year
Time trends in BMI of 19 year olds
Female
Male
Mean BMI (kg/m²)
32
30
28
26
24
22
20
18
16
1990
2000
2010
2020
Year
Height−for−age trajectories (2000 birth cohort)
Female
Male
Mean height (cm)
200
190
180
170
160
150
140
130
120
110
100
90
5
7
9
11
13
15
17
19
Age (years)
BMI−for−age trajectories (2000 birth cohort)
Female
Male
Mean BMI (kg/m²)
32
30
28
26
24
22
20
18
16
14
12
10
5
7
9
11
13
15
17
19
Age (years)

Mali

Time trends in height of 19 year olds

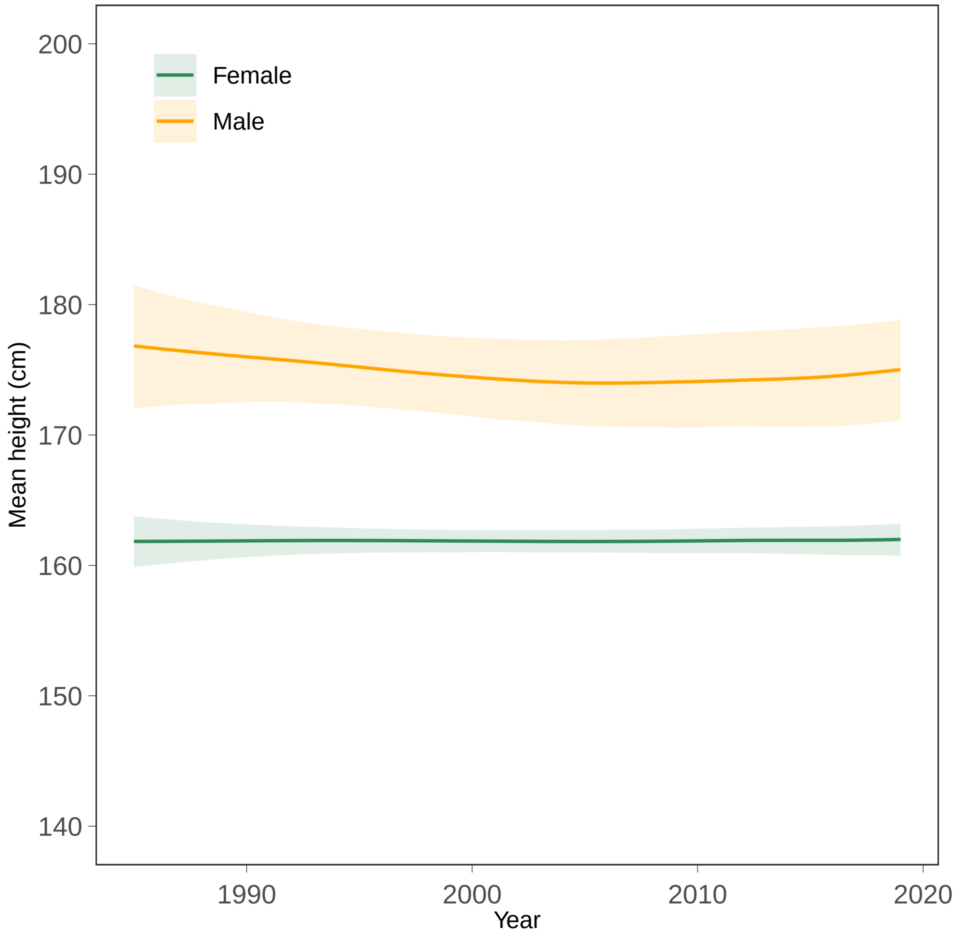

Time trends in BMI of 19 year olds

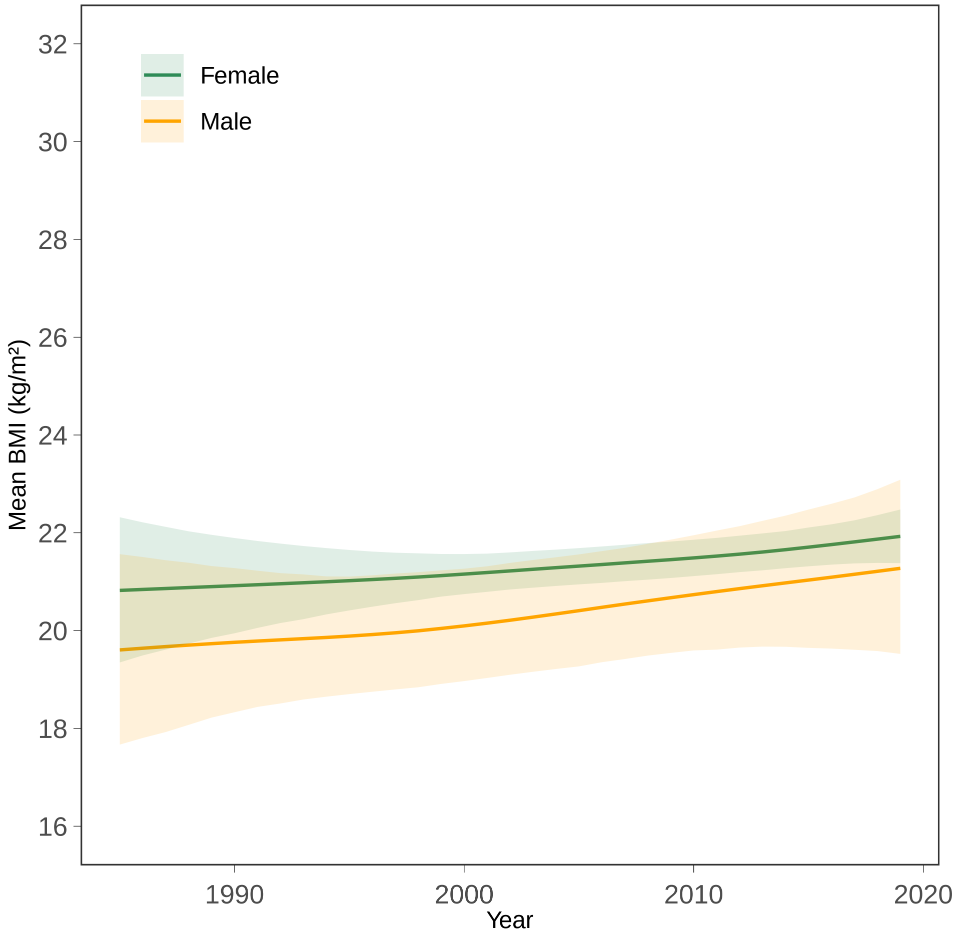

Height−for−age trajectories (2000 birth cohort)

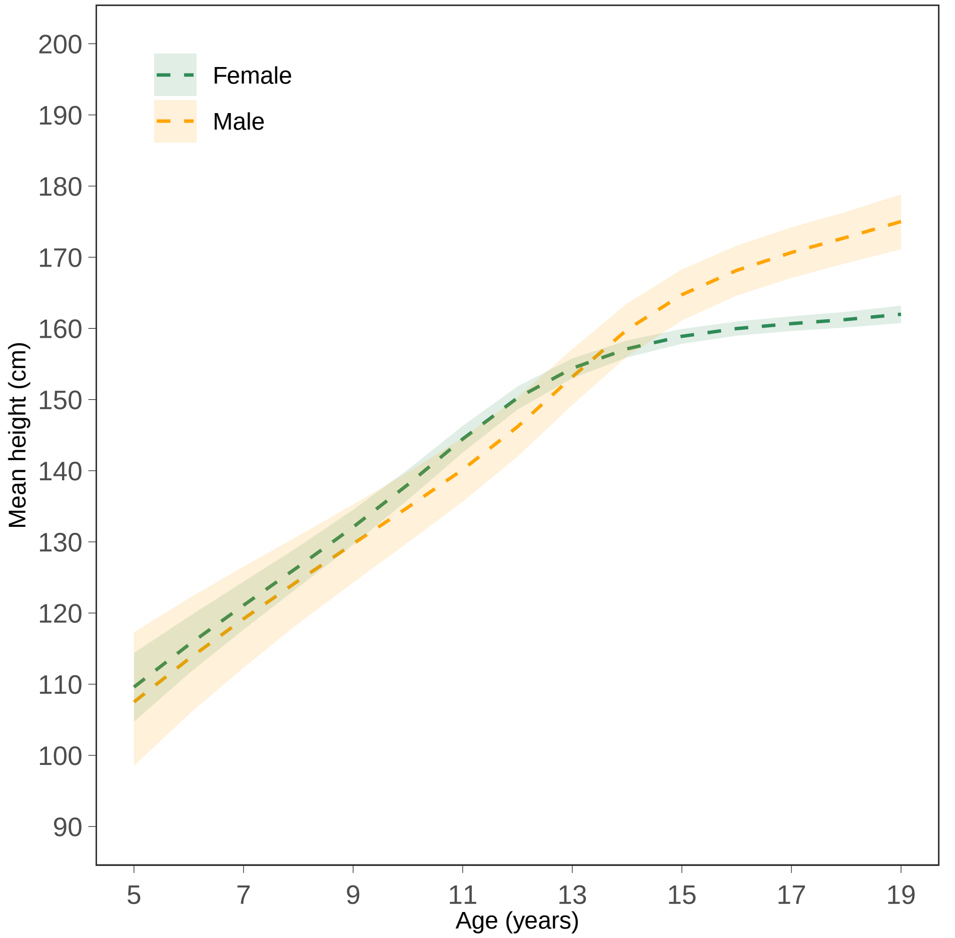

BMI−for−age trajectories (2000 birth cohort)

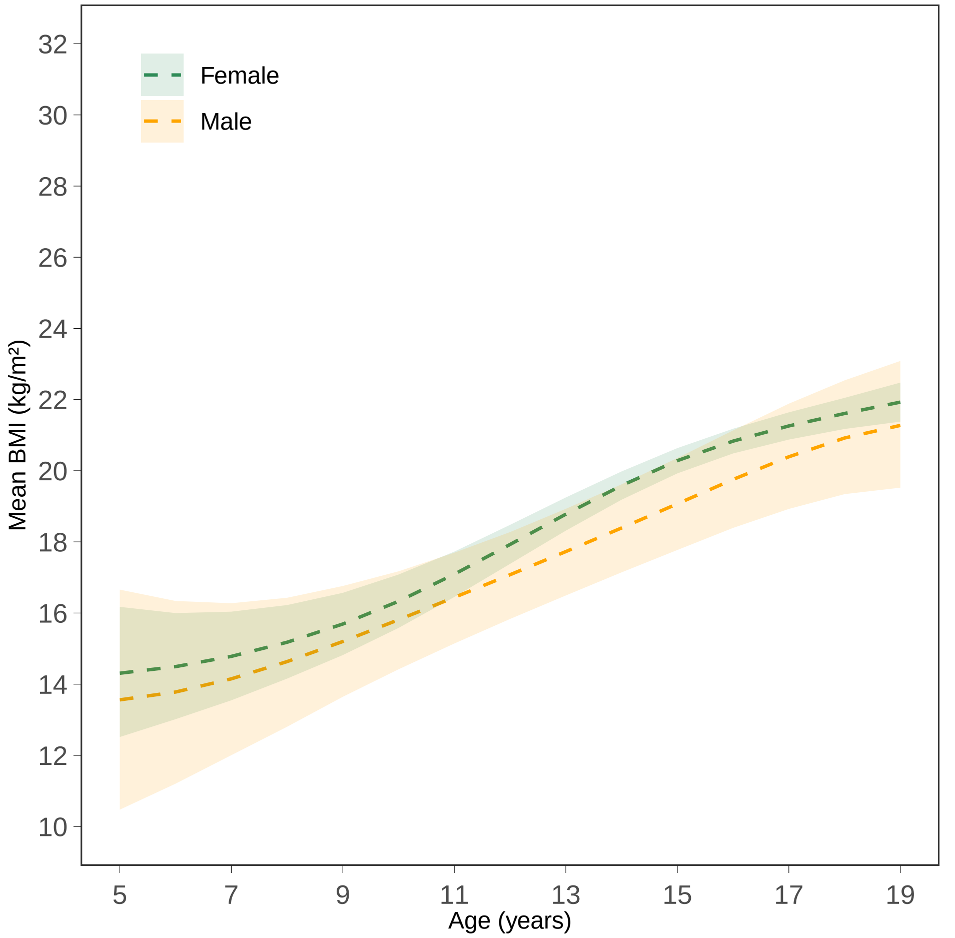

Malta

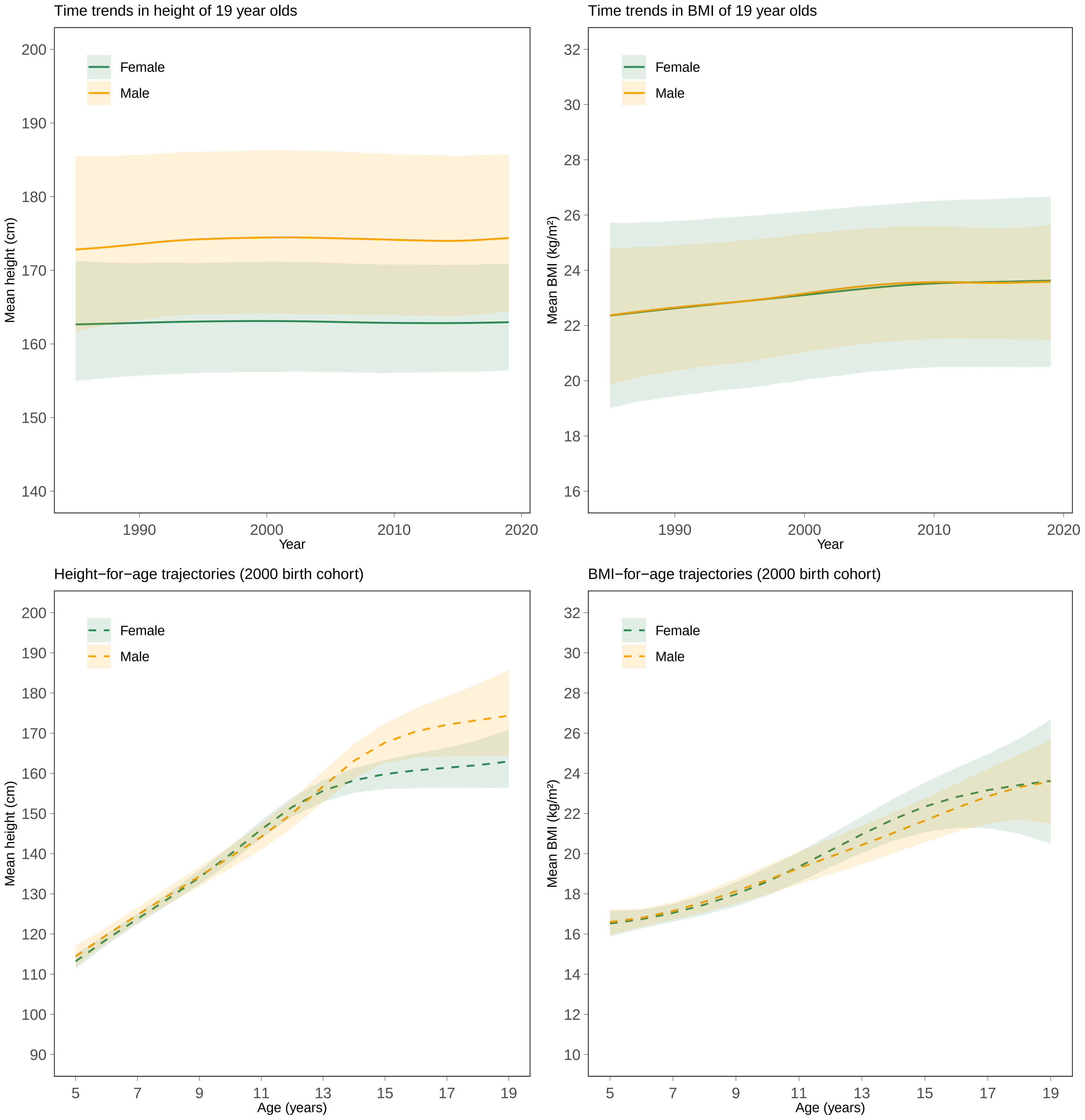
Time trends in height of 19 year olds
Female
Male
Mean height (cm)
200
190
180
170
160
150
140
1990
2000
2010
2020
Year
Time trends in BMI of 19 year olds
Female
Male
Mean BMI (kg/m²)
32
30
28
26
24
22
20
18
16
1990
2000
2010
2020
Year
Height−for−age trajectories (2000 birth cohort)
Female
Male
Mean height (cm)
200
190
180
170
160
150
140
130
120
110
100
90
5
7
9
11
13
15
17
19
Age (years)
BMI−for−age trajectories (2000 birth cohort)
Female
Male
Mean BMI (kg/m²)
32
30
28
26
24
22
20
18
16
14
12
10
5
7
9
11
13
15
17
19
Age (years)

Marshall Islands

Time trends in height of 19 year olds

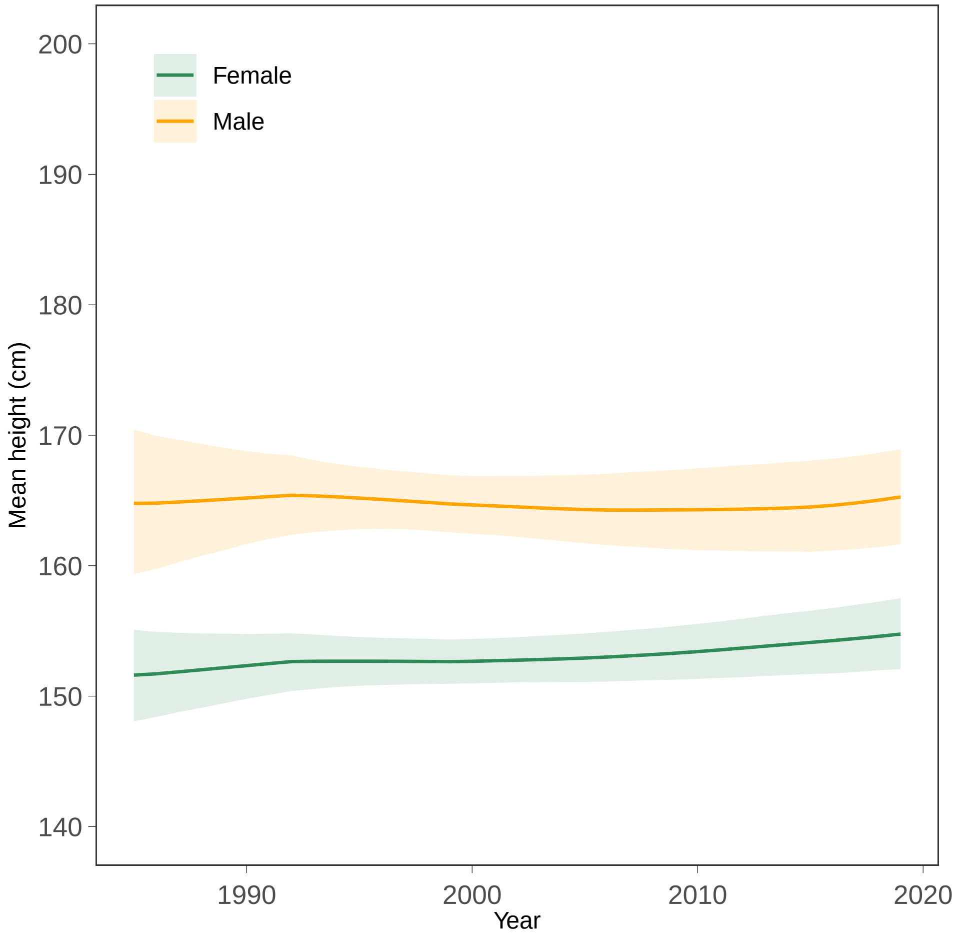

Time trends in BMI of 19 year olds

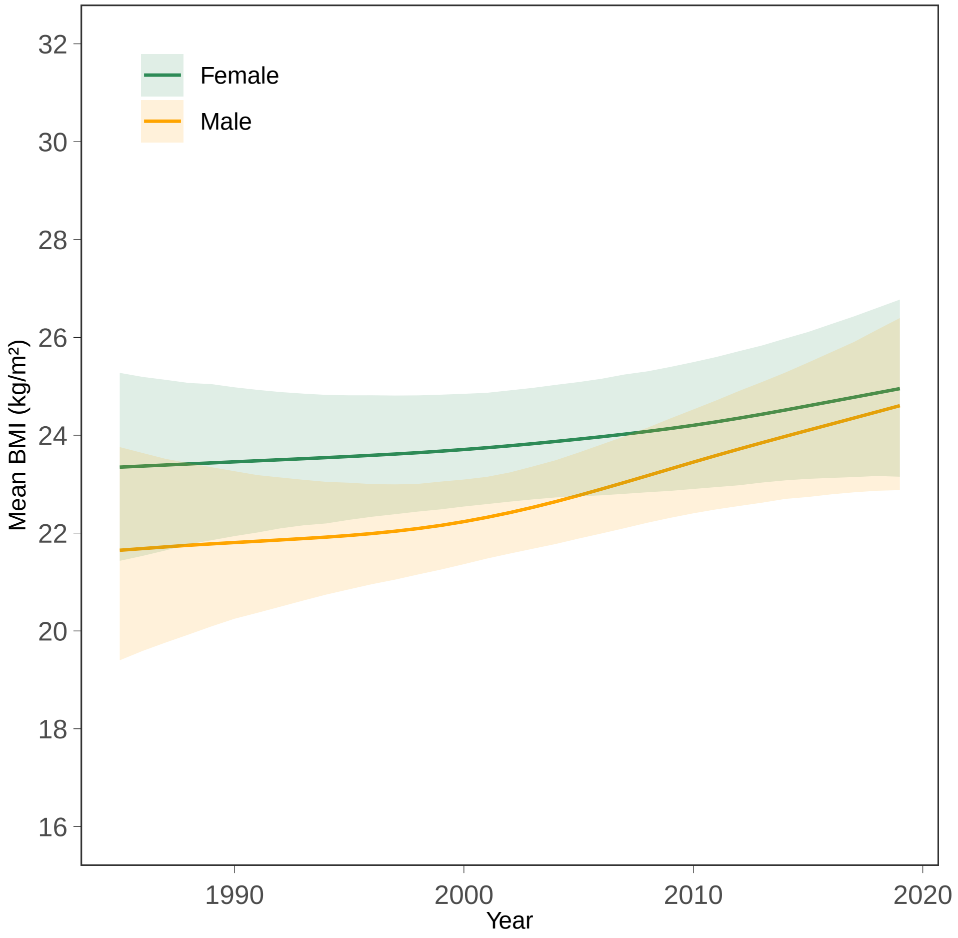

Height−for−age trajectories (2000 birth cohort)

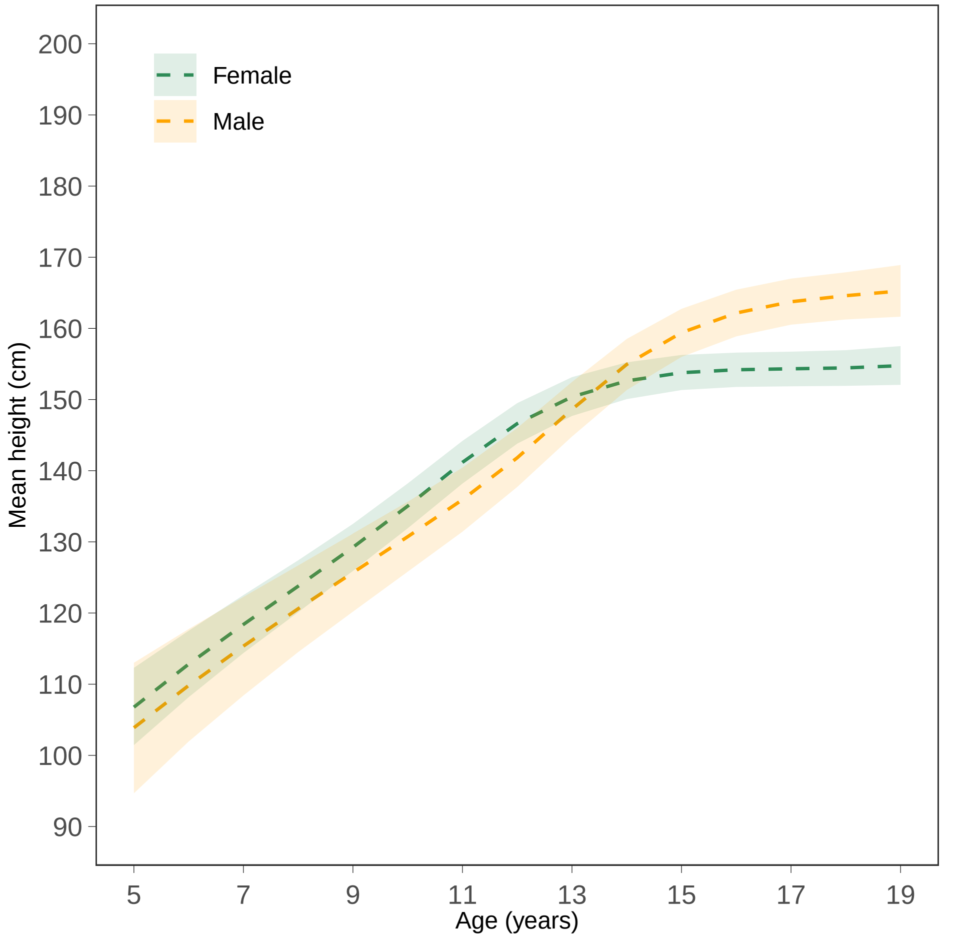

BMI−for−age trajectories (2000 birth cohort)

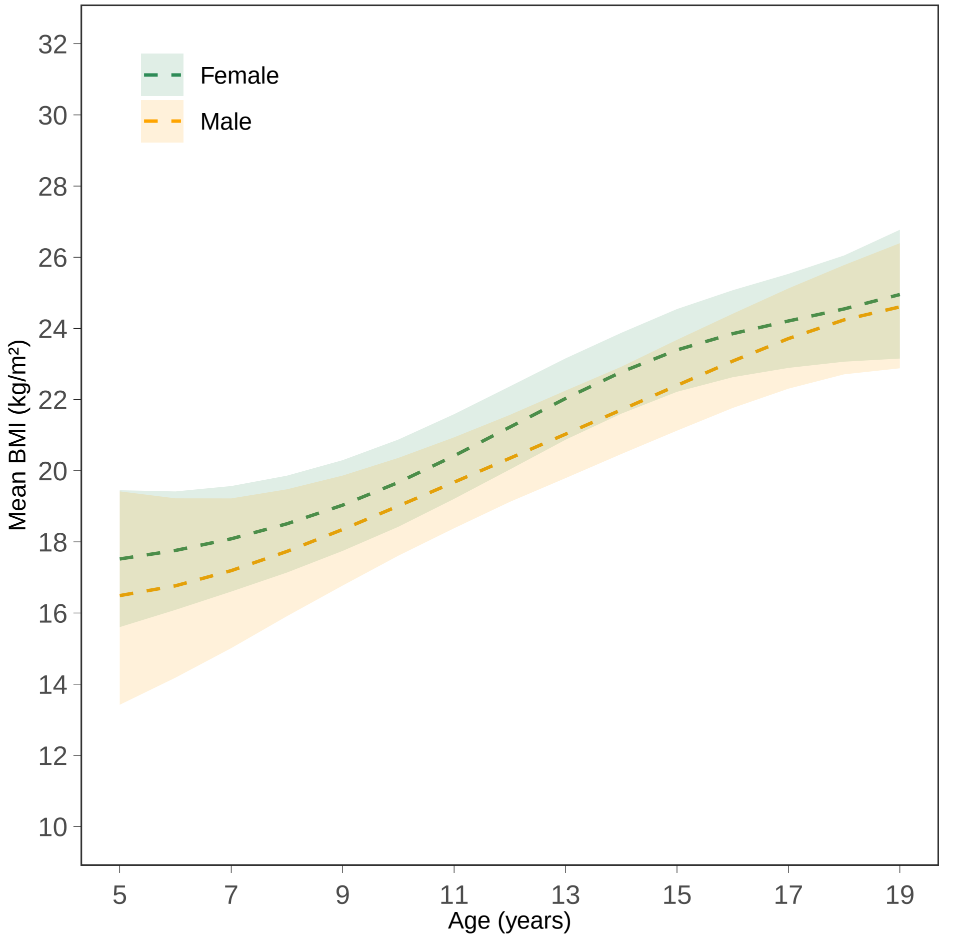

Mauritania

Time trends in height of 19 year olds

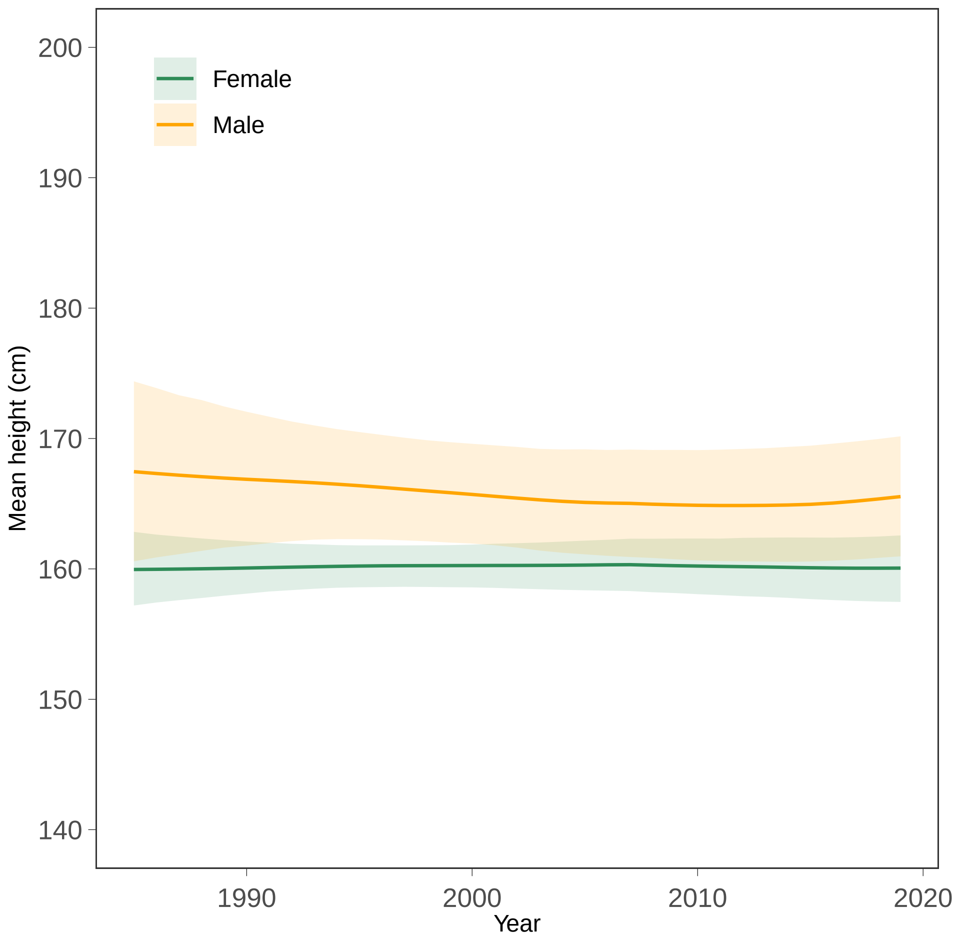

Time trends in BMI of 19 year olds

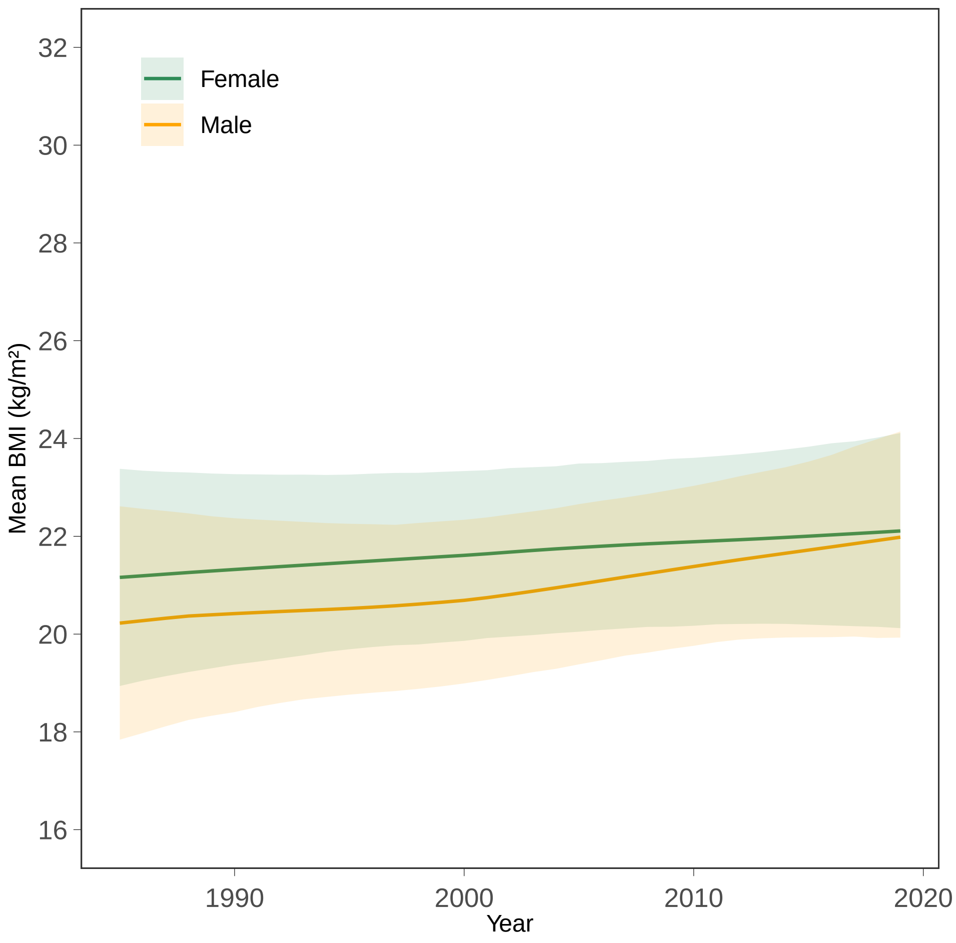

Height−for−age trajectories (2000 birth cohort)

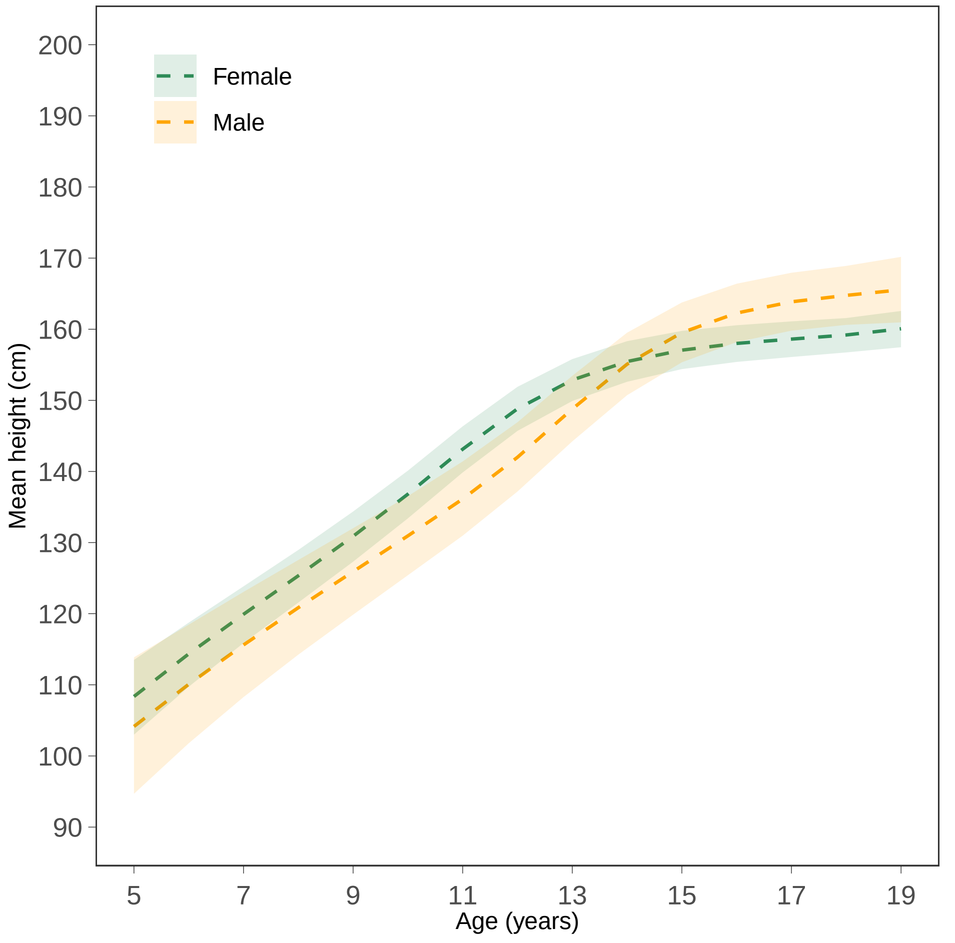

BMI−for−age trajectories (2000 birth cohort)

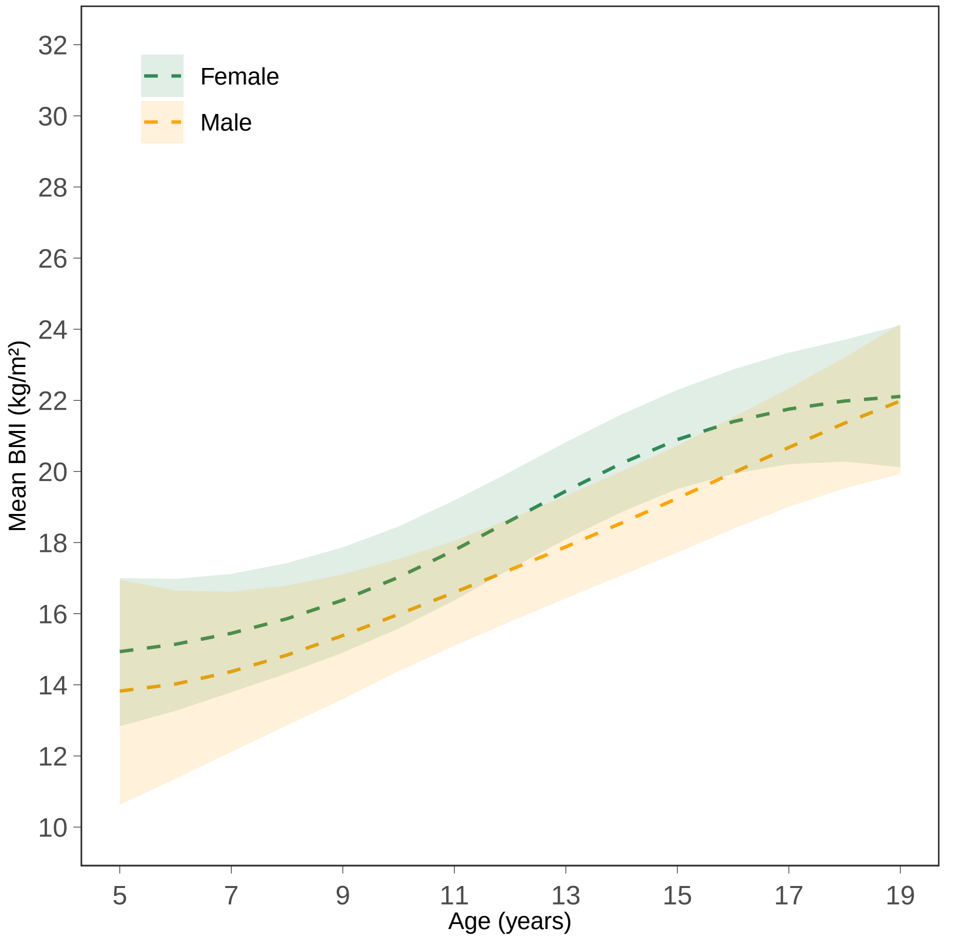

Mauritius

Time trends in height of 19 year olds

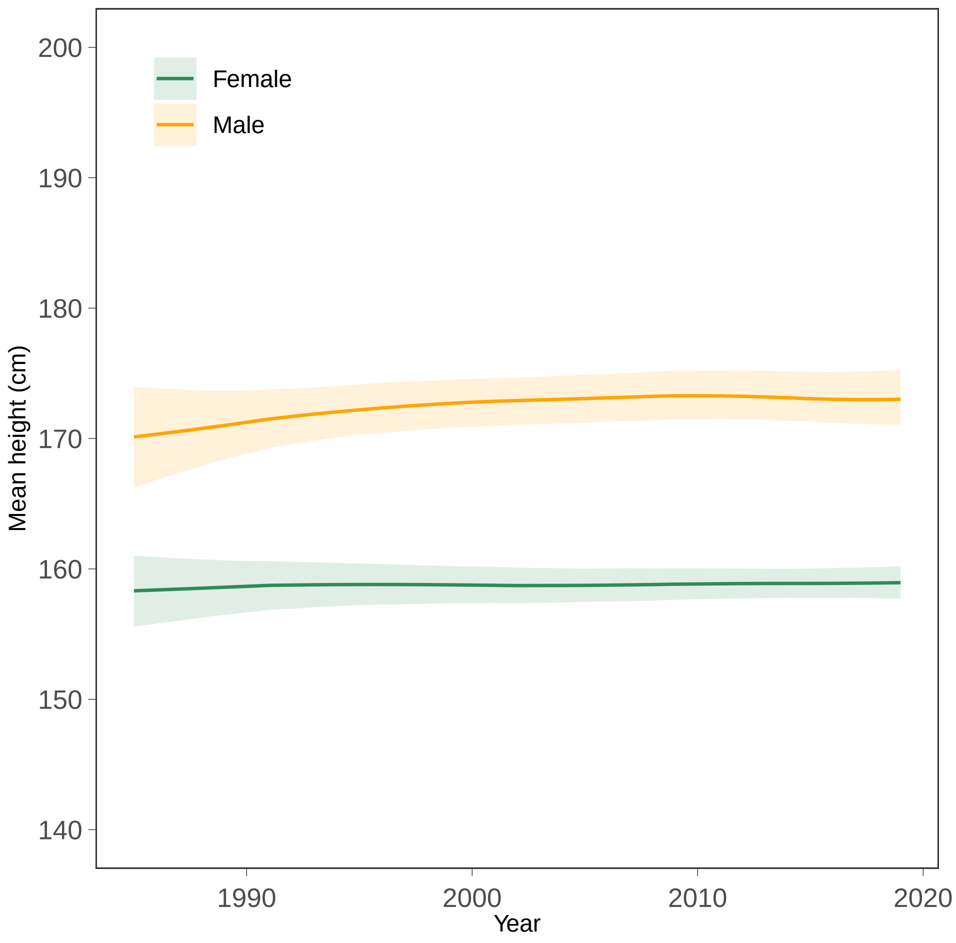

Time trends in BMI of 19 year olds

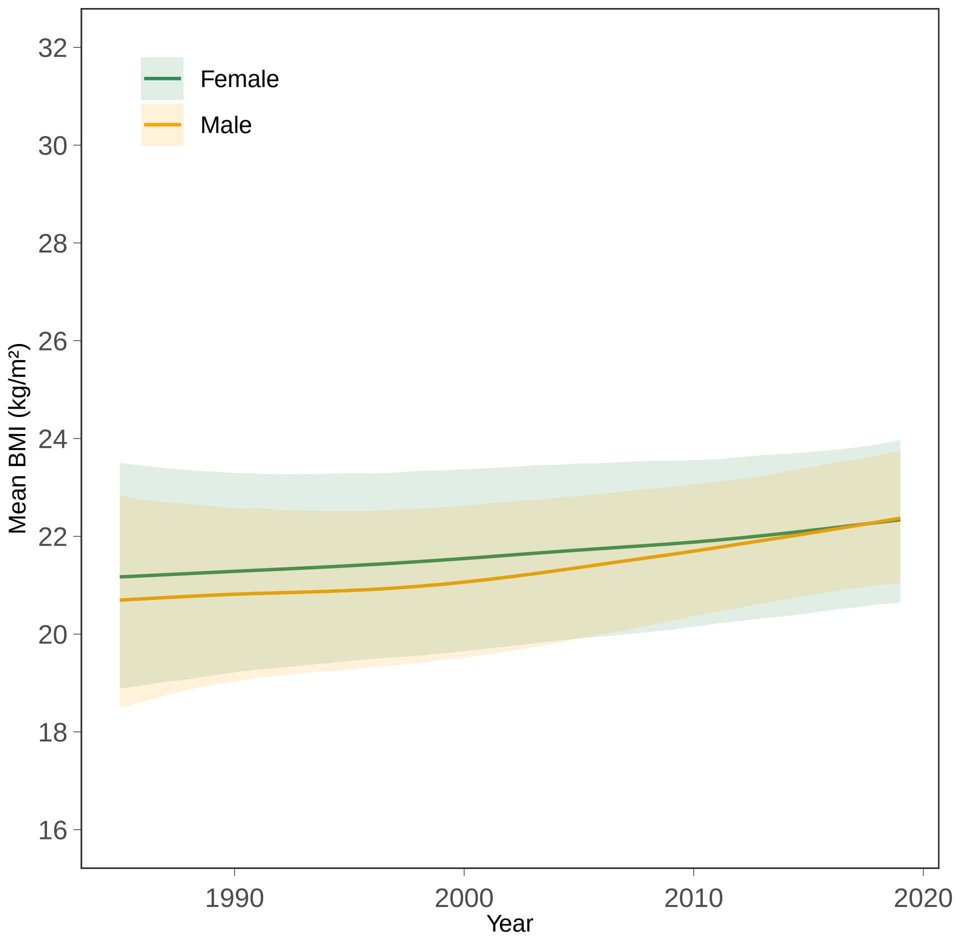

Height−for−age trajectories (2000 birth cohort)

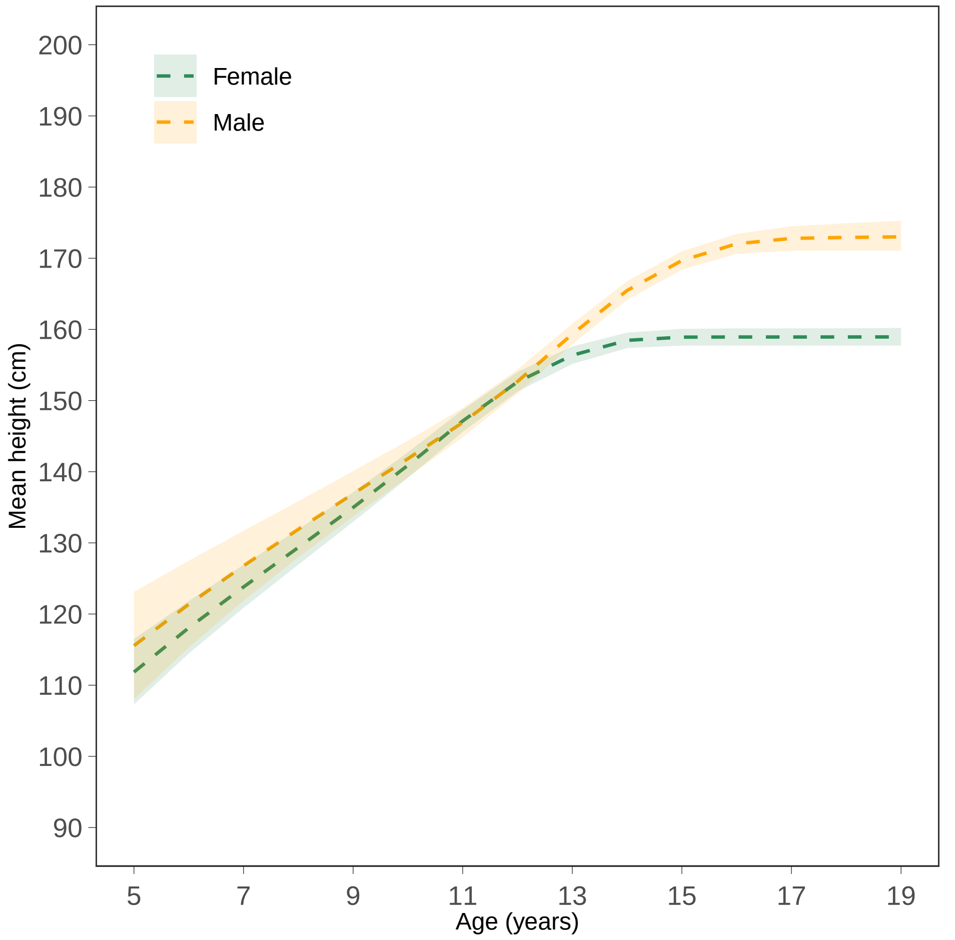

BMI−for−age trajectories (2000 birth cohort)

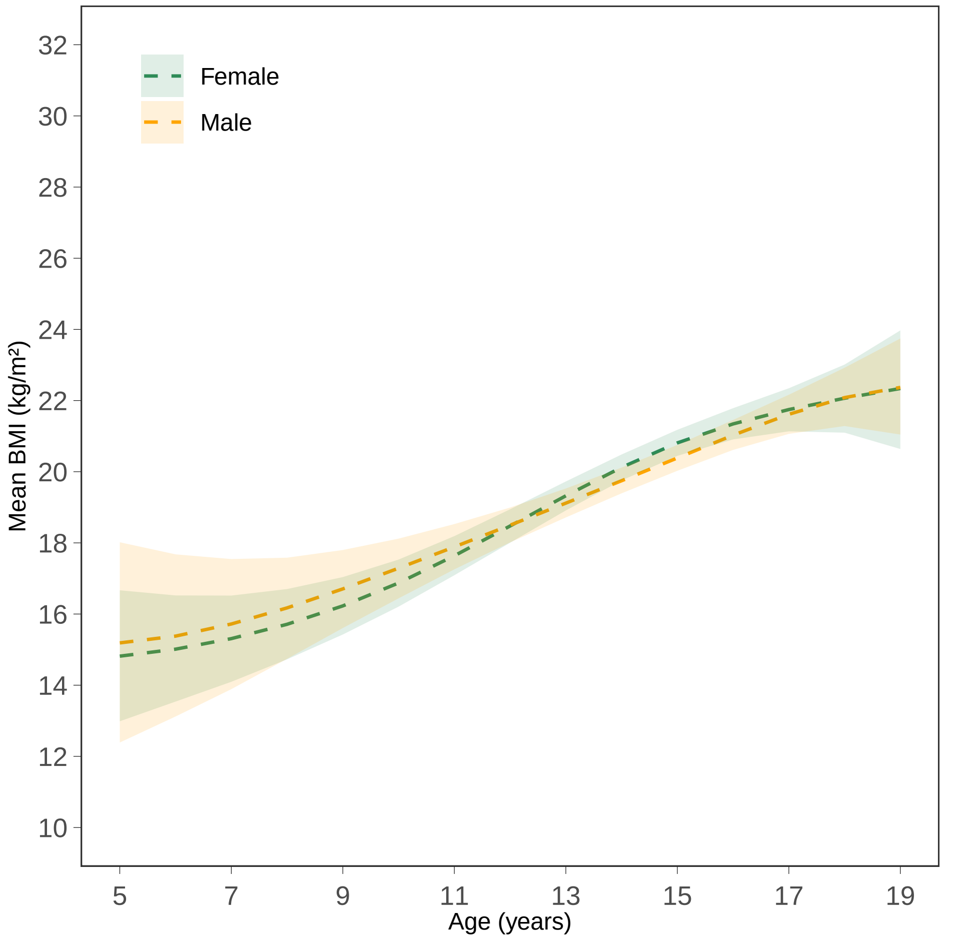

Mexico

Time trends in height of 19 year olds

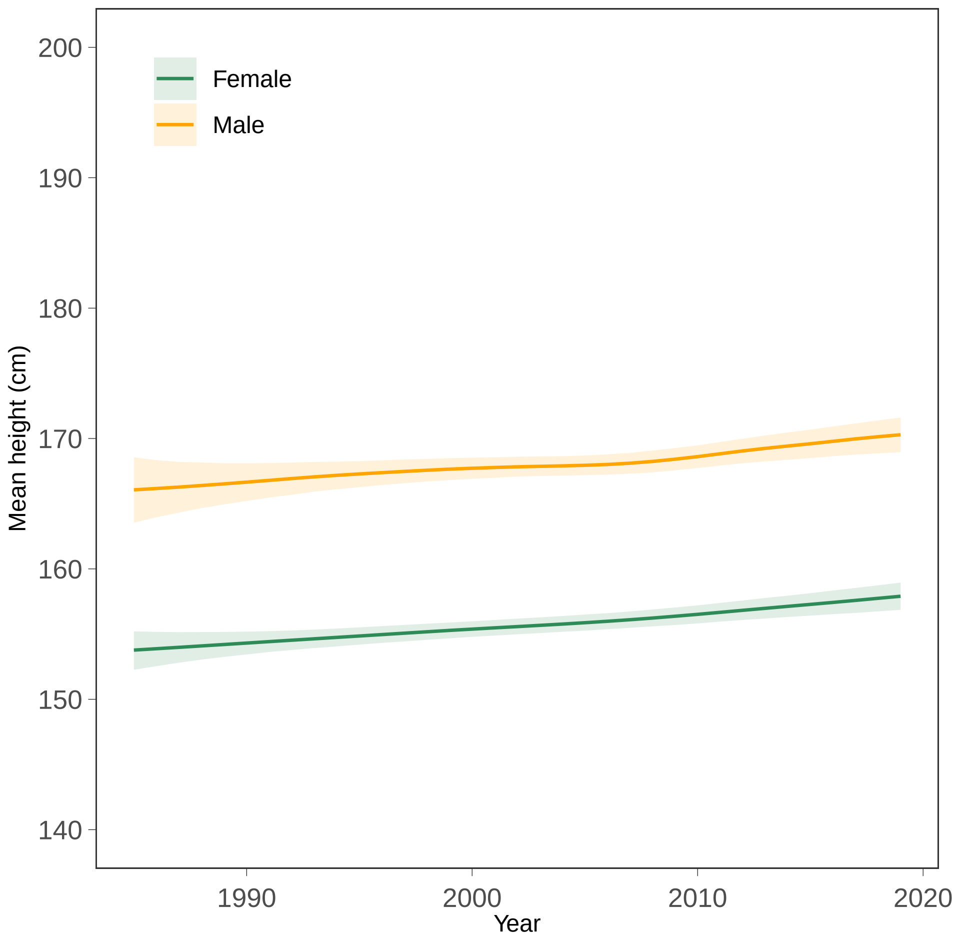

Time trends in BMI of 19 year olds

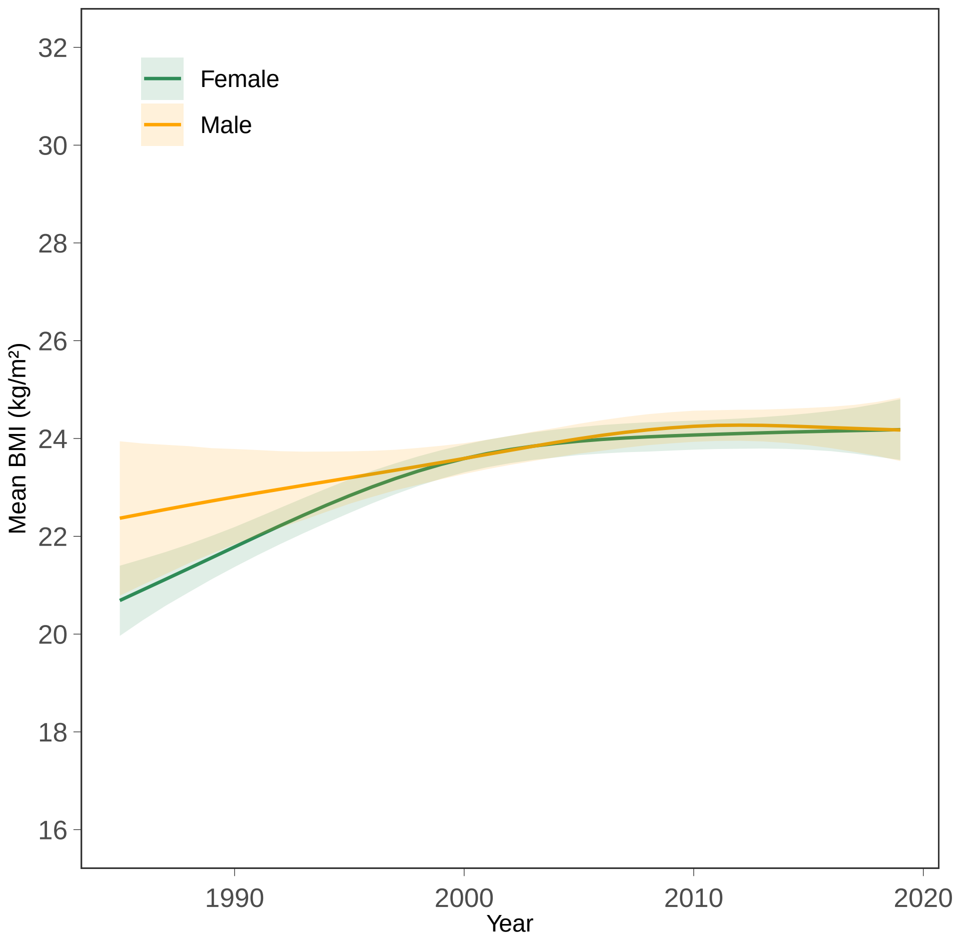

Height−for−age trajectories (2000 birth cohort)

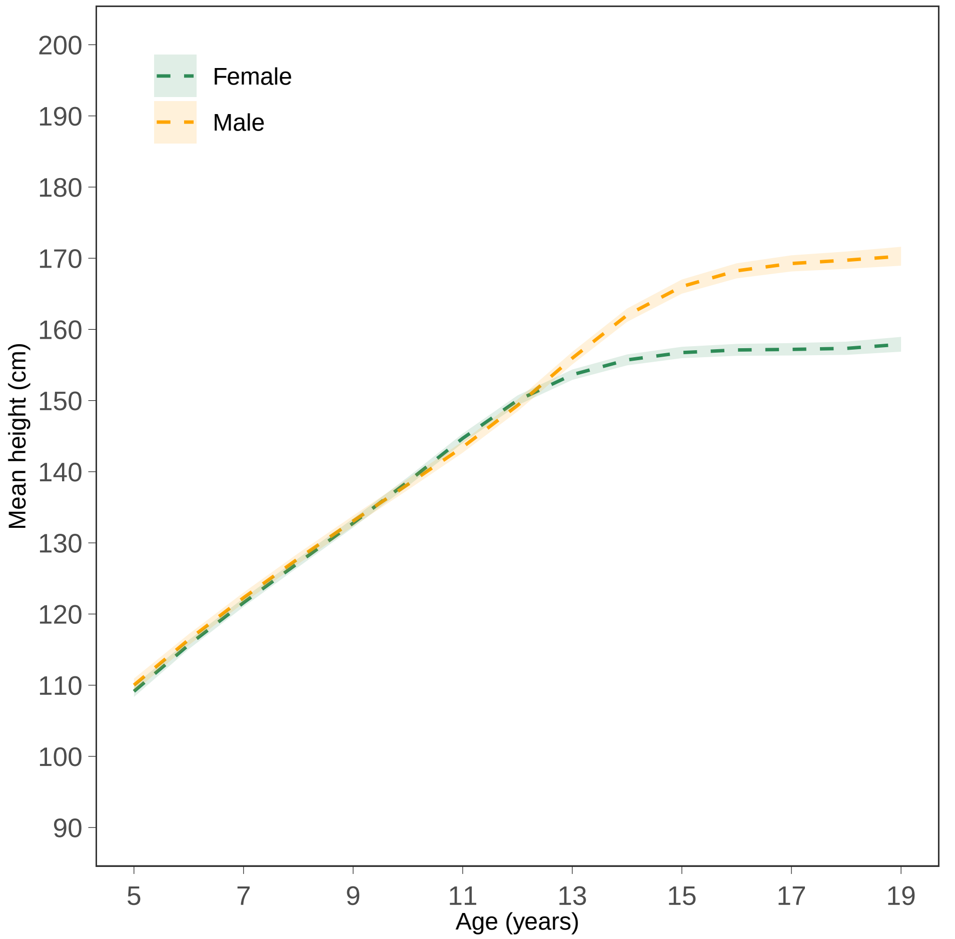

BMI−for−age trajectories (2000 birth cohort)

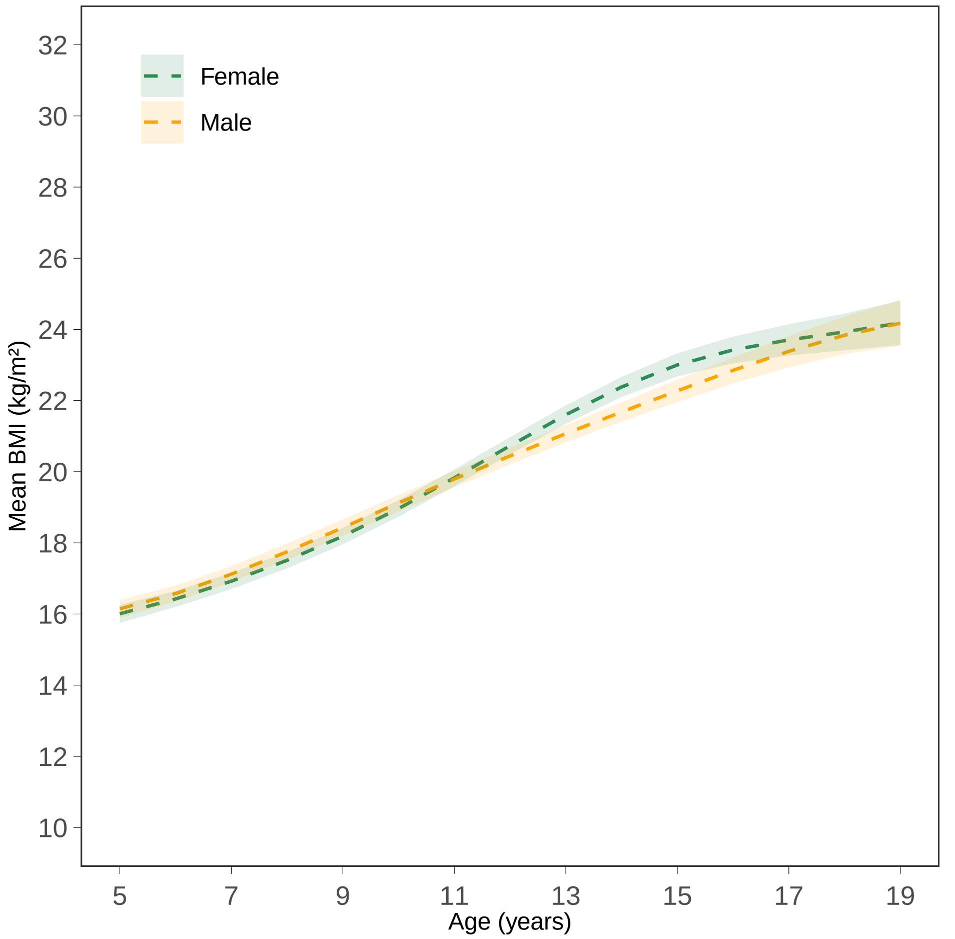

Micronesia (Federated States of)

Time trends in height of 19 year olds

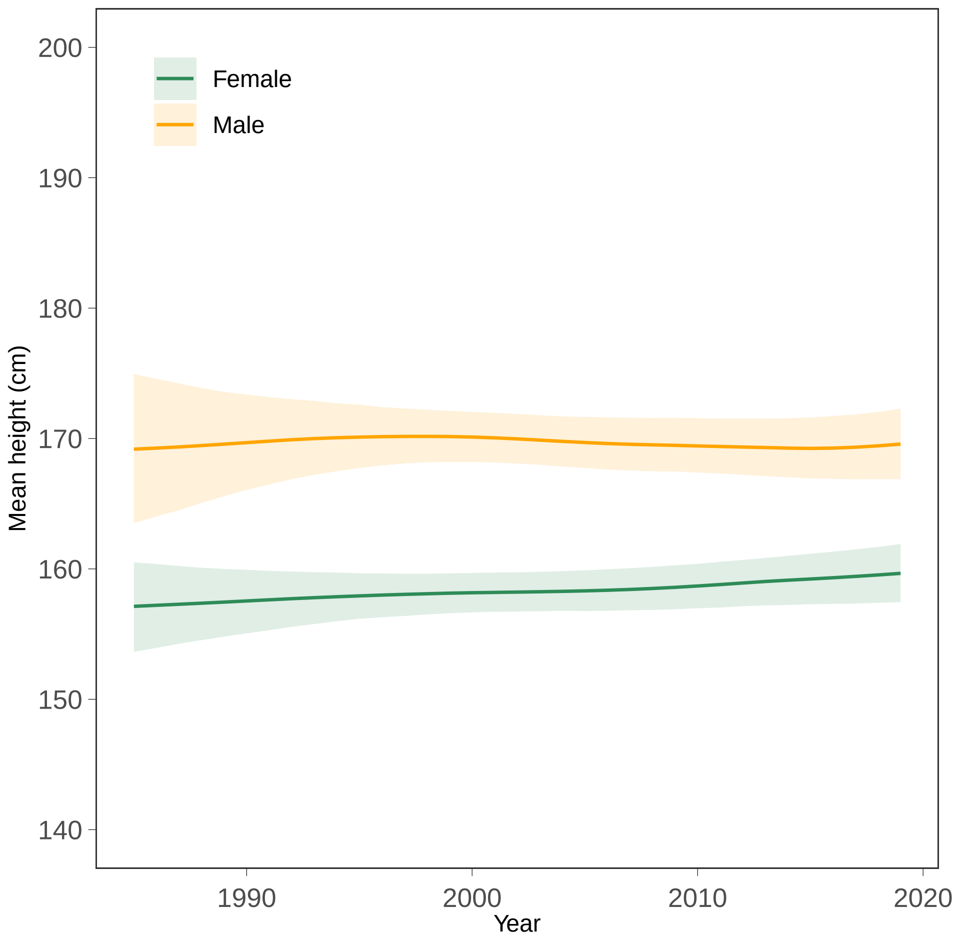

Time trends in BMI of 19 year olds

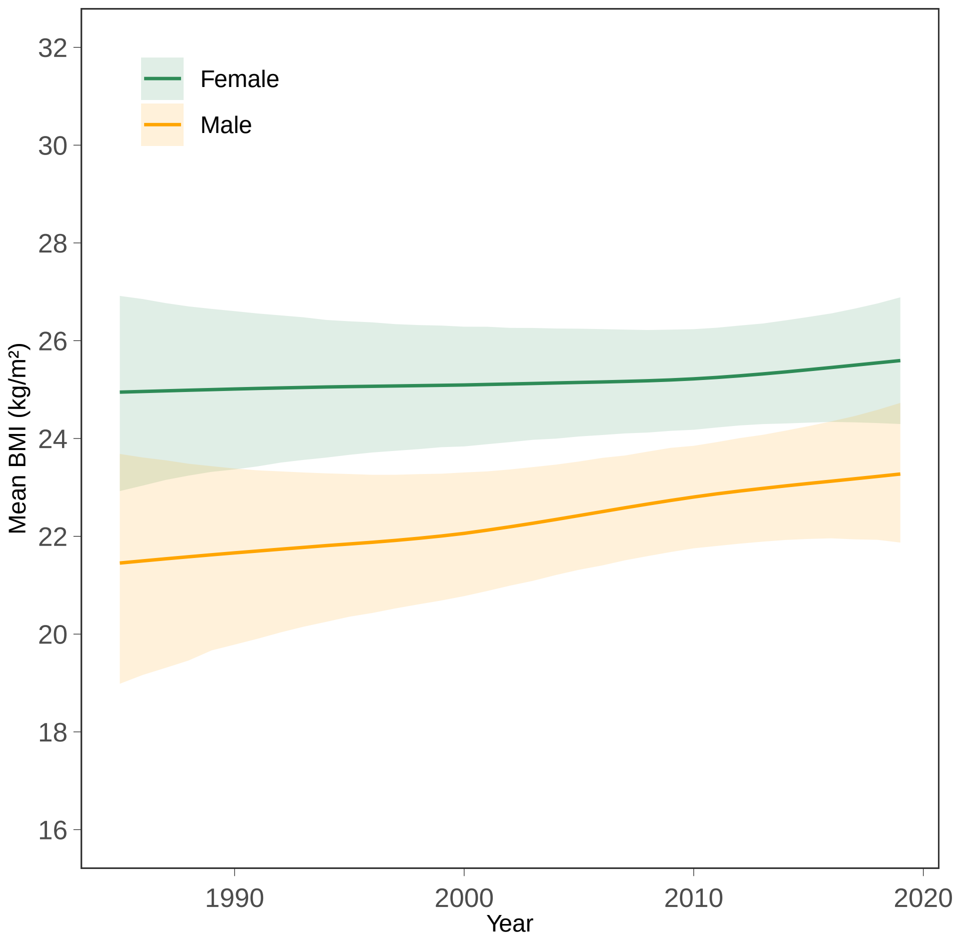

Height−for−age trajectories (2000 birth cohort)

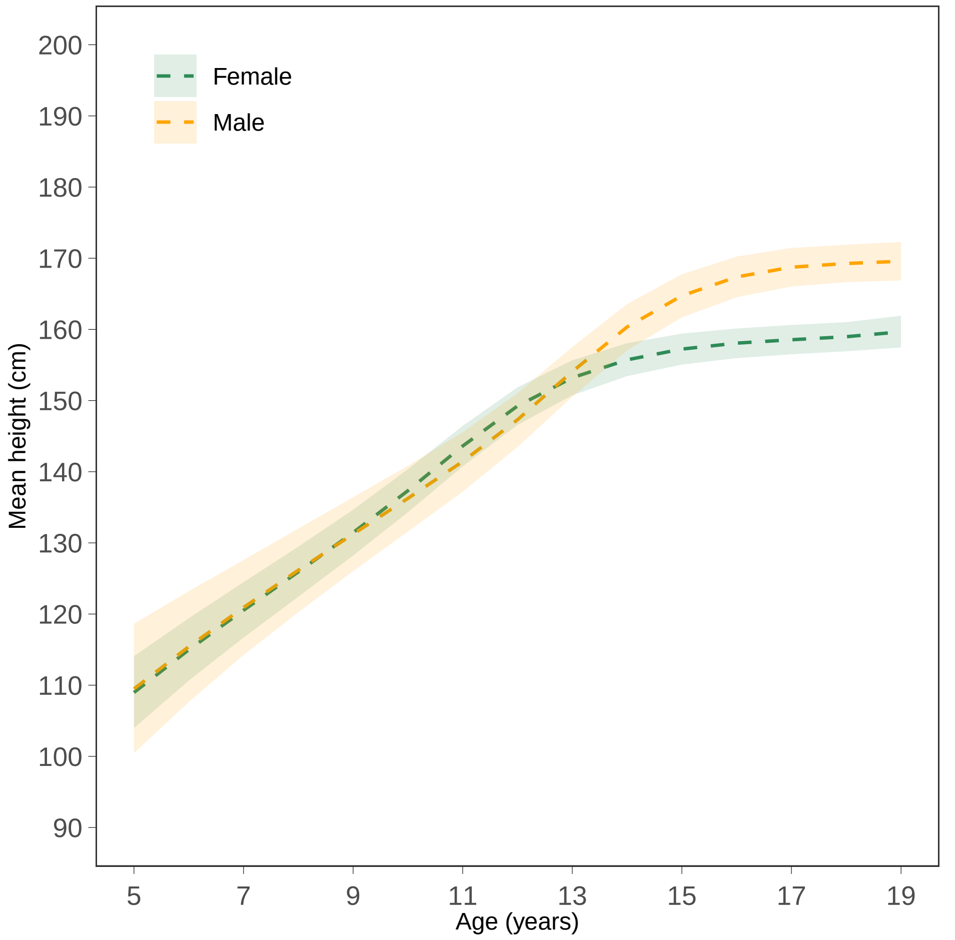

BMI−for−age trajectories (2000 birth cohort)

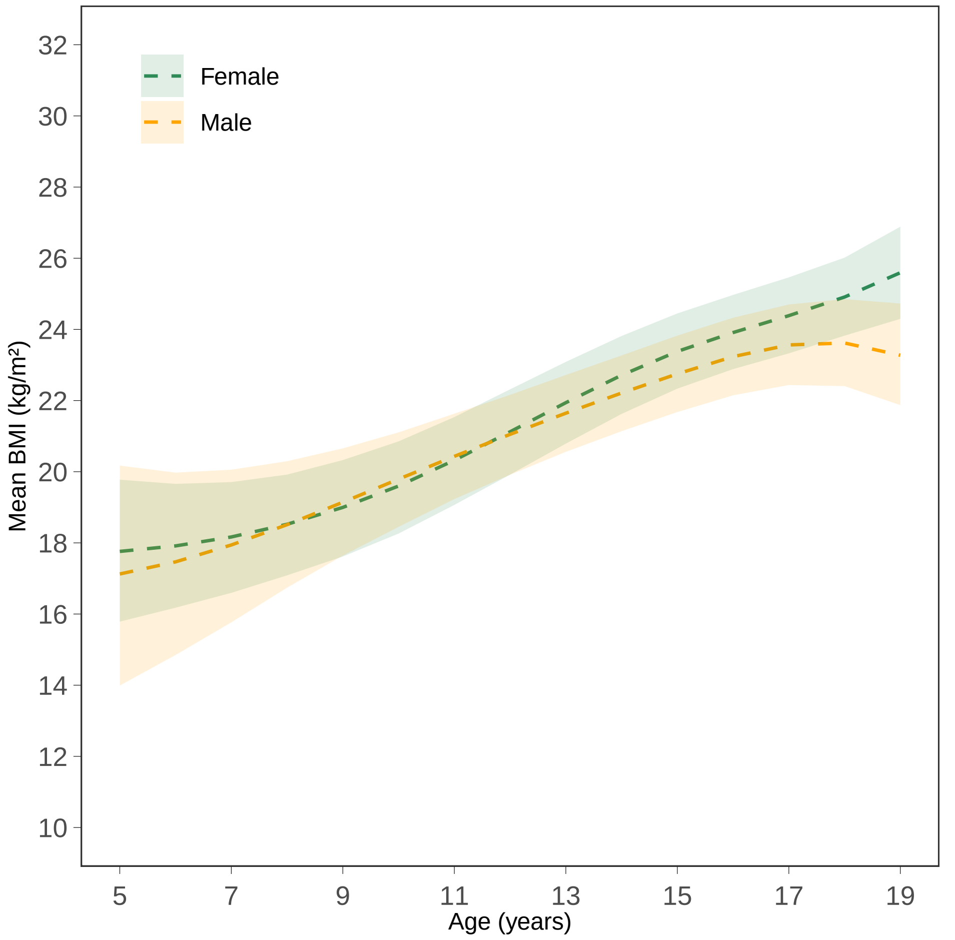

Moldova

Time trends in height of 19 year olds

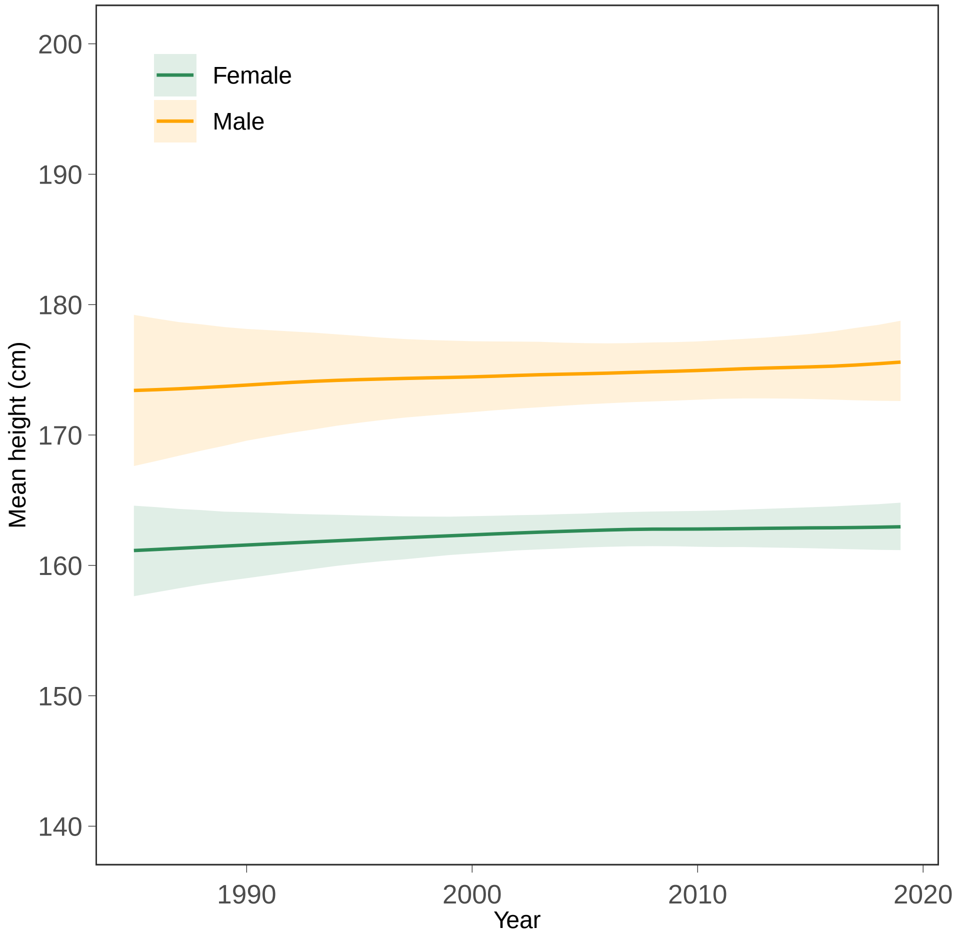

Time trends in BMI of 19 year olds

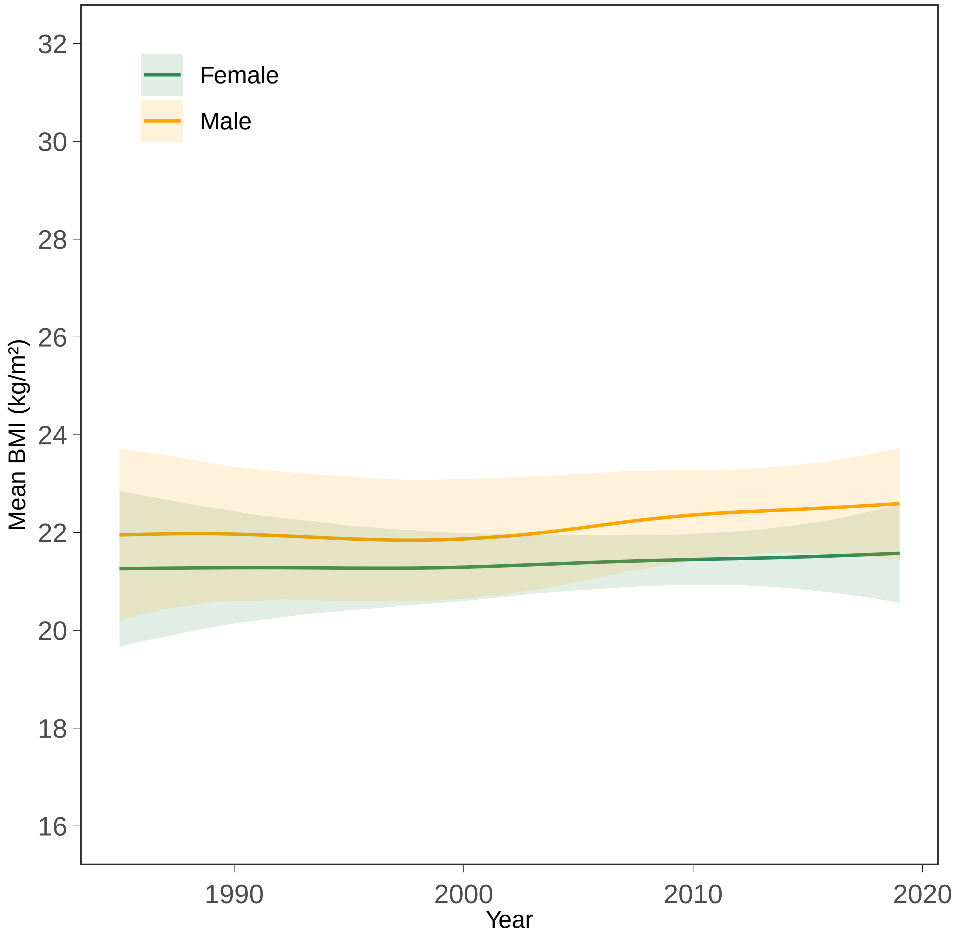

Height−for−age trajectories (2000 birth cohort)

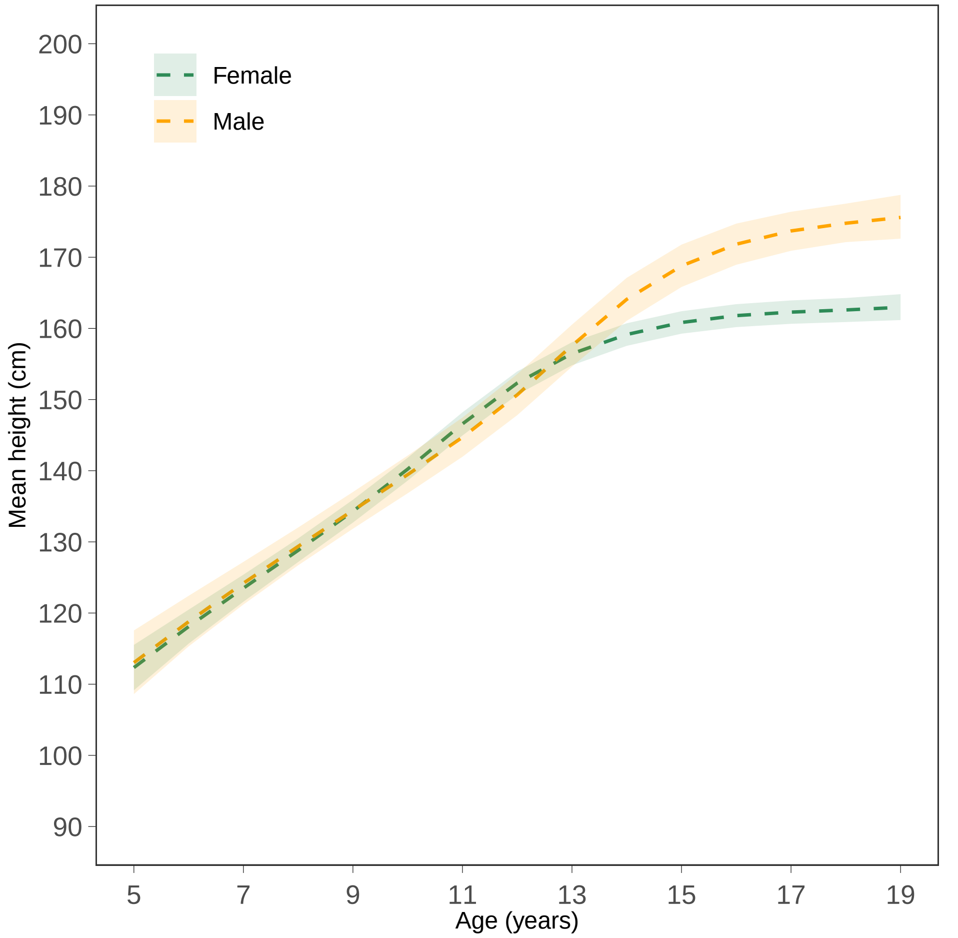

BMI−for−age trajectories (2000 birth cohort)

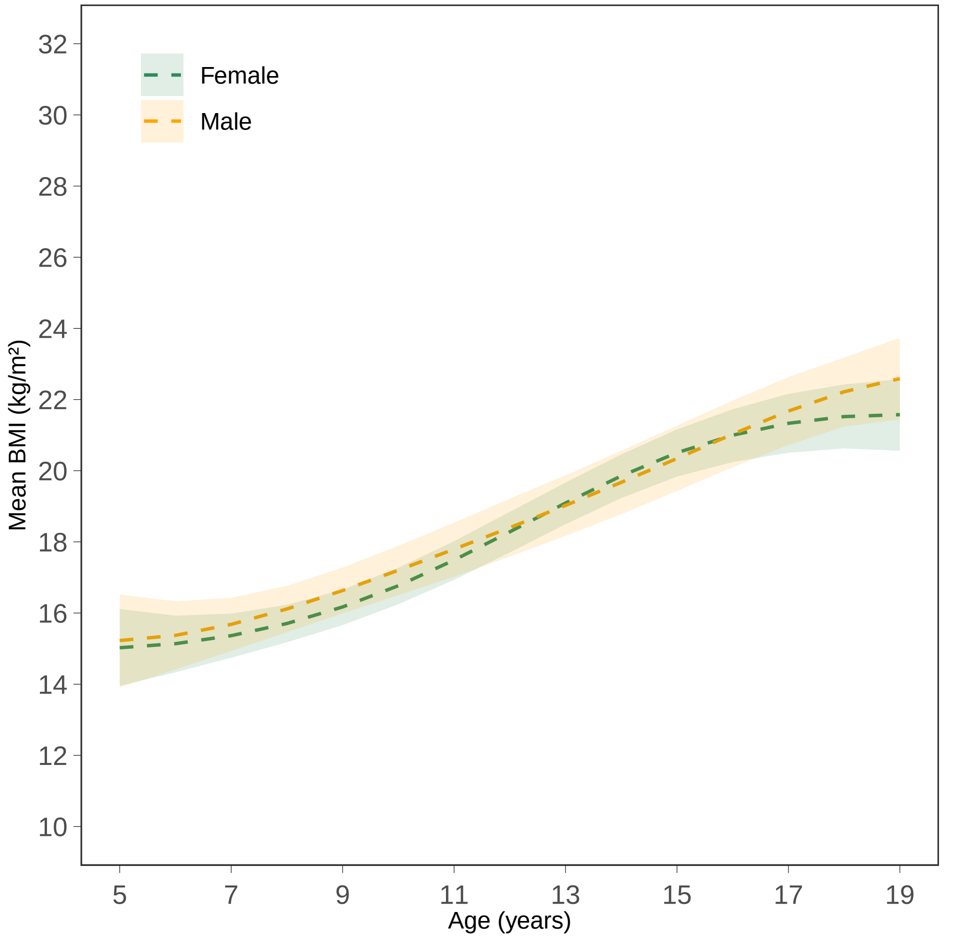

Mongolia

Time trends in height of 19 year olds

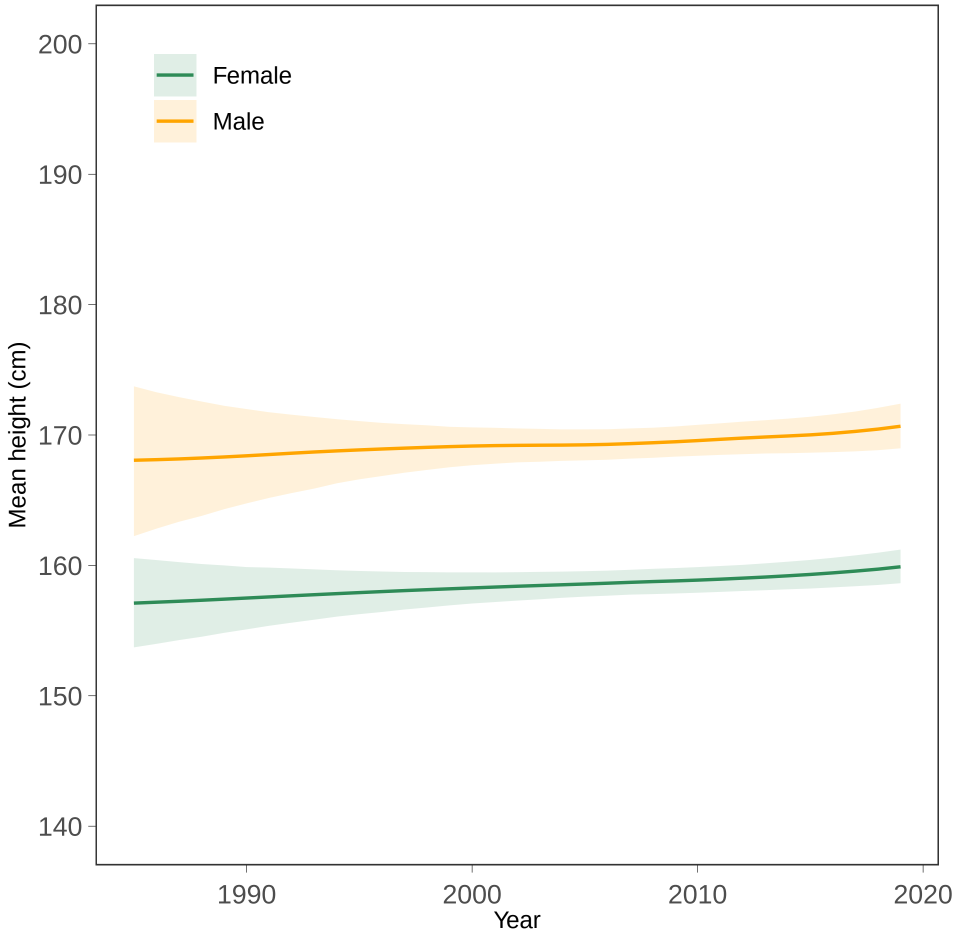

Time trends in BMI of 19 year olds

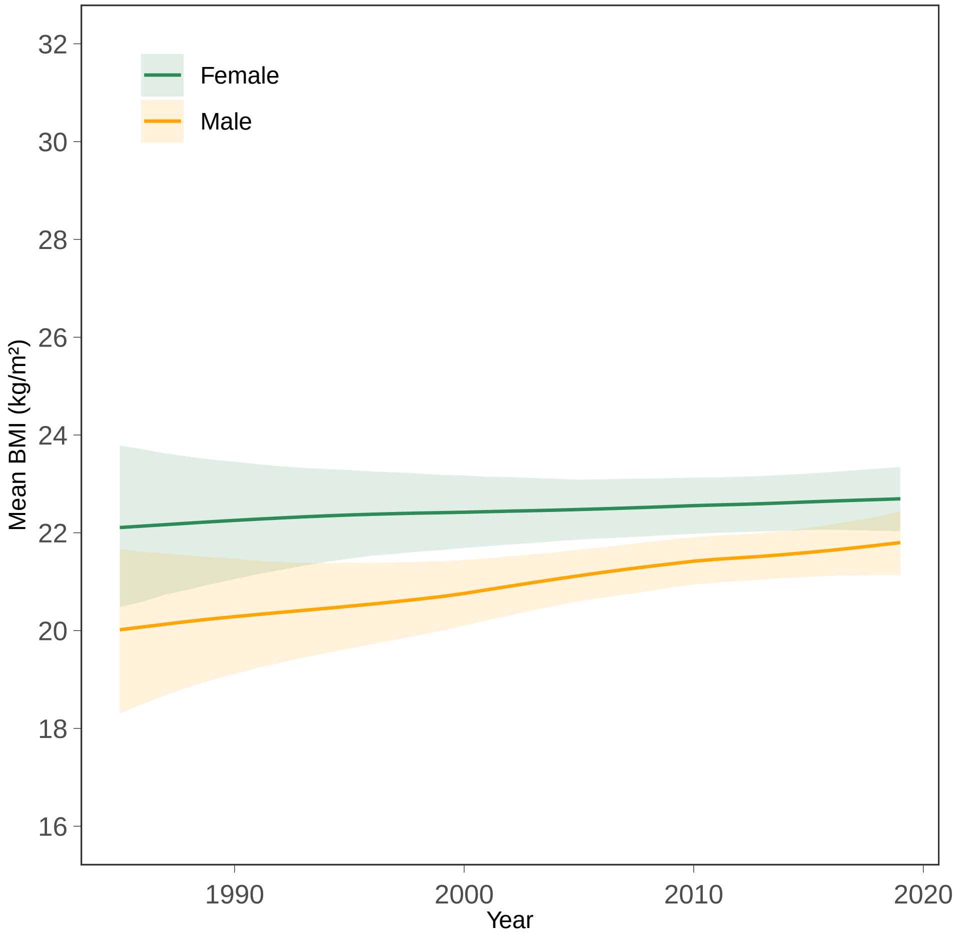

Height−for−age trajectories (2000 birth cohort)

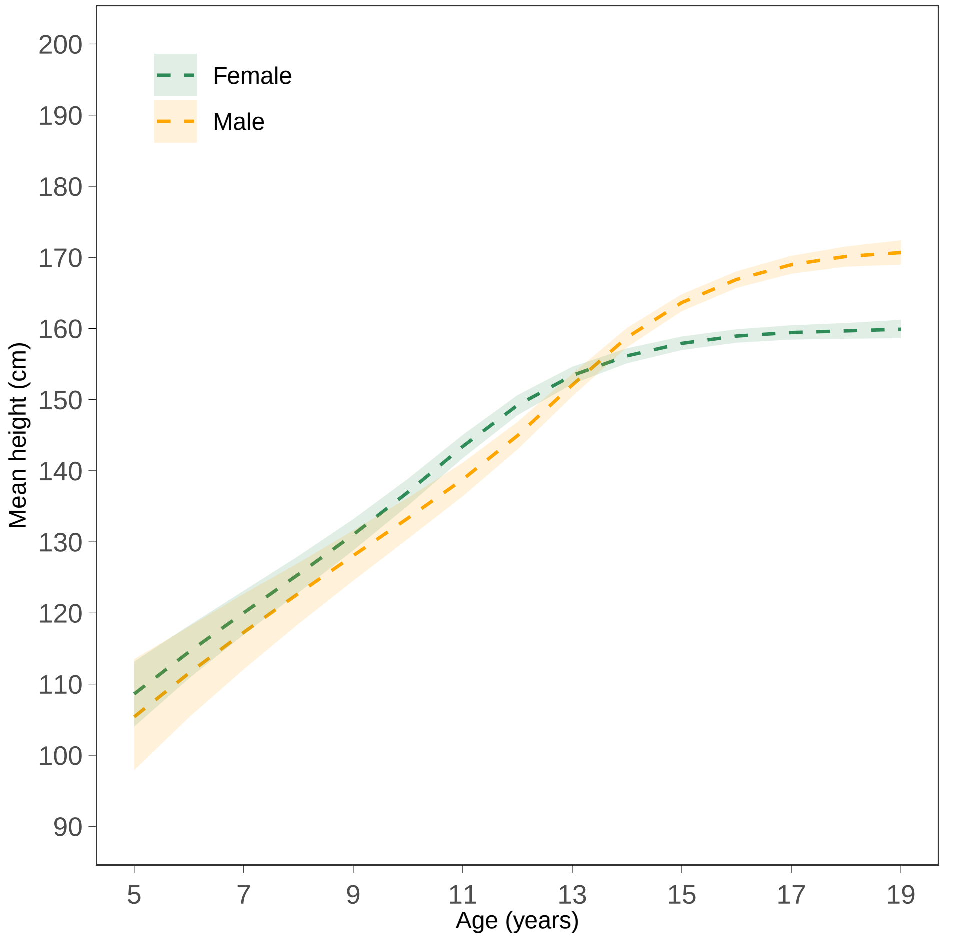

BMI−for−age trajectories (2000 birth cohort)

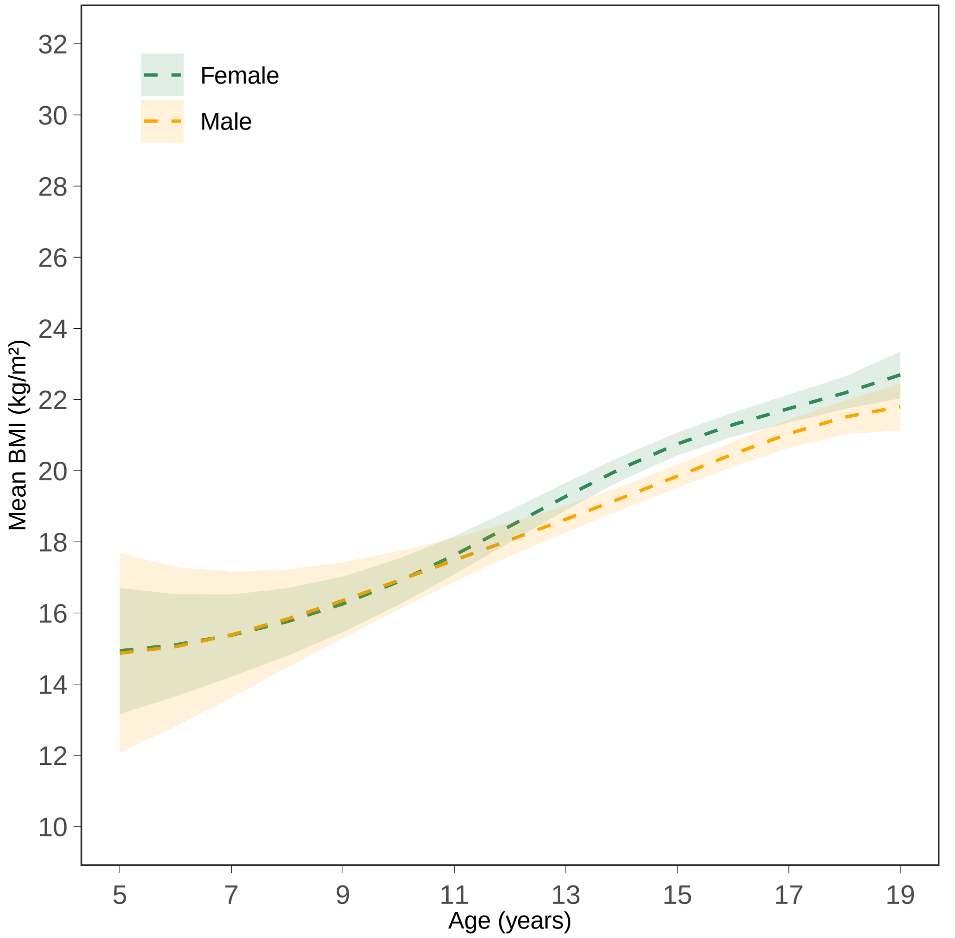

Montenegro

Time trends in height of 19 year olds

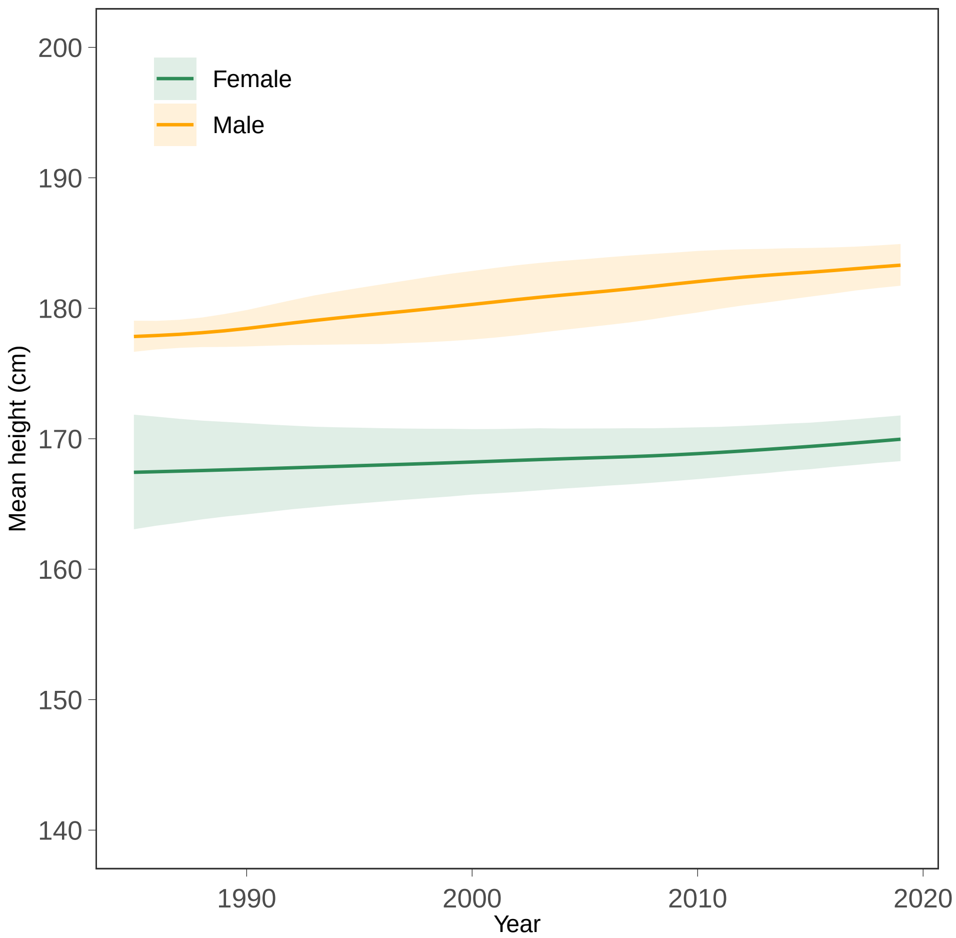

Time trends in BMI of 19 year olds

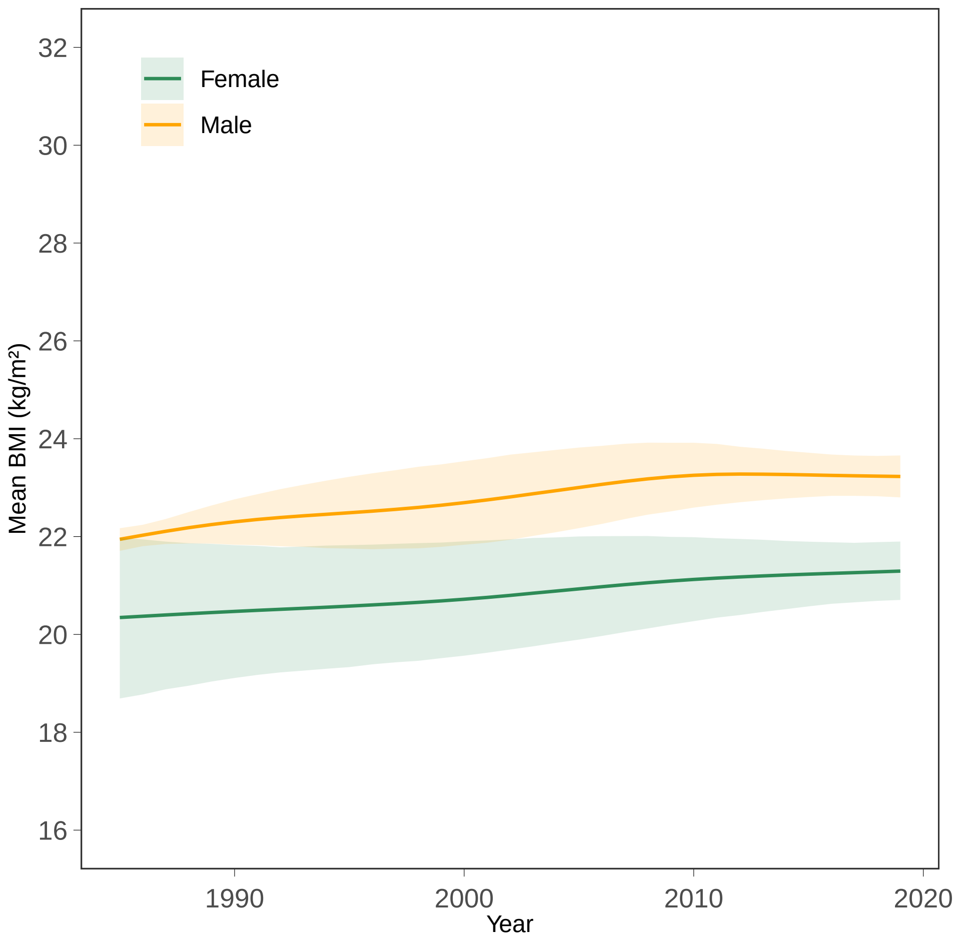

Height−for−age trajectories (2000 birth cohort)

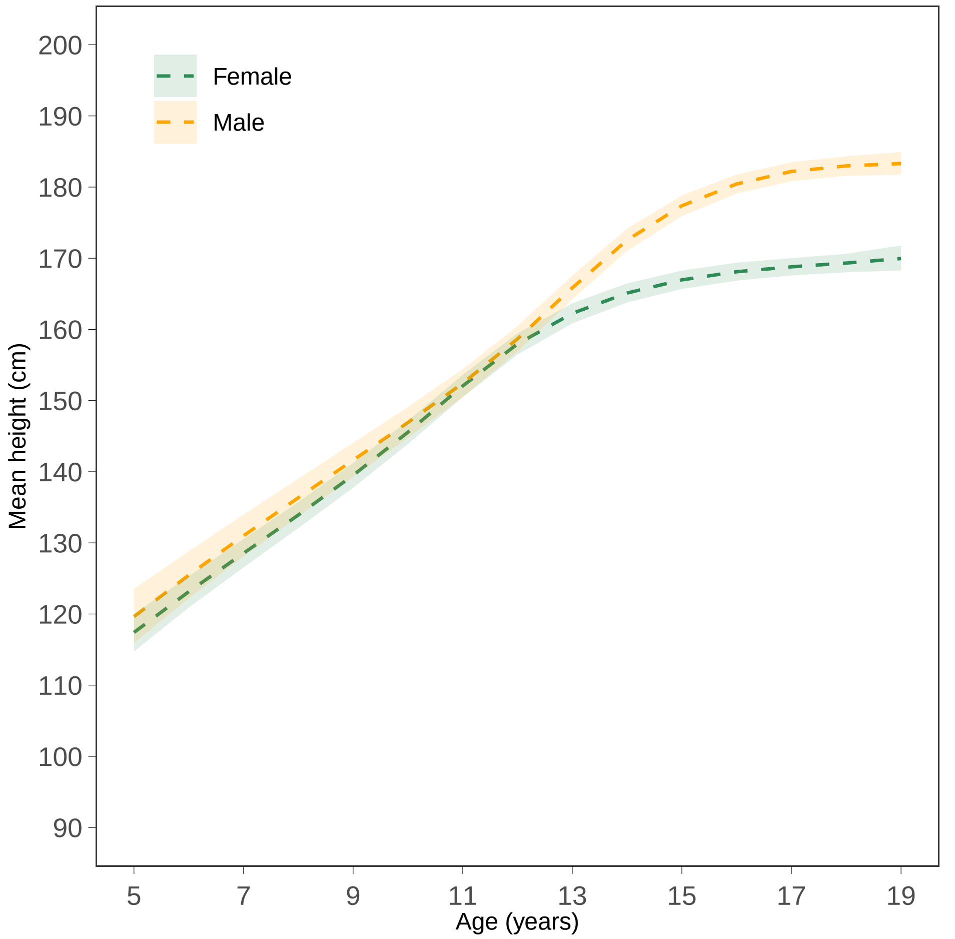

BMI−for−age trajectories (2000 birth cohort)

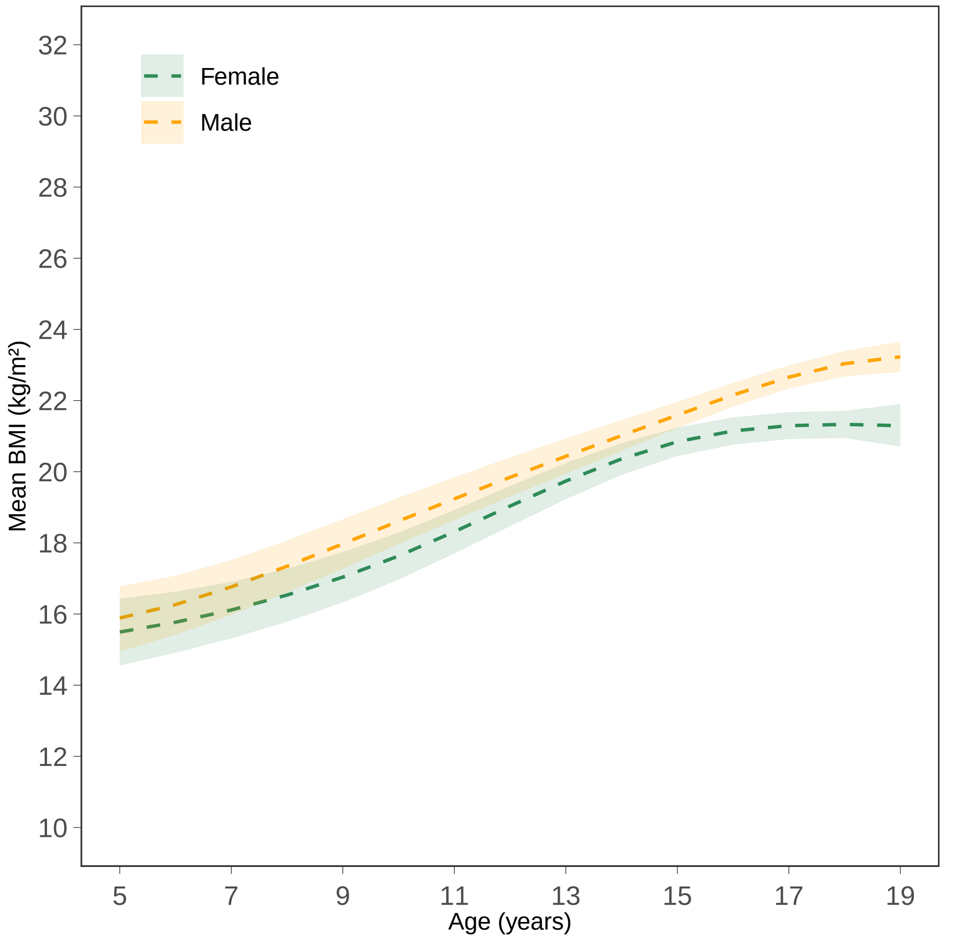

Morocco

Time trends in height of 19 year olds

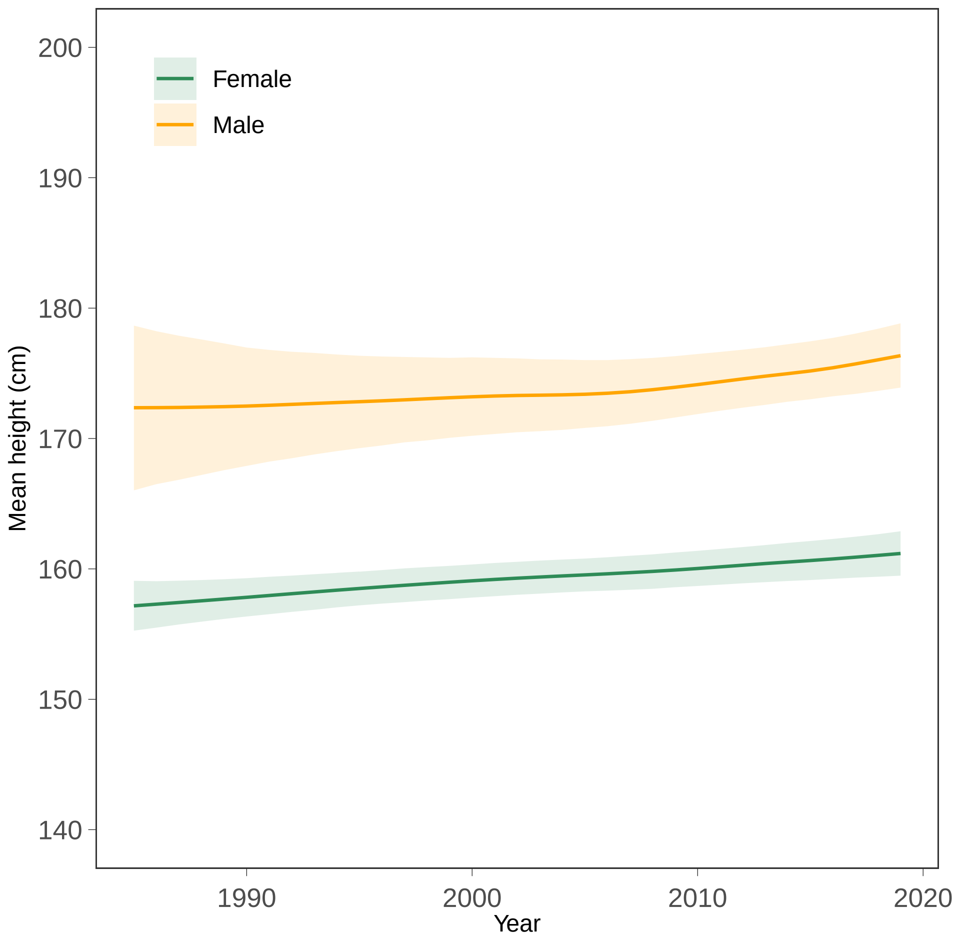

Time trends in BMI of 19 year olds

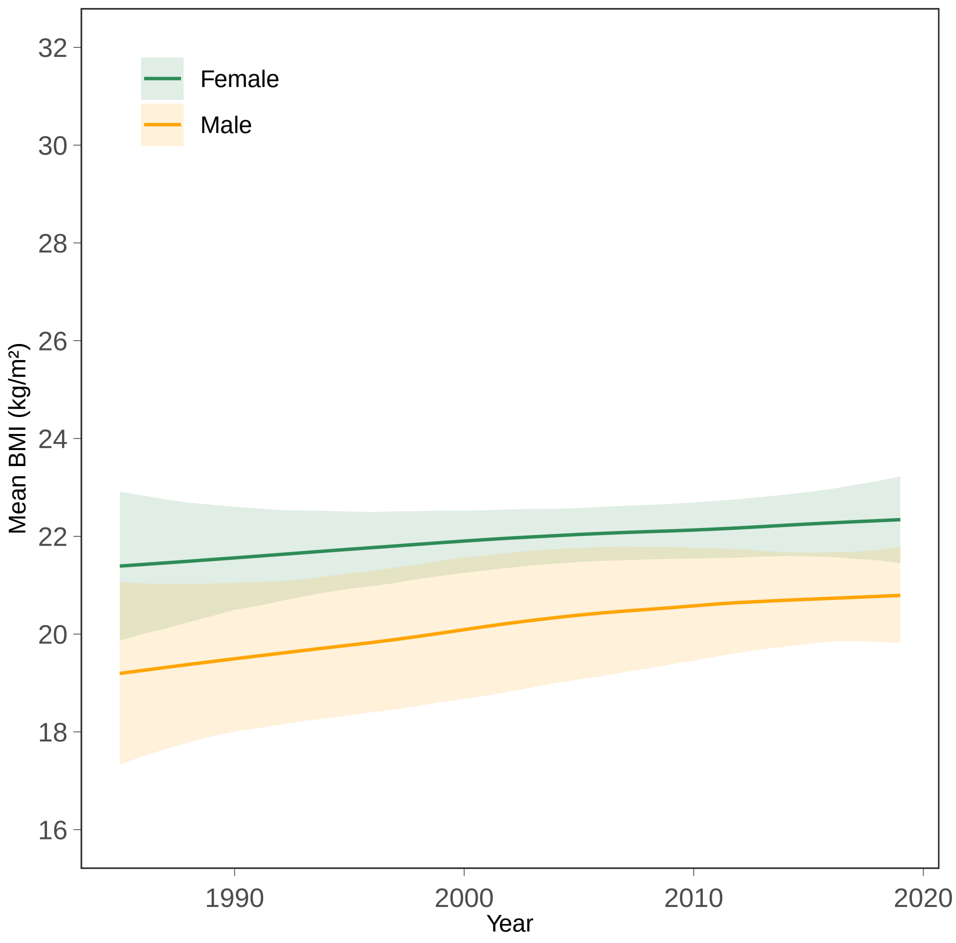

Height−for−age trajectories (2000 birth cohort)

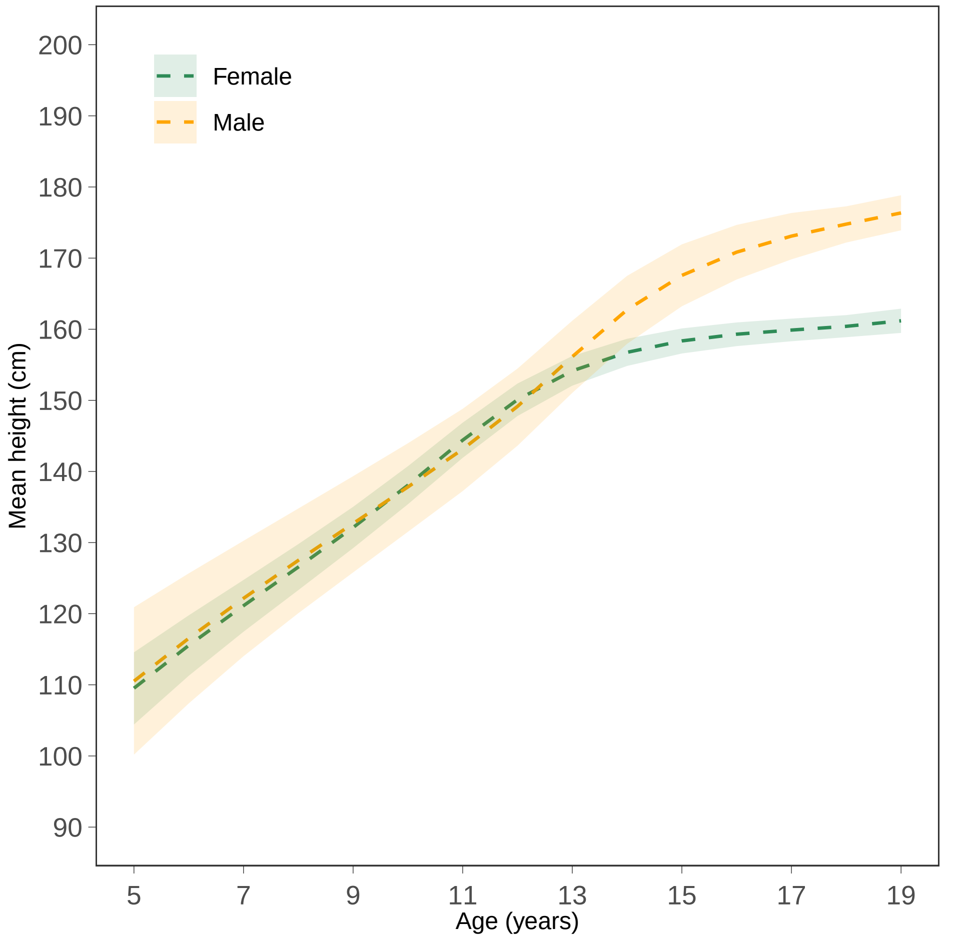

BMI−for−age trajectories (2000 birth cohort)

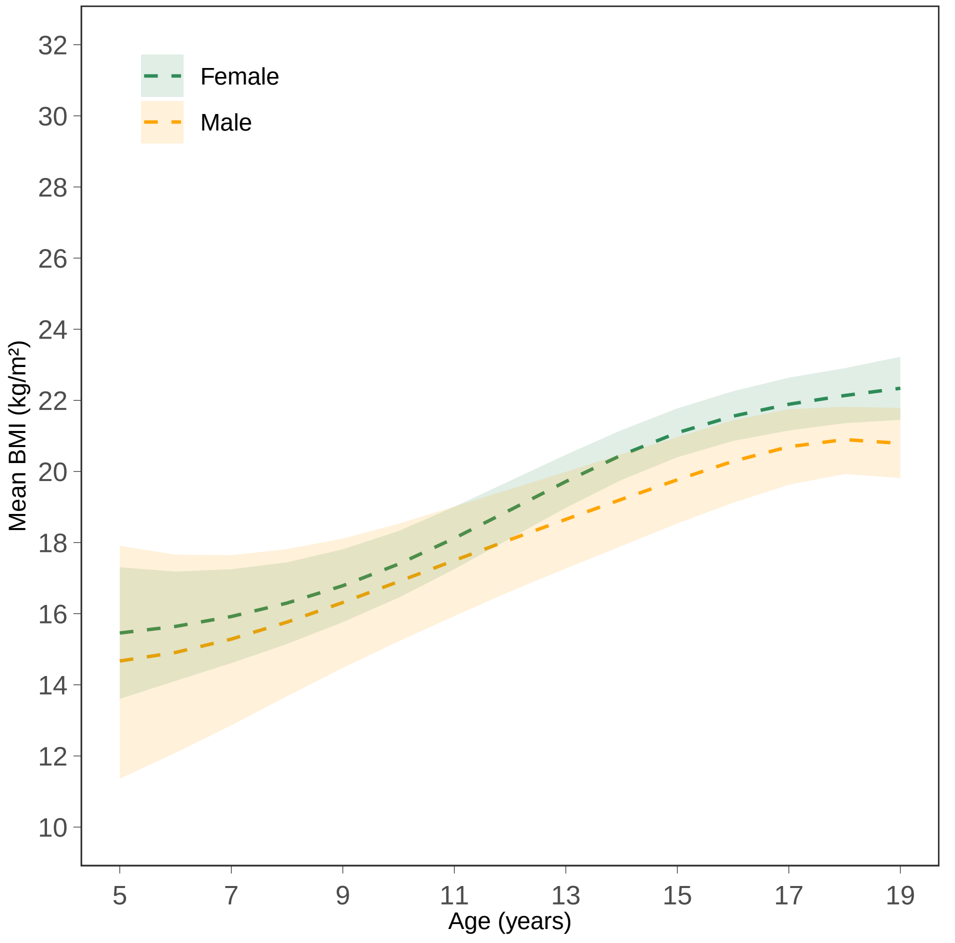

Mozambique

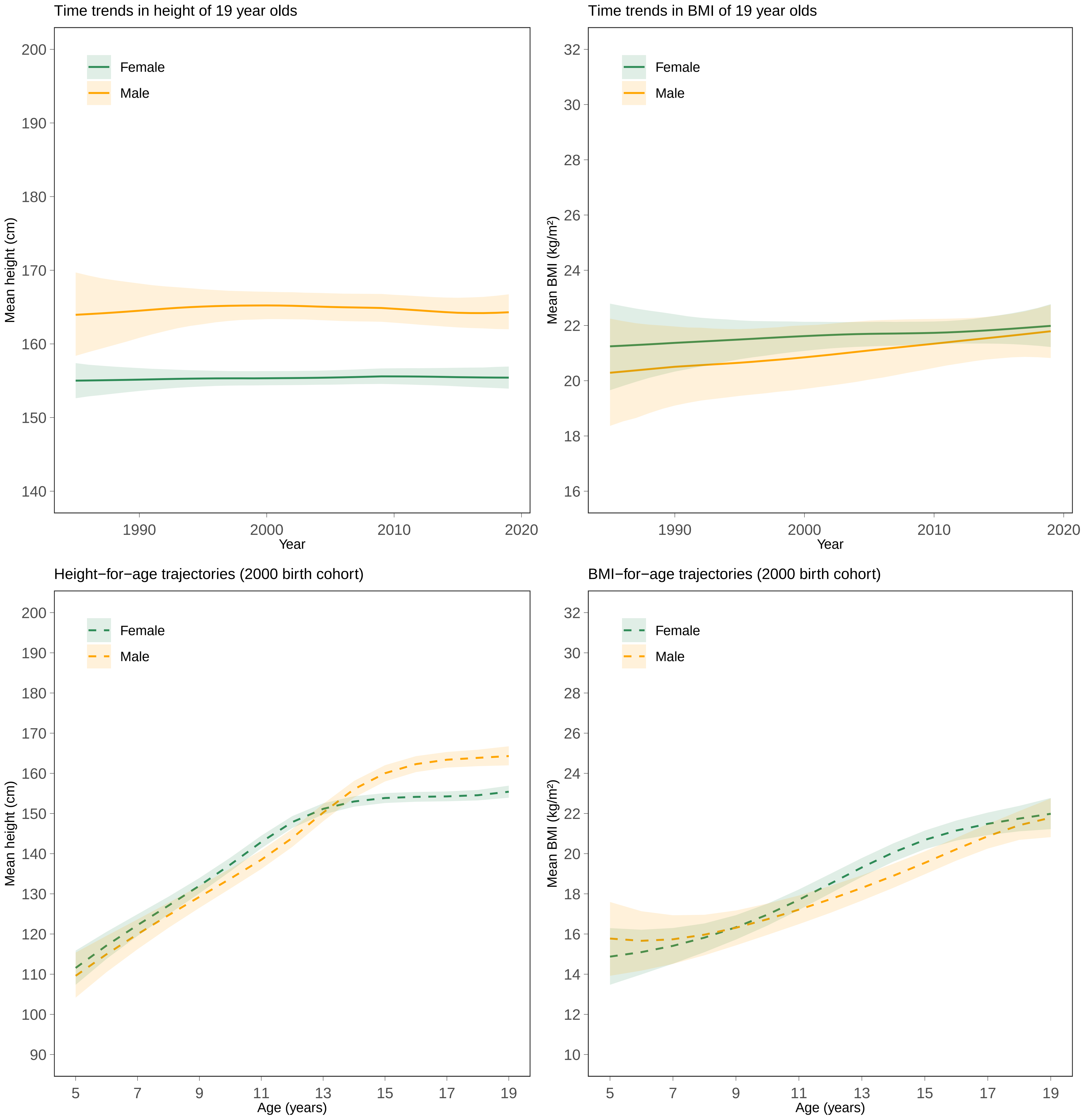

Time trends in height of 19 year olds
Female
Male
Mean height (cm)
200
190
180
170
160
150
140
1990
2000
2010
2020
Year
Time trends in BMI of 19 year olds
Female
Male
Mean BMI (kg/m²)
32
30
28
26
24
22
20
18
16
1990
2000
2010
2020
Year
Height−for−age trajectories (2000 birth cohort)
Female
Male
Mean height (cm)
200
190
180
170
160
150
140
130
120
110
100
90
5
7
9
11
13
15
17
19
Age (years)
BMI−for−age trajectories (2000 birth cohort)
Female
Male
Mean BMI (kg/m²)
32
30
28
26
24
22
20
18
16
14
12
10
5
7
9
11
13
15
17
19
Age (years)

Myanmar

Time trends in height of 19 year olds

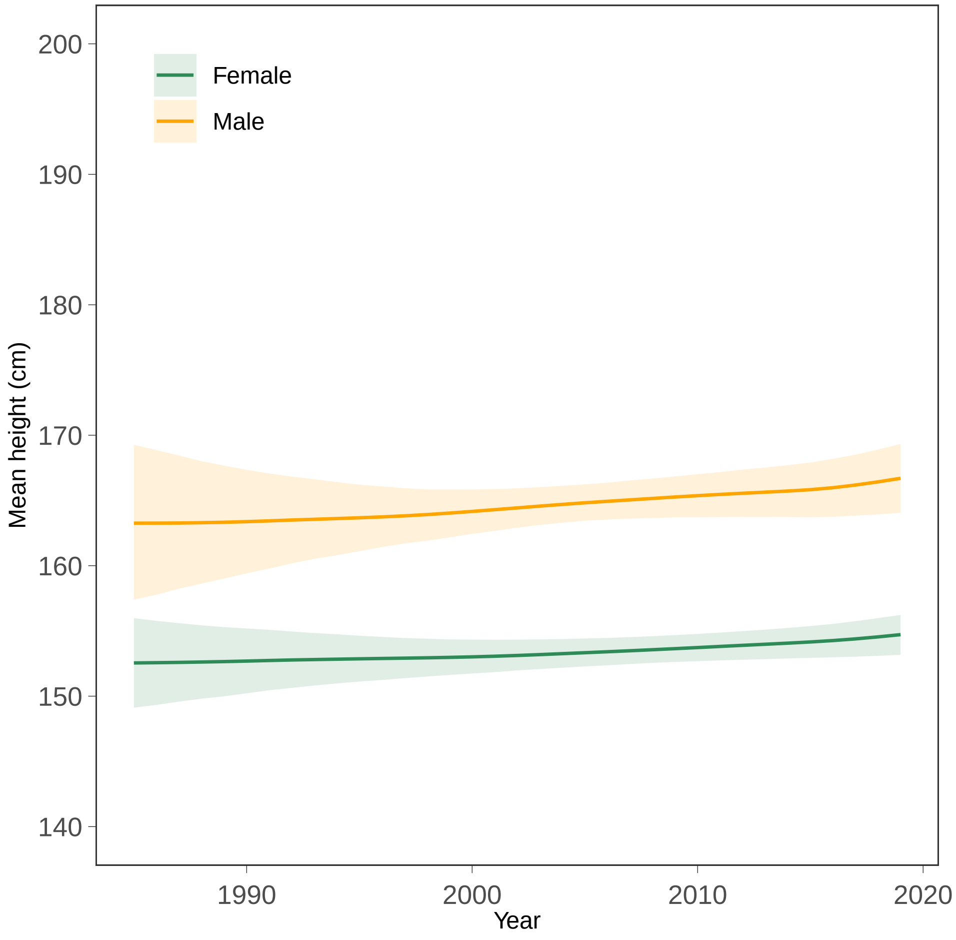

Time trends in BMI of 19 year olds

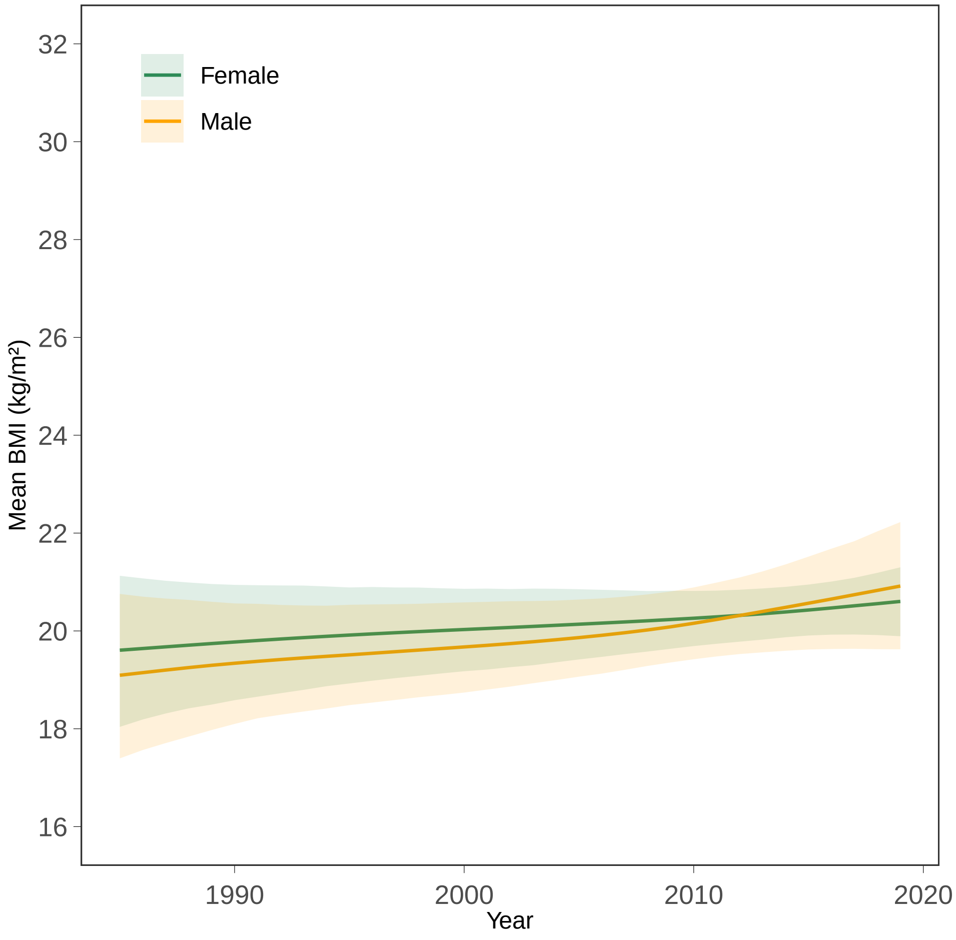

Height−for−age trajectories (2000 birth cohort)

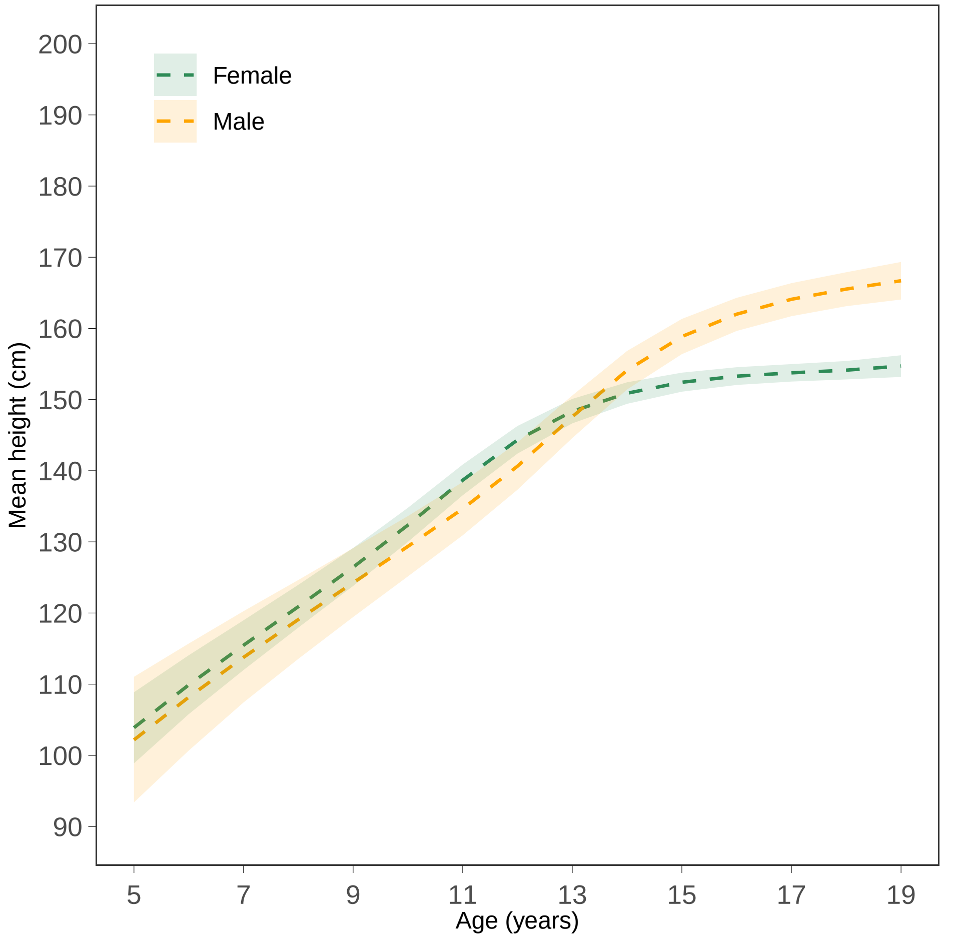

BMI−for−age trajectories (2000 birth cohort)

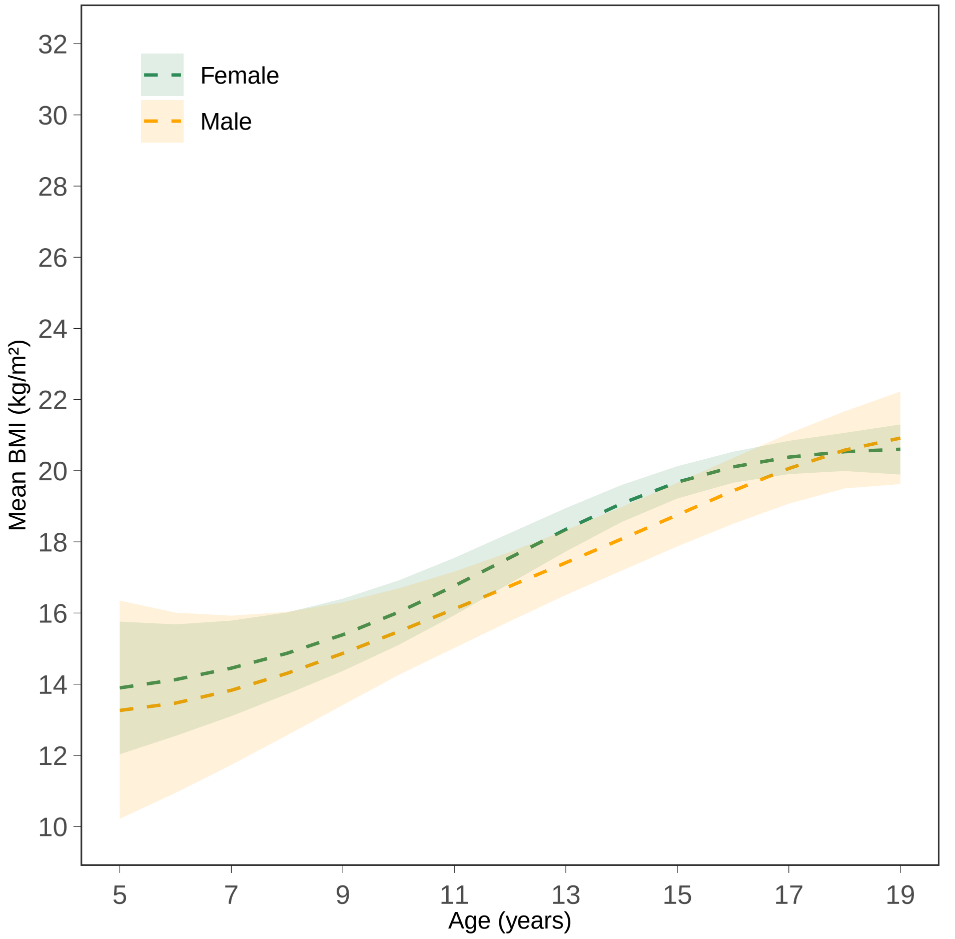

Namibia

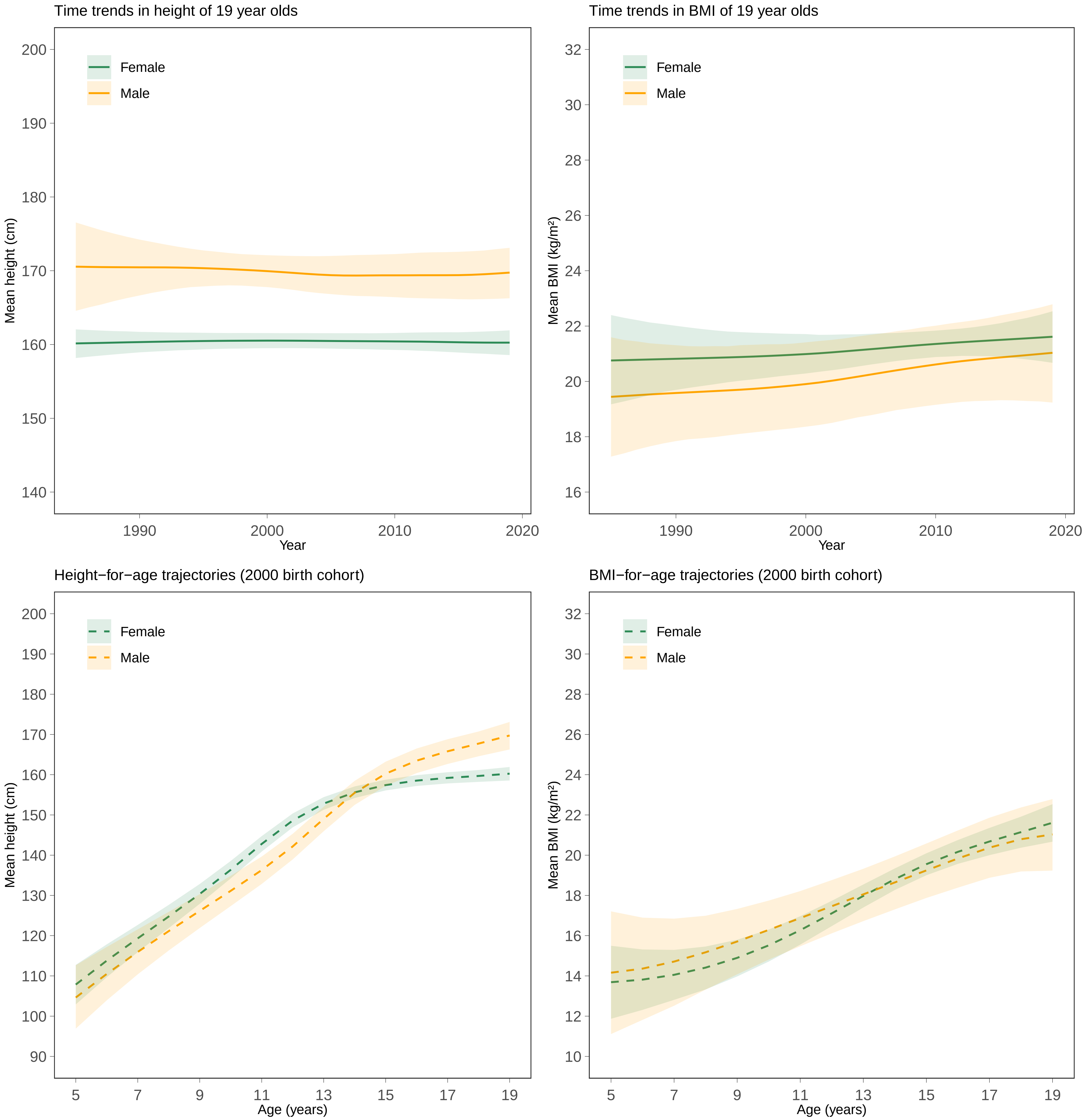
Time trends in height of 19 year olds
Female
Male
Mean height (cm)
Year
Time trends in BMI of 19 year olds
Female
Male
Mean BMI (kg/m²)
Year
Height−for−age trajectories (2000 birth cohort)
Female
Male
Mean height (cm)
Age (years)
BMI−for−age trajectories (2000 birth cohort)
Female
Male
Mean BMI (kg/m²)
Age (years)

Nauru

Time trends in height of 19 year olds

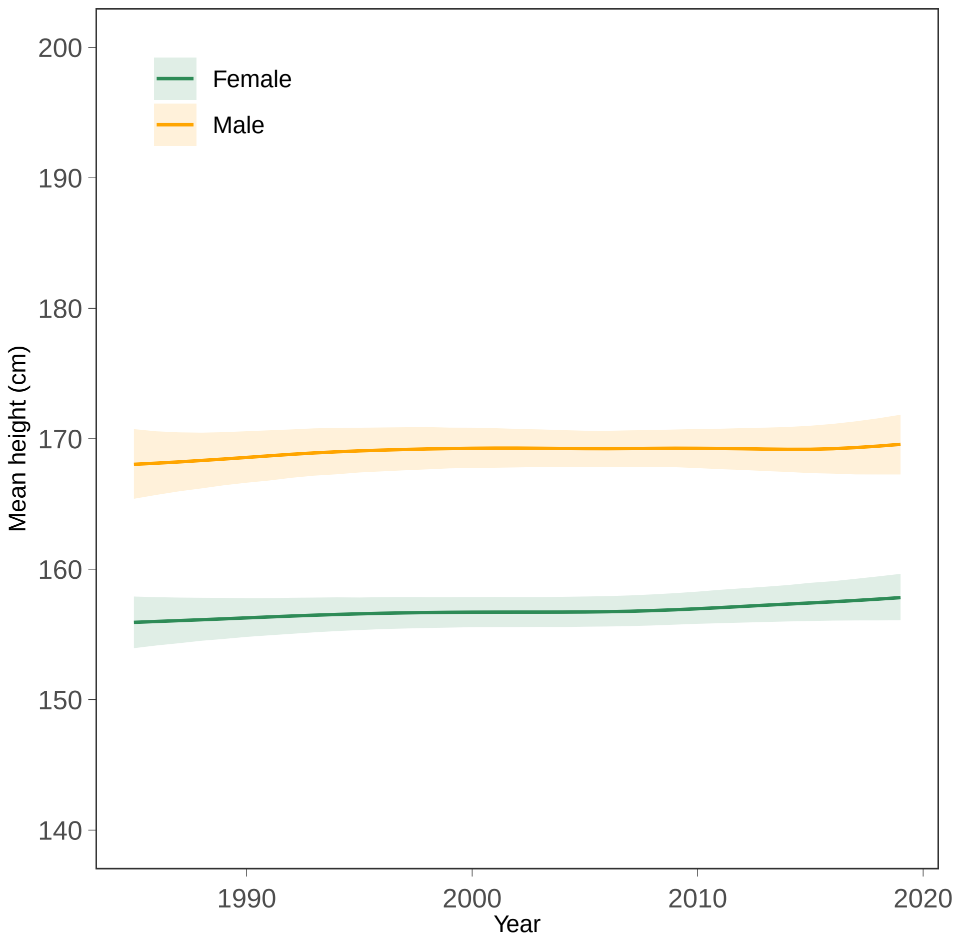

Time trends in BMI of 19 year olds

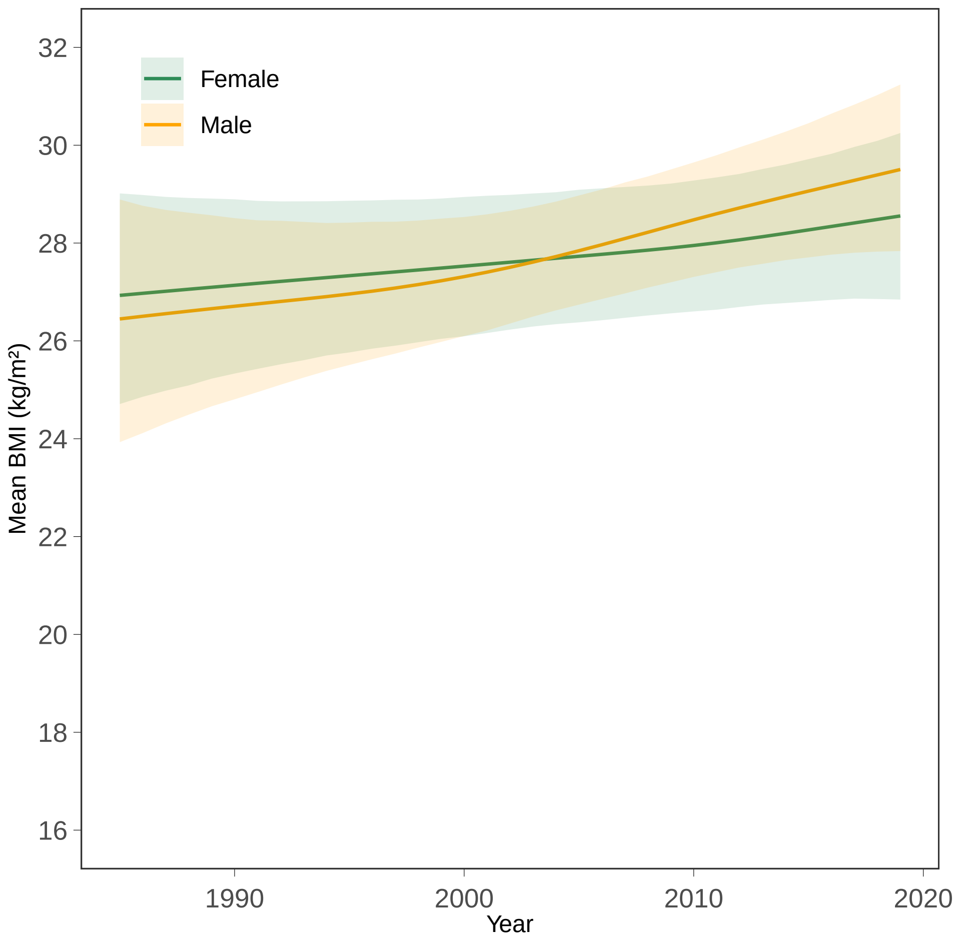

Height−for−age trajectories (2000 birth cohort)

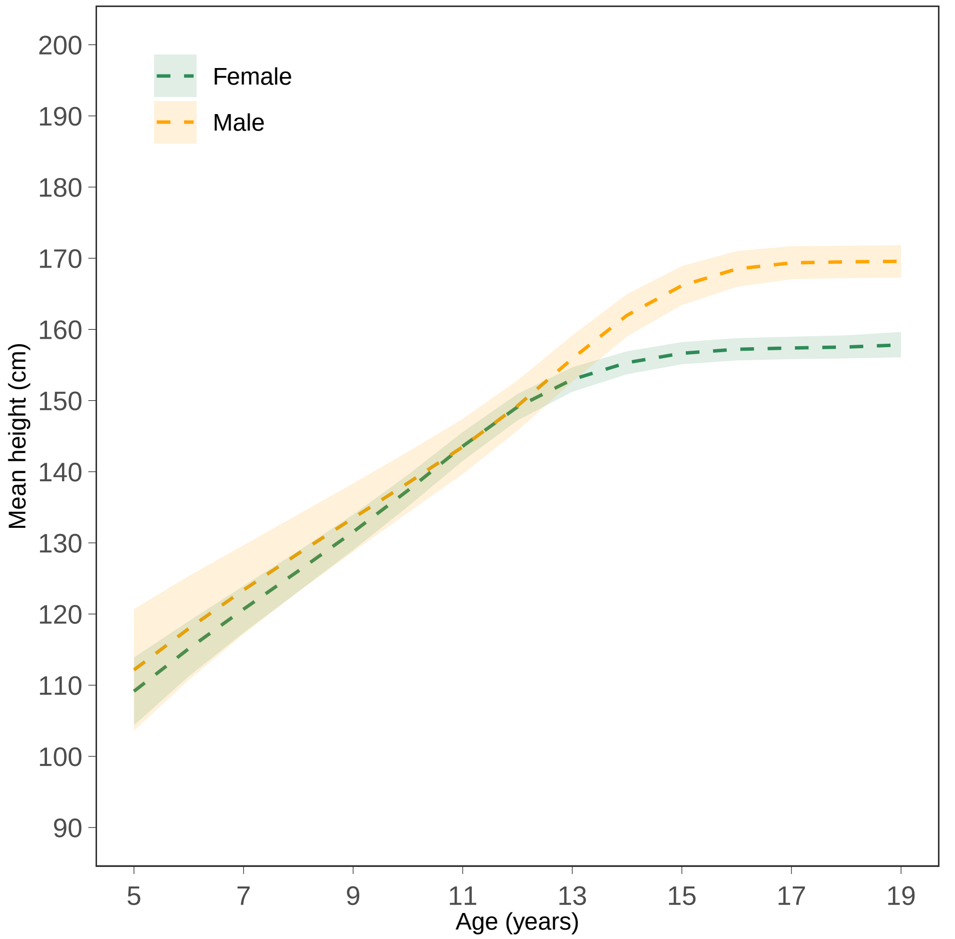

BMI−for−age trajectories (2000 birth cohort)

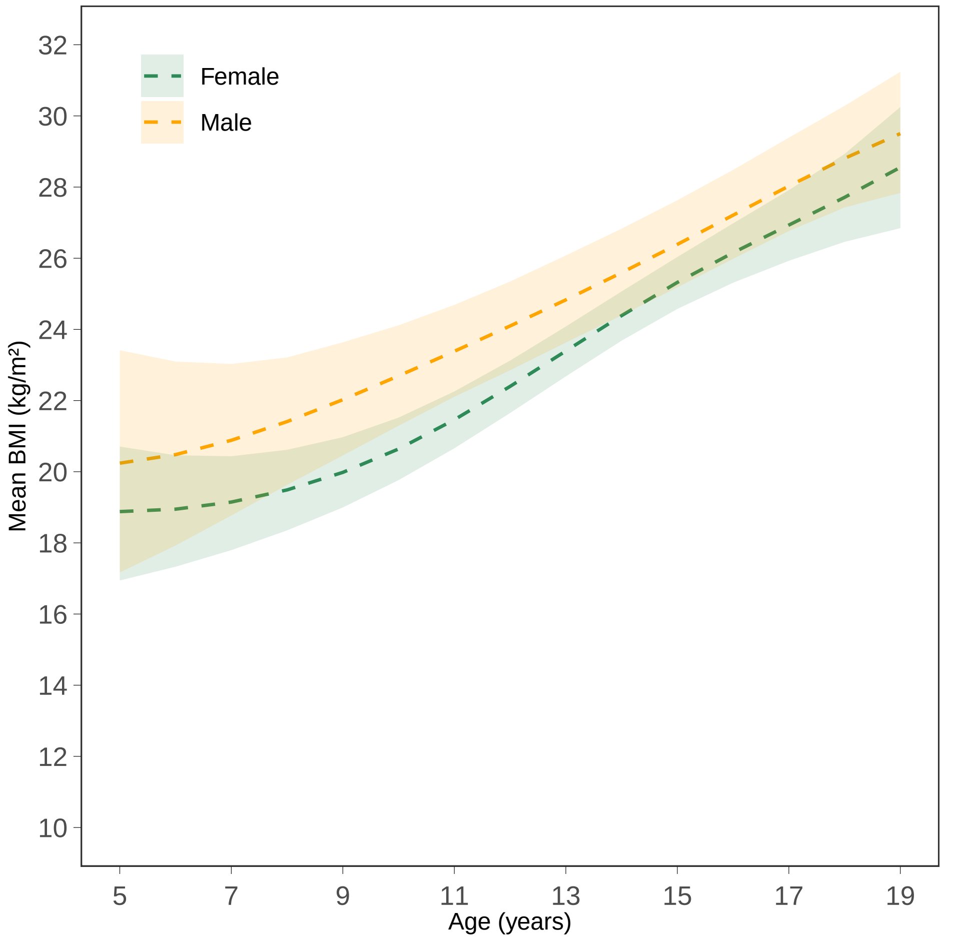

Nepal

Time trends in height of 19 year olds

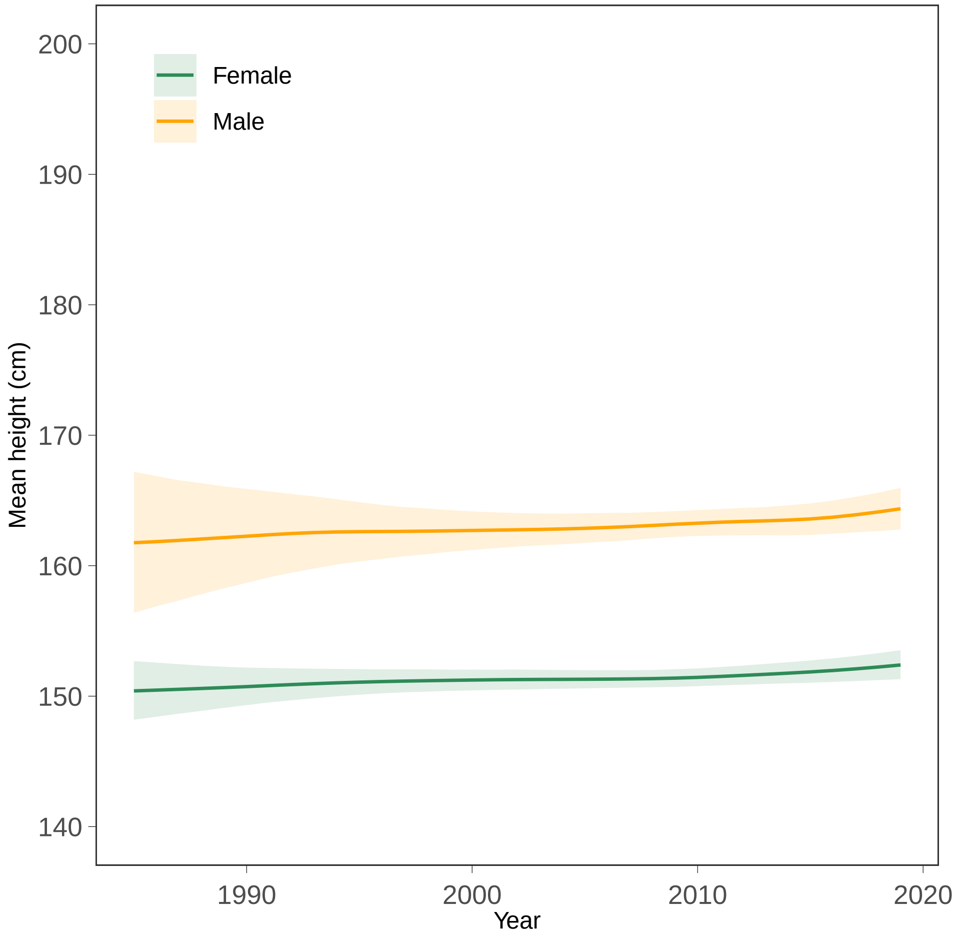

Time trends in BMI of 19 year olds

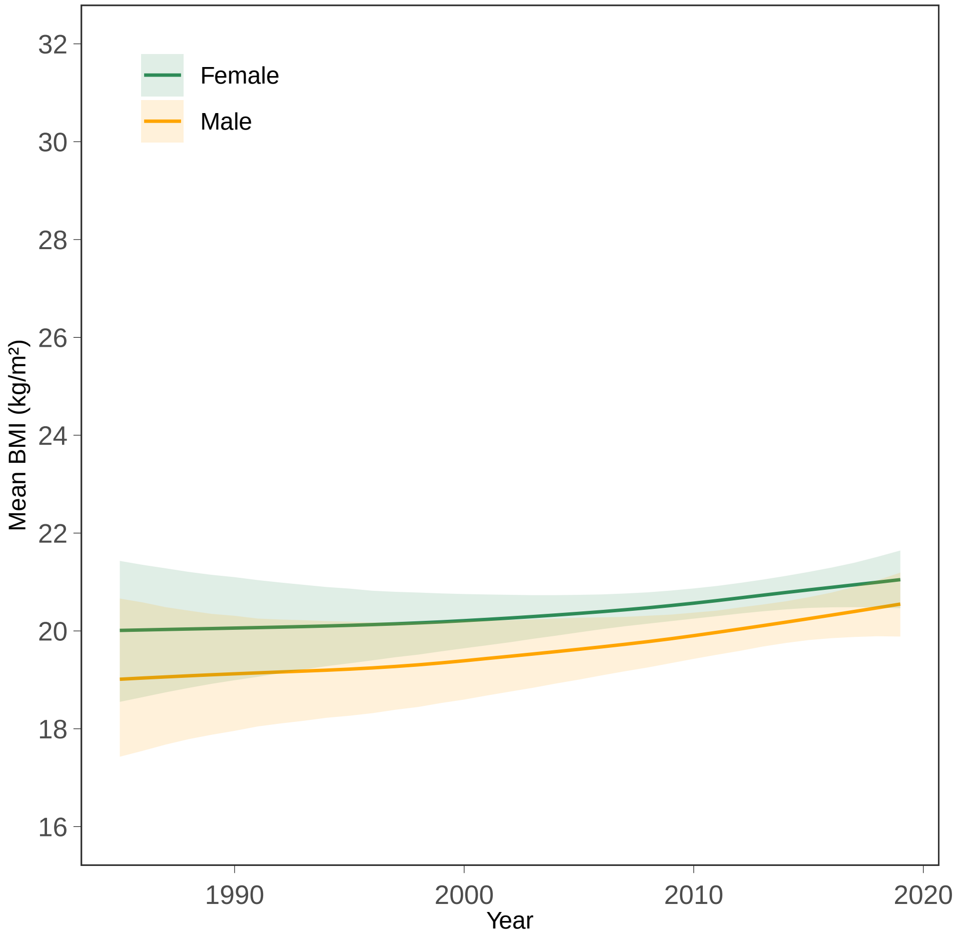

Height−for−age trajectories (2000 birth cohort)

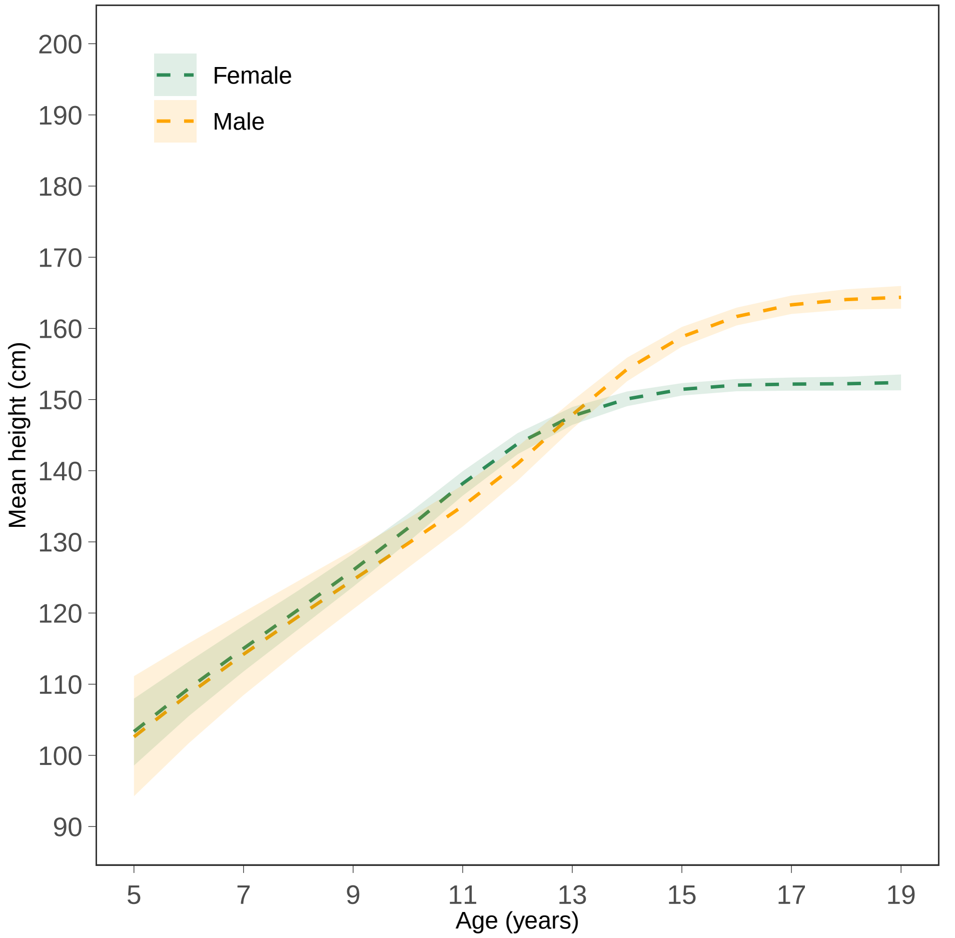

BMI−for−age trajectories (2000 birth cohort)

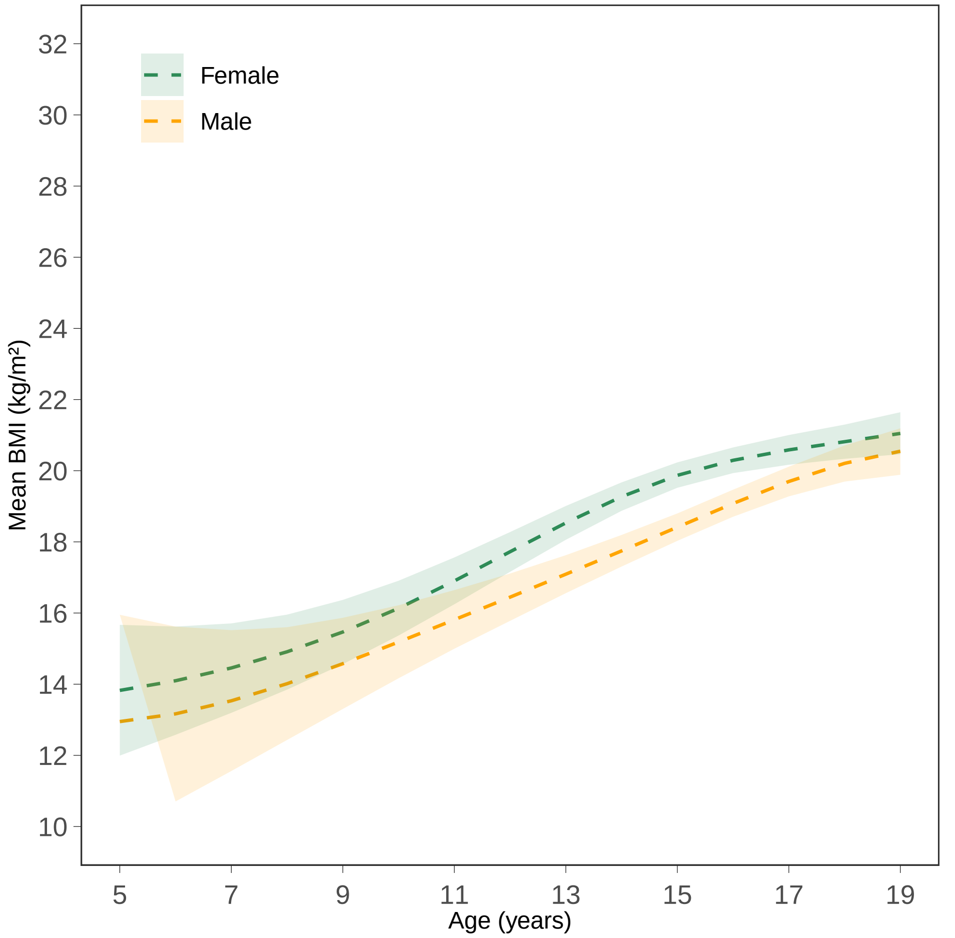

Netherlands

Time trends in height of 19 year olds

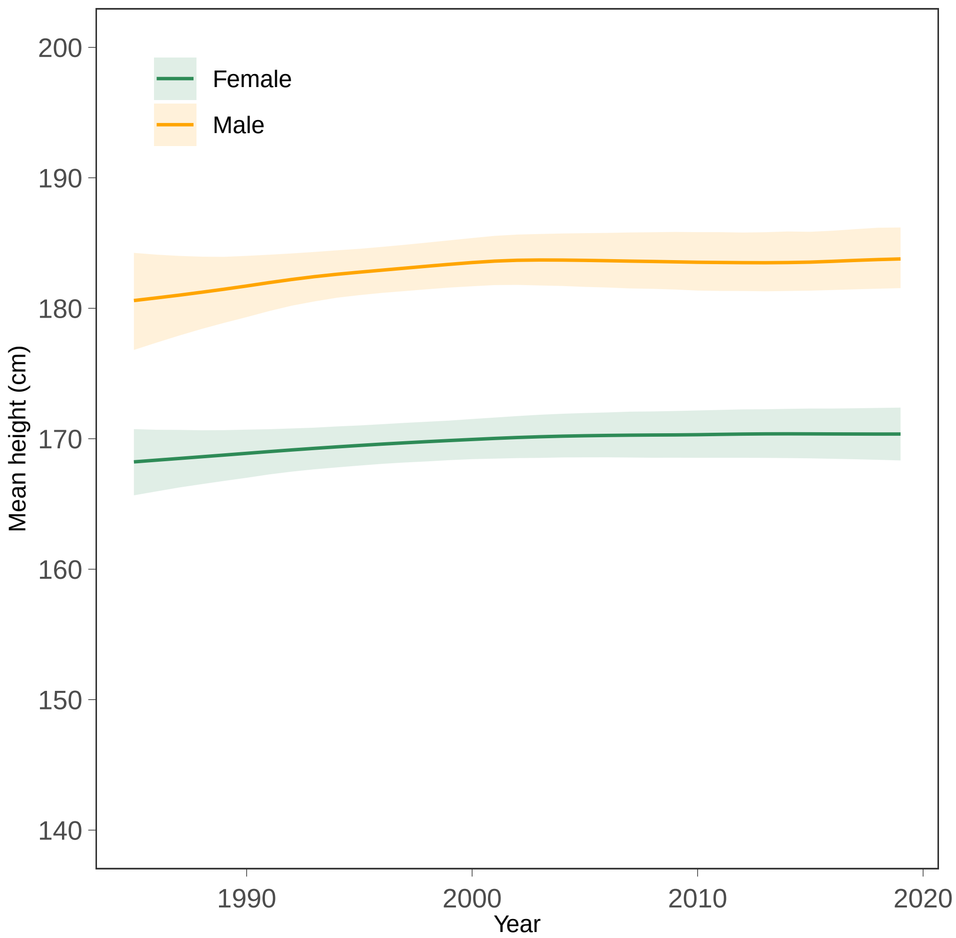

Time trends in BMI of 19 year olds

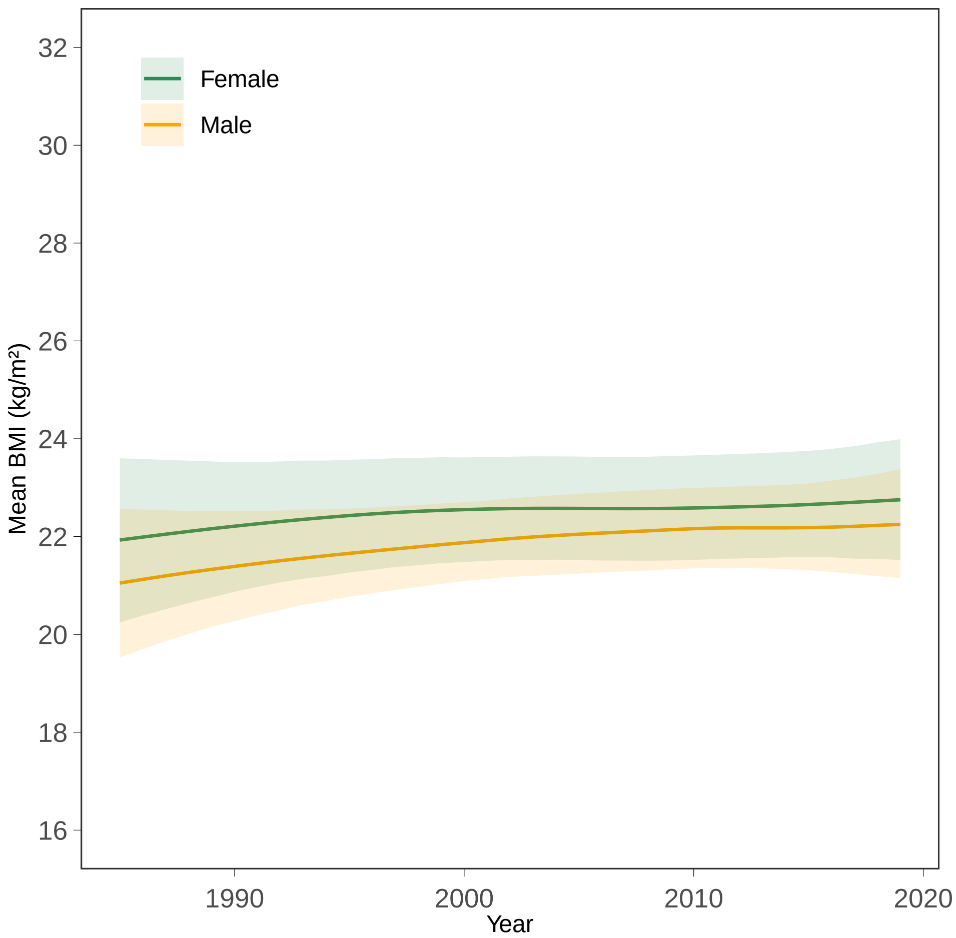

Height−for−age trajectories (2000 birth cohort)

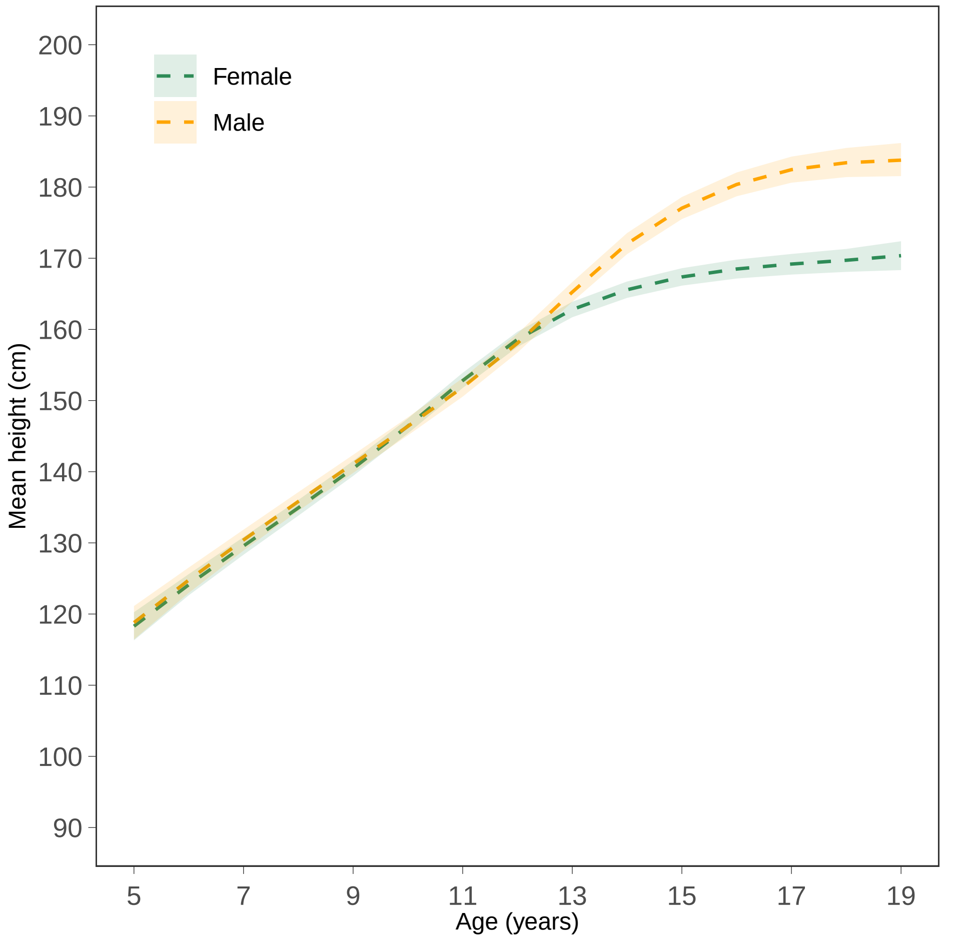

BMI−for−age trajectories (2000 birth cohort)

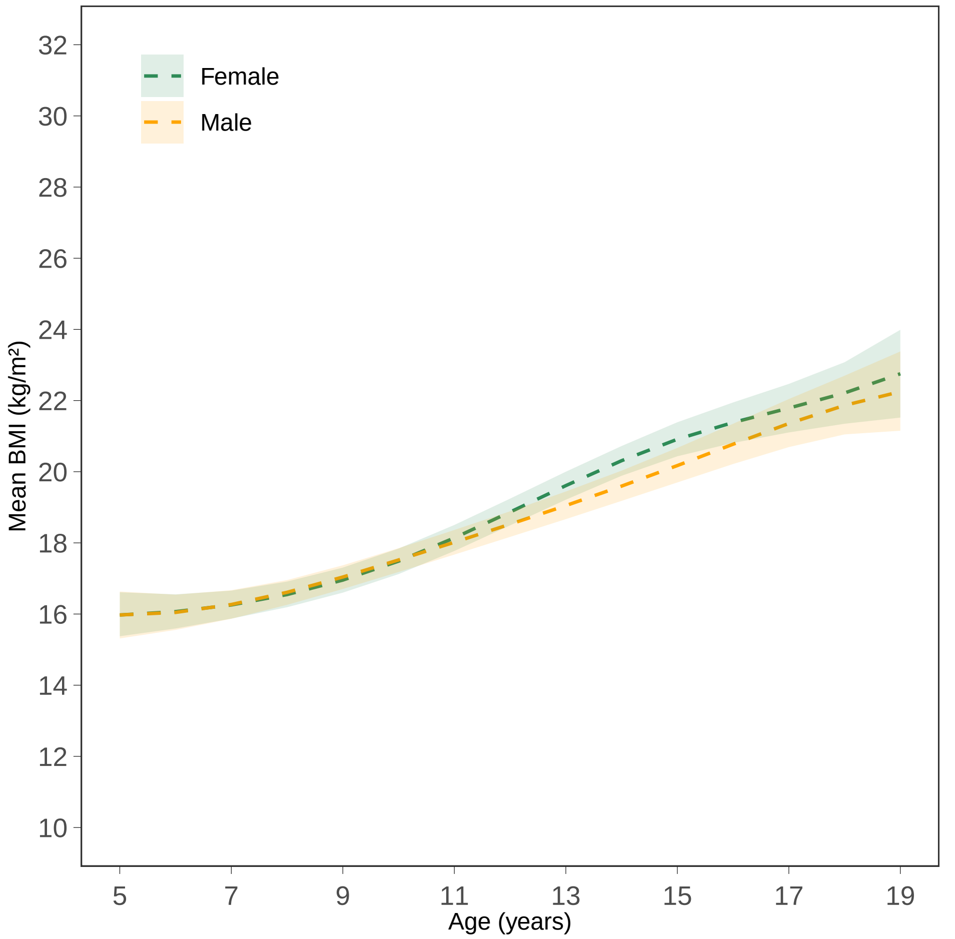

New Zealand

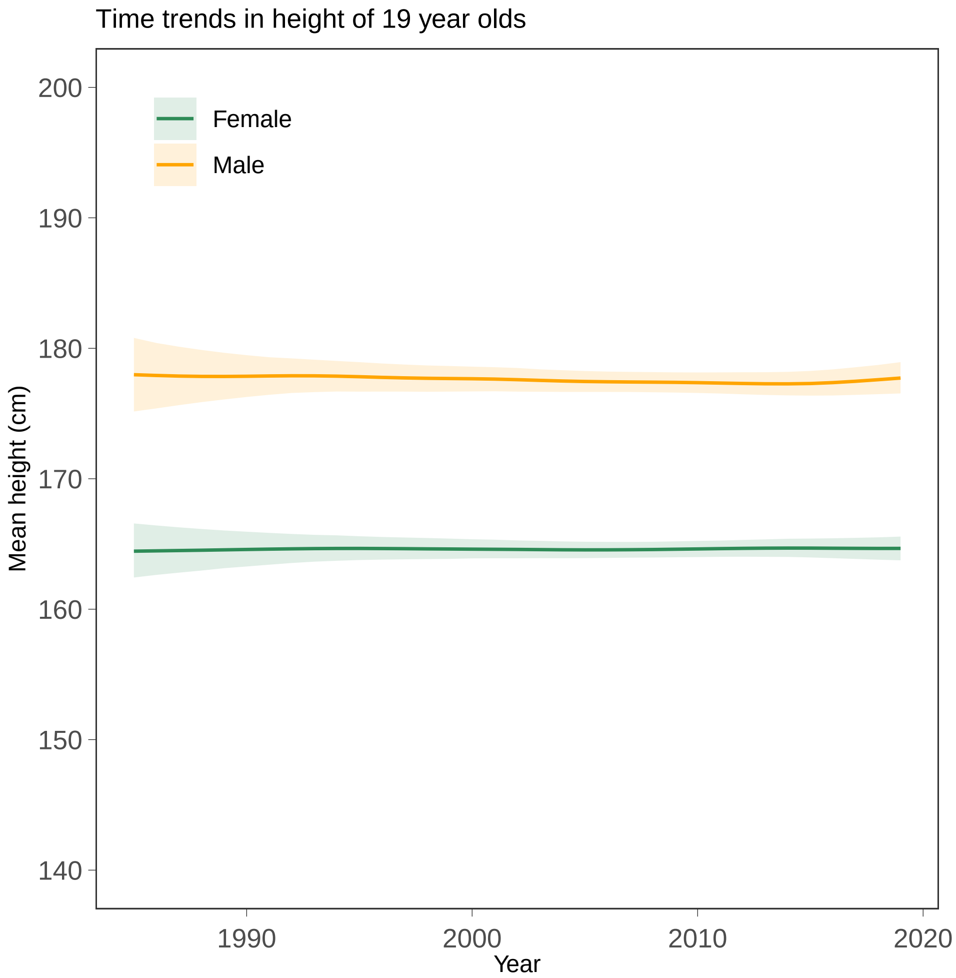
Time trends in height of 19 year olds
Female
Male
Mean height (cm)
200
190
180
170
160
150
140
1990
2000
2010
2020
Year

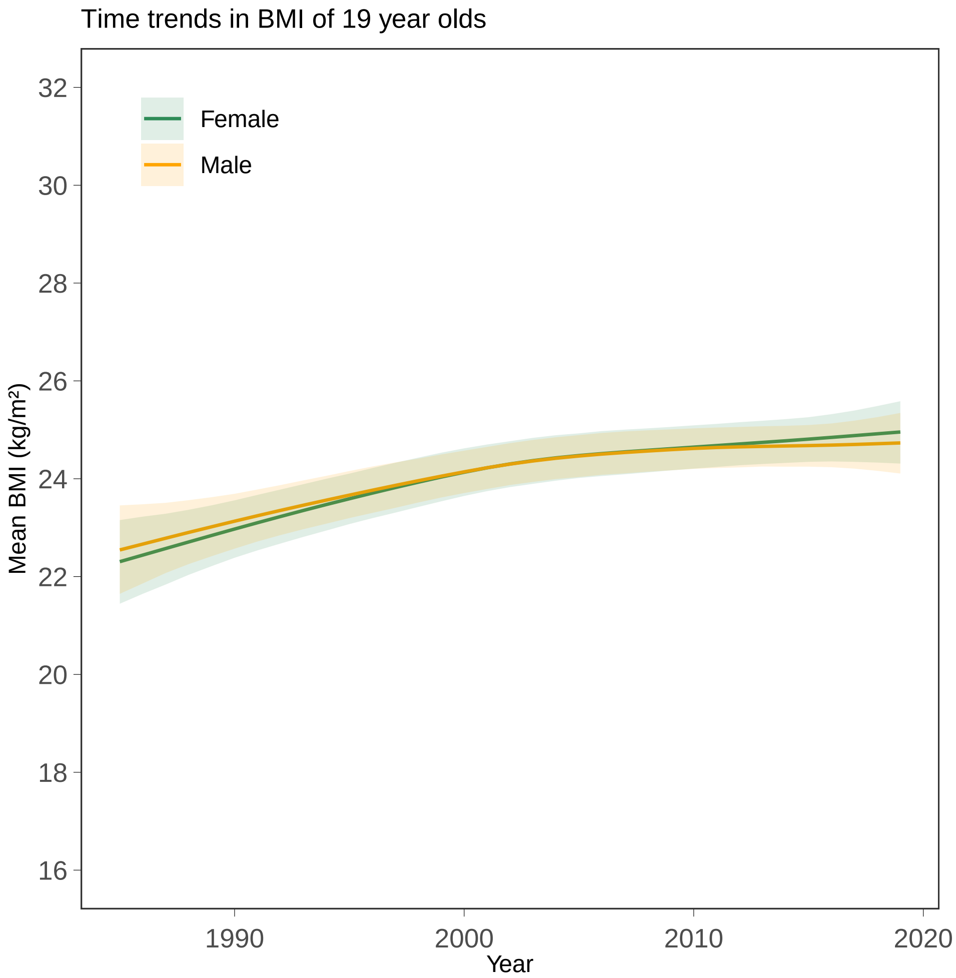
Time trends in BMI of 19 year olds
Female
Male
Mean BMI (kg/m²)
32
30
28
26
24
22
20
18
16
1990
2000
2010
2020
Year

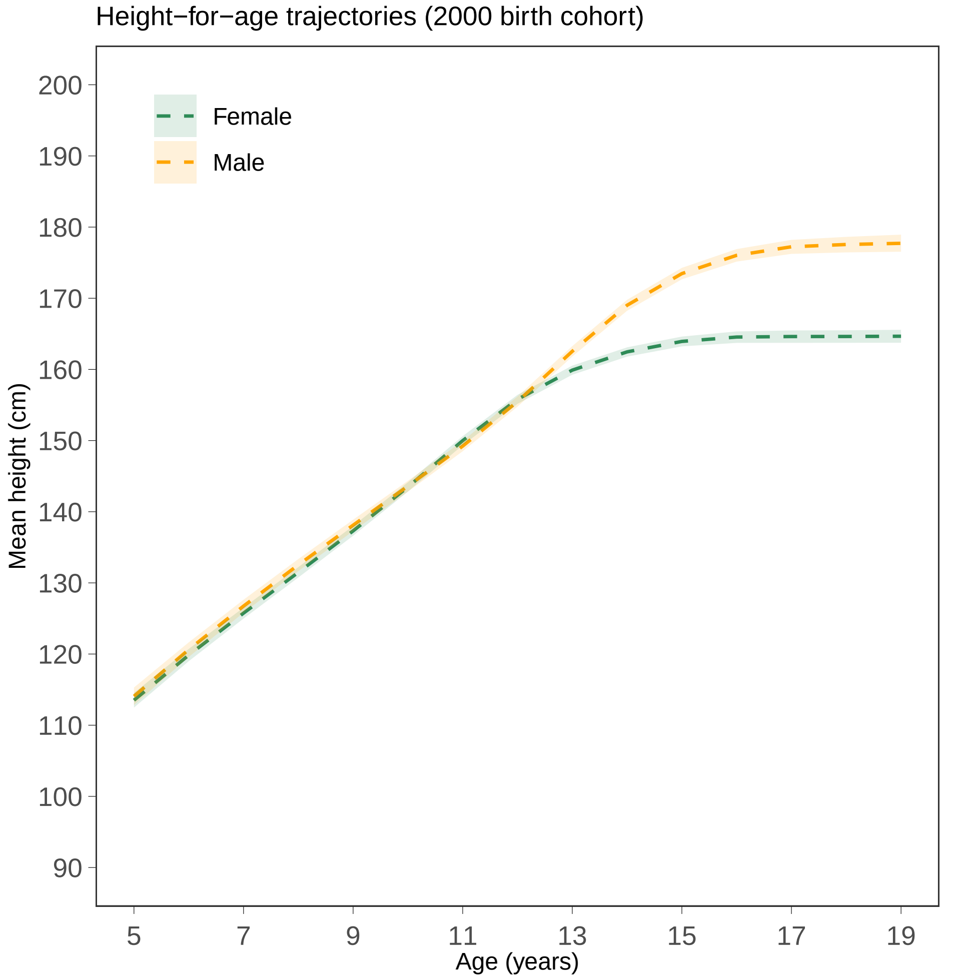
Height−for−age trajectories (2000 birth cohort)
Female
Male
Mean height (cm)
200
190
180
170
160
150
140
130
120
110
100
90
5
7
9
11
13
15
17
19
Age (years)

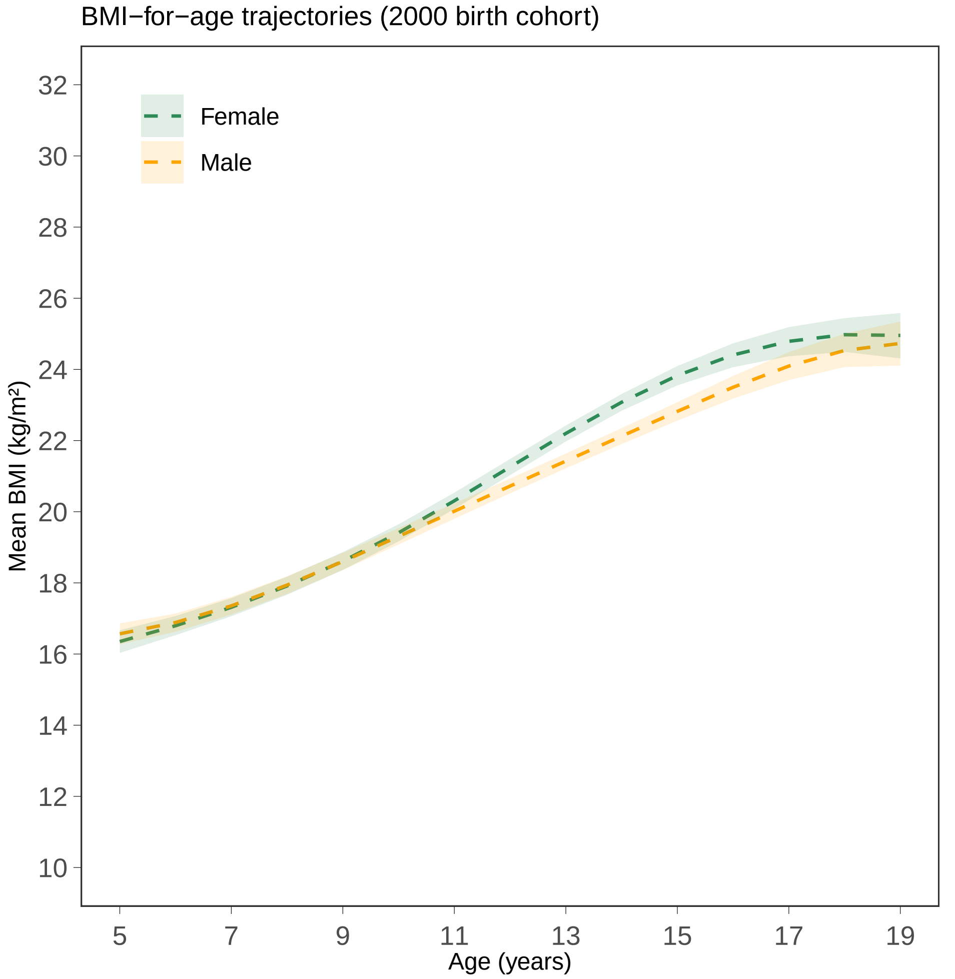
BMI−for−age trajectories (2000 birth cohort)
Female
Male
Mean BMI (kg/m²)
32
30
28
26
24
22
20
18
16
14
12
10
5
7
9
11
13
15
17
19
Age (years)

Nicaragua

Time trends in height of 19 year olds

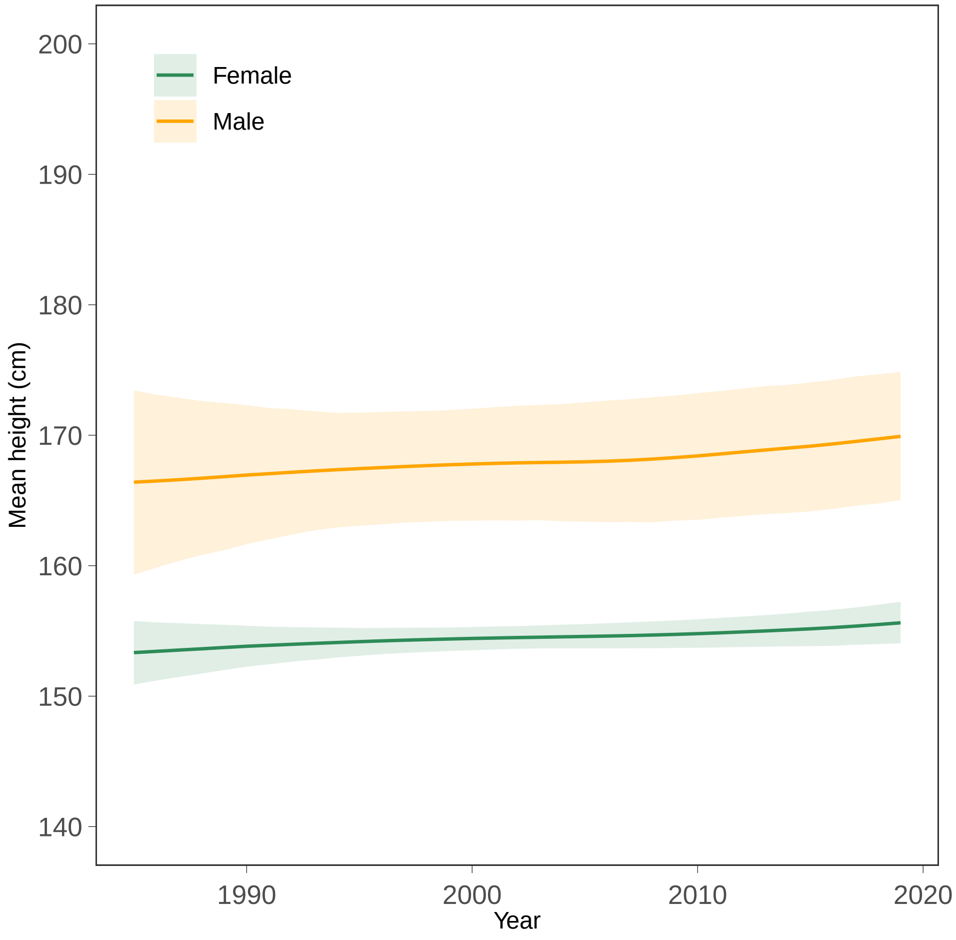

Time trends in BMI of 19 year olds

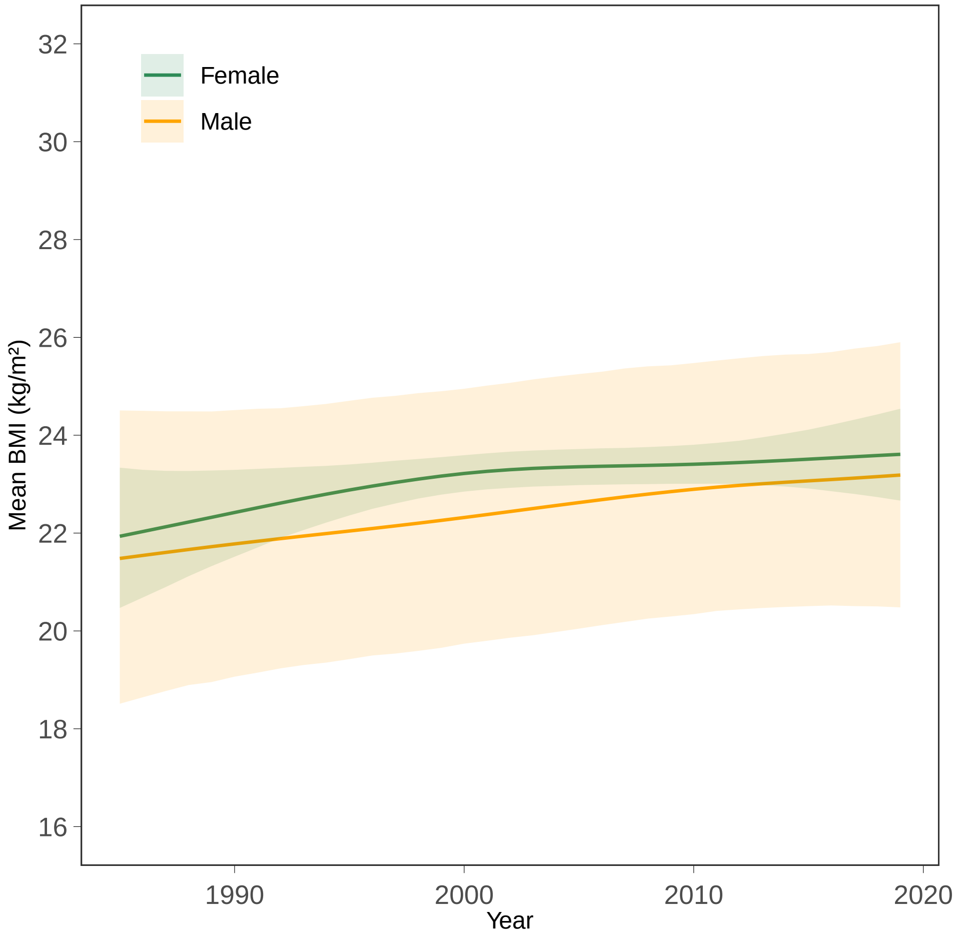

Height−for−age trajectories (2000 birth cohort)

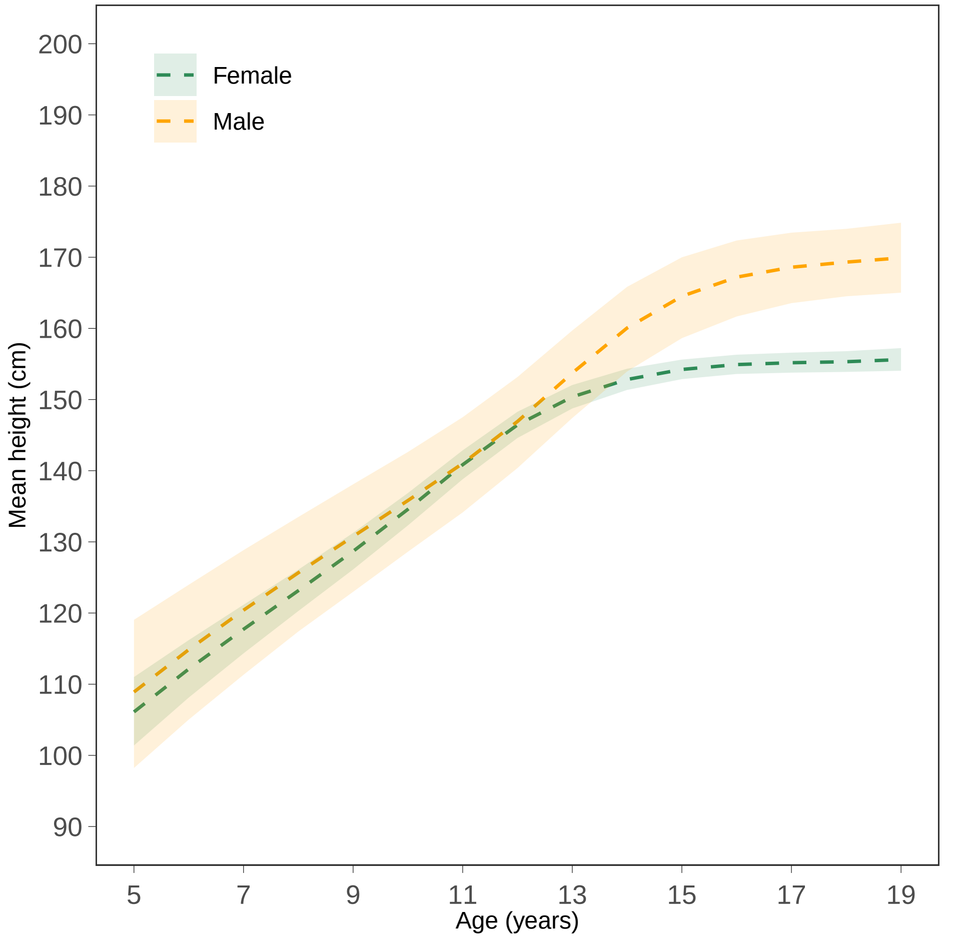

BMI−for−age trajectories (2000 birth cohort)

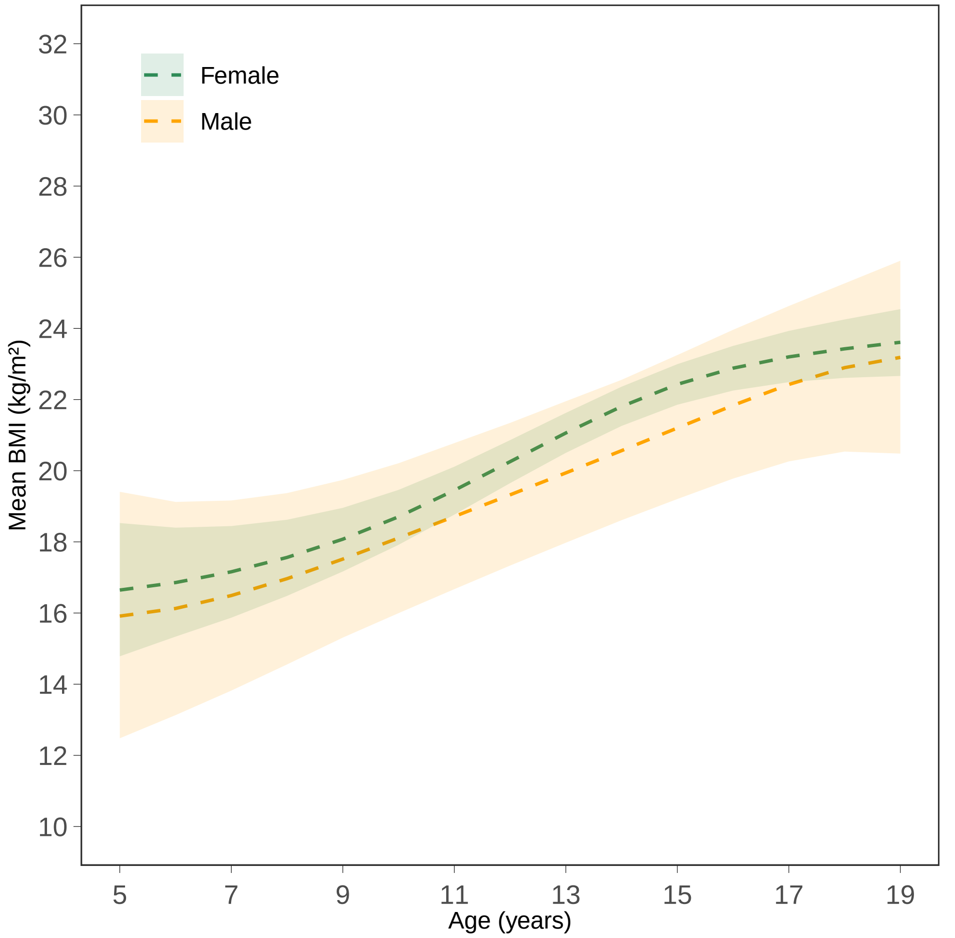

Niger

Time trends in height of 19 year olds

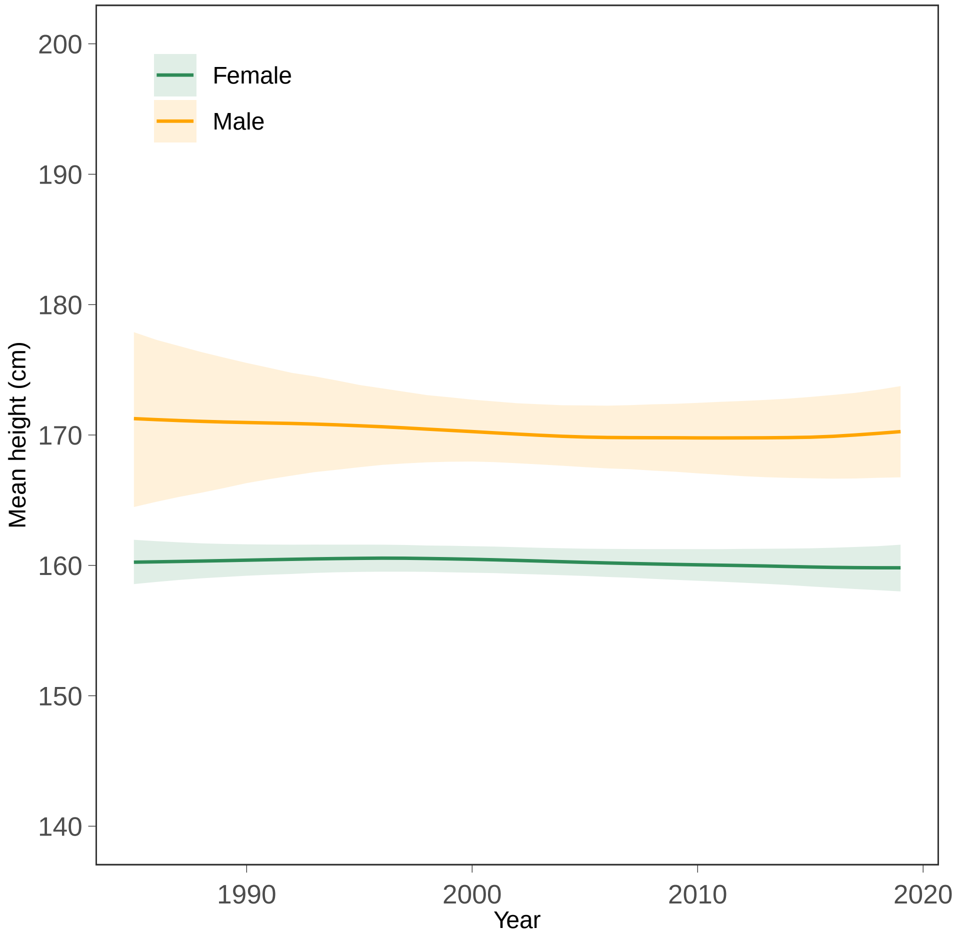

Time trends in BMI of 19 year olds

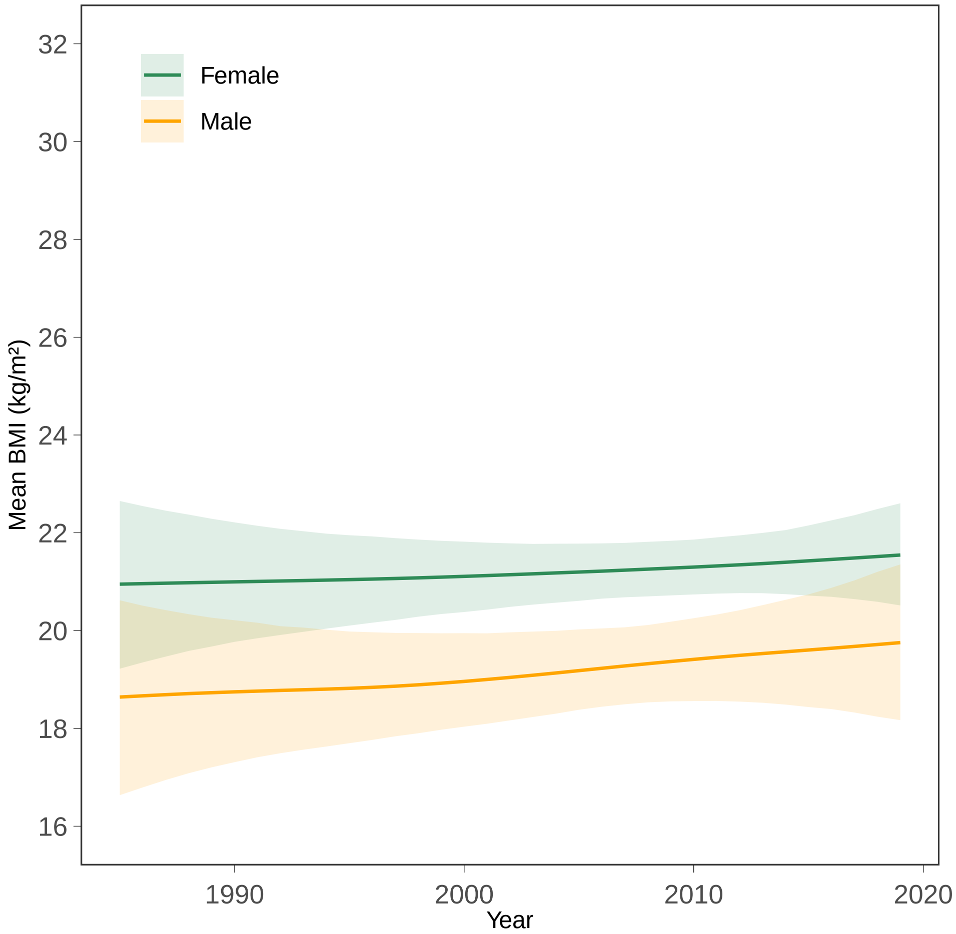

Height−for−age trajectories (2000 birth cohort)

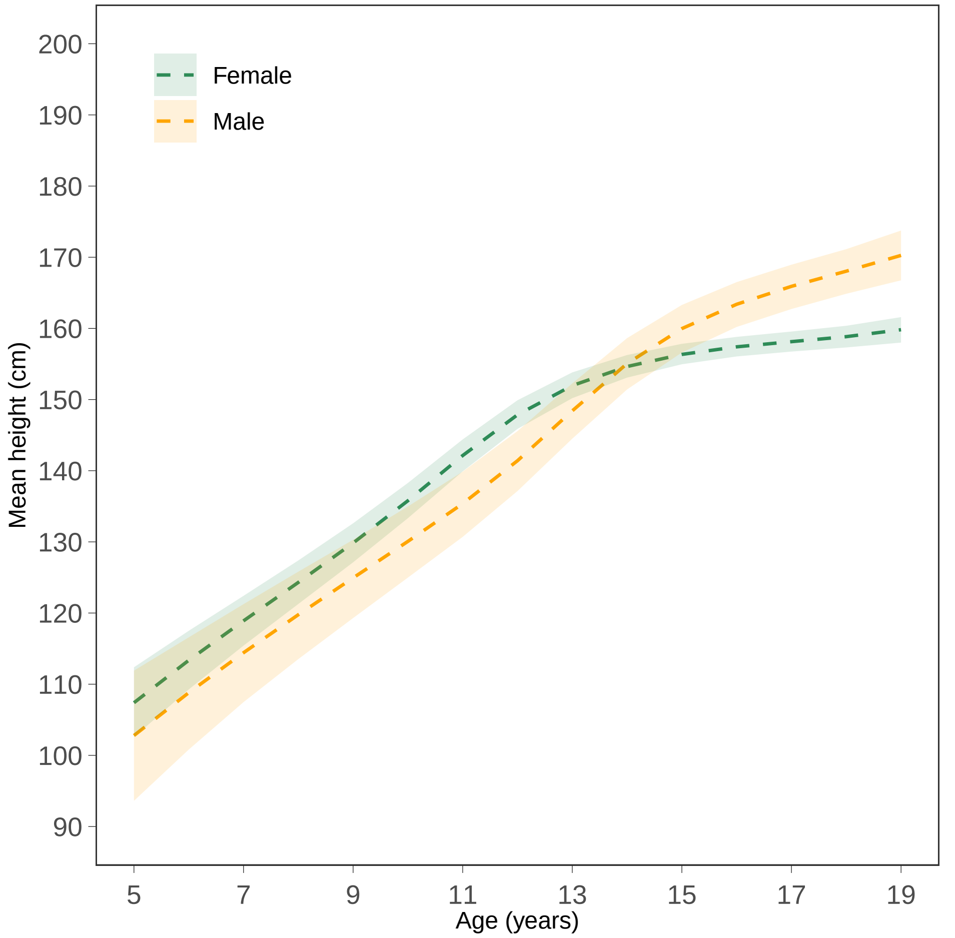

BMI−for−age trajectories (2000 birth cohort)

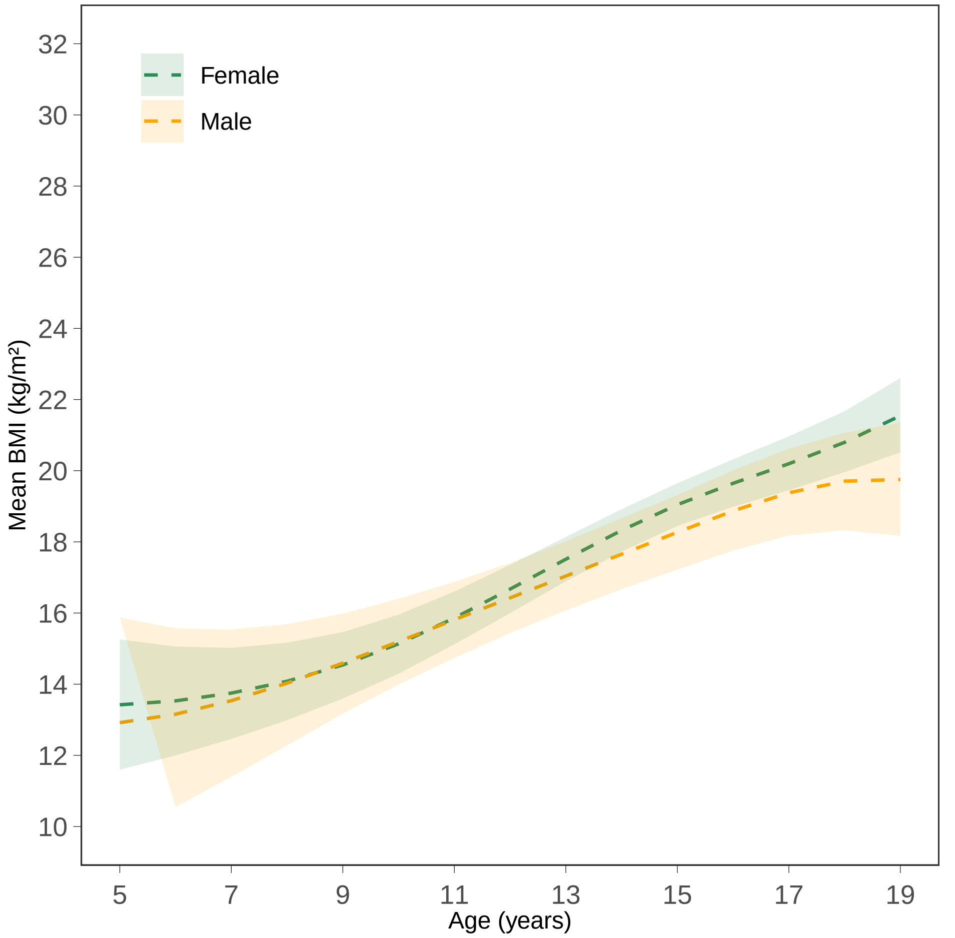

Nigeria

Time trends in height of 19 year olds

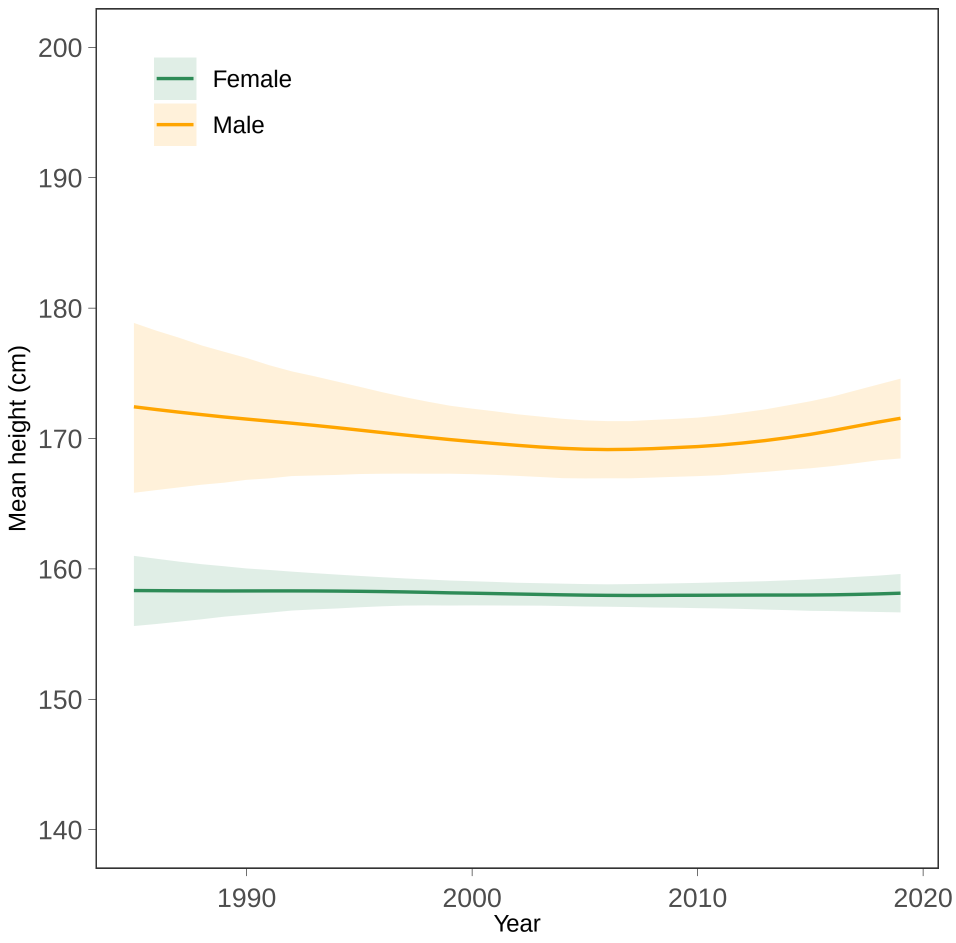

Time trends in BMI of 19 year olds

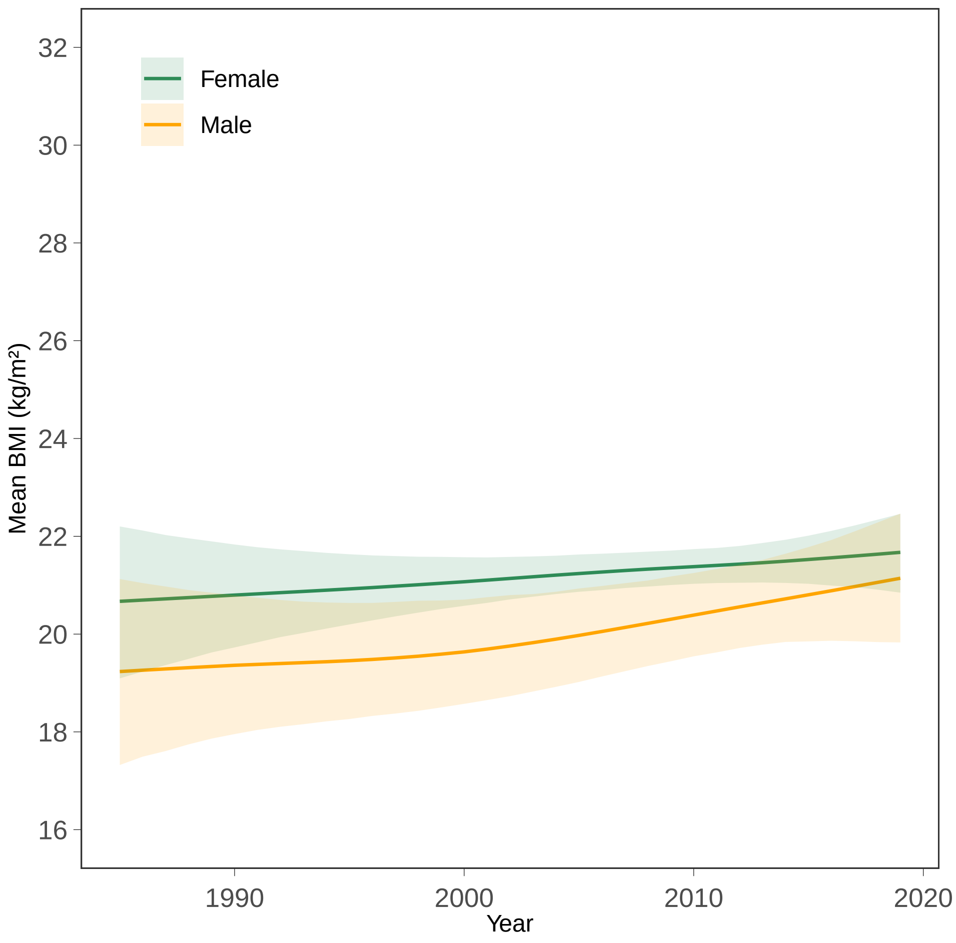

Height−for−age trajectories (2000 birth cohort)

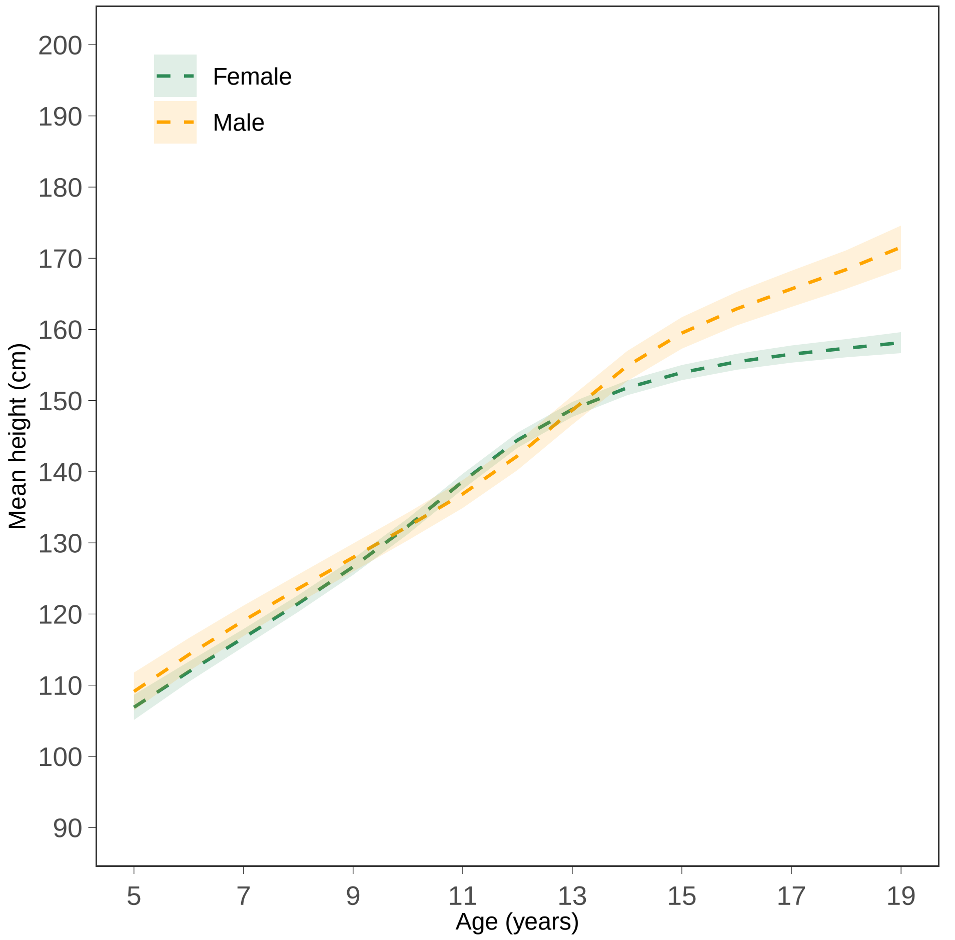

BMI−for−age trajectories (2000 birth cohort)

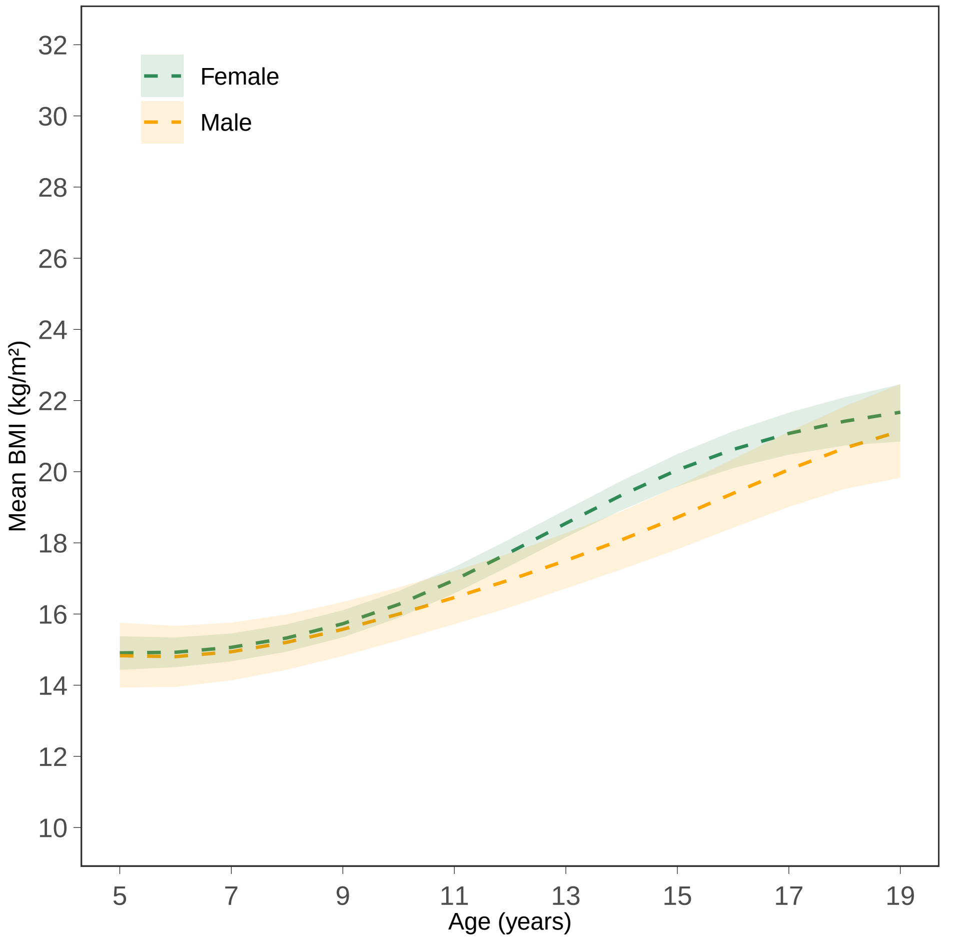

Niue

Time trends in height of 19 year olds

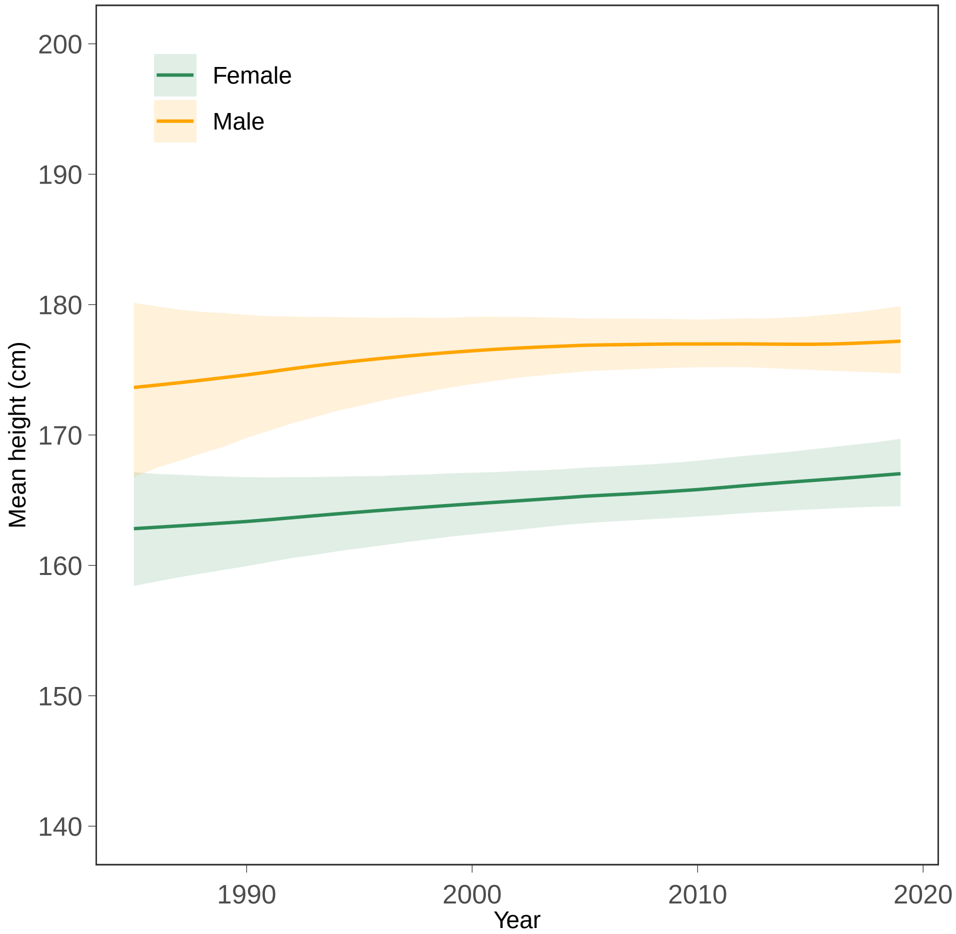

Time trends in BMI of 19 year olds

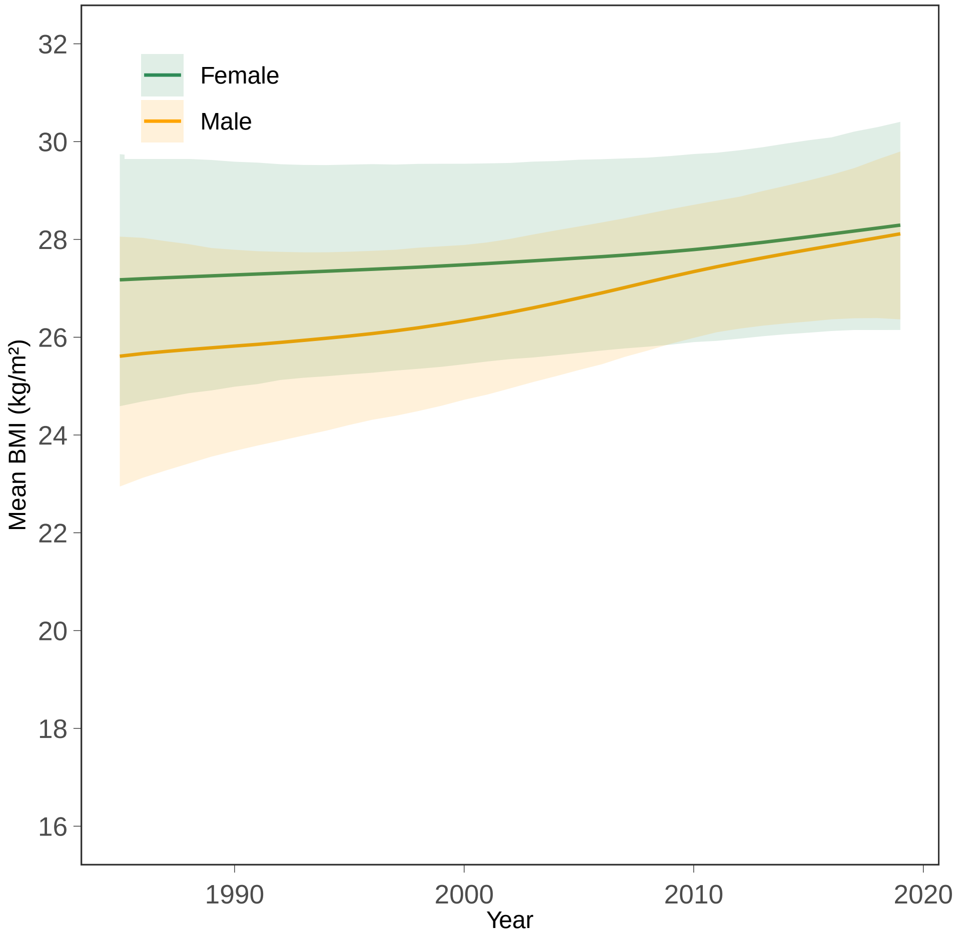

Height−for−age trajectories (2000 birth cohort)

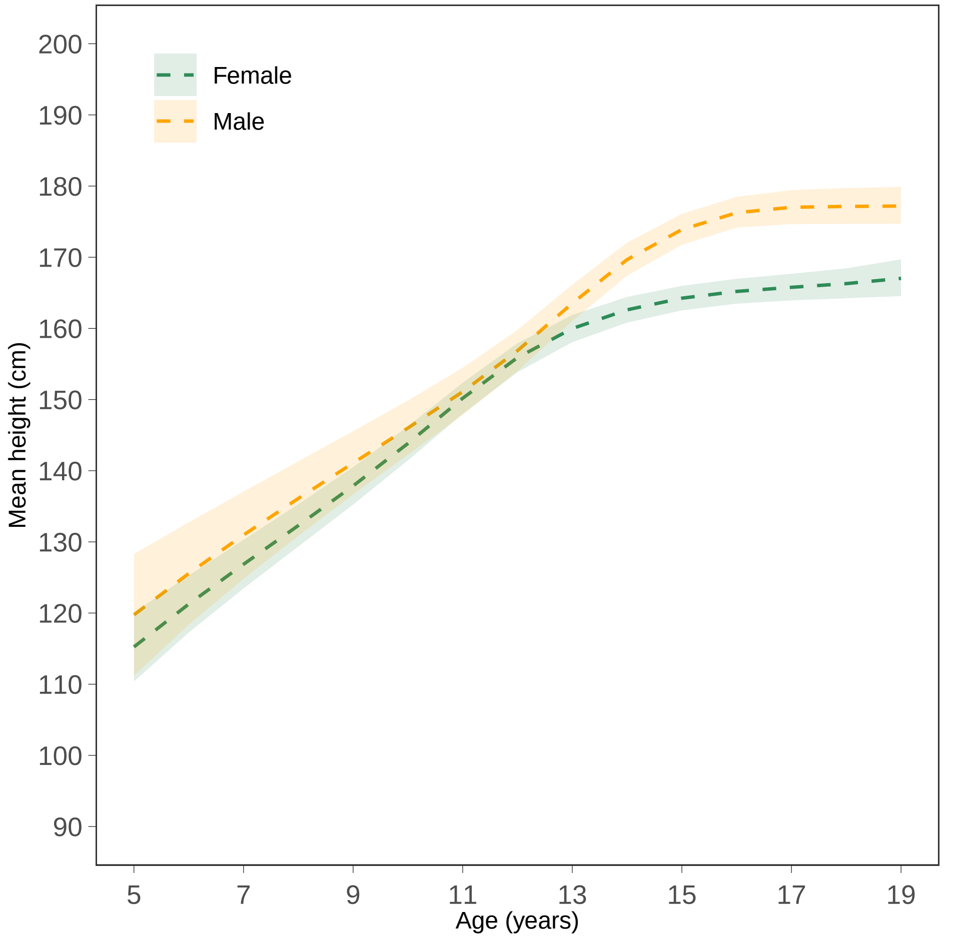

BMI−for−age trajectories (2000 birth cohort)

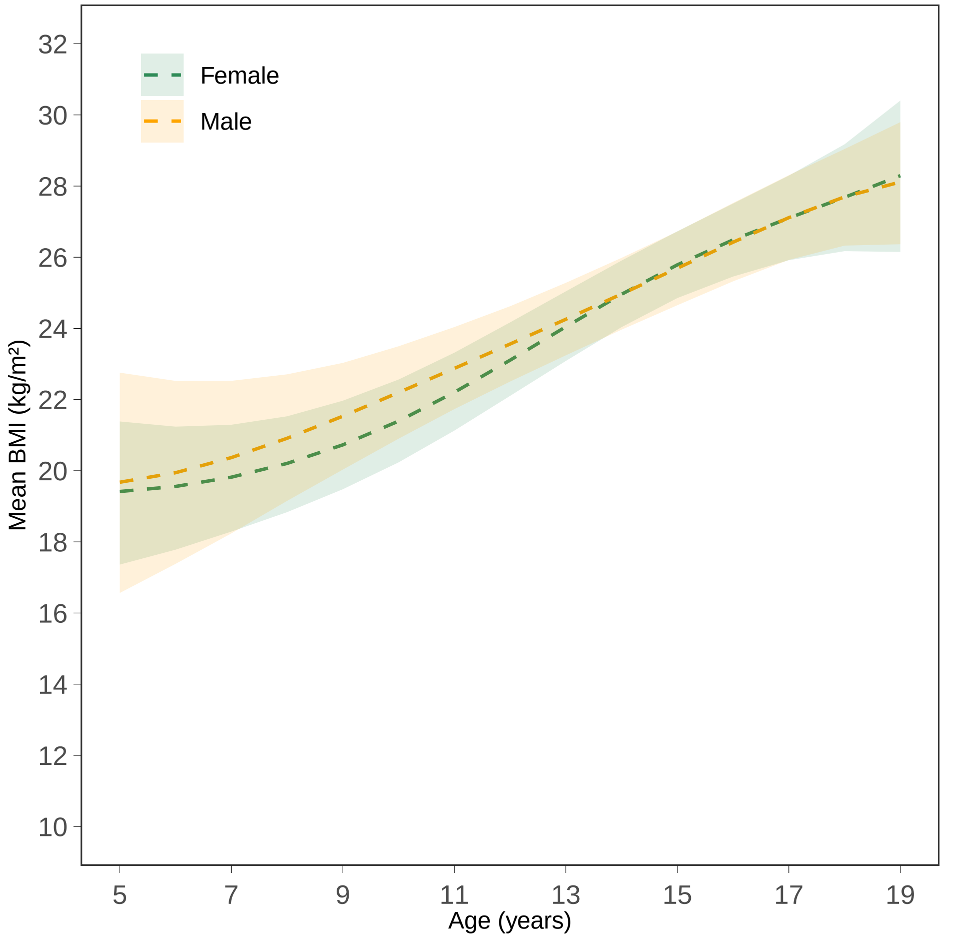

North Korea

Time trends in height of 19 year olds

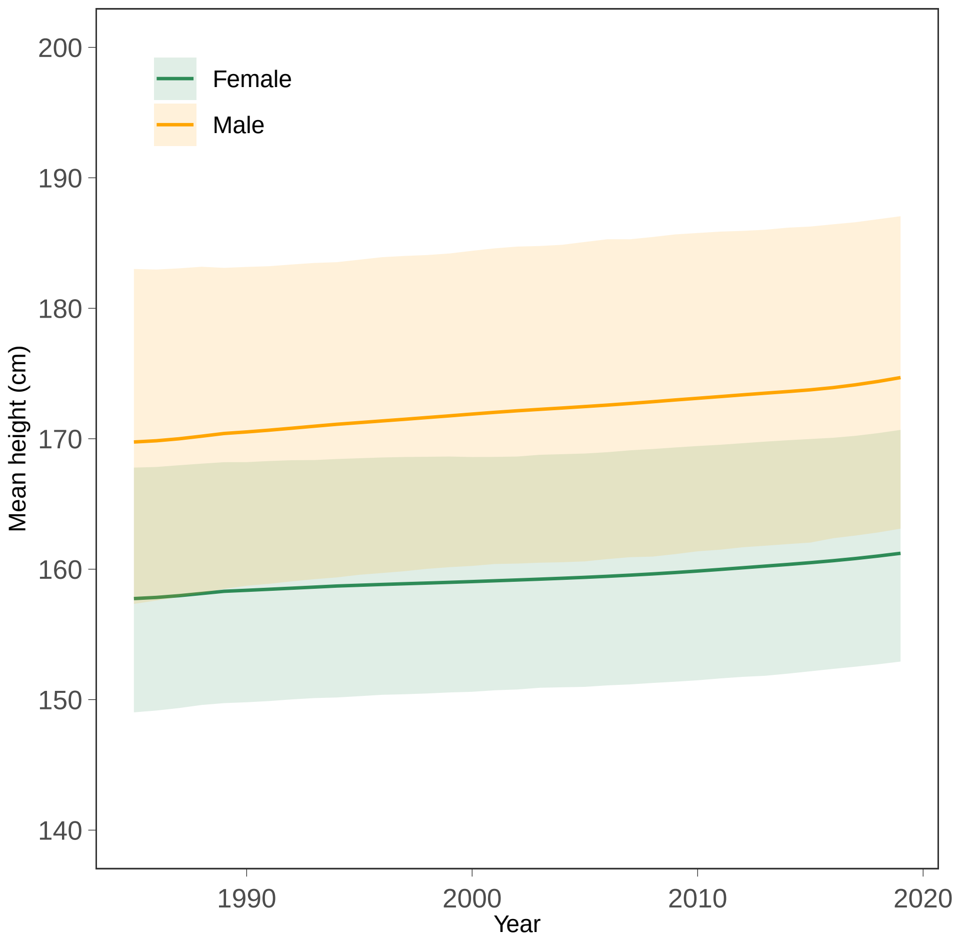

Time trends in BMI of 19 year olds

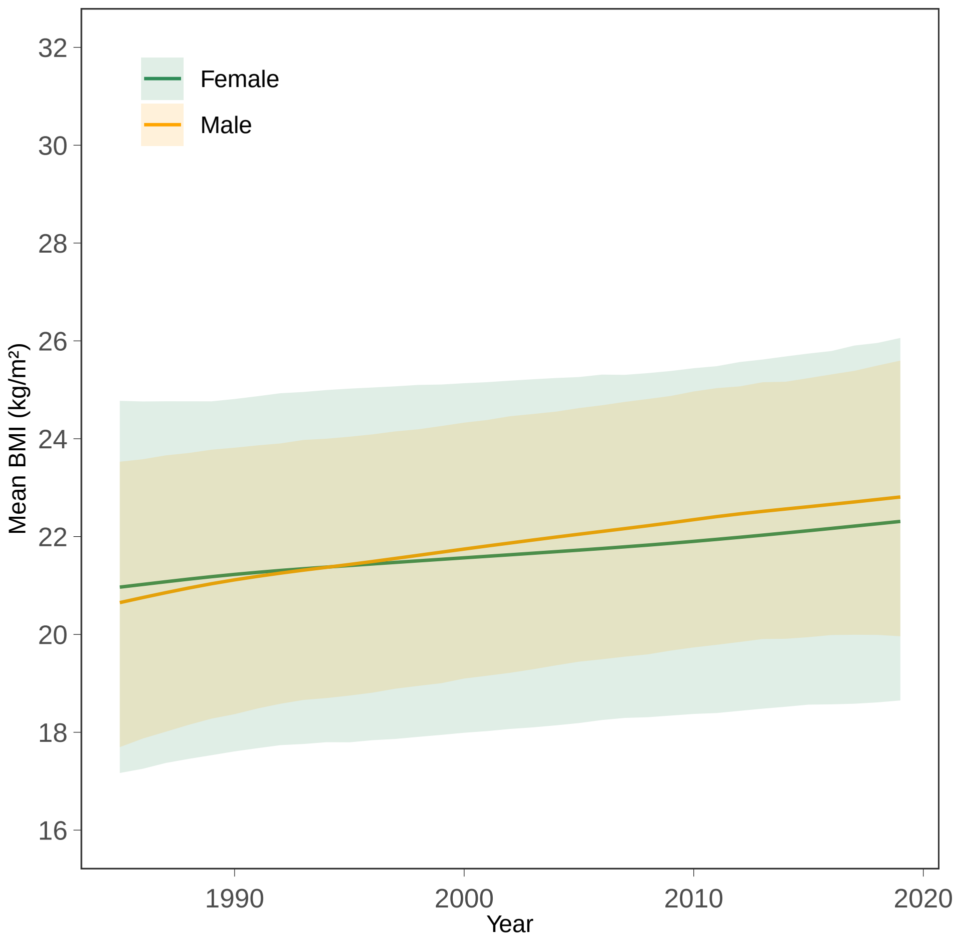

Height−for−age trajectories (2000 birth cohort)

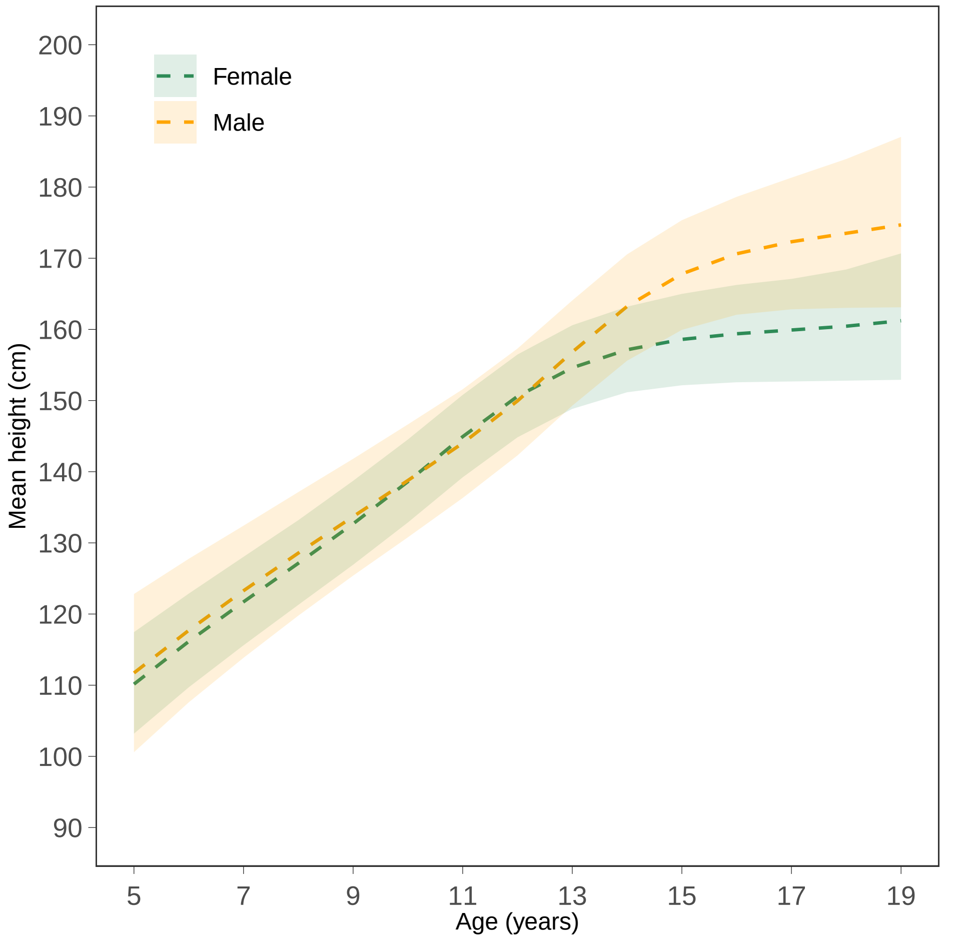

BMI−for−age trajectories (2000 birth cohort)

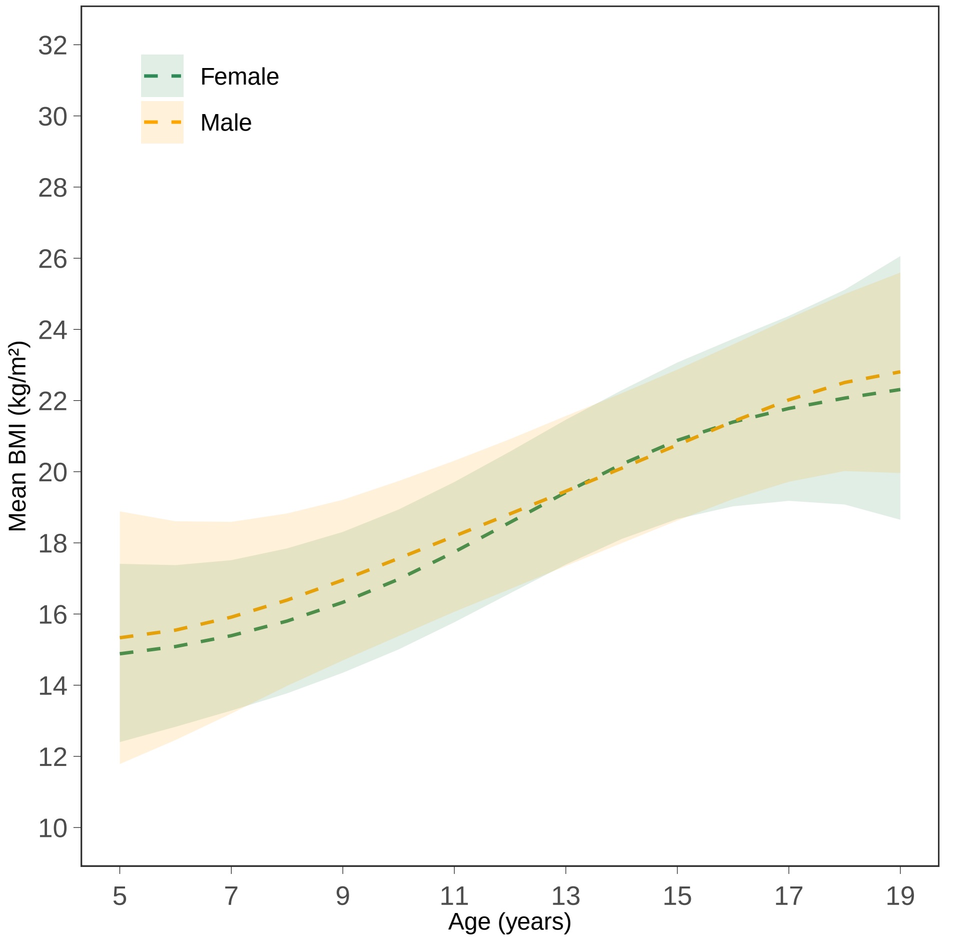

North Macedonia

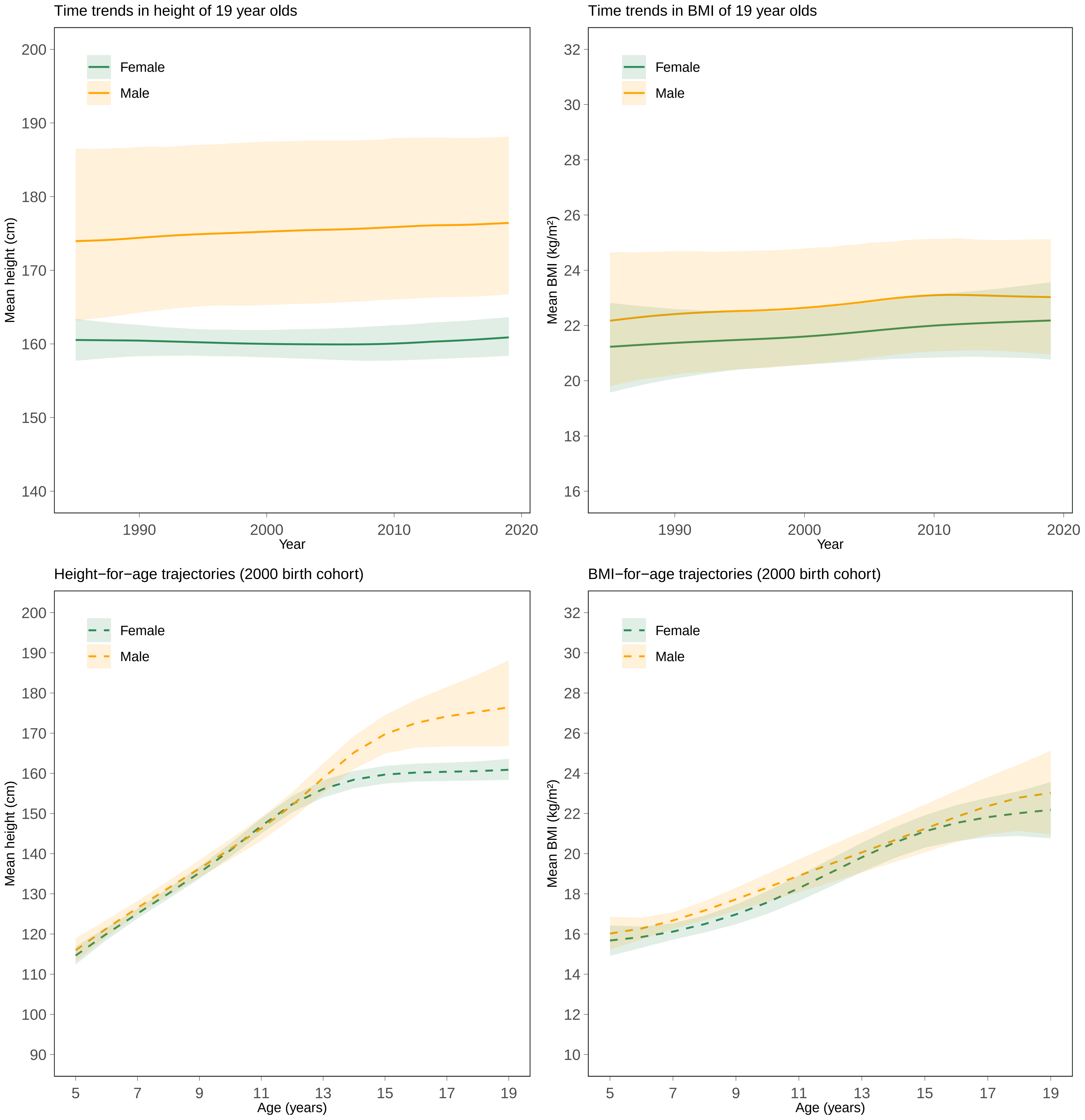

Norway

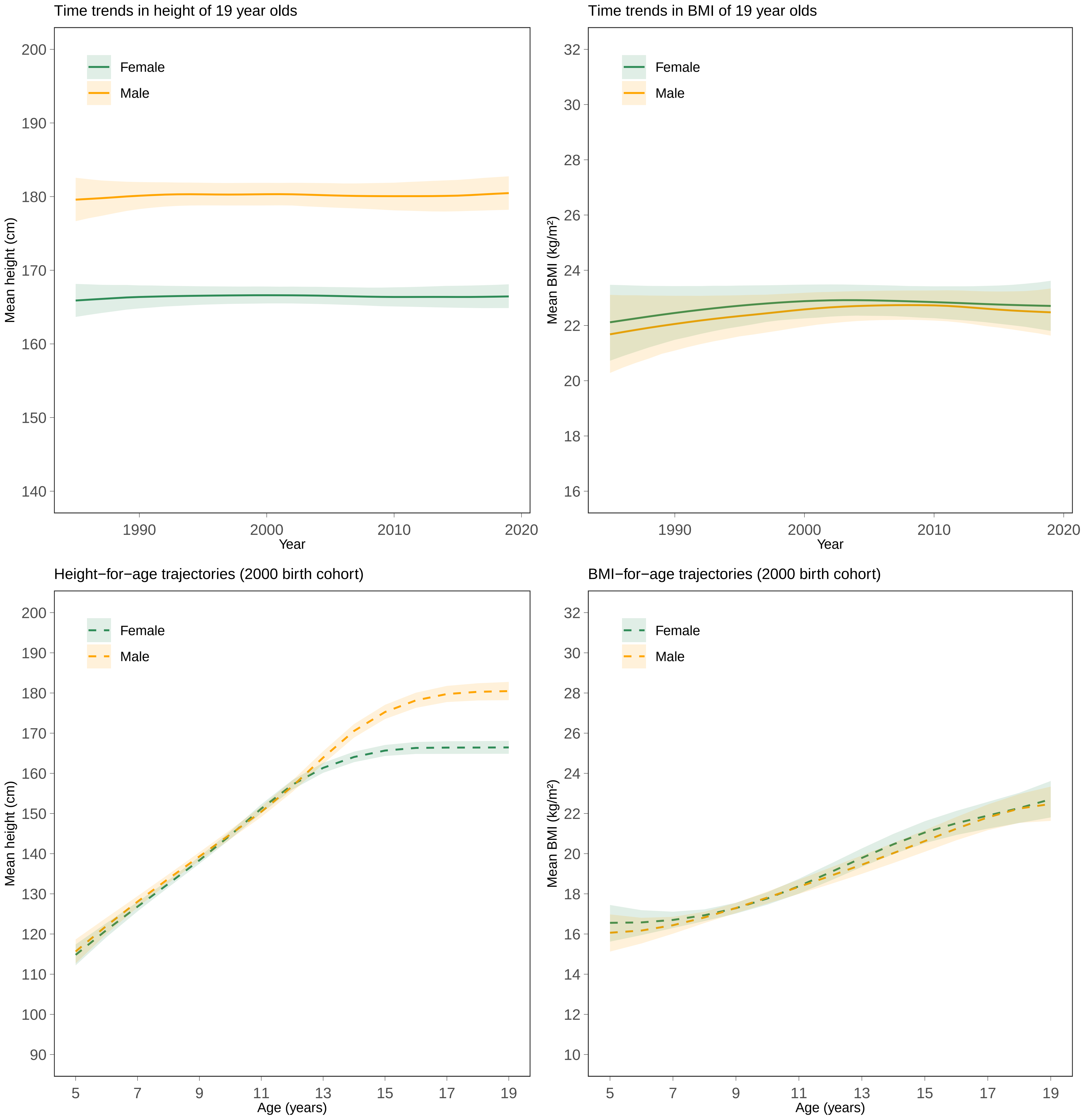
Time trends in height of 19 year olds
Female
Male
Mean height (cm)
Year
Time trends in BMI of 19 year olds
Female
Male
Mean BMI (kg/m²)
Year
Height−for−age trajectories (2000 birth cohort)
Female
Male
Mean height (cm)
Age (years)
BMI−for−age trajectories (2000 birth cohort)
Female
Male
Mean BMI (kg/m²)
Age (years)

Oman

Time trends in height of 19 year olds

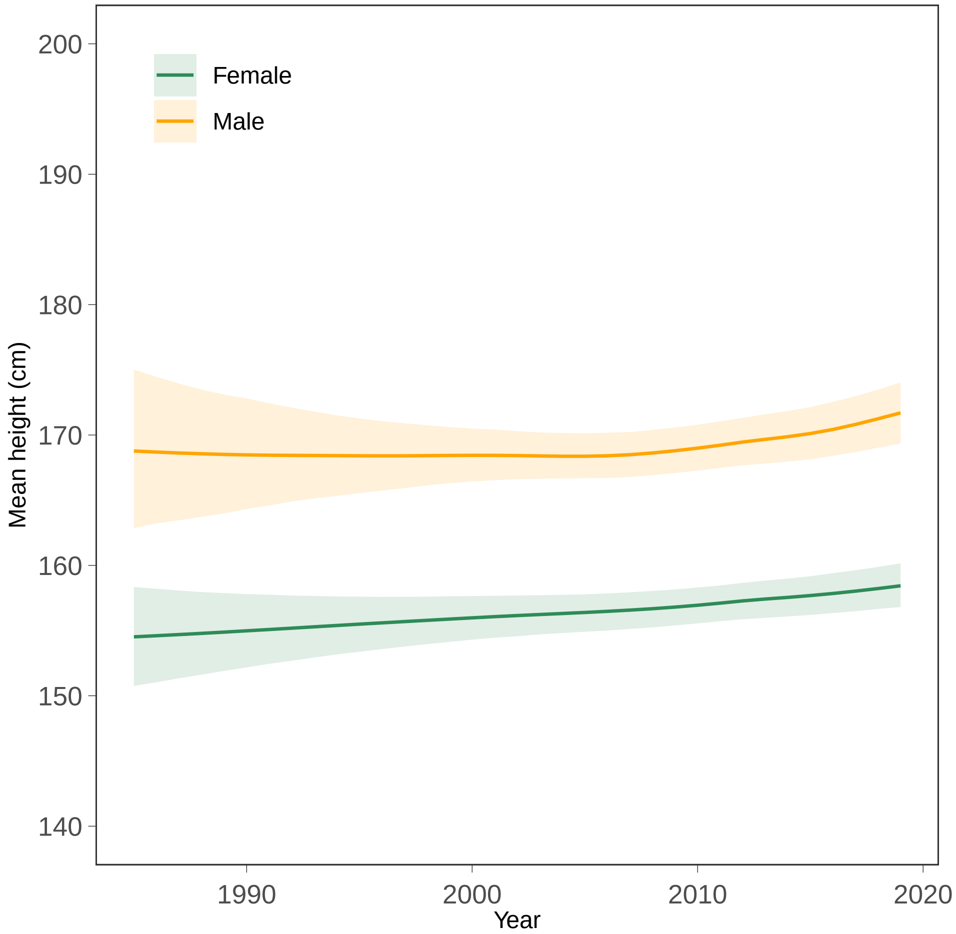

Time trends in BMI of 19 year olds

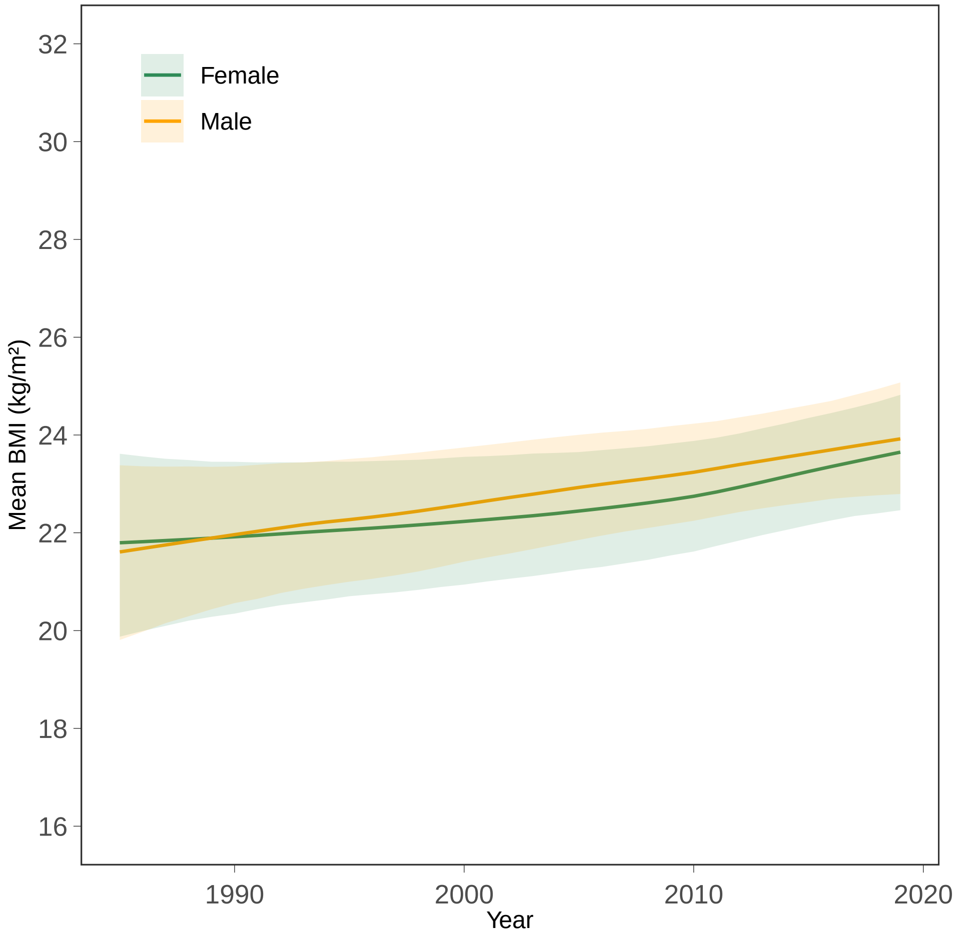

Height−for−age trajectories (2000 birth cohort)

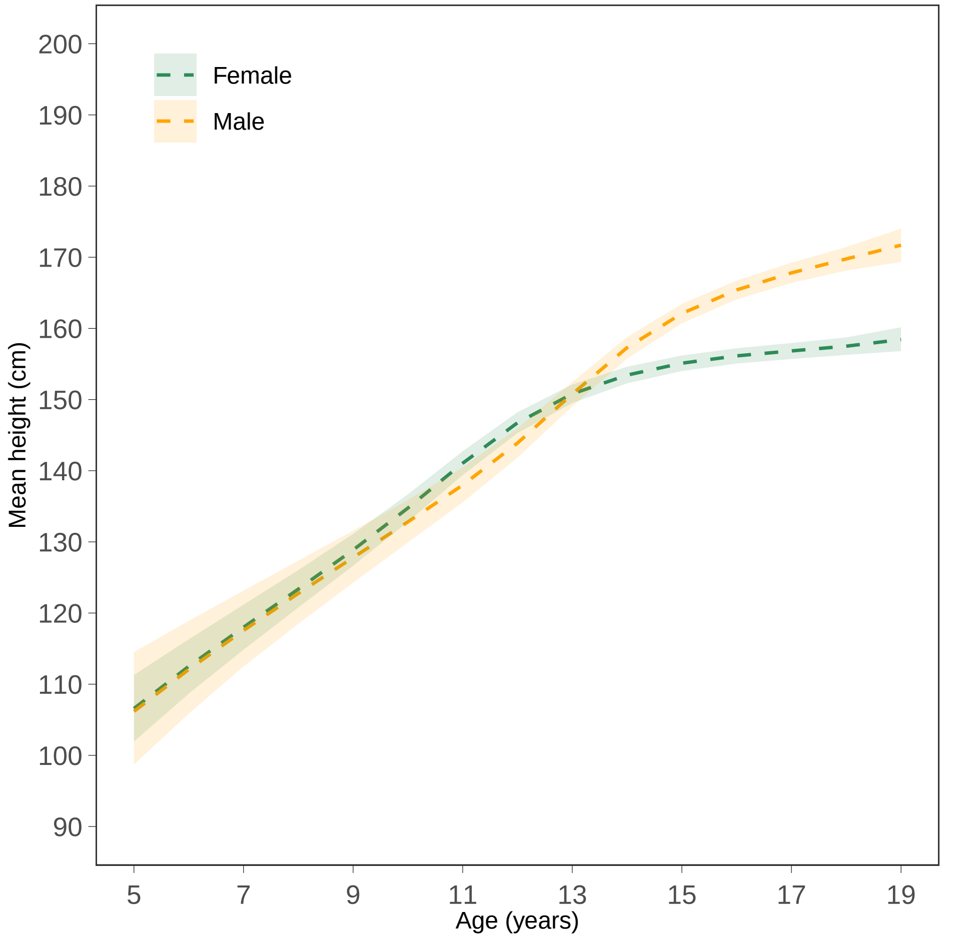

BMI−for−age trajectories (2000 birth cohort)

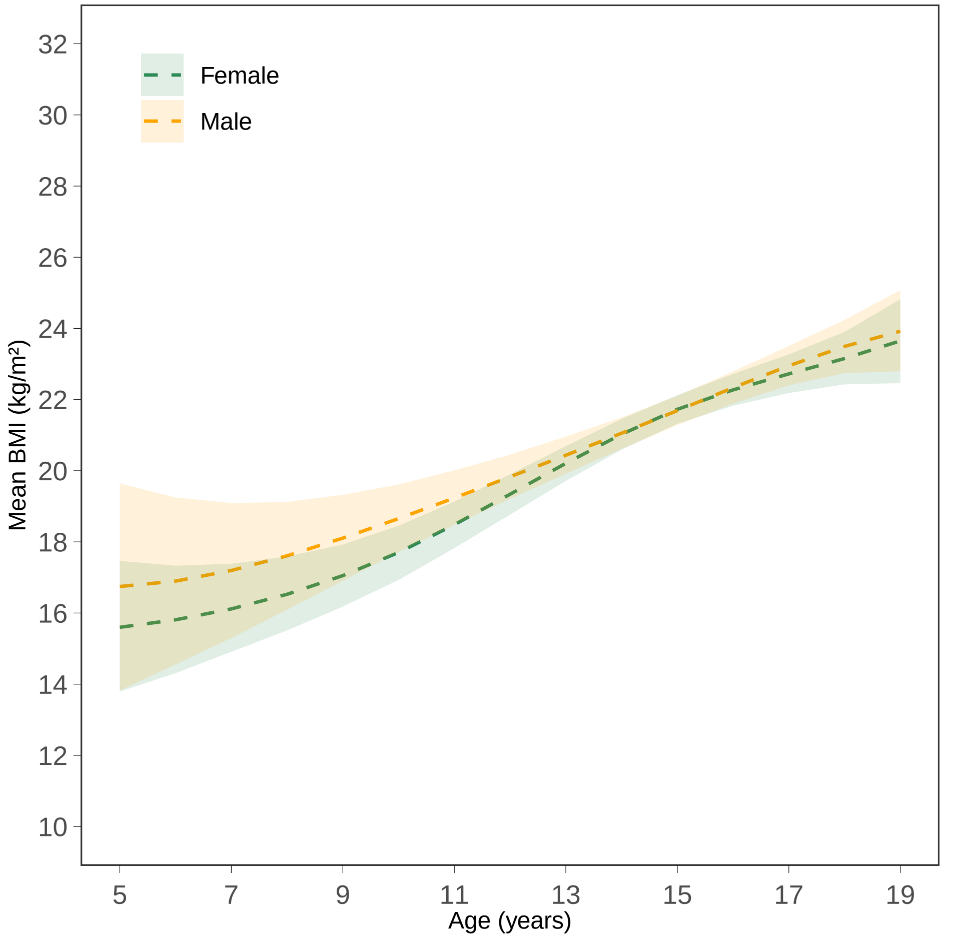

Pakistan

Time trends in height of 19 year olds

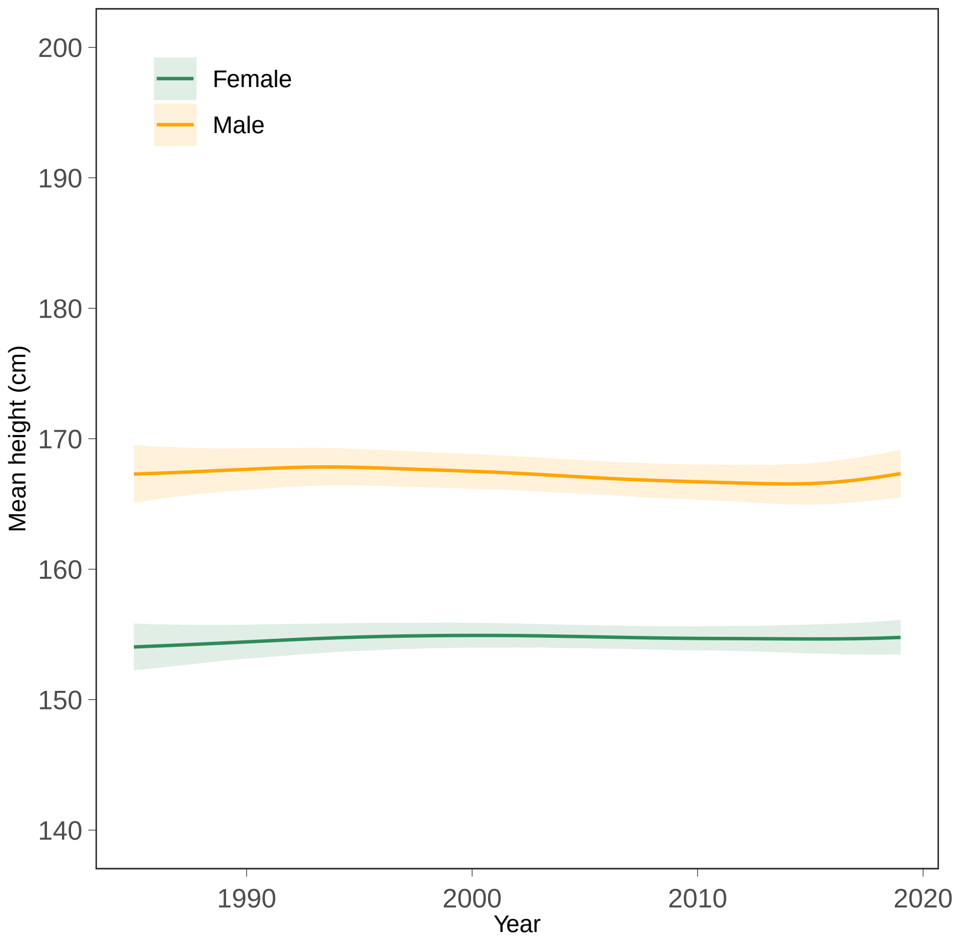

Time trends in BMI of 19 year olds

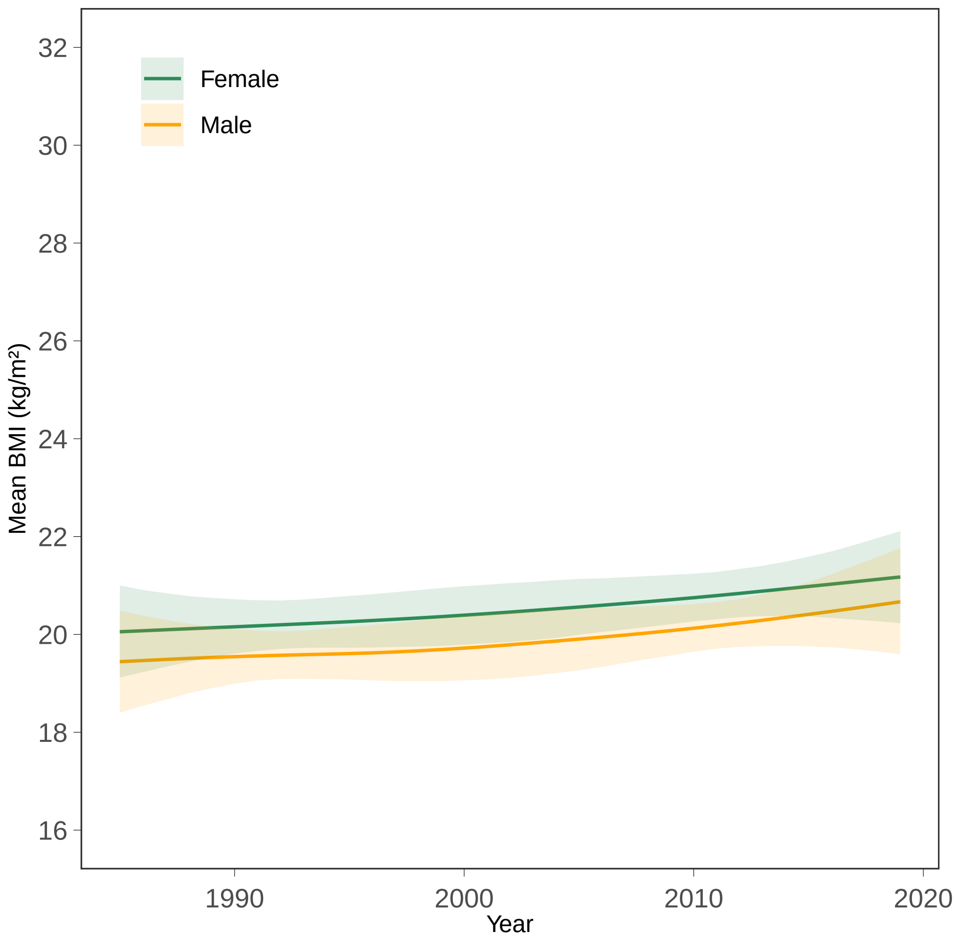

Height−for−age trajectories (2000 birth cohort)

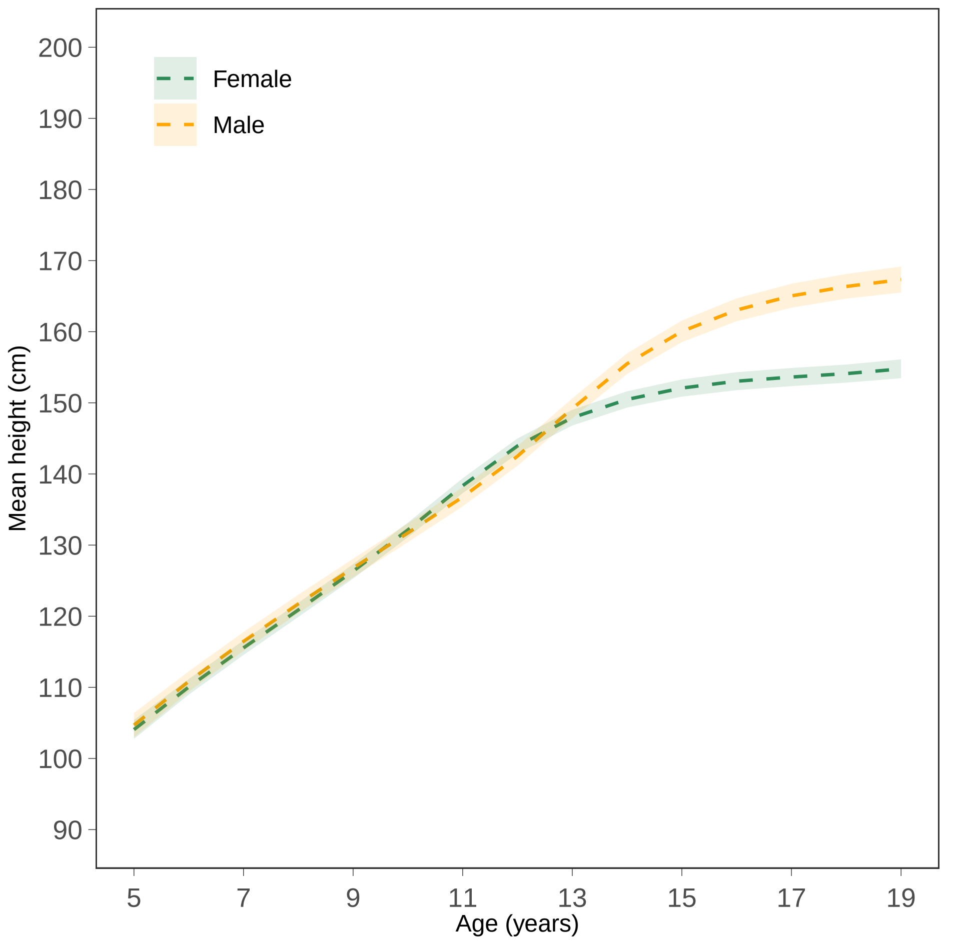

BMI−for−age trajectories (2000 birth cohort)

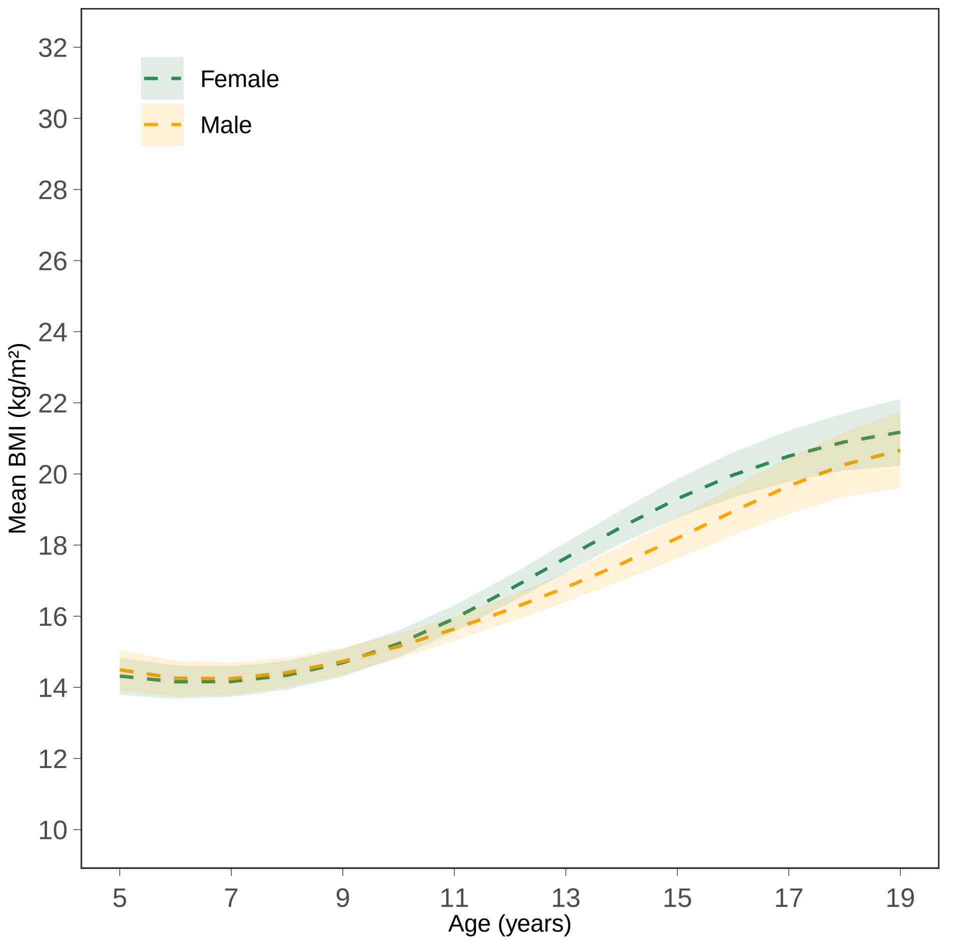

Palau

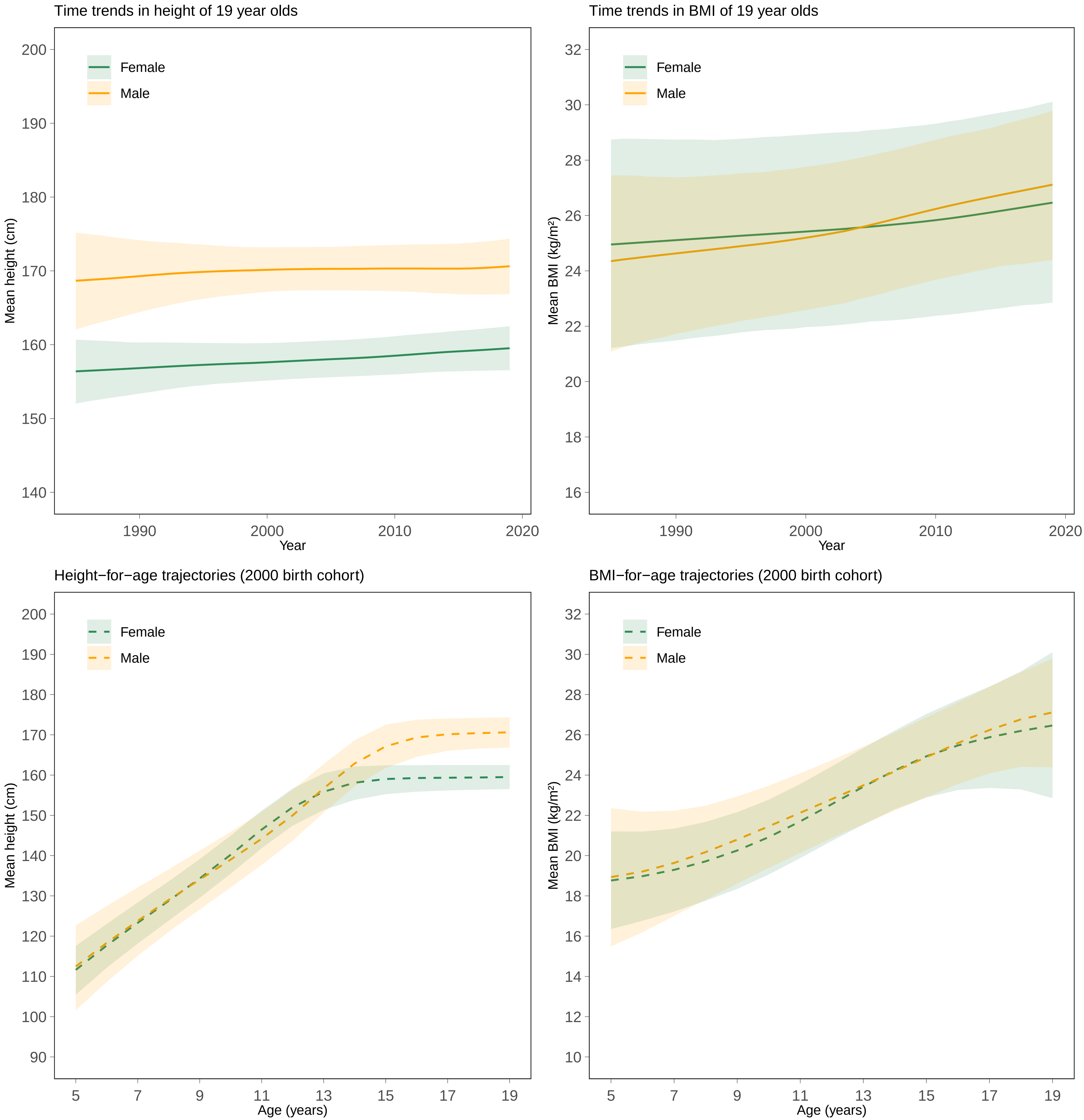
Time trends in height of 19 year olds
Female
Male
Mean height (cm)
Year
Time trends in BMI of 19 year olds
Mean BMI (kg/m²)
Height−for−age trajectories (2000 birth cohort)
Age (years)
BMI−for−age trajectories (2000 birth cohort)

Panama

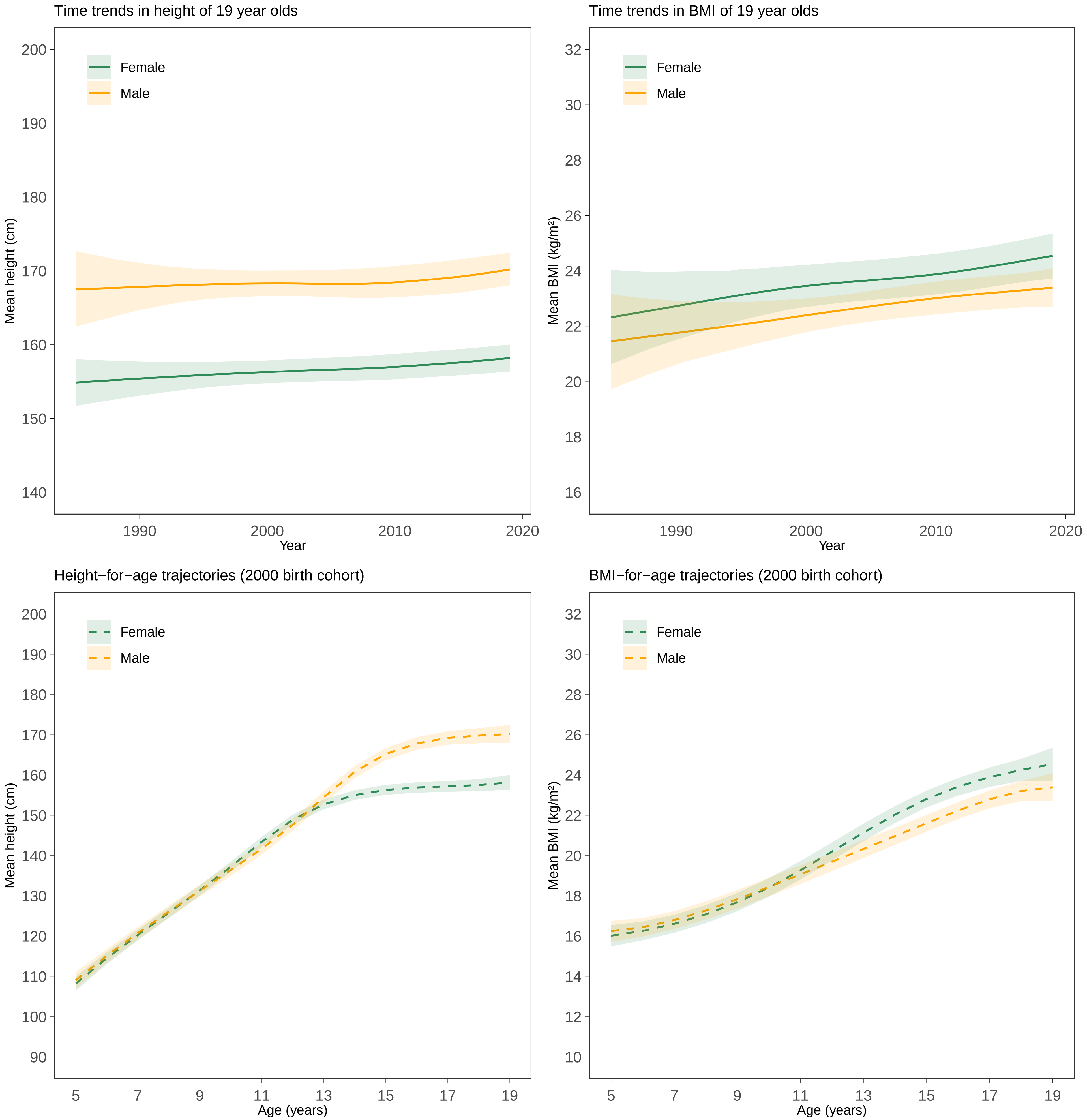

Time trends in height of 19 year olds
Female
Male
Mean height (cm)
Year
Time trends in BMI of 19 year olds
Mean BMI (kg/m²)
Height−for−age trajectories (2000 birth cohort)
Age (years)
BMI−for−age trajectories (2000 birth cohort)

Papua New Guinea

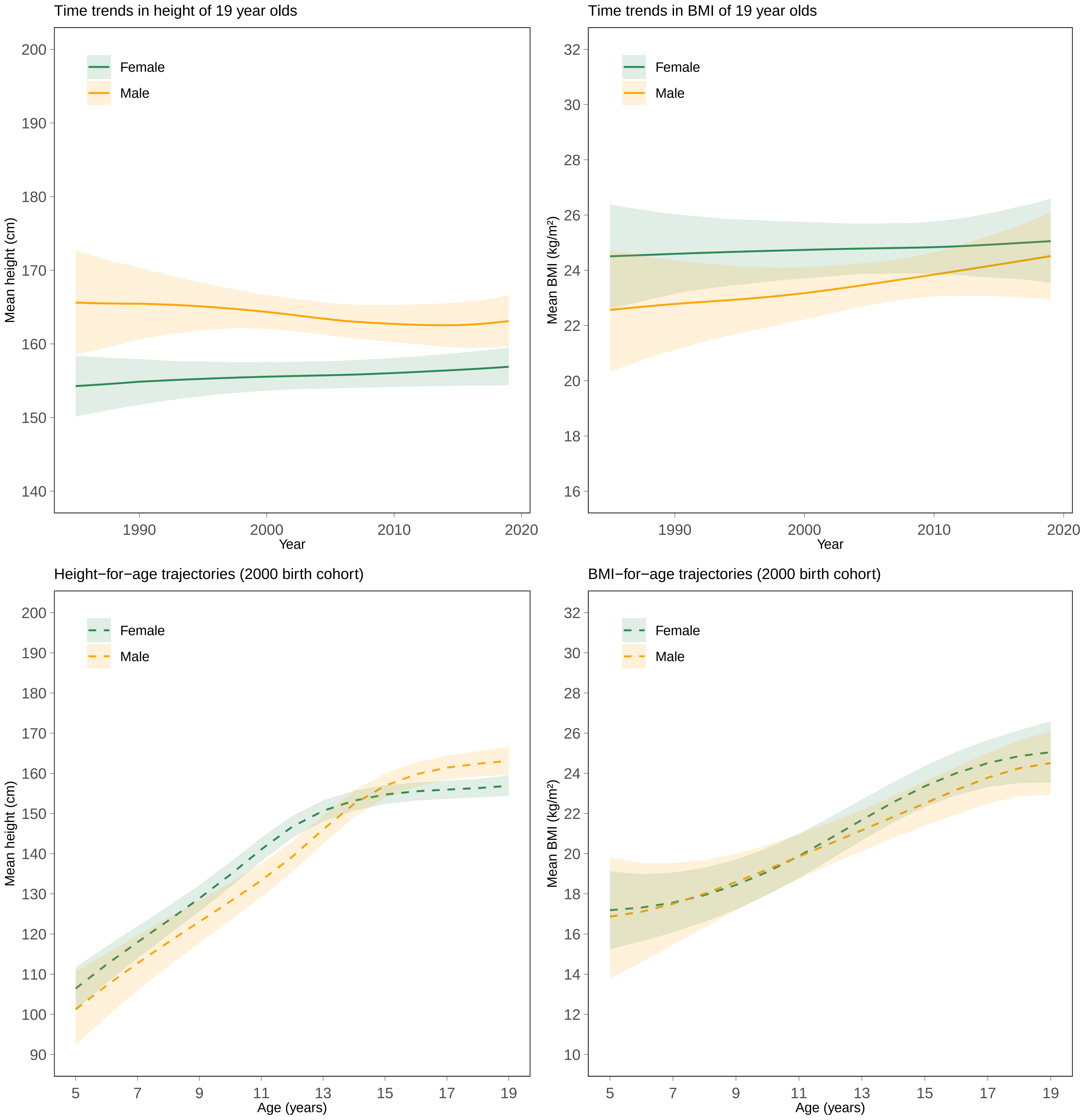

Paraguay

Time trends in height of 19 year olds

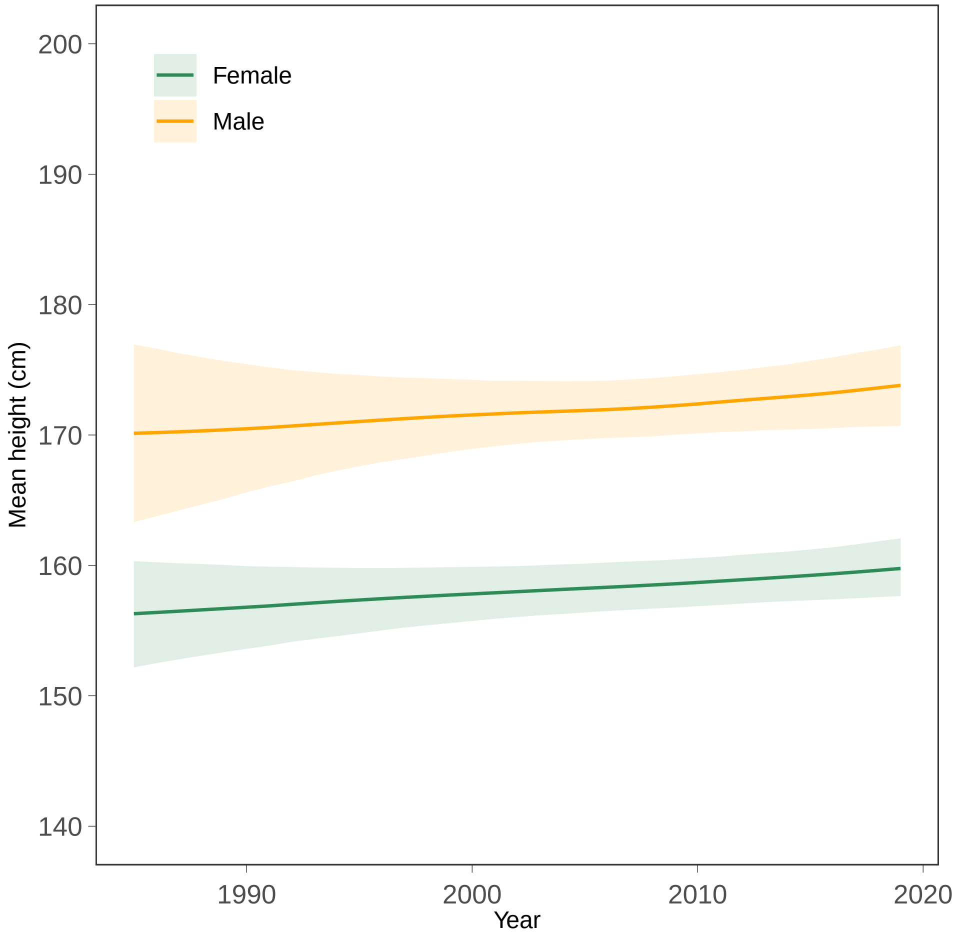

Time trends in BMI of 19 year olds

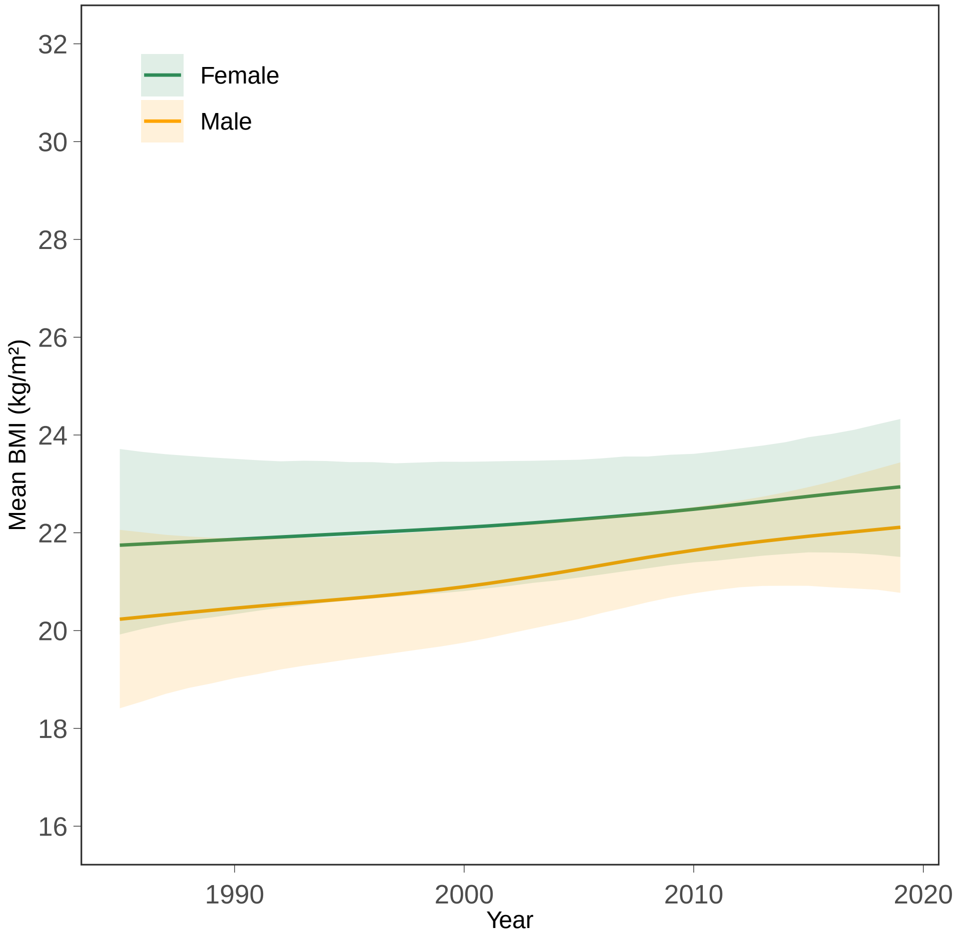

Height−for−age trajectories (2000 birth cohort)

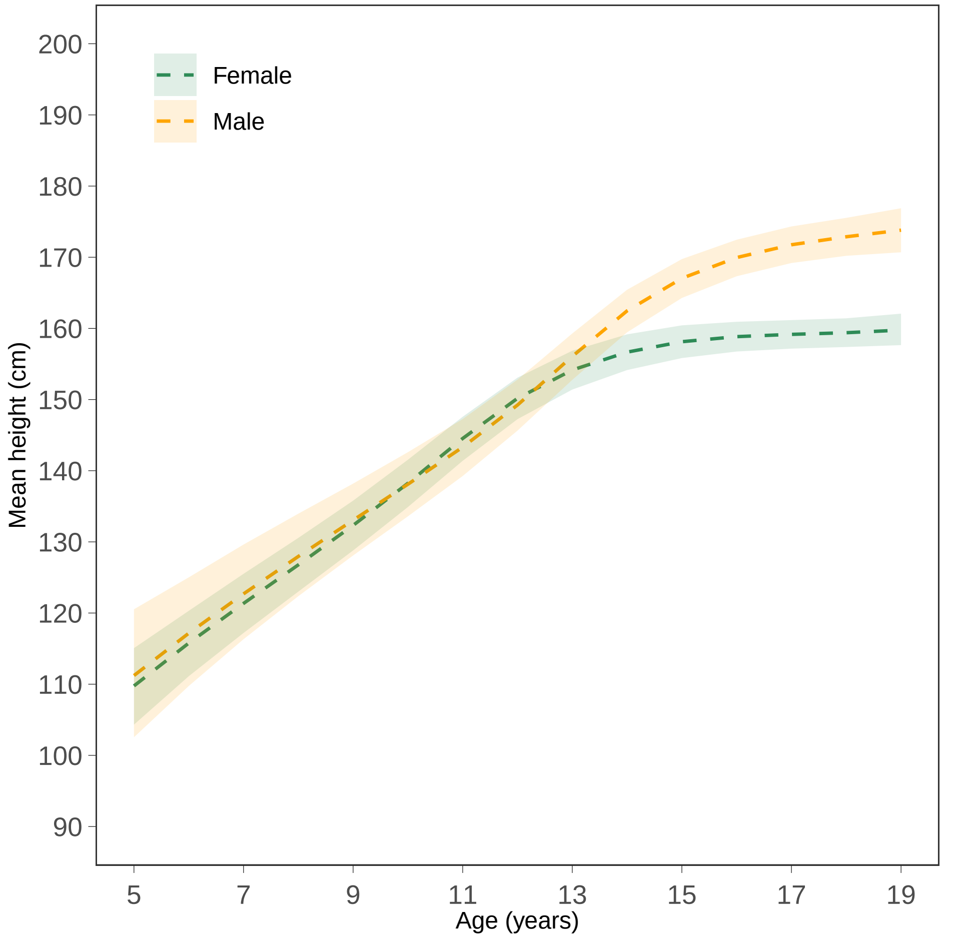

BMI−for−age trajectories (2000 birth cohort)

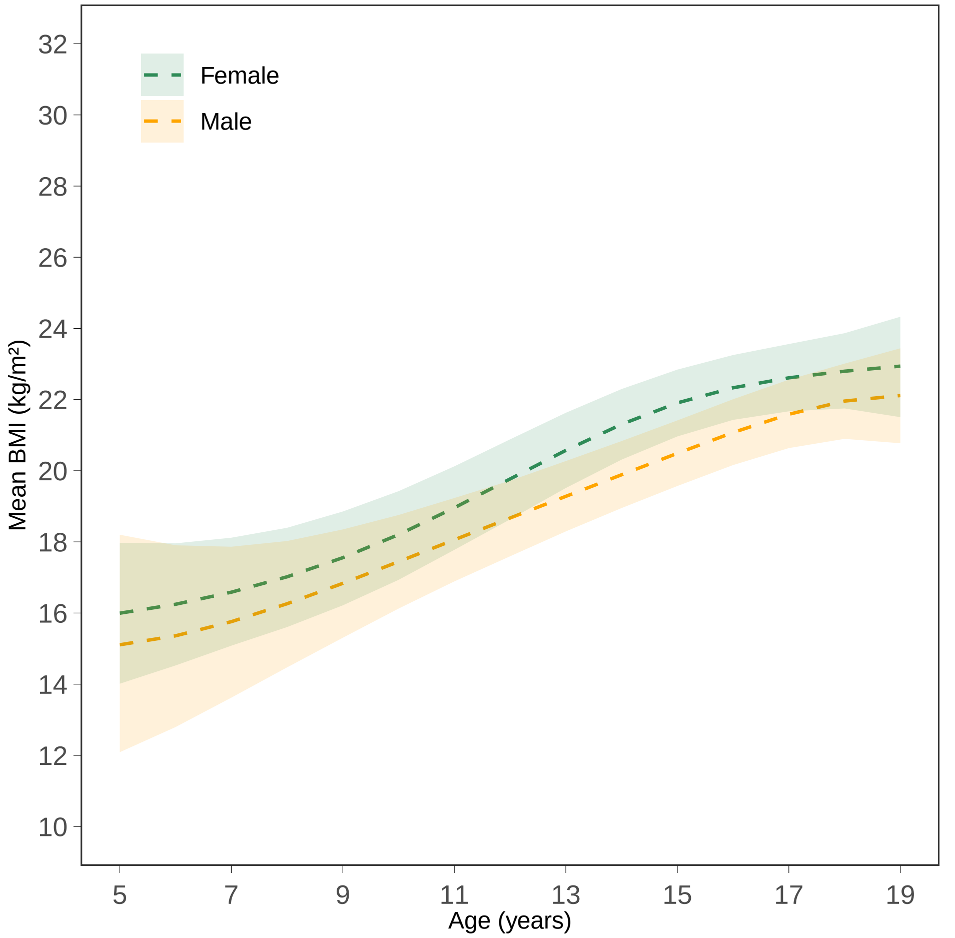

Peru

Time trends in height of 19 year olds

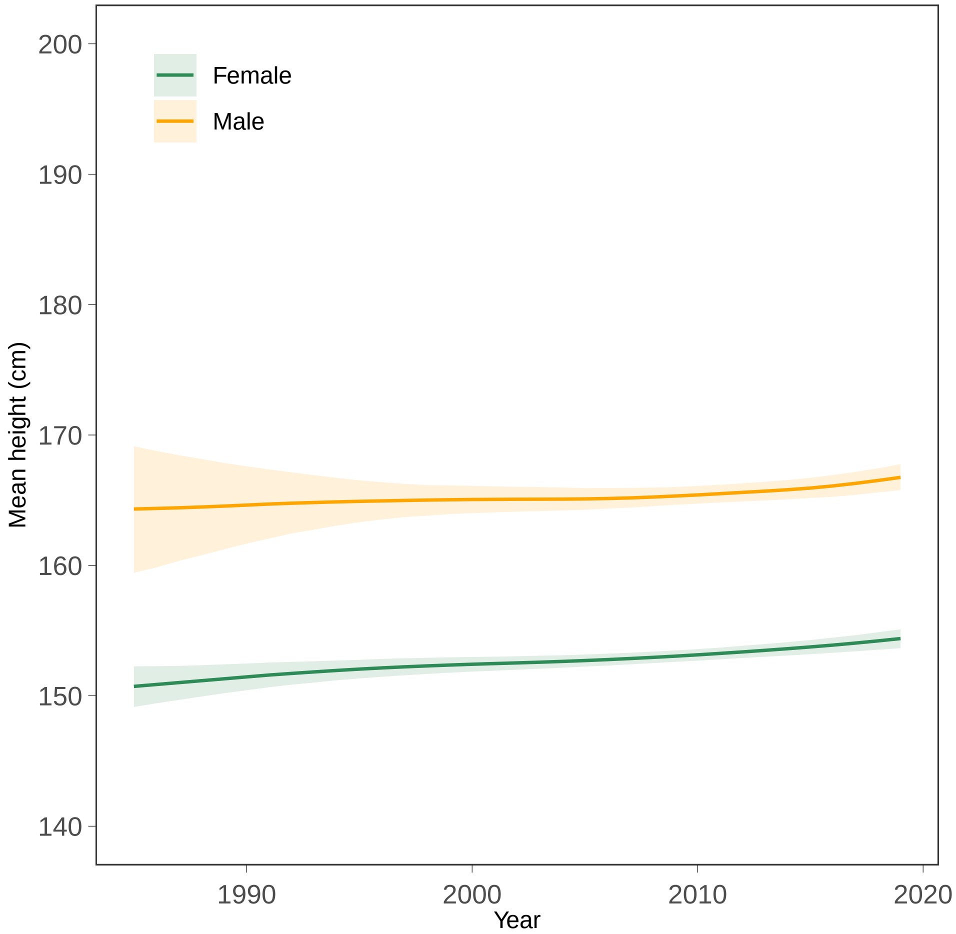

Time trends in BMI of 19 year olds

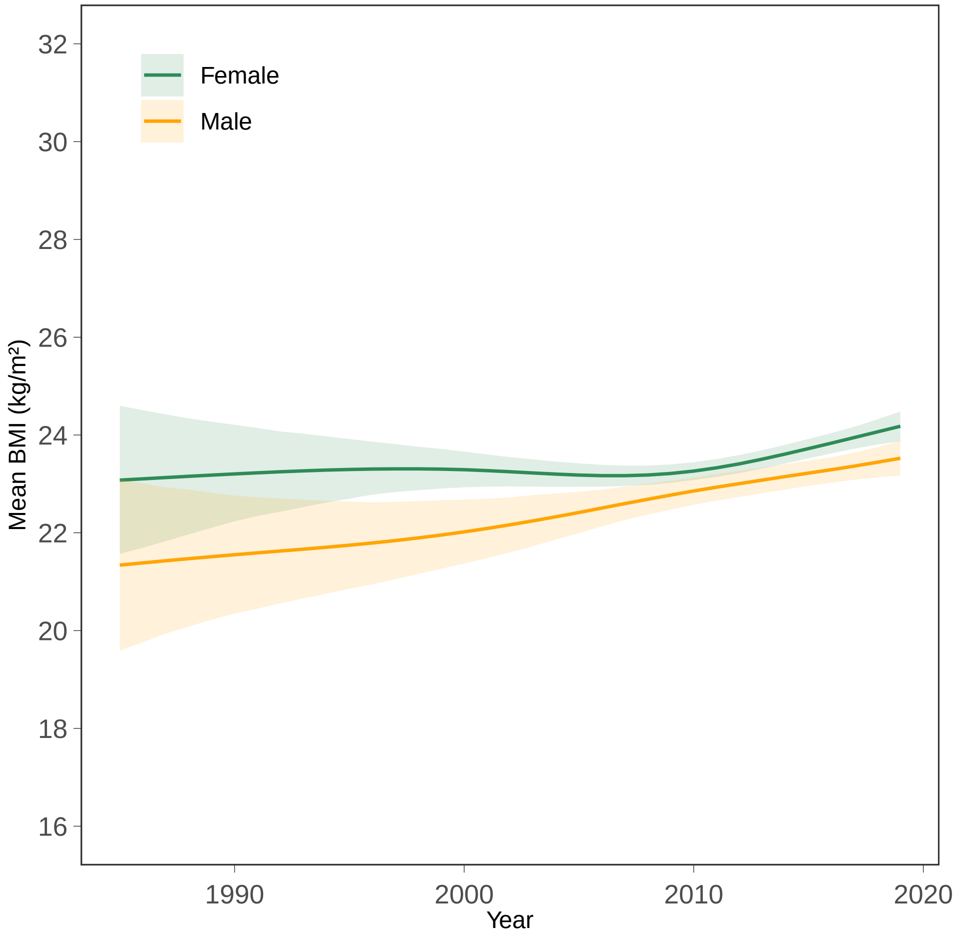

Height−for−age trajectories (2000 birth cohort)

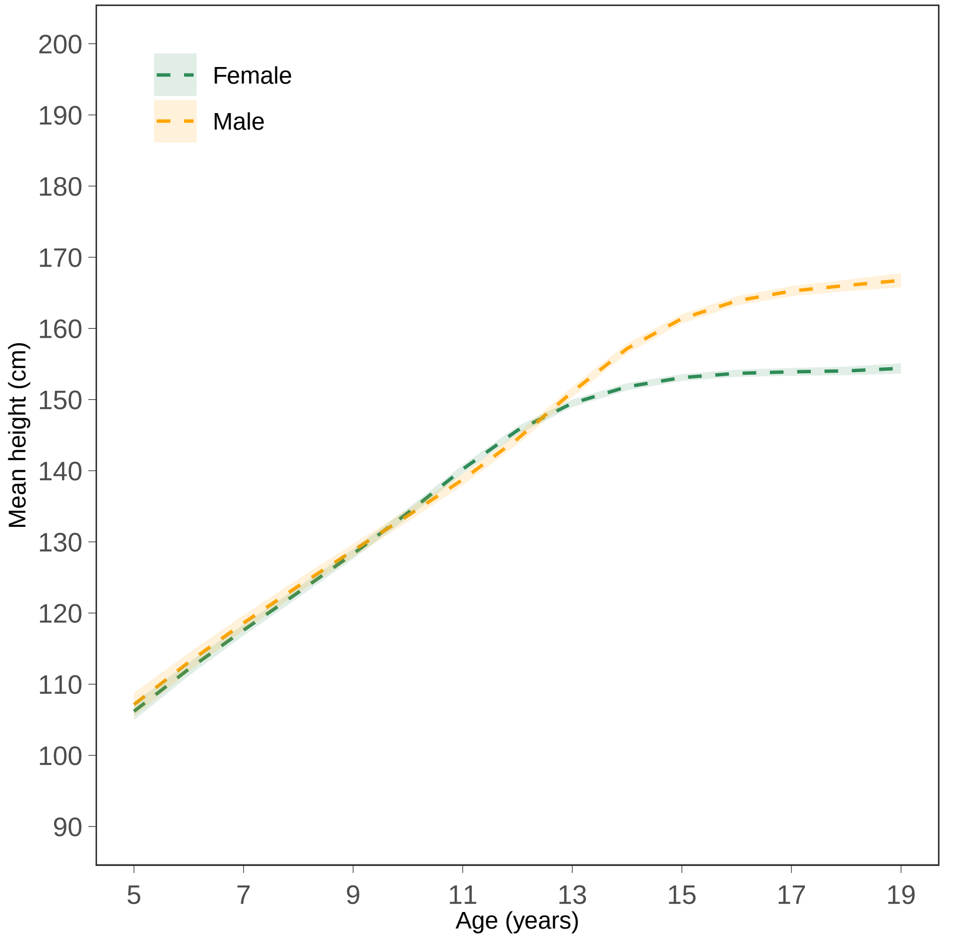

BMI−for−age trajectories (2000 birth cohort)

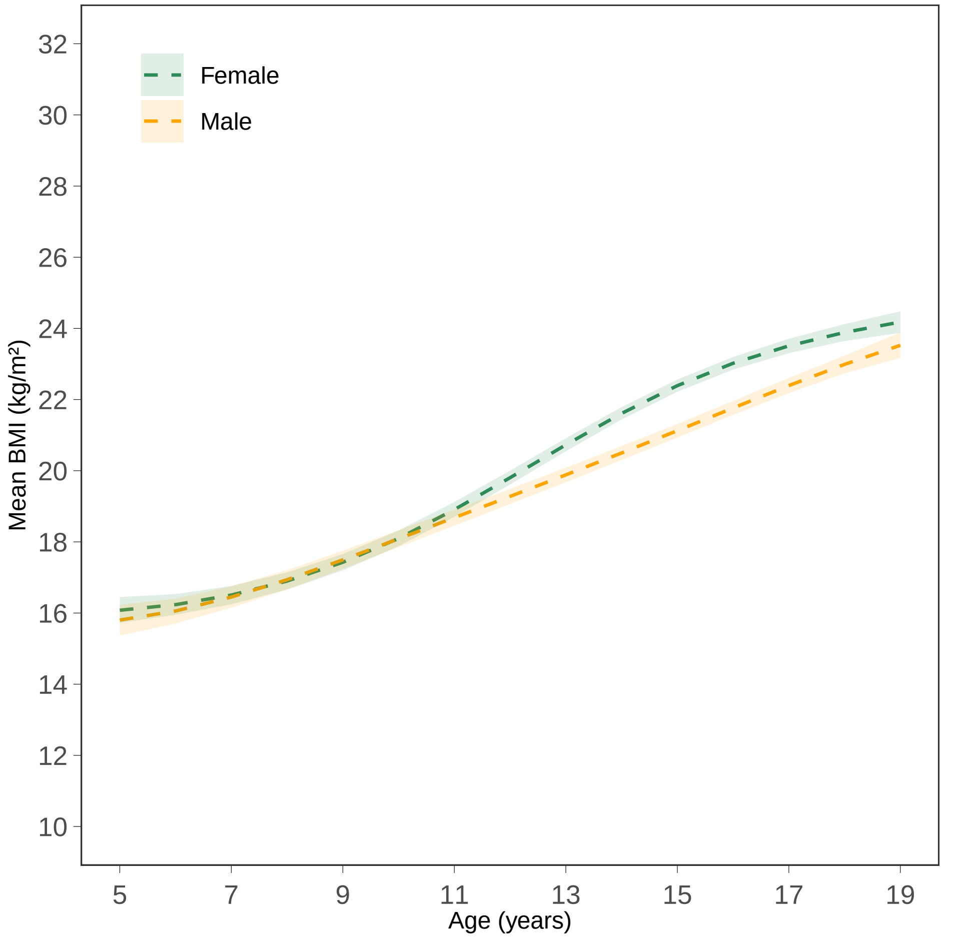

Philippines

Time trends in height of 19 year olds

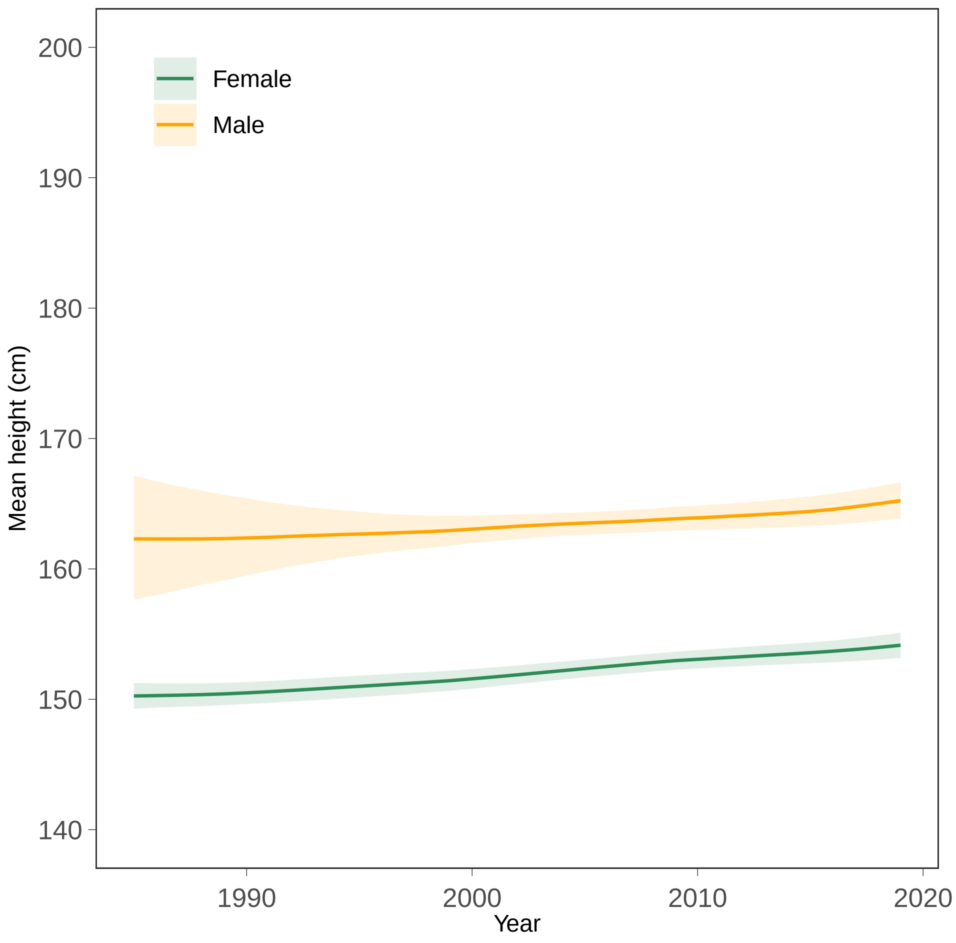

Time trends in BMI of 19 year olds

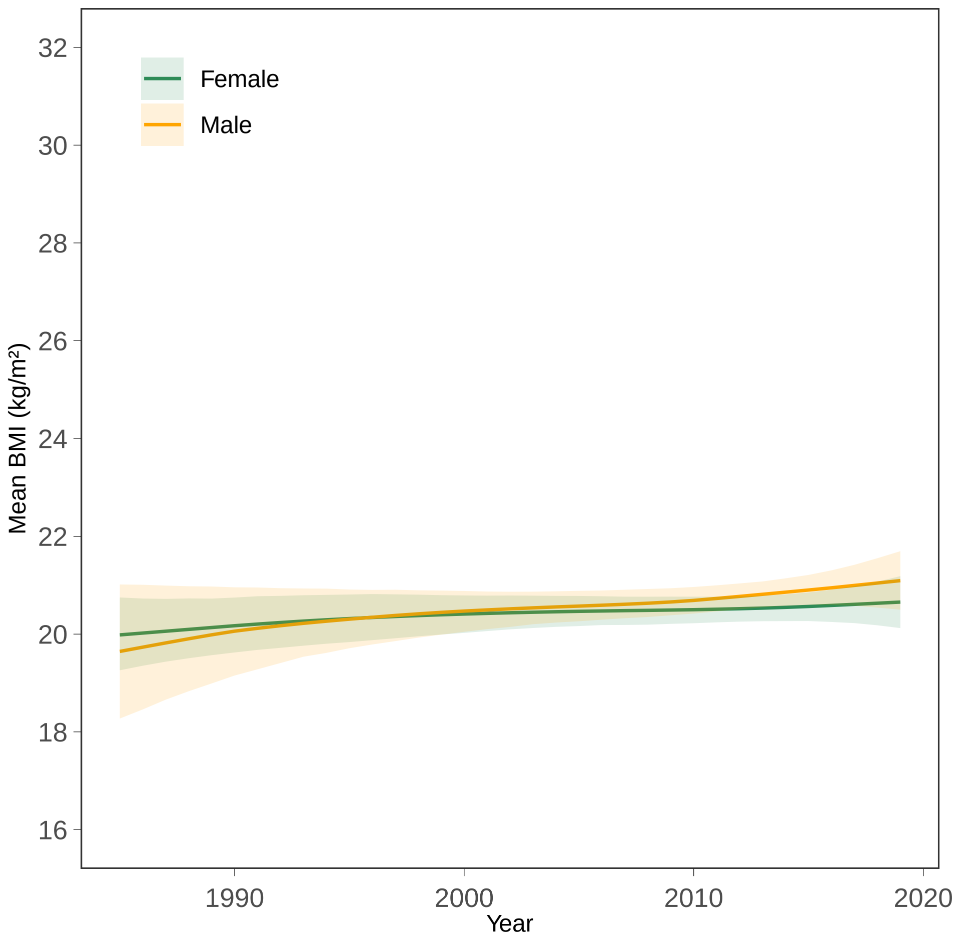

Height−for−age trajectories (2000 birth cohort)

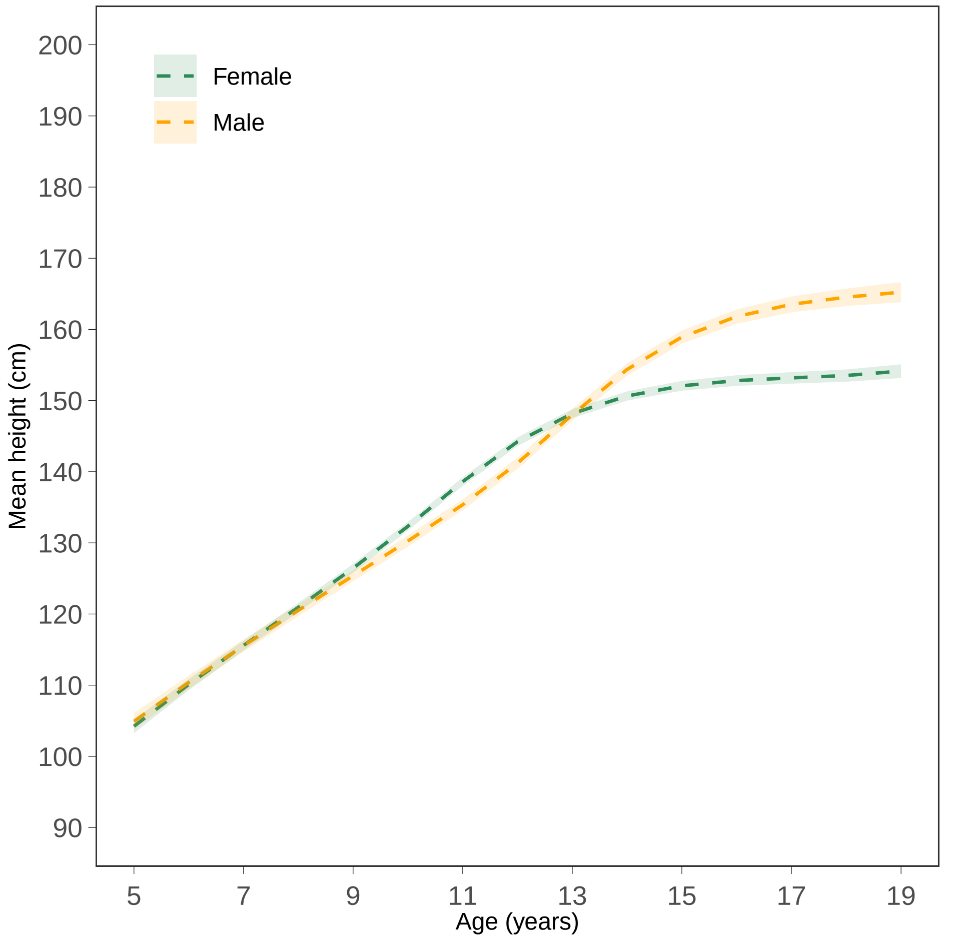

BMI−for−age trajectories (2000 birth cohort)

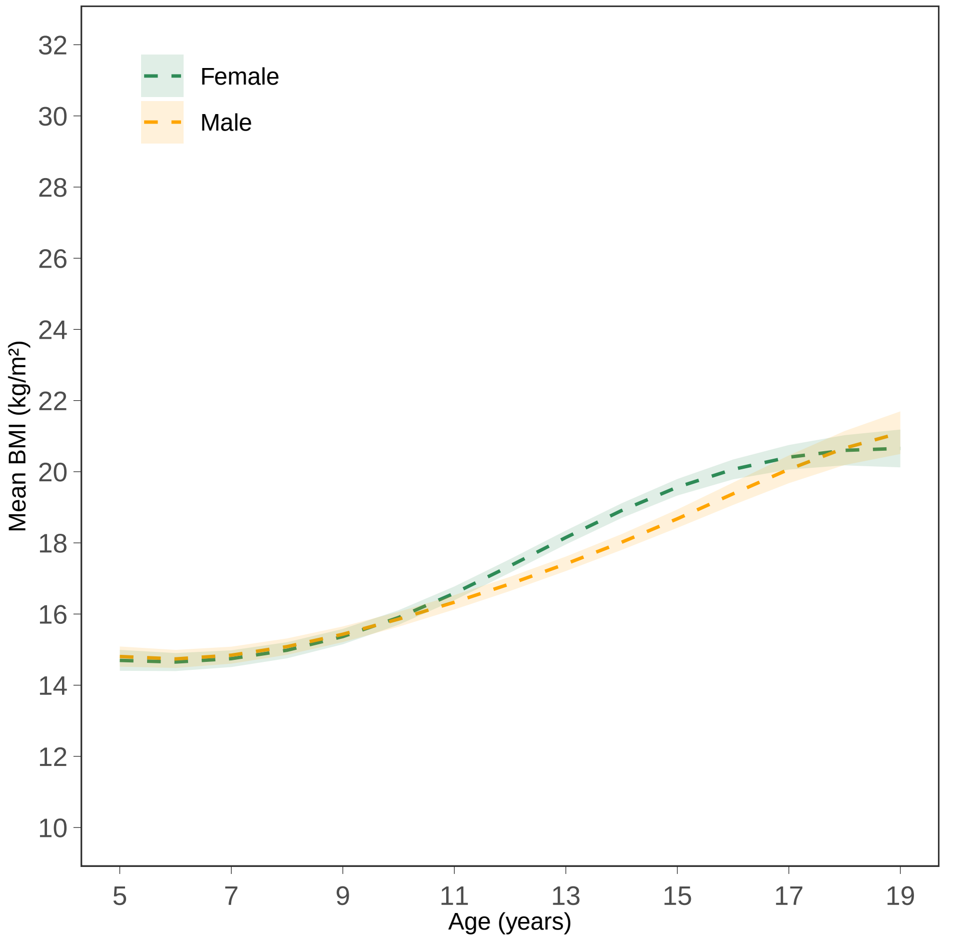

Poland

Time trends in height of 19 year olds

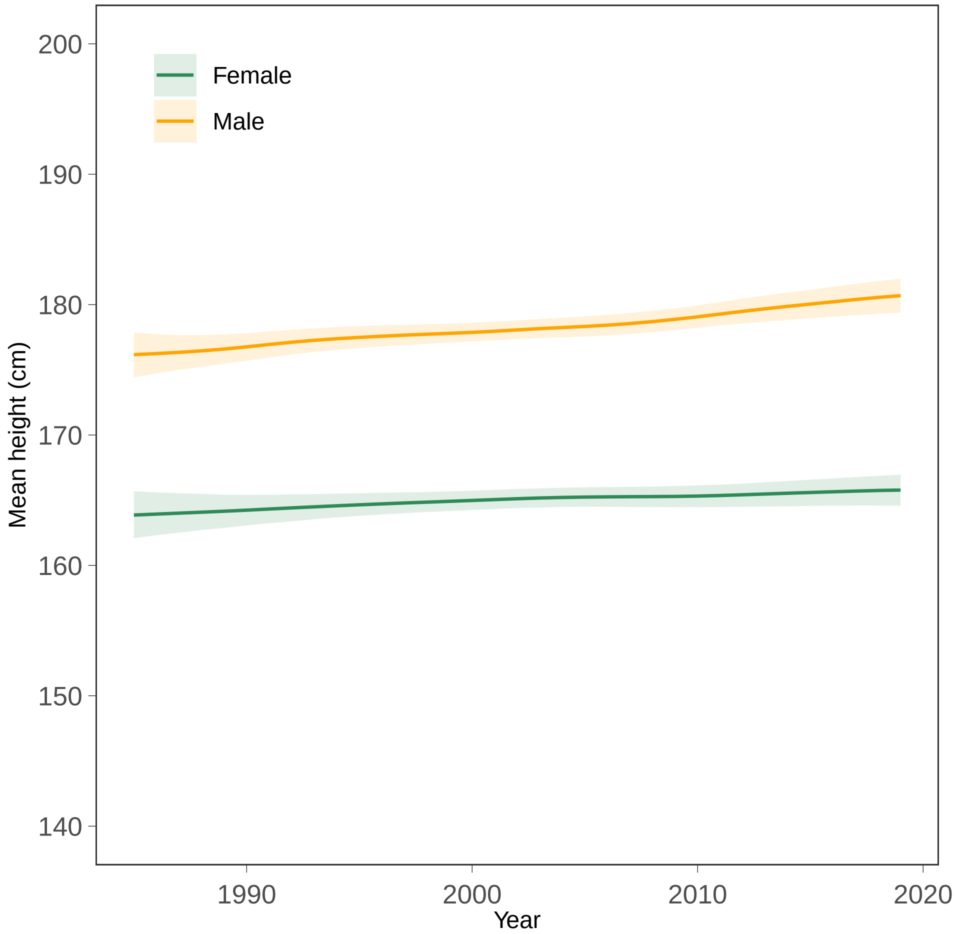

Time trends in BMI of 19 year olds

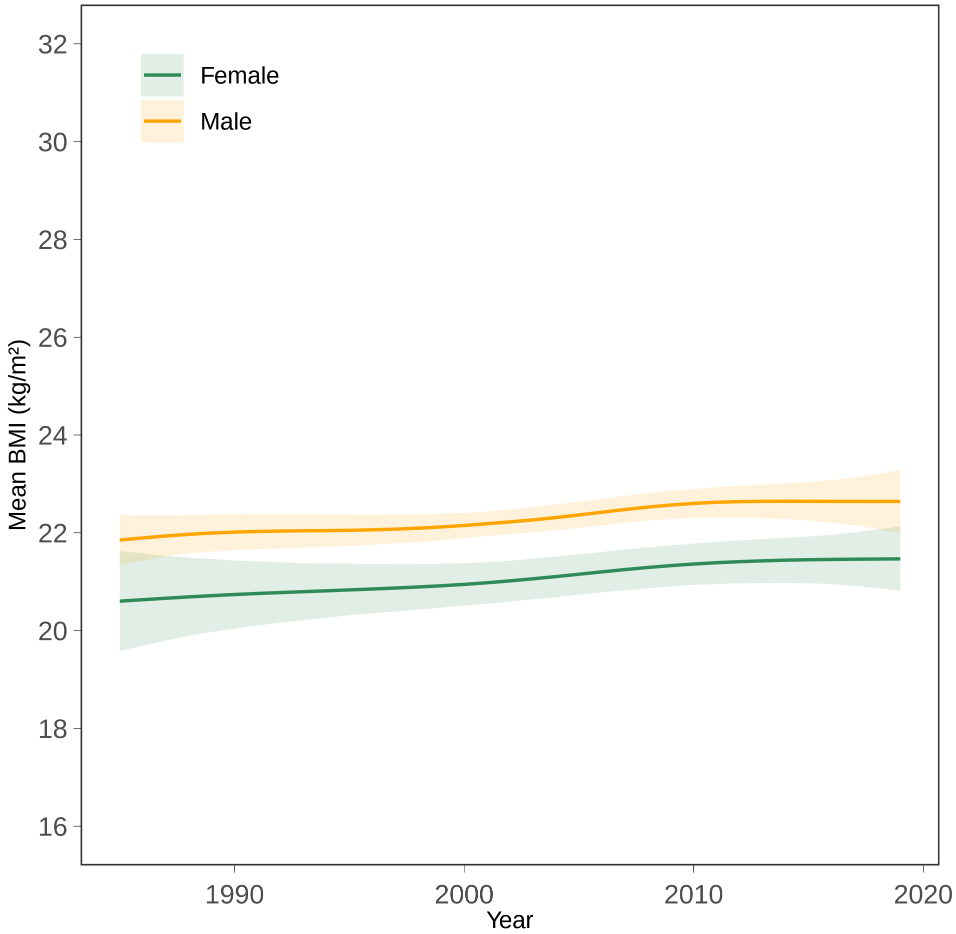

Height−for−age trajectories (2000 birth cohort)

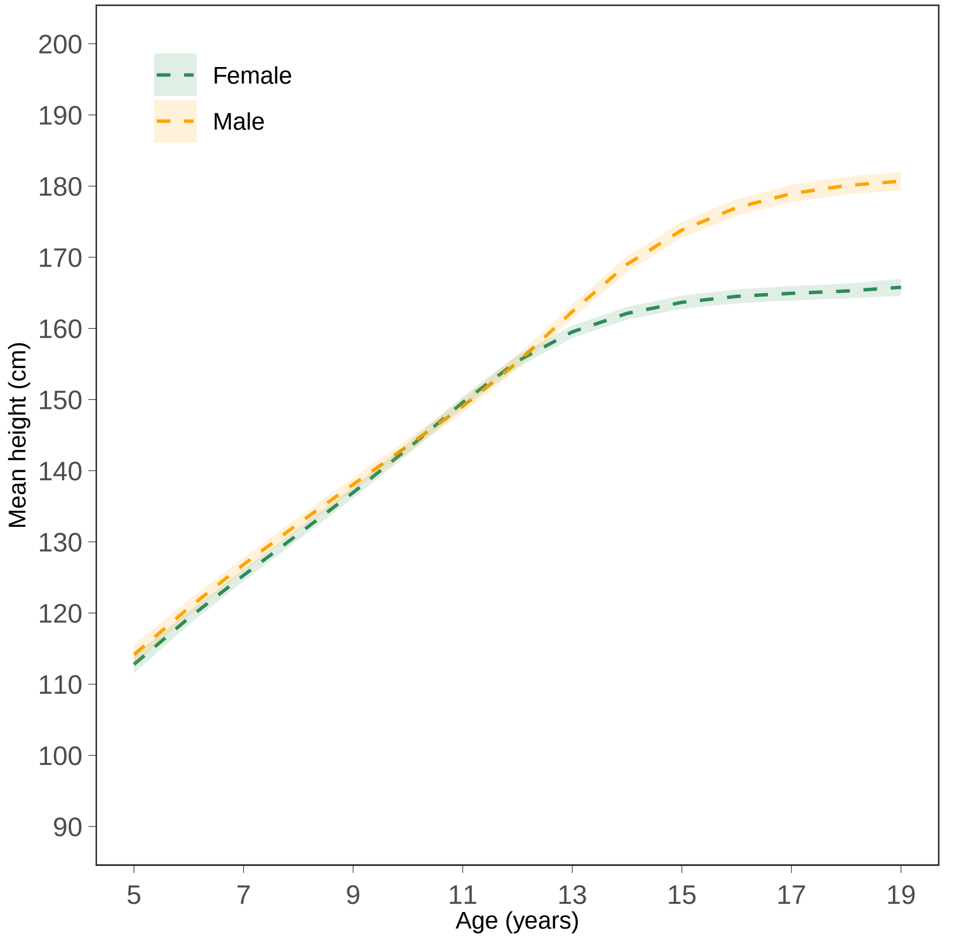

BMI−for−age trajectories (2000 birth cohort)

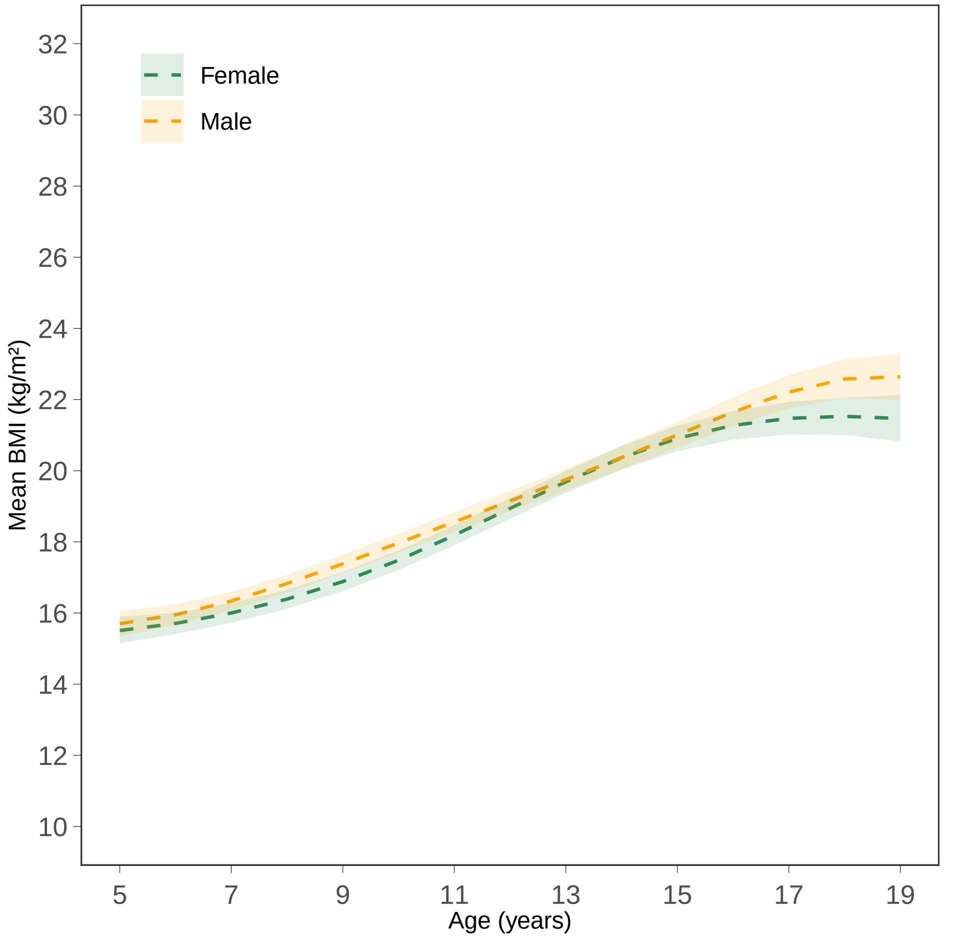

Portugal

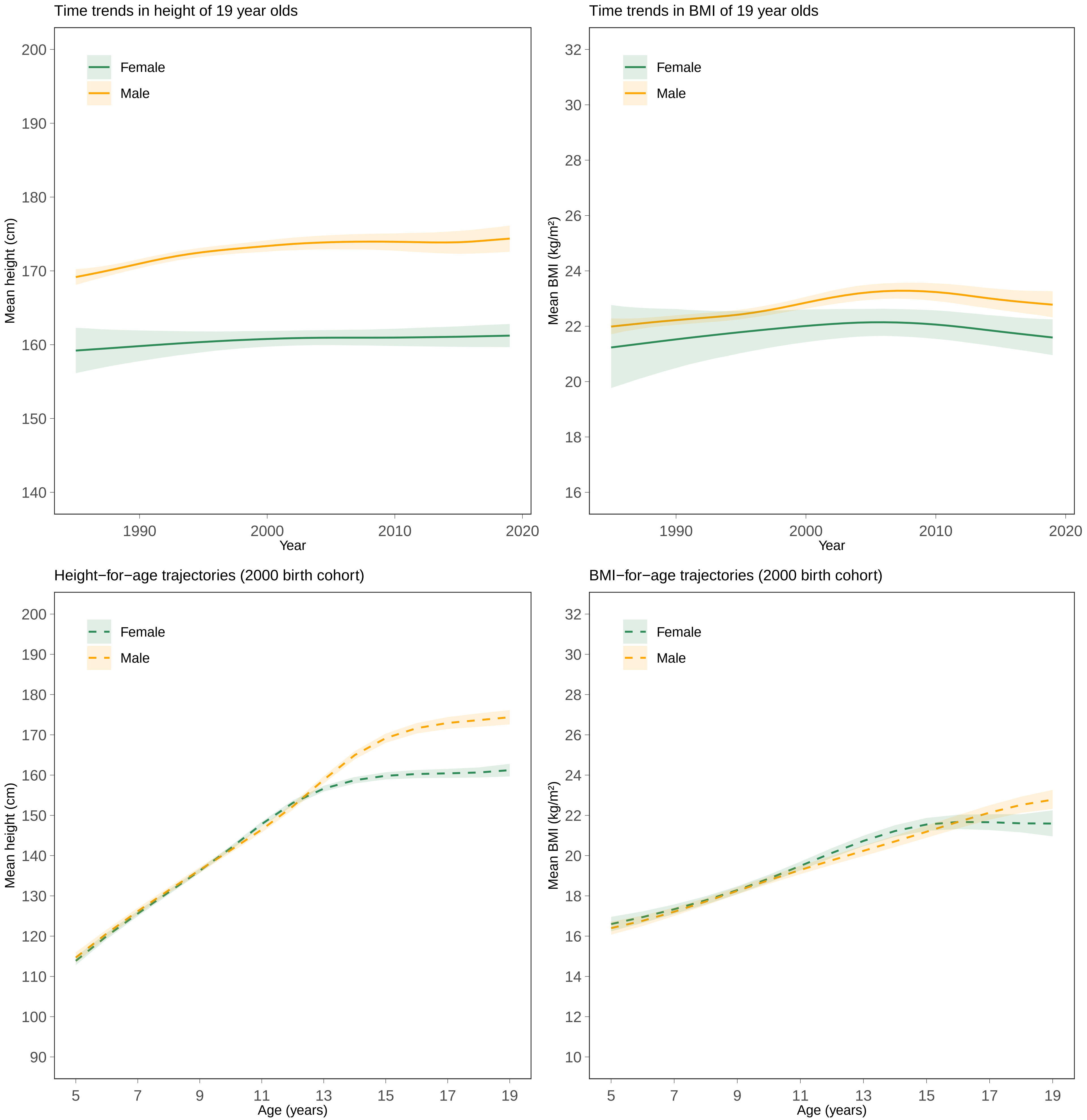
Time trends in height of 19 year olds
Female
Male
Mean height (cm)
Year
Time trends in BMI of 19 year olds
Mean BMI (kg/m²)
Height−for−age trajectories (2000 birth cohort)
Age (years)
BMI−for−age trajectories (2000 birth cohort)

Puerto Rico

Time trends in height of 19 year olds

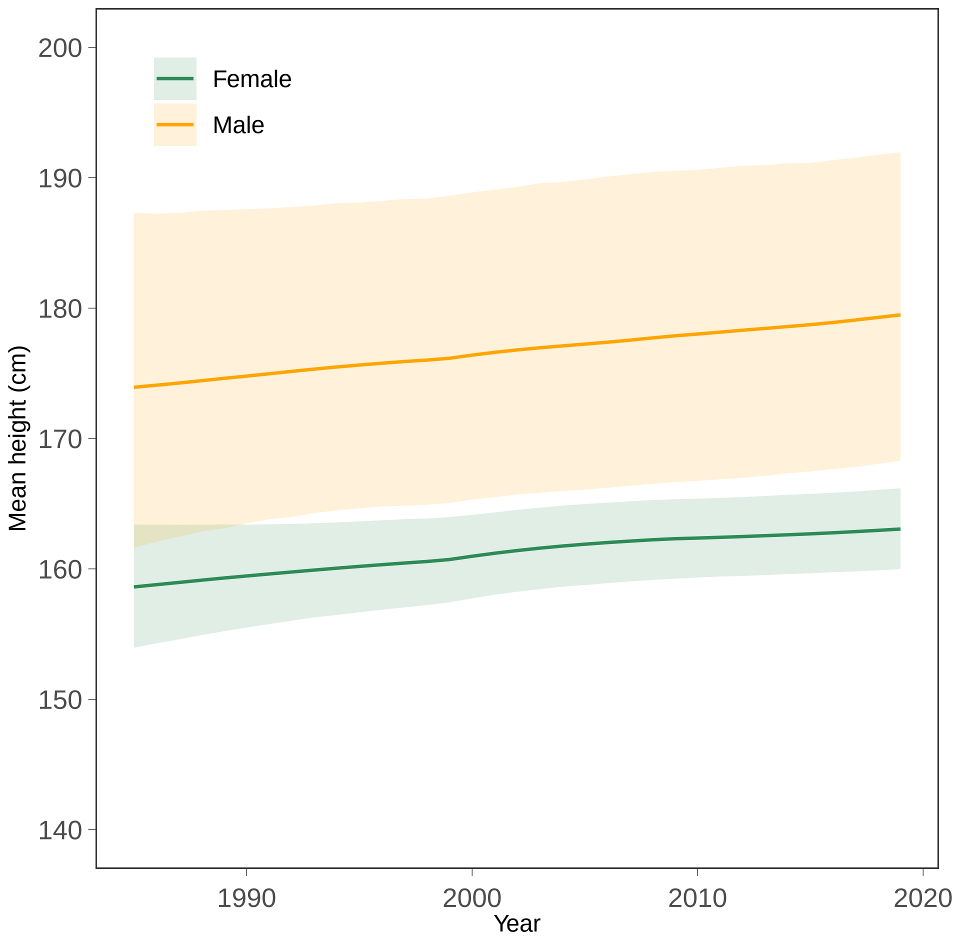

Time trends in BMI of 19 year olds

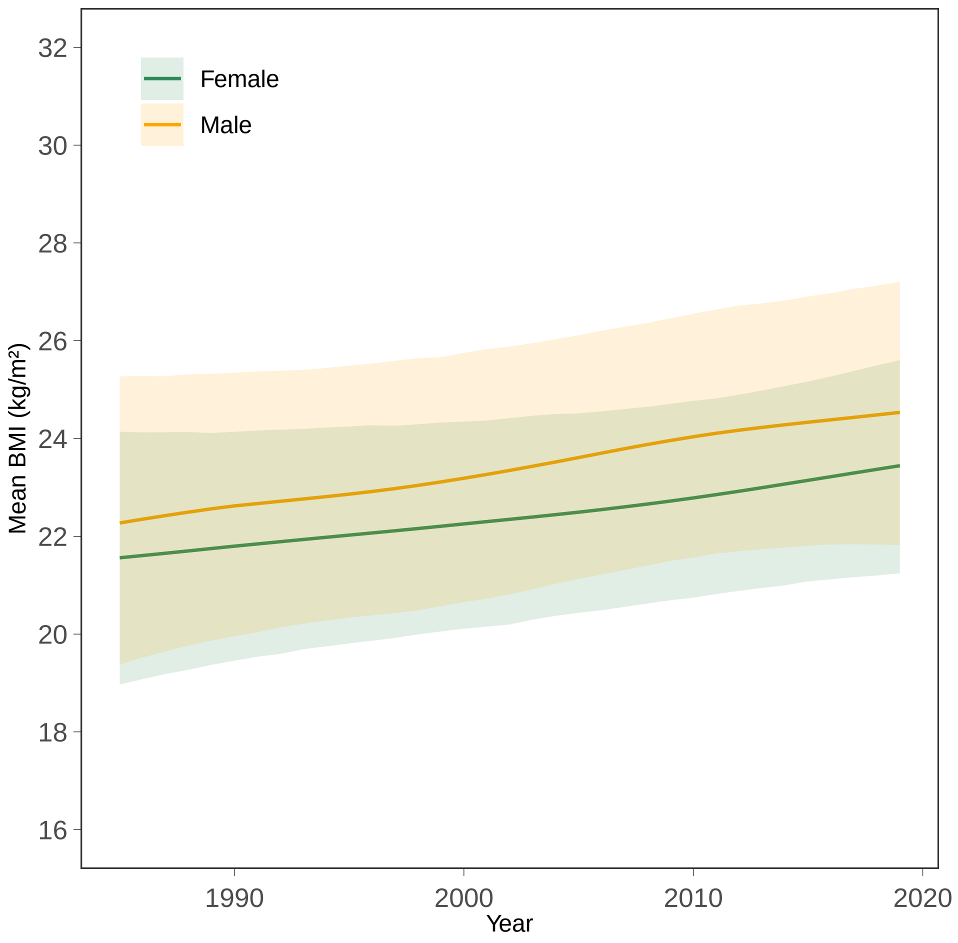

Height−for−age trajectories (2000 birth cohort)

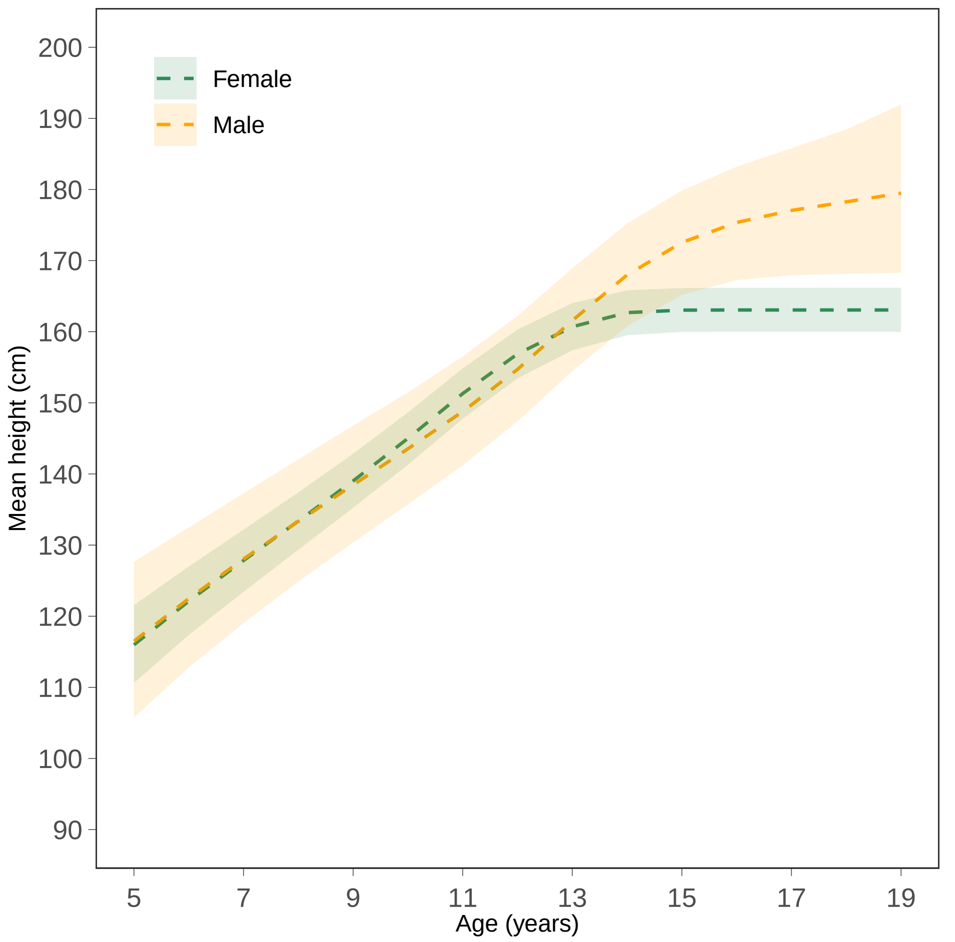

BMI−for−age trajectories (2000 birth cohort)

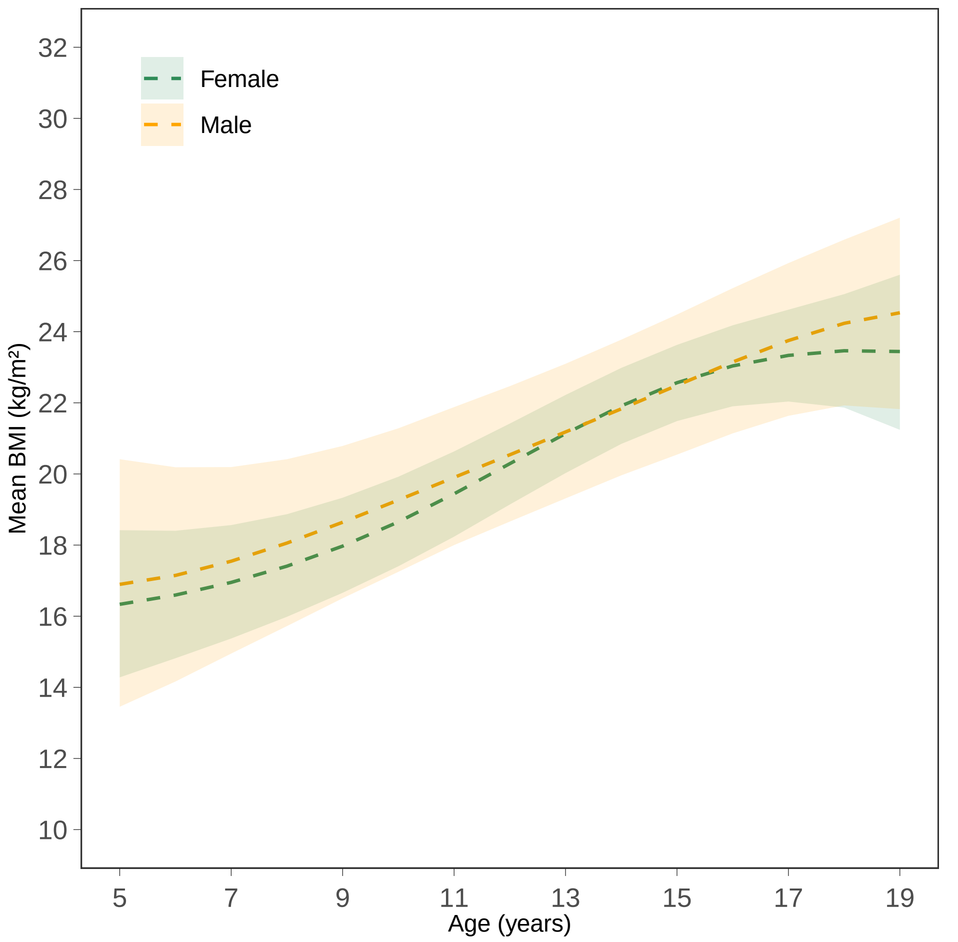

Qatar

Time trends in height of 19 year olds

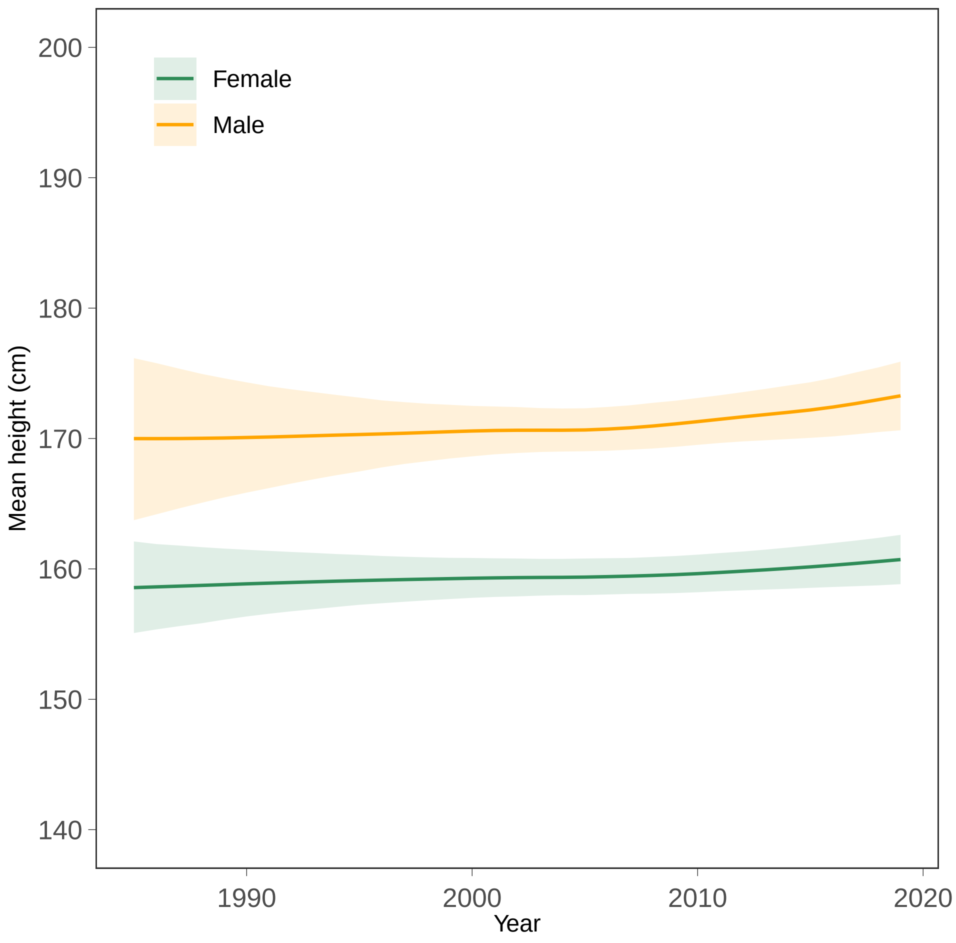

Time trends in BMI of 19 year olds

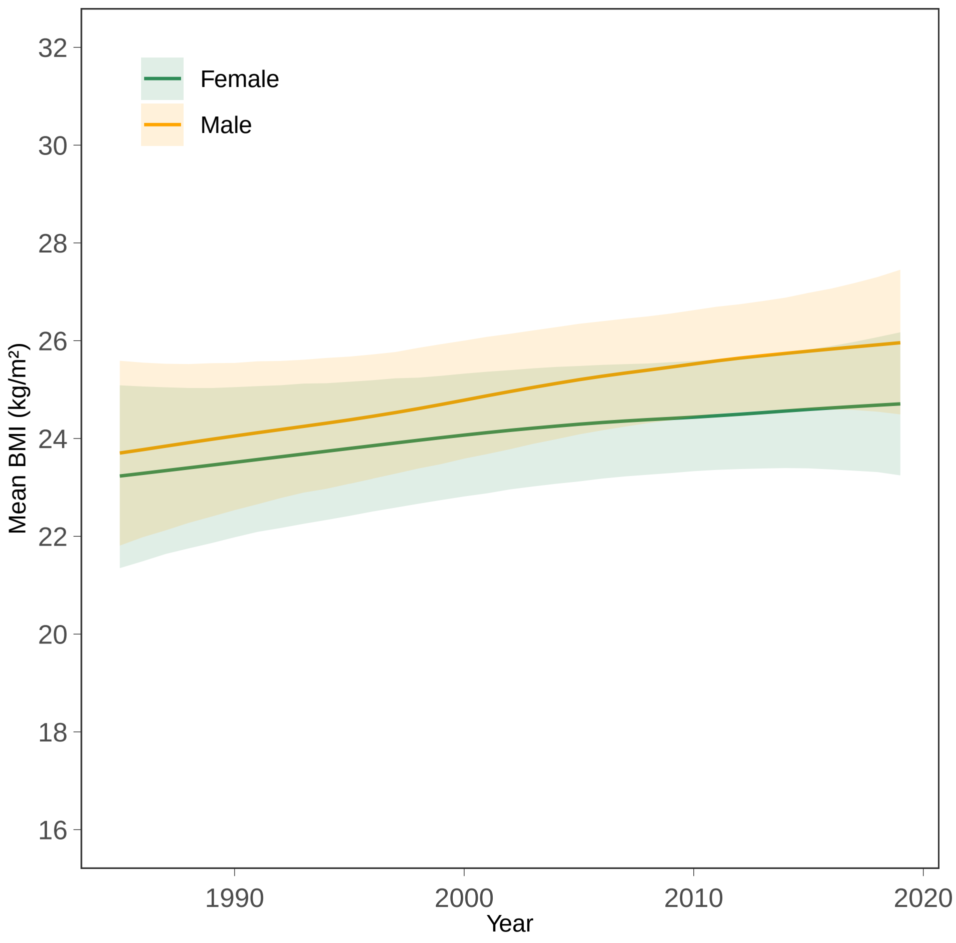

Height−for−age trajectories (2000 birth cohort)

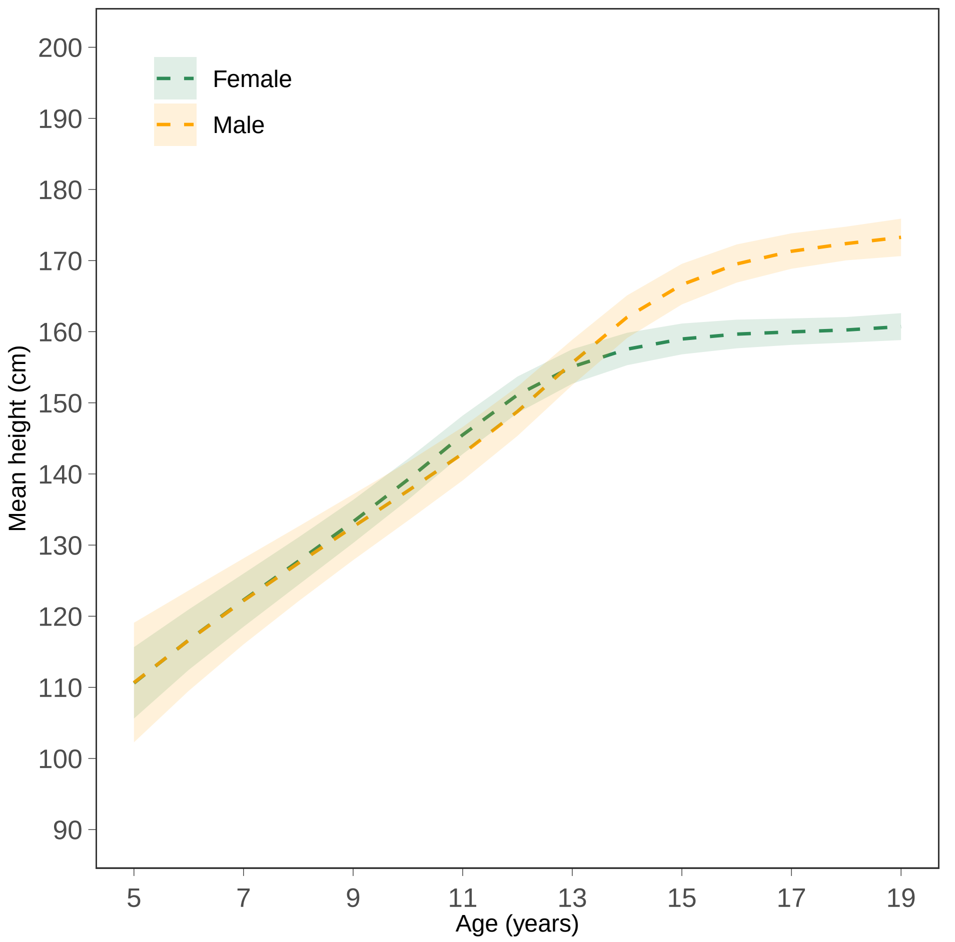

BMI−for−age trajectories (2000 birth cohort)

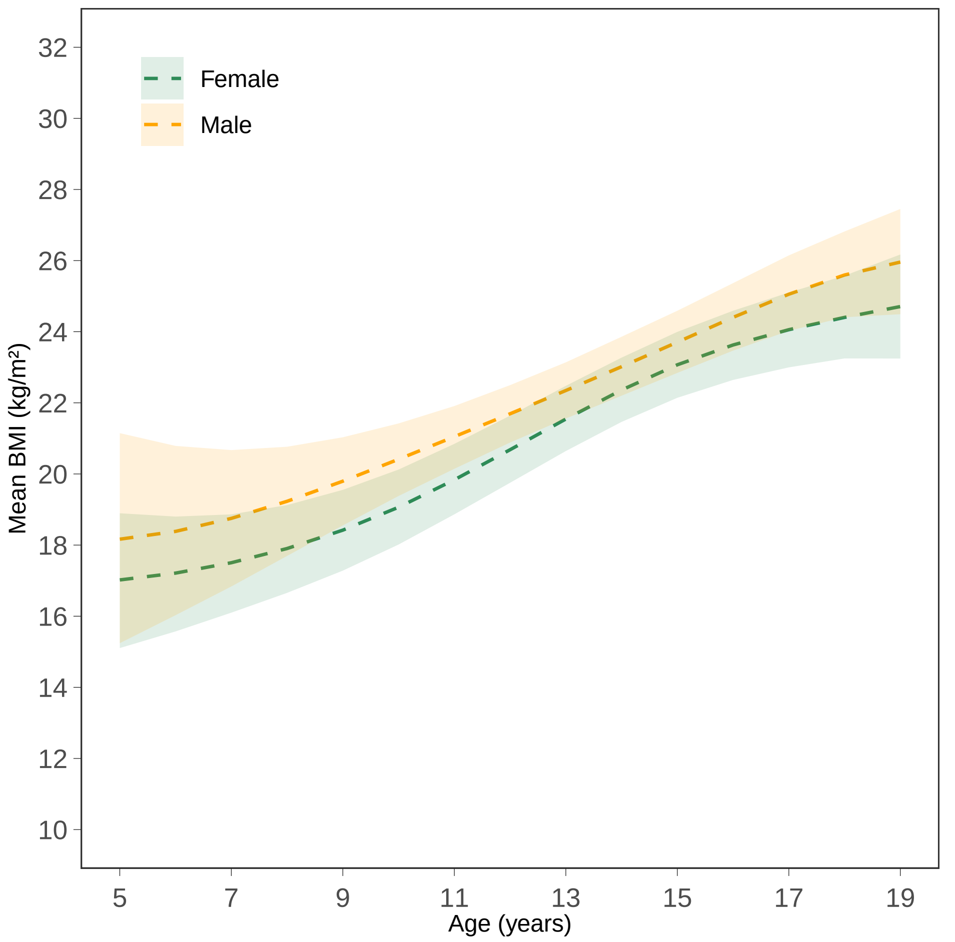

Romania

Time trends in height of 19 year olds

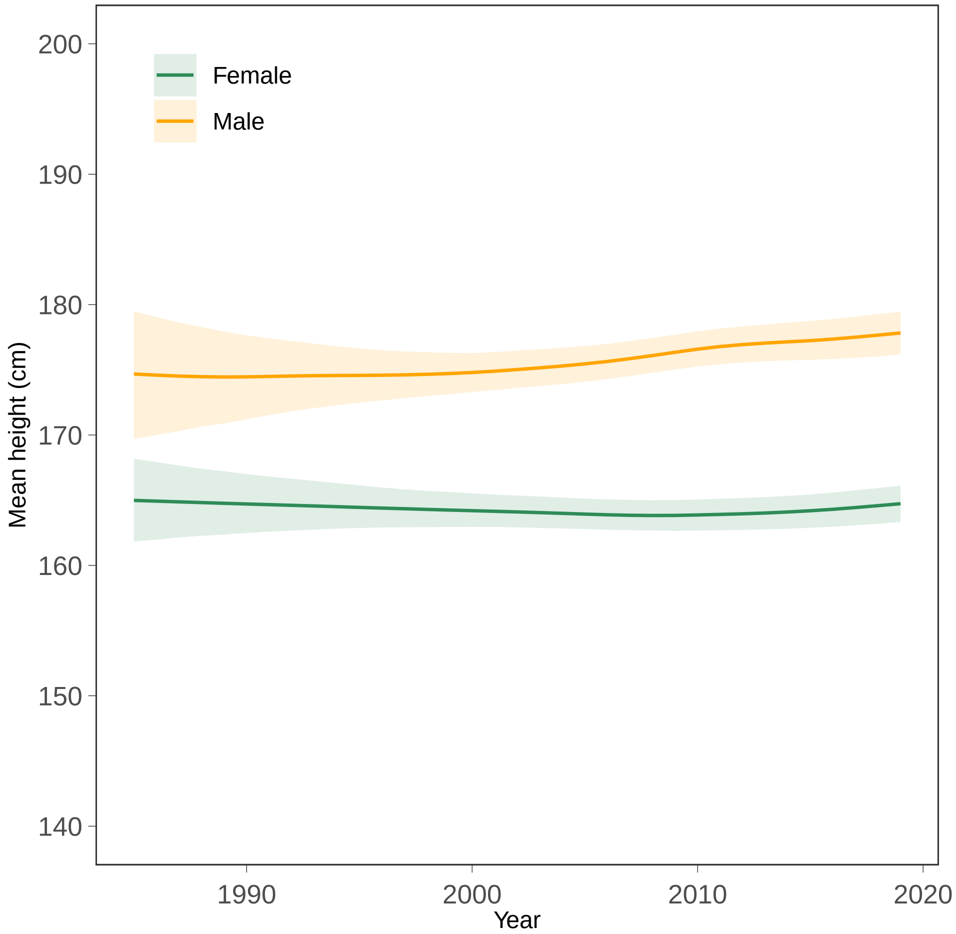

Time trends in BMI of 19 year olds

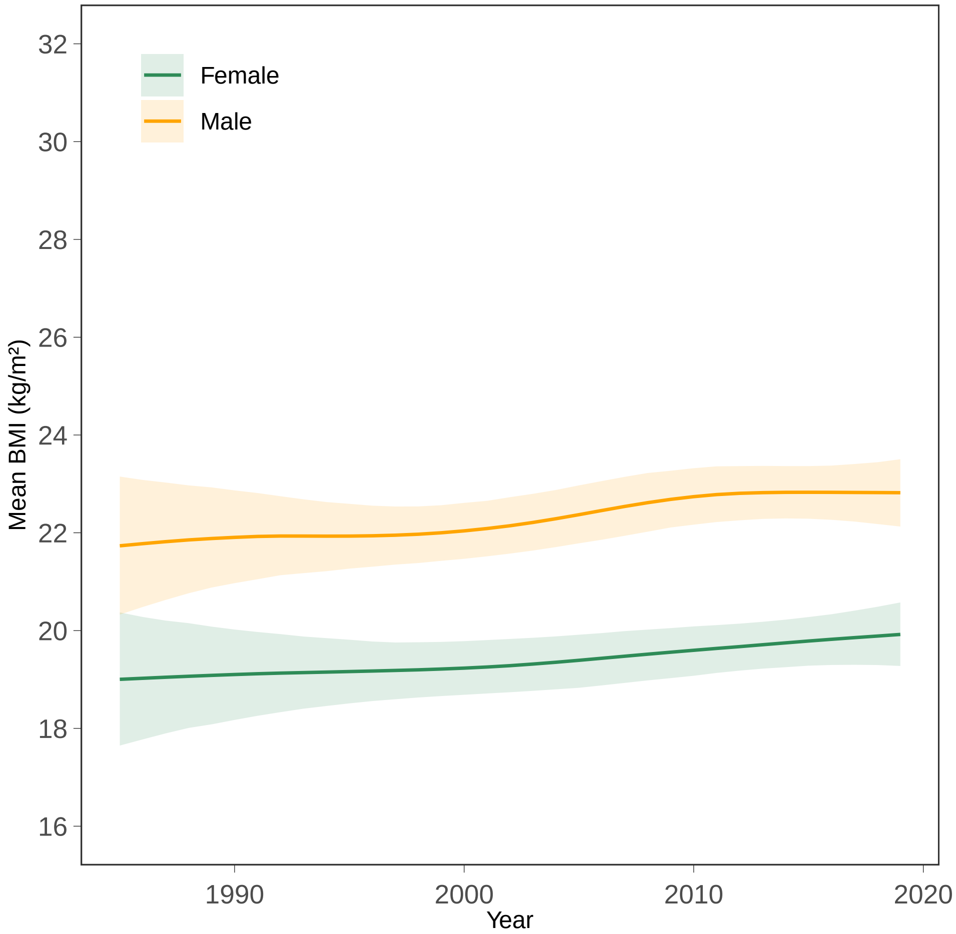

Height−for−age trajectories (2000 birth cohort)

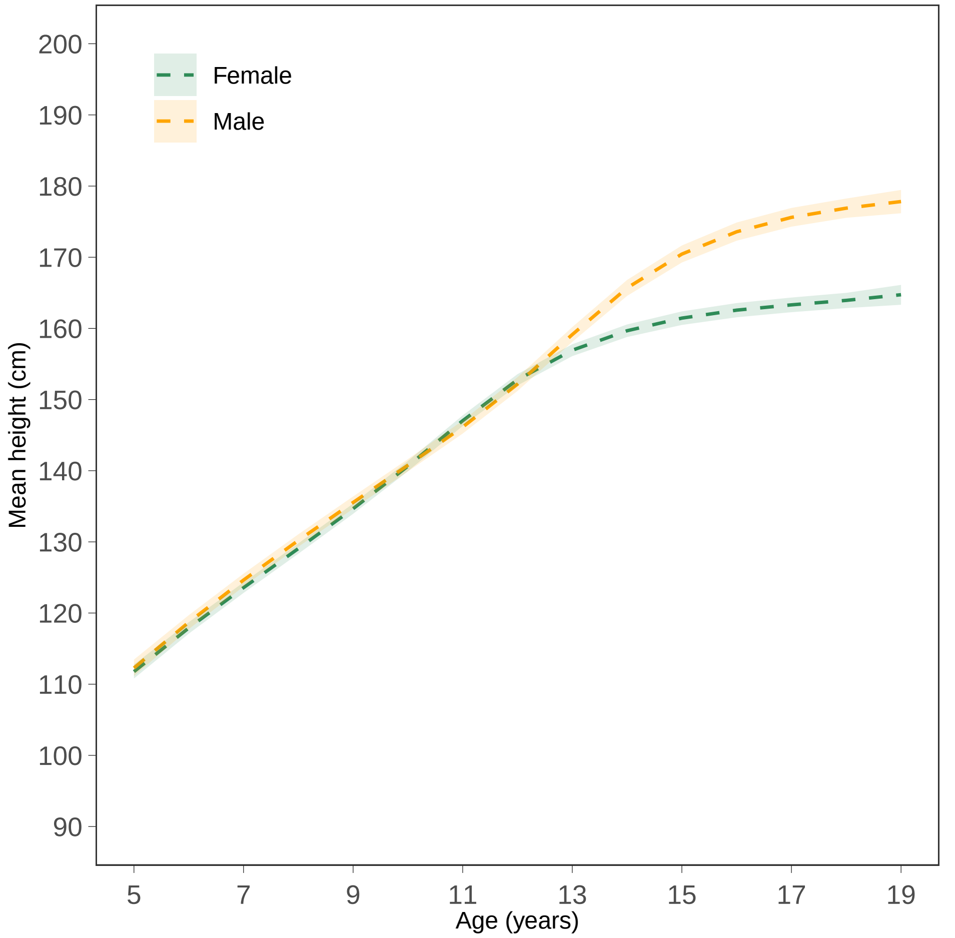

BMI−for−age trajectories (2000 birth cohort)

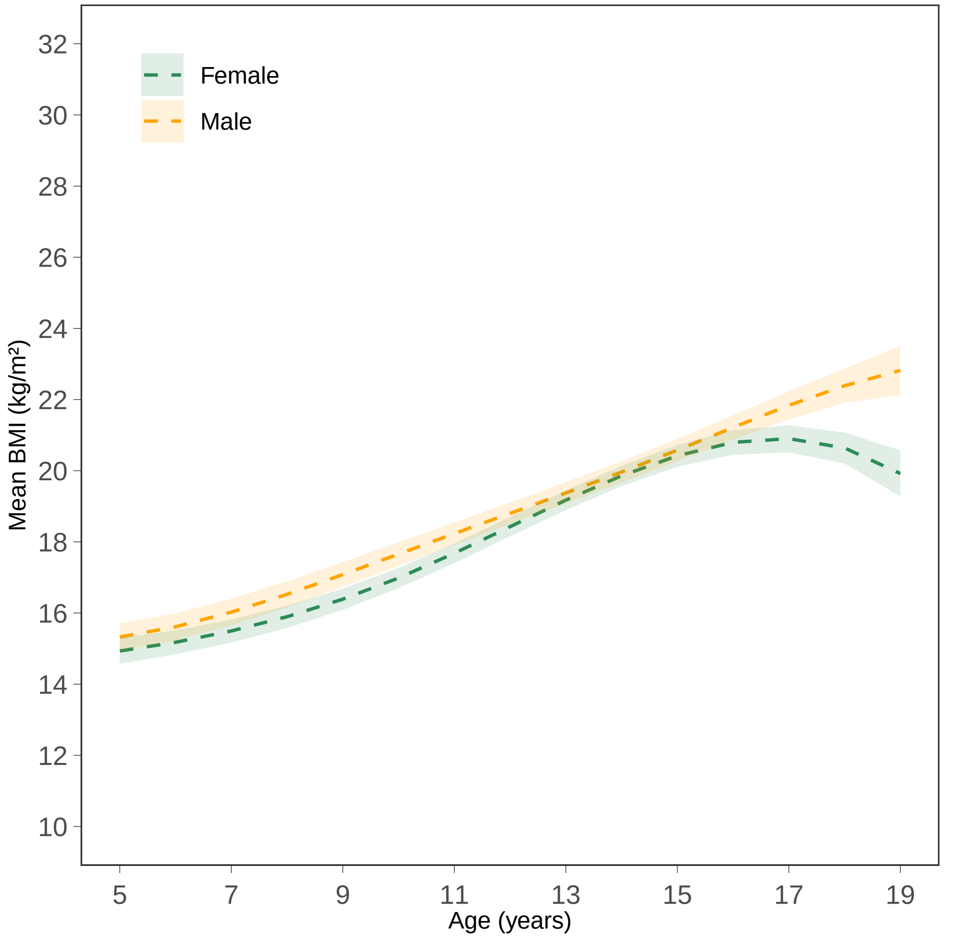

Russian Federation

Time trends in height of 19 year olds

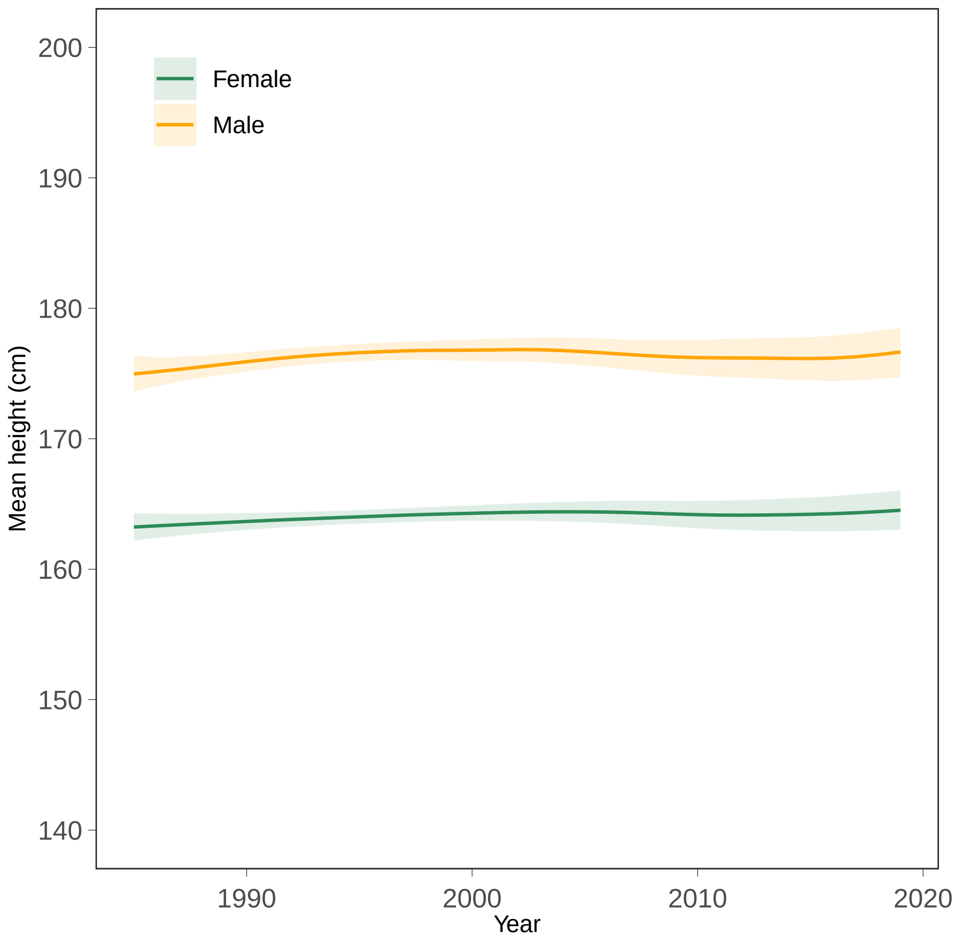

Time trends in BMI of 19 year olds

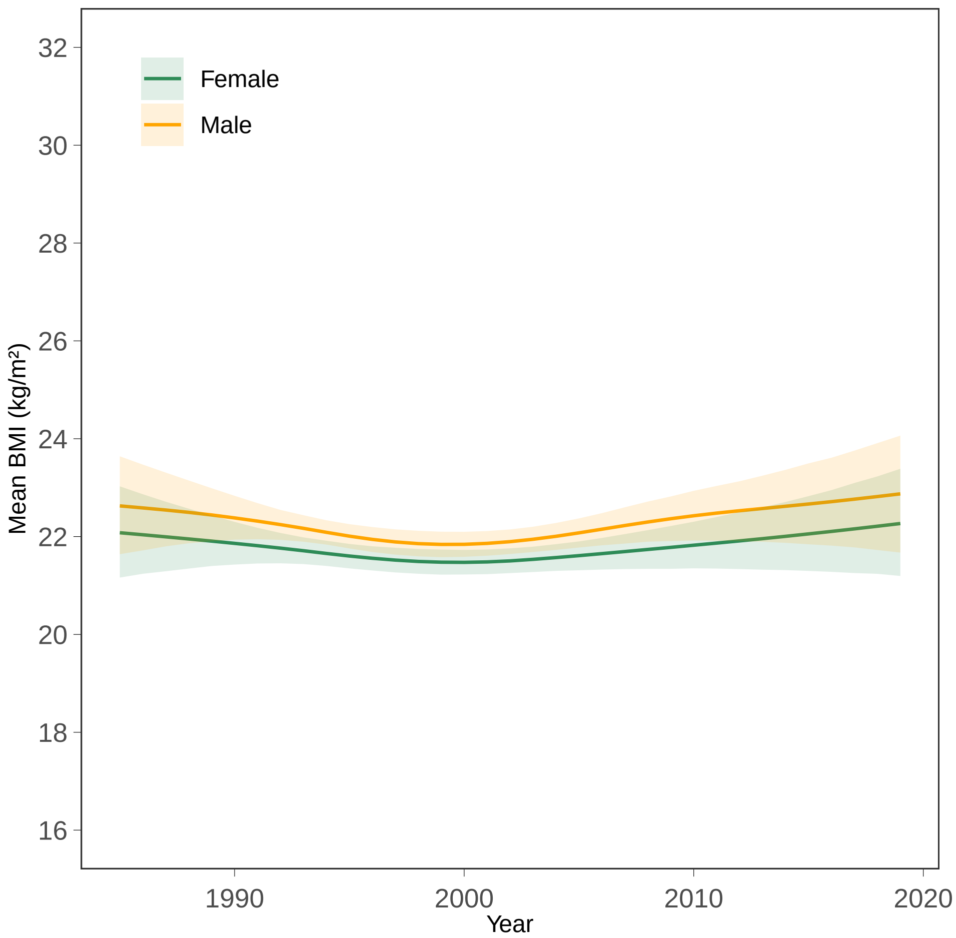

Height−for−age trajectories (2000 birth cohort)

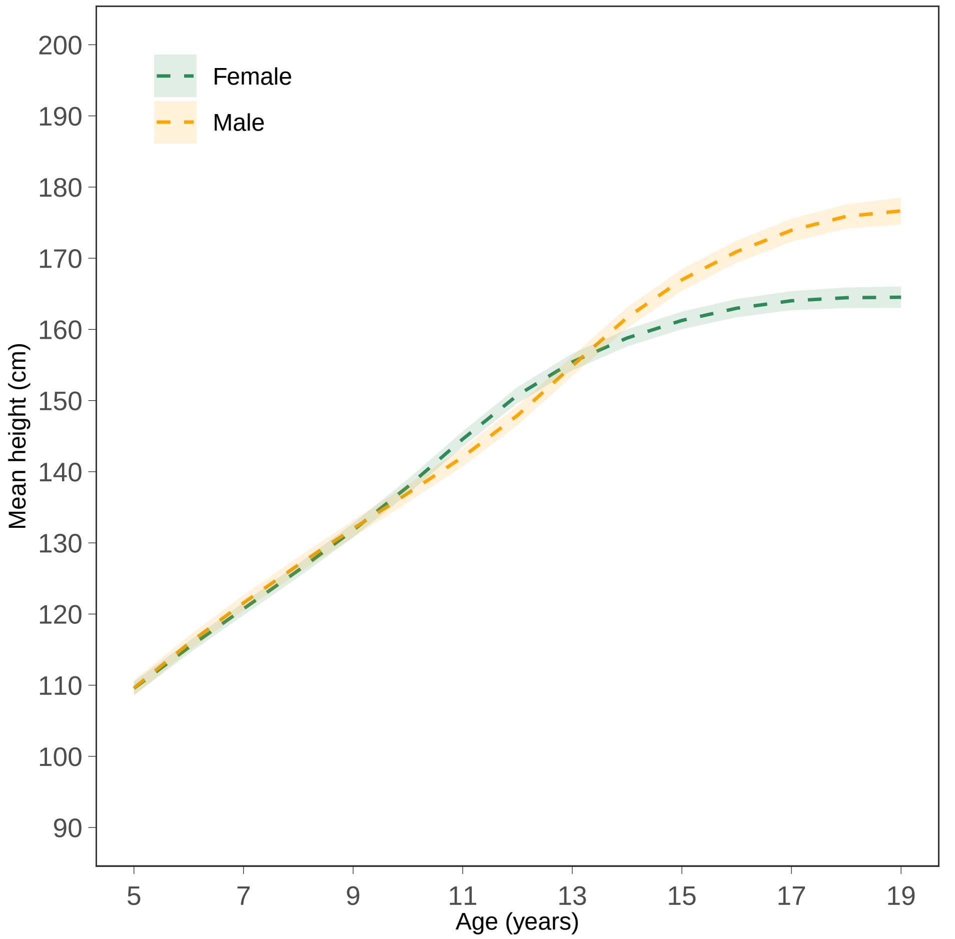

BMI−for−age trajectories (2000 birth cohort)

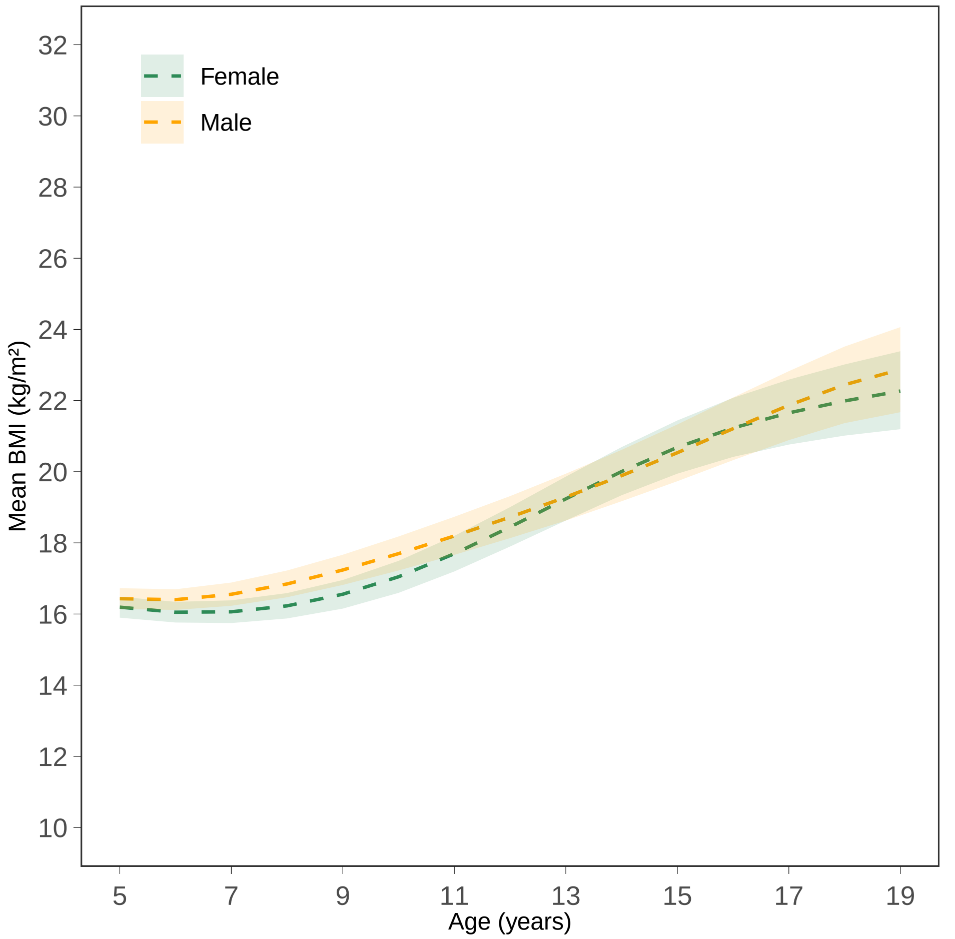

Rwanda

Time trends in height of 19 year olds

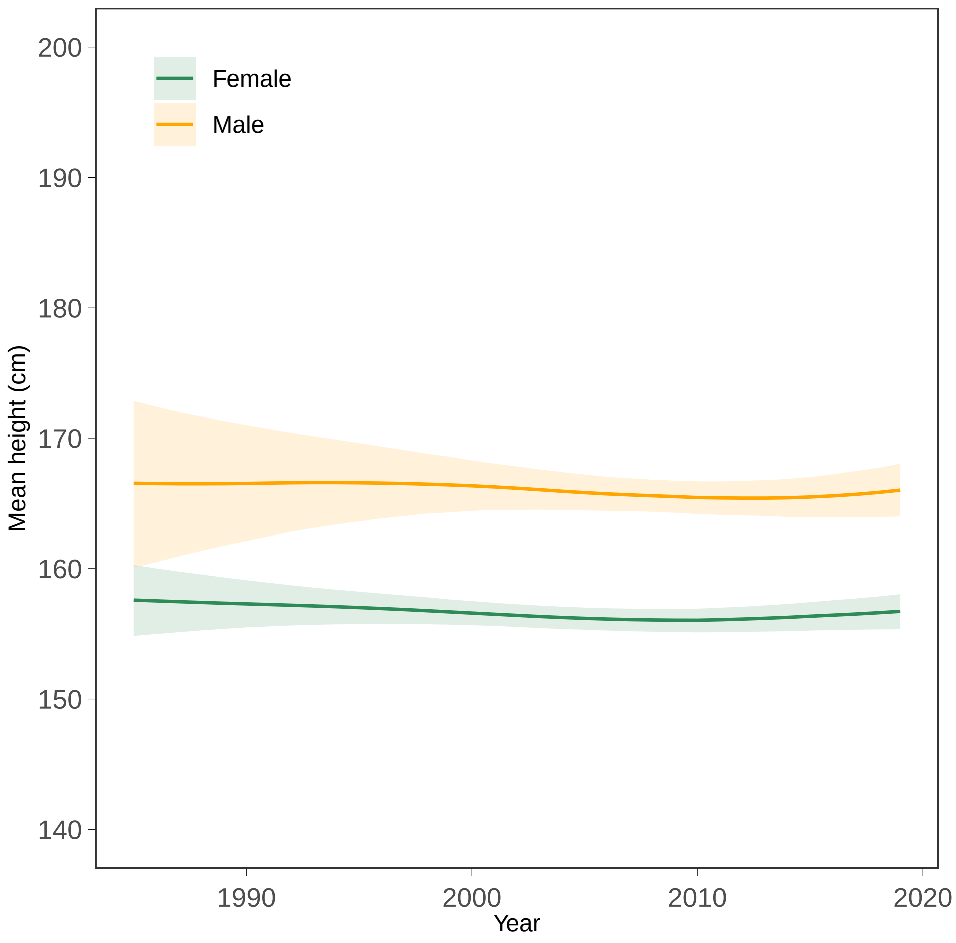

Time trends in BMI of 19 year olds

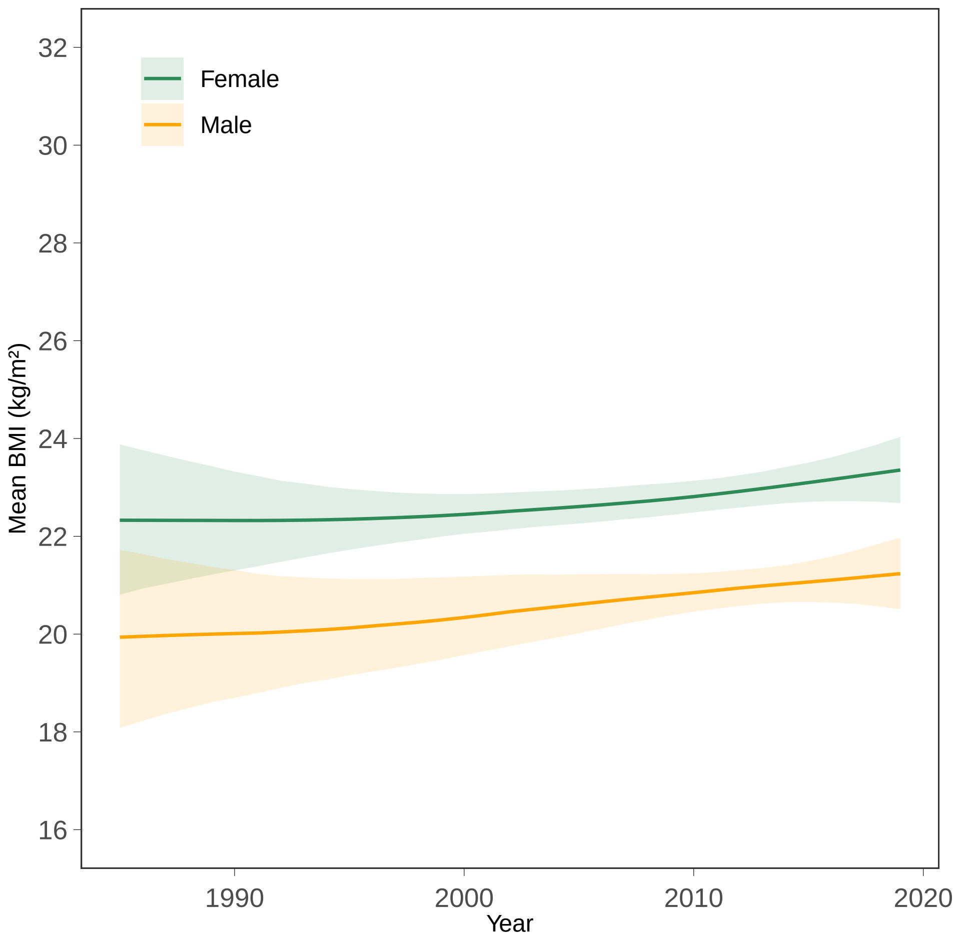

Height−for−age trajectories (2000 birth cohort)

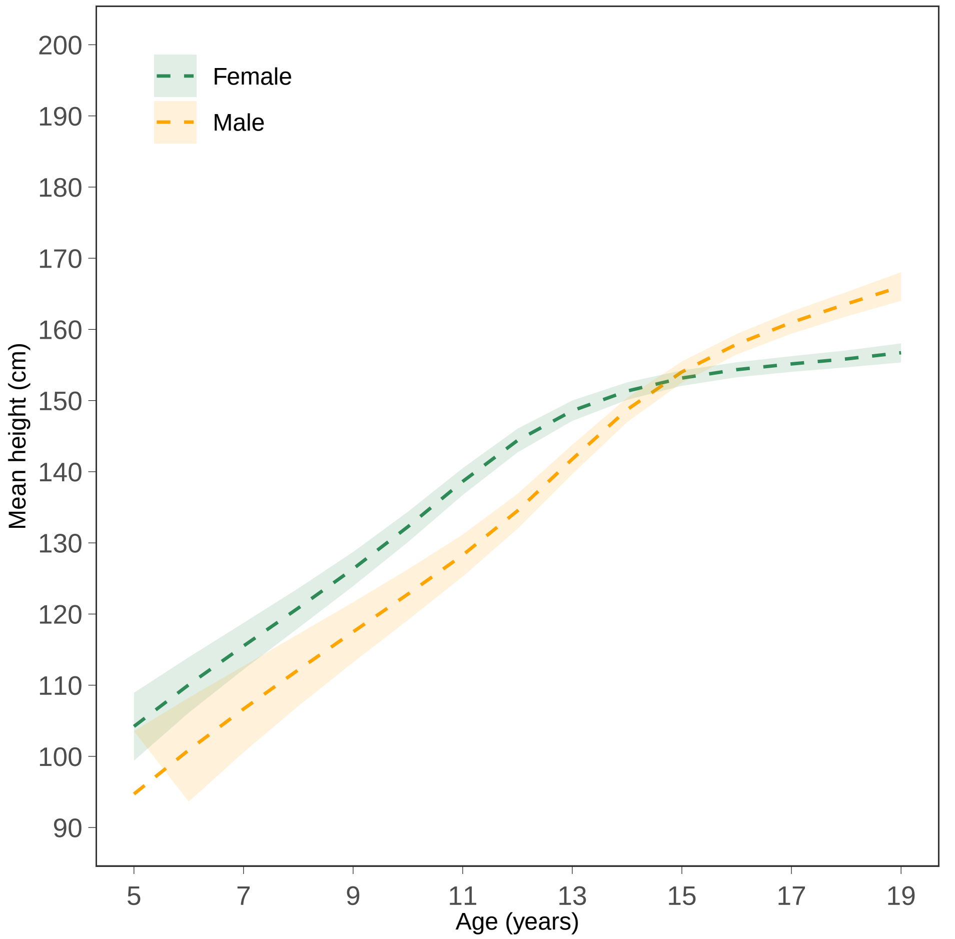

BMI−for−age trajectories (2000 birth cohort)

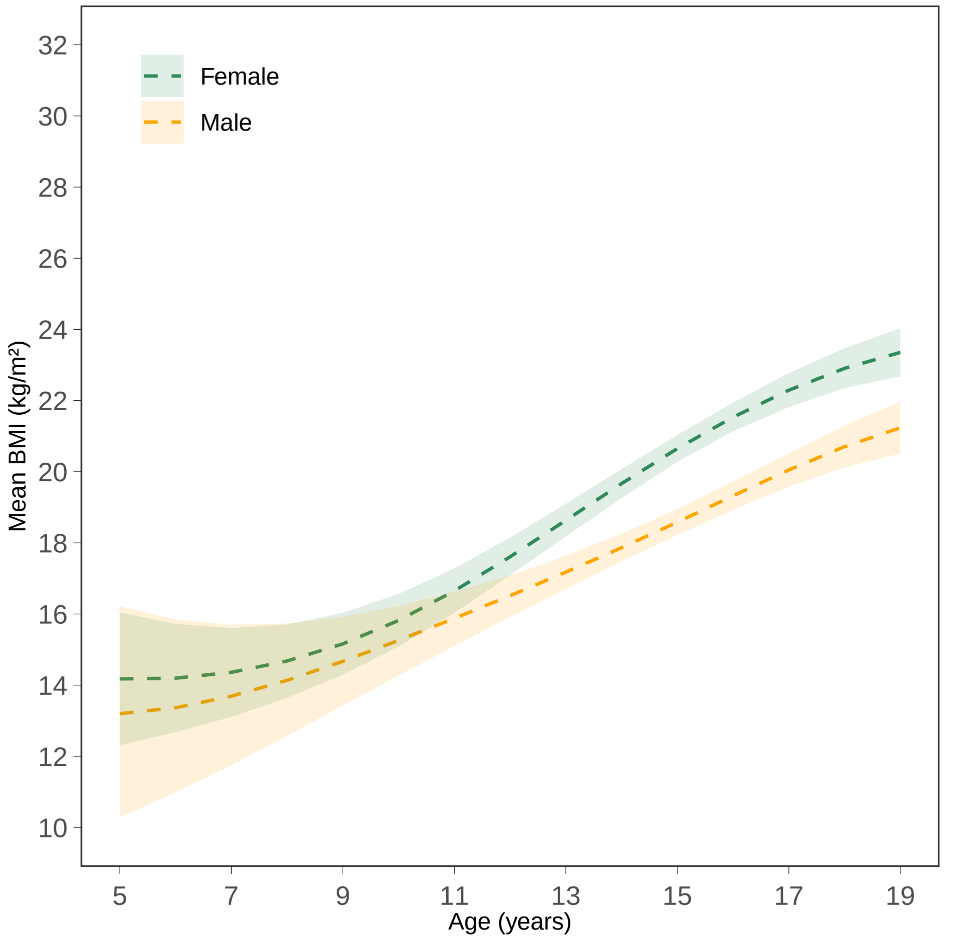

Saint Kitts and Nevis

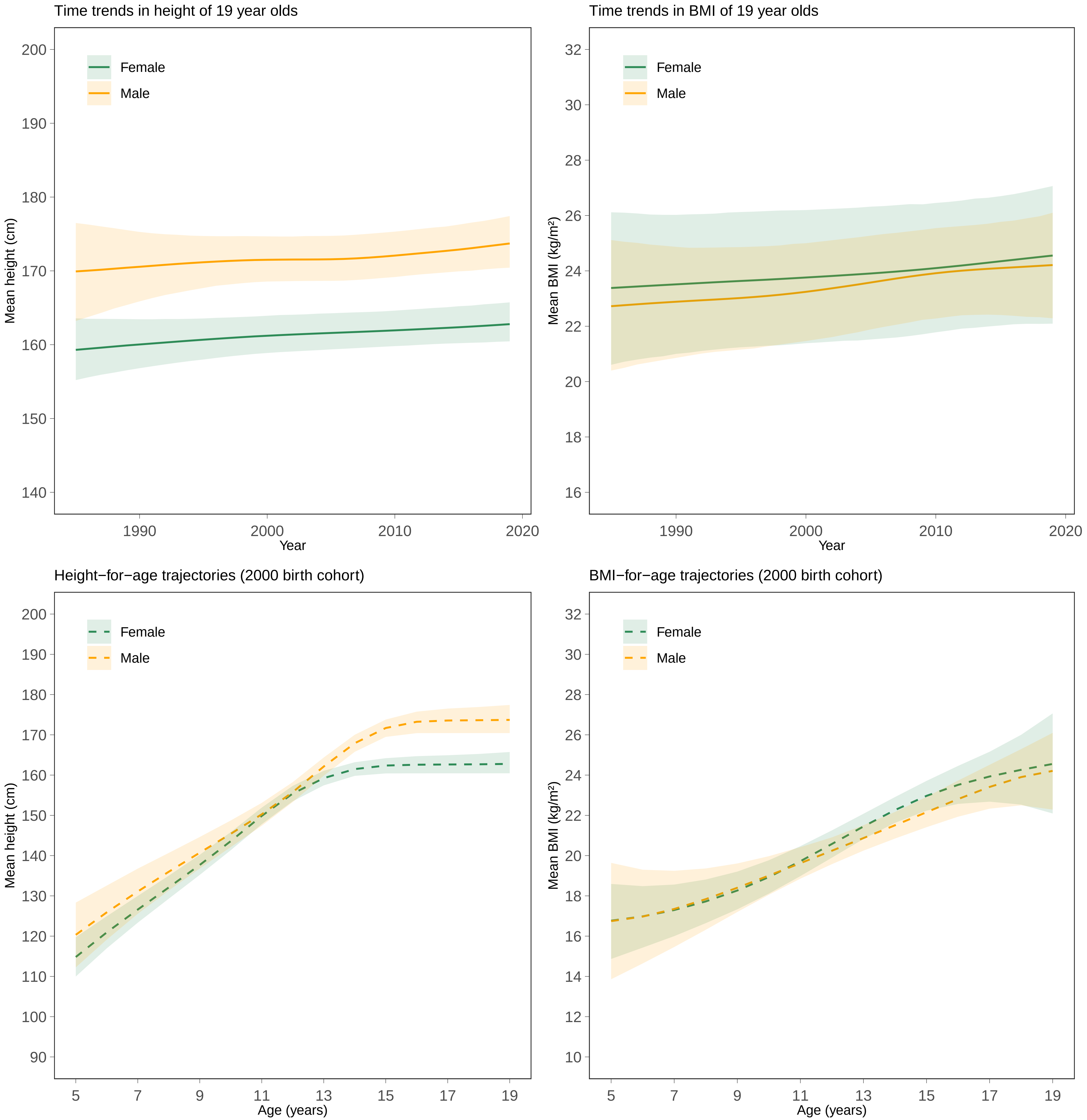

Saint Lucia

Time trends in height of 19 year olds

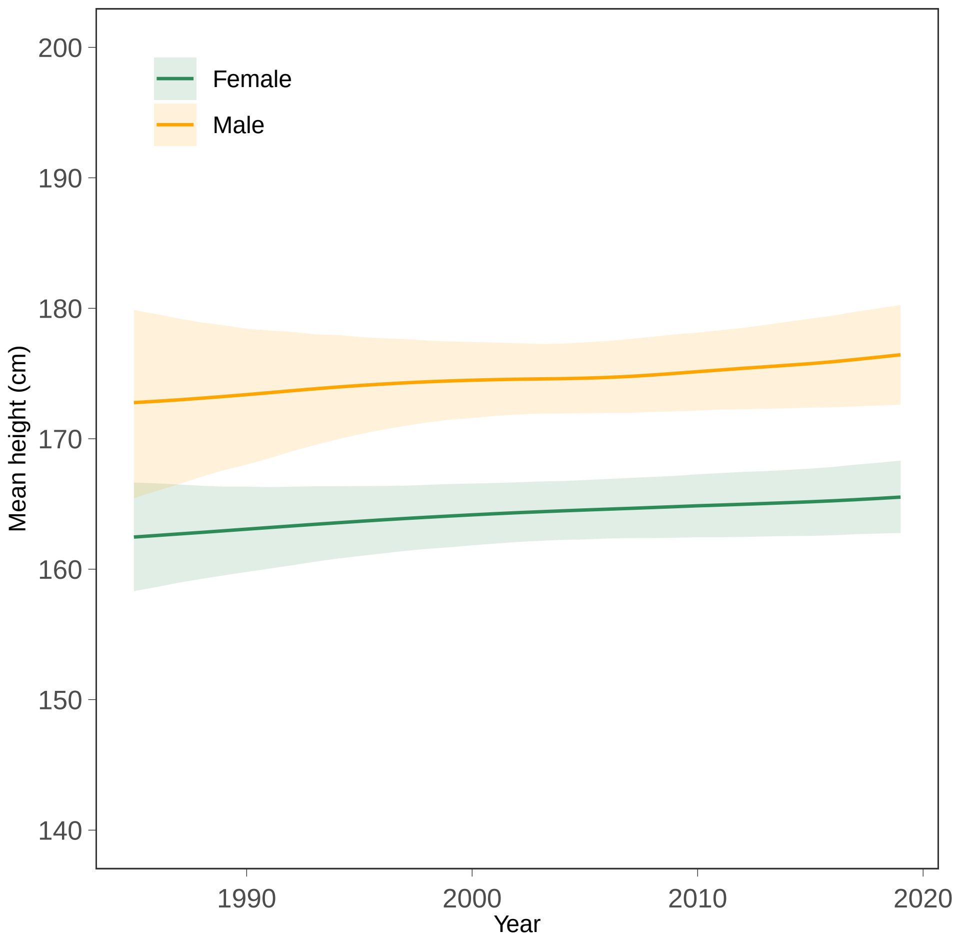

Time trends in BMI of 19 year olds

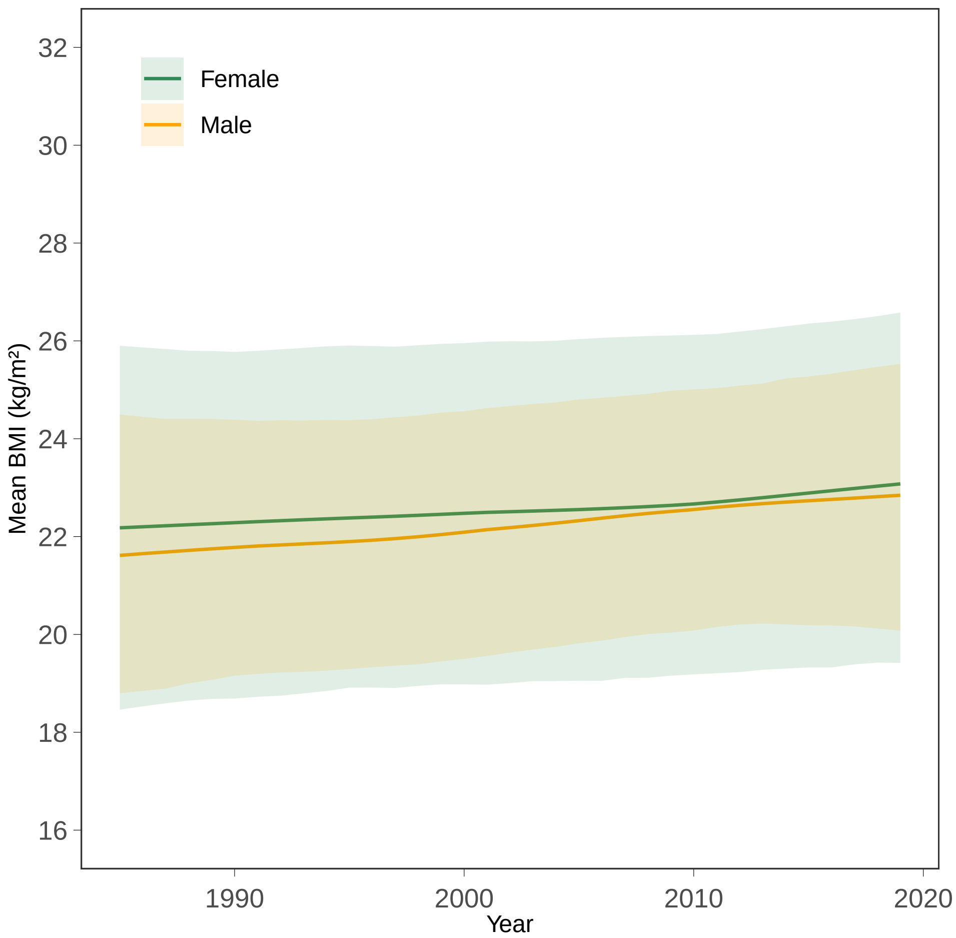

Height−for−age trajectories (2000 birth cohort)

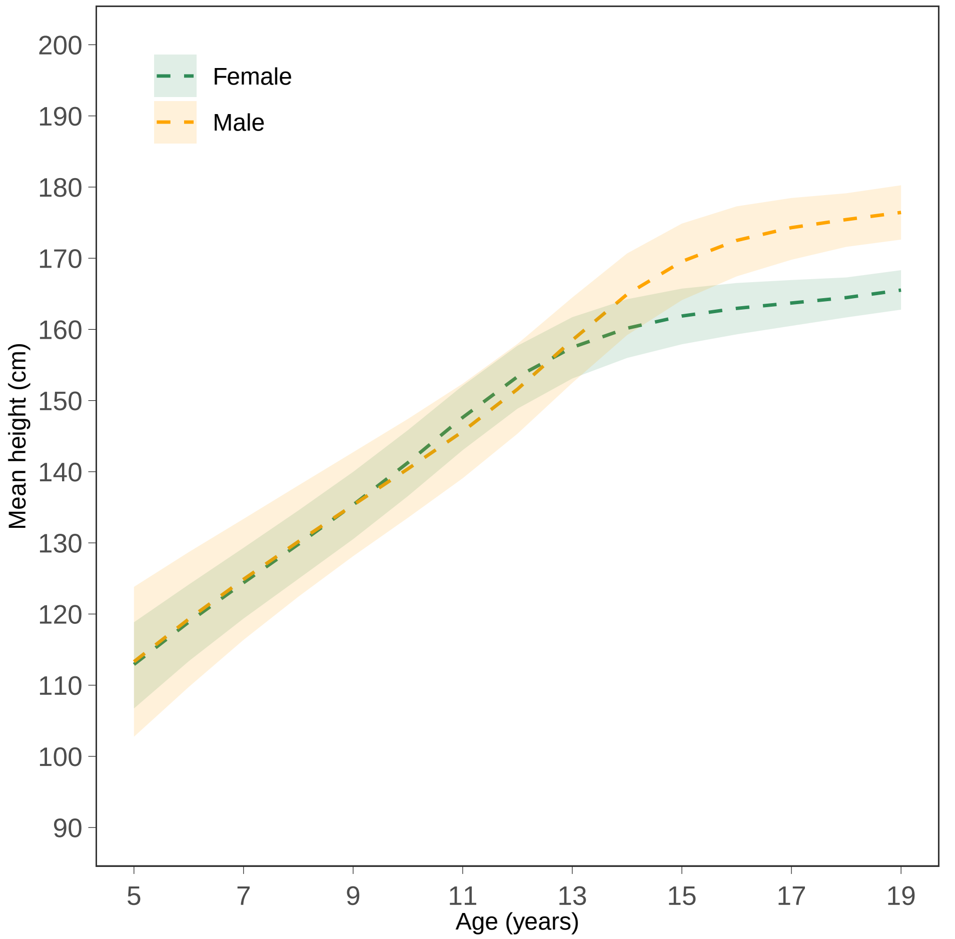

BMI−for−age trajectories (2000 birth cohort)

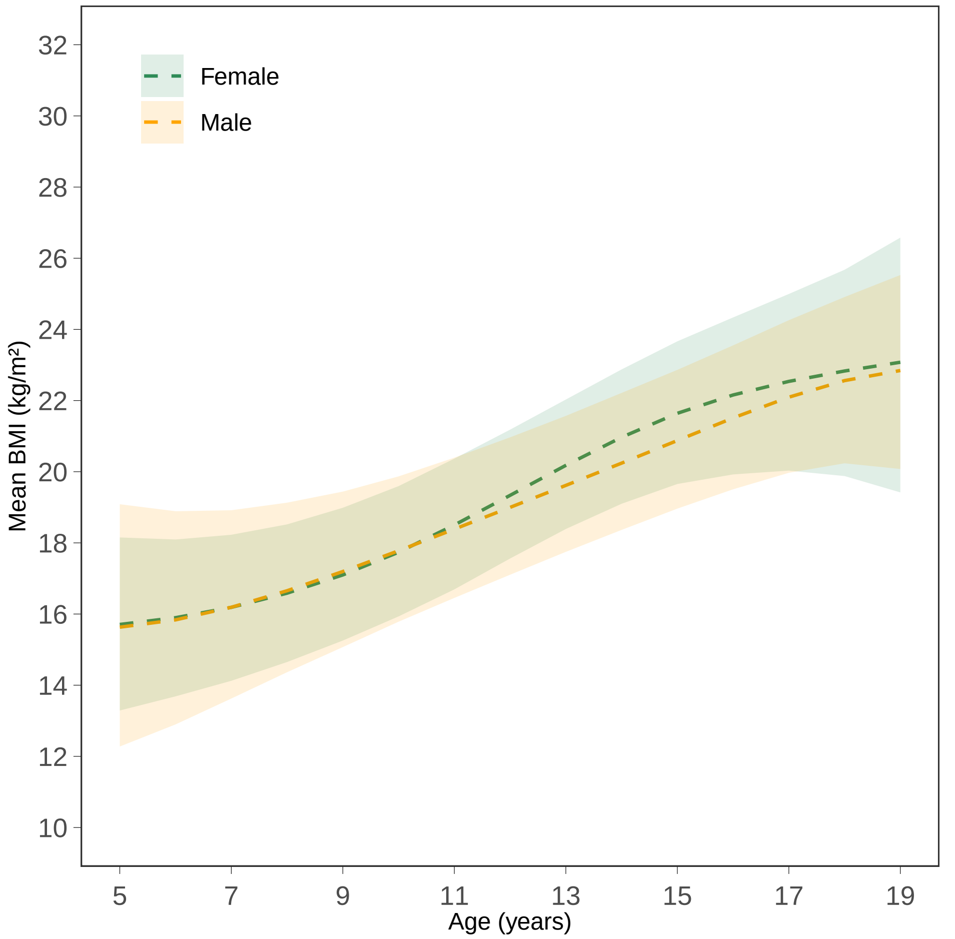

Saint Vincent and the Grenadines

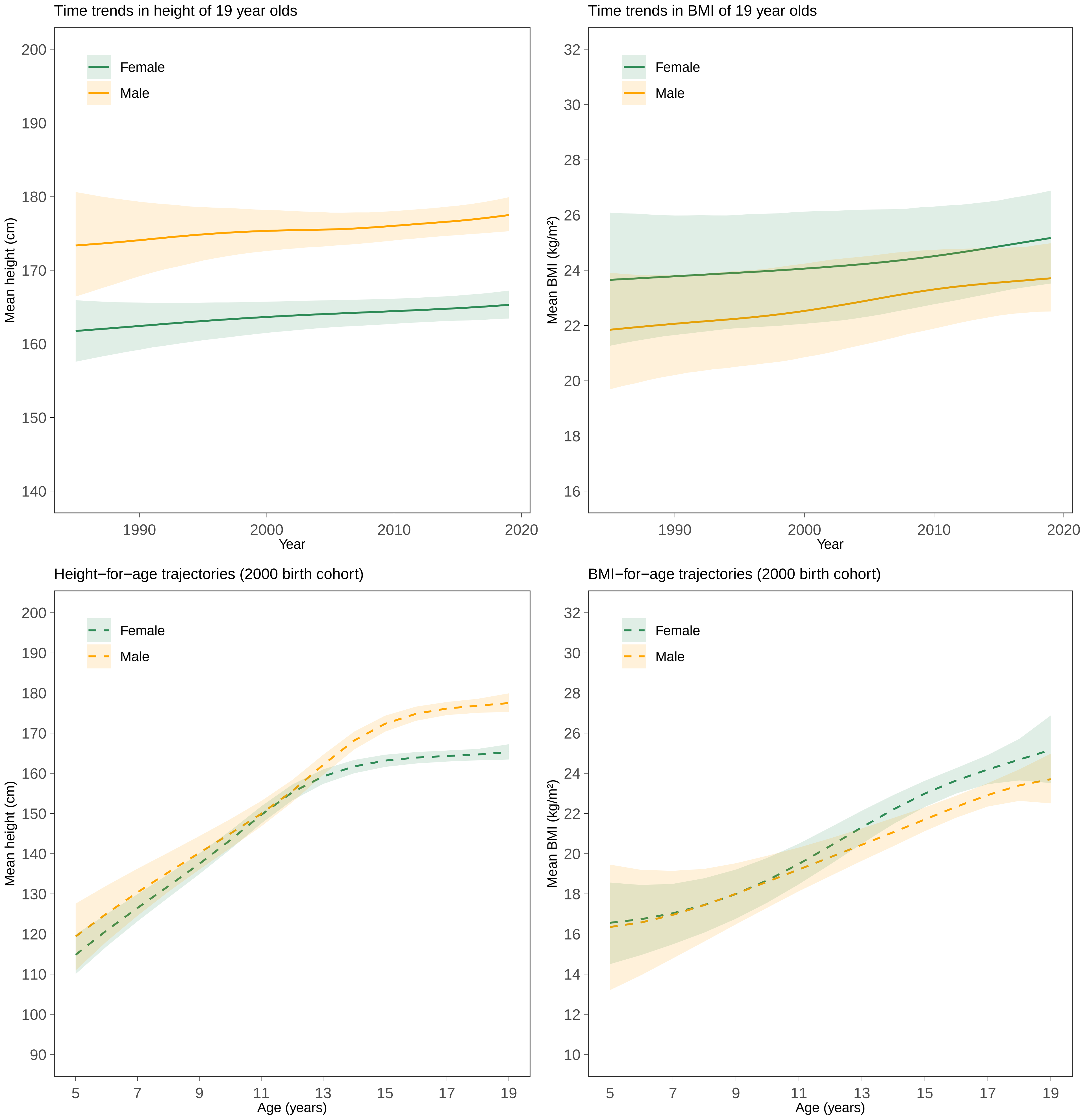

Samoa

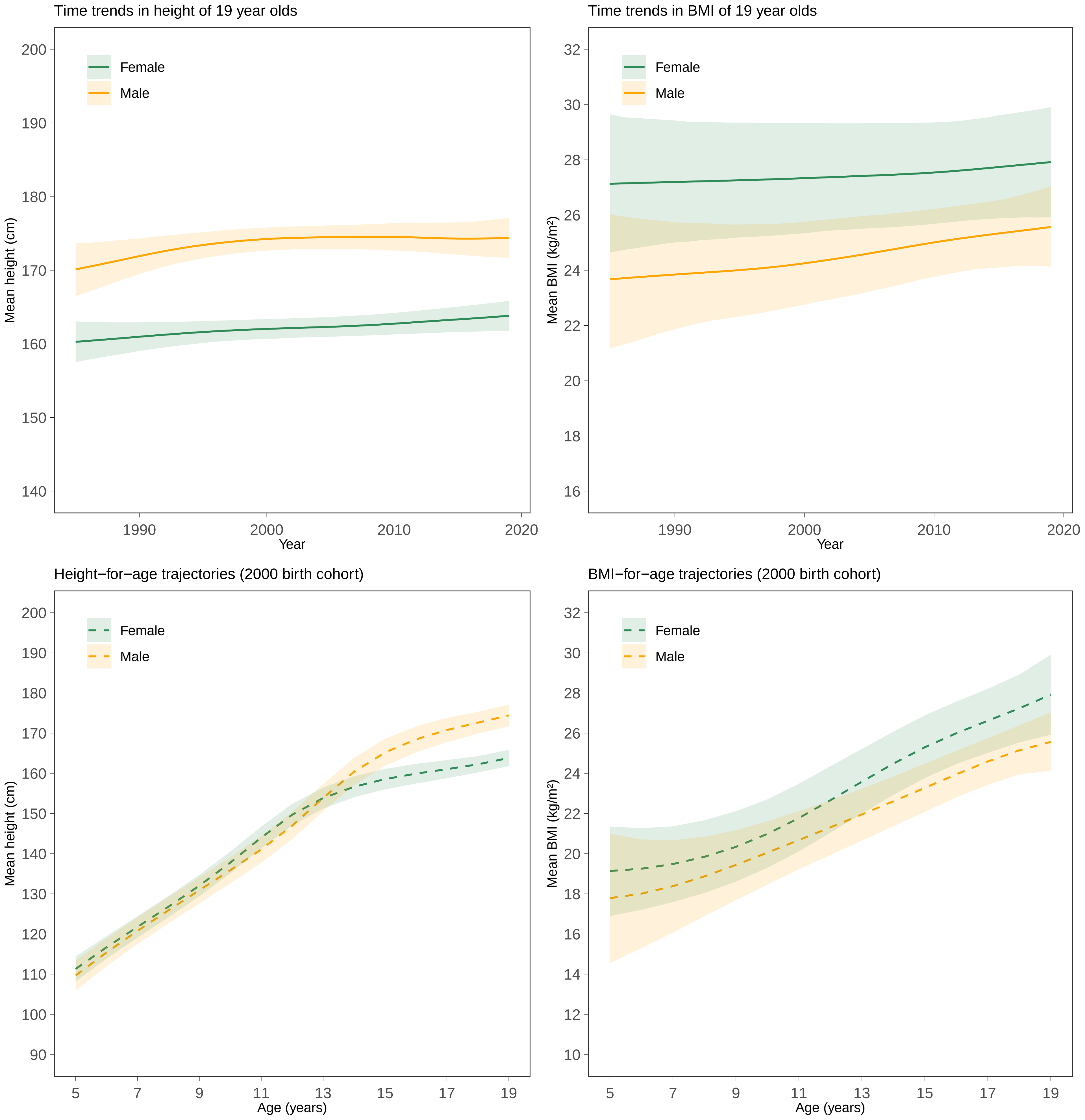

Time trends in height of 19 year olds
Female
Male
Mean height (cm)
200
190
180
170
160
150
140
1990
2000
2010
2020
Year
Time trends in BMI of 19 year olds
Female
Male
Mean BMI (kg/m²)
32
30
28
26
24
22
20
18
16
1990
2000
2010
2020
Year
Height−for−age trajectories (2000 birth cohort)
Female
Male
Mean height (cm)
200
190
180
170
160
150
140
130
120
110
100
90
5
7
9
11
13
15
17
19
Age (years)
BMI−for−age trajectories (2000 birth cohort)
Female
Male
Mean BMI (kg/m²)
32
30
28
26
24
22
20
18
16
14
12
10
5
7
9
11
13
15
17
19
Age (years)

Sao Tome and Principe

Time trends in height of 19 year olds

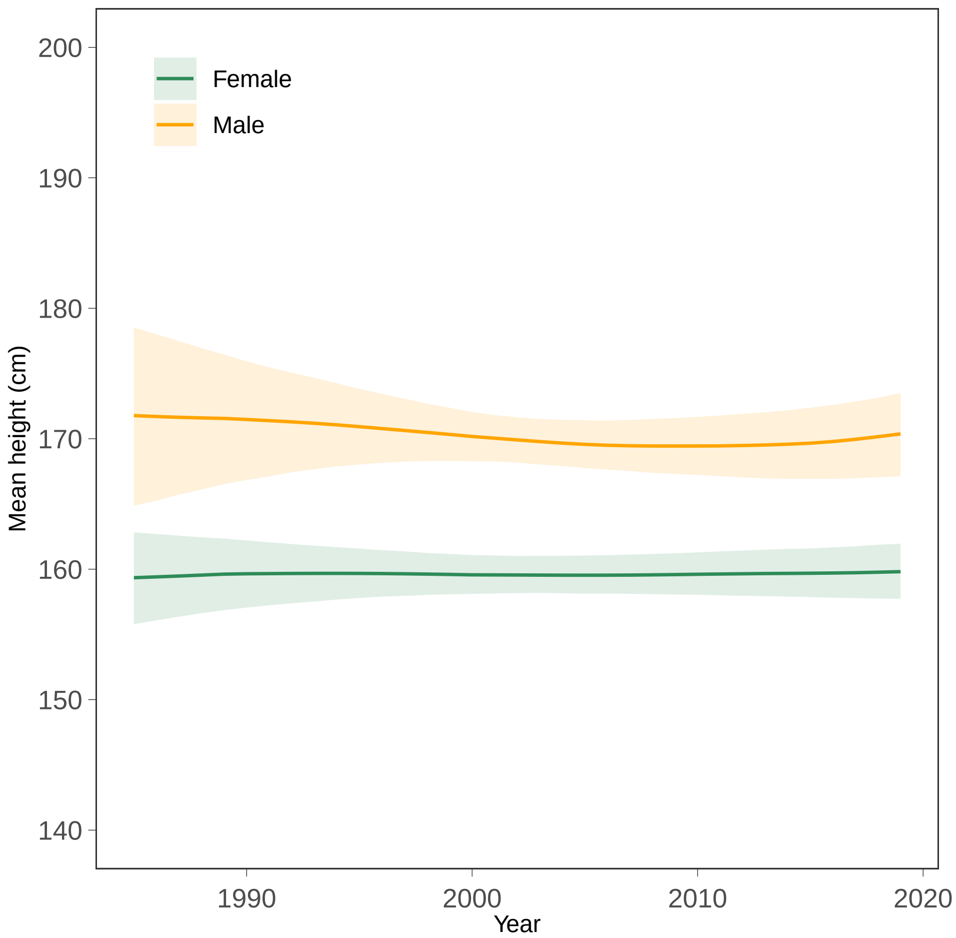

Time trends in BMI of 19 year olds

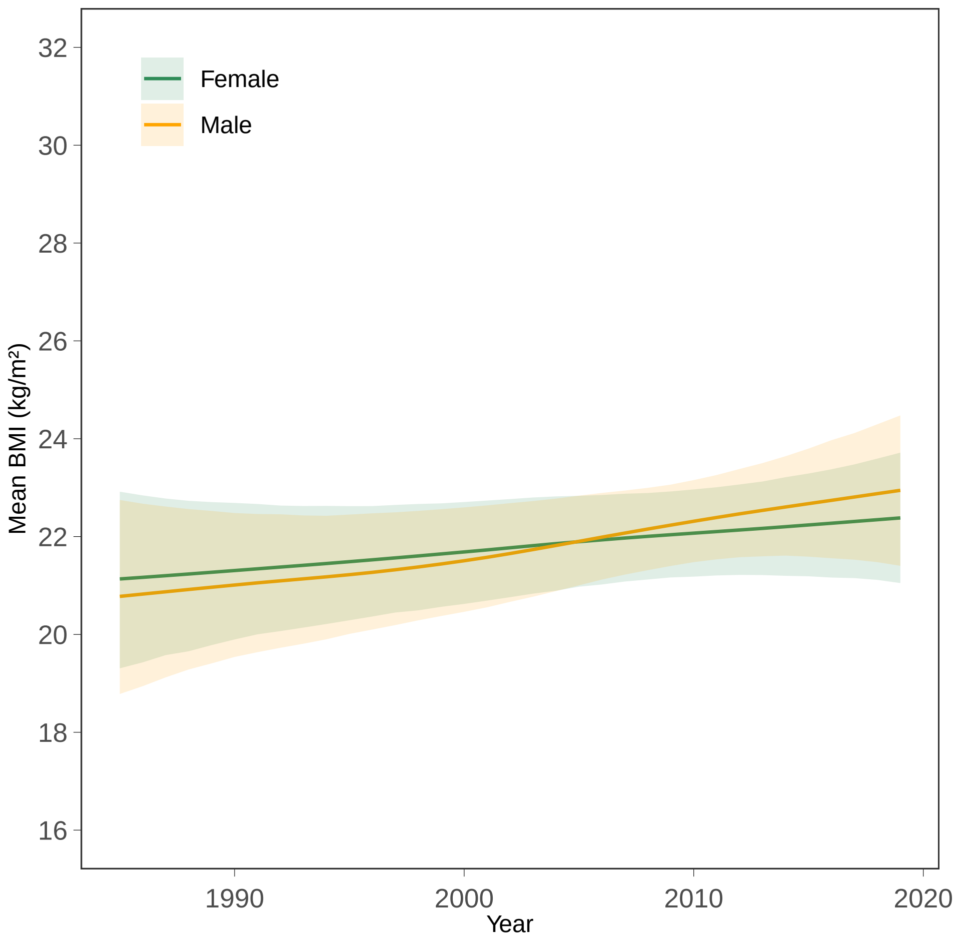

Height−for−age trajectories (2000 birth cohort)

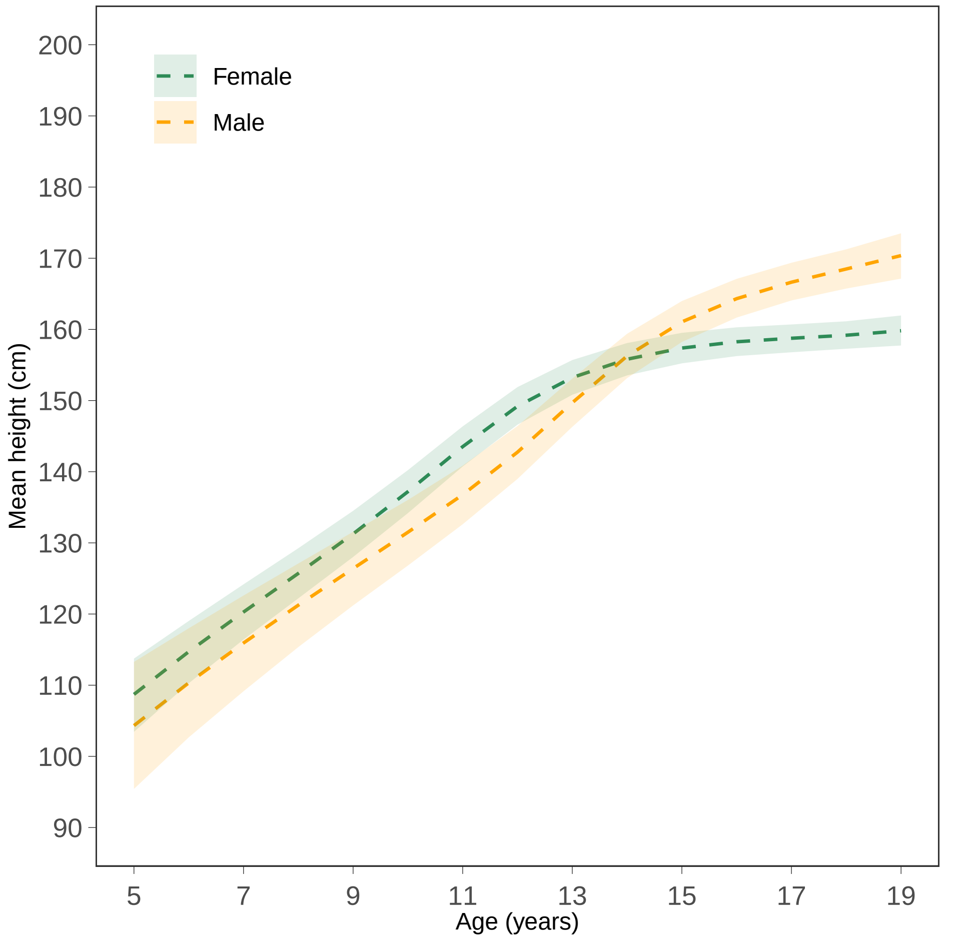

BMI−for−age trajectories (2000 birth cohort)

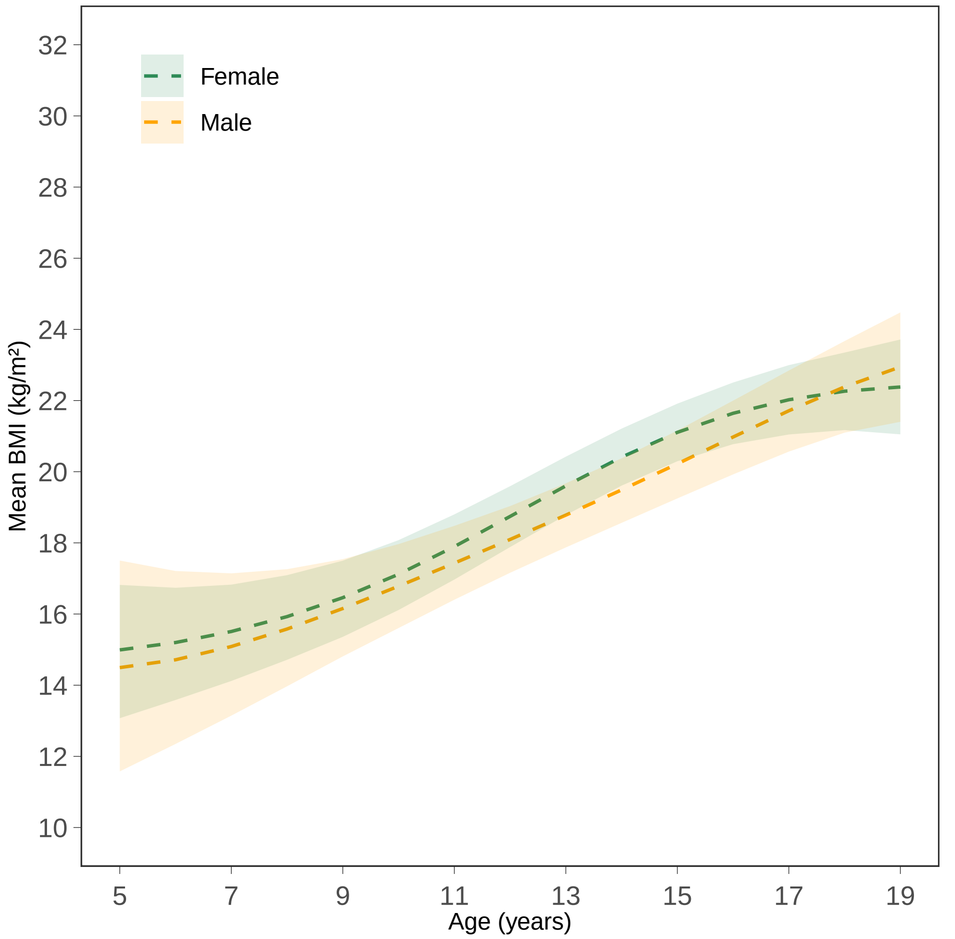

Saudi Arabia

Time trends in height of 19 year olds

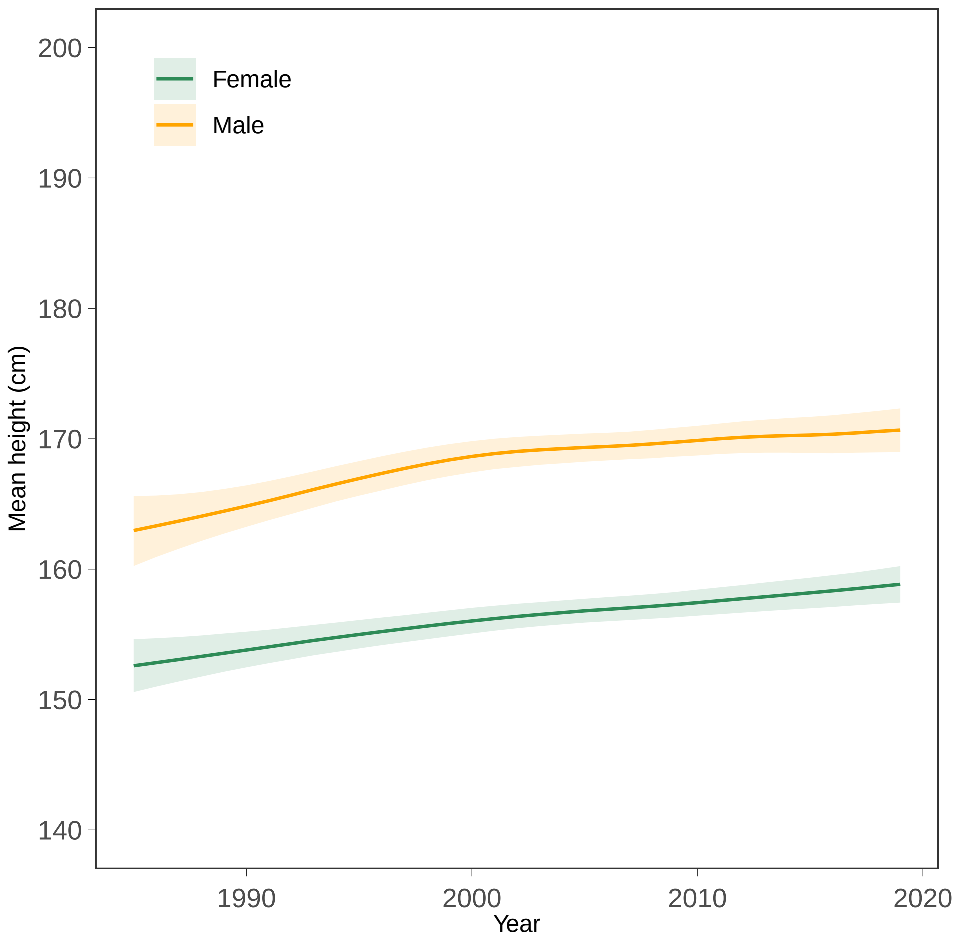

Time trends in BMI of 19 year olds

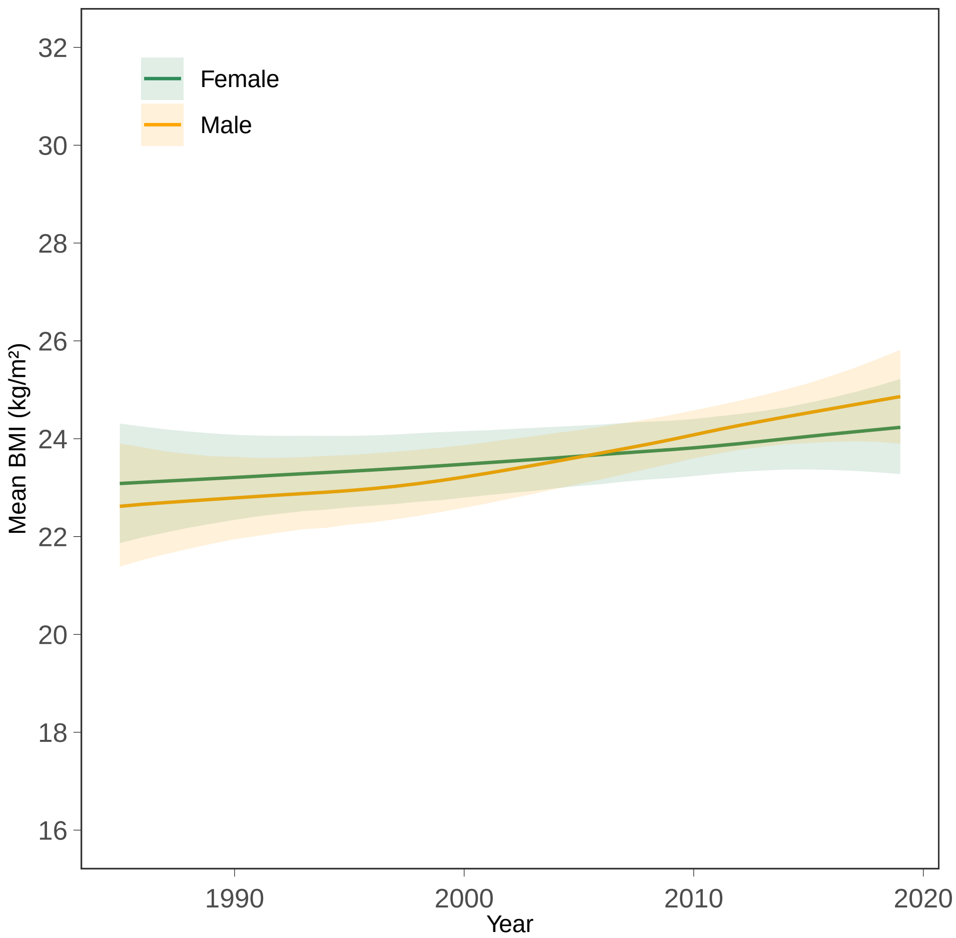

Height−for−age trajectories (2000 birth cohort)

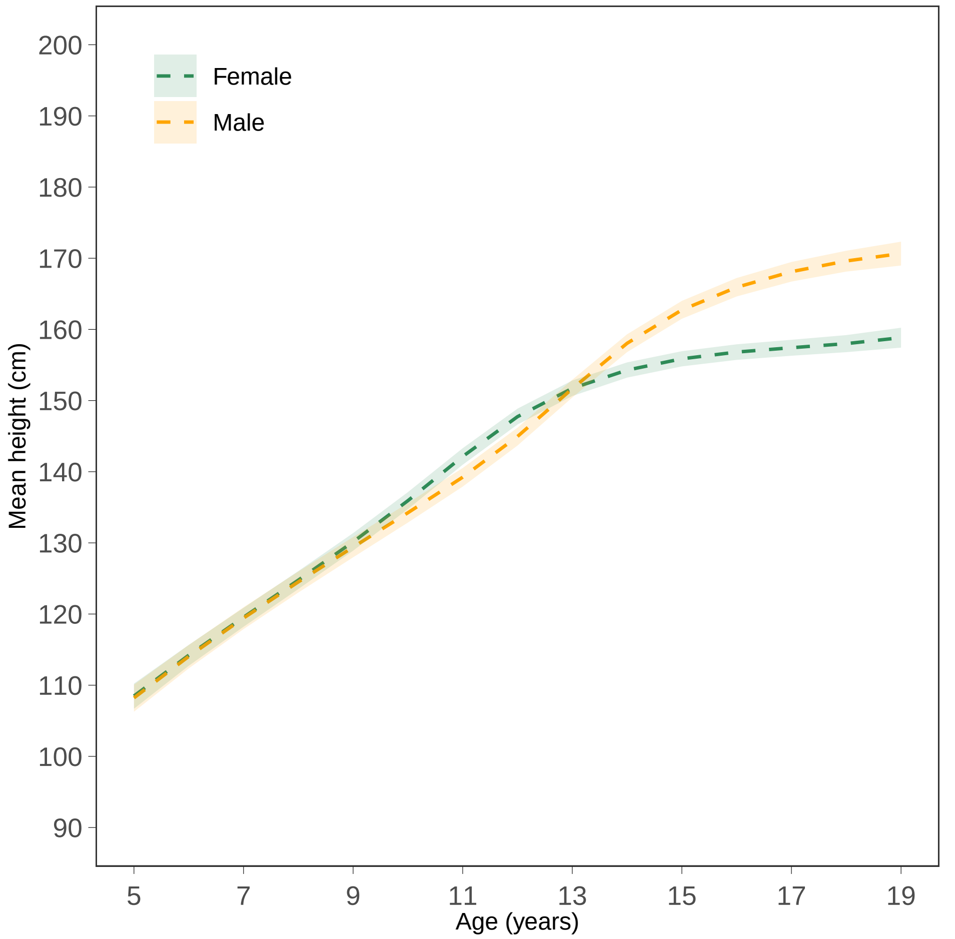

BMI−for−age trajectories (2000 birth cohort)

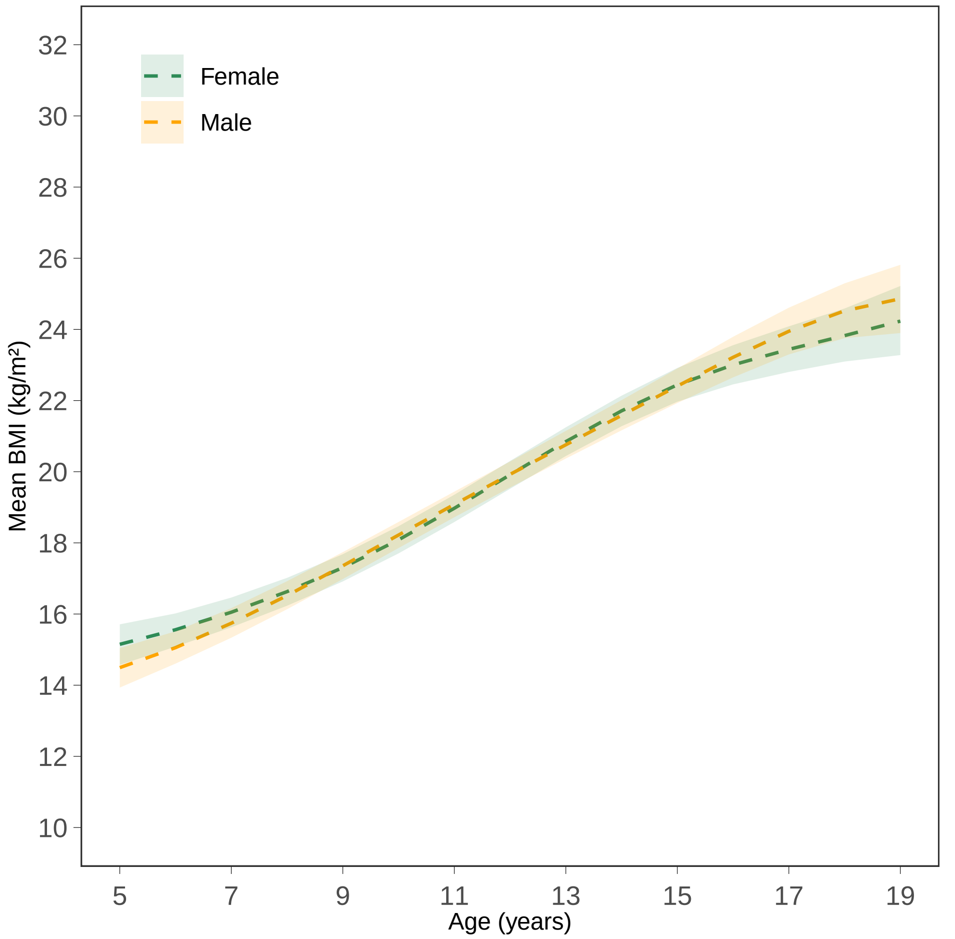

Senegal

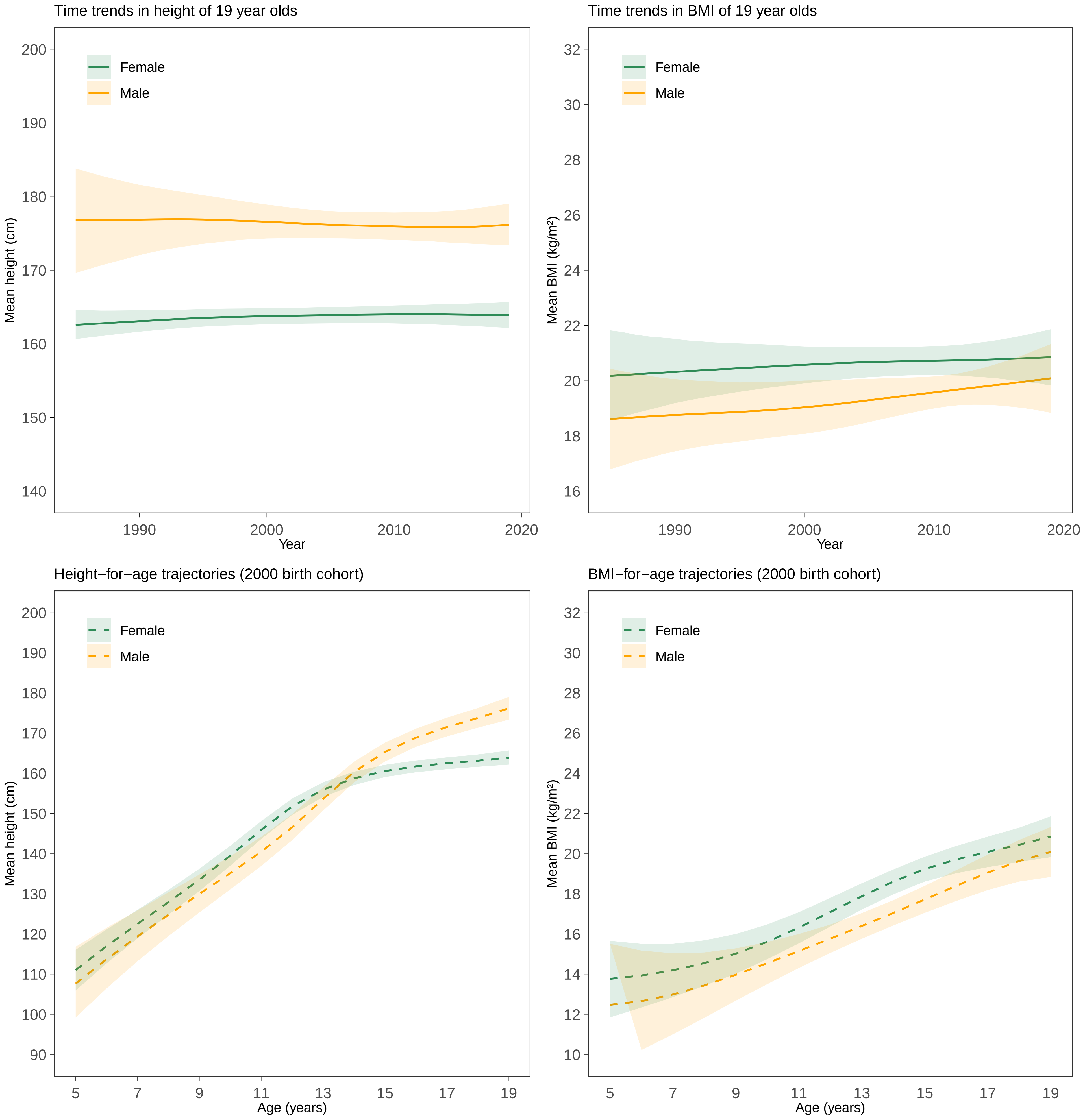

Time trends in height of 19 year olds
Female
Male
Mean height (cm)
200
190
180
170
160
150
140
1990
2000
2010
2020
Year
Time trends in BMI of 19 year olds
Female
Male
Mean BMI (kg/m²)
32
30
28
26
24
22
20
18
16
1990
2000
2010
2020
Year
Height−for−age trajectories (2000 birth cohort)
Female
Male
Mean height (cm)
200
190
180
170
160
150
140
130
120
110
100
90
5
7
9
11
13
15
17
19
Age (years)
BMI−for−age trajectories (2000 birth cohort)
Female
Male
Mean BMI (kg/m²)
32
30
28
26
24
22
20
18
16
14
12
10
5
7
9
11
13
15
17
19
Age (years)

Serbia

Time trends in height of 19 year olds

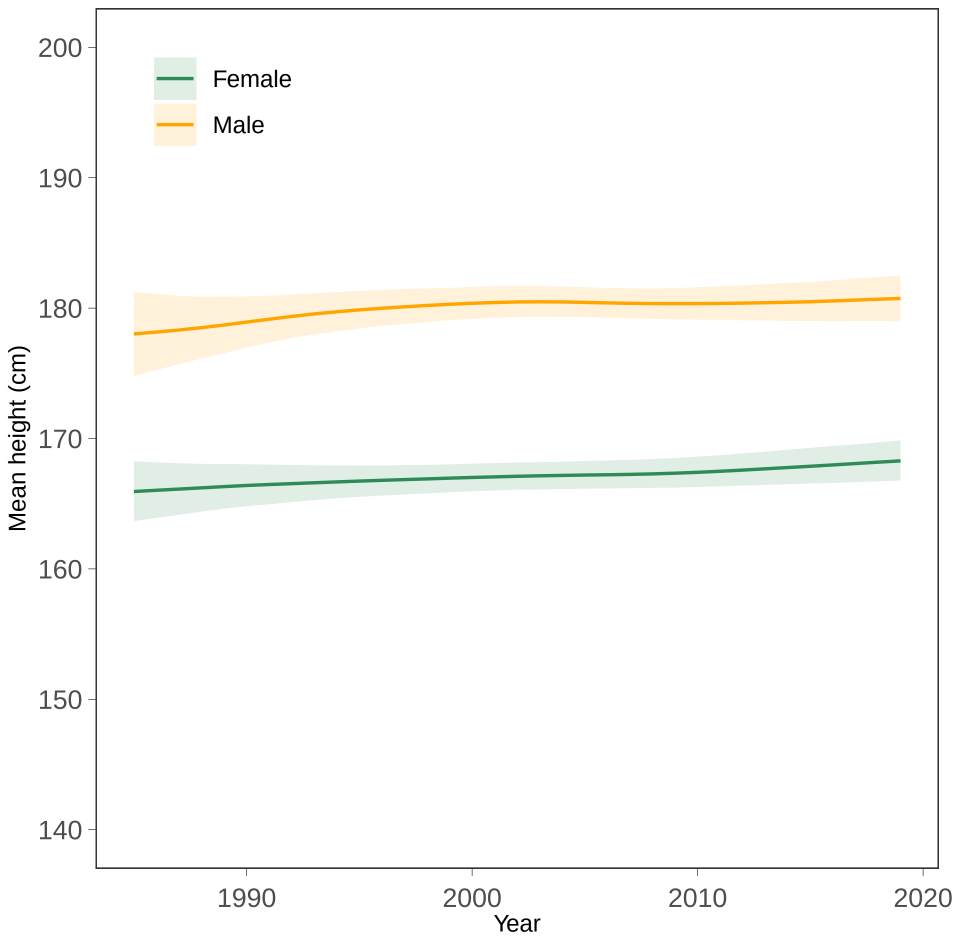

Time trends in BMI of 19 year olds

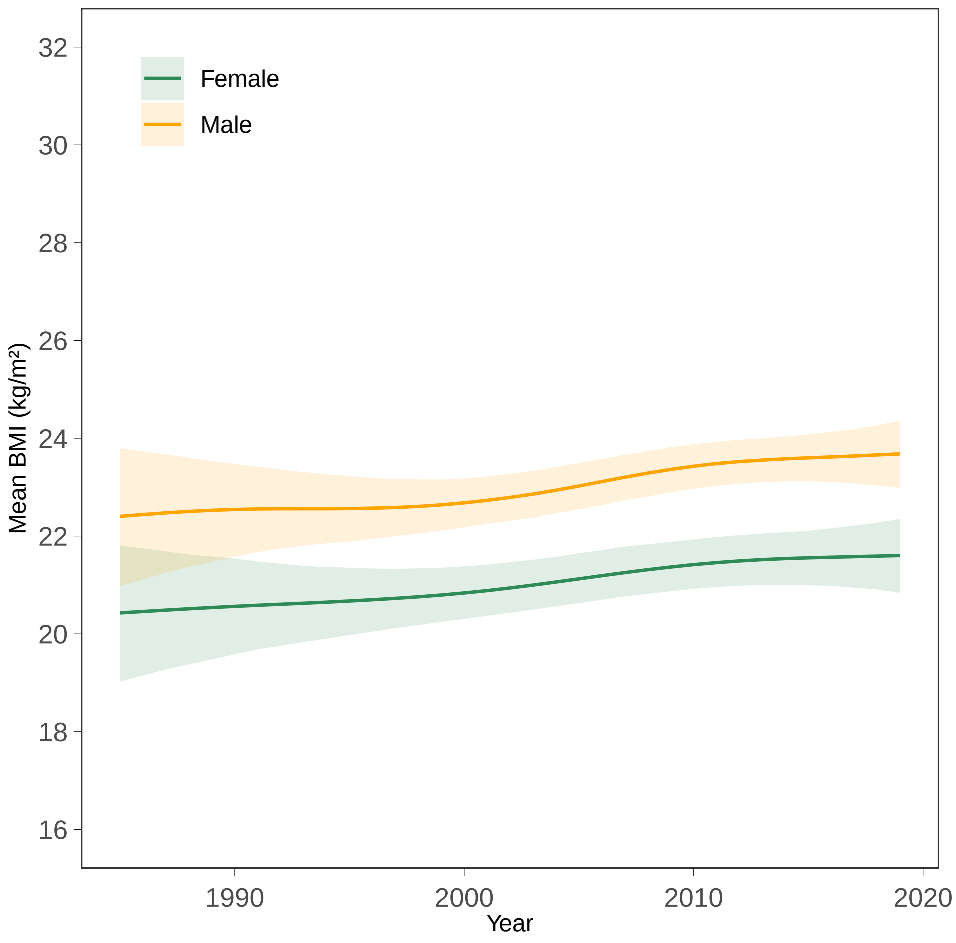

Height−for−age trajectories (2000 birth cohort)

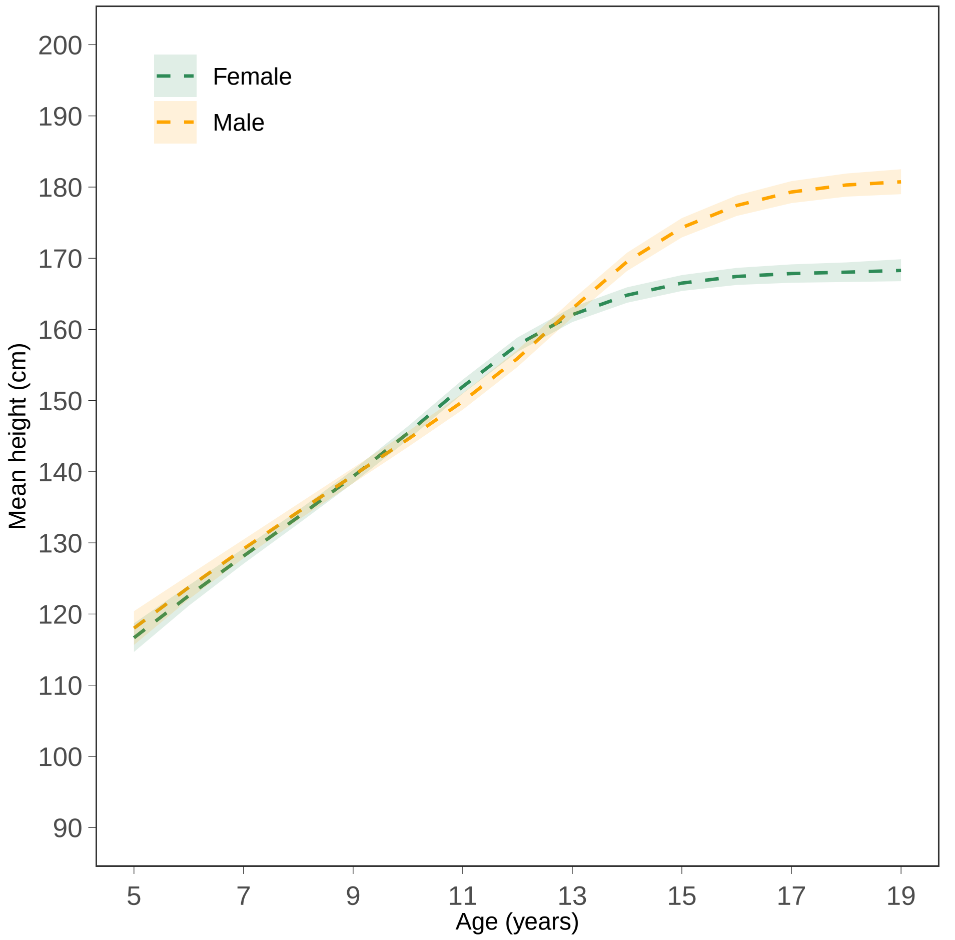

BMI−for−age trajectories (2000 birth cohort)

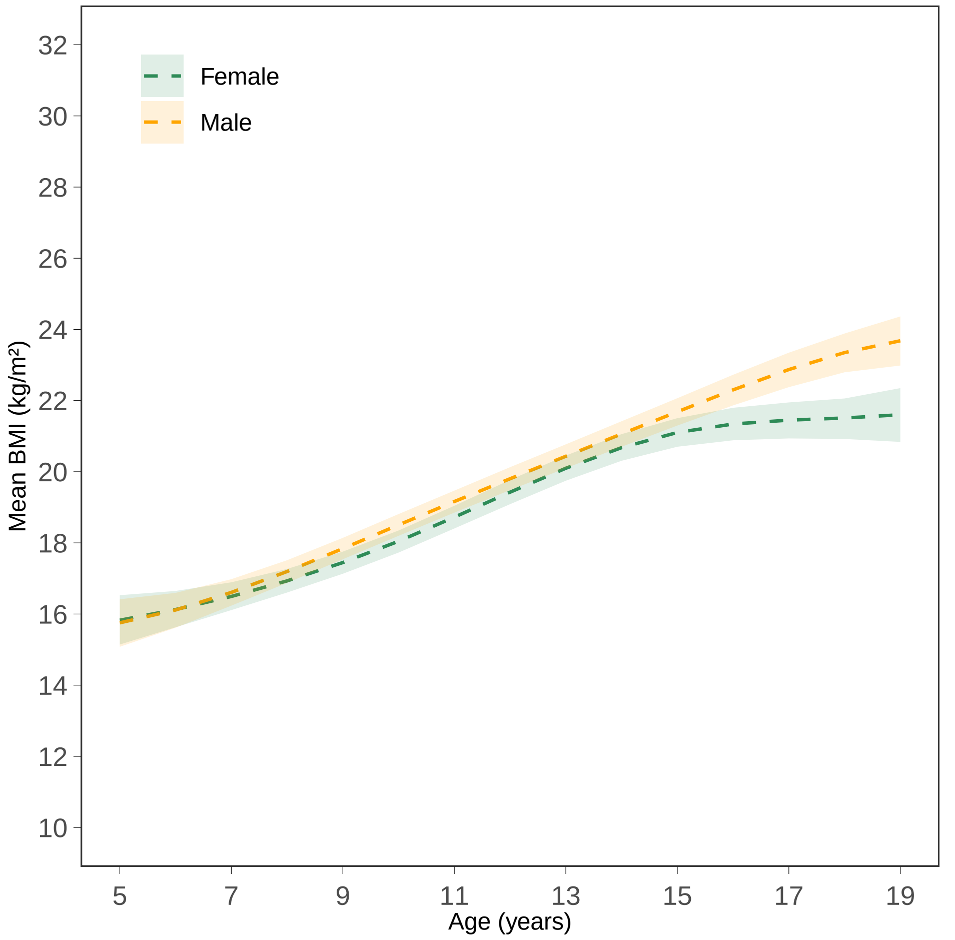

Seychelles

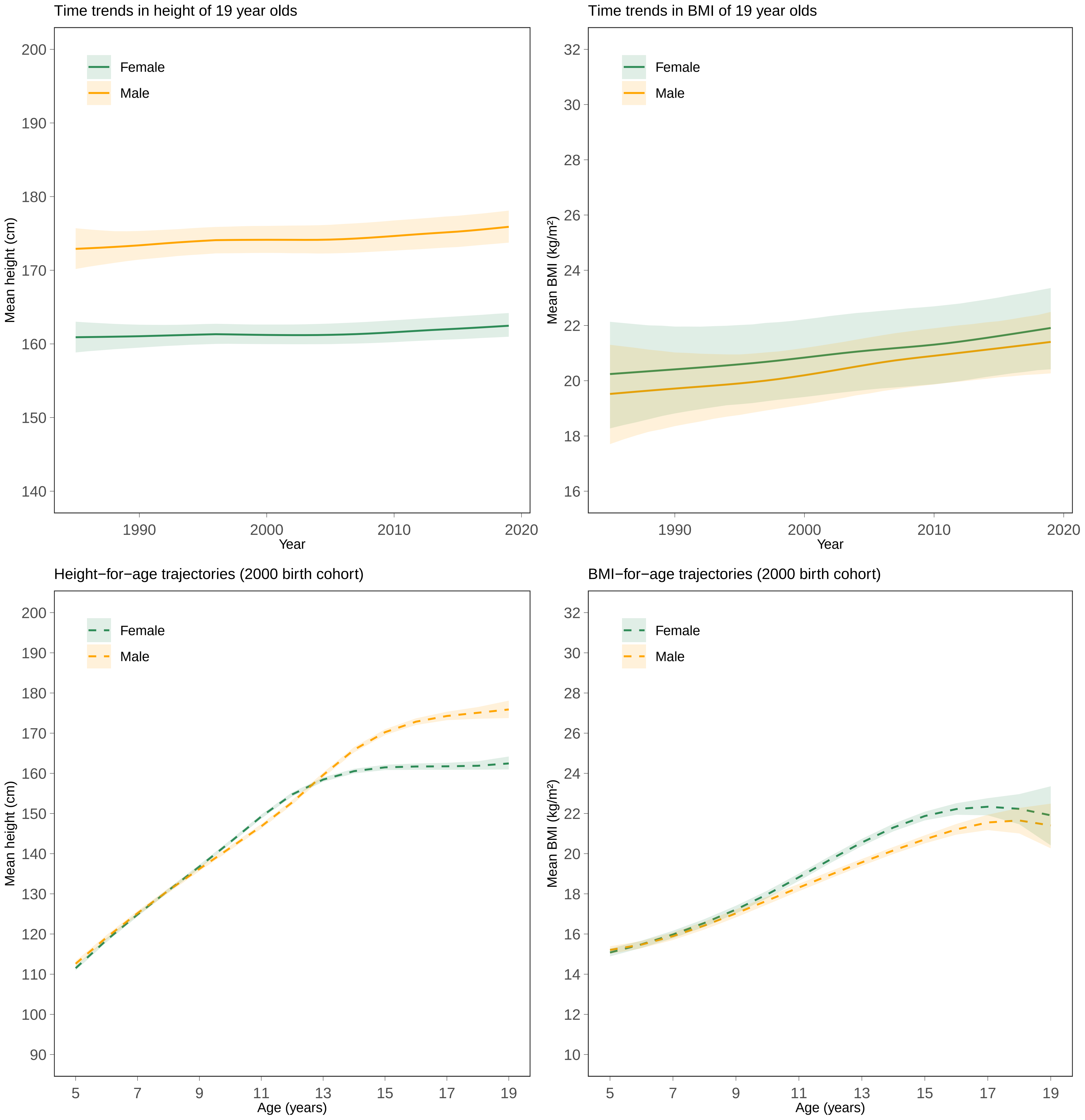
Time trends in height of 19 year olds
Female
Male
Mean height (cm)
200
190
180
170
160
150
140
1990
2000
2010
2020
Year
Time trends in BMI of 19 year olds
Female
Male
Mean BMI (kg/m²)
32
30
28
26
24
22
20
18
16
1990
2000
2010
2020
Year
Height−for−age trajectories (2000 birth cohort)
Female
Male
Mean height (cm)
200
190
180
170
160
150
140
130
120
110
100
90
5
7
9
11
13
15
17
19
Age (years)
BMI−for−age trajectories (2000 birth cohort)
Female
Male
Mean BMI (kg/m²)
32
30
28
26
24
22
20
18
16
14
12
10
5
7
9
11
13
15
17
19
Age (years)

Sierra Leone

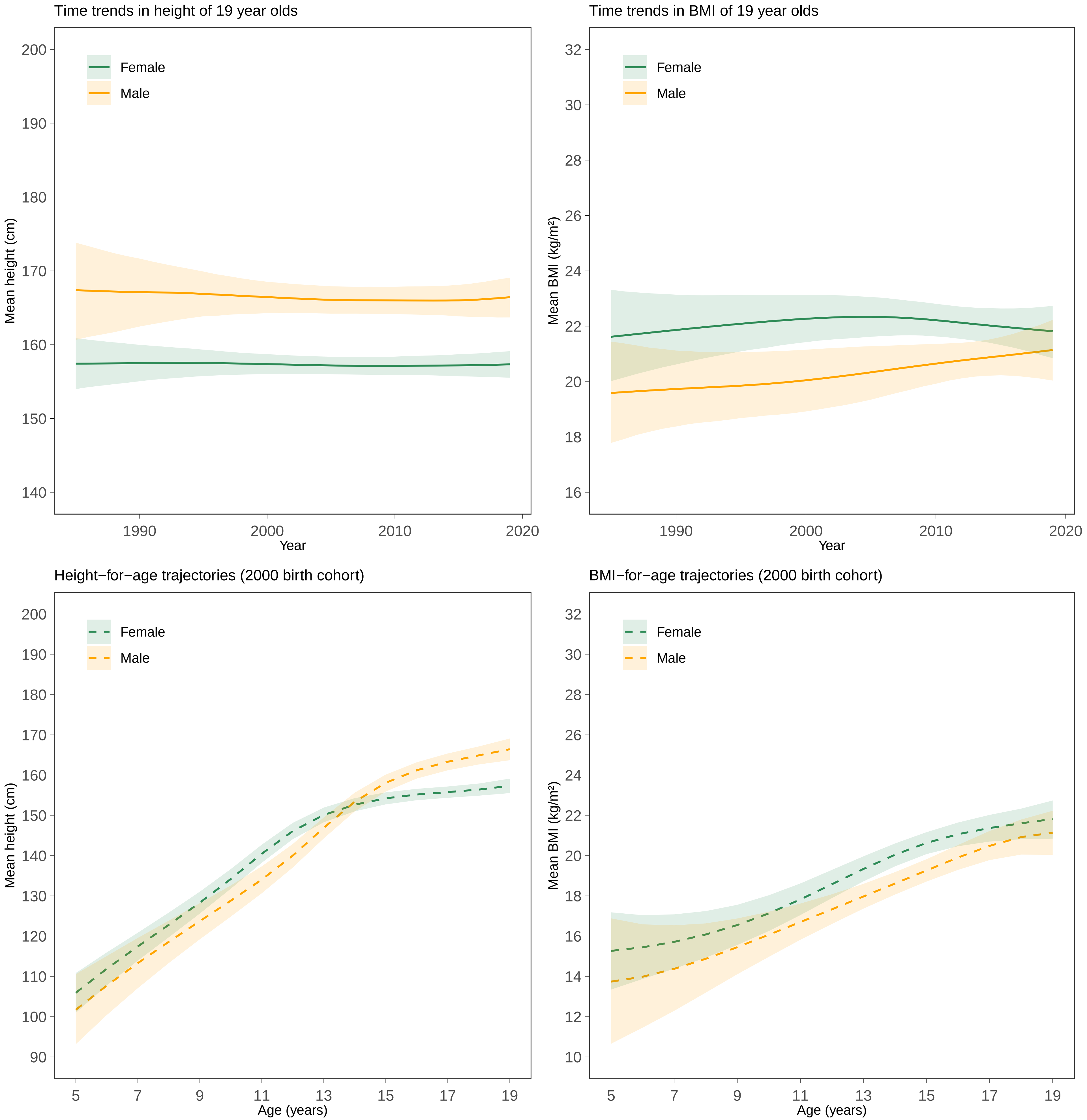

Singapore

Time trends in height of 19 year olds

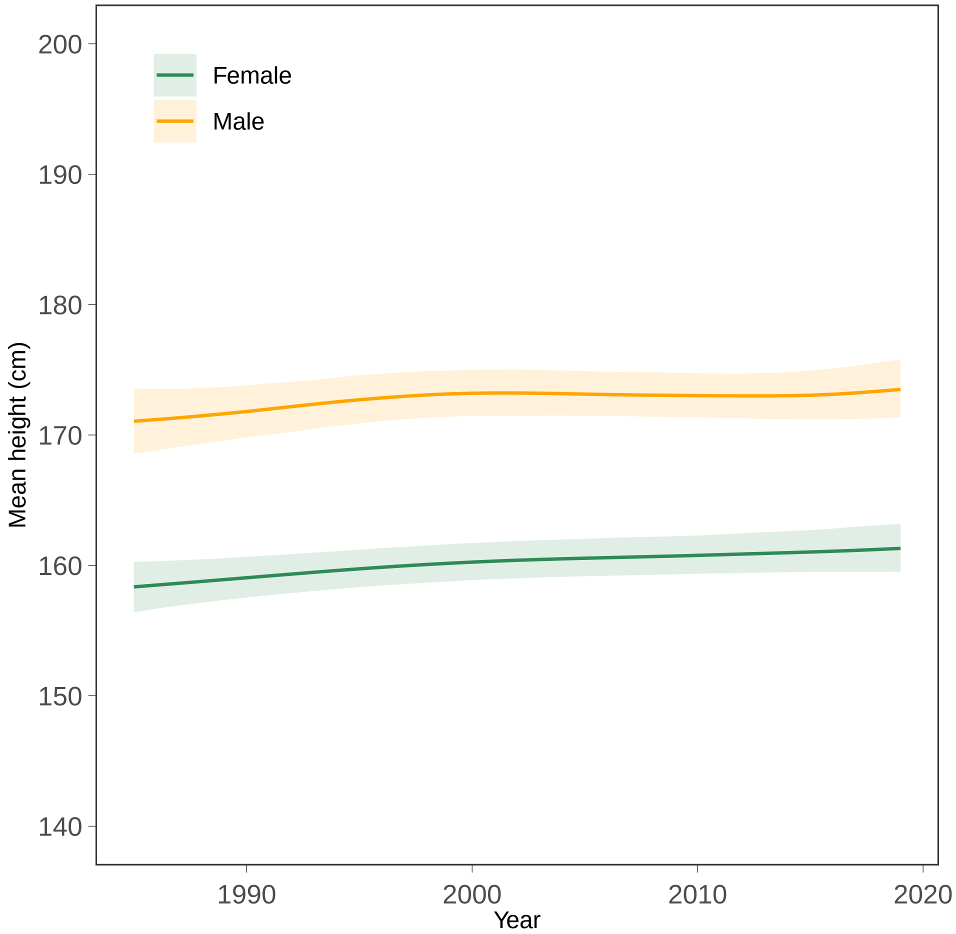

Time trends in BMI of 19 year olds

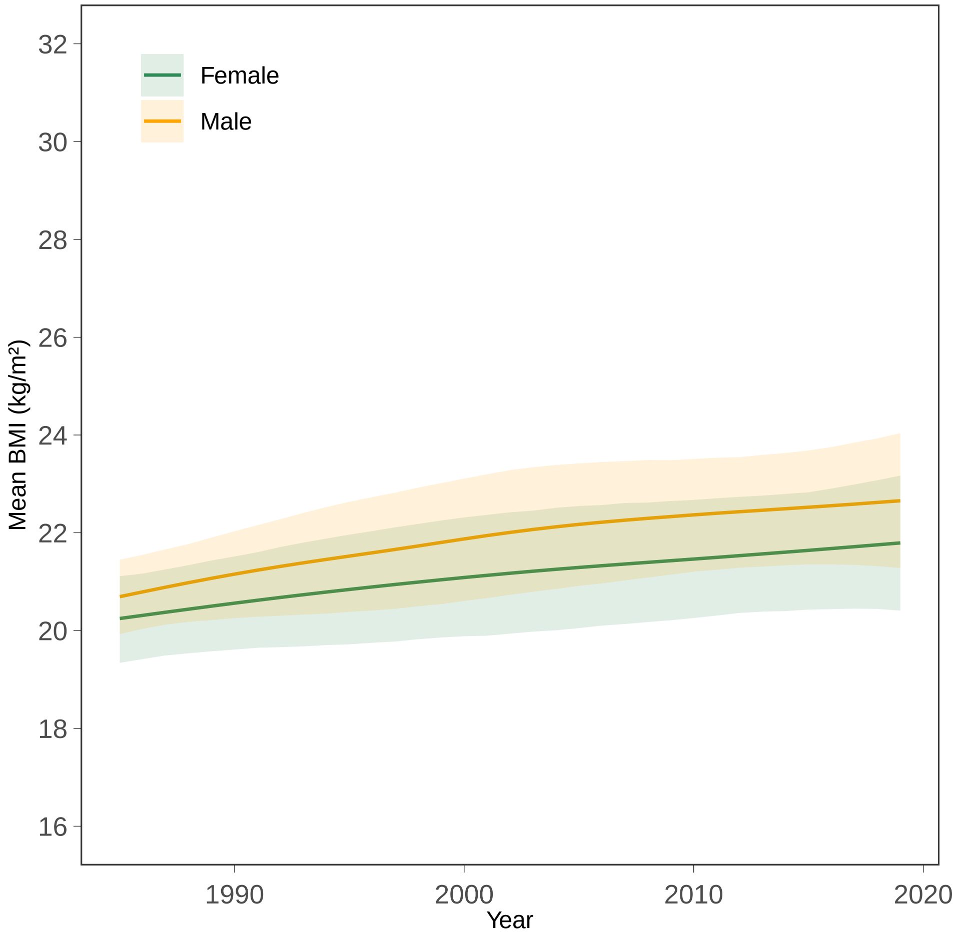

Height−for−age trajectories (2000 birth cohort)

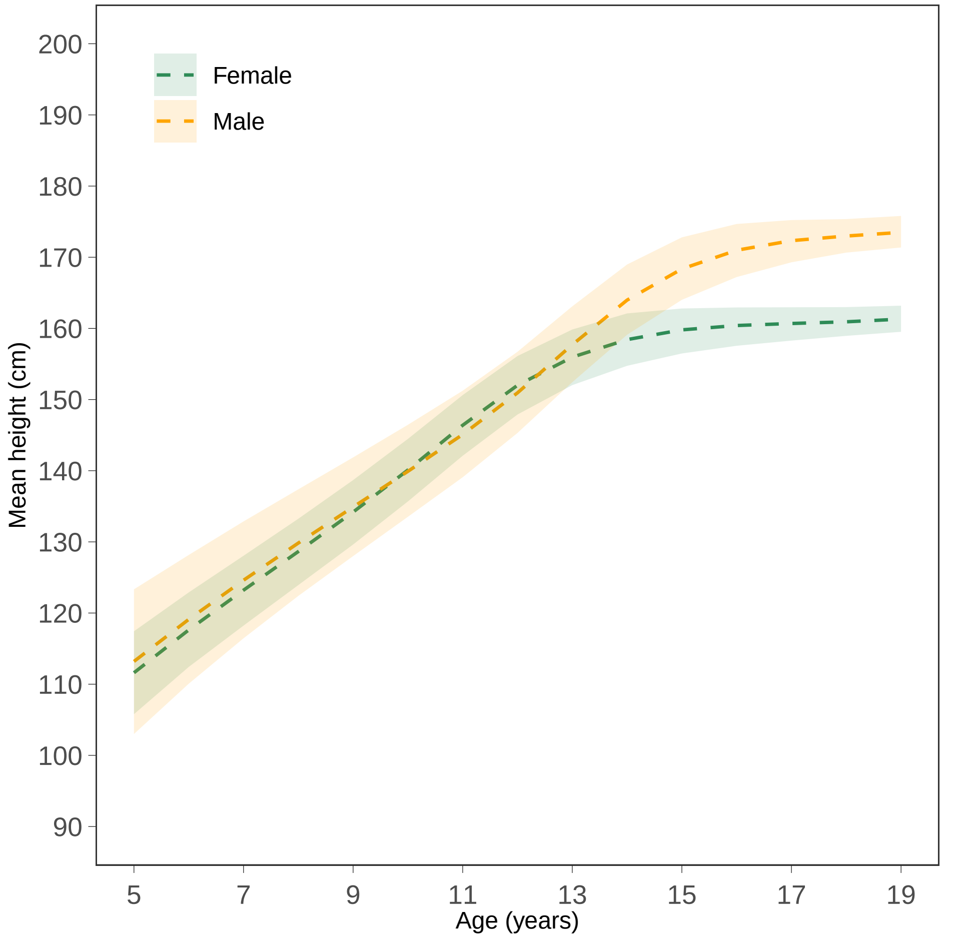

BMI−for−age trajectories (2000 birth cohort)

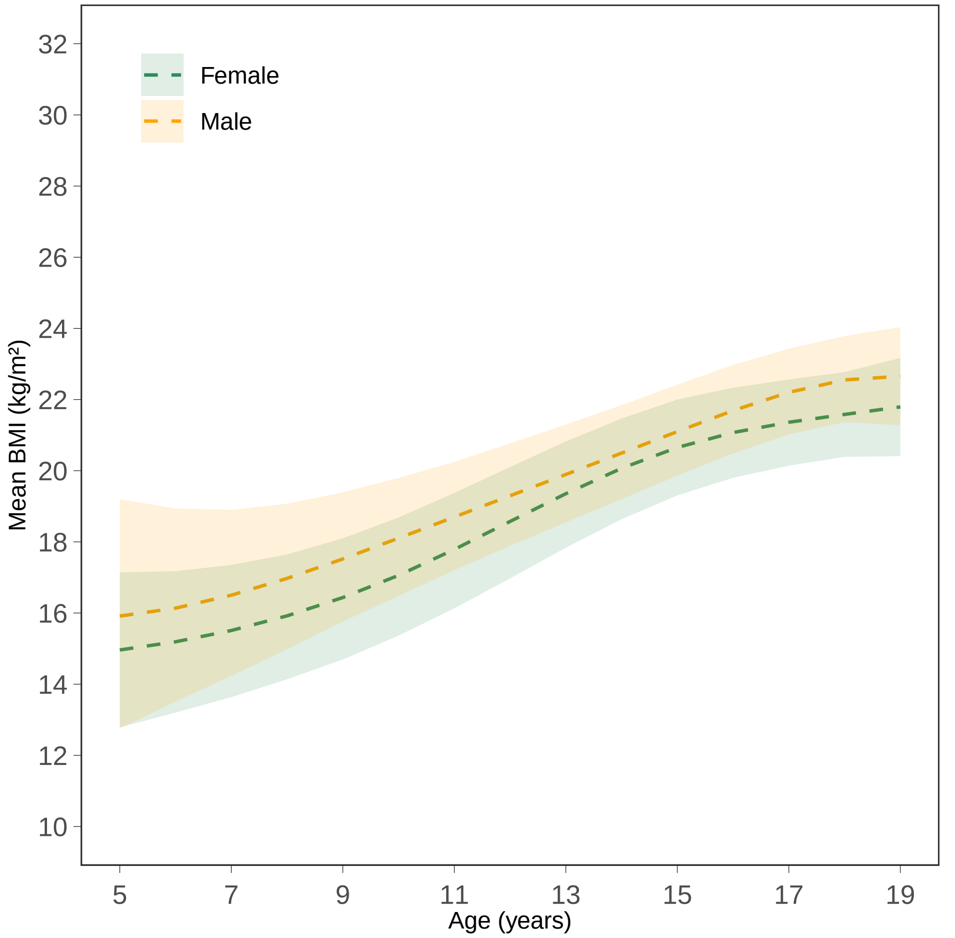

Slovakia

Time trends in height of 19 year olds

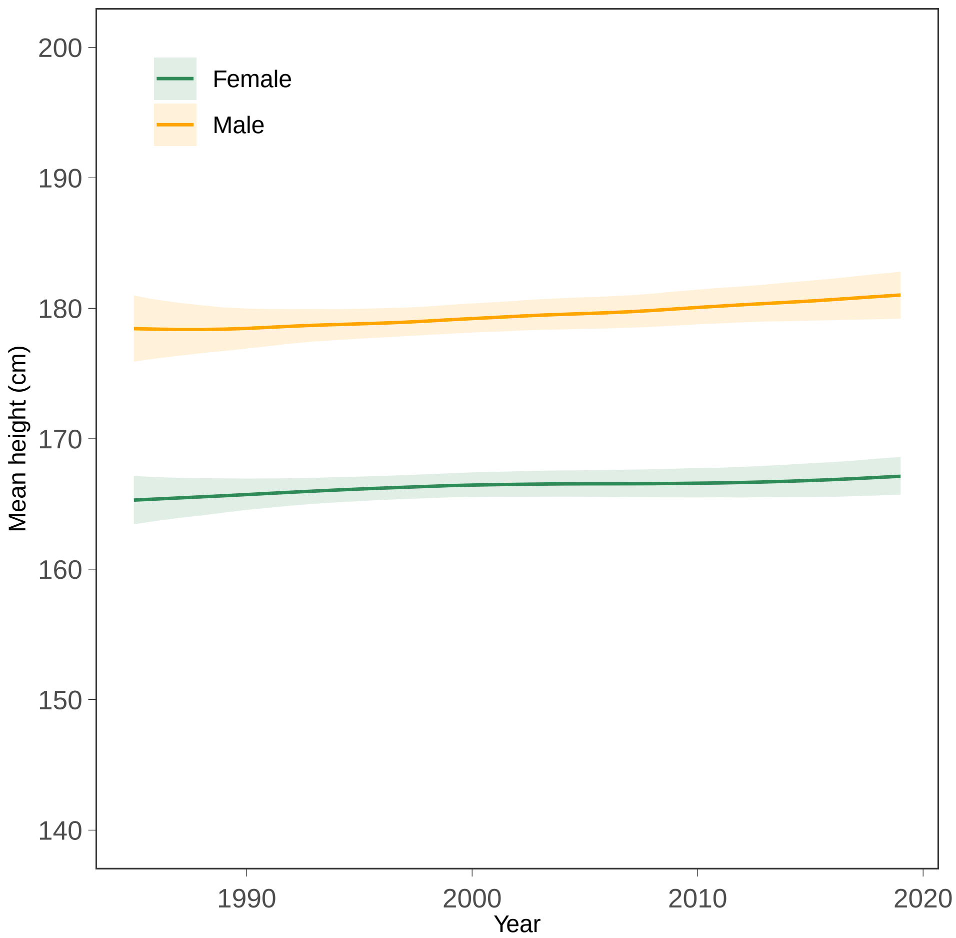

Time trends in BMI of 19 year olds

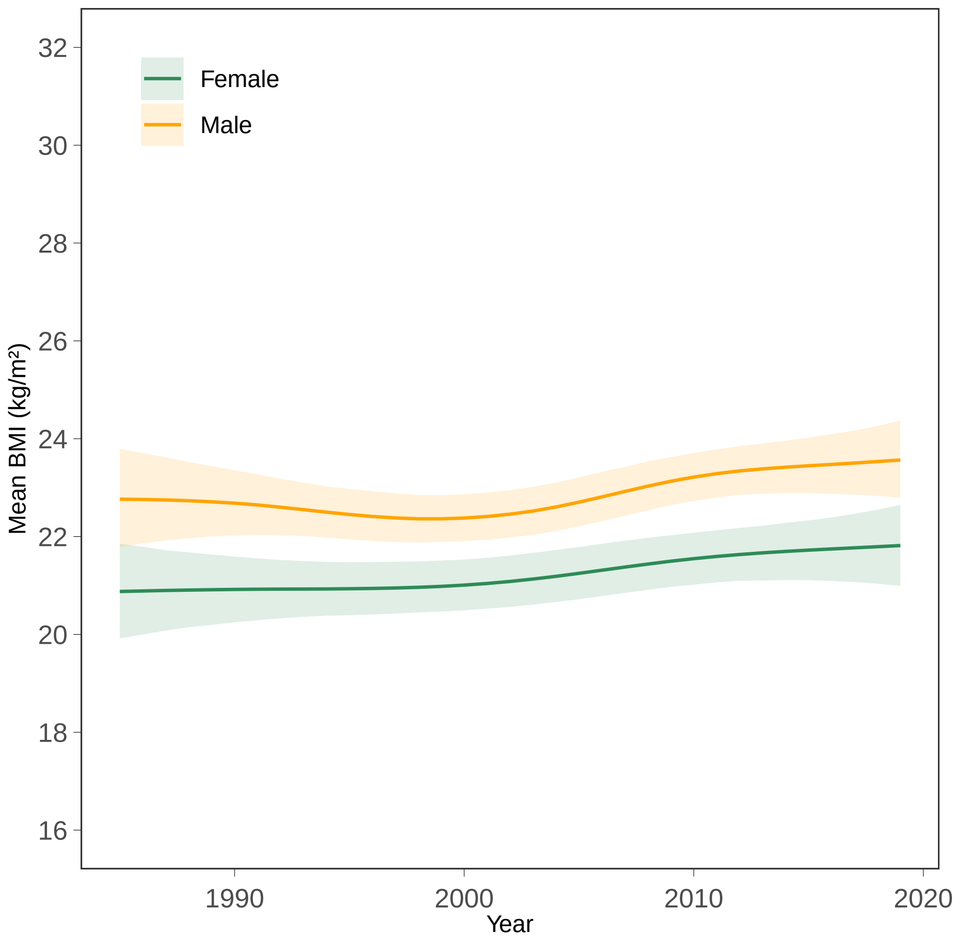

Height−for−age trajectories (2000 birth cohort)

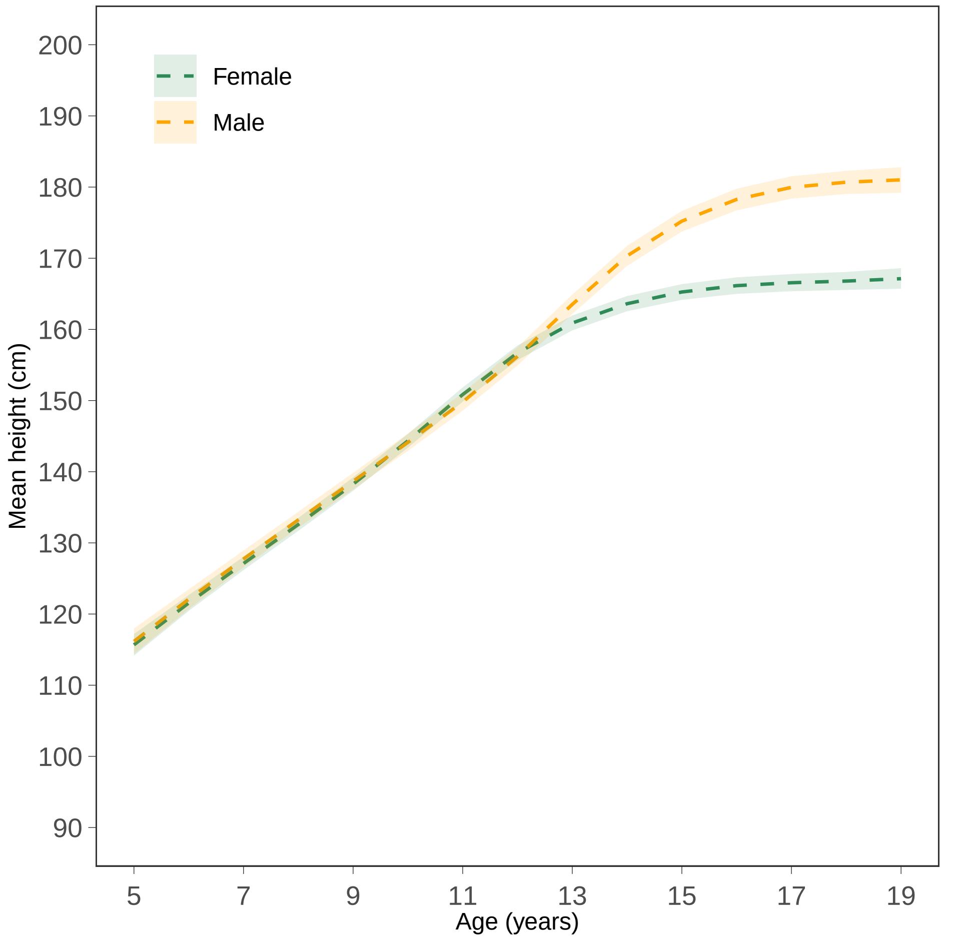

BMI−for−age trajectories (2000 birth cohort)

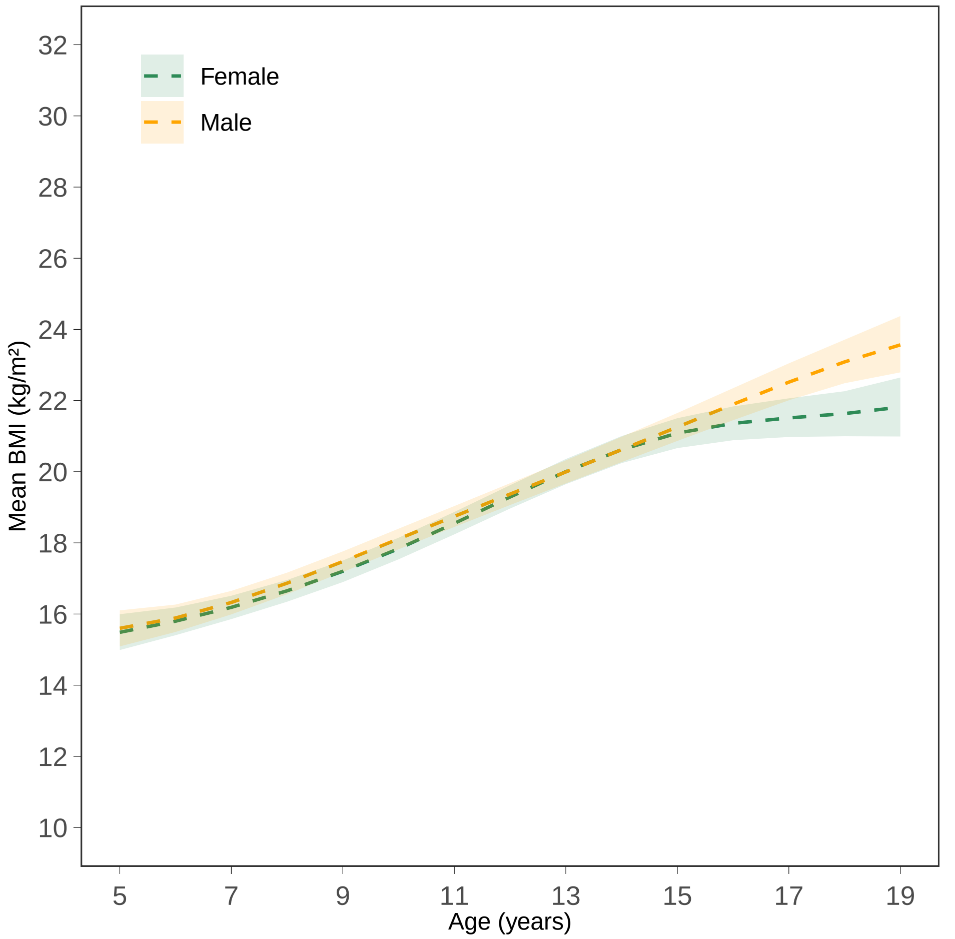

Slovenia

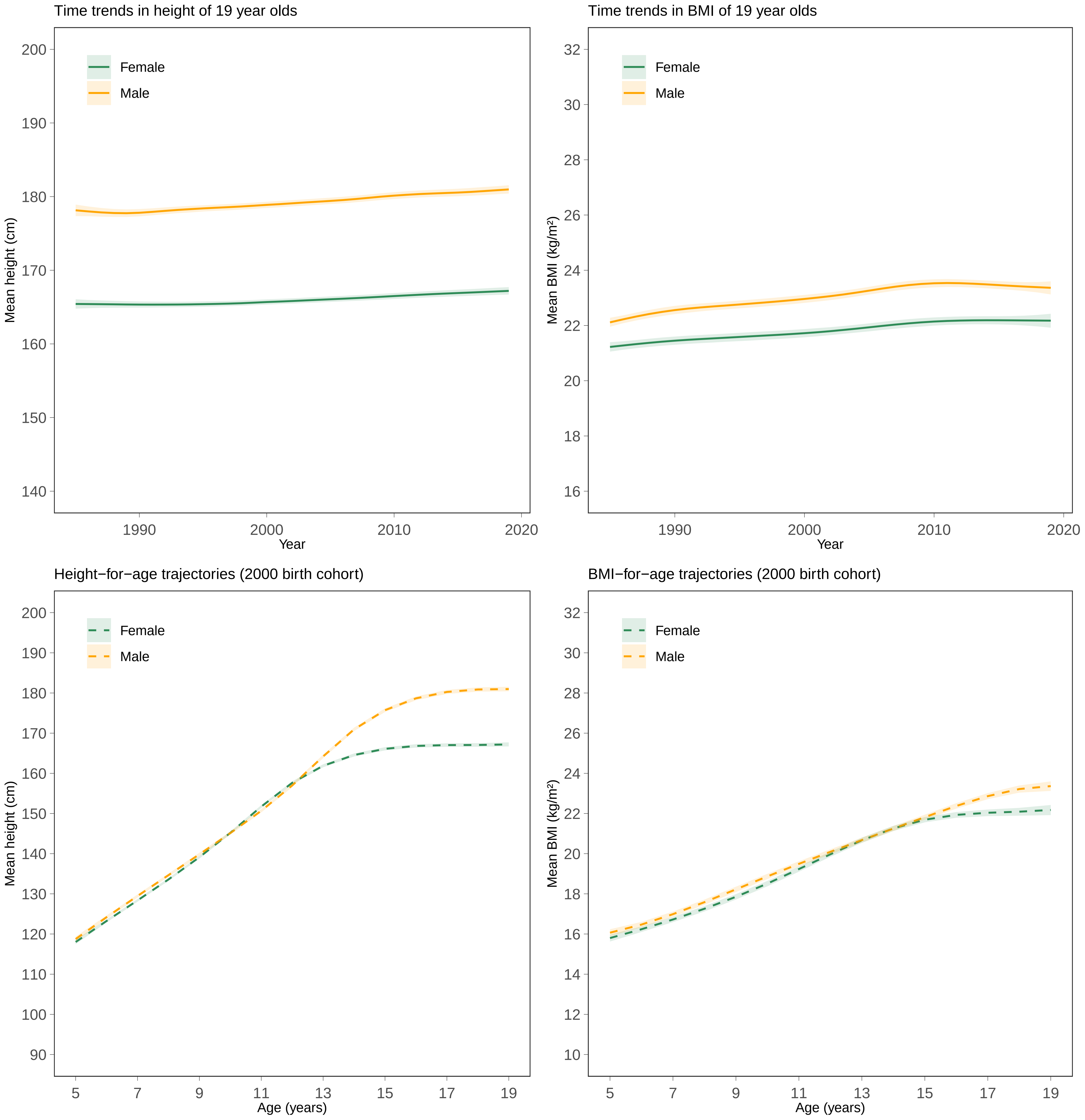

Time trends in height of 19 year olds
Female
Male
Mean height (cm)
Year
Time trends in BMI of 19 year olds
Female
Male
Mean BMI (kg/m²)
Year
Height−for−age trajectories (2000 birth cohort)
Female
Male
Mean height (cm)
Age (years)
BMI−for−age trajectories (2000 birth cohort)
Female
Male
Mean BMI (kg/m²)
Age (years)

Solomon Islands

Time trends in height of 19 year olds

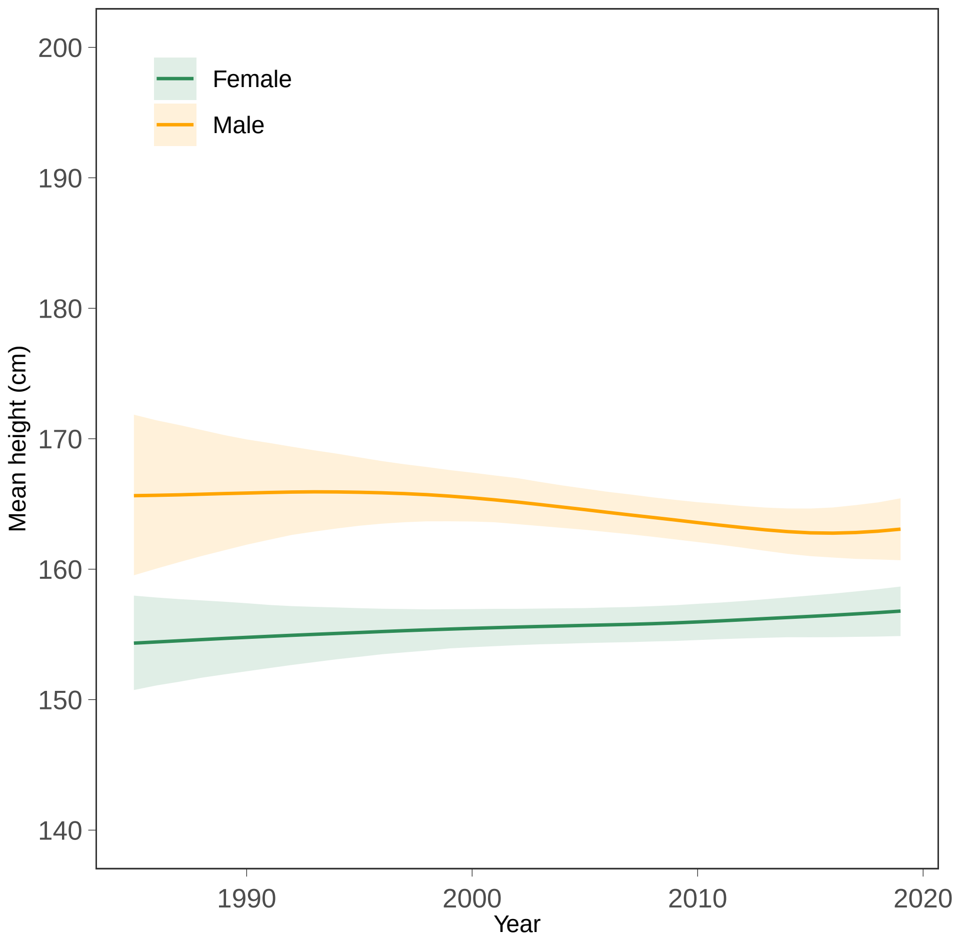

Time trends in BMI of 19 year olds

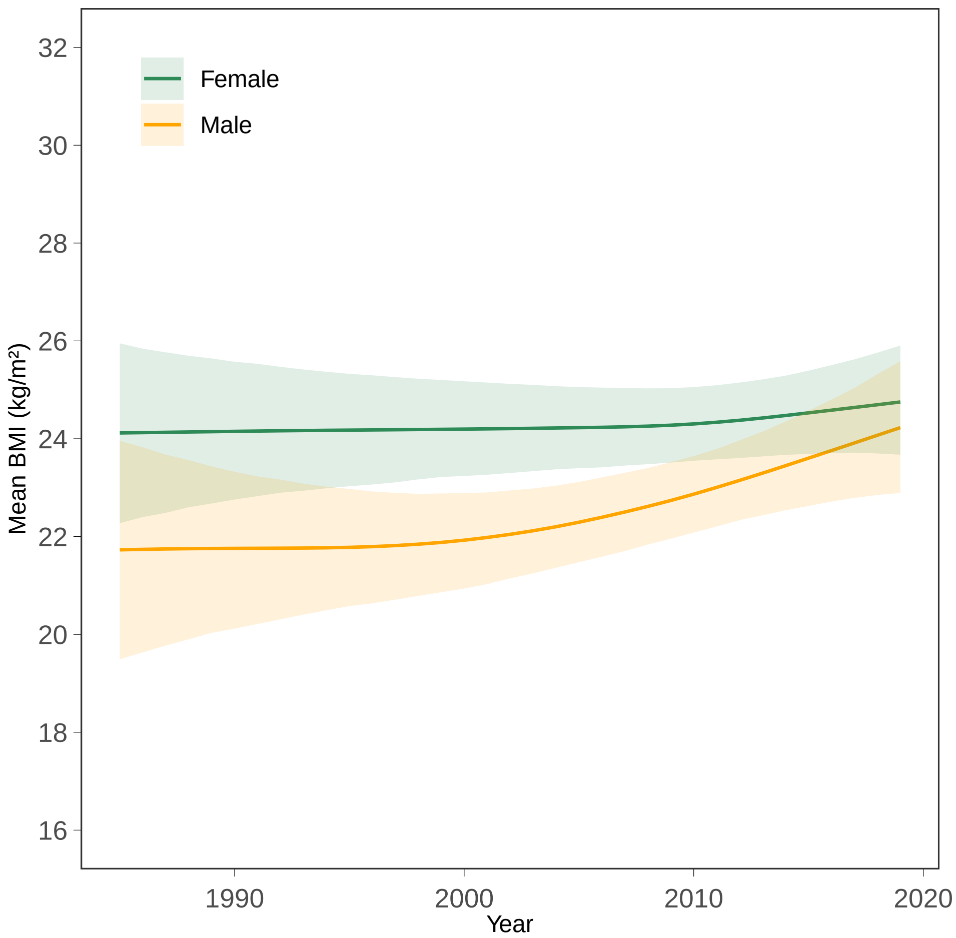

Height−for−age trajectories (2000 birth cohort)

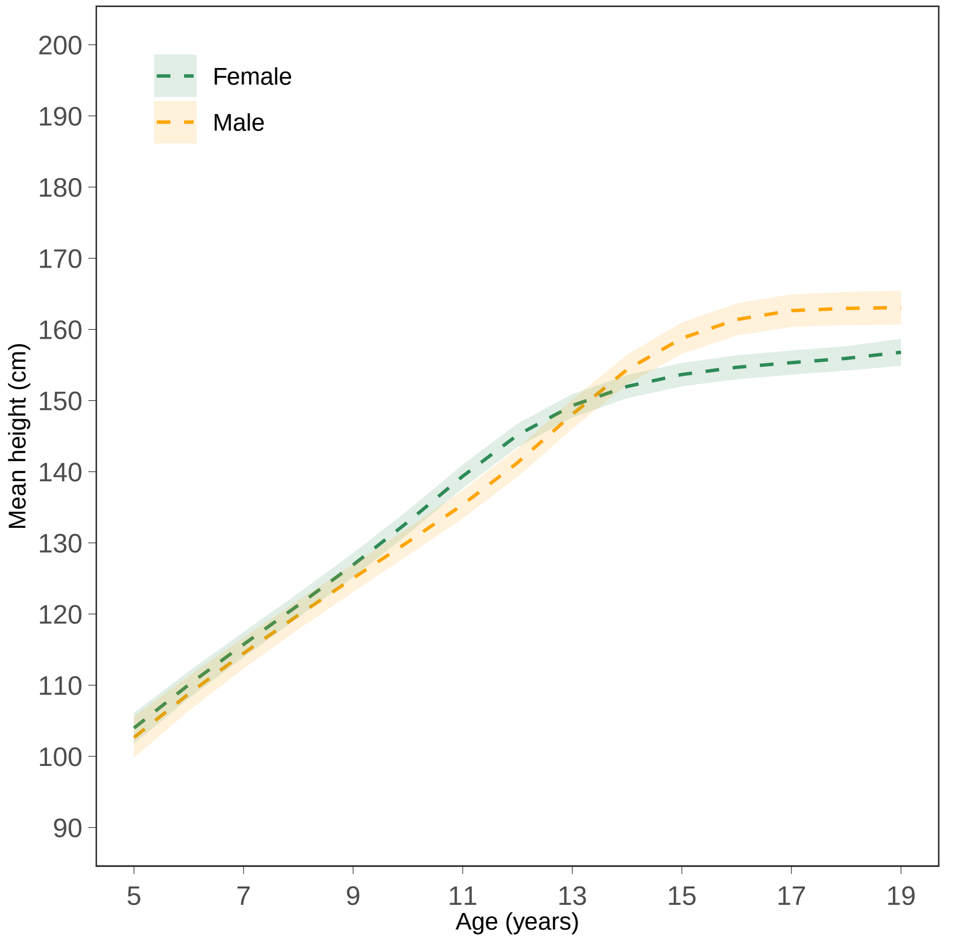

BMI−for−age trajectories (2000 birth cohort)

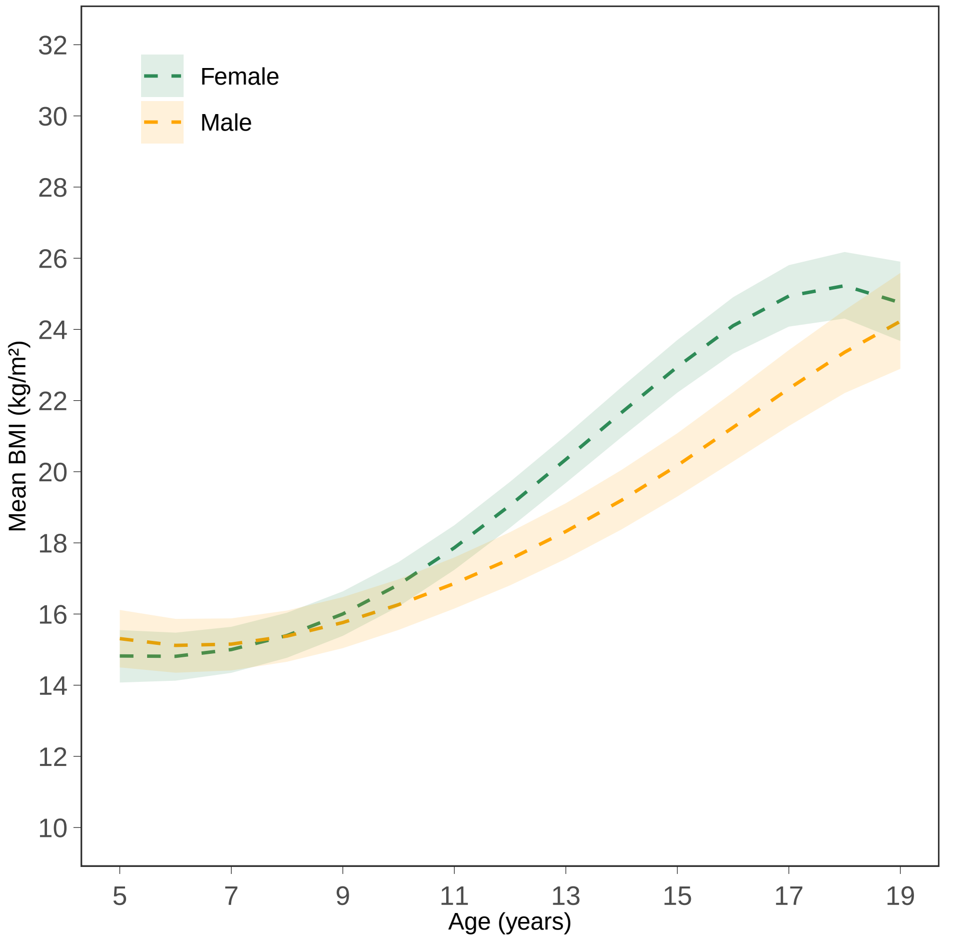

Somalia

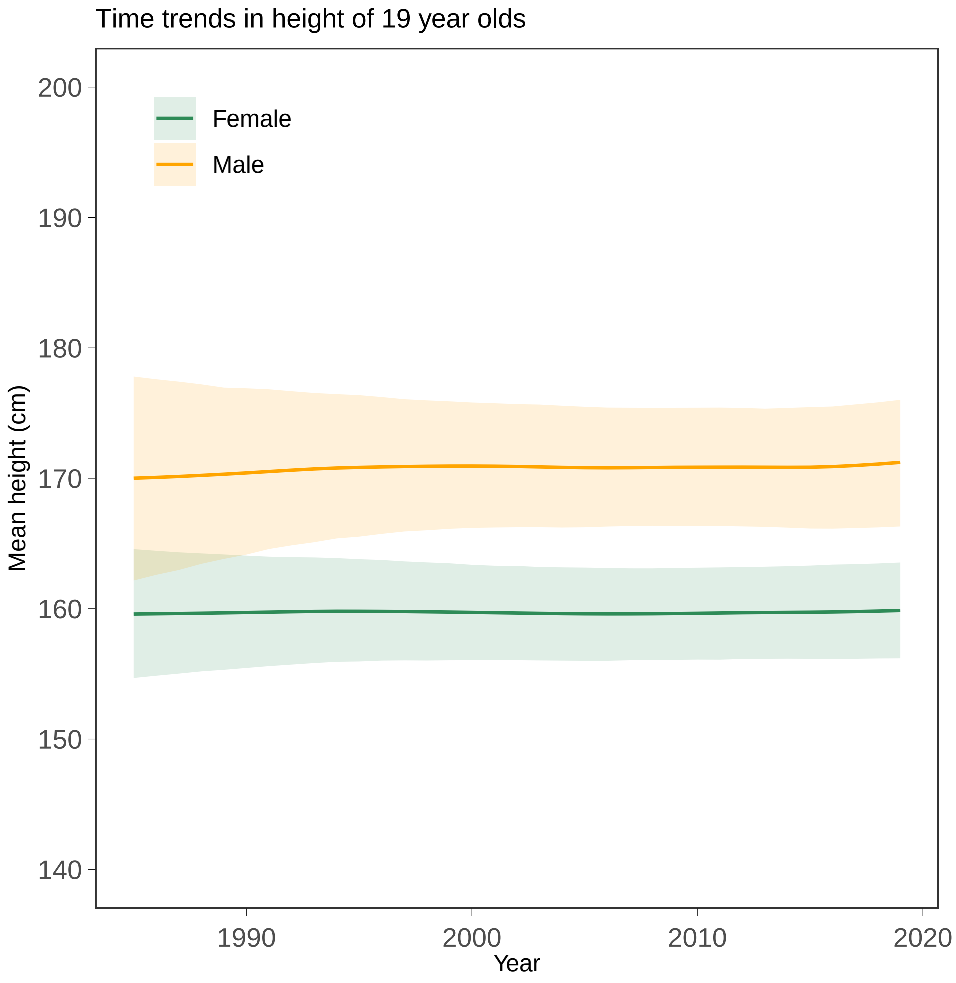
Time trends in height of 19 year olds
Female
Male
Mean height (cm)
Year
140
150
160
170
180
190
200
1990
2000
2010
2020

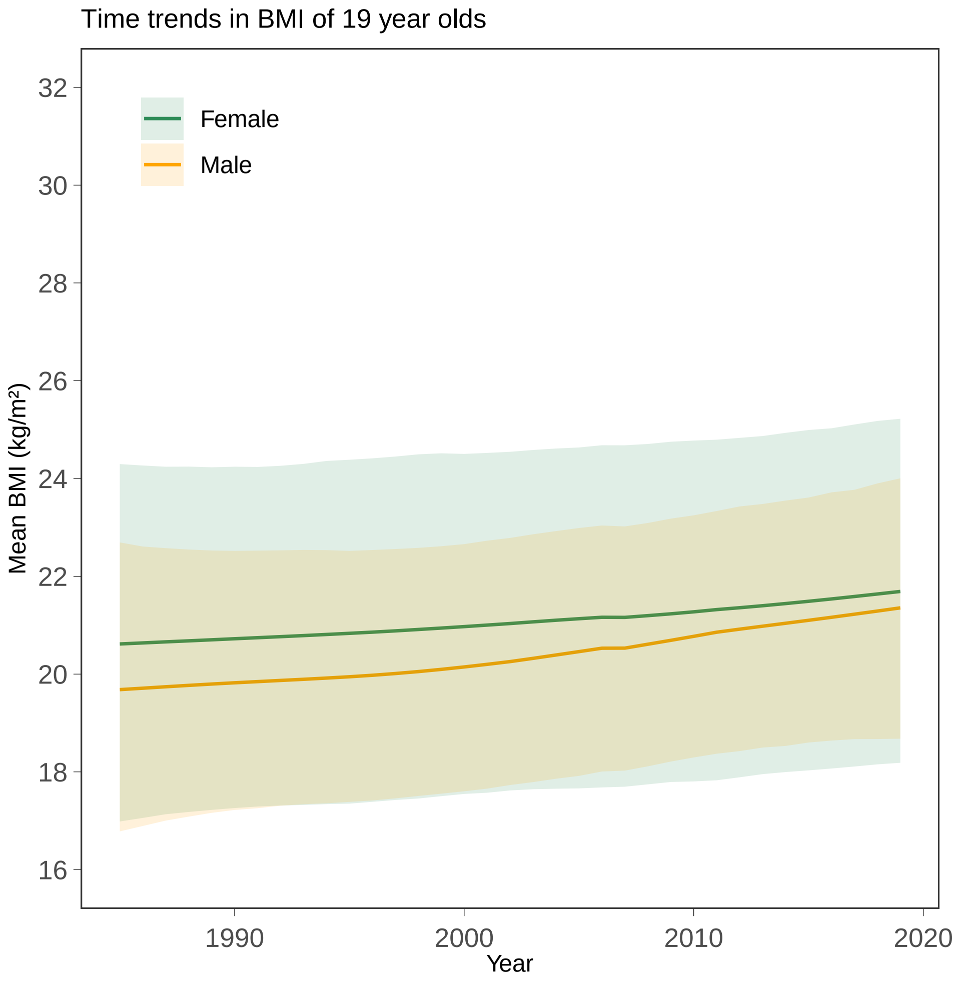
Time trends in BMI of 19 year olds
Female
Male
Mean BMI (kg/m²)
Year
16
18
20
22
24
26
28
30
32
1990
2000
2010
2020

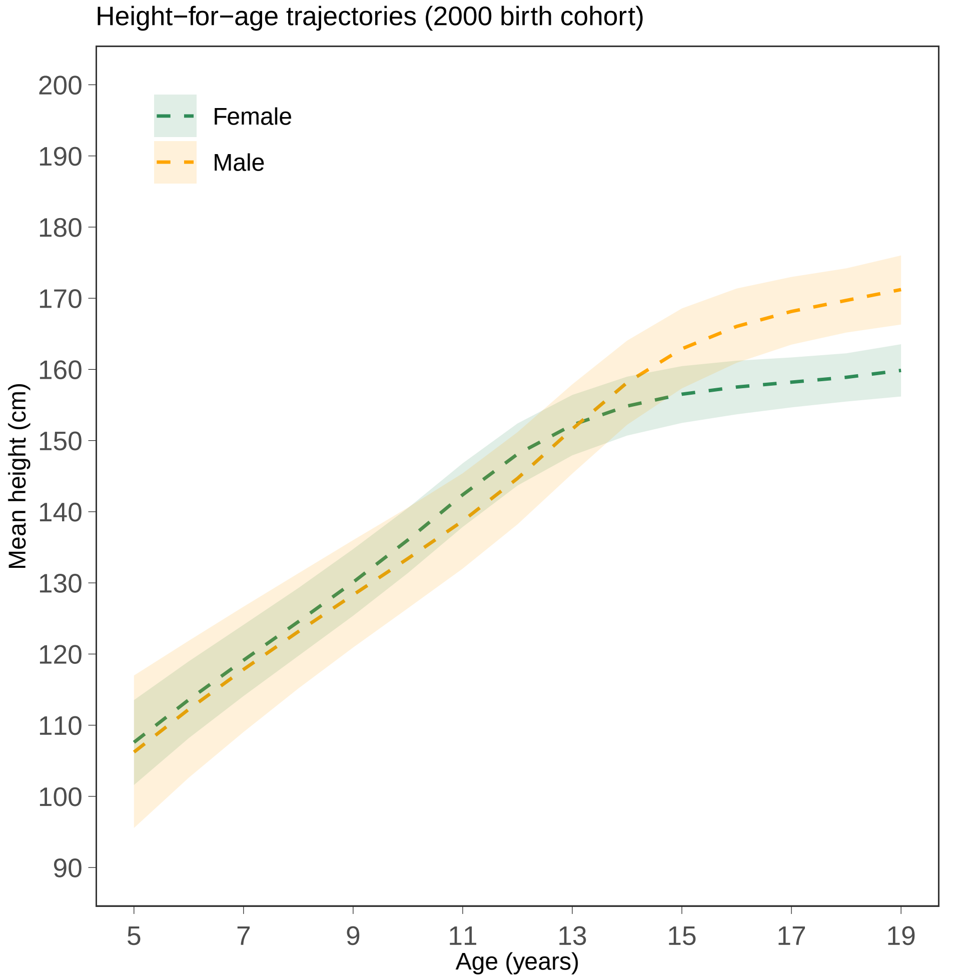
Height−for−age trajectories (2000 birth cohort)
Female
Male
Mean height (cm)
Age (years)
90
100
110
120
130
140
150
160
170
180
190
200
5
7
9
11
13
15
17
19

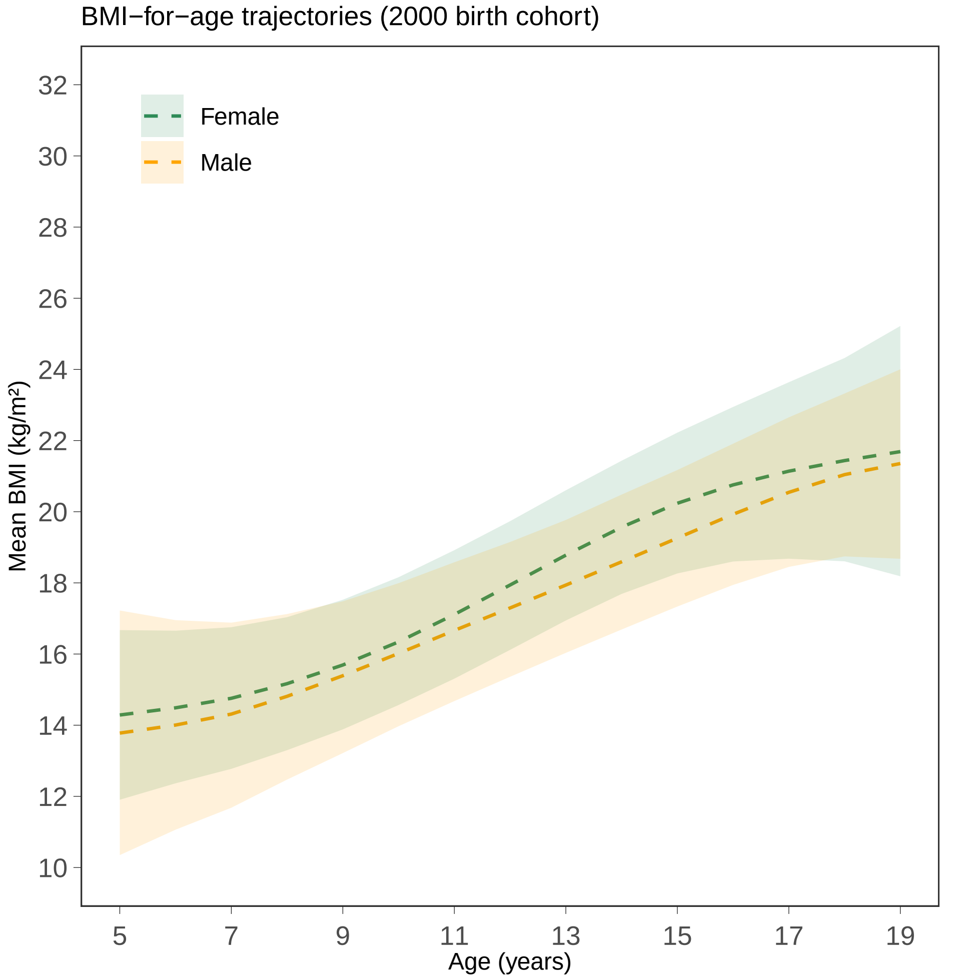
BMI−for−age trajectories (2000 birth cohort)
Female
Male
Mean BMI (kg/m²)
Age (years)
10
12
14
16
18
20
22
24
26
28
30
32
5
7
9
11
13
15
17
19

South Africa

Time trends in height of 19 year olds

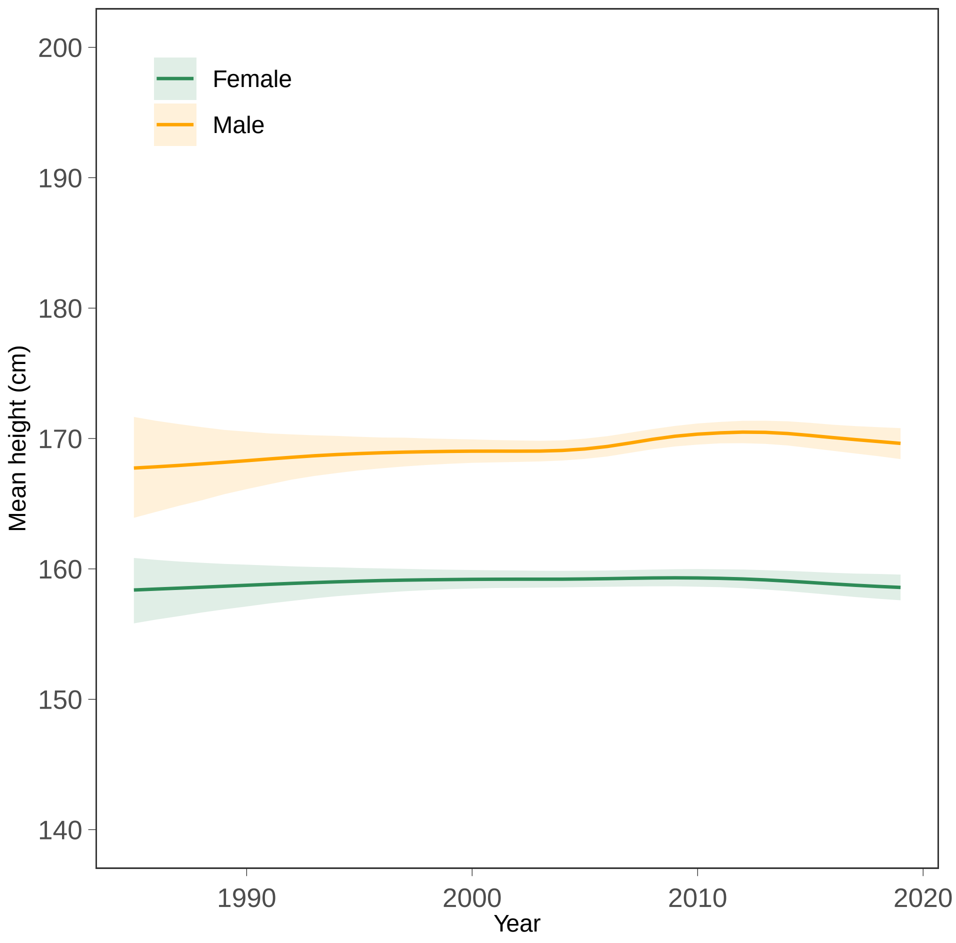

Time trends in BMI of 19 year olds

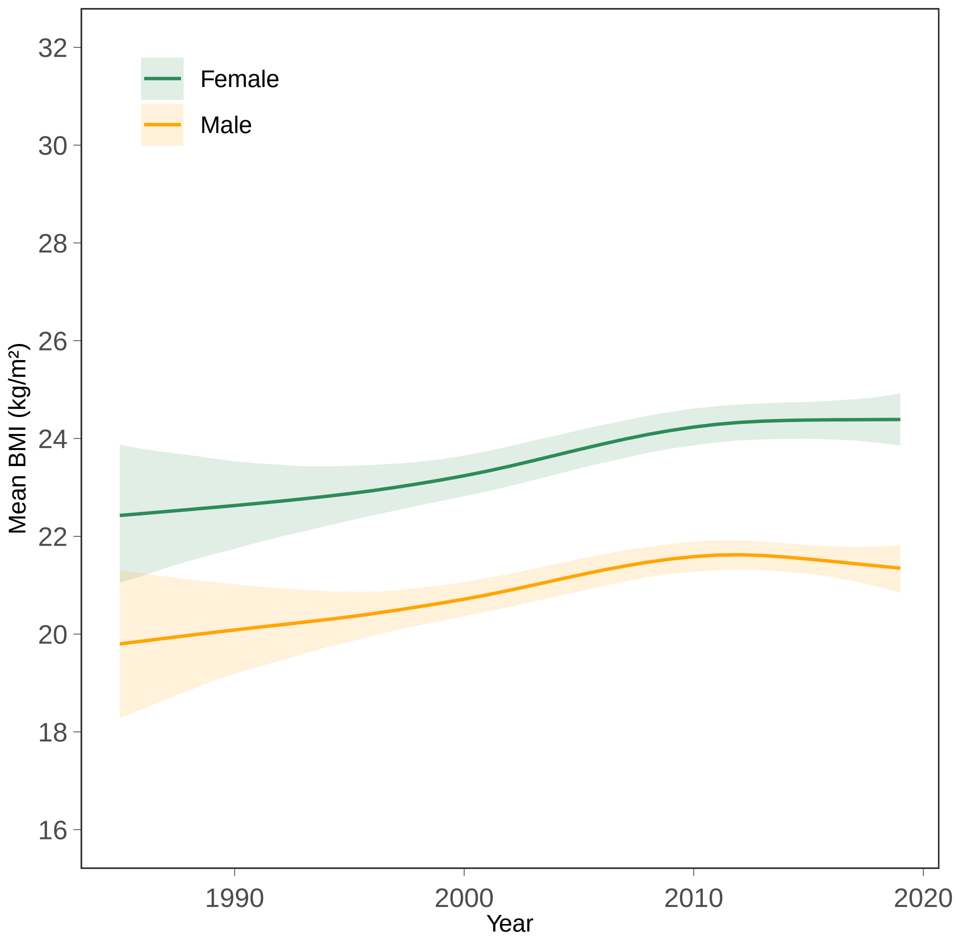

Height−for−age trajectories (2000 birth cohort)

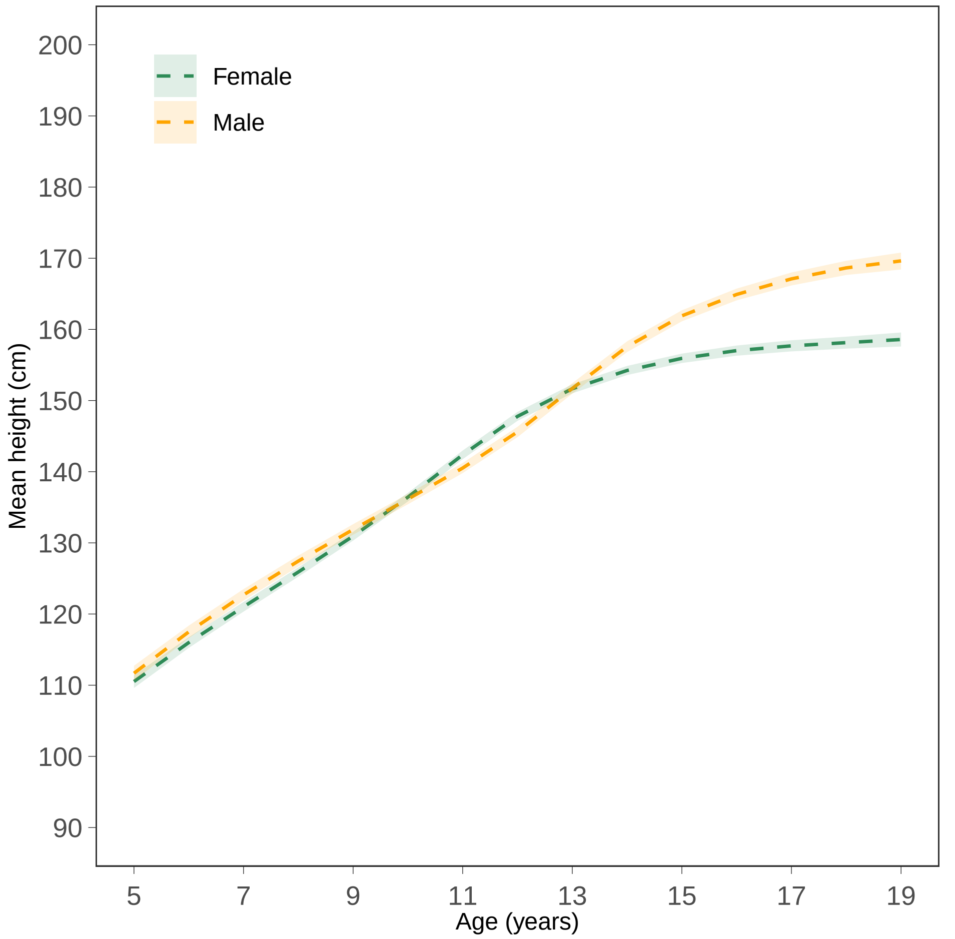

BMI−for−age trajectories (2000 birth cohort)

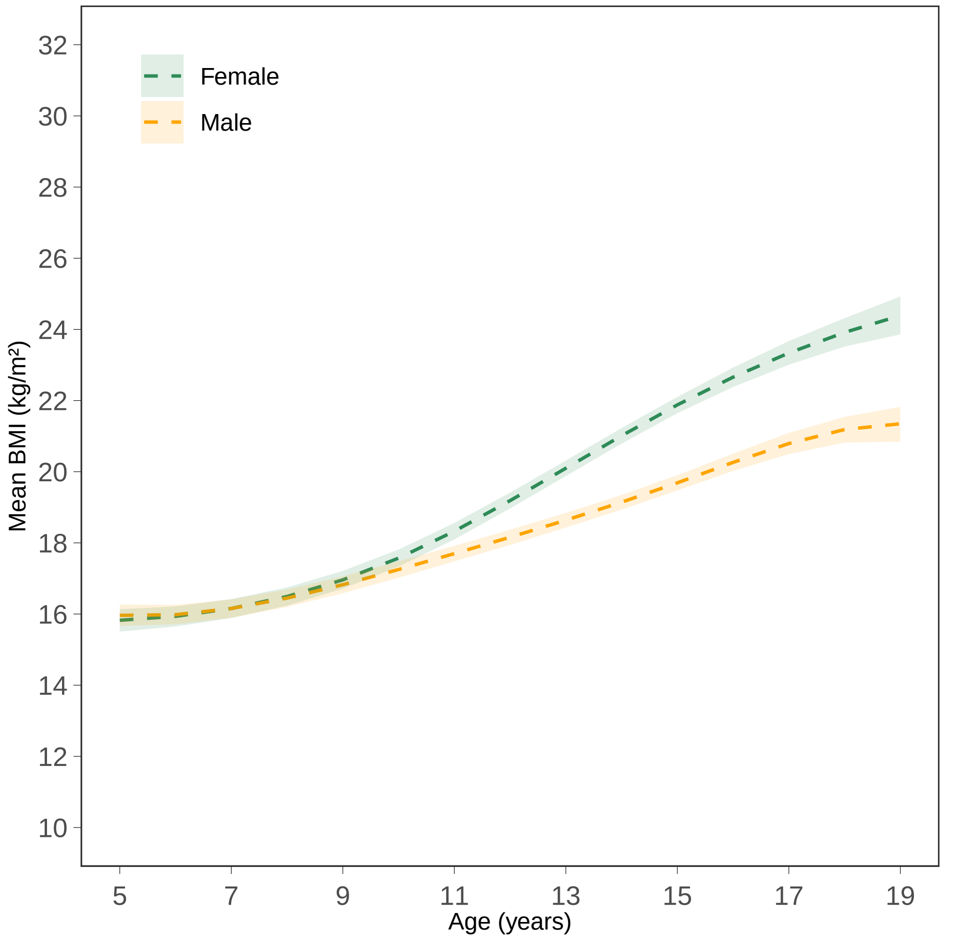

South Korea

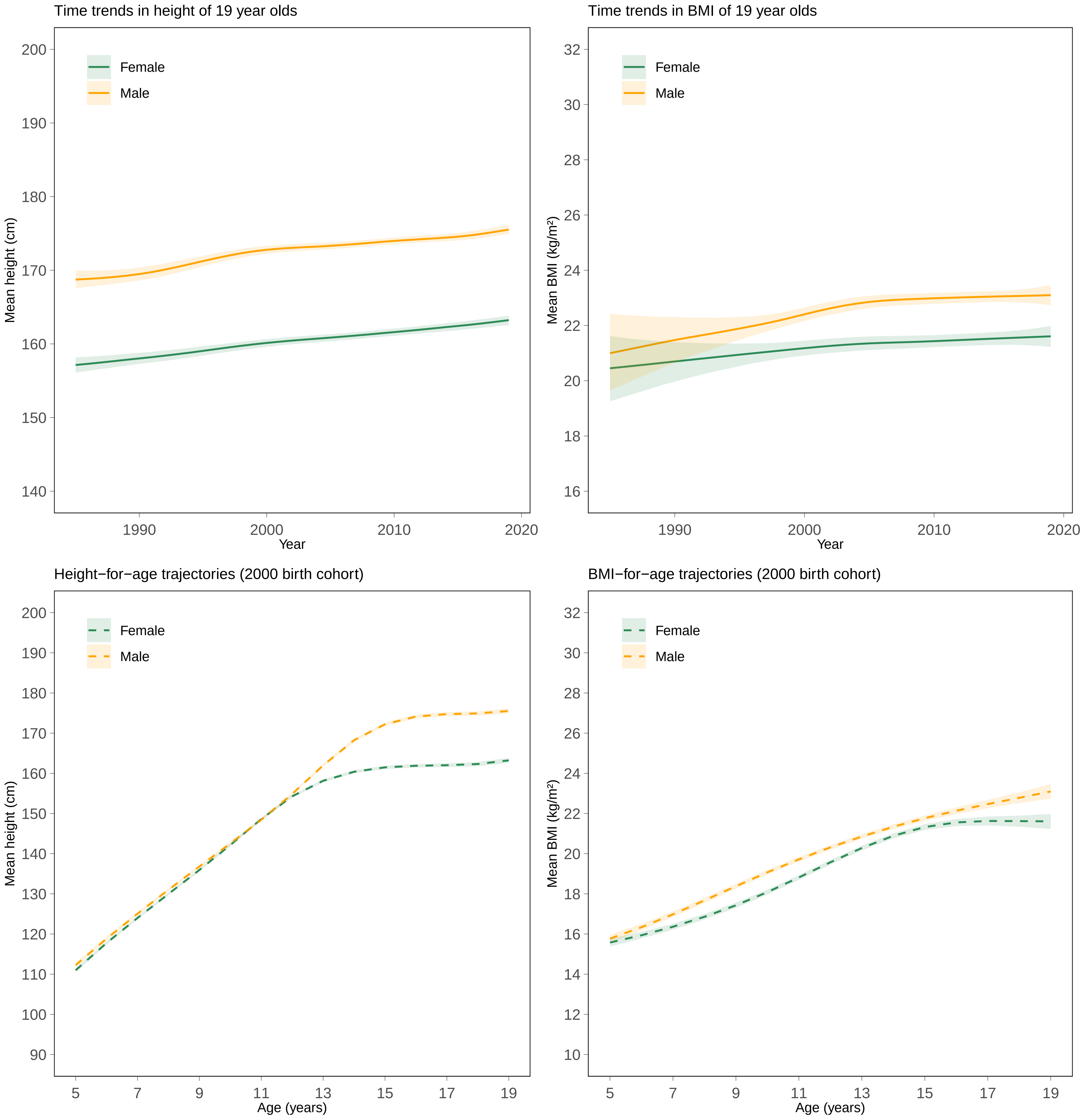
Time trends in height of 19 year olds
Female
Male
Mean height (cm)
Year
200
190
180
170
160
150
140
1990
2000
2010
2020
Time trends in BMI of 19 year olds
Female
Male
Mean BMI (kg/m²)
Year
32
30
28
26
24
22
20
18
16
1990
2000
2010
2020
Height−for−age trajectories (2000 birth cohort)
Female
Male
Mean height (cm)
Age (years)
200
190
180
170
160
150
140
130
120
110
100
90
5
7
9
11
13
15
17
19
BMI−for−age trajectories (2000 birth cohort)
Female
Male
Mean BMI (kg/m²)
Age (years)
32
30
28
26
24
22
20
18
16
14
12
10
5
7
9
11
13
15
17
19

Spain

Time trends in height of 19 year olds

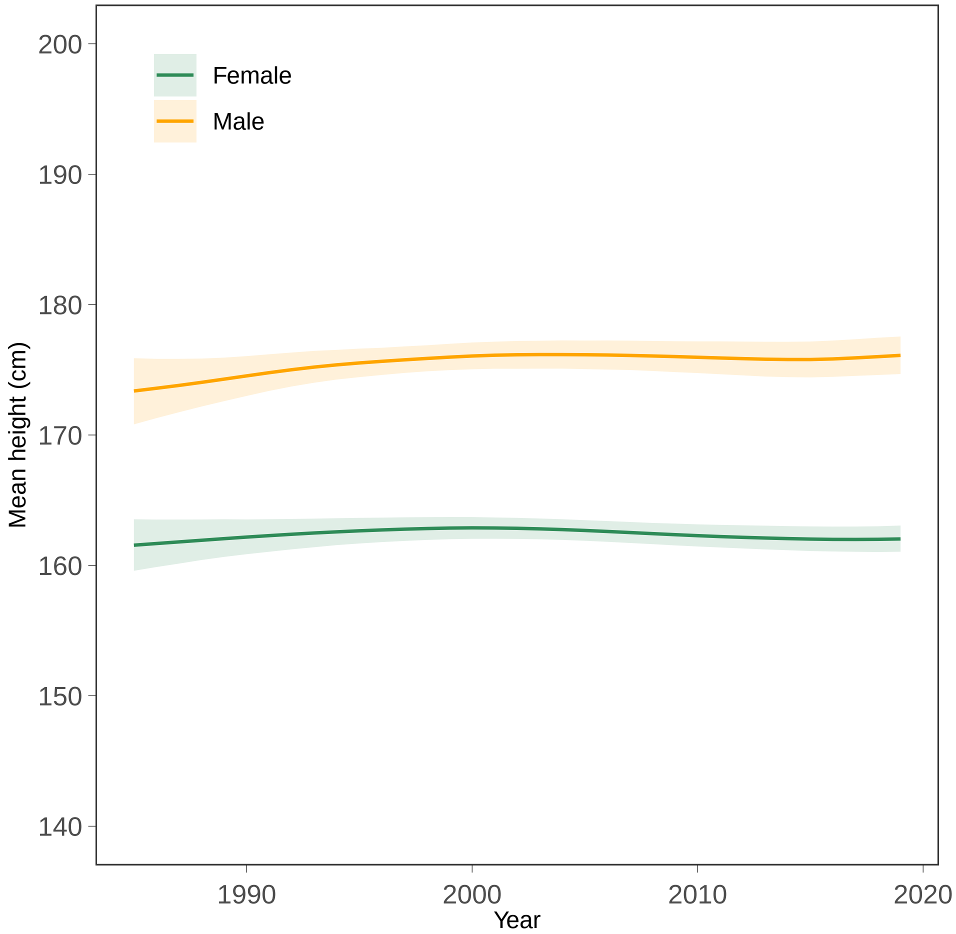

Time trends in BMI of 19 year olds

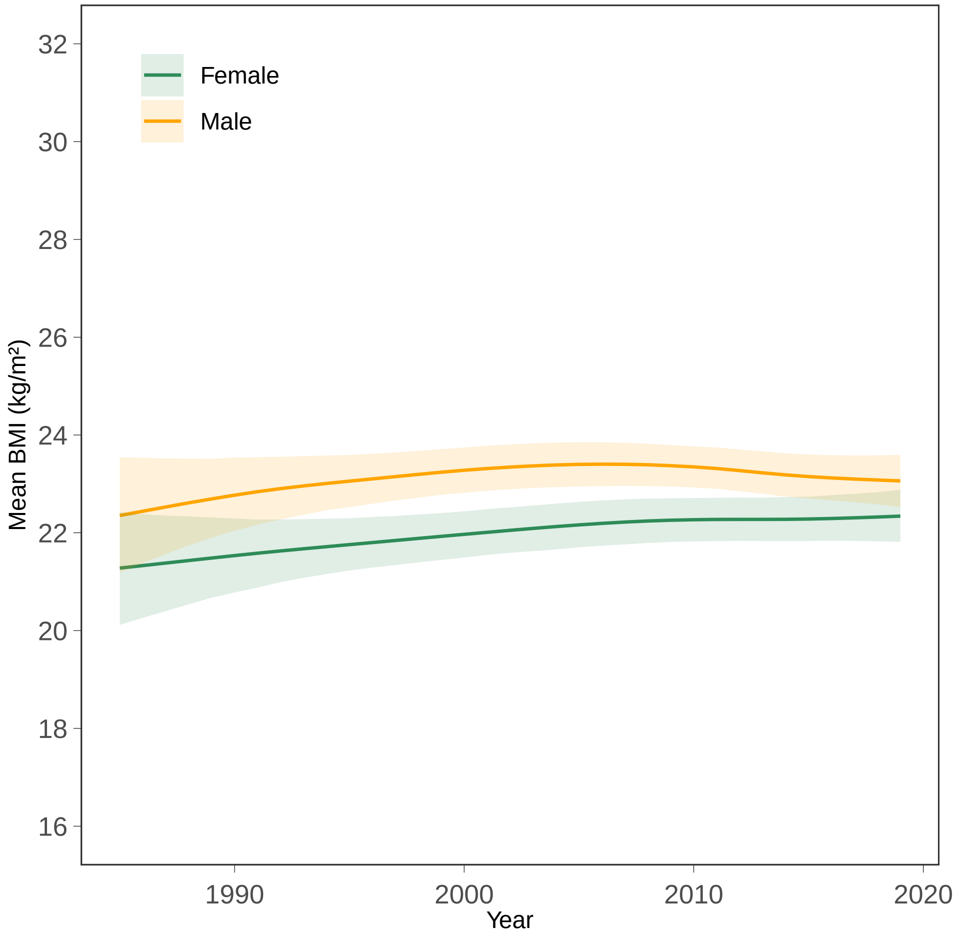

Height−for−age trajectories (2000 birth cohort)

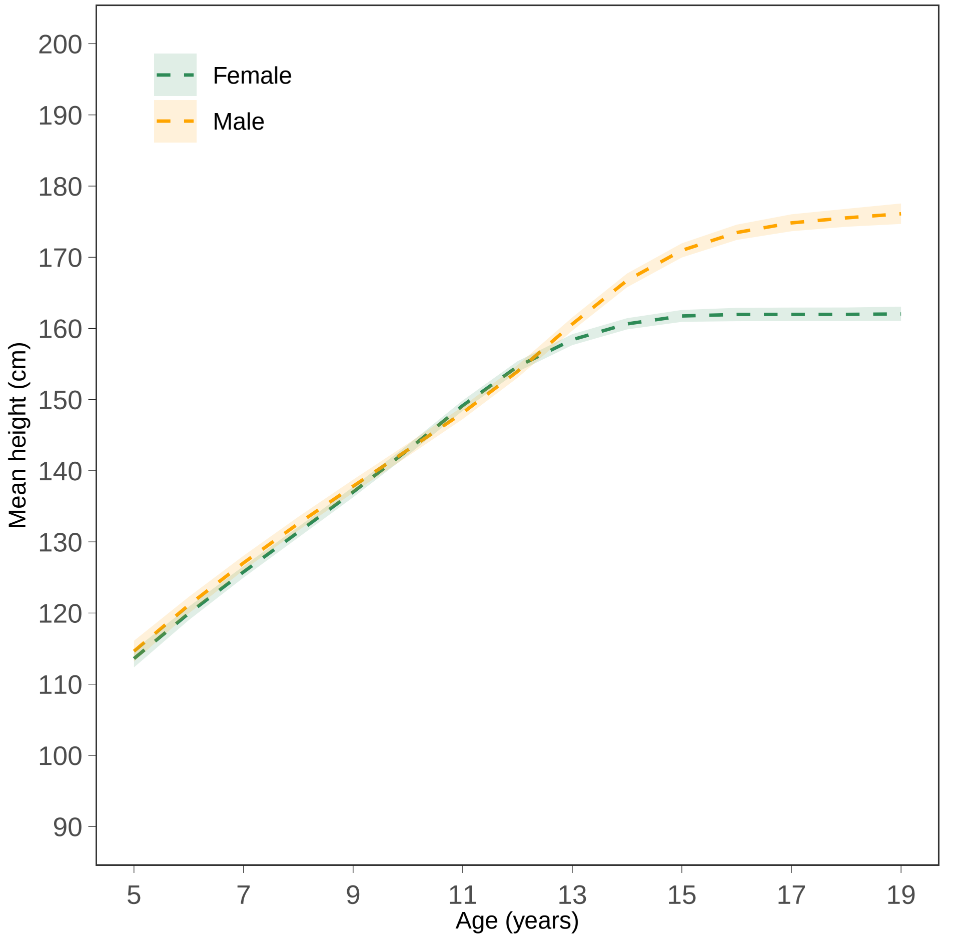

BMI−for−age trajectories (2000 birth cohort)

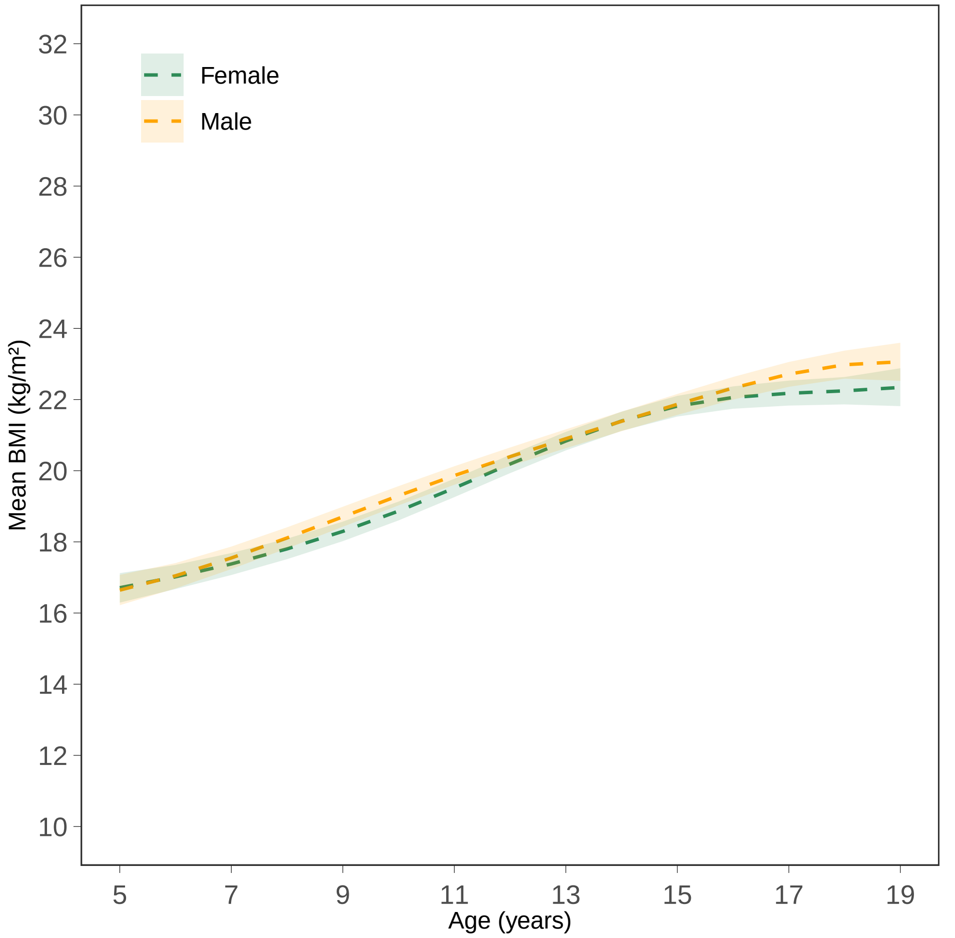

Sri Lanka

Time trends in height of 19 year olds

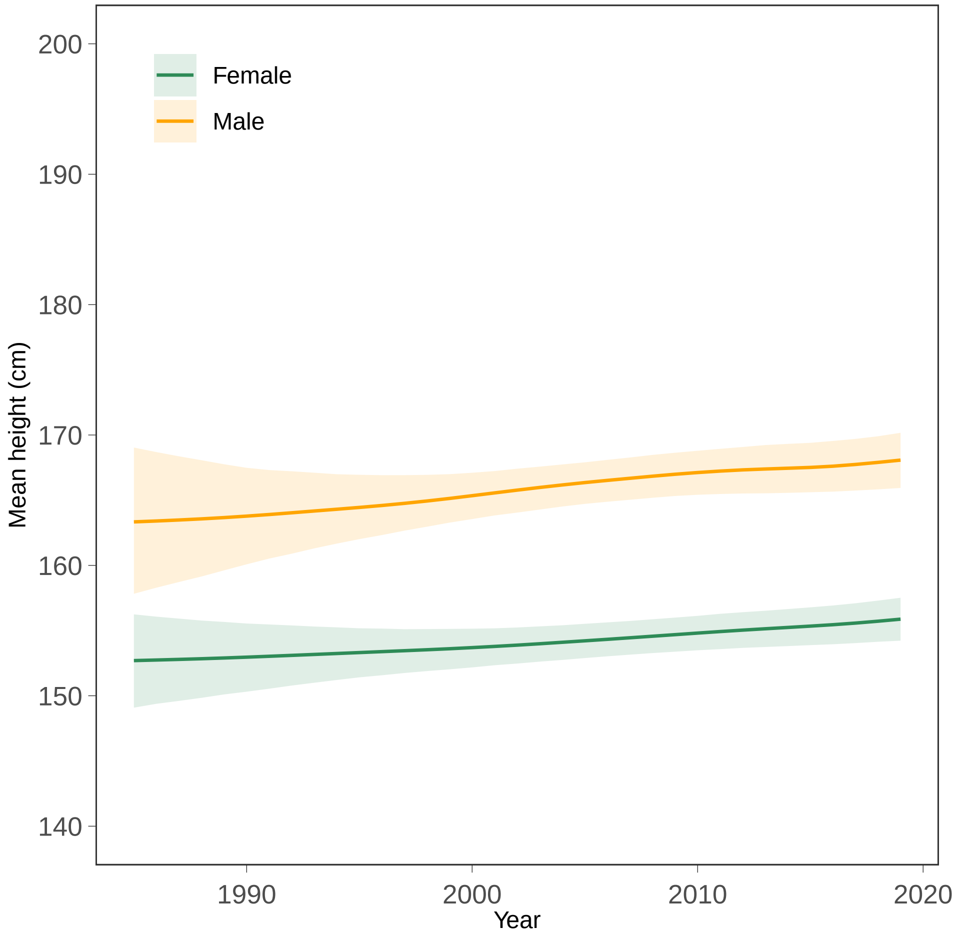

Time trends in BMI of 19 year olds

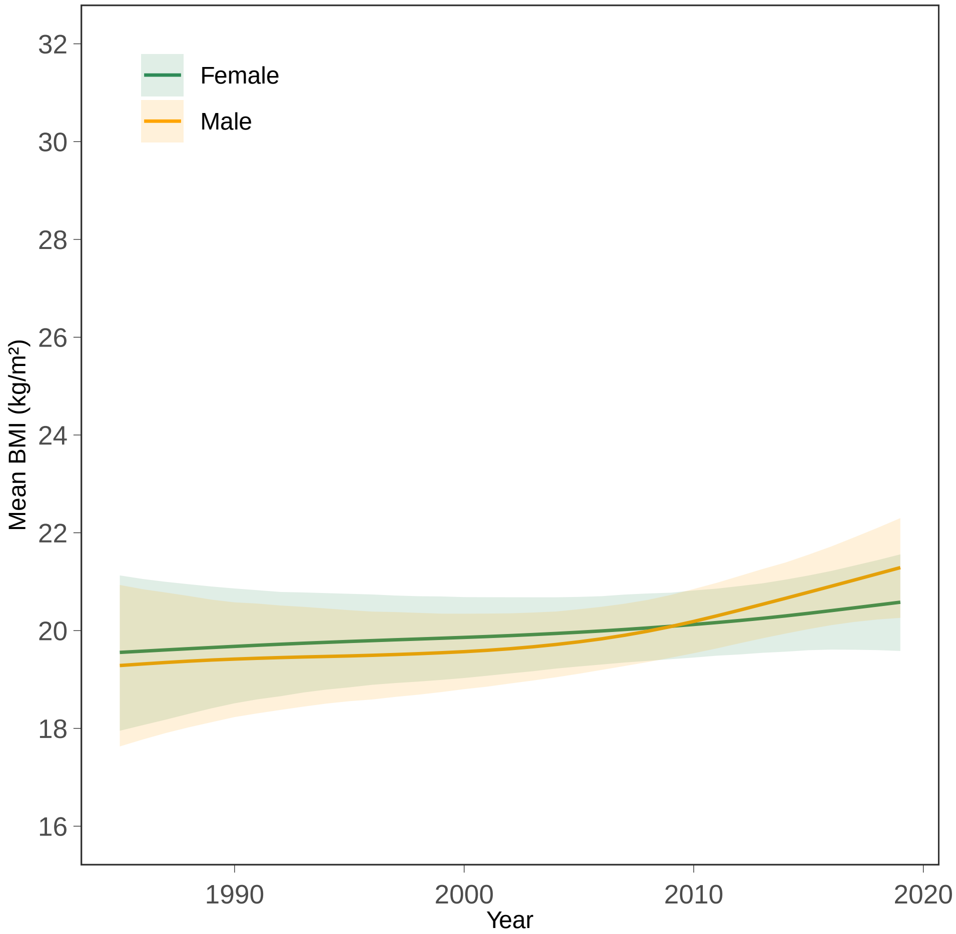

Height−for−age trajectories (2000 birth cohort)

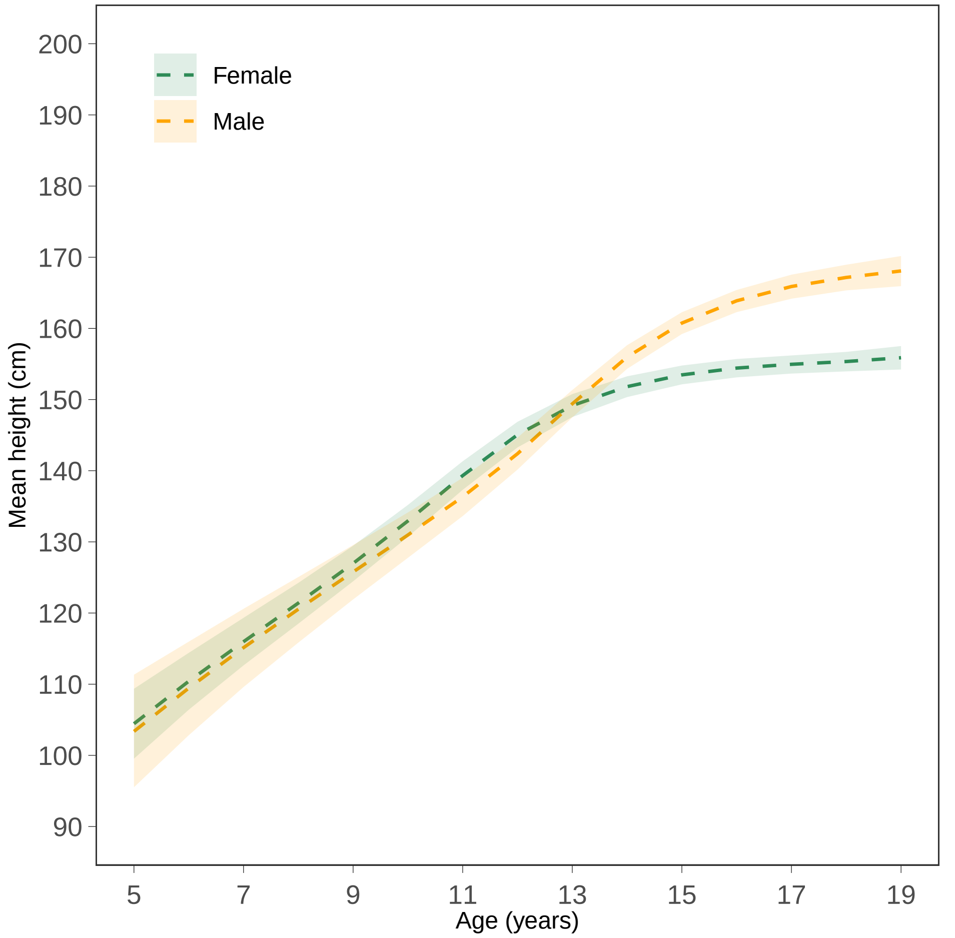

BMI−for−age trajectories (2000 birth cohort)

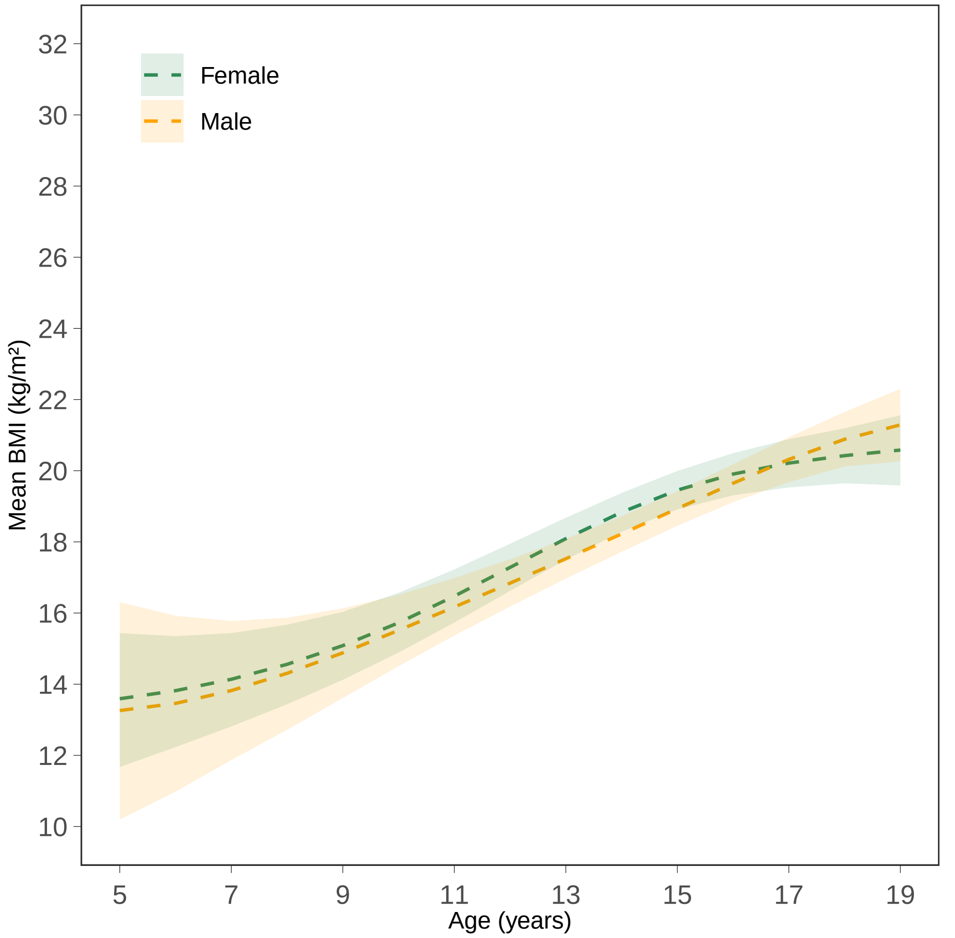

State of Palestine

Time trends in height of 19 year olds

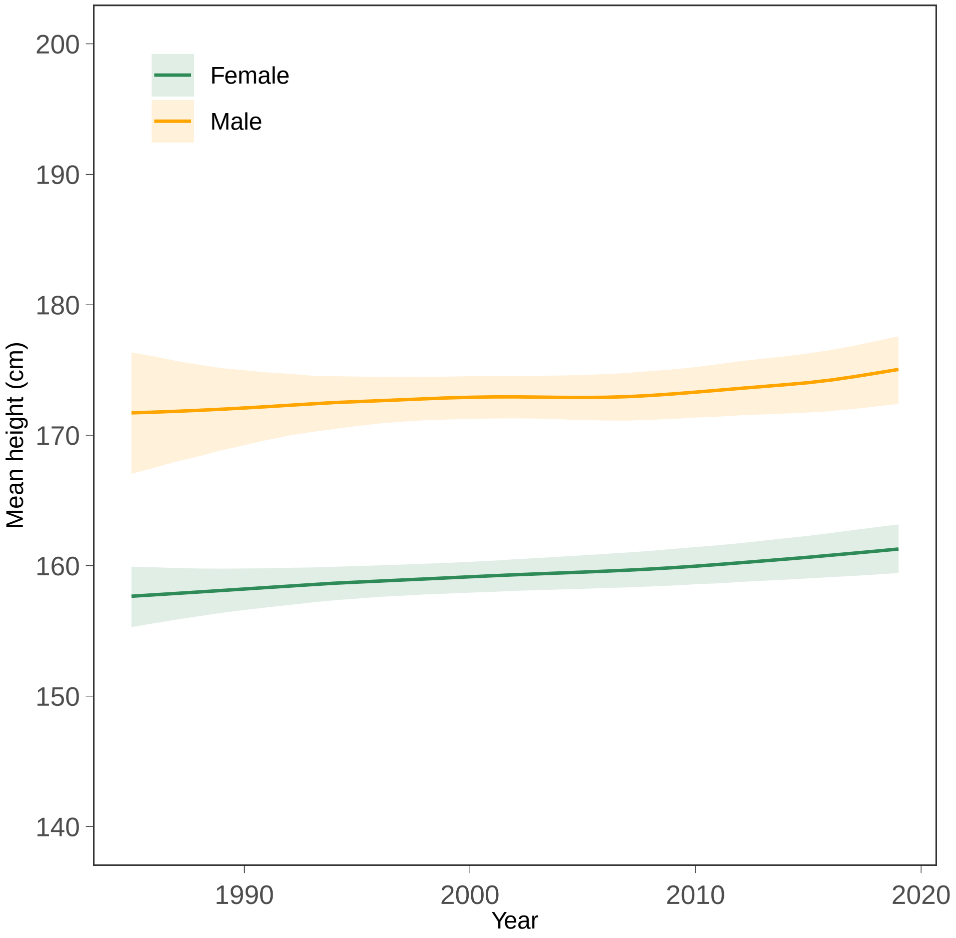

Time trends in BMI of 19 year olds

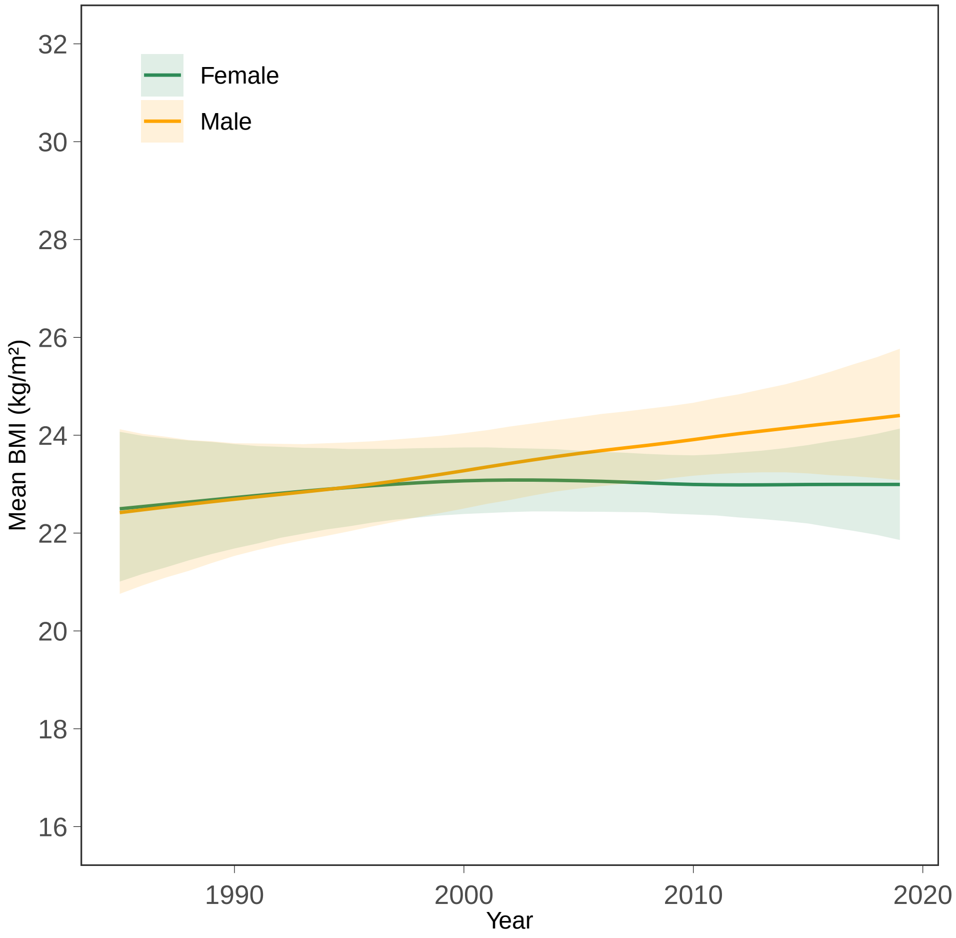

Height−for−age trajectories (2000 birth cohort)

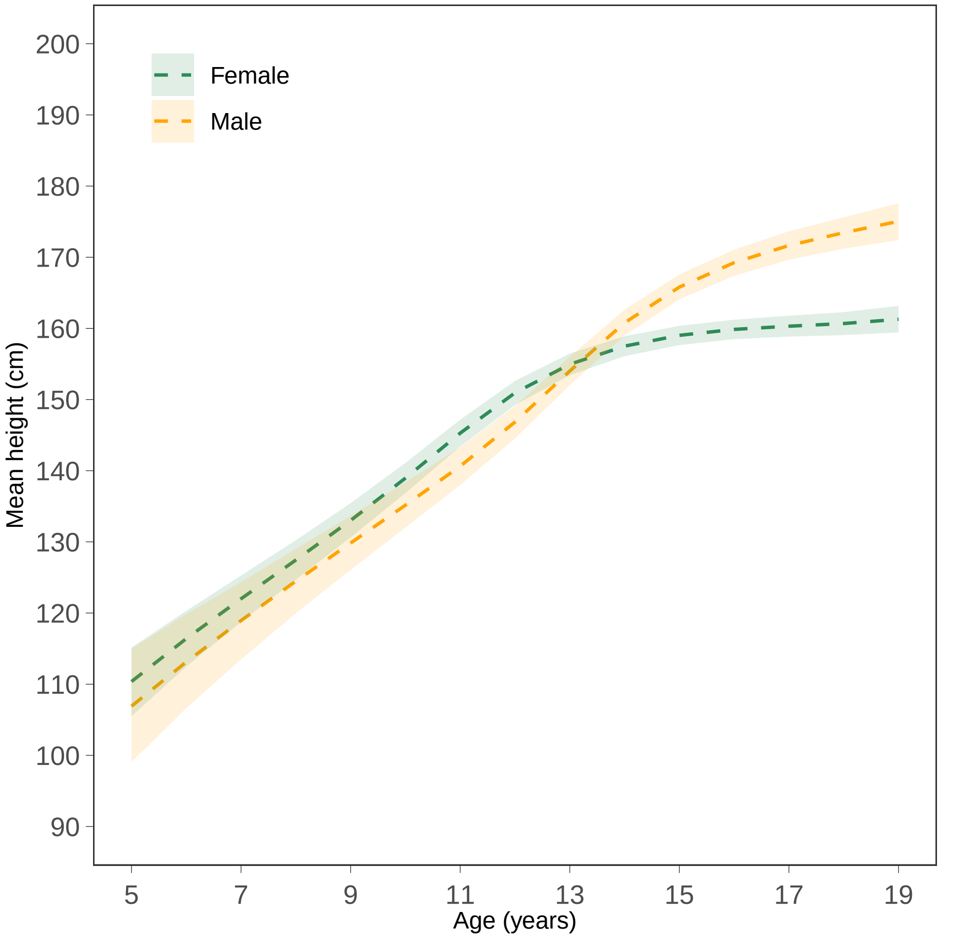

BMI−for−age trajectories (2000 birth cohort)

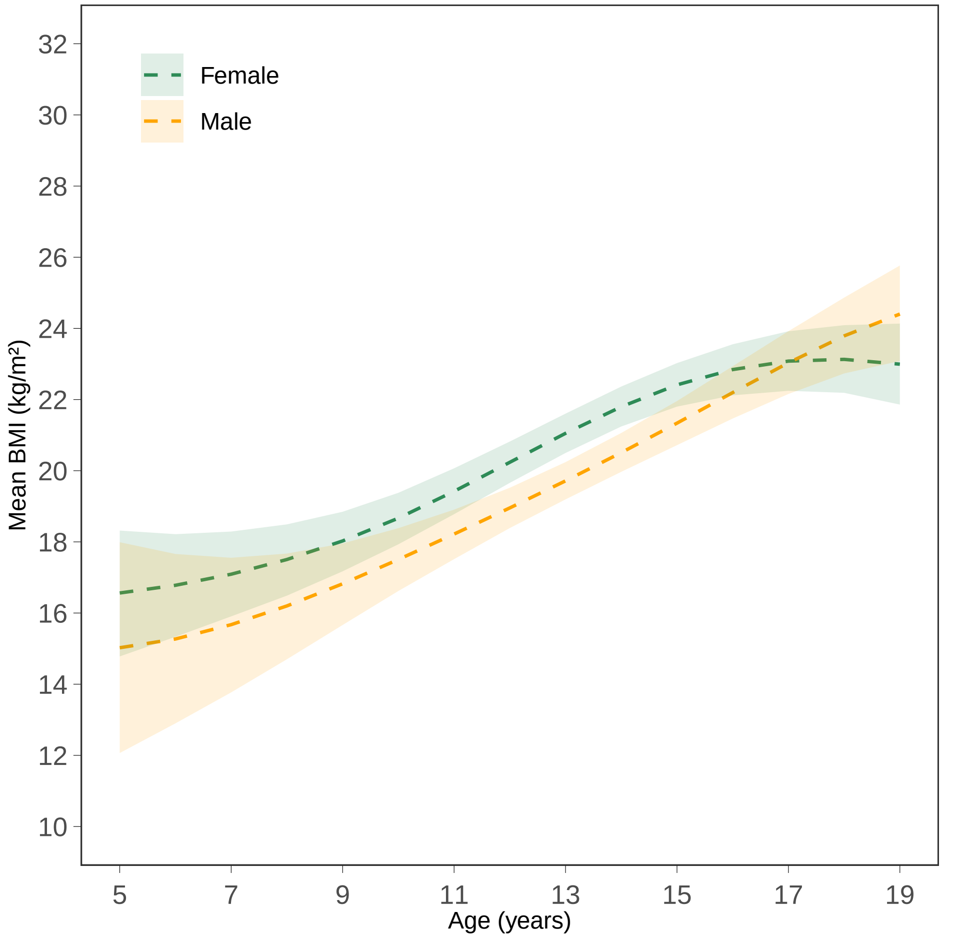

Sudan

Time trends in height of 19 year olds

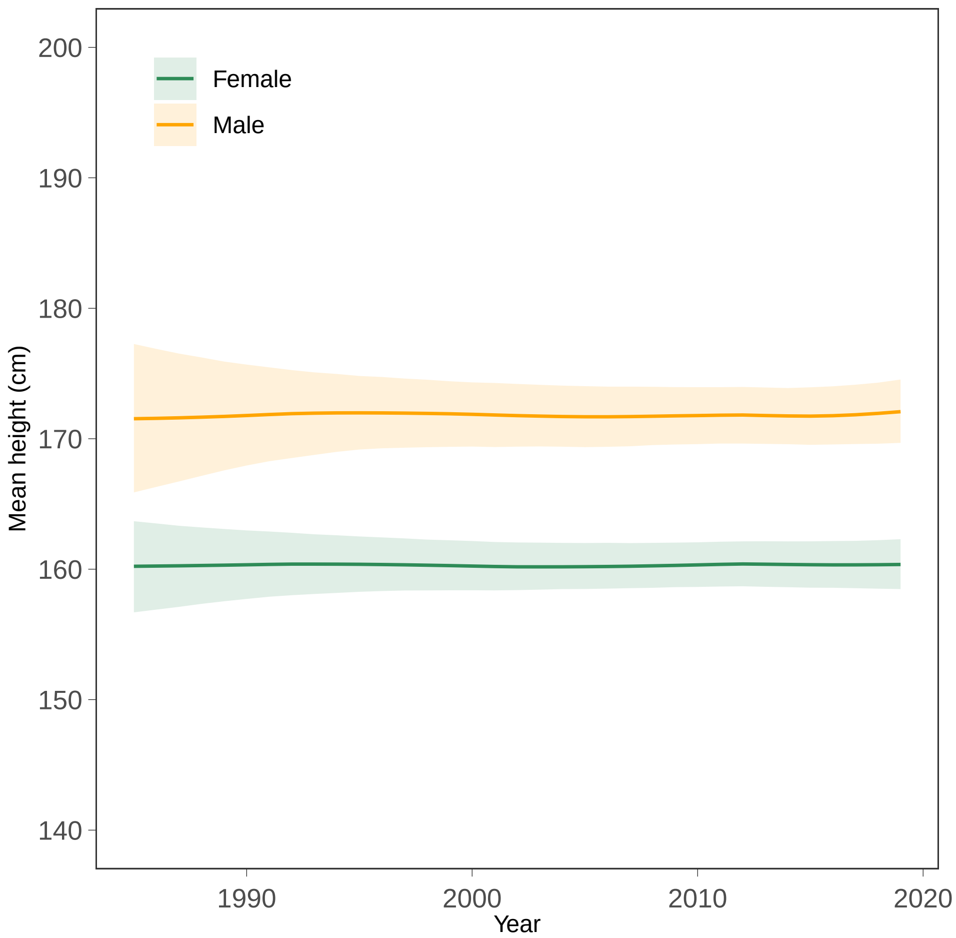

Time trends in BMI of 19 year olds

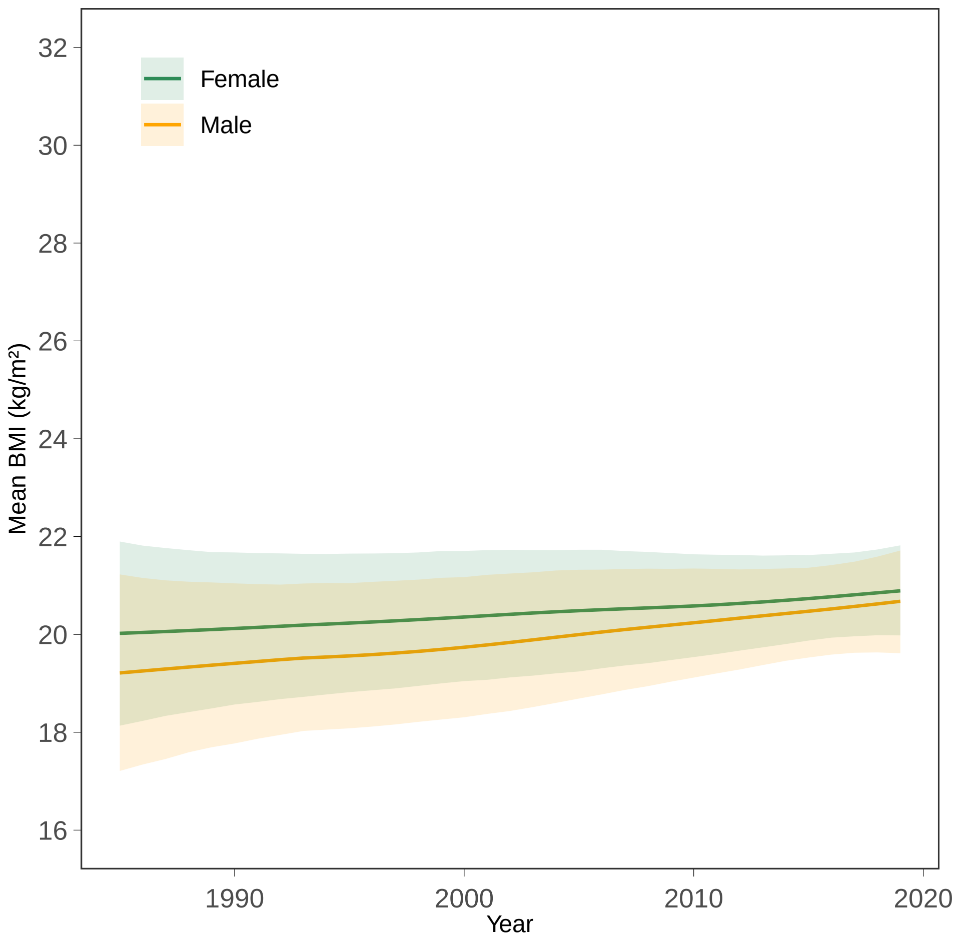

Height−for−age trajectories (2000 birth cohort)

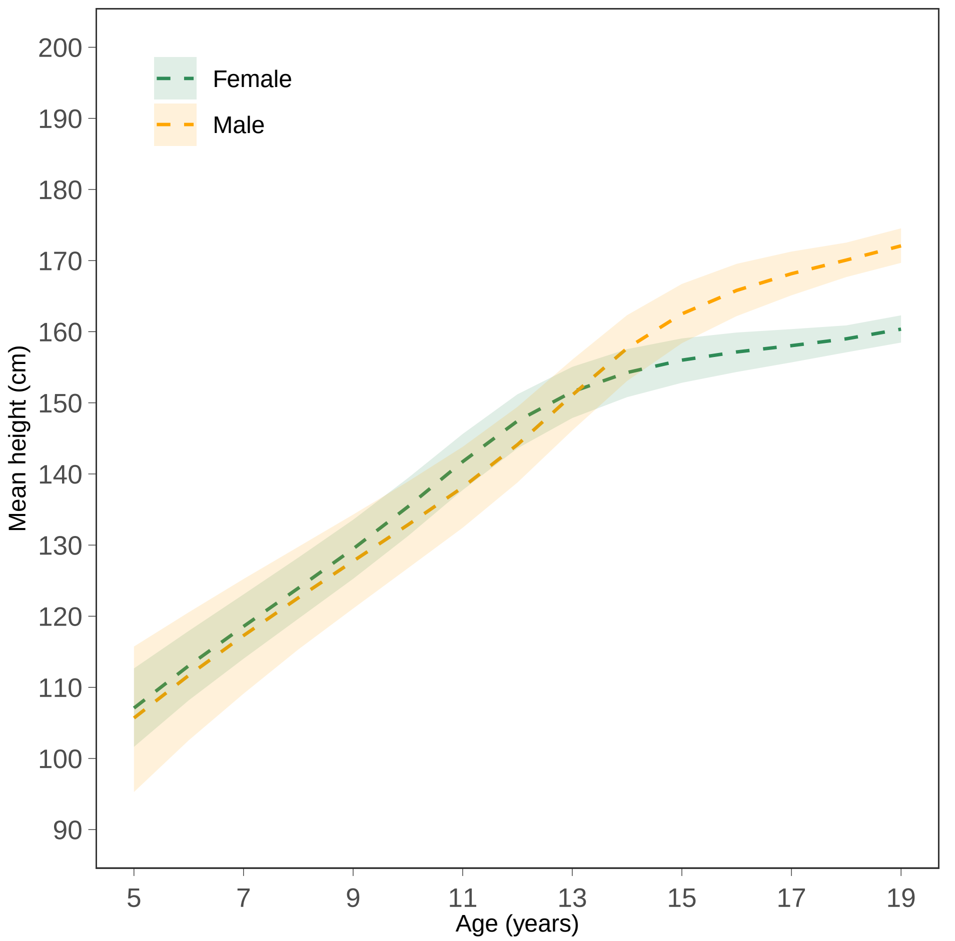

BMI−for−age trajectories (2000 birth cohort)

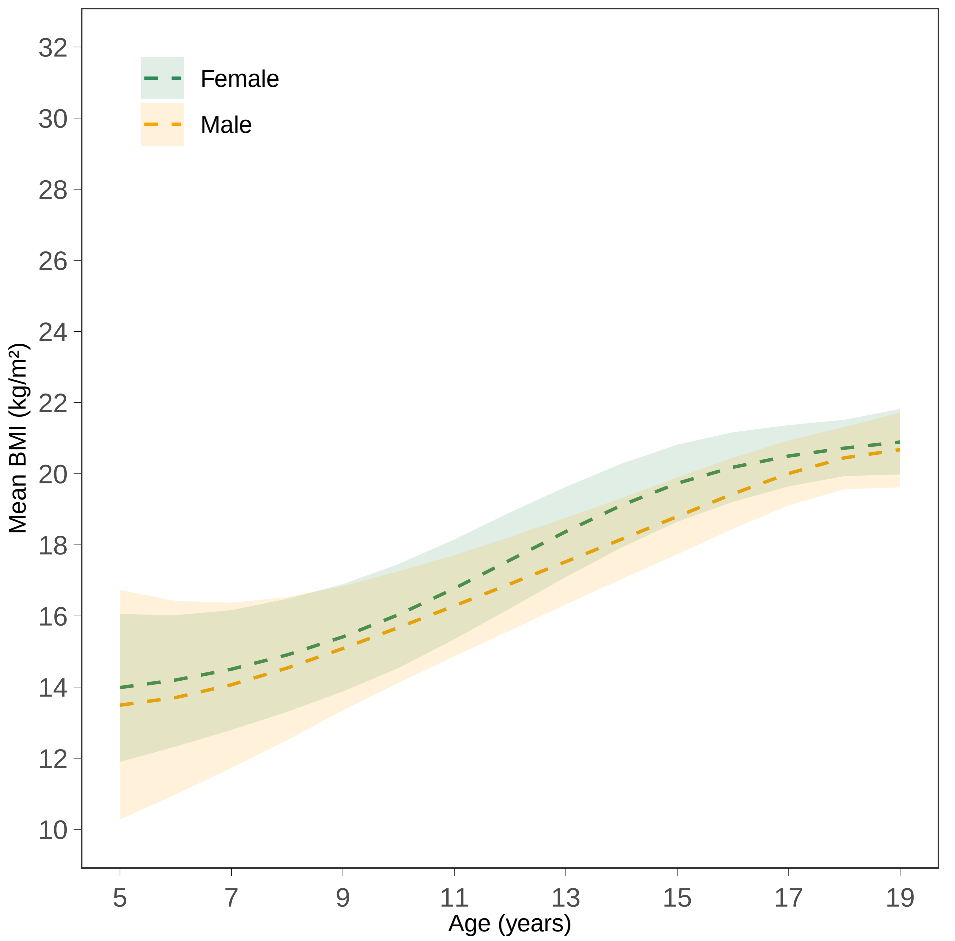

Suriname

Time trends in height of 19 year olds

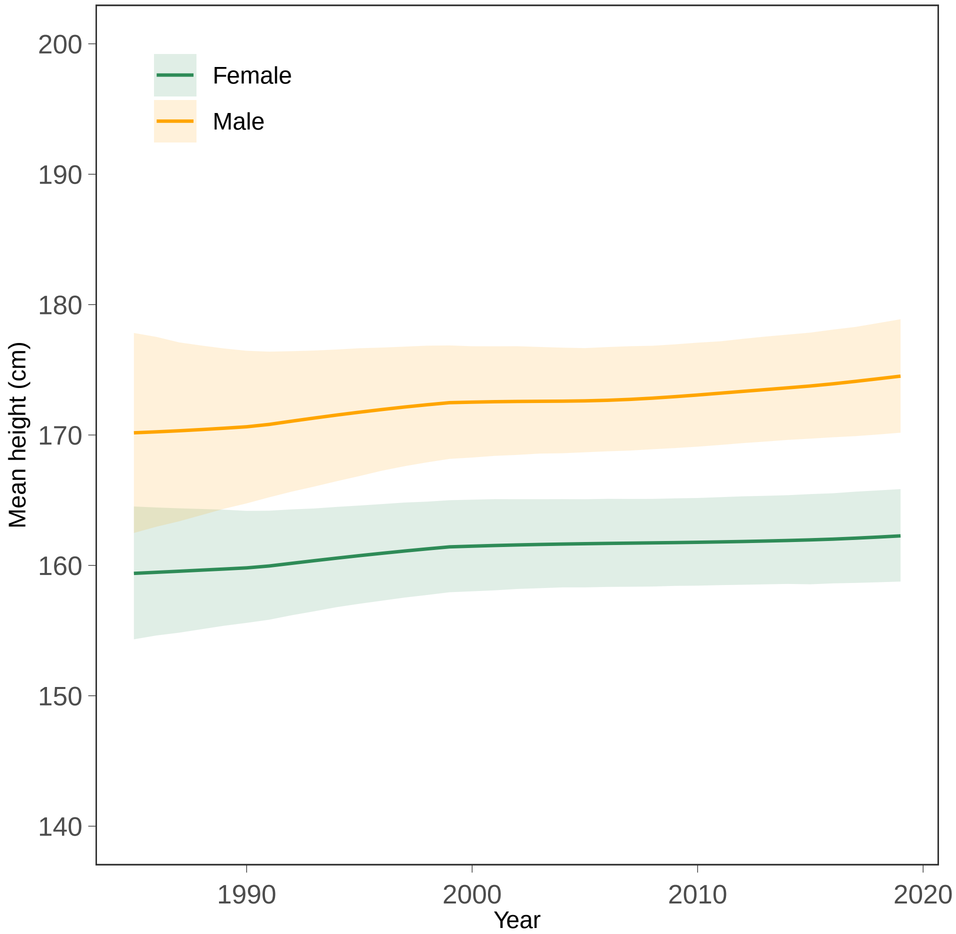

Time trends in BMI of 19 year olds

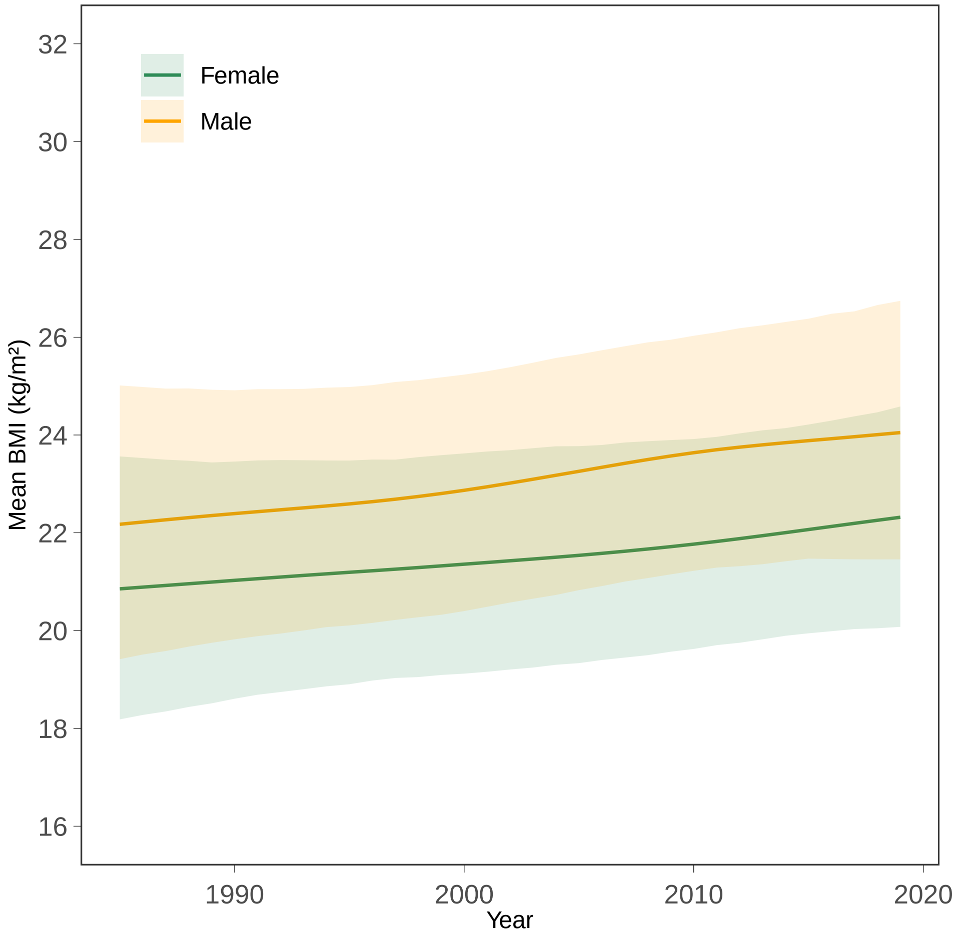

Height−for−age trajectories (2000 birth cohort)

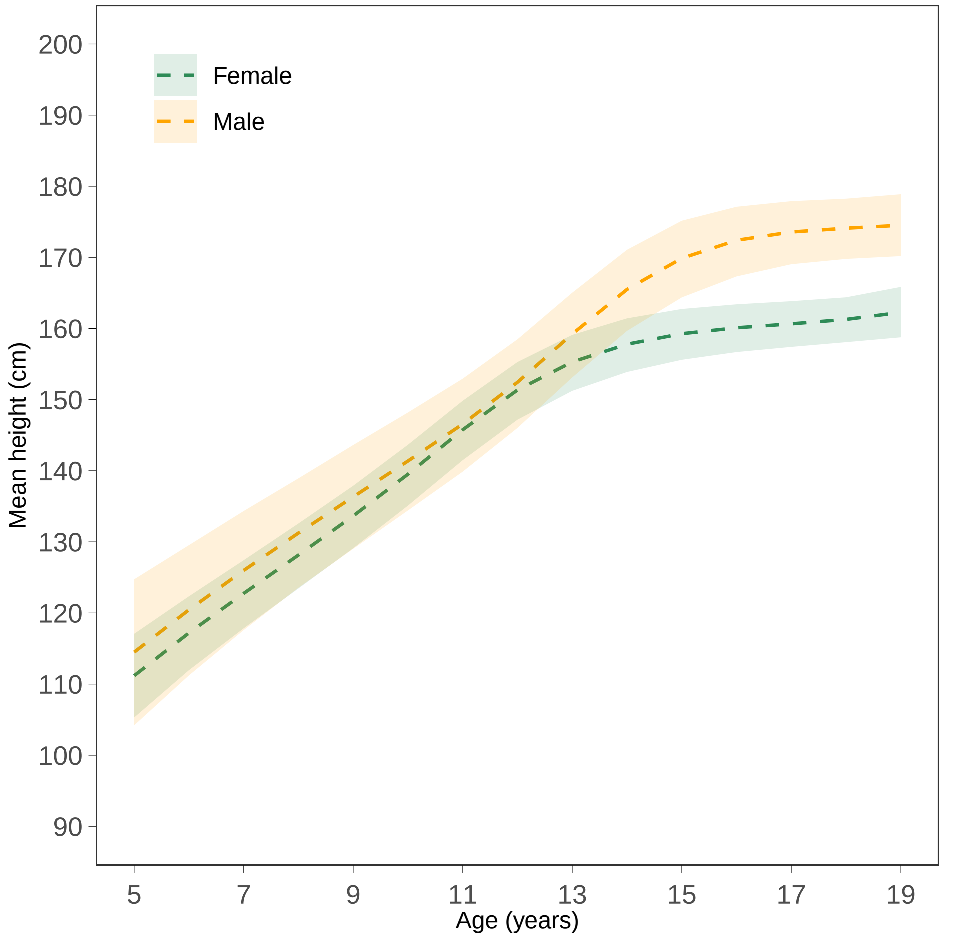

BMI−for−age trajectories (2000 birth cohort)

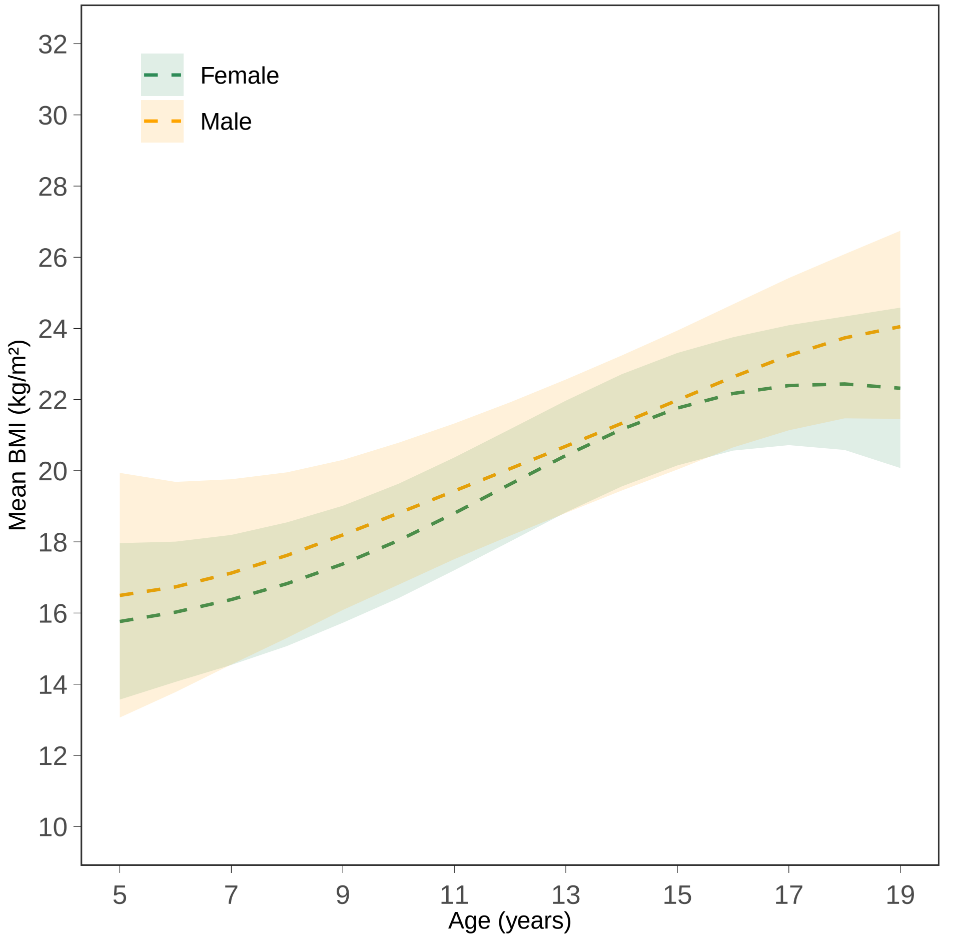

Sweden

Time trends in height of 19 year olds

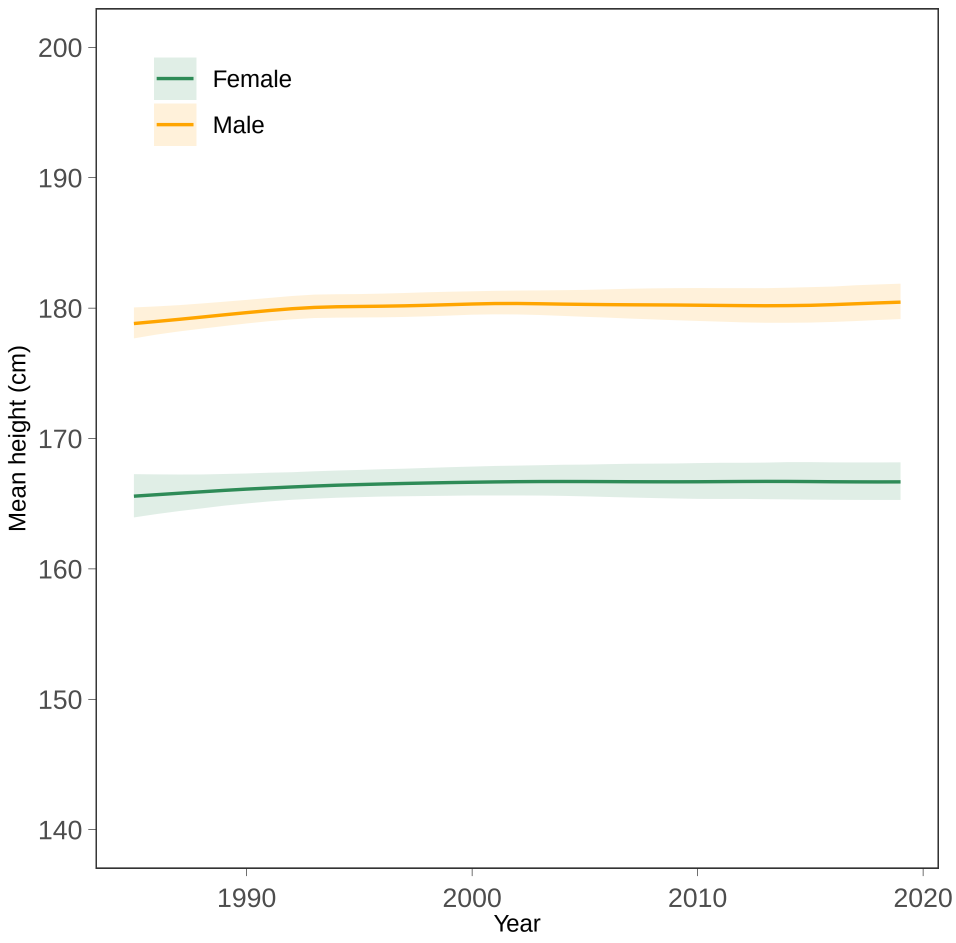

Time trends in BMI of 19 year olds

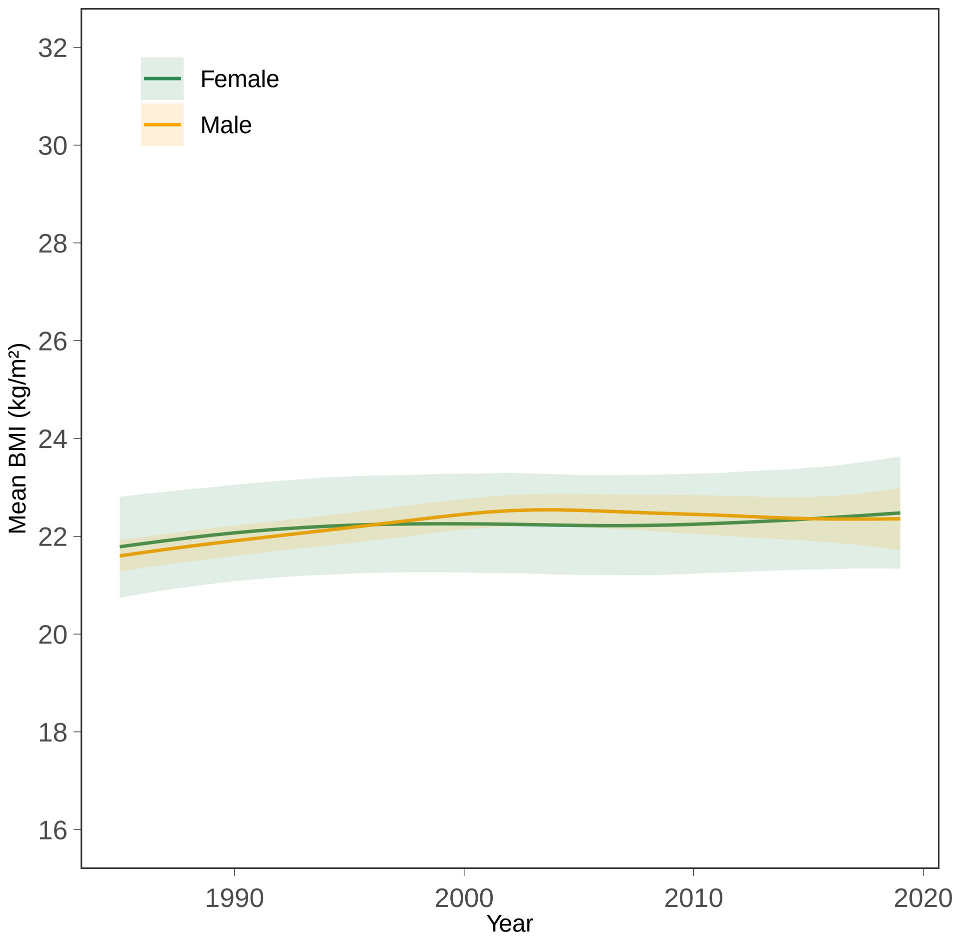

Height−for−age trajectories (2000 birth cohort)

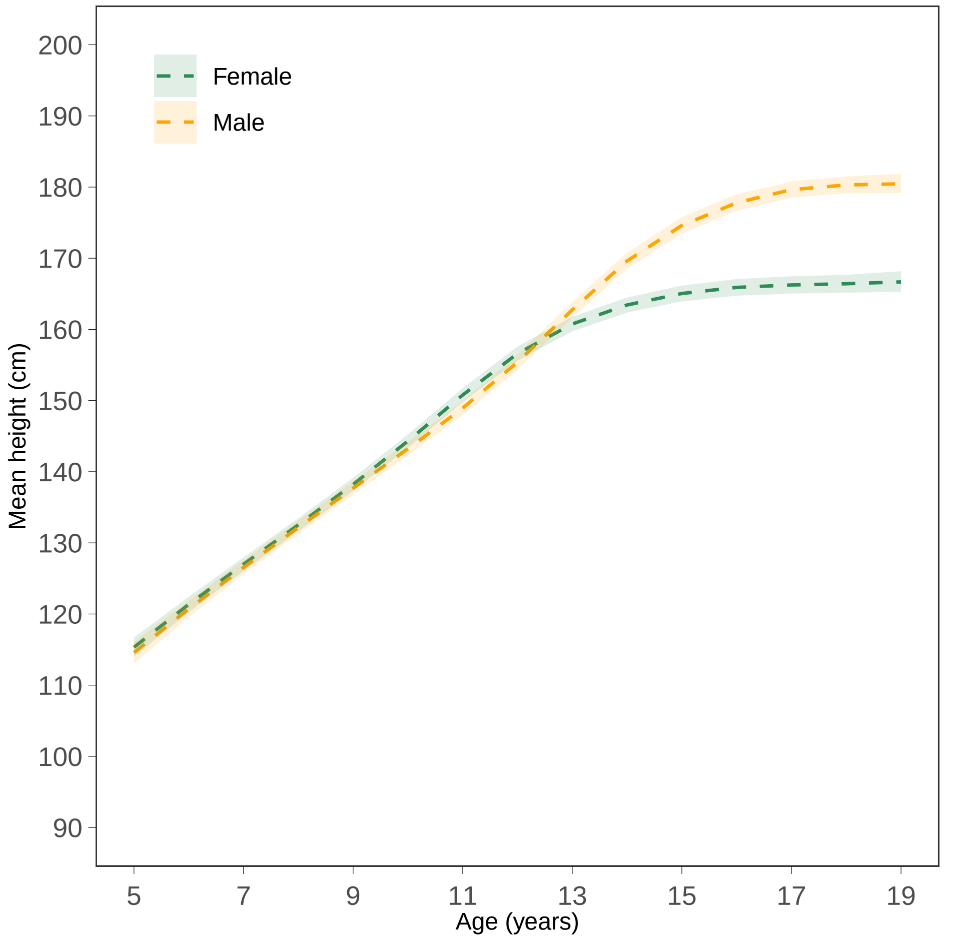

BMI−for−age trajectories (2000 birth cohort)

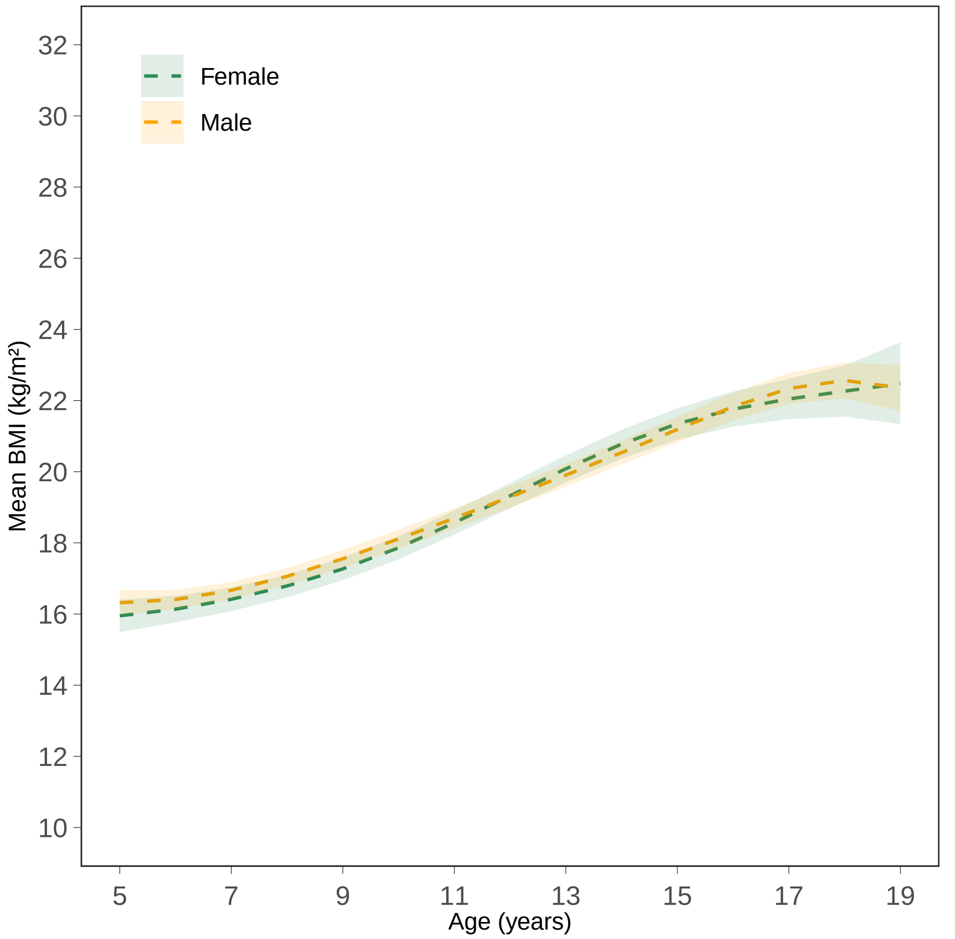

Switzerland

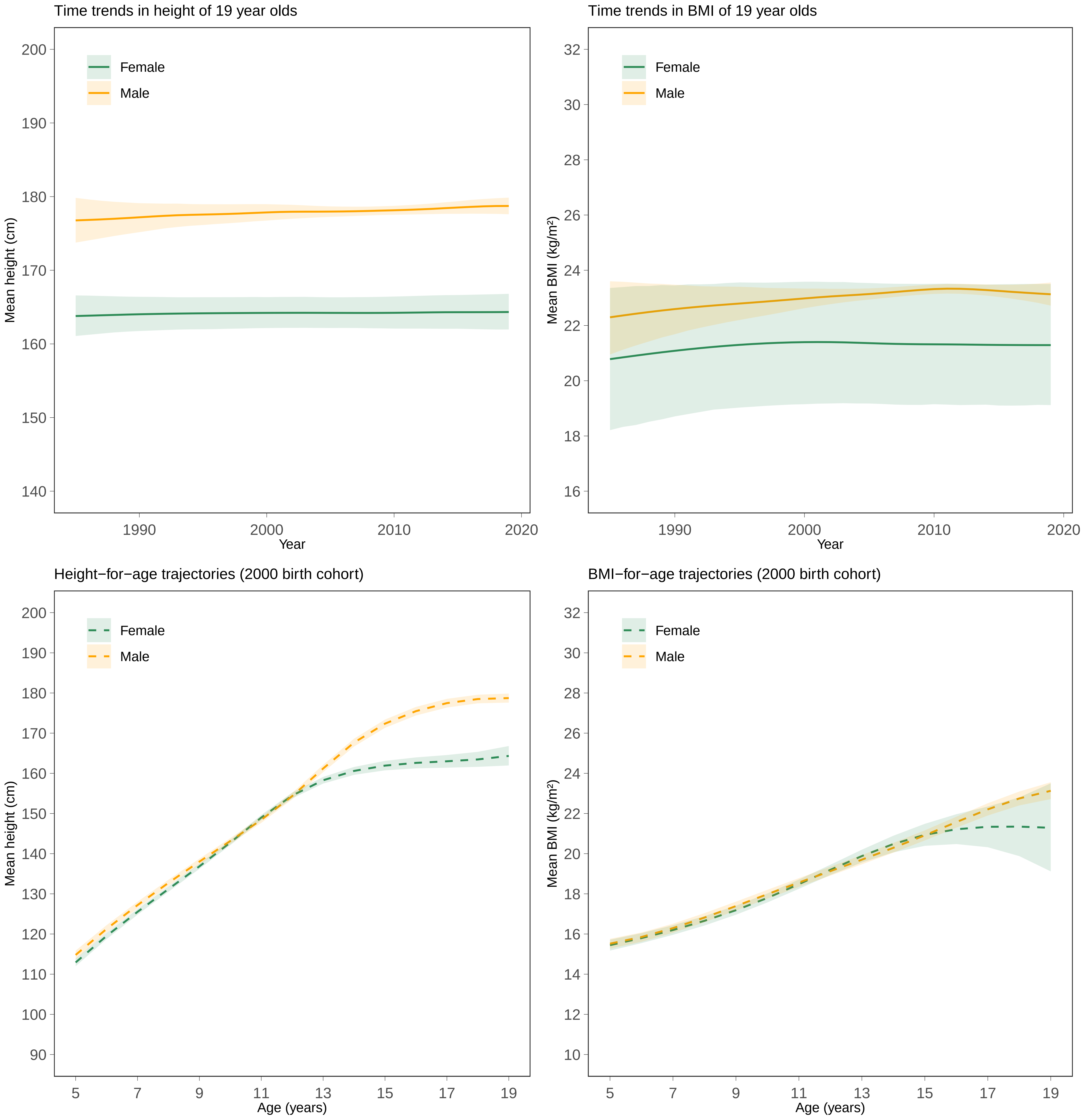

Syrian Arab Republic

Time trends in height of 19 year olds

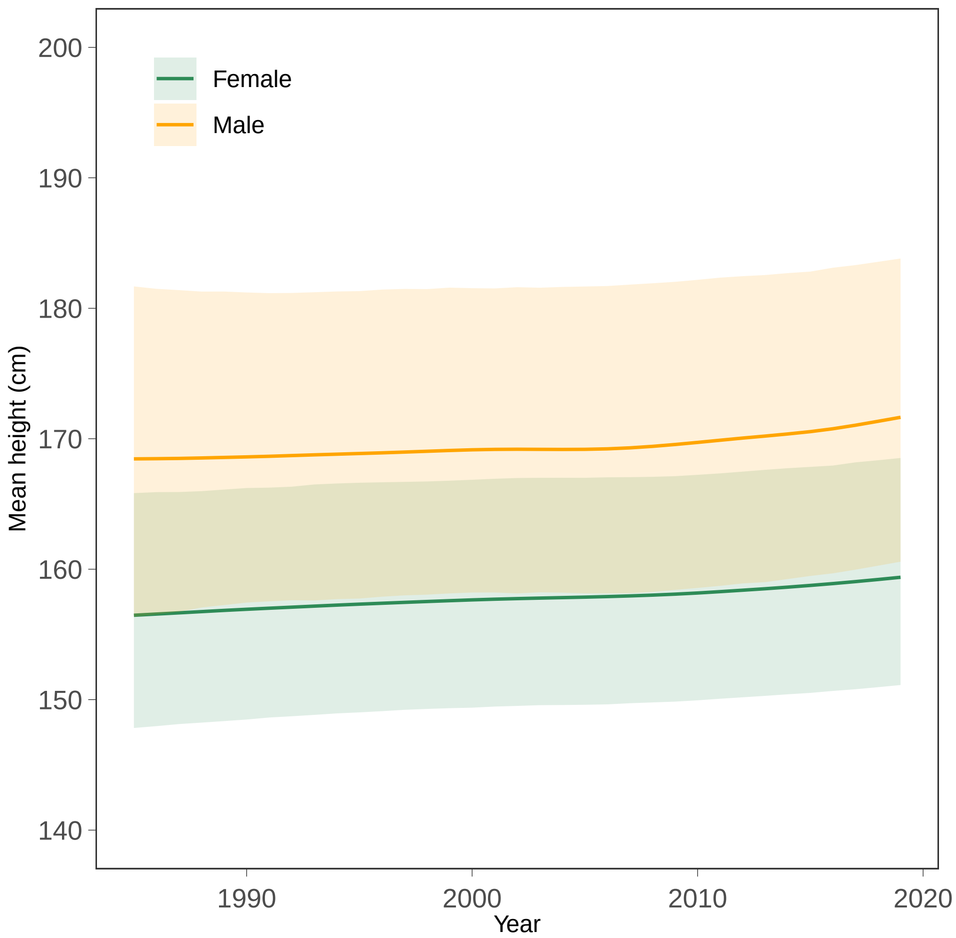

Time trends in BMI of 19 year olds

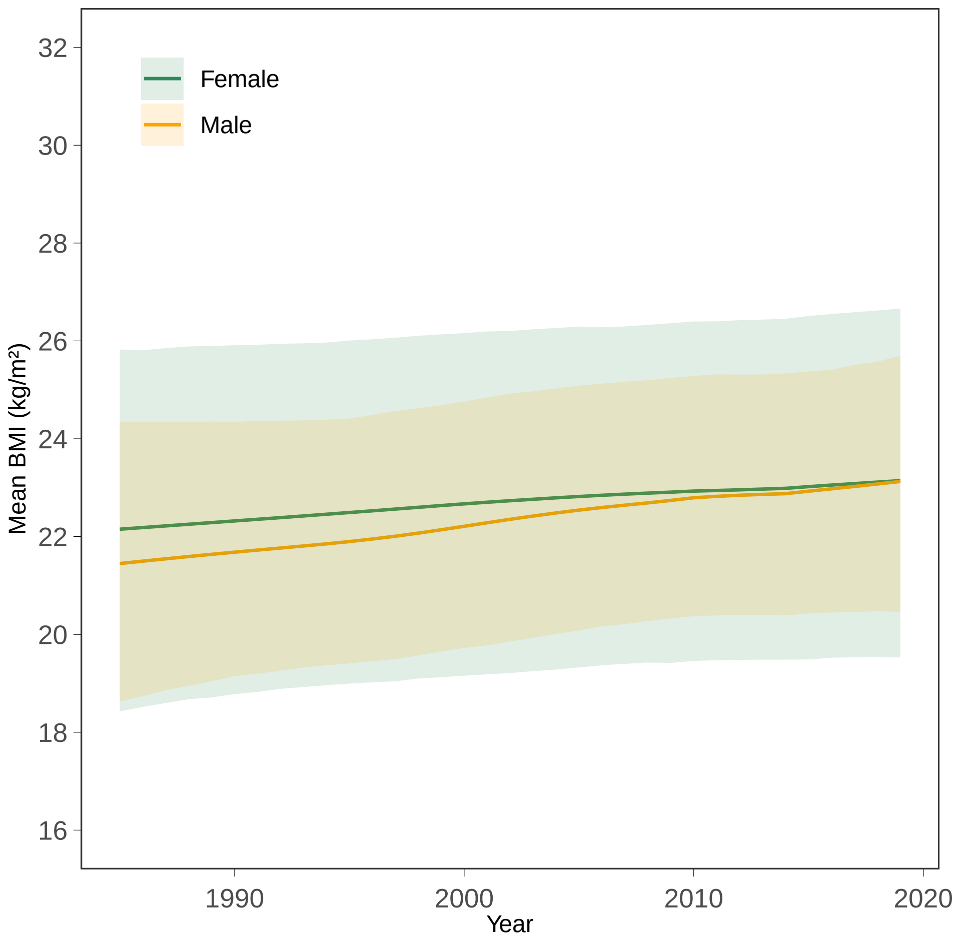

Height−for−age trajectories (2000 birth cohort)

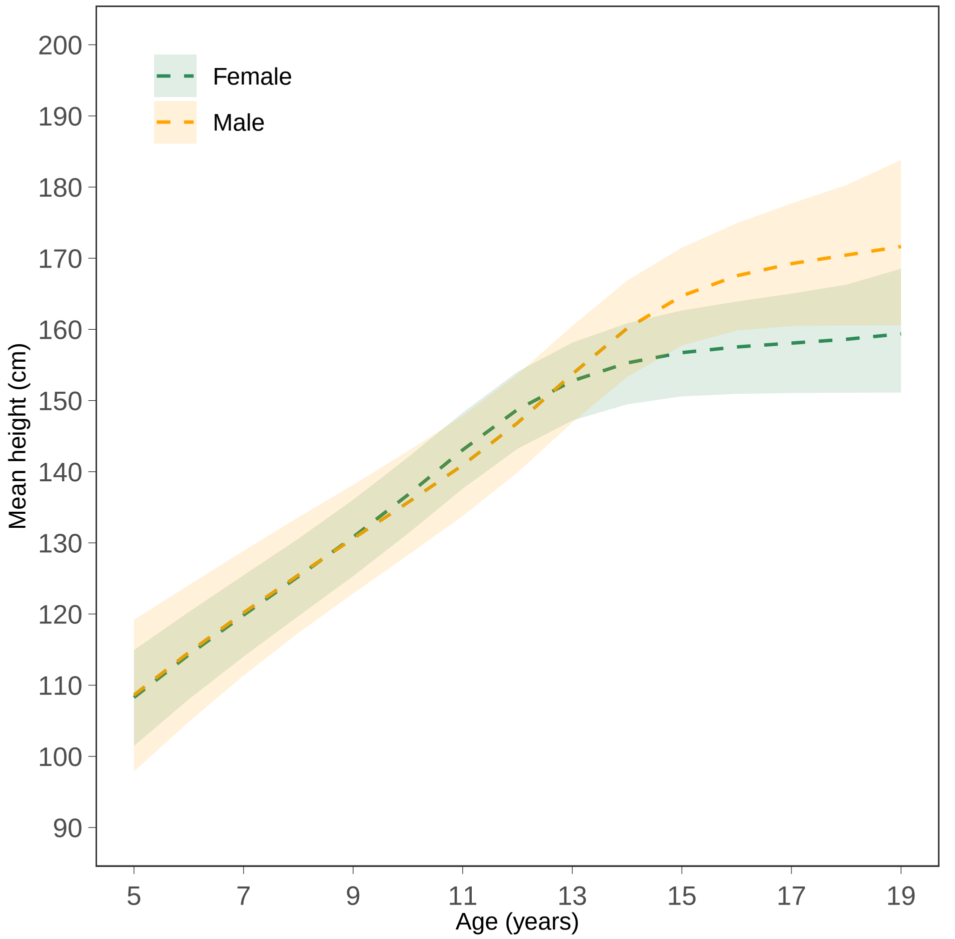

BMI−for−age trajectories (2000 birth cohort)

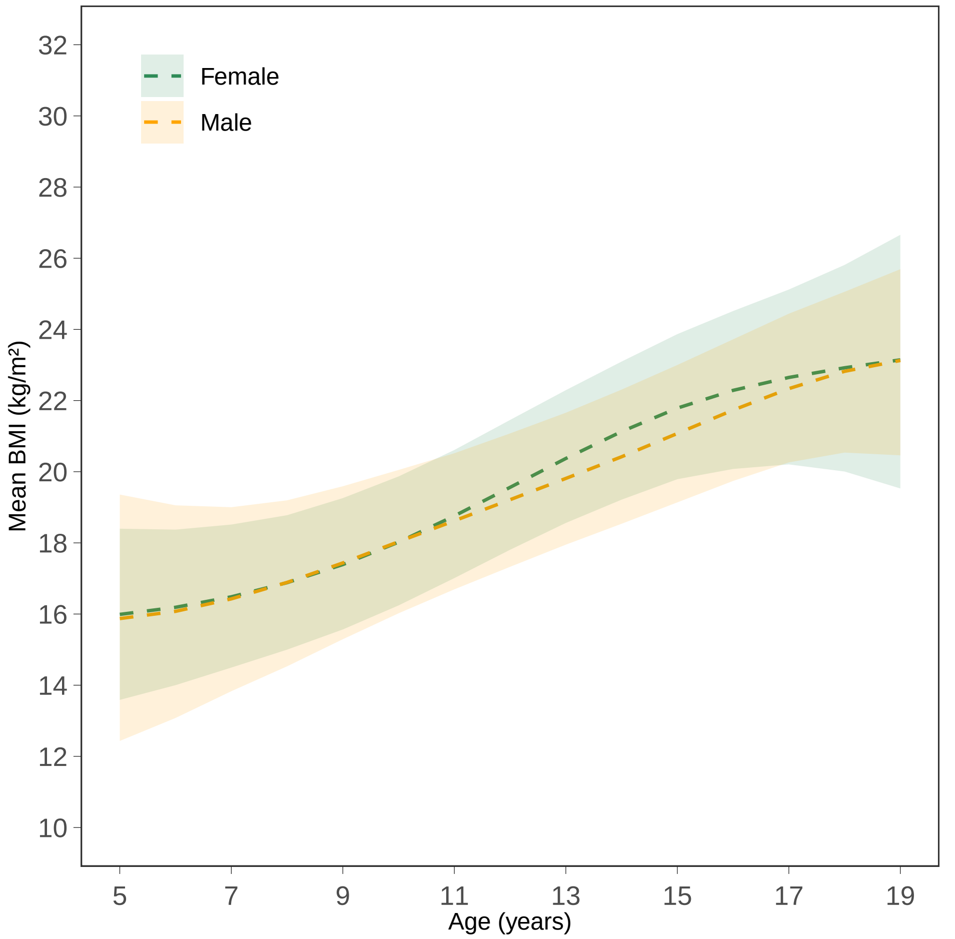

Taiwan (province of China)

Time trends in height of 19 year olds

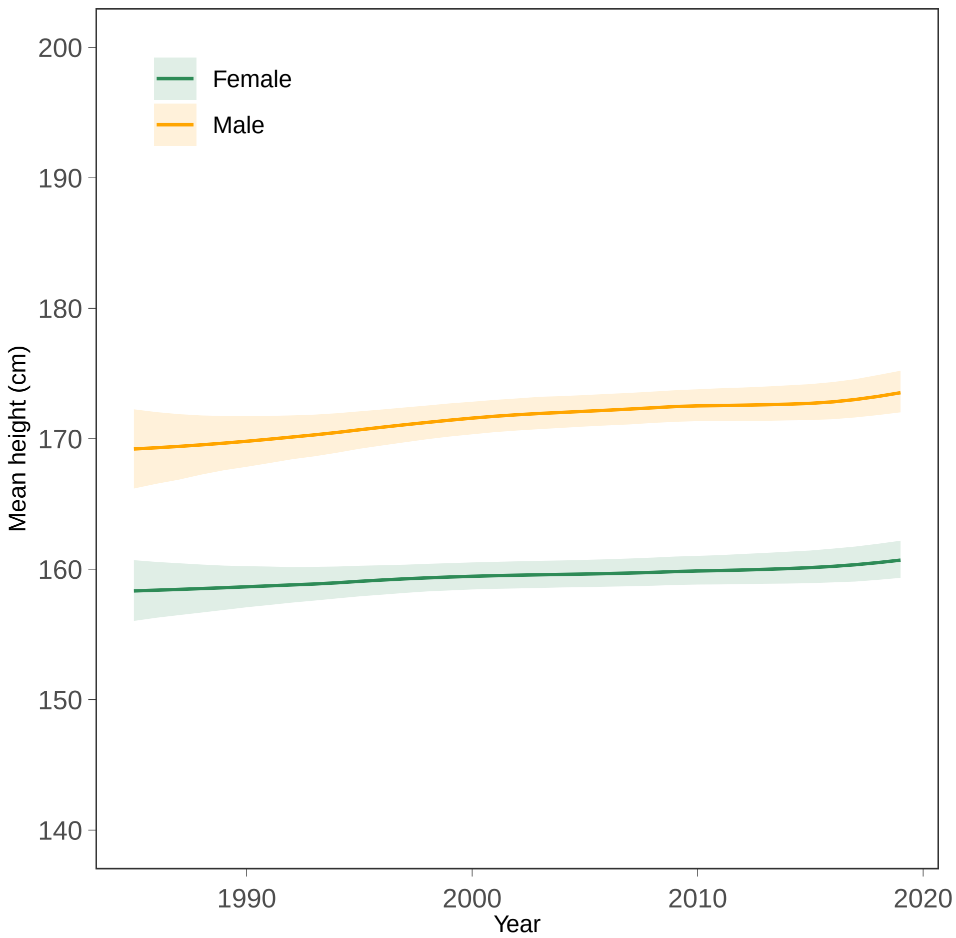

Time trends in BMI of 19 year olds

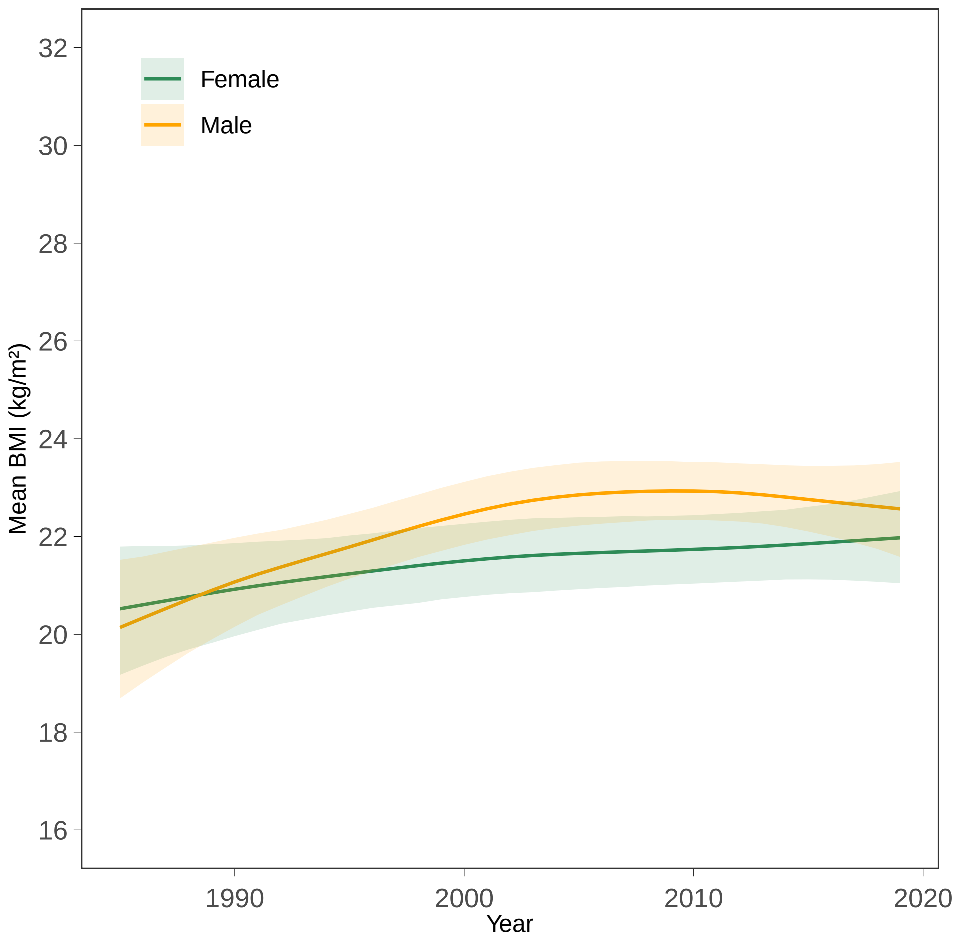

Height−for−age trajectories (2000 birth cohort)

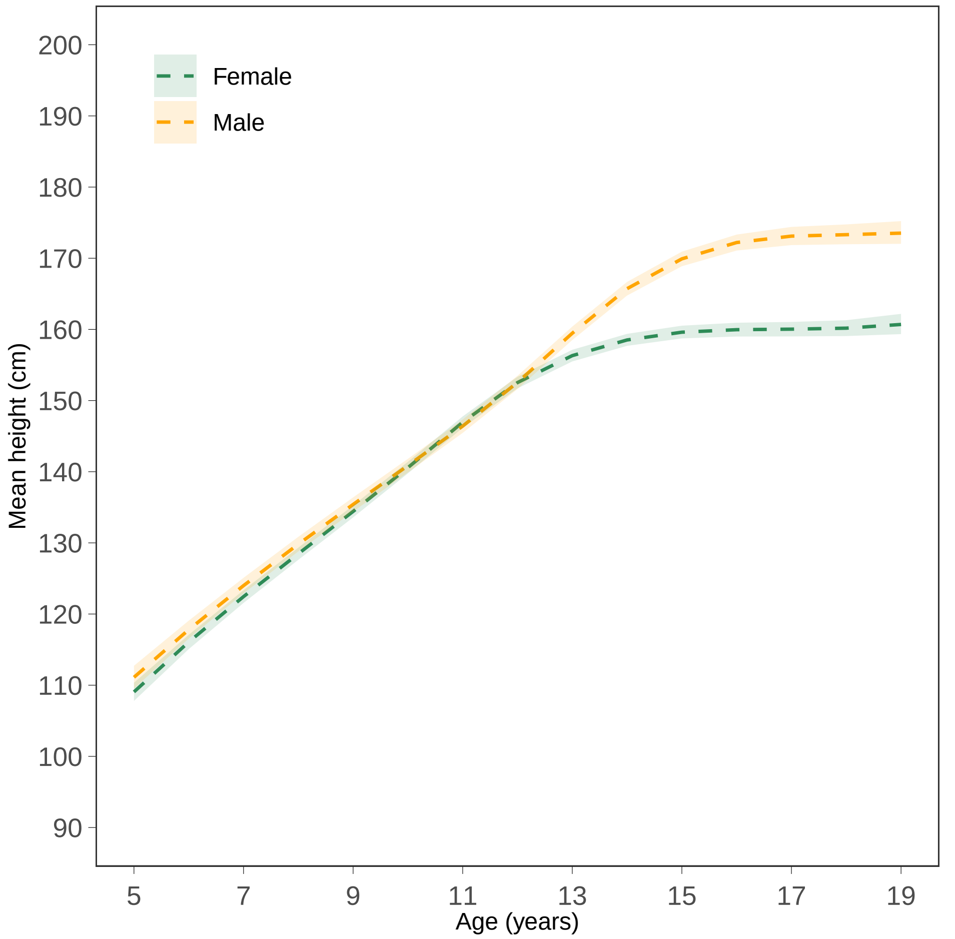

BMI−for−age trajectories (2000 birth cohort)

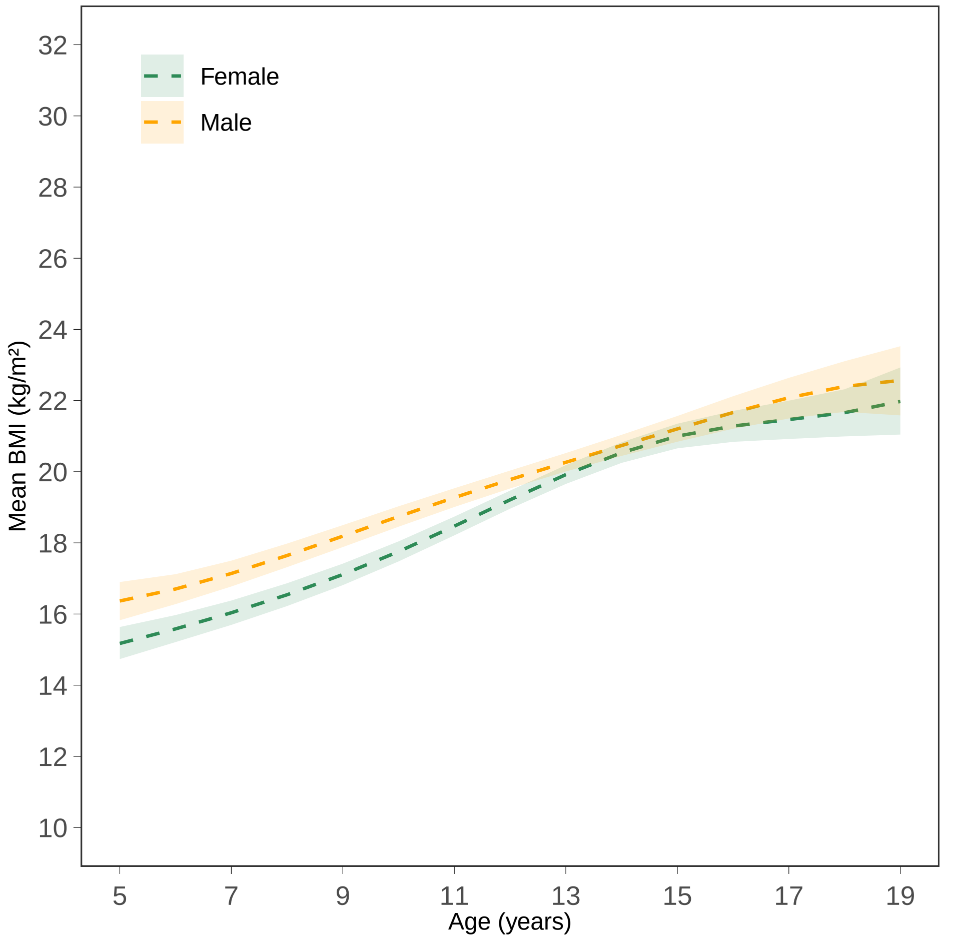

Tajikistan

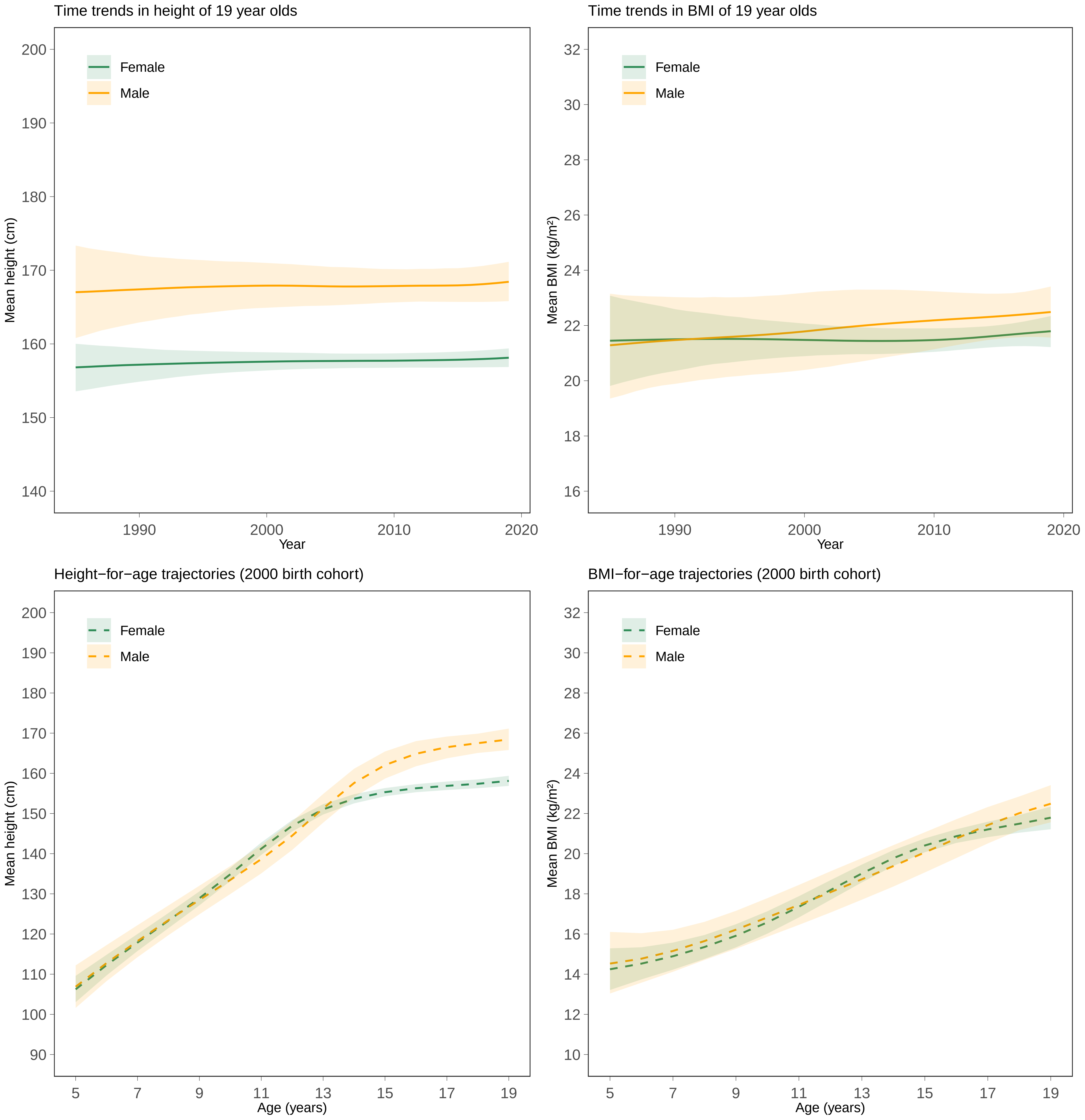

Time trends in height of 19 year olds
Female
Male
Mean height (cm)
Year
Time trends in BMI of 19 year olds
Mean BMI (kg/m²)
Height−for−age trajectories (2000 birth cohort)
Age (years)
BMI−for−age trajectories (2000 birth cohort)

Tanzania

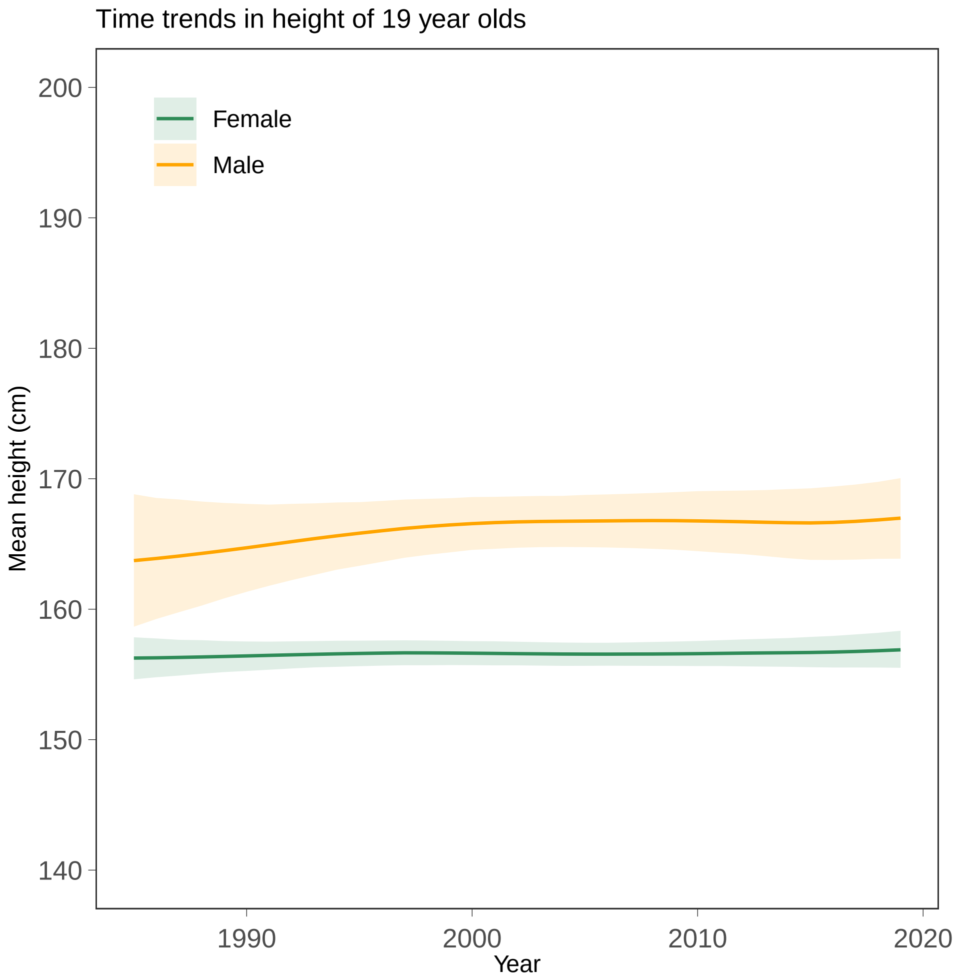

Time trends in height of 19 year olds
Female
Male
Mean height (cm)
200
190
180
170
160
150
140
1990
2000
2010
2020
Year

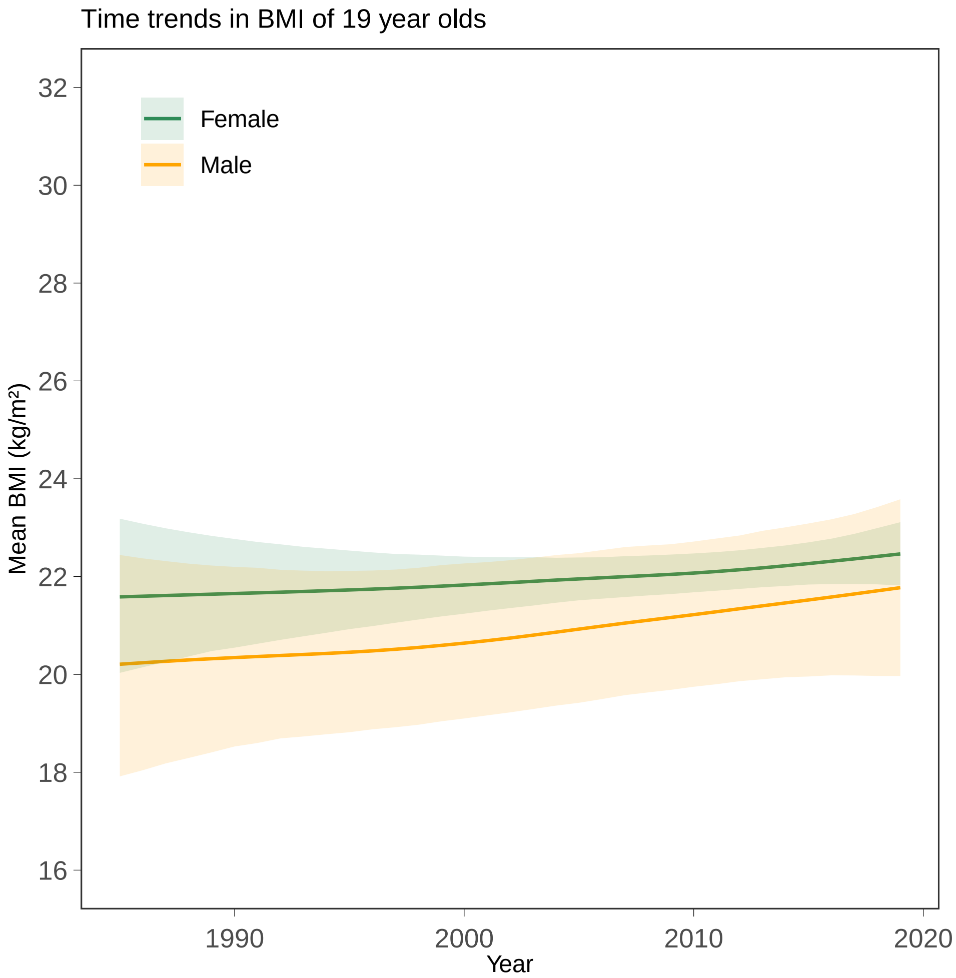

Time trends in BMI of 19 year olds
Female
Male
Mean BMI (kg/m²)
32
30
28
26
24
22
20
18
16
1990
2000
2010
2020
Year

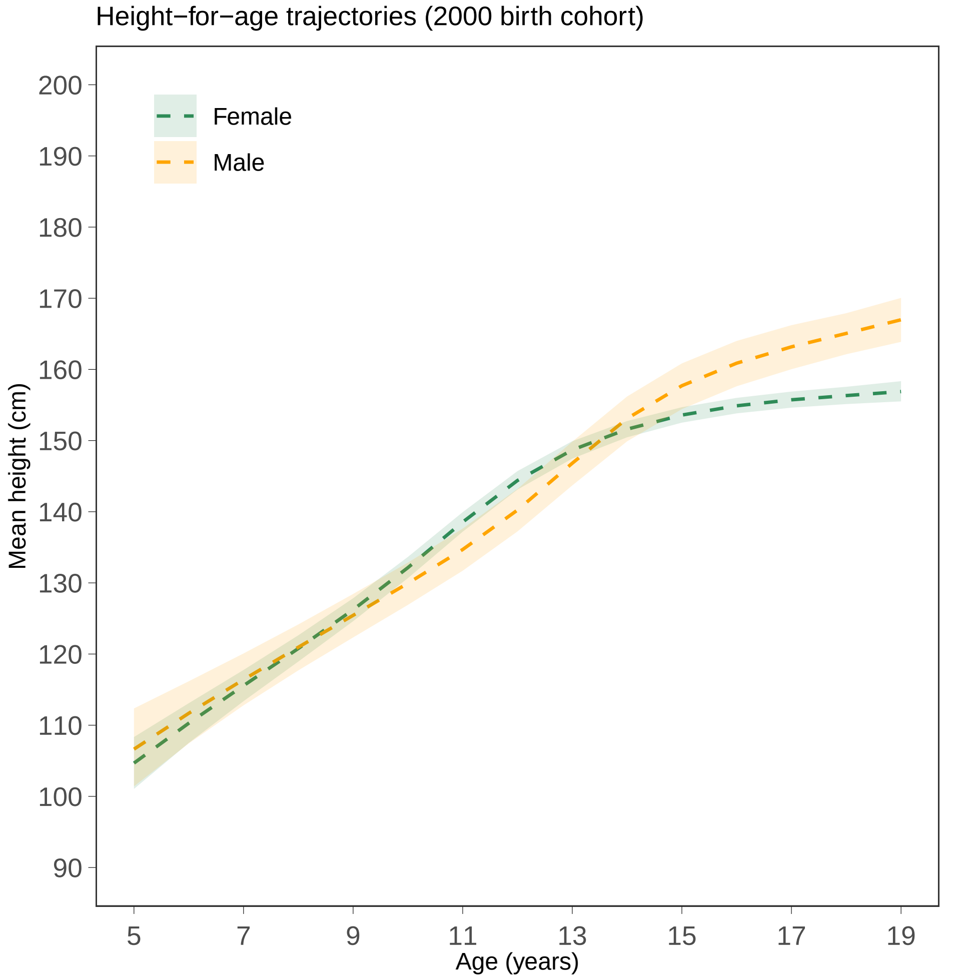

Height−for−age trajectories (2000 birth cohort)
Female
Male
Mean height (cm)
200
190
180
170
160
150
140
130
120
110
100
90
5
7
9
11
13
15
17
19
Age (years)

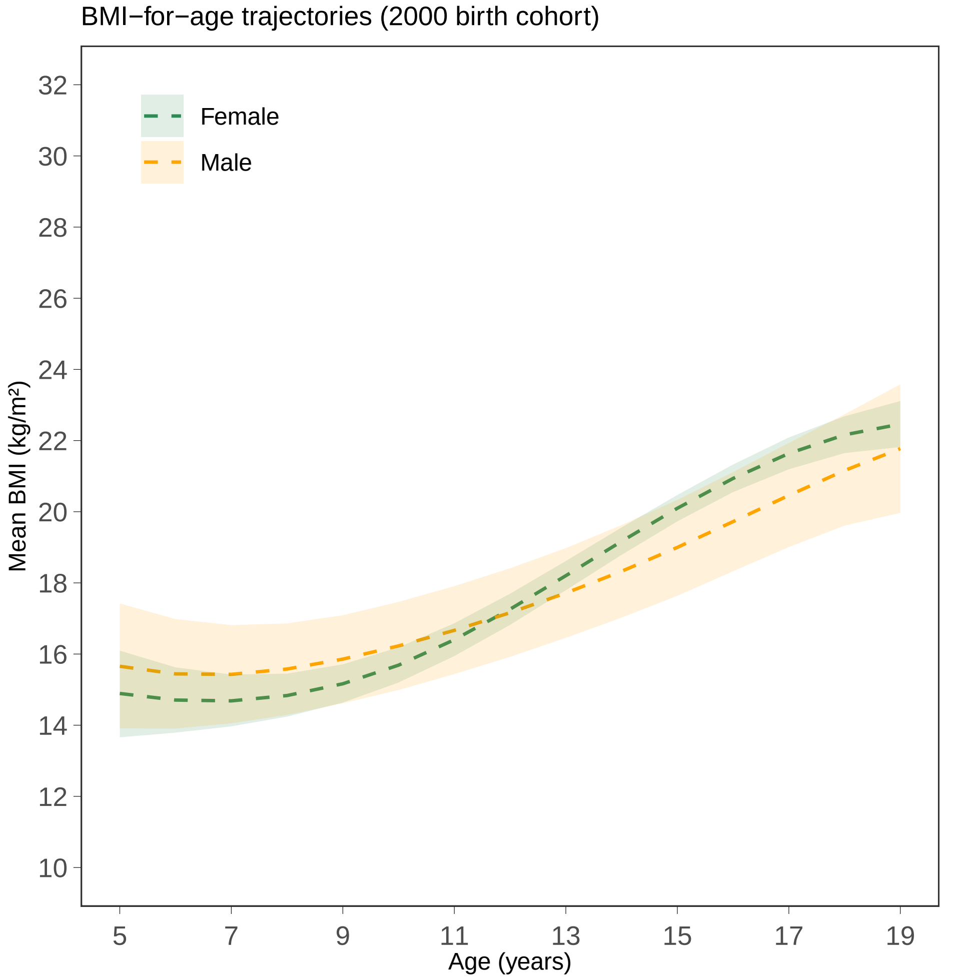

BMI−for−age trajectories (2000 birth cohort)
Female
Male
Mean BMI (kg/m²)
32
30
28
26
24
22
20
18
16
14
12
10
5
7
9
11
13
15
17
19
Age (years)

Thailand

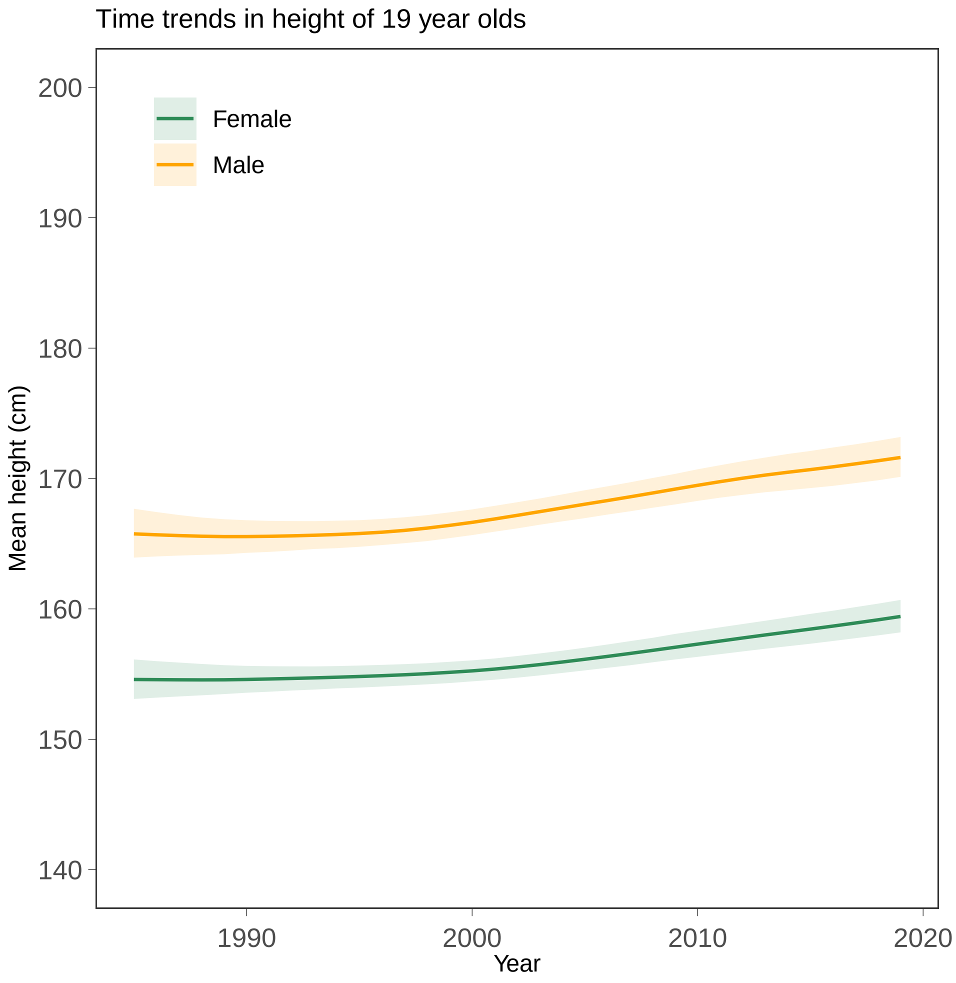
Time trends in height of 19 year olds
Female
Male
Mean height (cm)
200
190
180
170
160
150
140
1990
2000
2010
2020
Year

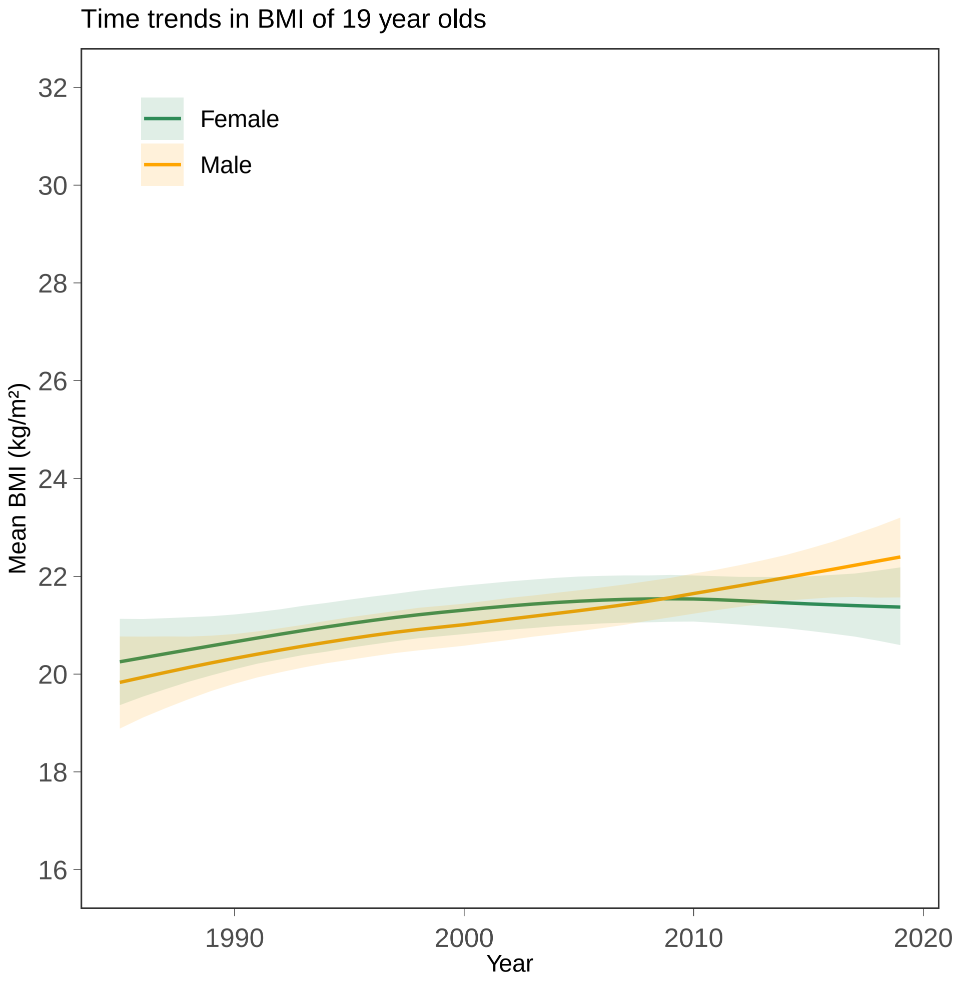
Time trends in BMI of 19 year olds
Female
Male
Mean BMI (kg/m²)
32
30
28
26
24
22
20
18
16
1990
2000
2010
2020
Year

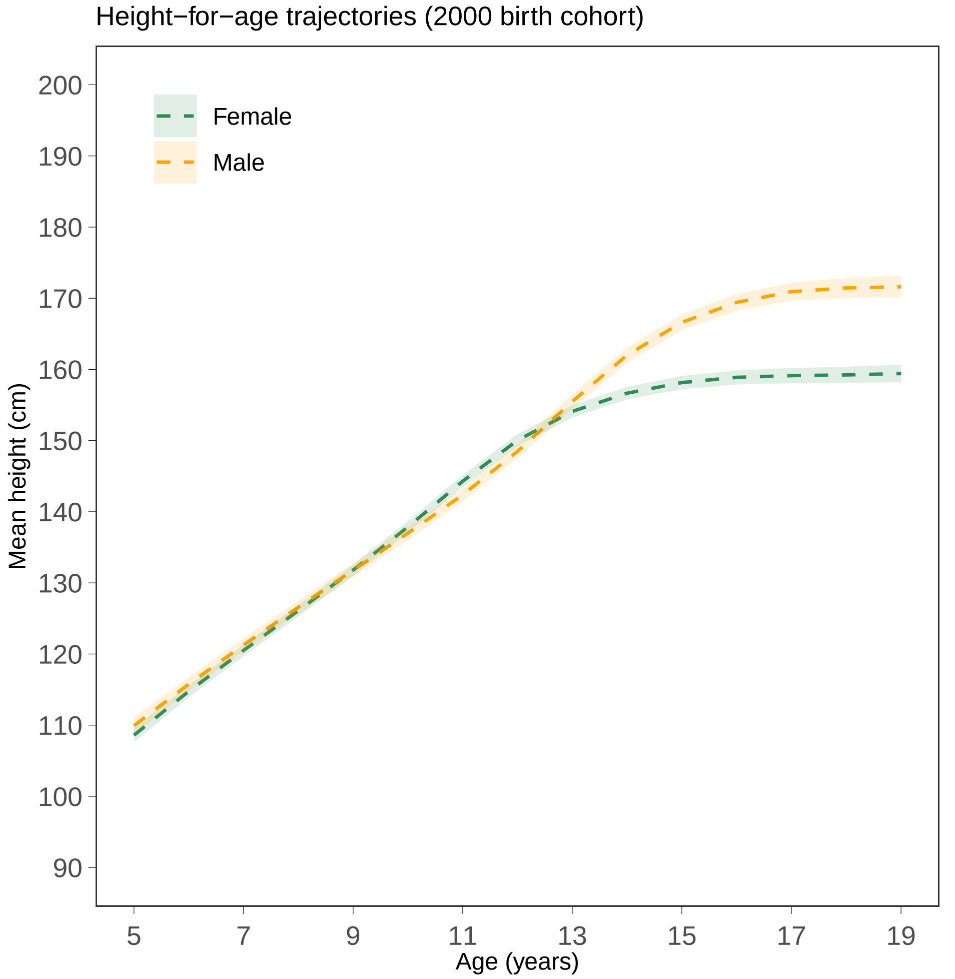
Height−for−age trajectories (2000 birth cohort)
Female
Male
Mean height (cm)
200
190
180
170
160
150
140
130
120
110
100
90
5
7
9
11
13
15
17
19
Age (years)

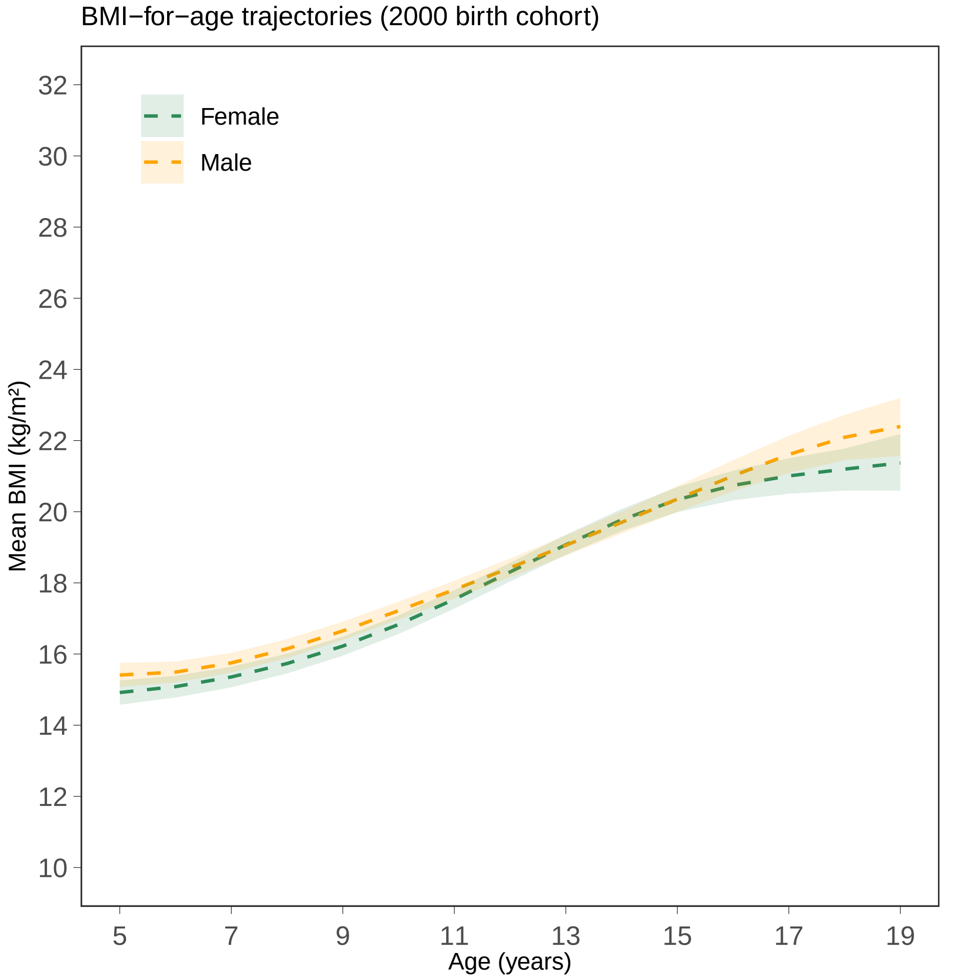
BMI−for−age trajectories (2000 birth cohort)
Female
Male
Mean BMI (kg/m²)
32
30
28
26
24
22
20
18
16
14
12
10
5
7
9
11
13
15
17
19
Age (years)

The Gambia

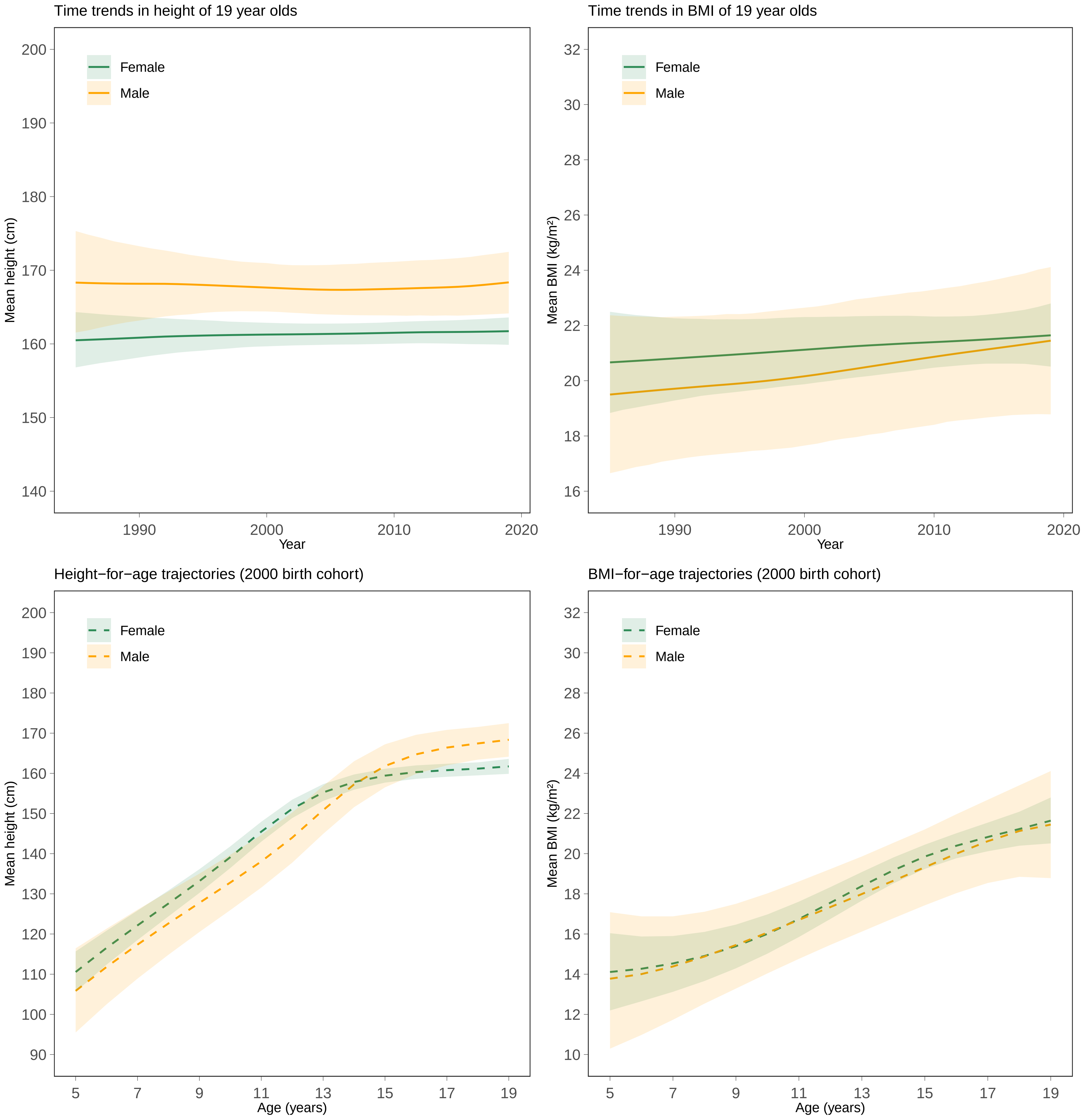
Time trends in height of 19 year olds
Female
Male
Mean height (cm)
200
190
180
170
160
150
140
1990
2000
2010
2020
Year
Time trends in BMI of 19 year olds
Female
Male
Mean BMI (kg/m²)
32
30
28
26
24
22
20
18
16
1990
2000
2010
2020
Year
Height−for−age trajectories (2000 birth cohort)
Female
Male
Mean height (cm)
200
190
180
170
160
150
140
130
120
110
100
90
5
7
9
11
13
15
17
19
Age (years)
BMI−for−age trajectories (2000 birth cohort)
Female
Male
Mean BMI (kg/m²)
32
30
28
26
24
22
20
18
16
14
12
10
5
7
9
11
13
15
17
19
Age (years)

Timor−Leste

Time trends in height of 19 year olds

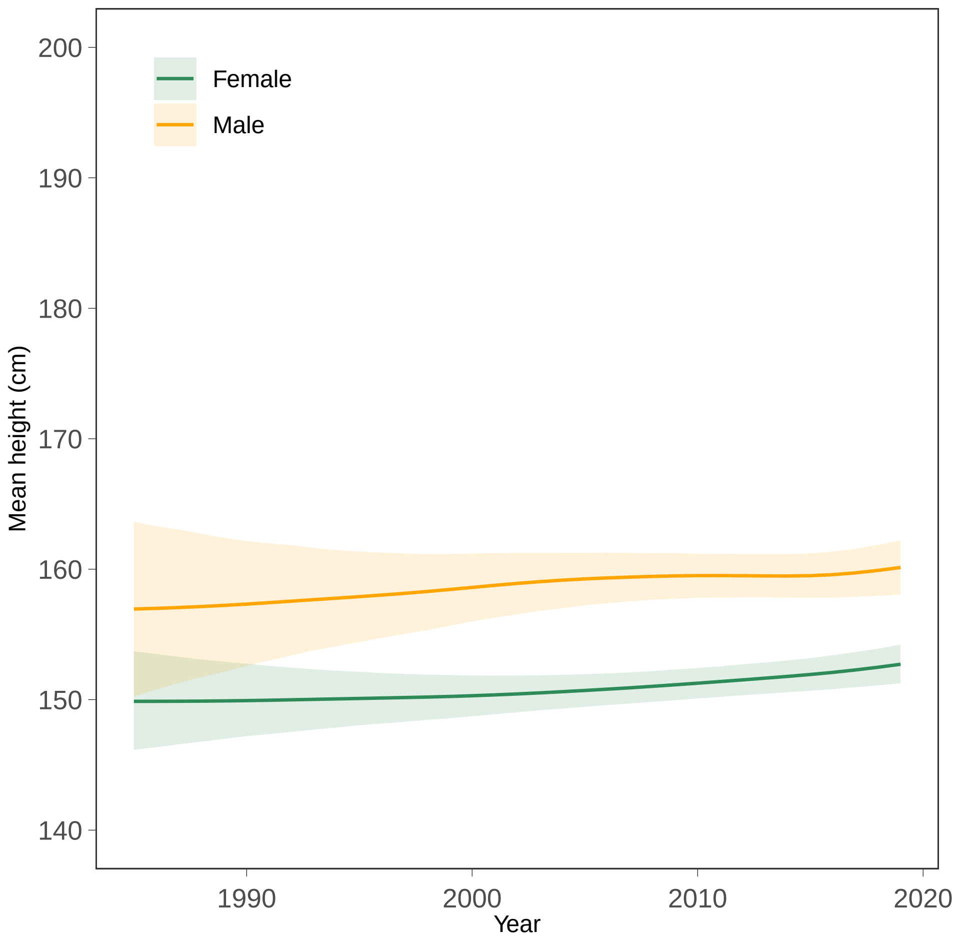

Time trends in BMI of 19 year olds

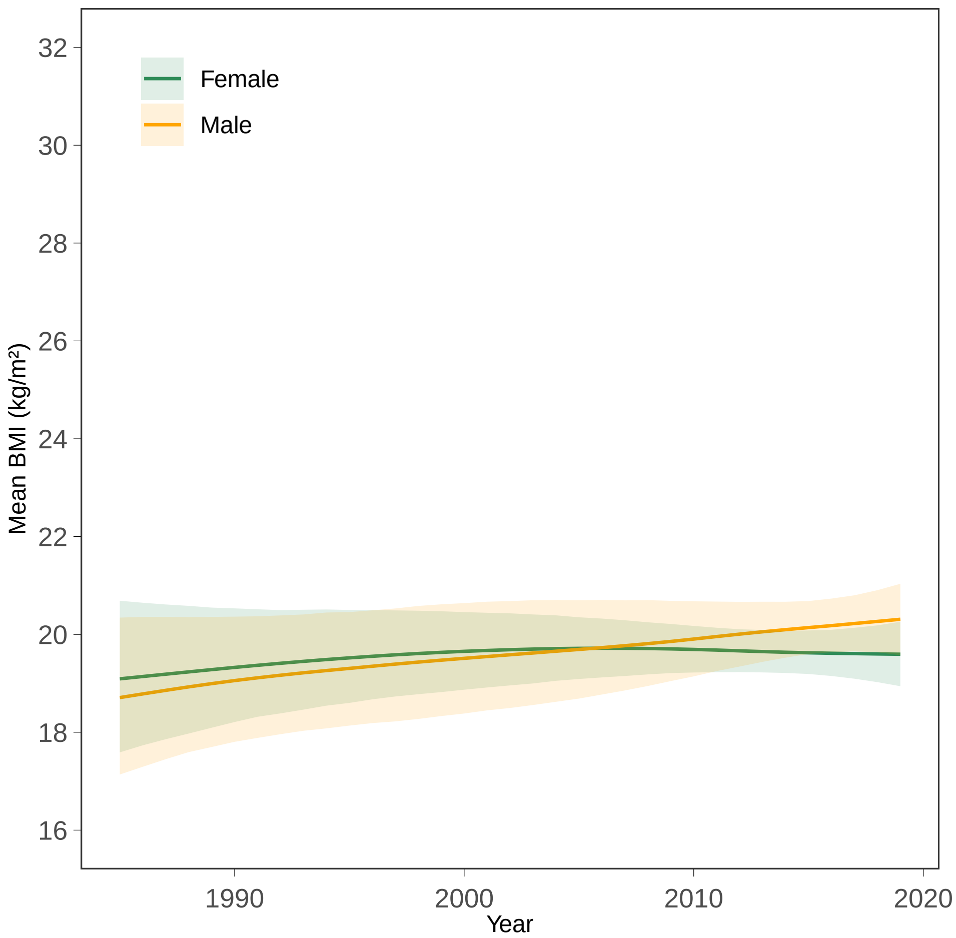

Height−for−age trajectories (2000 birth cohort)

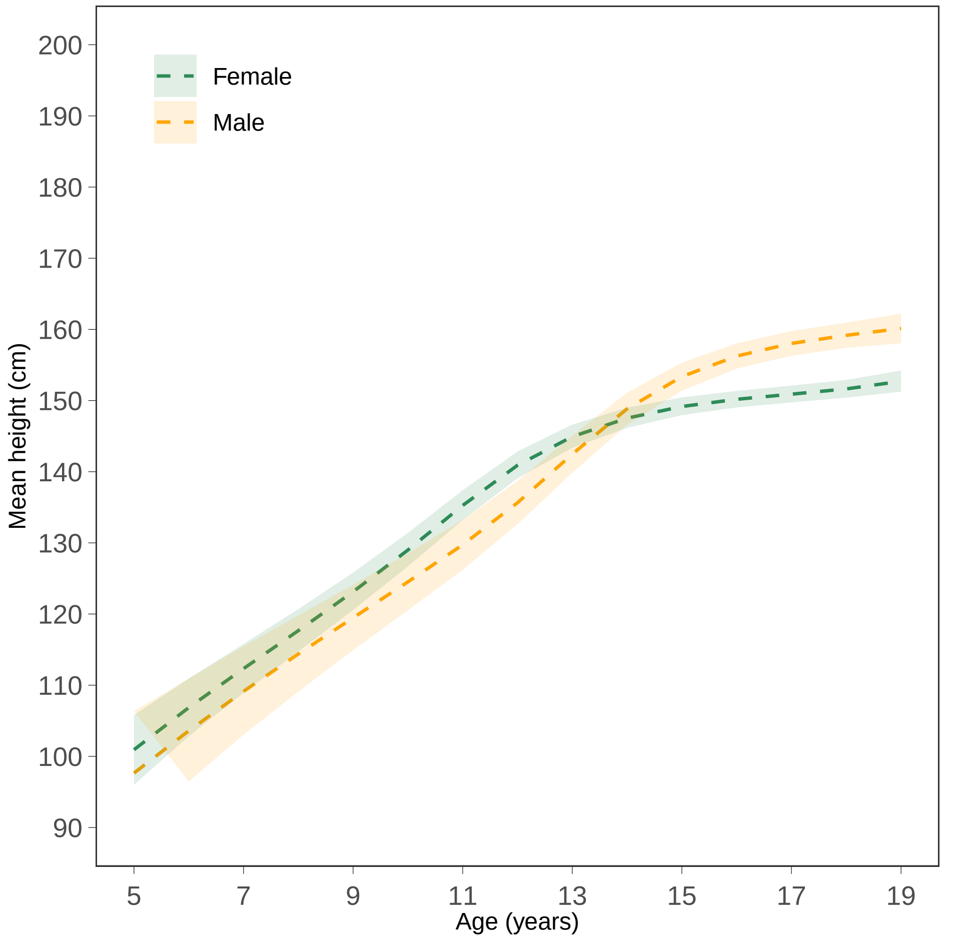

BMI−for−age trajectories (2000 birth cohort)

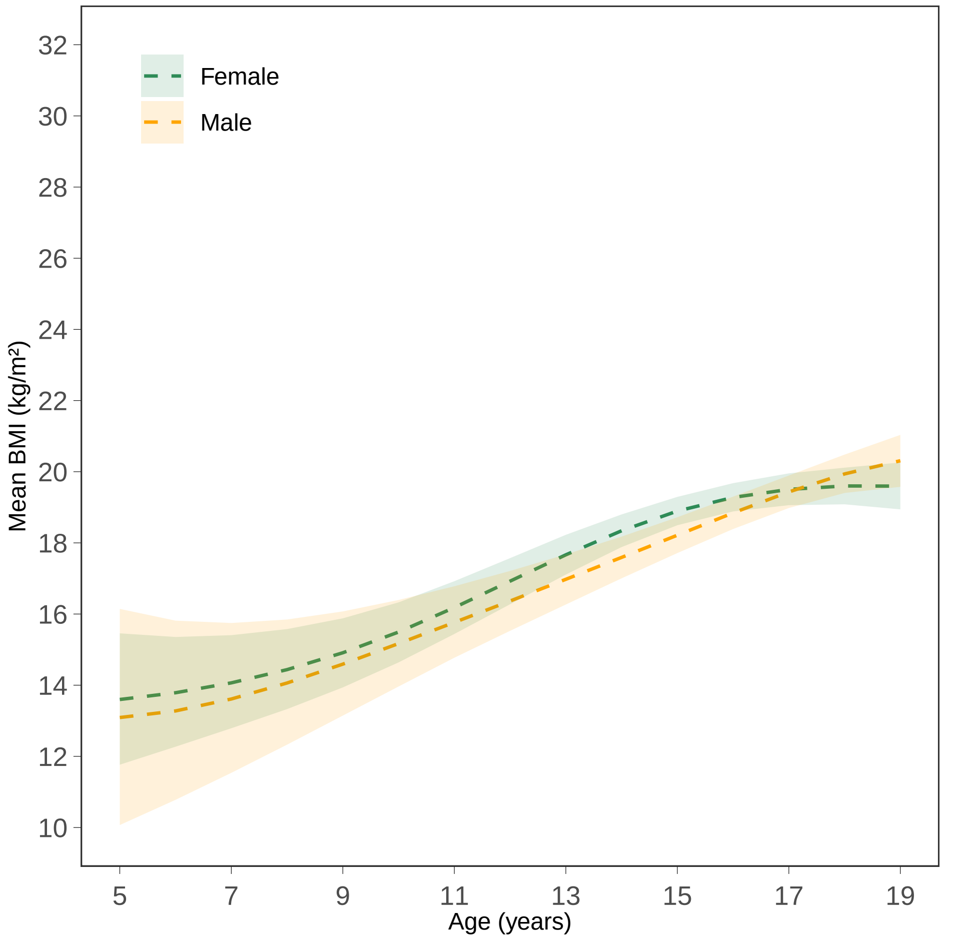

Togo

Time trends in height of 19 year olds

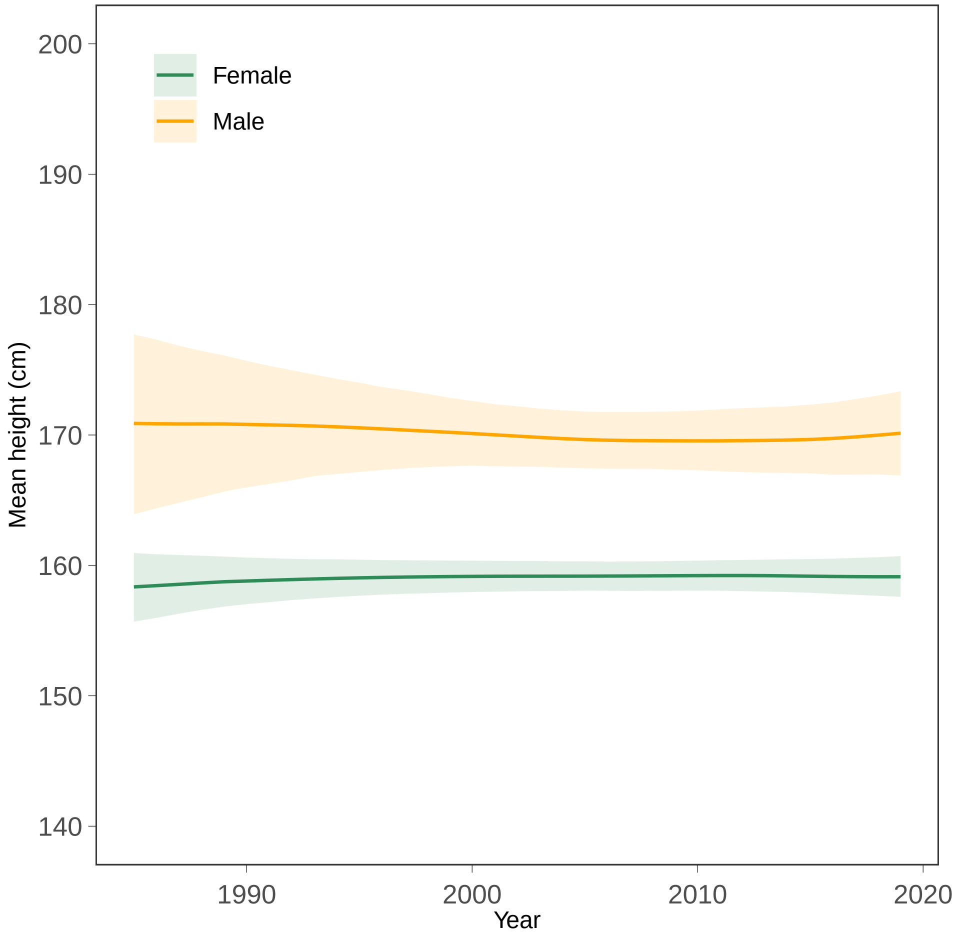

Time trends in BMI of 19 year olds

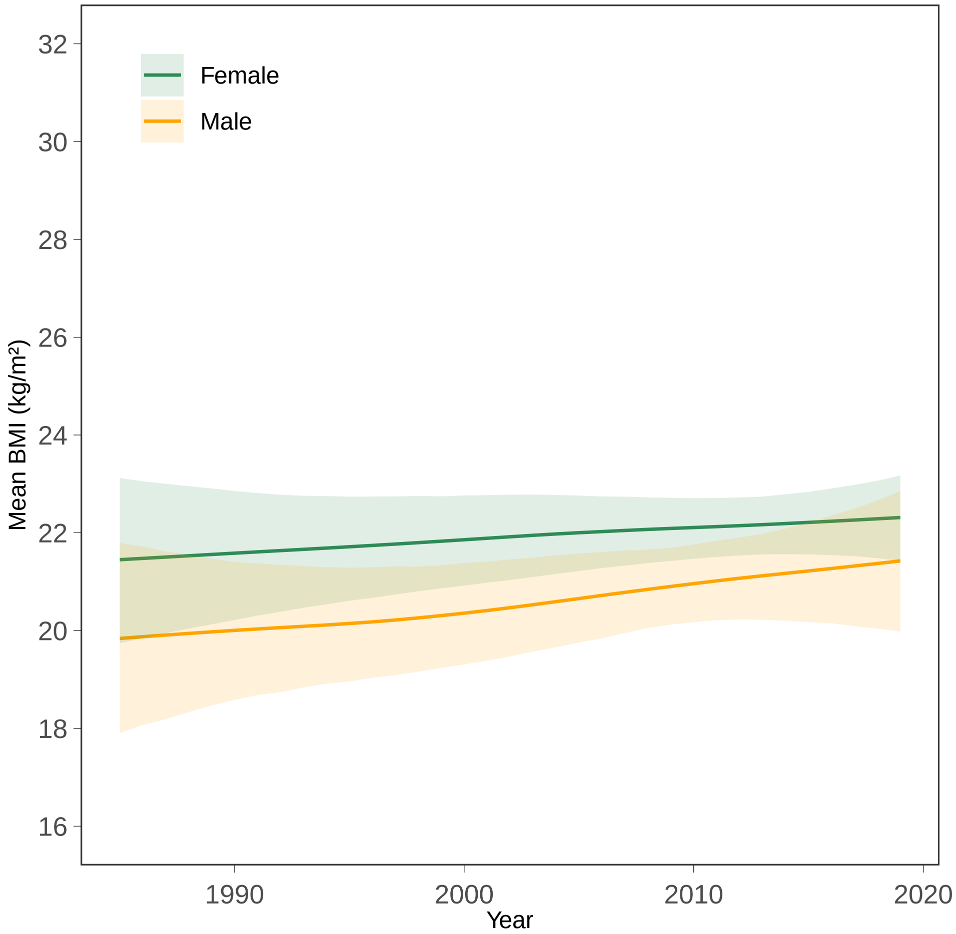

Height−for−age trajectories (2000 birth cohort)

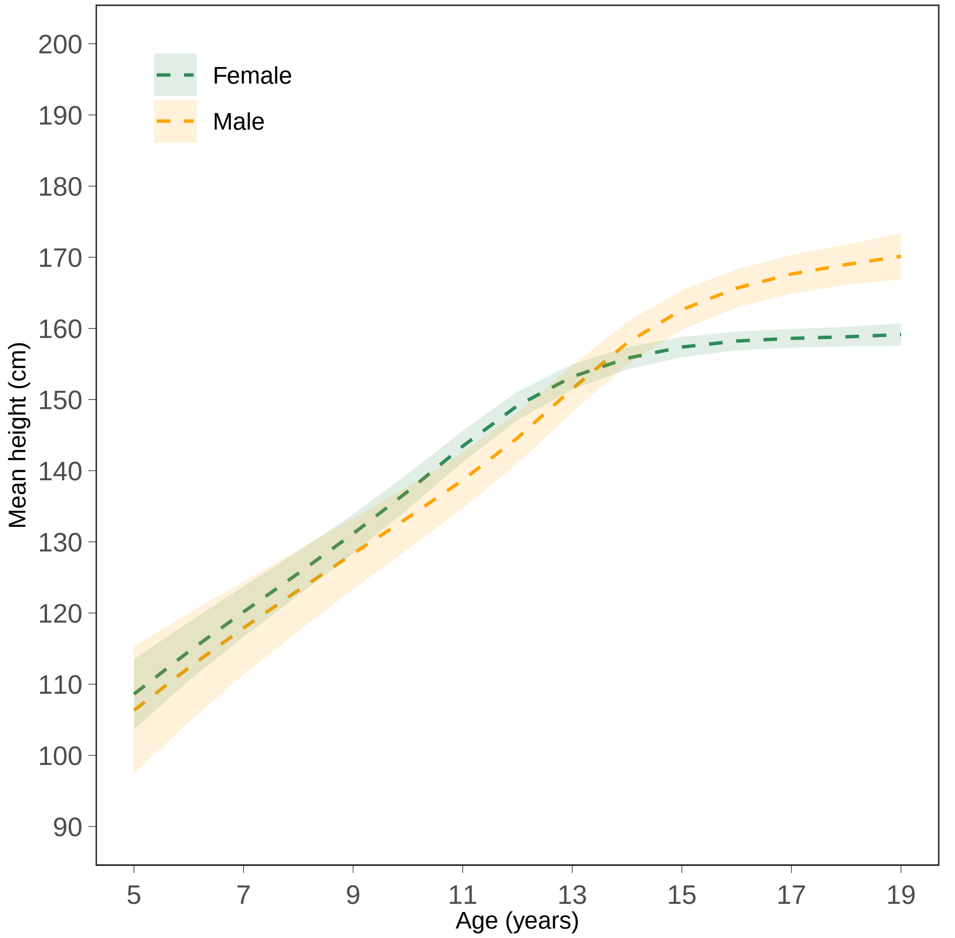

BMI−for−age trajectories (2000 birth cohort)

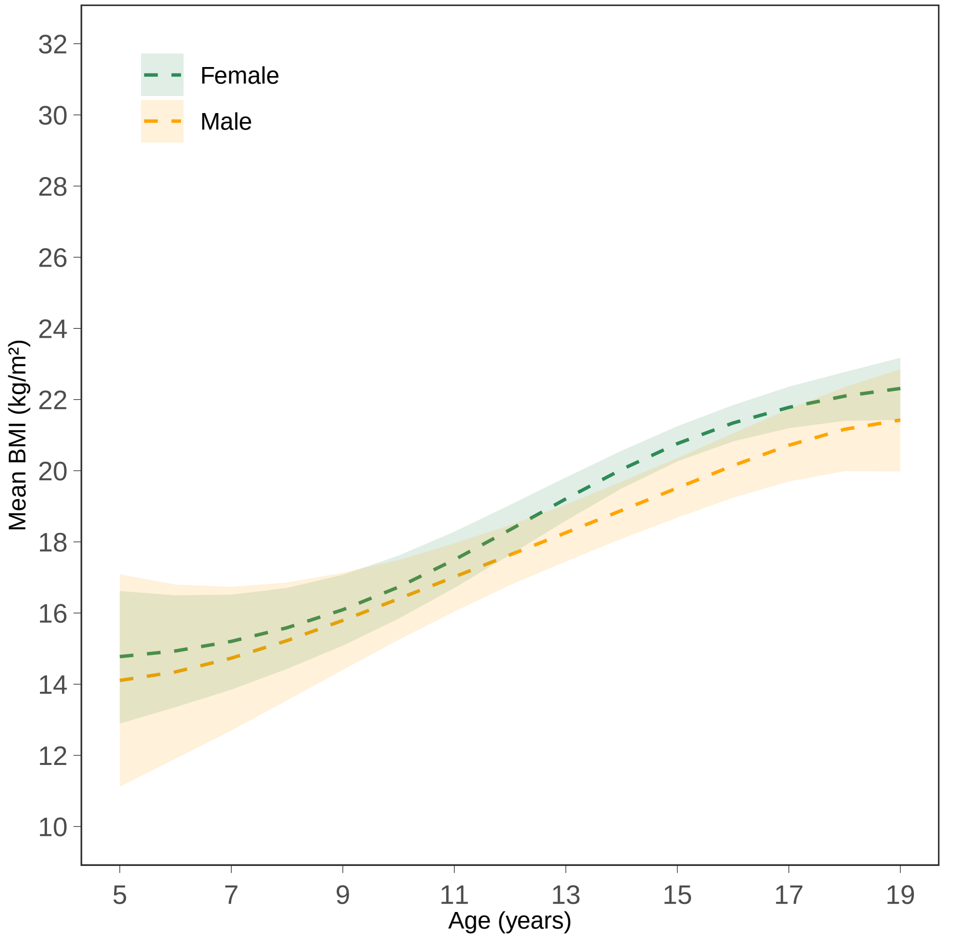

Tokelau

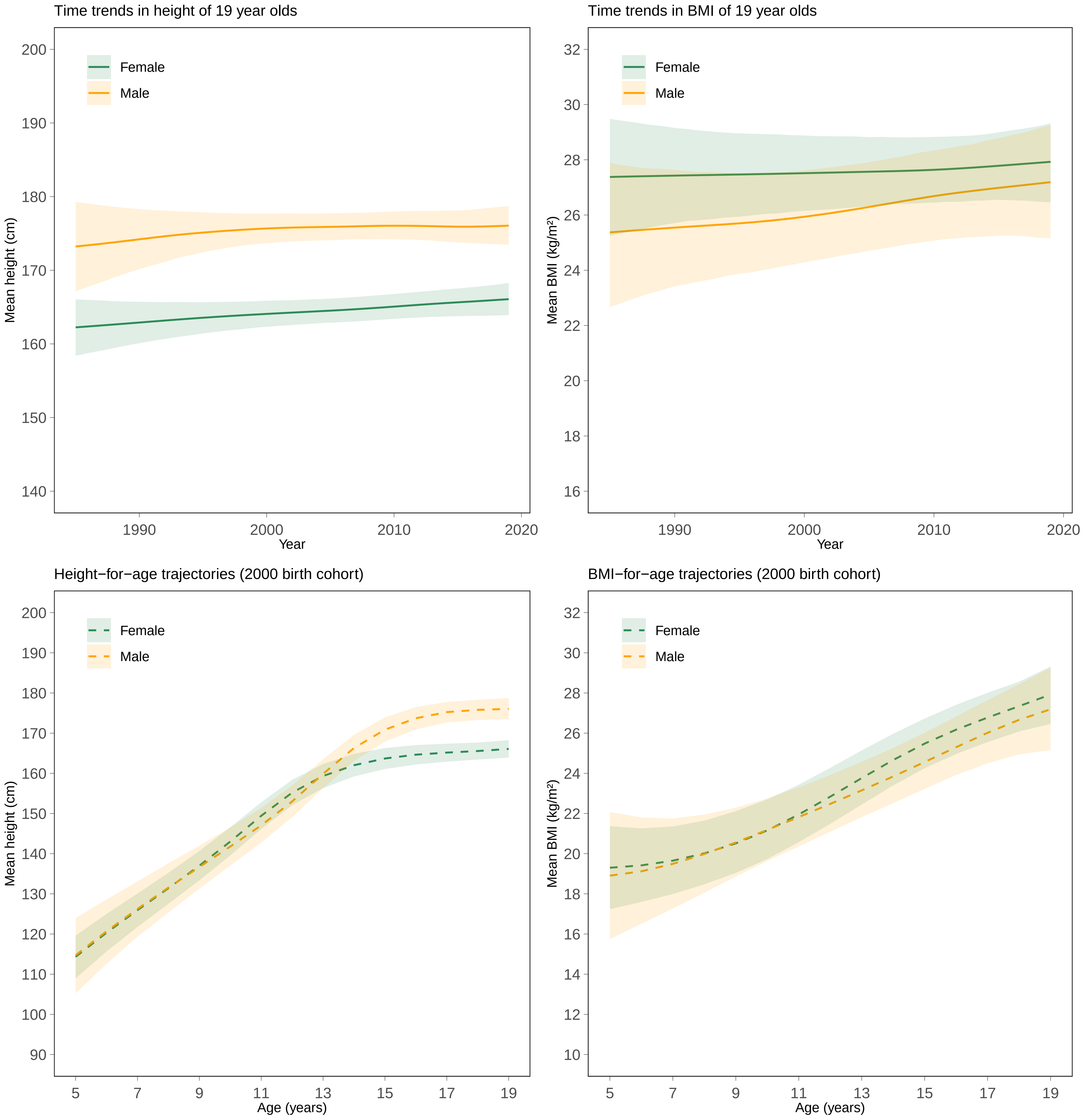
Time trends in height of 19 year olds
Female
Male
Mean height (cm)
Year
Time trends in BMI of 19 year olds
Female
Male
Mean BMI (kg/m²)
Year
Height−for−age trajectories (2000 birth cohort)
Female
Male
Mean height (cm)
Age (years)
BMI−for−age trajectories (2000 birth cohort)
Female
Male
Mean BMI (kg/m²)
Age (years)

Tonga

Time trends in height of 19 year olds

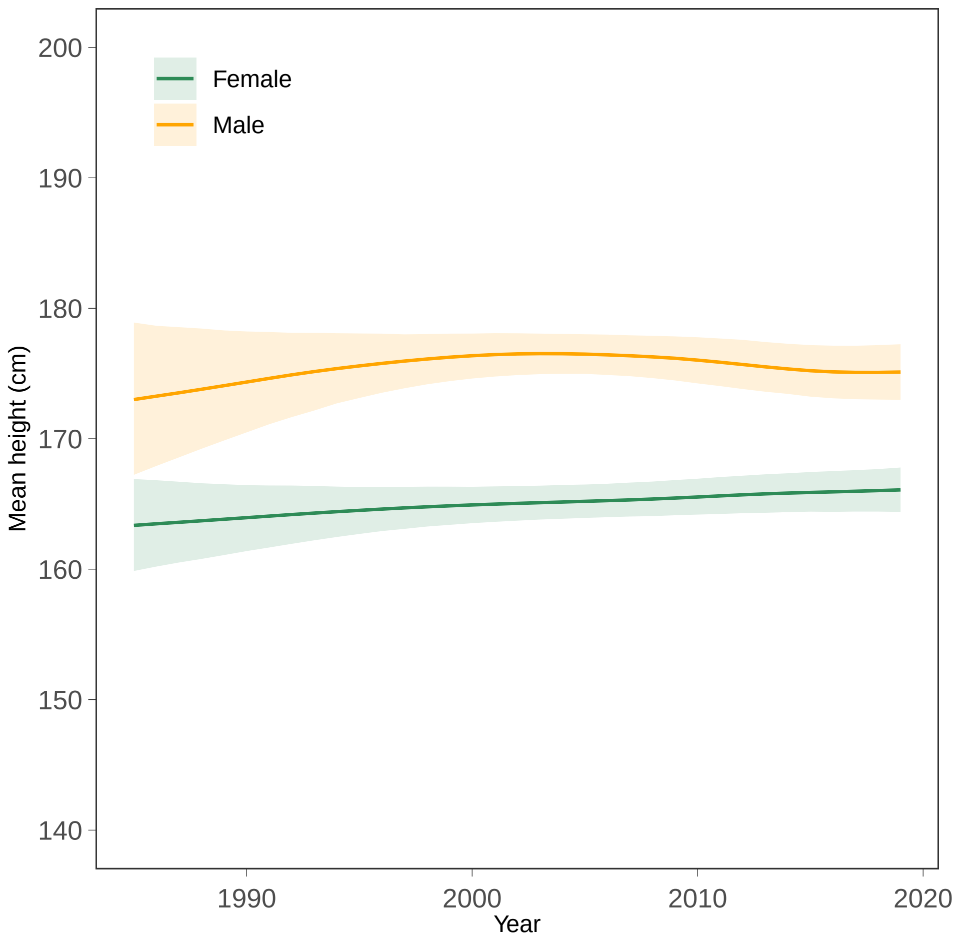

Time trends in BMI of 19 year olds

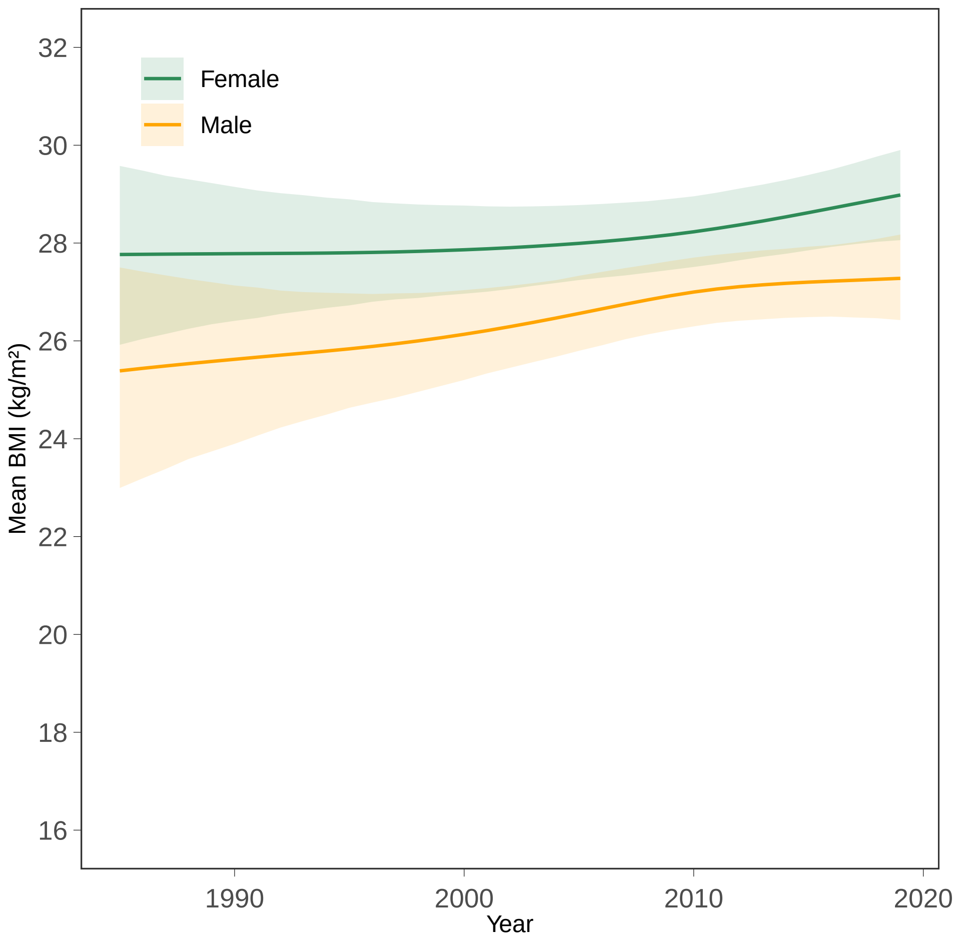

Height−for−age trajectories (2000 birth cohort)

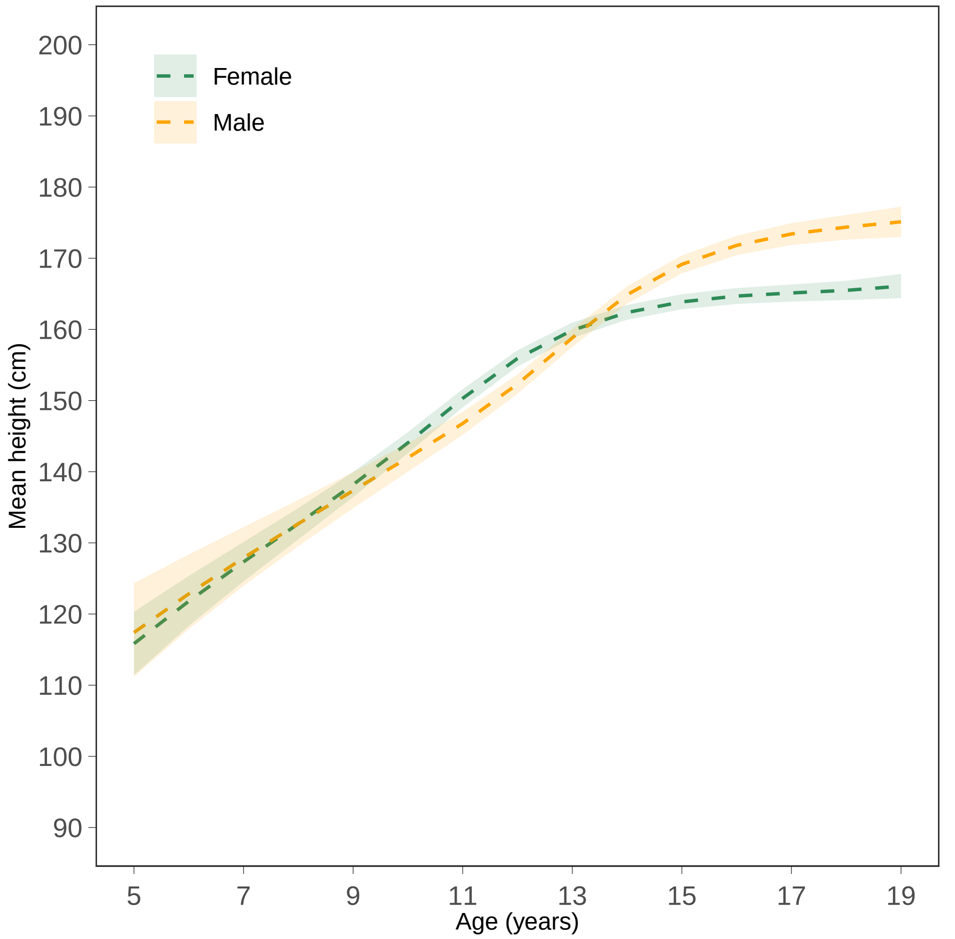

BMI−for−age trajectories (2000 birth cohort)

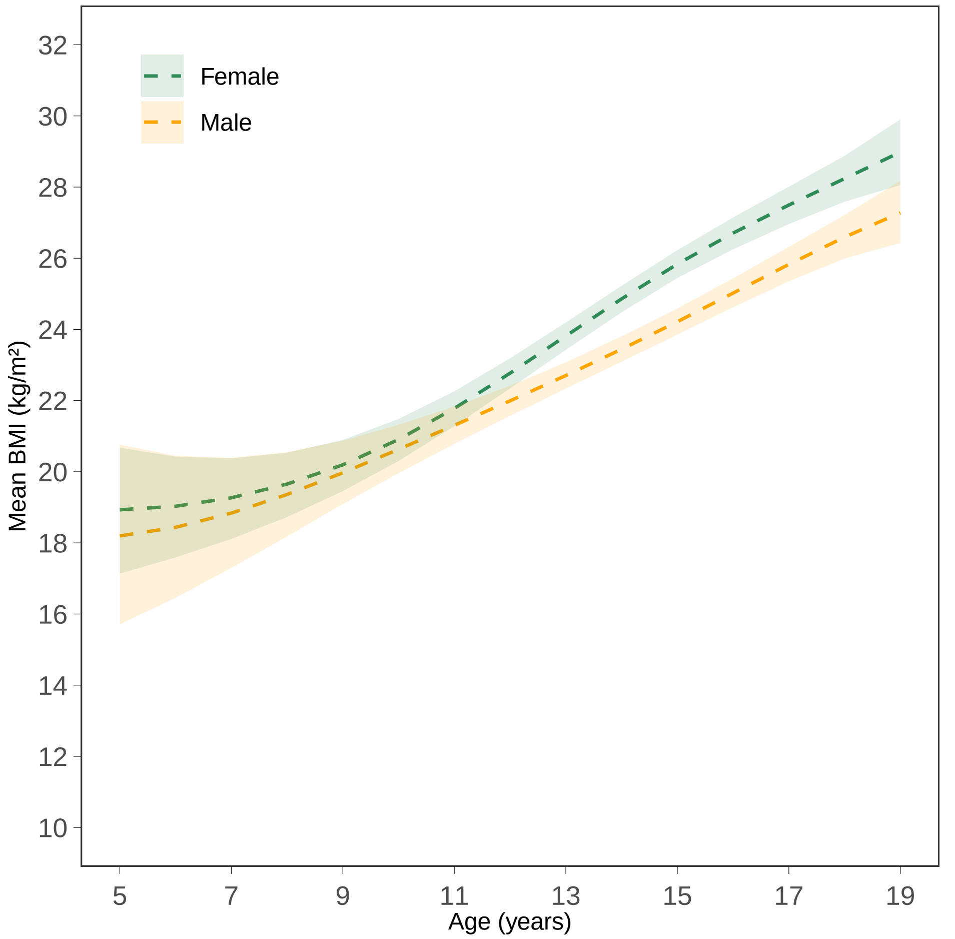

Trinidad and Tobago

Time trends in height of 19 year olds

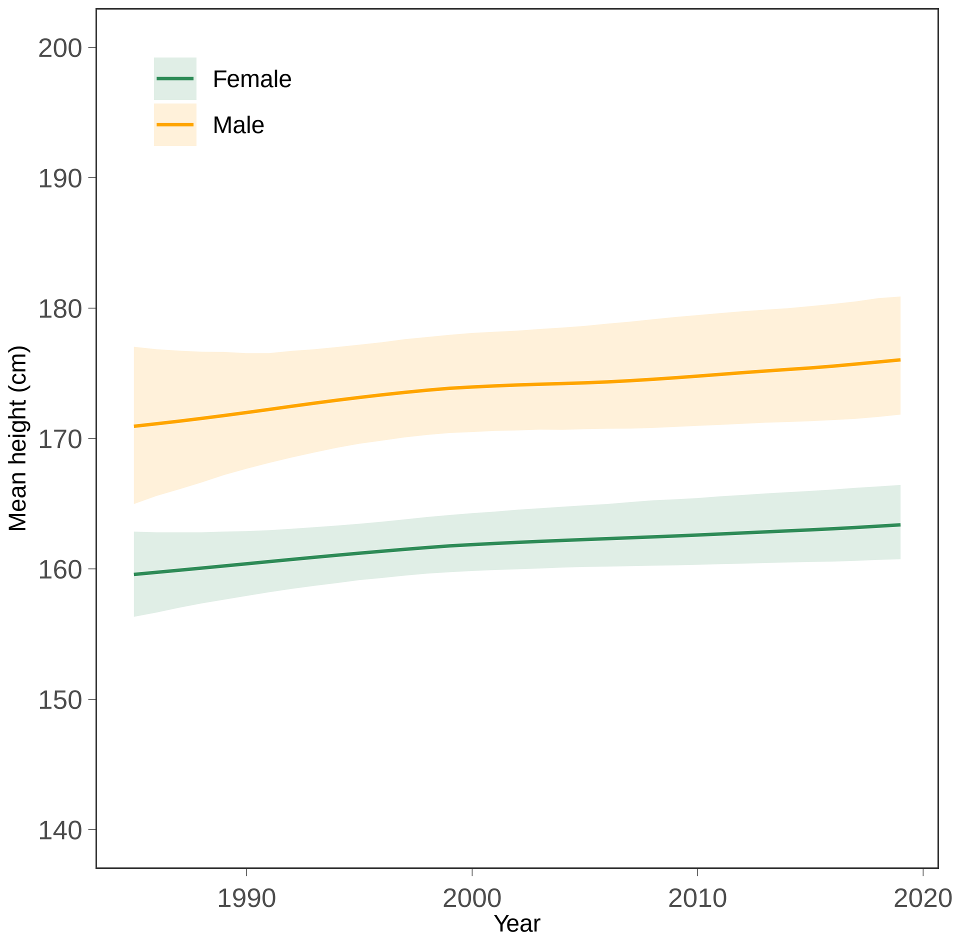

Time trends in BMI of 19 year olds

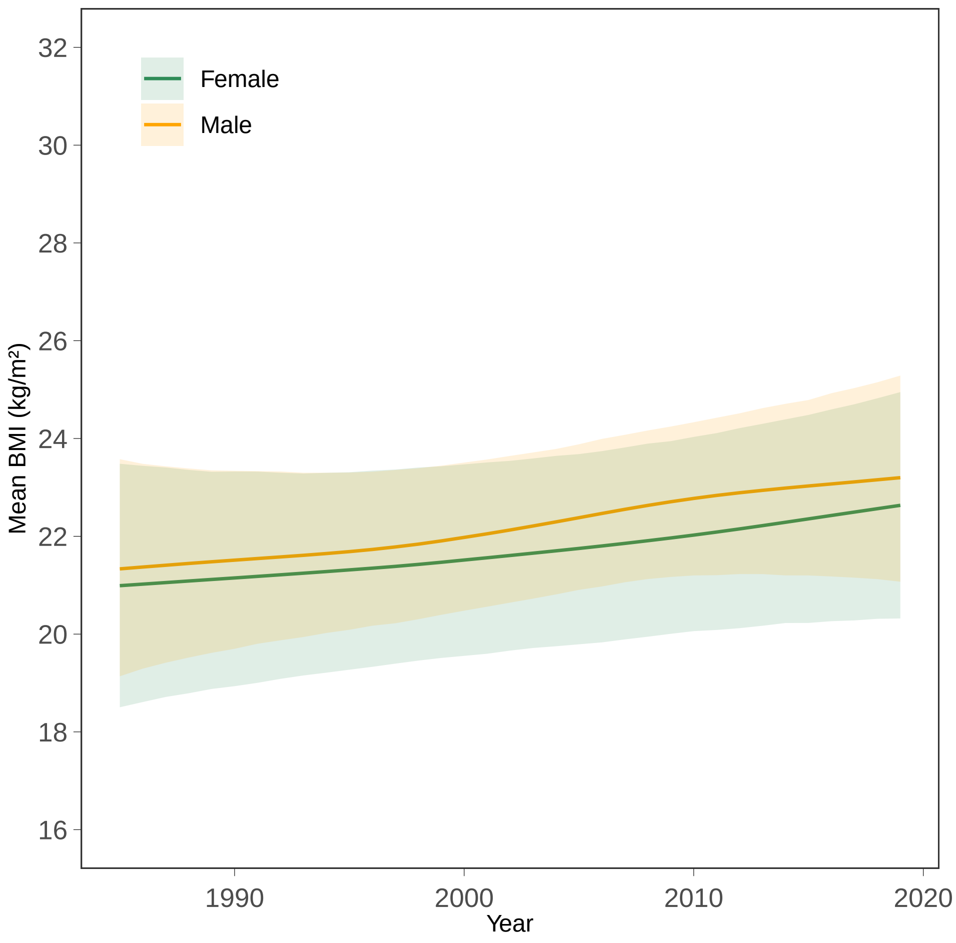

Height−for−age trajectories (2000 birth cohort)

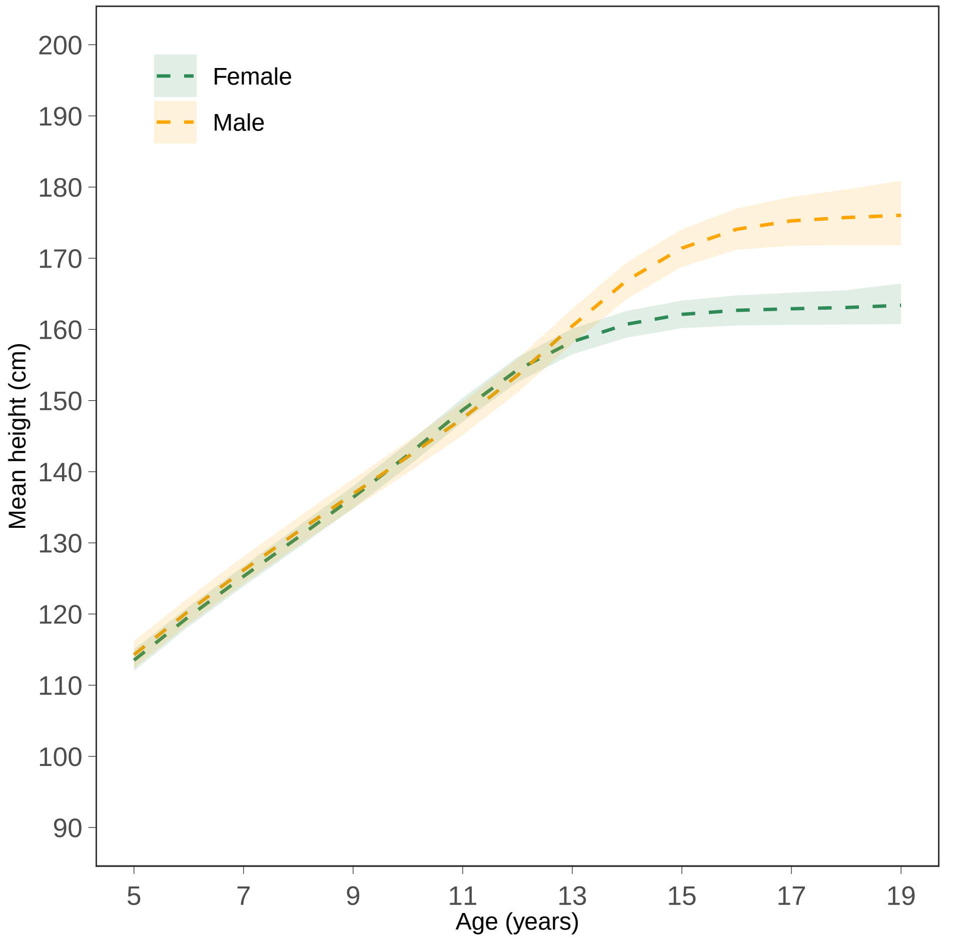

BMI−for−age trajectories (2000 birth cohort)

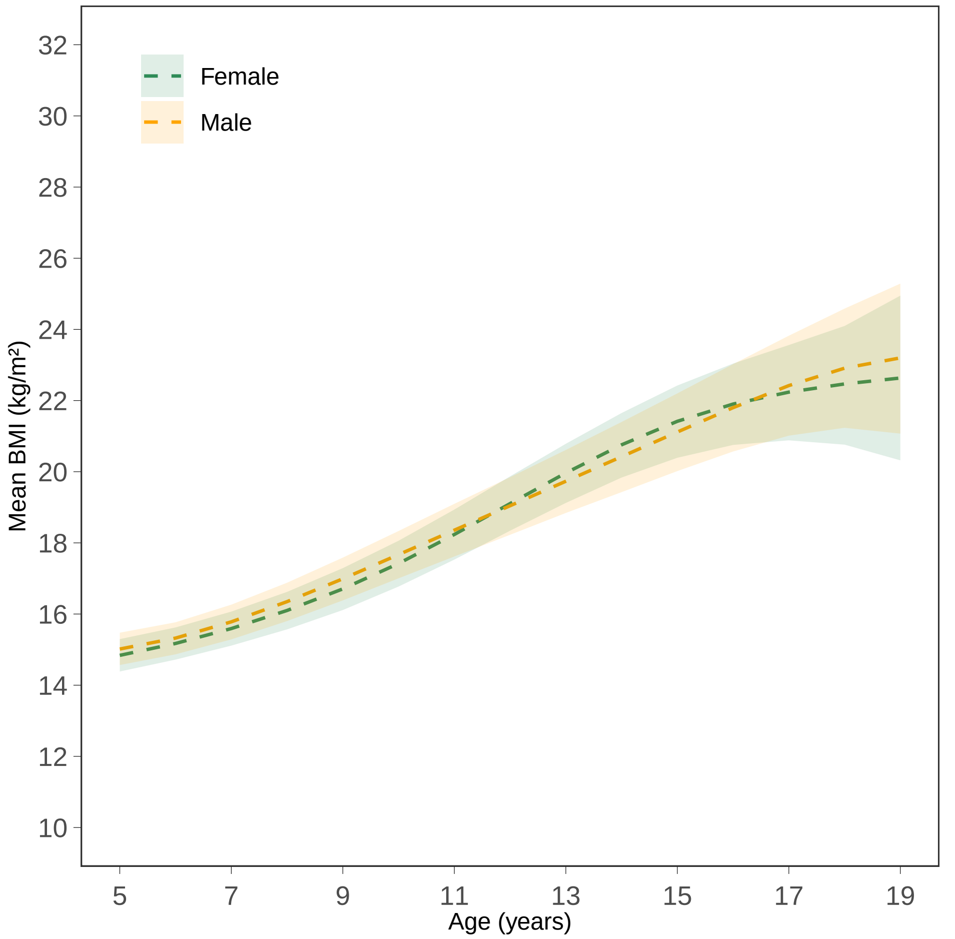

Tunisia

Time trends in height of 19 year olds

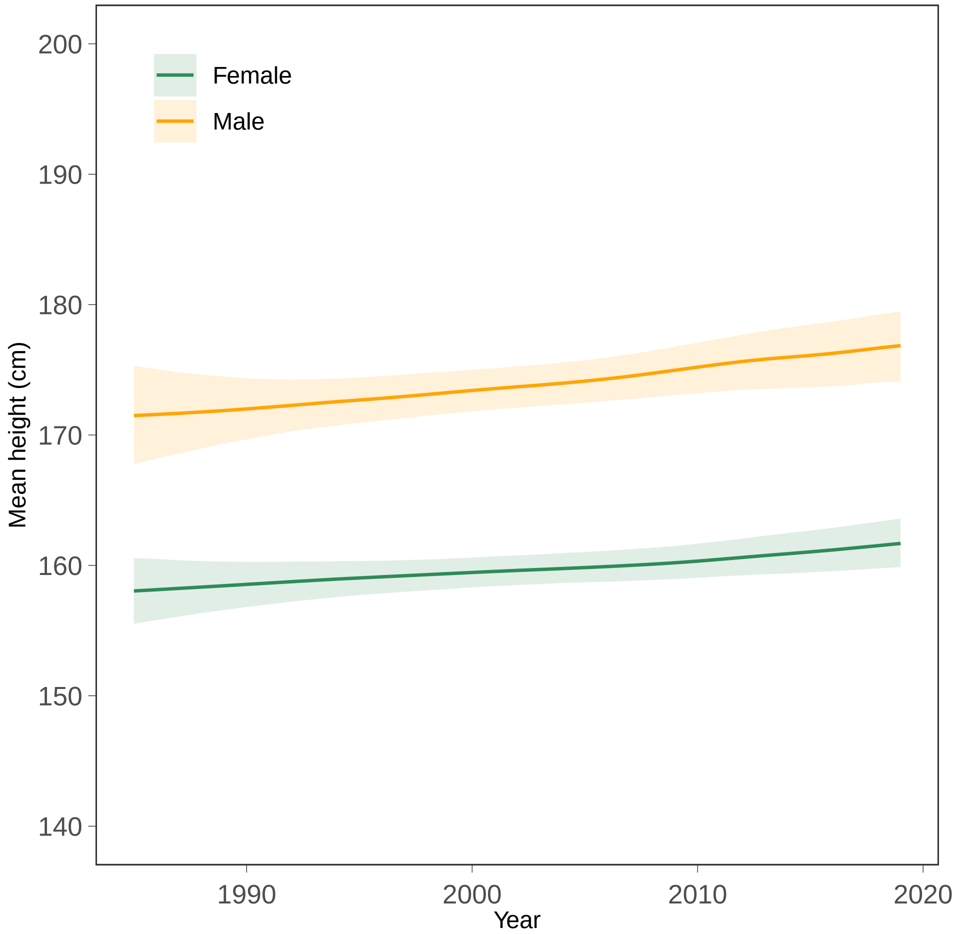

Time trends in BMI of 19 year olds

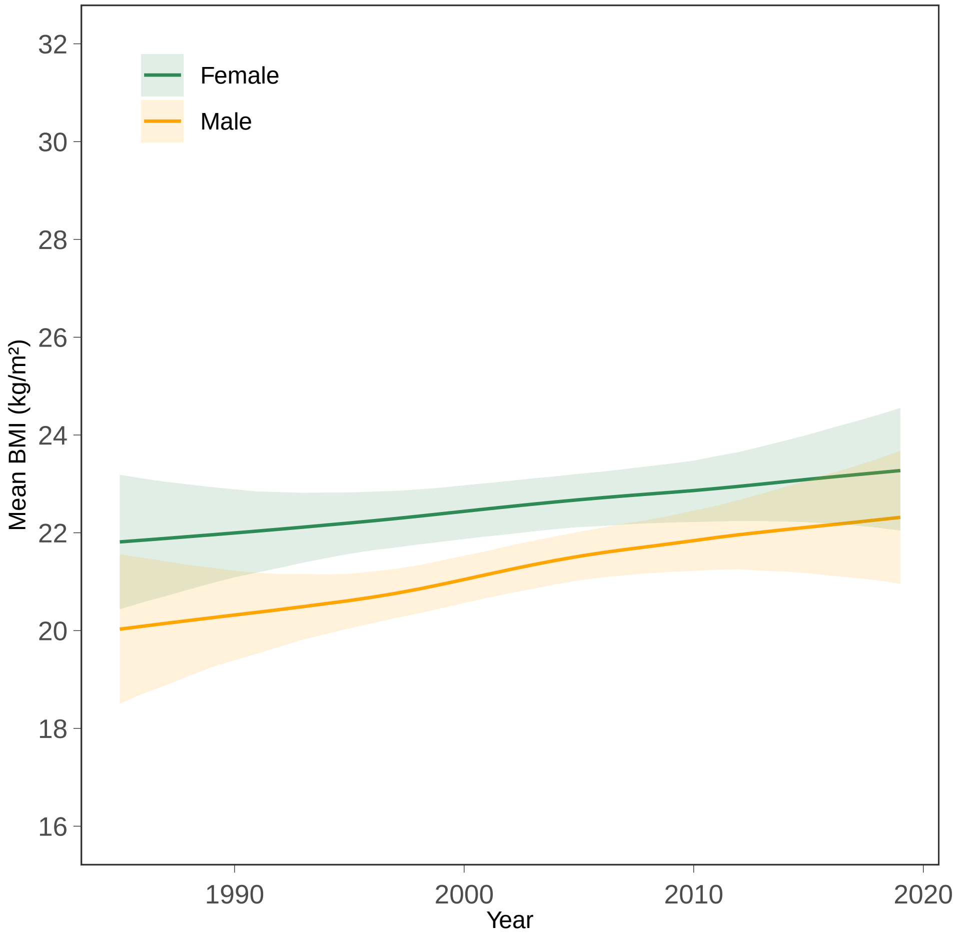

Height−for−age trajectories (2000 birth cohort)

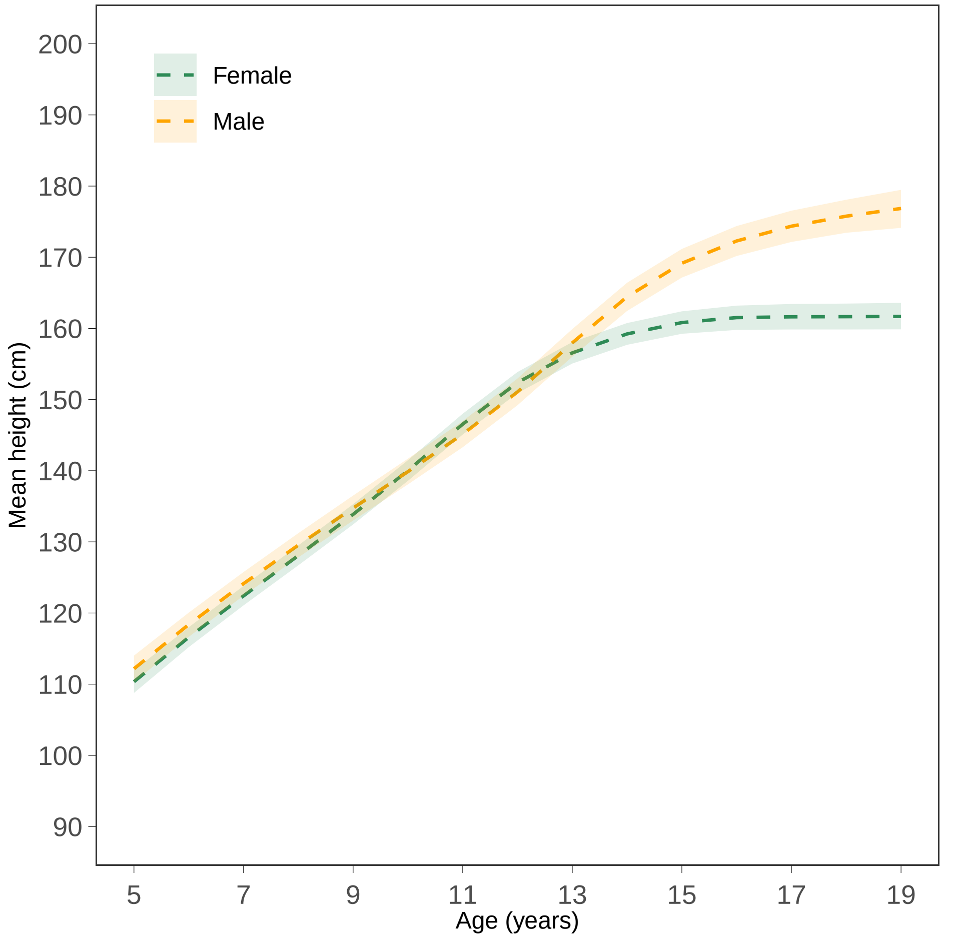

BMI−for−age trajectories (2000 birth cohort)

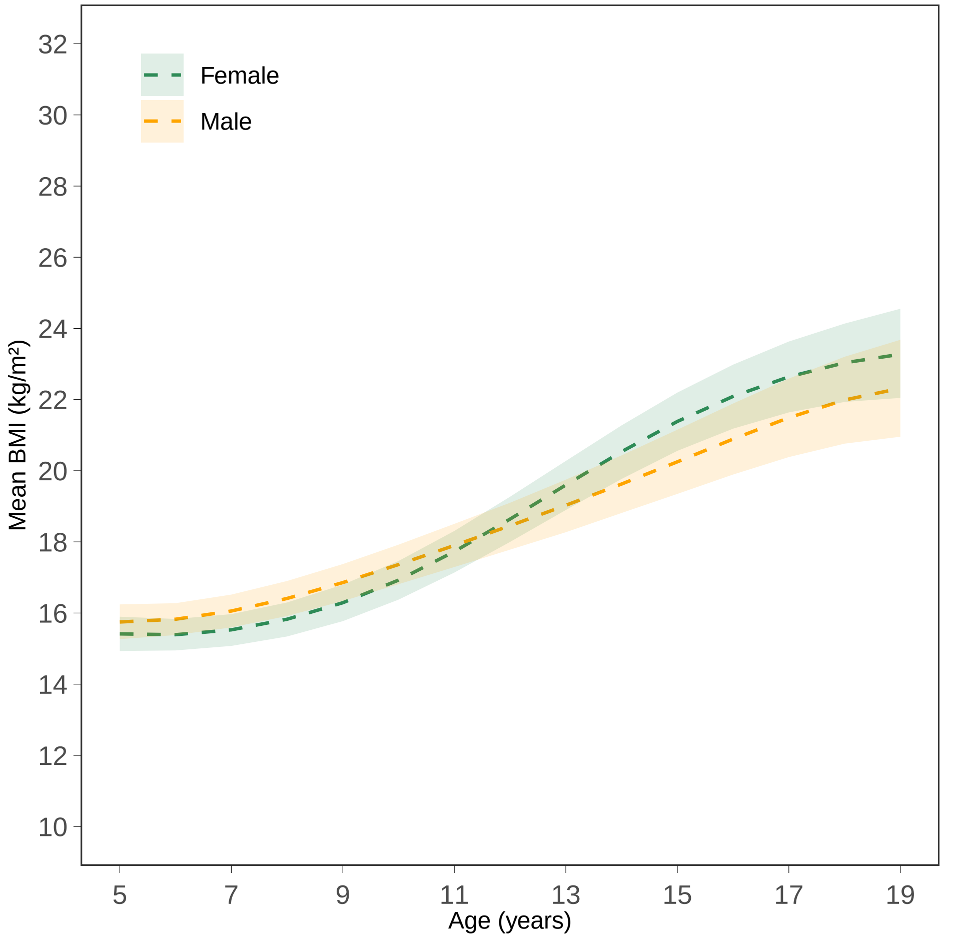

Turkey

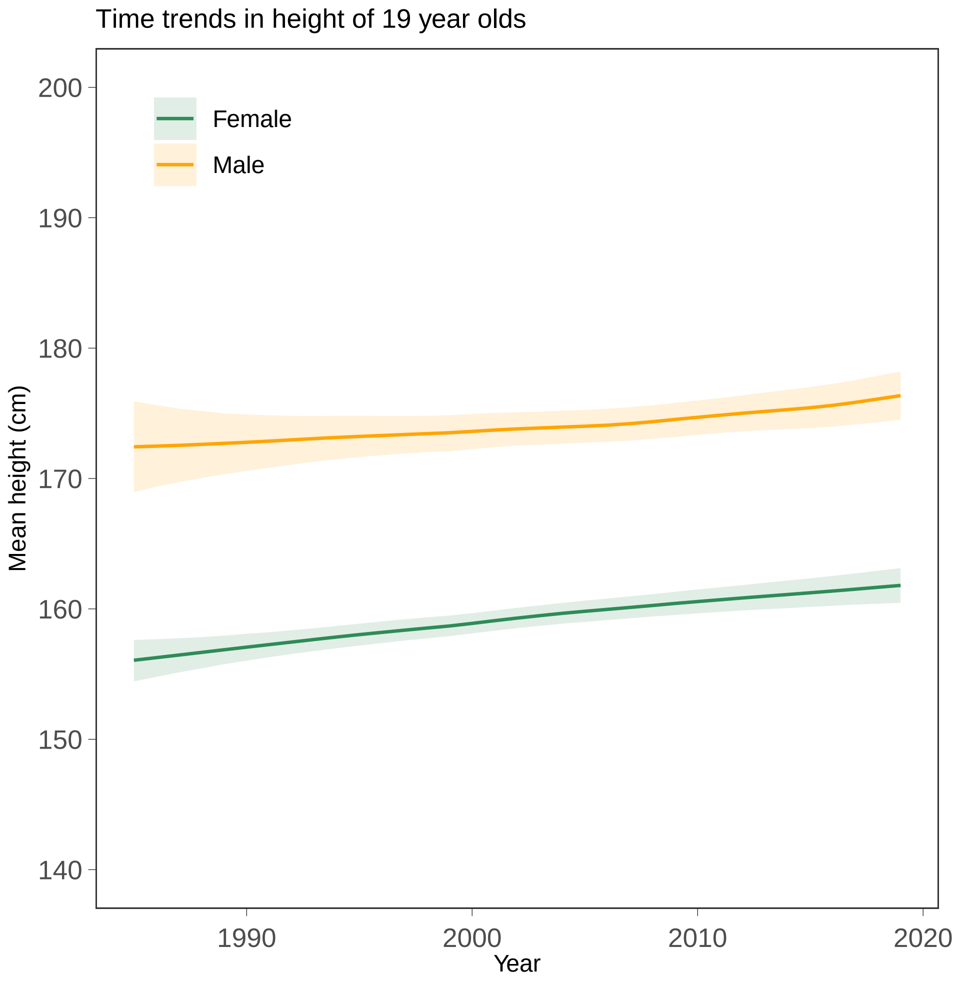
Time trends in height of 19 year olds
Female
Male
200
190
180
170
160
150
140
Mean height (cm)
1990
2000
2010
2020
Year

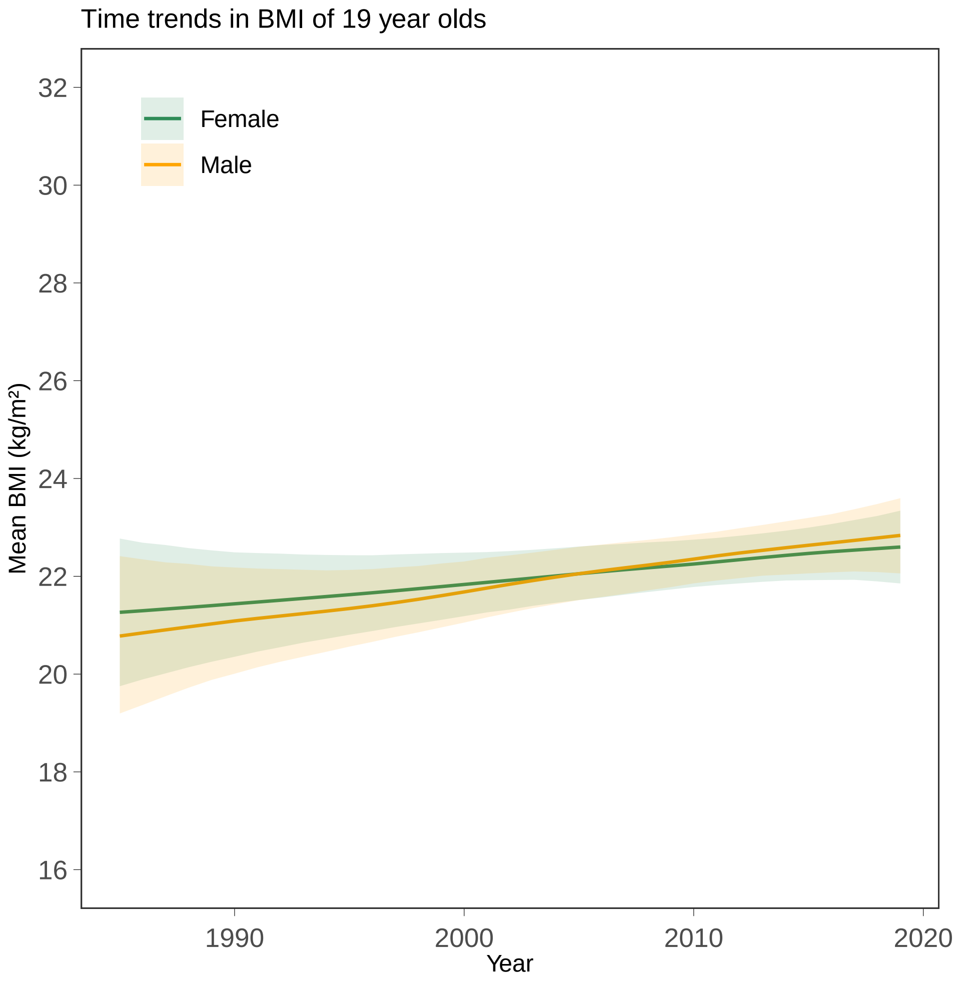
Time trends in BMI of 19 year olds
Female
Male
32
30
28
26
24
22
20
18
16
Mean BMI (kg/m²)
1990
2000
2010
2020
Year

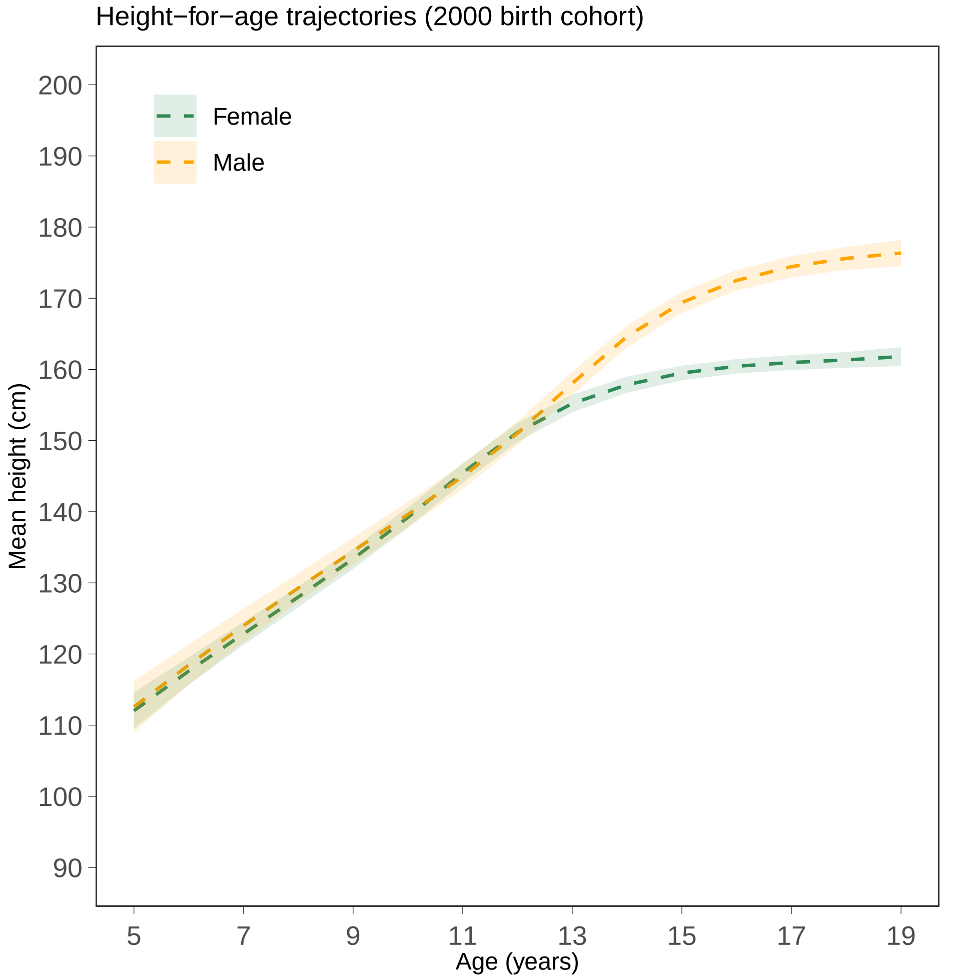
Height−for−age trajectories (2000 birth cohort)
Female
Male
200
190
180
170
160
150
140
130
120
110
100
90
Mean height (cm)
5
7
9
11
13
15
17
19
Age (years)

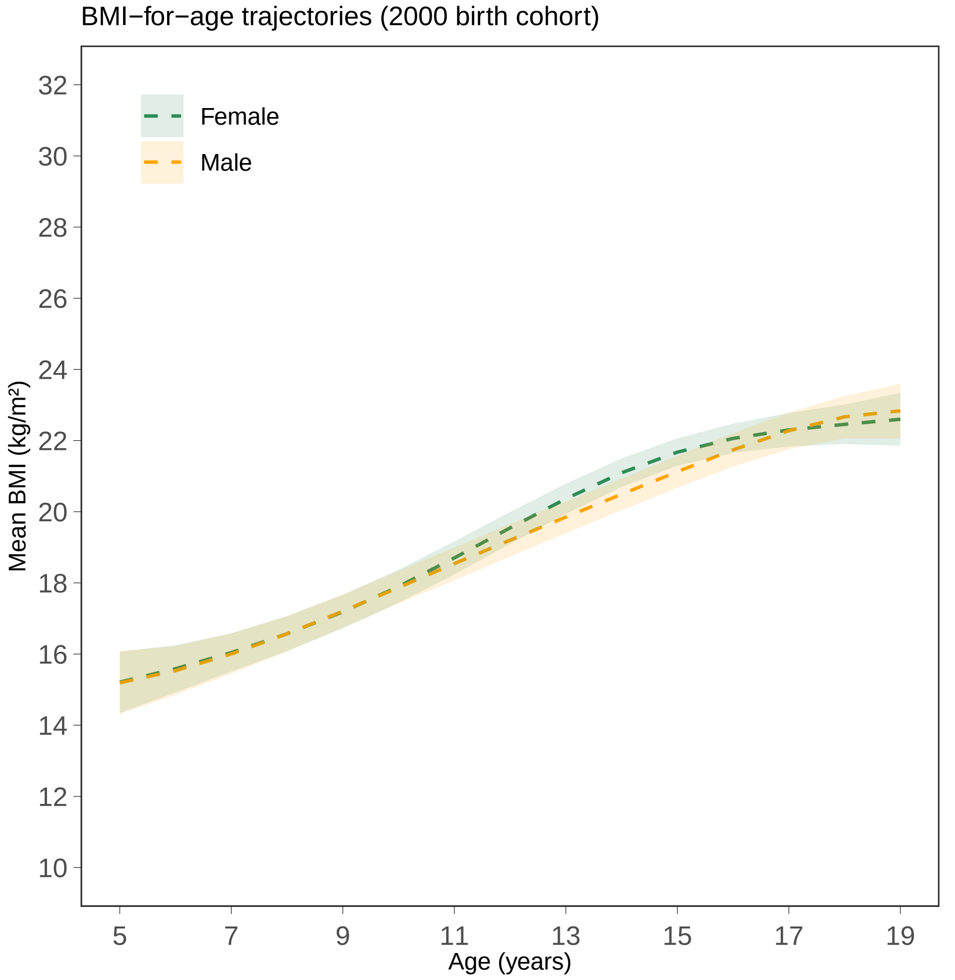
BMI−for−age trajectories (2000 birth cohort)
Female
Male
32
30
28
26
24
22
20
18
16
14
12
10
Mean BMI (kg/m²)
5
7
9
11
13
15
17
19
Age (years)

Turkmenistan

Time trends in height of 19 year olds

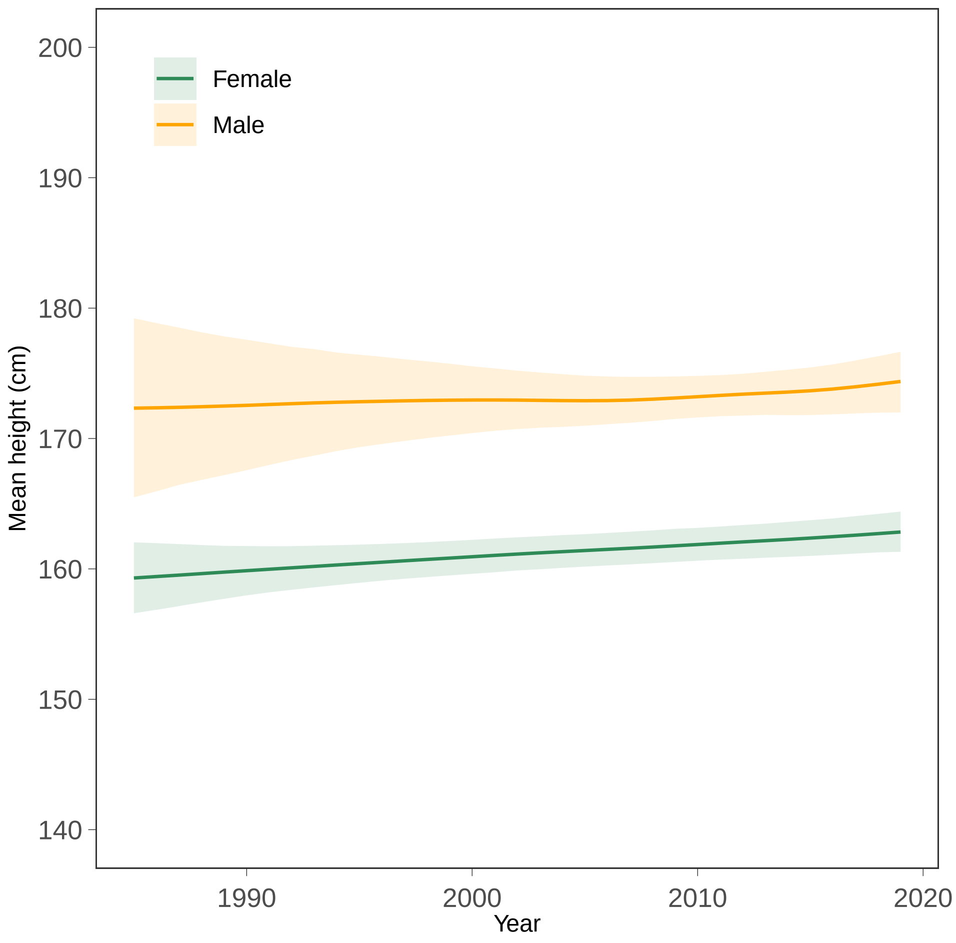

Time trends in BMI of 19 year olds

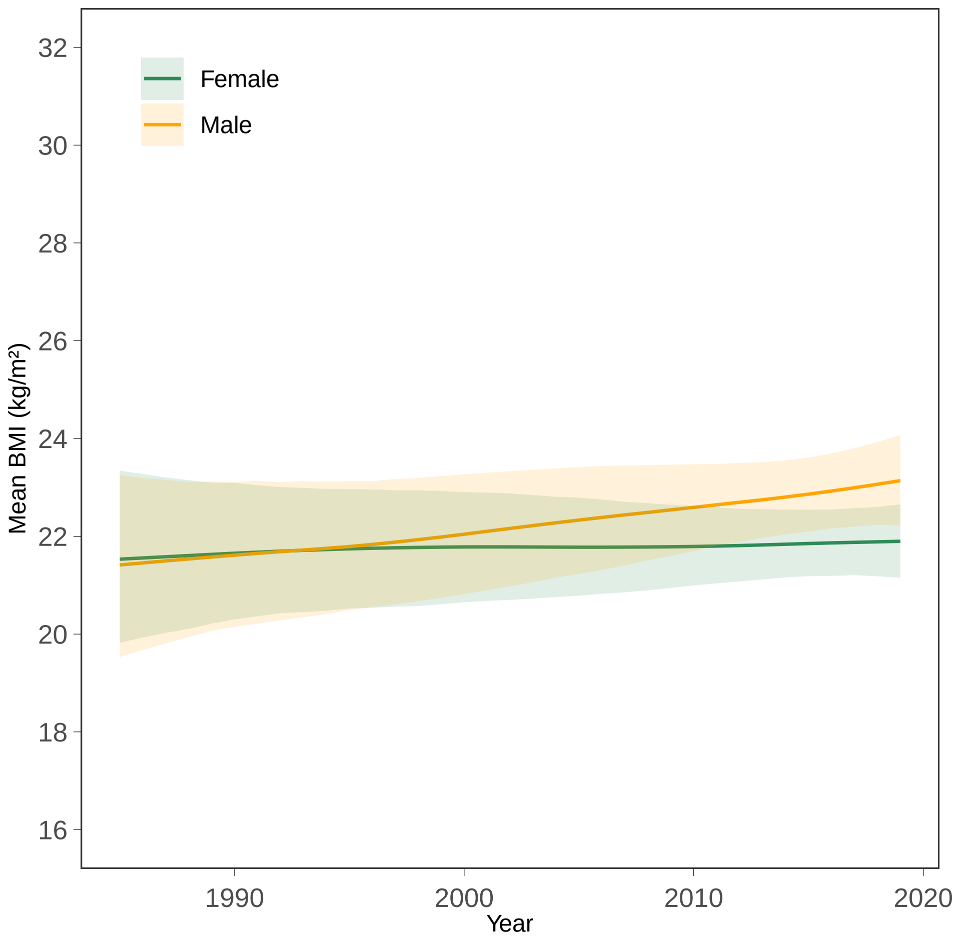

Height−for−age trajectories (2000 birth cohort)

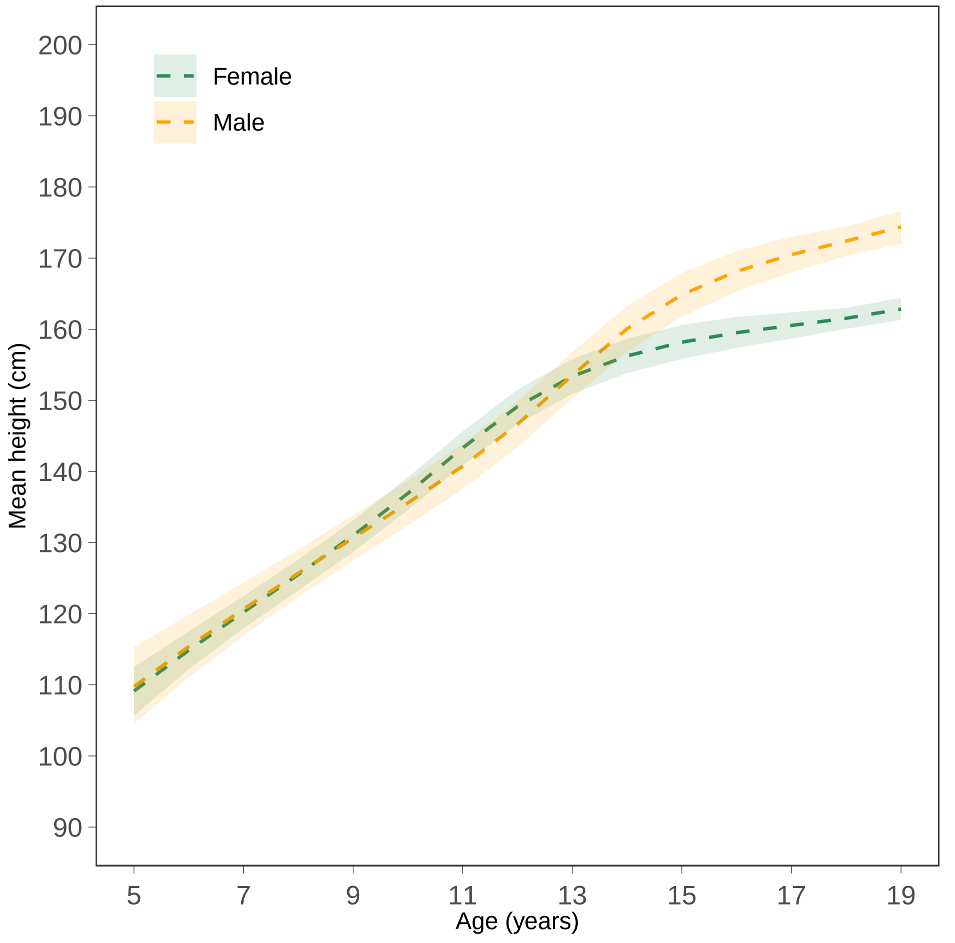

BMI−for−age trajectories (2000 birth cohort)

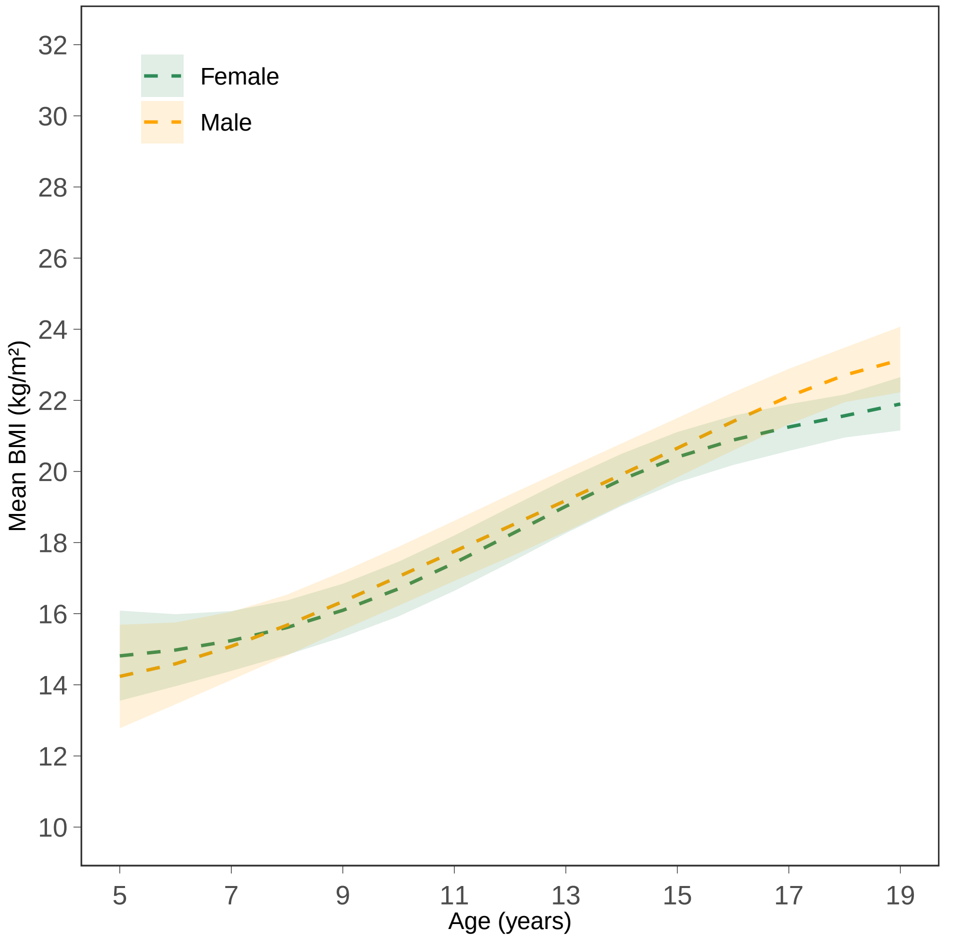

Tuvalu

Time trends in height of 19 year olds

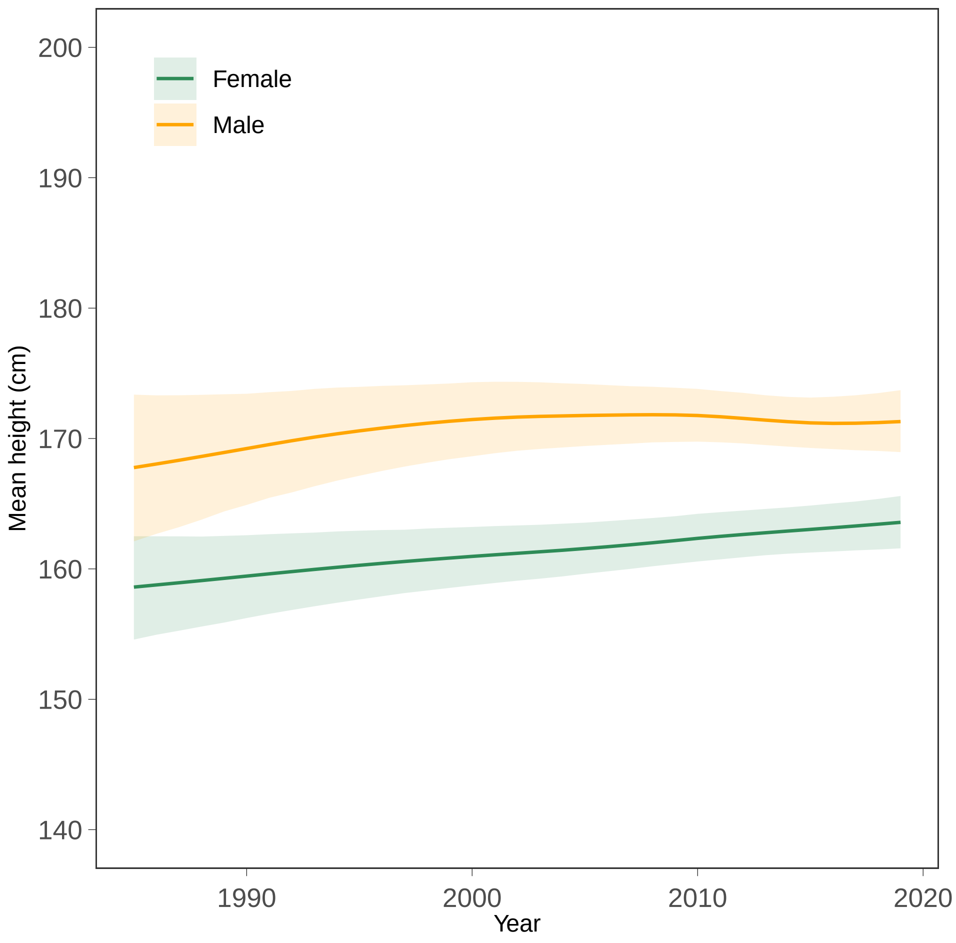

Time trends in BMI of 19 year olds

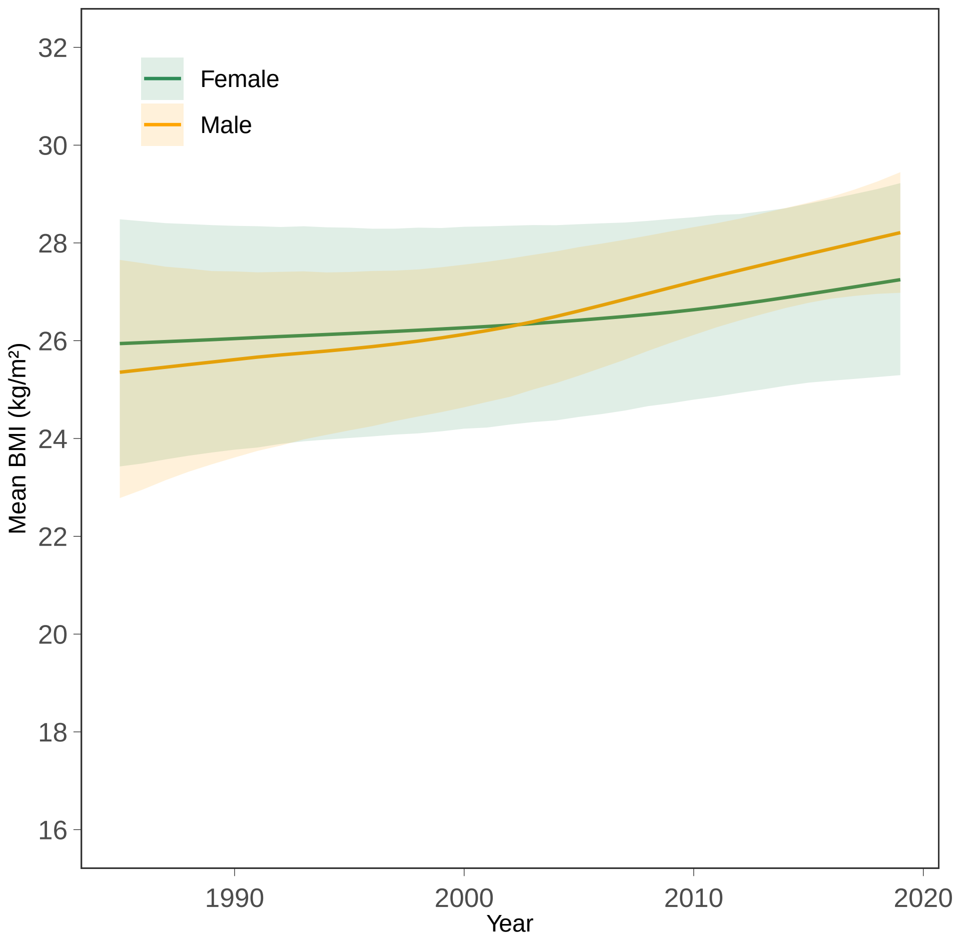

Height−for−age trajectories (2000 birth cohort)

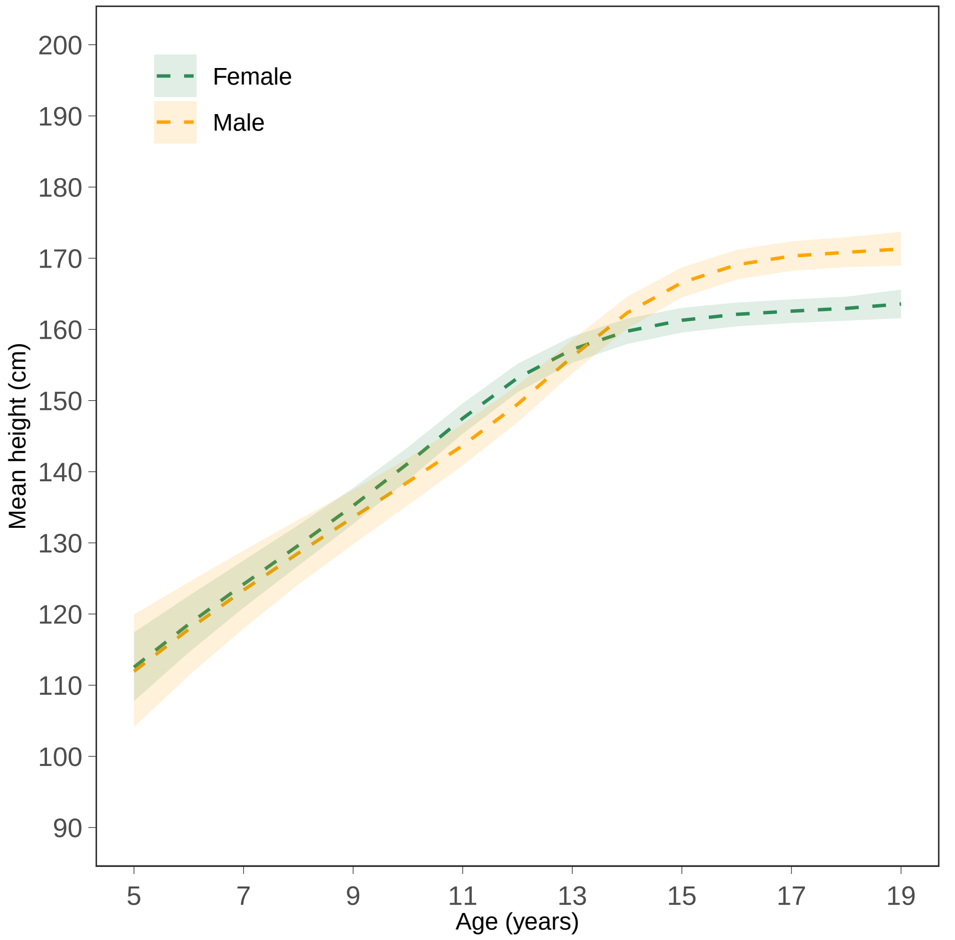

BMI−for−age trajectories (2000 birth cohort)

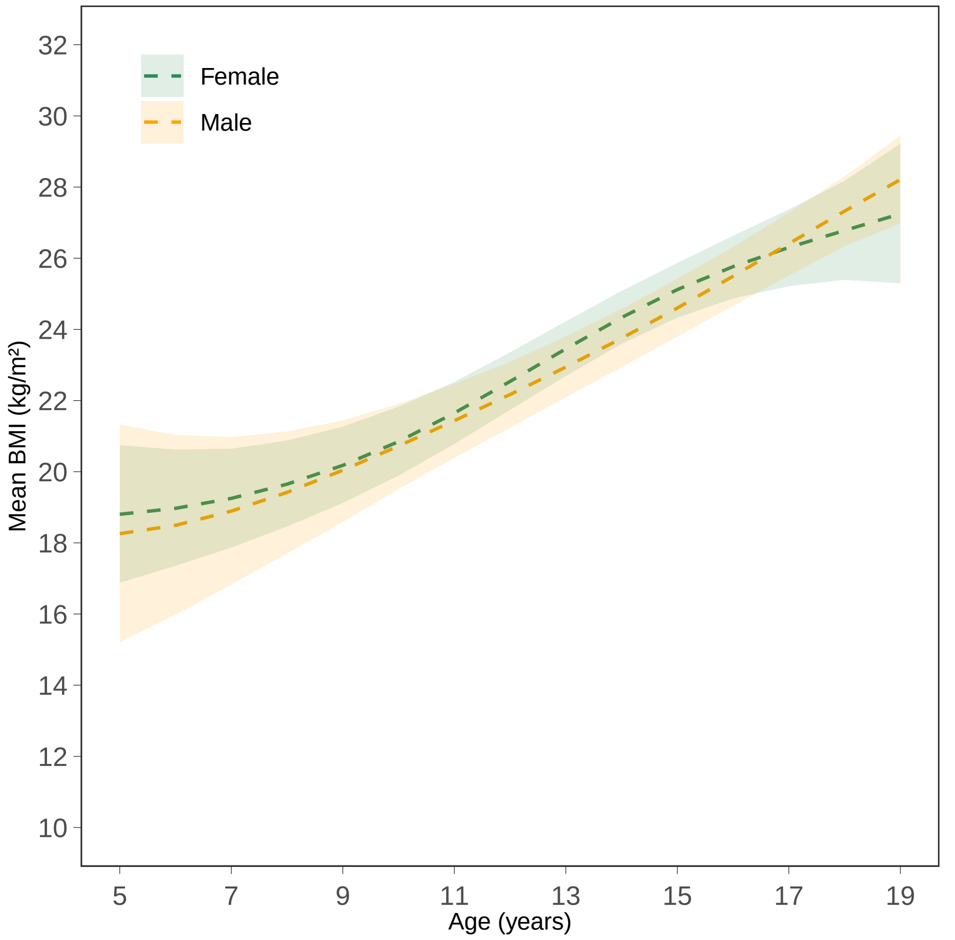

Uganda

Time trends in height of 19 year olds

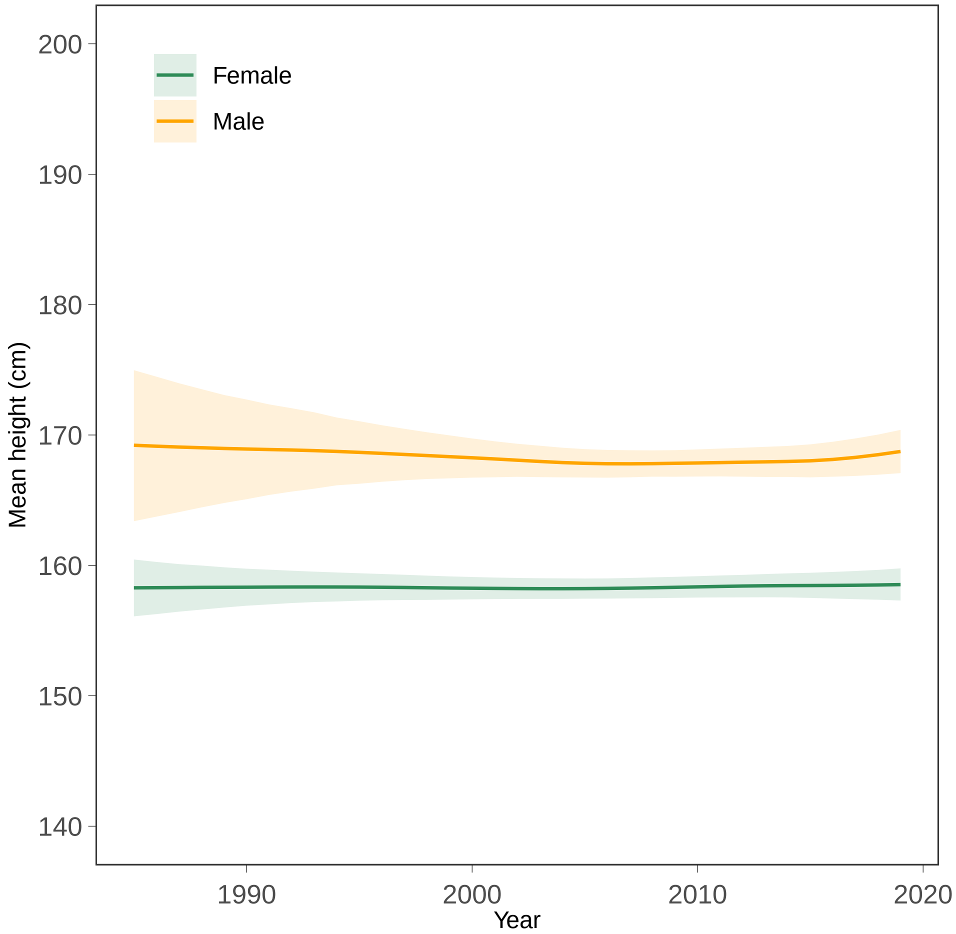

Time trends in BMI of 19 year olds

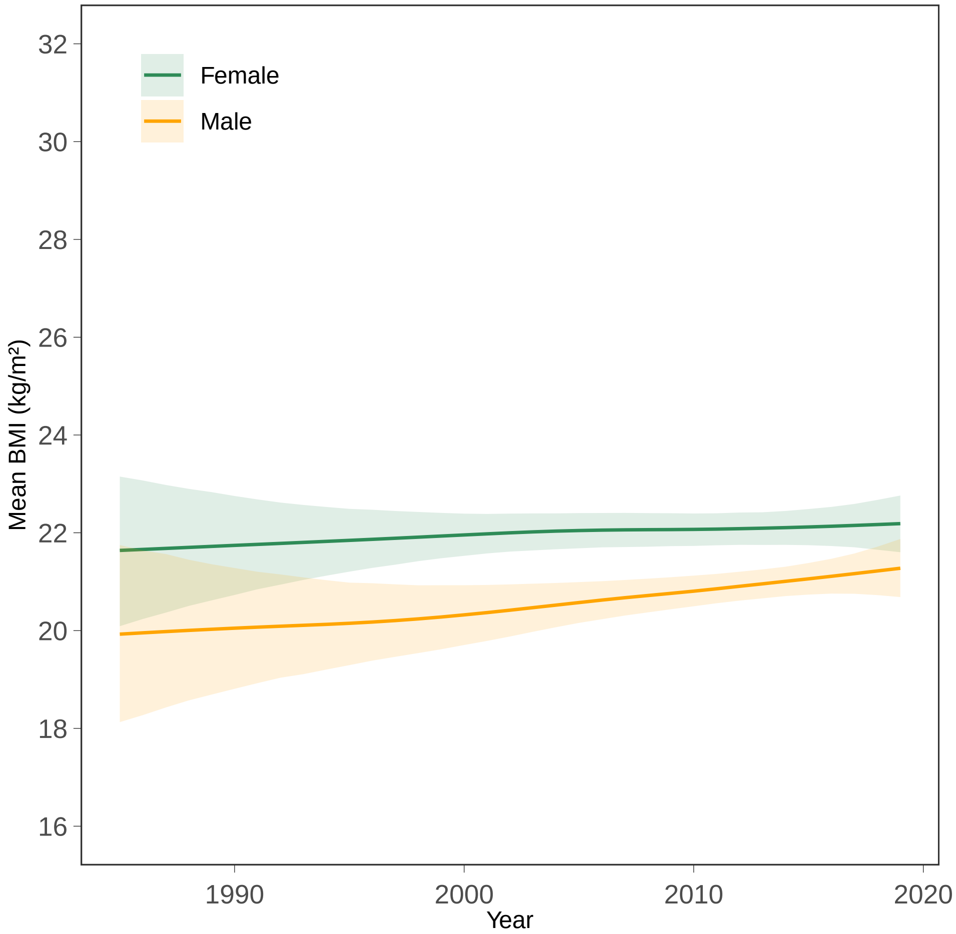

Height−for−age trajectories (2000 birth cohort)

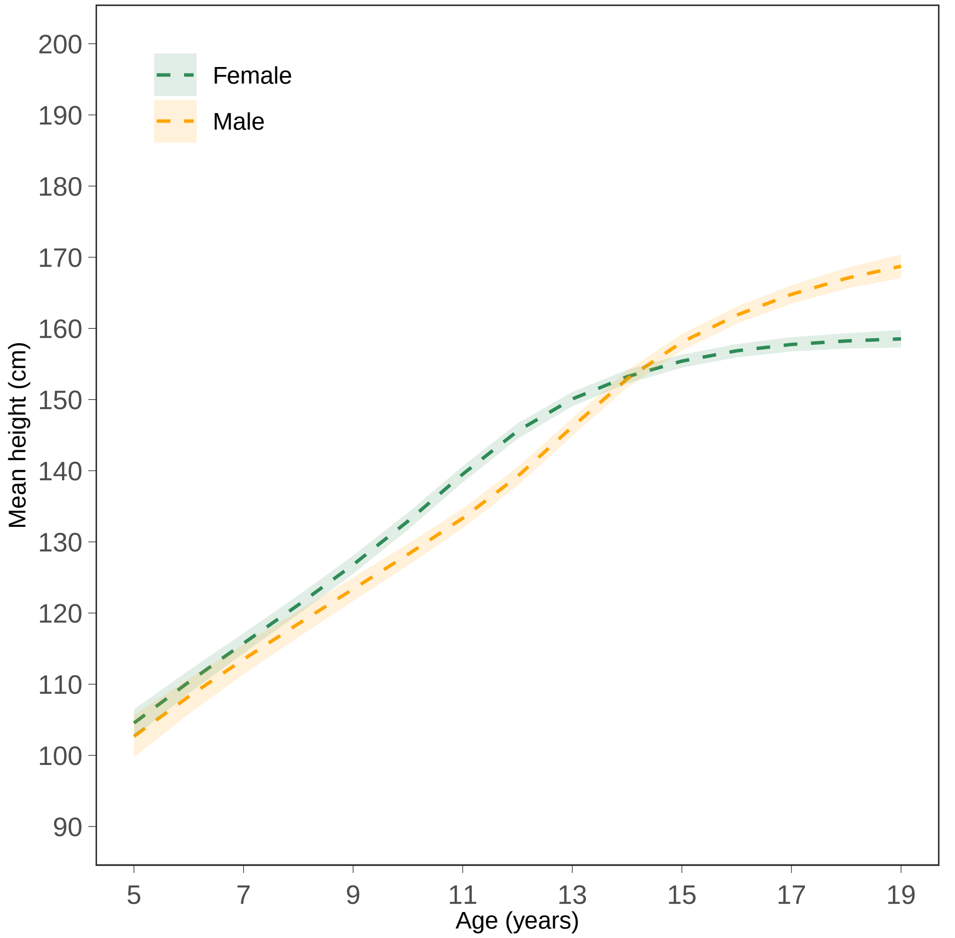

BMI−for−age trajectories (2000 birth cohort)

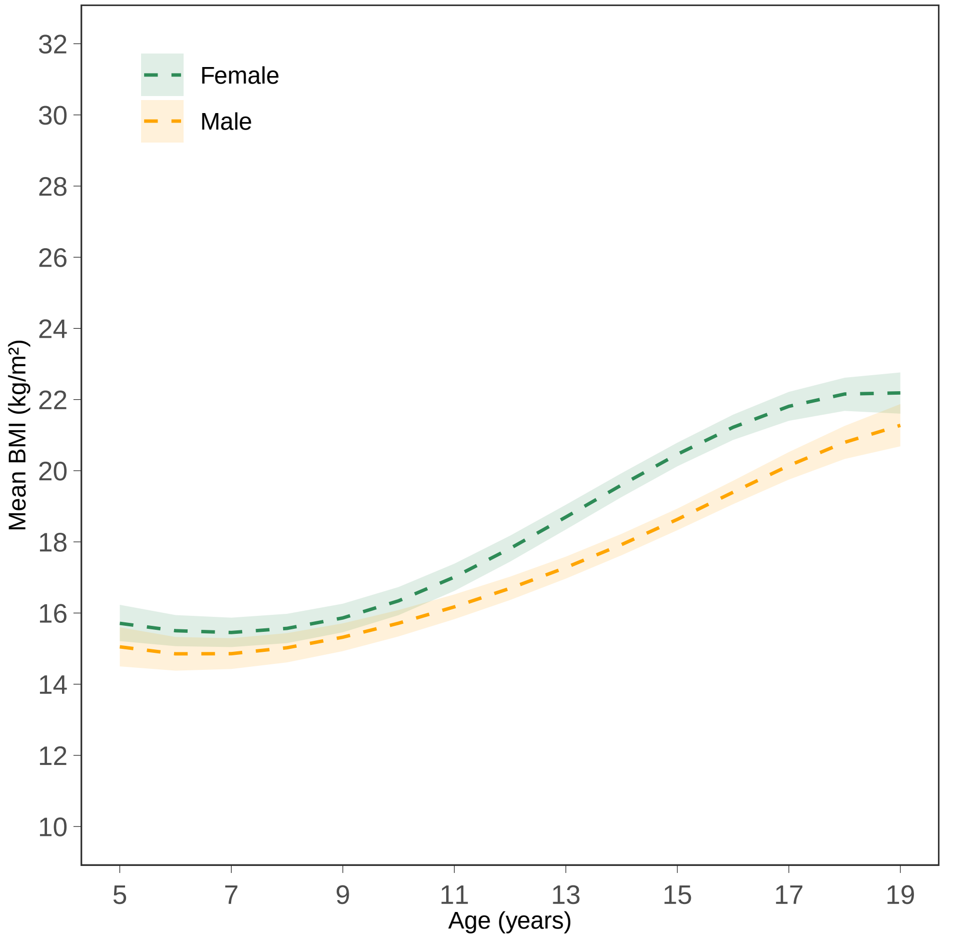

Ukraine

Time trends in height of 19 year olds

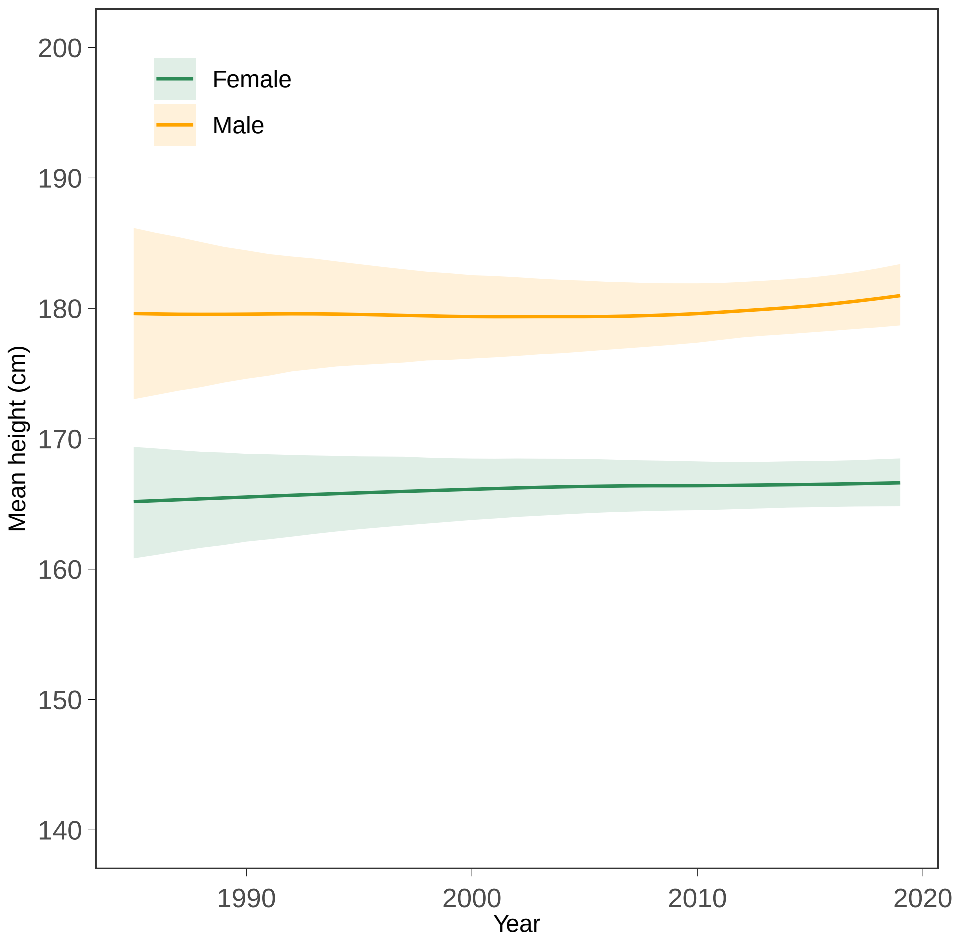

Time trends in BMI of 19 year olds

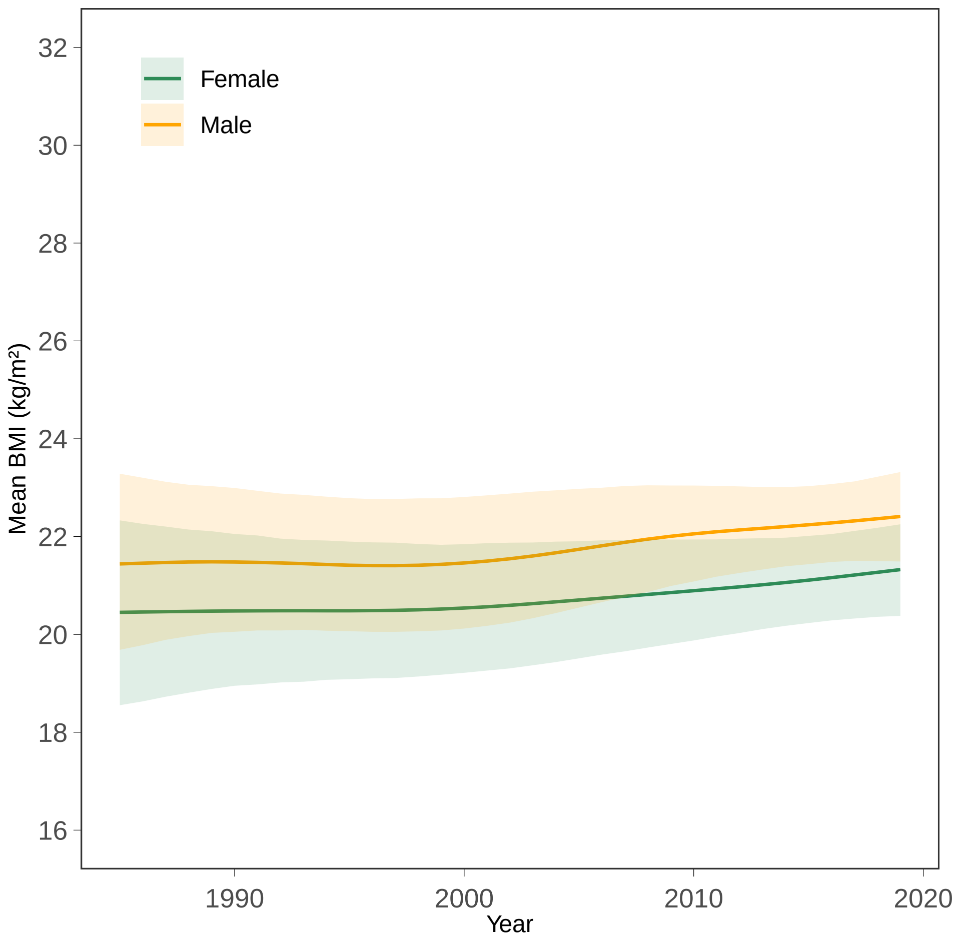

Height−for−age trajectories (2000 birth cohort)

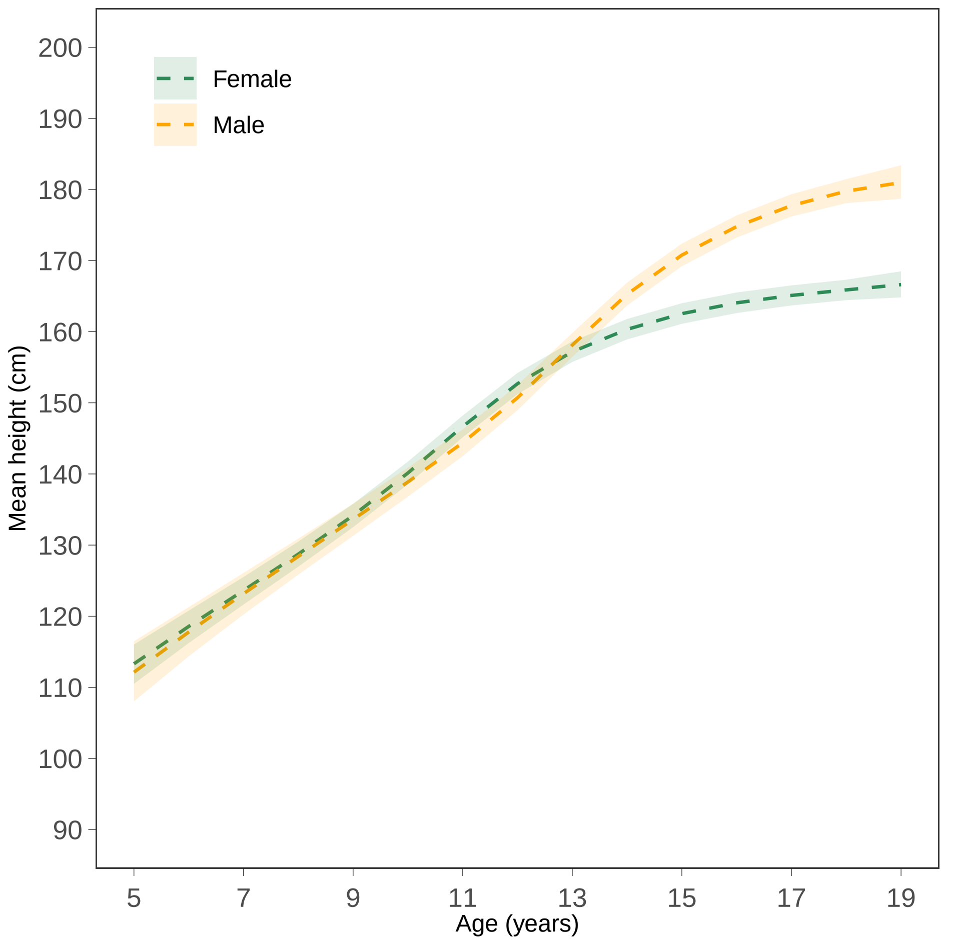

BMI−for−age trajectories (2000 birth cohort)

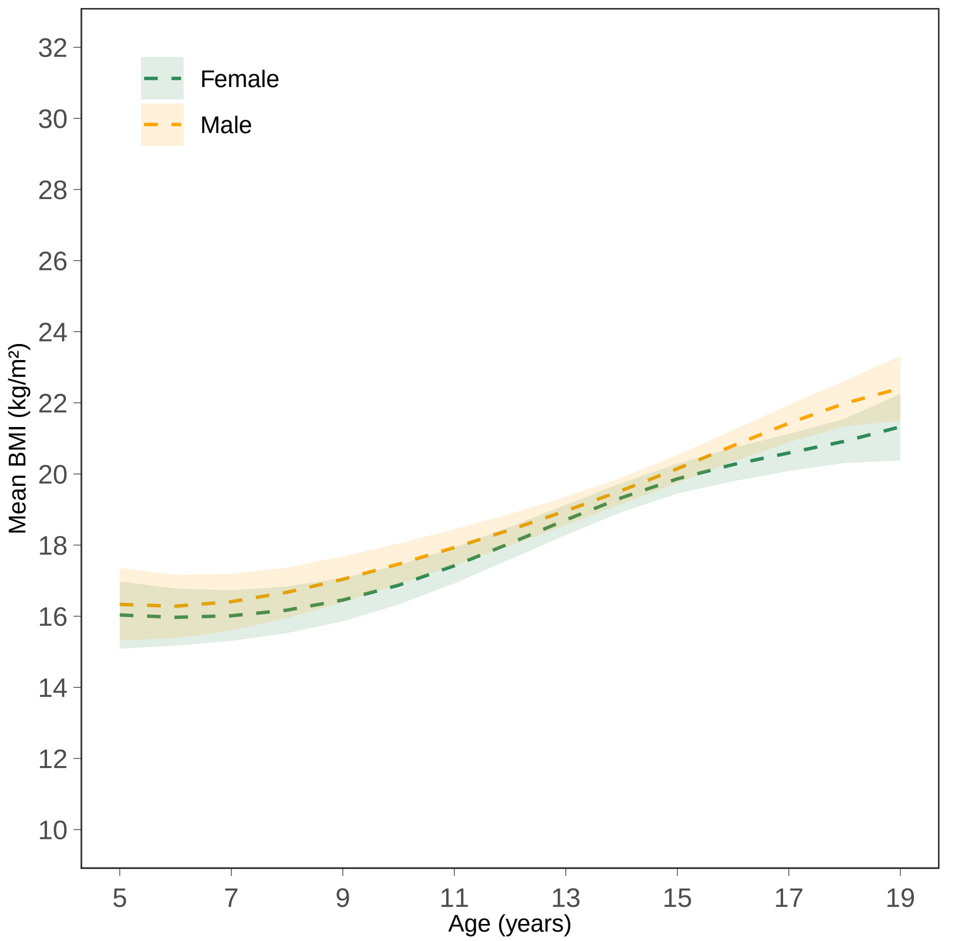

United Arab Emirates

Time trends in height of 19 year olds

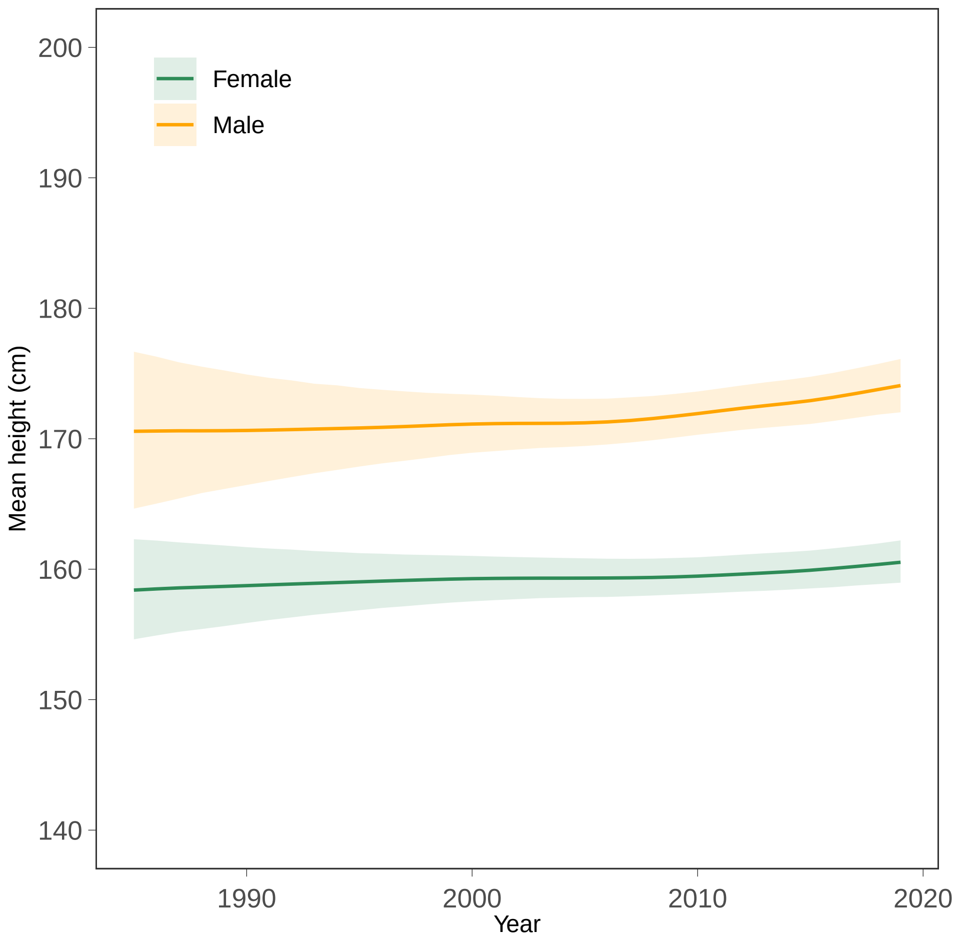

Time trends in BMI of 19 year olds

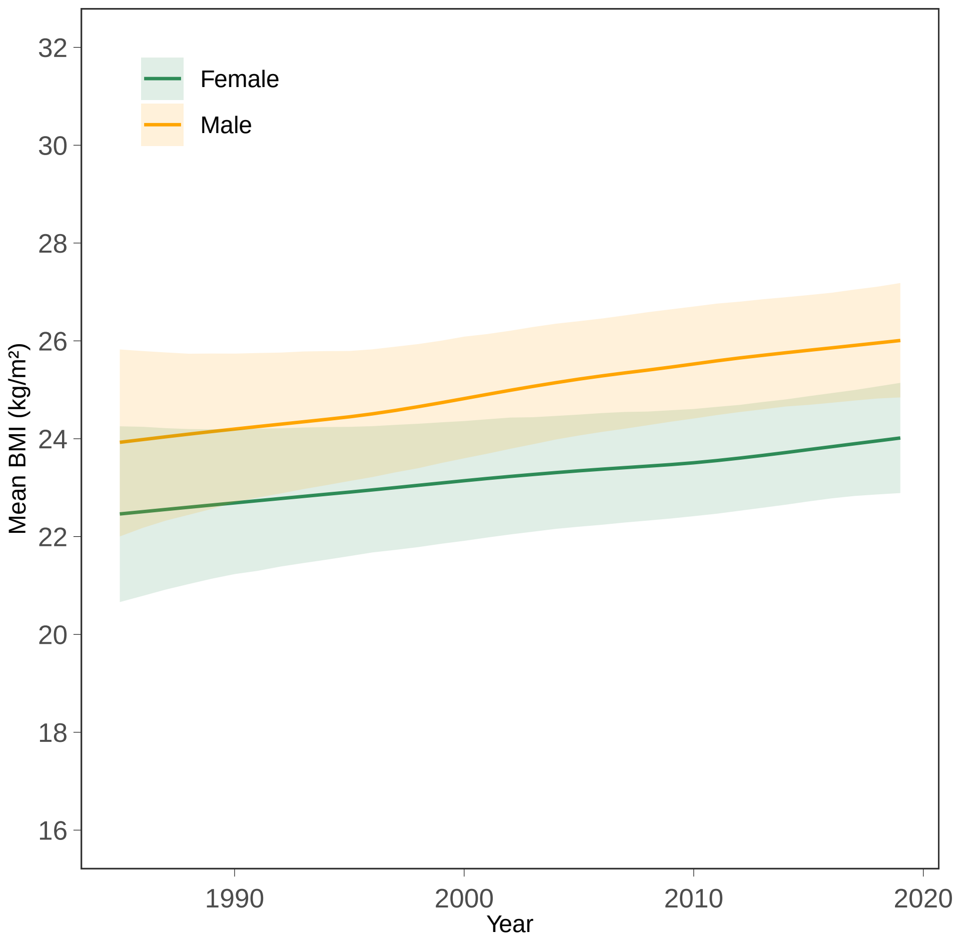

Height−for−age trajectories (2000 birth cohort)

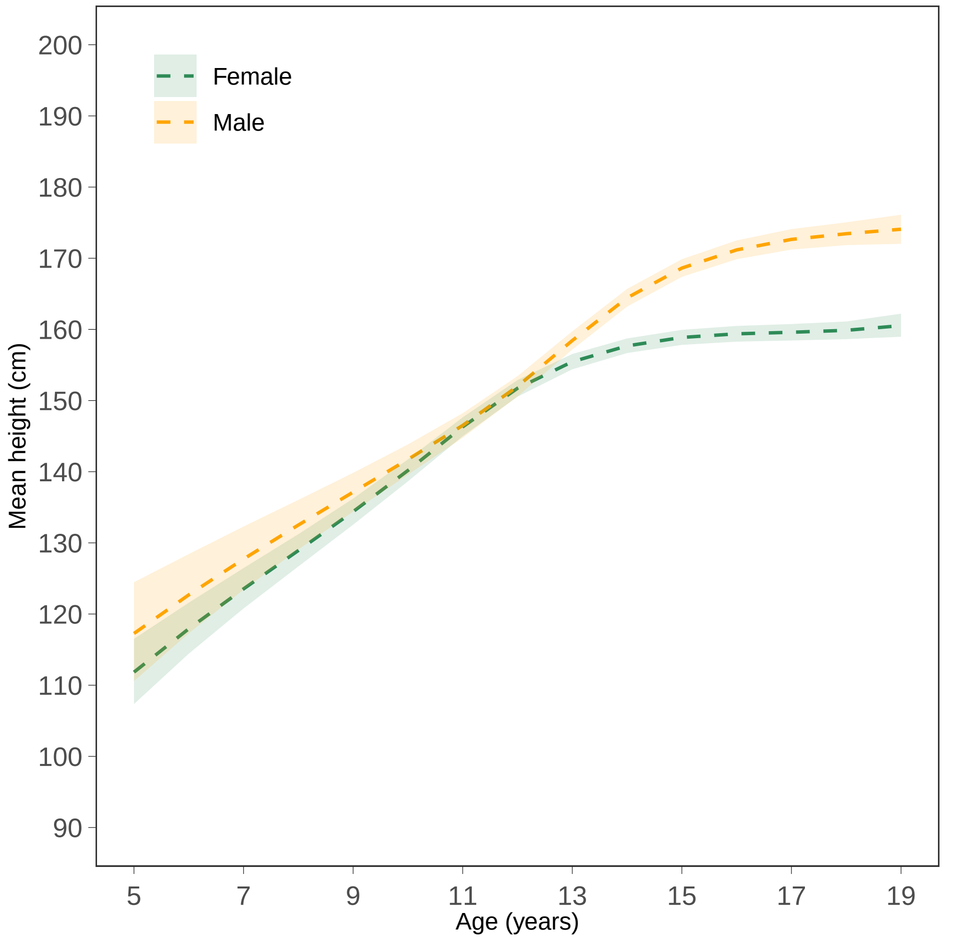

BMI−for−age trajectories (2000 birth cohort)

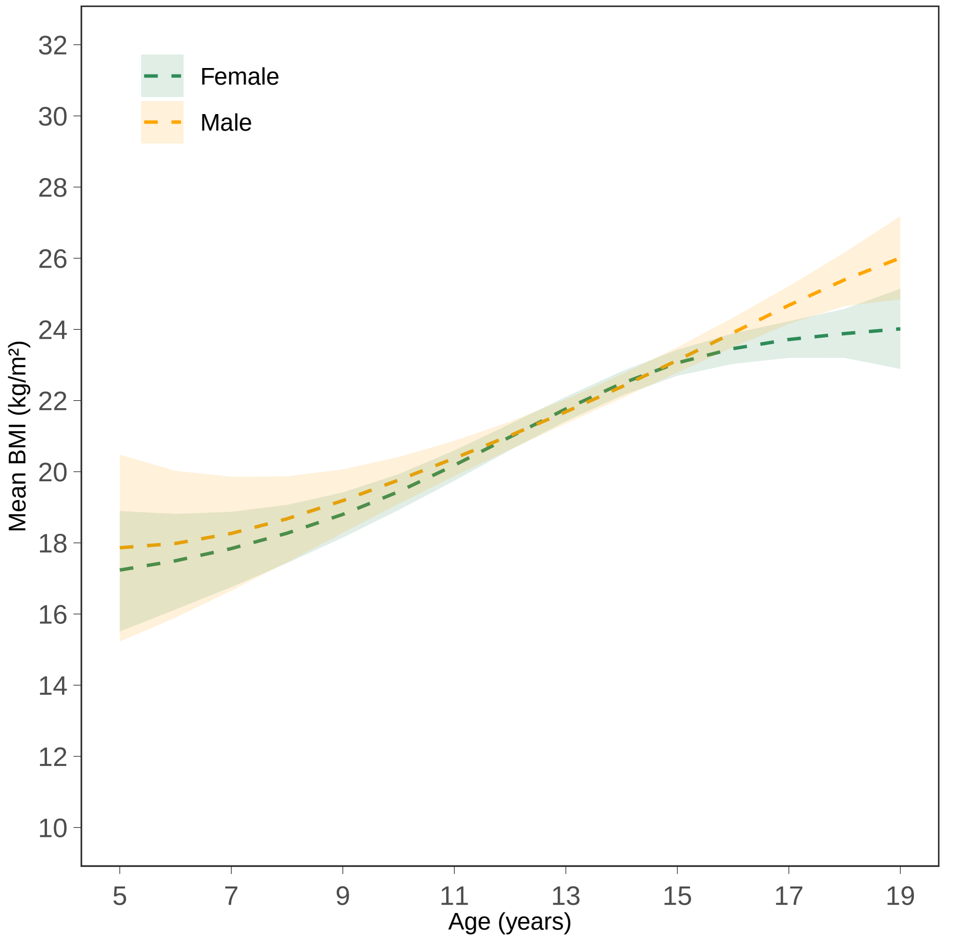

United Kingdom

Time trends in height of 19 year olds

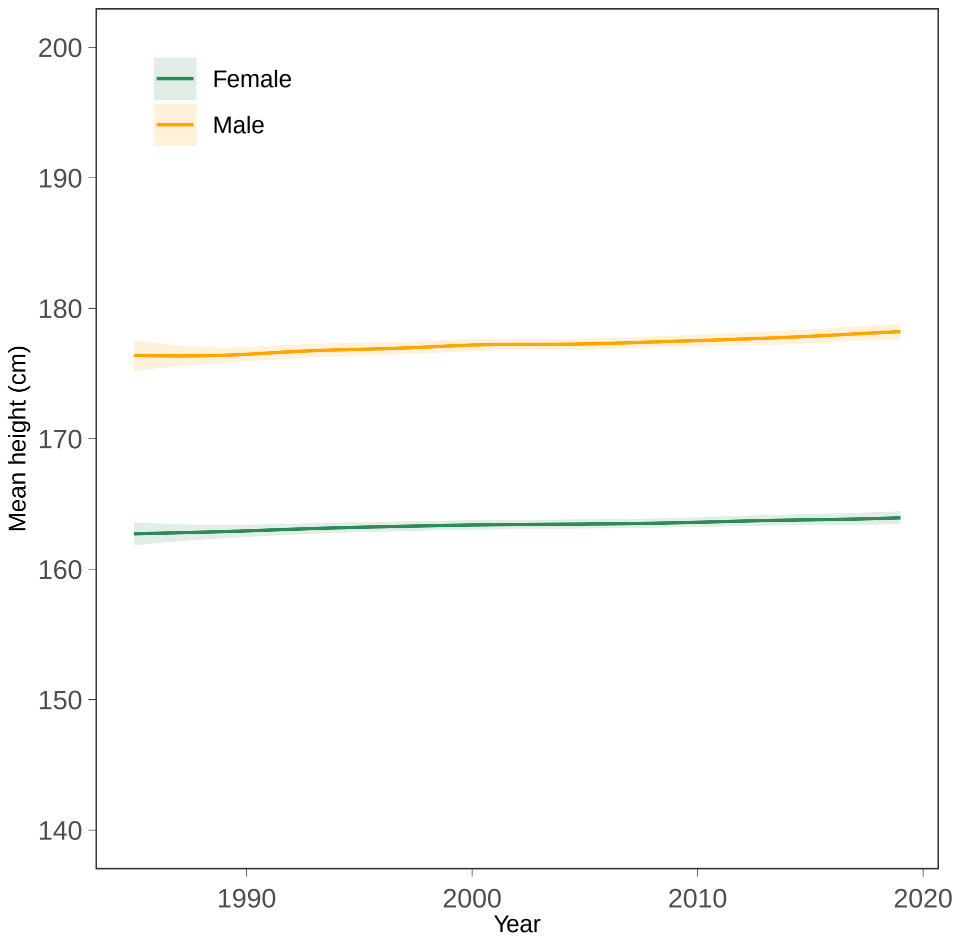

Time trends in BMI of 19 year olds

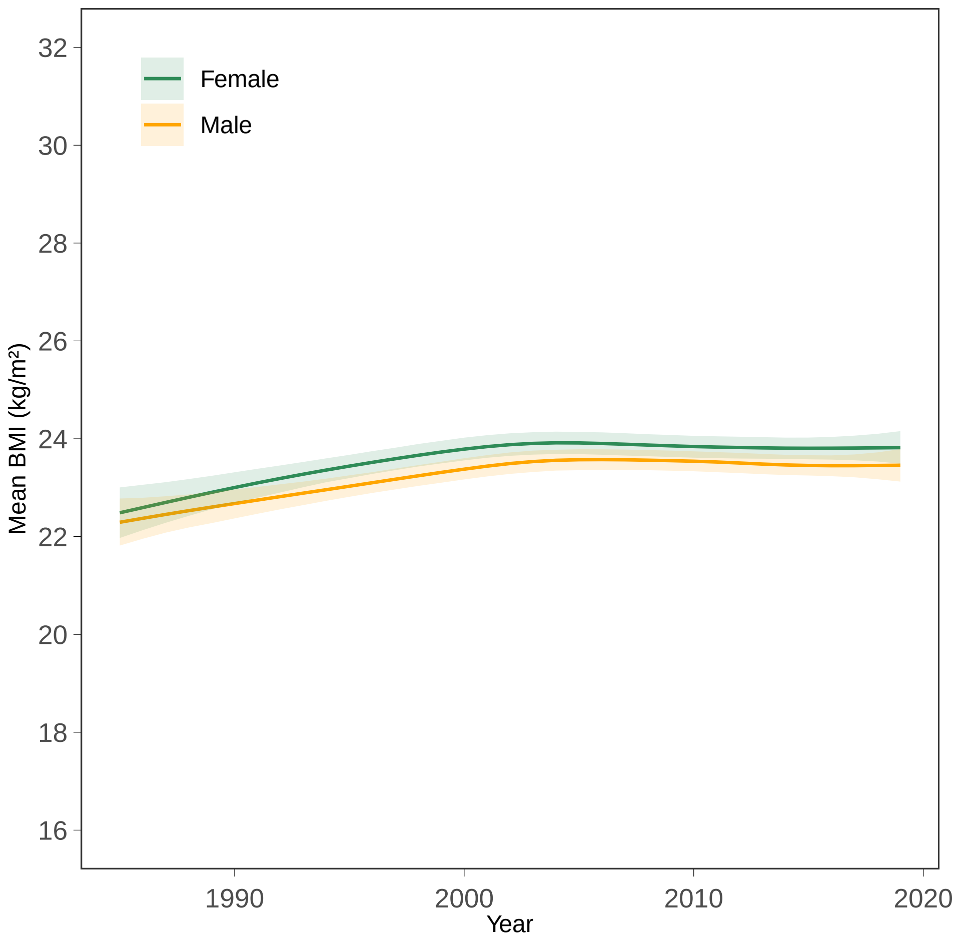

Height−for−age trajectories (2000 birth cohort)

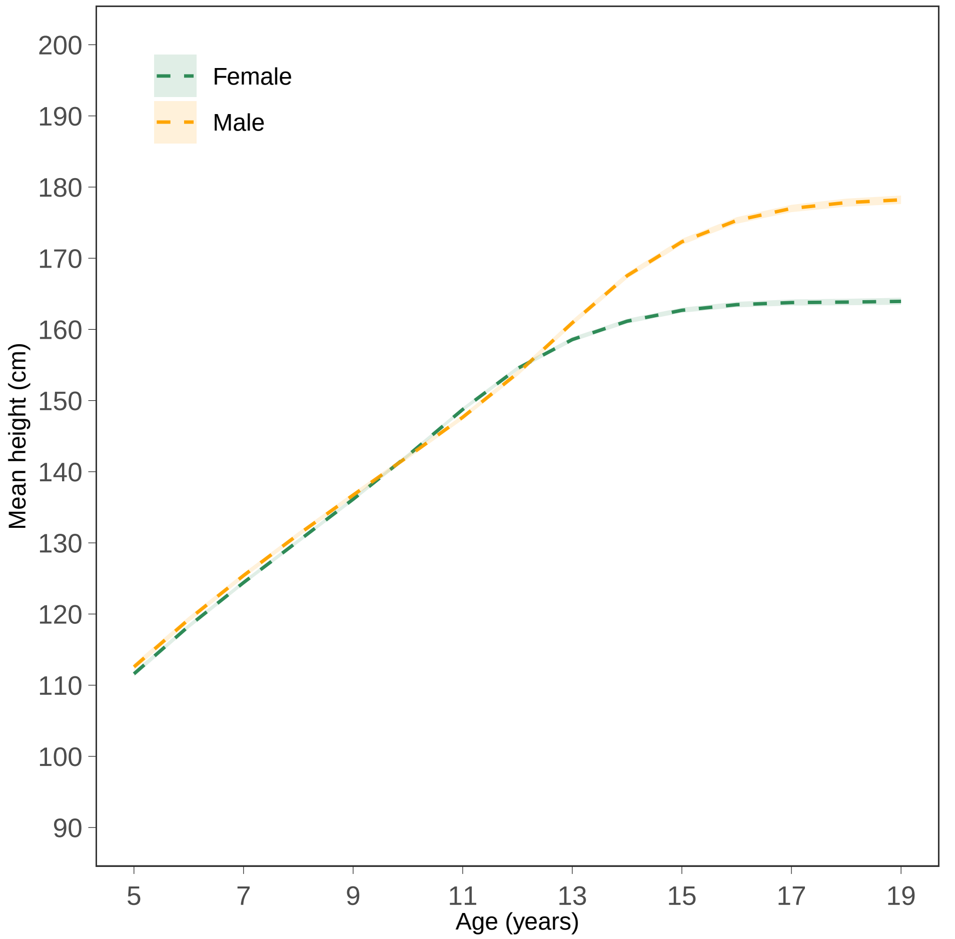

BMI−for−age trajectories (2000 birth cohort)

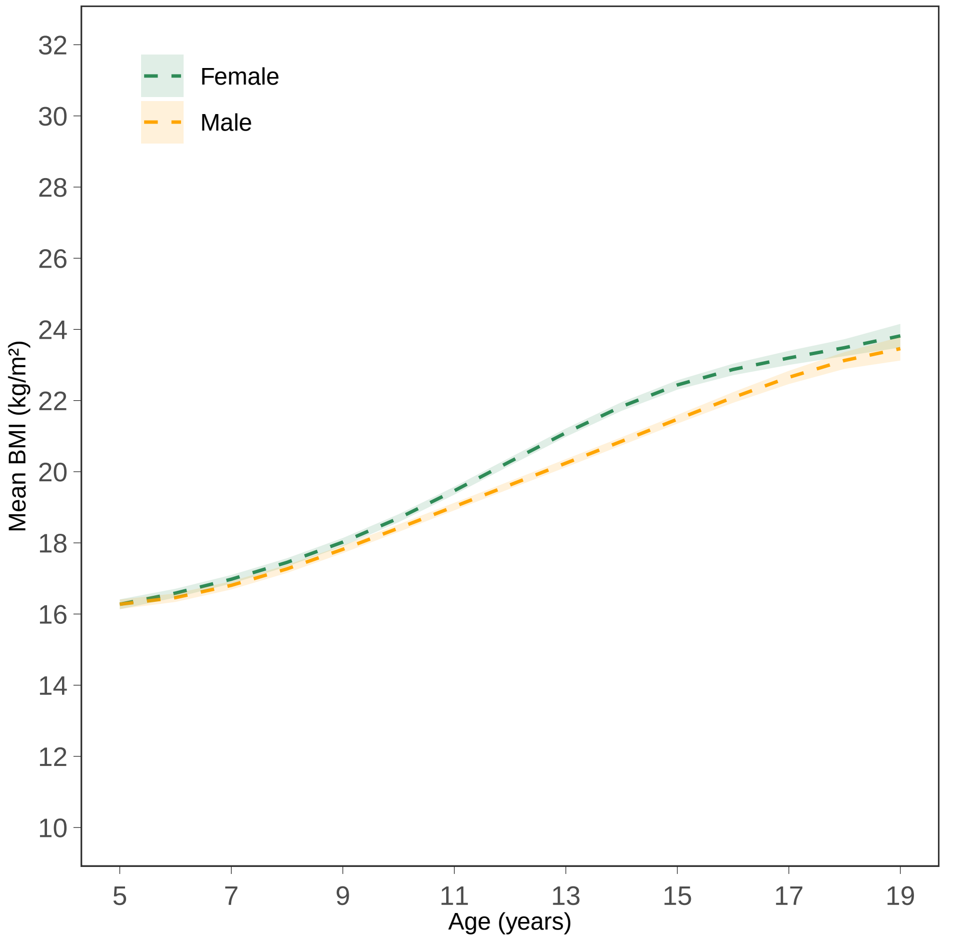

United States of America

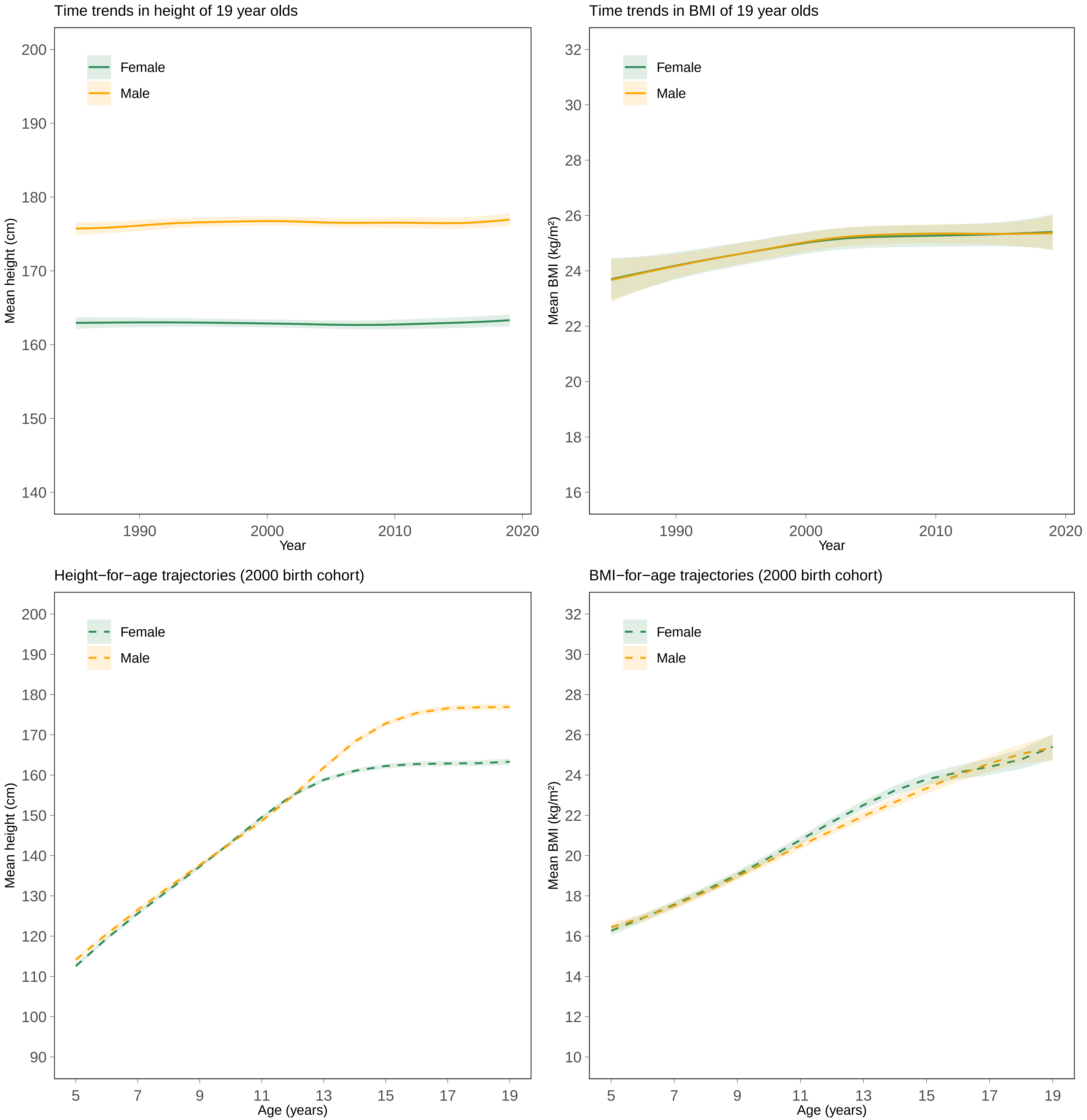

Uruguay

Time trends in height of 19 year olds

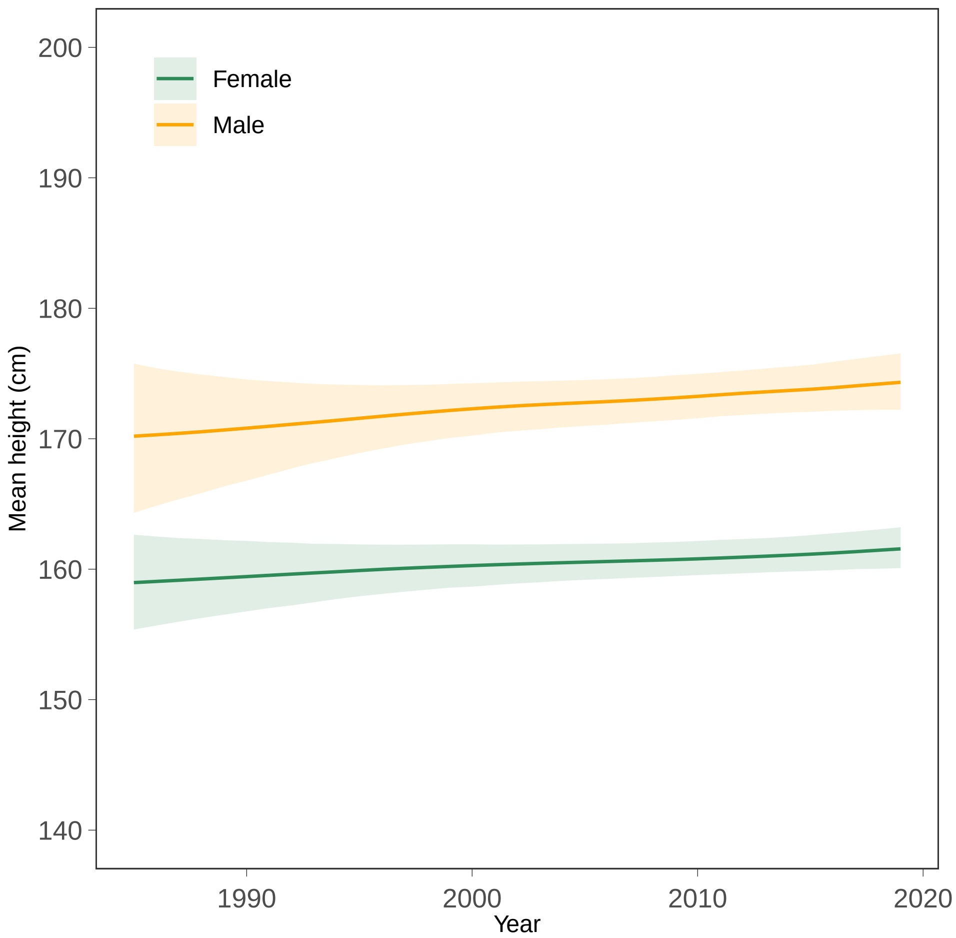

Time trends in BMI of 19 year olds

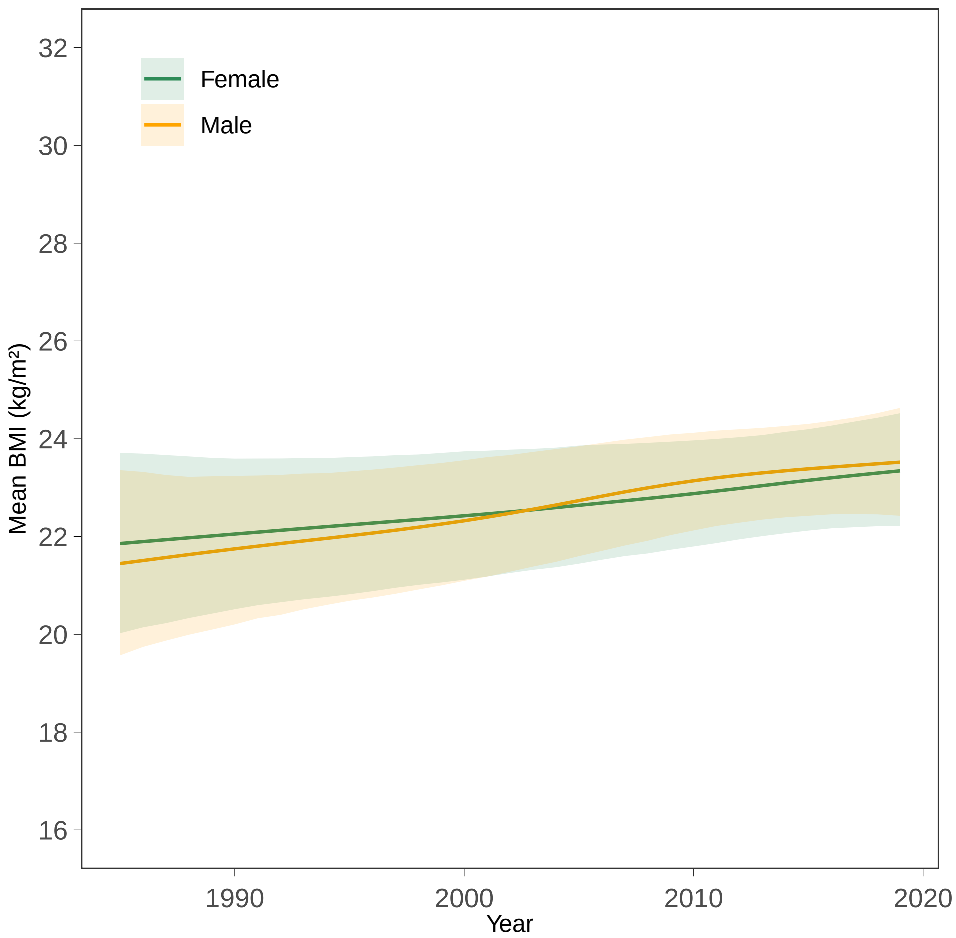

Height−for−age trajectories (2000 birth cohort)

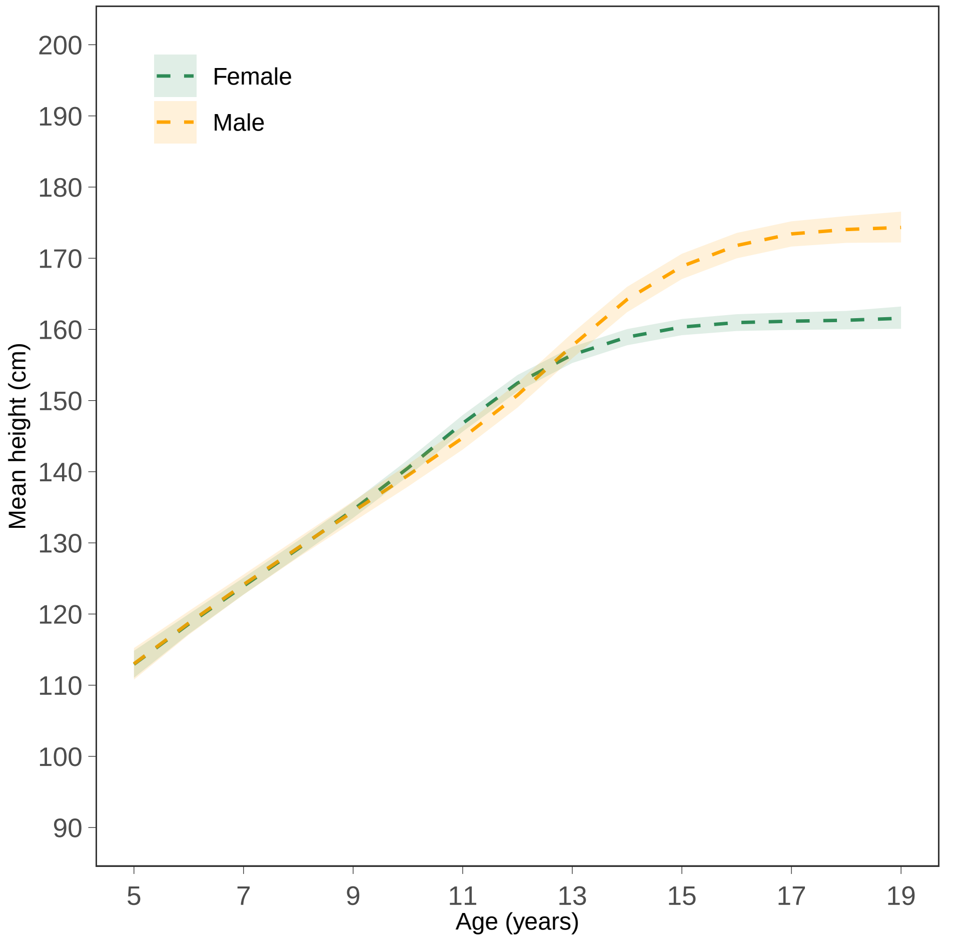

BMI−for−age trajectories (2000 birth cohort)

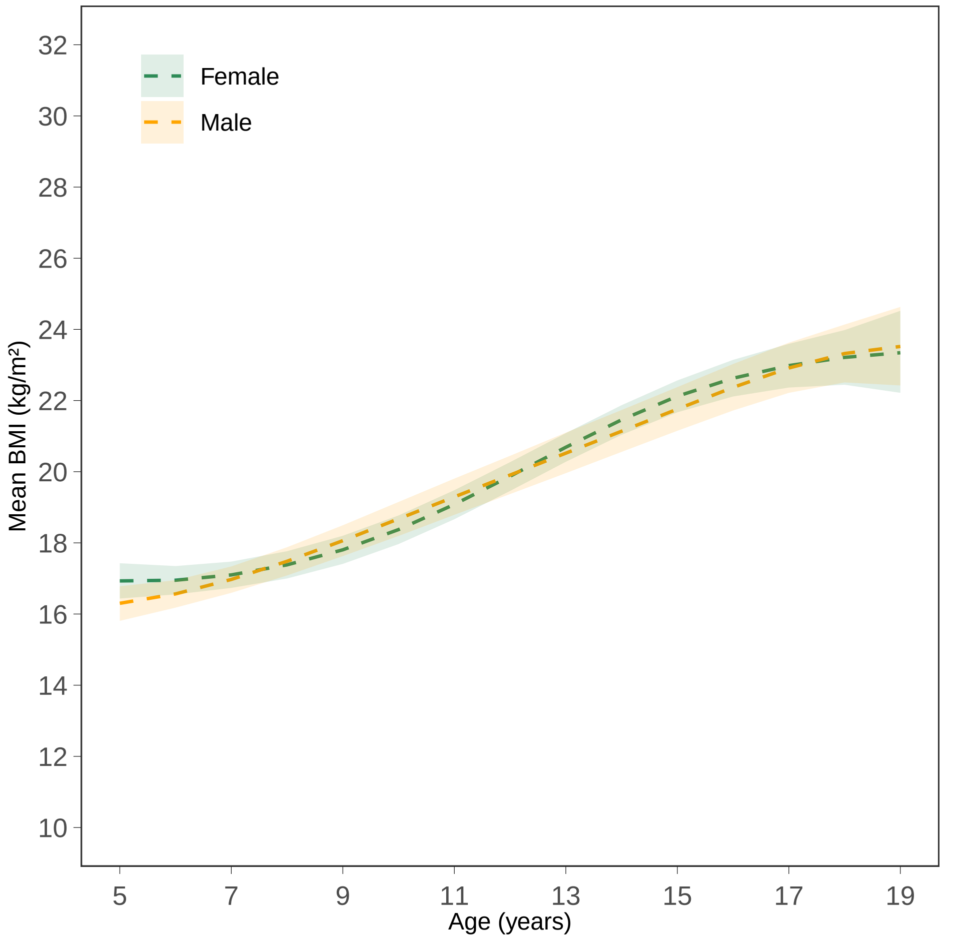

Uzbekistan

Time trends in height of 19 year olds

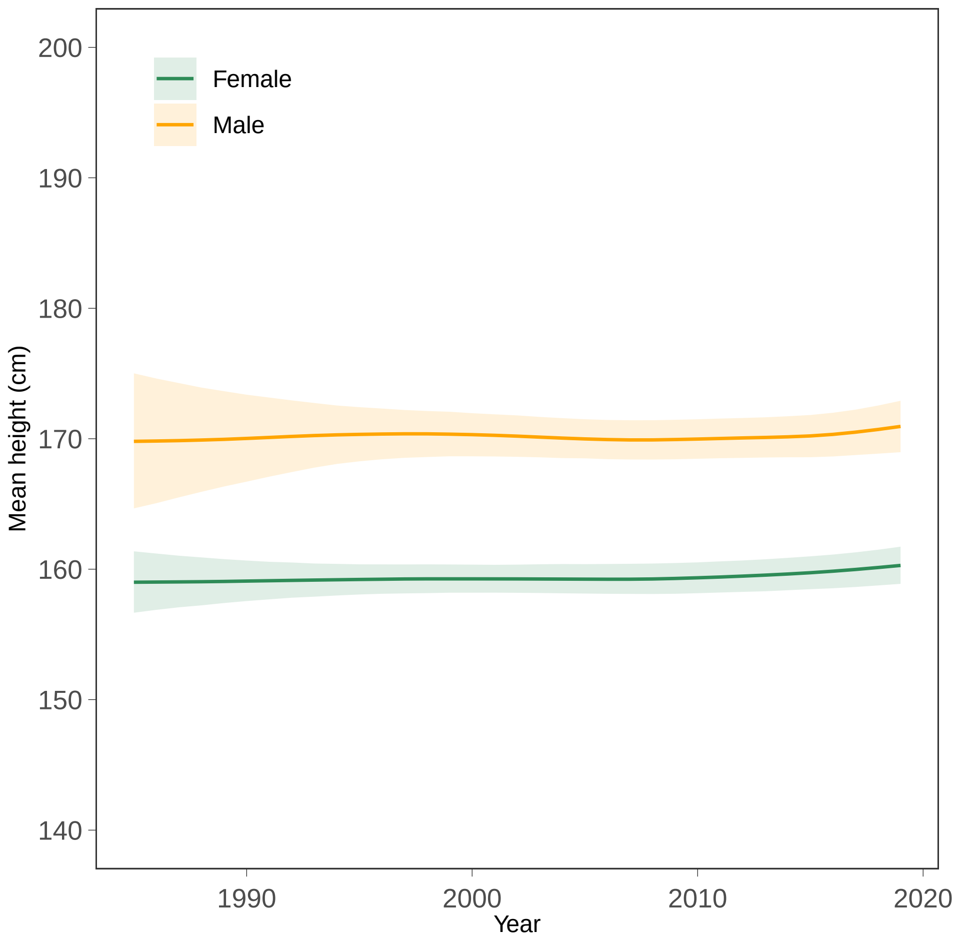

Time trends in BMI of 19 year olds

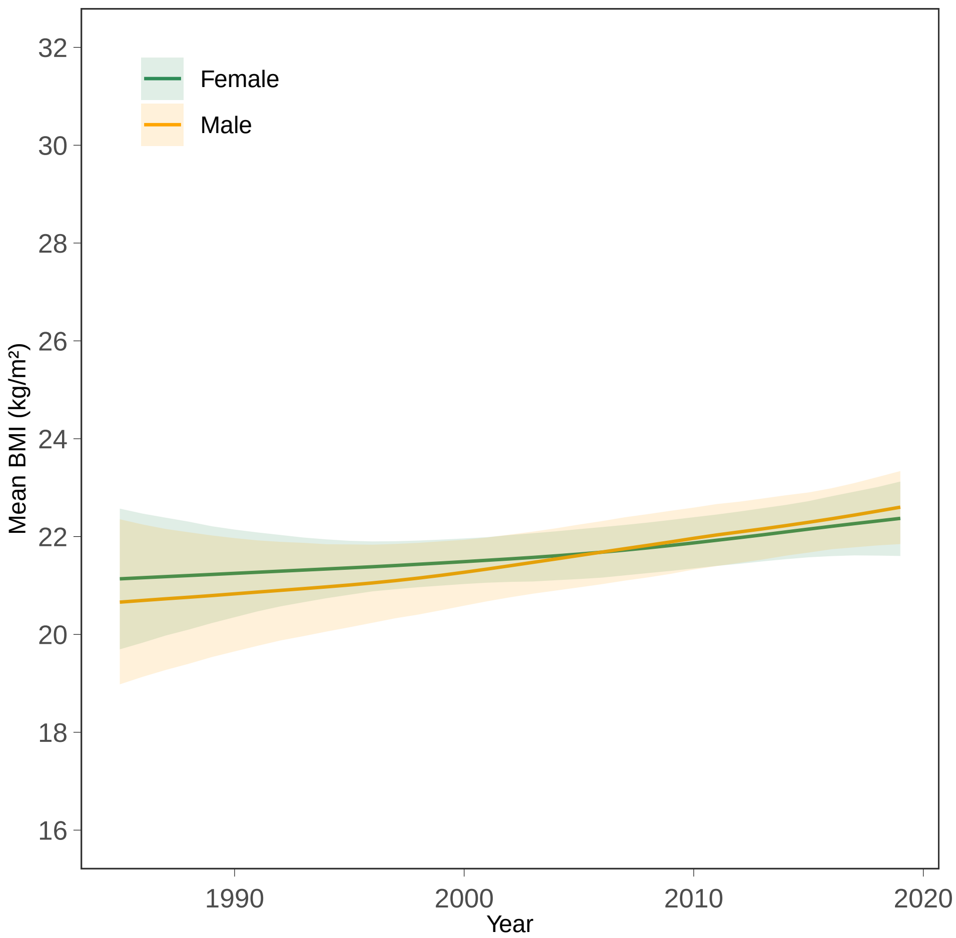

Height−for−age trajectories (2000 birth cohort)

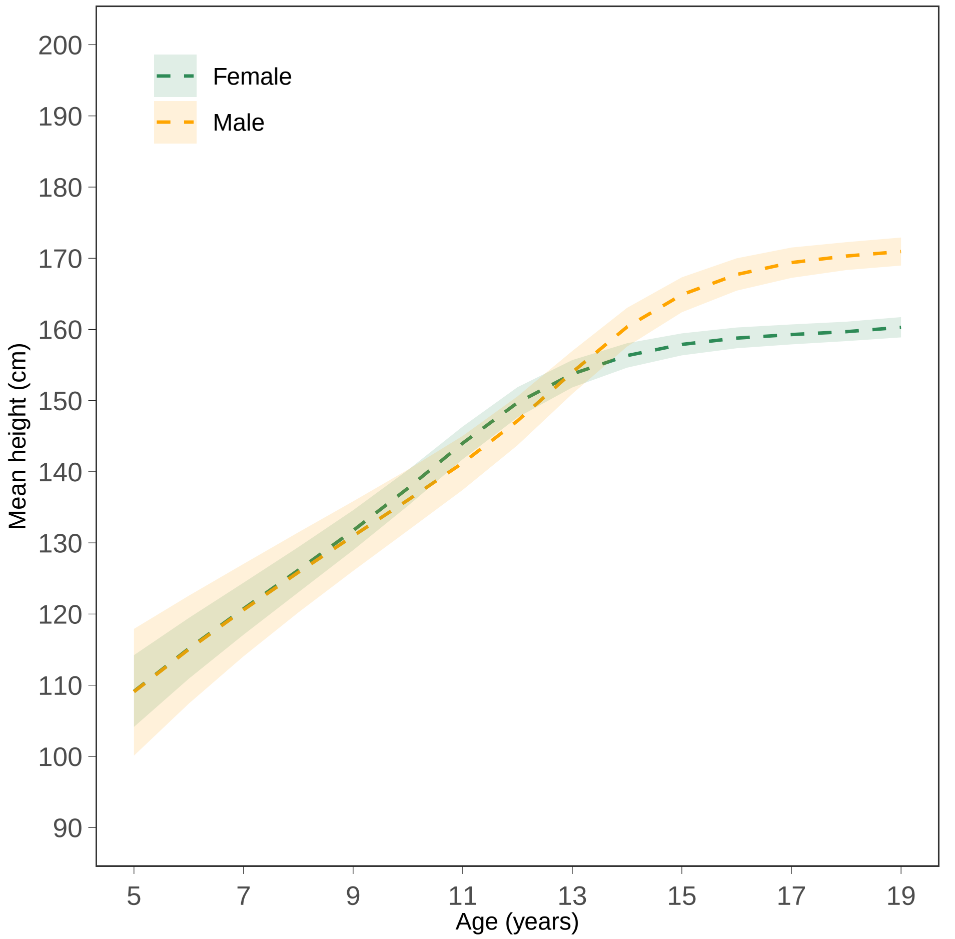

BMI−for−age trajectories (2000 birth cohort)

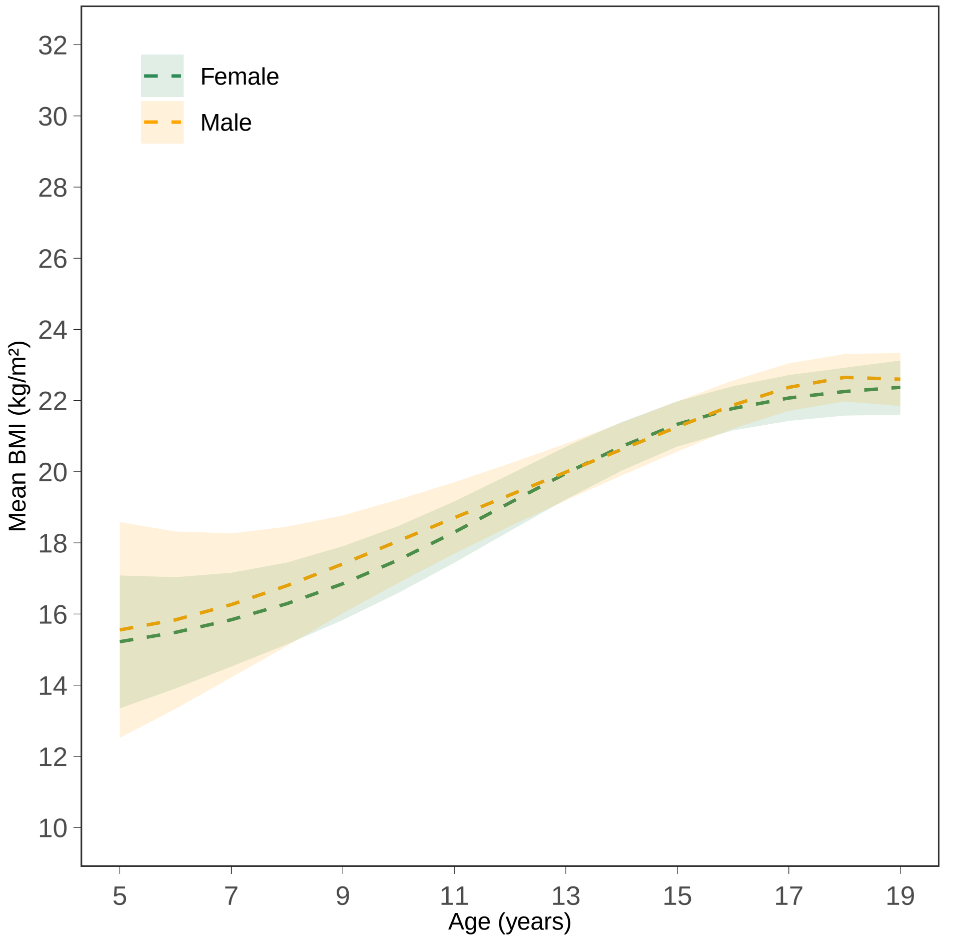

Vanuatu

Time trends in height of 19 year olds

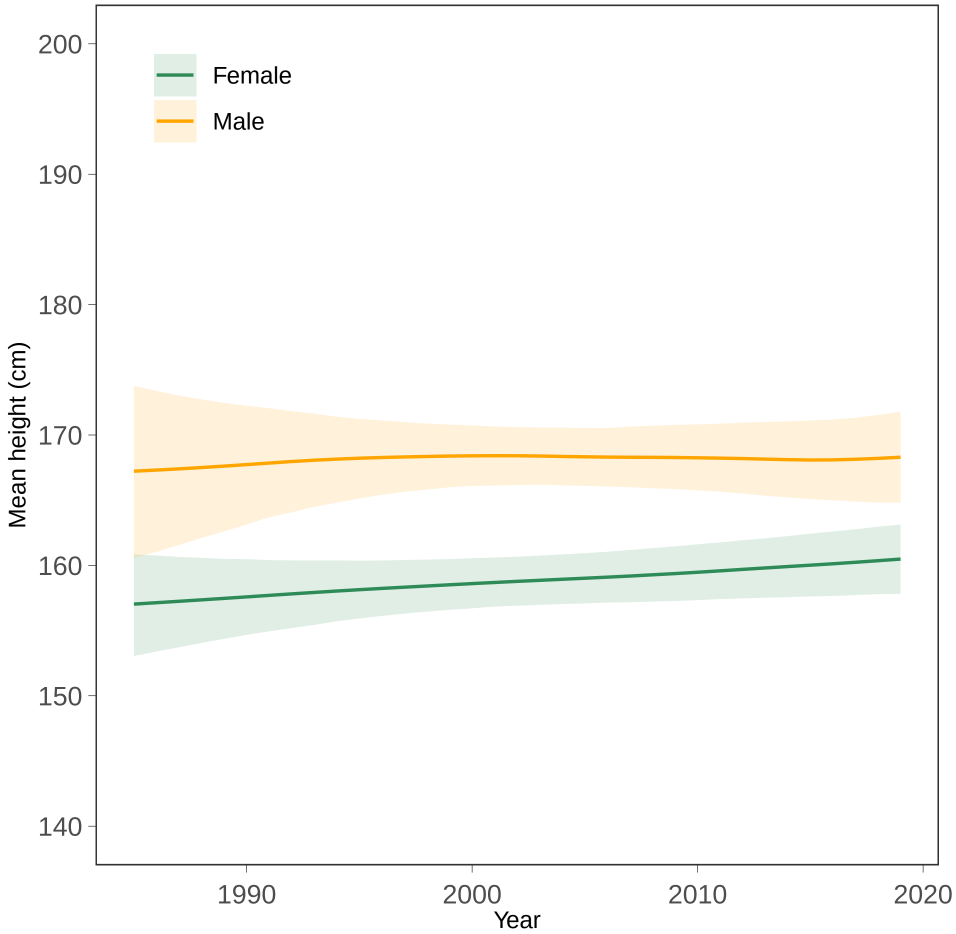

Time trends in BMI of 19 year olds

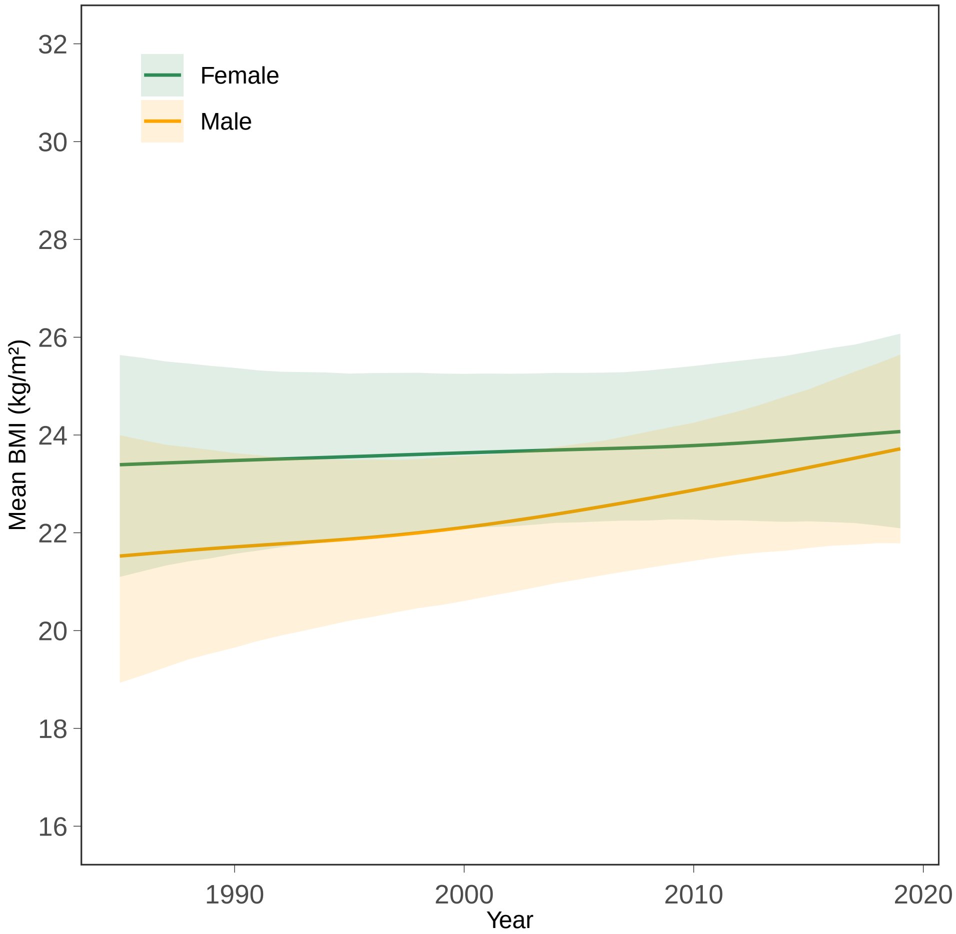

Height−for−age trajectories (2000 birth cohort)

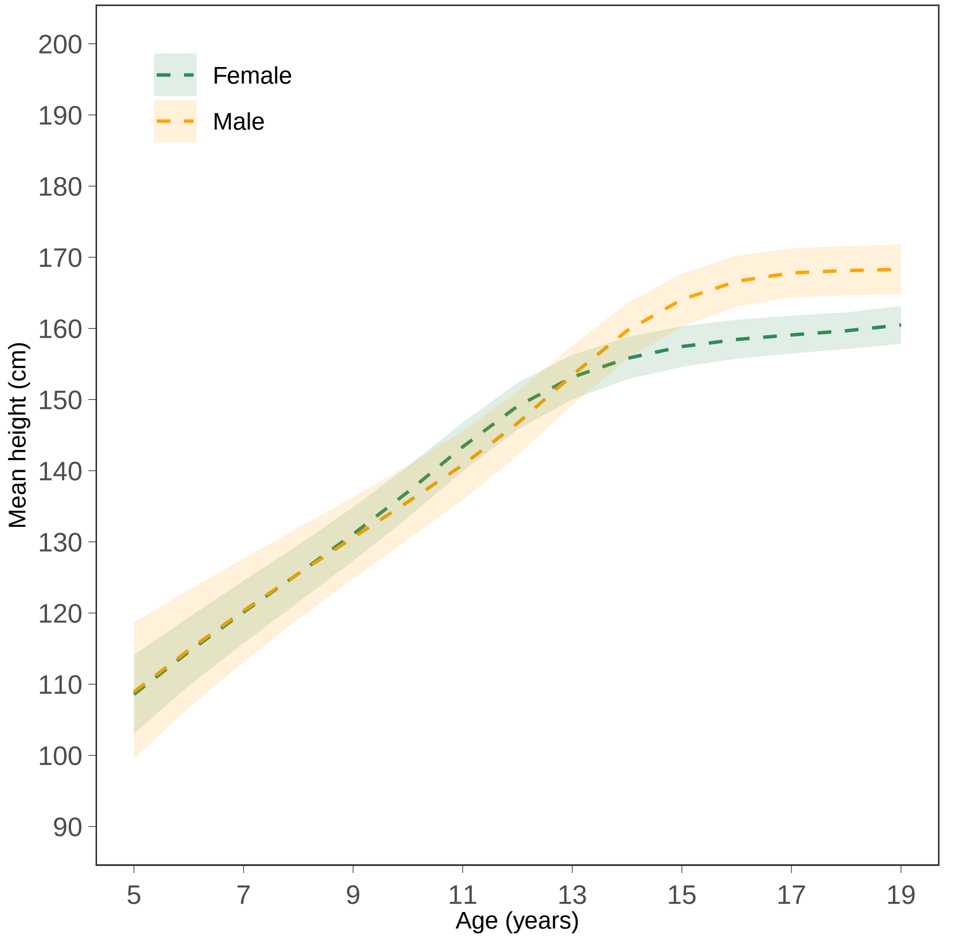

BMI−for−age trajectories (2000 birth cohort)

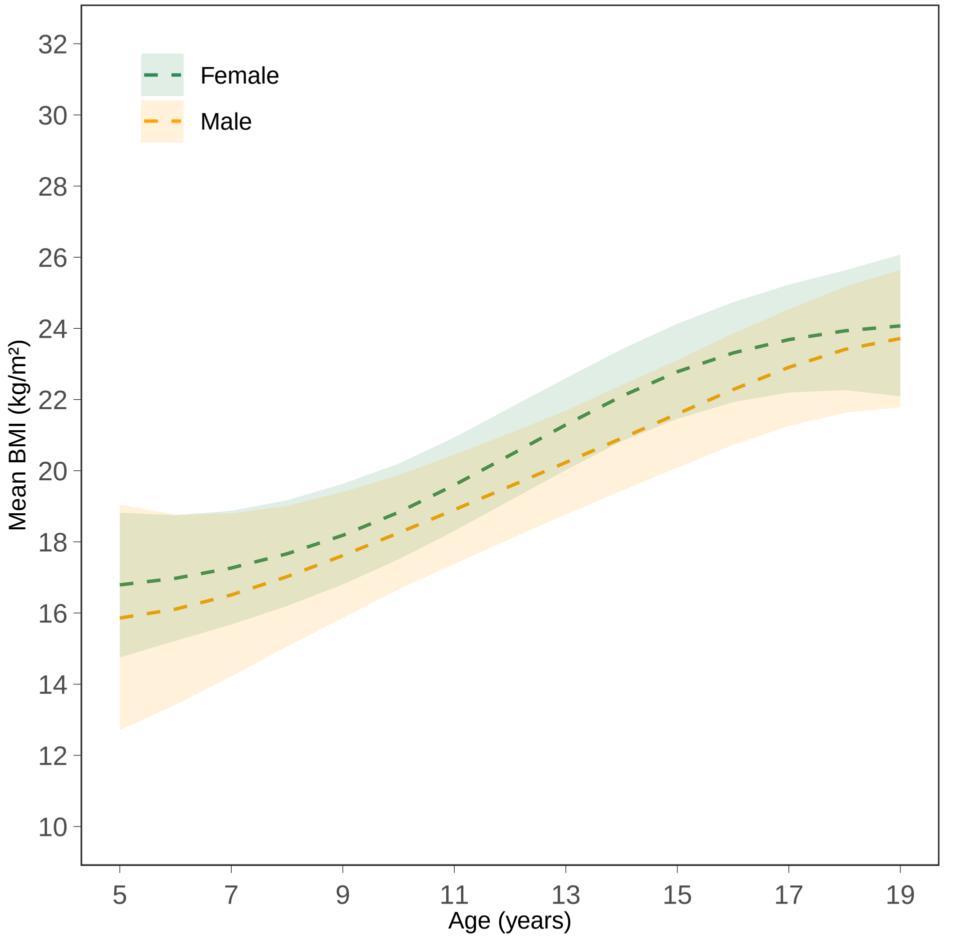

Venezuela

Time trends in height of 19 year olds

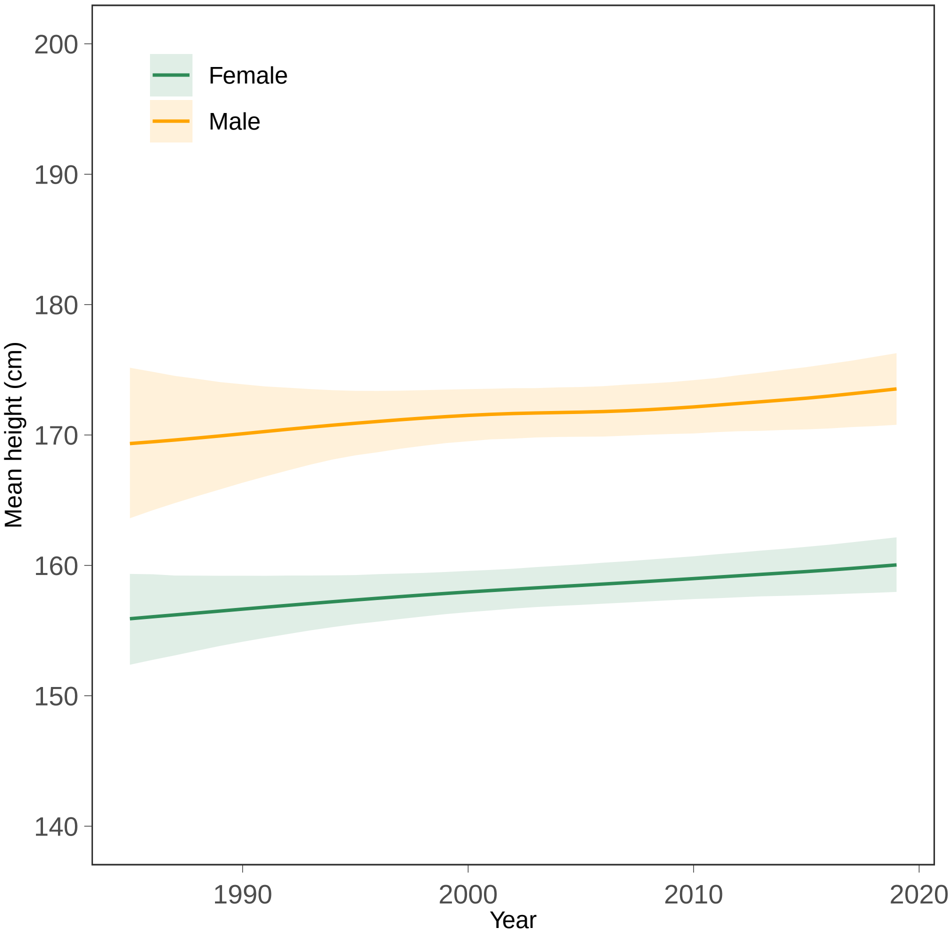

Time trends in BMI of 19 year olds

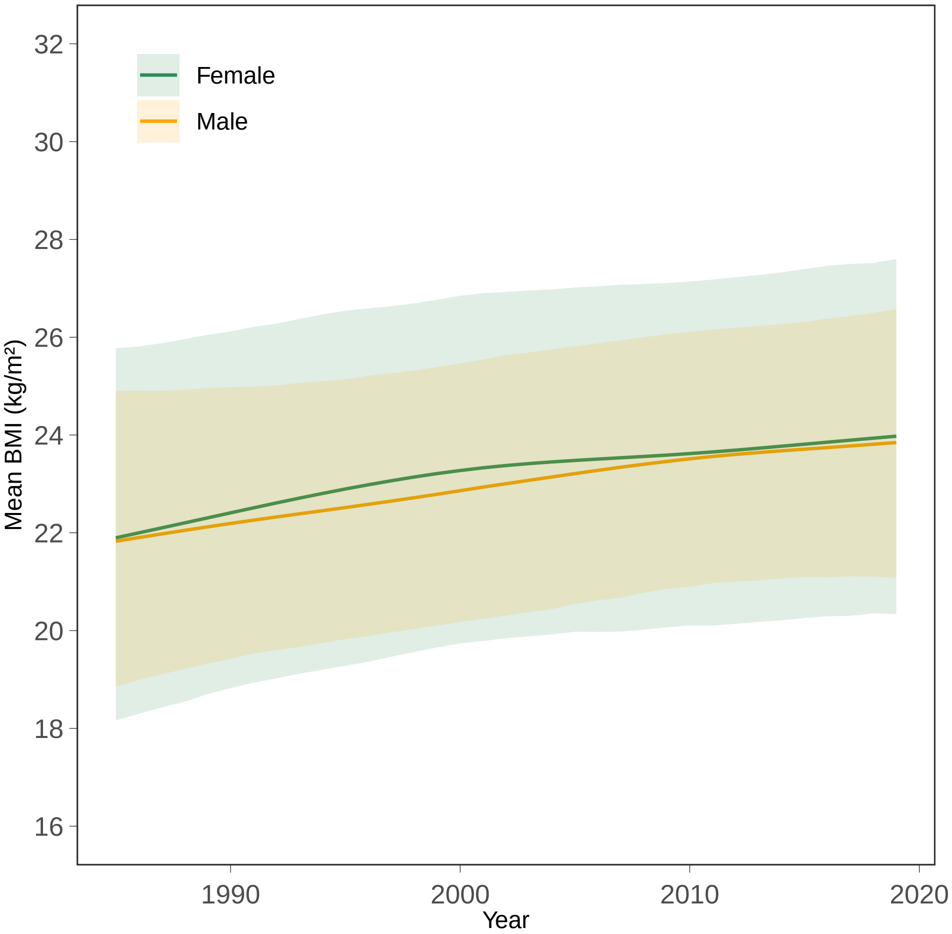

Height−for−age trajectories (2000 birth cohort)

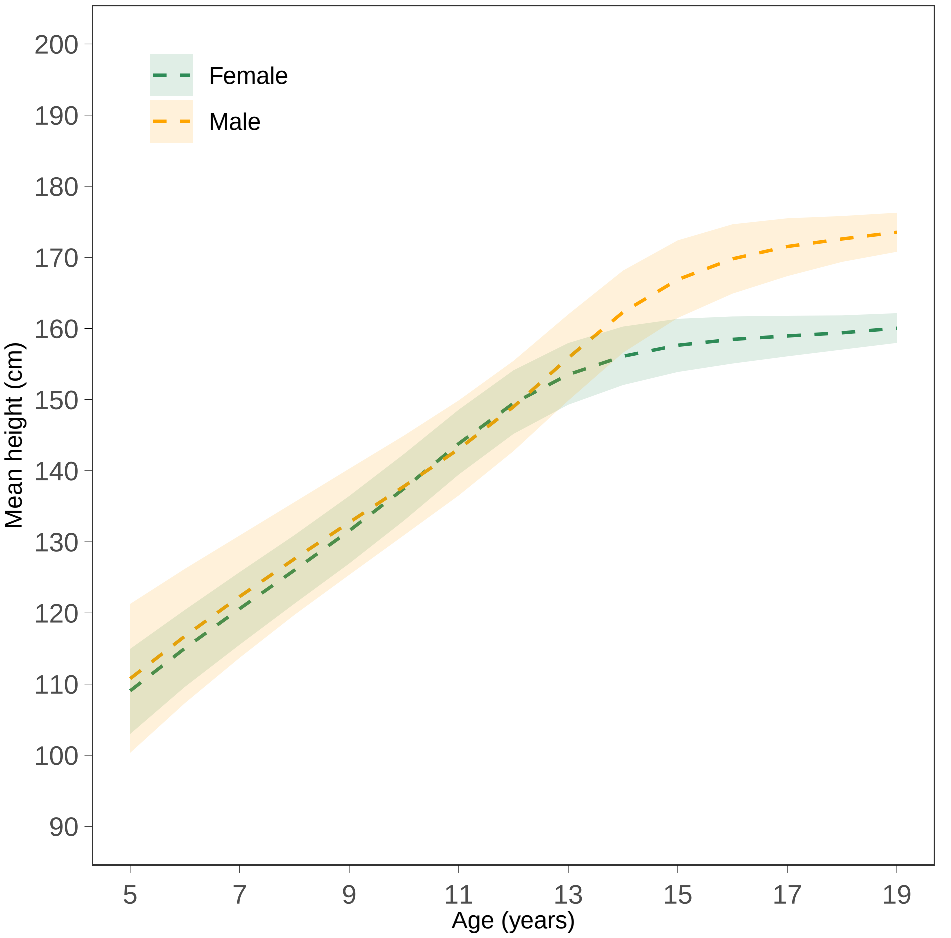

BMI−for−age trajectories (2000 birth cohort)

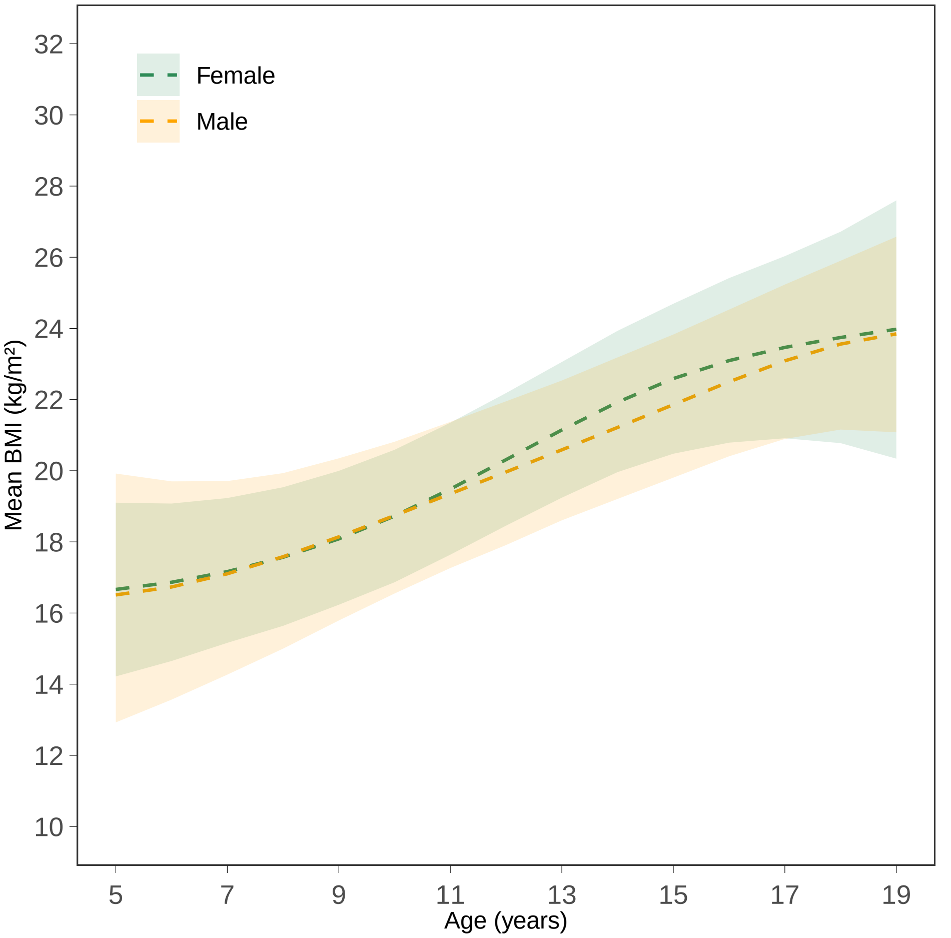

Viet Nam

Time trends in height of 19 year olds

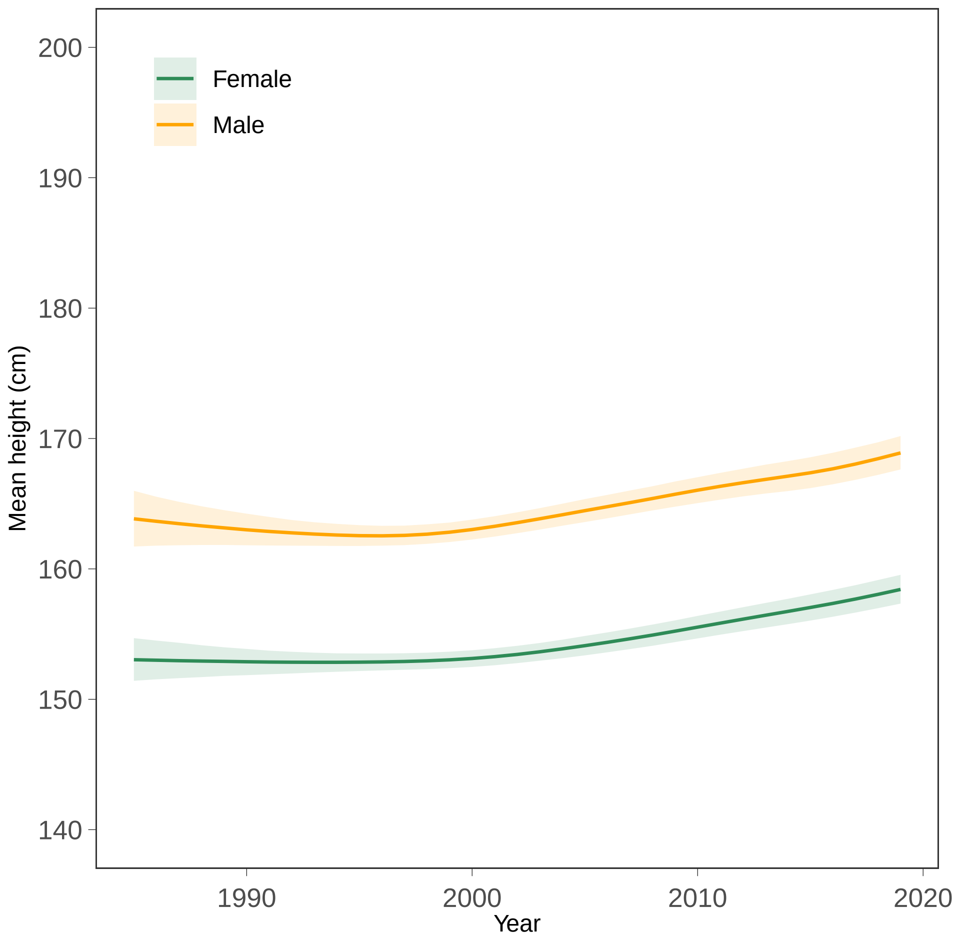

Time trends in BMI of 19 year olds

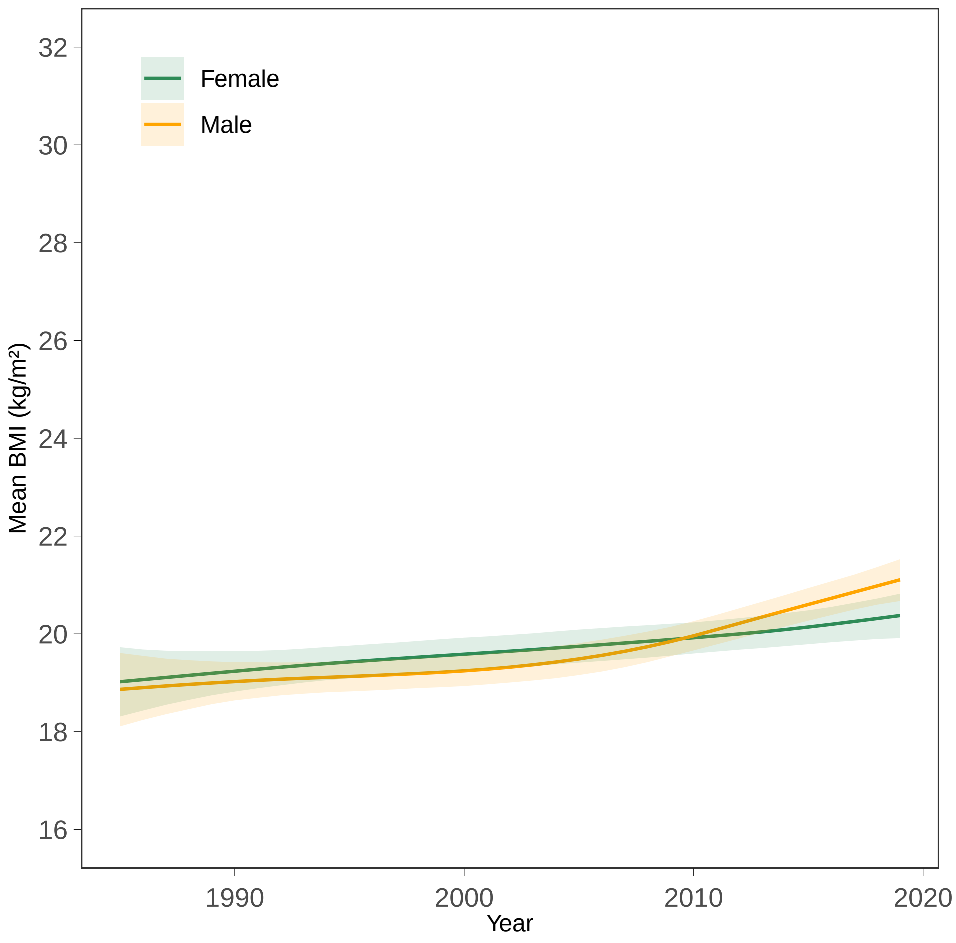

Height−for−age trajectories (2000 birth cohort)

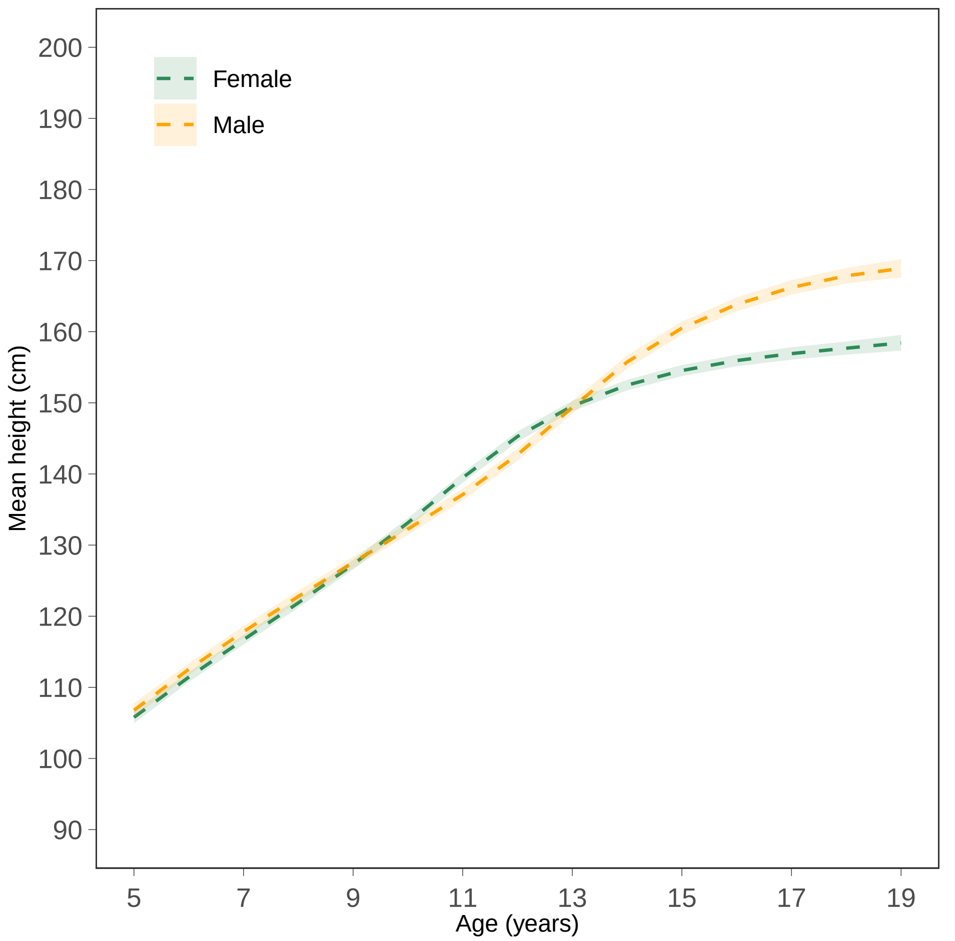

BMI−for−age trajectories (2000 birth cohort)

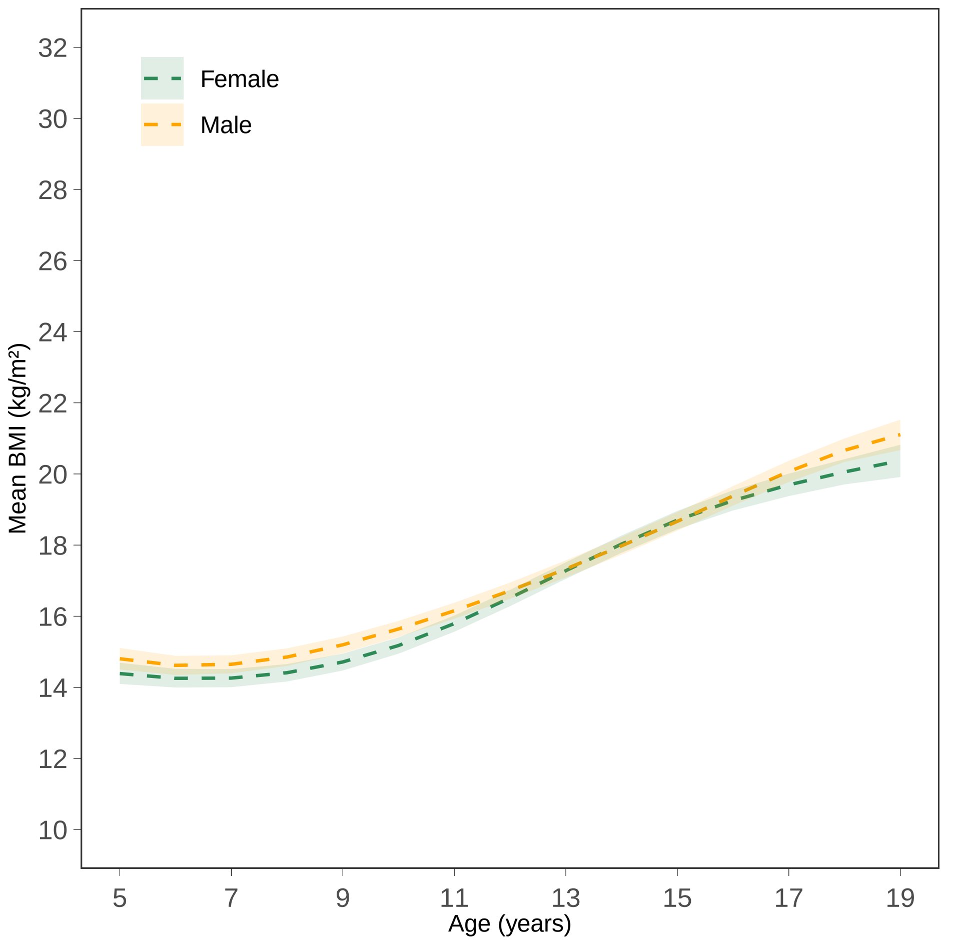

Yemen

Time trends in height of 19 year olds

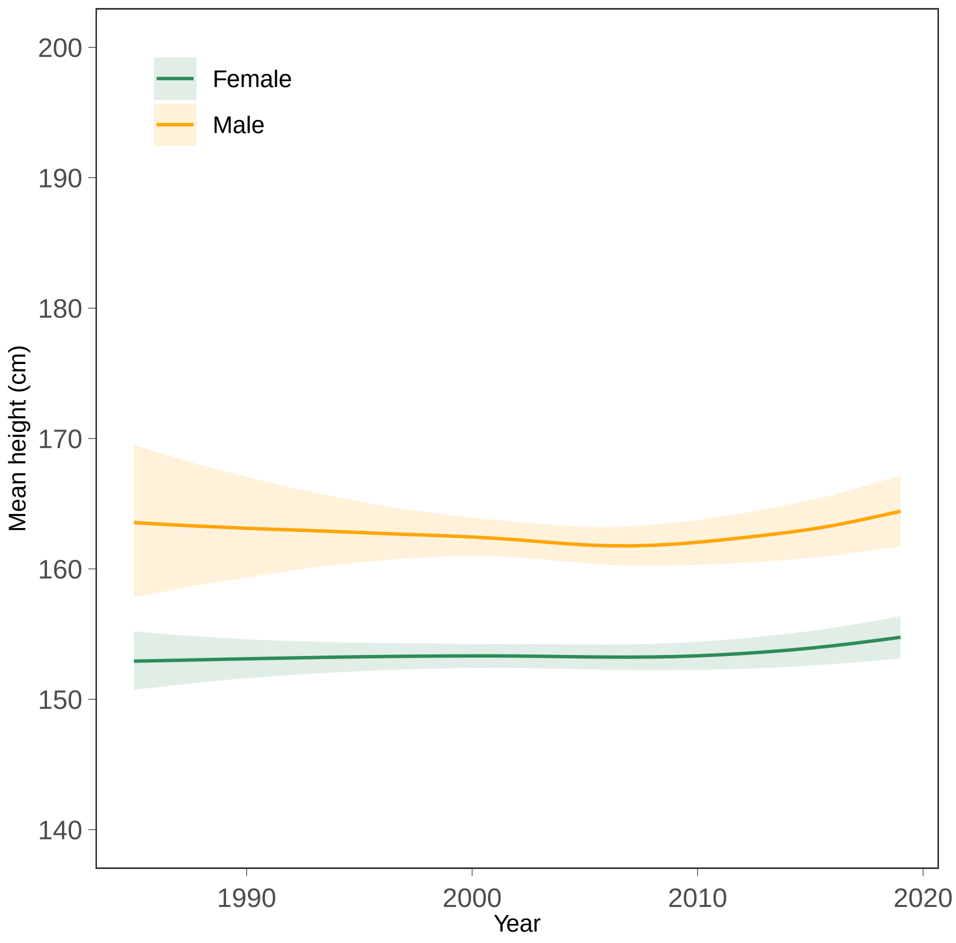

Time trends in BMI of 19 year olds

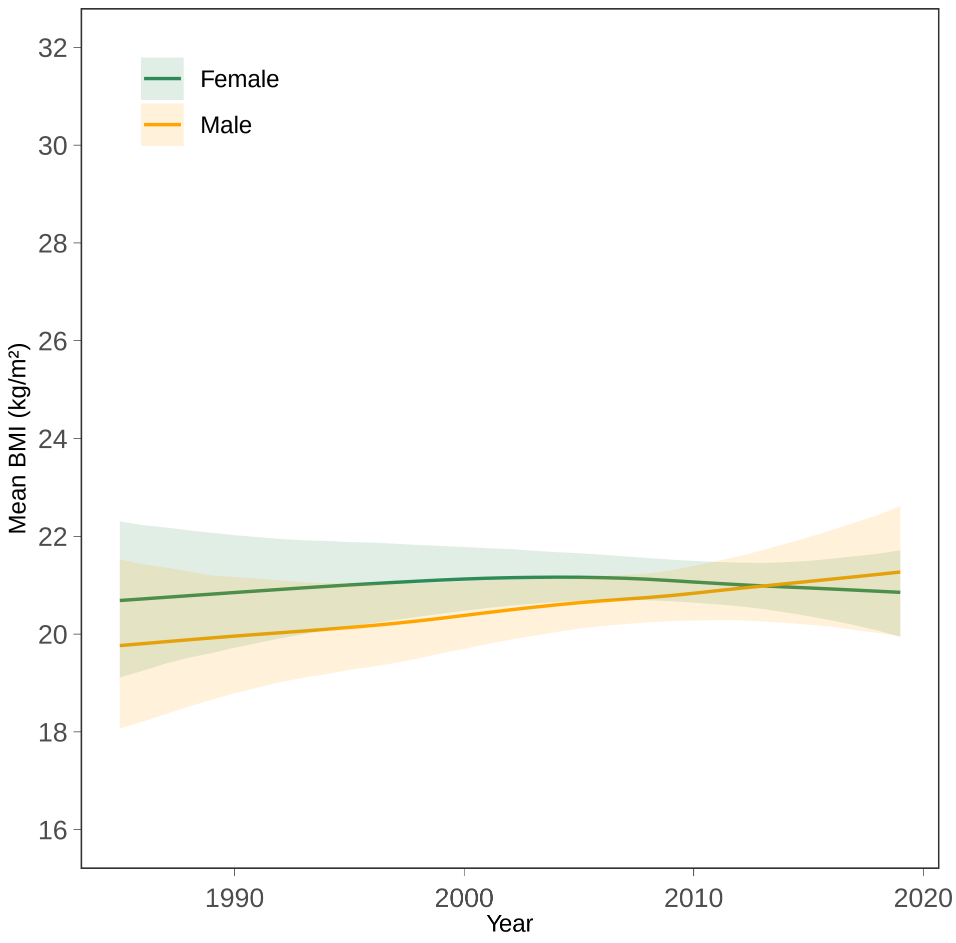

Height−for−age trajectories (2000 birth cohort)

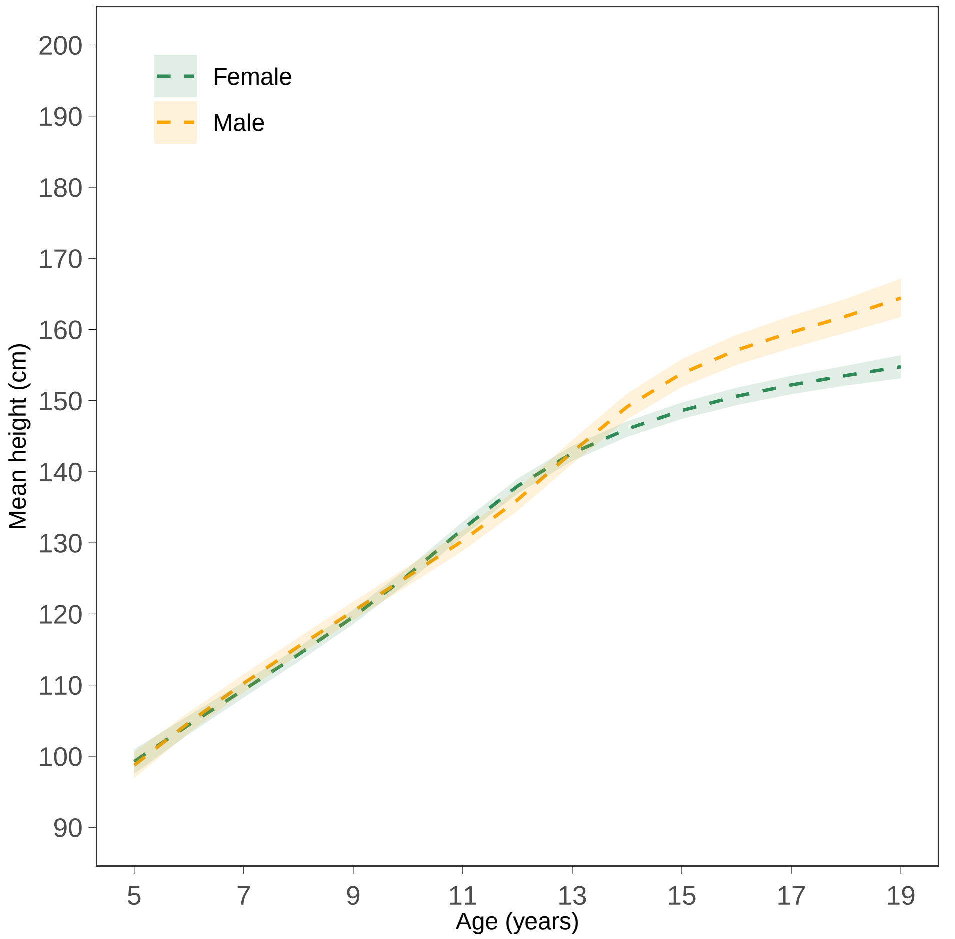

BMI−for−age trajectories (2000 birth cohort)

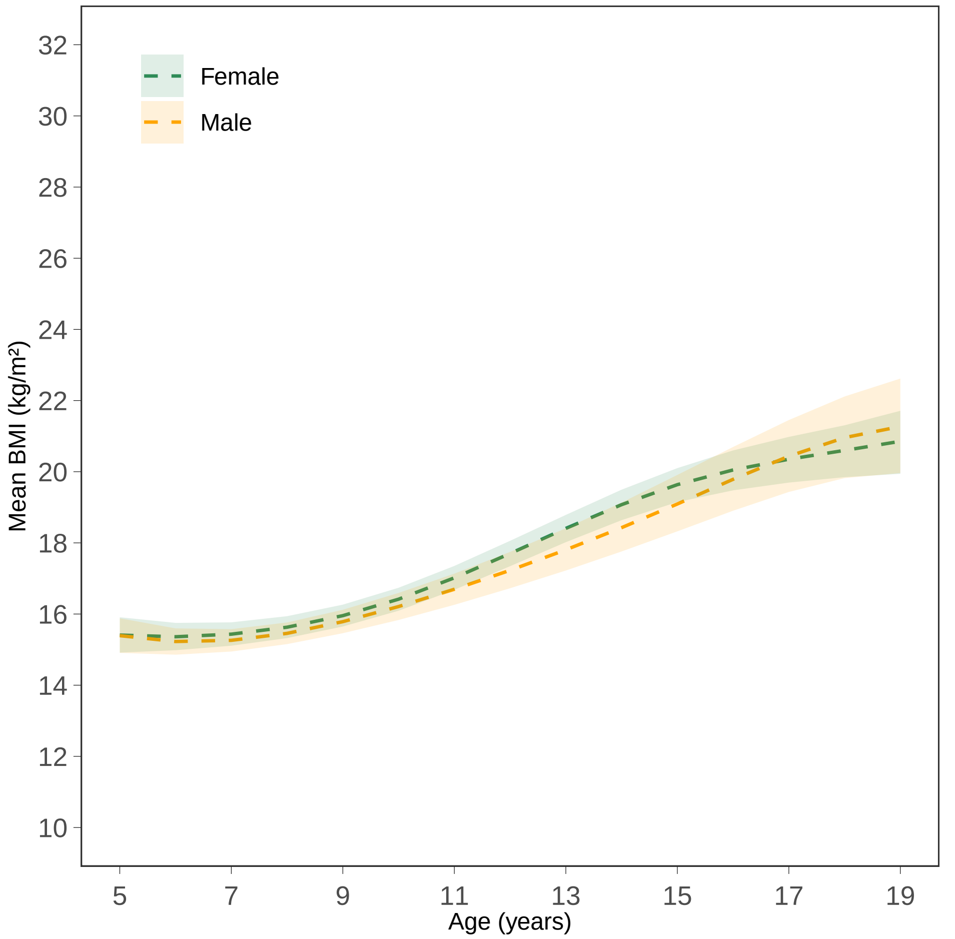

Zambia

Time trends in height of 19 year olds

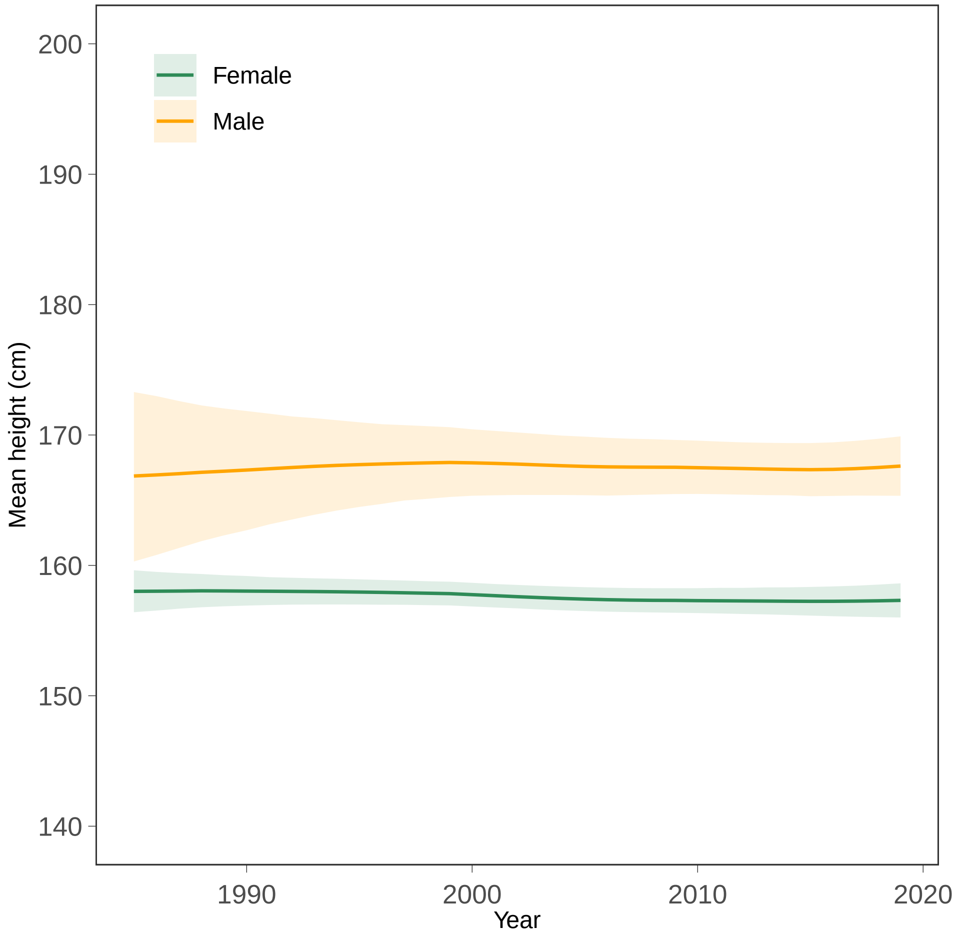

Time trends in BMI of 19 year olds

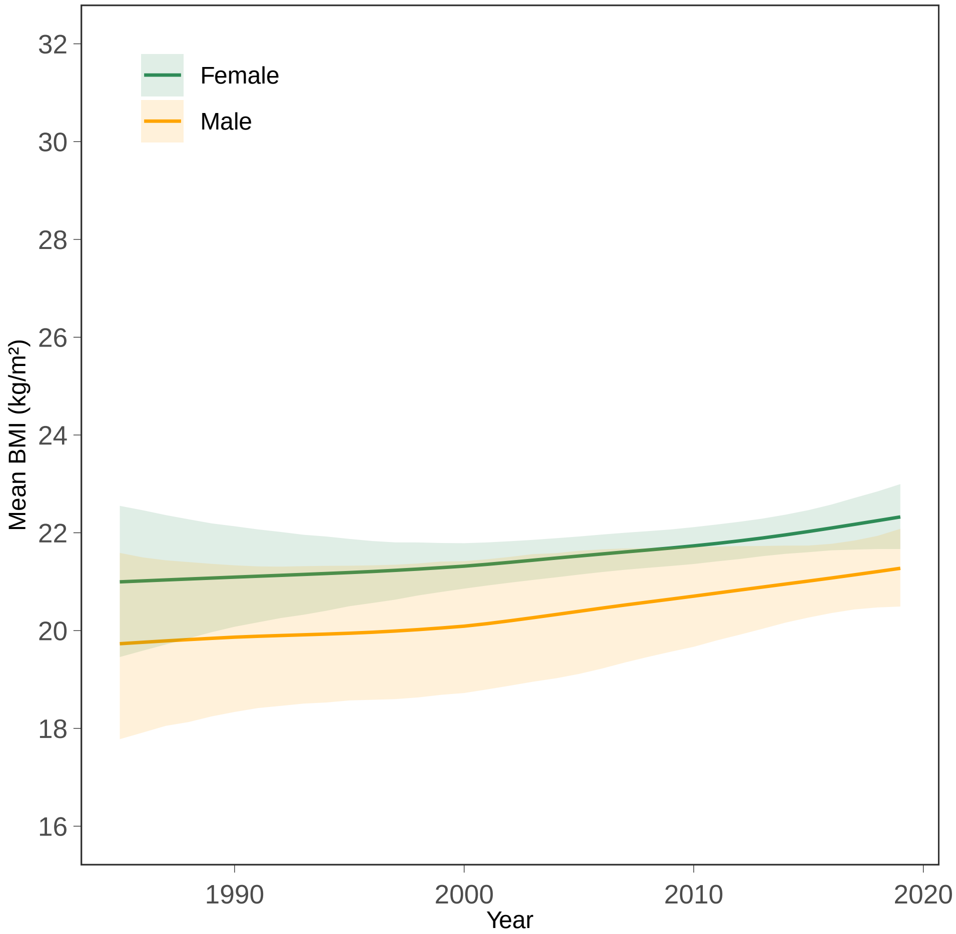

Height−for−age trajectories (2000 birth cohort)

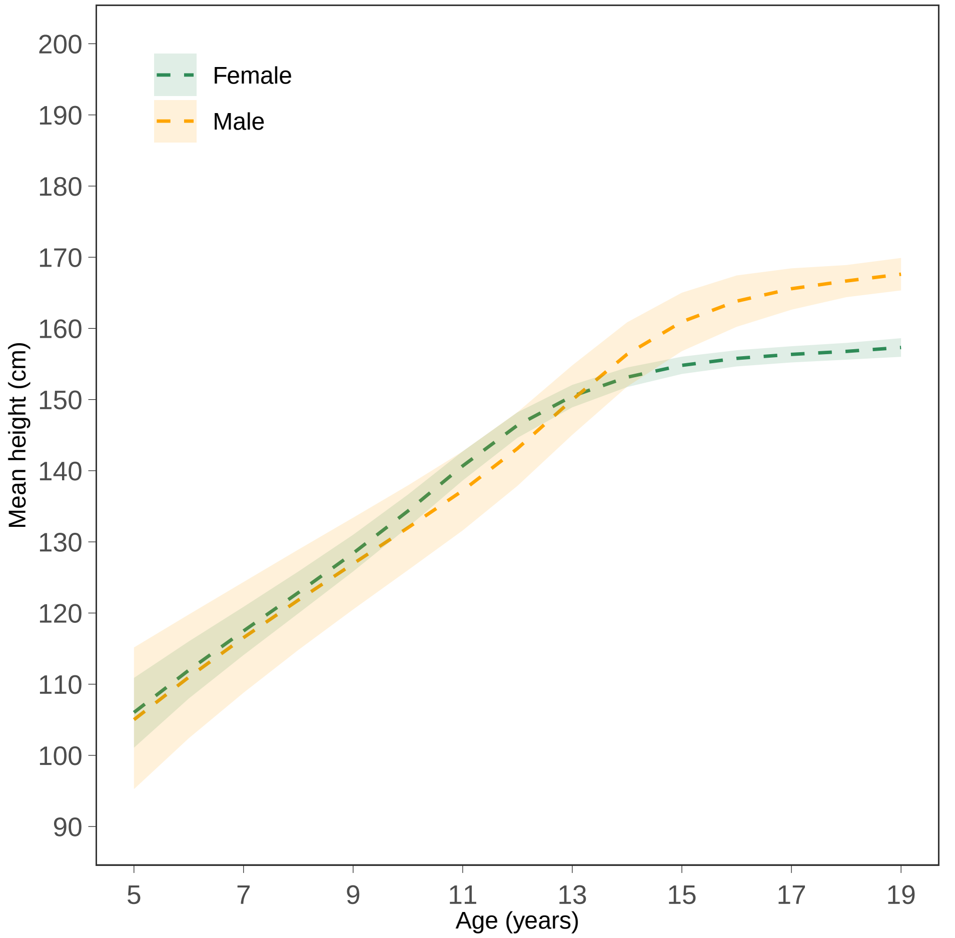

BMI−for−age trajectories (2000 birth cohort)

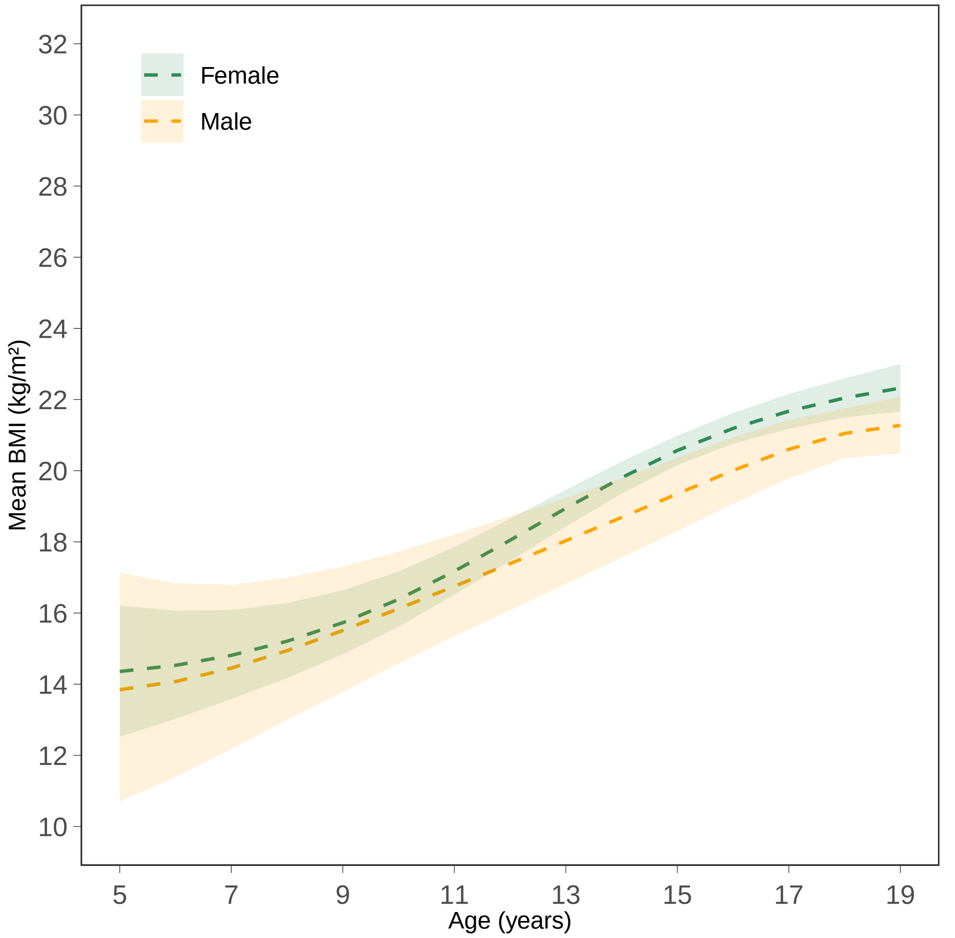

Zimbabwe

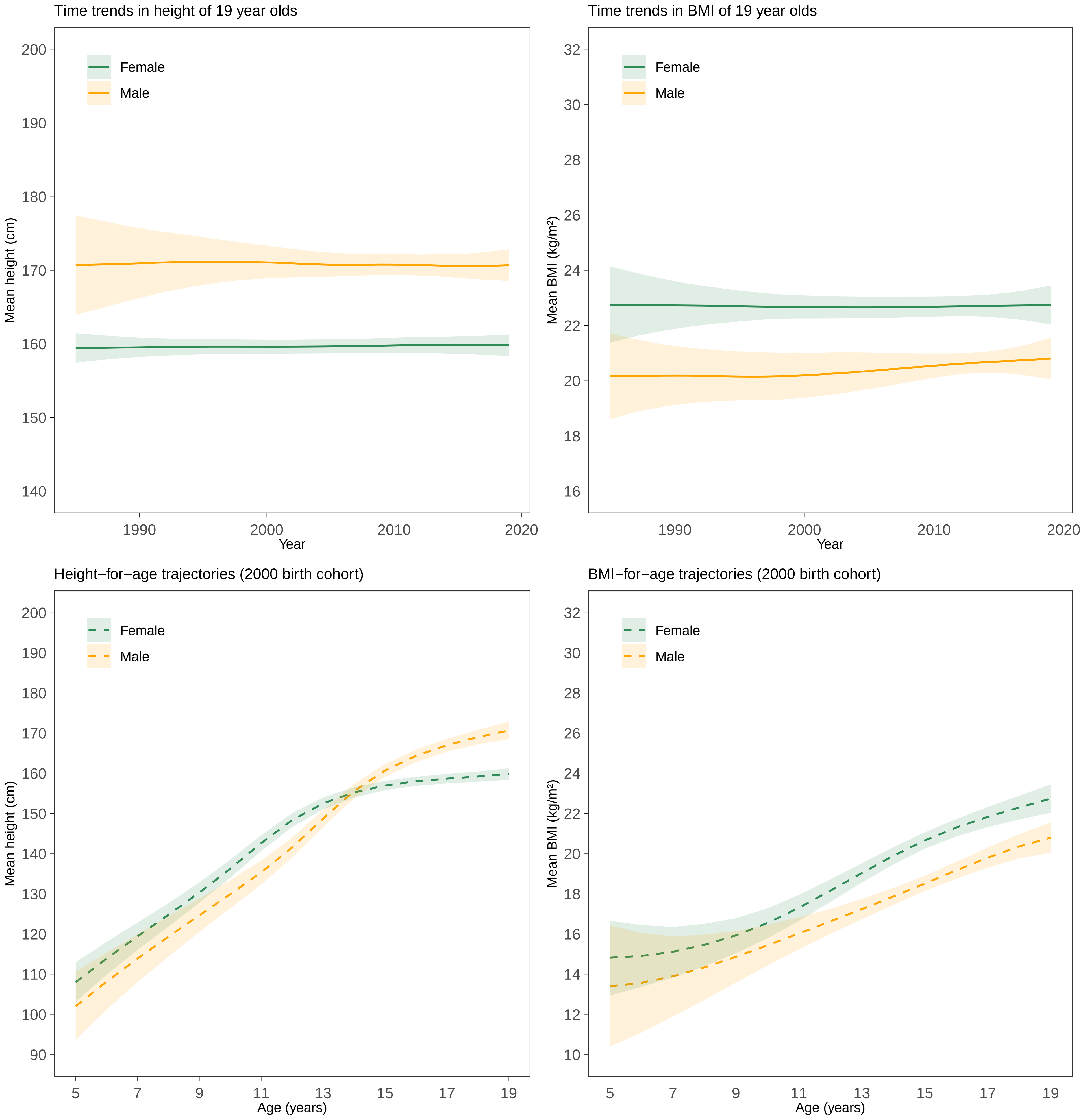

Time trends in height of 19 year olds
Female
Male
Mean height (cm)
Year
Time trends in BMI of 19 year olds
Mean BMI (kg/m²)
Height−for−age trajectories (2000 birth cohort)
Age (years)
BMI−for−age trajectories (2000 birth cohort)

Appendix Figure 5. Age trajectory of height and BMI for 2019 19-year-olds. The figure shows z-scores for mean height and BMI of 19-year-olds in 2019 (i.e., those born in 2000) at each age from 5 years to 19 years. Each cell represents the z-score, calculated based on the WHO growth reference[16], for a given age. Countries are ordered by region and within each region by the height of 19-year-old girls.

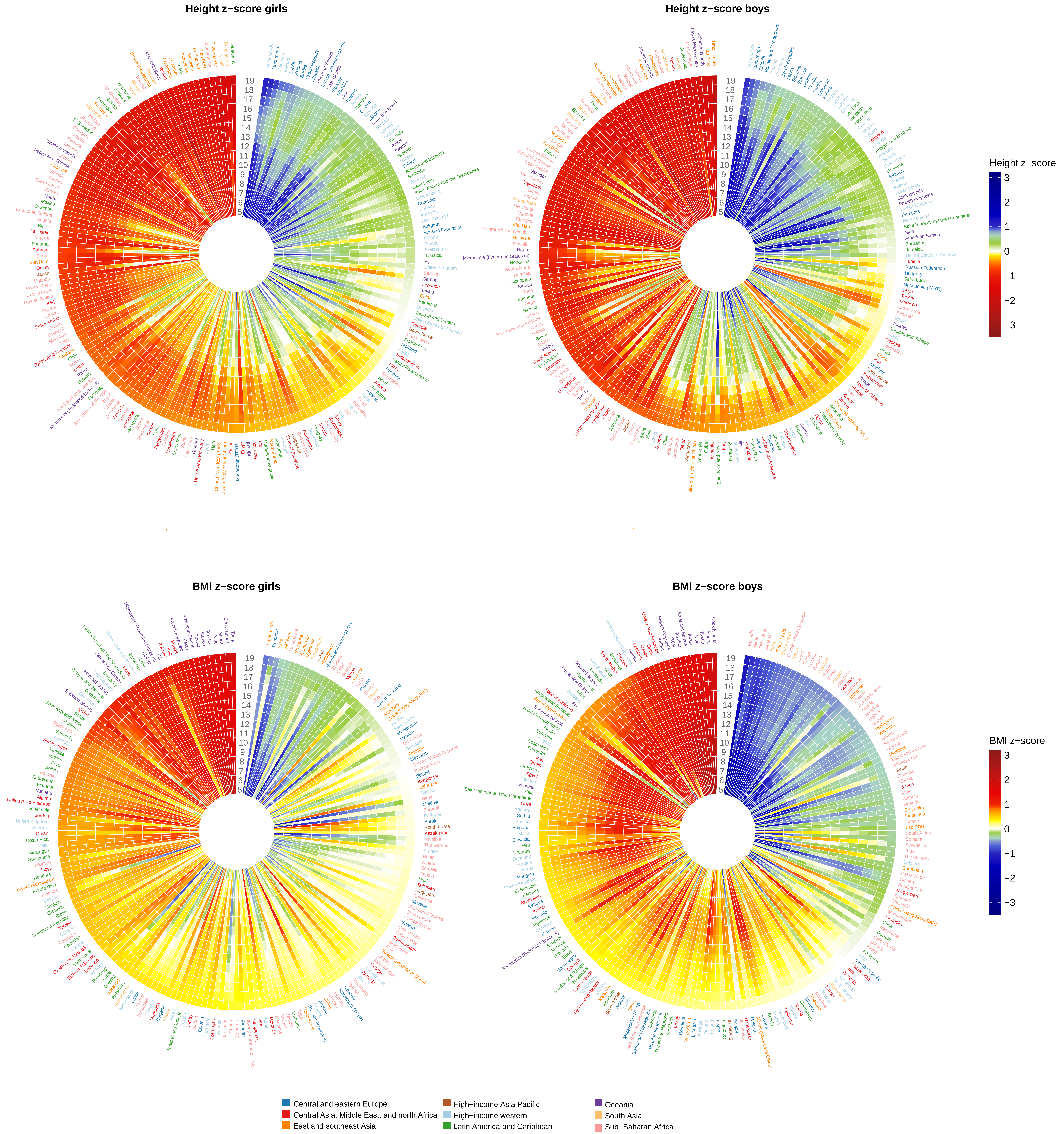

Height z−score girls
Height z−score boys
BMI z−score girls
BMI z−score boys
Height z−score
BMI z−score
Central and eastern Europe
Central Asia, Middle East, and north Africa
East and southeast Asia
High−income Asia Pacific
High−income western
Latin America and Caribbean
Oceania
South Asia
Sub−Saharan Africa

Appendix Figure 6. Difference between the height and BMI of boys and girls born who were 19 years old in 2019, by age. The figure shows the sex difference for mean height and BMI of 19-year-olds in 2019 (i.e., those born in 2000) at each age from 5 years to 19 years. Countries are ordered by region and within each region by the height of 19-year-old girls.

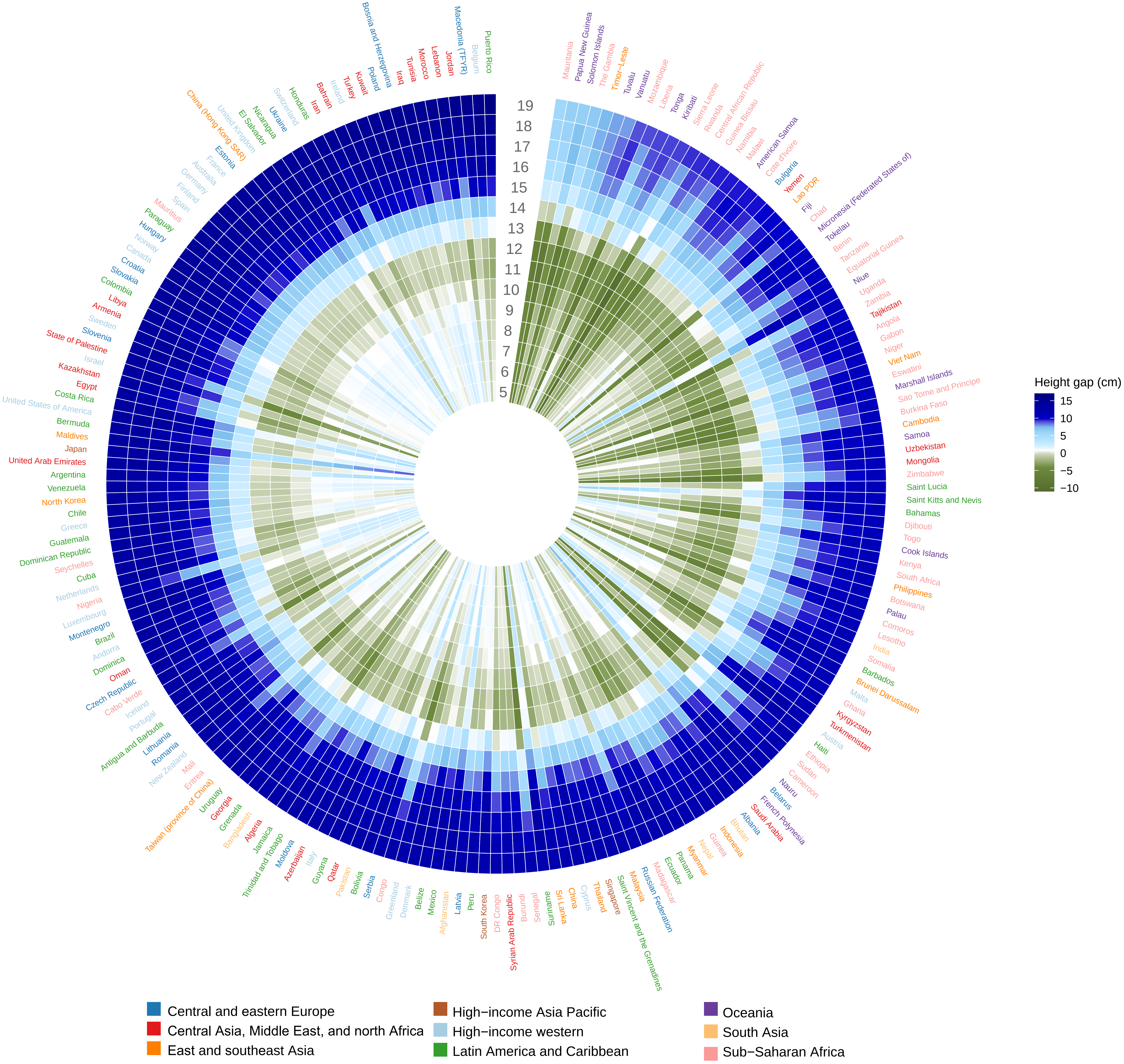

19
18
17
16
15
14
13
12
11
10
9
8
7
6
5
Mauritania
Papua New Guinea
Solomon Islands
The Gambia
Timor−Leste
Tuvalu
Vanuatu
Mozambique
Liberia
Tonga
Kiribati
Sierra Leone
Rwanda
Central African Republic
Guinea Bissau
Namibia
Malawi
American Samoa
Cote d'Ivoire
Bulgaria
Yemen
Lao PDR
Fiji
Chad
Micronesia (Federated States of)
Tokelau
Benin
Tanzania
Equatorial Guinea
Niue
Uganda
Zambia
Tajikistan
Angola
Gabon
Niger
Viet Nam
Eswatini
Marshall Islands
Sao Tome and Principe
Burkina Faso
Cambodia
Samoa
Uzbekistan
Mongolia
Zimbabwe
Saint Lucia
Saint Kitts and Nevis
Bahamas
Djibouti
Togo
Cook Islands
Kenya
South Africa
Philippines
Botswana
Palau
Comoros
Lesotho
India
Somalia
Barbados
Brunei Darussalam
Malta
Ghana
Kyrgyzstan
Turkmenistan
Austria
Haiti
Ethiopia
Sudan
Cameroon
Nauru
Belarus
French Polynesia
Saudi Arabia
Albania
Bhutan
Indonesia
Guinea
Nepal
Myanmar
Panama
Ecuador
Madagascar
Russian Federation
Malaysia
Saint Vincent and the Grenadines
Singapore
Thailand
Cyprus
China
Sri Lanka
Suriname
Senegal
Burundi
Syrian Arab Republic
DR Congo
South Korea
Peru
Latvia
Afghanistan
Mexico
Belize
Denmark
Greenland
Congo
Serbia
Bolivia
Pakistan
Qatar
Guyana
Italy
Azerbaijan
Moldova
Trinidad and Tobago
Jamaica
Algeria
Bangladesh
Grenada
Georgia
Uruguay
Taiwan (province of China)
Eritrea
Mali
New Zealand
Romania
Lithuania
Antigua and Barbuda
Portugal
Iceland
Cabo Verde
Czech Republic
Oman
Dominica
Andorra
Brazil
Montenegro
Luxembourg
Nigeria
Netherlands
Cuba
Seychelles
Dominican Republic
Guatemala
Greece
Chile
North Korea
Venezuela
Argentina
United Arab Emirates
Japan
Maldives
Bermuda
United States of America
Costa Rica
Egypt
Kazakhstan
Israel
State of Palestine
Slovenia
Sweden
Armenia
Libya
Colombia
Slovakia
Croatia
Canada
Norway
Hungary
Paraguay
Mauritius
Spain
Finland
Germany
Australia
France
Estonia
China (Hong Kong SAR)
United Kingdom
El Salvador
Nicaragua
Ukraine
Switzerland
Honduras
Bahrain
Iran
Ireland
Turkey
Kuwait
Poland
Bosnia and Herzegovina
Iraq
Tunisia
Morocco
Lebanon
Jordan
Macedonia (TFYR)
Belgium
Puerto Rico
Height gap (cm)
15
10
5
0
−5
−10
Central and eastern Europe
Central Asia, Middle East, and north Africa
East and southeast Asia
High−income Asia Pacific
High−income western
Latin America and Caribbean
Oceania
South Asia
Sub−Saharan Africa

References

1. NCD Risk Factor Collaboration (NCD-RisC). Worldwide trends in body-mass index, underweight, overweight, and obesity from 1975 to 2016: a pooled analysis of 2416 population-based measurement studies in 128.9 million children, adolescents, and adults. Lancet 2017; 390(10113): 2627-42.
2. Danaei G, Finucane MM, Lin JK, et al. National, regional, and global trends in systolic blood pressure since 1980: systematic analysis of health examination surveys and epidemiological studies with 786 country-years and 5.4 million participants. Lancet 2011; 377(9765): 568-77.
3. Danaei G, Finucane MM, Lu Y, et al. National, regional, and global trends in fasting plasma glucose and diabetes prevalence since 1980: systematic analysis of health examination surveys and epidemiological studies with 370 country-years and 2.7 million participants. Lancet 2011; 378(9785): 31-40.
4. Farzadfar F, Finucane MM, Danaei G, et al. National, regional, and global trends in serum total cholesterol since 1980: systematic analysis of health examination surveys and epidemiological studies with 321 country-years and 3.0 million participants. Lancet 2011; 377(9765): 578-86.
5. Finucane MM, Stevens GA, Cowan MJ, et al. National, regional, and global trends in body-mass index since 1980: systematic analysis of health examination surveys and epidemiological studies with 960 country-years and 9.1 million participants. Lancet 2011; 377(9765): 557-67.
6. Cline MG, Meredith KE, Boyer JT, Burrows B. Decline of Height with Age in Adults in a General-Population Sample - Estimating Maximum Height and Distinguishing Birth Cohort Effects from Actual Loss of Stature with Aging. Human Biology 1989; 61(3): 415-25.
7. Hayes AJ, Clarke PM, Lung TWC. Change in bias in self-reported body mass index in Australia between 1995 and 2008 and the evaluation of correction equations. Popul Health Metr 2011;9(1): 53.
8. Gorber SC, Tremblay M, Moher D, Gorber B. Diagnostic in obesity comorbidities - A comparison of direct vs. self-report measures for assessing height, weight and body mass index: a systematic review. Obesity Reviews 2007; 8(4): 307-26.
9. Ezzati M, Martin H, Skjold S, Hoorn SV, Murray CJL. Trends in national and state-level obesity in the USA after correction for self-report bias: analysis of health surveys. J Roy Soc Med 2006; 99(5): 250-7.
10. Finucane MM, Paciorek CJ, Danaei G, Ezzati M. Bayesian Estimation of Population-Level Trends in Measures of Health Status. Stat Sci 2014; 29(1): 18-25.
11. NCD Risk Factor Collaboration (NCD-RisC). A century of trends in adult human height. Elife 2016; 5: e13410.
12. Deaton A. Height, health, and development. Proc Natl Acad Sci U S A 2007; 104(33): 13232-7.
13. Rue H, Held L. Gaussian Markov random fields: theory and applications: Chapman and Hall, Boca Raton; 2005.
14. Cameron N, Barry B. Human growth and development: Academic Press An Elsevier Science Company 525 B Street, Suite 1900, San Diego, California 92101-4495, USA; 2012.
15. Tanner JM, Whitehouse RH, Marubini E, Resele LF. The adolescent growth spurt of boys and girls of the Harpenden growth study. Ann Hum Biol 1976; 3(2): 109-26.
16. de Onis M, Onyango AW, Borghi E, Siyam A, Nishida C, Siekmann J. Development of a WHO growth reference for school-aged children and adolescents. B World Health Organ 2007; 85(9): 660-7
17. de Leeuw J, Hornik K, Mair P. Isotone Optimization in R: Pool-Adjacent-Violators Algorithm (PAVA) and Active Set Methods. J Stat Softw 2009; 32(5): 1-24.

18. Best MJ, Chakravarti N. Active Set Algorithms for Isotonic Regression - a Unifying Framework. Math Program 1990; 47(3): 425-39.
19. NCD Risk Factor Collaboration (NCD-RisC). Worldwide trends in diabetes since 1980: a pooled analysis of 751 population-based studies with 4.4 million participants. Lancet 2016; 387(10027): 1513-30.
20. NCD Risk Factor Collaboration (NCD-RisC). Worldwide trends in blood pressure from 1975 to 2015: a pooled analysis of 1479 population-based measurement studies with 19.1 million participants. Lancet 2017; 389(10064): 37-55.
21. Xu Y, Hang L. Height inequalities and their change trends in China during 1985-2010: results from 6 cross-sectional surveys on children and adolescents aged 7-18 years. BMC Public Health 2017; 17(473): 1-11.
